# Cancer Screening and Prevention in MENA and Mediterranean Populations: A Multi-Level Analysis of Barriers, Knowledge Gaps, and Interventions Across Indigenous and Diaspora Communities

**DOI:** 10.3390/diseases14010010

**Published:** 2025-12-28

**Authors:** Sebahat Gozum, Omar F. Nimri, Mohammed Abdulridha Merzah, Rui Vitorino

**Affiliations:** 1 Department of Public Health Nursing, School of Nursing, Akdeniz University, Antalya 07070, Türkiye; 2 Public Health—Epidemiology at Jordan Center for Disease Control, JCDC, Jordan Cancer Society, Amman 11954, Jordan; 3 Department of Public Health and Epidemiology, Faculty of General Medicine, University of Debrecen, H-4028 Debrecen, Hungary; 4 Department of Community Health, Polytechnic College-Karbala, Al-Furat Al-Awsat Technical University, Kufa 54001, Iraq; 5 Institute of Biomedicine—iBiMED, Department of Medical Sciences, University of Aveiro, 3810-193 Aveiro, Portugal; 6 Cardiovascular R&D Centre—UnIC@RISE, Department of Surgery and Physiology, Faculty of Medicine, University of Porto, 4099-002 Porto, Portugal

**Keywords:** cancer screening, MENA and Mediterranean populations, health disparities, diaspora and refugee health, culturally adapted interventions, digital and AI-assisted screening

## Abstract

Cancer is one of the biggest health burdens for women in the Middle East and North Africa (MENA), with the incidence of breast, cervical and colorectal cancer on the rise. Although preventive measures such as the HPV vaccination and population-based screening are available, access to them remains very unequal. Women in rural, low-income and refugee communities face additional barriers, cultural stigmatisation, low health literacy, gender norms and fragile health systems, leading to delayed diagnoses and poorer outcomes. This review summarises the results of 724 peer-reviewed publications to assess the current situation of cancer screening in MENA and Mediterranean countries. The studies were classified into four dimensions: cancer type (breast, cervical, colorectal), behavioural constructs (awareness, uptake, education), vulnerability factors (e.g., migrants, refugees, low-literacy groups), and geography (indigenous MENA populations versus diaspora and Mediterranean immigrant communities). The results show large inequalities in access and participation due to fragmented policies, socio-cultural resistance and infrastructure gaps. Nevertheless, promising approaches are emerging: community-led outreach, mobile screening programmes, AI-assisted triage and culturally appropriate digital health interventions. Comparisons between the local and diaspora populations make it clear that systemic and cultural barriers persist even in well-equipped facilities. Closing the screening gap requires a culturally sensitive, digitally enabled and policy aligned approach. Key priorities include engaging religious and community leaders, promoting men’s engagement in women’s health and securing sustainable funding. With coordinated action across all sectors, MENA countries can build inclusive screening programmes that reach vulnerable women and reduce preventable cancer mortality.

## 1. Introduction

Cancer remains a major public health issue worldwide and is becoming an increasing concern for women in low- and middle-income countries. In the Middle East and North Africa (MENA), changes in population, lifestyle, and reproductive patterns are leading to a significant rise in preventable cancers, particularly breast, cervical, and colorectal cancers. The latest WHO Global Cancer Observatory (GLOBOCAN 2022) reports that breast cancer is the leading cause of cancer death among women in MENA, with approximately 140,000 new cases and over 50,000 deaths each year. Cervical cancer is less common but continues to rise, especially in countries without regular HPV vaccination programs. In 2022, more than 19,000 new cases were expected in North Africa and the Middle East. Meanwhile, colorectal cancer has become the second most common cancer among women in high-income MENA countries such as Qatar and the UAE, due to changing dietary habits and increased life expectancy [1].

Despite national efforts to expand screening, early detection remains significantly underutilized in the MENA region. According to the World Health Organization (WHO), fewer than 30% of eligible women in lower-resource MENA settings undergo regular breast cancer screening, and HPV vaccination rates remain below 10% in most countries. In contrast, Lebanon and Tunisia, which have well-organized screening systems, report lower cervical cancer mortality rates, highlighting the importance of evidence-based early detection methods [1,2]. However, substantial disparities in awareness, access, and follow-up persist, particularly among rural women, refugees, low-income populations, and MENA diaspora communities living in Western countries, where cultural and language barriers further hinder participation in routine screening programs. To ensure conceptual and geographic clarity, this review differentiates four population cohorts according to where they are assessed: (1) Indigenous MENA populations, referring to individuals residing within Middle Eastern and North African countries; (2) Mediterranean populations, assessed in countries bordering the Mediterranean Basin; (3) MENA diaspora communities living in Europe; and (4) MENA diaspora communities living in North America. This distinction allows us to interpret findings within their appropriate health-system, cultural, and policy contexts and prevents conflation between indigenous and migrant populations. Cervical cancer is becoming more common in the MENA region, even though it is largely preventable through human papillomavirus (HPV) vaccination and regular Pap tests. This increase is due to insufficient use of these preventive measures in the past. Countries such as Algeria and Lebanon have developed cost-effective models demonstrating that HPV vaccination is a sound public health investment. However, implementation remains limited because of cultural sensitivities, vaccine hesitancy, and unreliable health infrastructure. Public awareness of HPV and cervical cancer remains low, which is particularly concerning among adolescents and young women, who are a key target group for vaccination programs [3,4]. Colorectal cancer (CRC), once considered a disease primarily affecting older adults in high-income countries, is becoming increasingly common among younger populations in MENA countries. Limited public awareness, lack of structured screening programs, and the stigma associated with gastrointestinal symptoms hinder timely diagnosis and negatively impact treatment outcomes. Fecal occult blood testing (FOBT), colonoscopy, and risk stratification tools are not always accessible, and targeted interventions are needed to increase awareness and improve screening among the general public [5,6].

## 2. Methods

### 2.1. Search Strategy and Databases

A systematic literature search was conducted using Web of Science (File S1: Bibliometric data). The searches included all records published from January 2000 to December 2024. Boolean strings combined cancer type (breast, cervical, colorectal), behavioural constructs (screening uptake, awareness, education), population vulnerability (migrants, refugees, rural/low-literacy groups), and geographic terms (Middle East, North Africa, Mediterranean, Arab diaspora).

### 2.2. Eligibility Criteria

Studies were included if they met all of the following criteria:

- Study type: peer-reviewed empirical studies (quantitative, qualitative, or mixed-methods);
- Population: women or mixed-gender cohorts from indigenous MENA, Mediterranean Basin residents, or MENA diaspora populations in Europe or North America;
- Outcomes: cancer prevention, screening uptake, awareness, HPV vaccine knowledge, or early detection behaviour for breast, cervical, or colorectal cancer;
- Geographic scope: limited to the Middle East, North Africa, Southern Europe, and Western host countries with significant MENA migrant communities.

Exclusion criteria included:

- non-peer-reviewed sources (excluding specified grey literature reports),
- studies not addressing screening or early detection,
- studies outside the defined geographic scope,
- papers lacking extractable population-specific data.

### 2.3. Screening and Selection Process

Two independent reviewers screened titles and abstracts after removing duplicates. Full-text assessment was conducted using pre-established criteria. Disagreements were resolved by consensus. The final dataset comprised 724 peer-reviewed studies that met the inclusion criteria.

### 2.4. Data Collection and Synthesis

Data were extracted based on four analytical dimensions:

(1) cancer type (breast, cervical, colorectal),
(2) behavioural constructs (awareness, uptake, education),
(3) vulnerability factors (migrants, refugees, low-literacy populations),
(4) geography (indigenous vs. diaspora populations).

#### 2.4.1. Literature Search and Selection Criteria

Overall, while some MENA countries have made progress in developing cancer control strategies and implementing screening programs, the region continues to face systemic barriers, including staff shortages, fragmented data systems, gender inequalities in access to healthcare, and continued political instability in certain areas. In addition, regional disparities between urban and rural populations and between indigenous and displaced populations highlight the urgent need for a contextualized approach to cancer prevention and screening. To ensure that this review is based on comprehensive and representative evidence, we systematically analyzed a corpus of 724 peer-reviewed publications addressing cancer prevention, education and early detection in vulnerable populations in the Mediterranean, North Africa and the Middle East (MENA). The search strategy combined several dimensions: (1) cancer type of breast, cervical and colorectal cancer; (2) behavioral constructs such as screening uptake, awareness, education, invitation strategies; (3) population vulnerability, targeting migrants, low-income communities, ethnic minorities and individuals with low health literacy; and (4) geographical relevance, limiting the results to studies covering the Mediterranean, Southern Europe, North Africa or the Middle East. In addition to peer-reviewed articles, grey literature such as WHO regional cancer strategy reports and Global Breast Alliance briefings were screened and selectively incorporated where they provided meaningful insights into national cancer control planning and screening implementation in low-resource settings. To avoid geographic ambiguity, all visual representations in this review were restricted to countries in the Middle East, North Africa, and the Mediterranean Basin, excluding regions outside the defined scope.

Within this pool of literature, breast cancer proved to be the most comprehensively studied cancer type with 272 studies, followed by colorectal cancer with 118 studies and cervical cancer with 92 studies. Despite these differences, all three cancer types were well represented, providing comparative insights into cancer-specific screening pathways. Importantly, a substantial number of studies were conducted in MENA countries, including 64 on cervical cancer, 87 on breast cancer and 40 on colorectal cancer. These studies cover a wide range of contexts, from urban hospitals in Lebanon and Morocco to outreach programs in Algeria and Jordan.

The dataset also included a subset of studies looking at refugee and displaced populations (*n* = 9), reflecting the increased vulnerability of these groups to exclusion from preventive health services. Of the 724 studies analyzed, 472 (65%) were conducted in Western contexts, particularly in the United States, and focused on MENA immigrant or Arab diaspora communities. This geographic imbalance adds complexity to interpreting the findings in relation to indigenous MENA populations. These diaspora-focused studies provided important insights into how cultural, linguistic, and systemic barriers persist even in well-resourced healthcare settings. Their inclusion provides a valuable contrast to the challenges at home and illuminates how both structural and socio-cultural determinants operate across borders.

This diverse and geographically stratified evidence base not only justifies the scope of this review, but also enables a comparative analysis of barriers, interventions and outcomes for indigenous and immigrant MENA populations and for different cancer types. The following synthesis builds on this extensive literature and aims to identify both common challenges and context-specific opportunities to improve screening and uptake of cancer screening in these underrepresented groups. Drawn from 724 peer-reviewed publications, the keyword coincidence map provides a multidimensional overview of the scientific landscape addressing cancer prevention, early detection and health inequalities in Mediterranean and MENA populations. This visual representation shows the main thematic structures and links between disciplines from nutritional epidemiology to molecular oncology and health policy.

A densely populated cluster centered on the concept of the “Mediterranean diet” lies at the heart of the network. This concept is emerging as a key link in research on reducing cancer risk. Its prominence and extensive connections to terms such as “coronary heart disease,” “inflammation,” “mortality,” and “pattern” indicate a shared interest in diet as a modifiable risk factor for various non-communicable diseases, including cancer. Additional links to “food intake frequency questionnaire,” “cohort study,” and “validation” demonstrate that the methodology relies on nutritional assessment and long-term population research.

A large portion of the map, anchored by this core, focuses on breast cancer. This area is frequently associated with terms like “risk,” “expression,” “mutation,” “BRCA,” and “gene.” Here, genetic susceptibility, biomarker profiling, and tumor biology intersect in translational and clinical research. The terms “apoptosis,” “in vitro,” “activation,” and “antioxidant” highlight the significant laboratory component of the literature, particularly experimental studies investigating the anti-cancer effects of bioactive compounds (such as oleic acid, hydroxytyrosol, phenols, and olive oil) found in the Mediterranean diet.

In contrast, the cervical cancer cluster appears in the lower left corner of the map, representing the area of public health and implementation science in the literature. Key terms include “HPV,” “awareness,” “Pap smear,” “acceptance,” and “screening,” along with country-specific terms like “Lebanon,” “Tunisia,” and “Middle East.” This cluster reflects substantial efforts to understand and increase cervical cancer screening rates among vulnerable and traditionally underserved populations. Connections to terms such as “attitudes,” “education,” “adolescents,” and “self-efficacy” underscore the importance of psychosocial and behavioral factors in determining screening uptake among women in the MENA region. The map also displays a socio-political layer, featuring terms such as “barriers,” “policy,” “access,” “services,” and “health literacy,” which indicate systemic and structural issues. The terms “women,” “population,” “impact,” and “refugee” are closely linked, reflecting increasing research on disparities in healthcare access between immigrant and indigenous populations. This intersectional space underscores the importance of gender, migration status, and socioeconomic vulnerability in determining who benefits from screening initiatives. The close proximity of epidemiology, public health, and biomarker-related clusters further demonstrates the field’s shift toward more integrated and translational approaches. Additionally, terms like “machine learning,” “systems biology,” and “methylation” highlight growing interest in computational and omics-based methods for early disease detection and personalized screening models.

**Figure 1:**
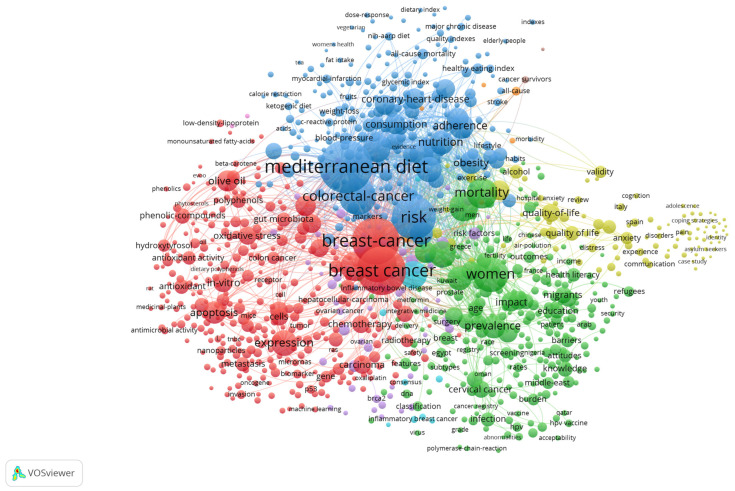
Co-occurrence network of cancer prevention and screening research across the Middle East, North Africa, and Mediterranean regions (724 studies). The map displays only the countries relevant to this review: the Middle East, North Africa, and Mediterranean Basin nations, as well as studies on MENA diaspora populations. Sub-Saharan Africa and other non-Mediterranean regions were removed to avoid geographic misinterpretation. Nodes represent author keywords, with node size reflecting frequency of occurrence. Colours denote thematic clusters that co-occur within the literature. Colours denote groups of concepts that tend to co-occur. A large blue cluster centres on the Mediterranean diet, cardiovascular risk, nutritional epidemiology, and adherence indices. A substantial purple-green cluster links breast cancer, colorectal cancer, women’s health, awareness, and screening outcomes in Mediterranean and MENA populations. The red cluster includes laboratory and field research topics such as phenolic compounds, bioactive compounds in olive oil, oxidative stress, apoptosis, and chemopreventive mechanisms. The yellow cluster highlights behavioural and psychosocial predictors, including health literacy, anxiety, migration status, refugees, and cultural barriers.

The co-occurrence map reveals extensive interconnected research, emphasizing breast and cervical cancer screening, the role of diet and lifestyle in prevention, and the need for culturally sensitive, policy-driven interventions. While 65% of the studies focus on MENA and Mediterranean diaspora populations in Western countries, the remaining evidence highlights the distinct vulnerabilities of at-risk groups within indigenous MENA and Mediterranean regions, including rural women, low-income populations, and refugees. The spatial distribution of terms illustrates the increasing integration of biomedical, behavioral, and systemic domains in advancing equitable cancer prevention. This review summarizes evidence on screening behaviors, barriers, and intervention strategies in MENA countries, focusing on breast, cervical, and colorectal cancer. By linking regional epidemiological data with findings from studies on immigrants and refugees, we aim to develop targeted, culturally sensitive public health strategies and inform future research and policy initiatives for cancer prevention. Thus, Figure 1 summarizes the main thematic trends identified in the 724 peer-reviewed studies included in this evaluation. The term co-occurrence network depicts a highly integrated research landscape, where cancer prevention, screening, nutritional epidemiology, molecular processes, and population health are closely connected. The relative sizes and interconnections of nodes indicate that a limited set of topics, including the Mediterranean diet, breast cancer, colorectal cancer, and cardiometabolic risk, dominates current research. This study reveals significant regional and demographic disparities in the evidence base. Approximately 65% of the research focuses on MENA and Mediterranean diaspora communities living in Western countries, while far fewer studies are conducted within indigenous MENA populations. This disparity shapes the thematic framework, with research agendas more closely reflecting Western health systems, screening infrastructures, and study designs than local MENA contexts. Despite this bias, several persistent patterns emerge. The Mediterranean diet plays a central role in the framework, serving as a connecting concept among cardiovascular disease, cancer risk, mortality, and preventive practices. Breast and colorectal cancers are the most prominent oncological clusters, linking epidemiological risk factors, screening practices, and molecular and experimental studies on chemopreventive mechanisms. Behavioral and psychosocial factors, such as health literacy, migratory status, cultural barriers, and access to care, consistently appear as interconnected themes, highlighting their impact on screening uptake. The co-occurrence map shows that current research on cancer prevention in Mediterranean and MENA populations is diverse but unevenly distributed. While diaspora-focused research provides important insights into cultural and structural barriers, the limited data from indigenous MENA contexts restricts direct regional generalization. These findings highlight the need for more locally focused research to supplement existing diaspora-based knowledge and to inform context-specific cancer prevention and screening practices.

**Figure 2:**
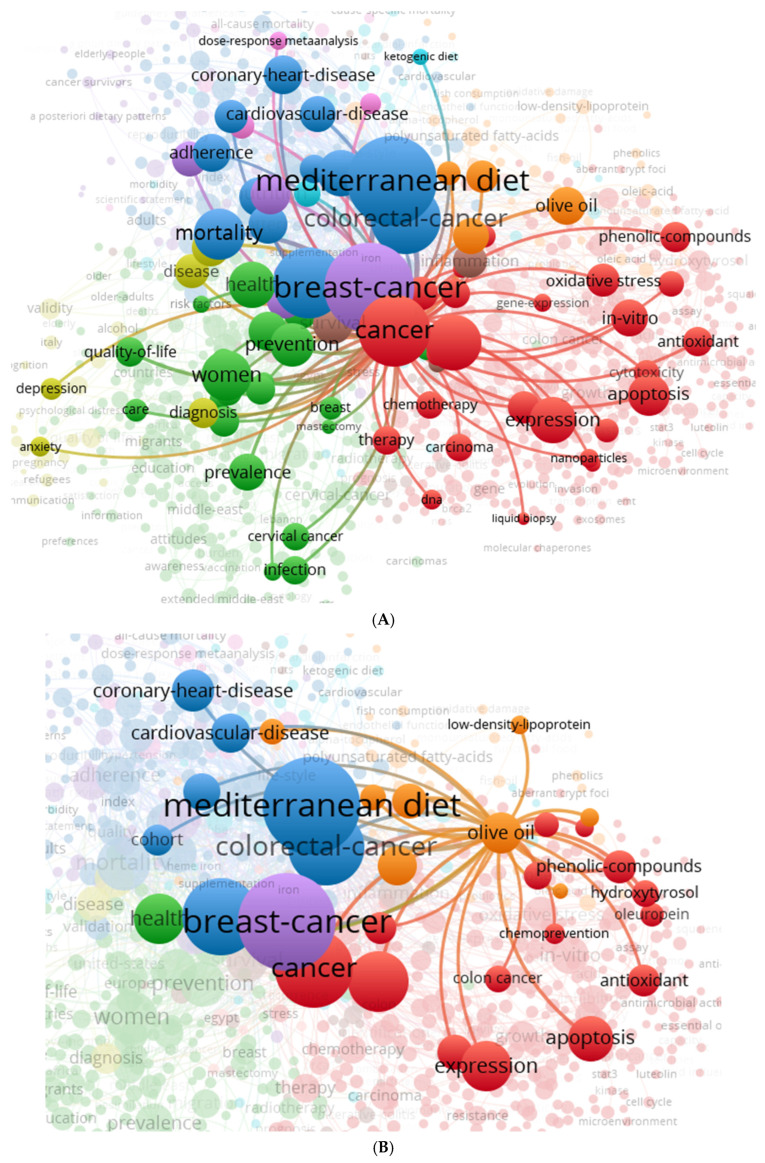

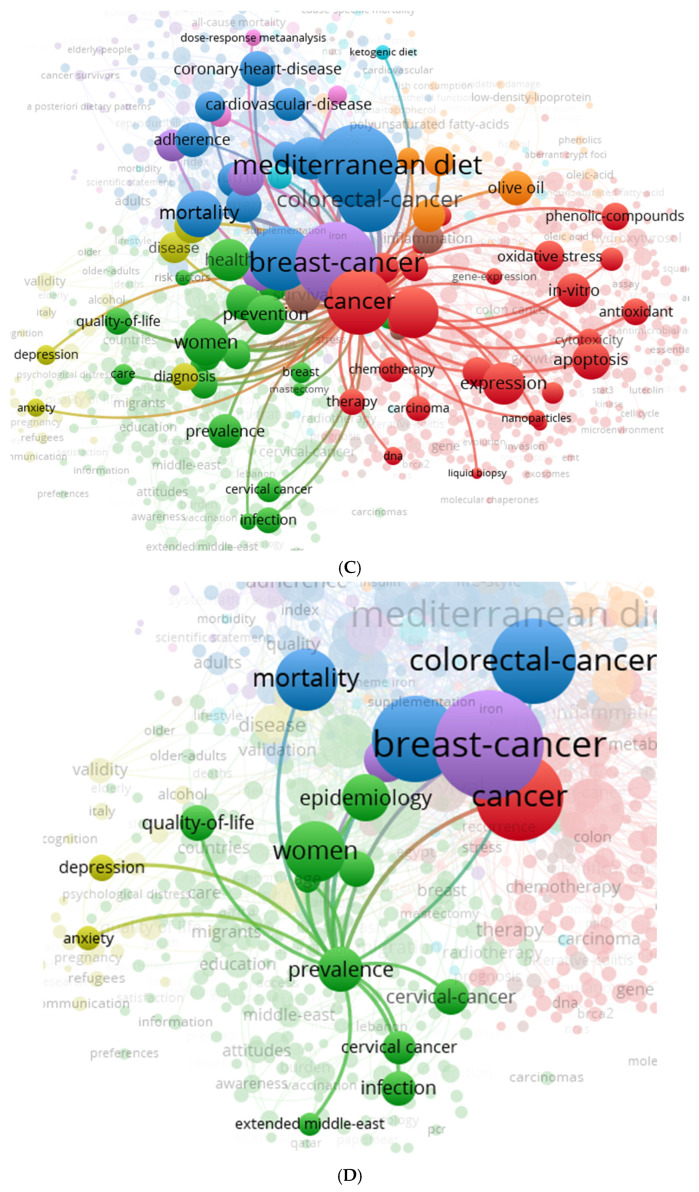
(**A**). A close-up of the Mediterranean diet–cancer interaction cluster. This panel illustrates the close links between the Mediterranean diet, breast cancer, colorectal cancer, risk, and heart disease. Strong co-occurrence links indicate that much of the research integrates nutrition, cancer epidemiology, and chronic disease prevention. Keywords such as olive oil, polyphenols, mortality, and adherence suggest a focus on modifiable dietary patterns as risk factors in Mediterranean and MENA populations. (**B**). A closer look at the olive oil and molecular mechanisms subcluster. This zoom highlights the connection between dietary bioactives (such as olive oil, phenolic compounds, and hydroxytyrosol) and experimental oncology themes (including apoptosis, oxidative stress, and gene expression). The strong link between breast and colon cancer reflects ongoing interest in chemopreventive pathways associated with Mediterranean diets. (**C**). A closer look at the cardiometabolic effects of the Mediterranean diet. This cluster connects the Mediterranean diet to coronary heart disease, cardiovascular disease, mortality, blood sugar control, and lifestyle. These associations exemplify the traditional cardioprotective literature, which is increasingly integrated with cancer screening and chronic disease prevention paradigms in the Mediterranean and MENA regions. (**D**) A close-up of the factors affecting women’s health and population-based screening. This panel focuses on women, prevalence, cervical cancer, breast cancer, infection, health literacy, migrants, refugees, and attitudes. The strong links in this cluster demonstrate how psychosocial, educational, and structural barriers influence screening uptake in both indigenous MENA populations and diaspora communities.

The discrepancies outlined show that cancer screening inequities in MENA and Mediterranean populations arise from the interplay of cultural, social, and systemic factors, rather than from isolated obstacles. Figure 2 presents a logic model to operationalize these findings for intervention design, translating identified determinants into modifiable targets for action. The framework maps upstream cultural norms and socioeconomic limitations onto intermediary health system processes, such as provider engagement, service accessibility, and continuity of care, culminating in screening outcomes. This logic model clarifies causal pathways and intervention points, providing a systematic framework for integrating culturally tailored education, community navigation, and systemic improvements. Accordingly, Figure 2 illustrates four principal thematic clusters extracted from the global co-occurrence network shown in Figure 1. The Mediterranean diet–cancer interaction cluster (Figure 2A) shows a significant co-occurrence between the Mediterranean diet and breast cancer, colorectal cancer, cardiovascular disease, and overall risk, with prevalent keywords such as olive oil, polyphenols, mortality, and dietary adherence. The olive oil and molecular mechanisms subcluster (Figure 2B) is characterized by the simultaneous presence of olive oil-derived phenolic compounds and hydroxytyrosol with oxidative stress, apoptosis, and gene expression, with breast and colorectal cancer as the main oncological keywords. The cardiometabolic cluster (Figure 2C) shows a frequent co-occurrence of the Mediterranean diet with coronary heart disease, cardiovascular disease, mortality, glycemic management, and lifestyle-related phrases, with partial overlap with cancer-related keywords. The women’s health and screening cluster (Figure 2D) includes high-frequency terms associated with women, prevalence, breast and cervical cancer, infection, and screening, along with health literacy, migration status, refugee populations, and attitudes, as observed in studies among both indigenous MENA populations and diaspora settings.

#### 2.4.2. The Importance of Early Detection and Prevention

Early detection and prevention are generally recognized as cornerstones of effective cancer control. In the context of the MENA region, these strategies are particularly important given the increasing burden of cancer and the systemic challenges that delay diagnosis and treatment. Cancers such as breast, cervical and colorectal cancers are highly detectable at an early stage, and when detected at an early stage, patient outcomes improve significantly while treatment costs and morbidity decrease substantially [7].

Screening programs such as mammography for breast cancer, Pap smears and HPV testing for cervical cancer, and fecal occult blood tests or colonoscopy for colorectal cancer have been shown to reduce mortality through early detection of precancerous lesions or asymptomatic disease. Prevention through vaccination, especially in the case of HPV-related cervical cancer, holds great potential for change [8]. The World Health Organization’s Global Strategy to Eliminate Cervical Cancer as a Public Health Problem targets 90% of girls to be fully vaccinated with the HPV vaccine by the age of 15. However, in many MENA countries, national HPV vaccination programs continue to be implemented inconsistently or not at all. Despite model studies demonstrating the cost-effectiveness of such measures, uptake is low, even in pilot programs, due to the hesitant attitude towards the vaccine and the lack of political prioritization [9,10,11,12,13].

From a public health perspective, investment in prevention and early detection provides significant benefits, both in lives saved and reduced healthcare costs. Efforts to promote culturally appropriate health education, patient navigation, and community engagement have shown promise in increasing screening rates and immunization coverage among both indigenous and diaspora populations of MENA countries [14]. Digital health technologies and artificial intelligence also offer new ways to assess and counsel individuals about their risks, especially in underserved or conflict-affected areas. Making services available to the MENA population is not sufficient; it is also necessary to build trust, cultural competence, and health literacy among the target groups. This is particularly important for vulnerable groups, such as refugees and immigrants, who may have limited access to healthcare and maintain traditional health beliefs. A shift toward evidence-based and inclusive investment in preventive oncology for both indigenous MENA populations and diaspora communities could significantly improve cancer-related outcomes across these settings.

## 3. HPV and Cervical Cancer in the MENA Population

### Knowledge Gaps, Vaccine Introduction and Cost-Effectiveness Models

Cancer remains a major burden for women across both indigenous MENA populations and diaspora communities in Western countries. Although it is largely due to persistent infection with high-risk types of human papillomavirus (HPV), the region lags behind global benchmarks in both awareness and prevention. The studies included in this review consistently point to profound gaps in knowledge among adolescents, young women and even healthcare professionals regarding HPV transmission, the link between HPV and cervical cancer, and the existence and purpose of the HPV vaccine [15,16,17].

A study of female college students in Lebanon found that only 42% had heard of HPV, and even fewer knew it was linked to cervical cancer. In Algeria and Jordan, where misconceptions about HPV and the vaccine were common, similar knowledge gaps were observed. Healthcare providers were hesitant to recommend the vaccine because they lacked sufficient information and were uncomfortable discussing sexually transmitted infections in general [18]. As a result, very few people in the MENA region are vaccinated. Most countries do not include HPV vaccination in their national immunization programs. Pilot programs exist, but they are limited to private health facilities or donor-funded initiatives. For example, in Lebanon, fewer than 10% of teenage girls have been vaccinated due to high costs and insufficient public funding. Studies of refugees in the US and Europe found even lower vaccination rates, with cultural barriers and lack of provider recommendations cited as the main reasons [19,20,21].

Despite these challenges, cost-effectiveness models offer compelling support for national implementation. A study examining the introduction of the HPV vaccine in Algeria concluded that vaccinating 10-year-old girls could be highly cost-effective, with incremental cost-effectiveness ratios (ICERs) well below WHO-recommended thresholds. These models suggest that HPV vaccination, if delivered efficiently, could significantly and cost-effectively reduce the burden of cervical cancer, especially when combined with screening measures such as Pap smears or HPV DNA testing [22,23].

School-based vaccination strategies, peer education and physician-led communication campaigns have been suggested as culturally acceptable approaches to improve uptake. However, few countries have implemented such measures nationwide. This implementation gap reflects a lack of prioritization in the public health agenda, although it is well justified from an epidemiological and economic perspective [24].

To address cervical cancer caused by HPV in the MENA region, a multi-pronged approach is necessary. This should include raising awareness, overcoming cultural barriers, and incorporating the HPV vaccine into national immunization programs. To reduce preventable deaths among women in the MENA region, cost-effective and culturally appropriate solutions based on local data and public engagement in health issues are essential [25,26,27,28,29]. Given the low uptake and high prevalence of cervical cancer, prioritizing HPV vaccination in regional public health initiatives is imperative. School-based vaccination programs for girls aged 9 to 14, tailored to local cultural contexts and supported by educational resources for parents and teachers, offer a feasible and effective strategy to mitigate the long-term impact of cancer.

## 4. Disparities and Indicators in Screening

### 4.1. Cultural, Socioeconomic, and Systemic Influences

In the MENA region, cancer screening remains underutilized, especially for breast, cervical, and colorectal cancers. This underuse results not only from limited resources and infrastructure but also from deeply rooted cultural beliefs, socioeconomic disparities, and systemic barriers within the healthcare system. Understanding these multifactorial predictors is crucial for designing interventions that increase screening rates among MENA women and reduce avoidable cancer deaths [30].

#### 4.1.1. Cultural Elements

Cultural beliefs and norms related to modesty, fatalism, and gender roles significantly influence screening behaviors. In many MENA countries, women are hesitant to undergo screening, particularly pelvic examinations or mammography, due to feelings of shame, the need for male physician consent, or fear of social stigma. Studies in Jordan and Lebanon show that religious and cultural sensitivities hinder women’s participation in cervical and breast cancer screening programs. Cultural norms also remain a major barrier to obtaining Pap smears and mammograms among MENA immigrants in Western countries, including the United States, especially when services lack linguistic or cultural appropriateness [31].

#### 4.1.2. Socioeconomic Indicators

Socioeconomic status, including educational attainment, income, and employment, is a significant predictor of screening participation. Women with low levels of education are consistently less likely to seek screening due to low health literacy, limited awareness of cancer risks, and reduced ability to navigate healthcare systems. In Lebanon and Algeria, research has shown that low-income women are markedly underrepresented in structured screening programs. Concerns about cost, especially for women without insurance or access to public healthcare, often lead them to delay or forgo screening altogether [32,33].

#### 4.1.3. Barriers in the System

Even when individuals are aware of the issue and want to participate, systemic problems within the health system create additional challenges. These include insufficient primary care infrastructure, lack of national screening programs, irregular or opportunistic screening methods, and inadequate follow-up systems.

Healthcare providers also present a significant barrier. In the MENA region, many doctors do not routinely recommend screening unless patients request it. This missed opportunity for early detection is often due to limited time, insufficient training, and discomfort discussing cancer risks with patients. Long wait times, long distances to clinics, and a shortage of female healthcare providers further reduce screening rates among women. Refugees and displaced individuals, who often live in underserved or unstable areas, face additional obstacles, including legal and financial exclusion from national health systems [34]. These factors frequently interact and reinforce one another. For example, a woman with a low income may have difficulty accessing a clinic, and even if she does, she may encounter a provider who lacks training in culturally sensitive communication, making her less likely to seek follow-up care. This intersectional perspective highlights the need for comprehensive, multilevel approaches that address individual, community, and health system determinants [35].

### 4.2. Indigenous vs. Diaspora MENA Populations

In line with the predefined cohort structure, the comparisons presented below distinguish between populations assessed in their countries of origin (indigenous MENA) and those assessed within host countries in Europe or North America (diaspora), thereby avoiding conflation of structurally distinct healthcare environments. Geographic context further complicates differences in cancer screening among the MENA population. Immigrants in host countries face distinct obstacles compared to women in their countries of origin. While both groups share cultural norms and potential knowledge gaps, immigrant women from MENA countries often encounter additional challenges related to migration status, navigating unfamiliar healthcare systems, and socio-political marginalization.

For example, studies of Arab-American and MENA immigrant women in the US show they are much less likely to be screened for cervical and breast cancer than the general population. Some reasons for this gap include limited English proficiency and lack of translated materials, distrust or unfamiliarity with Western healthcare systems, lower likelihood of receiving a doctor’s recommendation, fear of stigma, especially regarding HPV and sexually transmitted infections, and stress from immigration and competing survival priorities.

Insurance coverage is also a significant issue. Many MENA migrants in host countries lack access to healthcare, particularly those who are temporary, undocumented, or refugees. Even when services are theoretically available, cultural insensitivity and the scarcity of female or Arabic-speaking healthcare providers can discourage utilization.

Women in MENA countries, by contrast, often face a different set of challenges. These are primarily due to weak health systems, irregular screening practices, and political instability. For example, cervical screening in Lebanon and Algeria is often sporadic rather than systematic, and many rural clinics lack the staff or resources to perform Pap smears or follow-up biopsies. National screening programs, where they exist, are frequently underfunded and inconsistently implemented.

**Figure 3:**
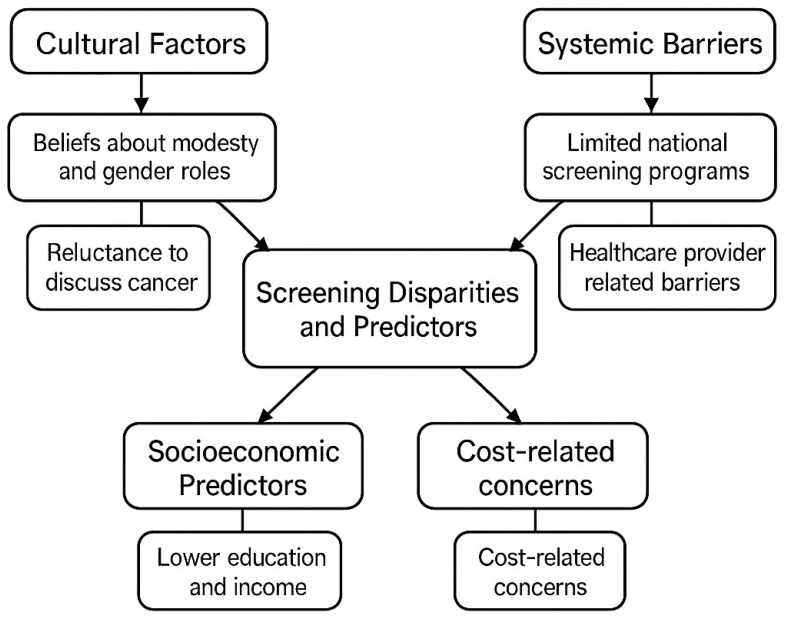
Conceptual diagram illustrating the main factors contributing to cancer screening inequalities and predictors in the MENA and Mediterranean populations. The framework highlights four main areas: cultural factors (e.g., beliefs about modesty and reluctance to discuss cancer), systemic barriers (e.g., limited national screening infrastructure and provider-related challenges), socioeconomic predictors (e.g., income, education level, and out-of-pocket costs), and access-related constraints. These interrelated elements jointly shape inequalities in awareness, access, and participation in breast, cervical, and colorectal cancer screening programs.

Interestingly, some immigrant MENA groups in high-income countries report greater exposure to public health messages and increased awareness, despite poorer socioeconomic conditions. However, this does not always translate into higher screening rates due to persistent structural and cultural barriers. This duality underscores the need for context-sensitive strategies that address the diverse realities of MENA women based on their environment. For immigrant patients, culturally appropriate patient navigation, interpreter services, and participation in public health programs are essential. For the indigenous population, priorities include strengthening primary care, educating healthcare providers, and institutionalizing screening through national campaigns and educational initiatives in schools (Figure 3).

Importantly, men’s involvement in women’s health decisions should not be overlooked. In many MENA households, men play an important role in granting permission for medical visits or allocating family resources. Engaging men through targeted awareness campaigns and their participation in community-based initiatives could improve support for women’s participation in screening programmes and counter patriarchal barriers.

## 5. Culturally Adapted Interventions

### 5.1. Case Studies of Tailored Navigation and Education Programs

Cultural stigma, rooted in ideas about modesty, sexuality, and fatalism, continues to prevent people in some MENA countries from getting screened. Low health literacy and taboos around discussing cancer or reproductive health often worsen this stigma. To address these issues, it is necessary not only to improve access to information but also to adapt messages to align with cultural values such as family well-being and religious teachings about caring for one’s health. To improve cancer screening for underserved populations, such as women from the MENA region, it is essential to ensure that services are accessible and culturally relevant. Culturally appropriate interventions are increasingly recognized as effective strategies to reduce disparities in screening uptake. These approaches focus on tailoring messages, access methods, and care delivery to the sociocultural and linguistic needs of target populations. The following models and case studies demonstrate how culturally appropriate education, patient navigation, and community engagement have successfully increased cancer screening rates among people in MENA countries and other marginalized groups.

### 5.2. Health Literacy and Culturally Sensitive Education

One of the most reliable indicators that people will not get screened is a lack of health knowledge. Culturally sensitive education programs aim to clarify and contextualize health information, often using visual aids, narrative accounts, and linguistically appropriate content. Balamou et al. (2023) [36] launched a tailored health education program in France for underserved migrant women facing low literacy and language barriers [37]. The initiative significantly improved understanding of screening recommendations for breast, cervical, and colorectal cancer by using images and simpler language. In Portugal, Gama et al. (2024) [38] applied a health literacy framework to develop educational events for African and Brazilian migrant women in Lisbon, resulting in increased awareness of cervical cancer and greater willingness to participate in screening.

Hamdiui et al. (2022) [39] developed a culturally appropriate video intervention for Turkish and Moroccan-Dutch women in the Netherlands. The video incorporated religious sensitivity, language adaptation, and trusted community voices. This approach increased positive attitudes toward cervical cancer screening and trust in public health systems. These case studies show that health education is most effective when it takes into account cultural values, religious beliefs, and preferred communication methods. Culturally appropriate framing, such as emphasizing family well-being or religious compatibility, can increase engagement, especially in MENA countries and among Muslim-majority populations.

### 5.3. Patient Navigation and Language Mediation

Patient navigation strategies provide logistical and emotional support to help people through complex or unfamiliar medical procedures. They are particularly effective with immigrants who have limited familiarity with the system.

In Italy, Palazzi et al. (2016) [40] used telephone-based call-back systems combined with language mediation in the city of Cesena. This program successfully improved migrant women’s participation in cervical cancer screening by bridging communication gaps and easing administrative challenges. In Spain, several programs in Barcelona [41] have shown that personalized invitation letters, telephone follow-ups and the involvement of community mediators increase rates of cervical and breast cancer screening in neighborhoods with a high proportion of migrants [42].

The Marseille study by Piana et al. (2007) [43] went a step further by offering free screening and holding information sessions in the community to build trust and reduce anxiety. The inclusion of culturally relevant facilitators and peer speakers created a safe environment for discussion, which in turn encouraged participation.

In each of these cases, navigation efforts were more effective when they combined practical support (scheduling, reminders) with cultural mediation and emotional reassurance, especially in communities where healthcare is perceived as intimidating or inaccessible.

### 5.4. Peer Educator and Community-Led Models

Community involvement is essential for sustainable behavior change. Interventions in which local women are trained as peer educators or community health workers have proven to be particularly effective in promoting cancer prevention.

In Lorraine, France, Cambon et al. introduced a participatory, peer-led program targeting precarious women from immigrant communities. These peer educators, who often have the same cultural background as the target group, fostered trust and credibility so that the message was better received. Similarly, in Marseille, Legendre et al. (2024) [44] used trained health mediators from local communities to provide targeted education. This strategy led to increased uptake of breast, cervical and colorectal cancer screening, especially among women with limited education or health literacy. Such bottom-up models empower communities, increase local capacity and reinforce shared norms around screening. They also counteract the historical mistrust of medicine that is widespread among migrants and refugees. In addition to peer educators, the involvement of local religious leaders and trusted community influencers can further increase the uptake and reach of cancer prevention interventions. In many MENA communities, imams, spiritual leaders and respected elders play a critical role in shaping health behaviours and social norms. Training these individuals to deliver culturally sensitive health messages in mosques, at community gatherings or via social media could strengthen advocacy and reduce stigma, particularly around issues such as HPV vaccination and cervical screening. Peer educator models have significant potential for use in low-resource settings in the MENA region. Their success relies on engaging trusted community members to address gaps in language, culture, and education. This makes them well suited to areas where health infrastructure is weak or underfunded. With tailored training, integration into primary care outreach initiatives, and collaboration with local NGOs and religious leaders, these models could be effectively expanded to increase cancer screening participation among underserved populations in both rural and urban areas. Mobile health platforms and WhatsApp-based peer groups offer promising opportunities for cost-effective information dissemination and follow-up in settings with limited formal healthcare access.

### 5.5. Integration into Primary Care and Support at System Level

Cultural adaptation alone is not enough if it is not integrated into the wider healthcare system. Effective interventions need to fit into primary care workflows, leverage relationships with providers and ensure continuity of care.

In a French cluster-randomized controlled trial, Durand et al. (2021) [45] trained GPs to provide visual educational materials tailored to adults with low literacy skills. This simple, scalable intervention led to a significant increase in colorectal cancer screening rates in the intervention clinics. Importantly, GPs were perceived as a credible and accessible source of health advice, especially in populations that previously lacked confidence in the medical system. The success of this approach underscores the need to train healthcare professionals in cultural competence and to develop standardized tools to facilitate risk communication, motivational interviewing, and shared decision making among diverse patient populations.

Taken together, these case studies provide tangible evidence that culturally appropriate interventions, when implemented at multiple levels and with community involvement, can significantly reduce cancer screening disparities in MENA and other vulnerable populations. Education, navigation and community participation, when embedded in primary care and public health infrastructures, are not only effective but also necessary to achieve equitable cancer prevention outcomes. Policy makers and practitioners need to prioritize these strategies in national screening programs, especially in areas of high ethnic diversity, migration and social exclusion [46]. Scaling primary care-based cancer screening programmes in resource-limited MENA contexts faces numerous challenges. Financial barriers persist where screening lacks public funding or where healthcare spending prioritises curative over preventive care. Structural issues, such as insufficient staff, lack of electronic health records, and underdeveloped referral pathways, hinder continuity of care and follow-up. Political instability and fragmented governance in some MENA countries further undermine programme sustainability. Addressing these barriers requires simultaneous investment, task-shifting (such as involving nurses or community health workers), and integrating low-cost screening technologies into existing primary care systems.

## 6. Challenges in the Implementation of Health Policy

Although the burden of cancer is increasingly recognized in MENA countries, the translation of evidence into effective, equitable screening programs remains uneven. Numerous structural, systemic and political challenges have hampered the development and implementation of national screening strategies for breast, cervical and colorectal cancer. These challenges are exacerbated by competing health priorities, conflict-related instability and underfunded health systems [47]. Thus, several grey literature reports from international bodies such as the Global Breast Cancer Initiative have highlighted similar barriers to screening implementation in MENA contexts, including uneven resource distribution, limited national registries, and underfunding of community-based outreach programs.

### 6.1. Infrastructural and Systemic Gaps

Basic health infrastructure remains a fundamental barrier to cancer prevention in much of the MENA region. While some high-income countries such as the Gulf States have the resources to implement advanced screening programs, lower and middle-income countries often lack essential components such as:

- Trained primary care providers and gynecologists
- Laboratory services for cytology or HPV DNA testing
- Colposcopy and biopsy follow-up services
- Electronic data systems for screening reminders and follow-up

A review of cervical cancer screening services in Algeria and Jordan, for example, found that many public facilities are limited to opportunistic Pap testing, with no standardized protocols or follow-up pathways. Screening is often not integrated into routine primary care, and rural clinics in particular are not adequately equipped to provide early detection and referral.

Even in urban areas where services do exist, the fragmentation of care between public, private and NGO-run services lead to inefficiencies and missed opportunities. The lack of centralized data makes monitoring and quality control at the national level difficult. These gaps are particularly acute in conflict zones, where both healthcare infrastructure and data systems are often lacking. There is an urgent need for disaggregated, country-specific data, especially in fragile situations, to inform customized action. Without reliable epidemiological and service delivery data, planning and evaluating cancer screening programs remains a challenge in many parts of the MENA region.

### 6.2. Access to Healthcare and Equal Opportunities

Geographic, economic and social barriers prevent many women, especially those in rural or marginalized urban communities, from accessing preventive healthcare. Out-of-pocket costs, transportation barriers and lack of paid time off from work disproportionately affect low-income women. In some countries, screening is not covered by general health plans, leaving many women to rely on sporadic NGO programs or private providers.

In addition, refugees and displaced persons—a growing population group in conflict areas such as Lebanon, Jordan and Palestine—are often left out of state health provision altogether. Their access to preventive services is highly dependent on external assistance and is often not continuous or comprehensive.

Social factors, such as women’s lack of autonomy in health decisions and the stigmatization of cancer, further inhibit the uptake of screening. Cultural taboos around pelvic exams and fear of cancer diagnoses were cited as psychological barriers to screening uptake, even in facilities where services are physically available.

### 6.3. Political Instability and Competing Health Priorities

Many MENA countries have delayed investing in public health due to political and economic challenges. Chronic underfunding, civil unrest, and shifting donor priorities often result in cancer screening being postponed in favor of more urgent issues such as maternal and child health, infectious diseases, and emergency care.

For example, the recent economic collapse in Lebanon has significantly affected public health services, making it difficult to sustain screening programs. In areas like Syria, Iraq, and parts of Libya where conflict persists, basic medical care has ceased, and cancer screening is nearly nonexistent. Even in more stable countries, health ministries may prioritize diseases with more immediate short-term impacts or those that attract greater international donor interest [48]. Although there is evidence that HPV vaccination and cervical cancer screening are cost-effective, policymakers are often slow to implement changes. Progress is hindered by a lack of political will, poor coordination between ministries (such as health and education), and weak advocacy platforms [49,50].

Implementing cancer screening policies in the MENA region is extremely challenging due to infrastructure problems, unequal access, and political fragmentation. To address these gaps, regional strategies should prioritize:

- Integrating screening into routine primary care
- Expanding services in rural areas
- Ensuring long-term funding and policy stability
- Collaborating across sectors to align health, education, and community development

Without these measures, even the most effective interventions will struggle to significantly reduce cancer disparities in MENA countries.

## 7. Possibilities and Future Directions

### 7.1. AI-Supported Education, Digital Health Literacy and Policy Recommendations

While cancer prevention in the MENA region faces numerous systemic and socio-cultural barriers, there are some promising opportunities at the intersection of technology, health education and policy reform. By leveraging innovations in artificial intelligence (AI), digital communication platforms, and evidence-based policymaking, MENA countries can create scalable, culturally sensitive solutions to improve cancer screening, particularly among women and underserved groups [51,52]. Several countries in the MENA and Mediterranean regions have begun testing AI-powered cancer screening systems. These are real-world examples of digital innovation. For instance, the United Arab Emirates is using AI-based mammography platforms in its national breast cancer screening programs. The SEHA health system in Abu Dhabi reports that this has accelerated the time to diagnosis. In Saudi Arabia, histopathological image analysis is being used to help detect colorectal cancer early with artificial intelligence. Egypt has also started using AI to assist with diagnosis in public hospitals to detect cervical cancer more quickly, especially in under-resourced areas. These efforts demonstrate a growing push in the region to use AI for cancer screening and early diagnosis that can be scaled up and delivered at low cost.

### 7.2. AI-Powered Outreach and Risk Stratification

Artificial intelligence (AI) and machine learning (ML) offer significant potential for personalizing cancer screening strategies and optimizing resource allocation in low-resource settings. AI-driven models can help:

- Identify high-risk individuals using electronic health records or self-reported data with minimal input
- Automate reminder systems tailored to language, literacy or screening history
- Prediction of non-compliance and suggestions for personalized nudges or navigator support

In high-income MENA countries (e.g., UAE, Saudi Arabia), innovative digital health ecosystems are already exploring AI applications in public health. Extending these tools to screening algorithms and culturally adapted chatbot interfaces (in Arabic, Tamazight, etc.) could help bridge the gap between awareness and participation, especially among digitally connected youth and urban women.

However, equal treatment must be ensured: prediction algorithms must be transparent, must not contain biases against minorities or migrants, and must be embedded in a framework that protects privacy. Investment in open, region-specific datasets and collaboration between health authorities and data scientists will be critical to the utility of AI in real-world screening programs in MENA countries.

### 7.3. Digital Health Literacy and Mobile Health Interventions

Cell phones are widely used across the MENA region, including in resource-poor and refugee settings. This presents a great opportunity for mobile health (mHealth) interventions to support cancer prevention [53,54]. Examples include:

- SMS-based appointment reminders and health alerts
- Video education via WhatsApp in local dialects
- QR codes that lead to visual explanations of Pap smears or mammograms
- Teleconsultation apps with culturally competent providers

Improving digital health literacy, i.e., the ability to access, understand and act on digital health information, is critical. Community-based training initiatives, school-based digital health programs for youth, and partnerships with women’s cooperatives or mosques can play a central role in bridging the digital divide [54]. Digital tools must also be developed together with the target groups to ensure their usability, relevance and trust. Pilot projects in Lebanon and Morocco have shown that visual and voice-activated mobile applications can support informed screening decisions even in populations with low literacy levels.

### 7.4. Suggestions for Policies to Support Fair and Long-Term Growth

Policy changes are necessary for sustained improvements in access to and use of screening. Based on the evidence summarized in this review, the following policy recommendations are proposed for MENA governments and stakeholders:

1) Integrate screening for cervical, breast, and colorectal cancer into maternal and child health services.
  - Leverage vaccination campaigns (such as HPV) to coordinate with other interventions.
2) Launch cost-effective national HPV vaccination programs. Use school-based models to reach girls before puberty. Seek support from Gavi or WHO for vaccine procurement and distribution.
3) Institutionalize community health workers and mediators.
  - Train culturally competent navigators to serve in clinics, refugee centers, and community settings.
  - Provide certification, fair compensation, and digital tools to expand their reach.
4) Ensure providers receive training that is both gender-sensitive and culturally sensitive. This will address provider bias and ensure patients are treated with respect and understanding. Incorporate cross-cultural communication skills into medical and nursing education.
5) Strengthen data systems and evaluation frameworks. Use AI and digital registries to monitor participation, follow-up, and outcomes. Disaggregate reports by gender, location, and migration status.
6) Promote cross-border collaboration and knowledge sharing by establishing regional consortia (such as the Maghreb-Eastern Med Cancer Screening Network) and facilitating the exchange of best practices, digital tools, and joint procurement agreements.
7) Ensure sustainable funding mechanisms for cancer screening programs.
  - Allocate long-term budget lines in national health plans.
  - Explore public–private partnerships and international donor engagement to maintain program continuity, especially in low-income and conflict-affected countries.

Combining new technologies with cultural sensitivity and a practical public health approach is essential for cancer prevention in MENA countries. By adopting AI, building digital health capacity, and developing inclusive policy frameworks, MENA countries can close persistent gaps in cancer screening and set an example for others in advancing global health equity. Political will, sustained investment, and collaboration are all critical to achieving this transformation.

## 8. Conclusions

Deep-seated structural, economic, and socio-cultural obstacles continue to impede cancer prevention and screening in the MENA region. Although effective tools such as HPV vaccines, mammograms, and fecal occult blood tests exist, their use remains limited, especially among vulnerable groups like migrants, low-income women, and displaced people. Fragmented implementation of screening programs, low awareness, and poor coordination between ministries continue to slow progress. However, the review indicates promising ways forward. Culturally appropriate interventions, including peer navigator models, multilingual communication strategies, school-based education, and policy advocacy, have been shown to increase participation. Additionally, integrating digital platforms and artificial intelligence into early detection strategies offers scalable and cost-effective solutions, particularly in settings with limited resources or political instability.

To close existing gaps, a combination of evidence-based policy, community empowerment, and new technologies is required. Public health, education, civil society, and digital health stakeholders must collaborate to build a robust screening infrastructure, ensure equitable access, and adapt strategies to the diverse cultural and socio-economic contexts of the MENA region. Only through such comprehensive efforts can all women, regardless of background or income, access life-saving cancer screening services.

## Data Availability

No new data were created or analyzed in this study.

## Supplementary Materials

The following supporting information can be downloaded at: https://www.mdpi.com/article/10.3390/diseases14010010/s1, File S1: Bibliometric data.
