# Supplementary material for: Cancer Screening and Prevention in MENA and Mediterranean Populations: A Multi-Level Analysis of Barriers, Knowledge Gaps, and Interventions Across Indigenous and Diaspora Communities
Source: Diseases. 2025 Dec 28;14(1):10. doi: 10.3390/diseases14010010 (PMC12839870; doi:10.3390/diseases14010010)
Supplement: Supplementary file 1 [file diseases-14-00010-s001.zip › diseases-3989750-supplementary.pdf]

PT AU BA BE GP AF BF CA TI SO SE BS LA DT CT CY CL SP HO DE ID AB C1 C3 RP EM RI OI FU FP FX CR NR TC Z9 U1 U2 PU PI PA SN EI BN J9 JI PD PY VL IS PN SU SI MA BP EP AR DI DL D2 EA PG WC WE SC GA PM OA HC HP DA UT

J Kim, JJ; Sharma, M; O'Shea, M; Sweet, S; Diaz, M; Sancho-Garnier, H; Seoud, M Kim, Jane J.; Sharma, Monisha; O'Shea, Meredith; Sweet, Steven; Diaz, Mireia; Sancho-Garnier, Helene; Seoud, Muhieddine Model-Based Impact and Cost-Effectiveness of Cervical Cancer Prevention in the Extended Middle East and North Africa (EMENA) VACCINE English Article HPV; Mathematical model; Cost-effectiveness; Extended Middle East; North Africa HUMAN-PAPILLOMAVIRUS; HPV VACCINE; MATHEMATICAL-MODELS; ECONOMIC-IMPACT; LATIN-AMERICA; HEALTH; KNOWLEDGE; AWARENESS; STUDENTS; WOMEN To date, no studies have evaluated the cost-effectiveness of human papillomavirus (HPV) vaccination in countries in the Extended Middle East and North Africa (EMENA) region. We synthesized population and epidemiologic data for 20 EMENA countries using a model-based approach to estimate averted cervical cancer cases and deaths, disability-adjusted life years (DALYs) and cost-effectiveness ratios (I$ [international dollars] per DALY averted) associated with HPV vaccination of pre-adolescent girls. We utilized additional epiderniologic data from Algeria, Lebanon, and Turkey to evaluate select cervical cancer screening strategies either alone or in combination with vaccination. Results showed that pre-adolescent vaccination of five consecutive birth cohorts at 70% coverage has the potential to prevent over 180,000 cervical cancer cases. Cases averted varied by country, largely due to differences in cancer burden and population size; 69% of cases averted occurred in the three GAVI-eligible countries in EMENA. Despite the low cervical cancer incidence in EMENA, we found that HPV vaccination was cost-effective using a threshold of each country's gross domestic product per capita (a common metric for evaluating cost-effectiveness) in all but five countries at a cost per vaccinated girl of I$25 ($5 per dose). However, cost-effectiveness diminished with increasing vaccine cost; at a cost of I$200 per vaccinated girl, HPV vaccination was cost-effective in only five countries. When the cost per vaccinated girl exceeded I$50 in Lebanon and Turkey and I$150 in Algeria, screening alone was most attractive. We identified opportunities to improve upon current national screening guidelines, involving less frequent screening every 3-5 years. While pre-adolescent HPV vaccination promises to be a cost-effective strategy in most EMENA countries at low costs, decision makers will need to consider many other factors, such as affordability, acceptability, feasibility, and competing health priorities, when making decisions about cervical cancer prevention. This article forms part of a regional report entitled "Comprehensive Control of HPV Infections and Related Diseases in the Extended Middle East and North Africa Region" Vaccine Volume 31, Supplement 6, 2013. Updates of the progress in the field are presented in a separate monograph entitled "Comprehensive Control of HPV Infections and Related Diseases" Vaccine Volume 30, Supplement 5, 2012. (C) 2013 Elsevier Ltd. All rights reserved.

[Kim, Jane J.; Sharma, Monisha; O'Shea, Meredith; Sweet,

Steven] Harvard Univ, Sch Publ Hlth, Dept Hlth Policy & Management, Ctr Hlth Decis Sci, Boston, MA 02115 USA; [Diaz, Mireia] Catalan Inst Oncol ICO, Canc Epidemiol Res Program, Unit Infect & Canc UNIC, Barcelona, Spain; [Sancho-Garnier, Helene] Univ Montpellier, CRLC Val dAurelle, F-34059 Montpellier, France; [Seoud, Muhieddine] Amer Univ Beirut, Med Ctr, Dept Obstet & Gynecol, Beirut, Lebanon Harvard University; Harvard T.H. Chan School of Public Health; Institut Catala d'Oncologia; UNICANCER; Universite de Montpellier; Institut Regional du Cancer Montpellier / Val d'Aurelle (ICM); American University of Beirut Kim, JJ (corresponding author), Harvard Univ, Sch Publ Hlth, Dept Hlth Policy & Management, Ctr Hlth Decis Sci, Boston, MA 02115 USA.
jkim@hsph.harvard.edu Diaz, Mireia/H-1593-2015 Diaz, Mireia/0000-0001-9360-4548 Bill and Melinda Gates Foundation [30505]; Instituto de Salud Carlos III (Spanish Government) [RCESPC03/09, RTICESP C03/10, RTIC RD06/0020/0095, RD12/0036/0056, CIBERESP]; Agencia de Gestio d'Ajuts Universitaris i de Recerca-Generalitat de Catalunya (Catalonian Government) [AGAUR2005SGR00695, AGAUR 2009SGR126] Bill and Melinda Gates Foundation(Bill & Melinda Gates Foundation); Instituto de Salud Carlos III (Spanish Government)(Instituto de Salud Carlos III); Agencia de Gestio d'Ajuts Universitaris i de Recerca-Generalitat de Catalunya (Catalonian Government) This study was funded by the Bill and Melinda Gates Foundation (30505), who had no role in the design and conduct of the study; collection, management, analysis, and interpretation of the data; or preparation, review, or approval of the report. The authors gratefully acknowledge the Global HPV and Cervical Cancer Modeling Team at the Center for Health Decision Science at the Harvard School of Public Health. The work of Mireia Diaz and the Unit of Infections and Cancer regarding this chapter was partially supported by public grants from the Instituto de Salud Carlos III (Spanish Government) (grants RCESPC03/09, RTICESP C03/10, RTIC RD06/0020/0095, RD12/0036/0056 and CIBERESP) and from the Agencia de Gestio d'Ajuts Universitaris i de Recerca-Generalitat de Catalunya (Catalonian Government) (grants AGAUR2005SGR00695 and AGAUR 2009SGR126), who had no role in data collection, analysis or interpretation of results. 51 31 33 0 23
ELSEVIER SCI LTD OXFORD THE BOULEVARD, LANGFORD LANE, KIDLINGTON, OXFORD OX5 1GB, OXON, ENGLAND 0264-410X 1873-2518
VACCINE Vaccine DEC 30 2013 31
6 G65 G77
10.1016/j.vaccine.2012.06.096
http://dx.doi.org/10.1016/j.vaccine.2012.06.096
13 Immunology; Medicine, Research & Experimental
Science Citation Index Expanded (SCI-EXPANDED)
Immunology; Research & Experimental Medicine 289LR 24331822
2025-06-24 WOS:000329684500008
J Koutrakou, P; Trigoni, M; Sarafis, P; Tzavara, C; Nikolentzos, A; Vassilakou, T; Sergentanis, TN
Koutrakou, Panagiota; Trigoni, Maria; Sarafis, Pavlos; Tzavara, Chara; Nikolentzos, Athanasios; Vassilakou, Tonia; Sergentanis, Theodoros N. Knowledge and Perceptions of Greek Students about Human Papilloma Virus, Vaccination and Cervical Cancer Screening CHILDREN-BASEL English
Article health literacy; youth; adolescents; cervical cancer; vaccination UNIVERSITY-STUDENTS; NURSING-STUDENTS; HPV INFECTION; ACCEPTANCE; AWARENESS;

PREVENTION; BELIEFS; RISK INTRODUCTION: Human papillomavirus (HPV) is the main cause of cervical cancer; the level of HPV-related knowledge among young students remains however questionable. The purpose of the present study was to investigate knowledge pertaining to HPV, cervical cancer screening, and vaccination among students in the Nursing Department and Department of Social Work of the Hellenic Mediterranean University of Crete, Greece. METHODS: This was a questionnaire-based, cross-sectional study involving 371 first-year and third-year students of the two Departments. Multivariate linear and logistic regression analysis was performed to examine factors associated with knowledge related to HPV, cervical cancer screening, and HPV vaccination. RESULTS: Only 22.1% of students knew all the ways of HPV transmission and only 5.9% knew the whole spectrum of cancers that HPV could cause. The vaccination rate for HPV was 33.7%. The majority of students used the Internet as the main source of information (62.3%). Students' sociodemographic characteristics, including age, marital status, and Department of studies were associated with knowledge about HPV. CONCLUSIONS: The present study highlights knowledge gaps and indicates the need for thorough health education strategies on HPV, targeting families and young people. [Koutrakou, Panagiota; Trigoni, Maria; Sarafis, Pavlos; Nikolentzos, Athanasios; Sergentanis, Theodoros N.] Hellen Open Univ, Sch Social Sci, Patras 26335, Greece; [Sarafis, Pavlos] Univ Thessaly, Gen Dept Lamia, Lamia 35100, Greece; [Tzavara, Chara] Natl & Kapodistrian Univ Athens, Ctr Hlth Serv Res, Med Sch, Dept Hyg Epidemiol & Med Stat, Athens 11527, Greece; [Vassilakou, Tonia; Sergentanis, Theodoros N.] Univ West Attica, Sch Publ Hlth, Dept Publ Hlth Pol, Athens 11521, Greece
Hellenic Open University; National & Kapodistrian University of Athens; University of West Attica Sergentanis, TN (corresponding author), Hellen Open Univ, Sch Social Sci, Patras 26335, Greece.; Sergentanis, TN (corresponding author), Univ West Attica, Sch Publ Hlth, Dept Publ Hlth Pol, Athens 11521, Greece.
tsergentanis@uniwa.gr Sergentanis, Theodoros/AAD-8303-2019; Vassilakou, Nair Tonia/R-6967-2017 Vassilakou, Nair Tonia/0000-0002-9993-1897; Sergentanis, Theodoros N./0000-0002-9355-5528; Sarafis, Pavlos/0000-0001-9967-5152
29 2 2 2 7 MDPI BASEL ST ALBAN-ANLAGE 66, CH-4052 BASEL, SWITZERLAND 2227-9067 CHILDREN-BASEL Children-Basel DEC 2022 9 12
1807 10.3390/children9121807
http://dx.doi.org/10.3390/children9121807 11
Pediatrics Science Citation Index Expanded (SCI-EXPANDED)
Pediatrics 7D6UR 36553251 gold, Green Published
2025-06-24 WOS:000900623300001
J Harper, DM; Tariq, M; Alhawli, A; Syed, N; Patel, MR; Resnicow, K Harper, Diane M.; Tariq, Madiha; Alhawli, Asraa; Syed, Nadia; Patel, Minal R.; Resnicow, Ken
Comparative predictors for cervical cancer screening in Southeast Michigan for Middle Eastern-North African (MENA), White and African American/black women PREVENTIVE MEDICINE
English Article
Middle East-North African (MENA); Health disparities; cancer screening; Cervical cancer screening; Female; Religion ARAB AMERICANS; ASSOCIATION; BREAST; BLACK The cervical cancer screening behaviors of Arab American women are not adequately understood, in part because Middle Eastern North African (MENA)

descent is not a US Census category. Others have shown decreased cervical cancer screening in this race of women. Our primary aim is to evaluate the predictors of cervical cancer screening among MENA, White and Black women of southeast Michigan. A community-wide health survey reached MENA, White and Black populations asking self-report questions about health behaviors, attitudes, and medical history. Cervical cancer screening was considered up-to-date if it was reported to have occurred within the past three years. Survey responses were limited to women 30-65 years old and were analyzed with inferential and logistic regression models to determine risk factors for cervical cancer screening. Overall, 78% reported cervical cancer screening within the past three years. MENA women screened less often if time in the US was less than ten years (aOR 0.24 (0.05, 0.76)) compared to more than ten years and if single (aOR 0.27 (0.07, 0.97)) compared to married. Religion was not associated with screening in any study population. Those of all races without insurance screened significantly less often than those with insurance. The barriers to cervical cancer screening among MENA women are not associated with religion but instead with lack of insurance and length of time residing in the US. [Harper, Diane M.] Univ Michigan, Dept Family Med, Sch Med, Ann Arbor, MI 48109 USA; [Harper, Diane M.] Univ Michigan, Sch Med, Dept Obstet & Gynecol, Ann Arbor, MI 48109 USA; [Harper, Diane M.] Univ Michigan, Coll Literature Sci & Arts, Dept Womens Studies, Ann Arbor, MI 48109 USA; [Tariq, Madiha; Alhawli, Asraa; Syed, Nadia] Arab Community Ctr Econ & Social Serv ACCESS, 2651 Saulino Court, Dearborn, MI 48120 USA; [Patel, Minal R.; Resnicow, Ken] Univ Michigan, Sch Publ Hlth, Dept Hlth Behav & Hlth Educ, Ann Arbor, MI 48109 USA; [Resnicow, Ken] Univ Michigan, Rogel Canc Ctr, Outreach & Hlth Dispar Res, Ann Arbor, MI 48109 USA; [Resnicow, Ken] Univ Michigan, Sch Publ Hlth, Ctr Hlth Communicat Res, Ann Arbor, MI 48109 USAUniversity of Michigan System; University of Michigan; University of Michigan System; University of Michigan; University of Michigan System; University of Michigan; University of Michigan System; University of Michigan; University of Michigan System; University of Michigan; University of Michigan System; University of Michigan Harper, DM (corresponding author), 1018 Fuller St, Ann Arbor, MI 48104 USA. harperdi@med.umich.edu; mtariq@accesscommunity.org; aalhawili@accesscommunity.org; nsyed@accesscommunity.org; minalrp@umich.edu; kresnic@umich.edu HARPER, DIANE/I-2180-2014
NIH through the Michigan Institute for Clinical and Health Research [UL1TR002240]; University of Michigan Rogel Cancer Center [P30CA046592-29-S4]; National Cancer Institute [P30CA046592] Funding Source: NIH RePORTER; National Center for Advancing Translational Sciences [UL1TR002240] Funding Source: NIH RePORTER NIH through the Michigan Institute for Clinical and Health Research; University of Michigan Rogel Cancer Center; National Cancer Institute(United States Department of Health & Human ServicesNational Institutes of Health (NIH) - USANIH National Cancer Institute (NCI)); National Center for Advancing Translational Sciences(United States Department of Health & Human ServicesNational Institutes of Health (NIH) - USANIH National Center for Advancing Translational Sciences (NCATS)) This work was supported by NIH through the Michigan Institute for Clinical and Health Research UL1TR002240 and The University of Michigan Rogel Cancer Center P30CA046592-29-S4 grants 40 9 9 0 1 ACADEMIC PRESS INC ELSEVIER SCIENCE SAN DIEGO

525 B ST, STE 1900, SAN DIEGO, CA 92101-4495 USA 0091-7435 1096-0260 PREV MED Prev. Med. JUN 2022 159 107054
10.1016/j.ypmed.2022.107054
http://dx.doi.org/10.1016/j.ypmed.2022.107054 MAY 2022 12 Public, Environmental & Occupational Health; Medicine, General & Internal Science Citation Index Expanded (SCI-EXPANDED) Public, Environmental & Occupational Health; General & Internal Medicine 1L3DZ 35460718 2025-06-24 WOS:000799173100021
J Harper, DM; Plegue, M; Sen, A; Gorin, SS; Jimbo, M; Patel, MR; Resnicow, K Harper, Diane M.; Plegue, Melissa; Sen, Ananda; Gorin, Sherri Sheinfeld; Jimbo, Mas; Patel, Minal R.; Resnicow, Ken Predictors of screening for cervical and colorectal cancer in women 50-65 years old in a multi-ethnic population PREVENTIVE MEDICINE REPORTS
English Article Cervical cancer screening; Colorectal cancer screening; Females; 50-65 years old; Middle Eastern-North American (MENA); ethnicity
UNITED-STATES; MEN; ADHERENCE Middle Eastern/North Africa (MENA) women are often not identified in cancer screening studies. The aim of this study was to determine the rates and predictors of cervical and colorectal cancer (CRC) screening for women 50-65 years of three race/ethnicities. White, black and MENA women of Southeast Michigan were surveyed once in 2019 for demographics, health care barriers, chronic diseases, and cancer screening updates using in-person, telephone, and online methods. Descriptive statistics and multivariate multinomial logistic regression were used to predict up-to-date colorectal cancer and cervical cancer screening. All analyses were adjusted by local population weights for comparability and generalizability. 394 women participated with 54% up-to-date on both screenings, 21% for cervical cancer screening alone, and 12% for CRC alone. Women more likely to be up-to-date for only cervical cancer screening compared to both cancer screens are younger (aOR 0.83 (95% CI 0.76, 0.92), are of MENA descent (7.97 (2.46, 25.76) and have no insurance (9.41 (1.07, 82.92). There are no predictors for women being up-to-date for CRC screening alone compared to both screens. Among women 50-65 years old, being up-to-date in cervical cancer screening is unrelated to being up-to-date for CRC screening. Compared to Healthy People 2020, there are significant gaps in cervical and CRC screening among women 50-65 years old of all races, but particularly among women of MENA descent who are even less likely to have CRC screening than cervical cancer screening.
[Harper, Diane M.; Plegue, Melissa; Sen, Ananda; Gorin, Sherri Sheinfeld; Jimbo, Mas] Univ Michigan, Dept Family Med, Ann Arbor, MI 48109 USA; [Harper, Diane M.] Univ Michigan, Dept Obstet & Gynecol, Ann Arbor, MI 48109 USA; [Harper, Diane M.] Univ Michigan, Dept Womens & Gender Studies, Ann Arbor, MI 48109 USA; [Sen, Ananda] Univ Michigan, Dept Biostat, Ann Arbor, MI 48109 USA; [Patel, Minal R.; Resnicow, Ken] Univ Michigan, Dept Hlth Behav & Hlth Educ, Ann Arbor, MI 48109 USA University of Michigan System; University of Michigan; University of Michigan System; University of Michigan; University of Michigan System; University of Michigan; University of Michigan System; University of Michigan; University of Michigan System; University of Michigan
Harper, DM (corresponding author), 1018 Fuller St, Ann Arbor, MI 48105 USA. harperdi@med.umich.edu;

petrelim@med.umich.edu; anandas@umich.edu; ssgorin@med.umich.edu; mjimbo@med.umich.edu; minalrp@umich.edu; kresnic@umich.edu
Anand, Amit/D-4232-2013; Harper, Diane/I-2180-2014 Harper, Diane/0000-0001-7648-883X
National Cancer Institute Grant [P30CA046592-29-S4]; National Center for Advancing Translational Science Grant [UL1TR001070] National Cancer Institute Grant(United States Department of Health & Human ServicesNational Institutes of Health (NIH) - USANIH National Cancer Institute (NCI)); National Center for Advancing Translational Science Grant This work was supported by the National Cancer Institute Grant P30CA046592-29-S4 and the National Center for Advancing Translational Science Grant UL1TR001070. 36 12 12 2 4 ELSEVIER
AMSTERDAM RADARWEG 29, 1043 NX AMSTERDAM, NETHERLANDS
2211-3355 PREV MED REP Prev. Med. Rep. JUN 2021
22 101375
10.1016/j.pmedr.2021.101375
http://dx.doi.org/10.1016/j.pmedr.2021.101375 APR 2021 7 Public, Environmental & Occupational Health Science Citation Index Expanded (SCI-EXPANDED); Social Science Citation Index (SSCI) Public, Environmental & Occupational Health
SP9DF 33996388 Green Published, gold 2025-06-24 WOS:000659961900046
J Percac-Lima, S; Ashburner, JM; Bond, B; Oo, SA; Atlas, SJ Percac-Lima, Sanja; Ashburner, Jeffrey M.; Bond, Barbara; Oo, Sarah A.; Atlas, Steven J.
Decreasing Disparities in Breast Cancer Screening in Refugee Women Using Culturally Tailored Patient Navigation JOURNAL OF GENERAL INTERNAL MEDICINE English Article
breast cancer screening; patient navigation; vulnerable populations; disparities HEALTH-CARE; BARRIERS; COMMUNITY; KNOWLEDGE; ATTITUDES; UPDATE; STATE Patient navigator (PN) programs can improve breast cancer screening in low income, ethnic/racial minorities. Refugee women have low breast cancer screening rates, but it has not been shown that PN is similarly effective. Evaluate whether a PN program for refugee women decreases disparities in breast cancer screening. Retrospective program evaluation of an implemented intervention. Women who self-identified as speaking Somali, Arabic, or Serbo-Croatian (Bosnian) and were eligible for breast cancer screening at an urban community health center (HC). Comparison groups were English-speaking and Spanish-speaking women eligible for breast cancer screening in the same HC. Patient navigators educated women about breast cancer screening, explored barriers to screening, and tailored interventions individually to help complete screening. Adjusted 2-year mammography rates from logistic regression models for each calendar year accounting for clustering by primary care physician. Rates in refugee women were compared to English-speaking and Spanish-speaking women in the year before implementation of the PN program and over its first 3 years. There were 188 refugee (36 Somali, 48 Arabic, 104 Serbo-Croatian speaking), 2,072 English-speaking, and 2,014 Spanish-speaking women eligible for breast cancer screening over the 4-year study period. In the year prior to implementation of the program, adjusted mammography rates were lower among refugee women (64.1 %, 95 % CI: 49-77 %) compared to English-speaking (76.5 %, 95 % CI: 69 %-83 %) and Spanish-speaking (85.2 %, 95 % CI: 79 %-90 %) women. By the end of 2011, screening rates increased in refugee women (81.2 %, 95 % CI: 72 %-88 %), and were similar to the rates

in English-speaking (80.0 %, 95 % CI: 73 %-86 %) and Spanish-speaking (87.6 %, 95 % CI: 82 %-91 %) women. PN increased screening rates in both younger and older refugee women. Linguistically and culturally tailored PN decreased disparities over time in breast cancer screening among female refugees from Somalia, the Middle East and Bosnia. [Percac-Lima, Sanja; Oo, Sarah A.] Massachusetts Gen Hosp, Chelsea HealthCare Ctr, Chelsea, MA 02150 USA; [Percac-Lima, Sanja; Ashburner, Jeffrey M.; Atlas, Steven J.] Massachusetts Gen Hosp, Dept Med, Div Gen Med, Boston, MA 02114 USA; [Bond, Barbara] Massachusetts Gen Hosp, Ctr Canc, Boston, MA USA; [Bond, Barbara] Bridgewater State Univ, Bridgewater, MA USA; [Percac-Lima, Sanja; Oo, Sarah A.] Massachusetts Gen Hosp, Ctr Community Hlth Improvement, Boston, MA 02114 USA Harvard University; Harvard University Medical Affiliates; Massachusetts General Hospital; Harvard University; Harvard University Medical Affiliates; Massachusetts General Hospital; Harvard University; Harvard University Medical Affiliates; Massachusetts General Hospital; Massachusetts System of Public Higher Education; Bridgewater State University; Harvard University; Harvard University Medical Affiliates; Massachusetts General Hospital Percac-Lima, S (corresponding author), Massachusetts Gen Hosp, Chelsea HealthCare Ctr, 151 Everett Ave, Chelsea, MA 02150 USA. spercaclima@partners.org

Susan G. Komen for Cure Massachusetts Affiliate Foundation; Agency for Health Care Research and Quality [R18 HS018161]

Susan G. Komen for Cure Massachusetts Affiliate Foundation; Agency for Health Care Research and Quality(United States Department of Health & Human ServicesAgency for Healthcare Research & Quality) This program was funded by Susan G. Komen for Cure Massachusetts Affiliate Foundation. Drs. Percac-Lima and Atlas are supported in part from a grant from the Agency for Health Care Research and Quality (R18 HS018161). 30 75

87 0 23 SPRINGER NEW YORK 233 SPRING ST, NEW YORK, NY 10013 USA 0884-8734 1525-1497 J GEN INTERN MED J. Gen. Intern. Med. NOV 2013 28 11

1463 1468 10.1007/s11606-013-2491-4

http://dx.doi.org/10.1007/s11606-013-2491-4 6

Health Care Sciences & Services; Medicine, General & Internal Science Citation Index Expanded (SCI-EXPANDED); Social Science Citation Index (SSCI) Health Care Sciences & Services; General & Internal Medicine 236DQ 23686510 Green Published

2025-06-24 WOS:000325774800017

J Harper, DM; Tariq, M; Alhawli, A; Syed, N; Patel, M; Resnicow, K Harper, Diane M.; Tariq, Madiha; Alhawli, Asraa; Syed, Nadia; Patel, Minal; Resnicow, Ken

Cancer risk perception and physician communication behaviors on cervical cancer and colorectal cancer screening ELIFE

English Article

WOMEN; RECOMMENDATION; ADHERENCE; AMERICANS; FATALISM; ARAB

Background: Women 50-65 years of age have the lowest cervical and colorectal cancer (CRC) screening rates among ages recommended for screening. The primary aim of this work is to determine how cancer risk perceptions and provider communication behaviors, in addition to known demographic factors, influence the uptake of both cervical and CRC screening or a single screen among women in southeast Michigan. Methods: Fourteen health services and communication behavior questions were adapted from the Health Information National Trends Survey (HINTS) and administered to a

multiethnic sample of adults in southeast Michigan. The outcome variable was self-reported up-to-date cervical cancer and/or CRC screening as defined by the United States Preventive Services Task Force (USPSTF). Demographic and cancer risk/communication behavior responses of the four screening populations (both tests, one test, no tests) were analyzed with multinomial regression for all comparisons. Results: Of the 394 respondents, 54% were up to date for both cervical and CRC screening, 21% were up to date with only cervical cancer screening and 12% were up to date for only CRC screening. Of the 14 risk perception and communication behavior questions, only 'Did your primary care physician (PCP) involve you in the decisions about your health care as much as you wanted?' was significantly associated with women having both screens compared to only cervical cancer screening (aOR 1.67; 95% CI: 1.08, 2.57). The multivariate model showed age, and Middle East and North African (MENA) ethnicity and Black race, in addition to PCP-patient dyad decision-making to be associated with the cancer screenings women completed. Conclusions: Optimizing PCP-patient decision-making in health care may increase opportunities for both cervical cancer and CRC screening either in the office or by self-sampling. Understanding the effects of age and the different interventional strategies needed for MENA women compared to Black women will inform future intervention trials aimed to increase both cancer screenings. [Harper, Diane M.] Univ Michigan, Dept Family Med, Sch Med, Ann Arbor, MI 48109 USA; [Harper, Diane M.] Univ Michigan, Dept Obstet & Gynecol, Sch Med, Ann Arbor, MI 48109 USA; [Harper, Diane M.] Univ Michigan, Coll Literature Sci & Arts, Dept Womens Studies, Ann Arbor, MI 48109 USA; [Tariq, Madiha; Alhawli, Asraa; Syed, Nadia] Arab Community Ctr Econ & Social Serv ACCESS, Dearborn, MI USA; [Patel, Minal] Univ Michigan, Dept Hlth Behav & Hlth Educ, Sch Publ, Ann Arbor, MI 48109 USA; [Patel, Minal; Resnicow, Ken] Univ Michigan, Outreach & Hlth Dispar Res, Rogel Canc Ctr, Ann Arbor, MI 48109 USA; [Resnicow, Ken] Univ Michigan, Sch Publ Hlth, Ctr Hlth Commun Res, Ann Arbor, MI 48109 USA University of Michigan System; University of Michigan; University of Michigan System; University of Michigan; University of Michigan System; University of Michigan; University of Michigan System; University of Michigan; University of Michigan System; University of Michigan; University of Michigan System; University of Michigan Harper, DM (corresponding author), Univ Michigan, Dept Family Med, Sch Med, Ann Arbor, MI 48109 USA.; Harper, DM (corresponding author), Univ Michigan, Dept Obstet & Gynecol, Sch Med, Ann Arbor, MI 48109 USA.; Harper, DM (corresponding author), Univ Michigan, Coll Literature Sci & Arts, Dept Womens Studies, Ann Arbor, MI 48109 USA. harperdi@med.umich.edu Harper, Diane/I-2180-2014 Harper, Diane/0000-0001-7648-883X National Cancer Institute [P30CA046592-29-S4]; National Center for Advancing Translational Sciences [UL1TR002240]; National Cancer Institute [P30CA046592] Funding Source: NIH RePORTER; National Center for Advancing Translational Sciences [UL1TR002240] Funding Source: NIH RePORTER National Cancer Institute(United States Department of Health & Human ServicesNational Institutes of Health (NIH) - USANIH National Cancer Institute (NCI)); National Center for Advancing Translational Sciences(United States Department of Health & Human ServicesNational Institutes of Health (NIH) - USANIH National Center for Advancing Translational Sciences (NCATS)); National Cancer Institute(United States Department of Health & Human ServicesNational Institutes of Health (NIH) -

USANIH National Cancer Institute (NCI)); National Center for Advancing Translational Sciences(United States Department of Health & Human ServicesNational Institutes of Health (NIH) - USANIH National Center for Advancing Translational Sciences (NCATS)) National Cancer Institute P30CA046592-29-S4 Ken Resnicow National Center for Advancing Translational Sciences UL1TR002240 Diane M Harper 44 14 16 0 7
eLIFE SCIENCES PUBL LTD CAMBRIDGE SHERATON HOUSE, CASTLE PARK, CAMBRIDGE, CB3 0AX, ENGLAND 2050-084X
ELIFE eLife AUG 24 2021 10
e70003 10.7554/eLife.70003
http://dx.doi.org/10.7554/eLife.70003 14
Biology Science Citation Index Expanded (SCI-EXPANDED); Social Science Citation Index (SSCI) Life Sciences & Biomedicine - Other Topics UG8VR 34427182 gold, Green Published, Green Submitted 2025-06-24
WOS:000689523000001
J Sharma, M; Seoud, M; Kim, JJ Sharma, Monisha; Seoud, Muhieddine; Kim, Jane J. Cost-effectiveness of increasing cervical cancer screening coverage in the Middle East: An example from Lebanon VACCINE
English Article Cervical cancer; Screening; Middle East; Lebanon; Cost-effectiveness; Modeling HUMAN-PAPILLOMAVIRUS VACCINATION; HPV-18 VACCINATION; EPIDEMIOLOGY; PREVENTION; CAREHPV; IMPACT; MODEL Background: Most cervical cancer (CC) cases in Lebanon are detected at later stages and associated with high mortality. There is no national organized CC screening program so screening is opportunistic and limited to women who can pay out-of-pocket. Therefore, a small percentage of women receive repeated screenings while most are under-or never screened. We evaluated the cost-effectiveness of increasing screening coverage and extending intervals. Methods: We used an individual-based Monte Carlo model simulating HPV and CC natural history and screening. We calibrated the model to epidemiological data from Lebanon, including CC incidence and HPV type distribution. We evaluated cytology and HPV DNA screening for women aged 25-65 years, varying coverage from 20 to 70% and frequency from 1 to 5 years. Results: At 20% coverage, annual cytologic screening reduced lifetime CC risk by 14% and had an incremental cost-effectiveness ratio of 1$80,670/year of life saved (YLS), far exceeding Lebanon's gross domestic product (GDP) per capita (1$17,460), a commonly cited cost-effectiveness threshold. By comparison, increasing cytologic screening coverage to 50% and extending screening intervals to 3 and 5 years provided greater CC reduction (26.1% and 21.4, respectively) at lower costs compared to 20% coverage with annual screening. Screening every 5 years with HPV DNA testing at 50% coverage provided greater CC reductions than cytology at the same frequency (23.4%) and was cost-effective assuming a cost of 1518 per HPV test administered (1$12,210/YLS); HPV DNA testing every 4 years at 50% coverage was also cost-effective at the same cost per test (1$16,340). Increasing coverage of annual cytology was not found to be cost-effective. Conclusion: Current practice of repeated cytology in a small percentage of women is inefficient. Increasing coverage to 50% with extended screening intervals provides greater health benefits at a reasonable cost and can more equitably distribute health gains. Novel HPV DNA strategies offer greater CC reductions and may be more cost-effective than cytology. (C) 2016 Elsevier

Ltd. All rights reserved.   [Sharma, Monisha] Univ Washington, Dept Epidemiol, 1959 NE Pacific St, Seattle, WA 98195 USA; [Seoud, Muhieddine] Amer Univ Beirut, Dept Obstet & Gynecol, Gynecol Oncol, Beirut, Lebanon; [Kim, Jane J.] Harvard TH Chan Sch Publ Hlth, Ctr Hlth Decis Sci, Boston, MA 02115 USA   University of Washington; University of Washington Seattle; American University of Beirut; Harvard University; Harvard T.H. Chan School of Public Health   Sharma, M (corresponding author), Univ Washington, Dept Epidemiol, 1959 NE Pacific St, Seattle, WA 98195 USA.
msharma04@gmail.com   GSK; MSD; Roche diagnostics GSK(GlaxoSmithKline); MSD; Roche diagnostics MS and JJK have disclosed no potential conflicts of interest. MS (Dr. Seoud) is on the medical advisory board of GSK, has received honoraria and travel grants for lecturing from GSK, MSD and Roche diagnostics and has received research grant money from GSK and MSD.   28   9   9   0   12   ELSEVIER SCI LTD OXFORD
THE BOULEVARD, LANGFORD LANE, KIDLINGTON, OXFORD OX5 1GB, OXON, ENGLAND   0264-410X   1873-2518   VACCINE   Vaccine
JAN 23   2017   35   4   564   569
10.1016/j.vaccine.2016.12.015
http://dx.doi.org/10.1016/j.vaccine.2016.12.015
6   Immunology; Medicine, Research & Experimental
Science Citation Index Expanded (SCI-EXPANDED)
Immunology; Research & Experimental Medicine EK1XQ 28017434
2025-06-24 WOS:000393721500010
J   Jafari, M; Laraqui, A; Baba, W; Benmokhtar, S; El Zaitouni, S; Ali, AA; Bounaim, A; Moujahid, M; Tanz, R; Mahfoud, T; Sbitti, Y; El Annaz, H; Abi, R; Tagajdid, MR; El Kochri, S; Lahlou, IA; El Hsaini, H; Belayachi, L; Benjouad, A; Ichou, M; En-Nya, A; Ennibi, K   Jafari, Meryem; Laraqui, Abdelilah; Baba, Walid; Benmokhtar, Soukaina; El Zaitouni, Sara; Ali, Abdelmounaim Ait; Bounaim, Ahmed; Moujahid, Mountassir; Tanz, Rachid; Mahfoud, Tarik; Sbitti, Yassir; El Annaz, Hicham; Abi, Rachid; Tagajdid, Mohamed Rida; El Kochri, Safae; Lahlou, Idriss Amine; El Hsaini, Houda; Belayachi, Lamiae; Benjouad, Abdelaziz; Ichou, Mohammed; En-Nya, Amina; Ennibi, Khalid   Prevalence and patterns of mutations in RAS/RAF/MEK/ERK/MAPK signaling pathway in colorectal cancer in North Africa BMC CANCER   English
Article   KRAS; NRAS; And BRAF mutations; Colorectal cancer; Lifestyle factors; North Africa RAS P21 ONCOPROTEIN; K-RAS; FUSOBACTERIUM-NUCLEATUM; MOROCCAN PATIENTS; KRAS MUTATIONS; BRAF; GENE; CARCINOGENESIS; POPULATION; OVEREXPRESSION   Background Our review discuss (i) the findings from analyzed data that have examined KRAS, NRAS and BRAF mutations in patients with colorectal cancer (CRC) in North Africa and to compare its prevalence with that shown in other populations and (ii) the possible role of dietary and lifestyle factors with CRC risk. Methods Using electronic databases, a systematic literature search was performed for the KRAS, NRAS, and BRAF mutations in CRC patients from Morocco, Tunisia, Algeria and Lybia. Results Seventeen studies were identified through electronic searches with six studies conducted in Morocco, eight in Tunisia, two in Algeria, and one in Libya. A total of 1843 CRC patients were included 576 (31.3%) in Morocco, 641 (34.8%) in Tunisia, 592 (32.1%) in Algeria, and 34 (1.8%) in Libya. Overall, the average age of patients was 52.7 years old. Patients were predominantly male (56.6%). The mutation rates of KRAS, NRAS and BRAF were 46.4%, 3.2% and 3.5% of all patients, respectively. A

broad range of reported KRAS mutation frequencies have been reported in North Africa countries. The KRAS mutation frequency was 23.9% to 51% in Morocco, 23.1% to 68.2% in Tunisia, 31.4% to 50% in Algeria, and 38.2% in Libya. The G12D was the most frequently identified KRAS exon 2 mutations (31.6%), followed by G12V (25.4%), G13D (15.5%), G12C (10.2%), G12A (6.9%), and G12S (6.4%). G12R, G13V, G13C and G13R are less than 5%. There are important differences among North Africa countries. In Morocco and Tunisia, there is a higher prevalence of G12D mutation in KRAS exon 2 (approximate to 50%). The most frequently mutation type in KRAS exon 3 was Q61L (40%). A59T and Q61E mutations were also found. In KRAS exon 4, the most common mutation was A146T (50%), followed by K117N (33.3%), A146P (8.3%) and A146V (8.3%). Conclusion KRAS mutated CRC patients in North Africa have been identified with incidence closer to the European figures. Beside established anti-CRC treatment, better understanding of the causality of CRC can be established by combining epidemiology and genetic/epigenetic on CRC etiology. This approach may be able to significantly reduce the burden of CRC in North Africa. [Jafari, Meryem; Laraqui, Abdelilah; Baba, Walid; El Annaz, Hicham; Abi, Rachid; Tagajdid, Mohamed Rida; El Kochri, Safae; Lahlou, Idriss Amine; Ennibi, Khalid] Mohammed V Univ Rabat, Mohammed V Mil Teaching Hosp, Fac Med & Pharm, Lab Virol,Sequencing Unit,Ctr Virol Infect & Trop, Rabat, Morocco; [Jafari, Meryem; Baba, Walid; Benmokhtar, Soukaina; El Zaitouni, Sara; En-Nya, Amina] Mohammed V Univ Rabat, Fac Sci, Genom Ctr Human Pathol, Lab Biol Human Pathol,Dept Biol, Rabat, Morocco; [Ali, Abdelmounaim Ait; Bounaim, Ahmed; Moujahid, Mountassir] Mohammed V Univ Rabat, Mohammed V Mil Teaching Hosp, Fac Med & Pharm, Dept Digest Surg, Rabat, Morocco; [Tanz, Rachid; Mahfoud, Tarik; Sbitti, Yassir; Ichou, Mohammed] Mohammed V Univ Rabat, Mohammed V Mil Teaching Hosp, Fac Med & Pharm, Dept Med Oncol, Rabat, Morocco; [El Hsaini, Houda; Belayachi, Lamiae; Benjouad, Abdelaziz] Int Univ Rabat, Int Fac Dent Med, Coll Hlth Sci, Rabat, Morocco; [Ennibi, Khalid] Mohammed V Univ Rabat, Mohammed V Mil Teaching Hosp, Fac Med & Pharm, Ctr Virol Infect & Trop Dis, Rabat, Morocco Mohammed V University in Rabat; Ibn sina University Hospital Center of Rabat; Mohammed V University in Rabat; Mohammed V University in Rabat; Ibn sina University Hospital Center of Rabat; Mohammed V University in Rabat; Ibn sina University Hospital Center of Rabat; Universite Internationale de Rabat; Mohammed V University in Rabat; Ibn sina University Hospital Center of Rabat Jafari, M (corresponding author), Mohammed V Univ Rabat, Mohammed V Mil Teaching Hosp, Fac Med & Pharm, Lab Virol,Sequencing Unit,Ctr Virol Infect & Trop, Rabat, Morocco.; Jafari, M (corresponding author), Mohammed V Univ Rabat, Fac Sci, Genom Ctr Human Pathol, Lab Biol Human Pathol,Dept Biol, Rabat, Morocco. meryem.jafari@um5r.ac.ma JAFARI, MERYEM/HKM-7981-2023; Benmokhtar, Soukaina/KHZ-8666-2024 benmokhtar, soukaina/0000-0002-0628-3190; Jafari, Meryem/0000-0002-2892-9108 90 7 7 0 7 BMC LONDON CAMPUS, 4 CRINAN ST, LONDON N1 9XW, ENGLAND 1471-2407 BMC CANCER BMC Cancer NOV 7 2022 22 1 1142
10.1186/s12885-022-10235-w
http://dx.doi.org/10.1186/s12885-022-10235-w 14
Oncology Science Citation Index Expanded (SCI-EXPANDED)
Oncology 5Z2CT 36344948 Green Submitted, gold, Green Published 2025-06-24 WOS:000879785300001

J Ziadi, W; Boussetta, S; Elkamel, S; Pakstis, AJ; Kidd, KK; Medimegh, I; Elgaaied, AB; Cherni, L Ziadi, Wafa; Boussetta, Sami; Elkamel, Sarra; Pakstis, Andrew J.; Kidd, Kenneth K.; Medimegh, Imen; Elgaaied, Amel Ben Ammar; Cherni, Lotfi STAT3 polymorphisms in North Africa and its implication in breast cancerMOLECULAR GENETICS & GENOMIC MEDICINE English Article

breast cancer; miR-3606-5p; North Africa; rs7211777; STAT3

SIGNAL-TRANSDUCER; TRANSCRIPTION FACTORS; GENE POLYMORPHISMS; PROSTATE-CANCER; ASSOCIATION; ACTIVATOR; INFLAMMATION; EXPRESSION; ROLES; CELLS Background Only a few studies have investigated the association of single nucleotide polymorphisms in STAT3 gene with the susceptibility to cancer and response to chemotherapy. Our aim was to determine the allele frequencies of rs3869550, rs957971, and rs7211777 at the STAT3 gene in North African populations and compare them to 1000 genomes populations, and to investigate their relation with cancer. Methods The targeted SNPs have been analyzed in six Tunisian populations and a sample of Libyans using TaqMan (R) Assay. The results were compared to 1000 Genomes Project population samples. Targeting of the regions encompassing the three SNPs by micro-ARN was assessed using miR databases. Results The analysis of the 3 SNPs showed that North African populations were close to South Asians. As expected, African populations presented a significant frequency of the ancestral CCG haplotype in contrast to other populations where the fully derived TGA haplotype was more frequent. The presence and diversity of rare haplotypes at STAT3 in North African populations could have been generated by recombination between the two major haplotypes. A screening of the micro-RNA databases showed that the STAT3 region with the mutated allele of rs7211777 (G>A) could be targeted by miR hsa-miR-3606-5p, which also targets genes involved in breast cancer. [Ziadi, Wafa; Boussetta, Sami; Elkamel, Sarra; Medimegh, Imen; Elgaaied, Amel Ben Ammar; Cherni, Lotfi] Univ Tunis El Manar, Fac Sci Tunis, Lab Genet Immunol & Human Pathol, Tunis 2092, Tunisia; [Pakstis, Andrew J.; Kidd, Kenneth K.] Yale Univ, Sch Med, Dept Genet, New Haven, CT 06510 USA; [Cherni, Lotfi] Univ Monastir, High Inst Biotechnol, Monastir, Tunisia Universite de Tunis-El-Manar; Faculte des Sciences de Tunis (FST); Yale University; Universite de Monastir Ziadi, W (corresponding author), Univ Tunis El Manar, Fac Sci Tunis, Lab Genet Immunol & Human Pathol, Tunis 2092, Tunisia. wefetou@gmail.com L, CHERNI/J-2849-2013; Boussetta, Sami/AAG-8913-2019; ElKamel, Sarra/O-6509-2015

LOTFI, CHERNI/0000-0002-7050-6539; ElKamel, Sarra/0000-0002-1110-5225; Boussetta, Sami/0000-0003-3994-6701 Tunisian Ministry of Higher Education and Scientific Research; University of Tunis El Manar; U.S. National Institute of Justice Tunisian Ministry of Higher Education and Scientific Research(Ministry of Higher Education & Scientific Research of Tunisia); University of Tunis El Manar(Universite de Tunis-El-Manar); U.S. National Institute of Justice(US National Institute of Justice) This work was partially supported by the Tunisian Ministry of Higher Education and Scientific Research as well as by the University of Tunis El Manar. Data generation at Yale was supported by grants from the U.S. National Institute of Justice. 47 0 0

0 2 WILEY HOBOKEN 111 RIVER ST, HOBOKEN 07030-5774, NJ USA 2324-9269 MOL GENET GENOM MED Mol. Genet. Genom. Med. AUG 2021 9 8

e1744 10.1002/mgg3.1744
http://dx.doi.org/10.1002/mgg3.1744 JUL 2021 11
Genetics & Heredity Science Citation Index Expanded (SCI-EXPANDED) Genetics & Heredity UJ0PT 34251094 Green Published 2025-06-24 WOS:000671832900001
J Bamia, C; Lagiou, P; Buckland, G; Grioni, S; Agnoli, C; Taylor, AJ; Dahm, CC; Overvad, K; Olsen, A; Tjonneland, A; Cottet, V; Boutron-Ruault, MC; Morois, S; Grote, V; Teucher, B; Boeing, H; Buijsse, B; Trichopoulos, D; Adarakis, G; Tumino, R; Naccarati, A; Panico, S; Palli, D; Bueno-de-Mesquita, HB; van Duijnhoven, FJB; Peeters, PHM; Engeset, D; Skeie, G; Lund, E; Sánchez, MJ; Barricarte, A; Huerta, JM; Quirós, JR; Dorronsoro, M; Ljuslinder, I; Palmqvist, R; Drake, I; Key, TJ; Khaw, KT; Wareham, N; Romieu, I; Fedirko, V; Jenab, M; Romaguera, D; Norat, T; Trichopoulou, A Bamia, Christina; Lagiou, Pagona; Buckland, Genevieve; Grioni, Sara; Agnoli, Claudia; Taylor, Aliki J.; Dahm, Christina C.; Overvad, Kim; Olsen, Anja; Tjonneland, Anne; Cottet, Vanessa; Boutron-Ruault, Marie-Christine; Morois, Sophie; Grote, Verena; Teucher, Birgit; Boeing, Heiner; Buijsse, Brian; Trichopoulos, Dimitrios; Adarakis, George; Tumino, Rosario; Naccarati, Alessio; Panico, Salvatore; Palli, Domenico; Bueno-de-Mesquita, H. Bas; van Duijnhoven, Fraenzel J. B.; Peeters, Petra H. M.; Engeset, Dagrun; Skeie, Guri; Lund, Eiliv; Sanchez, Maria-Jose; Barricarte, Aurelio; Huerta, Jose-Maria; Ramon Quiros, J.; Dorronsoro, Miren; Ljuslinder, Ingrid; Palmqvist, Richard; Drake, Isabel; Key, Timothy J.; Khaw, Kay-Tee; Wareham, Nick; Romieu, Isabelle; Fedirko, Veronika; Jenab, Mazda; Romaguera, Dora; Norat, Teresa; Trichopoulou, Antonia Mediterranean diet and colorectal cancer risk: results from a European cohort
EUROPEAN JOURNAL OF EPIDEMIOLOGY English
Article Mediterranean diet; Colorectal cancer; Cohort study PHYSICAL-ACTIVITY; RECTAL CANCERS; EPIC COHORT; NUTRITION; ADHERENCE; PATTERNS; SURVIVAL; HEALTH; METAANALYSIS; POPULATION The authors investigated the association of adherence to Mediterranean diet with colorectal cancer (CRC) risk in the European Prospective Investigation into Cancer and nutrition study. Adherence to Mediterranean diet was expressed through two 10-unit scales, the Modified Mediterranean diet score (MMDS) and the Centre-Specific MMDS (CSMMDS). Both scales share the same dietary components but differ in the cut-off values that were used for these components in the construction of the scales. Adjusted hazard ratios (HR) for the associations of these scales with CRC incidence were estimated. After 5,296,617 person-years of follow-up, 4,355 incident CRC cases were identified. A decreased risk of CRC, of 8 and 11 % was estimated when comparing the highest (scores 6-9) with the lowest (scores 0-3) adherence to CSMMDS and MMDS respectively. For MMDS the HR was 0.89 (95 % confidence interval (CI): 0.80, 0.99). A 2-unit increment in either Mediterranean scale was associated with a borderline statistically significant 3 to 4 % reduction in CRC risk (HR for MMDS: 0.96; 95 % CI: 0.92, 1.00). These associations were somewhat more evident, among women, were mainly manifested for colon cancer risk and their magnitude was not altered when alcohol was excluded from MMDS. These findings suggest that following a Mediterranean diet may have a modest beneficial effect on CRC risk. [Bamia, Christina; Lagiou, Pagona; Trichopoulou, Antonia] Univ Athens, Sch Med, Dept Hyg Epidemiol & Med Stat, WHO Collaborating Ctr Food & Nutr Policies, GR-11527 Athens, Greece;

[Lagiou, Pagona; Trichopoulos, Dimitrios] Harvard Univ, Sch Publ Hlth, Dept Epidemiol, Boston, MA 02115 USA; [Lagiou, Pagona; Trichopoulos, Dimitrios; Trichopoulou, Antonia] Acad Athens, Bur Epidemiol Res, Athens, Greece; [Buckland, Genevieve] Catalan Inst Oncol ICO, Unit Nutr Environm & Canc, Canc Epidemiol Res Programme, Barcelona, Spain; [Grioni, Sara] IRCCS Ist Nazl Tumori, Nutr Epidemiol Unit, Milan, Italy; [Agnoli, Claudia] Fdn IRCCS Ist Nazl Tumori, Epidemiol & Prevent Unit, Milan, Italy; [Taylor, Aliki J.] Univ Birmingham, Dept Publ Hlth Epidemiol & Biostat, Birmingham, W Midlands, England; [Dahm, Christina C.; Overvad, Kim] Aarhus Univ, Epidemiol Sect, Dept Publ Hlth, Aarhus, Denmark; [Olsen, Anja; Tjonneland, Anne] Danish Canc Soc Res Ctr, Copenhagen, Denmark; [Cottet, Vanessa; Boutron-Ruault, Marie-Christine; Morois, Sophie] Inst Gustave Roussy, Ctr Res Epidemiol & Populat Hlth, INSERM, U1018, F-94805 Villejuif, France; [Cottet, Vanessa; Boutron-Ruault, Marie-Christine; Morois, Sophie] Paris South Univ, UMRS 1018, F-94805 Villejuif, France; [Grote, Verena; Teucher, Birgit] German Canc Res Ctr, Heidelberg, Germany; [Boeing, Heiner; Buijsse, Brian] German Inst Human Nutr Potsdam Rehbrucke, Dept Epidemiol, Potsdam, Germany; [Trichopoulos, Dimitrios; Adarakis, George; Trichopoulou, Antonia] Hellen Hlth Fdn, Athens, Greece; [Tumino, Rosario] Civile MP Arezzo Hosp, Canc Registry Histopathol Unit, ASP, Ragusa, Italy; [Naccarati, Alessio] Human Genet Fdn HuGeF, Turin, Italy; [Panico, Salvatore] Univ Naples Federico II, Dept Clin & Expt Med, Naples, Italy; [Palli, Domenico] ISPO, Canc Res & Prevent Inst, Mol & Nutrit Epidemiol Unit, Florence, Italy; [Bueno-de-Mesquita, H. Bas; van Duijnhoven, Fraenzel J. B.] Natl Inst Publ Hlth & Environm RIVM, Bilthoven, Netherlands; [Bueno-de-Mesquita, H. Bas] Univ Med Ctr, Dept Gastroenterol & Hepatol, Utrecht, Netherlands; [van Duijnhoven, Fraenzel J. B.] Wageningen Univ, Div Human Nutr, NL-6700 AP Wageningen, Netherlands; [Peeters, Petra H. M.] Univ Med Ctr Utrecht, Julius Ctr, Utrecht, Netherlands; [Peeters, Petra H. M.; Romaguera, Dora; Norat, Teresa] Univ London Imperial Coll Sci Technol & Med, Sch Publ Hlth, Fac Med, Dept Epidemiol & Biostat, London, England; [Engeset, Dagrun; Skeie, Guri; Lund, Eiliv] Univ Tromso, Dept Community Med, Tromso, Norway; [Sanchez, Maria-Jose] Andalusian Sch Publ Hlth, Granada, Spain; [Sanchez, Maria-Jose; Barricarte, Aurelio; Huerta, Jose-Maria; Dorronsoro, Miren] CIBER Epidemiol & Salud Publ CIBERESP, Madrid, Spain; [Barricarte, Aurelio] Navarre Publ Hlth Inst, Pamplona, Spain; [Barricarte, Aurelio] Navarre Publ Univ, Navarra, Spain; [Huerta, Jose-Maria] Murcia Reg Hlth Council, Dept Epidemiol, Murcia, Spain; [Ramon Quiros, J.] Publ Hlth Directorate, Asturias, Spain; [Dorronsoro, Miren] BioDonostia Res Inst, Publ Hlth Div Gipuzkoa, Dept Hlth, San Sebastian, Spain; [Ljuslinder, Ingrid] Umea Univ, Dept Radiat Sci, Umea, Sweden; [Palmqvist, Richard] Umea Univ, Dept Med Biosci, Umea, Sweden; [Drake, Isabel] Lund Univ, Nutr Epidemiol Res Grp, Dept Clin Sci, Malmo, Sweden; [Key, Timothy J.] Univ Oxford, Nuffield Dept Clin Med, Canc Epidemiol Unit, Oxford, England; [Khaw, Kay-Tee] Univ Cambridge, Cambridge, England; [Wareham, Nick] MRC, Epidemiol Unit, Cambridge, England; [Romieu, Isabelle; Fedirko, Veronika; Jenab, Mazda] Int Agcy Res Canc IARC WHO, Lyon, France National & Kapodistrian University of Athens; Athens Medical School; World Health Organization; Harvard University; Harvard T.H. Chan School of Public Health; Academy of Athens; Institut Catala d'Oncologia; Fondazione IRCCS Istituto Nazionale Tumori Milan; Fondazione IRCCS Istituto Nazionale Tumori

Milan; University of Birmingham; Aarhus University; Danish Cancer Society; Universite Paris Saclay; Institut National de la Sante et de la Recherche Medicale (Inserm); UNICANCER; Gustave Roussy; Institut National de la Sante et de la Recherche Medicale (Inserm); Universite Paris Saclay; Helmholtz Association; German Cancer Research Center (DKFZ); Leibniz Association; Deutsches Institut fur Ernahrungsforschung Potsdam-Rehbrucke (DIfE); Civile M.P. Arezzo Hospital; University of Naples Federico II; Netherlands National Institute for Public Health & the Environment; Utrecht University; Utrecht University Medical Center; Wageningen University & Research; Utrecht University; Utrecht University Medical Center; Imperial College London; UiT The Arctic University of Tromso; Escuela Andaluza de Salud Publica; CIBER - Centro de Investigacion Biomedica en Red; CIBERESP; Public Health Institute of Navarra; Universidad Publica de Navarra; Murcia Regional Health Council; Umea University; Umea University; Lund University; University of Oxford; University of Cambridge; World Health Organization; International Agency for Research on Cancer (IARC)   Bamia, C (corresponding author), Univ Athens, Sch Med, Dept Hyg Epidemiol & Med Stat, WHO Collaborating Ctr Food & Nutr Policies, 75 Mikras Asias St, GR-11527 Athens, Greece.   cbamia@nut.uoa.gr   Engeset, Dagrun/AFW-5378-2022; Jenab, Mehdi/L-2515-2019; taylor, aliki/AAM-1702-2021; Trichopoulos, Dimitrios/G-6825-2012; van Duijnhoven, Fränzel/GWC-0059-2022; Teucher, Birgit/J-6380-2015; TRICHOPOULOU, ANTONIA/ABF-8727-2021; Romaguera, Dora/AAB-2852-2020; Khaw, Kay-Tee/AAZ-3209-2021; Panico, Salvatore/K-6506-2016; Sánchez, María/HOC-7747-2023; Tjonneland, Anne/AGU-0320-2022; Boutron-Ruault, Marie-Christine/H-3936-2014; Cottet, Vanessa/ABE-3236-2020; Dahm, Christina/G-9787-2014; Agnoli, Claudia/K-5916-2016; Huerta, Jose Maria/N-8654-2015; SANCHEZ-PEREZ, MARIA JOSE/D-1087-2011; grioni, Sara/K-5320-2016
Buckland, Genevieve/0000-0003-2060-6598; PALLI, Domenico/0000-0002-5558-2437; Olsen, Anja/0000-0003-4788-503X; Naccarati, Alessio/0000-0001-5774-0905; Dahm, Christina/0000-0003-0481-2893; Panico, Salvatore/0000-0002-5498-8312; Agnoli, Claudia/0000-0003-4472-1179; Huerta, Jose Maria/0000-0002-9637-3869; Lund, Eiliv/0000-0002-8071-8711; Skeie, Guri/0000-0003-2476-4251; SANCHEZ-PEREZ, MARIA JOSE/0000-0003-4817-0757; Tjonneland, Anne/0000-0003-4385-2097; Engeset, Dagrun/0000-0001-6946-2949; Jenab, Mazda/0000-0002-0573-1852; Drake, Isabel/0000-0002-6500-6310; grioni, Sara/0000-0002-5891-8426; Overvad, Kim/0000-0001-6429-7921; Romaguera, Dora/0000-0002-5762-8558; tumino, rosario/0000-0003-2666-414X Word Cancer Research Fund [2003/18, 2007/13]; European Commission (DG-SANCO); International Agency for Research on Cancer; Danish Cancer Society (Denmark); Ligue Contre le Cancer; Institut Gustave Roussy; Mutuelle Generale de l'Education Nationale; Institut National de la Sante et de la Recherche Medicale (INSERM) (France); Deutsche Krebshilfe; German Cancer Research Center (DKFZ); Federal Ministry of Education and Research (Germany); Hellenic Health Foundation; Stavros Niarchos Foundation (Greece); Italian Association for Research on Cancer (AIRC); National Research Council; AIRE-ONLUS Ragusa; AVIS Ragusa; Sicilian Government (Italy); Dutch Ministry of Public Health, Welfare and Sports (VWS); Netherlands Cancer Registry (NKR); LK Research Funds; Dutch Prevention Funds; Dutch ZON (Zorg Onderzoek Nederland); World Cancer Research Fund (WCRF); Statistics Netherlands (The Netherlands); Nordforsk and Nordic Center of Excellence programme on Food, Nutrition and Health (Norway);

Health Research Fund (FIS); Regional Government of Andalucia; Regional Government of Asturias; Regional Government of Basque Country; Regional Government of Murcia [6236]; Navarra and ISCIII RETIC (Spain) [RD06/0020]; Swedish Cancer Society; Swedish Scientific Council; Regional Government of Skane and Vasterbotten (Sweden); Cancer Research UK; Medical Research Council; Stroke Association; British Heart Foundation; Department of Health; Food Standards Agency; Welcome Trust (United Kingdom); [ERC-2009-AdG 232997] Word Cancer Research Fund; European Commission (DG-SANCO)(European Union (EU)European Commission Joint Research Centre); International Agency for Research on Cancer; Danish Cancer Society (Denmark)(Danish Cancer Society); Ligue Contre le Cancer(Ligue nationale contre le cancer); Institut Gustave Roussy; Mutuelle Generale de l'Education Nationale; Institut National de la Sante et de la Recherche Medicale (INSERM) (France)(Institut National de la Sante et de la Recherche Medicale (Inserm)); Deutsche Krebshilfe(Deutsche Krebshilfe); German Cancer Research Center (DKFZ)(Helmholtz Association); Federal Ministry of Education and Research (Germany)(Federal Ministry of Education & Research (BMBF)); Hellenic Health Foundation; Stavros Niarchos Foundation (Greece); Italian Association for Research on Cancer (AIRC)(Fondazione AIRC per la ricerca sul cancro); National Research Council; AIRE-ONLUS Ragusa; AVIS Ragusa; Sicilian Government (Italy); Dutch Ministry of Public Health, Welfare and Sports (VWS); Netherlands Cancer Registry (NKR); LK Research Funds; Dutch Prevention Funds; Dutch ZON (Zorg Onderzoek Nederland)(Netherlands Organization for Scientific Research (NWO)); World Cancer Research Fund (WCRF)(World Cancer Research Fund International (WCRF)); Statistics Netherlands (The Netherlands)(Netherlands Government); Nordforsk and Nordic Center of Excellence programme on Food, Nutrition and Health (Norway)(NordForsk); Health Research Fund (FIS)(Instituto de Salud Carlos III); Regional Government of Andalucia(Junta de Andalucia); Regional Government of Asturias(Principality of Asturias); Regional Government of Basque Country(Basque Government); Regional Government of Murcia; Navarra and ISCIII RETIC (Spain); Swedish Cancer Society(Swedish Cancer Society); Swedish Scientific Council; Regional Government of Skane and Vasterbotten (Sweden); Cancer Research UK(Cancer Research UK); Medical Research Council(UK Research & Innovation (UKRI)Medical Research Council UK (MRC)); Stroke Association; British Heart Foundation(British Heart Foundation); Department of Health; Food Standards Agency; Welcome Trust (United Kingdom)(Wellcome Trust); This work was supported by the Word Cancer Research Fund (grants number 2003/18 and 2007/13). The coordination of EPIC is financially supported by the European Commission (DG-SANCO) and the International Agency for Research on Cancer. The national cohorts are supported by: Danish Cancer Society (Denmark); Ligue Contre le Cancer, Institut Gustave Roussy, Mutuelle Generale de l'Education Nationale, and Institut National de la Sante et de la Recherche Medicale (INSERM) (France); Deutsche Krebshilfe, German Cancer Research Center (DKFZ) and Federal Ministry of Education and Research (Germany); Hellenic Health Foundation and Stavros Niarchos Foundation (Greece); Italian Association for Research on Cancer (AIRC), National Research Council and AIRE-ONLUS Ragusa, AVIS Ragusa, Sicilian Government (Italy); Dutch Ministry of Public Health, Welfare and Sports (VWS), Netherlands Cancer Registry (NKR), LK Research Funds, Dutch Prevention Funds, Dutch ZON (Zorg Onderzoek

Nederland), World Cancer Research Fund (WCRF) and Statistics Netherlands (The Netherlands); ERC-2009-AdG 232997 and Nordforsk and Nordic Center of Excellence programme on Food, Nutrition and Health (Norway); Health Research Fund (FIS), Regional Governments of Andalucia, Asturias, Basque Country, Murcia (No 6236) and Navarra and ISCIII RETIC (RD06/0020) (Spain); Swedish Cancer Society, Swedish Scientific Council and Regional Government of Skane and Vasterbotten (Sweden); Cancer Research UK, Medical Research Council, Stroke Association, British Heart Foundation, Department of Health, Food Standards Agency and Welcome Trust (United Kingdom). 35 139 155 0 46 SPRINGER DORDRECHT VAN GODEWIJCKSTRAAT 30, 3311 GZ DORDRECHT, NETHERLANDS 0393-2990 EUR J EPIDEMIOL Eur. J. Epidemiol. APR 2013 28 4 317 328 10.1007/s10654-013-9795-x http://dx.doi.org/10.1007/s10654-013-9795-x 12 Public, Environmental & Occupational Health Science Citation Index Expanded (SCI-EXPANDED) Public, Environmental & Occupational Health 143VI 23579425 2025-06-24 WOS:000318896000005
J Gereige, JD; Zhang, L; Boehmer, U Gereige, Jessica D.; Zhang, Li; Boehmer, Ulrike The Sexual Health of Women in Lebanon: Are There Differences by Sexual Orientation? LGBT HEALTH English Article access to care; cervical cancer screening; female homosexuality; Middle East and North Africa; sexual health; women's health EXTENDED MIDDLE-EAST; CERVICAL-CANCER; UNITED-STATES; LESBIANS; PREVALENCE; MINORITY; MEN; DISPARITIES; KNOWLEDGE; BEHAVIOR Purpose: From studies conducted in Western countries (United States, United Kingdom, and Australia), we know that the sexual health of sexual minority women (SMW) differs in key ways from that of heterosexual women (HSW). To date, the sexual health of SMW living in the Middle East and North Africa region has not been studied. The purpose of this study was to compare the sexual health of SMW and HSW living in Lebanon. Methods: SMW and HSW living in Lebanon (N=95) completed an anonymous, self-administered survey. SMW's risk perceptions and health promoting and sexual behaviors were compared to those of HSW. We examined differences by sexual orientation by using t tests and Fisher's exact tests. Results: The 45 SMW and 50 HSW had similar demographic characteristics. Significantly more SMW had heard of human papillomavirus, but only 22% of women from both groups knew of its association with abnormal Papanicolaou tests. Cervical cancer screening rates were similar in SMW and HSW, although remarkably low (42%) compared with rates in Western countries. Significantly more SMW (18%) reported difficulty with access to care than HSW (0%). Forty-four percent of SMW reported discomfort in disclosing their sexual orientation to their healthcare provider and 61% reported that healthcare providers lacked sensitivity toward lesbian, gay, bisexual, and transgender needs. Unwanted sexual contact occurred more frequently in SMW (53%) than HSW (23%). Conclusion: The sexual health of women is affected by sociocultural factors. SMW living in Lebanon have unique health needs that should be addressed within their sociocultural context. [Gereige, Jessica D.] Massachusetts Gen Hosp, Dept Internal Med, 55 Fruit St, Boston, MA 02114 USA; [Gereige, Jessica D.] Harvard Med Sch, Boston, MA USA; [Zhang, Li] Boston Univ, Sch Publ Hlth, Dept Biostat, Boston, MA USA;

[Boehmer, Ulrike] Boston Univ, Sch Publ Hlth, Dept Community Hlth Sci, Boston, MA USA   Harvard University; Harvard University Medical Affiliates; Massachusetts General Hospital; Harvard University; Harvard Medical School; Boston University; Boston University Gereige, JD (corresponding author), Massachusetts Gen Hosp, Dept Internal Med, 55 Fruit St, Boston, MA 02114 USA.
jgereige@mgh.harvard.edu   Gereige, Jessica/0000-0003-0755-1131; Boehmer, Ulrike/0000-0003-0097-5927
52   6   6   0   6   MARY ANN LIEBERT, INC
NEW ROCHELLE   140 HUGUENOT STREET, 3RD FL, NEW ROCHELLE, NY 10801 USA   2325-8292   2325-8306   LGBT HEALTH LGBT Health   JAN   2018   5   1   45   53
10.1089/lgbt.2017.0031
http://dx.doi.org/10.1089/lgbt.2017.0031   NOV 2017
9   Public, Environmental & Occupational Health   Science Citation Index Expanded (SCI-EXPANDED); Social Science Citation Index (SSCI)   Public, Environmental & Occupational Health
FS5FC 29130791   2025-06-24
WOS:000416444100001
J   Escrich, E; Moral, R; Solanas, M   Escrich, Eduard; Moral, Raquel; Solanas, Montserrat   Olive oil, an essential component of the Mediterranean diet, and breast cancer   PUBLIC HEALTH NUTRITION   English
Article   Mediterranean diet; Olive oil; Breast cancer   HIGH CORN-OIL; INDUCED MAMMARY-CARCINOMA; FATTY-ACID; LIPID INFLUENCE; OLEIC-ACID; RAT; APOPTOSIS; TUMORS; RISK; EXPRESSION   Objective: The Mediterranean diet has been related to a lower risk of some chronic diseases, including cancer. We aim to gain insight into the effects of the main source of fat of this diet on breast cancer, the most common type of malignancy in women. Design: Data from sixteen experimental series analysing the effects of dietary lipids on mammary carcinogenesis in an animal model, in the context of the international literature on the Mediterranean diet, olive oil and breast cancer risk. Setting: Experimental and human data on the effects of olive oil and Mediterranean diet on breast cancer. Subjects: An animal model of induced breast cancer and other human and experimental studies in the literature. Results: Diets rich in extra virgin olive oil (EVOO) exert a negative modulatory effect on experimental breast cancer to a weak promoting effect, much lower than that obtained with a high-corn oil diet. EVOO confers to the mammary adenocarcinomas a clinical behaviour and morphological features compatible with low tumour aggressiveness. This differential effect, in relation to other dietary lipids, may be related to a lower effect on body weight and sexual maturation. In addition, EVOO induced different molecular changes in tumours, such as in the composition of cell membranes, activity of signalling proteins and gene expression. All these modifications could induce lower proliferation, higher apoptosis and lower DNA damage. These results, together with the favourable effect of olive oil reported in the literature when it is consumed in moderate quantities, suggest a beneficial influence of EVOO on breast cancer risk. Conclusions: Consumption of EVOO in moderate quantities and throughout the lifetime appears to be a healthy choice and may favourably influence breast cancer risk.
[Escrich, Eduard; Moral, Raquel; Solanas, Montserrat] Univ Autonoma Barcelona, Dept Cell Biol Physiol & Immunol, Physiol Unit, Sch Med, E-08193 Barcelona, Spain Autonomous University of

Barcelona Escrich, E (corresponding author), Univ Autonoma Barcelona, Dept Cell Biol Physiol & Immunol, Physiol Unit, Sch Med, E-08193 Barcelona, Spain.   Eduard.Escrich@uab.es Garcia, Montserrat/O-4684-2016; Moral, Raquel/H-2122-2015 SOLANAS GARCIA, MONTSERRAT/0000-0003-2949-1344; Moral, Raquel/0000-0002-2738-6121
'Plan Nacional de I+D+I' [AGL2006-07691/ALI]; Fundacion Patrimonio Comunal Olivarero [FPCO2008-165.396]; Agencia para el Aceite de Oliva del Ministerio de Medio Ambiente y de Medio Rural y Marino [AAO2008-165.471]; Organizacion Interprofesional del Aceite de Oliva Espanol [OIP2009-165.646]; Departaments d'Agricultura, Alimentacioi Accio Rural, i de Salut de la Generalitat de Catalunya [GC2010-165.000]   'Plan Nacional de I+D+I'; Fundacion Patrimonio Comunal Olivarero; Agencia para el Aceite de Oliva del Ministerio de Medio Ambiente y de Medio Rural y Marino; Organizacion Interprofesional del Aceite de Oliva Espanol; Departaments d'Agricultura, Alimentacioi Accio Rural, i de Salut de la Generalitat de Catalunya(Generalitat de Catalunya)
The present study was supported by grants from the 'Plan Nacional de I+D+I 2004-2007' (AGL2006-07691/ALI); 'Fundacion Patrimonio Comunal Olivarero 2008-2012' (FPCO2008-165.396); 'Agencia para el Aceite de Oliva del Ministerio de Medio Ambiente y de Medio Rural y Marino 2008-2012' (AAO2008-165.471); 'Organizacion Interprofesional del Aceite de Oliva Espanol 2009-2013' (OIP2009-165.646) and the 'Departaments d'Agricultura, Alimentacioi Accio Rural, i de Salut de la Generalitat de Catalunya' (GC2010-165.000). The sponsors had no role in the study design, data collection and analysis, interpretation of results, the preparation of the manuscript, the decision to submit the manuscript for publication or the writing of the manuscript. E. E. conceived the study, its design, and coordinated the project. All authors are involved with the study design and review of the literature, data analysis and interpretation, made critical revisions of the paper, and provided approval for its publication. The authors thank Raquel Escrich and Irmgard Costa for their collaboration on these studies.   77   58   65   0   26
CAMBRIDGE UNIV PRESS   CAMBRIDGE   EDINBURGH BLDG, SHAFTESBURY RD, CB2 8RU CAMBRIDGE, ENGLAND   1368-9800   1475-2727
PUBLIC HEALTH NUTR   Public Health Nutr.   DEC   2011
14   12A   SI   2323 2332
10.1017/S1368980011002588
http://dx.doi.org/10.1017/S1368980011002588   10
Public, Environmental & Occupational Health; Nutrition & Dietetics Science Citation Index Expanded (SCI-EXPANDED)
Public, Environmental & Occupational Health; Nutrition & Dietetics 871IF 22166191   Bronze   2025-06-24
WOS:000298730800009
J   Ghebrendrias, S; Pfeil, S; Crouthamel, B; Chalmiers, M; Kully, G; Mody, S   Ghebrendrias, Selemawit; Pfeil, Sarah; Crouthamel, Bonnie; Chalmiers, Morgen; Kully, Gennifer; Mody, Sheila   An Examination of Misconceptions and Their Impact on Cervical Cancer Prevention Practices among Sub-Saharan African and Middle Eastern Refugees   HEALTH EQUITY
English   Article
cervical cancer screening; HPV vaccination; pap test; refugee women   IMMIGRANT; COMMUNITY   Objective: The purpose of the study was to understand cervical cancer screening and prevention practices of refugee women in San Diego, California and identify desired components of a cervical cancer screening

toolkit. Methods: We conducted a qualitative study utilizing semi-structured focus groups and identified common themes via grounded theory analysis. Results: There were 53 female refugee participants from Sub-Saharan Africa and the Middle East. Over half of all women surveyed expressed a fear of pelvic exams and loss of modesty as barriers to seeking gynecologic care, with nearly 34% avoiding routine pap tests. Of the 18 participants who were asked if they were aware of the Human Papilloma Virus (HPV) vaccination, only one had heard of the vaccine and none had received it for themselves or their children. Over 60% of participants were interested in educational materials surrounding HPV and pap tests. Conclusion: There is a significant lack of knowledge regarding cervical cancer screening and HPV vaccination among refugee women in San Diego, California. Refugee women in this study were interested in multi-modal educational materials as part of a cervical cancer screening toolkit. [Ghebrendrias, Selemawit] Univ Calif San Diego, Dept Obstet Gynecol & Reprod Sci, Div Family Planning, San Diego, CA 92103 USA; [Pfeil, Sarah; Chalmiers, Morgen; Kully, Gennifer; Mody, Sheila] Univ Calif San Diego, Dept Obstet & Gynecol, San Diego, CA 92103 USA; [Crouthamel, Bonnie] Univ Calif Davis, Dept Obstet & Gynecol, Davis, CA 95616 USA   University of California System; University of California San Diego; University of California System; University of California San Diego; University of California System; University of California Davis Mody, S (corresponding author), Univ Calif San Diego, Dept Obstet Gynecol & Reprod Sci, Div Family Planning, 9300 Campus Point Dr,MC 7433, La Jolla, CA 92037 USA. smody@ucsd.edu   Chalmiers, Morgen/JEZ-7148-2023

Chalmiers, Morgen/0000-0001-6174-3076 Academy of Clinician Scholars at the University of California, San Diego   Academy of Clinician Scholars at the University of California, San Diego

This study was made possible by UWEAST. Funding was provided by the Doris A Howell Foundation for Women Health Research and Academy of Clinician Scholars at the University of California, San Diego.   28   9   9   0   0   MARY ANN LIEBERT, INC

NEW ROCHELLE   140 HUGUENOT STREET, 3RD FL, NEW ROCHELLE, NY 10801 USA   2473-1242   HEALTH EQUITY   Health Equity   JUN 1 2021 5   1   382   389

10.1089/heq.2020.0125

http://dx.doi.org/10.1089/heq.2020.0125   8

Public, Environmental & Occupational Health Emerging Sources Citation Index (ESCI)   Public, Environmental & Occupational Health   SO5JV 34095709   gold, Green Published

2025-06-24 WOS:000659009400002

J   Siraj, AK; Bu, R; Azam, S; Qadri, Z; Iqbal, K; Parvathareddy, SK; Al-Dayel, F; Al-Kuraya, KS

Siraj, Abdul Khalid; Bu, Rong; Azam, Saud; Qadri, Zeeshan; Iqbal, Kaleem; Parvathareddy, Sandeep Kumar; Al-Dayel, Fouad; Al-Kuraya, Khawla S.   Whole Exome-Wide Association Identifies Rare Variants in APC Associated with High-Risk Colorectal Cancer in the Middle East   CANCERS

English   Article

colorectal cancer; exome-wide association study; rare variants; APC; high-risk CRC; Middle East; sequence kernel association test SIGNALING PATHWAYS; DIAGNOSIS; GROWTH; GENE

Background: Colorectal cancer (CRC) displays a complex pattern of inheritance. It is postulated that much of the missing heritability of CRC is enriched in high-impact rare alleles, which

might play a crucial role in the etiology and susceptibility of CRC. Methods: In this study, an exome-wide association analysis was performed in 146 patients with high-risk CRC in the Middle East and 1395 healthy controls. The aim was to identify rare germline variants in coding regions and their splicing sites associated with high-risk CRC in the Middle Eastern population. Results: Rare inactivating variants (RIVs) in APC had the strongest association with high-risk CRC (6/146 in cases vs. 1/1395 in controls, OR = 59.7, p = 5.13 x 10(-12)), whereas RIVs in RIMS1, an RAS superfamily member, were significantly associated with high-risk CRC (5/146 case vs. 2/1395 controls, OR = 24.7, p = 2.03 x 10(-8)). Rare damaging variants in 17 genes were associated with high-risk CRC at the exome-wide threshold (p < 2.5 x 10(-6)). Based on the sequence kernel association test, nonsynonymous variants in six genes (TNXB, TAP2, GPSM3, ADGRG4, TMEM229A, and ANKRD33B) had a significant association with high-risk CRC. RIVs in APC-the most common high-penetrance genetic factor-were associated with patients with high-risk CRC in the Middle East. Individuals who inherited APC RIVs had an approximate 60-fold increased risk of developing CRC and were likely to develop the disease earlier. Conclusions: We identified new potential CRC predisposition variants in other genes that could play a role in CRC inheritance. However, large collaborative studies are needed to confirm the association of these variants with high-risk CRC. These results provide information for counseling patients with high-risk CRC and their families in our population. [Siraj, Abdul Khalid; Bu, Rong; Azam, Saud; Qadri, Zeeshan; Iqbal, Kaleem; Parvathareddy, Sandeep Kumar; Al-Kuraya, Khawla S.] King Faisal Specialist Hosp & Res Ctr, Human Canc Genom Res, POB 3354, Riyadh 11211, Saudi Arabia; [Al-Dayel, Fouad] King Faisal Specialist Hosp & Res Ctr, Dept Pathol, POB 3354, Riyadh 11211, Saudi Arabia King Faisal Specialist Hospital & Research Center; King Faisal Specialist Hospital & Research Center Al-Kuraya, KS (corresponding author), King Faisal Specialist Hosp & Res Ctr, Human Canc Genom Res, POB 3354, Riyadh 11211, Saudi Arabia.
asiraj@kfshrc.edu.sa; rbu@kfshrc.edu.sa; sjeelani@kfshrc.edu.sa; sqadri96@kfshrc.edu.sa; miqbal@kfshrc.edu.sa; psandeepkumar@kfshrc.edu.sa; dayelf@kfshrc.edu.sa; kkuraya@kfshrc.edu.sa Siraj, Abdul/IQW-1179-2023; Iqbal, Kaleem/GXH-7291-2022; Al-Kuraya, Khawla/AFQ-7946-2022; Bu, Rong/JBI-9735-2023; Parvathareddy, Sandeep Kumar/JBI-9330-2023; Qadri, Syed/KYP-5564-2024; Azam, Saud/JCN-9987-2023 Azam, Saud/0000-0002-8091-5280
49 0 0 0 0 MDPI BASEL MDPI AG, Grosspeteranlage 5, CH-4052 BASEL, SWITZERLAND 2072-6694
CANCERS Cancers NOV 2024 16 21
3720 10.3390/cancers16213720
http://dx.doi.org/10.3390/cancers16213720 11
Oncology Science Citation Index Expanded (SCI-EXPANDED)
Oncology L6Z0C 39518157 gold 2025-06-24
WOS:001352166200001
J Bencina, G; Ugrekhelidze, D; Shoel, H; Oliver, E; Meiwald, A; Hughes, R; Eiden, A; Weston, G Bencina, Goran; Ugrekhelidze, Dzhumber; Shoel, Hayley; Oliver, Edward; Meiwald, Anne; Hughes, Robert; Eiden, Amanda; Weston, Georgie
The indirect costs of vaccine-preventable cancer mortality in the Middle East and North Africa (MENA) JOURNAL OF MEDICAL ECONOMICS English Article

Health economics; hepatitis B; prevention and control; indirect costs; human papillomavirus; vaccine; I18; I1; I; I10

CLINICAL-TRIALS; GLOBAL BURDEN; INFECTIONS; IMPACT

BackgroundInfections are responsible for similar to 13% of cancer cases worldwide, with human papillomavirus (HPV) and hepatitis B (HBV) among the infections associated with cancer for which vaccines are available. The aim of this study was to estimate the indirect cost of premature mortality related to cancers caused by HPV and HBV in Middle East and North Africa (MENA) countries.MethodsThe number of deaths and years of life lost (YLL) in 2019 from four HPV-related cancers: cervical cancer, oral cavity cancer, laryngeal cancer, and oropharynx cancer, as well as HBV-related liver cancer were sourced from the Institute for Health Metrics Evaluation (IHME) Global Burden of Disease database. HPV-attributable fractions were applied to deaths and YLL. The human capital approach was used to measure productivity loss, through value of YLL (VYLL), and estimated using gross domestic product per capita (World Bank; in USD). Seventeen countries in the MENA region were included. Four countries in the region were not included due to data availability.ResultsIn 2019, there were 11,645 potentially vaccine-preventable cancer-related deaths across the MENA region. This resulted in an indirect cost of $1,688,821,605, with 76.1% of this accrued in the Middle East ($1,284,923,633). The number of deaths in the Middle East (5,986) were similar to Northern Africa (5,659) but YLL were higher in Northern Africa (179,425) compared to the Middle East (169,207). The highest indirect cost per death occurred in Qatar ($1,378,991), compared to $14,962 in Sudan. Oral cavity cancer had the highest VYLL per death ($186,084).ConclusionsThere is a high burden of premature mortality and indirect costs of potentially vaccine-preventable cancer-related deaths in the MENA region. Improved vaccination program implementation, increased vaccine coverage of HPV and HBV vaccinations, and continued prioritization of public health measures, such as screening, could effectively reduce premature mortality and associated costs. [Bencina, Goran] Ctr Observat & Real World Evidence, Madrid, Spain; [Ugrekhelidze, Dzhumber] Ctr Observat & Real World Evidence, Zurich, Switzerland; [Shoel, Hayley; Oliver, Edward; Meiwald, Anne; Hughes, Robert; Weston, Georgie] Adelphi Values PROVE, Bollington, England; [Eiden, Amanda] Merck & Co Inc, Ctr Observat & Real World Evidence, Rahway, NJ USA Adelphi Group Ltd; Merck & Company; Merck & Company USAMeiwald, A (corresponding author), Adelphi Values PROVE, Adelphi Mill SK10 5JB, Bollington, England.

anne.meiwald@adelphivalues.com Merck Sharp Dohme LLC; Merck & Co., Inc., Rahway Merck Sharp Dohme LLC; Merck & Co., Inc., Rahway This work was funded by Merck Sharp & Dohme LLC, a subsidiary of Merck & Co., Inc., Rahway, NJ, USA. GB, DU, and AE are employees of Merck & Co and contributed to model validation and suggested revisions to the manuscript. 50

3 3 1 3 TAYLOR & FRANCIS LTD ABINGDON 2-4 PARK SQUARE, MILTON PARK, ABINGDON OR14 4RN, OXON, ENGLAND

1369-6998 1941-837X J MED ECON J. Med. Econ. DEC 31 2024 27 1 1036 1045

10.1080/13696998.2024.2384264

http://dx.doi.org/10.1080/13696998.2024.2384264

10 Economics; Health Care Sciences & Services; Health Policy & Services; Medicine, General & Internal Science Citation Index Expanded (SCI-EXPANDED) Business & Economics;

Health Care Sciences & Services; General & Internal Medicine
A8O0S 39046303 gold 2025-06-24
WOS:001285065200001
J Siraj, AK; Bu, R; Iqbal, K; Parvathareddy, SK; Masoodi, T; Siraj, N; Al-Rasheed, M; Kong, Y; Ahmed, SO; Al-Obaisi, KAS; Victoria, IG; Arshad, M; Al-Dayel, F; Abduljabbar, A; Ashari, LH; Al-Kuraya, KS Siraj, Abdul K.; Bu, Rong; Iqbal, Kaleem; Parvathareddy, Sandeep K.; Masoodi, Tariq; Siraj, Nabil; Al-Rasheed, Maha; Kong, Yan; Ahmed, Saeeda O.; Al-Obaisi, Khadija A. S.; Victoria, Ingrid G.; Arshad, Maham; Al-Dayel, Fouad; Abduljabbar, Alaa; Ashari, Luai H.; Al-Kuraya, Khawla S. POLE and POLD1 germline exonuclease domain pathogenic variants, a rare event in colorectal cancer from the Middle EastMOLECULAR GENETICS & GENOMIC MEDICINE
English Article
colorectal cancers; Middle East; POLD1; POLE; variant
PAPILLARY THYROID-CANCER; MUTATIONS; POLYPOSIS; DIAGNOSIS; ATTITUDES; ADENOMAS; SPECTRUM; GENE Background Colorectal cancer (CRC) is a major contributor to morbidity and mortality related to cancer. Only similar to 5% of all CRCs occur as a result of pathogenic variants in well-defined CRC predisposing genes. The frequency and effect of exonuclease domain pathogenic variants ofPOLEandPOLD1genes in Middle Eastern CRCs is still unknown. Methods Targeted capture sequencing and Sanger sequencing technologies were employed to investigate the germline exonuclease domain pathogenic variants ofPOLEandPOLD1in Middle Eastern CRCs. Immunohistochemical analysis of POLE and POLD1 was performed to look for associations between protein expression and clinico-pathological characteristics. Results Five damaging or possibly damaging variants (0.44%) were detected in 1,135 CRC cases, four inPOLEgene (0.35%, 4/1,135) and one (0.1%, 1/1,135) inPOLD1gene. Furthermore, low POLE protein expression was identified in 38.9% (417/1071) cases and a significant association with lymph node involvement (p = .0184) and grade 3 tumors (p = .0139) was observed. Whereas, low POLD1 expression was observed in 51.9% (555/1069) of cases and was significantly associated with adenocarcinoma histology (p = .0164), larger tumor size (T3 and T4 tumors;p = .0012), and stage III tumors (p = .0341). Conclusion POLEandPOLD1exonuclease domain pathogenic variants frequency in CRC cases was very low and these exonuclease domain pathogenic variants might be rare causative events of CRC in the Middle East.POLEandPOLD1can be included in multi-gene panels to screen CRC patients. [Siraj, Abdul K.; Bu, Rong; Iqbal, Kaleem; Parvathareddy, Sandeep K.; Masoodi, Tariq; Siraj, Nabil; Al-Rasheed, Maha; Kong, Yan; Ahmed, Saeeda O.; Al-Obaisi, Khadija A. S.; Victoria, Ingrid G.; Arshad, Maham; Al-Kuraya, Khawla S.] King Faisal Specialist Hosp & Res Ctr, Res Ctr, Human Canc Genom Res, Iyadh, Saudi Arabia; [Al-Dayel, Fouad] King Faisal Specialist Hosp & Res Ctr, Dept Pathol & Lab Med, Riyadh, Saudi Arabia; [Abduljabbar, Alaa; Ashari, Luai H.] King Faisal Specialist Hosp & Res Ctr, Colorectal Sect, Dept Surg, Riyadh, Saudi Arabia King Faisal Specialist Hospital & Research Center; King Faisal Specialist Hospital & Research Center; King Faisal Specialist Hospital & Research Center Al-Kuraya, KS (corresponding author), King Faisal Specialist Hosp & Res Ctr, Res Ctr, Human Canc Genom Res, MBC 98-16,POB 3,354, Riyadh 11211, Saudi Arabia.
kkuraya@kfshrc.edu.sa Alrasheed, Maha/AGZ-9897-2022; Iqbal, Kaleem/GXH-7291-2022; Siraj, Nabil/IQU-4189-2023; Bu, Rong/JBI-

9735-2023; Siraj, Abdul/IQW-1179-2023; Parvathareddy, Sandeep Kumar/JBI-9330-2023; Masoodi, Tariq/C-9843-2012; Alkuraya, Khawla/AFQ-7946-2022 Iqbal, Muhammad Kaleem/0000-0001-5634-5030; , Nabil/0000-0002-9412-5909; Masoodi, Tariq/0000-0002-9186-6349; Alkuraya, Khawla/0000-0002-4126-3419; Al Dayel, Fouad/0000-0001-6175-9051 47 11 12 0 3
WILEY HOBOKEN 111 RIVER ST, HOBOKEN 07030-5774, NJ USA
2324-9269 MOL GENET GENOM MED Mol. Genet. Genom. Med. AUG 2020 8 8
e1368 10.1002/mgg3.1368
http://dx.doi.org/10.1002/mgg3.1368 JUN 2020 11
Genetics & Heredity Science Citation Index Expanded (SCI-EXPANDED) Genetics & Heredity NG7XH 32567205 gold, Green Published 2025-06-24 WOS:000542652700001
J Soliman, AS; Schairer, C Soliman, Amr S.; Schairer, Catherine Considerations in setting up and conducting epidemiologic studies of cancer in middle- and low-income countries: the experience of a case-control study of inflammatory breast cancer in North Africa in the past 10 years
CANCER MEDICINE English Article
Breast cancer; Egypt; epidemiology; field methods; Morocco; North Africa; Tunisia This article illustrates some issues we faced during our experience in conducting an epidemiologic case-control study of inflammatory breast cancer in North Africa. We expect that some of the questions we had to ask in order to address these issues might be helpful to others in setting up epidemiologic studies in developing regions. We describe our experience from different angles including the use of multiple sites to achieve adequate sample size, standardizing diagnosis of disease, identifying cancer cases at the time of diagnosis, control selection procedures, logistics of study implementation, questionnaire development and interviewing, biologic specimens, and procedures for protection of human subjects. We have developed a brief checklist to summarize important issues for conducting future epidemiologic studies in these or similar low- or middle-income countries. [Soliman, Amr S.] Univ Michigan, Sch Publ Hlth, Dept Epidemiol, Ann Arbor, MI 48109 USA; [Schairer, Catherine] Natl Canc Inst, Div Canc Epidemiol & Genet, Bethesda, MD USA
University of Michigan System; University of Michigan; National Institutes of Health (NIH) - USA; NIH National Cancer Institute (NCI) Soliman, AS (corresponding author), Univ Nebraska Med Ctr, Coll Publ Hlth, Dept Epidemiol, 984395 Nebraska Med Ctr, Omaha, NE 68198 USA. amr.soliman@unmc.edu National Cancer Institute [R25 CA112383]; AVON Foundation [N012091]; Office of International Affairs of the National Cancer Institute; National Cancer Institute Intramural Research Program; National Cancer Institute [ZIACP010182] Funding Source: NIH RePORTER
National Cancer Institute(United States Department of Health & Human ServicesNational Institutes of Health (NIH) - USANIH National Cancer Institute (NCI)); AVON Foundation; Office of International Affairs of the National Cancer Institute; National Cancer Institute Intramural Research Program(United States Department of Health & Human ServicesNational Institutes of Health (NIH) - USANIH National Cancer Institute (NCI)); National Cancer Institute(United States Department of Health & Human ServicesNational Institutes of Health (NIH) - USANIH National Cancer Institute (NCI)) This work was supported, in part, by

grant R25 CA112383 from the National Cancer Institute, grant N012091 from the AVON Foundation, the Office of International Affairs of the National Cancer Institute, and the National Cancer Institute Intramural Research Program. 45 14 16 0 2 WILEY-BLACKWELL HOBOKEN 111 RIVER ST, HOBOKEN 07030-5774, NJ USA 2045-7634 CANCER MED-US Cancer Med. DEC 2012 1 3 338 349 10.1002/cam4.36 http://dx.doi.org/10.1002/cam4.36 12 Oncology Science Citation Index Expanded (SCI-EXPANDED) Oncology V36KG 23342283 Green Published, gold 2025-06-24 WOS:000209210400006
J Badre-Esfahani, S; Larsen, MB; Seibæk, L; Petersen, L; Blaakær, J; Andersen, B Badre-Esfahani, S.; Larsen, M. B.; Seibaek, L.; Petersen, Lk; Blaakaer, J.; Andersen, B. Low attendance by non-native women to human papillomavirus vaccination and cervical cancer screening - A Danish nationwide register-based cohort study PREVENTIVE MEDICINE REPORTS English Article
Human papilloma virus; Vaccination; Screening; Non-participation; Non-attendance; Denmark; Socio-economic status; NationalityETHNIC-MINORITY; IMMUNIZATION; EXPERIENCES; IMMIGRANTS; PREVENTION; COUNTRIES; BARRIERS; DENMARK; GIRLS
Background: Cervical cancer is preventable through human papillomavirus vaccination and cervical cancer screening. However, possibly due to systemic, individual (e.g. low socio-economic staus) and socio-cultural barriers, it is likely that non-natives, especially non-westerns, are more prone to attend neither vaccination nor screening (combined non-attendance). This is disturbing as the non-native population in Denmark is predicted to rise to 21% by 2060. We aimed to investigate differences in combined non-attendance by nativity and region of origin, and to analyse the association between country of origin and combined non-attendance adjusted for socio-economic status. Setting: 1.6.2007-31.12.2016 Denmark. Methods: Logistic regression was performed to estimate crude and adjusted odds ratios with 95% confidence intervals for combined non-attendance. Results: 170,158 women were included. Overall combined non-attendance was 11.8% [11.7-12.0]; 10.0% [9.8-10.1] for native women and 27.1% [26.4-27.7] for non-native women, with highest degrees among Middle-Eastern and North-Africans (30.1% [29.2-30.9]). Even when adjusted for socio-economics, women from Middle-East and North-Africa had substantially higher odds of combined non-attendance than natives (adj. OR = 7.5 [6.3-8.9] for Somali women). Conclusion: Denmark has a relatively low degree of combined non-attendance. However, cervical cancer preventive programmes seem to be better tailored to the needs of native women and do not appear to cater sufficiently to the needs of the fast-growing non-native populations, particularly not to the needs of Middle-Eastern and North African women. In order to secure more just cervical cancer prevention, future studies are recommended to develop tailored intervention sensitive to the need of non-native women. [Badre-Esfahani, S.; Larsen, M. B.; Andersen, B.] Randers Reg Hosp, Dept Publ Hlth Programmes, Skovlyvej 15, DK-8930 Randers NO, Denmark; [Badre-Esfahani, S.; Seibaek, L.] Aarhus Univ Hosp, Dept Gynaecol & Obstet, Palle Juul Jensens Blvd 99, DK-8200 Aarhus N, Denmark; [Petersen, Lk; Blaakaer, J.] Odense Univ Hosp, Dept Gynaecol & Obstet, JB Winslows Vej 2, DK-5000 Odense C, Denmark; [Petersen,

Lk] Univ Southern Denmark, OPEN Open Patient Data Explorat Network, Odense, Denmark; [Badre-Esfahani, S.] Aarhus Univ, Dept Clin Med, Aarhus, Denmark; [Blaakaer, J.] Univ Southern Denmark, Dept Clin Res, Odense, Denmark   Aarhus University; Aarhus University; University of Southern Denmark; Odense University Hospital; University of Southern Denmark; Aarhus University; University of Southern Denmark   Badre-Esfahani, S (corresponding author), Randers Reg Hosp, Dept Publ Hlth Programmes, Skovlyvej 15, DK-8930 Randers NO, Denmark.; Badre-Esfahani, S (corresponding author), Aarhus Univ Hosp, Dept Gynaecol & Obstet, Palle Juul Jensens Blvd 99, DK-8200 Aarhus N, Denmark.; Badre-Esfahani, S (corresponding author), Aarhus Univ, Dept Clin Med, Aarhus, Denmark.   sarbad@rm.dk   Larsen, Mette/AAD-6779-2019; Larsen, Mette Bach/T-6816-2017; Andersen, Berit/J-3402-2017; petersen, lone kjeld/R-6025-2017 Larsen, Mette Bach/0000-0002-0727-5571; Seibaek, Lene/0000-0002-2396-8669; Andersen, Berit/0000-0003-4074-6504; Koed Badre-Esfahani, Sara/0000-0003-1940-9482; petersen, lone kjeld/0000-0002-1424-6170; Badre-Esfahani, Sara/0000-0002-1500-9273; Blaakaer, Jan/0000-0003-3726-0589 Family Hede Nielsen's Foundation; Helsefonden   Family Hede Nielsen's Foundation; Helsefonden This study was funded by the Family Hede Nielsen's Foundation and Helsefonden. The sponsors had no influence on the scientific process.   62   6   6   0   5   ELSEVIER   AMSTERDAM   RADARWEG 29, 1043 NX AMSTERDAM, NETHERLANDS   2211-3355   PREV MED REP   Prev. Med. Rep.   SEP   2020   19   101106   10.1016/j.pmedr.2020.101106   http://dx.doi.org/10.1016/j.pmedr.2020.101106   7   Public, Environmental & Occupational Health   Science Citation Index Expanded (SCI-EXPANDED); Social Science Citation Index (SSCI)   Public, Environmental & Occupational Health   RV0ZB 32426214   Green Published, gold   2025-06-24   WOS:000645565500010
J   Safaeian, F; Ghaemimood, S; El-Khatib, Z; Enayati, S; Mirkazemi, R; Reeder, B   Safaeian, Fereshteh; Ghaemimood, Shidrokh; El-Khatib, Ziad; Enayati, Sahba; Mirkazemi, Roksana; Reeder, Bruce   Burden of Cervical Cancer in the Eastern Mediterranean Region During the Years 2000 and 2017: Retrospective Data Analysis of the Global Burden of Disease Study   JMIR PUBLIC HEALTH AND SURVEILLANCE   English   Article   cervical cancer; Eastern Mediterranean Region; burden of disease; cancer; burden; inequality; mortality; preventable disease   EXTENDED MIDDLE-EAST; ATTITUDES   Background: Cervical cancer is a growing health concern, especially in resource-limited settings. Objective: The objective of this study was to assess the burden of cervical cancer mortality and disability-adjusted life years (DALYs) in the Eastern Mediterranean Region (EMR) and globally between the years 2000 and 2017 by using a pooled data analysis approach. Methods: We used an ecological approach at the country level. This included extracting data from publicly available databases and linking them together in the following 3 steps: (1) extraction of data from the Global Burden of Disease (GBD) study in the years 2000 and 2017, (2) categorization of EMR countries according to the World Bank gross domestic product per capita, and (3) linking age-specific population data from the Population Statistics Division of the United Nations (20-29 years, 30-49 years, and >50 years) and GBD's data with gross national income per capita and globally extracted

data, including cervical cancer mortality and DALY numbers and rates per country. The cervical cancer mortality rate was provided by the GBD study using the following formula: number of cervical cancer deaths x 100,000/female population in the respective age group. Results: The absolute number of deaths due to cervical cancer increased from the year 2000 (n=6326) to the year 2017 (n=8537) in the EMR; however, the mortality rate due to this disease decreased from the year 2000 (2.7 per 100,000) to the year 2017 (2.5 per 100,000). According to age-specific data, the age group >= 50 years showed the highest mortality rate in both EMR countries and globally, and the age group of 20-29 years showed the lowest mortality rate both globally and in the EMR countries. Further, the rates of cervical cancer DALYs in the EMR were lower compared to the global rates (2.7 vs 6.8 in 2000 and 2.5 vs 6.8 in 2017 for mortality rate per 100,000; 95.8 vs 222.2 in 2000 and 86.3 vs 211.8 in 2017 for DALY rate per 100,000; respectively). However, the relative difference in the number of DALYs due to cervical cancer between the year 2000 and year 2017 in the EMR was higher than that reported globally (34.9 vs 24.0 for the number of deaths and 23.5 vs 18.1 for the number of DALYs, respectively). Conclusions: We found an increase in the burden of cervical cancer in the EMR as per the data on the absolute number of deaths and DALYs. Further, we found that the health care system has an increased number of cases to deal with, despite the decrease in the absolute number of deaths and DALYs. Cervical cancer is preventable if human papilloma vaccination is taken and early screening is performed. Therefore, we recommend identifying effective vaccination programs and interventions to reduce the burden of this disease. [Safaeian, Fereshteh; Ghaemimood, Shidrokh; Mirkazemi, Roksana] Bahai Inst Higher Educ, Publ Hlth Grad Studies, Tehran, Iran; [El-Khatib, Ziad] Univ Quebec Abitibi Temiscamingue, World Hlth Programme, Quebec City, PQ, Canada; [Enayati, Sahba] Kompetenzctr Gesundheit, Internal Med & Cardiol, Wels, Austria; [Reeder, Bruce] Univ Saskatchewan, Dept Community Hlth & Epidemiol, Saskatoon, SK, Canada University of Quebec; University Quebec Abitibi-Temiscamingue; University of Saskatchewan Ghaemimood, S (corresponding author), Bahai Inst Higher Educ, Publ Hlth Grad Studies, Tehran, Iran.
shidrokh.ghaemi@gmail.com El-Khatib, Ziad/B-5161-2017; Mirkazemi, Roksana/LFT-3869-2024 Reeder, Bruce/0000-0001-9079-514X; Mirkazemi, Roksana/0000-0001-7749-1096
28 11 12 0 22 JMIR PUBLICATIONS, INC TORONTO
130 QUEENS QUAY East, Unit 1100, TORONTO, ON M5A 0P6, CANADA
2369-2960 JMIR PUBLIC HLTH SUR JMIR Public Health Surveill. MAY 2021 7 5
e22160 10.2196/22160
http://dx.doi.org/10.2196/22160 17 Public, Environmental & Occupational Health Science Citation Index Expanded (SCI-EXPANDED); Social Science Citation Index (SSCI)
Public, Environmental & Occupational Health SP1HO 33978592
Green Published, gold 2025-06-24
WOS:000659422700020
J Schairer, C; Hablas, A; Eldein, IAS; Gaafar, R; Rais, H; Mezlini, A; Ben Ayed, F; Ben Ayoub, W; Benider, A; Tahri, A; Khouchani, M; Aboulazm, D; Karkouri, M; Eissa, S; Pfeiffer, RM; Gadalla, SM; Swain, SM; Merajver, SD; Brown, LM; Soliman, AS
Schairer, Catherine; Hablas, Ahmed; Eldein, Ibrahim AbdelBar Seif; Gaafar, Rabab; Rais, Henda; Mezlini, Amel;

Ben Ayed, Farhat; Ben Ayoub, Wided; Benider, Abdellatif; Tahri, Ali; Khouchani, Mouna; Aboulazm, Dalia; Karkouri, Mehdi; Eissa, Saad; Pfeiffer, Ruth M.; Gadalla, Shahinaz M.; Swain, Sandra M.; Merajver, Sofia D.; Brown, Linda Morris; Soliman, Amr S.

Clinico-pathologic and mammographic characteristics of inflammatory and non-inflammatory breast cancer at six centers in North Africa   BREAST CANCER RESEARCH AND TREATMENT

English   Article

Inflammatory breast cancer; Diagnostic criteria; Egypt; Tunisia; Morocco CARCINOMA; DIAGNOSIS; CONSENSUS; TUNISIA; PROGRAM

PurposeWe describe the clinico-pathologic and mammographic characteristics of inflammatory breast cancer (IBC) and non-IBC cases enrolled in a case-control study. Because IBC is a clinico-pathologic entity with rapid appearance of erythema and other signs, its diagnosis is based on clinical observation and thus, by necessity, subjective. Therefore, we evaluate our cases by photographic review by outside expert clinicians and by degree of adherence to the two most recent definitions of IBC: the international expert panel consensus statement and American Joint Committee on Cancer (AJCC) 8th edition (we used the slightly less restrictive 7th edition definition for our study).MethodsWe enrolled 267 IBC and 274 age- and geographically matched non-IBC cases at 6 sites in Egypt, Tunisia, and Morocco in a case-control study of IBC conducted between 2009 and 2015. We collected clinico-pathologic and mammographic data and standardized medical photographs of the breast.ResultsWe identified many differences between IBC and non-IBC cases: 54.5% versus 68.8% were estrogen receptor-positive, 39.9% versus 14.8% human epidermal growth factor receptor 2-positive, 91% versus 4% exhibited erythema, 63% versus 97% had a mass, and 57% versus 10% had mammographic evidence of skin thickening. Seventy-six percent of IBC cases adhered to the expert panel consensus statement and 36% to the AJCC definition; 86 percent were confirmed as IBC by either photographic review or adherence to the consensus statement.ConclusionsWe successfully identified distinct groups of IBC and non-IBC cases. The reliability of IBC diagnosis would benefit from expert review of standardized medical photographs and associated clinical information. [Schairer, Catherine; Pfeiffer, Ruth M.; Gadalla, Shahinaz M.] NCI, Div Canc Epidemiol & Genet, NIH, Bethesda, MD 20892 USA; [Schairer, Catherine] NCI, 9609 Med Ctr Dr,Rm 6E340, Rockville, MD 20850 USA; [Hablas, Ahmed; Eldein, Ibrahim AbdelBar Seif] Gharbiah Canc Soc, Tanta, Egypt; [Gaafar, Rabab; Aboulazm, Dalia; Eissa, Saad] Natl Canc Inst, Cairo, Egypt; [Rais, Henda; Mezlini, Amel; Ben Ayoub, Wided] Inst Salah Azaiz, Tunis, Tunisia; [Ben Ayed, Farhat] Assoc Fight Canc, Tunis, Tunisia; [Benider, Abdellatif; Karkouri, Mehdi] Ibn Rochd Oncol Ctr, Casablanca, Morocco; [Tahri, Ali] Clin Specialisee Menara, Marrakech, Morocco; [Khouchani, Mouna] Univ Hosp Ctr Mohammed VI, Marrakech, Morocco; [Swain, Sandra M.] Georgetown Univ, Lombardi Comprehens Canc Ctr, Washington, DC USA; [Merajver, Sofia D.] Univ Michigan, Ann Arbor, MI 48109 USA; [Brown, Linda Morris] RTI Int, Rockville, MD USA; [Soliman, Amr S.] CUNY, Sch Med, New York, NY 10031 USA National Institutes of Health (NIH) - USA; NIH National Cancer Institute (NCI); NIH National Cancer Institute-Division of Cancer Epidemiology & Genetics; National Institutes of Health (NIH) - USA; NIH National Cancer Institute (NCI); Egyptian Knowledge Bank (EKB); National Cancer Institute - Egypt; Cairo University; Universite de Tunis-El-Manar; Institut Salah Azaiez;

Hassan II University of Casablanca; Ibn Rochd University Hospital Center of Casablanca; Mohammed VI University Hospital Center of Marrakech; Cadi Ayyad University of Marrakech; Georgetown University; University of Michigan System; University of Michigan; Research Triangle Institute; City University of New York (CUNY) System Schairer, C (corresponding author), NCI, Div Canc Epidemiol & Genet, NIH, Bethesda, MD 20892 USA.; Schairer, C (corresponding author), NCI, 9609 Med Ctr Dr,Rm 6E340, Rockville, MD 20850 USA. schairec@exchange.nih.gov Hablas, Ahmed/AFK-5458-2022; Pfeiffer, Ruth/F-4748-2011; Gadalla, Shahinaz/C-4005-2015 Swain, Sandra/0000-0002-1320-3830; Gadalla, Shahinaz/0000-0002-3255-8143; Brown, Linda/0000-0002-2181-9627 National Cancer Institute Intramural Research Program; Breast Cancer Research Foundation; Metavivor Foundation; Cancer Epidemiology Education in Special Populations (CEESP) Program [R25 CA112383]; National Cancer Institute [ZIACP010182] Funding Source: NIH RePORTER
National Cancer Institute Intramural Research Program(United States Department of Health & Human ServicesNational Institutes of Health (NIH) - USANIH National Cancer Institute (NCI)); Breast Cancer Research Foundation; Metavivor Foundation(ACEV Foundation); Cancer Epidemiology Education in Special Populations (CEESP) Program; National Cancer Institute(United States Department of Health & Human ServicesNational Institutes of Health (NIH) - USANIH National Cancer Institute (NCI)) The National Cancer Institute Intramural Research Program (CS), the Breast Cancer Research Foundation (SDM), the Metavivor Foundation (SDM), The Cancer Epidemiology Education in Special Populations (CEESP) Program-Grant R25 CA112383 (ASS). 28 13 15 0 3
SPRINGER NEW YORK 233 SPRING ST, NEW YORK, NY 10013 USA
0167-6806 1573-7217 BREAST CANCER RES TR Breast Cancer Res. Treat. JUL 2019 176 2
407 417 10.1007/s10549-019-05237-5
http://dx.doi.org/10.1007/s10549-019-05237-5 11
Oncology Science Citation Index Expanded (SCI-EXPANDED)
Oncology IC1RT 31006821 Green Accepted
2025-06-24 WOS:000470737500017
J Petimar, J; Park, YMM; Smith-Warner, SA; Fung, TT; Sandler, DP Petimar, Joshua; Park, Yong-Moon Mark; Smith-Warner, Stephanie A.; Fung, Teresa T.; Sandler, Dale P.
Dietary index scores and invasive breast cancer risk among women with a family history of breast cancer AMERICAN JOURNAL OF CLINICAL NUTRITION English Article
breast cancer; dietary index; cohort studies; DASH diet; Mediterranean diet; Alternative Healthy Eating Index DASH-STYLE DIET; MEDITERRANEAN DIET; POOLED ANALYSIS; REGRESSION-MODELS; LIFE-STYLE; ADHERENCE; CONSUMPTION; DISEASE; QUALITY; METAANALYSIS Background: Many epidemiologic studies have analyzed the relations of individual foods and nutrients and breast cancer risk with inconsistent results. Few studies have examined recommendation-based dietary indices and breast cancer risk. Objective: The aim of this study was to determine associations between recommendation-based dietary index scores and incident invasive breast cancer. Methods: The Sister Study is a prospective cohort of 50,884 US women (baseline: 2003-2009) who had a sister with breast cancer but no prior breast cancer themselves. We created scores for the Dietary Approaches to Stop Hypertension (DASH) diet, Alternative Mediterranean Diet (AMED), and Alternative Healthy Eating Index-2010 (AHEI-2010) from

dietary intakes estimated by a baseline-validated Block food-frequency questionnaire (FFQ). We used Cox regression to estimate multivariable-adjusted HRs and 95% CIs for total invasive breast cancer risk and by estrogen receptor (ER), progesterone receptor (PR), and human epidermal growth factor receptor-2 (HER2) status. Results: We documented 1,700 invasive breast cancer cases through 2015 (mean follow-up, 7.6 y). Individuals in the highest quartile of DASH scores had a lower risk of invasive breast cancer compared with those in the lowest quartile (HR: 0.78; 95% CI: 0.67, 0.90; P-trend = 0.001), with stronger associations for ER(HR: 0.61; 95% CI: 0.40, 0.94; P-trend = 0.006) as well as ER-/PR- and ER-/PR-/HER2-subtypes. AHEI-2010 (HR for highest compared with lowest quartile: 0.90; 95% CI: 0.78, 1.03; P-trend = 0.15) and AMED (HR for highest compared with lowest quartile: 0.90; 95% CI: 0.77, 1.06; P-trend = 0.07) were weakly and nonsignificantly associated with breast cancer risk, but after excluding alcohol, AHEI-2010 was inversely associated with risk of ER-/PR-(HR: 0.64; 95% CI: 0.42, 0.98; Ptrend = 0.04) and ER-/PR-/HER2-subtypes. We did not observe any significant interactions by menopausal status or other participant characteristics. Conclusions: DASH scores were inversely associated with breast cancer risk; DASH and AHEI-2010 scores excluding alcohol were particularly inversely associated with risk of ER-/PR-and ER-/PR/HER2-breast cancers. This trial was registered at clinicaltrials.gov as NCT00047970. [Petimar, Joshua; Smith-Warner, Stephanie A.; Fung, Teresa T.] Harvard TH Chan Sch Publ Hlth, Dept Nutr, Boston, MA USA; [Petimar, Joshua; Smith-Warner, Stephanie A.] Harvard TH Chan Sch Publ Hlth, Dept Epidemiol, Boston, MA USA; [Park, Yong-Moon Mark; Sandler, Dale P.] NIEHS, Epidemiol Branch, NIH, Res Triangle Pk, NC 27709 USA; [Fung, Teresa T.] Simmons Univ, Dept Nutr, Boston, MA USA

Harvard University; Harvard T.H. Chan School of Public Health; Harvard University; Harvard T.H. Chan School of Public Health; National Institutes of Health (NIH) - USA; NIH National Institute of Environmental Health Sciences (NIEHS); Simmons University Park, YMM; Sandler, DP (corresponding author), NIEHS, Epidemiol Branch, NIH, Res Triangle Pk, NC 27709 USA.

mark.park@nih.gov; sandler@niehs.nih.gov PARK, YONG-MOON ("MARK")/ABA-2765-2021; Sandler, Dale/E-5110-2019 PARK, YONG-MOON ("MARK")/0000-0002-5879-6879; Fung, Teresa/0000-0002-0159-482X; Sandler, Dale/0000-0002-6776-0018 Intramural Research Program of the NIH, National Institute of Environmental Health Sciences [ZO1-ES-044005]; Breast Cancer Research Foundation award; [T32 HL 098048] Intramural Research Program of the NIH, National Institute of Environmental Health Sciences(United States Department of Health & Human ServicesNational Institutes of Health (NIH) - USANIH National Institute of Environmental Health Sciences (NIEHS)); Breast Cancer Research Foundation award; This work was funded, in part, by the Intramural Research Program of the NIH, National Institute of Environmental Health Sciences (ZO1-ES-044005 to DPS). JP is supported by T32 HL 098048. SAS-W is supported by a Breast Cancer Research Foundation award. 56

28 30 0 4 OXFORD UNIV PRESS OXFORD

GREAT CLARENDON ST, OXFORD OX2 6DP, ENGLAND 0002-9165

1938-3207 AM J CLIN NUTR Am. J. Clin. Nutr. MAY

2019 109 5 1393 1401

10.1093/ajcn/nqy392 http://dx.doi.org/10.1093/ajcn/nqy392

9 Nutrition & Dietetics Science Citation Index Expanded (SCI-EXPANDED) Nutrition & Dietetics IM5WZ

30968114	Green Published, hybrid			2025-06-24	WOS:000478066000018
J	Castelló, A; Boldo, E; Pérez-Gómez, B; Lope, V; Altzibar, JM; Martín, V; Castaño-Vinyals, G; Guevara, M; Dierssen-Sotos, T; Tardón, A; Moreno, V; Puig-Vives, M; Llorens-Ivorra, C; Alguacil, J; Gómez-Acebo, I; Castilla, J; Gracìa-Lavedán, E; Dávila-Batista, V; Kogevinas, M; Aragonés, N; Amiano, P; Pollán, M
	Castello, Adela; Boldo, Elena; Perez-Gomez, Beatriz; Lope, Virginia; Altzibar, Jone M.; Martin, Vicente; Castano-Vinyals, Gemma; Guevara, Marcela; Dierssen-Sotos, Trinidad; Tardon, Adonina; Moreno, Victor; Puig-Vives, Montserrat; Llorens-Ivorra, Cristobal; Alguacil, Juan; Gomez-Acebo, Ines; Castilla, Jesus; Gracia-Lavedan, Esther; Davila-Batista, Veronica; Kogevinas, Manolis; Aragones, Nuria; Amiano, Pilar; Pollan, Marina
	Adherence to the Western, Prudent and Mediterranean dietary patterns and breast cancer risk: MCC-Spain study	MATURITAS
		English	Article
	Mediterranean diet; Western diet; Breast neoplasms; Prevention and control; Population attributable fraction	FOOD; AMERICAN; SUBTYPES	Objective: To externally validate the previously identified effect on breast cancer risk of the Western, Prudent and Mediterranean dietary patterns. Study design: MCC-Spain is a multicase-control study that collected epidemiological information on 1181 incident cases of female breast cancer and 1682 healthy controls from 10 Spanish provinces. Three dietary patterns derived in another Spanish case-control study were analysed in the MCC-Spain study. These patterns were termed Western (high intakes of fatty and sugary products and red and processed meat), Prudent (high intakes of low-fat dairy products, vegetables, fruits, whole grains and juices) and Mediterranean (high intake of fish, vegetables, legumes, boiled potatoes, fruits, olives, and vegetable oil, and a low intake of juices). Their association with breast cancer was assessed using logistic regression models with random province-specific intercepts considering an interaction with menopausal status. Risk according to tumour subtypes based on oestrogen (ER), progesterone (PR) and human epidermal growth factor 2 (HER2) receptors (ER +/PR + HER2; HER2+; ER- /PR- & HER2-) -was evaluated with multinomial regression models. Main outcome measures: Breast cancer and histological subtype. Results: Our results confirm most of the associations found in the previous case-control study. A high adherence to the Western dietary pattern seems to increase breast cancer risk in both premenopausal women (OR4th vs 1st quartile (95% CI):1.68 (1.02;2.79); ORISD-increase (95% CI):1.19 (1.02;1.40)) and postmenopausal women (OR4th vs 1st quartile(95% CI):1.48(1.07;2.05); ORISD-increase(95% CO: 1.14 (1.01;1.29)). While high adherence to the Prudent pattern did not show any effect on breast cancer, the Mediterranean dietary pattern seemed to be protective, but only among postmenopausal women (OR4th vs 1st quartile (95% CI): 0.72 (95% CI 0.53;0.98); p-int = 0.075). There were no significant differences by tumour subtype. Conclusion: Dietary recommendations based on a departure from the Western dietary pattern in favour of the Mediterranean diet could reduce breast cancer risk in the general population.
	[Castello, Adela; Boldo, Elena; Perez-Gomez, Beatriz; Lope, Virginia; Aragones, Nuria; Pollan, Marina] Carlos III Inst Hlth, Natl Ctr Epidemiol, Canc Epidemiol Unit, Ave Monforte de Lemos 5, Madrid 28029, Spain; [Castello, Adela; Boldo, Elena; Perez-Gomez,

Beatriz; Lope, Virginia; Altzibar, Jone M.; Castano-Vinyals, Gemma; Dierssen-Sotos, Trinidad; Tardon, Adonina; Moreno, Victor; Gracia-Lavedan, Esther; Kogevinas, Manolis; Aragones, Nuria; Amiano, Pilar; Pollan, Marina] Carlos III Inst Hlth, Consortium Biomed Res Epidemiol & Publ Hlth CIBER, Ave Monforte de Lemos 5, Madrid 28029, Spain; [Castello, Adela; Boldo, Elena; Perez-Gomez, Beatriz; Lope, Virginia; Aragones, Nuria; Pollan, Marina] IIS Puerta Hierro IDIPHIM, Oncol & Hematol Area, Canc Epidemiol Res Grp, Manuel Falla 1, Madrid 28222, Spain; [Altzibar, Jone M.] Osakidetza Hlth Basque Reg Serv, Breast Canc Screening Program, Ave Navarra 4, San Sebastian 20013, Spain; [Martin, Vicente] Univ Leon, Grp Invest Interacc Gen Ambiente & Salud, Campus Vegazana S-N, E-24071 Leon, Spain; [Castano-Vinyals, Gemma; Gracia-Lavedan, Esther; Kogevinas, Manolis] ISGlobal, Ctr Res Environm Epidemiol CREAL, Carrer Doctor Aiguader 88, Barcelona 08003, Spain; [Castano-Vinyals, Gemma; Gracia-Lavedan, Esther; Kogevinas, Manolis] Univ Pompeu Fabra, Carrer Doctor Aiguader 88, Barcelona 08003, Spain; [Castano-Vinyals, Gemma; Kogevinas, Manolis] Hosp del Mar Med Res Inst IMIM, Carrer Doctor Aiguader 88, Barcelona 08003, Spain; [Guevara, Marcela; Castilla, Jesus] Publ Hlth Inst Navarra, Early Detect Sect, Calle Leyre 15, Pamplona 31003, Spain; [Dierssen-Sotos, Trinidad; Gomez-Acebo, Ines] Univ Cantabria, IDIVAL, Ave Cardenal Herrera Oria S-N, Santander 39011, Spain; [Tardon, Adonina] Univ Oviedo, Fac Med, Inst Univ Oncol, Planta 7,Campus El Cristo B, E-33006 Oviedo, Spain; [Moreno, Victor] IDIBELL Catalan Inst Oncol, Gran Via Km 2-7, Lhospitalet De Llobregat 08907, Spain; [Moreno, Victor] Univ Barcelona, Fac Med, Dept Clin Sci, Campus Bellvitge,Feixa Llarga S-N, Lhospitalet De Llobregat 08907, Spain; [Puig-Vives, Montserrat] Autonomous Govt Catalonia, Girona Biomed Res Inst IdiBGi, Catalan Inst Oncol, Epidemiol Unit,Oncol Coordinat Plan,Dept Hlth, Girona 17004, Spain; [Puig-Vives, Montserrat] Autonomous Govt Catalonia, Girona Biomed Res Inst IdiBGi, Catalan Inst Oncol, Girona Canc Registry,Oncol Coordinat Plan,Dept Hl, Girona 17004, Spain; [Llorens-Ivorra, Cristobal] Conselleria Sanidad Universal & Salud Pabl, Ctr Salud Publ Denia, Plaza Jaime 1,5, Denia 03700, Spain; [Llorens-Ivorra, Cristobal] Fdn El Fomento Invest Sanitaria & Biomed Comunida, Avda Cataluna 21, Valencia 46020, Spain; [Alguacil, Juan] Univ Huelva, Ctr Invest Salud & Medio Ambiente CYSMA, Campus Univ El Carmen, Huelva 21071, Spain; [Amiano, Pilar] Govt Basque Country, Publ Hlth Dept Gipuzkoa, Ave Navarra 4, San Sebastian 20013, Spain; [Amiano, Pilar] Biodonostia Res Inst, Paseo Dr Beguiristain S-N, San Sebastian 20014, Spain   Instituto de Salud Carlos III; CIBER - Centro de Investigacion Biomedica en Red; CIBERESP; Instituto de Salud Carlos III; Universidad de Leon; Pompeu Fabra University; Centre de Recerca en Epidemiologia Ambiental (CREAL); ISGlobal; Pompeu Fabra University; Hospital del Mar Research Institute; Hospital del Mar; Public Health Institute of Navarra; Universidad de Cantabria; University of Oviedo; Institut Catala d'Oncologia; Institut d'Investigacio Biomedica de Bellvitge (IDIBELL); University of Barcelona; Universitat de Girona; Girona University Hospital Dr. Josep Trueta; Institut d'Investigacio Biomedica de Girona (IDIBGI); Institut Catala d'Oncologia; Universitat de Girona; Girona University Hospital Dr. Josep Trueta; Institut d'Investigacio Biomedica de Girona (IDIBGI); Institut Catala d'Oncologia; Universidad de Huelva; Basque Government   Castelló, A (corresponding author), Carlos III Inst Hlth, Natl Ctr Epidemiol, Canc Epidemiol Unit, Ave

Monforte de Lemos 5, Madrid 28029, Spain. acastello@isciii.es; eiboldo@isciii.es; bperez@isciii.es; vicarvajal@isciii.es; JONEMIREN.ALTZIBARAROTZENA@osakidetza.eus; vicente.martin@unileon.es; gemma.castano@isglobal.orgl; mguevare@cfnavarra.es; dierssent@unican.es; atardon@uniovi.es; v.moreno@iconcologia.net; mpuig.icogirona@gmail.com; llorens_cri@gva.es; alguacil@dbasp.uhu.es; ines.gomez@unican.es; jesus.castilla.catalan@cfnavarra.es; esther.gracia@isglobal.org; vdavb@unileon.es; manolis.kogevinas@isglobal.org; naragones@isciii.es; epicss-san@ej-gv.es; mpollan@isciii.es

Lopez-Garcia, Esther/A-8894-2019; Kogevinas, Manolis/C-3918-2017; Batista, Veronica/E-8082-2017; Boldo, Elena/L-6610-2014; Castello, Adela/C-3829-2014; Castilla, Jesus/B-9048-2008; Moreno, Victor/A-1697-2010; Martin, Vicente/A-1597-2008; Castano-Vinyals, Gemma/H-7021-2017; Perez-Gomez, Beatriz/C-4715-2012; Aragones, Nuria/O-5962-2015; Guevara, Marcela/B-4473-2015; Lope, Virginia/S-4774-2016; Alguacil Ojeda, Juan/D-4463-2019; Gomez-Acebo, Ines/C-9122-2018; Tardon, Adonina/B-6563-2014; Pollan, Marina/M-3259-2014

ALTZIBAR, JONE MIREN/0000-0001-7037-7944; Castello, Adela/0000-0002-1308-9927; Castilla, Jesus/0000-0002-6396-7265; Moreno, Victor/0000-0002-2818-5487; Martin, Vicente/0000-0003-0552-2804; Castano-Vinyals, Gemma/0000-0003-4468-1816; Perez-Gomez, Beatriz/0000-0002-4299-8214; Aragones, Nuria/0000-0003-0983-2156; Guevara, Marcela/0000-0001-9242-6364; Lope, Virginia/0000-0002-6986-4021; Davila Batista, Veronica/0000-0001-8888-395X; Gracia-Lavedan, Esther/0000-0002-0104-3980; Dierssen-Sotos, Trinidad/0000-0002-6127-0077; Alguacil Ojeda, Juan/0000-0003-2703-9725; Gomez-Acebo, Ines/0000-0001-8793-8314; Tardon, Adonina/0000-0001-5150-1209; Pollan, Marina/0000-0002-4328-1565

Carlos III Institute of Health grants [PI12/00488, PI12/00265, PI12/00715, PI12/01270, PI09/00773, PI08/1770]; Spanish Ministry of Economy and Competitiveness [IJCI-2014-20900]; Consejeria de Salud de la Junta de Andalucia [PI-0571-2009, PI-0306-2011]; Spanish Federation of Breast Cancer Patients [FECMA: EPY 1169-10]; Association of Women with Breast Cancer from Elche [AMACMEC: EPY 1394/15]; Marques de Valdecilla foundation [API 10/09]; Accion Transversal del Cancer Carlos III Institute of Health grants; Spanish Ministry of Economy and Competitiveness(Spanish Government); Consejeria de Salud de la Junta de Andalucia(Junta de Andalucia); Spanish Federation of Breast Cancer Patients; Association of Women with Breast Cancer from Elche; Marques de Valdecilla foundation; Accion Transversal del Cancer(Instituto de Salud Carlos III) The study was funded by Carlos III Institute of Health grants (PI12/00488, PI12/00265, PI12/00715, PI12/01270, PI09/00773 and PI08/1770), by the Spanish Ministry of Economy and Competitiveness (IJCI-2014-20900) and by Consejeria de Salud de la Junta de Andalucia (PI-0571-2009 and PI-0306-2011) competitive calls including peer review for scientific quality. Additional funding was provided by the Spanish Federation of Breast Cancer Patients (FECMA: EPY 1169-10), the Association of Women with Breast Cancer from Elche (AMACMEC: EPY 1394/15), the Marques de Valdecilla foundation (grant API 10/09), and by Accion Transversal del Cancer, approved by the Spanish Ministry Council on October 11, 2007. None of the funders played any role in conducting research or writing the paper. 31 61 67

1 34 ELSEVIER IRELAND LTD CLARE ELSEVIER HOUSE, BROOKVALE PLAZA, EAST PARK SHANNON, CO, CLARE, 00000, IRELAND

0378-5122 1873-4111 MATURITAS Maturitas SEP 2017

103 8 15
10.1016/j.maturitas.2017.06.020
http://dx.doi.org/10.1016/j.maturitas.2017.06.020
8 Geriatrics & Gerontology; Obstetrics & Gynecology
Science Citation Index Expanded (SCI-EXPANDED)
Geriatrics & Gerontology; Obstetrics & Gynecology FD6UR
28778338 Green Accepted 2025-06-24
WOS:000407663900003
J Cao, S; Liu, LC; Zhu, QR; Zhu, Z; Zhou, JY; Wei, PM; Wu, M Cao, Shang; Liu, Linchen; Zhu, Qianrang; Zhu, Zheng; Zhou, Jinyi; Wei, Pingmin; Wu, Ming
Adherence to the Vegetable-Fruit-Soy Dietary Pattern, a Reference From Mediterranean Diet, Protects Against Postmenopausal Breast Cancer Among Chinese Women FRONTIERS IN NUTRITION
English Article breast cancer; Mediterranean diet; cancer prevention; molecular subtype; vegetable-fruit-soy diet POOLED ANALYSIS; RISK; CAROTENOIDS; MORTALITY; CONSUMPTION; NUTRITION; SURVIVAL; QUALITY; HABITS; COHORT
BackgroundThe diet-center hypothesis has gained much support from the apparent protective effect of the Mediterranean diet on breast cancer. However, the evidence of the association between Mediterranean diet adherence and breast cancer molecular subtypes remains small, especially in non-Mediterranean populations. MethodsThe subjects from the Chinese Wuxi Exposure and Breast Cancer Study, a population-based case-control study, included 818 patients and 935 healthy controls. A validated food frequency questionnaire used for diet assessment and a modified version of the alternate Mediterranean Diet Score, which is called the alternate Chinese Diet Score, was developed to assess adherence to a migrated Chinese version of the Mediterranean diet, which we called the vegetable-fruit-soy dietary pattern. Soy foods, rapeseed oil, and coarse cereals replaced legumes, olive oil, and whole grains reflecting the cuisine of the region. We examined the association between the vegetable-fruit-soy diet adherence and breast cancer risk, stratified by menopause status (pre- or postmenopausal) and receptor status [estrogen-receptor (ER), progesterone-receptor (PR) status, and human epidermal growth factor 2 (HER2)] oncogene expression, followed by five specific combinations (ER+, ER-, ER+/PR+,ER-/PR-, and ER-/PR-/HER2-). ResultsThe results suggest that the vegetable-fruit-soy dietary pattern was inversely associated with postmenopausal breast cancer risk [4th vs. 1st quartile, odds ratio (OR) = 0.57, 95%CI = 0.41, 0.80; P trend < 0.001] and that the inverse association was somewhat stronger to detect among ER- subtypes (OR = 0.63; 95%CI = 0.37, 0.94; P trend = 0.003) and ER-/PR-subtypes (OR = 0.64; 95%CI = 0.41, 0.93; P trend = 0.012). We did not observe any significant association between the vegetable-fruit-soy diet characteristics and ER+ subtype, as well as between PR+ and ER+/PR+ subtypes. ConclusionThe favorable influence from the Mediterranean diet may also apply to Chinese women. The vegetable-fruit-soy dietary pattern may reduce the risk of postmenopausal breast cancer, particularly among ER- subtype, and ER-/PR-subtype.
[Cao, Shang; Wei, Pingmin; Wu, Ming] Southeast Univ, Dept Epidemiol & Hlth Stat, Nanjing, Peoples R China; [Liu, Linchen] Southeast Univ, Zhongda Hosp, Sch Med, Dept Rheumatol, Nanjing, Peoples R China; [Zhu, Qianrang; Zhu, Zheng; Zhou, Jinyi; Wu, Ming] Jiangsu Prov Ctr Dis Control & Prevent, Dept Chron Dis Control, Nanjing, Peoples R China Southeast University - China;

Southeast University - China; Jiangsu Provincial Center for Disease Control & PreventionWu, M (corresponding author), Southeast Univ, Dept Epidemiol & Hlth Stat, Nanjing, Peoples R China.; Wu, M (corresponding author), Jiangsu Prov Ctr Dis Control & Prevent, Dept Chron Dis Control, Nanjing, Peoples R China.
mingwu@seu.edu.cn QIANRANG, ZHU/JOJ-6927-2023
World Cancer Research Fund [2011/RFA/473] World Cancer Research Fund(World Cancer Research Fund International (WCRF))
This study was supported by World Cancer Research Fund (2011/RFA/473). 43 9 9 3 27 FRONTIERS MEDIA SA LAUSANNE AVENUE DU TRIBUNAL FEDERAL 34, LAUSANNE, CH-1015, SWITZERLAND 2296-861X FRONT NUTR Front. Nutr. MAR 29 2022 9 800996
10.3389/fnut.2022.800996
http://dx.doi.org/10.3389/fnut.2022.800996 12
Nutrition & Dietetics Science Citation Index Expanded (SCI-EXPANDED) Nutrition & Dietetics 0T7RD 35425800 gold, Green Published 2025-06-24 WOS:000787161000001
J Castro-Espin, C; Bonet, C; Crous-Bou, M; Nadal-Zaragoza, N; Tjonneland, A; Mellemkjær, L; Hajji-Louati, M; Truong, T; Katzke, V; Le Cornet, C; Schulze, MB; Jannasch, F; Masala, G; Sieri, S; Panico, S; Di Girolamo, C; Skeie, G; Borch, KB; Olsen, KS; Sánchez, MJ; Amiano, P; Chirlaque, MD; Guevara, M; Sund, M; Bodén, S; Gunter, MJ; Gonzalez-Gil, EM; Weiderpass, E; Aguilera-Buenosvinos, I; Tsilidis, KK; Heath, AK; Aune, D; Dossus, L; Agudo, A Castro-Espin, Carlota; Bonet, Catalina; Crous-Bou, Marta; Nadal-Zaragoza, Nuria; Tjonneland, Anne; Mellemkjaer, Lene; Hajji-Louati, Mariem; Truong, Therese; Katzke, Verena; Le Cornet, Charlotte; Schulze, Matthias B.; Jannasch, Franziska; Masala, Giovanna; Sieri, Sabina; Panico, Salvatore; Di Girolamo, Chiara; Skeie, Guri; Borch, Kristin Benjaminsen; Olsen, Karina Standahl; Sanchez, Maria-Jose; Amiano, Pilar; Chirlaque, Maria-Dolores; Guevara, Marcela; Sund, Malin; Boden, Stina; Gunter, Marc J.; Gonzalez-Gil, Esther M.; Weiderpass, Elisabete; Aguilera-Buenosvinos, Inmaculada; Tsilidis, Kostas K.; Heath, Alicia K.; Aune, Dagfinn; Dossus, Laure; Agudo, Antonio Association of Mediterranean diet with survival after breast cancer diagnosis in women from nine European countries: results from the EPIC cohort study BMC MEDICINE English Article
Mediterranean diet; Breast cancer; Cancer survivors; Dietary patterns; Prospective studies ADHERENCE; DEATH; RISK
BackgroundThe Mediterranean diet has been associated with lower risk of breast cancer (BC) but evidence from prospective studies on the role of Mediterranean diet on BC survival remains sparse and conflicting. We aimed to investigate whether adherence to Mediterranean diet prior to diagnosis is associated with overall and BC-specific mortality.MethodsA total of 13,270 incident breast cancer cases were identified from an initial sample of 318,686 women in 9 countries from the European Prospective Investigation into Cancer and Nutrition (EPIC) study. Adherence to Mediterranean diet was estimated through the adapted relative Mediterranean diet (arMED), a 16-point score that includes 8 key components of the Mediterranean diet and excludes alcohol. The degree of adherence to arMED was classified as low (score 0-5), medium (score 6-8), and high (score 9-16). Multivariable Cox proportional hazards models were used to analyze the association between the arMED score and overall mortality, and

Fine-Gray competing risks models were applied for BC-specific mortality.ResultsAfter a mean follow-up of 8.6 years from diagnosis, 2340 women died, including 1475 from breast cancer. Among all BC survivors, low compared to medium adherence to arMED score was associated with a 13% higher risk of all-cause mortality (HR 1.13, 95%CI 1.01-1.26). High compared to medium adherence to arMED showed a non-statistically significant association (HR 0.94; 95% CI 0.84-1.05). With no statistically significant departures from linearity, on a continuous scale, a 3-unit increase in the arMED score was associated with an 8% reduced risk of overall mortality (HR3-unit 0.92, 95% CI: 0.87-0.97). This result sustained when restricted to postmenopausal women and was stronger among metastatic BC cases (HR3-unit 0.81, 95% CI: 0.72-0.91).ConclusionsConsuming a Mediterranean diet before BC diagnosis may improve long-term prognosis, particularly after menopause and in cases of metastatic breast cancer. Well-designed dietary interventions are needed to confirm these findings and define specific dietary recommendations. [Castro-Espin, Carlota; Bonet, Catalina; Crous-Bou, Marta; Nadal-Zaragoza, Nuria; Agudo, Antonio] Catalan Inst Oncol ICO, Unit Nutr & Canc, Lhospitalet De Llobregat, Spain; [Castro-Espin, Carlota; Bonet, Catalina; Crous-Bou, Marta; Nadal-Zaragoza, Nuria; Agudo, Antonio] Bellvitge Bio Med Res Inst IDIBELL, Nutr & Canc Grp, Epidemiol Publ Hlth Canc Prevent & Palliat Care P, Lhospitalet De Llobregat, Spain; [Crous-Bou, Marta] Harvard T H Chan Sch Publ Hlth, Dept Epidemiol, Boston, MA 02115 USA; [Tjonneland, Anne; Mellemkjaer, Lene] Danish Canc Soc Res Ctr, Copenhagen, Denmark; [Tjonneland, Anne] Univ Copenhagen, Dept Publ Hlth, Copenhagen, Denmark; [Hajji-Louati, Mariem; Truong, Therese] Univ Paris Saclay, UVSQ, Inserm Exposome Hered Canc & Hlth Team, CESP U1018,Gustave Roussy, Villejuif, France; [Katzke, Verena; Le Cornet, Charlotte] German Canc Res Ctr, Heidelberg, Germany; [Schulze, Matthias B.; Jannasch, Franziska] German Inst Human Nutr Potsdam Rehbrucke, Dept Mol Epidemiol, Nuthetal, Germany; [Schulze, Matthias B.] Univ Potsdam, Inst Nutrit Sci, Nuthetal, Germany; [Masala, Giovanna] Inst Canc Res Prevent & Clin Network ISPRO, Florence, Italy; [Sieri, Sabina] Fondazione IRCCS Ist Nazl Tumori Milano, Epidemiol & Prevent Unit, Via Venezian 1, I-120133 Milan, Italy; [Sieri, Sabina] Univ Naples Federico II, Dipartimento Medicina Clin Chirurg, Naples, Italy; [Di Girolamo, Chiara] Univ Turin, Dept Clin & Biol Sci, Ctr Biostat Epidemiol & Publ Hlth C BEPH, Regione Gonzole 10, Orbassano, TO, Italy; [Skeie, Guri; Borch, Kristin Benjaminsen; Olsen, Karina Standahl] UiT Arctic Univ Norway, Dept Community Med, Tromso, Norway; [Sanchez, Maria-Jose] Escuela Andaluza Salud Publ EASP, Granada 18011, Spain; [Sanchez, Maria-Jose] Inst Invest Biosanitaria ibs GRANADA, Granada 18012, Spain; [Sanchez, Maria-Jose; Amiano, Pilar; Guevara, Marcela] Ctr Invest Biomed Red Epidemiol Salud Publ CIBERE, Madrid 28029, Spain; [Sanchez, Maria-Jose] Univ Granada, Dept Prevent Med & Publ Hlth, Granada 18071, Spain; [Amiano, Pilar] Minist Hlth Basque Govt, Sub Directorate Publ Hlth & Addict Gipuzkoa, San Sebastian, Spain; [Amiano, Pilar] Biodonostia Hlth Res Inst, Epidemiol Chron & Communicable Dis Grp, San Sebastian, Spain; [Chirlaque, Maria-Dolores] Univ Murcia, Dept Epidemiol, Reg Hlth Council, IMIB Arrixaca, Murcia, Spain; [Chirlaque, Maria-Dolores] CIBER Epidemiol & Publ Hlth CIBERESP, Madrid, Spain; [Guevara, Marcela] Inst Salud Publ & Laboral Navarra, Pamplona 31003, Spain; [Guevara, Marcela] Navarra Inst Hlth Res IdiSNA, Pamplona 31008,

Spain; [Sund, Malin] Umea Univ, Dept Surg & Perioperat Sci Surg, Umea, Sweden; [Sund, Malin] Univ Helsinki, Dept Surg, Helsinki, Finland; [Sund, Malin] Helsinki Univ Hosp, Helsinki, Finland; [Boden, Stina] Umea Univ, Dept Clin Sci Pediat, Umea, Sweden; [Gunter, Marc J.; Gonzalez-Gil, Esther M.; Weiderpass, Elisabete; Aguilera-Buenosvinos, Inmaculada; Dossus, Laure] World Hlth Org, Int Agcy Res Canc, Lyon, France; [Tsilidis, Kostas K.; Heath, Alicia K.; Aune, Dagfinn] Imperial Coll London, Sch Publ Hlth, Dept Epidemiol & Biostat, London, England; [Aguilera-Buenosvinos, Inmaculada] Univ Navarra, Dept Prevent Med & Publ Hlth, Pamplona, Spain; [Tsilidis, Kostas K.] Univ Ioannina, Dept Hyg & Epidemiol, Sch Med, Ioannina, Greece; [Aune, Dagfinn] Oslo New Univ Coll, Dept Nutr, Oslo, Norway; [Aune, Dagfinn] Oslo Univ Hosp Ulleval, Dept Endocrinol Morbid Obes & Prevent Med, Oslo, Norway Institut Catala d'Oncologia; Harvard University; Harvard T.H. Chan School of Public Health; Danish Cancer Society; University of Copenhagen; Universite Paris Saclay; UNICANCER; Gustave Roussy; Institut National de la Sante et de la Recherche Medicale (Inserm); Helmholtz Association; German Cancer Research Center (DKFZ); Leibniz Association; Deutsches Institut fur Ernahrungsforschung Potsdam-Rehbrucke (DIfE); University of Potsdam; University of Naples Federico II; University of Turin; UiT The Arctic University of Tromso; Escuela Andaluza de Salud Publica; Instituto de Investigacion Biosanitaria IBS Granada; CIBER - Centro de Investigacion Biomedica en Red; CIBERESP; University of Granada; Basque Government; Instituto de Investigacion Sanitaria Biogipuzkoa; Hospital Clinico Universitario Virgen de la Arrixaca; University of Murcia; CIBER - Centro de Investigacion Biomedica en Red; CIBERESP; Public Health Institute of Navarra; University of Navarra; Umea University; University of Helsinki; University of Helsinki; Helsinki University Central Hospital; Umea University; World Health Organization; International Agency for Research on Cancer (IARC); Imperial College London; University of Navarra; University of Ioannina; University of Oslo   Castro-Espin, C (corresponding author), Catalan Inst Oncol ICO, Unit Nutr & Canc, Lhospitalet De Llobregat, Spain.; Castro-Espin, C (corresponding author), Bellvitge Bio Med Res Inst IDIBELL, Nutr & Canc Grp, Epidemiol Publ Hlth Canc Prevent & Palliat Care P, Lhospitalet De Llobregat, Spain.   carlota.castroespin@gmail.com

Tjonneland, Anne/AGU-0320-2022; Weiderpass, Elisabete/M-4029-2016; Crous-Bou, Marta/A-9499-2019; Chirlaque, Maria-Dolores/KCL-3322-2024; Aune, Dagfinn/AFP-4300-2022; Dossus, Laure/AAW-9097-2021; Di Girolamo, Chiara/AAB-9735-2019; Sánchez, María/JTS-8758-2023; Gil-Agudo, Ángel/ABD-3065-2021; Bonet, Catalina/AFR-7176-2022; Sieri, Sabina/K-4667-2016; Gunter, Marc/AAP-8621-2020; Panico, Salvatore/K-6506-2016; Schulze, Matthias B./AAH-6906-2021; Heath, Alicia/AAO-4007-2020; Gonzalez Gil, Esther Maria/AAA-8040-2020; Truong, Therese/A-2837-2013; Guevara, Marcela/B-4473-2015Schulze, Matthias B./0000-0002-0830-5277; Heath, Alicia/0000-0001-6517-1300; Gonzalez Gil, Esther Maria/0000-0003-2005-8229; Tjonneland, Anne/0000-0003-4385-2097; Sund, Malin/0000-0002-7516-9543; Castro Espin, Carlota/0000-0001-9050-7590; Aune, Dagfinn/0000-0002-4533-1722; Truong, Therese/0000-0002-2943-6786; Guevara, Marcela/0000-0001-9242-6364

Instituto de Salud Carlos III [FI19/00197]; European Social Fund; AECC Scientific Foundation [PRYES211366AGUD]; International Agency for Research on Cancer (IARC); Department of Epidemiology and Biostatistics, School of Public Health, Imperial College

London; Cancer Research UK [14136, C8221/A29017]; Medical Research Council (United Kingdom) [1000143, MR/M012190/1]; MRC [MR/M012190/1] Funding Source: UKRI Instituto de Salud Carlos III(Instituto de Salud Carlos IIISpanish Government); European Social Fund(European Social Fund (ESF)); AECC Scientific Foundation; International Agency for Research on Cancer (IARC)(World Health Organization); Department of Epidemiology and Biostatistics, School of Public Health, Imperial College London; Cancer Research UK(Cancer Research UK); Medical Research Council (United Kingdom)(UK Research & Innovation (UKRI)Medical Research Council UK (MRC)); MRC(UK Research & Innovation (UKRI)Medical Research Council UK (MRC)) Carlota Castro-Espin is funded by Instituto de Salud Carlos III through the Grant FI19/00197 (co-funded by the European Social Fund. ESF investing in your future). This study is funded by the AECC Scientific Foundation (Project PRYES211366AGUD). The coordination of EPIC is financially supported by International Agency for Research on Cancer (IARC) and by the Department of Epidemiology and Biostatistics, School of Public Health, Imperial College London, which has additional infrastructure support provided by the NIHR Imperial Biomedical Research Centre (BRC). The national cohorts are supported by: Danish Cancer Society (Denmark); Ligue Contre le Cancer, Institut Gustave Roussy, Mutuelle Generale de l'Education Nationale, Institut National de la Sante et de la Recherche Medicale (INSERM) (France); German Cancer Aid, German Cancer Research Centre (DKFZ), German Institute of Human Nutri-tion Potsdam-Rehbruecke (DIfE), Federal Ministry of Education and Research (BMBF) (Germany); Associazione Italiana per la Ricerca sul Cancro-AIRC-Italy, Compagnia di SanPaolo and National Research Council (Italy); Dutch Ministry of Public Health, Welfare and Sports (VWS), Netherlands Cancer Registry (NKR), LK Research Funds, Dutch Prevention Funds, Dutch ZON (Zorg Onderzoek Nederland), World Cancer Research Fund (WCRF), Statistics Netherlands (The Netherlands); Health Research Fund (FIS)-Instituto de Salud Carlos III (ISCIII), Regional Governments of Andalucia, Asturias, Basque Country, Murcia and Navarra, and the Catalan Institute of Oncology-ICO (Spain); Swedish Cancer Society, Swedish Research Council and County Councils of Skane and Vaesterbotten (Sweden); Cancer Research UK (14136 to EPIC-Norfolk; C8221/A29017 to EPIC-Oxford), Medical Research Council (1000143 to EPIC-Norfolk; MR/M012190/1 to EPIC-Oxford) (United Kingdom). The funders of this study had no role in the decisions about the analysis or interpretation of the data, or preparation, review or approval of the manuscript. 30 16 16 0 14 BMC LONDON CAMPUS, 4 CRINAN ST, LONDON N1 9XW, ENGLAND 1741-7015 BMC MED BMC Med. JUN 26 2023 21 1 225 10.1186/s12916-023-02934-3 http://dx.doi.org/10.1186/s12916-023-02934-3 11 Medicine, General & Internal Science Citation Index Expanded (SCI-EXPANDED) General & Internal Medicine L2LZ7 37365585 gold, Green Published 2025-06-24 WOS:001021638200002

J Bennacef, AC; Khodja, AA; Abou-Bekr, FA; Ndao, T; Holl, R; Bencina, G Bennacef, Ali-Chakib; Khodja, Aomar Ammar; Abou-Bekr, Fadl Allah; Ndao, Tidiane; Holl, Ryan; Bencina, Goran Costs and Resource Use Among Patients with Cervical Cancer, Cervical Intraepithelial Neoplasia, and Genital Warts in Algeria JOURNAL OF HEALTH ECONOMICS AND OUTCOMES RESEARCH

English Article

papillomavirus infections; sexually transmitted viral diseases; genital warts; North Africa epidemiology; Algeria epidemiology; burden of illness; cost of illness EXTENDED MIDDLE-EAST; HUMAN-PAPILLOMAVIRUS INFECTIONS; BURDEN; PREVENTION; MANAGEMENT; DISEASES; EUROPE; WOMEN; HPV Background: Cervical cancer rates in North Africa have risen in the last 10 years, suggesting that this region might benefit from cervical cancer screening and HPV vaccination programs. To assess the potential benefits of cervical cancer screening and HPV vaccination in North African countries, country-specific data on the prevalence and burden of HPV-related conditions are needed. Objectives: To describe the patterns and estimate the costs of management of cervical cancer, cervical intraepithelial neoplasia (CIN), and genital warts in Algeria. Methods: This was a descriptive analysis of questionnaire data obtained from a panel of 15 oncologists, gynecologists, and dermatologists (n=5 each). Data on diagnostic and treatment patterns, recurrence, and healthcare resource use (HCRU) were obtained. The costs (in Algerian dinars) associated with diagnosis, treatment, and recurrence were estimated. Results: Diagnosis of CIN was obtained by cytology tests or lesion biopsies; for cervical cancer, lesion biopsies, MRI, and CT scans were the most common diagnostic tests. For CIN, 70% of gynecologists and oncologists regularly or always used conization as a treatment. Treatments used regularly or always for cervical cancer included chemotherapy (80%), hysterectomy (70%), and radiation (70%). Annual HCRU per institution included 20 outpatient visits and 15 hospitalizations for CIN, and 50 outpatient visits and 11 hospitalizations for cervical cancer. For genital warts, diagnostic tests performed regularly or always included assays for hepatitis B, hepatitis C, HIV, and syphilis; cervical cytology; and colposcopy. Cryotherapy was the universal first-line treatment. Median per-patient costs associated with diagnosis, treatment, and recurrence were 6750, 19 750, and 77 750, respectively, for CIN; 53 750, 650 000, and 431 250, respectively, for cervical cancer; and 16 075, 15 500, and 9250, respectively, for genital warts. Discussion: These results give an estimate of the HCRU and cost of cervical cancer, CIN, and genital warts and highlight the need to assess more precisely the epidemiology of these diseases in Algeria. Conclusions: This study investigated the management of patients with cervical cancer, CIN, or genital warts in Algeria and provided the first estimates of diagnosis and treatment patterns, HCRU, and costs associated with these conditions. These resource use and cost estimates highlight the need to develop prevention strategies for HPV-related pathologies. [Bennacef, Ali-Chakib] Merck Sharp & Dohme Ltd, Algiers, Algeria; [Khodja, Aomar Ammar] Univ Hosp Mustapha Bacha, Algiers, Algeria; [Abou-Bekr, Fadl Allah] Univ Hosp Sidi Bel Abbes, Sidi Bel Abbes, Algeria; [Ndao, Tidiane] Merck Sharp & Dohme Ltd, Casablanca, Morocco; [Holl, Ryan] Merck Sharp & Dohme Int GmbH, Kriens, Switzerland; [Bencina, Goran] Merck Sharp & Dohme Ltd, Madrid, Spain Merck & Company; Merck & Company Spain

Bennacef, AC (corresponding author), Merck Sharp & Dohme Ltd, Algiers, Algeria. ali.chakib.bennacef@merck.com

25 1 1 0 1 COLUMBIA DATA ANALYTICS, LLC NEW YORK 145 HUDSON ST., SUITE 205, NEW YORK, NJ 10013 USA 2327-2236 J HEALTH ECON OUTCOM

J. Health Econ. Outcome. Res. 2022 9 1

31 38 10.36469/jheor.2022.31049
http://dx.doi.org/10.36469/jheor.2022.31049 8
Economics; Health Care Sciences & Services; Health Policy & Services Emerging Sources Citation Index (ESCI) Business & Economics; Health Care Sciences & Services L2US9 35224126
Green Published, gold 2025-06-24
WOS:001021867200005
J Barrubés, L; Babio, N; Mena-Sánchez, G; Toledo, E; Ramírez-Sabio, JB; Estruch, R; Ros, E; Fitó, M; Aros, F; Fiol, M; Santos-Lozano, JM; Serra-Majem, L; Pintó, X; Martínez-González, MA; Sorlí, JV; Basora, J; Salas-Salvadó, J Barrubes, Laura; Babio, Nancy; Mena-Sanchez, Guillermo; Toledo, Estefania; Ramirez-Sabio, Judith B.; Estruch, Ramon; Ros, Emilio; Fito, Montserrat; Aros, Fernando; Fiol, Miquel; Manuel Santos-Lozano, Jose; Serra-Majem, Lluis; Pinto, Xavier; Angel Martinez-Gonzalez, Miguel; Vicente Sorli, Jose; Basora, Josep; Salas-Salvado, Jordi
PREvencion Con DIeta MEDiterDairy Product Consumption and Risk of Colorectal Cancer in an Older Mediterranean Population at High Cardiovascular Risk INTERNATIONAL JOURNAL OF CANCER
English Article
colorectal cancer; mediterranean diet; dairy products; milk; PREDIMED study CONJUGATED LINOLEIC-ACID; VITAMIN-D; COHORT; DIET; CALCIUM; FOOD; METAANALYSIS; MECHANISMS; DISEASE; MILK
Prospective studies have reported an inverse association between the consumption of total dairy products and milk and the risk of colorectal cancer (CRC). Nonetheless, there is little and inconsistent evidence regarding subtypes of dairy product and CRC risk. We assessed the associations between the consumption of total dairy products, their different subtypes and CRC risk in older Mediterranean individuals at high cardiovascular risk. We analyzed data from 7,216 men and women (55-80 years) without CRC at baseline from the PREvencion con DIeta MEDiterranea study. Individuals were recruited between 2003 and 2009 and followed up until December 2012. At baseline and yearly thereafter, consumption of total and specific dairy products was assessed using a validated 137-item food-frequency questionnaire. Cox proportional hazards ratios (HRs) of CRC incidence were estimated for tertiles of mean consumption of dairy products during the follow-up. During a median [interquartile range] follow-up of 6.0 [4.4-7.3] years, we documented 101 incident CRC cases. In the multivariable-adjusted models, HRs and 95% confidence intervals (CIs) of CRC for the comparison of extreme tertiles of total dairy product and low-fat milk consumption were 0.55 (95% CI: 0.31-0.99; p-trend = 0.037) and 0.54 (95% CI: 0.32-0.92; p-trend = 0.022), respectively. No significant associations with other dairy products (whole-fat and low-fat dairy products; total, low-fat and whole-fat yogurt; cheese; total, low-fat and whole-fat milk; concentrated full-fat dairy products, sugar-enriched dairy products and fermented dairy products) were found. A high consumption of total dairy products and low-fat milk was significantly associated with a reduced CRC risk. [Barrubes, Laura; Babio, Nancy; Mena-Sanchez, Guillermo; Basora, Josep; Salas-Salvado, Jordi] Univ Rovira & Virgili, Dept Biochem & Biotechnol, Human Nutr Unit, Reus, Spain; [Barrubes, Laura; Babio, Nancy; Mena-Sanchez, Guillermo; Toledo, Estefania; Estruch, Ramon; Ros, Emilio; Fito, Montserrat; Aros, Fernando; Fiol, Miquel; Manuel Santos-Lozano, Jose; Serra-Majem, Lluis; Pinto, Xavier; Angel Martinez-Gonzalez, Miguel; Vicente Sorli, Jose; Basora,

Josep; Salas-Salvado, Jordi] Inst Salud Carlos III ISCIII, CIBER Fisiopatol Obesidad & Nutr CIBEROBN, Madrid, Spain; [Toledo, Estefania; Angel Martinez-Gonzalez, Miguel] Univ Navarra, Navarra Inst Hlth Res, Dept Prevent Med & Publ Hlth, Pamplona, Spain; [Ramirez-Sabio, Judith B.; Vicente Sorli, Jose] Univ Valencia, Dept Prevent Med, Valencia, Spain; [Ramirez-Sabio, Judith B.] Hosp Sagunto, Serv Oncol, Valencia, Spain; [Estruch, Ramon] Univ Barcelona, Hosp Clin, Dept Internal Med, Barcelona, Spain; [Estruch, Ramon; Ros, Emilio] August Pi i Sunyer Biomed Res Inst IDIBAPS, Barcelona, Spain; [Ros, Emilio] Univ Barcelona, Hosp Clin, Dept Lipids, Barcelona, Spain; [Fito, Montserrat] Inst Hosp Mar Invest Med IMIM, Cardiovasc Risk & Nutr Res REGICOR Grp, Barcelona, Spain; [Aros, Fernando] Univ Hosp Araba, Dept Cardiol, Vitoria, Spain; [Fiol, Miquel] Balear Isl Hlth Res Inst IdISBa, Palma De Mallorca, Spain; [Fiol, Miquel] Son Espases Hosp, Palma De Mallorca, Spain; [Manuel Santos-Lozano, Jose] Ctr Salud Univ San Pablo, Dist Sanitario Atenc Primaria Sevilla, Dept Family Med, Res Unit, Seville, Spain; [Serra-Majem, Lluis] Univ Las Palmas Gran Canaria, Res Inst Biomed & Hlth Sci, Las Palmas Gran Canaria, Spain; [Pinto, Xavier] Hosp Univ Bellvitge, Internal Med, Lipids & Vasc Risk Unit, Lhospitalet De Llobregat, Spain	Universitat Rovira i Virgili; CIBER - Centro de Investigacion Biomedica en Red; CIBEROBN; University of Navarra; University of Valencia; University of Barcelona; Hospital Clinic de Barcelona; University of Barcelona; Hospital Clinic de Barcelona; IDIBAPS; University of Barcelona; Hospital Clinic de Barcelona; Hospital del Mar Research Institute; University Hospital of Araba; Institut Investigacio Sanitaria Illes Balears (IdISBa); Hospital Universitari Son Espases; Universidad de Las Palmas de Gran Canaria; Institut d'Investigacio Biomedica de Bellvitge (IDIBELL); Bellvitge University Hospital	Babio, N; Salas-Salvadó, J (corresponding author), Rovira & Virgili Univ, Fac Med & Hlth Sci, Biochem & Biotechnol Dept, Human Nutr Unit, 21 St Llorenc St, Reus 43201, Spain.	nancy.babio@urv.cat; jordi.salas@urv.cat	Sorlí, José/L-8758-2014; Estruch, Ramon/AAZ-3723-2020; Toledo, Estefania/H-6211-2014; Santos, João/HHZ-5595-2022; Babio, Nancy/AAN-2715-2020; Serra-Majem, Lluis/I-6708-2019; Pintó, Xavier/AGI-4297-2022; Martinez-Gonzalez, Miguel/AAE-7669-2019; Santos Lozano, Jose Manuel/J-9312-2018; Salas-Huetos, Albert/A-8509-2011; Salas-Salvado, Jordi/C-7229-2017; Fito Colomer, Montse/C-1822-2012; , MIQUEL FIOL SALA/F-6793-2016	Basora, Josep/0000-0003-0278-1149; Santos Lozano, Jose Manuel/0000-0001-7097-5653; Salas-Huetos, Albert/0000-0001-5914-6862; Sorli, Jose V/0000-0002-0130-2006; MENA SANCHEZ, GUILLERMO/0000-0002-3790-130X; Ramirez-Sabio, Judith Begona/0000-0002-4554-6360; Salas-Salvado, Jordi/0000-0003-2700-7459; Pinto Sala, Xavier/0000-0002-2216-2444; Fito Colomer, Montse/0000-0002-1817-483X; BABIO SANCHEZ, NANCY/0000-0003-3527-5277; , MIQUEL FIOL SALA/0000-0002-5370-1391	41	27	27	0	28
WILEY	HOBOKEN	111 RIVER ST, HOBOKEN 07030-5774, NJ USA
0020-7136	1097-0215	INT J CANCER	Int. J. Cancer
SEP 15	2018	143	6	1356	1366
10.1002/ijc.31540
http://dx.doi.org/10.1002/ijc.31540	11
Oncology	Science Citation Index Expanded (SCI-EXPANDED)
Oncology	GP7ZH	29663376	Bronze	2025-06-24	WOS:000441128700009

J Laitman, Y; Friebel, TM; Yannoukakos, D; Fostira, F; Konstantopoulou, I; Figlioli, G; Bonanni, B; Manoukian, S; Zuradelli, M; Tondini, C; Pasini, B; Peterlongo, P; Plaseska-Karanfilska, D; Jakimovska, M; Majidzadeh, K; Zarinfam, S; Loizidou, MA; Hadjisavvas, A; Michailidou, K; Kyriacou, K; Behar, DM; Bernstein Molho, R; Ganz, P; James, P; Parsons, MT; Sallam, A; Olopade, OI; Seth, A; Chenevix - Trench, G; Leslie, G; McGuffog, L; Marafie, MJ; Megarbane, A; Al-Mulla, F; Rebbeck, TR; Friedman, E Laitman, Yael; Friebel, Tara M.; Yannoukakos, Drakoulis; Fostira, Florentia; Konstantopoulou, Irene; Figlioli, Gisella; Bonanni, Bernardo; Manoukian, Siranoush; Zuradelli, Monica; Tondini, Carlo; Pasini, Barbara; Peterlongo, Paolo; Plaseska-Karanfilska, Dijana; Jakimovska, Milena; Majidzadeh, Keivan; Zarinfam, Shiva; Loizidou, Maria A.; Hadjisavvas, Andreas; Michailidou, Kyriaki; Kyriacou, Kyriacos; Behar, Doron M.; Bernstein Molho, Rinat; Ganz, Patricia; James, Paul; Parsons, Michael T.; Sallam, Aminah; Olopade, Olufunmilayo I.; Seth, Arun; Chenevix - Trench, Georgia; Leslie, Goska; McGuffog, Lesley; Marafie, Makia J.; Megarbane, Andre; Al-Mulla, Fahd; Rebbeck, Timothy R.; Friedman, Eitan The spectrum of BRCA1 and BRCA2 pathogenic sequence variants in Middle Eastern, North African, and South European countries HUMAN MUTATION English Article BRCA1 BRCA2 mutational spectrum; first pass genotyping; inherited breast cancer; Middle East; North Africa; underserved populations BREAST-CANCER PATIENTS; FOUNDER MUTATIONS; HAPLOTYPE ANALYSIS; ASHKENAZI JEWISH; OVARIAN-CANCER; RECURRENT; POPULATION; RISK; IDENTIFICATION; PREVALENCE BRCA1 BRCA2 mutational spectrum in the Middle East, North Africa, and Southern Europe is not well characterized. The unique history and cultural practices characterizing these regions, often involving consanguinity and inbreeding, plausibly led to the accumulation of population-specific founder pathogenic sequence variants (PSVs). To determine recurring BRCA PSVs in these locales, a search in PUBMED, EMBASE, BIC, and CIMBA was carried out combined with outreach to researchers from the relevant countries for unpublished data. We identified 232 PSVs in BRCA1 and 239 in BRCA2 in 25 of 33 countries surveyed. Common PSVs that were detected in four or more countries were c.5266dup (p.Gln1756Profs), c.181T>G (p.Cys61Gly), c.68_69del (p.Glu23Valfs), c.5030_5033del (p.Thr1677Ilefs), c.4327C>T (p.Arg1443Ter), c.5251C>T (p.Arg1751Ter), c.1016dup (p.Val340Glyfs), c.3700_3704del (p.Val1234Glnfs), c.4065_4068del (p.Asn1355Lysfs), c.1504_1508del (p.Leu502Alafs), c.843_846del (p.Ser282Tyrfs), c.798_799del (p.Ser267Lysfs), and c.3607C>T (p.Arg1203Ter) in BRCA1 and c.2808_2811del (p.Ala938Profs), c.5722_5723del (p.Leu1908Argfs), c.9097dup (p.Thr3033Asnfs), c.1310_1313del (p. p.Lys437Ilefs), and c.5946del (p.Ser1982Argfs) for BRCA2. Notably, some mutations (e.g., p.Asn257Lysfs (c.771_775del)) were observed in unrelated populations. Thus, seemingly genotyping recurring BRCA PSVs in specific populations may provide first pass BRCA genotyping platform. [Laitman, Yael; Friedman, Eitan] Sheba Med Ctr, Inst Human Genet, Susanne Levy Gertner Oncogenet Unit, IL-52621 Tel Hashomer, Israel; [Friebel, Tara M.; Rebbeck, Timothy R.] Dana Farber Canc Inst, Boston, MA 02115 USA; [Yannoukakos, Drakoulis; Fostira, Florentia; Konstantopoulou, Irene] Natl Ctr Sci Res Demokritos, INRASTES, Mol Diagnost Lab, Athens, Greece; [Figlioli, Gisella; Peterlongo, Paolo] FIRC Inst Mol Oncol, IFOM, Genome

Diagnost Program, Milan, Italy; [Bonanni, Bernardo] European Inst Oncol IRCCS, IEO, Div Canc Prevent & Genet, Milan, Italy; [Manoukian, Siranoush] Fdn IRCCS Ist Nazl Tumori INT, Dept Med Oncol & Hematol, Unit Med Genet, Milan, Italy; [Zuradelli, Monica] Humanitas Canc Ctr, Med Oncol & Hematol Dept, Milan, Italy; [Tondini, Carlo] Osped Papa Giovanni XXIII, Dept Med Oncol, Bergamo, Italy; [Pasini, Barbara] Univ Turin, Dept Med Sci, Turin, Italy; [Plaseska-Karanfilska, Dijana; Jakimovska, Milena] Macedonian Acad Sci & Arts, Res Ctr Genet Engn & Biotechnol, Skopje, North Macedonia; [Majidzadeh, Keivan] Motamed Canc Inst, ACECR, Breast Canc Res Ctr, Dept Genet, Tehran, Iran; [Zarinfam, Shiva; Loizidou, Maria A.; Hadjisavvas, Andreas; Michailidou, Kyriaki; Kyriacou, Kyriacos] Cyprus Sch Mol Med, Cyprus Inst Neurol & Genet, Dept Elect Microscopy Mol Pathol, Nicosia, Cyprus; [Behar, Doron M.] Igentify, Tirat Hacrmel, Israel; [Bernstein Molho, Rinat] Sheba Med Ctr, Inst Oncol, Tel Hashomer, Israel; [Bernstein Molho, Rinat; Friedman, Eitan] Tel Aviv Univ, Sackler Sch Med, Tel Aviv, Israel; [Ganz, Patricia] Univ Calif Los Angeles, Jonsson Comprehens Canc Ctr, Div Canc Prevent & Control Res, Sch Med, Los Angeles, CA 90024 USA; [Ganz, Patricia] Univ Calif Los Angeles, Jonsson Comprehens Canc Ctr, Div Canc Prevent & Control Res, Sch Publ Hlth, Los Angeles, CA 90024 USA; [James, Paul] Univ Melbourne, Sir Peter MacCallum Dept Oncol, Parkville Familial Canc Peter MacCallum Canc Ctr, Melbourne, Vic, Australia; [Parsons, Michael T.; Chenevix - Trench, Georgia] Queensland Inst Med Res, QIMR Berghofer Med Res Inst, Dept Genet & Computat Biol, Brisbane, Qld, Australia; [Sallam, Aminah; Olopade, Olufunmilayo I.] Univ Chicago, Ctr Clin Canc Genet, Chicago, IL 60637 USA; [Seth, Arun] Univ Toronto, Fac Med, Dept Lab Med & Pathobiol, Toronto, ON, Canada; [Leslie, Goska; McGuffog, Lesley] Univ Cambridge, Dept Publ Hlth & Primary Care, Ctr Canc Genet Epidemiol, Cambridge, England; [Marafie, Makia J.] Matern Hosp, Kuwait Med Genet Ctr, Kuwait, Kuwait; [Megarbane, Andre] Inst Jerome Lejeune, Paris, France; [Al-Mulla, Fahd] Dasman Diabet Inst, Dept Genet & Bioinformat, Kuwait, Kuwait; [Rebbeck, Timothy R.] Harvard TH Chan Sch Publ Hlth, Boston, MA USA Chaim Sheba Medical Center; Tel Aviv University; Harvard University; Harvard University Medical Affiliates; Dana-Farber Cancer Institute; National Centre of Scientific Research "Demokritos"; IFOM - FIRC Institute of Molecular Oncology; IRCCS European Institute of Oncology (IEO); Fondazione IRCCS Istituto Nazionale Tumori Milan; ASST Papa Giovanni XXIII; University of Turin; Academic Center for Education, Culture & Research (ACECR); Cyprus Institute of Neurology & Genetics; Tel Aviv University; Chaim Sheba Medical Center; Tel Aviv University; Sackler Faculty of Medicine; University of California System; University of California Los Angeles; University of California Los Angeles Medical Center; David Geffen School of Medicine at UCLA; UCLA Jonsson Comprehensive Cancer Center; UCLA Jonsson Comprehensive Cancer Center; University of California System; University of California Los Angeles; University of Melbourne; Peter Maccallum Cancer Center; QIMR Berghofer Medical Research Institute; University of Chicago; University of Toronto; University of Cambridge; Dasman Diabetes Institute (DDI); Harvard University; Harvard T.H. Chan School of Public Health   Friedman, E (corresponding author), Sheba Med Ctr, Inst Human Genet, Susanne Levy Gertner Oncogenet Unit, IL-52621 Tel Hashomer, Israel.

eitan.friedman@sheba.health.gov.il   Loizidou, Maria/C-

6607-2009; Seth, Arun/AAI-7618-2020; Michailidou, Kyriaki/ABD-5122-2020; Konstantopoulou, Irene/Y-3074-2018; Chenevix-Trench, Georgia/AAV-2014-2020; Peterlongo, Paolo/Q-4717-2018; James, Paul/G-2943-2014; Bonanni, Bernardo/AAM-7928-2020; Megarbane, Andre/ABD-5574-2021; Rebbeck, Timothy/GLU-8348-2022; Fostira, Florentia/Z-5730-2019; Zuradelli, Monica/AAC-2254-2020; manoukian, siranoush/E-7132-2017; Karanfilska, Dijana/P-6096-2018; Friebel-Klingner, Tara/ABF-4212-2021; Pasini, Barbara/AHI-2004-2022; Al-Mulla, Fahd/E-2068-2015; Konstantopoulou, Irene/B-5309-2009
Friebel, Tara/0000-0002-3726-9527; Peterlongo, Paolo/0000-0001-6951-6855; Bernstein Molho, Rinat/0000-0002-4068-9514; Pasini, Barbara/0000-0002-4373-1212; Fostira, Florentia/0000-0003-2751-2332; Ganz, Patricia/0000-0002-1841-4143; Chenevix-Trench, Georgia/0000-0002-1878-2587; Megarbane, andre/0000-0003-0714-2469; Olopade, Olufunmilayo/0000-0002-9936-1599; Figlioli, Gisella/0000-0002-0740-1363; Al-Mulla, Fahd/0000-0001-5409-3829; Bonanni, Bernardo/0000-0003-3589-2128; Yannoukakos, Drakoulis/0000-0001-7509-3510; Konstantopoulou, Irene/0000-0002-0470-0309; Michailidou, Kyriaki/0000-0001-7065-1237; James, Paul/0000-0002-4361-4657; Parsons, Michael/0000-0003-3242-8477 Cancer Research UK [20861] Funding Source: Medline Cancer Research UK(Cancer Research UK) 42 36 37 0 2 WILEY HOBOKEN 111 RIVER ST, HOBOKEN 07030-5774, NJ USA 1059-7794 1098-1004 HUM MUTAT Hum. Mutat. NOV 2019 40 11 E1 E23 10.1002/humu.23842 http://dx.doi.org/10.1002/humu.23842 23 Genetics & Heredity Science Citation Index Expanded (SCI-EXPANDED) Genetics & Heredity JF6FU 31209999 gold, Green Published, Green Submitted 2025-06-24 WOS:000491484200001
J Buckland, G; Travier, N; Cottet, V; González, CA; Luján-Barroso, L; Agudo, A; Trichopoulou, A; Lagiou, P; Trichopoulos, D; Peeters, PH; May, A; Bueno-de-Mesquita, HB; Duijnhoven, FJB; Key, TJ; Allen, N; Khaw, KT; Wareham, N; Romieu, I; McCormack, V; Boutron-Ruault, M; Clavel-Chapelon, F; Panico, S; Agnoli, C; Palli, D; Tumino, R; Vineis, P; Amiano, P; Barricarte, A; Rodríguez, L; Sanchez, MJ; Chirlaque, MD; Kaaks, R; Teucher, B; Boeing, H; Bergmann, MM; Overvad, K; Dahm, CC; Tjonneland, A; Olsen, A; Manjer, J; Wirfält, E; Hallmans, G; Johansson, I; Lund, E; Hjartåker, A; Skeie, G; Vergnaud, AC; Norat, T; Romaguera, D; Riboli, E Buckland, G.; Travier, N.; Cottet, V.; Gonzalez, C. A.; Lujan-Barroso, L.; Agudo, A.; Trichopoulou, A.; Lagiou, P.; Trichopoulos, D.; Peeters, P. H.; May, A.; Bueno-de-Mesquita, H. B.; Duijnhoven, F. J. Bvan; Key, T. J.; Allen, N.; Khaw, K. T.; Wareham, N.; Romieu, I.; McCormack, V.; Boutron-Ruault, M.; Clavel-Chapelon, F.; Panico, S.; Agnoli, C.; Palli, D.; Tumino, R.; Vineis, P.; Amiano, P.; Barricarte, A.; Rodriguez, L.; Sanchez, M. J.; Chirlaque, M. D.; Kaaks, R.; Teucher, B.; Boeing, H.; Bergmann, M. M.; Overvad, K.; Dahm, C. C.; Tjonneland, A.; Olsen, A.; Manjer, J.; Wirfalt, E.; Hallmans, G.; Johansson, I.; Lund, E.; Hjartaker, A.; Skeie, G.; Vergnaud, A. C.; Norat, T.; Romaguera, D.; Riboli, E. Adherence to the mediterranean diet and risk of breast cancer in the European prospective investigation into cancer and nutrition cohort study INTERNATIONAL JOURNAL OF CANCER English Article breast cancer; Europe; Mediterranean diet; prospective studies FATTY-ACID; PATTERNS; ESTROGEN; CALIBRATION; RATIONALE; VALIDITY; DISEASE;

GROWTH; CELLS   Epidemiological evidence suggests that the Mediterranean diet (MD) could reduce the risk of breast cancer (BC). As evidence from the prospective studies remains scarce and conflicting, we investigated the association between adherence to the MD and risk of BC among 335,062 women recruited from 1992 to 2000, in ten European countries, and followed for 11 years on average. Adherence to the MD was estimated through an adapted relative Mediterranean diet (arMED) score excluding alcohol. Cox proportional hazards regression models were used while adjusting for BC risk factors. A total of 9,009 postmenopausal and 1,216 premenopausal first primary incident invasive BC were identified (5,862 estrogen or progesterone receptor positive [ER+/PR+] and 1,018 estrogen and progesterone receptor negative [ER/PR]). The arMED was inversely associated with the risk of BC overall and in postmenopausal women (high vs. low arMED score; hazard ratio [HR] = 0.94 [95% confidence interval [CI]: 0.88, 1.00] ptrend = 0.048, and HR = 0.93 [95% CI: 0.87, 0.99] ptrend = 0.037, respectively). The association was more pronounced in ER/PR tumors (HR = 0.80 [95% CI: 0.65, 0.99] ptrend = 0.043). The arMED score was not associated with BC in premenopausal women. Our findings show that adherence to a MD excluding alcohol was related to a modest reduced risk of BC in postmenopausal women, and this association was stronger in receptor-negative tumors. The results support the potential scope for BC prevention through dietary modification.

[Buckland, G.; Travier, N.; Gonzalez, C. A.; Lujan-Barroso, L.; Agudo, A.] Catalan Inst Oncol ICO IDIBELL, Canc Epidemiol Res Programme, Unit Nutr Environm & Canc, Barcelona 08907, Spain; [Travier, N.] Univ Barcelona, Bellvitge Biomed Res Inst, Dept Clin Sci, Barcelona, Spain; [Cottet, V.; Boutron-Ruault, M.; Clavel-Chapelon, F.] Inst Gustave Roussy, INSERM, Ctr Res Epidemiol & Populat Hlth, U1018, F-94805 Villejuif, France; [Cottet, V.] Paris South Univ, UMRS 1018, Villejuif, France; [Trichopoulou, A.; Lagiou, P.] Univ Athens, Sch Med, WHO Collaborating Ctr Food & Nutr Policies, Dept Hyg Epidemiol & Med Stat, GR-11527 Athens, Greece; [Trichopoulou, A.] Hellen Hlth Fdn, Athens, Greece; [Lagiou, P.; Trichopoulos, D.] Harvard Univ, Sch Publ Hlth, Dept Epidemiol, Boston, MA 02115 USA; [Lagiou, P.; Trichopoulos, D.] Acad Athens, Bur Epidemiol Res, Athens, Greece; [Peeters, P. H.; May, A.] Univ Med Ctr Utrecht, Julius Ctr, Utrecht, Netherlands; [Peeters, P. H.; Vineis, P.; Vergnaud, A. C.; Norat, T.; Romaguera, D.; Riboli, E.] Univ London Imperial Coll Sci Technol & Med, Fac Med, Sch Publ Hlth, Dept Epidemiol & Biostat, London, England; [Bueno-de-Mesquita, H. B.; Duijnhoven, F. J. Bvan] Natl Inst Publ Hlth & Environm RIVM, Bilthoven, Netherlands; [Bueno-de-Mesquita, H. B.; Duijnhoven, F. J. Bvan] Univ Med Ctr Utrecht, Dept Gastroenterol & Hepatol, Utrecht, Netherlands; [Duijnhoven, F. J. Bvan] Wageningen Univ, Div Human Nutr, NL-6700 AP Wageningen, Netherlands; [Key, T. J.; Allen, N.] Univ Oxford, Canc Epidemiol Unit, Oxford, England; [Khaw, K. T.] Univ Cambridge, Dept Publ Hlth & Primary Care, Cambridge, England; [Wareham, N.] Inst Metab Sci, MRC, Epidemiol Unit, Cambridge, England; [Romieu, I.; McCormack, V.] Int Agcy Res Canc, F-69372 Lyon, France; [Panico, S.] Univ Naples Federico II, Dept Clin & Expt Med, Naples, Italy; [Agnoli, C.] Fdn IRCCS, Ist Nazl Tumori, Nutr Epidemiol Unit, Milan, Italy; [Palli, D.] Res & Prevent Inst ISPO, Mol & Nutrit Epidemiol Unit, Florence, Italy; [Tumino, R.] Canc Registry & Histopathol Unit, Ragusa, Italy; [Barricarte, A.] Publ Hlth Inst Navarra, Pamplona, Spain; [Rodriguez, L.] Publ Hlth

Directorate, Oviedo Asturias, Spain; [Sanchez, M. J.] Andalusian Sch Publ Hlth, Granada, Spain; [Chirlaque, M. D.] Murcia Reg Hlth Author, Dept Epidemiol, Murcia, Spain; [Kaaks, R.; Teucher, B.] German Canc Res Ctr, Heidelberg, Germany; [Boeing, H.; Bergmann, M. M.] German Inst Human, Dept Epidemiol, Nuthetal, Nuthetal, Germany; [Overvad, K.; Dahm, C. C.] Sch Publ Hlth, Dept Epidemiol, Aarhus C, Denmark; [Dahm, C. C.] Aarhus Univ Hosp, Dept Cardiol, Aalborg, Jutland, Denmark; [Tjonneland, A.; Olsen, A.] Inst Canc Epidemiol Diet Canc & Hlth, Danish Canc Soc, Copenhagen, Denmark; [Manjer, J.; Wirfalt, E.] Lund Univ, Clin Res Ctr, Dept Clin Sci Malmo, Malmo, Sweden; [Hallmans, G.] Umea Univ, Dept Publ Hlth & Clin Med, Umea, Sweden; [Johansson, I.] Umea Univ, Dept Odontol, Umea, Sweden; [Lund, E.; Skeie, G.] Univ Tromso, Dept Community Med, Tromso, Norway; [Hjartaker, A.] Univ Oslo, Inst Basic Med Sci, Dept Nutr, Oslo, NorwayInstitut d'Investigacio Biomedica de Bellvitge (IDIBELL); Institut Catala d'Oncologia; Institut d'Investigacio Biomedica de Bellvitge (IDIBELL); University of Barcelona; Institut National de la Sante et de la Recherche Medicale (Inserm); UNICANCER; Gustave Roussy; Universite Paris Saclay; Institut National de la Sante et de la Recherche Medicale (Inserm); Universite Paris Saclay; National & Kapodistrian University of Athens; World Health Organization; Athens Medical School; Harvard University; Harvard T.H. Chan School of Public Health; Academy of Athens; Utrecht University; Utrecht University Medical Center; Imperial College London; Netherlands National Institute for Public Health & the Environment; Utrecht University; Utrecht University Medical Center; Wageningen University & Research; University of Oxford; University of Cambridge; University of Cambridge; World Health Organization; International Agency for Research on Cancer (IARC); University of Naples Federico II; Fondazione IRCCS Istituto Nazionale Tumori Milan; Public Health Institute of Navarra; Escuela Andaluza de Salud Publica; Murcia Regional Health Council; Helmholtz Association; German Cancer Research Center (DKFZ); Aarhus University; Danish Cancer Society; Lund University; Umea University; Umea University; UiT The Arctic University of Tromso; University of Oslo Buckland, G (corresponding author), Catalan Inst Oncol ICO IDIBELL, Canc Epidemiol Res Programme, Unit Nutr Environm & Canc, Avda Gran Via 199-203, Barcelona 08907, Spain. gbuckland@iconcologia.net

Clavel-Chapelon, Francoise/G-6733-2014; Romaguera, Dora/AAB-2852-2020; Hjartåker, Anette/D-6220-2011; Khaw, Kay-Tee/AAZ-3209-2021; Tjonneland, Anne/AGU-0320-2022; Lujan-Barroso, Leila/AAD-3166-2021; Teucher, Birgit/J-6380-2015; TRICHOPOULOU, ANTONIA/ABF-8727-2021; Cottet, Vanessa/ABE-3236-2020; Panico, Salvatore/K-6506-2016; May, Anne/G-9183-2011; Sánchez, María/HOC-7747-2023; Chirlaque, Maria-Dolores/KCL-3322-2024; Riboli, Elio/A-4357-2009; Trichopoulos, Dimitrios/G-6825-2012; McCormack, Valerie/NAZ-3472-2025; Agnoli, Claudia/K-5916-2016; Agudo, Antonio/J-1805-2016; Dahm, Christina/G-9787-2014; Gonzalez, Carlos A/O-4651-2014; SANCHEZ-PEREZ, MARIA JOSE/D-1087-2011 Panico, Salvatore/0000-0002-5498-8312; McCormack, Valerie/0000-0001-7397-3442; Agnoli, Claudia/0000-0003-4472-1179; tumino, rosario/0000-0003-2666-414X; Gueranger, Anne-Claire/0000-0003-0728-9313; Riboli, Elio/0000-0001-6795-6080; Skeie, Guri/0000-0003-2476-4251; Romaguera, Dora/0000-0002-5762-8558; Tjonneland, Anne/0000-0003-4385-2097; Lujan-Barroso, Leila/0000-0001-6224-1764; Olsen, Anja/0000-0003-4788-503X; PALLI, Domenico/0000-0002-5558-2437; Travier, Noemie/0000-0001-5228-7769; Buckland, Genevieve/0000-0003-2060-

6598; Agudo, Antonio/0000-0001-9900-5677; Dahm, Christina/0000-0003-0481-2893; Lund, Eiliv/0000-0002-8071-8711; Gonzalez, Carlos A/0000-0003-2822-9715; Overvad, Kim/0000-0001-6429-7921; SANCHEZ-PEREZ, MARIA JOSE/0000-0003-4817-0757 European Commission [QLG1-CT-2001-01049]; European Commission (SANCO); Ligtre le Cancer; Institut Gustave Roussy; Mutuelle Generale de l'Education Nationale; Institut National de la Sante et de la Recherche Medicale (INSERM) (France); Compagnia di San Paolo (Naples, Italy); German Cancer Aid; German Cancer Research Center; German Federal Ministry of Education and Research; Danish Cancer Society; Health Research Fund (FIS) of the Spanish Ministry of Health RTICC 'Red Tematica de Investigacion Cooperativa en Cancer [C03/10R06/0020]; Associazione Italiana per la Ricerca sul Cancro Funding Source: Custom European Commission(European Union (EU)European Commission Joint Research Centre); European Commission (SANCO)(European Union (EU)European Commission Joint Research Centre); Ligtre le Cancer; Institut Gustave Roussy; Mutuelle Generale de l'Education Nationale; Institut National de la Sante et de la Recherche Medicale (INSERM) (France)(Institut National de la Sante et de la Recherche Medicale (Inserm)); Compagnia di San Paolo (Naples, Italy)(Compagnia di San Paolo); German Cancer Aid(Deutsche Krebshilfe); German Cancer Research Center; German Federal Ministry of Education and Research(Federal Ministry of Education & Research (BMBF)); Danish Cancer Society(Danish Cancer Society); Health Research Fund (FIS) of the Spanish Ministry of Health RTICC 'Red Tematica de Investigacion Cooperativa en Cancer; Associazione Italiana per la Ricerca sul Cancro(Fondazione AIRC per la ricerca sul cancro) Grant sponsors: The European Commission FP5 project (QLG1-CT-2001-01049), "Europe Against Cancer" Programme of the European Commission (SANCO); Ligtre le Cancer, Institut Gustave Roussy, Mutuelle Generale de l'Education Nationale, Institut National de la Sante et de la Recherche Medicale (INSERM) (France); Compagnia di San Paolo (Naples, Italy); Mutuelle Generale de l'Education Nationale; German Cancer Aid; German Cancer Research Center; German Federal Ministry of Education and Research; Danish Cancer Society; Health Research Fund (FIS) of the Spanish Ministry of Health RTICC 'Red Tematica de Investigacion Cooperativa en Cancer; (Grant numbers: C03/10, R06/0020); the partiue concipating regional governments and institutions of Spain; Cancer Research, United Kingdom; Medical Research Council, United Kingdom; The Stroke Association, United Kingdom; British Heart Foundation; Department of Health, United Kingdom; Food Standards Agency, United Kingdom; The Wellcome Trust, United Kingdom; The Hellenic Health Foundation; The Stavros Niarchos Foundation; The Hellenic Ministry of Health (Greece); Italian Association for Research on Cancer (AIRC); Dutch Ministry of Public Health, Welfare and Sports (VWS), Netherlands Cancer Registry (NKR), LK Research Funds, Dutch Prevention Funds, Dutch ZON (Zorg Onderzoek Nederland), World Cancer Research Fund (WCRF), Statistics Netherlands (The Netherlands); Swedish Cancer Society; Swedish Scientific Council; Regional Government of Skane, Sweden; Helga-Nordforsk Centre of Excellence Programme in Food and Nutrition (Norway) 45 171 178 0 104 WILEY HOBOKEN 111 RIVER ST, HOBOKEN 07030-5774, NJ USA
0020-7136 1097-0215 INT J CANCER Int. J. Cancer
JUN 15 2013 132 12 2918 2927
10.1002/ijc.27958
http://dx.doi.org/10.1002/ijc.27958 10

Oncology	Science Citation Index Expanded (SCI-EXPANDED)	Oncology	126DV	23180513	Bronze			2025-06-24	WOS:000317593100022
J	Kulhánová, I; Bray, F; Fadhil, I; Al-Zahrani, AS; El-Basmy, A; Anwar, WA; Al-Omari, A; Shamseddine, A; Znaor, A; Soerjomataram, I			Kulhanova, Ivana; Bray, Freddie; Fadhil, Ibtihal; Al-Zahrani, Ali Saeed; El-Basmy, Amani; Anwar, Wagida A.; Al-Omari, Amal; Shamseddine, Ali; Znaor, Ariana; Soerjomataram, Isabelle			Profile of cancer in the Eastern Mediterranean region: The need for action	CANCER EPIDEMIOLOGY			English	Article			Cancer; Mortality; Incidence; Risk factors; Eastern Mediterranean region	HEPATITIS-C VIRUS; GLOBAL BURDEN; BREAST-CANCER; HEALTH OUTCOMES; CERVICAL-CANCER; AIR-POLLUTION; DISEASE; EGYPT; SCHISTOSOMIASIS; SMOKING	Background: Many countries in the Eastern Mediterranean region (EMR) are undergoing marked demographic and socioeconomic transitions that are increasing the cancer burden in region. We sought to examine the national cancer incidence and mortality profiles as a support to regional cancer control planning in the EMR. Methods: GLOBOCAN 2012 data were used to estimate cancer incidence and mortality by country, cancer type, sex and age in 22 EMR countries. We calculated age- standardized incidence and mortality rates (per 100,000) using direct method of standardization. Results: The cancer incidence and mortality rates vary considerably between countries in the EMR. Incidence rates were highest in Lebanon (204 and 193 per 100,000 in males and females, respectively). Mortality rates were highest in Lebanon (119) and Egypt (121) among males and in Somalia (117) among females. The profile of common cancers differs substantially by sex. For females, breast cancer is the most common cancer in all 22 countries, followed by cervical cancer, which ranks high only in the lower-income countries in the region. For males, lung, prostate, and colorectal cancer in combination represent almost 30% of the cancer burden in countries that have attained very high levels of human development. Conclusions: The most common cancers are largely amenable to preventive strategies by primary and/or secondary prevention, hence a need for effective interventions tackling lifestyle risk factors and infections. The high mortality observed from breast and cervical cancer highlights the need to break the stigmas and improve awareness surrounding these cancers. (C) 2017 Published by Elsevier Ltd.	[Kulhanova, Ivana; Bray, Freddie; Znaor, Ariana; Soerjomataram, Isabelle] Int Agcy Res Canc, Sect Canc Surveillance, 150 Cours Albert Thomas, F-69008 Lyon, France; [Fadhil, Ibtihal] WHO, Reg Off Eastern Mediterranean, Cairo, Egypt; [Al-Zahrani, Ali Saeed] King Faisal Specialist Hosp & Res Ctr, Riyadh, Saudi Arabia; [El-Basmy, Amani] Kuwait Canc Control Ctr, Epidemiol & Canc Registry Dept, Kuwait, Kuwait; [Anwar, Wagida A.] Ain Shams Univ, Dept Commun Environm & Occupat Med, Fac Med, Cairo, Egypt; [Al-Omari, Amal] King Hussein Canc Ctr, Amman, Jordan; [Shamseddine, Ali] Amer Univ Beirut, Div Hematol & Oncol, Dept Internal Med, Med Ctr, Beirut, Lebanon	World Health Organization; International Agency for Research on Cancer (IARC); World Health Organization; Egyptian Knowledge Bank (EKB); World Health Organization Egypt; King Faisal Specialist Hospital & Research Center; Egyptian Knowledge Bank (EKB); Ain Shams University; King Hussein Cancer Center; American University of Beirut	Kulhánová, I (corresponding author), Int Agcy Res

Canc, Sect Canc Surveillance, 150 Cours Albert Thomas, F-69008 Lyon, France.	kulhanovai@fellows.iarc.fr	Znaor, Ariana/H-6718-2019; Fadhil, Ibtihal/AAF-4123-2021; Al Omari, Amal/GXW-0942-2022; Kulhanova, Ivana/B-9253-2019Kulhanova, Ivana/0000-0002-9688-1548; Bray, Freddie/0000-0002-3248-7787; Al Omari, Amal/0000-0001-7571-3097	IARC WHO EMRO 2nd Action Plan [GR-IARC-2015-05-08-02]	IARC WHO EMRO 2nd Action Plan	Funding of this project has been provided through the IARC WHO EMRO 2nd Action Plan in the areas of cancer surveillance and risk factors 2015 (grant number: GR-IARC-2015-05-08-02).	43	51	54	0	13	ELSEVIER SCI LTD	OXFORD	THE BOULEVARD, LANGFORD LANE, KIDLINGTON, OXFORD OX5 1GB, OXON, ENGLAND	1877-7821	1877-783X		CANCER EPIDEMIOL	Cancer Epidemiol.	APR	2017	47
125	132		10.1016/j.canep.2017.01.009
http://dx.doi.org/10.1016/j.canep.2017.01.009
8	Oncology; Public, Environmental & Occupational Health
Science Citation Index Expanded (SCI-EXPANDED)	Oncology; Public, Environmental & Occupational Health	EO5BK	28268206
2025-06-24	WOS:000396707800018
J	Simkhada, P; van Teijlingen, E; Gurung, M; Wasti, SP
Simkhada, Padam; van Teijlingen, Edwin; Gurung, Manju; Wasti, Sharada P.		A survey of health problems of Nepalese female migrants workers in the Middle-East and Malaysia	BMC INTERNATIONAL HEALTH AND HUMAN RIGHTS
English	Article
Health problems; Migration; Exploitation; South Asia; Nepal; Women; Gulf countries; Malaysia	DOMESTIC WORKERS; HONG-KONG
Background: Nepal is a key supplier of labour for countries in the Middle East, India and Malaysia. As many more men than women leave Nepal to work abroad, female migrant workers are a minority and very much under-researched. The aim of the study was to explore the health problems of female Nepalese migrants working in the Middle-East and Malaysia. Methods: The study was conducted among 1010 women who were registered as migrant returnees at an organisation called Pourakhi Nepal. Secondary data were extracted from the records of the organisation covering the five-year period of July 2009 to July 2014. Results: The 1010 participants were aged 14 to 51 with a median age of 31 (IQR: 38-25) years. A quarter of respondents (24%) reported having experienced health problems while in the country of employment. Fever, severe illness and accidents were the most common health problems reported. Working for unlimited periods of time and not being able to change one's place of work were independently associated with a greater likelihood of health problems. Logistic regression shows that migrant women who are illiterate [OR = 1.56, 95% CI: 1.02 to 2.38, p = 0.042], who had changed their workplace [OR = 1.63, 95% CI: 1.14 to 2.32, p = 0.007], who worked unlimited periods of time [OR = 1.64, 95% CI: 1.44 to 1.93, p = 0.020], had been severely maltreated or tortured in the workplace [OR = 1.84, 95% CI: 1.15 to 2.92, p = 0.010], were not being paid on time [OR = 2.38, 95% CI: 1.60 to 3.55, p = 0.038] and migrant women who had family problems at home [OR = 3.48, CI 95%: 1.22 to 9.98, p = 0.020] were significantly associated with health problems in their host country in the Middle East. Conclusion: Female migrant workers face various work-related health risks, which are often related to exploitation. The Government of Nepal should initiate awareness campaigns about health risks and rights in relation to health care services in the host countries. Recruiting agencies/employers

should provide information on health risks and training for preventive measures. Raising awareness among female migrant workers can make a change in their working lives. [Simkhada, Padam] Liverpool John Moores Univ, Publ Hlth Inst, Henry Cotton Bldg,15-21 Webster St, Liverpool L3 2ET, Merseyside, England; [Simkhada, Padam; van Teijlingen, Edwin] Tribhuvan Univ, Manmohan Mem Inst Hlth Sci, Kirtipur, Nepal; [Simkhada, Padam; van Teijlingen, Edwin] Pokhara Univ, Nobel Coll, Pokhara, Nepal; [van Teijlingen, Edwin] Bournemouth Univ, Fac Hlth & Social Sci, Bournemouth, Dorset, England; [Gurung, Manju] Pourakhi Nepal, Makhamali Marg, Kathmandu, Nepal; [Wasti, Sharada P.] Georgetown Univ, Inst Reprod Hlth, Washington, DC USA Liverpool John Moores University; Tribhuvan University; Bournemouth University; Georgetown University Simkhada, P (corresponding author), Liverpool John Moores Univ, Publ Hlth Inst, Henry Cotton Bldg,15-21 Webster St, Liverpool L3 2ET, Merseyside, England.; Simkhada, P (corresponding author), Tribhuvan Univ, Manmohan Mem Inst Hlth Sci, Kirtipur, Nepal.; Simkhada, P (corresponding author), Pokhara Univ, Nobel Coll, Pokhara, Nepal. p.p.simkhada@ljmu.ac.uk

/CAE-7572-2022; Wasti, Sharada/AAO-7742-2021; Simkhada, Padam/LKK-2820-2024; van Teijlingen, Edwin/A-3727-2010 van Teijlingen, Edwin/0000-0001-5523-8583; Wasti, Dr. Sharada Prasad/0000-0001-8833-7801; Simkhada, Padam/0000-0002-5706-6479

Liverpool John Moores University, UK; Bournemouth University, UK Liverpool John Moores University, UK; Bournemouth University, UK This study had financial support from Liverpool John Moores University, UK and Bournemouth University, UK.

34 34 34 0 18 BIOMED CENTRAL LTD LONDON

236 GRAYS INN RD, FLOOR 6, LONDON WC1X 8HL, ENGLAND

1472-698X BMC INT HEALTH HUM R BMC Int. Health Hum. Rights. JAN 18 2018 18

4 10.1186/s12914-018-0145-7

http://dx.doi.org/10.1186/s12914-018-0145-7 7

Health Policy & Services; Public, Environmental & Occupational Health Social Science Citation Index (SSCI)

Health Care Sciences & Services; Public, Environmental & Occupational Health FS9HG 29347938 Green Published, gold

2025-06-24 WOS:000422727600001

J Figlioli, G; Kvist, A; Tham, E; Soukupova, J; Kleiblova, P; Muranen, TA; Andrieu, N; Azzollini, J; Balmaña, J; Barroso, A; Benítez, J; Bertelsen, B; Blanco, A; Bonanni, B; Borg, Å; Brunet, J; Calistri, D; Calvello, M; Chvojka, S; Cortesi, L; Darder, E; Del Valle, J; Diez, O; Eon-Marchais, S; Fostira, F; Gensini, F; Houdayer, C; Janatova, M; Kiiski, J; Konstantopoulou, I; Kubelka-Sabit, K; Lázaro, C; Lesueur, F; Manoukian, S; Marcinkute, R; Mickys, U; Moncoutier, V; Myszka, A; Tu, ND; Nielsen, FC; Norvilas, R; Olah, E; Osorio, A; Papi, L; Peissel, B; Peixoto, A; Plaseska-Karanfilska, D; Pócza, T; Rossing, M; Rudaitis, V; Santamariña, M; Santos, C; Smichkoska, S; Southey, MC; Stoppa-Lyonnet, D; Teixeira, M; Törngren, T; Toss, A; Urioste, M; Vega, A; Vlckova, Z; Yannoukakos, D; Zampiga, V; Kleibl, Z; Radice, P; Nevanlinna, H; Ehrencrona, H; Janavicius, R; Peterlongo, P

Figlioli, Gisella; Kvist, Anders; Tham, Emma; Soukupova, Jana; Kleiblova, Petra; Muranen, Taru A.; Andrieu, Nadine; Azzollini, Jacopo; Balmana, Judith; Barroso, Alicia; Benitez, Javier; Bertelsen, Birgitte; Blanco, Ana; Bonanni, Bernardo; Borg, Ake; Brunet, Joan; Calistri, Daniele; Calvello, Mariarosaria; Chvojka, Stepan; Cortesi, Laura; Darder, Esther; Del

Valle, Jesus; Diez, Orland; Eon-Marchais, Severine; Fostira, Florentia; Gensini, Francesca; Houdayer, Claude; Janatova, Marketa; Kiiski, Johanna, I; Konstantopoulou, Irene; Kubelka-Sabit, Katerina; Lazaro, Conxi; Lesueur, Fabienne; Manoukian, Siranoush; Marcinkute, Ruta; Mickys, Ugnius; Moncoutier, Virginie; Myszka, Aleksander; Tu Nguyen-Dumont; Nielsen, Finn Cilius; Norvilas, Rimvydas; Olah, Edith; Osorio, Ana; Papi, Laura; Peissel, Bernard; Peixoto, Ana; Plaseska-Karanfilska, Dijana; Pocza, Timea; Rossing, Maria; Rudaitis, Vilius; Santamarina, Marta; Santos, Catarina; Smichkoska, Snezhana; Southey, Melissa C.; Stoppa-Lyonnet, Dominique; Teixeira, Manuel; Torngren, Therese; Toss, Angela; Urioste, Miguel; Vega, Ana; Vlckova, Zdenka; Yannoukakos, Drakoulis; Zampiga, Valentina; Kleibl, Zdenek; Radice, Paolo; Nevanlinna, Heli; Ehrencrona, Hans; Janavicius, Ramunas; Peterlongo, Paolo ENIGMA Consortium; ENIGMA Consortium; GENESIS Study Collaborators; SWE-BRCA Grp The Spectrum of FANCM Protein Truncating Variants in European Breast Cancer Cases CANCERS English Article breast cancer predisposition; breast cancer risk factors; FANCM truncating variants; mutation spectrum; PTVs RISK; MUTATIONS; BRCA1; C.5791C-GREATER-THAN-T; ANEMIA; GENE Germline protein truncating variants (PTVs) in the FANCM gene have been associated with a 2-4-fold increased breast cancer risk in case-control studies conducted in different European populations. However, the distribution and the frequency of FANCM PTVs in Europe have never been investigated. In the present study, we collected the data of 114 European female breast cancer cases with FANCM PTVs ascertained in 20 centers from 13 European countries. We identified 27 different FANCM PTVs. The p.Gln1701* PTV is the most common PTV in Northern Europe with a maximum frequency in Finland and a lower relative frequency in Southern Europe. On the contrary, p.Arg1931* seems to be the most common PTV in Southern Europe. We also showed that p.Arg658*, the third most common PTV, is more frequent in Central Europe, and p.Gln498Thrfs*7 is probably a founder variant from Lithuania. Of the 23 rare or unique FANCM PTVs, 15 have not been previously reported. We provide here the initial spectrum of FANCM PTVs in European breast cancer cases. [Figlioli, Gisella; Peterlongo, Paolo] IFOM, Genome Diagnost Program, FIRC Inst Mol Oncol, I-20139 Milan, Italy; [Kvist, Anders; Borg, Ake; Torngren, Therese] Lund Univ, Dept Clin Sci Lund, Div Oncol & Pathol, SE-22381 Lund, Sweden; [Tham, Emma] Karolinska Univ Hosp, Dept Clin Genet, S-17176 Stockholm, Sweden; [Tham, Emma] Karolinska Inst, Dept Mol Med, S-17176 Stockholm, Sweden; [Soukupova, Jana; Janatova, Marketa; Kleibl, Zdenek] Charles Univ Prague, Fac Med 1, Inst Biochem & Expt Oncol, Prague 12853, Czech Republic; [Kleiblova, Petra] Gen Univ Hosp, Inst Biol & Med Genet, Prague 12800, Czech Republic; [Kleiblova, Petra] Charles Univ Prague, Fac Med 1, Prague 12800, Czech Republic; [Muranen, Taru A.; Kiiski, Johanna, I; Nevanlinna, Heli] Helsinki Univ Hosp, Dept Obstet & Gynecol, Helsinki 00029, Finland; [Muranen, Taru A.; Kiiski, Johanna, I; Nevanlinna, Heli] Univ Helsinki, HUS, Helsinki 00029, Finland; [Andrieu, Nadine; Eon-Marchais, Severine; Lesueur, Fabienne; GENESIS Study Collaborators] PSL Univ, Inst Curie, U900, INSERM, F-75005 Paris, France; [Andrieu, Nadine; Eon-Marchais, Severine; Lesueur, Fabienne] Mines ParisTech, F-77300 Fontainebleau, France; [Azzollini, Jacopo; Manoukian, Siranoush; Peissel, Bernard] IRCCS Ist Nazl Tumori, Dept Med Oncol & Hematol, Unit Med Genet Fdn, I-

20133 Milan, Italy; [Balmana, Judith; Diez, Orland] Vall dHebron Inst Oncol VHIO, Hereditary Canc Grp, Barcelona 08035, Spain; [Balmana, Judith] Univ Hosp Vall dHebron, Dept Med Oncol, Barcelona 08035, Spain; [Barroso, Alicia; Benitez, Javier; Osorio, Ana] Spanish Natl Canc Res Ctr, Human Genet Grp, Human Canc Genet Programme, Madrid 28029, Spain; [Benitez, Javier; Osorio, Ana] Spanish Network Rare Dis CIBERER, Madrid 28029, Spain; [Benitez, Javier] Spanish Natl Canc Res Ctr, Genotyping Unit, CEGEN, Human Canc Genet Programme, Madrid 28029, Spain; [Bertelsen, Birgitte; Nielsen, Finn Cilius; Rossing, Maria] Copenhagen Univ Hosp, Rigshosp, Ctr Genom Med, DK-2100 Copenhagen, Denmark; [Blanco, Ana; Santamarina, Marta; Vega, Ana] Fdn Publ Galega Med Xenam SERGAS, Santiago De Compostela 15706, Spain; [Blanco, Ana; Santamarina, Marta; Vega, Ana] Inst Invest Sanitaria Santiago de Compostela IDIS, Santiago De Compostela 15706, Spain; [Blanco, Ana; Santamarina, Marta; Vega, Ana] Ctr Invest Red Enfermedades Raras CIBERER, Madrid 28029, Spain; [Bonanni, Bernardo; Calvello, Mariarosaria] European Inst Oncol IRCCS, IEO, Div Canc Prevent & Genet, I-20141 Milan, Italy; [Brunet, Joan; Darder, Esther; Del Valle, Jesus; Lazaro, Conxi] CIBERONC, CIBERONC, Catalan Inst Oncol, Hereditary Canc Program,ONCOBELL,IDIBELL,IDIBGI,I, Barcelona 08908, Spain; [Calistri, Daniele; Zampiga, Valentina] Ist Sci Romagnolo Studio & Cura Tumori IRST IRCCS, Biosci Lab, I-47014 Meldola, Italy; [Chvojka, Stepan] Ctr Med Genet & Reprod Med, Gennet, Prague 17000, Czech Republic; [Cortesi, Laura; Toss, Angela] Univ Modena Hosp, I-41124 Modena, Italy; [Diez, Orland] Univ Hosp Vall dHebron, Area Mol & Clin Genet, Barcelona 08035, Spain; [ENIGMA Consortium; ENIGMA Consortium] QIMR Berghofer Med Res Inst, Brisbane, Qld, Australia; [Fostira, Florentia; Konstantopoulou, Irene; Yannoukakos, Drakoulis] Natl Ctr Sci Res Demokritos, Mol Diagnost Lab, InRASTES, Athens 15310, Greece; [Gensini, Francesca; Papi, Laura] Univ Florence, Dept Expt & Clin Biomed Sci, I-50134 Florence, Italy; [Houdayer, Claude] Genet Dept, F-76000 Rouen, France; [Houdayer, Claude] Normandy Univ, INSERM, Normandy Ctr Genom & Personalized Med, Rouen Univ Hosp,UNIROUEN,U1245, Rouen, France; [Kubelka-Sabit, Katerina] Clin Hosp Acibadem Sistina, Dept Histopathol & Cytol, Skopje 1000, North Macedonia; [Marcinkute, Ruta; Norvilas, Rimvydas; Janavicius, Ramunas] Vilnius Univ Hosp, Hereditary Canc Ctr, Hematol Oncol & Transfus Med Ctr, Santaros Klin, LT-08410 Vilnius, Lithuania; [Mickys, Ugnius] Vilnius Univ Hosp, Santaros Klin, Natl Ctr Pathol, LT-08410 Vilnius, Lithuania; [Moncoutier, Virginie; Stoppa-Lyonnet, Dominique] Paris Descartes Univ, INSERM, U830, Serv Genet,Inst Curie, F-75005 Paris, France; [Ehrencrona, Hans; SWE-BRCA Grp] Lund Univ, Dept Lab Med, Div Clin Genet, SE-22100 Lund, Sweden; [Myszka, Aleksander] Univ Rzeszow, Inst Med Sci, PL-35310 Rzeszow, Poland; [Tu Nguyen-Dumont; Southey, Melissa C.] Monash Univ, Sch Clin Sci, Precis Med, Monash Hlth, Clayton 3168, Australia; [Tu Nguyen-Dumont; Southey, Melissa C.] Univ Melbourne, Dept Clin Pathol, Melbourne 3010, Australia; [Norvilas, Rimvydas; Janavicius, Ramunas] State Res Inst Ctr Innovat Med, Dept Expt Prevent & Clin Med, LT-08410 Vilnius, Lithuania; [Olah, Edith; Pocza, Timea] Natl Inst Oncol, Dept Mol Genet, H-1122 Budapest, Hungary; [Peixoto, Ana; Santos, Catarina; Teixeira, Manuel] Portuguese Oncol Inst Porto IPO Porto, Dept Genet, P-4200072 Porto, Portugal; [Plaseska-Karanfilska, Dijana] Macedonian Acad Sci & Arts, Res Ctr Genet Engn & Biotechnol Georgi D Efremov, Skopje 1000, North Macedonia; [Rudaitis, Vilius] Vilnius Univ

Hosp, Santaros Klin, Dept Gynaecol, Ctr Obster & Gynaecol, LT-08410 Vilnius, Lithuania; [Smichkoska, Snezhana] Ss Cyril & Methodius Univ Skopje, Med Fac, Univ Clin Radiotherapy & Oncol, Skopje 1000, North Macedonia; [Teixeira, Manuel] Univ Porto, Biomed Sci Inst, P-4050313 Porto, Portugal; [Urioste, Miguel] Spanish Natl Canc Res Ctr, Familial Canc Clin Unit, Human Canc Genet Programme, Madrid 28029, Spain; [Vlckova, Zdenka] GHC Genet, Dept Med Genet, Prague 11000, Czech Republic; [Radice, Paolo] Fdn IRCCS Ist Nazl Tumori, Dept Res, Unit Mol Bases Genet Risk & Genet Testing, I-20133 Milan, Italy; [Ehrencrona, Hans] Reg Skane, Lab Med, Off Med Serv, Dept Clin Genet & Pathol, SE-22100 Lund, Sweden

IFOM - FIRC Institute of Molecular Oncology; Lund University; Karolinska Institutet; Karolinska University Hospital; Karolinska Institutet; Charles University Prague; General University Hospital Prague; Charles University Prague; University of Helsinki; Helsinki University Central Hospital; University of Helsinki; Universite PSL; UNICANCER; Institut Curie; Institut National de la Sante et de la Recherche Medicale (Inserm); Universite PSL; MINES ParisTech; Fondazione IRCCS Istituto Nazionale Tumori Milan; Vall d'Hebron Institut d'Oncologia (VHIO); Hospital Universitari Vall d'Hebron; Centro Nacional de Investigaciones Oncologicas (CNIO); CIBER - Centro de Investigacion Biomedica en Red; CIBERER; Centro Nacional de Investigaciones Oncologicas (CNIO); University of Copenhagen; Copenhagen University Hospital; Rigshospitalet; CIBER - Centro de Investigacion Biomedica en Red; CIBERER; IRCCS European Institute of Oncology (IEO); Institut d'Investigacio Biomedica de Bellvitge (IDIBELL); Universitat de Girona; Girona University Hospital Dr. Josep Trueta; Institut d'Investigacio Biomedica de Girona (IDIBGI); CIBER - Centro de Investigacion Biomedica en Red; CIBERONC; University of Barcelona; Institut Catala d'Oncologia; IRCCS Meldola (IRST); Universita di Modena e Reggio Emilia; Universita di Modena e Reggio Emilia Hospital; Hospital Universitari Vall d'Hebron; QIMR Berghofer Medical Research Institute; National Centre of Scientific Research "Demokritos"; University of Florence; Universite de Rouen Normandie; CHU de Rouen; Institut National de la Sante et de la Recherche Medicale (Inserm); Vilnius University Hospital Santariskiu Klinikos; Vilnius University Hospital Santariskiu Klinikos; Universite PSL; UNICANCER; Institut Curie; Institut National de la Sante et de la Recherche Medicale (Inserm); Universite Paris Cite; Lund University; University of Rzeszow; Monash Health; Monash University; University of Melbourne; State Research Institute Centre for Innovative Medicine; National Institute of Oncology Hungary; Portuguese Institute of Oncology; Vilnius University Hospital Santariskiu Klinikos; Saints Cyril & Methodius University of Skopje; Universidade do Porto; Centro Nacional de Investigaciones Oncologicas (CNIO); Fondazione IRCCS Istituto Nazionale Tumori Milan Peterlongo, P (corresponding author), IFOM, Genome Diagnost Program, FIRC Inst Mol Oncol, I-20139 Milan, Italy. gisella.figlioli@ifom.eu; Anders.Kvist@med.lu.se; Emma.Tham@ki.se; jana.soukupova@lf1.cuni.cz; pekleje@lf1.cuni.cz; taru.a.muranen@helsinki.fi; nadine.andrieu@curie.fr; Jacopo.Azzollini@istitutotumori.mi.it; Jbalmana@vhebron.net; abarroso@cnio.es; jbenitez@cnio.es; Birgitte.Bertelsen@regionh.dk; ana.blanco@usc.es; bernardo.bonanni@ieo.it; ake.borg@med.lu.se; Jbrunet@iconcologia.net; daniele.calistri@irst.emr.it; mariarosaria.calvello@ieo.it; stepan.chvojka@gennet.cz;

hbc@unimore.it; edarder@iconcologia.net;
jdelvalle@iconcologia.net; odiez@vhio.net; Severine.Eon-Marchais@curie.fr; florentia_fostira@hotmail.com;
francesca.gensini@unifi.it; claude.houdayer@chu-rouen.fr;
mjana@lfl.cuni.cz; reenakon@gmail.com;
katerina.kubelka@acibademsistina.mk; conxi.lazaro@gmail.com;
fabienne.lesueur@curie.fr;
Siranoush.Manoukian@istitutotumori.mi.it;
ruta.marcinkute@gmail.com; ugnius.mickys@vpc.lt;
virginie.moncoutier@curie.fr; amyszka@univ.rzeszow.pl; tu.nguyen-dumont@monash.edu; finn.cilius.nielsen@regionh.dk;
rimvydas.norvilas@santa.lt; e.olah@oncol.hu; aosorio@cnio.es;
laura.papi@unifi.it; bernard.peissel@istitutotumori.mi.it;
analuisamoura@ipoporto.min-saude.pt; dijana@manu.edu.mk;
timipocza@oncol.hu; caroline.maria.rossing@regionh.dk;
Vilius.Rudaitis@santa.lt; santamarinapena@gmail.com;
catarinasantos@ipoporto.min-saude.pt; smicko@t.mk;
melissa.southey@monash.edu; Dominique.StoppaLyonnet@curie.fr;
manuelteixeira@ipoporto.min-saude.pt; Therese.Torngren@med.lu.se;
angela.toss@unimore.it; murioste@cnio.es; ana.vega@usc.es;
Vlckova@ghc.cz; yannouka@gmail.com; valentina.zampiga@irst.emr.it;
zdekleje@lfl.cuni.cz; Paolo.Radice@istitutotumori.mi.it;
Heli.Nevanlinna@hus.fi; hans.ehrencrona@skane.se;
Ramunas.Janavicius@santa.it; paolo.peterlongo@ifom.eu   Myszka, Aleksander/HZH-6617-2023; GARCIA, CONXI/Q-2410-2016; Calvello, Mariarosaria/AAM-8866-2020; Peterlongo, Paolo/Q-4717-2018;
Southey, Melissa/CAA-7064-2022; Soukupová, Jana/ABI-2077-2020;
Tan, Yen/AAW-2357-2021; Janavicius, Ramunas/NGS-2944-2025; Vega, Ana/R-4758-2019; manoukian, siranoush/E-7132-2017; Azzollini, Jacopo/E-7134-2017; Peixoto, Ana/KPA-1199-2024; Kubelka-Sabit, Katerina/AAC-5828-2020; Mickys, Ugnius/GRO-6315-2022; Calistri, Daniele/K-5788-2016; Fostira, Florentia/Z-5730-2019; Nielsen, Finn/AAA-4926-2020; Bonanni, Bernardo/AAM-7928-2020; Karanfilska, Dijana/P-6096-2018; Zampiga, Valentina/AAA-6198-2021;
Konstantopoulou, Irene/Y-3074-2018; Peissel, Bernard/E-8187-2017;
del Valle, Jesús/AAE-2216-2022; balmaña, judith/AAH-1908-2019;
ortiz, Javier/H-5232-2015; Cortesi, Laura/J-6898-2019; ANDRIEU, Nadine/H-4255-2014; Muranen, Taru/AAC-8418-2019; Blanco, Ana/NDR-9630-2025; del Valle, Jesus/I-2527-2015; Kleiblova, Petra/K-9899-2017; Brunet, Joan/C-5292-2018; Ehrencrona, Hans/M-5619-2014;
Rodrigues Teixeira, Manuel Antonio/E-4885-2011; Kleibl, Zdenek/A-2009-2008; Lesueur, Fabienne/R-2178-2017; Konstantopoulou, Irene/B-5309-2009; Osorio, Ana/I-4324-2014; GENSINI, FRANCESCA/LPQ-3536-2024   Kiiski, Johanna I./0000-0002-4089-7209; Peterlongo, Paolo/0000-0001-6951-6855; Santamarina, Marta/0000-0002-2022-4162; Houdayer, Claude/0000-0002-5190-0389;
Muranen, Taru/0000-0002-5895-1808; Nguyen-Dumont, Tu/0000-0002-6217-0182; del Valle, Jesus/0000-0003-3607-7045; Janatova, Marketa/0000-0003-2816-6769; Santos, Catarina/0000-0002-9102-757X;
Santamarina, Marta/0000-0001-5616-9946; Kvist, Anders/0000-0002-1358-0695; Kleiblova, Petra/0000-0002-4806-9854; Cortesi, Laura/0000-0001-8950-8561; Brunet, Joan/0000-0003-1945-3512;
Zampiga, Valentina/0000-0002-7356-8153; Ehrencrona, Hans/0000-0002-5589-3622; Vega, Ana/0000-0002-7416-5137; Rodrigues Teixeira, Manuel Antonio/0000-0002-4896-5982; Kubelka-Sabit, Katerina/0000-0002-3941-2219; Peixoto, Ana/0000-0002-1404-5897; Kleibl, Zdenek/0000-0003-2050-9667; Lesueur, Fabienne/0000-0001-7404-4549;
Soukupova, Jana/0000-0003-2413-1542; Calvello, Mariarosaria/0000-

0003-2113-8503; Figlioli, Gisella/0000-0002-0740-1363; Myszka, Aleksander/0000-0002-2931-7920; Konstantopoulou, Irene/0000-0002-0470-0309; Osorio, Ana/0000-0001-8124-3984; GENSINI, FRANCESCA/0000-0001-6669-420X Associazione Italiana Ricerca sul Cancro (AIRC; IG2015) [16732]; Fondazione Umberto Veronesi; Italian Ministry of Health; Region Stockholm (ALF); Ministry of Health of the Czech Republic [NV16-29959A]; Instituto de Salud Carlos III [PI16/00440, PI19/00640]; European Regional Development Fund (ERDF); Spanish Network on Rare Diseases (CIBERER); BRIDGES project H2020; Ligue Nationale contre le Cancer [PRE05/DSL, PRE07/DSL, PRE11/NA]; French National Institute of Cancer (INCa grant) [b2008-029/LL-LC]; comprehensive cancer center SiRIC, (Site de Recherche Integree sur le Cancer) [INCa-DGOS-4654]; Helsinki University Hospital Research Fund; Sigrid Juselius Foundation; cancer Society of Finland; Spanish Health Research Foundation, Instituto de Salud Carlos III (ISCIII) through Research Activity Intensification Program [INT15/00070, INT16/00154, INT17/00133]; Spanish Health Research Foundation, Instituto de Salud Carlos III (ISCIII) through Centro de Investigacion Biomedica en Red de Enferemdades Raras CIBERER [ACCI 2016: ER17P1AC7112/2018]; Autonomous Government of Galicia (Consolidation and structuring program) [IN607B]; Fundacion Mutua Madrilena; Australian National Health and Medical Research Council [APP1029974, APP1074383]; Victorian Life Sciences Computation Initiative grant on its Peak Computing Facility, an initiative of the Victorian Government [VR0182]; Hungarian Research Grants [KTIA-OTKA CK-80745, NKFI OTKA K-112228]; Research Council of Lithuania [SEN18/2015, P-MIP-20-25]; Carlos III National Health Institute - FEDER funds-a way to build Europe [PI16/00563, PI19/00553]; Carlos III National Health Institute - FEDER funds-a way to build Europe [CIBERONC]; Government of Catalonia [Pla estrategic de recerca i innovacio en salut (PERIS) Project MedPerCan] [2017SGR1282, 2017SGR496]; CERCA program Associazione Italiana Ricerca sul Cancro (AIRC; IG2015)(Fondazione AIRC per la ricerca sul cancro); Fondazione Umberto Veronesi(Fondazione Umberto Veronesi); Italian Ministry of Health(Ministry of Health, Italy); Region Stockholm (ALF); Ministry of Health of the Czech Republic(Ministry of Health, Czech Republic); Instituto de Salud Carlos III(Instituto de Salud Carlos IIISpanish Government); European Regional Development Fund (ERDF)(European Union (EU)); Spanish Network on Rare Diseases (CIBERER); BRIDGES project H2020; Ligue Nationale contre le Cancer(Ligue nationale contre le cancer); French National Institute of Cancer (INCa grant); comprehensive cancer center SiRIC, (Site de Recherche Integree sur le Cancer); Helsinki University Hospital Research Fund; Sigrid Juselius Foundation(Sigrid Juselius Foundation); cancer Society of Finland; Spanish Health Research Foundation, Instituto de Salud Carlos III (ISCIII) through Research Activity Intensification Program; Spanish Health Research Foundation, Instituto de Salud Carlos III (ISCIII) through Centro de Investigacion Biomedica en Red de Enferemdades Raras CIBERER; Autonomous Government of Galicia (Consolidation and structuring program); Fundacion Mutua Madrilena(Instituto de Salud Carlos III); Australian National Health and Medical Research Council(National Health & Medical Research Council (NHMRC) of Australia); Victorian Life Sciences Computation Initiative grant on its Peak Computing Facility, an initiative of the Victorian Government; Hungarian Research Grants; Research Council of Lithuania(Research Council of Lithuania

(LMTLT)); Carlos III National Health Institute - FEDER funds-a way to build Europe; Carlos III National Health Institute - FEDER funds-a way to build Europe [CIBERONC]; Government of Catalonia [Pla estrategic de recerca i innovacio en salut (PERIS) Project MedPerCan]; CERCA program   This research was partially funded by Associazione Italiana Ricerca sul Cancro (AIRC; IG2015 no.16732) to P. Peterlongo, a fellowship from Fondazione Umberto Veronesi to G. Figlioli and by the Italian Ministry of Health with Ricerca Corrente and 5x1000 funds. E. Tham is supported by Region Stockholm (ALF). The Czech study was supported by a grant of the Ministry of Health of the Czech Republic NV16-29959A. CNIO study was partially supported by projects PI16/00440 and PI19/00640, supported by the Instituto de Salud Carlos III, cofunded by European Regional Development Fund (ERDF), the Spanish Network on Rare Diseases (CIBERER) and BRIDGES project H2020. Financial support for GENESIS resource and genotyping was provided by the Ligue Nationale contre le Cancer (grants PRE05/DSL, PRE07/DSL, PRE11/NA), the French National Institute of Cancer (INCa grant No b2008-029/LL-LC) and the comprehensive cancer center SiRIC, (Site de Recherche Integree sur le Cancer: Grant INCa-DGOS-4654). HEBCS was funded by Helsinki University Hospital Research Fund, Sigrid Juselius Foundation, The cancer Society of Finland. A.Vega is supported by the Spanish Health Research Foundation, Instituto de Salud Carlos III (ISCIII) through Research Activity Intensification Program (contract grant numbers: INT15/00070, INT16/00154, INT17/00133), and through Centro de Investigacion Biomedica en Red de Enferemdades Raras CIBERER (ACCI 2016: ER17P1AC7112/2018); Autonomous Government of Galicia (Consolidation and structuring program: IN607B), and by the Fundacion Mutua Madrilena (call 2018). This work was supported by the Australian National Health and Medical Research Council (APP1029974 and APP1074383) and by a Victorian Life Sciences Computation Initiative grant (number VR0182) on its Peak Computing Facility, an initiative of the Victorian Government. T.N-D is a Career Development Fellow of the National Breast Cancer Foundation (Australia, ECF-17-001). M.C.S. is a National Health and Medical Research Council (Australia) Senior Research Fellow. The Hungarian Breast and Ovarian Cancer Study was supported by Hungarian Research Grants KTIA-OTKA CK-80745 and NKFI OTKA K-112228 to E. Olah. Lithuanian study was supported by The Research Council of Lithuania grant SEN18/2015 and P-MIP-20-25 to R. Janavicius. The ICO was supported by the Carlos III National Health Institute funded by FEDER funds-a way to build Europe-[PI16/00563, PI19/00553 and CIBERONC]; the Government of Catalonia [Pla estrategic de recerca i innovacio en salut (PERIS) Project MedPerCan, 2017SGR1282 and 2017SGR496]; and CERCA program.
15 10 11 0 15 MDPI BASEL ST ALBAN-ANLAGE 66, CH-4052 BASEL, SWITZERLAND 2072-6694 CANCERS
Cancers FEB 2020 12 2
292 10.3390/cancers12020292
http://dx.doi.org/10.3390/cancers12020292 10
Oncology Science Citation Index Expanded (SCI-EXPANDED)
Oncology KY3ND 31991861 Green Published, Green Accepted, gold 2025-06-24 WOS:000522477300038
J Mansori, K; Khazaei, S; Khosravi Shadmani, F; Hanis, SM; Jenabi, E; Soheylizad, M; Sani, M; Ayubi, E
Mansori, Kamyar; Khazaei, Salman; Khosravi Shadmani, Fatemeh; Hanis, Shiva Mansouri; Jenabi, Ensiyeh; Soheylizad,

Mokhtar; Sani, Mohadeseh; Ayubi, Erfan Global Inequalities in Cervical Cancer Incidence and Mortality MIDDLE EAST JOURNAL OF CANCER English Article

Human Development Index; Cervical cancer; Correlation; GLOBOCAN Cancer Project HUMAN-PAPILLOMAVIRUS; SURVIVAL; COUNTRIES; BURDEN; PREVALENCE; PATTERNS; OBESITY; REGIONS; TRENDS; BREAST Background: Cervical cancer is the third most common cancer in women after breast and colorectal cancers, and one of the leading causes of cancer death among women worldwide. The aim of this study is to determine the associations of cervical cancer incidence and mortality rates with the Human Development Index. Methods: Information of the incidence and mortality rates for cervical cancer were obtained from the GLOBOCAN Cancer Project for 2012 and data for the Human Development Index for 2013 from the World Bank database. We used linear regression models to assess the Human Development Index effect on cervical cancer occurrence rates. Inequality in the age-standardized incidence and mortality rates of cervical cancer according to the Human Development Index were assessed by the concentration index. Results: The results showed substantially higher cervical cancer incidence and mortality rates in regions with low and medium Human Development Index compared to regions that had substantially elevated Human Development Index. The death and incidence from cervical cancer were more concentrated in low Human Development Index countries. There was a significant negative association between the cervical cancer incidence and mortality rates with all the components of the Human Development Index, including life expectancy (B=-0.98, P<0.001), mean years of schooling (B=-1.86, P<0.001), gross national income (B=-0.38, P<0.001), urbanization level (B=-0.29, P<0.001), and age standardized obesity (B=-0.45, P<0.001). Conclusion: Cervical cancer is a significant public health problem in countries with low Human Development Index and requires the implementation of prevention programs and screening for early detection and treatment. [Mansori, Kamyar] Gonabad Univ Med Sci, Social Dev & Hlth Promot Res Ctr, Gonabad, Iran; [Mansori, Kamyar] Iran Univ Med Sci, Dept Epidemiol, Sch Publ Hlth, Tehran, Iran; [Khazaei, Salman] Hamadan Univ Med Sci, Sch Publ Hlth, Dept Epidemiol, Hamadan, Iran; [Khosravi Shadmani, Fatemeh] Shahid Beheshti Univ Med Sci, Sch Publ Hlth, Dept Epidemiol, Tehran, Iran; [Hanis, Shiva Mansouri] Dezful Univ Med Sci, Sch Publ Hlth, Dezful, Iran; [Jenabi, Ensiyeh] Hamadan Univ Med Sci, Pediat Dev Disorders Res Ctr, Hamadan, Iran; [Soheylizad, Mokhtar] Hamadan Univ Med Sci, Sch Publ Hlth, Hamadan, Iran; [Sani, Mohadeseh] Zabol Univ Med Sci, Sch Med, Zabol, Iran; [Ayubi, Erfan] Zahedan Univ Med Sci, Sch Med, Dept Community Med, Zahedan, Iran Iran University of Medical Sciences; Hamadan University of Medical Sciences; Shahid Beheshti University Medical Sciences; Hamadan University of Medical Sciences; Hamadan University of Medical Sciences; Zahedan University of Medical Sciences Ayubi, E (corresponding author), Zahedan Univ Med Sci, Sch Med, Dept Community Med, Zahedan, Iran.

aubi65@gmail.com Ayubi, Erfan/AAD-3213-2019; Khazaei, Salman/O-4553-2019; Shadmani, Fatemeh/Y-9533-2019; Jenabi, Ensiyeh/S-6919-2017 Khazaei, Salman/0000-0001-5918-2310; Jenabi, Ensiyeh/0000-0002-4536-0814; Soheylizad, Mokhtar/0000-0002-0900-0125 38 7 8 1 4

SHIRAZ UNIV MEDICAL SCIENCESSHIRAZ NEMAZEE HOSPITAL, SHIRAZ, 71934, IRAN 2008-6709 2008-6687 MIDDLE EAST J

CANCER Middle East J. Cancer JUL 2018 9 3
235 242 8
Oncology Emerging Sources Citation Index (ESCI) Oncology
GL0OX 2025-06-24 WOS:000436790600010
J Gany, F; Ayash, C; Raad, N; Wu, ML; Roberts-Eversley, N; Mahmoud, H; Fouad, Y; Fahmy, Y; Asar, H; Salama, A; El-Shinawi, M
Gany, Francesca; Ayash, Claudia; Raad, Noor; Wu, Minlun; Roberts-Eversley, Nicole; Mahmoud, Hani; Fouad, Yousef; Fahmy, Yara; Asar, Hadeel; Salama, Ahmed; El-Shinawi, Mohamed
Financial and food security challenges of Egyptian women undergoing breast cancer treatment SUPPORTIVE CARE IN CANCER English Article
Breast cancer; Egypt; Financial toxicity; Food insecurity; Patient navigation PATIENT NAVIGATION; CARE; DIAGNOSIS; COUNTRIES; BARRIERS; IMPACT
Background Breast cancer treatment is an established cause of financial toxicity, and associated costs may contribute to higher mortality and morbidity rates. In Egypt, breast cancer incidence and mortality rates are among the highest in the Middle East. Late-stage diagnosis is common, and disease occurs at an earlier age than in Europe and North America. Out-of-pocket payments are the primary means of financing healthcare in Egypt, and socioeconomic factors have been shown to significantly impact access to cancer screening and treatment. Methods An observational cross-sectional study was conducted among breast cancer patients at Ain Shams University Hospitals in Cairo from 2013 to 2015. Results One hundred women with breast cancer participated. There was a high need for financial assistance (66.0%) and patients with financial needs had great difficulty affording medications (80.0%). A number of patients had lost their jobs following diagnosis, with 32.7% employed prior to diagnosis and 15.3% afterwards. Nearly one-half of participants were classified as food insecure, and nearly one-third reported difficulty affording transportation costs. Conclusions This is the first study to describe socioeconomic needs and financial impact among a cohort of Egyptian women undergoing breast cancer treatment. The findings highlight the financial impact of breast cancer treatment on a cohort of Egyptian breast cancer patients and the need for a multidisciplinary approach to help them access and mitigate the costs of treatment. Recommendations include implementing patient financial navigation services and producing printed materials to inform patients of resources to help mitigate the treatment's financial impact.
[Gany, Francesca; Ayash, Claudia; Raad, Noor; Wu, Minlun; Roberts-Eversley, Nicole; Mahmoud, Hani] Mem Sloan Kettering Canc Ctr, Immigrant Hlth & Canc Dispar Serv, Dept Psychiat & Behav Sci, 1275 York Ave, New York, NY 10021 USA; [Fouad, Yousef; Fahmy, Yara; Asar, Hadeel; Salama, Ahmed; El-Shinawi, Mohamed] Ain Shams Univ, Dept Gen Surg, Cairo, Egypt
Memorial Sloan Kettering Cancer Center; Egyptian Knowledge Bank (EKB); Ain Shams University Ayash, C (corresponding author), Mem Sloan Kettering Canc Ctr, Immigrant Hlth & Canc Dispar Serv, Dept Psychiat & Behav Sci, 1275 York Ave, New York, NY 10021 USA.
ayashc@mskcc.org Fouad, Yousef/AAJ-1730-2021; Fahmy, Yara/AAV-9647-2020 Fahmy, Yara/0000-0002-9883-5270; Asar, Hadeel/0009-0005-9792-5142; Fouad, Yousef/0000-0002-2954-2917
National Cancer Institute [P30 CA008748]; Olayan Group
National Cancer Institute(United States Department of Health & Human ServicesNational Institutes of Health (NIH) - USANIH

National Cancer Institute (NCI)); Olayan Group This work was supported by The Olayan Group and National Cancer Institute (P30 CA008748). 38 11 11 0 9 SPRINGER NEW YORK
ONE NEW YORK PLAZA, SUITE 4600, NEW YORK, NY, UNITED STATES
0941-4355 1433-7339 SUPPORT CARE CANCER Support. Care Cancer DEC 2020 28 12 5787 5794
10.1007/s00520-020-05426-9
http://dx.doi.org/10.1007/s00520-020-05426-9 MAR 2020
8 Oncology; Health Care Sciences & Services; Rehabilitation Science Citation Index Expanded (SCI-EXPANDED); Social Science Citation Index (SSCI) Oncology; Health Care Sciences & Services; Rehabilitation PI3HG 32221669 Green Accepted 2025-06-24 WOS:000521856700002
J Hirko, KA; Willett, WC; Hankinson, SE; Rosner, BA; Beck, AH; Tamimi, RM; Eliassen, AH Hirko, Kelly A.; Willett, Walter C.; Hankinson, Susan E.; Rosner, Bernard A.; Beck, Andrew H.; Tamimi, Rulla M.; Eliassen, A. Heather
Healthy dietary patterns and risk of breast cancer by molecular subtype BREAST CANCER RESEARCH AND TREATMENT
English Article
Breast cancer; Diet; Patterns; Molecular subtypes GENE-EXPRESSION PATTERNS; MEDITERRANEAN DIET; POOLED ANALYSIS; ESTROGEN-RECEPTOR; VEGETABLE INTAKE; EATING INDEX; QUALITY; ADHERENCE; COHORT; REPRODUCIBILITY We examined associations between dietary quality indices and breast cancer risk by molecular subtype among 100,643 women in the prospective Nurses' Health Study (NHS) cohort, followed from 1984 to 2006. Dietary quality scores for the Alternative Healthy Eating Index (AHEI), alternate Mediterranean diet (aMED), and Dietary Approaches to Stop Hypertension (DASH) dietary patterns were calculated from semi-quantitative food frequency questionnaires collected every 2-4 years. Breast cancer molecular subtypes were defined according to estrogen receptor (ER), progesterone receptor, human epidermal growth factor 2 (HER2), cytokeratin 5/6 (CK5/6), and epidermal growth factor receptor status from immunostained tumor microarrays in combination with histologic grade. Cox proportional hazards models, adjusted for age and breast cancer risk factors, were used to estimate hazard ratios (HRs) and 95 % confidence intervals (CIs). Competing risk analyses were used to assess heterogeneity by subtype. We did not observe any significant associations between the AHEI or aMED dietary patterns and risk of breast cancer by molecular subtype. However, a significantly reduced risk of HER2-type breast cancer was observed among women in 5th versus 1st quintile of the DASH dietary pattern [n = 134 cases, Q5 vs. Q1 HR (95 % CI) = 0.44 (0.25-0.77)], and the inverse trend across quintiles was significant (p trend = 0.02). We did not observe any heterogeneity in associations between AHEI (p (het) = 0.25), aMED (p (het) = 0.71), and DASH (p (het) = 0.12) dietary patterns and breast cancer by subtype. Adherence to the AHEI, aMED, and DASH dietary patterns was not strongly associated with breast cancer molecular subtypes. [Hirko, Kelly A.] Michigan State Univ, Dept Epidemiol & Biostat, 909 Fee Rd, E Lansing, MI 48824 USA; [Willett, Walter C.; Hankinson, Susan E.; Rosner, Bernard A.; Tamimi, Rulla M.; Eliassen, A. Heather] Brigham & Womens Hosp, Dept Med, Channing Div Network Med, 909 Fee Rd, Boston, MA 02115 USA; [Willett, Walter C.; Hankinson, Susan E.; Rosner, Bernard A.; Beck, Andrew H.; Tamimi, Rulla M.; Eliassen, A. Heather] Harvard Univ, Sch Med, 909 Fee Rd, Boston, MA 02115 USA; [Willett, Walter

C.; Hankinson, Susan E.; Tamimi, Rulla M.; Eliassen, A. Heather] Harvard TH Chan Sch Publ Hlth, Dept Epidemiol, 909 Fee Rd, Boston, MA USA; [Willett, Walter C.] Harvard TH Chan Sch Publ Hlth, Dept Nutr, Boston, MA USA; [Hankinson, Susan E.] Univ Massachusetts, Dept Biostat & Epidemiol, Amherst, MA 01003 USA; [Beck, Andrew H.] Beth Israel Deaconess Med Ctr, Dept Pathol, Boston, MA 02215 USA
Michigan State University; Harvard University; Harvard University Medical Affiliates; Brigham & Women's Hospital; Harvard University; Harvard Medical School; Harvard University; Harvard T.H. Chan School of Public Health; Harvard University; Harvard T.H. Chan School of Public Health; University of Massachusetts System; University of Massachusetts Amherst; Harvard University; Harvard University Medical Affiliates; Beth Israel Deaconess Medical Center Hirko, KA (corresponding author), Michigan State Univ, Dept Epidemiol & Biostat, 909 Fee Rd, E Lansing, MI 48824 USA. khirko@epi.msu.edu Hirko, Kelly/AFN-8256-2022; Willett, Walter/E-2352-2013 Eliassen, A Heather/0000-0002-3961-6609; hirko, kelly/0000-0002-0050-655X NIH [UM1 CA186107, P01 CA087969]; [R25 CA098566]; [T32 CA009001] NIH(United States Department of Health & Human ServicesNational Institutes of Health (NIH) - USA); ; This research was supported by the NIH UM1 CA186107 (Meir Stampfer) and P01 CA087969 (Meir Stampfer). KA Hirko is supported by the R25 CA098566 and the T32 CA009001 training grants. We would like to thank the participants and staff of the Nurses' Health Study for their valuable contributions as well as the following state cancer registries for their help: AL, AZ, AR, CA, CO, CT, DE, FL, GA, ID, IL, IN, IA, KY, LA, ME, MD, MA, MI, NE, NH, NJ, NY, NC, ND, OH, OK, OR, PA, RI, SC, TN, TX, VA, WA, and WY. The authors assume full responsibility for analyses and interpretation of these data. 68 45 49 1 13 SPRINGER NEW YORK 233 SPRING ST, NEW YORK, NY 10013 USA 0167-6806 1573-7217 BREAST CANCER RES TR Breast Cancer Res. Treat. FEB 2016 155 3 579 588 10.1007/s10549-016-3706-2 http://dx.doi.org/10.1007/s10549-016-3706-2 10 Oncology Science Citation Index Expanded (SCI-EXPANDED) Oncology DF0UR 26872903 Green Accepted 2025-06-24 WOS:000371055100019
J Hamdi, Y; Boujemaa, M; Ben Rekaya, M; Ben Hamda, C; Mighri, N; El Benna, H; Mejri, N; Labidi, S; Daoud, N; Naouali, C; Messaoud, O; Chargui, M; Ghedira, K; Boubaker, MS; Mrad, R; Boussen, H; Abdelhak, S Hamdi, Yosr; Boujemaa, Maroua; Ben Rekaya, Mariem; Ben Hamda, Cherif; Mighri, Najah; El Benna, Houda; Mejri, Nesrine; Labidi, Soumaya; Daoud, Nouha; Naouali, Chokri; Messaoud, Olfa; Chargui, Mariem; Ghedira, Kais; Boubaker, Mohamed Samir; Mrad, Ridha; Boussen, Hamouda; Abdelhak, Sonia PEC Consortium Family specific genetic predisposition to breast cancer: results from Tunisian whole exome sequenced breast cancer cases JOURNAL OF TRANSLATIONAL MEDICINE English Article
Breast cancer; Exome sequencing; Family specific predisposition; Non BRCA Tunisian families SUSCEPTIBILITY GENE; CYSTIC-FIBROSIS; RARE MUTATIONS; RISK; IDENTIFICATION; DNA; POLYMORPHISMS; ASSOCIATION; POPULATION; REVEALS Background: A family history of breast cancer has long been thought to indicate the presence of inherited genetic events that predispose to this disease. In North Africa, many specific epidemio-genetic characteristics have been observed in breast cancer families when

compared to Western populations. Despite these specificities, the majority of breast cancer genetics studies performed in North Africa remain restricted to the investigation of the BRCA1 and BRCA2 genes. Thus, comprehensive data at a whole exome or whole genome level from local patients are lacking. Methods: A whole exome sequencing (WES) of seven breast cancer Tunisian families have been performed using a family-based approach. We focused our analysis on BC-TN-F001 family that included two affected members that have been sequenced using WES. Relevant variants identified in BC-TN-F001 have been confirmed using Sanger sequencing. Then, we conducted an integrative analysis by combining our results with those from other WES studies in order to figure out the genetic transmission model of the newly identified genes. Biological network construction and protein-protein interactions analyses have been performed to decipher the molecular mechanisms likely accounting for the role of these genes in breast cancer risk. Results: Sequencing, filtering strategies, and validation analysis have been achieved. For BC-TN-F001, no deleterious mutations have been identified on known breast cancer genes. However, 373 heterozygous, exonic and rare variants have been identified on other candidate genes. After applying several filters, 12 relevant high-risk variants have been selected. Our results showed that these variants seem to be inherited in a family specific model. This hypothesis has been confirmed following a thorough analysis of the reported WES studies. Enriched biological process and proteinprotein interaction networks resulted in the identification of four novel breast cancer candidate genes namely MMS19, DNAH3, POLK and KATB6. Conclusions: In this first WES application on Tunisian breast cancer patients, we highlighted the impact of next generation sequencing technologies in the identification of novel breast cancer candidate genes which may bring new insights into the biological mechanisms of breast carcinogenesis. Our findings showed that the breast cancer predisposition in non-BRCA families may be ethnic and/or family specific. [Hamdi, Yosr; Boujemaa, Maroua; Ben Rekaya, Mariem; Mighri, Najah; Naouali, Chokri; Messaoud, Olfa; Chargui, Mariem; Boubaker, Mohamed Samir; Abdelhak, Sonia] Univ Tunis El Manar, Lab Biomed Genom & Oncogenet, Inst Pasteur Tunis, LR16IPT05,13 Pl Pasteur BP 74, Tunis 1002, Tunisia; [Ben Hamda, Cherif; Ghedira, Kais] Univ Tunis El Manar, Lab Bioinformat Biomath & Biostat, Inst Pasteur Tunis, LR16IPT09, Tunis, Tunisia; [Ben Hamda, Cherif] Carthage Univ, Fac Sci Bizerte, Tunis, Tunisia; [El Benna, Houda; Mejri, Nesrine; Labidi, Soumaya; Daoud, Nouha; Boussen, Hamouda; PEC Consortium] Abderrahmane Mami Hosp, Dept Med Oncol, Ariana, Tunisia; [Mrad, Ridha] Charles Nicolle Hosp, Dept Human Genet, Tunis, Tunisia

Pasteur Network; Universite de Tunis-El-Manar; Institut Pasteur Tunis; Pasteur Network; Universite de Tunis-El-Manar; Institut Pasteur Tunis; Universite de Carthage; Universite de Tunis-El-Manar; Hopital Abderrahmene Mami; Universite de Tunis-El-Manar; Hopital Charles Nicolle Hamdi, Y (corresponding author), Univ Tunis El Manar, Lab Biomed Genom & Oncogenet, Inst Pasteur Tunis, LR16IPT05,13 Pl Pasteur BP 74, Tunis 1002, Tunisia.

yosr.hamdi.82@gmail.com M'rad, Ridha/GLU-4806-2022; Messaoud, Olfa/I-8967-2019; Boussen, Hamouda/AAI-2796-2020; Boujemaa, Maroua/JVY-7909-2024; Hamdi, Yosr/GLT-7117-2022; Abdelhak, Sonia/L-2831-2013 Ben rekaya, Mariem/0000-0001-7176-5201; Hamdi, Yosr/0000-0002-2815-1834; Boujemaa, Maroua/0000-0001-7409-9240; Ben Hamda, Cherif/0000-0003-0985-4881; Abdelhak,

Sonia/0000-0001-8466-5525; MESSAOUD, Olfa/0000-0003-4547-0180
Tunisian Ministry of Public Health [PEC4-TUN]; Tunisian Ministry of Higher Education and Scientific Research [LR11IPT05, LR16IPT05]; E.C. [295097] Tunisian Ministry of Public Health(Ministry of Public Health in Tunisia); Tunisian Ministry of Higher Education and Scientific Research(Ministry of Higher Education & Scientific Research of Tunisia); E.C.(European Union (EU)European Commission Joint Research Centre) This work was supported by the Tunisian Ministry of Public Health (PEC4-TUN), the Tunisian Ministry of Higher Education and Scientific Research (LR11IPT05 and LR16IPT05) and by the E.C. Grant Agreement No 295097 for FP7 project GM-NCD-Inco. 55 28 30 1
10 BMC LONDON CAMPUS, 4 CRINAN ST, LONDON N1 9XW, ENGLAND 1479-5876 J TRANSL MED J. Transl. Med.
JUN 7 2018 16 158
10.1186/s12967-018-1504-9
http://dx.doi.org/10.1186/s12967-018-1504-9 13
Medicine, Research & Experimental Science Citation Index Expanded (SCI-EXPANDED) Research & Experimental Medicine
GJ0YD 29879995 Green Published, gold 2025-06-24 WOS:000434979600004
J Nosrati, A; Naghshvar, F; Torabizadeh, Z; Haghshenas, M; Sangsefidi, H Nosrati, Anahita; Naghshvar, Farshad; Torabizadeh, Zhila; Haghshenas, Mohammadreza; Sangsefidi, Hadi Relationship between Human Papilloma Virus and Colorectal Cancer in Northern Iran MIDDLE EAST JOURNAL OF CANCER English Article
Colorectal cancer; Human papilloma virus; PCR
COLON; INFECTION; ASSOCIATION; DNA; CARCINOMAS; FREQUENCY
Background: Colorectal cancer is one of the most common malignancies worldwide with more than one million new cases. According to the Ministry of Health and Medical Education of Iran, colorectal cancer is the third most common cancer in Iran. Many risk factors are known causes of this disease. However, the molecular mechanisms associated with colorectal cancer are still under investigation. Recent studies have shown that some viruses, particularly human papilloma virus, may be associated with the pathology of colorectal cancer. Methods: This case-control study examined 95 colorectal cancer and 95 normal colon tissue paraffin blocks (control) to identify the relationship between human papilloma virus and colorectal cancer by polymerase chain reaction. Results: Clinicopathological data that included sex, age, tumor grade, stage and location were recorded. All tumor and control groups (totally: 190 samples) were negative in terms of the human papilloma virus genome. No relationship between clinicopathological data and human papilloma virus genome was identified. Conclusions: Regardless of other risk factors for colorectal cancer, a number of studies in different parts of the world have shown that human papilloma virus may be an important factor in the increasing incidence of colorectal cancer. However, we have found no association between human papilloma virus and colorectal cancer in this study. [Nosrati, Anahita; Naghshvar, Farshad; Torabizadeh, Zhila; Sangsefidi, Hadi] Mazandaran Univ Med Sci, Imam Khomeini Hosp, Dept Pathol, Sari, Iran; [Haghshenas, Mohammadreza] Mazandaran Univ Med Sci, Dept Virol, Fac Med, Sari, Iran Mazandaran University of Medical Sciences; Tehran University of Medical Sciences; Mazandaran University of Medical Sciences
Torabizadeh, Z (corresponding author), Mazandaran Univ Med

Sci, Imam Khomeini Hosp, Dept Pathol, Sari, Iran.
zhtorabi@yahoo.com Naghshvar, Farshad/R-7280-2018
36 5 6 0 0 SHIRAZ UNIV MEDICAL SCIENCES SHIRAZ NEMAZEE HOSPITAL, SHIRAZ, 71934, IRAN 2008-6709 2008-6687 MIDDLE EAST J CANCER Middle East J. Cancer OCT 2015 6 4 237 241 5 Oncology Emerging Sources Citation Index (ESCI) Oncology DR0LS
2025-06-24 WOS:000379599600005
J Dey, S; Soliman, AS; Hablas, A; Seifeldein, IA; Ismail, K; Ramadan, M; El-Hamzawy, H; Wilson, ML; Banerjee, M; Boffetta, P; Harford, J; Merajver, SD Dey, Subhojit; Soliman, Amr S.; Hablas, Ahmad; Seifeldein, Ibrahim A.; Ismail, Kadry; Ramadan, Mohamed; El-Hamzawy, Hesham; Wilson, Mark L.; Banerjee, Mousumi; Boffetta, Paolo; Harford, Joe; Merajver, Sofia D. Urban-rural differences in breast cancer incidence in Egypt (1999-2006) BREAST English
Article Breast cancer; Incidence; Urban-rural; xenoestrogens; Egypt PERSISTENT ORGANIC POLLUTANTS; PASSIVE AIR SAMPLERS; BISPHENOL-A; ORGANOCHLORINE PESTICIDES; POLYCHLORINATED-BIPHENYLS; US POPULATION; WASTE-WATER; ENVIRONMENT; EXPOSURE; MORTALITY Objective: To describe urban rural differences in breast cancer incidence in Gharbiah, Egypt and to investigate if these differences could be explained by known risk factors of breast cancer. Methods: We used data from the population-based cancer registry of Gharbiah, Egypt to assess breast cancer incidence from 1999 through 2006. The Egyptian census provided data on district-specific population, age, and urban rural classification. Incidence patterns of breast cancer by district and age-specific urban rural differences were analyzed. Results: Overall, incidence rate of breast cancer was three to four times higher in urban areas than in rural areas (60.9/10(5)-year for urban areas versus 17.8/10(5)-year for rural areas; IRR = 3.73, 95% CI = 3.30, 4.22). Urban areas had consistently higher incidence of breast cancer across all age-groups for all years. Higher incidence of breast cancer was also seen in the more developed districts of Tanta and El-Mehalla. Conclusions: Higher incidence of breast cancer in urban and more developed populations might be related to higher exposure to xenoestrogens, as well as other endocrine disruptors and genotoxic substances. (C) 2010 Elsevier Ltd. All rights reserved. [Dey, Subhojit; Soliman, Amr S.; Wilson, Mark L.] Univ Michigan, Sch Publ Hlth, Dept Epidemiol, Ann Arbor, MI 48109 USA; [Hablas, Ahmad; Ismail, Kadry] Gharbiah Canc Soc, Tanta, Gharbiah, Egypt; [Seifeldein, Ibrahim A.; Ramadan, Mohamed; El-Hamzawy, Hesham] Tanta Canc Ctr, Tanta, Gharbiah, Egypt; [Banerjee, Mousumi] Univ Michigan, Sch Publ Hlth, Dept Biostat, Ann Arbor, MI 48109 USA; [Boffetta, Paolo] Mt Sinai Sch Med, Tisch Canc Inst, New York, NY USA; [Harford, Joe] NCI, Off Int Affairs, Bethesda, MD 20892 USA; [Merajver, Sofia D.] Univ Michigan, Ctr Comprehens Canc, Ann Arbor, MI 48109 USA
University of Michigan System; University of Michigan; Egyptian Knowledge Bank (EKB); Tanta University; University of Michigan System; University of Michigan; Icahn School of Medicine at Mount Sinai; National Institutes of Health (NIH) - USA; NIH National Cancer Institute (NCI); University of Michigan System; University of Michigan Dey, S (corresponding author), Univ Michigan, Sch Publ Hlth, Dept Epidemiol, 109 Observ St, Ann Arbor, MI 48109 USA. subhojit@umich.edu Boffetta, Paolo/AAI-7767-

2021 Harford, Joe Bryan/0000-0002-6681-6315 Middle East Cancer Consortium, National Cancer Institute, Bethesda [R25 CA112383, R03 CA117350,5, R03 CA117355, P30 CA46592]; Burroughs Wellcome Fund; Breast Cancer Research Foundation; Department of Epidemiology, University of Michigan School of Public Health; Rackham Graduate School of the University of Michigan; National Cancer Institute [P30CA046592] Funding Source: NIH RePORTER Middle East Cancer Consortium, National Cancer Institute, Bethesda(United States Department of Health & Human ServicesNational Institutes of Health (NIH) - USANIH National Cancer Institute (NCI)); Burroughs Wellcome Fund(Burroughs Wellcome Fund); Breast Cancer Research Foundation; Department of Epidemiology, University of Michigan School of Public Health(University of Michigan System); Rackham Graduate School of the University of Michigan; National Cancer Institute(United States Department of Health & Human ServicesNational Institutes of Health (NIH) - USANIH National Cancer Institute (NCI)) This work was supported by the Middle East Cancer Consortium, National Cancer Institute, Bethesda [R25 CA112383, R03 CA117350,5 P30 CA46592], the Burroughs Wellcome Fund [SDM], and the Breast Cancer Research Foundation [SDM]. Block Grant of the Department of Epidemiology, University of Michigan School of Public Health; and the Travel Grant of the Rackham Graduate School of the University of Michigan to [SD]. 45
52 53 0 10 CHURCHILL LIVINGSTONE EDINBURGH
JOURNAL PRODUCTION DEPT, ROBERT STEVENSON HOUSE, 1-3 BAXTERS PLACE, LEITH WALK, EDINBURGH EH1 3AF, MIDLOTHIAN, SCOTLAND
0960-9776 1532-3080 BREAST Breast OCT 2010
19 5 417 423
10.1016/j.breast.2010.04.005
http://dx.doi.org/10.1016/j.breast.2010.04.005
7 Oncology; Obstetrics & Gynecology Science Citation Index Expanded (SCI-EXPANDED) Oncology; Obstetrics & Gynecology 673XF 20452771 Green Accepted, hybrid
2025-06-24 WOS:000283696800021
J Chaichian, S; Khateri, S; Moradi, Y; Khosravi Shadmani, F; Mansori, K; Khazaei, Z; Moradpour, F; Varse, F
Chaichian, Shahla; Khateri, Sorour; Moradi, Yousef; Khosravi Shadmani, Fatemeh; Mansori, Kamyar; Khazaei, Zaher; Moradpour, Farhad; Varse, Fatemeh Trends in Cervical Cancer Incidence in Iran from 2003 to 2009 MIDDLE EAST JOURNAL OF CANCER English Article
Cervical cancer; Trend of incidence; Epidemiology
HUMAN-PAPILLOMAVIRUS; ORAL-CONTRACEPTIVES; CYTOLOGY; RISK; NEOPLASIA; HISTORY; BREAST; CELLS Background: Cancer is the second leading cause of death worldwide and the third in Iran. Among cancers, cervical cancer is the third leading cause of death in women. Thus, recognizing the epidemiology and trends of cervical cancer can be effective for planning and policy-making. This study aims to investigate the incidence and trends of cervical cancer in Iran due to the few studies that have addressed this issue and the unclear trend for cervical cancer in Iran. Methods: This study re-analyzed existing data from the cancer data recording system in Iran during years 2003 to 2009. We used available data from the National Cancer Registry and Center for Disease Control of the Ministry of Health, Treatment and Medical Education. Stata software (version 11) was used for data analysis and the significance of the incidence trend diagram was derived with WINPEPI software. Results: Assessment of the National Cancer

Registry statistics from 2003 to 2009 showed an increased trend in cervical cancer from 2003 to 2008 and a decreased trend from 2008 to 2009. During this period, there were 4273 cases of cervical cancer registered. From these, 394 cases were registered in 2003 which peaked at 907 cases in 2009. The registered cancer cases had an approximately 3-fold increase during this period. Most provinces reported an increased trend of incidence in cervical cancer. Conclusion: Based on the results of this study, the incidence of cervical cancer is increasing in Iran, especially in the central regions of the country. Therefore, considering the growing trend of cancer, we recommend early detection through screening programs, public awareness, and public training programs that particularly target high risk populations. [Chaichian, Shahla] Islamic Azad Univ, Tehran Med Sci Branch, Minimally Invas Techn Res Ctr Women, Tehran, Iran; [Khateri, Sorour] Kurdistan Univ Med Sci, Student Res Comm, Sanandaj, Iran; [Moradi, Yousef] Iran Univ Med Sci, Pars Hosp, Pars Adv & Minimally Invas Med Manners Res Ctr, Tehran, Iran; [Khosravi Shadmani, Fatemeh] Shahid Beheshti Univ Med Sci, Sch Publ Hlth, Dept Epidemiol, Tehran, Iran; [Mansori, Kamyar] Gonabad Univ Med Sci, Social Dev & Hlth Promot Res Ctr, Gonabad, Iran; [Mansori, Kamyar] Islamic Azad Univ, Sch Nursing & Midwifery, Sannandaj Branch, Sanandaj, Iran; [Khazaei, Zaher] Dezful Univ Med Sci, Dezful, Iran; [Moradpour, Farhad; Varse, Fatemeh] Iran Univ Med Sci, Sch Publ Hlth, Dept Epidemiol, Tehran, Iran Islamic Azad University; Kurdistan University of Medical Sciences; Iran University of Medical Sciences; Shahid Beheshti University Medical Sciences; Islamic Azad University; Iran University of Medical Sciences Moradi, Y (corresponding author), Iran Univ Med Sci, Pars Hosp, Pars Adv & Minimally Invas Med Manners Res Ctr, Tehran, Iran.
yousefmoradi211@yahoo.com Shadmani, Fatemeh/Y-9533-2019; Moradi, Yousef/LPQ-4665-2024; Moradpour, Farhad/HGA-9768-2022; Chaichian, Shahla/S-9196-2017; Moradi, Yousef/AAV-5696-2021
Chaichian, Shahla/0000-0001-5772-8711; Moradpour, Farhad/0000-0002-4449-1978; Moradi, Yousef/0000-0002-2936-5930
24 17 18 0 0 SHIRAZ UNIV MEDICAL SCIENCES SHIRAZ NEMAZEE HOSPITAL, SHIRAZ, 71934, IRAN 2008-6709 2008-6687 MIDDLE EAST J CANCER Middle East J. Cancer JAN 2018 9 1 57 63 7 Oncology Emerging Sources Citation Index (ESCI) Oncology GB0PY
2025-06-24 WOS:000428750200009
J Fung, TT; Hu, FB; Wu, KN; Chiuve, SE; Fuchs, CS; Giovannucci, E Fung, Teresa T.; Hu, Frank B.; Wu, Kana; Chiuve, Stephanie E.; Fuchs, Charles S.; Giovannucci, Edward The Mediterranean and Dietary Approaches to Stop Hypertension (DASH) diets and colorectal cancer AMERICAN JOURNAL OF CLINICAL NUTRITION English Article
NIH-AARP DIET; CORONARY-HEART-DISEASE; IGF-BINDING-PROTEINS; GROWTH-FACTOR-I; C-PEPTIDE; RISK; INSULIN; QUESTIONNAIRE; METAANALYSIS; ADHERENCE
Background Although the Mediterranean diet has been studied for cancer mortality and the Dietary Approaches to Stop Hypertension (DASH) diet shares similarities with the Mediterranean diet few studies have specifically examined these 2 diets and incident colorectal cancer Objective The objective was to prospectively assess the association between the Alternate Mediterranean Diet (aMed) and the DASH style diet scores and risk

of colorectal cancer in middle aged men and women Design A total of 87 256 women and 45 490 men (age 30-55 y for women and 40-75 y for men at baseline) without a history of cancer were followed for <= 26 y The aMed and DASH scores were calculated for each participant by using dietary information that was assessed <= 7 times during follow up Relative risks (RRs) for colorectal cancer were computed with adjustment for potential confounders Results We documented 1432 cases of incident colorectal cancer among women and 1032 cases in men Comparing top with bottom quintiles of the DASH score the pooled RR for total colorectal cancer was 080 (95% CI 0 70 0 91 P for trend = 0 0001) The corresponding RR for DASH score and colon cancer was 081 (95% CI 0 69 0 95 P for trend = 0 002) There was a suggestion of an inverse association with rectal cancer with a pooled RR-of 0 73 (95% CI 0 55 0 98 P for trend = 0 31) when comparing top with bottom quintiles of DASH score No association was observed with aMed score Conclusion Adherence to the DASH diet (which involves higher intakes of whole grains fruit and vegetables moderate amounts of low fat dairy and lower amounts of red or processed meats desserts and sweetened beverages) was associated with a lower risk of colorectal cancer Am J Clin Nutr 2010 92 1429-35 [Fung, Teresa T.] Simmons Coll, Dept Nutr, Boston, MA 02115 USA; [Fung, Teresa T.; Hu, Frank B.; Wu, Kana; Chiuve, Stephanie E.; Giovannucci, Edward] Harvard Univ, Sch Publ Hlth, Dept Nutr, Boston, MA 02115 USA; [Hu, Frank B.; Giovannucci, Edward] Harvard Univ, Sch Publ Hlth, Dept Epidemiol, Boston, MA 02115 USA; [Fuchs, Charles S.] Dana Farber Canc Inst, Boston, MA 02115 USA; [Hu, Frank B.; Giovannucci, Edward] Brigham & Womens Hosp, Dept Med, Channing Lab, Boston, MA USA; [Chiuve, Stephanie E.] Brigham & Womens Hosp, Dept Med, Div Prevent Med, Boston, MA USA; Harvard Univ, Sch Med, Boston, MA USA Simmons University; Harvard University; Harvard T.H. Chan School of Public Health; Harvard University; Harvard T.H. Chan School of Public Health; Harvard University; Harvard University Medical Affiliates; Dana-Farber Cancer Institute; Harvard University; Harvard University Medical Affiliates; Brigham & Women's Hospital; Harvard University; Harvard University Medical Affiliates; Brigham & Women's Hospital; Harvard University; Harvard Medical School
Fung, TT (corresponding author), Simmons Coll, Dept Nutr, 300 Fenway, Boston, MA 02115 USA. Hu, Frank/C-1919-2013; Giovannucci, Edward/ADE-8028-2022 Chiuve, Stephanie/0000-0002-3524-8917 National Institutes of Health [CA98859, CA87969, HL60712] National Institutes of Health(United States Department of Health & Human ServicesNational Institutes of Health (NIH) - USA) Supported by the National Institutes of Health (grant nos CA98859 CA87969 and HL60712) 29 181 204 0 32
OXFORD UNIV PRESS OXFORD GREAT CLARENDON ST, OXFORD OX2 6DP, ENGLAND 0002-9165 1938-3207 AM J CLIN NUTR Am. J. Clin. Nutr. DEC 2010 92 6 1429
1435 10.3945/ajcn.2010.29242
http://dx.doi.org/10.3945/ajcn.2010.29242 7
Nutrition & Dietetics Science Citation Index Expanded (SCI-EXPANDED) Nutrition & Dietetics 690HM 21097651 Green Published, Bronze 2025-06-24 WOS:000284993600021
J Figlioli, G; Billaud, A; Wang, Q; Bolla, MK; Dennis, J; Lush, M; Kvist, A; Adank, MA; Ahearn, TU; Antonenkova, NN; Auvinen, P; Behrens, S; Bermisheva, M; Bogdanova, N; Bojesen, SE; Bonanni, B; Brüning, T; Camp, NJ; Campbell, A; Castelao, JE; Cessna, MH; Czene, K; Devilee, P; Dörk, T; Eriksson, M; Fasching,

PA; Flyger, H; Gabrielson, M; Gago-Dominguez, M; García-Closas, M; Glendon, G; Garcia, EG; González-Neira, A; Grassmann, F; Guénel, P; Hahnen, E; Hamann, U; Hillemanns, P; Hooning, MJ; Hoppe, R; Howell, A; Humphreys, K; Jakubowska, A; Khusnutdinova, EK; Kristensen, VN; Lindblom, A; Loizidou, MA; Lubinski, J; Mannermaa, A; Maurer, T; Mavroudis, D; Newman, WG; Obi, N; Panayiotidis, M; Radice, P; Rashid, MU; Rhenius, V; Ruebner, M; Saloustros, E; Sawyer, EJ; Schmidt, MK; Schmutzler, RK; Shah, MT; Southey, MC; Tomlinson, I; Truong, T; van Veen, EM; Wendt, C; Yang, XHR; Michailidou, K; Dunning, AM; Pharoah, PDP; Easton, DF; Andrulis, IL; Evans, DG; Hollestelle, A; Chang-Claude, J; Milne, RL; Peterlongo, P Figlioli, Gisella; Billaud, Amandine; Wang, Qin; Bolla, Manjeet K.; Dennis, Joe; Lush, Michael; Kvist, Anders; Adank, Muriel A.; Ahearn, Thomas U.; Antonenkova, Natalia N.; Auvinen, Paeivi; Behrens, Sabine; Bermisheva, Marina; Bogdanova, Natalia, V; Bojesen, Stig E.; Bonanni, Bernardo; Bruening, Thomas; Camp, Nicola J.; Campbell, Archie; Castelao, Jose E.; Cessna, Melissa H.; Czene, Kamila; Devilee, Peter; Doerk, Thilo; Eriksson, Mikael; Fasching, Peter A.; Flyger, Henrik; Gabrielson, Marike; Gago-Dominguez, Manuela; Garcia-Closas, Montserrat; Glendon, Gord; Garcia, Encarna Gomez; Gonzalez-Neira, Anna; Grassmann, Felix; Guenel, Pascal; Hahnen, Eric; Hamann, Ute; Hillemanns, Peter; Hooning, Maartje J.; Hoppe, Reiner; Howell, Anthony; Humphreys, Keith; Jakubowska, Anna; Khusnutdinova, Elza K.; Kristensen, Vessela N.; Lindblom, Annika; Loizidou, Maria A.; Lubinski, Jan; Mannermaa, Arto; Maurer, Tabea; Mavroudis, Dimitrios; Newman, William G.; Obi, Nadia; Panayiotidis, Mihalis, I; Radice, Paolo; Rashid, Muhammad U.; Rhenius, Valerie; Ruebner, Matthias; Saloustros, Emmanouil; Sawyer, Elinor J.; Schmidt, Marjanka K.; Schmutzler, Rita K.; Shah, Mitul T.; Southey, Melissa C.; Tomlinson, Ian; Truong, Therese; van Veen, Elke M.; Wendt, Camilla; Yang, Xiaohong R.; Michailidou, Kyriaki; Dunning, Alison M.; Pharoah, Paul D. P.; Easton, Douglas F.; Andrulis, Irene L.; Evans, D. Gareth; Hollestelle, Antoinette; Chang-Claude, Jenny; Milne, Roger L.; Peterlongo, Paolo NBCS Collaborators; kConFab Investigators Spectrum and Frequency of Germline FANCM Protein-Truncating Variants in 44,803 European Female Breast Cancer Cases CANCERS English Article breast cancer predisposition; breast cancer risk factors; FANCM PTVs spectrum; protein truncating variants; PTVs RISK; GENE; C.5791C-GREATER-THAN-T; SUSCEPTIBILITY Simple Summary Mutations in the FANCM gene may cause a particular type of breast cancer known as ER-negative. In this study, we describe the geographic distribution of 66 different FANCM mutations identified in 44,803 female breast cancer cases from Europe, USA, Canada and Australia. We found that the FANCM:p.Gln1701* mutation is most common in Northern Europe and has lower frequencies in Southern European countries. In contrast, the FANCM:p.Gly1906Alafs*12 mutation is most common in Southern Europe and rarer in Central and Northern Europe. We found that the FANCM:p.Arg658* mutation is most prevalent in Central Europe and that the FANCM:p.Gln498Thrfs*7 mutation originates from Lithuania. Finally, we showed that many and varied FANCM mutations are present in Southwestern and Central Europeans while a much more limited range of mutations is present in Northeastern Europeans. The knowledge of this geographic distribution of FANCM mutations is important to establish more efficient genetic testing strategies in specific

populations. FANCM germline protein truncating variants (PTVs) are moderate-risk factors for ER-negative breast cancer. We previously described the spectrum of FANCM PTVs in 114 European breast cancer cases. In the present, larger cohort, we report the spectrum and frequency of four common and 62 rare FANCM PTVs found in 274 carriers detected among 44,803 breast cancer cases. We confirmed that p.Gln1701* was the most common PTV in Northern Europe with lower frequencies in Southern Europe. In contrast, p.Gly1906Alafs*12 was the most common PTV in Southern Europe with decreasing frequencies in Central and Northern Europe. We verified that p.Arg658* was prevalent in Central Europe and had highest frequencies in Eastern Europe. We also confirmed that the fourth most common PTV, p.Gln498Thrfs*7, might be a founder variant from Lithuania. Based on the frequency distribution of the carriers of rare PTVs, we showed that the FANCM PTVs spectra in Southwestern and Central Europe were much more heterogeneous than those from Northeastern Europe. These findings will inform the development of more efficient FANCM genetic testing strategies for breast cancer cases from specific European populations.
[Figlioli, Gisella; Billaud, Amandine; Peterlongo, Paolo] IFOM ETS AIRC Inst Mol Oncol, Genome Diagnost Program, I-20139 Milan, Italy; [Wang, Qin; Bolla, Manjeet K.; Dennis, Joe; Lush, Michael; Michailidou, Kyriaki; Pharoah, Paul D. P.; Easton, Douglas F.] Univ Cambridge, Ctr Canc Genet Epidemiol, Dept Publ Hlth & Primary Care, Cambridge CB1 8RN, England; [Kvist, Anders] Lund Univ, Dept Clin Sci Lund, Div Oncol, S-22185 Lund, Sweden; [Adank, Muriel A.] Antoni Leeuwenhoek Hosp, Netherlands Canc Inst, Family Canc Clin, NL-1066 CX Amsterdam, Netherlands; [Ahearn, Thomas U.; Garcia-Closas, Montserrat; Yang, Xiaohong R.] NCI, NIH, Dept Hlth & Human Serv, Div Canc Epidemiol & Genet, Bethesda, MD 20892 USA; [Antonenkova, Natalia N.; Bogdanova, Natalia, V] NN Alexandrov Res Inst Oncol & Med Radiol, Minsk 223040, BELARUS; [Auvinen, Paeivi; Mannermaa, Arto] Univ Eastern Finland, Translat Canc Res Area, Kuopio 70210, Finland; [Auvinen, Paeivi] Univ Eastern Finland, Inst Clin Med Oncol, Kuopio 70210, Finland; [Auvinen, Paeivi] Kuopio Univ Hosp, Canc Ctr, Dept Oncol, Kuopio 70210, Finland; [Behrens, Sabine; Chang-Claude, Jenny] German Canc Res Ctr, Div Canc Epidemiol, D-69120 Heidelberg, Germany; [Bermisheva, Marina] Russian Acad Sci, Inst Biochem & Genet, Ufa Fed Res Ctr, Ufa 450054, Russia; [Bogdanova, Natalia, V] Hannover Med Sch, Dept Radiat Oncol, D-30625 Hannover, Germany; [Bogdanova, Natalia, V; Doerk, Thilo; Hillemanns, Peter] Hannover Med Sch, Gynaecol Res Unit, D-30625 Hannover, Germany; [Bojesen, Stig E.] Copenhagen Univ Hosp, Herlev & Gentofte Hosp, Copenhagen Gen Populat Study, DK-2730 Herlev, Denmark; [Bojesen, Stig E.] Copenhagen Univ Hosp, Herlev & Gentofte Hosp, Dept Clin Biochem, DK-2730 Herlev, Denmark; [Bojesen, Stig E.] Univ Copenhagen, Fac Hlth & Med Sci, DK-2200 Copenhagen, Denmark; [Bonanni, Bernardo] European Inst Oncol IRCCS, Div Canc Prevent & Genet, IEO, I-20141 Milan, Italy; [Bruening, Thomas] Inst Ruhr Univ Bochum IPA, Inst Prevent & Occupat Med German Social Accid In, D-44789 Bochum, Germany; [Camp, Nicola J.] Univ Utah, Dept Internal Med, Salt Lake City, UT 84112 USA; [Camp, Nicola J.] Univ Utah, Huntsman Canc Inst, Salt Lake City, UT 84112 USA; [Campbell, Archie] Univ Edinburgh, Inst Genet & Canc, Ctr Genom & Expt Med, Edinburgh EH4 2XU, Scotland; [Campbell, Archie] Univ Edinburgh, Usher Inst Populat Hlth Sci & Informat, Edinburgh EH16 4UX, Scotland; [Castelao, Jose E.] Xerencia Xest Integrada Vigo SERGAS, Oncol & Genet Unit, Inst

Invest Sanitaria Galicia IISGS, Vigo 36312, Spain; [Cessna, Melissa H.] Intermt Hlth, Salt Lake City, UT 84111 USA; Univ Oslo, Inst Clin Med, Fac Med, N-0450 Oslo, Norway; [NBCS Collaborators] Cyprus Inst Neurol & Genet, Biostat Unit, CY-2371 Nicosia, Cyprus; [Czene, Kamila; Eriksson, Mikael; Gabrielson, Marike; Grassmann, Felix; Humphreys, Keith] Karolinska Inst, Dept Med Epidemiol & Biostat, S-17176 Stockholm, Sweden; [Devilee, Peter] Leiden Univ, Med Ctr, Dept Pathol, NL-2333 ZA Leiden, Netherlands; [Devilee, Peter] Leiden Univ, Dept Human Genet, Med Ctr, NL-2333 ZA Leiden, Netherlands; [Fasching, Peter A.] Univ Hosp Erlangen, Friedrich Alexander Univ Erlangen Nuremberg, Comprehens Canc Ctr Erlangen EMN, Dept Gynecol & Obstet, D-91054 Erlangen, Germany; [Flyger, Henrik] Copenhagen Univ Hosp, Herlev & Gentofte Hosp, Dept Breast Surg, DK-2730 Herlev, Denmark; [Gago-Dominguez, Manuela] Complejo Hosp Univ Santiago, Inst Invest Sanitaria Santiago Compostela FIDIS F, IDIS Canc Genet & Epidemiol Grp, Genom Med Grp,SERGAS, Santiago De Compostela 15706, Spain; [Glendon, Gord] Lunenfeld Tanenbaum Res Inst Mt Sinai Hosp, Fred A Litwin Ctr Canc Genet, Toronto, ON M5G 1X5, Canada; [Glendon, Gord; Andrulis, Irene L.] Univ Hlth Network, Lab Med Program, Toronto, ON M5G 2C4, Canada; [Gonzalez-Neira, Anna] Maastricht Univ, Dept Clin Genet, Med Ctr, NL-6229 HX Maastricht, Netherlands; [Grassmann, Felix] Spanish Natl Canc Res Ctr CNIO, Human Genotyping Unit CeGen, Madrid 28029, Spain; [Grassmann, Felix] Hlth & Med Univ, Inst Clin Res & Syst Med, Dept Med, D-14467 Potsdam, Germany; [Guenel, Pascal] Univ Paris Saclay, UVSQ, CESP U1018, INSERM,Exposome Hered Canc & Hlth Team,Gustave Ro, F-94805 Villejuif, France; [Hahnen, Eric] Univ Cologne, Fac Med, Ctr Familial Breast & Ovarian Canc, D-50937 Cologne, Germany; [Hahnen, Eric; Schmutzler, Rita K.] Univ Hosp Cologne, Univ Cologne, D-50937 Cologne, Germany; [Hahnen, Eric] Univ Cologne, Fac Med, Ctr Integrated Oncol CIO, D-50937 Cologne, Germany; [Hamann, Ute] German Canc Res Ctr, Mol Genet Breast Canc, D-69120 Heidelberg, Germany; [Hooning, Maartje J.; Hollestelle, Antoinette] Erasmus MC Canc Inst, Dept Med Oncol, NL-3015 GD Rotterdam, Netherlands; [Hoppe, Reiner] Dr Margarete Fischer Bosch Inst Clin Pharmacol, D-70376 Stuttgart, Germany; [Hoppe, Reiner] Univ Tubingen, D-72074 Tubingen, Germany; [Howell, Anthony] Univ Manchester, Div Canc Sci, Manchester M13 9PL, England; [Jakubowska, Anna] Pomeranian Med Univ, Int Hereditary Canc Ctr, Dept Genet & Pathol, PL-71252 Szczecin, Poland; [Jakubowska, Anna] Pomeranian Med Univ, Independent Lab Mol Biol & Genet Diagnost, PL-71252 Szczecin, Poland; [Khusnutdinova, Elza K.] Ufa Univ Sci & Technol, Dept Genet & Fundamental Med, Ufa 450076, Russia; [Kristensen, Vessela N.] Oslo Univ Hosp, Dept Med Genet, N-0379 Oslo, Norway; [Kristensen, Vessela N.] Univ Oslo, N-0379 Oslo, Norway; [Lindblom, Annika] Karolinska Inst, Dept Mol Med & Surg, S-17176 Stockholm, Sweden; [Lindblom, Annika] Karolinska Univ Hosp, Dept Clin Genet, S-17176 Stockholm, Sweden; [Loizidou, Maria A.; Panayiotidis, Mihalis, I] Cyprus Inst Neurol & Genet, Dept Canc Genet Therapeut & Ultrastruct Pathol, CY-2371 Nicosia, Cyprus; [Mannermaa, Arto] Univ Eastern Finland, Inst Clin Med Pathol & Forens Med, Kuopio 70210, Finland; [Mannermaa, Arto] Kuopio Univ Hosp, Biobank Eastern Finland, Kuopio 70210, Finland; [Maurer, Tabea; Chang-Claude, Jenny] Univ Med Ctr Hamburg Eppendorf, Univ Canc Ctr Hamburg UCCH, Canc Epidemiol Grp, D-20246 Hamburg, Germany; [Mavroudis, Dimitrios] Univ Hosp Heraklion, Dept Med Oncol, Iraklion 71110, Greece; [Newman, William G.; van Veen, Elke M.] Univ Manchester, Fac Biol Med & Hlth, Sch Biol Sci, Div

Evolut Infect & Genom, Manchester M13 9PL, England; [Newman, William G.; van Veen, Elke M.] Manchester Univ NHS Fdn Trust, St Marys Hosp, Manchester Ctr Genom Med, Manchester Acad Hlth Sci Ctr, Manchester M13 9WL, England; [Obi, Nadia] Univ Med Ctr Hamburg Eppendorf, Inst Med Biometry & Epidemiol, D-20246 Hamburg, Germany; [Radice, Paolo] Fdn IRCCS Ist Nazl Tumori INT, Dept Expt Oncol, Unit Predict Med Mol Bases Genet Risk, I-20133 Milan, Italy; [Rashid, Muhammad U.] Shaukat Khanum Mem Canc Hosp & Res Ctr SKMCH & RC, Dept Basic Sci, Lahore 54000, Pakistan; [Rhenius, Valerie] Univ Cambridge, Ctr Canc Genet Epidemiol, Dept Oncol, Cambridge CB1 8RN, England; [Saloustros, Emmanouil] Univ Hosp Larissa, Dept Oncol, Larisa 41110, Greece; [Sawyer, Elinor J.] Kings Coll London, Comprehens Canc Ctr, Sch Canc & Pharmaceut Sci, Guys Campus, London SE1 9RT, England; [Schmidt, Marjanka K.] Netherlands Canc Inst, Div Mol Pathol, NL-1066 CX Amsterdam, Netherlands; [Schmidt, Marjanka K.] Antoni Leeuwenhoek Hosp, Netherlands Canc Inst, Div Psychosocial Res & Epidemiol, NL-1066 CX Amsterdam, Netherlands; [Schmidt, Marjanka K.] Leiden Univ, Dept Clin Genet, Med Ctr, NL-2333 ZA Leiden, Netherlands; [Schmutzler, Rita K.] Univ Cologne, Fac Med, Ctr Mol Med Cologne CMMC, D-50931 Cologne, Germany; [Schmutzler, Rita K.] Univ Hosp Cologne, Univ Cologne, D-50931 Cologne, Germany; [Southey, Melissa C.] Monash Univ, Sch Clin Sci Monash Hlth, Precis Med, Clayton, Vic 3168, Australia; [Southey, Melissa C.] Univ Melbourne, Dept Clin Pathol, Melbourne, Vic 3000, Australia; [Southey, Melissa C.] Canc Council Victoria, Canc Epidemiol Div, Melbourne, Vic 3004, Australia; [Tomlinson, Ian] Univ Edinburgh, Canc Res Ctr, Edinburgh EH4 2XU, Scotland; [Wendt, Camilla] Soder Sjukhuset, Karolinska Inst, Dept Clin Sci & Educ, S-11883 Stockholm, Sweden; [Pharoah, Paul D. P.] Cedars Sinai Med Ctr, Dept Computat Biomed, West Hollywood, CA 90069 USA; [Andrulis, Irene L.] Univ Toronto, Dept Mol Genet, Toronto, ON M5S 1A8, Canada; [Milne, Roger L.] Univ Melbourne, Ctr Epidemiol & Biostat, Melbourne Sch Populat & Global Hlth, Melbourne, Vic 3010, Australia IFOM - FIRC Institute of Molecular Oncology; University of Cambridge; Lund University; Netherlands Cancer Institute; National Institutes of Health (NIH) - USA; NIH National Cancer Institute (NCI); NIH National Cancer Institute- Division of Cancer Epidemiology & Genetics; University of Eastern Finland; University of Eastern Finland; Kuopio University Hospital; University of Eastern Finland; University of Eastern Finland Hospital; Helmholtz Association; German Cancer Research Center (DKFZ); Russian Academy of Sciences; Institute of Biochemistry & Genetics of Ufa Science Centre of the RAS; Hannover Medical School; Hannover Medical School; University of Copenhagen; Copenhagen University Hospital; University of Copenhagen; Copenhagen University Hospital; University of Copenhagen; IRCCS European Institute of Oncology (IEO); Ruhr University Bochum; Utah System of Higher Education; University of Utah; Utah System of Higher Education; University of Utah; Huntsman Cancer Institute; University of Edinburgh; University of Edinburgh; Intermountain Healthcare; Intermountain Medical Center; University of Oslo; Cyprus Institute of Neurology & Genetics; Karolinska Institutet; Leiden University; Leiden University Medical Center (LUMC); Leiden University - Excl LUMC; Leiden University; Leiden University Medical Center (LUMC); Leiden University - Excl LUMC; University of Erlangen Nuremberg; University of Copenhagen; Copenhagen University Hospital; Complexo Hospitalario Universitario de Santiago de Compostela; Universidade de Santiago de Compostela;

University of Toronto; Sinai Health System Toronto; Lunenfeld Tanenbaum Research Institute; University of Toronto; University Health Network Toronto; Maastricht University; Centro Nacional de Investigaciones Oncologicas (CNIO); Universite Paris Saclay; Institut National de la Sante et de la Recherche Medicale (Inserm); University of Cologne; University of Cologne; University of Cologne; Helmholtz Association; German Cancer Research Center (DKFZ); Erasmus University Rotterdam; Erasmus MC; Erasmus MC Cancer Institute; Eberhard Karls University of Tubingen; Eberhard Karls University Hospital; Eberhard Karls University of Tubingen; University of Manchester; Pomeranian Medical University; Pomeranian Medical University; Ufa University of Science & Technology; University of Oslo; University of Oslo; Karolinska Institutet; Karolinska Institutet; Karolinska University Hospital; Cyprus Institute of Neurology & Genetics; University of Eastern Finland; Kuopio University Hospital; University of Eastern Finland; University of Eastern Finland Hospital; University of Hamburg; University Medical Center Hamburg-Eppendorf; University Hospital of Heraklion; University of Manchester; Manchester University NHS Foundation Trust; University of Manchester; University of Hamburg; University Medical Center Hamburg-Eppendorf; Fondazione IRCCS Istituto Nazionale Tumori Milan; Shaukat Khanum Memorial Cancer Hospital & Research Centre; University of Cambridge; General University Hospital of Larissa; University of London; King's College London; Netherlands Cancer Institute; Netherlands Cancer Institute; Leiden University; Leiden University Medical Center (LUMC); Leiden University - Excl LUMC; University of Cologne; University of Cologne; Monash University; University of Melbourne; Cancer Council Victoria; University of Edinburgh; Karolinska Institutet; Sodersjukhuset Hospital; Cedars Sinai Medical Center; University of Toronto; University of Melbourne Peterlongo, P (corresponding author), IFOM ETS AIRC Inst Mol Oncol, Genome Diagnost Program, I-20139 Milan, Italy.

gisella.figlioli@ifom.eu; paolo.peterlongo@ifom.eu

Paayiotidis, Mihalis/KOC-9449-2024; Romero, Atocha/AAS-2862-2021; Southey, Melissa/CAA-7064-2022; Gabrielson, Marike/KIC-7122-2024; Andrulis, Irene/E-7267-2013; Guenel, Pascal/AAS-7688-2021; Michailidou, Kyriaki/ABD-5122-2020; Hoppe, Reiner/LZI-7325-2025; Flyger, Henrik/AAM-6494-2021; Evans, D/AAB-4308-2022; Gomez, Encarna/E-6022-2018; Pharoah, Paul/V-6658-2019; shah, Mitul/KIC-1534-2024; Bermisheva, Marina/J-6305-2018; Lubinski, Jan/C-4420-2017; Khusnutdinova, Elza/A-4810-2013; Campbell, Archie/AAZ-1757-2021; Gonzalez-Neira, Anna/C-5791-2015; truong, therese/A-2837-2013; Gago-Dominguez, Manuela/AAP-6611-2021; Peterlongo, Paolo/Q-4717-2018; van Veen, Elke/ABA-3250-2021; Billaud, Amandine/JDM-3056-2023; DEVILEE, PETER/ABR-2140-2022; Bogdanova, Natalia V./Y-2205-2019; Schmidt, Marjanka/HPE-6946-2023; Bonanni, Bernardo/AAM-7928-2020; Loizidou, Maria/C-6607-2009; Fasching, Peter/ABH-9912-2020; van Veen, Elke/JHU-3928-2023; qianglong, wang/HIK-3742-2022; Jakubowska, Anna/O-8050-2014; Saloustros, Emmanouil/AAC-8235-2020; Guenel, Pascal/H-3728-2018; Dork, Thilo/J-8620-2012; Garcia-Closas, Montserrat/F-3871-2015   Bojesen, Stig/0000-0002-4061-4133; Newman, William/0000-0002-6382-4678; Bogdanova, Natalia/0000-0002-9736-4593; Guenel, Pascal/0000-0002-8359-518X; Figlioli, Gisella/0000-0002-0740-1363; Grassmann, Felix/0000-0003-1390-7528; Lindblom, Annika/0000-0001-7675-7569; Bermisheva, Marina/0000-0002-0584-3969; Dork, Thilo/0000-0002-9458-0282; Kvist, Anders/0000-0002-1358-0695; Garcia-Closas, Montserrat/0000-

0003-1033-2650; Gomez-Garcia, Encarna/0000-0003-4999-8847; Howell, Anthony/0000-0002-6233-719X; van Veen, Elke/0000-0001-8618-2332
Associazione Italiana Ricerca sul Cancro (AIRC) [IG22860]; Fondazione Umberto Veronesi; European Union's Horizon 2020 Research and Innovation Programme [16634935, 633784]; Government of Canada through Genome Canada; Canadian Institutes of Health Research; Ministere de l'Economie et de l'Innovation du Quebec through Genome Quebec; Quebec Breast Cancer Foundation; National Cancer Institute Intramural Research Program, National Institutes of Health; European Union Horizon 2020 research and innovation program BRIDGES [634935]; Wellcome Trust [v203477/Z/16/Z]; Dutch Cancer Society [NKI 2007-3839, 2009 4363, 207800/Z/17/Z, 70492, Do761/15-1, NWO 91109024]; Dutch Ministry of Health, Welfare and Sport; ELAN-Fond of the University Hospital of Erlangen; NIHR Comprehensive Biomedical Research Centre, Guy's AMP; St. Thomas' NHS Foundation Trust; King's College London, United Kingdom; Oxford Biomedical Research Centre; Accion Estrategica de Salud del Instituto de Salud Carlos III [NKI1998-1854]; FEDER [NKI2004-3088]; Accion Estrategica de Salud del Instituto de Salud Carlos III FIS Intrasalud [NKI2007-3756]; Programa Grupos Emergentes, Cancer Genetics Unit, Instituto de Investigacion Biomedica Galicia Sur; Xerencia de Xestion Integrada de Vigo-SERGAS, Instituto de Salud Carlos III, Spain; Conselleria de Industria Programa Sectorial de Investigacion Aplicada, PEME I + De I + D Suma del Plan Gallego de Investigacion, Desarrollo e Innovacion Tecnologica de la Conselleriade Industria de la Xunta de Galicia, Spain [NKI 12535]; Fomento de la Investigacion ClinicaIndependiente, Ministerio de Sanidad, Servicios Sociales e Igualdad, Spain [FIS PI12/02125]; Dietmar-Hopp Foundation; Helmholtz Society; Consejo Nacional de Ciencia y Tecnologia (CONACyT) [PI17/00918]; National Cancer Institute (NCI) [PI13/01136, 10CSA012E]; University of Crete; Fondation de France; Institut National du Cancer (INCa); Ligue Nationale contre le Cancer; Agence Nationale de SecuriteSanitaire, de l'Alimentation, de l'Environnement et du Travail (ANSES); Agence Nationale de la Recherche (ANR); Chief Physician Johan Boserup and Lise Boserup Fund; Danish Medical Research Council; Herlev and Gentofte Hospital; Instituto de Salud Carlos III, the Red Tematica de Investigacion Cooperativa en Cancer; Asociacion Espanola Contra el Cancer; Fondo de Investigacion Sanitario [EC11-192, ALUD-2002-C01-7462]; NIHR [R01CA120120]; NIHR Manchester Biomedical Research Centre [K24CA169004]; German Cancer Aid [PI11/00923, PI12/00070]; Federal Ministry of Education and Research, Germany [PGfAR0707-10031]; European Regional Development Fund; Free State of Saxony, Germany [IS-BRC-1215-20007, 70114178, 01GY1901]; Federal Ministry of Education and Research (BMBF) Germany [713-241202, 14505/2470, 14575/2470, 01KW9975/5]; Robert Bosch Foundation, Stuttgart; Deutsches Krebsforschungszentrum (DKFZ), Heidelberg; Institute for Prevention and Occupational Medicine of the German Social AccidentInsurance, Institute of the Ruhr University Bochum (IPA), Bochum; Department of Internal Medicine, Johanniter GmbH Bonn, Johanniter Krankenhaus, Bonn, Germany; Chief Scientist Office of the Scottish Government Health Directorates [01KW9976/8]; Scottish Funding Council [01KW9977/0]; Medical Research Council UK; Wellcome Trust (Wellcome Trust Strategic Award "STratifying Resilience and DepressionLongitudinally" (STRADL)) [01KW0114]; Wellcome Trust Seed Award "Temporal trends inincidence and mortality of molecular subtypes of breast cancer to inform public

health, policy andprevention" [CZD/16/6]; Deutsche Krebshilfe e.v. [HR03006]; German Cancer Research Center (DKFZ); German Research Foundation (DFG) [104036/Z/14/Z]; Claudia von Schilling Foundation for Breast Cancer Research; Lower Saxonian Cancer Society; Helsinki University Hospital Research Fund; Sigrid Juselius Foundation; Cancer Foundation Finland; Netherlands Organisation of Scientific Research [110005]; Pink Ribbon grants [2014-187.WO76, NWO 184.021.007/CP46]; BBMRI [12-054]; Transcan grant JTC 2012 Cancer [RUS08/017]; Friends of Hannover Medical School; Rudolf Bartling Foundation; German Federal Ministry of Research and Education [17-44-020498]; Russian Foundation for Basic Research [17-29-0601, 18-29-09129, 020-220-08-2197]; Government of Russian Federation [AAAA-A16-116020350032-1]; Ministry of Science and Higher Education of the Russian Federation [400413]; Stockholm County Council; Karolinska Institutet; Swedish Cancer Society; Gustav V Jubilee foundation; Bert von Kantzows foundation; Maerit and Hans Rausings Initiative Against Breast Cancer; Government Funding (VTR) of Kuopio University Hospital grants; Cancer Fund of North Savo; Finnish Cancer Organizations; University of Eastern Finland; National Breast Cancer Foundation; National Health and Medical Research Council (NHMRC); Queensland Cancer Fund; Cancer Council New South Wales; Cancer Council South Australia; Cancer Foundation of Western Australia; Cancer Council Tasmania; National Health and Medical Research Council of Australia (NHMRC) [400281, 199600, 0104/13]; Hamburg Cancer Society; Cyprus Research Promotion Foundation [0104/17, 209057]; VicHealth; Cancer Council Victoria; Australian National Health and Medical Research Council [396414, 1074383]; [110837]; Wellcome Trust [207800/Z/17/Z]
Funding Source: Wellcome Trust Associazione Italiana Ricerca sul Cancro (AIRC)(Fondazione AIRC per la ricerca sul cancro); Fondazione Umberto Veronesi(Fondazione Umberto Veronesi); European Union's Horizon 2020 Research and Innovation Programme(Horizon 2020); Government of Canada through Genome Canada(Genome Canada); Canadian Institutes of Health Research(Canadian Institutes of Health Research (CIHR)); Ministere de l'Economie et de l'Innovation du Quebec through Genome Quebec; Quebec Breast Cancer Foundation; National Cancer Institute Intramural Research Program, National Institutes of Health; European Union Horizon 2020 research and innovation program BRIDGES(Horizon 2020); Wellcome Trust(Wellcome Trust); Dutch Cancer Society(KWF Kankerbestrijding); Dutch Ministry of Health, Welfare and Sport; ELAN-Fond of the University Hospital of Erlangen; NIHR Comprehensive Biomedical Research Centre, Guy's AMP; St. Thomas' NHS Foundation Trust; King's College London, United Kingdom; Oxford Biomedical Research Centre; Accion Estrategica de Salud del Instituto de Salud Carlos III(Instituto de Salud Carlos III); FEDER(European Union (EU)Spanish Government); Accion Estrategica de Salud del Instituto de Salud Carlos III FIS Intrasalud; Programa Grupos Emergentes, Cancer Genetics Unit, Instituto de Investigacion Biomedica Galicia Sur; Xerencia de Xestion Integrada de Vigo-SERGAS, Instituto de Salud Carlos III, Spain; Conselleria de Industria Programa Sectorial de Investigacion Aplicada, PEME I + De I + D Suma del Plan Gallego de Investigacion, Desarrollo e Innovacion Tecnologica de la Conselleriade Industria de la Xunta de Galicia, Spain; Fomento de la Investigacion ClinicaIndependiente, Ministerio de Sanidad, Servicios Sociales e Igualdad, Spain; Dietmar-Hopp Foundation(ACEV Foundation); Helmholtz Society(Helmholtz Association); Consejo Nacional de

Ciencia y Tecnologia (CONACyT)(Consejo Nacional de Ciencia y Tecnologia (CONACyT)); National Cancer Institute (NCI)(United States Department of Health & Human ServicesNational Institutes of Health (NIH) - USANIH National Cancer Institute (NCI)); University of Crete; Fondation de France(Fondation de France); Institut National du Cancer (INCa)(Institut National du Cancer (INCA) France); Ligue Nationale contre le Cancer(Ligue nationale contre le cancer); Agence Nationale de SecuriteSanitaire, de l'Alimentation, de l'Environnement et du Travail (ANSES)(Agence Nationale de la Recherche (ANR)); Agence Nationale de la Recherche (ANR)(Agence Nationale de la Recherche (ANR)); Chief Physician Johan Boserup and Lise Boserup Fund; Danish Medical Research Council(Danish Medical Research CouncilUK Research & Innovation (UKRI)Medical Research Council UK (MRC)); Herlev and Gentofte Hospital; Instituto de Salud Carlos III, the Red Tematica de Investigacion Cooperativa en Cancer; Asociacion Espanola Contra el Cancer; Fondo de Investigacion Sanitario; NIHR(National Institutes of Health Research (NIHR)); NIHR Manchester Biomedical Research Centre(National Institutes of Health Research (NIHR)); German Cancer Aid(Deutsche Krebshilfe); Federal Ministry of Education and Research, Germany(Federal Ministry of Education & Research (BMBF)); European Regional Development Fund(European Union (EU)); Free State of Saxony, Germany; Federal Ministry of Education and Research (BMBF) Germany(Federal Ministry of Education & Research (BMBF)); Robert Bosch Foundation, Stuttgart; Deutsches Krebsforschungszentrum (DKFZ), Heidelberg; Institute for Prevention and Occupational Medicine of the German Social AccidentInsurance, Institute of the Ruhr University Bochum (IPA), Bochum; Department of Internal Medicine, Johanniter GmbH Bonn, Johanniter Krankenhaus, Bonn, Germany; Chief Scientist Office of the Scottish Government Health Directorates(Chief Scientist Office - Scotland); Scottish Funding Council; Medical Research Council UK(UK Research & Innovation (UKRI)Medical Research Council UK (MRC)); Wellcome Trust (Wellcome Trust Strategic Award "STratifying Resilience and DepressionLongitudinally" (STRADL))(Wellcome Trust); Wellcome Trust Seed Award "Temporal trends inincidence and mortality of molecular subtypes of breast cancer to inform public health, policy andprevention"; Deutsche Krebshilfe e.v.(Deutsche Krebshilfe); German Cancer Research Center (DKFZ)(Helmholtz Association); German Research Foundation (DFG)(German Research Foundation (DFG)); Claudia von Schilling Foundation for Breast Cancer Research; Lower Saxonian Cancer Society; Helsinki University Hospital Research Fund; Sigrid Juselius Foundation(Sigrid Juselius Foundation); Cancer Foundation Finland; Netherlands Organisation of Scientific Research(Netherlands Organization for Scientific Research (NWO)); Pink Ribbon grants; BBMRI; Transcan grant JTC 2012 Cancer; Friends of Hannover Medical School; Rudolf Bartling Foundation; German Federal Ministry of Research and Education; Russian Foundation for Basic Research(Russian Foundation for Basic Research (RFBR)Spanish Government); Government of Russian Federation; Ministry of Science and Higher Education of the Russian Federation; Stockholm County Council(Region Stockholm); Karolinska Institutet(Karolinska Institutet); Swedish Cancer Society(Swedish Cancer Society); Gustav V Jubilee foundation; Bert von Kantzows foundation; Maerit and Hans Rausings Initiative Against Breast Cancer; Government Funding (VTR) of Kuopio University Hospital grants; Cancer Fund of North Savo; Finnish Cancer Organizations; University of Eastern

Finland; National Breast Cancer Foundation; National Health and Medical Research Council (NHMRC)(National Health & Medical Research Council (NHMRC) of Australia); Queensland Cancer Fund; Cancer Council New South Wales(Cancer Council New South Wales); Cancer Council South Australia(Cancer Council South Australia); Cancer Foundation of Western Australia(Cancer Council Western Australia); Cancer Council Tasmania(Cancer Council Tasmania); National Health and Medical Research Council of Australia (NHMRC)(National Health & Medical Research Council (NHMRC) of Australia); Hamburg Cancer Society; Cyprus Research Promotion Foundation(Research and Innovation Foundation (RIF)); VicHealth; Cancer Council Victoria(Canadian Institutes of Health Research (CIHR)Cancer Council Victoria); Australian National Health and Medical Research Council(National Health & Medical Research Council (NHMRC) of Australia); ; Wellcome Trust(Wellcome Trust)

This study was supported by Associazione Italiana Ricerca sul Cancro (AIRC; IG22860) to Paolo Peterlongo and by a fellowship from Fondazione Umberto Veronesi to Gisella Figlioli. BCAC is funded by the European Union's Horizon 2020 Research and Innovation Programme (grant numbers 634935 and 633784 for BRIDGES and B-CAST, respectively), and the PERSPECTIVE I&I project, funded by the Government of Canada through Genome Canada and the Canadian Institutes of Health Research, the Ministere de l'Economie et de l'Innovation du Quebec through Genome Quebec, the Quebec Breast Cancer Foundation. The EU Horizon 2020 Research and Innovation Programme funding source had no role in study design, data collection, data analysis, data interpretation or writing of the report. Additional funding for BCAC is provided via the Confluence project which is funded with intramural funds from the National Cancer Institute Intramural Research Program, National Institutes of Health. The BRIDGES panel sequencing was supported by the European Union Horizon 2020 research and innovation program BRIDGES (grant number, 634935) and the Wellcome Trust (v203477/Z/16/Z). The ABCS study was supported by the Dutch Cancer Society [grants NKI 2007-3839; 2009 4363] and an institutional grant of the Dutch Cancer Society and of the Dutch Ministry of Health, Welfare and Sport. The work of the BBCC was partly funded by ELAN-Fond of the University Hospital of Erlangen. For BIGGS, ES is supported by NIHR Comprehensive Biomedical Research Centre, Guy's & St. Thomas' NHS Foundation Trust in partnership with King's College London, United Kingdom. IT is supported by the Oxford Biomedical Research Centre. The BREast Oncology GAlician Network (BREOGAN) is funded by Accion Estrategica de Salud del Instituto de Salud Carlos III FIS PI12/02125/Cofinanciado and FEDER PI17/00918/Cofinanciado FEDER; Accion Estrategica de Salud del Instituto de Salud Carlos III FIS Intrasalud (PI13/01136); Programa Grupos Emergentes, Cancer Genetics Unit, Instituto de Investigacion Biomedica Galicia Sur. Xerencia de Xestion Integrada de Vigo-SERGAS, Instituto de Salud Carlos III, Spain; Grant 10CSA012E, Conselleria de Industria Programa Sectorial de Investigacion Aplicada, PEME I + D e I + D Suma del Plan Gallego de Investigacion, Desarrollo e Innovacion Tecnologica de la Conselleria de Industria de la Xunta de Galicia, Spain; Grant EC11-192. Fomento de la Investigacion Clinica Independiente, Ministerio de Sanidad, Servicios Sociales e Igualdad, Spain; and Grant FEDER-Innterconecta. Ministerio de Economia y Competitividad, Xunta de Galicia, Spain. The BSUCH study was supported by the Dietmar-Hopp Foundation, the Helmholtz Society

and the German Cancer Research Center (DKFZ). The CAMA study was funded by Consejo Nacional de Ciencia y Tecnologia (CONACyT) (SALUD-2002-C01-7462). Sample collection and processing was funded in part by grants from the National Cancer Institute (NCI R01CA120120 and K24CA169004). CCGP is supported by funding from the University of Crete. The CECILE study was supported by Fondation de France, Institut National du Cancer (INCa), Ligue Nationale contre le Cancer, Agence Nationale de Securite Sanitaire, de l'Alimentation, de l'Environnement et du Travail (ANSES), Agence Nationale de la Recherche (ANR). The CGPS was supported by the Chief Physician Johan Boserup and Lise Boserup Fund, the Danish Medical Research Council and Herlev and Gentofte Hospital.r The CNIO-BCS was supported by the Instituto de Salud Carlos III, the Red Tematica de Investigacion Cooperativa en Cancer and grants from the Asociacion Espanola Contra el Cancer and the Fondo de Investigacion Sanitario (PI11/00923 and PI12/00070). FHRISK and PROCAS are funded from NIHR grant PGfAR 0707-10031. DGE, AH and WGN are supported by the NIHR Manchester Biomedical Research Centre (IS-BRC-1215-20007). The GC-HBOC (German Consortium of Hereditary Breast and Ovarian Cancer) is supported by the German Cancer Aid (grant no 110837 and 70114178, coordinator: Rita K. Schmutzler, Cologne) and the Federal Ministry of Education and Research, Germany (grant no 01GY1901). This work was also funded by the European Regional Development Fund and Free State of Saxony, Germany (LIFE-Leipzig Research Centre for Civilization Diseases, project numbers 713-241202, 713-241202, 14505/2470, 14575/2470). The GENICA was funded by the Federal Ministry of Education and Research (BMBF) Germany grants 01KW9975/5, 01KW9976/8, 01KW9977/0 and 01KW0114, the Robert Bosch Foundation, Stuttgart, Deutsches Krebsforschungszentrum (DKFZ), Heidelberg, the Institute for Prevention and Occupational Medicine of the German Social Accident Insurance, Institute of the Ruhr University Bochum (IPA), Bochum, as well as the Department of Internal Medicine, Johanniter GmbH Bonn, Johanniter Krankenhaus, Bonn, Germany. Generation Scotland (GENSCOT) received core support from the Chief Scientist Office of the Scottish Government Health Directorates [CZD/16/6] and the Scottish Funding Council [HR03006]. Genotyping of the GS:SFHS samples was carried out by the Genetics Core Laboratory at the Edinburgh Clinical Research Facility, University of Edinburgh, Scotland and was funded by the Medical Research Council UK and the Wellcome Trust (Wellcome Trust Strategic Award "STratifying Resilience and Depression Longitudinally" (STRADL) Reference 104036/Z/14/Z). Funding for identification of cases and contribution to BCAC funded in part by the Wellcome Trust Seed Award "Temporal trends in incidence and mortality of molecular subtypes of breast cancer to inform public health, policy and prevention" Reference 207800/Z/17/Z. The GESBC was supported by the Deutsche Krebshilfe e. V. [70492] and the German Cancer Research Center (DKFZ). The HABCS study was supported by German Research Foundation (DFG Do761/15-1), the Claudia von Schilling Foundation for Breast Cancer Research, by the Lower Saxonian Cancer Society, and by the Rudolf Bartling Foundation. The HEBCS was financially supported by the Helsinki University Hospital Research Fund, the Sigrid Juselius Foundation and the Cancer Foundation Finland. The HEBON study is supported by the Dutch Cancer Society grants NKI1998-1854, NKI2004-3088, NKI2007-3756, NKI 12535, the Netherlands Organisation of Scientific Research grant NWO 91109024, the Pink Ribbon grants

110005 and 2014-187.WO76, the BBMRI grant NWO 184.021.007/CP46, and the Transcan grant JTC 2012 Cancer 12-054. The HMBCS was supported by the German Research Foundation (DFG Do761/151), a grant from the Friends of Hannover Medical School, and by the Rudolf Bartling Foundation. The HUBCS was supported by German Research Foundation (DFG Do761/15-1), a grant from the German Federal Ministry of Research and Education (RUS08/017), B.M. was supported by grant 17-44-020498, 17-29-06014 of the Russian Foundation for Basic Research, D.P. was supported by grant 18-29-09129 of the Russian Foundation for Basic Research, E.r K was supported by the mega grant from the Government of Russian Federation (2020-220-08-2197), and the study was performed as part of the assignment of the Ministry of Science and Higher Education of the Russian Federation (No AAAAA-16-116020350032-1). Financial support for KARBAC was provided through the regional agreement on medical training and clinical research (ALF) between Stockholm County Council and Karolinska Institutet, the Swedish Cancer Society, The Gustav V Jubilee foundation and Bert von Kantzows foundation. The KARMA study was supported by Marit and Hans Rausings Initiative Against Breast Cancer. The KBCP was financially supported by the special Government Funding (VTR) of Kuopio University Hospital grants, Cancer Fund of North Savo, the Finnish Cancer Organizations and by the strategic funding of the University of Eastern Finland. kConFab was supported by a grant from the National Breast Cancer Foundation, and previously by the National Health and Medical Research Council (NHMRC), the Queensland Cancer Fund, the Cancer Councils of New South Wales, Victoria, Tasmania and South Australia and the Cancer Foundation of Western Australia. Financial support for the AOCS was provided by the United States Army Medical Research and Materiel Command [DAMD17-01-1-0729], Cancer Council Victoria, Queensland Cancer Fund, Cancer Council New South Wales, Cancer Council South Australia, The Cancer Foundation of Western Australia, Cancer Council Tasmania and the National Health and Medical Research Council of Australia (NHMRC; 400413, 400281, 199600). G.C.T. and P.W. were supported by the NHMRC. RB was a Cancer Institute NSW Clinical Research Fellow. The MARIE study was supported by the Deutsche Krebshilfe e.V. [70-2892-BR I, 106332, 108253, 108419, 110826, 110828], the Hamburg Cancer Society, the German Cancer Research Center (DKFZ) and the Federal Ministry of Education and Research (BMBF) Germany [01KH0402]. The MASTOS study was supported by "Cyprus Research Promotion Foundation" grants 0104/13 and 0104/17, and the Cyprus Institute of Neurology and Genetics. MBCSG is supported by grants from the Italian Association for Cancer Research (AIRC). The Melbourne Collaborative Cohort Study (MCCS) cohort recruitment was funded by VicHealth and Cancer Council Victoria. The MCCS was further augmented by Australian National Health and Medical Research Council grants 209057, 396414 and 1074383 and by infrastructure provided by Cancer Council Victoria. Cases and their vital status were ascertained through the Victorian Cancer Registry and the Australian Institute of Health and Welfare, including the National Death Index and the Australian Cancer Database. The NBCS has received funding from the K.G. Jebsen Centre for Breast Cancer Research; the Research Council of Norway grant 193387/V50 (to A-L Borresen-Dale and V.N. Kristensen) and grant 193387/H10 (to A-L Borresen-Dale and V.N. Kristensen), South Eastern Norway Health Authority (grant 39346 to A-L Borresen-Dale) and the Norwegian Cancer Society (to A-L Borresen-

Dale and V.N. Kristensen). The NBHS was supported by NIH grant R01CA100374. Biological sample preparation was conducted the Survey and Biospecimen Shared Resource, which is supported by P30 CA68485. The Ontario Familial Breast Cancer Registry (OFBCR) was supported by grant U01CA164920 from the USA National Cancer Institute of the National Institutes of Health.r The content of this manuscript does not necessarily reflect the views or policies of the National Cancer Institute or any of the collaborating centers in the Breast Cancer Family Registry (BCFR), nor does mention of trade names, commercial products, or organizations imply endorsement by the USA Government or the BCFR. The PBCS was funded by Intramural Research Funds of the National Cancer Institute, Department of Health and Human Services, USA. Genotyping for PLCO was supported by the Intramural Research Program of the National Institutes of Health, NCI, Division of Cancer Epidemiology and Genetics. The PLCO is supported by the Intramural Research Program of the Division of Cancer Epidemiology and Genetics and supported by contracts from the Division of Cancer Prevention, National Cancer Institute, National Institutes of Health. The RBCS was funded by the Dutch Cancer Society (DDHK 2004-3124, DDHK 2009-4318). The SASBAC study was supported by funding from the Agency for Science, Technology and Research of Singapore (A*STAR), the US National Institute of Health (NIH) and the Susan G. Komen Breast Cancer Foundation. SEARCH was funded by Cancer Research UK [C490/A10124, C490/A16561] and supported by the UK National Institute for Health Research Biomedical Research Centre at the University of Cambridge. The University of Cambridge has received salary support for PDPP from the NHS in the East of England through the Clinical Academic Reserve. SKKDKFZS was supported by the DKFZ. The SZBCS was supported by Grant PBZ_KBN_122/P05/2004 and the program of the Minister of Science and Higher Education under the name "Regional Initiative of Excellence" in 2019-2022 project number 002/RID/2018/19 amount of financing 12,000,000 PLN. UBCS was supported by funding from National Cancer Institute (NCI) grant R01 CA163353 (to N.J. Camp) and the Women's Cancer Center at the Huntsman Cancer Institute (HCI). Data collection for UBCS was supported by the Utah Population Database (UPDB) and Utah Cancer Registry (UCR). Partial support for all datasets within the UPDB was provided by the University of Utah HCI and the HCI Cancer Center Support grant, P30 CA2014 from the NCI. The UCR is funded by the NCI's SEER Program, Contract No. HHSN261201800016I, the US Centers for Disease Control and Prevention's National Program of Cancer Registries, Cooperative Agreement No. NU58DP006320, with additional support from the University of Utah and Huntsman Cancer Foundation. 31 0 0 1 7 MDPI BASEL ST ALBAN-ANLAGE 66, CH-4052 BASEL, SWITZERLAND 2072-6694
CANCERS Cancers JUL 2023 15 13
3313 10.3390/cancers15133313
http://dx.doi.org/10.3390/cancers15133313 16
Oncology Science Citation Index Expanded (SCI-EXPANDED)
Oncology P1YI2 37444426 Green Published, Green Submitted, gold 2025-06-24 WOS:001048663200001
J Freirj, MA; Khadra, MM; Abu Farsakh, HA; Saleh, HH; Ijmail, AA; Rahal, BO; Waldali, MH; Najeeb, NS; Tahtamouni, LH
Freirj, M. A.; Khadra, M. M.; Abu Farsakh, H. A.; Saleh, H. H.; Ijmail, A. A.; Rahal, B. O.; Waldali, M. H.; Najeeb, N. S.; Tahtamouni, L. H. Low rate of cervical

cancer among women with rising incidence of cervical cytological abnormalities. The unlearnt lesson EUROPEAN JOURNAL OF GYNAECOLOGICAL ONCOLOGY English Article
Pap smear; Cervical cancer; HPV; Cervical screening program; Jordan EXTENDED MIDDLE-EAST; HUMAN-PAPILLOMAVIRUS; NORTH-AFRICA; PAP-SMEAR; PREVENTION; ADENOCARCINOMA; KNOWLEDGE Objectives: Cervical cancer is preceded by a group of epithelial cell abnormalities. However, there is insufficient data on cervical abnormalities in Jordan and the Middle East at large. The current study aimed at determining the prevalence of different cytological abnormalities in women in Jordan. In addition. it aimed at assessing the age specific cytological abnormalities in these women and analyzing the changing trends of epithelial cell abnormalities in cervical smear over a period of 15 years compared in three periods of five years each. Materials and Methods: 6,455 conventional cervical Papanicolaou (Pap) smear results obtained between January 2000 and December 2014 were retrospectively analyzed. Results: Out of the 6,454 Pap smears analyzed, 5,645 (87.5%) were found adequate for reporting. A total number of 801 (14.2 %) cases had cervical epithelial abnormalities. A significant increase in cytological abnormalities was observed between 2000 and 2014. In addition, a significant increasing trend in cervical cytological abnormalities was noted between 2000 and 2014. The highest percentage of cytological abnormalities (20.1%) was found in women younger than 25 years old. In all of the age groups, the low-grade squamous intraepithelial lesions (LSIL) cytological abnormality was the most prevalent. Conclusions: Invasive cervical cancer is still a killer for young women in the developing countries. The present study may reflect a change in the sociosexual behavior over the last 15 years. The current work highlights the importance of awareness campaigns on the importance of cervical smear and the urgent need for initiating a cervical screening program in Jordan.
[Freirj, M. A.; Khadra, M. M.; Rahal, B. O.] Univ Jordan, Dept Obstet & Gynecol, Fac Med, Queen Rania St, Amman 11942, Jordan; [Abu Farsakh, H. A.; Ijmail, A. A.] First Med Labs, Amman, Jordan; [Saleh, H. H.] Elite Moms Clin, Amman, Jordan; [Waldali, M. H.] Specialty Hosp, Obstet & Gynecol Dept, Amman, Jordan; [Najeeb, N. S.; Tahtamouni, L. H.] Hashemite Univ, Dept Biol & Biotechnol, Fac Sci, Zarqa, Jordan University of Jordan; Hashemite University Freirj, MA (corresponding author), Univ Jordan, Dept Obstet & Gynecol, Fac Med, Queen Rania St, Amman 11942, Jordan. Mazen2k@yahoo.com Khadra, Maysa/M-5399-2017; Tahtamouni, Lubna/AAT-4121-2020 khadra, Maysa/0000-0003-3042-5686; ABDALLAH, NISSREEN/0000-0002-0765-8230 University of Jordan, Amman-Jordan University of Jordan, Amman-Jordan The authors are grateful for the University of Jordan, Amman-Jordan for supporting this work. 27 0 0 0 2 I R O G CANADA, INC MONTREAL 4900 COTE ST-LUC, APT#212, MONTREAL, QUEBEC H3W 2H3, CANADA 0392-2936 EUR J GYNAECOL ONCOL
Eur. J. Gynaecol. Oncol. 2018 39 1
96 100 10.12892/ejgo3722.2018
http://dx.doi.org/10.12892/ejgo3722.2018 5
Oncology; Obstetrics & Gynecology Science Citation Index Expanded (SCI-EXPANDED) Oncology; Obstetrics & Gynecology
GF0LK 2025-06-24 WOS:000431621900019
J Krusinska, B; Wadolowska, L; Slowinska, MA; Biernacki, M; Drozdowski, M; Chadzynski, T Krusinska, Beata;

Wadolowska, Lidia; Slowinska, Malgorzata Anna; Biernacki, Maciej; Drozdowski, Marek; Chadzynski, Tomasz Associations of Dietary Patterns and Metabolic-Hormone Profiles with Breast Cancer Risk: A Case-Control Study NUTRIENTS English Article breast cancer; dietary pattern; Mediterranean diet; hormones; metabolic syndrome MEDITERRANEAN DIET; CHOLESTEROL; PROLACTIN; COHORT; SERUM; ADHERENCE; ESTROGEN; INSULIN; OBESITY Breast cancer is the most diagnosed cancer in women worldwide. Studies regarding complex breast cancer aetiology are limited and the results are inconclusive. We investigated the associations between dietary patterns (DPs), metabolic-hormone profiles (M-HPs), and breast cancer risk. This case-control study involved 420 women aged 40-79 years from north-eastern Poland, including 190 newly-diagnosed breast cancer cases. The serum concentration of lipid components, glucose, and hormones (oestradiol, progesterone, testosterone, prolactin, cortisol, insulin) was marked in 129 post-menopausal women (82 controls, 47 cases). The food frequency consumption was collected using a validated 62-item food frequency questionnaire. A posteriori DPs or M-HPs were derived with a Principal Component Analysis (PCA). Three DPs: Non-Healthy', Prudent', and Margarine and Sweetened Dairy' and two M-HPs: Metabolic-Syndrome' and High-Hormone' were identified. The Polish-adapted Mediterranean Diet' (Polish-aMED') score was calculated. The risk of breast cancer risk was three-times higher (odds ratio (OR): 2.90; 95% confidence interval (95% Cl): 1.62-5.21; p < 0.001) in the upper tertile of the Non-Healthy' pattern (reference: bottom tertile) and five-times higher (OR: 5.34; 95% Cl: 1.84-15.48; p < 0.01) in the upper tertile of the High-Hormone' profile (reference: bottom tertile). There was a positive association of Metabolic-Syndrome' profile and an inverse association of Polish-aMED' score with the risk of breast cancer, which disappeared after adjustment for confounders. No significant association between Prudent' or Margarine and Sweetened Dairy' DPs and cancer risk was revealed. Concluding, a pro-healthy diet is insufficient to reduce the risk of breast cancer in peri- and postmenopausal women. The findings highlight the harmful effect of the High-Hormone' profile and the Non-Healthy' dietary pattern on breast cancer risk. In breast cancer prevention, special attention should be paid to decreasing the adherence to the Non-Healthy' pattern by reducing the consumption of highly processed food and foods with a high content of sugar and animal fat. There is also a need to monitor the concentration of multiple sex hormones in the context of breast cancer risk.

[Krusinska, Beata; Wadolowska, Lidia; Slowinska, Malgorzata Anna; Chadzynski, Tomasz] Univ Warmia & Mazury, Dept Human Nutr, Sloneczna 45f, PL-10718 Olsztyn, Poland; [Biernacki, Maciej] Univ Warmia & Mazury, Dept Surg, PL-11041 Olsztyn, Poland; [Drozdowski, Marek] Univ Warmia & Mazury, Dept Lab Med, PL-11041 Olsztyn, Poland University of Warmia & Mazury; University of Warmia & Mazury; University of Warmia & Mazury Krusinska, B (corresponding author), Univ Warmia & Mazury, Dept Human Nutr, Sloneczna 45f, PL-10718 Olsztyn, Poland.

beata.krusinska@uwm.edu.pl; lidia.wadolowska@uwm.edu.pl; malgorzata.slowinska@uwm.edu.pl; maciej.biernacki@uwm.edu.pl; marek.drozdowski@uwm.edu.pl; tomasz.chadzynski@uwm.edu.pl

Wądołowska, Lidia/L-1458-2019; Wadolowska, Lidia/O-8463-2018

Stasiewicz, Beata/0000-0003-0718-9101; Wadolowska, Lidia/0000-0001-8571-9935 Polish Ministry of Science and Higher

Education - Roche Diagnostics Company Polish Ministry of Science and Higher Education - Roche Diagnostics Company This research was funded by Polish Ministry of Science and Higher Education. The part of chemical reagents was funded by the Roche Diagnostics Company. 62 19 19 0 12 MDPI BASEL ST ALBAN-ANLAGE 66, CH-4052 BASEL, SWITZERLAND 2072-6643 NUTRIENTS Nutrients DEC 2018 10 12 2013 10.3390/nu10122013 http://dx.doi.org/10.3390/nu10122013 23 Nutrition & Dietetics Science Citation Index Expanded (SCI-EXPANDED) Nutrition & Dietetics HG6EG 30572623 Green Published, Green Submitted, gold 2025-06-24 WOS:000455073200194

J Costantini, S; Guerriero, E; Teta, R; Capone, F; Caso, A; Sorice, A; Romano, G; Ianora, A; Ruocco, N; Budillon, A; Costantino, V; Costantini, M Costantini, Susan; Guerriero, Eliana; Teta, Roberta; Capone, Francesca; Caso, Alessia; Sorice, Angela; Romano, Giovanna; Ianora, Adrianna; Ruocco, Nadia; Budillon, Alfredo; Costantino, Valeria; Costantini, Maria Evaluating the Effects of an Organic Extract from the Mediterranean Sponge Geodia cydonium on Human Breast Cancer Cell Lines INTERNATIONAL JOURNAL OF MOLECULAR SCIENCES English Article breast cancer; cytotoxicity; metabolomics; cytokines; sponges CARIBBEAN SPONGE; NATURAL-PRODUCTS; ANTITUMOR; METABOLISM; NUCLEOSIDES; PATHWAY Marine sponges are an excellent source of bioactive secondary metabolites for pharmacological applications. In the present study, we evaluated the chemistry, cytotoxicity and metabolomics of an organic extract from the Mediterranean marine sponge Geodia cydonium, collected in coastal waters of the Gulf of Naples. We identified an active fraction able to block proliferation of breast cancer cell lines MCF-7, MDA-MB231, and MDA-MB468 and to induce cellular apoptosis, whereas it was inactive on normal breast cells (MCF-10A). Metabolomic studies showed that this active fraction was able to interfere with amino acid metabolism, as well as to modulate glycolysis and glycosphingolipid metabolic pathways. In addition, the evaluation of the cytokinome profile on the polar fractions of three treated breast cancer cell lines (compared to untreated cells) demonstrated that this fraction induced a slight anti-inflammatory effect. Finally, the chemical entities present in this fraction were analyzed by liquid chromatography high resolution mass spectrometry combined with molecular networking. [Costantini, Susan; Guerriero, Eliana; Capone, Francesca; Sorice, Angela; Budillon, Alfredo] Ist Nazl Tumori Fdn G Pascale IRCCS, Expt Pharmacol Unit, I-80131 Naples, Italy; [Teta, Roberta; Caso, Alessia; Costantino, Valeria] Univ Naples Federico II, Dept Pharm, Via Domenico Montesano 49, I-80131 Naples, Italy; [Romano, Giovanna; Ianora, Adrianna] Stn Zool Anton Dohrn, Dept Integrat Marine Ecol, I-80121 Naples, Italy; [Ruocco, Nadia; Costantini, Maria] Stn Zool Anton Dohrn, Dept Biol & Evolut Marine Organisms, I-80121 Naples, Italy; [Ruocco, Nadia] Univ Naples Federico II, Dept Biol, Complesso Univ Monte St Angelo,Via Cinthia, I-80126 Naples, Italy; [Ruocco, Nadia] CNR, Bioorgan Chem Unit, Inst Biomol Chem, Via Campi Flegrei 34, I-80078 Naples, Italy IRCCS Fondazione Pascale; University of Naples Federico II; Stazione Zoologica Anton Dohrn; Stazione Zoologica Anton Dohrn; University of Naples Federico II; Consiglio Nazionale delle Ricerche (CNR)

Costantini, M (corresponding author), Stn Zool Anton Dohrn, Dept Biol & Evolut Marine Organisms, I-80121 Naples, Italy.
s.costantini@istitutotumori.na.it; e.guerriero@istitutotumori.na.it; roberta.teta@unina.it; f.capone@istitutotumori.na.it; alessia.caso@unina.it; a.sorice@istitutotumori.na.it; romano@szn.it; ianora@szn.it; nadia.ruocco@szn.it; a.budillon@istitutotumori.na.it; costanti@unina.it; maria.costantini@szn.it Costantini, Susan/J-9914-2018; Ianora, Adrianna/O-3408-2019; Costantini, Maria/AGH-4080-2022; Ruocco, Nadia/HGA-2143-2022; Costantino, Valeria/AAN-1960-2020; Romano, Giovanna/F-4561-2015; Teta, Roberta/C-8359-2011; Budillon, Alfredo/K-4763-2016; Capone, Francesca/AAS-7458-2021 Budillon, Alfredo/0000-0002-6330-6053; Costantini, Susan/0000-0002-7538-4657; CASO, Alessia/0000-0002-5395-2457; Romano, Giovanna/0000-0002-4898-7153; TETA, Roberta/0000-0003-0817-7743; Sorice, Angela/0000-0003-4900-6107; Capone, Francesca/0000-0002-5402-6228 PhD (PhD in Biology, University of Naples Federico II) fellowship - Stazione Zoologica Anton Dohrn; Bio-Organic Chemistry Unit of the Institute of Biomolecular Chemistry-CNR, Pozzuoli, Naples PhD (PhD in Biology, University of Naples Federico II) fellowship - Stazione Zoologica Anton Dohrn; Bio-Organic Chemistry Unit of the Institute of Biomolecular Chemistry-CNR, Pozzuoli, Naples Firstly, we thank the "Parco Sommerso di Baia" in Naples for providing Geodia cydonium and the Fishing Service of Stazione Zoologica Anton Dohrn. We also thank Davide Caramiello from the Marine Resources for Research Unit (Stazione Zoologica) for his technical support in sponge maintenance. Nadia Ruocco has been supported by a PhD (PhD in Biology, University of Naples Federico II) fellowship co-funded by the Stazione Zoologica Anton Dohrn and Bio-Organic Chemistry Unit of the Institute of Biomolecular Chemistry-CNR, Pozzuoli, Naples.
38 14 14 0 19 MDPI BASEL ST ALBAN-ANLAGE 66, CH-4052 BASEL, SWITZERLAND 1422-0067 INT J MOL SCI Int. J. Mol. Sci. OCT 2017 18 10
2112 10.3390/ijms18102112
http://dx.doi.org/10.3390/ijms18102112 16
Biochemistry & Molecular Biology; Chemistry, Multidisciplinary Science Citation Index Expanded (SCI-EXPANDED) Biochemistry & Molecular Biology; Chemistry FM0QL
28991212 Green Published, gold 2025-06-24
WOS:000414671800096
J Castelló, A; Pollán, M; Buijsse, B; Ruiz, A; Casas, AM; Baena-Cañada, JM; Lope, V; Antolín, S; Ramos, M; Muñoz, M; Lluch, A; de Juan-Ferré, A; Jara, C; Jimeno, MA; Rosado, P; Díaz, E; Guillem, V; Carrasco, E; Pérez-Gómez, B; Vioque, J; Boeing, H; Martín, M Castello, A.; Pollan, M.; Buijsse, B.; Ruiz, A.; Casas, A. M.; Baena-Canada, J. M.; Lope, V.; Antolin, S.; Ramos, M.; Munoz, M.; Lluch, A.; de Juan-Ferre, A.; Jara, C.; Jimeno, M. A.; Rosado, P.; Diaz, E.; Guillem, V.; Carrasco, E.; Perez-Gomez, B.; Vioque, J.; Boeing, H.; Martin, M.
GEICAM Spanish Mediterranean diet and other dietary patterns and breast cancer risk: case-control EpiGEICAM study
BRITISH JOURNAL OF CANCER English Article
breast neoplasms; dietary patterns; aMED; AHEI; principal component analysis; Mediterranean pattern PLASMA-CONCENTRATIONS; MULTIPLE IMPUTATION; OLIVE OIL; WOMEN; METAANALYSIS; REPRODUCIBILITY; CONSUMPTION; ADHERENCE; VALIDITY; QUALITY Background: Although there are solid

findings regarding the detrimental effect of alcohol consumption, the existing evidence on the effect of other dietary factors on breast cancer (BC) risk is inconclusive. This study aimed to evaluate the association between dietary patterns and risk of BC in Spanish women, stratifying by menopausal status and tumour subtype, and to compare the results with those of Alternate Healthy Index (AHEI) and Alternate Mediterranean Diet Score (aMED). Methods: We recruited 1017 incident BC cases and 1017 matched healthy controls of similar age (+/- 5 years) without a history of BC. The association between 'a priori' and 'a posteriori' developed dietary patterns and BC in general and according to menopausal status and intrinsic tumour subtypes (ER+/PR+ and HER2-; HER2+; and ER-/PR- and HER2-) was evaluated using logistic and multinomial regression models. Results: Adherence to the Western dietary pattern was related to higher risk of BC (OR for the top vs the bottom quartile 1.46 (95% CI 1.06-2.01)), especially in premenopausal women (OR = 1.75; 95% CI 1.14-2.67). In contrast, the Mediterranean pattern was related to a lower risk (OR for the top quartile vs the bottom quartile 0.56 (95% CI 0.40-0.79)). Although the deleterious effect of the Western pattern was similarly observed in all tumour subtypes, the protective effect of our Mediterranean pattern was stronger for triple-negative tumours (OR = 0.32; 95% CI 0.15-0.66 and P-heterogeneity = 0.04). No association was found between adherence to the Prudent pattern and BC risk. The associations between 'a priori' indices and BC risk were less marked (OR for the top vs the bottom quartile of AHEI = 0.69; 95% CI 0.51-0.94 and aMED = 0.74; 95% CI 0.46-1.18)). Conclusions: Our results confirm the harmful effect of a Western diet on BC risk, and add new evidence on the benefits of a diet rich in fruits, vegetables, legumes, oily fish and vegetable oils for preventing all BC subtypes, and particularly triple-negative tumours. [Castello, A.; Pollan, M.; Lope, V.; Perez-Gomez, B.] Inst Salud Carlos III, Natl Ctr Epidemiol, Canc Epidemiol Unit, Madrid 28029, Spain; [Castello, A.; Pollan, M.; Lope, V.; Perez-Gomez, B.; Vioque, J.] Carlos III Inst Hlth, Consortium Biomed Res Epidemiol & Publ Hlth CIBER, Madrid 28029, Spain; [Castello, A.; Buijsse, B.; Boeing, H.] German Inst Human Nutr Potsdam Rehbrucke, Dept Epidemiol, D-14558 Nuthetal, Germany; [Ruiz, A.; Guillem, V.] Inst Valenciano Oncol, Med Oncol Unit, Valencia 46009, Spain; [Casas, A. M.; Diaz, E.] Hosp Virgen de Rocio, Med Oncol Unit, Seville 41013, Spain; [Baena-Canada, J. M.; Rosado, P.] Hosp Puerta del Mar, Med Oncol Unit, Cadiz 11009, Spain; [Antolin, S.] Complejo Hosp Univ, Med Oncol Unit, La Coruna 15006, Spain; [Ramos, M.] Ctr Oncol Galicia, Med Oncol Unit, La Coruna 15009, Spain; [Munoz, M.] Hosp Clin I Prov Barcelona, Med Oncol Unit, Barcelona 08036, Spain; [Lluch, A.] Univ Valencia, Dept Hematol Oncol, Hosp Clin, INCLIVA, Valencia 46010, Spain; [de Juan-Ferre, A.] Hosp Marques de Valdecilla, Med Oncol Unit, Santander 39008, Spain; [Jara, C.] Fdn Hosp Alcorco, Med Oncol Unit, Madrid 28922, Spain; [Jimeno, M. A.] Spanish Breast Canc Res Grp GEICAM Headquarters, Madrid 28703, Spain; [Vioque, J.] Univ Miguel Hernandez, Dept Publ Hlth, Sant Joan Dalacant 03550, Spain; [Martin, M.] Hosp Clin Univ San Carlos, Med Oncol Unit, Madrid 28040, Spain; [Martin, M.] Univ Complutense, Hlth Res Inst Gregorio Maranon, Madrid 28007, Spain

Instituto de Salud Carlos III; CIBER - Centro de Investigacion Biomedica en Red; CIBERESP; Instituto de Salud Carlos III; Leibniz Association; Deutsches Institut fur

Ernahrungsforschung Potsdam-Rehbrucke (DIfE); Instituto Valenciano De Oncologia; Universidad de Cadiz; Hospital Universitario Puerta del Mar; University of Valencia; Hospital Universitario Marques de Valdecilla (HUMV); GEICAM; Universidad Miguel Hernandez de Elche; Complutense University of Madrid Pollán, M (corresponding author), Inst Salud Carlos III, Natl Ctr Epidemiol, Canc Epidemiol Unit, Av Monforte de Lemos 5, Madrid 28029, Spain.
mpollan@isciii.es Lluch, Ana/R-5493-2019; Vioque, Jesus/A-1066-2008; Carrasco Bañuelos, Eva/HMP-7611-2023; Martin, Miguel/V-6589-2019; Muñoz, Montserrat/AAO-9617-2020; Perez-Gomez, Beatriz/C-4715-2012; Pollan, Marina/M-3259-2014; Castello, Adela/C-3829-2014; Lope, Virginia/S-4774-2016 Lluch, Ana/0000-0003-2766-407X; Perez-Gomez, Beatriz/0000-0002-4299-8214; Vioque, Jesus/0000-0002-2284-148X; MARTIN, MIGUEL/0000-0001-9237-3231; Antolin Novoa, Silvia/0000-0002-0110-3349; Pollan, Marina/0000-0002-4328-1565; Castello, Adela/0000-0002-1308-9927; Munoz Mateu, Montserrat/0000-0001-7772-1437; Lope, Virginia/0000-0002-6986-4021 Fundacion Cientifica Asociacion Espanola Contra el Cancer (AECC) (Scientific Foundation of the Spanish Association Against Cancer); Fundacion Cerveza y Salud (Beer and Health Foundation); Sociedad Espanola de Oncologia Medica (SEOM) (Spanish Society of Medical Oncology); Federacion de Mujeres con Cancer de Mama (FECMA) (Association of Women with Breast Cancer); Fondo de Investigacion Sanitaria (FIS) (Health Research Fund) [CD110/00018]
Fundacion Cientifica Asociacion Espanola Contra el Cancer (AECC) (Scientific Foundation of the Spanish Association Against Cancer); Fundacion Cerveza y Salud (Beer and Health Foundation); Sociedad Espanola de Oncologia Medica (SEOM) (Spanish Society of Medical Oncology); Federacion de Mujeres con Cancer de Mama (FECMA) (Association of Women with Breast Cancer); Fondo de Investigacion Sanitaria (FIS) (Health Research Fund) This work was supported by the Fundacion Cientifica Asociacion Espanola Contra el Cancer (AECC) (Scientific Foundation of the Spanish Association Against Cancer); Fundacion Cerveza y Salud 2005 (Beer and Health Foundation 2005); Sociedad Espanola de Oncologia Medica (SEOM) (Spanish Society of Medical Oncology); Federacion de Mujeres con Cancer de Mama (FECMA) (Association of Women with Breast Cancer) and Fondo de Investigacion Sanitaria (FIS) (Health Research Fund) CD110/00018. 46 151 156 1 36
SPRINGERNATURE LONDON CAMPUS, 4 CRINAN ST, LONDON, N1 9XW, ENGLAND 0007-0920 1532-1827 BRIT J CANCER Br. J. Cancer SEP 23 2014 111 7 1454
1462 10.1038/bjc.2014.434
http://dx.doi.org/10.1038/bjc.2014.434 9
Oncology Science Citation Index Expanded (SCI-EXPANDED)
Oncology AR1DT 25101568 Green Published, hybrid
2025-06-24 WOS:000343323100024
J Haridass, V; Ziogas, A; Neuhausen, SL; Anton-Culver, H; Odegaard, A Haridass, Vikram; Ziogas, Argyrios; Neuhausen, Susan L.; Anton-Culver, Hoda; Odegaard, Andrew
Diet Quality Scores Inversely Associated with Postmenopausal Breast Cancer Risk Are Not Associated with Premenopausal Breast Cancer Risk in the California Teachers Study
JOURNAL OF NUTRITION English Article
diet quality; diet pattern; aMED; AHEI-2010; DASH; Paleolithic index; breast cancer MEDITERRANEAN DIET; PATTERN SCORES; ADHERENCE; METAANALYSIS; CONSUMPTION; INDEXES; COHORT; WOMEN Background: Evidence for the association

between diet and breast cancer risk is inconsistent Thus, research that compares indexes of overall diet quality may provide new insight. Objective: We examined the association between diet quality indexes and pre- and postmenopausal breast cancer risk in a large prospective cohort. Methods: This was a prospective analysis of 96,959 women, aged 22-104 y, in the California Teachers Study cohort (1995-2011). Diet quality was characterized by 4 different indexes. Specifically, we examined Alternate Mediterranean Diet (aMED), Alternative Healthy Eating Index-2010 (AHEI-2010), Dietary Approaches to Stop Hypertension (DASH), and Paleolithic index (PALEO) scores with the risk of developing breast cancer. We used multivariable Cox proportional hazards regression models to derive HRs and 95% CIs for breast cancer risk. Results: In the analysis of 42,517 women at risk of premenopausal breast cancer, there was no association between any of the indexes and incident breast cancer (346 cases). In the analysis of 54,442 women at risk of postmenopausal breast cancer at baseline, higher AHEI-2010, aMED, and DASH scores were inversely associated with incident breast cancer (3523 incident cases). Respectively, HRs (95% CIs) comparing quintile 5 to quintile 1 (reference) for AHEI-2010, aMED, and DASH indexes were 0.87 (0.78, 0.97; P-trend = 0.004), 0.91 (0.82, 1.02; P-trend = 0.03), and 0.89 (0.80, 1.00; P-trend = 0.03). The PALEO score was not associated with postmenopausal breast cancer (HR for quintile 5 compared with quintile 1: 1.05; 95% CI: 0.94, 1.17). Conclusions: Diet quality indexes that emphasize intake of whole grains, vegetables, fruits, legumes, and nuts and seeds and de-emphasize red and processed meats and sugar-sweetened beverages were modestly associated with a lower risk of incident postmenopausal breast cancer risk. However, they were not associated with premenopausal breast cancer, and the PALEO score was not associated with cancer risk regardless of menopausal status. [Haridass, Vikram; Ziogas, Argyrios; Anton-Culver, Hoda; Odegaard, Andrew] Univ Calif Irvine, Sch Med, Dept Epidemiol, Irvine, CA 92717 USA; [Neuhausen, Susan L.] City Hope Natl Med Ctr, Beckman Res Inst, Dept Populat Sci, Duarte, CA USA

University of California System; University of California Irvine; City of Hope; Beckman Research Institute of City of Hope

Odegaard, A (corresponding author), Univ Calif Irvine, Sch Med, Dept Epidemiol, Irvine, CA 92717 USA. aodegaar@uci.edu

National Cancer Institute (NCI) [R01 CA77398]; California Breast Cancer Research Fund [97-10500]; California Department of Public Health as part of the statewide cancer-reporting program [103885]; NCI's Surveillance, Epidemiology, and End Results Program [HHSN261201000036C, HHSN261201000034C, HHSN261201000035C]; CDC's National Program of Cancer Registries [1U58 DP000807-01] National Cancer Institute (NCI)(United States Department of Health & Human ServicesNational Institutes of Health (NIH) - USANIH National Cancer Institute (NCI)); California Breast Cancer Research Fund; California Department of Public Health as part of the statewide cancer-reporting program; NCI's Surveillance, Epidemiology, and End Results Program(United States Department of Health & Human ServicesNational Institutes of Health (NIH) - USANIH National Cancer Institute (NCI)); CDC's National Program of Cancer RegistriesSupported in part by grants R01 CA77398 from the National Cancer Institute (NCI), and contract 97-10500 from the California Breast Cancer Research Fund. The collection of data on cancer incidence used in this study was

supported by the California Department of Public Health as part of the statewide cancer-reporting program mandated by California Health and Safety Code Section 103885; the NCI's Surveillance, Epidemiology, and End Results Program under contract HHSN261201000036C awarded to the Cancer Prevention Institute of California; contract HHSN261201000035C awarded to the University of Southern California; contract HHSN261201000034C awarded to the Public Health Institute; and the CDC's National Program of Cancer Registries, under agreement 1U58 DP000807-01 awarded to the Public Health Institute. 42 28 30 0 8 OXFORD UNIV PRESS OXFORD GREAT CLARENDON ST, OXFORD OX2 6DP, ENGLAND
0022-3166 1541-6100 J NUTR J. Nutr. NOV 2018
148 11 1830 1837
10.1093/jn/nxy187 http://dx.doi.org/10.1093/jn/nxy187
8 Nutrition & Dietetics Science Citation Index Expanded (SCI-EXPANDED) Nutrition & Dietetics GY9KL
30247577 Bronze 2025-06-24
WOS:000448963200015
J Tseng, M; Sellers, TA; Vierkant, RA; Kushi, LH; Vachon, CM
Tseng, Marilyn; Sellers, Thomas A.; Vierkant, Robert A.; Kushi, Lawrence H.; Vachon, Celine M.
Mediterranean Diet and Breast Density in the Minnesota Breast Cancer Family Study NUTRITION AND CANCER-AN INTERNATIONAL JOURNAL English Article
MAMMOGRAPHIC PARENCHYMAL PATTERNS; POSTMENOPAUSAL WOMEN; FOLLOW-UP; RISK; COHORT; POPULATION; ESTROGEN; SURVIVAL; TRIAL; FAT Mediterranean populations' lower breast cancer incidence has been attributed to a traditional Mediterranean diet, but few studies have quantified Mediterranean dietary pattern intake in relation to breast cancer. We examined the association of a Mediterranean diet scale (MDS) with mammographic breast density as a surrogate marker for breast cancer risk. Participants completed a dietary questionnaire and provided screening mammograms for breast density assessment using a computer-assisted method. Among 1,286 women, MDS was not clearly associated with percent density in multivariate linear regression analyses. Because of previous work suggesting dietary effects limited to smokers, we conducted stratified analyses and found MDS and percent density to be significantly, inversely associated among current smokers (beta = -1.68, P = 0.002) but not among nonsmokers (beta = -0.08, P = 0.72; P for interaction = 0.008). Our results confirm a previous suggestion that selected dietary patterns may be protective primarily in the presence of procarcinogenic compounds such as those found in tobacco smoke. [Tseng, Marilyn] Fox Chase Canc Ctr, Div Populat Sci, Philadelphia, PA 19111 USA; [Sellers, Thomas A.] H Lee Moffitt Canc Ctr & Res Inst, Div Canc Prevent & Control, Tampa, FL USA; [Vierkant, Robert A.] Mayo Clin, Coll Med, Dept Hlth Sci Res, Div Biostat, Rochester, MN USA; [Kushi, Lawrence H.] Kaiser Permanente, Div Res, Oakland, CA USA; [Vachon, Celine M.] Mayo Clin, Coll Med, Dept Hlth Sci Res, Div Epidemiol, Rochester, MN USA Fox Chase Cancer Center; H Lee Moffitt Cancer Center & Research Institute; Mayo Clinic; Kaiser Permanente; Mayo Clinic Tseng, M (corresponding author), Fox Chase Canc Ctr, Div Populat Sci, 333 Cottman Ave, Philadelphia, PA 19111 USA. m_tseng@fccc.edu Vierkant, Robert/AAP-4927-2020; Tseng, Marilyn/B-9334-2016 Kushi, Lawrence/0000-0001-9136-1175; Tseng, Marilyn/0000-0002-9969-9055; Vierkant, Robert/0000-0001-6242-5221
National Institutes of Health [5 R03 CA097779-02] National

Institutes of Health(United States Department of Health & Human ServicesNational Institutes of Health (NIH) - USA) The authors thank Ms. Fang-Fang Wu for her work in reading mammograms and estimation of mammographic percent density. This work was supported by Grant 5 R03 CA097779-02 from the National Institutes of Health. 39 18 19 0 4 ROUTLEDGE JOURNALS, TAYLOR & FRANCIS LTD ABINGDON 2-4 PARK SQUARE, MILTON PARK, ABINGDON OX14 4RN, OXON, ENGLAND 0163-5581 1532-7914 NUTR CANCER Nutr. Cancer 2008 60 6
703 709 10.1080/01635580802233991
http://dx.doi.org/10.1080/01635580802233991 7
Oncology; Nutrition & Dietetics Science Citation Index Expanded (SCI-EXPANDED) Oncology; Nutrition & Dietetics
389QI 19005969 Green Submitted, Green Accepted
2025-06-24 WOS:000262109200001
J Gulle, BT; Kiran, P; Celik, SG; Varol, ZS; Siyve, N; Emecen, AN; Duzel, H Gulle, Bugra Taygun; Kiran, Pinar; Celik, Saadet Goksu; Varol, Zeynep Sedef; Siyve, Neslisah; Emecen, Ahmet Naci; Duzel, Hilal Awareness and acceptance of human papillomavirus vaccine in the Middle East: A systematic review, meta-analysis, and meta-regression of 159 studies EPIDEMIOLOGY & INFECTION English
Article human papillomavirus; vaccine acceptance; vaccine awareness; Middle East; meta-analysis; meta-regression CERVICAL-CANCER; HPV-VACCINATION; UNIVERSITY-STUDENTS; ADOLESCENT GIRLS; RISK-FACTORS; WOMENS KNOWLEDGE; VIRUS INFECTION; FAMILY PHYSICIANS; MOTHERS KNOWLEDGE; NURSING-STUDENTS
Cervical cancer, closely linked to human papillomavirus (HPV) infection, is a major global health concern. Our study aims to fill the gap in understanding HPV vaccine awareness and acceptance in the Middle East, where national immunization programs are often lacking and cultural perceptions hinder acceptance. This systematic review and meta-analysis adhered to Preferred Reporting Items for Systematic Reviews and Meta-Analyses guidelines. A comprehensive literature search across several databases was conducted on 5 September 2023. We included quantitative studies on HPV vaccine awareness and acceptance in Middle Eastern countries. Data extraction and quality assessment were conducted independently by multiple reviewers to ensure accuracy. Statistical analyses, including subgroup analyses, were performed using R to calculate pooled estimates, assess heterogeneity, and publication bias. We reviewed 159 articles from 15 Middle Eastern countries, focusing on 93,730 participants, predominantly female and healthcare workers. HPV vaccine awareness was found to be 41.7% (95% CI 37.4%-46.1%), with higher awareness among healthcare workers. The pooled acceptance rate was 45.6% (95% CI 41.3%-50.1%), with similar rates between healthcare and non-healthcare workers. Our study highlights the critical need for increased HPV vaccine awareness and acceptance in the Middle East, emphasizing the importance of integrating the vaccine into national immunization programs and addressing cultural and religious factors to improve public health outcomes. [Gulle, Bugra Taygun; Kiran, Pinar; Emecen, Ahmet Naci] Dokuz Eylul Univ, Fac Med, Dept Publ Hlth, Div Epidemiol, Izmir, Turkiye; [Celik, Saadet Goksu; Siyve, Neslisah] Dokuz Eylul Univ, Izmir, Turkiye; [Varol, Zeynep Sedef] Izmir Prov Hlth Directorate, Communicable Dis Unit, Izmir, Turkiye; [Duzel, Hilal] Izmir Kemalpasa Dist Hlth Directorate, Publ Hlth Dept, Izmir, Turkiye Dokuz Eylul

University; Dokuz Eylul University; Ministry of Health - Turkey
Gulle, BT (corresponding author), Dokuz Eylul Univ, Fac Med, Dept Publ Hlth, Div Epidemiol, Izmir, Turkiye.
bugrataygun.gulle@deu.edu.trDüzel, Hilal/AAS-1644-2020; Şiyve, Neslişah/AAH-3218-2020; varol, zeynep/LDF-6550-2024; Kıran, Pınar/JEZ-3221-2023; Gülle, Buğra Taygun/AAY-4060-2020; Celik, Saadet Goksu/GYA-5781-2022; Emecen, Ahmet Naci/AAV-7383-2020
Emecen, Ahmet Naci/0000-0003-3995-0591; KIRAN, PINAR/0000-0003-4247-4098 191 0 0 21 21
CAMBRIDGE UNIV PRESS CAMBRIDGE EDINBURGH BLDG, SHAFTESBURY RD, CB2 8RU CAMBRIDGE, ENGLAND 0950-2688 1469-4409
EPIDEMIOL INFECT Epidemiol. Infect. DEC 10 2024
152 e165
10.1017/S0950268824001596
http://dx.doi.org/10.1017/S0950268824001596 13
Public, Environmental & Occupational Health; Infectious Diseases Science Citation Index Expanded (SCI-EXPANDED)
Public, Environmental & Occupational Health; Infectious Diseases O9R3P 39655623 gold 2025-06-24
WOS:001374406900001
J Jarosz, E; Gugushvili, A Jarosz, Ewa; Gugushvili, Alexi Parental education, health literacy and children's adult body height JOURNAL OF BIOSOCIAL SCIENCE English Article
Body height; Parental education; Functional literacy
LABOR-MARKET; BIRTH-WEIGHT; FOLLOW-UP; WOMEN; MORTALITY; COUNTRIES; STATURE; INEQUALITIES; ASSOCIATION; MIGRATION Human anthropometric traits, while significantly determined by genetic factors, are also affected by an individual's early life environment. An adult's body height is a valid indicator of their living conditions in childhood. Parental education has been shown to be one of the key covariates of individuals' health and height, both in childhood and adulthood. Parental functional literacy has been demonstrated to be another important determinant of child health, but this has largely been overlooked in studies on height. The objective of this study was to analyse the associations between parents' education, their functional literacy and their children's adult body height. The study used data for 39,240 individuals from the 2016 wave of the nationally representative Life in Transition Survey (LITS) conducted in 34 countries in Southern and Eastern Europe, the Middle East and Central Asia. Using linear and Poisson models, regression adjustment treatment estimators and multilevel mixed-effects linear regressions, the study analysed the links between mother's and father's educational attainment, parental functional literacy, measured by the number of books in the childhood home, and children's adult height. The models also included other individual and contextual covariates of height. The results demonstrated that mother's educational attainment and parental functional literacy have independent associations with children's adult body height. Sufficient literacy skills of the parent may have a positive effect on children's growth even if parental education is low. These associations remained significant across time. The study also provides evidence of a widening of the height gap for men born in the period just before and after systemic transition in post-socialist societies, which may suggest an increase in social differences in early living standards. [Jarosz, Ewa] Polish Acad Sci, Inst Philosophy & Sociol, Warsaw, Poland; [Jarosz, Ewa] Univ

Oxford, Ctr Time Use Res, Dept Sociol, Oxford, England; [Gugushvili, Alexi] Erasmus Univ, Dept Publ Adm & Sociol, Rotterdam, Netherlands; [Gugushvili, Alexi] Univ Oxford, Dept Social Policy & Intervent, Oxford, England   Polish Academy of Sciences; Institute of Philosophy & Sociology of the Polish Academy of Sciences; University of Oxford; Erasmus University Rotterdam; Erasmus University Rotterdam - Excl Erasmus MC; University of Oxford   Jarosz, E (corresponding author), Polish Acad Sci, Inst Philosophy & Sociol, Warsaw, Poland.; Jarosz, E (corresponding author), Univ Oxford, Ctr Time Use Res, Dept Sociol, Oxford, England.   ejarosz@ifispan.waw.pl
70   19   19   0   13   CAMBRIDGE UNIV PRESS NEW YORK   32 AVENUE OF THE AMERICAS, NEW YORK, NY 10013-2473 USA   0021-9320   1469-7599   J BIOSOC SCI   J. Biosoc. Sci.   SEP   2020   52   5   696   718   PII S0021932019000737   10.1017/S0021932019000737   http://dx.doi.org/10.1017/S0021932019000737   23
Demography; Public, Environmental & Occupational Health; Social Sciences, Biomedical Social Science Citation Index (SSCI)
Demography; Public, Environmental & Occupational Health; Biomedical Social Sciences   OA1QJ 31722763   Green Published
2025-06-24 WOS:000577568800005
J   Dey, S; Soliman, AS; Hablas, A; Seifeldin, IA; Ismail, K; Ramadan, M; El-Hamzawy, H; Wilson, ML; Banerjee, M; Boffetta, P; Harford, J; Merajver, SD   Dey, Subhojit; Soliman, Amr S.; Hablas, Ahmad; Seifeldin, Ibrahim A.; Ismail, Kadry; Ramadan, Mohamed; El-Hamzawy, Hesham; Wilson, Mark L.; Banerjee, Mousumi; Boffetta, Paolo; Harford, Joe; Merajver, Sofia D.   Urban-rural differences in breast cancer incidence by hormone receptor status across 6 years in Egypt
BREAST CANCER RESEARCH AND TREATMENT   English
Article   Breast cancer incidence; Hormone receptor status; Mammary stem cells; Xenoestrogens; Egypt   PERSISTENT ORGANIC POLLUTANTS; ESTROGEN-RECEPTOR; RISK-FACTORS; ENVIRONMENTAL-POLLUTANTS; BISPHENOL-A; STEM-CELLS; EPIDEMIOLOGY; TUMOR; IDENTIFICATION; POPULATION
Breast cancer incidence is higher in developed countries with higher rates of estrogen receptor positive (ER+) tumors. ER+ tumors are caused by estrogenic exposures although known exposures explain approximately 50% of breast cancer risk. Unknown risk factors causing high breast cancer incidence exist that are estrogenic and development-related. Xenoestrogens are such risk factors but are difficult to study since developed countries lack unexposed populations. Developing countries have urban-rural populations with differential exposure to xenoestrogens. This study assessed urban-rural breast cancer incidence classified by hormone receptor status using data from Gharbiah population-based cancer registry in Egypt from 2001 to 2006. Urban ER+ incidence rate (per 100,000 women) was 2-4 times (IRR = 3.36, 95% CI = 4.84, 2.34) higher than rural incidence rate. ER-incidence rate was 2-3 times (IRR = 1.86, 95% CI = 2.38, 1.45) higher in urban areas than in rural areas. Our findings indicate that urban women may probably have a higher exposure to xenoestrogens. [Dey, Subhojit; Soliman, Amr S.; Wilson, Mark L.] Univ Michigan, Sch Publ Hlth, Dept Epidemiol, Ann Arbor, MI 48109 USA; [Hablas, Ahmad; Ismail, Kadry] Gharbiah Canc Soc, Tanta, Gharbiah, Egypt; [Seifeldin, Ibrahim A.; Ramadan, Mohamed; El-Hamzawy, Hesham] Tanta Canc Ctr, Tanta, Gharbiah, Egypt; [Banerjee, Mousumi] Univ Michigan, Sch

Publ Hlth, Dept Biostat, Ann Arbor, MI 48109 USA; [Boffetta, Paolo] Int Agcy Res Canc, F-69372 Lyon, France; [Harford, Joe] NCI, Off Int Affairs, Bethesda, MD 20892 USA; [Merajver, Sofia D.] Univ Michigan, Ctr Comprehens Canc, Ann Arbor, MI 48109 USA
University of Michigan System; University of Michigan; Egyptian Knowledge Bank (EKB); Tanta University; University of Michigan System; University of Michigan; World Health Organization; International Agency for Research on Cancer (IARC); National Institutes of Health (NIH) - USA; NIH National Cancer Institute (NCI); University of Michigan System; University of Michigan   Soliman, AS (corresponding author), Univ Michigan, Sch Publ Hlth, Dept Epidemiol, 109 Observ St, Ann Arbor, MI 48109 USA.
asoliman@umich.edu   Boffetta, Paolo/AAI-7767-2021
Harford, Joe Bryan/0000-0002-6681-6315 Middle East Cancer Consortium, National Cancer Institute, Bethesda [R25 CA112383, R03 CA117350, 5 P30 CA46592]; Burroughs Wellcome Fund; Breast Cancer Research Foundation; Department of Epidemiology, University of Michigan School of Public Health; Rackham Graduate School of the University of Michigan; National Cancer Institute [P30CA046592] Funding Source: NIH RePORTERMiddle East Cancer Consortium, National Cancer Institute, Bethesda(United States Department of Health & Human ServicesNational Institutes of Health (NIH) - USANIH National Cancer Institute (NCI)); Burroughs Wellcome Fund(Burroughs Wellcome Fund); Breast Cancer Research Foundation; Department of Epidemiology, University of Michigan School of Public Health(University of Michigan System); Rackham Graduate School of the University of Michigan; National Cancer Institute(United States Department of Health & Human ServicesNational Institutes of Health (NIH) - USANIH National Cancer Institute (NCI))   We are grateful to Dr. Hoda Gad, Mr. Khaled Daboos and other personnel of Tanta Cancer Center and Gharbiah Cancer Society for the valuable assistance they provided for this project. The authors declare that they have no commercial or other associations that might pose a conflict of interest in connection with this article. Funding This work was supported by the Middle East Cancer Consortium, National Cancer Institute, Bethesda [R25 CA112383, R03 CA117350,5 P30 CA46592], the Burroughs Wellcome Fund [SDM], and the Breast Cancer Research Foundation [SDM]. Block Grant of the Department of Epidemiology, University of Michigan School of Public Health; and the Travel Grant of the Rackham Graduate School of the University of Michigan to [S. Dey].
50   49   51   0   3   SPRINGER   NEW YORK   233 SPRING ST, NEW YORK, NY 10013 USA 0167-6806   BREAST CANCER RES TR   Breast Cancer Res. Treat.   FEB   2010 120   1
149   160   10.1007/s10549-009-0427-9   http://dx.doi.org/10.1007/s10549-009-0427-9
12   Oncology   Science Citation Index Expanded (SCI-EXPANDED) Oncology   545OK 19548084   Green Accepted
2025-06-24 WOS:000273743500016
J   Tsui, J; Saraiya, M; Thompson, T; Dey, A; Richardson, L
Tsui, Jennifer; Saraiya, Mona; Thompson, Trevor; Dey, Achintya; Richardson, Lisa   Cervical cancer screening among foreign-born women by birthplace and duration in the United States   JOURNAL OF WOMENS HEALTH
English   Article; Proceedings Paper 14th Annual Congress on Womens Health JUN 03-06, 2006 Hilton Head Isl, SC
HUMAN-PAPILLOMAVIRUS VACCINE; PACIFIC ISLANDER WOMEN; HEALTH-INSURANCE; CITIZENSHIP STATUS; BREAST; AMERICAN;

IMMIGRANTS; BEHAVIORS; ATTITUDES; ACCULTURATION Objective: Mortality rates for cervical cancer have increased among foreign-born women in the United States in the last two decades. Previous research indicates that rates of Pap testing are lower among foreign-born women than in U.S.-born women. This study identifies screening rates among foreign-born women by birthplace and duration in the United States. Methods: We used data from 4 years (1998, 1999, 2000, 2003) of the National Health Interview Survey (NHIS) to estimate Pap testing rates by birthplace (Mexico, Central America, Caribbean, South America, Europe, Russia, Africa, Middle East, India, Asia, and Southeast Asia) and percent of lifetime spent in the United States for women aged >= 18 years (n = 70,775). Rates were age standardized to the 2000 U. S. population. Results: After adjusting for demographic characteristics and health indicators, we found that 18.6% (95% CI 16.7, 20.6) of recent immigrants (<25% of lifetime in the United States) and 9.9% (95% CI 9.0, 10.8) of established immigrants (>= 25% of lifetime in the United States) never received a Pap test in their lifetime compared with 5.8% (95% CI 5.5, 6.1) of U.S.-born women. Adjusted prevalence of never receiving a Pap test was highest among women from Asia, Southeast Asia, and India (19.6%), South America (12.7%), Mexico (11.2%), Caribbean (11.0%), Europe (9.9%), and Central America (9.2%). Conclusions: Significant differences exist in rates of screening for cervical cancer between foreign-born groups by birthplace and by duration in the United States. Nationally and locally funded screening programs may benefit from these findings in developing screening strategies for foreign-born women. [Tsui, Jennifer] Assoc Sch Publ Hlth, Los Angeles, CA USA; [Saraiya, Mona; Thompson, Trevor; Richardson, Lisa] Univ Calif Los Angeles, Sch Publ Hlth, Dept Hlth Serv, Los Angeles, CA 90024 USA; [Dey, Achintya] Ctr Dis Control & Prevent, Natl Ctr Hlth Stat, Coordinating Ctr Hlth Informat & Serv, Hyattsville, MD 20782 USA University of California System; University of California Los Angeles; Centers for Disease Control & Prevention - USA; CDC National Center for Health Statistics (NCHS) Saraiya, M (corresponding author), Ctr Dis Control & Prevent, Div Canc Prevent & Control, Epidemiol & Appl Res Branch, 4770 Buford Highway MS K-55, Atlanta, GA 30341 USA.
msaraiya@cdc.gov Tsui, Jennifer/ABE-3743-2020Richardson, Lisa/0000-0002-9555-7674 53 95 118
0 18 MARY ANN LIEBERT, INC NEW ROCHELLE 140 HUGUENOT STREET, 3RD FL, NEW ROCHELLE, NY 10801 USA 1540-9996
1931-843X J WOMENS HEALTH J. Womens Health DEC 2007
16 10 1447 1457
10.1089/jwh.2006.0279
http://dx.doi.org/10.1089/jwh.2006.0279 11
Public, Environmental & Occupational Health; Medicine, General & Internal; Obstetrics & Gynecology; Women's Studies
Science Citation Index Expanded (SCI-EXPANDED); Social Science Citation Index (SSCI); Conference Proceedings Citation Index - Science (CPCI-S); Conference Proceedings Citation Index - Social Science & Humanities (CPCI-SSH) Public, Environmental & Occupational Health; General & Internal Medicine; Obstetrics & Gynecology; Women's Studies 248MW 18062760
2025-06-24 WOS:000252158400008
J Petimar, J; Smith-Warner, SA; Fung, TT; Rosner, B; Chan, AT; Hu, FB; Giovannucci, EL; Tabung, FK Petimar, Joshua; Smith-Warner, Stephanie A.; Fung, Teresa T.; Rosner,

Bernard; Chan, Andrew T.; Hu, Frank B.; Giovannucci, Edward L.; Tabung, Fred K. Recommendation-based dietary indexes and risk of colorectal cancer in the Nurses' Health Study and Health Professionals Follow-up Study AMERICAN JOURNAL OF CLINICAL NUTRITION English Article colorectal cancer; dietary index; cohort studies; DASH diet; Mediterranean diet; Alternative Healthy Eating Index POLYUNSATURATED FATTY-ACIDS; DASH-STYLE DIET; VITAMIN-D; MEDITERRANEAN DIET; REGRESSION-MODELS; QUALITY INDEXES; POOLED ANALYSIS; CALCIUM INTAKE; COLON-CANCER; RED MEAT Background: Many dietary indexes exist for chronic disease prevention, but the optimal dietary pattern for colorectal cancer prevention is unknown. Objective: We sought to determine associations between adherence to various dietary indexes and incident colorectal cancer in 2 prospective cohort studies. Design: We followed 78,012 women in the Nurses' Health Study and 46,695 men in the Health Professionals Follow-up Study from 1986 and 1988, respectively, until 2012. We created dietary index scores for the Dietary Approaches to Stop Hypertension (DASH) diet, Alternative Mediterranean Diet (AMED), and Alternative Healthy Eating Index-2010 (AHEI-2010) and used Cox regression to estimate HRs and 95% CIs for risk of colorectal cancer (CRC) and by anatomic subsite. We also conducted latency analyses to examine associations between diet and CRC risk during different windows of exposure. We conducted analyses in men and women separately, and subsequently pooled these results in a random-effects meta-analysis. Results: We documented 2690 colorectal cancer cases. Pooled multivariable HRs for colorectal cancer risk comparing the highest to lowest quintile of diet scores were 0.89 (95% CI: 0.74, 1.08; P-trend = 0.10) for DASH, 0.89 (95% CI: 0.73, 1.10; P-trend = 0.31) for AMED, and 0.95 (95% CI: 0.83, 1.09; P-trend = 0.56) for AHEI-2010 (P-heterogeneity >= 0.07 for all). In sex-specific analyses, we observed stronger associations in men for all dietary indexes (DASH: multivariable HR = 0.81, 95% CI: 0.66, 0.98; P-trend = 0.003; AMED: multivariable HR = 0.80, 95% CI: 0.65, 0.98; P-trend = 0.02; AHEI-2010: multivariable HR = 0.88, 95% CI: 0.72, 1.07; P-trend = 0.04) than in women (multivariable HRs range from 0.98 to 1.01). Conclusions: Adherence to the DASH, AMED, and AHEI-2010 diets was inversely associated with colorectal cancer risk in men. These diets were not associated with colorectal cancer risk in women. [Petimar, Joshua; Smith-Warner, Stephanie A.; Fung, Teresa T.; Hu, Frank B.; Giovannucci, Edward L.; Tabung, Fred K.] Harvard TH Chan Sch Publ Hlth, Dept Nutr, Boston, MA 02115 USA; [Petimar, Joshua; Smith-Warner, Stephanie A.; Hu, Frank B.; Giovannucci, Edward L.; Tabung, Fred K.] Harvard TH Chan Sch Publ Hlth, Dept Epidemiol, Boston, MA 02115 USA; [Rosner, Bernard] Harvard TH Chan Sch Publ Hlth, Dept Biostat, Boston, MA USA; [Fung, Teresa T.] Simmons Coll, Dept Nutr, Boston, MA 02115 USA; [Rosner, Bernard; Chan, Andrew T.; Hu, Frank B.; Giovannucci, Edward L.] Brigham & Womens Hosp, Dept Med, Channing Div Network Med, 75 Francis St, Boston, MA 02115 USA; [Chan, Andrew T.] Massachusetts Gen Hosp, Clin & Translat Epidemiol Unit, Boston, MA 02114 USA; [Chan, Andrew T.] Harvard Med Sch, Boston, MA USA; [Chan, Andrew T.] Massachusetts Gen Hosp, Div Gastroenterol, Boston, MA 02114 USA; [Tabung, Fred K.] Ohio State Univ, Coll Med, Dept Internal Med, Div Med Oncol, Columbus, OH 43210 USA Harvard University; Harvard T.H. Chan School of Public Health; Harvard University; Harvard T.H. Chan School of Public Health; Harvard

University; Harvard T.H. Chan School of Public Health; Simmons University; Harvard University; Harvard University Medical Affiliates; Brigham & Women's Hospital; Harvard University; Harvard University Medical Affiliates; Massachusetts General Hospital; Harvard University; Harvard Medical School; Harvard University; Harvard University Medical Affiliates; Massachusetts General Hospital; University System of Ohio; Ohio State University
Tabung, FK (corresponding author), Harvard TH Chan Sch Publ Hlth, Dept Nutr, Boston, MA 02115 USA.; Tabung, FK (corresponding author), Harvard TH Chan Sch Publ Hlth, Dept Epidemiol, Boston, MA 02115 USA.; Tabung, FK (corresponding author), Ohio State Univ, Coll Med, Dept Internal Med, Div Med Oncol, Columbus, OH 43210 USA. fred.tabung@osumc.edu Tabung, Fred/AAQ-7823-2021; Hu, Frank/C-1919-2013; Chan, Andrew/ADM-9271-2022; Giovannucci, Edward/ADE-8028-2022 Fung, Teresa/0000-0002-0159-482X National Cancer Institute [K99CA207736]; NIH [UM1CA167552, P01 CA55075, UM1CA186107, P01 CA87969] National Cancer Institute(United States Department of Health & Human ServicesNational Institutes of Health (NIH) - USANIH National Cancer Institute (NCI)); NIH(United States Department of Health & Human ServicesNational Institutes of Health (NIH) - USA) This work was supported by National Cancer Institute grant #K99CA207736 to FKT. The HPFS and NHS cohorts are supported by NIH grants UM1CA167552 (HPFS), P01 CA55075 (HPFS), UM1CA186107 (NHS), and P01 CA87969 (NHS). 70 53 59 1 13 OXFORD UNIV PRESS OXFORD GREAT CLARENDON ST, OXFORD OX2 6DP, ENGLAND 0002-9165 1938-3207 AM J CLIN NUTR Am. J. Clin. Nutr. NOV 2018 108 5 1092 1103 10.1093/ajcn/nqy171 http://dx.doi.org/10.1093/ajcn/nqy171 12 Nutrition & Dietetics Science Citation Index Expanded (SCI-EXPANDED) Nutrition & Dietetics HH9XK 30289433 Green Published, Bronze 2025-06-24 WOS:000456094800020
J Apostolou, P; Fostira, F; Papamentzelopoulou, M; Michelli, M; Panopoulos, C; Fountzilas, G; Konstantopoulou, I; Voutsinas, GE; Yannoukakos, D Apostolou, Paraskevi; Fostira, Florentia; Papamentzelopoulou, Myrto; Michelli, Maria; Panopoulos, Christos; Fountzilas, George; Konstantopoulou, Irene; Voutsinas, Gerassimos E.; Yannoukakos, Drakoulis
CHEK2 c.1100delC allele is rarely identified in Greek breast cancer cases CANCER GENETICS English Article 1100delC; BRCA; breast cancer; CHEK2; hereditary TUMOR CHARACTERISTICS; CHEK2-ASTERISK-1100DELC VARIANT; HEREDITARY BREAST; GERMLINE MUTATION; CHK2 KINASE; EARLY-ONSET; SUSCEPTIBILITY; 1100DELC; FAMILIES; GENE The CHEK2 gene encodes a protein kinase that plays a crucial role in maintenance of genomic integrity and the DNA repair mechanism. CHEK2 germline mutations are associated with increased risk of breast cancer and other malignancies. From a clinical perspective, the most significant mutation identified is the c.1100delC mutation, which is associated with an approximately 25% lifetime breast cancer risk. The distribution of this mutation shows wide geographical variation; it is more prevalent in the Northern European countries and less common, or even absent, in Southern Europe. In order to estimate the frequency of the CHEK2 c.1100delC mutation in Greek breast cancer patients, we genotyped 2,449 patients (2,408 females and 41 males), which was the largest series ever tested for c.1100delC. The mean age of female and male breast cancer diagnosis was 49 and 59 years, respectively. All

patients had previously tested negative for the Greek BRCA1 founder and recurrent mutations. The CHEK2 c.1100delC mutation was detected in 0.16% (4 of 2,408) of females, all of whom were diagnosed with breast cancer before the age of 50 years. Only one c.1100delC carrier was reported with breast cancer family history. The present study indicates that the CHEK2 c.1100delC mutation does not contribute substantially to hereditary breast cancer in patients of Greek descent. [Apostolou, Paraskevi; Fostira, Florentia; Papamentzelopoulou, Myrto; Michelli, Maria; Konstantopoulou, Irene; Yannoukakos, Drakoulis] Natl Ctr Sci Res Demokritos, INRASTES, Mol Diagnost Lab, Athens, Greece; [Apostolou, Paraskevi] Univ Athens, Fac Biol, Dept Genet & Biotechnol, Lab Human Genet, Athens 11528, Greece; [Panopoulos, Christos] Agios Savvas Anticanc Hosp, Dept Med Oncol 2, Athens, Greece; [Fountzilas, George] Aristotle Univ Thessaloniki, Sch Med, Papageorgiou Hosp, Dept Med Oncol, GR-54006 Thessaloniki, Greece; [Voutsinas, Gerassimos E.] Natl Ctr Sci Res Demokritos, Inst Biosci & Applicat, Lab Environm Mutagenesis & Carcinogenesis, Athens, Greece National Centre of Scientific Research "Demokritos"; National & Kapodistrian University of Athens; Papageorgiou Hospital; Aristotle University of Thessaloniki; National Centre of Scientific Research "Demokritos"

Yannoukakos, D (corresponding author), Natl Ctr Sci Res Demokritos, INRASTES, Mol Diagnost Lab, Athens, Greece.

yannouka@gmail.com Konstantopoulou, Irene/Y-3074-2018; Papamentzelopoulou, Myrto/ABE-6985-2021; Fountzilas, George/ABF-2139-2020; Fostira, Florentia/Z-5730-2019; Konstantopoulou, Irene/B-5309-2009 Konstantopoulou, Irene/0000-0002-0470-0309; Fountzilas, George/0000-0001-8045-2186; michelli, maria/0000-0002-2755-1721; Yannoukakos, Drakoulis/0000-0001-7509-3510 research program ARISTEIA; European Union (European Social Fund, ESF) [ARISTEIA 39]; Greek national funds through the Operational Program "Education and Lifelong Learning" of the National Strategic Reference Framework (NSRF)-Research Funding Program of the General Secretariat for Research & Technology (ARISTEIA 39)

research program ARISTEIA; European Union (European Social Fund, ESF)(European Union (EU)European Social Fund (ESF)); Greek national funds through the Operational Program "Education and Lifelong Learning" of the National Strategic Reference Framework (NSRF)-Research Funding Program of the General Secretariat for Research & Technology (ARISTEIA 39) We thank the patients for their participation in this study. P.A. acknowledges support from the research program ARISTEIA. This research was co-financed by the European Union (European Social Fund, ESF) (ARISTEIA 39, P. BROCA) and Greek national funds through the Operational Program "Education and Lifelong Learning" of the National Strategic Reference Framework (NSRF)-Research Funding Program of the General Secretariat for Research & Technology (ARISTEIA 39, P. BROCA), which invests in knowledge society through the ESF. 37

14 14 0 6 ELSEVIER SCIENCE INC NEW YORK 360 PARK AVE SOUTH, NEW YORK, NY 10010-1710 USA 2210-7762 2210-7770

CANCER GENET-NY Cancer Genet. APR 2015 208 4

129 134

10.1016/j.cancergen.2015.02.006

http://dx.doi.org/10.1016/j.cancergen.2015.02.006

6 Oncology; Genetics & Heredity Science Citation Index Expanded (SCI-EXPANDED) Oncology; Genetics & Heredity

CI6YZ 25835597 2025-06-24
WOS:000354911400004
J Aguilera-Buenosvinos, I; Martínez-González, MA; Zazpe, I; Romanos-Nanclares, A; Sánchez-Bayona, R; Toledo, E
Aguilera-Buenosvinos, Inmaculada; Angel Martinez-Gonzalez, Miguel; Zazpe, Itziar; Romanos-Nanclares, Andrea; Sanchez-Bayona, Rodrigo; Toledo, Estefania Associations between overall, healthful, and unhealthful low-fat dietary patterns and breast cancer risk in a Mediterranean cohort: The SUN project
NUTRITION English Article
Breast cancer; Cohort; Low -fat diet; Dietary patterns; Prospective cohort study; Epidemiology FOOD-FREQUENCY QUESTIONNAIRE; CARDIOVASCULAR-DISEASE; FOLLOW-UP; VALIDATION; NUTRITION; MORTALITY; INSULIN; INDEX; ACIDS Objectives: Dietary patterns may have a greater influence on human health than individual foods or nutrients, and they are also of substantial interest in the field of breast cancer prevention. Beyond the adequate balance of macronutrients, evidence indicates that the quality of macronutrient sources may play an important role in health outcomes. We sought to examine the relationship between healthful and unhealthful low-fat dietary patterns in relation to breast cancer. Methods: We used observational data from a Mediterranean cohort study (the Seguimiento Universidad de Navarra project). We prospectively followed 10 930 middle-aged women initially free of breast cancer during a median follow-up of 12.1 y. We calculated an overall, an unhealthful, and a healthful low-fat diet score, based on a previously validated 136-item food frequency questionnaire and grouped participants into ter -tiles. Incident breast cancer-overall and stratified by menopausal status-was the primary outcome. It was self-reported by participants and confirmed based on medical reports or consultation of the National Death Index. We used multivariable Cox regression models adjusted for potential confounders. Results: During 123 297 person-years of follow-up, 150 cases of incident breast cancer were confirmed. No signifi-cant associations were observed for overall or premenopausal breast cancer. For postmenopausal women, we observed a significant association for moderate adherence to the unhealthful low-fat dietary score and postmeno-pausal breast cancer (comparing tertile 2 to tertile 1; hazard ratio = 2.18; 95% confidence interval, 1.15-4.13). Conclusions: In conclusion, no clear associations were observed, although more research is needed to address the association between an unhealthful dietary pattern and postmenopausal breast cancer risk. (c) 2023 The Authors. Published by Elsevier Inc. This is an open access article under the CC BY-NC-ND license (http://creativecommons.org/licenses/by-nc-nd/4.0/)
[Aguilera-Buenosvinos, Inmaculada; Angel Martinez-Gonzalez, Miguel; Zazpe, Itziar; Toledo, Estefania] Univ Navarra, Dept Prevent Med & Publ Hlth, Pamplona, Spain; [Aguilera-Buenosvinos, Inmaculada; Angel Martinez-Gonzalez, Miguel; Toledo, Estefania] IdiSNA, Navarra Inst Hlth Res, Pamplona, Spain; [Angel Martinez-Gonzalez, Miguel; Toledo, Estefania] Ctr Invest Biomed Red Area Fisiol Obes & Nutr CIB, Madrid, Spain; [Angel Martinez-Gonzalez, Miguel] Harvard Univ, Harvard TH Chan Sch Publ Hlth, Dept Nutr, Boston, MA USA; [Zazpe, Itziar] Univ Navarra, Dept Nutr & Food Sci & Physiol, Irunlarrea, Spain; [Romanos-Nanclares, Andrea] Harvard Med Sch, Brigham & Womens Hosp, Dept Med, Channing Div Network Med, Boston, MA USA; [Sanchez-Bayona, Rodrigo] Hosp Univ 12

Octubre, Med Oncol Dept, Madrid, Spain University of Navarra; University of Navarra; Harvard University; Harvard T.H. Chan School of Public Health; University of Navarra; Harvard University; Harvard University Medical Affiliates; Brigham & Women's Hospital; Harvard Medical School; Hospital Universitario 12 de Octubre Toledo, E (corresponding author), Univ Navarra, Dept Prevent Med & Publ Hlth, Pamplona, Spain.; Toledo, E (corresponding author), IdiSNA, Navarra Inst Hlth Res, Pamplona, Spain.; Toledo, E (corresponding author), Ctr Invest Biomed Red Area Fisiol Obes & Nutr CIB, Madrid, Spain. etoledo@unav.es

García, Miguel/F-9630-2010; Toledo, Estefania/H-6211-2014; Zazpe, Itziar/B-1970-2017 Aguilera Buenosvinos, Inmaculada/0000-0002-9484-7303 49 1 1 0 2

ELSEVIER SCIENCE INC NEW YORK STE 800, 230 PARK AVE, NEW YORK, NY 10169 USA 0899-9007 1873-1244 NUTRITION

Nutrition MAY 2023 109

111967 10.1016/j.nut.2022.111967

http://dx.doi.org/10.1016/j.nut.2022.111967 FEB 2023

10 Nutrition & Dietetics Science Citation Index Expanded (SCI-EXPANDED) Nutrition & Dietetics J3QR3 36738657 Green Published, hybrid 2025-06-24 WOS:001008796300001

J Khodakarami, N; Clifford, GM; Yavari, P; Farzaneh, F; Salehpour, S; Broutet, N; Bathija, H; Heideman, DAM; van Kemenade, FJ; Meijer, CJLM; Hosseini, SJ; Franceschi, S

Khodakarami, Nahid; Clifford, Gary M.; Yavari, Parvin; Farzaneh, Farah; Salehpour, Saghar; Broutet, Natalie; Bathija, Heli; Heideman, Danielle A. M.; van Kemenade, Folkert J.; Meijer, Chris J. L. M.; Hosseini, Seyed Jalil; Franceschi, Silvia

Human papillomavirus infection in women with and without cervical cancer in Tehran, Iran INTERNATIONAL JOURNAL OF CANCER English Article

human papillomavirus; prevalence; cervical cancer; Iran REPUBLIC-OF-CHINA; REGISTRY; PREVALENCE; WORLDWIDE; CITY No data exist on the population prevalence of, or risk factors for, human papillomavirus (HPV) infection in Iran or the Middle East. Cervical specimens were obtained from 825 married women aged 1859 years from the general population of Tehran, Iran and from 45 locally diagnosed invasive cervical cancers (ICC) according to the standardized protocol of the International Agency for Research on Cancer HPV Prevalence Surveys. HPV was detected and genotyped using a GP5+/6+ PCR-based assay. HPV prevalence in the general population was 7.8% (95% confidence interval: 6.09.8) (5.1% of high-risk types), with no significant variation by age. HPV positivity was significantly higher among divorced women, women in polygamous marriages and those reporting husband's absence from home for >7 nights/month. HPV16/18 accounted for 30 and 82.2% of HPV-positive women in the general population and ICC, respectively. Cervical cancer prevention policies should take into account the relatively low HPV prevalence in this population.

[Clifford, Gary M.; Franceschi, Silvia] Int Agcy Res Canc, F-69372 Lyon 08, France; [Khodakarami, Nahid; Farzaneh, Farah; Salehpour, Saghar; Hosseini, Seyed Jalil] Shahid Beheshti Univ Med Sci, Infertil & Reprod Hlth Res Ctr, Tehran, Iran; [Yavari, Parvin] Shahid Beheshti Univ Med Sci, Dept Community Med, Sch Med, Tehran, Iran; [Yavari, Parvin] Iranian Epidemiol Assoc, Tehran, Iran; [Yavari, Parvin] Shahid Beheshti Univ Med Sci, Genom Res Ctr, Tehran, Iran; [Broutet, Natalie; Bathija, Heli] World Hlth Org, Geneva, Switzerland; [Heideman, Danielle A. M.; van Kemenade,

Folkert J.; Meijer, Chris J. L. M.] Vrije Univ Amsterdam Med Ctr, Dept Pathol, Amsterdam, Netherlands World Health Organization; International Agency for Research on Cancer (IARC); Shahid Beheshti University Medical Sciences; Shahid Beheshti University Medical Sciences; Shahid Beheshti University Medical Sciences; World Health Organization; Vrije Universiteit Amsterdam; VU UNIVERSITY MEDICAL CENTER Clifford, GM (corresponding author), Int Agcy Res Canc, 150 Cours Albert Thomas, F-69372 Lyon 08, France. clifford@iarc.fr salehpour, saghar/GMX-1727-2022; Farzaneh, Farah/AAG-4379-2020; Khodakarami, Nima/M-8601-2019; Hosseini, Jalil/AAU-9002-2021; franceschi, silvia/M-2452-2014
franceschi, silvia/0000-0003-4181-8071; Khodakarami, Nahid/0000-0001-6469-8717; Farzaneh, Farah/0000-0002-5062-4386; van Kemenade, Folkert J./0000-0002-4225-4354; Farzaneh, Farah/0000-0003-3600-1374; Hosseini, Jalil/0000-0003-3741-1543
Bill & Melinda Gates Foundation [35537]; UNDP/UNFPA/WHO/World Bank [A65119]; Association for International Cancer Research [08-0213]; Institut National du Cancer [07/3D1514/PL-89-05/NG-LC] Bill & Melinda Gates Foundation(Bill & Melinda Gates FoundationBill & Melinda Gates Foundation Grand Challenges Explorations InitiativeCGIAR); UNDP/UNFPA/WHO/World Bank(World Health Organization); Association for International Cancer Research; Institut National du Cancer(Institut National du Cancer (INCA) France) Grant sponsor: Bill & Melinda Gates Foundation; Grant number: 35537; Grant sponsor: UNDP/UNFPA/WHO/World Bank Special Programme of Research, Development and Research Training in Human Reproduction; Grant number: A65119; Grant sponsor: Association for International Cancer Research; Grant number: 08-0213; Grant sponsor: Institut National du Cancer; Grant number: 07/3D1514/PL-89-05/NG-LC
25 56 57 0 8 WILEY-BLACKWELL HOBOKEN 111 RIVER ST, HOBOKEN 07030-5774, NJ USA 0020-7136 INT J CANCER Int. J. Cancer JUL 15 2012 131 2
E156 E161 10.1002/ijc.26488
http://dx.doi.org/10.1002/ijc.26488 6
Oncology Science Citation Index Expanded (SCI-EXPANDED)
Oncology 946KN 22038830 2025-06-24
WOS:000304350600018
J Al-Thawadi, H; Ghabreau, L; Aboulkassim, T; Yasmeen, A; Vranic, S; Batist, G; Al Moustafa, AE Al-Thawadi, Hamda; Ghabreau, Lina; Aboulkassim, Tahar; Yasmeen, Amber; Vranic, Semir; Batist, Gerald; Al Moustafa, Ala-Eddin
Co-Incidence of Epstein-Barr Virus and High-Risk Human Papillomaviruses in Cervical Cancer of Syrian Women FRONTIERS IN ONCOLOGY English Article
Epstein-Barr virus; high-risk human papillomaviruses; cervical cancer; Syrian women; cancer phenotype BREAST-CANCER; TISSUE MICROARRAY; EPITHELIAL-CELLS; HPV TYPE-16; EBV; CARCINOGENESIS; ASSOCIATION; METASTASIS; EXPRESSION; CARCINOMA
Epstein-Barr virus (EBV) has been recently shown to be co-present with high-risk human papillomaviruses (HPVs) in human cervical cancer; thus, these oncoviruses play an important role in the initiation and/or progression of this cancer. Accordingly, our group has recently viewed the presence and genotyping distribution of high-risk HPVs in cervical cancer in Syrian women; our data pointed out that HPVs are present in 42/44 samples (95%). Herein, we aim to explore the co-prevalence of EBV and high-risk HPVs in 44 cervical cancer tissues from Syrian women using polymerase

chain reaction, immunohistochemistry, and tissue microarray analyses. We found that EBV and high-risk HPVs are co-present in 15/44 (34%) of the samples. However, none of the samples was exclusively EBV-positive. Additionally, we report that the co-expression of LMP1 and E6 genes of EBV and high-risk HPVs, respectively, is associated with poorly differentiated squamous cell carcinomas phenotype; this is accompanied by a strong and diffuse overexpression of Id-1 (93% positivity), which is an important regulator of cell invasion and metastasis. These data imply that EBV and HPVs are co-present in cervical cancer samples in the Middle East area including Syria and their co-presence is associated with a more aggressive cancer phenotype. Future investigations are needed to elucidate the exact role of EBV and HPVs cooperation in cervical carcinogenesis. [Al-Thawadi, Hamda; Vranic, Semir; Al Moustafa, Ala-Eddin] Qatar Univ, Coll Med, Doha, Qatar; [Ghabreau, Lina] Univ Aleppo, Pathol Dept, Fac Med, Aleppo, Syria; [Ghabreau, Lina; Al Moustafa, Ala-Eddin] Syrian Soc Canc, Syrian Res Canc Ctr, Aleppo, Syria; [Aboulkassim, Tahar; Yasmeen, Amber; Batist, Gerald] Sir Mortimer B Davis Jewish Hosp, Lady Davis Inst Med Res, Segal Canc Ctr, Montreal, PQ, Canada; [Batist, Gerald; Al Moustafa, Ala-Eddin] McGill Univ, Oncol Dept, Montreal, PQ, Canada; [Al Moustafa, Ala-Eddin] Qatar Univ, Coll Med, Doha, Qatar; [Al Moustafa, Ala-Eddin] Qatar Univ, Biomed Res Ctr, Doha, Qatar Qatar University; University of Aleppo; Lady Davis Institute; Jewish General Hospital - Montreal; McGill University; Qatar University; Qatar University Al Moustafa, AE (corresponding author), Qatar Univ, Coll Med, Doha, Qatar.; Al Moustafa, AE (corresponding author), Syrian Soc Canc, Syrian Res Canc Ctr, Aleppo, Syria.; Al Moustafa, AE (corresponding author), McGill Univ, Oncol Dept, Montreal, PQ, Canada.; Al Moustafa, AE (corresponding author), Qatar Univ, Coll Med, Doha, Qatar.; Al Moustafa, AE (corresponding author), Qatar Univ, Biomed Res Ctr, Doha, Qatar. aalmoustafa@qu.edu.qa ; Vranic, Semir/J-4113-2012 Ghabreau, Lina/0009-0000-5769-3459; Vranic, Semir/0000-0001-9743-7265 Qatar University [GCC-2017-002 QU/KU, QUCG-CMED-2018\2019-3] Qatar University(Qatar UniversityQatar National Research Fund (QNRF)) We would like to thank Mrs. A. Kassab for her critical reading of the manuscript. This work was supported by Qatar University grants # GCC-2017-002 QU/KU and QUCG-CMED-2018\2019-3. 37 26 28 0 4 FRONTIERS MEDIA SA LAUSANNE AVENUE DU TRIBUNAL FEDERAL 34, LAUSANNE, CH-1015, SWITZERLAND 2234-943X FRONT ONCOL Front. Oncol. JUL 2 2018 8 250
10.3389/fonc.2018.00250
http://dx.doi.org/10.3389/fonc.2018.00250 6
Oncology Science Citation Index Expanded (SCI-EXPANDED)
Oncology GL2QY 30035100 gold, Green Published
2025-06-24 WOS:000436970000004

J Huang, ZZ; Shi, Y; Bao, PP; Cai, H; Hong, Z; Ding, D; Jackson, J; Shu, XO; Dai, Q Huang, Zhezhou; Shi, Yan; Bao, Pingping; Cai, Hui; Hong, Zhen; Ding, Ding; Jackson, James; Shu, Xiao-Ou; Dai, Qi Associations of dietary intake and supplement use with post-therapy cognitive recovery in breast cancer survivors BREAST CANCER RESEARCH AND TREATMENT English Article
Breast cancer survivor; Cognition; Diet; Supplement
ALZHEIMERS-DISEASE; MEDITERRANEAN DIET; PATTERNS; CHINESE; RISK; CHEMOTHERAPY; PERFORMANCE; DEMENTIA; DECLINE Cognitive

impairment induced by cancer therapy is common and can be long lasting after completion of therapy. Little is known on factors that influence recovery from the impairment. We evaluated the associations of dietary intake and supplement use with post-therapy cognitive recovery in a large cohort of breast cancer survivors. This study included 1047 breast cancer patients aged 20-75 who were recruited to the Shanghai Breast Cancer Survival Study between 2002 and 2006 at approximately 6.5 months post-cancer diagnosis. Two cognitive assessments covering immediate memory, delayed memory, verbal fluency, and attention, were conducted at 18 and 36 months post-diagnosis. We used food frequency questionnaire to collect information on their dietary intake and supplement use between 18 and 36 months post-diagnosis. Linear regression models were used to examine the associations of dietary intake and supplement use with mean cognitive scores at 36 months post-diagnosis and with differences in cognitive scores between 18 and 36 months post-diagnosis. Higher vegetable, fruit and fish intake, supplementation with vitamin B and vitamin E, and tea drinking were associated with higher cognitive scores, while alcohol drinking was associated with lower cognitive scores at 36 months post-diagnosis. Vegetable intake was positively associated with improvement in verbal fluency, while tea drinking and fish oil supplementation were associated with greater improvements in delayed memory between 18 and 36 months post-diagnosis. Our results indicate that higher vegetable intake, tea drinking, and fish oil supplementation may help post-therapy cognitive recovery for cancer patients. [Huang, Zhezhou; Shi, Yan; Bao, Pingping] Shanghai Municipal Ctr Dis Control & Prevent, Div Noncommunicable Dis & Injury, 1380 Zhongshan West Rd, Shanghai 200336, Peoples R China; [Cai, Hui; Shu, Xiao-Ou; Dai, Qi] Vanderbilt Univ, Sch Med, Dept Med, Div Epidemiol, Nashville, TN 37203 USA; [Hong, Zhen; Ding, Ding] Fudan Univ, Huashan Hosp, Dept Neurol, Shanghai 200040, Peoples R China; [Jackson, James] Vanderbilt Univ, Sch Med, Div Allergy Pulm & Crit Care Med, Dept Med, Nashville, TN 37203 USA Shanghai Center for Disease Control & Prevention; Vanderbilt University; Fudan University; Vanderbilt University

Shi, Y (corresponding author), Shanghai Municipal Ctr Dis Control & Prevent, Div Noncommunicable Dis & Injury, 1380 Zhongshan West Rd, Shanghai 200336, Peoples R China.

shiyan@scdc.sh.cn Jackson, James/LRT-7855-2024; Shu, Xiao'Ou/KPA-5613-2024; ding, ding/LTD-3007-2024 Huang, Zhezhou/0000-0002-4043-698X Shanghai Municipal Commission of Health and Family Planning [20164Y0027, 15GWZK0801]; National Natural Science Foundation of China [81402734]; Department of Defense Breast Cancer Research Program [DAMD 17-02-1-0607]; National Cancer Institute [R01 CA118229] Shanghai Municipal Commission of Health and Family Planning; National Natural Science Foundation of China(National Natural Science Foundation of China (NSFC)); Department of Defense Breast Cancer Research Program(United States Department of Defense); National Cancer Institute(United States Department of Health & Human ServicesNational Institutes of Health (NIH) - USANIH National Cancer Institute (NCI)) The authors thank the patients and the investigators who participated in this study. This work was supported by the Shanghai Municipal Commission of Health and Family Planning (20164Y0027 to Zhezhou Huang; 15GWZK0801 to Zhezhou Huang, PI: Fan Wu), the National Natural Science Foundation of China (81402734 to Pingping Bao), the Department of

Defense Breast Cancer Research Program (DAMD 17-02-1-0607 to Xiao-Ou Shu), and the National Cancer Institute (R01 CA118229 to Xiao-Ou Shu). 34 14 14 1 12 SPRINGER NEW YORK
ONE NEW YORK PLAZA, SUITE 4600, NEW YORK, NY, UNITED STATES
0167-6806 1573-7217 BREAST CANCER RES TR Breast Cancer Res. Treat. AUG 2018 171 1
189 198 10.1007/s10549-018-4805-z
http://dx.doi.org/10.1007/s10549-018-4805-z 10
Oncology Science Citation Index Expanded (SCI-EXPANDED)
Oncology GN0KU 29744676 2025-06-24
WOS:000438656200020
J Mourouti, N; Kontogianni, MD; Papavagelis, C; Psaltopoulou, T; Kapetanstrataki, MG; Plytzanopoulou, P; Vassilakou, T; Malamos, N; Linos, A; Panagiotakos, DB Mourouti, Niki; Kontogianni, Meropi D.; Papavagelis, Christos; Psaltopoulou, Theodora; Kapetanstrataki, Melpo G.; Plytzanopoulou, Petrini; Vassilakou, Tonia; Malamos, Nikolaos; Linos, Athena; Panagiotakos, Demosthenes B. Whole Grain Consumption and Breast Cancer: A Case-Control Study in Women JOURNAL OF THE AMERICAN COLLEGE OF NUTRITION English Article
breast cancer; whole grain consumption; dietary fiber consumption CARDIOVASCULAR-DISEASE; DIETARY FIBER; COLORECTAL-CANCER; RISK; METAANALYSIS; WEIGHT Objective: Whole grain consumption has long been associated with human health. However, its relationship with breast cancer remains not well understood and appreciated. The aim of this work was to evaluate the association between whole grain consumption and breast cancer in women. Methods: A case-control study was designed. Two hundred and fifty consecutive, newly diagnosed breast cancer female patients (56 +/- 12years) and 250 one-to-one age-matched controls were enrolled. A standardized, validated questionnaire assessing various sociodemographic, clinical, lifestyle, and dietary characteristics was applied through face-to-face interviews. Moreover, data on regular consumption of whole grains (i.e., never/rarely, 1-6times/week, >7times/week) were recorded. Overall dietary habits were assessed through the level of adherence to the Mediterranean diet using the MedDietScore (theoretical range 0-55). Results: Whole grain consumption of more than 7times/week was associated with a 0.49-fold (odds ratio = 0.49; 95% confidence interval, 0.29, 0.82) lower likelihood of having breast cancer, after adjustments were made. Conclusions: This study suggested that whole grain consumption more than 7times/week was consistently associated with reduced risk of breast cancer. [Mourouti, Niki; Kontogianni, Meropi D.; Papavagelis, Christos; Kapetanstrataki, Melpo G.; Panagiotakos, Demosthenes B.] Harokopio Univ, Sch Hlth Sci & Educ, Dept Nutr & Dietet, Athens, Greece; [Psaltopoulou, Theodora; Linos, Athena] Univ Athens, Sch Med, Dept Hyg Epidemiol & Med Stat, GR-11527 Athens, Greece; [Plytzanopoulou, Petrini; Vassilakou, Tonia] Natl Sch Publ Hlth, Dept Biochem & Nutr, Athens, Greece; [Malamos, Nikolaos] Gen Hosp Elena Venizelos, Pathol Oncol Dept, Athens, Greece Harokopio University Athens; National & Kapodistrian University of Athens; Athens Medical School; National & Kapodistrian University of Athens Panagiotakos, DB (corresponding author), 46 Paleon Polemiston St, Glifadha 16674, Greece.
d.b.panagiotakos@usa.net Kontogianni, Meropi/AAN-6530-2021; Psaltopoulou, Theodora/AAA-6878-2020; Panagiotakos, Demosthenes/K-8294-2019; Vassilakou, Nair Tonia/R-6967-2017

Kontogianni, Meropi/0000-0003-3168-8867; Vassilakou, Nair Tonia/0000-0002-9993-1897; Mourouti, Niki/0000-0002-7383-5928; Kapetanstrataki, Melpo/0000-0003-4964-3691
25 33 35 0 9 ROUTLEDGE JOURNALS, TAYLOR & FRANCIS LTD ABINGDON 2-4 PARK SQUARE, MILTON PARK, ABINGDON OX14 4RN, OXON, ENGLAND 0731-5724 1541-1087 J AM COLL NUTR
J. Am. Coll. Nutr. FEB 17 2016 35 2
143 149 10.1080/07315724.2014.963899
http://dx.doi.org/10.1080/07315724.2014.963899
7 Nutrition & Dietetics Science Citation Index Expanded (SCI-EXPANDED) Nutrition & Dietetics DG5AU 25915188
2025-06-24 WOS:000372085400008
J Kolar, M; Luksic, I; Gabrovec, B Kolar, Marusa; Luksic, Igor; Gabrovec, Branko Public opinion on the eligibility of health care for migrants and refugees in Slovenia EASTERN MEDITERRANEAN HEALTH JOURNAL
English Article delivery of health care; health care sectors; refugees; transients; migrants CRISIS; EUROPE Background: Worldwide, more than 200 million people have left their home country, and international migration from the Middle East to Europe is increasing. The journey and the poor living conditions cause numerous health problems. Migrants show significant differences in lifestyle, health beliefs and risk factors compared with native populations and this can impact access to health systems and participation in prevention programmes. Aims: Our aim was to measure the attitude of survey participants to migrants and to define up to what level migrants are entitled to health care from the viewpoint of Slovenian citizens. Methods: This survey was carried out in January 2019 and included 311 respondents. We applied a quantitative, nonexperimental sampling method. We used a structured survey questionnaire based on an overview, a national survey on the experiences of patients in hospitals and user satisfaction with medical services of basic health care at the primary level. Results: A large proportion of the respondents agreed that migrants should receive emergency or full health care provision, that there is no need to limit their health rights and that they do not feel that their own rights are compromised by the rights of migrants. Over 80% agreed with health protection for women and for children. Conclusion: The findings offer a basis for supplementing the existing, or designing a new, model of health care provision for migrants in Slovenia, focusing on the provision of health protection and care as a fundamental human right.
[Kolar, Marusa; Luksic, Igor] Univ Ljubljana, Fac Social Sci, Ljubljana, Slovenia; [Gabrovec, Branko] Natl Inst Publ Hlth, Ljubljana, Slovenia University of Ljubljana Gabrovec, B (corresponding author), Natl Inst Publ Hlth, Ljubljana, Slovenia.
branko.gabrovec@nijz.si
31 0 0 2 8 WHO EASTERN MEDITERRANEAN REGIONAL OFFICE NASR CITY, CAIRO P. O. BOX 7608, NASR CITY, CAIRO, EGYPT 1020-3397 1687-1634 E MEDITERR HEALTH J
East Mediterr. Health J. DEC 2021 27 12
1182 1188 10.26719/emhj.21.063
http://dx.doi.org/10.26719/emhj.21.063 7
Health Care Sciences & Services; Health Policy & Services; Public, Environmental & Occupational Health Science Citation Index Expanded (SCI-EXPANDED); Social Science Citation Index (SSCI) Health Care Sciences & Services; Public, Environmental

& Occupational Health YA1WC 35137386 gold 2025-06-24 WOS:000738131200007
S Coughlin, SS Ahmad, A Coughlin, Steven S. Epidemiology of Breast Cancer in Women BREAST CANCER METASTASIS AND DRUG RESISTANCE: CHALLENGES AND PROGRESS, 2ND EDITION Advances in Experimental Medicine and Biology English Article; Book Chapter Alcohol; Breast cancer; Diet; Epidemiology; Genetics; Physical activity DIETARY GLYCEMIC INDEX; BODY-MASS INDEX; RECREATIONAL PHYSICAL-ACTIVITY; POPULATION ATTRIBUTABLE RISK; POSTMENOPAUSAL HORMONE USE; GROWTH-FACTOR-I; MAMMOGRAPHIC DENSITY; AFRICAN-AMERICAN; MEDITERRANEAN DIET; LIFE-COURSE Epidemiologic studies have contributed importantly to current knowledge of environmental and genetic risk factors for breast cancer. Worldwide, breast cancer is an important cause of human suffering and premature mortality among women. In the United States, breast cancer accounts for more cancer deaths in women than any site other than lung cancer. A variety of risk factors for breast cancer have been well-established by epidemiologic studies including race, ethnicity, family history of cancer, and genetic traits, as well as modifiable exposures such as increased alcohol consumption, physical inactivity, exogenous hormones, and certain female reproductive factors. Younger age at menarche, parity, and older age at first full-term pregnancy may influence breast cancer risk through long-term effects on sex hormone levels or by other biological mechanisms. Recent studies have suggested that triple negative breast cancers may have a distinct etiology. Genetic variants and mutations in genes that code for proteins having a role in DNA repair pathways and the homologous recombination of DNA double stranded breaks (APEX1, BRCA1, BRCA2, XRCC2, XRCC3, ATM, CHEK2, PALB2, RAD51, XPD), have been implicated in some cases of breast cancer. [Coughlin, Steven S.] Augusta Univ, Med Coll Georgia, Dept Populat Hlth Sci, Div Epidemiol, Augusta, GA 30912 USA University System of Georgia; Augusta University Coughlin, SS (corresponding author), Augusta Univ, Med Coll Georgia, Dept Populat Hlth Sci, Div Epidemiol, Augusta, GA 30912 USA. scoughlin@augusta.edu 113 244 250 1 77 SPRINGER INTERNATIONAL PUBLISHING AG CHAM GEWERBESTRASSE 11, CHAM, CH-6330, SWITZERLAND 0065-2598 2214-8019 978-3-030-20301-6; 978-3-030-20300-9 ADV EXP MED BIOL Adv.Exp.Med.Biol. 2019 1152 9 29 10.1007/978-3-030-20301-6_2 http://dx.doi.org/10.1007/978-3-030-20301-6_2 10.1007/978-3-030-20301-6 21 Oncology; Medicine, Research & Experimental; Pharmacology & Pharmacy Book Citation Index – Science (BKCI-S); Science Citation Index Expanded (SCI-EXPANDED) Oncology; Research & Experimental Medicine; Pharmacology & Pharmacy BO4LH 31456177 Y N 2025-06-24 WOS:000514550100003
J Alegria-Lertxundi, I; Aguirre, C; Bujanda, L; Fernández, FJ; Polo, F; Ordovás, JM; Etxezarraga, MC; Zabalza, I; Larzabal, M; Portillo, I; de Pancorbo, MM; Garcia-Etxebarria, K; Rocandio, AM; Arroyo-Izaga, M Alegria-Lertxundi, Iker; Aguirre, Carmelo; Bujanda, Luis; Fernandez, Francisco J.; Polo, Francisco; Ordovas, Jose Ma; Etxezarraga, Ma Carmen; Zabalza, Inaki; Larzabal, Mikel; Portillo, Isabel; de Pancorbo, Marian M.; Garcia-Etxebarria, Koldo; Rocandio, Ana Ma; Arroyo-Izaga, Marta Food groups, diet quality and colorectal cancer

risk in the Basque Country WORLD JOURNAL OF GASTROENTEROLOGY English Article

Colorectal cancer; Food group; Dietary quality; Mediterranean diet; Risk-factors; Case-control study

MEDITERRANEAN DIET; POOLED ANALYSIS; FATTY-ACIDS; PREVENTION; QUESTIONNAIRE; MECHANISMS; FREQUENCY; PATTERNS; DISEASE; FIBER BACKGROUND The results obtained to date concerning food groups, diet quality and colorectal cancer (CRC) risk vary according to criteria used and the study populations. AIM To study the relationships between food groups, diet quality and CRC risk, in an adult population of the Basque Country (North of Spain). METHODS This observational study included 308 patients diagnosed with CRC and 308 age- and sex-matched subjects as controls. During recruitment, dietary, anthropometric, lifestyle, socioeconomic, demographic and health status information was collected. Adherence to the dietary recommendations was evaluated utilizing the Healthy Eating Index for the Spanish Diet and the MedDietScore. Conditional logistic regressions were used to evaluate the associations of food group intakes, diet quality scores, categorized in tertiles, with CRC risk. RESULTS The adjusted models for potential confounding factors showed a direct association between milk and dairy products consumption, in particular high-fat cheeses [odds ratio (OR) third tertilevsfirst tertile = 1.87, 95% confidence intervals (CI): 1.11-3.16], and CRC risk. While the consumption of fiber-containing foods, especially whole grains (OR third tertilevsfirst tertile = 0.62, 95%CI: 0.39-0.98), and fatty fish (OR third tertilevsfirst tertile = 0.53, 95%CI: 0.27-0.99) was associated with a lower risk for CRC. Moreover, higher MD adherence was associated with a reduced CRC risk in adjusted models (OR third tertilevsfirst tertile = 0.40, 95%CI: 0.20-0.80). CONCLUSION Direct associations were found for high-fat cheese, whereas an inverse relation was reported for fiber-containing foods and fatty fish, as well as adherence to a Mediterranean dietary pattern. [Alegria-Lertxundi, Iker; Arroyo-Izaga, Marta] Univ Basque Country, UPV EHU, Fac Pharm, Dept Pharm & Food Sci, Paseo Univ 7, Vitoria 01006, Spain; [Aguirre, Carmelo] Galdakao Usansolo Univ Hosp, Pharmacovigilance Unit, Osakidetza 48960, Galdakao, Spain; [Bujanda, Luis] Univ Basque Country, UPV EHU, Ctr Invest Biomed Red Enfermedades Hepaticas & Di, Donostia Univ Hosp Biodonostia,Dept Gastroenterol, San Sebastian 20014, Spain; [Fernandez, Francisco J.] Galdakao Usansolo Univ Hosp, Dept Gastroenterol, Osakidetza 48960, Galdakao, Spain; [Polo, Francisco] Basurto Univ Hosp, Dept Gastroenterol, Bilbao 48013, Spain; [Ordovas, Jose Ma] Tufts Univ, Jean Mayer Human Nutr Res Ctr Aging, Nutr & Genom Lab, Boston, MA 02111 USA; [Ordovas, Jose Ma] IMDEA Food, Madrid 28049, Spain; [Etxezarraga, Ma Carmen] Basurto Univ Hosp, Dept Pathol, Bilbao 48013, Spain; [Etxezarraga, Ma Carmen] Univ Basque Country, UPV EHU, Dept Phys & Surg Special, Leioa 48940, Spain; [Zabalza, Inaki] Galdakao Usansolo Univ Hosp, Dept Pathol, Osakidetza 48960, Osakidetza, Spain; [Larzabal, Mikel] Donostia Hosp Biodonostia, Dept Pathol, Ctr Invest Biomed Red Enfermedades Hepaticas & Di, San Sebastian 20014, Spain; [Portillo, Isabel] Colorectal Canc Screening Programme, Bilbao 48011, Spain; [de Pancorbo, Marian M.; Rocandio, Ana Ma] Univ Basque Country, UPV EHU, BIOMICs Res Grp, Vitoria 01006, Spain; [Garcia-Etxebarria, Koldo] Ctr Invest Biomed Red Enfermedades Hepaticas & Di, Gastrointestinal Genet Grp, Biodonostia, San Sebastian 20014, Spain University of Basque

Country; CIBER - Centro de Investigacion Biomedica en Red; CIBEREHD; University of Basque Country; Basurto Hospital; Tufts University; IMDEA Food Institute; Basurto Hospital; University of Basque Country; CIBER - Centro de Investigacion Biomedica en Red; CIBEREHD; University of Basque Country; Instituto de Investigacion Sanitaria Biogipuzkoa; CIBER - Centro de Investigacion Biomedica en Red; CIBEREHD Arroyo-Izaga, M (corresponding author), Univ Basque Country, UPV EHU, Fac Pharm, Dept Pharm & Food Sci, Paseo Univ 7, Vitoria 01006, Spain. marta.arroyo@ehu.eus de Pancorbo, Marian/ADQ-0719-2022; LERTXUNDI, IKER/R-6018-2016; Pérez Fernández, Francisco/ISU-9112-2023; Garcia-Etxebarria, Koldo/AAQ-4638-2021; Bujanda, Luis/AAB-2485-2021; Stefanadis, Christodoulos/ABH-2232-2020; Arroyo-Izaga, Marta/F-2139-2013
Portillo, Isabel/0000-0002-3871-3423; Bujanda, Luis/0000-0002-4353-9968; Stefanadis, Christodoulos/0000-0001-5974-6454; Arroyo-Izaga, Marta/0000-0001-5592-4241; M. de Pancorbo, Marian/0000-0002-8081-0702 Department of Health and Consumer Affairs, Basque Government [2011111153]; Saiotek, Basque Government [S-PE12UN058]; Basque Government [PRE_2015_2_0084]; United States Department of AgricultureAgricultural Research Service [58-1950-4-003] Department of Health and Consumer Affairs, Basque Government; Saiotek, Basque Government(Basque Government); Basque Government(Basque Government); United States Department of AgricultureAgricultural Research Service(United States Department of Agriculture (USDA)USDA Agricultural Research Service) Supported by the Department of Health and Consumer Affairs, Basque Government, No. 2011111153; Saiotek, Basque Government, No. S-PE12UN058; Pre-doctoral grant from the Basque Government, No. PRE_2015_2_0084; and United States Department of AgricultureAgricultural Research Service, No. 58-1950-4-003.
64 16 18 2 22 BAISHIDENG PUBLISHING GROUP INC
PLEASANTON 7041 Koll Center Parkway, Suite 160, PLEASANTON, CA, UNITED STATES 1007-9327 2219-2840 WORLD J GASTROENTERO World J. Gastroenterol. JUL 28 2020 26
28 4108 4125
10.3748/wjg.v26.i28.4108
http://dx.doi.org/10.3748/wjg.v26.i28.4108 18
Gastroenterology & Hepatology Science Citation Index Expanded (SCI-EXPANDED) Gastroenterology & Hepatology
NA7VS 32821073 Green Published, hybrid
2025-06-24 WOS:000560025900007
J Nasseri, K Nasseri, Kiumarss
Breast Cancer in the Middle Eastern Population of California, 1988-2004 BREAST JOURNAL English
Article breast cancer; California; California Cancer Registry; Middle Eastern; salmon bias; survival MORTALITY; MIGRANTS This report presents the patterns of incidence, survival, and mortality of breast cancer in the Middle Eastern (ME) population of California. Cases were identified through surname recognition and population estimates were obtained from census public use files. Rates, trends, and survival in this ethnic group were compared with the non-Hispanic White (NHW) of California, as well as natives in the Middle East. Age-adjusted incidence rates for the insitu (22.8), invasive (126.2), and mortality (23.2) in ME women were significantly lower than similar rates of 26.0, 146.9, and 30.6 in the NHW women. Incidence rate in ME women in California was higher than rates in women in the Middle East. Lower rates for early stage and higher

rates for late stage diagnoses in this ethnic population suggest lack of optimal access to preventive healthcare. Relative survival in the two groups is negatively associated with stage at diagnosis and is slightly higher in ME women, probably due to large numbers of lost to follow-up in ME women suggesting the presence of salmon bias. Positive association with socioeconomic standing was detected only in the NHW women. Incidence of breast cancer in ME men was significantly higher than that of NHW men. [Nasseri, Kiumarss] Inst Publ Hlth, Calif Canc Registry, Santa Barbara, CA USA Public Health Institute Nasseri, K (corresponding author), Inst Publ Hlth, Calif Canc Registry, 1825 Bell St,Ste 203, Sacramento, CA 95825 USA. QNasserei@-west.net
California Department of Health Services [103885]; National Cancer Institute [CA103457]; Centers for Disease Control and Prevention National Program of Cancer Registries California Department of Health Services; National Cancer Institute(United States Department of Health & Human ServicesNational Institutes of Health (NIH) - USANIH National Cancer Institute (NCI)); Centers for Disease Control and Prevention National Program of Cancer Registries(United States Department of Health & Human ServicesCenters for Disease Control & Prevention - USA) The author would like to express his gratitude to Mark Allen, MS from the California Cancer Registry for linking the California Cancer Registry data-base with the ME surname list, and Thomas H. Tylor, PhD for constructive comments and help. The collection of cancer data used in this study was supported by the California Department of Health Services as part of the statewide cancer reporting program mandated by California Health and Safety Code Section 103885, the National Cancer Institute's Surveillance, Epidemiology and End Results Program, and Centers for Disease Control and Prevention National Program of Cancer Registries. The ideas and opinions expressed herein are those of the author and endorsement by the State of California, Department of Health Services, the National Cancer Institute, and the Centers for Disease Control and Prevention is not intended and should not be inferred. This work was supported by the grant CA103457 from the National Cancer Institute to Kiumarss Nasseri and was approved by the Institutional Review Board of the Public Health Institute. These results were partially presented at the Annual Meeting of the North American Association of Central Cancer Registries (NAACCR), Detroit, Michigan, 2006. 35 11 14 0 2
WILEY HOBOKEN 111 RIVER ST, HOBOKEN 07030-5774, NJ USA
1075-122X 1524-4741 BREAST J Breast J. MAR-APR 2009 15 2 182 188
10.1111/j.1524-4741.2009.00694.x
http://dx.doi.org/10.1111/j.1524-4741.2009.00694.x
7 Oncology; Obstetrics & Gynecology Science Citation Index Expanded (SCI-EXPANDED) Oncology; Obstetrics & Gynecology 417PG 19292805 gold 2025-06-24
WOS:000264087900010
J Mourouti, N; Kontogianni, MD; Papavagelis, C; Plytzanopoulou, P; Vassilakou, T; Malamos, N; Linos, A; Panagiotakos, DB Mourouti, Niki; Kontogianni, Meropi D.; Papavagelis, Christos; Plytzanopoulou, Petrini; Vassilakou, Tonia; Malamos, Nikolaos; Linos, Athena; Panagiotakos, Demosthenes B. Adherence to the Mediterranean Diet is Associated With Lower Likelihood of Breast Cancer: A Case-Control Study NUTRITION AND CANCER-AN INTERNATIONAL JOURNAL

English Article
CARDIOVASCULAR-DISEASE; HEALTHY-ADULTS; OLIVE OIL; RISK; PATTERNS; ALCOHOL Mediterranean diet has long been associated with human health. However, its relationship with breast cancer remains not well understood and appreciated. The aim of this work was to evaluate the association between adherence to the Mediterranean diet and its inherent constituents, with breast-cancer. Two-hundred-and-fifty consecutive, newly diagnosed breast-cancer female patients (56 +/- 12 yr) and 250, 1-to-1 age-matched with the patients, controls, were studied. A standardized, validated questionnaire assessing various sociodemographic, clinical, lifestyle, and dietary characteristics, was applied through face-to-face interviews. Adherence to the Mediterranean diet was evaluated using the 11-components MedDietScore (theoretical range 0-55). Multiple logistic regression was applied to test the research hypothesis, whereas discriminant analysis was used to explore the strength of each component in relation to the outcome. One unit increase in the MedDietScore (i.e., greater adherence to the Mediterranean diet) was associated with 9% lower likelihood of having breast cancer (odds ratio = 0.91; 95% confidence interval, 0.86, 0.97). Decomposition of the MedDietScore revealed that the most important components and with beneficial effect were nonrefined cereals, vegetables, fruits, and alcohol, followed by red meat, but with unfavorable effect. A dietary recommendation for healthy eating, close to the Mediterranean dietary pattern, seems promising for breast cancer prevention.[Mourouti, Niki; Kontogianni, Meropi D.; Papavagelis, Christos; Panagiotakos, Demosthenes B.] Harokopio Univ, Dept Nutr & Dietet, Athens, Greece; [Plytzanopoulou, Petrini; Vassilakou, Tonia] Natl Sch Publ Hlth, Dept Nutr & Biochem, Athens, Greece; [Malamos, Nikolaos] Gen Hosp Elena Venizelos, Pathol Oncol Dept, Athens, Greece; [Linos, Athena] Univ Athens, Sch Med, Dept Hyg Epidemiol & Med Stat, GR-11527 Athens, Greece Harokopio University Athens; National & Kapodistrian University of Athens; Athens Medical School; National & Kapodistrian University of Athens Panagiotakos, DB (corresponding author), 46 Paleon Polemiston St, Glifadha 16674, Greece. d.b.panagiotakos@usa.net
Panagiotakos, Demosthenes/K-8294-2019; Kontogianni, Meropi/AAN-6530-2021; Vassilakou, Nair Tonia/R-6967-2017Mourouti, Niki/0000-0002-7383-5928; Vassilakou, Nair Tonia/0000-0002-9993-1897; Kontogianni, Meropi/0000-0003-3168-8867 Graduate Program of the Department of Nutrition and Dietetics, Harokopio University in Athens, GreeceGraduate Program of the Department of Nutrition and Dietetics, Harokopio University in Athens, Greece
The study was partially funded by the Graduate Program of the Department of Nutrition and Dietetics, Harokopio University in Athens, Greece. 35 31 39 0 23 ROUTLEDGE JOURNALS, TAYLOR & FRANCIS LTD ABINGDON 2-4 PARK SQUARE, MILTON PARK, ABINGDON OX14 4RN, OXON, ENGLAND 0163-5581
1532-7914 NUTR CANCER Nutr. Cancer JUL 2014 66
5 810 817
10.1080/01635581.2014.916319
http://dx.doi.org/10.1080/01635581.2014.916319
8 Oncology; Nutrition & Dietetics Science Citation Index Expanded (SCI-EXPANDED) Oncology; Nutrition & Dietetics
AJ9DC 24847911 2025-06-24
WOS:000338006000005

J	Christodoulou, A; Ajzajian, J; Su, DJ; Wang, HM; Roupa, Z; Farazi, PA		Christodoulou, Andria; Ajzajian, Jirayr; Su, Dejun; Wang, Hongmei; Roupa, Zoe; Farazi, Paraskevi A.		Awareness of human papilloma virus and cervical cancer prevention among Cypriot female healthcare workers
ECANCERMEDICALSCIENCE		English	Article
human papilloma virus; cervical cancer prevention; awareness; healthcare professionals; Cyprus HPV INFECTION; BEHAVIORS; KNOWLEDGE; VACCINES	Background: Cervical cancer incidence varies around the world with the highest rates in Eastern Africa and the lowest rates in Western Asia. In Cyprus, a small Mediterranean island, cervical cancer incidence was 6.4 per 100,000 in 2013. HPV is an established risk factor for cervical cancer with HPV-16 and HPV-18 being the most common carcinogenic strains. Cervical cancer is preventable through primary (HPV vaccination) and secondary (Pap and HPV tests) prevention. These prevention methods should be promoted, however, in order to design a cancer prevention programme and the awareness and characteristics of populations should be investigated so that prevention programmes can be targeted specifically to them. Methods: In this work, we sought to investigate awareness of HPV and cervical cancer prevention among female healthcare workers in Cyprus. To achieve this, we conducted a 60-item survey among 200 healthcare professionals in randomly selected hospitals in two different cities within Cyprus. Results: Our results revealed that nearly 10% of our participants reported not ever having had a Pap test. 88.5% of the healthcare workers knew about HPV and 86.5% reported that HPV is transmitted through sexual intercourse. 83.5% of the participants were willing to vaccinate themselves for cervical cancer prevention. Conclusion: Even though awareness and vaccination acceptance were relatively high, they are still not optimal for healthcare professionals who play an essential role in health promotion. We suggest the design of educational programmes to target this population and improve their knowledge so that they can promote cervical cancer prevention in their health practice.
[Christodoulou, Andria; Ajzajian, Jirayr; Roupa, Zoe] Univ Nicosia, 46 Makedonitissas Ave, CY-2417 Nicosia, Cyprus; [Christodoulou, Andria] Cyprus Univ Technol, Cyprus Inst Environm & Publ Hlth, CY-3036 Limassol, Cyprus; [Su, Dejun] Univ Nebraska Med Ctr, Coll Publ Hlth, Dept Hlth Promot, Omaha, NE 68198 USA; [Wang, Hongmei] Univ Nebraska Med Ctr, Dept Hlth Serv Res & Adm, Coll Publ Hlth, Omaha, NE 68198 USA; [Farazi, Paraskevi A.] Univ Nebraska Med Ctr, Dept Epidemiol, Coll Publ Hlth, 984395 Nebraska Med Ctr, Omaha, NE 68198 USAUniversity of Nicosia; Cyprus University of Technology; University of Nebraska System; University of Nebraska Medical Center; University of Nebraska System; University of Nebraska Medical Center; University of Nebraska System; University of Nebraska Medical Center	Farazi, PA (corresponding author), Univ Nebraska Med Ctr, Dept Epidemiol, Coll Publ Hlth, 984395 Nebraska Med Ctr, Omaha, NE 68198 USA.
evi.farazi@unmc.edu		Su, Dejun/0000-0002-7723-3262; Wang, Hongmei/0000-0003-0810-064X	ecancer (UK Charity) [1176307]
ecancer (UK Charity)	Publication costs for this article were supported by ecancer (UK Charity number 1176307).		25
5	5	0	3	eCancer Global Foundation	Bristol
13 King Square Avenue, Bristol, UNITED KINGDOM	1754-6605
ECANCERMEDICALSCIENC	eCancerMedicalScience	NOV 20	2019	13			978

10.3332/ecancer.2019.978
http://dx.doi.org/10.3332/ecancer.2019.978 10
Oncology Emerging Sources Citation Index (ESCI) Oncology
JZ4NE 31921349 Green Published, gold, Green Submitted
2025-06-24 WOS:000505078100001
J Rossner, P; Gammon, MD; Zhang, YJ; Terry, MB; Hibshoosh, H; Memeo, L; Mansukhani, M; Long, CM; Garbowski, G; Agrawal, M; Kalra, TS; Gaudet, MM; Teitelbaum, SL; Neugut, AI; Santella, RM
Rossner, Pavel, Jr.; Gammon, Marilie D.; Zhang, Yu-Jing; Terry, Mary Beth; Hibshoosh, Hanina; Memeo, Lorenzo; Mansukhani, Mahesh; Long, Chang-Min; Garbowski, Gail; Agrawal, Meenakshi; Kalra, Tara S.; Gaudet, Mia M.; Teitelbaum, Susan L.; Neugut, Alfred I.; Santella, Regina M.
Mutations in p53, p53 protein overexpression and breast cancer survival JOURNAL OF CELLULAR AND MOLECULAR MEDICINE
English Article
breast cancer; p53 mutations; p53 overexpression; survival
CARCINOMA IN-SITU; PROGNOSTIC VALUE; GENE-MUTATIONS; RISK-FACTORS; IMMUNOHISTOCHEMICAL EXPRESSION; SURVEYOR(TM) NUCLEASE; MISSENSE MUTATIONS; TP53 MUTATION; MARKERS; GRADE p53 is an important tumour suppressor gene that encodes p53 protein, a molecule involved in cell cycle regulation and has been inconsistently linked to breast cancer survival. Using archived tumour tissue from a population-based sample of 859 women diagnosed with breast cancer between 1996 and 1997, we determined p53 mutations in exons 5-8 and p53 protein overexpression. We examined the association of p53 mutations with overexpression and selected tumour clinical parameters. We assessed whether either p53 marker was associated with survival through 2002, adjusting for other tumour markers and prognostic factors. The prevalence of protein overexpression in the tumour was 36% (307/859) and of any p53 mutation was 15% (128/859). p53 overexpression was positively associated with the presence of any p53 mutation (odds ratio [OR] = 2.2, 95% confidence interval [CI] = 1.5-3.2), particularly missense mutations (ER = 7.0, 95% CI = 3.6-13.7). Negative oestrogen and progesterone receptor (ER/PR) status was positively associated with both p53 protein overexpression (= 2.6, 95% CI = 1.7-4.0) and p53 mutation (OR = 3.9, 95% CI = 2.4-6.5). Any p53 mutation and missense mutations, but not p53 protein overexpression, were associated with breast cancer-specific mortality (hazard ratio [HR] = 1.7, 95% CI = 1.0-2.8; HR = 2.0, 95% CI = 1.1-3.6, respectively) and all-cause mortality (HR = 1.5, 95% CI = 1.0-2.4; HR = 2.0, 95% CI = 1.2-3.4, respectively); nonsense mutations were associated only with breast cancer-specific mortality (HR = 3.0, 95% CI = 1.1-8.1). These associations however did not remain after adjusting for ER/PR status. Thus, in this population-based cohort of women with breast cancer, although p53 protein overexpression and p53 mutations were associated with each other, neither independently impacted breast cancer-specific or all-causing mortality, after considering ER/PR status. [Rossner, Pavel, Jr.] AS CR, Inst Expt Med, Lab Genet Ecotoxicol, Prague 14220, Czech Republic; [Rossner, Pavel, Jr.; Zhang, Yu-Jing; Long, Chang-Min; Garbowski, Gail; Agrawal, Meenakshi; Santella, Regina M.] Columbia Univ, Dept Environm Hlth Sci, Mailman Sch Publ Hlth, New York, NY USA; [Gammon, Marilie D.; Gaudet, Mia M.] Univ N Carolina, Sch Publ Hlth, Dept Epidemiol, Chapel Hill, NC USA; [Terry, Mary Beth; Kalra, Tara S.; Neugut, Alfred I.] Columbia Univ, Dept Epidemiol, Mailman Sch Publ Hlth,

New York, NY USA; [Hibshoosh, Hanina; Mansukhani, Mahesh] Columbia Univ, Coll Phys & Surg, Dept Pathol, New York, NY USA; [Memeo, Lorenzo] Mediterranean Inst Oncol, Pathol Unit, Catania, Italy; [Teitelbaum, Susan L.] Mt Sinai Sch Med, Dept Community & Prevent Med, New York, NY USA Czech Academy of Sciences; Institute of Experimental Medicine of the Czech Academy of Sciences; Columbia University; University of North Carolina; University of North Carolina Chapel Hill; Columbia University; Columbia University; Mediterranean Institute of Oncology; Icahn School of Medicine at Mount Sinai Rossner, P (corresponding author), AS CR, Inst Expt Med, Lab Genet Ecotoxicol, Vvi,Videnska 1083, Prague 14220, Czech Republic. prossner@biomed.cas.cz Zhang, Yujing/ABB-3590-2021; Rossner, Pavel/AAI-5789-2020 Rossner, Pavel/0000-0001-6921-5446; Terry, Mary Beth/0000-0002-4106-5033; Memeo, Lorenzo/0000-0003-4251-7203 National Cancer Institute [U01 CA/ES66572, P30ES09089, P30ES10126, K07CA90685-02]; National Institute of Environmental Health Sciences; US Army [BC972772]; Breast Cancer Research Foundation National Cancer Institute(United States Department of Health & Human ServicesNational Institutes of Health (NIH) - USANIH National Cancer Institute (NCI)); National Institute of Environmental Health Sciences(United States Department of Health & Human ServicesNational Institutes of Health (NIH) - USANIH National Institute of Environmental Health Sciences (NIEHS)); US Army(United States Department of DefenseUnited States Army); Breast Cancer Research Foundation This work was funded in part by grants U01 CA/ES66572, P30ES09089, P30ES10126 and K07CA90685-02 from the National Cancer Institute and the National Institute of Environmental Health Sciences; awards from the US Army (BC972772), Breast Cancer Research Foundation and Women at Risk Program and gifts from private citizens. The authors have no conflicts of interest to declare. 56 38 41 2 40 WILEY HOBOKEN 111 RIVER ST, HOBOKEN 07030-5774, NJ USA 1582-1838 1582-4934 J CELL MOL MED J. Cell. Mol. Med. SEP 2009 13 9B 3847 3857 10.1111/j.1582-4934.2008.00553.x http://dx.doi.org/10.1111/j.1582-4934.2008.00553.x 11 Cell Biology; Medicine, Research & Experimental Science Citation Index Expanded (SCI-EXPANDED) Cell Biology; Research & Experimental Medicine 551BC 19602056 Green Accepted, hybrid, Green Published 2025-06-24 WOS:000274179300075

J Leufkens, AM; Van Duijnhoven, FJB; Boshuizen, HC; Siersema, PD; Kunst, AE; Mouw, T; Tjonneland, A; Olsen, A; Overvad, K; Boutron-Ruault, MC; Clavel-Chapelon, F; Morois, S; Krogh, V; Tumino, R; Panico, S; Polidoro, S; Palli, D; Kaaks, R; Teucher, B; Pischon, T; Trichopoulou, A; Orfanos, P; Goufa, I; Peeters, PHM; Skeie, G; Braaten, T; Rodríguez, L; Lujan-Barroso, L; Sánchez-Pérez, MJ; Navarro, C; Barricarte, A; Zackrisson, S; Almquist, M; Hallmans, G; Palmqvist, R; Tsilidis, KK; Khaw, KT; Wareham, N; Gallo, V; Jenab, M; Riboli, E; Bueno-de-Mesquita, HB

Leufkens, Anke M.; Van Duijnhoven, Franzel J. B.; Boshuizen, Hendriek C.; Siersema, Peter D.; Kunst, Anton E.; Mouw, Traci; Tjonneland, Anne; Olsen, Anja; Overvad, Kim; Boutron-Ruault, Marie-Christine; Clavel-Chapelon, Francoise; Morois, Sophie; Krogh, Vittorio; Tumino, Rosario; Panico, Salvatore; Polidoro, Silvia; Palli, Domenico; Kaaks, Rudolf; Teucher, Birgit; Pischon, Tobias; Trichopoulou, Antonia; Orfanos, Philippos; Goufa, Ioulia; Peeters, Petra H. M.; Skeie, Guri; Braaten, Tonje;

Rodriguez, Laudina; Lujan-Barroso, Leila; Sanchez-Perez, Maria-Jose; Navarro, Carmen; Barricarte, Aurelio; Zackrisson, Sophia; Almquist, Martin; Hallmans, Goran; Palmqvist, Richard; Tsilidis, Konstantinos K.; Khaw, Kay-Tee; Wareham, Nick; Gallo, Valentina; Jenab, Mazda; Riboli, Elio; Bueno-de-Mesquita, H. Bas
Educational level and risk of colorectal cancer in EPIC with specific reference to tumor location INTERNATIONAL JOURNAL OF CANCER English Article
colorectal cancer; tumor location; educational level
SOCIOECONOMIC-STATUS; UNITED-STATES; NUTRITION; COLON; SURVIVAL; HEALTH; WOMEN; INEQUALITIES; DEPRIVATION; RECTUM
Existing evidence is inconclusive on whether socioeconomic status (SES) and educational inequalities influence colorectal cancer (CRC) risk, and whether low or high SES/educational level is associated with developing CRC. The aim of our study was to investigate the relationship between educational level and CRC. We studied data from 400,510 participants in the EPIC (European Prospective Investigation into Cancer and Nutrition) study, of whom 2,447 developed CRC (colon: 1,551, rectum: 896, mean follow-up 8.3 years). Cox proportional hazard regression analysis stratified by age, gender and center, and adjusted for potential confounders were used to estimate hazard ratios (HR) and 95% confidence intervals (95% CI). Relative indices of inequality (RII) for education were estimated using Cox regression models. We conducted separate analyses for tumor location, gender and geographical region. Compared with participants with college/university education, participants with vocational secondary education or less had a nonsignificantly lower risk of developing CRC. When further stratified for tumor location, adjusted risk estimates for the proximal colon were statistically significant for primary education or less (HR 0.73, 95% CI 0.57-0.94) and for vocational secondary education (HR 0.76, 95% CI 0.58-0.98). The inverse association between low education and CRC risk was particularly found in women and Southern Europe. These associations were statistically significant for CRC, for colon cancer and for proximal colon cancer. In conclusion, CRC risk, especially in the proximal colon, is lower in subjects with a lower educational level compared to those with a higher educational level. This association is most pronounced in women and Southern Europe. [Leufkens, Anke M.; Siersema, Peter D.; Bueno-de-Mesquita, H. Bas] Univ Med Ctr, Dept Gastroenterol & Hepatol, Utrecht, Netherlands; [Leufkens, Anke M.; Van Duijnhoven, Franzel J. B.; Boshuizen, Hendriek C.; Bueno-de-Mesquita, H. Bas] Natl Inst Publ Hlth & Environm RIVM, Bilthoven, Netherlands; [Van Duijnhoven, Franzel J. B.; Peeters, Petra H. M.] Univ Med Ctr, Julius Ctr Hlth Sci & Primary Care, Utrecht, Netherlands; [Kunst, Anton E.] Univ Amsterdam, Acad Med Ctr, Dept Publ Hlth, NL-1105 AZ Amsterdam, Netherlands; [Mouw, Traci] Univ London Imperial Coll Sci Technol & Med, Div Epidemiol Publ Hlth & Primary Care, Fac Med, London, England; [Tjonneland, Anne; Olsen, Anja] Danish Canc Soc, Inst Canc Epidemiol, Copenhagen, Denmark; [Overvad, Kim] Aarhus Univ, Sch Publ Hlth, Dept Epidemiol, DK-8000 Aarhus C, Denmark; [Boutron-Ruault, Marie-Christine; Clavel-Chapelon, Francoise; Morois, Sophie] Inst Gustave Roussy, INSERM, U1018, Ctr Res Epidemiol & Populat Hlth, F-94805 Villejuif, France; [Boutron-Ruault, Marie-Christine; Clavel-Chapelon, Francoise; Morois, Sophie] Paris S Univ, UMRS 1018, F-94805 Villejuif, France; [Krogh, Vittorio] Fdn IRCCS Ist Nazl Tumori, Dept Prevent &

Predict Med, Milan, Italy; [Tumino, Rosario] Civile MP Arezzo Hosp, Canc Registry & Histopathol Unit, Ragusa, Italy; [Panico, Salvatore] Univ Naples Federico II, Dipartimento Med Clin & Sperimentale, I-80138 Naples, Italy; [Polidoro, Silvia] Human Genet Fdn Hugef, Turin, Italy; [Palli, Domenico] ISPO Florence, Canc Res & Prevent Inst, Mol & Nutr Epidemiol Unit, Florence, Italy; [Kaaks, Rudolf; Teucher, Birgit] German Canc Res Ctr, Dept Canc Epidemiol, D-6900 Heidelberg, Germany; [Pischon, Tobias] German Inst Human Nutr Potsdam Rehbrucke, Dept Epidemiol, Nuthetal, Germany; [Trichopoulou, Antonia; Orfanos, Philippos; Goufa, Ioulia] Univ Athens, Sch Med, Dept Hyg Epidemiol & Med Stat, WHO Collaborating Ctr Food & Nutr Policies, GR-11527 Goudi, Greece; [Trichopoulou, Antonia; Orfanos, Philippos; Goufa, Ioulia] Hellen Hlth Fdn, GR-11527 Athens, Greece; [Skeie, Guri; Braaten, Tonje] Univ Tromso, Inst Community Med, N-9001 Tromso, Norway; [Rodriguez, Laudina] Hlth & Hlth Care Serv Council, Publ Hlth & Participat Directorate, Asturias, Spain; [Lujan-Barroso, Leila] Catalan Inst Oncol, Canc Epidemiol Res Programme, Unit Nutr Environm & Canc, Barcelona, Spain; [Sanchez-Perez, Maria-Jose] Andalusian Sch Publ Hlth, Granada, Spain; [Sanchez-Perez, Maria-Jose; Navarro, Carmen; Barricarte, Aurelio] CIBER Epidemiol & Salud Publ CIBERESP, Madrid, Spain   Utrecht University; Utrecht University Medical Center; Netherlands National Institute for Public Health & the Environment; Utrecht University; Utrecht University Medical Center; University of Amsterdam; Academic Medical Center Amsterdam; Imperial College London; Danish Cancer Society; Aarhus University; Universite Paris Saclay; Institut National de la Sante et de la Recherche Medicale (Inserm); UNICANCER; Gustave Roussy; Institut National de la Sante et de la Recherche Medicale (Inserm); Universite Paris Saclay; Fondazione IRCCS Istituto Nazionale Tumori Milan; Civile M.P. Arezzo Hospital; University of Naples Federico II; Helmholtz Association; German Cancer Research Center (DKFZ); Leibniz Association; Deutsches Institut fur Ernahrungsforschung Potsdam-Rehbrucke (DIfE); World Health Organization; National & Kapodistrian University of Athens; UiT The Arctic University of Tromso; Institut Catala d'Oncologia; Escuela Andaluza de Salud Publica; CIBER - Centro de Investigacion Biomedica en Red; CIBERESP

Bueno-de-Mesquita, HB (corresponding author), Natl Inst Publ Hlth & Environm, POB 1, NL-3720 BA Bilthoven, Netherlands.

Bas.Bueno.De.Mesquita@rivm.nl   Krogh, Vittorio/AAA-9171-2019; Siersema, Peter/V-1636-2019; Kunst, Anton/M-3021-2014; Almquist, Martin/H-7209-2019; Panico, Salvatore/K-6506-2016; Tjonneland, Anne/AGU-0320-2022; van Duijnhoven, Fränzel/GWC-0059-2022; Boshuizen, Hendriek/B-3718-2014; Boutron-Ruault, Marie-Christine/H-3936-2014; Orfanos, Philippos/AAL-2606-2021; Jenab, Mehdi/L-2515-2019; Clavel-Chapelon, Francoise/G-6733-2014; TRICHOPOULOU, ANTONIA/ABF-8727-2021; Sánchez, María/JTS-8758-2023; Polidoro, Silvia/GYJ-3517-2022; Lujan-Barroso, Leila/AAD-3166-2021; Teucher, Birgit/J-6380-2015; Pischon, Tobias/HGE-8577-2022; Khaw, Kay-Tee/AAZ-3209-2021; Riboli, Elio/A-4357-2009; Krogh, Vittorio/K-2628-2016; Gallo, Valentina/X-1139-2018; Boshuizen, Hendriek/M-3415-2014; SANCHEZ-PEREZ, MARIA JOSE/D-1087-2011

Kunst, Anton/0000-0002-3313-5273; Skeie, Guri/0000-0003-2476-4251; Polidoro, Silvia/0000-0003-2968-0575; Riboli, Elio/0000-0001-6795-6080; Panico, Salvatore/0000-0002-5498-8312; tumino, rosario/0000-0003-2666-414X; Orfanos, Philippos/0000-0002-9949-3137; Jenab, Mazda/0000-0002-0573-1852; Almquist,

Martin/0000-0002-0953-1188; Olsen, Anja/0000-0003-4788-503X; Krogh, Vittorio/0000-0003-0122-8624; Navarro, Carmen/0000-0001-8896-7483; Gallo, Valentina/0000-0002-1268-8629; Lujan-Barroso, Leila/0000-0001-6224-1764; Boshuizen, Hendriek/0000-0002-3916-9095; Overvad, Kim/0000-0001-6429-7921; Pischon, Tobias/0000-0003-1568-767X; Tjonneland, Anne/0000-0003-4385-2097; PALLI, Domenico/0000-0002-5558-2437; Tsilidis, Konstantinos/0000-0002-8452-8472; SANCHEZ-PEREZ, MARIA JOSE/0000-0003-4817-0757European Commission; Ligue contre le Cancer; Societe 3M; Mutuelle Generale de l'Education Nationale; Institut National de la Sante et de la Recherche Medicale (INSERM) (France); German Cancer Aid; German Cancer Research Center; Federal Ministry of Education and Research (Germany); Danish Cancer Society (Denmark); Health Research Fund (FIS) of the Spanish Ministry of Health; Cancer Research UK; Medical Research Council; Stroke Association; British Heart Foundation; Department of Health; Food Standards Agency; Wellcome Trust (United Kingdom); Hellenic Ministry of Health; Stavros Niarchos Foundation; Hellenic Health Foundation (Greece); Italian Association for Research on Cancer; National Research Council (Italy); Dutch Ministry of Public Health, Welfare and Sports (VWS); Netherlands Cancer Registry (NKR); LK Research Funds; Dutch Prevention Funds; Dutch ZON (Zorg Onderzoek Nederland); World Cancer Research Fund (WCRF); Statistics Netherlands (The Netherlands); Swedish Cancer Society; Swedish Scientific Council; Regional Government of Skane (Sweden); Norwegian Research Council and NordForsk (Norway) European Commission(European Union (EU)European Commission Joint Research Centre); Ligue contre le Cancer(Ligue nationale contre le cancer); Societe 3M(3M); Mutuelle Generale de l'Education Nationale; Institut National de la Sante et de la Recherche Medicale (INSERM) (France)(Institut National de la Sante et de la Recherche Medicale (Inserm)); German Cancer Aid(Deutsche Krebshilfe); German Cancer Research Center; Federal Ministry of Education and Research (Germany)(Federal Ministry of Education & Research (BMBF)); Danish Cancer Society (Denmark)(Danish Cancer Society); Health Research Fund (FIS) of the Spanish Ministry of Health(Instituto de Salud Carlos III); Cancer Research UK(Cancer Research UK); Medical Research Council(UK Research & Innovation (UKRI)Medical Research Council UK (MRC)); Stroke Association; British Heart Foundation(British Heart Foundation); Department of Health; Food Standards Agency; Wellcome Trust (United Kingdom)(Wellcome Trust); Hellenic Ministry of Health; Stavros Niarchos Foundation; Hellenic Health Foundation (Greece); Italian Association for Research on Cancer(Fondazione AIRC per la ricerca sul cancro); National Research Council (Italy)(Consiglio Nazionale delle Ricerche (CNR)); Dutch Ministry of Public Health, Welfare and Sports (VWS); Netherlands Cancer Registry (NKR); LK Research Funds; Dutch Prevention Funds; Dutch ZON (Zorg Onderzoek Nederland)(Netherlands Organization for Scientific Research (NWO)); World Cancer Research Fund (WCRF)(World Cancer Research Fund International (WCRF)); Statistics Netherlands (The Netherlands)(Netherlands Government); Swedish Cancer Society(Swedish Cancer Society); Swedish Scientific Council; Regional Government of Skane (Sweden); Norwegian Research Council and NordForsk (Norway)(Research Council of NorwayNordForsk) Grant sponsors: European Commission: Public Health and Consumer Protection Directorate 1993-2004, Research Directorate-General 2005, Ligue contre le Cancer, Societe 3M, Mutuelle Generale de l'Education Nationale, Institut National de

la Sante et de la Recherche Medicale (INSERM) (France), German Cancer Aid, German Cancer Research Center, Federal Ministry of Education and Research (Germany), Danish Cancer Society (Denmark), Health Research Fund (FIS) of the Spanish Ministry of Health, The participating regional governments and institutions (Spain), Cancer Research UK, Medical Research Council, Stroke Association, British Heart Foundation, Department of Health, Food Standards Agency, the Wellcome Trust (United Kingdom), Hellenic Ministry of Health, the Stavros Niarchos Foundation and the Hellenic Health Foundation (Greece), Italian Association for Research on Cancer, National Research Council (Italy), Dutch Ministry of Public Health, Welfare and Sports (VWS), Netherlands Cancer Registry (NKR), LK Research Funds, Dutch Prevention Funds, Dutch ZON (Zorg Onderzoek Nederland), World Cancer Research Fund (WCRF), Statistics Netherlands (The Netherlands), Swedish Cancer Society, Swedish Scientific Council, Regional Government of Skane (Sweden), The Norwegian Research Council and NordForsk (Norway) 32
42 44 0 14 WILEY-BLACKWELL HOBOKEN 111 RIVER ST, HOBOKEN 07030-5774, NJ USA 0020-7136 INT J CANCER Int. J. Cancer FEB 1 2012 130 3
622 630 10.1002/ijc.26030
http://dx.doi.org/10.1002/ijc.26030 9
Oncology Science Citation Index Expanded (SCI-EXPANDED); Social Science Citation Index (SSCI) Oncology 869MP 21412763
Bronze 2025-06-24 WOS:000298602500014
J Fung, TT; Chiuve, SE; Willett, WC; Hankinson, SE; Hu, FB; Holmes, MD Fung, Teresa T.; Chiuve, Stephanie E.; Willett, Walter C.; Hankinson, Susan E.; Hu, Frank B.; Holmes, Michelle D. Intake of specific fruits and vegetables in relation to risk of estrogen receptor-negative breast cancer among postmenopausal women BREAST CANCER RESEARCH AND TREATMENT
English Article
Breast cancer; Diet; Estrogen receptor; Incidence; Risk
PROSPECTIVE COHORT; POOLED ANALYSIS; CAROTENOIDS; QUESTIONNAIRE; TOCOPHEROLS; VALIDATION; DISEASE; CELLS In previous studies of postmenopausal women, overall intake of fruits and vegetables groups has been inversely associated with estrogen receptor-negative (ER-) breast cancer. In this analysis, we prospectively examined the associations of specific fruits and vegetables with risk of ER- postmenopausal breast cancer among 75,929 women aged 38-63 years at baseline and followed for up to 24 years. Dietary data were collected seven times during this period. Cox proportional hazard models were used, adjusting for potential confounders, including a modified Alternate Mediterranean Diet score. We ascertained 792 incident cases of ER- postmenopausal breast cancer. The multivariate relative risk (RR) for every 2 servings/week consumption for total berries was 0.82 (95 % CI = 0.71-0.96, p = 0.01), and the RR for women who consumed at least one serving of blueberries a week was 0.69 (95 % CI = 0.50-0.95, p = 0.02) compared with non-consumers. Also, the RR for consuming at least 2 servings of peaches/nectarines per week was 0.59 (95 % CI = 0.37-0.93, p = 0.02). Risk of ER- breast cancer was not associated with intakes of other specific fruits or vegetables. In conclusion, higher intake of berries and peaches was associated with lower risk of ER- breast cancer among postmenopausal women. These results are considered exploratory and need to be confirmed in further studies. [Fung, Teresa T.] Simmons Coll, Dept Nutr, Boston, MA 02115 USA; [Fung, Teresa T.;

Chiuve, Stephanie E.; Willett, Walter C.; Hankinson, Susan E.; Hu, Frank B.] Harvard Univ, Sch Publ Hlth, Dept Nutr, Boston, MA 02115 USA; [Chiuve, Stephanie E.; Willett, Walter C.; Hu, Frank B.; Holmes, Michelle D.] Harvard Univ, Sch Med, Brigham & Womens Hosp, Channing Div,Network Med,Dept Med, Boston, MA 02115 USA; [Hankinson, Susan E.] Univ Massachusetts Amherst, Sch Publ Hlth & Hlth Sci, Dept Publ Hlth, Amherst, MA 01003 USA Simmons University; Harvard University; Harvard T.H. Chan School of Public Health; Harvard University; Harvard University Medical Affiliates; Brigham & Women's Hospital; Harvard Medical School; University of Massachusetts System; University of Massachusetts Amherst
Fung, TT (corresponding author), Simmons Coll, Dept Nutr, 300 Fenway, Boston, MA 02115 USA. fung@simmons.edu Holmes, Michelle/GLS-8692-2022; Willett, Walter/E-2352-2013; Hu, Frank/C-1919-2013 Chiuve, Stephanie/0000-0002-3524-8917 NIH [CA87969, HL60712, CA95589, 1U54CA155626-01] NIH(United States Department of Health & Human ServicesNational Institutes of Health (NIH) - USA) We would like to thank the participants and staff of the Nurses' Health Study, for their valuable contributions as well as the following state cancer registries for their help: AL, AZ, AR, CA, CO, CT, DE, FL, GA, ID, IL, IN, IA, KY, LA, ME, MD, MA, MI, NE, NH, NJ, NY, NC, ND, OH, OK, OR, PA, RI, SC, TN, TX, VA, WA, WY. NIH Grants CA87969, HL60712, CA95589, and 1U54CA155626-01. 20 47 50 0 27 SPRINGER NEW YORK 233 SPRING ST, NEW YORK, NY 10013 USA 0167-6806
BREAST CANCER RES TR Breast Cancer Res. Treat. APR 2013
138 3 925 930
10.1007/s10549-013-2484-3
http://dx.doi.org/10.1007/s10549-013-2484-3 6
Oncology Science Citation Index Expanded (SCI-EXPANDED)
Oncology 131FG 23532538 Green Accepted
2025-06-24 WOS:000317977300027
J Castro-Quezada, I; Sánchez-Villegas, A; Martínez-González, MA; Salas-Salvadó, J; Corella, D; Estruch, R; Schröder, H; Alvarez-Pérez, J; Ruiz-López, MD; Artacho, R; Ros, E; Bulló, M; Sorli, JV; Fitó, M; Ruiz-Gutiérrez, V; Toledo, E; Buil-Cosiales, P; García Rodríguez, A; Lapetra, J; Pintó, X; Salaverría, I; Tur, JA; Romaguera, D; Tresserra-Rimbau, A; Serra-Majem, L
Castro-Quezada, Itandehui; Sanchez-Villegas, Almudena; Martinez-Gonzalez, Miguel A.; Salas-Salvado, Jordi; Corella, Dolores; Estruch, Ramon; Schroeder, Helmut; Alvarez-Perez, Jacqueline; Ruiz-Lopez, Maria D.; Artacho, Reyes; Ros, Emilio; Bullo, Monica; Sorli, Jose V.; Fito, Montserrat; Ruiz-Gutierrez, Valentina; Toledo, Estefania; Buil-Cosiales, Pilar; Garcia Rodriguez, Antonio; Lapetra, Jose; Pinto, Xavier; Salaverria, Itziar; Tur, Josep A.; Romaguera, Dora; Tresserra-Rimbau, Anna; Serra-Majem, Lluis PREDIMED Study InvestigatorsGlycemic index, glycemic load and invasive breast cancer incidence in postmenopausal women: The PREDIMED study EUROPEAN JOURNAL OF CANCER PREVENTION English Article
breast cancer; glycemic index; glycemic load; mediterranean diet; postmenopausal DENSITY-LIPOPROTEIN CHOLESTEROL; PHYSICAL-ACTIVITY QUESTIONNAIRE; FOOD-FREQUENCY QUESTIONNAIRE; MEDITERRANEAN DIET; CARBOHYDRATE INTAKE; DIABETES-MELLITUS; RISK; FIBER; METAANALYSIS; VALIDATION The objective of this study was to evaluate the prospective associations between dietary glycemic index (GI) and glycemic load (GL) and the risk for invasive breast cancer incidence in postmenopausal women at

high cardiovascular disease (CVD) risk. This study was conducted within the framework of the PREvencion con DIeta MEDiterranea (PREDIMED) study, a nutritional intervention trial for primary cardiovascular prevention. We included 4010 women aged between 60 and 80 years who were initially free from breast cancer but at high risk for CVD disease. Dietary information was collected using a validated 137-item food frequency questionnaire. We assigned GI values using the International Tables of GI and GL values. Cases were ascertained through yearly consultation of medical records and through consultation of the National Death Index. Only cases confirmed by results from cytology tests or histological evaluation were included. We estimated multivariable-adjusted hazard ratios for invasive breast cancer risk across tertiles of energy-adjusted dietary GI/GL using Cox regression models. We repeated our analyses using yearly repeated measures of GI/GL intakes. No associations were found between baseline dietary GI/GL and invasive breast cancer incidence. The multivariable hazard ratio and 95% confidence interval (CI) for the top tertile of dietary GI was 1.02 (95% CI: 0.42-2.46) and for dietary GL was 1.00 (95% CI: 0.44-2.30) when compared with the bottom tertile. Repeated-measures analyses yielded similar results. In sensitivity analyses, no significant associations were observed for women with obesity or diabetes. Dietary GI and GL did not appear to be associated with an increased risk for invasive breast cancer in postmenopausal women at high CVD risk. [Castro-Quezada, Itandehui; Sanchez-Villegas, Almudena; Alvarez-Perez, Jacqueline; Serra-Majem, Lluis] Univ Las Palmas Gran Canaria, Res Inst Biomed & Hlth Sci, Paseo Blas Cabrera Felipe Fisico S-N, Las Palmas Gran Canaria 35016, Spain; [Martinez-Gonzalez, Miguel A.; Salas-Salvado, Jordi; Corella, Dolores; Estruch, Ramon; Alvarez-Perez, Jacqueline; Ros, Emilio; Bullo, Monica; Sorli, Jose V.; Fito, Montserrat; Ruiz-Gutierrez, Valentina; Toledo, Estefania; Buil-Cosiales, Pilar; Garcia Rodriguez, Antonio; Lapetra, Jose; Pinto, Xavier; Salaverria, Itziar; Tur, Josep A.; Romaguera, Dora; Tresserra-Rimbau, Anna; Serra-Majem, Lluis] Spanish Govt, PREDIMED Prevent Mediterranean Diet Res Network R, Madrid, Spain; [Martinez-Gonzalez, Miguel A.; Salas-Salvado, Jordi; Corella, Dolores; Estruch, Ramon; Alvarez-Perez, Jacqueline; Ros, Emilio; Bullo, Monica; Sorli, Jose V.; Fito, Montserrat; Ruiz-Gutierrez, Valentina; Toledo, Estefania; Buil-Cosiales, Pilar; Garcia Rodriguez, Antonio; Lapetra, Jose; Pinto, Xavier; Salaverria, Itziar; Tur, Josep A.; Romaguera, Dora; Tresserra-Rimbau, Anna; Serra-Majem, Lluis] Spanish Govt, CIBER Physiopathol Obes & Nutr CIBEROBN CB06 03, Madrid, Spain; [Schroeder, Helmut] Spanish Govt, CIBER Epidemiol & Publ Hlth CIBERESP, Madrid, Spain; [Martinez-Gonzalez, Miguel A.; Toledo, Estefania] Univ Navarra, Sch Med, Dept Prevent Med & Publ Hlth, Navarra, Spain; [Buil-Cosiales, Pilar] Navarras Hlth Serv Osasunbidea, Pamplona, Spain; [Salas-Salvado, Jordi; Bullo, Monica] Univ Rovira & Virgili, Sch Med, Human Nutr Dept, Tarragona, Spain; [Corella, Dolores; Sorli, Jose V.] Univ Valencia, Sch Med, Dept Prevent Med, Valencia, Spain; [Estruch, Ramon] Hosp Clin Barcelona, Dept Internal Med, August Pi & Sunyer Biomed Res Inst IDIBAPS, Barcelona, Spain; [Schroeder, Helmut; Fito, Montserrat; Tresserra-Rimbau, Anna] Hosp del Mar Med Res Inst IMIM, Cardiovasc Risk Nutr Res Grp CARIN ULEC, Barcelona, Spain; [Ros, Emilio] Hosp Clin Barcelona, Lipid Clin, Endocrinol & Nutr Serv, August Pi & Sunyer Biomed Res Inst IDIBAPS, Barcelona, Spain; [Pinto, Xavier] Bellvitge Hosp, Internal Med Serv,

Barcelona, Spain; [Tresserra-Rimbau, Anna] Univ Barcelona, Sch Pharm, Dept Nutr & Bromatol, Barcelona, Spain; [Ruiz-Lopez, Maria D.; Artacho, Reyes] Campus Univ Cartuja, Sch Pharm, Dept Nutr & Food Sci, Granada, Spain; [Ruiz-Lopez, Maria D.] Inst Nutr & Food Technol, Granada, Spain; [Ruiz-Gutierrez, Valentina] CSIC, Inst Grasa, Grp Nutr & Lipid Metab, Seville, Spain; [Lapetra, Jose] San Pablo Hlth Ctr, Primary Care Div Sevilla, Dept Family Med, Seville, Spain; [Garcia Rodriguez, Antonio] Univ Malaga, Sch Med, Dept Prevent Med, Malaga, Spain; [Tur, Josep A.] Univ Balearic Isl, Community Nutr & Oxidat Stress Res Grp IUNICS, Lab Phys Act Sci, Mallorca, Spain; [Romaguera, Dora] Son Espases Univ Hosp, Hlth Res Inst Palma IdISPa, Mallorca, Spain Universidad de Las Palmas de Gran Canaria; CIBER - Centro de Investigacion Biomedica en Red; CIBEROBN; CIBER - Centro de Investigacion Biomedica en Red; CIBERESP; University of Navarra; Universitat Rovira i Virgili; University of Valencia; University of Barcelona; Hospital Clinic de Barcelona; IDIBAPS; Hospital del Mar Research Institute; Hospital del Mar; University of Barcelona; Hospital Clinic de Barcelona; IDIBAPS; University of Barcelona; Institut d'Investigacio Biomedica de Bellvitge (IDIBELL); Bellvitge University Hospital; University of Barcelona; Consejo Superior de Investigaciones Cientificas (CSIC); CSIC - Instituto de la Grasa (IG); Universidad de Malaga; Universitat de les Illes Balears; Hospital Universitari Son Espases; Institut Investigacio Sanitaria Illes Balears (IdISBa) Serra-Majem, L (corresponding author), Univ Las Palmas Gran Canaria, Res Inst Biomed & Hlth Sci, Paseo Blas Cabrera Felipe Fisico S-N, Las Palmas Gran Canaria 35016, Spain.

lluis.serra@ulpgc.es Tur, Josep/AAE-5748-2020; Tresserra-Rimbau, Anna/ABD-1099-2020; Romaguera, Dora/AAB-2852-2020; Estruch, Ramon/AAZ-3723-2020; Sanchez-Villegas, Almudena/T-6733-2019; Sorlí, José/L-8758-2014; Corella, Dolores/L-9888-2014; Lapetra, Jose/F-2552-2015; ÁLVAREZ-PÉREZ, JACQUELINE/V-4360-2017; Pintó, Xavier/AGI-4297-2022; Martinez-Gonzalez, Miguel/AAE-7669-2019; Serra-Majem, Lluis/I-6708-2019; Castro-Quezada, Itandehui/ABE-2687-2020; Schroder, Helmut/G-2586-2015; Bullo, Monica/F-2925-2016; Salas-Salvado, Jordi/C-7229-2017; Artacho, Reyes/B-2888-2018; Toledo, Estefania/H-6211-2014; Fito Colomer, Montse/C-1822-2012; Ruiz-Lopez, Maria Dolores/U-1925-2017

Sorli, Jose V/0000-0002-0130-2006; Castro-Quezada, Itandehui/0000-0003-4419-5690; Tresserra-Rimbau, Anna/0000-0002-7022-9041; Ros, Emilio/0000-0002-2573-1294; Schroder, Helmut/0000-0003-2231-5081; Pinto Sala, Xavier/0000-0002-2216-2444; Salaverria Lete, Itziar/0000-0002-9504-4668; Serra-Majem, Lluis/0000-0002-9658-9061; Buil-Cosiales, Pilar/0000-0002-8586-577X; Bullo, Monica/0000-0002-0218-7046; Salas-Salvado, Jordi/0000-0003-2700-7459; Artacho, Reyes/0000-0001-6405-9480; Toledo, Estefania/0000-0002-6263-4434; Romaguera, Dora/0000-0002-5762-8558; Sanchez Villegas, Almudena/0000-0001-7733-9238; Fito Colomer, Montse/0000-0002-1817-483X; Ruiz-Lopez, Maria Dolores/0000-0001-8523-0689

official funding agency for biomedical research of the Spanish government; Instituto de Salud Carlos III (ISCIII); Centro de Investigacion Biomedica en Red de Fisiopatologia de la Obesidad y Nutricion (CIBERobn) [RTIC G03/140, RTIC RD 06/0045]; Centro Nacional de Investigaciones Cardiovasculares [CNIC 06/2007]; Fondo de Investigacion Sanitaria - Fondo Europeo de Desarrollo Regional [PI04-2239, PI 05/2584, CP06/00100, PI07/0240, PI07/1138, PI07/0954, PI 07/0473, PI10/01407, PI10/02658, PI11/01647, PI11/01791, P11/02505, PI13/01090]; Ministerio de Ciencia e

Innovacion [AGL-2009-13906-C02, AGL2010-22319-C03]; Fundacion Mapfre; Agencia Canaria de Investigacion, Innovacion y Sociedad de la Informacion-EU FEDER [PI 2007/050]; Consejeria de Salud de la Junta de Andalucia [PI0105/2007]; Public Health Division of the Department of Health of the Autonomous Government of Catalonia; Generalitat Valenciana [ACOMP06109, GVACOMP2010-181, GVACOMP2011-151, CS2010-AP-111, CS2011-AP-042]; Regional Government of Navarra [P27/2011]; Regional Government of the Balearic Islands; FEDER funds [35/2011]; Consejo Nacional de Ciencia y Tecnologia de Mexico (CONACYT), Secretaria de Educacion Publica (SEP), Mexican Government official funding agency for biomedical research of the Spanish government; Instituto de Salud Carlos III (ISCIII)(Instituto de Salud Carlos IIISpanish Government); Centro de Investigacion Biomedica en Red de Fisiopatologia de la Obesidad y Nutricion (CIBERobn); Centro Nacional de Investigaciones Cardiovasculares; Fondo de Investigacion Sanitaria - Fondo Europeo de Desarrollo Regional; Ministerio de Ciencia e Innovacion(Spanish GovernmentInstituto de Salud Carlos III); Fundacion Mapfre; Agencia Canaria de Investigacion, Innovacion y Sociedad de la Informacion-EU FEDER; Consejeria de Salud de la Junta de Andalucia(Junta de Andalucia); Public Health Division of the Department of Health of the Autonomous Government of Catalonia; Generalitat Valenciana(Center for Forestry Research & Experimentation (CIEF)); Regional Government of Navarra; Regional Government of the Balearic Islands; FEDER funds(European Union (EU)); Consejo Nacional de Ciencia y Tecnologia de Mexico (CONACYT), Secretaria de Educacion Publica (SEP), Mexican Government This report has been supported by the official funding agency for biomedical research of the Spanish government, Instituto de Salud Carlos III (ISCIII), through grants provided to research networks specifically developed for the trial (RTIC G03/140, to R. E.; RTIC RD 06/0045, to M.A.M.-G.) and through Centro de Investigacion Biomedica en Red de Fisiopatologia de la Obesidad y Nutricion (CIBERobn); Centro Nacional de Investigaciones Cardiovasculares (CNIC 06/2007); Fondo de Investigacion Sanitaria - Fondo Europeo de Desarrollo Regional (PI04-2239, PI 05/2584, CP06/00100, PI07/0240, PI07/1138, PI07/0954, PI 07/0473, PI10/01407, PI10/02658, PI11/01647, PI11/01791, P11/02505 and PI13/01090); Ministerio de Ciencia e Innovacion (AGL-2009-13906-C02 and AGL2010-22319-C03); Fundacion Mapfre 2010; Agencia Canaria de Investigacion, Innovacion y Sociedad de la Informacion-EU FEDER (PI 2007/050); Consejeria de Salud de la Junta de Andalucia (PI0105/2007); Public Health Division of the Department of Health of the Autonomous Government of Catalonia; Generalitat Valenciana (ACOMP06109, GVACOMP2010-181, GVACOMP2011-151, CS2010-AP-111, and CS2011-AP-042); Regional Government of Navarra (P27/2011); Regional Government of the Balearic Islands and FEDER funds (35/2011), and I.C.-Q. is currently receiving a grant from Consejo Nacional de Ciencia y Tecnologia de Mexico (CONACYT), Secretaria de Educacion Publica (SEP), Mexican Government. 50 16 16 0 15
LIPPINCOTT WILLIAMS & WILKINS PHILADELPHIA TWO COMMERCE SQ, 2001 MARKET ST, PHILADELPHIA, PA 19103 USA 0959-8278
1473-5709 EUR J CANCER PREV Eur. J. Cancer Prev.
NOV 2016 25 6 524 532
10.1097/CEJ.0000000000000209
http://dx.doi.org/10.1097/CEJ.0000000000000209
9 Oncology Science Citation Index Expanded (SCI-

EXPANDED) Oncology DZ6VM 26633163 2025-06-24 WOS:000386000700006
J Cantalini, S; Guetto, R; Panichella, N
Cantalini, Stefano; Guetto, Raffaele; Panichella, Nazareno
Ethnic Wage Penalty and Human Capital Transferability: A Comparative Study of Recent Migrants in 11 European Countries INTERNATIONAL MIGRATION REVIEW
English Article ethnic penalty; labor market; Europe LABOR-MARKET; OCCUPATIONAL INTEGRATION; NONWESTERN IMMIGRANTS; SAMPLE SELECTION; CLASS ATTAINMENT; SOCIAL-MOBILITY; EDUCATION; EARNINGS; UNEMPLOYMENT; ASSIMILATION This article examines the ethnic wage penalty among migrants in 11 Western European countries. It aims to extend the literature on the models of migrant occupational inclusion in European labor markets by studying the wage gap and to disentangle whether the gross wage penalty experienced by foreign-born residents can be explained by human capital-related factors and/or by migrants' occupational segregation. Estimating probit models with sample selection on European Labour Force Survey data (2009-2016), we find that both male and female migrants experienced a larger gross wage penalty in Southern Europe, where they had lower education levels and faced stronger occupational segregation. In the other countries under study, we find a smaller gross wage penalty among foreign-born women. Results show that migrants from Eastern Europe were not systematically less penalized than migrants from Africa, Asia, and Latin America, except for men in Italy and Greece. Wage penalties were higher among tertiary-educated migrants, compared to their less-educated counterparts, only in Mediterranean countries, where the former were mainly concentrated at the bottom of the occupational structure. Finally, the acquisition of the highest education after migration reduced migrants' wage penalty, thanks to a better match between educational credentials and job allocation, especially in Southern Europe. Focusing on the ethnic wage penalty and on both human capital- and occupation-related factors of ethnic penalization highlights cross-country differences not yet explored by existing comparative research, allowing a new and more comprehensive picture of migrants' penalization in Europe. [Cantalini, Stefano] Stockholm Univ, Stockholm, Sweden; [Guetto, Raffaele] Univ Florence, Florence, Italy; [Panichella, Nazareno] Univ Milan, Via Conservatorio 7, Milan, Italy Stockholm University; University of Florence; University of Milan Panichella, N (corresponding author), Univ Milan, Via Conservatorio 7, Milan, Italy.
nazareno.panichella@unimi.itGuetto, Raffaele/AAB-3072-2020; PANICHELLA, NAZARENO/E-7230-2017 GUETTO, RAFFAELE/0000-0001-8052-9809; PANICHELLA, NAZARENO/0000-0002-7326-6817
74 14 14 1 17 SAGE PUBLICATIONS INC THOUSAND OAKS 2455 TELLER RD, THOUSAND OAKS, CA 91320 USA
0197-9183 1747-7379 INT MIGR REV Int. Migr. Rev.
MAR 2023 57 1 328 356
01979183221099481 10.1177/01979183221099481
http://dx.doi.org/10.1177/01979183221099481 MAY 2022
29 Demography Social Science Citation Index (SSCI)
Demography 9M8PU 2025-06-24
WOS:000798940800001
J Osterman, AL; Winer, RL; Gottlieb, GS; Sy, MP; Ba, S; Dembele, B; Toure, P; Dem, A; Seydi, M; Sall, F; Sow, PS; Kiviat, NB; Hawes, SE Osterman, Allison L.; Winer,

Rachel L.; Gottlieb, Geoffrey S.; Sy, Marie-Pierre; Ba, Selly; Dembele, Birama; Toure, Papa; Dem, Ahmadou; Seydi, Moussa; Sall, Fatima; Sow, Papa Salif; Kiviat, Nancy B.; Hawes, Stephen E.
Female genital mutilation and noninvasive cervical abnormalities and invasive cervical cancer in Senegal, West Africa: A retrospective study INTERNATIONAL JOURNAL OF CANCER English Article
cervical cancer; female genital mutilation; sub-Saharan Africa; Senegal HUMAN-PAPILLOMAVIRUS INFECTION; RISK; INFLAMMATION; COFACTORS; ETIOLOGY; WOMEN Female genital mutilation or cutting (FGM/C) is a traditional practice that affects a significant portion of women in sub-Saharan Africa, Egypt, areas of the Middle East and some countries in Asia. While clinical and epidemiological studies have established a close association between inflammation and carcinogenesis, particularly in epithelial cancers, the relationship between FGM/C and cervical cancer is not well known. We performed a secondary analysis using combined data from six research studies conducted in and around Dakar, Senegal from 1994 to 2012. Study subjects included both asymptomatic women who presented to outpatient clinics but were screened for cervical cancer, and women with cancer symptoms who were referred for cervical cancer treatment. We used unconditional logistic regression to estimate adjusted pooled odds ratios (ORs) and 95% confidence intervals (CI) for associations between FGM/C and (1) Invasive cervical cancer (ICC) and (2) noninvasive cervical abnormalities. After adjusting for confounding, women with ICC were 2.50 times more likely to have undergone FGM/C than women without cervical abnormalities (95% CI, 1.28-4.91). Restricting to HPV-positive women increased the strength of the association (OR = 4.23; 95% CI 1.73-10.32). No significant associations between FGM/C and noninvasive cervical abnormalities were observed, except in commercial sex workers with FGM/C (OR = 2.01; 95% CI 1.19-3.40). The potential increased risk for ICC suggested by our study warrants further examination. Study results may impact cancer prevention efforts in populations where FGM/C is practiced and draw awareness to the additional health risks associated with FGM/C. [Osterman, Allison L.; Gottlieb, Geoffrey S.; Hawes, Stephen E.] Univ Washington, Dept Global Hlth, Seattle, WA 98195 USA; [Winer, Rachel L.; Hawes, Stephen E.] Univ Washington, Dept Epidemiol, 1959 NE Pacific St,Box 357236, Seattle, WA 98195 USA; [Gottlieb, Geoffrey S.] Univ Washington, Dept Med, Seattle, WA USA; [Sy, Marie-Pierre; Ba, Selly; Dembele, Birama; Toure, Papa; Dem, Ahmadou; Seydi, Moussa; Sall, Fatima; Sow, Papa Salif] Ctr Hosp Univ Fann, Dakar, Senegal; [Kiviat, Nancy B.] Univ Washington, Dept Pathol, Seattle, WA 98195 USA
University of Washington; University of Washington Seattle; University of Washington; University of Washington Seattle; University of Washington; University of Washington Seattle; University of Washington; University of Washington Seattle
Hawes, SE (corresponding author), Univ Washington, Dept Epidemiol, 1959 NE Pacific St,Box 357236, Seattle, WA 98195 USA.
hawes@uw.edu National Institute of Allergy and Infectious Diseases [CA115713, CA111187, AI60466, AI48470, DE12925, CA62801]; National Institute of Dental and Craniofacial Research; National Cancer Institute; Abbott Molecular Diagnostics; ViiV Healthcare; Cerus Corporation; Janssen Pharmaceutica; Merck Co., Inc.; Alere Technologies; Gilead Sciences; Bill and Melinda Gates Foundation; University of Washington; US National Institutes

of Health National Institute of Allergy and Infectious Diseases(United States Department of Health & Human ServicesNational Institutes of Health (NIH) - USANIH National Institute of Allergy & Infectious Diseases (NIAID)); National Institute of Dental and Craniofacial Research(United States Department of Health & Human ServicesNational Institutes of Health (NIH) - USANIH National Institute of Dental & Craniofacial Research (NIDCR)); National Cancer Institute(United States Department of Health & Human ServicesNational Institutes of Health (NIH) - USANIH National Cancer Institute (NCI)); Abbott Molecular Diagnostics; ViiV Healthcare; Cerus Corporation; Janssen Pharmaceutica(Johnson & JohnsonJohnson & Johnson USAJanssen Biotech Inc); Merck Co., Inc.(Merck & Company); Alere Technologies; Gilead Sciences(Gilead Sciences); Bill and Melinda Gates Foundation(Bill & Melinda Gates Foundation); University of Washington(University of Washington); US National Institutes of Health(United States Department of Health & Human ServicesNational Institutes of Health (NIH) - USA) Grant sponsor: National Institute of Allergy and Infectious Diseases; Grant numbers: CA115713, CA111187, AI60466, AI48470, DE12925, CA62801; Grant sponsor: National Institute of Dental and Craniofacial Research; Grant sponsor: The National Cancer Institute; Grant sponsor: Abbott Molecular Diagnostics (GSG); Grant sponsor: ViiV Healthcare; Grant sponsor: Cerus Corporation; Grant sponsor: Janssen Pharmaceutica; Grant sponsor: Merck & Co., Inc.; Grant sponsor: Alere Technologies; Grant sponsor: Gilead Sciences; Grant sponsor: Bill and Melinda Gates Foundation; Grant sponsor: University of Washington; Grant sponsor: The US National Institutes of Health 27 12 13 0 13 WILEY HOBOKEN 111 RIVER ST, HOBOKEN 07030-5774, NJ USA 0020-7136 1097-0215 INT J CANCER Int. J. Cancer MAR 15 2019 144 6 1302 1312 10.1002/ijc.31829 http://dx.doi.org/10.1002/ijc.31829 11 Oncology Science Citation Index Expanded (SCI-EXPANDED) Oncology HM2TF 30144025 Bronze 2025-06-24 WOS:000459321900010

J Crujeiras, AB; Cueva, J; Vieito, M; Curiel, T; López-López, R; Pollán, M; Casanueva, FF Crujeiras, A. B.; Cueva, J.; Vieito, M.; Curiel, T.; Lopez-Lopez, R.; Pollan, M.; Casanueva, F. F. Association of breast cancer and obesity in a homogeneous population from Spain JOURNAL OF ENDOCRINOLOGICAL INVESTIGATION English Article Adipokines; breast cancer incidence; dietary patterns; obesity OVERWEIGHT; DIET; WEIGHT; FOOD; COHORT; WOMEN; RISK; SIZE Objective: To evaluate for the first time in Spain if the association between obesity and breast cancer prognosis is similar to that reported in other countries with non Mediterranean dietary patterns. Methods: Weight and height and other variables of interest, tumor characteristics and current clinical status 3 yr after diagnosis were retrieved from medical files of breast cancer women diagnosed during 2006. A total of 159 cases with complete information were studied and categorized according to the World Health Organization criteria in normal-/under-weight, overweight, and obese. Results: Among breast cancer patients, 70.4% were classified as overweight/obese and 29.6% as normal weight. Prevalence of obesity was high (38.4%) in comparison with information reported for healthy women of the same

region (27.11%) and was higher among post-menopausal patients and in women with low level of alcohol and tobacco consumption. Moreover, overweight/obese cases (79.5%) tended to have more often human epidermal growth factor receptor 2 status negative when compared with those with normal weight (70.2%; p=0.097) and the survival curves tended to be influenced by body mass index although without statistical significance. Conclusions: Overweight/obesity in a Mediterranean country is highly prevalent among breast cancer patients. Our results support a putative influence of obesity per se and not the alimentary patterns as a prognostic factor in breast cancer patients justifying the need to perform larger prospective studies. (J. Endocrinol. Invest. 35: 681-685, 2012) (C)2012, Editrice Kurt's [Crujeiras, A. B.; Casanueva, F. F.] Complejo Hosp Santiago de Compostela CHUS, IDIS, Lab Mol & Cellular Endocrinol, Santiago De Compostela, Spain; [Crujeiras, A. B.; Casanueva, F. F.] Inst Salud Carlos III, CIBER Fisiopatol Obesidad & Nutr CIBERobn, Madrid, Spain; [Cueva, J.; Vieito, M.; Curiel, T.; Lopez-Lopez, R.] Complejo Hosp Santiago de Compostela CHUS, Dept Med Oncol, Santiago De Compostela, Spain; [Pollan, M.] Natl Ctr Epidemiol, Canc & Environm Epidemiol Unit, Madrid, Spain; [Pollan, M.] Inst Salud Carlos III, CIBER Epidemiol & Salud Publ CIBERESP, Madrid, Spain Complexo Hospitalario Universitario de Santiago de Compostela; CIBER - Centro de Investigacion Biomedica en Red; CIBEROBN; Instituto de Salud Carlos III; Complexo Hospitalario Universitario de Santiago de Compostela; CIBER - Centro de Investigacion Biomedica en Red; CIBERESP; Instituto de Salud Carlos III Crujeiras, AB (corresponding author), Complejo Hosp Santiago CHUS, Area Mol & Cellular Endocrinol, Inst Invest Sanitaria, Lab 2, C Choupana S-N, Santiago De Compostela 15706, Spain.
anabelencrujeiras@hotmail.com Pollan, Marina/M-3259-2014; Crujeiras, Ana B/ABA-8866-2021 Pollan, Marina/0000-0002-4328-1565; Vieito, Maria/0000-0001-7531-343X; Crujeiras, Ana B/0000-0003-4392-0301; Lopez Lopez, Rafael/0000-0003-1315-655X
Xunta de Galicia (INCITE); Fundacion Mutua Madrilena; CIBERobn, an ISCIII iniciative; Instituto de Salud Carlos III
Xunta de Galicia (INCITE)(Xunta de Galicia); Fundacion Mutua Madrilena(Instituto de Salud Carlos III); CIBERobn, an ISCIII iniciative; Instituto de Salud Carlos III(Instituto de Salud Carlos IIISpanish Government) This work has been supported by Xunta de Galicia (INCITE 2009), Fundacion Mutua Madrilena (FMMA2010) and CIBERobn, an ISCIII iniciative. AB Crujeiras is funded by the Instituto de Salud Carlos III through a research-staff contract "Sara Borrell" 2009. 33 15 15 0
5 SPRINGER NEW YORK 233 SPRING ST, NEW YORK, NY 10013 USA 0391-4097 1720-8386 J ENDOCRINOL INVEST J. Endocrinol. Invest. JUL-AUG 2012 35 7
681 685 10.3275/8370
http://dx.doi.org/10.3275/8370 5
Endocrinology & Metabolism Science Citation Index Expanded (SCI-EXPANDED) Endocrinology & Metabolism 045PI 22522745
2025-06-24 WOS:000311708900013
J Al-Kuraya, K; Siraj, AK; Bavi, P; Al-Jommah, N; Ezzat, A; Sheikh, S; Amr, S; Al-Dayel, F; Simon, R; Guido, S
Al-Kuraya, K; Siraj, AK; Bavi, P; Al-Jommah, N; Ezzat, A; Sheikh, S; Amr, S; Al-Dayel, F; Simon, R; Guido, S High epidermal growth factor receptor amplification rate but tow mutation frequency in Middle East lung cancer population HUMAN

PATHOLOGY English Article
EGFR; epidermal growth factor receptor; mutation; amplification; gefitinib; Middle East; lung cancer GENE AMPLIFICATION; BREAST-CANCER; TISSUE MICROARRAYS; PROTEIN EXPRESSION; EGFR INHIBITORS; GEFITINIB; OVEREXPRESSION; CARCINOMAS; IMPACT Epidermal growth factor receptor (EGFR) exon 18-21 mutations were shown to be highly predictive of response to gefitinib (Iressa) therapy in lung cancer. Studies on Western and Japanese lung cancers have indicated substantial differences in the EGFR mutation frequency between these populations. To investigate the prevalence of EGFR in another distinct ethnic group, EGFR alterations were studied in 47 consecutive non small cell lung cancers from Saudi Arabia by immunohistochemistry, fluorescence in situ hybridization, and DNA sequencing. Detectable EGFR expression was seen in 69.8% of 43 interpretable cancers. Epidermal growth factor receptor amplification, present in 15.3% of 39 analyzable cancers, was strongly associated with high levels of EGFR expression (P = .0047). Only 1 exon 18-21 mutation was seen among 34 lung cancers that could be successfully sequenced. It is concluded that EGFR exon 18-21 mutations are rare in Middle East patients with lung cancer and occur in a similar range as in Western patients. The remarkable high rate of EGFR gene amplifications could potentially facilitate studies on the predictive role of gene copy number changes for response to anti-EGFR therapies in Middle East patient sets. (c) 2006 Elsevier Inc. All rights reserved. Univ Hamburg Eppendorf, Med Ctr, Dept Pathol, D-20246 Hamburg, Germany; King Faisal Specialist Hosp & Res Ctr, Riyadh 11211, Saudi Arabia
University of Hamburg; University Medical Center Hamburg-Eppendorf; King Faisal Specialist Hospital & Research Center
Simon, R (corresponding author), Univ Hamburg Eppendorf, Med Ctr, Dept Pathol, D-20246 Hamburg, Germany. Siraj, Abdul/IQW-1179-2023; Binjumah, Naif/JCE-8161-2023; Alkuraya, Khawla/AFQ-7946-2022; BAVI, PRASHANT/B-2123-2009 Simon, Ronald/0000-0003-0158-4258; Al-Jomah, Naif/0000-0001-6653-2403; Al Dayel, Fouad/0000-0001-6175-9051; Alkuraya, Khawla/0000-0002-4126-3419; BAVI, PRASHANT/0000-0002-7711-2911
21 19 22 0 0 W B SAUNDERS CO-ELSEVIER INC
PHILADELPHIA INDEPENDENCE SQUARE WEST CURTIS CENTER, STE 300, PHILADELPHIA, PA 19106-3399 USA 0046-8177 HUM PATHOL Hum. Pathol. APR 2006 37 4
453 457 10.1016/j.humpath.2005.12.004
http://dx.doi.org/10.1016/j.humpath.2005.12.004
5 Pathology Science Citation Index Expanded (SCI-EXPANDED) Pathology 031TR 16564920 2025-06-24 WOS:000236727500010
S Berrino, F; Villarini, A; De Petris, M; Raimondi, M; Pasanisi, P Bradlow, HL; Carruba, G Berrino, Franco; Villarini, Anna; De Petris, Michela; Raimondi, Milena; Pasanisi, Patrizia Adjuvant diet to improve hormonal and metabolic factors affecting breast cancer prognosis
ESTROGENS AND HUMAN DISEASESAnnals of the New York Academy of Sciences English Article; Proceedings Paper Meeting on Estrogens and Human Diseases MAY 15-21, 2006 Erice, ITALY
Ettore Majorana Fdn, Ctr Sci Culture breast cancer; hormonal and metabolic factors; diet GROWTH-FACTOR-I; DIANA RANDOMIZED-TRIAL; BODY-MASS INDEX; PREMENOPAUSAL WOMEN; SEX-HORMONES; POSTMENOPAUSAL WOMEN; BINDING-PROTEINS; WEIGHT CHANGE;

IGF-I; RISK Western lifestyle, characterized by reduced physical activity and a diet rich in fat, refined carbohydrates, and animal protein is associated with high prevalence of overweight, metabolic syndrome, insulin resistance, and high plasma levels of several growth factors and sex hormones. Most of these factors are associated with breast cancer risk and, in breast cancer patients, with increased risk of recurrences. Recent trials have proven that such a metabolic and endocrine imbalance can be favorably modified through comprehensive dietary modification, shifting from Western to Mediterranean and macrobiotic diet. Ist Nazl Tumori, Dept Prevent & Predict Med, I-20133 Milan, Italy Fondazione IRCCS Istituto Nazionale Tumori Milan Berrino, F (corresponding author), Ist Nazl Tumori, Dept Prevent & Predict Med, Via Venezian 1, I-20133 Milan, Italy. berrino@istitutotumori.mi.it Berrino, Franco/AAC-2364-2020; Villarini, Anna/K-2838-2017; Pasanisi, Patrizia/F-4908-2017 Berrino, Franco/0000-0002-4858-1866; Villarini, Anna/0000-0002-5789-6587; Pasanisi, Patrizia/0000-0001-6278-3491 44 27 28 0 12
WILEY-BLACKWELL MALDEN COMMERCE PLACE, 350 MAIN STREET, MALDEN 02148, MA USA 0077-8923 978-1-57331-669-9 ANN NY ACAD SCI Ann.NY Acad.Sci. 2006 1089
110 118 10.1196/annals.1386.023
http://dx.doi.org/10.1196/annals.1386.023 9
Oncology; Endocrinology & Metabolism; Multidisciplinary Sciences Conference Proceedings Citation Index - Science (CPCI-S); Science Citation Index Expanded (SCI-EXPANDED) Oncology; Endocrinology & Metabolism; Science & Technology - Other Topics
BFU92 17261760 2025-06-24
WOS:000244736800009

J Bozorgi, A; Khazaei, F; Bozorgi, M; Khazaei, M
Bozorgi, Azam; Khazaei, Fatemeh; Bozorgi, Maryam; Khazaei, Mozafar In Vitro Stem Cell Isolation from Human Breast Cancer MIDDLE EAST JOURNAL OF CANCER
English Article Breast cancer; Cancer stem cells; Isolation PROSPECTIVE IDENTIFICATION; TUMOR; ANTIGENS; SOX2; IRAN Background: Breast cancer (BC) is the most prevalent cancer among women worldwide with significant incidence and death rates. Nowadays, researchers hold that tumor formation, failure in therapy, and disease progression are all related to the presence of a small fraction of cancer cells with self-renewal capability known as "breast cancer stem cells" (BCSCs). Therefore, the study of this cancer cell population can be conducive to eradicating the tumor. The objective of the present study was to survey the existence and in vitro isolation of human BCSCs. Method: An in vitro research study was conducted under controlled laboratory settings to isolate, enrich, and identify breast cancer stem cells. Briefly, fresh breast tumors were carried to the lab immediately after surgery, followed by mechanical and enzymatic digestion (2 mg/ml collagenase I). Then, digested samples were passed through cell strainers (70 and 40 mu m), and obtained cell suspension was cultured under the serum-free medium supplemented with growth factors for 21 days. The expression of cluster of differentiation 44 (CD44) and cluster of differentiation 24 (CD24) surface markers was assessed using immunocytochemistry, and stem cell gene expression was analyzed via RT-PCR. Results: BCSCs were able to survive in serum-free conditions and form floating spheres in vitro. Cells obtained from mammospheres expressed CD44 as the

membranous and cytoplasmic pattern while CD24 expression was negative. Also, octameter-binding transcription factor 4 and SOX2 gene expression was observed in BCSCs. Conclusion: The presence of stem cells was confirmed in Iranian women BC, and an efficient in vitro mammosphere culture model was used to enrich and propagate BCSCs. In our opinion, this in vitro model could be a suitable method for isolating and enriching BCSCs. [Bozorgi, Azam; Khazaei, Fatemeh; Bozorgi, Maryam; Khazaei, Mozafar] Kermanshah Univ Med Sci, Fertil & Infertil Res Ctr, Hlth Technol Inst, Kermanshah, Iran Kermanshah University of Medical Sciences
Khazaei, M (corresponding author), Kermanshah Univ Med Sci, Fertil & Infertil Res Ctr, Hlth Technol Inst, Kermanshah, Iran.
mkhazaei1345@yahoo.com Khazaei, Mozafar/K-1615-2017 Bozorgi Zarrini, Azam/0000-0003-3274-6125 KermanshahUniversity of Medical Sciences [93521] KermanshahUniversity of Medical Sciences(Kermanshah University of Medical Sciences) This study was granted by KermanshahUniversity of Medical Sciences as an MSc. thesis project (No: 93521) conducted in the Fertility and Infertility Research Center. 34 0 0 2 5
SHIRAZ UNIV MEDICAL SCIENCES SHIRAZ NEMAZEE HOSPITAL, SHIRAZ, 71934, IRAN 2008-6709 2008-6687 MIDDLE EAST J CANCER Middle East J. Cancer JUL 2021 12 3
383 390 10.30476/mejc.2021.83672.1181
http://dx.doi.org/10.30476/mejc.2021.83672.1181
8 Oncology Emerging Sources Citation Index (ESCI)
Oncology UD5WW 2025-06-24
WOS:000687277900008
J Akin, H; Tözün, N Akin, Hakan; Tozun, Nurdan Diet, Microbiota, and Colorectal Cancer
JOURNAL OF CLINICAL GASTROENTEROLOGY English
Article colorectal cancer; microbiota; diet; dysbiosis; probiotics GUT MICROBIOTA; INTESTINAL MICROBIOTA; MEDITERRANEAN DIET; PREVENTION; METABOLITES; MICROFLORA; PROBIOTICS; CULTURE; DISEASE; HEALTH Colorectal cancer (CRC) is the third most common cancer in the world causing nearly 500,000 deaths every year. In addition to genetic background, environmental factors including diet and lifestyle are accepted as major contributors to adenoma and CRC development. Lifestyle factors include high BMI, obesity, and reduced physical activity. Growing interest and accumulating data on human microbiota implicate that host-microbe interplay has an important role in the development of metabolic, neoplastic, and inflammatory diseases. Findings from recent studies suggest that colon cancer risk is determined by the interaction between diet and gut microbiota. Dietary changes affect gut microbiota and conversely microbiota mediates the generation of dietary factors triggering colon cancer. Identification of the microbial communities associated with carcinogenesis is of crucial importance. Nowadays, with the evolvement of culture-independent molecular techniques, it has become possible to identify main bacterial species in healthy individuals, inflammatory conditions, and CRC. Some recent studies have shown the differences in intestinal microbiota between colon cancer patients and healthy individuals. Animal studies have provided a better understanding of interaction between pathobionts and symbionts in the development of colon cancer. There is no single causative organism identified in CRC; however, there is strong evidence that reduction of protective bacteria, increase in some bacteria (ie, fusobacterium members;

Bacteroides/Prevotella), and age-related changes in microbiota have an impact on adenoma or cancer development. Future studies will enable us to understand procarcinogenic and anticarcinogenic mechanisms and give insights to rational manipulation of the microbiota with prebiotics, probiotics, or dietary modifications.

[Akin, Hakan] Marmara Univ, Sch Med, Marmara Univ Inst Gastroenterol, Dept Gastroenterol, Istanbul, Turkey; [Tozun, Nurdan] Acibadem Univ, Sch Med, Acibadem Kozyatagi Hosp, Dept Gastroenterol, TR-34742 Istanbul, Turkey Marmara University; Acibadem Hospitals Group; Acibadem Hastaneleri; Acibadem University Tözün, N (corresponding author), Acibadem Univ, Sch Med, Acibadem Kozyatagi Hosp, Dept Gastroenterol, Inonu Cadd Okur Sok 20, TR-34742 Istanbul, Turkey.

nurdan.tozun@acibadem.edu.trTozun, Nurdan/D-6984-2015; AKIN, HAKAN/AAV-6581-2020 23 61 75

1 88 LIPPINCOTT WILLIAMS & WILKINS PHILADELPHIA

TWO COMMERCE SQ, 2001 MARKET ST, PHILADELPHIA, PA 19103 USA

0192-0790 1539-2031 J CLIN GASTROENTEROL J. Clin. Gastroenterol. NOV-DEC 2014 48 1

S67 S69 10.1097/MCG.0000000000000252

http://dx.doi.org/10.1097/MCG.0000000000000252

3 Gastroenterology & Hepatology Science Citation Index Expanded (SCI-EXPANDED) Gastroenterology & Hepatology

AX9UA 25291132 Bronze 2025-06-24

WOS:000347246300017

J Velazquez, FN; Viscardi, V; Montemage, J; Zhang, LQ; Trocchia, C; Delamont, MM; Ahmad, R; Hannun, YA; Obeid, LM; Snider, AJ Velazquez, Fabiola N.; Viscardi, Valentina; Montemage, Julia; Zhang, Leiqing; Trocchia, Carolena; Delamont, Megan M.; Ahmad, Rasheed; Hannun, Yusuf A.; Obeid, Lina M.; Snider, Ashley J. A Milk-Fat Based Diet Increases Metastasis in the MMTV-PyMT Mouse Model of Breast CancerNUTRIENTS English Article

diet; fatty acid; breast cancer; lung metastasis MAMMARY TUMORIGENESIS; MEDITERRANEAN DIET; RISK; ACIDS; OBESITY; HEALTH; ALPHA; WOMEN; ASSOCIATION; PATTERNS A high-fat diet (HFD) and obesity are risk factors for many diseases including breast cancer. This is particularly important with close to 40% of the current adult population being overweight or obese. Previous studies have implicated that Mediterranean diets (MDs) partially protect against breast cancer. However, to date, the links between diet and breast cancer progression are not well defined. Therefore, to begin to define and assess this, we used an isocaloric control diet (CD) and two HFDs enriched with either olive oil (OOBD, high in oleate, and unsaturated fatty acid in MDs) or a milk fat-based diet (MFBD, high in palmitate and myristate, saturated fatty acids in Western diets) in a mammary polyomavirus middle T antigen mouse model (MMTV-PyMT) of breast cancer. Our data demonstrate that neither MFBD or OOBD altered the growth of primary tumors in the MMTV-PyMT mice. The examination of lung metastases revealed that OOBD mice exhibited fewer surface nodules and smaller metastases when compared to MFBD and CD mice. These data suggest that different fatty acids found in different sources of HFDs may alter breast cancer metastasis.

[Velazquez, Fabiola N.; Viscardi, Valentina; Montemage, Julia; Zhang, Leiqing; Trocchia, Carolena; Hannun, Yusuf A.; Obeid, Lina M.; Snider, Ashley J.] SUNY Stony Brook, Dept Med, Stony Brook, NY 11794 USA; [Velazquez, Fabiola N.; Viscardi,

Valentina; Montemage, Julia; Zhang, Leiqing; Trocchia, Carolena; Hannun, Yusuf A.; Obeid, Lina M.; Snider, Ashley J.] SUNY Stony Brook, Canc Ctr, Stony Brook, NY 11794 USA; [Delamont, Megan M.] Univ Arizona, Coll Agr & Life Sci, Dept Nutr Sci, Tucson, AZ 85721 USA; [Ahmad, Rasheed; Snider, Ashley J.] Dasman Diabet Inst, Immunol & Microbiol Dept, Kuwait 15462, Kuwait; [Snider, Ashley J.] Univ Arizona, BI05 Inst, 1230 N Cherry Ave,BSRL 372, Tucson, AZ 85718 USA State University of New York (SUNY) System; Stony Brook University; State University of New York (SUNY) System; Stony Brook University; University of Arizona; Dasman Diabetes Institute (DDI); University of Arizona Snider, AJ (corresponding author), SUNY Stony Brook, Dept Med, Stony Brook, NY 11794 USA.; Snider, AJ (corresponding author), SUNY Stony Brook, Canc Ctr, Stony Brook, NY 11794 USA.; Snider, AJ (corresponding author), Dasman Diabet Inst, Immunol & Microbiol Dept, Kuwait 15462, Kuwait.; Snider, AJ (corresponding author), Univ Arizona, BI05 Inst, 1230 N Cherry Ave,BSRL 372, Tucson, AZ 85718 USA.
Fabiolalloelia.Velazquez@stonybrookmedicine.edu; ValentinaViscardi7@gmail.com; julia.montemage@gmail.com; leiqing.zhang.1@stonybrookmedicine.edu; carolena.trocchia@gmail.com; delamontmegan@gmail.com; rasheed.ahmad@dasmaninstitute.org; yusuf.hannun@stonybrookmedicine.edu; lina.obeid@stonybrookmedicine.edu; ashleysnider@arizona.edu
Ahmad, Rasheed/LMP-1937-2024Velazquez, Fabiola Noelia/0000-0001-5692-848X; Snider, Ashley/0000-0002-1515-4171; Ahmad, Rasheed/0000-0001-5746-0743 National Cancer Institute [P01-CA097132] National Cancer Institute(United States Department of Health & Human ServicesNational Institutes of Health (NIH) - USANIH National Cancer Institute (NCI)) This research was funded by National Cancer Institute, Grant P01-CA097132 (YAH, LMO, AJS).
51 3 3 2 11 MDPI BASEL ST ALBAN-ANLAGE 66, CH-4052 BASEL, SWITZERLAND 2072-6643 NUTRIENTS
Nutrients JUL 2021 13 7
2431 10.3390/nu13072431
http://dx.doi.org/10.3390/nu13072431 10
Nutrition & Dietetics Science Citation Index Expanded (SCI-EXPANDED) Nutrition & Dietetics TO0CO 34371939 Green Published, gold 2025-06-24 WOS:000676591700001
J McLeod, A; Wolf, P; Chapkin, RS; Davidson, LA; Ivanov, I; Berbaum, M; Williams, LR; Gaskins, HR; Ridlon, J; Sanchez-Flack, J; Blumstein, L; Schiffer, L; Hamm, A; Cares, K; Antonic, M; Bernabe, BP; Fitzgibbon, M; Tussing-Humphreys, L
McLeod, Andrew; Wolf, Patricia; Chapkin, Robert S.; Davidson, Laurie A.; Ivanov, Ivan; Berbaum, Michael; Williams, Lauren R.; Gaskins, H. Rex; Ridlon, Jason; Sanchez-Flack, Jen; Blumstein, Lara; Schiffer, Linda; Hamm, Alyshia; Cares, Kate; Antonic, Mirjana; Bernabe, Beatriz Penalver; Fitzgibbon, Marian; Tussing-Humphreys, Lisa Design of the Building Research in CRC prevention (BRIDGE-CRC) trial: a 6-month, parallel group Mediterranean diet and weight loss randomized controlled lifestyle intervention targeting the bile acid-gut microbiome axis to reduce colorectal cancer risk among African American/Black adults with obesity TRIALS English Article
Cancer health disparities; Colorectal cancer; Mediterranean diet; Weight loss; Gut microbiome; Bile acids; Nutrition GENE-EXPRESSION PROFILES; US ADULTS; QUALITY; COLONOCYTES; COMBINATION; METABOLISM; BIOMARKERS;

FREQUENCY; SURGERY; PROTEIN Background Among all racial/ethnic groups, people who identify as African American/Blacks have the second highest colorectal cancer (CRC) incidence in the USA. This disparity may exist because African American/Blacks, compared to other racial/ethnic groups, have a higher prevalence of risk factors for CRC, including obesity, low fiber consumption, and higher intakes of fat and animal protein. One unexplored, underlying mechanism of this relationship is the bile acid-gut microbiome axis. High saturated fat, low fiber diets, and obesity lead to increases in tumor promoting secondary bile acids. Diets high in fiber, such as a Mediterranean diet, and intentional weight loss may reduce CRC risk by modulating the bile acid-gut microbiome axis. The purpose of this study is to test the impact of a Mediterranean diet alone, weight loss alone, or both, compared to typical diet controls on the bile acid-gut microbiome axis and CRC risk factors among African American/Blacks with obesity. Because weight loss or a Mediterranean diet alone can reduce CRC risk, we hypothesize that weight loss plus a Mediterranean diet will reduce CRC risk the most.Methods This randomized controlled lifestyle intervention will randomize 192 African American/Blacks with obesity, aged 45-75 years to one of four arms: Mediterranean diet, weight loss, weight loss plus Mediterranean diet, or typical diet controls, for 6 months (48 per arm). Data will be collected at baseline, mid-study, and study end. Primary outcomes include total circulating and fecal bile acids, taurine-conjugated bile acids, and deoxycholic acid. Secondary outcomes include body weight, body composition, dietary change, physical activity, metabolic risk, circulating cytokines, gut microbial community structure and composition, fecal short-chain fatty acids, and expression levels of genes from exfoliated intestinal cells linked to carcinogenesis.Discussion This study will be the first randomized controlled trial to examine the effects of a Mediterranean diet, weight loss, or both on bile acid metabolism, the gut microbiome, and intestinal epithelial genes associated with carcinogenesis. This approach to CRC risk reduction may be especially important among African American/Blacks given their higher risk factor profile and increased CRC incidence. [McLeod, Andrew; Berbaum, Michael; Sanchez-Flack, Jen; Blumstein, Lara; Schiffer, Linda; Antonic, Mirjana; Fitzgibbon, Marian; Tussing-Humphreys, Lisa] Univ Illinois Chicago UIC, Inst Hlth Res & Policy, Chicago, IL 60608 USA; [Wolf, Patricia] Purdue Univ, Dept Nutr Sci, W Lafayette, IN USA; [Chapkin, Robert S.; Davidson, Laurie A.; Ivanov, Ivan] Texas A&M Univ, Dept Nutr, Program Integrat Nutr & Complex Dis, College Stn, TX USA; [Chapkin, Robert S.; Davidson, Laurie A.; Ivanov, Ivan] Texas A&M Univ, Ctr Environm Hlth Res, College Stn, TX USA; [Ivanov, Ivan] Texas A&M Univ, Dept Vet Physiol & Pharmacol, College Stn, TX USA; [Williams, Lauren R.] Univ Illinois, Mile Sq Hlth Ctr, Chicago, IL USA; [Gaskins, H. Rex; Ridlon, Jason] Univ Illinois, Dept Anim Sci, Urbana, IL USA; [Gaskins, H. Rex; Ridlon, Jason] Univ Illinois, Div Nutr Sci, Urbana, IL USA; [Gaskins, H. Rex; Ridlon, Jason] Univ Illinois, Carl R Woese Inst Genom Biol, Urbana, IL USA; [Gaskins, H. Rex; Ridlon, Jason] Univ Illinois, Canc Ctr Illinois, Urbana, IL USA; [Gaskins, H. Rex; Ridlon, Jason] Univ Illinois, Dept Biomed & Translat Sci, Urbana, IL USA; [Gaskins, H. Rex] Univ Illinois, Dept Pathobiol, Urbana, IL USA; [Gaskins, H. Rex] Univ Illinois, Dept Pediat, Chicago, IL 60637 USA; [Sanchez-Flack, Jen; Fitzgibbon, Marian; Tussing-Humphreys,

Lisa] Univ Illinois Canc Ctr, Univ Illinois Chicago, Chicago, IL USA; [Hamm, Alyshia; Cares, Kate; Tussing-Humphreys, Lisa] Univ Illinois, Dept Kinesiol & Nutr, Chicago, IL USA University of Illinois System; University of Illinois Chicago; Purdue University System; Purdue University; Texas A&M University System; Texas A&M University College Station; Texas A&M University System; Texas A&M University College Station; Texas A&M University System; Texas A&M University College Station; University of Illinois System; University of Illinois Chicago; University of Illinois Chicago Hospital; University of Illinois System; University of Illinois Urbana-Champaign; University of Illinois System; University of Illinois Urbana-Champaign; University of Illinois System; University of Illinois Urbana-Champaign; University of Illinois System; University of Illinois Urbana-Champaign; University of Illinois System; University of Illinois Urbana-Champaign; University of Illinois System; University of Illinois Urbana-Champaign; University of Illinois System; University of Illinois Chicago; University of Illinois Chicago Hospital; University of Illinois System; University of Illinois Chicago; University of Illinois System; University of Illinois Chicago; University of Illinois Chicago Hospital Fitzgibbon, M; Tussing-Humphreys, L (corresponding author), Univ Illinois Chicago UIC, Inst Hlth Res & Policy, Chicago, IL 60608 USA. mlf@uic.edu; ltussing@uic.edu
Wolf, Patricia/KOC-7260-2024; Bernabe, Beatriz/ABH-2834-2022; Ivanov, Ivan/E-6905-2012 McLeod, Andrew/0000-0002-7067-3441; Fitzgibbon, Marian/0000-0002-7735-2165 National Cancer Institute (NCI) [R01CA250390]; NCI [T32CA057699] National Cancer Institute (NCI)(United States Department of Health & Human ServicesNational Institutes of Health (NIH) - USANIH National Cancer Institute (NCI)); NCI(United States Department of Health & Human ServicesNational Institutes of Health (NIH) - USANIH National Cancer Institute (NCI)) This trial is funded by grant R01CA250390 from the National Cancer Institute (NCI). APM is supported by T32CA057699 (NCI). The funder shall have no influence over the design, implementation, conduct, results, or interpretations of the results of this trial 100 10 10
2 19 BMC LONDON CAMPUS, 4 CRINAN ST, LONDON N1 9XW, ENGLAND 1745-6215 TRIALS Trials FEB 15 2023 24 1 113
10.1186/s13063-023-07115-4
http://dx.doi.org/10.1186/s13063-023-07115-4 17
Medicine, Research & Experimental Science Citation Index Expanded (SCI-EXPANDED) Research & Experimental Medicine
9B8KU 36793105 Green Published, gold 2025-06-24 WOS:000934981600001
J Syed, N; Chintakuntlawar, AV; Vilasini, D; Al Salami, AM; Al Hasan, R; Afrooz, I; Chandani, KU; Chandani, AU; Chehal, A
Syed, Naveed; Chintakuntlawar, Ashish Vittalrao; Vilasini, Deepti; Al Salami, Aisha Mohamed; Al Hasan, Riad; Afrooz, Imrana; Chandani, Kanishka Uttam; Chandani, Ashok Uttam; Chehal, Aref Low testing rates and high BRCA prevalence: Poly (ADP-ribose) polymerase inhibitor use in Middle East BRCA/homologous recombination deficiency-positive cancer patients WORLD JOURNAL OF CLINICAL ONCOLOGY
English Article
Homologous recombination repair; BRCA1; BRCA2; Homologous recombination deficiency; Ovarian cancer; Breast cancer; Poly (ADP-ribose) polymerase inhibitors; Olaparib; DNA double-strand

breaks OVARIAN-CANCER; MAINTENANCE; GERMLINE; MUTATION; BREAST; OLAPARIB; THERAPY BACKGROUND Poly (ADP-ribose) polymerase inhibitors (PARPis) are approved as first-line therapies for breast cancer gene (BRCA)-positive, human epidermal growth factor receptor 2-negative locally advanced or metastatic breast cancer. They are also effective for new and recurrent ovarian cancers that are BRCA- or homologous recombination deficiency (HRD)-positive. However, data on these mutations and PARPi use in the Middle East are limited. AIM To assess BRCA/HRD prevalence and PARPi use in patients in the Middle East with breast/ovarian cancer. METHODS This was a single-center retrospective study of 57 of 472 breast cancer patients tested for BRCA mutations, and 25 of 65 ovarian cancer patients tested for HRD. These adult patients participated in at least four visits to the oncology service at our center between August 2021 and May 2023. Data were summarized using descriptive statistics and compared using counts and percentages. Response to treatment was assessed using Response Evaluation Criteria in Solid Tumors criteria. RESULTS Among the 472 breast cancer patients, 12.1% underwent BRCA testing, and 38.5% of 65 ovarian cancer patients received HRD testing. Pathogenic mutations were found in 25.6% of the tested patients: 26.3% breast cancers had germline BRCA (gBRCA) mutations and 24.0% ovarian cancers showed HRD. Notably, 40.0% of gBRCA-positive breast cancers and 66.0% of HRD-positive ovarian cancers were Middle Eastern and Asian patients, respectively. PARPi treatment was used in 5 (33.3%) gBRCA-positive breast cancer patients as first-line therapy (n = 1; 7-months progression-free), for maintenance (n = 2; > 15-months progression-free), or at later stages due to compliance issues (n = 2). Four patients (66.6%) with HRD-positive ovarian cancer received PARPi and all remained progression-free. CONCLUSION Lower testing rates but higher BRCA mutations in breast cancer were found. Ethnicity reflected United Arab Emirates demographics, with breast cancer in Middle Eastern and ovarian cancer in Asian patients. [Syed, Naveed] Sheikh Shakbout Med City, Dept Hematol & Oncol, Abu Dhabi 11001, U Arab Emirates; [Chintakuntlawar, Ashish Vittalrao] Mayo Clin, Dept Oncol, Rochester, NY 55905 USA; [Vilasini, Deepti; Al Salami, Aisha Mohamed; Al Hasan, Riad; Chandani, Ashok Uttam; Chehal, Aref] Sheikh Shakbout Med City, Dept Oncol, Abu Dhabi 11001, U Arab Emirates; [Afrooz, Imrana] Clin Res Sheikh Shakbout Med City, Abu Dhabi 11001, U Arab Emirates; [Chandani, Kanishka Uttam] Landmark Med Ctr, Dept Internal Med, Woonsocket, RI 02895 USA Mayo Clinic Syed, N (corresponding author), Sheikh Shakbout Med City, Dept Hematol & Oncol, Mafraq Area, Abu Dhabi 11001, U Arab Emirates.
naveed3642003@gmail.com Syed, Naveed/ITW-1333-2023 naveed, syed/0000-0002-8172-8129 34
0 0 1 2 BAISHIDENG PUBLISHING GROUP INC PLEASANTON 7041 Koll Center Parkway, Suite 160, PLEASANTON, CA, UNITED STATES 2218-4333 WORLD J CLIN ONCOL
World J. Clin. Oncol. JUL 24 2024 15 7
10.5306/wjco.v15.i7.848
http://dx.doi.org/10.5306/wjco.v15.i7.848 12
Oncology Emerging Sources Citation Index (ESCI) Oncology
A1S5V 39071455 Green Published, gold 2025-06-24 WOS:001280403000002
J Aragón, F; Perdigón, G; de LeBlanc, AD
Aragon, Felix; Perdigon, Gabriela; de Moreno de LeBlanc,

Alejandra Modification in the diet can induce beneficial effects against breast cancer WORLD JOURNAL OF CLINICAL ONCOLOGY English Article

Breast cancer; Nutrition; Probiotic; Fermented products

The population tends to consume foods that in addition to their nutritional values can offer some benefits to their health. There are many epidemiological evidences and research studies in animal models suggesting that diet plays an important role in breast cancer prevention or progression. This review summarized some of the relevant researches about nutrition and cancer during the last years, especially in breast cancer. The analysis of probiotics and fermented products containing lactic acid bacteria in cancer prevention and/or treatment was especially discussed. It was observed that a balance of fatty acids similar to those of traditional Mediterranean diet, the consumption of fruits and vegetables, dietary fiber intake, vitamin supplementation are, along with the intake of probiotic products, the most extensively studied by the negative association to breast cancer risk. The consumption of probiotics and fermented products containing lactic acid bacteria was associated to reduce breast cancer risk in some epidemiological studies. The use of animal models showed the modulation of the host's immune response as one of the important effects associated to the benefices observed with most probiotics. However; future assays in human are very important before the medical community can accept the addition of probiotic or fermented milks containing lactic acid bacteria as supplements for cancer patients. (C) 2014 Baishideng Publishing Group Inc. All rights reserved.

[Aragon, Felix; Perdigon, Gabriela; de Moreno de LeBlanc, Alejandra] Consejo Nacl Invest Cient & Tecn, CERELA, Ctr Referencia Lactobacilos, Chacabuco 145,T4000ILC, San Miguel De Tucuman, Tucuman, Argentina; [Perdigon, Gabriela] Univ Nacl Tucuman, Catedra Inmunol, Fac Bioquim Quim & Farm, San Miguel De Tucuman, Tucuman, Argentina Consejo Nacional de Investigaciones Cientificas y Tecnicas (CONICET); Universidad Nacional de Tucuman de LeBlanc, AD (corresponding author), Consejo Nacl Invest Cient & Tecn, CERELA, Ctr Referencia Lactobacilos, Chacabuco 145,T4000ILC, San Miguel De Tucuman, Tucuman, Argentina.demoreno@cerela.org.ar de Moreno de LeBlanc, Alejandra/R-1242-2018 de Moreno de LeBlanc, Alejandra/0000-0003-0352-374X

Consejo Nacional de Investigaciones Cientificas y Tecnicas (CONICET); Argentina and Consejo de Investigacion de la Universidad Nacional de Tucuman, Argentina [CIUNT-26/D442] Consejo Nacional de Investigaciones Cientificas y Tecnicas (CONICET)(Consejo Nacional de Investigaciones Cientificas y Tecnicas (CONICET)); Argentina and Consejo de Investigacion de la Universidad Nacional de Tucuman, Argentina Supported by Consejo Nacional de Investigaciones Cientificas y Tecnicas (CONICET); Argentina and Consejo de Investigacion de la Universidad Nacional de Tucuman, Argentina, No. CIUNT-26/D442 115 25 27 0 23 BAISHIDENG PUBLISHING GROUP INC PLEASANTON 8226 REGENCY DR, PLEASANTON, CA 94588 USA2218-4333 WORLD J CLIN ONCOL World J. Clin. Oncol. AUG 10 2014 5 3 455 464 10.5306/wjco.v5.i3.455 http://dx.doi.org/10.5306/wjco.v5.i3.455 10 Oncology Emerging Sources Citation Index (ESCI) Oncology VE3FR 25114859 Green Published, hybrid 2025-06-24 WOS:000439079600022

J Masala, G; Assedi, M; Bendinelli, B; Ermini, I; Sieri, S; Grioni, S; Sacerdote, C; Ricceri, F; Panico, S; Mattiello, A; Tumino, R; Giurdanella, MC; Berrino, F; Saieva, C; Palli, D Masala, Giovanna; Assedi, Melania; Bendinelli, Benedetta; Ermini, Ilaria; Sieri, Sabina; Grioni, Sara; Sacerdote, Carlotta; Ricceri, Fulvio; Panico, Salvatore; Mattiello, Amalia; Tumino, Rosario; Giurdanella, Maria Concetta; Berrino, Franco; Saieva, Calogero; Palli, Domenico Fruit and vegetables consumption and breast cancer risk: the EPIC Italy studyBREAST CANCER RESEARCH AND TREATMENT English Article Breast cancer; Vegetables; Fruit; Longitudinal study; Risk FOOD GROUPS; DIET; METAANALYSIS; WOMEN; MICRONUTRIENTS; PROJECT; DESIGN; COHORT The role of fruit and vegetables in breast cancer (BC) development has long been debated. A large variety of vegetables and fruit are consumed by Mediterranean populations, a favourable setting for evaluating the effects of these foods. The association between vegetables and fruit consumption, overall and by specific types, and BC risk was studied in the Italian section of the European Prospective Investigation into Cancer and Nutrition study. Over 31,000 women, aged 36-64 years, recruited in five Italian centers between 1993 and 1998, were available for analyses with dietary and lifestyle information and anthropometric measurements. After a median follow-up of 11.25 years, 1,072 invasive and in situ incident BC cases were identified. Cox proportional hazard models (adjusted for education, anthropometry, reproductive history, hormone replacement therapy, physical activity, alcohol consumption and smoking habits) showed an inverse association between consumption of all vegetables and BC risk (highest vs. lowest quintile HR 0.65; 95% CI 0.53-0.81, P for trend = 0.003). According to subtypes of vegetables, an inverse association emerged for increasing consumption of leafy vegetables (highest vs. lowest quintile HR 0.70; 95% CI 0.57-0.86, P for trend = 0.0001) and fruiting vegetables (highest vs. lowest quintile HR 0.75; 95% CI 0.60-0.94, P for trend = 0.01). An inverse association also emerged with increasing consumption of raw tomatoes (P for trend = 0.03). In contrast, no association of fruit, overall or by subtypes, with BC risk was found. In this Mediterranean population, a clear protective role of increasing vegetables consumption, mainly leafy and fruiting vegetables, on BC risk emerged. [Masala, Giovanna; Assedi, Melania; Bendinelli, Benedetta; Ermini, Ilaria; Saieva, Calogero; Palli, Domenico] Canc Res & Prevent Inst ISPO, Mol & Nutr Epidemiol Unit, I-50141 Florence, Italy; [Sieri, Sabina; Grioni, Sara] Ist Nazl Tumori, Nutr Epidemiol Unit, Fdn IRCSS, I-20133 Milan, Italy; [Sacerdote, Carlotta] Azienda Sanitaria Osped Molinette San Giovanni Ba, Ctr Canc Prevent CPO Piemonte, I-10126 Turin, Italy; [Sacerdote, Carlotta; Ricceri, Fulvio] Human Genet Fdn HuGeF, I-10126 Turin, Italy; [Panico, Salvatore; Mattiello, Amalia] Univ Naples Federico II, Dept Clin & Expt Med, I-80131 Naples, Italy; [Tumino, Rosario; Giurdanella, Maria Concetta] Canc Registry ASP 7, I-97100 Ragusa, Italy; [Berrino, Franco] Ist Nazl Tumori, Fdn IRCSS, Dept Prevent & Predict Med, I-20133 Milan, Italy ISPRO Istituto per lo studio, la prevenzione e la rete oncologica; Fondazione IRCCS Istituto Nazionale Tumori Milan; A.O.U. Citta della Salute e della Scienza di Torino; AOU San Giovanni Battista-Molinette; University of Naples Federico II; Fondazione IRCCS Istituto Nazionale Tumori Milan Palli, D (corresponding author), Canc Res & Prevent Inst

ISPO, Mol & Nutr Epidemiol Unit, Ponte Nuovo Palazzina 28 A,Via Oblate 4, I-50141 Florence, Italy. d.palli@ispo.toscana.it
Saieva, Calogero/AAC-2611-2019; Berrino, Franco/AAC-2364-2020; Masala, Giovanna/AAC-5474-2022; Panico, Salvatore/K-6506-2016; Sacerdote, Carlotta/K-3611-2018; Sieri, Sabina/K-4667-2016; RICCERI, FULVIO/I-9910-2018; Mattiello, Amalia/K-5112-2016; grioni, Sara/K-5320-2016 Sacerdote, Carlotta/0000-0002-8008-5096; tumino, rosario/0000-0003-2666-414X; Sieri, Sabina/0000-0001-5201-172X; RICCERI, FULVIO/0000-0001-8749-9737; Berrino, Franco/0000-0002-4858-1866; Masala, Giovanna/0000-0002-5758-9069; Panico, Salvatore/0000-0002-5498-8312; Mattiello, Amalia/0000-0003-3676-7353; Bendinelli, Benedetta/0000-0002-4796-1517; saieva, calogero/0000-0002-0117-1608; grioni, Sara/0000-0002-5891-8426; PALLI, Domenico/0000-0002-5558-2437 Associazione Italiana per la Ricerca sul Cancro (AIRC Milan); Regione Toscana, Ministero della Salute, Rome, Italy Associazione Italiana per la Ricerca sul Cancro (AIRC Milan)(Fondazione AIRC per la ricerca sul cancro); Regione Toscana, Ministero della Salute, Rome, Italy We wish to thank all EPIC Italy participants. The EPIC Italy collaboration has been supported by the Associazione Italiana per la Ricerca sul Cancro (AIRC Milan) and Programma Integrato Oncologia (PIO-Regione Toscana, Ministero della Salute, Rome, Italy). 24 57 67 0 22 SPRINGER NEW YORK 233 SPRING ST, NEW YORK, NY 10013 USA 0167-6806 1573-7217 BREAST CANCER RES TR Breast Cancer Res. Treat. APR 2012 132 3 SI 1127 1136 10.1007/s10549-011-1939-7 http://dx.doi.org/10.1007/s10549-011-1939-7 10 Oncology Science Citation Index Expanded (SCI-EXPANDED) Oncology 933RL 22215387 2025-06-24 WOS:000303379800035
J Beg, S; Siraj, AK; Prabhakaran, S; Bu, R; Al-Rasheed, M; Sultana, M; Qadri, Z; Al-Assiri, M; Sairafi, R; Al-Dayel, F; Al-Sanea, N; Uddin, S; Al-Kuraya, KS Beg, Shaham; Siraj, Abdul K.; Prabhakaran, Sarita; Bu, Rong; Al-Rasheed, Maha; Sultana, Mehar; Qadri, Zeeshan; Al-Assiri, Mohammed; Sairafi, Rami; Al-Dayel, Fouad; Al-Sanea, Nasser; Uddin, Shahab; Al-Kuraya, Khawla S. Molecular markers and pathway analysis of colorectal carcinoma in the Middle East CANCER
English Article alternate pathway; colorectal cancer; Middle East; molecular pathways; serrated pathway; traditional pathway ISLAND METHYLATOR PHENOTYPE; BRAF MUTATIONS; MICROSATELLITE INSTABILITY; SERRATED PATHWAY; MEAT CONSUMPTION; CANCER; PATTERNS; AGE; ASSOCIATIONS; METAANALYSIS BACKGROUNDColorectal cancer (CRC) is one of the most common cancers in the world. A newly proposed integrated pathway comprising traditional, alternate, and serrated pathways by genetic and epigenetic factors was defined recently and hypothesized to play a role in the pathogenesis of CRC; however, to the authors' knowledge, there is a paucity of information regarding these proposed molecular pathways in different ethnic groups. METHODSMolecular characterization of 770 CRC specimens was performed for microsatellite instability, BRAF, and KRAS by polymerase chain reaction and 500 cases for CpG island methylator phenotype (CIMP) high phenotype by MethyLight technology. Tumors were assigned to different molecular pathways and examined for clinicopathological correlation and survival analysis. RESULTSThe traditional pathway constituted 33.4% of CRC cases, the alternate

pathway comprised 11.6%, and the serrated molecular pathway accounted for only 0.8% of Middle Eastern CRC cases. Approximately 54.2% of CRC cases did not qualify to fit into any pathway and thus were designated as an unassigned group. Molecular pathways were found to be significantly associated with tumor site and grade. A subset of cases with an uncategorized pathway demonstrated a significant survival difference (P = .0079). CONCLUSIONSThe serrated pathway was found to account for a very low percentage of the CRC patient cohort in the current study. The unassigned group accounted for the majority of Middle Eastern CRC cases, and therefore methods of CRC pathway analysis might not be applicable to this ethnic group. The current study demonstrates the need to unravel the molecular genetic basis of this disease to further subcategorize these CRC cases. It also identifies a need for further studies on different populations for a better understanding of their exact role and incidence. Cancer 2015;121:3799-3808. (c) 2015 American Cancer Society. The results of the current study indicate the need to unravel the molecular genetic basis of colorectal carcinoma to further subcategorize colorectal carcinoma cases. They also emphasize the need to perform further studies on different populations to develop a better understanding of the exact role and incidence of genetic pathways. [Beg, Shaham; Siraj, Abdul K.; Prabhakaran, Sarita; Bu, Rong; Al-Rasheed, Maha; Sultana, Mehar; Qadri, Zeeshan; Uddin, Shahab; Al-Kuraya, Khawla S.] King Faisal Specialist Hosp & Res Ctr, Human Canc Genom Res, Riyadh 11211, Saudi Arabia; [Al-Assiri, Mohammed; Sairafi, Rami] Secur Forces Hosp, Dept Surg, Riyadh, Saudi Arabia; [Al-Dayel, Fouad] King Faisal Specialist Hosp & Res Ctr, Dept Pathol, Riyadh 11211, Saudi Arabia; [Al-Sanea, Nasser] King Faisal Specialist Hosp & Res Ctr, Dept Surg, Colorectal Unit, Riyadh 11211, Saudi Arabia; [Al-Kuraya, Khawla S.] Alfaisal Univ, Coll Med, Dept Pathol, Riyadh, Saudi Arabia King Faisal Specialist Hospital & Research Center; Security Forces Hospital - Saudi Arabia; King Faisal Specialist Hospital & Research Center; King Faisal Specialist Hospital & Research Center; Alfaisal University Al-Kuraya, KS (corresponding author), King Faisal Specialist Hosp & Res Canc, Human Canc Genom Res, MBC 98-16,POB 3354, Riyadh 11211, Saudi Arabia. Kkuraya@kfshrc.edu.sa Siraj, Abdul/IQW-1179-2023; Bu, Rong/JBI-9735-2023; Sairafi, Rami/AAB-1488-2020; Qadri, Syed/KYP-5564-2024; Alkuraya, Khawla/AFQ-7946-2022; Uddin, Shahab/AFH-8541-2022; Alsanea, Nasser/J-1565-2016
Alkuraya, Khawla/0000-0002-4126-3419; Beg, Shaham/0000-0002-6068-1595; Uddin, Shahab/0000-0003-1886-6710; Alsanea, Nasser/0000-0002-6336-5942; Qadri, Syed Zeeshan/0000-0003-4026-8987; Al Dayel, Fouad/0000-0001-6175-9051; Prabhakaran, Sarita/0000-0003-3252-2519 43 20 22
0 7 WILEY HOBOKEN 111 RIVER ST, HOBOKEN 07030-5774, NJ USA 0008-543X 1097-0142 CANCER-AM CANCER SOC
Cancer NOV 1 2015 121 21 3799
3808 10.1002/cncr.29580
http://dx.doi.org/10.1002/cncr.29580 10
Oncology Science Citation Index Expanded (SCI-EXPANDED)
Oncology CU1EH 26218848 Bronze 2025-06-24 WOS:000363262100010

J Schwingshackl, L; Schwedhelm, C; Hoffmann, G; Knüppel, S; Preterre, AL; Iqbal, K; Bechthold, A; De Henauw, S; Michels, N; Devleesschauwer, B; Boeing, H; Schlesinger, S
Schwingshackl, Lukas; Schwedhelm, Carolina; Hoffmann, Georg;

Knueppel, Sven; Preterre, Anne Laure; Iqbal, Khalid; Bechthold, Angela; De Henauw, Stefaan; Michels, Nathalie; Devleesschauwer, Brecht; Boeing, Heiner; Schlesinger, Sabrina Food groups and risk of colorectal cancer INTERNATIONAL JOURNAL OF CANCER English Article

food groups; diet; meta-analysis; dose-response; colorectal cancer DOSE-RESPONSE METAANALYSIS; MEDITERRANEAN DIET; TREND ESTIMATION; CALCIUM; COLON; MECHANISMS; MICROBIOTA; MORTALITY; ADHERENCE; PATTERNS The aim of this systematic review and meta-analysis was to summarize the evidence on the relationship between intake of 12 major food groups, including whole grains, refined grains, vegetables, fruit, nuts, legumes, eggs, dairy, fish, red meat, processed meat and sugar-sweetened beverages with risk of colorectal cancer (CRC). We conducted a systematic search in PubMed and Embase for prospective studies investigating the association between these 12 food groups and risk of CRC until April 2017. Summary risk ratios (RRs) and 95% confidence intervals (95% CI) were estimated using a random effects model for high vs. low intake categories, as well as for linear and nonlinear relationships. An inverse association was observed for whole grains (RR30g/d: 0.95, 95% CI 0.93, 0.97; n=9 studies), vegetables (RR100g/d: 0.97, 95% CI 0.96, 0.98; n=15), fruit (RR100g/d: 0.97, 95% CI 0.95, 0.99; n=16) and dairy (RR200g/d: 0.93, 95% CI 0.91, 0.94; n=15), while a positive association for red meat (RR100g/d: 1.12, 95% CI 1.06, 1.19; n=21) and processed meat (RR50g/d: 1.17, 95% CI 1.10, 1.23; n=16), was seen in the linear dose-response meta-analysis. Some evidence for nonlinear relationships was observed between vegetables, fruit and dairy and risk of colorectal cancer. Findings of this meta-analysis showed that a diet characterized by high intake of whole grains, vegetables, fruit and dairy products and low amounts of red meat and processed meat was associated with lower risk of CRC.

[Schwingshackl, Lukas; Schwedhelm, Carolina; Knueppel, Sven; Preterre, Anne Laure; Iqbal, Khalid; Boeing, Heiner] German Inst Human Nutr Potsdam Rehbruecke DIfE, Dept Epidemiol, Arthur Scheunert Allee 114-116, D-14558 Nuthetal, Germany; [Hoffmann, Georg] Univ Vienna, Dept Nutr Sci, Althanstr 14,UZA 2, A-1090 Vienna, Austria; [Bechthold, Angela] German Nutr Soc, Godesberger Allee 18, D-53175 Bonn, Germany; [De Henauw, Stefaan; Michels, Nathalie] Univ Ghent, Dept Publ Hlth, B-9000 Ghent, Belgium; [Devleesschauwer, Brecht] Sci Inst Publ Hlth WIV ISP, Dept Publ Hlth & Surveillance, Rue Juliette Wytsmanstr 14, B-1050 Brussels, Belgium; [Schlesinger, Sabrina] Heinrich Heine Univ Dusseldorf, Inst Biometry & Epidemiol, Leibniz Inst Diabet Res, German Diabet Ctr, D-40225 Dusseldorf, Germany Leibniz Association; Deutsches Institut fur Ernahrungsforschung Potsdam-Rehbrucke (DIfE); University of Vienna; Ghent University; Leibniz Association; Deutsches Diabetes-Zentrum (DDZ); Heinrich Heine University Dusseldorf Schwingshackl, L (corresponding author), Arthur Scheunert Allee 114-116, D-14558 Nuthetal, Germany.

lukas.schwingshackl@dife.de Iqbal, Khalid/AAD-7112-2022; Michels, Nathalie/C-2819-2012; Schlesinger, Sabrina/AAE-7640-2020; Devleesschauwer, Brecht/G-9895-2018; Hoffmann, Georg/B-9201-2013; Schwedhelm, Carolina/AAE-6756-2022; Schwingshackl, Lukas/AAC-4119-2019; Knuppel, Sven/AAI-7195-2020; Schwingshackl, Lukas/B-9220-2013 Knuppel, Sven/0000-0001-9006-9906; Schwingshackl, Lukas/0000-0003-3407-7594; Michels, Nathalie/0000-0002-3069-7254

46 232 248 1 134 WILEY

HOBOKEN 111 RIVER ST, HOBOKEN 07030-5774, NJ USA
0020-7136 1097-0215 INT J CANCER Int. J. Cancer
MAY 1 2018 142 9 1748 1758
10.1002/ijc.31198 http://dx.doi.org/10.1002/ijc.31198
11 Oncology Science Citation Index Expanded (SCI-EXPANDED) Oncology FY3OQ 29210053 Bronze Y N
2025-06-24 WOS:000426730600003
J Khodaei, SH; Sabetkam, S; Mazloumi, Z; Asl, KD; Rafat, A
Khodaei, Sepideh Hassanpour; Sabetkam, Shahnaz; Mazloumi, Zeinab; Asl, Khadijeh Dizaji; Rafat, Ali
Targeting cancer-cell mitochondria using Tigecycline improves radiotherapy response in colorectal cancer cell line
MUTATION RESEARCH-FUNDAMENTAL AND MOLECULAR MECHANISMS OF MUTAGENESIS English Article
Colorectal cancer; Tigecycline; Radiotherapy; Apoptosis RADIATION-THERAPY; INHIBITION; CARCINOMA; RESISTANT
Background: Colorectal cancer (CRC) is the third most common cancer worldwide and causes more than 50,000 deaths in the United States each year. Due to the limited therapeutic options and poor prognosis in CRC, extensive research and development of novel therapeutic methods is essential. In this regard, the presence of cancer stem cells with unlimited division ability is the main reason for the therapeutic resistance in CRC. Tigecycline is a pharmacological mitochondria inhibitor and blocks mitochondria-related cell proliferation in cancer cells. This study investigated the effects of Tigecycline combined with radiotherapy on CRC cell apoptosis. Methods: Human colorectal cancer cells (HCT-116) were treated with Tigecycline, and cell viability was measured with MTT assay. In the next step, the cells were exposed to radiation using a Siemens Primus 6 MV linear accelerator at radiation dose of 400 cGy. Finally, we evaluated cancer cell apoptosis, caspase-3 activity and apoptotic-related genes expression with AnnexinV/PI, flowcytometry and gene expression, respectively. Results: The MTT assay revealed an IC50 value of 93 mu M for Tigecycline after 48 hours. Mitochondria inhibition, at its IC50 value, sensitizes colorectal cancer cells to radiotherapy. Compared to monotherapy, the combination therapy increased the number of apoptotic cells and caspase-3 activity, up-regulated pro-apoptotic genes, and down-regulated anti-apoptotic genes. Conclusion: In conclusion, our data suggests that targeting mitochondria may represent a clinically relevant approach to enhance the sensitivity of colorectal cancer cells to therapy. These findings could provide new insights into cancer therapy and might be used as a novel method to improve the current state of CRC therapy. [Khodaei, Sepideh Hassanpour] Eastern Mediterranean Univ EMU, Dept Dent, Mersin 10, Famagusta, North Cyprus, Turkiye; [Sabetkam, Shahnaz] Univ Kyrenia, Fac Med, Dept Anat, Kyrenia, Northern Cyprus, Turkiye; [Mazloumi, Zeinab] Tabriz Univ Med Sci, Fac Adv Med Sci, Dept Med Appl Cell Sci, Tabriz, Iran; [Asl, Khadijeh Dizaji] Islamic Azad Univ, Dept Histopathol & Anat, TMSc, Tabriz, Iran; [Rafat, Ali] Kashan Univ Med Sci, Anat Sci Res Ctr, Inst Basic Sci, Kashan, Iran Eastern Mediterranean University; Tabriz University of Medical Science; Islamic Azad University Rafat, A (corresponding author), Kashan Univ Med Sci, Anat Sci Res Ctr, Inst Basic Sci, Kashan, Iran.
Rafata@tbzmed.ac.ir dizaji asl, khadijeh/KBQ-0968-2024
Rafat, Ali/0000-0002-5594-2906 Kashan University of Medical Sciences, Kashan, Iran [403070] Kashan University of

Medical Sciences, Kashan, Iran(Golestan University of Medical Sciences) The present work was financially supported by Grant No. 403070 from Kashan University of Medical Sciences, Kashan, Iran. 25 0 0 0 0 ELSEVIER AMSTERDAM RADARWEG 29, 1043 NX AMSTERDAM, NETHERLANDS 0027-5107 1873-135X MUTAT RES-FUND MOL M Mutat. Res.-Fundam. Mol. Mech. Mutagen. JAN-JUN 2025 830 111905 10.1016/j.mrfmmm.2025.111905 http://dx.doi.org/10.1016/j.mrfmmm.2025.111905 APR 2025 6 Biotechnology & Applied Microbiology; Genetics & Heredity; Toxicology Science Citation Index Expanded (SCI-EXPANDED) Biotechnology & Applied Microbiology; Genetics & Heredity; Toxicology 1QY0G 40233495 2025-06-24 WOS:001471543400001

J Toledo, E; Salas-Salvadó, J; Donat-Vargas, C; Buil-Cosiales, P; Estruch, R; Ros, E; Corella, D; Fitó, M; Hu, FB; Arós, F; Gómez-Gracia, E; Romaguera, D; Ortega-Calvo, M; Serra-Majem, L; Pintó, X; Schröder, H; Basora, J; Sorlí, JV; Bulló, M; Serra-Mir, M; Martínez-González, MA Toledo, Estefania; Salas-Salvado, Jordi; Donat-Vargas, Carolina; Buil-Cosiales, Pilar; Estruch, Ramon; Ros, Emilio; Corella, Dolores; Fito, Montserrat; Hu, Frank B.; Aros, Fernando; Gomez-Gracia, Enrique; Romaguera, Dora; Ortega-Calvo, Manuel; Serra-Majem, Lluis; Pinto, Xavier; Schroeder, Helmut; Basora, Josep; Vicente Sorli, Jose; Bullo, Monica; Serra-Mir, Merce; Martinez-Gonzalez, Miguel A. Mediterranean Diet and Invasive Breast Cancer Risk Among Women at High Cardiovascular Risk in the PREDIMED Trial A Randomized Clinical Trial JAMA INTERNAL MEDICINE English Article PHYSICAL-ACTIVITY QUESTIONNAIRE; OXIDATIVE DNA-DAMAGE; OLIVE OIL INTAKE; FAT; ADHERENCE; PATTERN; HEALTH; METAANALYSIS; VALIDATION; MORTALITY IMPORTANCE Breast cancer is the leading cause of female cancer burden, and its incidence has increased by more than 20% worldwide since 2008. Some observational studies have suggested that the Mediterranean dietmay reduce the risk of breast cancer. OBJECTIVE To evaluate the effect of 2 interventions with Mediterranean diet vs the advice to follow a low-fat diet (control) on breast cancer incidence. DESIGN, SETTING, AND PARTICIPANTS The PREDIMED study is a 1:1:1 randomized, single-blind, controlled field trial conducted at primary health care centers in Spain. From 2003 to 2009, 4282 women aged 60 to 80 years and at high cardiovascular disease risk were recruited after invitation by their primary care physicians. INTERVENTIONS Participants were randomly allocated to a Mediterranean diet supplemented with extra-virgin olive oil, a Mediterranean diet supplemented with mixed nuts, or a control diet (advice to reduce dietary fat). MAIN OUTCOMES AND MEASURES Breast cancer incidence was a prespecified secondary outcome of the trial for women without a prior history of breast cancer (n = 4152). RESULTS After a median follow-up of 4.8 years, we identified 35 confirmed incident cases of breast cancer. Observed rates (per 1000 person-years) were 1.1 for the Mediterranean diet with extra-virgin olive oil group, 1.8 for the Mediterranean diet with nuts group, and 2.9 for the control group. The multivariable-adjusted hazard ratios vs the control group were 0.32 (95% CI, 0.13-0.79) for the Mediterranean diet with extra-virgin olive oil group and 0.59 (95% CI, 0.26-1.35) for the Mediterranean diet with nuts group. In analyses with yearly cumulative updated dietary exposures, the

hazard ratio for each additional 5% of calories from extra-virgin olive oil was 0.72 (95% CI, 0.57-0.90). CONCLUSIONS AND RELEVANCE This is the first randomized trial finding an effect of a long-term dietary intervention on breast cancer incidence. Our results suggest a beneficial effect of a Mediterranean diet supplemented with extra-virgin olive oil in the primary prevention of breast cancer. These results come from a secondary analysis of a previous trial and are based on few incident cases and, therefore, need to be confirmed in longer-term and larger studies. [Toledo, Estefania; Donat-Vargas, Carolina] Univ Navarra, Sch Med, Dept Prevent Med & Publ Hlth, Navarra 31008, Spain; [Toledo, Estefania; Salas-Salvado, Jordi; Donat-Vargas, Carolina; Estruch, Ramon; Ros, Emilio; Corella, Dolores; Fito, Montserrat; Aros, Fernando; Gomez-Gracia, Enrique; Romaguera, Dora; Ortega-Calvo, Manuel; Serra-Majem, Lluis; Pinto, Xavier; Basora, Josep; Vicente Sorli, Jose; Bullo, Monica; Serra-Mir, Merce; Martinez-Gonzalez, Miguel A.] Inst Salud Carlos III, Ctr Invest Biomed Red Fisiopatol Obesidad & Nutr, Madrid, Spain; [Salas-Salvado, Jordi; Basora, Josep; Bullo, Monica] Univ Rovira & Virgili, Fac Med & Hlth Sci, Biochem Biotechnol Dept, Human Nutr Unit, E-43201 Reus, Spain; [Buil-Cosiales, Pilar] Serv Navarro Salud Osasunbidea, Navarra, Spain; [Buil-Cosiales, Pilar; Martinez-Gonzalez, Miguel A.] IdiSNA, Navarra Inst Hlth Res, Navarra, Spain; [Estruch, Ramon] Univ Barcelona, Hosp Clin, Inst Invest Biomed August Pi I Sunyer, Dept Internal Med, Barcelona, Spain; [Ros, Emilio; Serra-Mir, Merce] Univ Barcelona, Hosp Clin, Inst Invest Biomed August Pi I Sunyer, Lipid Clin,Dept Endocrinol & Nutr, Barcelona, Spain; [Corella, Dolores; Vicente Sorli, Jose] Univ Valencia, Dept Prevent Med, Valencia, Spain; [Fito, Montserrat] Hosp del Mar, Inst Recerca, Cardiovasc & Nutr Res Grp, Barcelona, Spain; [Hu, Frank B.] Harvard Univ, Sch Publ Hlth, Dept Nutr, Boston, MA 02115 USA; [Hu, Frank B.] Harvard Univ, Sch Publ Hlth, Dept Epidemiol, Boston, MA 02115 USA; [Hu, Frank B.] Harvard Univ, Brigham & Womens Hosp, Sch Med, Dept Med, Boston, MA 02115 USA; [Aros, Fernando] Univ Hosp Alava, Vitoria, Basque Country, Spain; [Gomez-Gracia, Enrique] Univ Malaga, Dept Prevent Med, E-29071 Malaga, Spain; [Romaguera, Dora] Hosp Univ Son Espases, Inst Invest Sanitaria Palma IdISPa, Palma De Mallorca, Spain; [Ortega-Calvo, Manuel] Primary Care Div Sevilla, Dept Family Med, Seville, Spain; [Serra-Majem, Lluis] Univ Las Palmas Gran Canaria, Res Inst Biomed & Hlth Sci, Las Palmas Gran Canaria, Spain; [Pinto, Xavier] Hosp Univ Bellvitge IDIBELL UB, Lipids & Vasc Risk Unit, Internal Med, Barcelona, Spain; [Schroeder, Helmut] Inst Catala Salut & IDiap Jordi Gol, Primary Care Div Barcelona, Barcelona, Spain; [Schroeder, Helmut] Inst Salud Carlos III, Ctr Invest Biomed Red Epidemiol & Salud Publ, Madrid, Spain University of Navarra; CIBER - Centro de Investigacion Biomedica en Red; CIBEROBN; Instituto de Salud Carlos III; Universitat Rovira i Virgili; Servicio Navarro de Salud - Osasunbidea; University of Barcelona; Hospital Clinic de Barcelona; IDIBAPS; University of Barcelona; Hospital Clinic de Barcelona; IDIBAPS; University of Valencia; Hospital del Mar Research Institute; Hospital del Mar; Autonomous University of Barcelona; Hospital Universitari Vall d'Hebron; Vall d'Hebron Institut de Recerca (VHIR); Harvard University; Harvard T.H. Chan School of Public Health; Harvard University; Harvard T.H. Chan School of Public Health; Harvard University; Harvard Medical School; Harvard University Medical Affiliates; Brigham & Women's Hospital; Universidad de Malaga; Hospital Universitari Son

Espases; Institut Investigacio Sanitaria Illes Balears (IdISBa); Universidad de Las Palmas de Gran Canaria; Institut d'Investigacio Biomedica de Bellvitge (IDIBELL); Bellvitge University Hospital; Instituto de Salud Carlos III; CIBER - Centro de Investigacion Biomedica en Red; CIBERESP Martínez-González, MA (corresponding author), Univ Navarra, Sch Med, Dept Prevent Med & Publ Hlth, Edificio Invest,2a Planta,C Irunlarrea 1, Navarra 31008, Spain.
mamartinez@unav.es   Pintó, Xavier/AGI-4297-2022; Donat, Carolina/AAS-3985-2021; Hu, Frank/C-1919-2013; Serra-Majem, Lluis/I-6708-2019; Corella, Dolores/L-9888-2014; Martinez-Gonzalez, Miguel/AAE-7669-2019; Estruch, Ramon/AAZ-3723-2020; Romaguera, Dora/AAB-2852-2020; Ortega-Calvo, Manuel/D-6960-2015; Sorlí, José/L-8758-2014; Toledo, Estefania/H-6211-2014; Bullo, Monica/F-2925-2016; Schroder, Helmut/G-2586-2015; Fito Colomer, Montse/C-1822-2012; Salas-Salvado, Jordi/C-7229-2017   Serra-Majem, Lluis/0000-0002-9658-9061; Romaguera, Dora/0000-0002-5762-8558; Basora, Josep/0000-0003-0278-1149; Toledo, Estefania/0000-0002-6263-4434; Sorli, Jose V/0000-0002-0130-2006; Donat, Carolina/0000-0002-4523-4148; Bullo, Monica/0000-0002-0218-7046; Buil-Cosiales, Pilar/0000-0002-8586-577X; Pinto Sala, Xavier/0000-0002-2216-2444; Ros, Emilio/0000-0002-2573-1294; Gomez Gracia, Enrique/0000-0002-1281-5798; Schroder, Helmut/0000-0003-2231-5081; Fito Colomer, Montse/0000-0002-1817-483X; Salas-Salvado, Jordi/0000-0003-2700-7459   Spanish government (Instituto de Salud Carlos III) [RTIC G03/140, RTIC RD 06/0045]; National Institutes of Health [1R01HL118264-01, 1R01DK102896]; Fondo de Investigacion Sanitaria-Fondo Europeo de Desarrollo Regional [PI04/0233, PI05/0976, PI07/0240, PI10/01407, PI10/02658, PI11/00049, PI11/02505, AGL2010-22319-C03-03]; Consejeria de Salud de la Junta de Andalucia [PI0105/2007]; Generalitat Valenciana, Spain [ACOMP/2013/165, ACOMP/2013/159] Spanish government (Instituto de Salud Carlos III)(Instituto de Salud Carlos III); National Institutes of Health(United States Department of Health & Human ServicesNational Institutes of Health (NIH) - USA); Fondo de Investigacion Sanitaria-Fondo Europeo de Desarrollo Regional; Consejeria de Salud de la Junta de Andalucia(Junta de Andalucia); Generalitat Valenciana, Spain(Center for Forestry Research & Experimentation (CIEF))   The supplemental foods used in the study were generously donated by Patrimonio Comunal Olivarero and Hojiblanca, Spain (EVOO); the California Walnut Commission, Sacramento, California (walnuts); and Borges SA (almonds) and La Morella Nuts (hazelnuts), both from Reus, Spain. The PREDIMED trial was supported by the official funding agency for biomedical research of the Spanish government (Instituto de Salud Carlos III) through grants provided to research networks specifically developed for the trial: RTIC G03/140 (Coordinator: R.E.) and RTIC RD 06/0045 (Coordinator: M.A.M.-G.). All investigators of the PREDIMED trial belong to Centro de Investigacion Biomedica en Red (CIBER), an initiative of Instituto de Salud Carlos III. We also acknowledge grants from the National Institutes of Health (1R01HL118264-01 and 1R01DK102896); Fondo de Investigacion Sanitaria-Fondo Europeo de Desarrollo Regional (PI04/0233, PI05/0976, PI07/0240, PI10/01407, PI10/02658, PI11/00049, PI11/02505 and AGL2010-22319-C03-03); Consejeria de Salud de la Junta de Andalucia (PI0105/2007); and the Generalitat Valenciana, Spain (ACOMP/2013/165 and ACOMP/2013/159).   40   358   374
0   73   AMER MEDICAL ASSOC   CHICAGO   330 N WABASH AVE, STE 39300, CHICAGO, IL 60611-5885 USA   2168-6106   2168-6114

JAMA INTERN MED JAMA Intern. Med. NOV 2015 175 11 1752 1760
10.1001/jamainternmed.2015.4838
http://dx.doi.org/10.1001/jamainternmed.2015.4838
9 Medicine, General & Internal Science Citation Index Expanded (SCI-EXPANDED) General & Internal Medicine CV7BO
26365989 Bronze, Green Submitted, Green Published, Green Accepted Y N 2025-06-24 WOS:000364427200006
J Fuhr, L; Basti, A; Brás, TS; Duarte, MF; Relógio, A
Fuhr, Luise; Basti, Alireza; Bras, Teresa Silva; Duarte, Maria F.; Relogio, Angela Antiproliferative Effects of Cynara Carduncululus in Colorectal Cancer Cells Are Modulated by the Circadian Clock INTERNATIONAL JOURNAL OF MOLECULAR SCIENCES English Article
circadian clock; colorectal cancer; plant-derived compounds; proliferation; treatment sensitivity; apoptosis; cytotoxicity GENE; CYNAROPICRIN; SENSITIVITY; EXPRESSION; HALLMARKS; PLANTS; MACC1 The circadian clock generates 24 h rhythms in behavioural, cellular and molecular processes. Malfunctions of the clock are associated with enhanced susceptibility to cancer, worse treatment response and poor prognosis. Clock-controlled genes are involved in cellular processes associated with tumour development and progression including metabolism of drugs and the cell cycle. Cynara cardunculus, a plant of the Asteraceae family, has been reported to have antiproliferative effects on breast cancer cells. Here, we used the human colorectal cancer (CRC) cell line HCT116 and its knockout variants for different core-clock genes (BMAL1, PER2, NR1D1), to investigate the treatment effect of C. cardunculus lipophilic leaf extract under different clock scenarios. Our results show a direct effect of C. cardunculus on the circadian phenotype of the cells, as indicated by alterations in the phase, amplitude, and period length of core-clock gene oscillations. Furthermore, our data indicate a role for the circadian clock in sensitivity to C. cardunculus treatment. In particular, the treatment inhibited proliferation and induced cytotoxicity and apoptosis in a clock knockout-specific manner, in CRC cells. These results point to a potential effect of C. cardunculus lipophilic leaf extracts as a modulator of the circadian clock, in addition to its anti-proliferative properties. [Fuhr, Luise; Basti, Alireza; Relogio, Angela] Charite Univ Med Berlin, D-10115 Berlin, Germany; [Fuhr, Luise; Basti, Alireza; Relogio, Angela] Free Univ Berlin, D-10115 Berlin, Germany; [Fuhr, Luise; Basti, Alireza; Relogio, Angela] Humboldt Univ, Inst Theoret Biol, D-10115 Berlin, Germany; [Fuhr, Luise; Basti, Alireza; Relogio, Angela] Humboldt Univ, Med Dept Hematol Oncol & Tumor Immunol, Mol Canc Res Ctr, D-10115 Berlin, Germany; [Basti, Alireza; Relogio, Angela] Appl Sci & Med Univ, MSH Med Sch Hamburg Univ, Inst Syst Med, Fac Human Med, D-20457 Hamburg, Germany; [Bras, Teresa Silva; Duarte, Maria F.] Alentejo Biotechnol Ctr Agr & Agrofood CEBAL, P-7801908 Beja, Portugal; [Bras, Teresa Silva; Duarte, Maria F.] Inst Politecn Beja IPBeja, P-7801908 Beja, Portugal; [Bras, Teresa Silva; Duarte, Maria F.] MED Mediterranean Inst Agr Environm & Dev, P-7801908 Beja, Portugal; [Bras, Teresa Silva; Duarte, Maria F.] CEBAL, CHANGE Global Change & Sustainabil Inst, P-7801908 Beja, Portugal Berlin Institute of Health; Free University of Berlin; Humboldt University of Berlin; Charite Universitatsmedizin Berlin; Free University of Berlin; Humboldt University of Berlin; Humboldt

University of Berlin; MSH Medical School Hamburg; Instituto Politecnico de Beja Relógio, A (corresponding author), Charite Univ Med Berlin, D-10115 Berlin, Germany.; Relógio, A (corresponding author), Free Univ Berlin, D-10115 Berlin, Germany.; Relógio, A (corresponding author), Humboldt Univ, Inst Theoret Biol, D-10115 Berlin, Germany.; Relógio, A (corresponding author), Humboldt Univ, Med Dept Hematol Oncol & Tumor Immunol, Mol Canc Res Ctr, D-10115 Berlin, Germany.; Relógio, A (corresponding author), Appl Sci & Med Univ, MSH Med Sch Hamburg Univ, Inst Syst Med, Fac Human Med, D-20457 Hamburg, Germany.
Bras, Teresa/AAY-6262-2021; Fuhr, Luise/F-2398-2017; Relogio, Angela/M-4959-2016; Duarte, Maria F./H-5177-2013
Relogio, Angela/0000-0002-9165-2439; Duarte, Maria F./0000-0002-2223-7784; Bras, Teresa/0000-0002-8526-6320; Akhondzadeh Basti, Alireza/0000-0002-0153-2784 Rolf M. Schwiete Stiftung; Berlin School of Integrative Oncology (BSIO) of the ChariteUniversitatsmedizin Berlin; Program Alentejo 2020, through the European Fund for Regional Development (FEDER) [LT20-03-0145-FEDER039495]; FCT [UIDB/05183/2020]; CHANGEGlobal Change and Sustainability Institute [LA/P/0121/2020] Rolf M. Schwiete Stiftung; Berlin School of Integrative Oncology (BSIO) of the ChariteUniversitatsmedizin Berlin; Program Alentejo 2020, through the European Fund for Regional Development (FEDER)(European Union (EU)); FCT(Fundacao para a Ciencia e a Tecnologia (FCT)); CHANGEGlobal Change and Sustainability Institute The work in the Relogio group was funded by the Rolf M. Schwiete Stiftung. AB was additionally funded by the Berlin School of Integrative Oncology (BSIO) of the ChariteUniversitatsmedizin Berlin. The work in the group of MFD is supported by Program Alentejo 2020, through the European Fund for Regional Development (FEDER) under the scope of MedCynaraBioTec-Selection of Cynara cardunculus genotypes for new biotechnological applications: the value chain improvement of cardoon, a well-adapted Mediterranean crop (ALT20-03-0145-FEDER039495). The authors also acknowledge FCT for Project UIDB/05183/2020 to Mediterranean Institute for Agriculture, Environment and Development (MED), and Project LA/P/0121/2020 to CHANGEGlobal Change and Sustainability Institute. 46 8 8 0 10 MDPI BASEL ST ALBAN-ANLAGE 66, CH-4052 BASEL, SWITZERLAND 1661-6596 1422-0067 INT J MOL SCI
Int. J. Mol. Sci. AUG 2022 23 16
9130 10.3390/ijms23169130
http://dx.doi.org/10.3390/ijms23169130 15
Biochemistry & Molecular Biology; Chemistry, Multidisciplinary Science Citation Index Expanded (SCI-EXPANDED) Biochemistry & Molecular Biology; Chemistry 4B3BF
36012399 Green Published, gold 2025-06-24
WOS:000845656800001
J Marzouk, D; Abd El Aal, W; Saleh, A; Sleem, H; Khyatti, M; Mazini, L; Hemminki, K; Anwar, WA Marzouk, Diaa; Abd El Aal, Wafaa; Saleh, Azza; Sleem, Hany; Khyatti, Meriem; Mazini, Loubna; Hemminki, Kari; Anwar, Wagida A.
Overview on health research ethics in Egypt and North Africa
EUROPEAN JOURNAL OF PUBLIC HEALTH English
Article EASTERN MEDITERRANEAN REGION; COMMITTEES Developing countries, including Egypt and North African countries, need to improve their quality of research by enhancing international cooperation and exchanges of scientific information, as well as competing for obtaining

international funds to support research activities. Research must comply with laws and other requirements for research that involves human subjects. The purpose of this article is to overview the status of health research ethics in Egypt and North African countries, with reference to other Middle Eastern countries. The EU and North African Migrants: Health and Health Systems project (EUNAM) has supported the revision of the status of health research ethics in Egypt and North African countries, by holding meetings and discussions to collect information about research ethics committees in Egypt, and revising the structure and guidelines of the committees, as well as reviewing the literature concerning ethics activities in the concerned countries. This overview has revealed that noticeable efforts have been made to regulate research ethics in certain countries in the Middle East. This can be seen in the new regulations, which contain the majority of protections mentioned in the international guidelines related to research ethics. For most of the internationally registered research ethics committees in North African countries, the composition and functionality reflect the international guidelines. There is growing awareness of research ethics in these countries, which extends to teaching efforts to undergraduate and postgraduate medical students. [Marzouk, Diaa; Anwar, Wagida A.] Ain Shams Univ, Fac Med, Dept Community Med, Cairo, Egypt; [Abd El Aal, Wafaa] Natl Res Ctr, Cairo, Egypt; [Saleh, Azza] Theodor Bilharz Res Inst, Giza, Egypt; [Sleem, Hany] Natl Hepatol & Trop Med Res Inst, Cairo, Egypt; [Khyatti, Meriem; Mazini, Loubna] Inst Pasteur Maroc, Lab Oncovirol, Casablanca, Morocco; [Hemminki, Kari] German Canc Res Ctr, Div Mol Genet Epidemiol, D-69120 Heidelberg, Germany; [Hemminki, Kari] Lund Univ, Ctr Primary Hlth Care Res, Malmo, SwedenEgyptian Knowledge Bank (EKB); Ain Shams University; Egyptian Knowledge Bank (EKB); National Research Centre (NRC); Egyptian Knowledge Bank (EKB); Theodor Bilharz Research Institute (TBRI); Egyptian Knowledge Bank (EKB); National Hepatology & Tropical Medicine Research Institute (NHTMRI); Helmholtz Association; German Cancer Research Center (DKFZ); Lund University Marzouk, D (corresponding author), Ain Shams Univ, Fac Med, Dept Commun Environm & Occupat Med, Ramses St, Cairo, Egypt. diaamarzouk@yahoo.com Abd El-Aal, Wafaa/0000-0002-3066-9982 European Union (EU) [260715]European Union (EU)(European Union (EU)CGIAR) The authors wish to thank EUNAM project members, supported by the European Union (EU FP7/2007-2013 grant 260715), for their guidance and scientific and financial support of the execution of this work. 20 17 17 0 5 OXFORD UNIV PRESS OXFORD GREAT CLARENDON ST, OXFORD OX2 6DP, ENGLAND 1101-1262 1464-360X EUR J PUBLIC HEALTH Eur. J. Public Health AUG 2014 24 1 87 91 10.1093/eurpub/cku110 http://dx.doi.org/10.1093/eurpub/cku110 5 Public, Environmental & Occupational Health Science Citation Index Expanded (SCI-EXPANDED); Social Science Citation Index (SSCI) Public, Environmental & Occupational Health CT7BX 25108003 Green Published, Bronze 2025-06-24 WOS:000362969300014

J Farazi, PA; Hadji, P; Roupa, Z Farazi, Paraskevi A.; Hadji, Panayiota; Roupa, Zoe Awareness of human papilloma virus and cervical cancer prevention among Greek female healthcare workers EUROPEAN JOURNAL OF CANCER PREVENTION English Article

awareness; cervical cancer; healthcare workers; human papilloma virus; prevention HPV INFECTION; RISK-FACTORS; SUSTAINED EFFICACY; PARTICLE VACCINE; CONTROLLED-TRIAL; SQUAMOUS-CELL; YOUNG-WOMEN; METAANALYSIS; DISEASES; KNOWLEDGE

The incidence rate of cervical cancer varies by geographic region, with less developed regions showing the highest rates. All risk factors for cervical cancer are actually preventable if appropriate lifestyle changes are adopted. In addition, vaccines protecting against the majority of human papilloma virus (HPV) high-risk types have been developed. Even though cervical cancer is preventable, not all women are aware of this or how it can be prevented. Thus, it is essential for every nation to assess the level of knowledge among women of cervical cancer and HPV prevention. In this work, we assessed the level of awareness and attitudes of Greek female healthcare workers on cervical cancer and HPV prevention through the delivery of a validated questionnaire between March and June 2012 in three hospitals in Greece. Our results show that there exist gaps in the knowledge of women on this topic, especially in terms of the newest information on cervical cancer prevention through HPV testing and vaccination. In fact, only 80% of surveyed women knew about the existence of HPV testing. We propose that more information needs to be transmitted to Greek women and men on HPV testing and vaccination. Even though the incidence of cervical cancer is not extremely high in Greece, this number can easily change, especially in the face of the economic crisis and the increasing rates of migration, which can result in higher rates of HPV infection in the population if no measures for HPV prevention are implemented. European Journal of Cancer Prevention 26: 330-335
Copyright (C) 2017 Wolters Kluwer Health, Inc. All rights reserved.
[Farazi, Paraskevi A.; Roupa, Zoe] Univ Nicosia, Dept Life & Hlth Sci, Nicosia, Cyprus; [Farazi, Paraskevi A.] Mediterranean Ctr Canc Res, Nicosia, Cyprus; [Farazi, Paraskevi A.] Univ Nebraska Med Ctr, Dept Epidemiol, 984395 Nebraska Med Ctr, Omaha, NE 68198 USA; [Hadji, Panayiota] Univ Thessaly, Dept Hlth Sci & Med, Larisa, Greece University of Nicosia; University of Nebraska System; University of Nebraska Medical Center; University of Thessaly Farazi, PA (corresponding author), Univ Nebraska Med Ctr, Dept Epidemiol, 984395 Nebraska Med Ctr, Omaha, NE 68198 USA. evi.farazi@unmc.edu
Farazi, Paraskevi/0000-0002-4375-5812
41 5 5 0 3 LIPPINCOTT WILLIAMS & WILKINS
PHILADELPHIA TWO COMMERCE SQ, 2001 MARKET ST, PHILADELPHIA, PA 19103 USA 0959-8278 1473-5709 EUR J CANCER PREV Eur. J. Cancer Prev. JUL 2017 26 4
330 335 10.1097/CEJ.0000000000000254
http://dx.doi.org/10.1097/CEJ.0000000000000254
6 Oncology Science Citation Index Expanded (SCI-EXPANDED) Oncology EX9JI 27254169 2025-06-24 WOS:000403573800009

J Kassem, NM; Emera, G; Kassem, HA; Medhat, N; Nagdy, B; Tareq, M; Moneim, RA; Abdulla, M; El Metenawy, WH
Kassem, Neemat M.; Emera, Gamal; Kassem, Hebatallah A.; Medhat, Nashwa; Nagdy, Basant; Tareq, Mustafa; Moneim, Rabab Abdel; Abdulla, Mohammed; El Metenawy, Wafaa H.
Clinicopathological features of Egyptian colorectal cancer patients regarding somatic genetic mutations especially in KRAS gene and microsatellite instability status: a pilot study
EGYPTIAN JOURNAL OF MEDICAL HUMAN GENETICS

English Article

Colorectal cancer; Targeted NGS; KRAS mutation; MSI

Background Colorectal cancer (CRC) is the third most common cause of cancer-related deaths which contributes to a significant public health problem worldwide with 1.8 million new cases and almost 861,000 deaths in 2018 according to the World Health Organization. It exhibits 7.4% of all diagnosed cancer cases in the region of the Middle East and North Africa. Molecular changes that happen in CRCs are chromosomal instability, microsatellite instability (MSI), and CpG island methylator phenotype. The human RAS family (KRAS, NRAS, and HRAS) is the most frequently mutated oncogenes in human cancer appearing in 45% of colon cancers. Determining MSI status across CRCs offers the opportunity to identify patients who are likely to respond to targeted therapies such as anti-PD-1. Therefore, a method to efficiently determine MSI status for every cancer patient is needed. Results KRAS mutations were detected in 31.6% of CRC patients, namely in older patients ($p = 0.003$). Codons 12 and 13 constituted 5/6 (83.3%) and 1/6 (16.7%) of all KRAS mutations, respectively. We found three mutations G12D, G12C, and G13D which occur as a result of substitution at c.35G>A, c.34G>T, and c.38G>A and have been detected in 4/6 (66.6%), 1/6 (16.7%), and 1/6 (16.7%) patients, respectively. Eleven (57.9%) patients had microsatellite instability-high (MSI-H) CRC. A higher percentage of MSI-H CRC was detected in female patients ($p = 0.048$). Eight patients had both MSI-H CRC and wild KRAS mutation with no statistical significance was found between MSI status and KRAS mutation in these studied patients. Conclusion In conclusion, considering that KRAS mutations confer resistance to EGFR inhibitors, patients who have CRC with KRAS mutation could receive more tailored management by defining MSI status. MSI-high patients have enhanced responsiveness to anti-PD-1 therapies. Thus, the question arises as to whether it is worth investigating this association in the routine clinical setting or not. Further studies with a larger number of patients are needed to assess the impact of MSI status on Egyptian CRC care.

[Kassem, Neemat M.; Kassem, Hebatallah A.; El Metenawy, Wafaa H.] Cairo Univ, Kasr Al Ainy Ctr Clin Oncol & Nucl Med, Sch Med, Clin & Chem Pathol Dept, Cairo, Egypt; [Emera, Gamal] Cairo Univ, Natl Canc Inst, Surg Dept, Cairo, Egypt; [Medhat, Nashwa; Nagdy, Basant; Tareq, Mustafa] Kasr Al Ainy Ctr Clin Oncol & Nucl Med, Mol Oncol Unit, Cairo, Egypt; [Moneim, Rabab Abdel; Abdulla, Mohammed] Cairo Univ, Sch Med, Clin Oncol Dept, Cairo, Egypt Egyptian Knowledge Bank (EKB); Cairo University; Egyptian Knowledge Bank (EKB); Cairo University; National Cancer Institute - Egypt; Egyptian Knowledge Bank (EKB); Cairo University Kassem, HA (corresponding author), Cairo Univ, Kasr Al Ainy Ctr Clin Oncol & Nucl Med, Sch Med, Clin & Chem Pathol Dept, Cairo, Egypt. Heba.kasem@hotmail.com Kassem, Hebatallah/X-3018-2019 Ahmed, Rabab/0000-0002-6729-5742; Kassem, Neemat/0000-0001-9295-2823; Tareq, Mustafa/0000-0002-6782-0973; Kassem, Hebatallah/0000-0002-6033-6839 Kasr Al Ainy Oncology Department, Cairo UniversityKasr Al Ainy Oncology Department, Cairo University This study was funded by the Kasr Al Ainy Oncology Department, Cairo University, which provides us with the needed kits. This study was performed in the Molecular Lab of Kasr Al-Ainy Oncology Department with the availability of all equipments needed in our study especially MiSeqDX and bioanalyzer.

25 9 9 0 4 SPRINGERNATURE LONDON

CAMPUS, 4 CRINAN ST, LONDON, N1 9XW, ENGLAND 1110-8630
2090-2441 EGYPTIAN J MED HUMAN Egypt. J. Med. Human Genet. NOV 19 2019 20 1
20 10.1186/s43042-019-0028-z
http://dx.doi.org/10.1186/s43042-019-0028-z 9
Genetics & Heredity Emerging Sources Citation Index (ESCI)
Genetics & Heredity WE4IG gold 2025-06-24 WOS:000705590800001
J Bu, R; Siraj, AK; Al-Obaisi, KAS; Beg, S; Al Hazmi, M; Ajarim, D; Tulbah, A; Al-Dayel, F; Al-Kuraya, KS
Bu, Rong; Siraj, Abdul K.; Al-Obaisi, Khadija A. S.; Beg, Shaham; Al Hazmi, Mohsen; Ajarim, Dahish; Tulbah, Asma; Al-Dayel, Fouad; Al-Kuraya, Khawla S. Identification of novel BRCA founder mutations in Middle Eastern breast cancer patients using capture and Sanger sequencing analysis INTERNATIONAL JOURNAL OF CANCER English Article
breast cancer; mutation; BRCA 1; BRCA 2 POPULATION-BASED SERIES; GERMLINE MUTATIONS; HEREDITARY BREAST; OVARIAN-CANCER; RISK; PREVALENCE; FREQUENCY; RECURRENT; WOMEN Ethnic differences of breast cancer genomics have prompted us to investigate the spectra of BRCA1 and BRCA2 mutations in different populations. The prevalence and effect of BRCA 1 and BRCA 2 mutations in Middle Eastern population is not fully explored. To characterize the prevalence of BRCA mutations in Middle Eastern breast cancer patients, BRCA mutation screening was performed in 818 unselected breast cancer patients using Capture and/or Sanger sequencing. 19 short tandem repeat (STR) markers were used for founder mutation analysis. In our study, nine different types of deleterious mutation were identified in 28 (3.4%) cases, 25 (89.3%) cases in BRCA 1 and 3 (10.7%) cases in BRCA 2. Seven recurrent mutations identified accounted for 92.9% (26/28) of all the mutant cases. Haplotype analysis was performed to confirm c.1140 dupG and c.4136_4137delCT mutations as novel putative founder mutation, accounting for 46.4% (13/28) of all BRCA mutant cases and 1.6% (13/818) of all the breast cancer cases, respectively. Moreover, BRCA 1 mutation was significantly associated with BRCA 1 protein expression loss (p=0.0005). Our finding revealed that a substantial number of BRCA mutations were identified in clinically high risk breast cancer from Middle East region. Identification of the mutation spectrum, prevalence and founder effect in Middle Eastern population facilitates genetic counseling, risk assessment and development of cost-effective screening strategy. [Bu, Rong; Siraj, Abdul K.; Al-Obaisi, Khadija A. S.; Beg, Shaham; Al Hazmi, Mohsen; Al-Kuraya, Khawla S.] King Faisal Specialist Hosp & Res Ctr, Res Ctr, Human Canc Genom Res, MBC 98-16,POB 3354, Riyadh 11211, Saudi Arabia; [Ajarim, Dahish] King Faisal Specialist Hosp & Res Ctr, Dept Oncol, MBC 98-16,POB 3354, Riyadh 11211, Saudi Arabia; [Tulbah, Asma] King Faisal Specialist Hosp & Res Ctr, Dept Pathol & Lab Med, MBC 98-16,POB 3354, Riyadh 11211, Saudi Arabia; [Al-Dayel, Fouad] King Faisal Specialist Hosp & Res Ctr, Dept Pathol, MBC 98-16,POB 3354, Riyadh 11211, Saudi Arabia King Faisal Specialist Hospital & Research Center; King Faisal Specialist Hospital & Research Center; King Faisal Specialist Hospital & Research Center; King Faisal Specialist Hospital & Research Center Al-Kuraya, KS (corresponding author), King Faisal Specialist Hosp & Res Ctr, Res Ctr, Human Canc Genom Res, MBC 98-16,POB 3354, Riyadh 11211, Saudi Arabia. Kkuraya@kfshrc.edu.sa Bu, Rong/JBI-9735-

2023; Siraj, Abdul/IQW-1179-2023; Alkuraya, Khawla/AFQ-7946-2022
Beg, Shaham/0000-0002-6068-1595; Alkuraya, Khawla/0000-0002-4126-3419; Al Dayel, Fouad/0000-0001-6175-9051
40 56 57 1 26 WILEY HOBOKEN 111 RIVER ST, HOBOKEN 07030-5774, NJ USA 0020-7136 1097-0215 INT J CANCER Int. J. Cancer SEP 1 2016 139 5
1091 1097 10.1002/ijc.30143
http://dx.doi.org/10.1002/ijc.30143 7
Oncology Science Citation Index Expanded (SCI-EXPANDED)
Oncology DP3TR 27082205 hybrid, Green Published
2025-06-24 WOS:000378418800014
J Zaidi, M; Collins, H; Ahmed, R; Lee, DN; Valdman, O; Poghosyan, H; Cooley, ME; Lemon, SC Zaidi, Maryum; Collins, Heidi; Ahmed, Rasha; Lee, Donghee Nicole; Valdman, Olga; Poghosyan, Hermine; Cooley, Mary E.; Lemon, Stephenie C. Experiences With Cancer Screenings Among Arabic-Speaking Refugee Women NURSING FOR WOMENS HEALTH
English Article
Arabic speaking; barriers; cancer screening; facilitators; preventive care; refugee; women MIDDLE-EAST; BREAST-CANCER; NORTH-AFRICA; INFORMATION; PREVENTION; BEHAVIORS; FATALISM
Objective: To explore breast, colorectal, and cervical cancer screening experiences among Arabic-speaking refugee women in Massachusetts. Design: Qualitative descriptive. Setting: The research was conducted in central Massachusetts, an area with a large refugee population from the Middle East. Despite cancer being the leading cause of death among Middle Eastern women aged 40 to 79 years, refugees experience significant disparities in cancer screenings. These refugees often pass through intermediary countries before permanently resettling, leading to preventive care becominga lower priority as other life stressors take precedence. Participants: Eleven women participated. Participants had an average age of 47 years; had come from Iraq, Syria, and Egypt; and had been in the United States an average of 7 years. Methods: In 2022, semistructured individual interviews were conducted in person or via Zoom. An interview guide, constructed based on components of the Health Equity Implementation Science Framework and an expert panel of partners, refugee women, and medical providers, was used. Conventional content analysis was used to analyze the data. Results: Three themes emerged: Facilitators of Cancer Screening, Barriers to Cancer Screening, and Interpreter-Related Factors. Women positively described providers'efforts to provide knowledge regarding screenings; however, language barriers, the invasive nature of some screenings, and scheduling appointments for screening posed significant obstacles. Conclusion: This study highlights the unique health challenges faced by Arabic-speaking refugee women, focusing on their beliefs, experiences in clinical encounters, and communication regarding cancer screenings. Nurses can address these challenges by providing culturally sensitive care and building relationships with community resources. [Zaidi, Maryum; Collins, Heidi] Univ Massachusetts, Solomont Sch Nursing, Lowell, MA 01854 USA; [Ahmed, Rasha] Trinity Coll, Hartford, CT USA; [Lee, Donghee Nicole; Valdman, Olga; Lemon, Stephenie C.] UMass Chan Med Sch, Worcester, MA USA; [Valdman, Olga] Worcester RISE Hlth Refugee & Immigrant Support &, Worcester, MA, Brazil; [Poghosyan, Hermine] Yale Univ, Sch Nursing, Hartford, CT USA; [Cooley, Mary E.] Dana Farber Canc Inst, Boston, MA USA University of

Massachusetts System; University of Massachusetts Lowell; Trinity College; University of Massachusetts System; University of Massachusetts Worcester; UMass Chan Medical School; Yale University; Harvard University; Harvard University Medical Affiliates; Dana-Farber Cancer Institute Zaidi, M (corresponding author), Univ Massachusetts, Solomont Sch Nursing, Lowell, MA 01854 USA. maryum_zaidi@uml.edu Poghosyan, Hermine/JBI-9905-2023 Lee, Donghee/0000-0002-9932-4376; Zaidi, Maryum/0000-0002-6417-7257; Collins Fantasia, Heidi/0000-0002-7646-1183 National Cancer Institute Fellowship [T32 CA172009]; National Cancer Institute; Food and Drug Administration Center for Tobacco Products [1K99CA281094-01A1] National Cancer Institute Fellowship(United States Department of Health & Human ServicesNational Institutes of Health (NIH) - USANIH National Cancer Institute (NCI)); National Cancer Institute(United States Department of Health & Human ServicesNational Institutes of Health (NIH) - USANIH National Cancer Institute (NCI)); Food and Drug Administration Center for Tobacco Products During the coure of the study, Dr. Zaidi was supported by National Cancer Institute Fellowship T32 CA172009 (Principle Investigator: Lemon) at UMass Chan Medical School, Worcester, MA. The fellowship covered all costs related to this study. Dr. Lee is supported by the National Cancer Institute and the Food and Drug Administration Center for Tobacco Products under award 1K99CA281094-01A1 (Principle Investigator: Lee) . NWH 69 0 0 0 0
ELSEVIER AMSTERDAM RADARWEG 29, 1043 NX AMSTERDAM, NETHERLANDS 1751-4851 1751-486X NURS WOMENS HEALTH
Nurs. Womens Health APR 2025 29 2
109 119 10.1016/j.nwh.2024.09.004
http://dx.doi.org/10.1016/j.nwh.2024.09.004 APR 2025
11 Nursing; Obstetrics & Gynecology Emerging Sources Citation Index (ESCI) Nursing; Obstetrics & Gynecology 1GP1J
39947246 2025-06-24 WOS:001464534300001
J Ramirez, MU; Clear, KYJ; Cornelius, Z; Bawaneh, A; Feliz-Mosquea, YR; Wilson, AS; Ruggiero, AD; Cruz-Diaz, N; Shi, LH; Kerr, BA; Soto-Pantoja, DR; Cook, KL Ramirez, Manuel U.; Clear, Kenysha Y. J.; Cornelius, Zipporah; Bawaneh, Alaa; Feliz-Mosquea, Yismeilin R.; Wilson, Adam S.; Ruggiero, Alistaire D.; Cruz-Diaz, Nildris; Shi, Lihong; Kerr, Bethany A.; Soto-Pantoja, David R.; Cook, Katherine L. Diet impacts triple-negative breast cancer growth, metastatic potential, chemotherapy responsiveness, and doxorubicin-mediated cardiac dysfunction PHYSIOLOGICAL REPORTS English
Article cardiac damage; doxorubicin; drug resistance; fish oil; lung metastases; Mediterranean diet; triple-negative breast cancer; Western diet
RISK-FACTOR; OBESITY; CARDIOTOXICITY; SURVIVAL; WOMEN; ACID; RATS Anthracyclines are standard-of-care chemotherapy for the treatment of triple-negative breast cancer (TNBC). However, high anthracyclines cumulative doses increase heart failure risk. Designing therapeutic strategies that ameliorate cardiac toxicities without compromising oncologic efficacy are important to improve TNBC outcomes and survivorship. The purpose of this study was to determine the impact of diet on TNBC chemotherapeutic responsiveness and development of chemotherapy-induced cardiac damage. Female BALB/c mice fed a control, Western, Mediterranean, or Western + fish oil diet were injected with 1 x 10(6) 4T1-luciferase TNBC into the mammary fat pad. Tumors grew for 21 days

before surgical tumor resection, then mice were treated with 3.3 mg/kg i.v. doxorubicin for 3 weeks. Vevo (R) cardiac ultrasound was performed. Female nu/nu mice were placed on diets before 1 x 10(5) MDA-MB-231-luciferase TNBC were injected via the tail vein to induce the development of lung metastases. Mice were treated with saline or 3.3 mg/kg i.v. doxorubicin for 3 weeks, and the development of metastases visualized by IVIS (R). Consumption of a high-fat diet increased TNBC growth regardless of dietary pattern. Western diet-fed mice developed lung metastases sooner and displayed increased lung metastatic lesion formation, which was not observed in Mediterranean diet-fed mice. Western diet-fed animals displayed worse cardiac function when compared with Mediterranean diet-fed animals. Hearts from Western diet-fed animals displayed increased fibrosis. Diet represents a modifiable component directly impacting tumor growth, antitumor chemotherapy efficacy, and cardiac toxicities. Our data suggest that the Mediterranean diet may reduce lung metastatic lesions formation and prevent the development of cardiac toxicities.D [Ramirez, Manuel U.; Clear, Kenysha Y. J.; Bawaneh, Alaa; Feliz-Mosquea, Yismeilin R.] Wake Forest Univ Hlth Sci, Dept Physiol & Pharmacol, Winston Salem, NC USA; [Ramirez, Manuel U.; Clear, Kenysha Y. J.; Cornelius, Zipporah; Bawaneh, Alaa; Feliz-Mosquea, Yismeilin R.; Wilson, Adam S.; Cruz-Diaz, Nildris; Soto-Pantoja, David R.; Cook, Katherine L.] Wake Forest Sch Med, Dept Surg Hypertens, Winston Salem, NC 27101 USA; [Ruggiero, Alistaire D.] Wake Forest Sch Med, Dept Pathol, Winston Salem, NC 27101 USA; [Cruz-Diaz, Nildris; Soto-Pantoja, David R.; Cook, Katherine L.] Wake Forest Sch Med, Cardiovasc Sci, Winston Salem, NC 27101 USA; [Shi, Lihong; Kerr, Bethany A.; Soto-Pantoja, David R.; Cook, Katherine L.] Wake Forest Sch Med, Dept Canc Biol, Winston Salem, NC USA; [Kerr, Bethany A.; Soto-Pantoja, David R.; Cook, Katherine L.] Wake Forest Sch Med, Comprehens Canc Ctr, Winston Salem, NC USA Wake Forest University; Wake Forest University School of Medicine; Wake Forest University; Wake Forest University; Wake Forest University; Wake Forest University; Wake Forest University Cook, KL (corresponding author), Wake Forest Sch Med, 575 N Patterson Ave,Suite 340, Winston Salem, NC 27101 USA. klcook@wakehealth.edu
Kerr, Bethany/D-1644-2012; Pantoja, David/ABB-3764-2020
Ruggiero, Alistaire/0000-0002-1942-2751Metavivor Research and Support; Susan G. Komen Foundation [CCR18547795]; American Cancer Society [RSG-16-204-01-NEC, 133727-RSG-19-150-01-LIB]; IRACDA PRIME K12 [1K12-GM102773]; NIH [UL1TR001420]; National Institute of General Medical Sciences [T32GM127261] Funding Source: NIH RePORTER Metavivor Research and Support; Susan G. Komen Foundation(Susan G. Komen Breast Cancer Foundation); American Cancer Society(American Cancer Society); IRACDA PRIME K12; NIH(United States Department of Health & Human ServicesNational Institutes of Health (NIH) - USA); National Institute of General Medical Sciences(United States Department of Health & Human ServicesNational Institutes of Health (NIH) - USANIH National Institute of General Medical Sciences (NIGMS))
Metavivor Research and Support; Susan G. Komen Foundation, Grant/Award Number: CCR18547795 (to K.L.C.); American Cancer Society, Grant/Award Number: RSG-16-204-01-NEC and 133727-RSG-19-150-01-LIB; American Cancer Society, Grant/Award Number: RSG-16-204-01-NEC (to K.L.C.) and 133727-RSG-19-150-01-LIB (to D.S.P.); IRACDA PRIME K12, Grant/Award Number: 1K12-GM102773; NIH, Grant/Award Number: UL1TR001420 34 4 5 1 7

WILEY HOBOKEN 111 RIVER ST, HOBOKEN 07030-5774, NJ USA
2051-817X PHYSIOL REP PHYSIOL. REP. APR 2022
10 8 e15192
10.14814/phy2.15192 http://dx.doi.org/10.14814/phy2.15192
15 Physiology Emerging Sources Citation Index (ESCI) Physiology 0O6HC 35439354 Green Published, gold
2025-06-24 WOS:000783623900001
J Shandiz, FH; Afzaljavan, F; Tajbakhsh, A; Rivadeh, M; Sharifi, N; Shakeri, MT; Pasdar, A Shandiz, Fatemeh Homaei; Afzaljavan, Fahimeh; Tajbakhsh, Amir; Rivadeh, Maryam; Sharifi, Nourieh; Shakeri, Mohammad Taghi; Pasdar, Alireza
Age-related Variation in Expression of Breast Cancer Tumour Markers in Iranian Patients MIDDLE EAST JOURNAL OF CANCER English Article
HER2; Breast cancer; Biomarkers; Ki-67; TP53; Triple negative OVER-EXPRESSION; P53; WOMEN; CARCINOMA; HER-2/NEU; PROGNOSIS; ACCUMULATION; ASSOCIATION; SURVIVAL; FEATURES
Background: There are believed to be several risk factors affecting the prognosis of breast cancer through their effect on the growth rate of tumour. In the present study, we investigated estrogen receptor (ER), progesterone receptor (PR), human epidermal growth factor receptor 2 (HER2), Ki-67, and tumor protein P53 (TP53) as well-known biomarkers, particularly in breast cancer prognosis, associated with age. Method: In a case-control study, 406 breast cancer patients were considered retrospectively. In order to extract the clinical and pathologic data, we employed the patients' records. The extracted information was compared between two groups: for patients under 40 (group I) and above 40 years of age (group II). Herein, the researchers performed statistical analysis using SPSS Ver16. Results: The most prevalent type of cancer in both groups was found to be invasive ductal carcinoma. The major method of treatment was modified radical mastectomy. According to our observations, grade 3 breast cancer was more common in group I. Lymph node involvement significantly increased in group I, while oestrogen and progesterone receptor expressions were less in this group. HER2, TP53, and Ki-67 oncogenes were overexpressed in group I compared with group II. Conclusion: Expression of HER2, TP53, and Ki-67 biomarkers and a reduction in the number of hormonal receptors in younger patients (<40YO) indicated that breast cancer might be more invasive in younger women with breast cancer and therefore, they might have poorer prognosis and less favourable outcomes.
[Shandiz, Fatemeh Homaei; Rivadeh, Maryam] Mashhad Univ Med Sci, Canc Res Ctr, Mashhad, Razavi Khorasan, Iran; [Afzaljavan, Fahimeh; Tajbakhsh, Amir; Pasdar, Alireza] Mashhad Univ Med Sci, Fac Med, Dept Med Genet & Mol Med, Mashhad, Razavi Khorasan, Iran; [Sharifi, Nourieh] Mashhad Univ Med Sci, Fac Med, Dept Pathol, Mashhad, Razavi Khorasan, Iran; [Shakeri, Mohammad Taghi] Mashhad Univ Med Sci, Fac Med, Dept Biostat, Mashhad, Razavi Khorasan, Iran; [Pasdar, Alireza] Univ Aberdeen, Med Sch, Div Appl Med, Foresterhill, Aberdeen, Scotland Mashhad University of Medical Sciences; Mashhad University of Medical Sciences; Mashhad University of Medical Sciences; Mashhad University of Medical Sciences; University of Aberdeen Pasdar, A (corresponding author), Mashhad Univ Med Sci, Fac Med, Dept Med Genet & Mol Med, Mashhad, Razavi Khorasan, Iran. Pasdara@mums.ac.ir shakeri, Mohammad/Q-4293-2017; homaee, fateme/AAY-3696-2020; Tajbakhsh, Amir/AAB-1925-2019; Afzaljavan, Fahimeh/AFK-8200-2022; Tajbakhsh,

Amir/HMV-4969-2023 Tajbakhsh, Amir/0000-0002-2311-6554; Pasdar, Alireza/0000-0002-7864-9729; Afzaljavan, Fahimeh/0000-0001-7413-6945 Mashhad University of Medical Sciences Mashhad University of Medical Sciences(Mashhad University of Medical Sciences) We would like to thank Mashhad University of Medical Sciences, Omid and Ghaem hospitals who supported the project.
30 0 0 0 1 SHIRAZ UNIV MEDICAL SCIENCES
SHIRAZ NEMAZEE HOSPITAL, SHIRAZ, 71934, IRAN 2008-6709
2008-6687 MIDDLE EAST J CANCER Middle East J. Cancer
APR 2021 12 2 269 275
10.30476/mejc.2020.83033.1127
http://dx.doi.org/10.30476/mejc.2020.83033.1127
7 Oncology Emerging Sources Citation Index (ESCI)
Oncology SM4OA 2025-06-24
WOS:000657585700013
J Simonsen, NR; Navajos, JFC; Martin-Moreno, JM; Strain, JJ; Huttunen, JK; Martin, BC; Thamm, M; Kardinaal, AFM; van't Veer, P; Kok, FJ; Kohlmeier, L Simonsen, NR; Navajos, JFC; Martin-Moreno, JM; Strain, JJ; Huttunen, JK; Martin, BC; Thamm, M; Kardinaal, AFM; van't Veer, P; Kok, FJ; Kohlmeier, L
Tissue stores of individual monounsaturated fatty acids and breast cancer: the EURAMIC study AMERICAN JOURNAL OF CLINICAL NUTRITION English Article
monounsaturated fat; oleic acid; fatty acids; breast cancer; adipose tissue; olive oil; EURAMIC Study; Europe SUBCUTANEOUS ADIPOSE-TISSUE; DIETARY-FAT; OLIVE OIL; MAMMARY TUMORIGENESIS; RISK-FACTORS; DISEASE; CONSUMPTION; GREECE; COHORT The strongest evidence that monunsaturated fat may influence breast cancer risk comes from studies of southern European populations, in whom intake of oleic acid sources, particularly olive oil, appears protective. No previous study has examined the relation of adipose tissue fatty acid content to breast cancer in such a population. We used adipose biopsies with diverse fat intake patterns gathered in 5 European centers, including southern Europe (Malaga, Spain), to test the hypothesis that stores of oleic acid or other monounsaturates are inversely associated with breast cancer. Gluteal fat aspirates were obtained from 291 postmenopausal incident breast cancer patients and 351 control subjects, frequency-matched for age and catchment area. Logistic regression was used to model breast cancer by monounsaturates, with established risk factors controlled for. Oleic acid showed a strong inverse association with breast cancer in the Spanish center. The odds ratio for the difference between 75th and 25th percentiles was 0.40 (95% CI: 0.28, 0.58) in Malaga and 1.27 (0.88, 1.85) in all other centers pooled, with a peak at 2.36 (1.01, 5.50) for Zeist. Palmitoleic and myristoleic acids showed evidence of an inverse association outside Spain, and cis-vaccenic acid showed a positive association in 3 centers. These data do not support the hypothesis that increasing tissue stores of oleic acid are protective against breast cancer in non-Spanish populations, This finding implies that the strong protective associations reported for olive oil intake in dietary studies may be due to some other protective components of the oil and not to the direct effect of oleic acid uptake. Alternatively, high olive oil intake may indicate some other protective aspect of the lifestyle of these women. Univ N Carolina, Dept Epidemiol, Chapel Hill, NC 27599 USA; Univ Malaga, Fac Med, Dept Prevent Med, E-29071 Malaga, Spain; Univ Ulster, Human Nutr Res Grp, Coleraine

BT52 1SA, Londonderry, North Ireland; Ctr Nacl Epidemiol, Dept Epidemiol & Biostat, Madrid, Spain; Natl Inst Publ Hlth, Helsinki, Finland; Univ Zurich, Inst Social & Prevent Med, CH-8006 Zurich, Switzerland; Robert Koch Inst, D-1000 Berlin, Germany; TNO, Nutr & Food Res Inst, NL-3700 AJ Zeist, Netherlands; Wageningen Univ Agr, Wageningen, Netherlands University of North Carolina; University of North Carolina Chapel Hill; Universidad de Malaga; Ulster University; Instituto de Salud Carlos III; Centro Nacional de Epidemiologia (CNE); University of Zurich; Robert Koch Institute; Netherlands Organization Applied Science Research; Wageningen University & Research Simonsen, NR (corresponding author), Univ N Carolina, Dept Epidemiol, CB 7400,Mc Gavran Greenberg Hall, Chapel Hill, NC 27599 USA. nsimonse@sph.unc.edu
Martin-Moreno, Jose/S-7733-2016 Fernandez-Crehuet Navajas, Joaquin/0000-0002-1173-9906 39 74 80
0 2 AMER SOC CLINICAL NUTRITION BETHESDA 9650 ROCKVILLE PIKE, SUBSCRIPTIONS, RM L-3300, BETHESDA, MD 20814-3998 USA 0002-9165 AM J CLIN NUTR Am. J. Clin. Nutr.
JUL 1998 68 1 134 141
10.1093/ajcn/68.1.134
http://dx.doi.org/10.1093/ajcn/68.1.134 8
Nutrition & Dietetics Science Citation Index Expanded (SCI-EXPANDED) Nutrition & Dietetics ZX598 9665107 Bronze
2025-06-24 WOS:000074534300020
J Buckland, G; Travier, N; Agudo, A; Fonseca-Nunes, A; Navarro, C; Lagiou, P; Demetriou, C; Amiano, P; Dorronsoro, M; Chirlaque, MD; Huerta, JM; Molina, E; Pérez, MJS; Ardanaz, E; Moreno-Iribas, C; Quirós, JR; Naska, A; Trichopoulos, D; Giurdanella, MC; Tumino, R; Agnoli, C; Grioni, S; Panico, S; Mattiello, A; Masala, G; Sacerdote, C; Polidoro, S; Palli, D; Trichopoulou, A; González, CA Buckland, Genevieve; Travier, Noemie; Agudo, Antonio; Fonseca-Nunes, Ana; Navarro, Carmen; Lagiou, Pagona; Demetriou, Christiana; Amiano, Pilar; Dorronsoro, Miren; Chirlaque, Maria-Dolores; Huerta, Jose-Maria; Molina, Esther; Sanchez Perez, Maria-Jose; Ardanaz, Eva; Moreno-Iribas, Conchi; Ramon Quiros, J.; Naska, Androniki; Trichopoulos, Dimitrios; Giurdanella, Maria Concetta; Tumino, Rosario; Agnoli, Claudia; Grioni, Sara; Panico, Salvatore; Mattiello, Amalia; Masala, Giovanna; Sacerdote, Carlotta; Polidoro, Silvia; Palli, Domenico; Trichopoulou, Antonia; Gonzalez, Carlos A. Olive oil intake and breast cancer risk in the Mediterranean countries of the European Prospective Investigation into Cancer and Nutrition study
INTERNATIONAL JOURNAL OF CANCER English
Article breast cancer; olive oil; EPIC cohort study DIET Although there is some evidence suggesting that olive oil could reduce breast cancer (BC) risk, the epidemiological data are still relatively limited, not entirely consistent and mainly based on casecontrol studies. Therefore, we prospectively assessed the association between olive oil and BC risk in postmenopausal women from the Mediterranean cohorts within the European Prospective Investigation into Cancer and Nutrition. The analysis included 62,284 postmenopausal women recruited from Spain, Italy and Greece who had complete dietary data (collected from validated country-specific dietary questionnaires). The risk of BC (overall and by hormone receptor subtypes) was assessed using hazards ratios (HRs) obtained from Cox proportional hazards regression, while adjusting for known BC

risk factors. After a mean follow-up of 9 years, 1,256 women were diagnosed with a primary incident invasive BC. The multivariate HRs for BC risk by olive oil intake (highest vs. lowest tertile of g/day/2,000 kcal) were 1.07 (95% CI = 0.911.25) in the adjusted model, 1.06 (95% CI = 0.911.24) in the model additionally adjusted for reproductive-related factors and 1.10 (95% CI = 0.921.31) for the model additionally adjusted for dietary factors. There was no association between olive oil and risk of estrogen or progesterone receptor-positive tumors, but a suggestion of a negative association with estrogens and progesterone receptor-negative tumors. The results from our prospective study showed that olive oil consumption during adult life was not associated with the risk of BC. However, larger prospective studies are still needed to explore possible differences related to hormone receptor status.

[Buckland, Genevieve; Travier, Noemie; Agudo, Antonio; Fonseca-Nunes, Ana; Gonzalez, Carlos A.] Catalan Inst Oncol ICO IDIBELL, Canc Epidemiol Res Programme, Unit Nutr Environm & Canc, Barcelona, Spain; [Navarro, Carmen; Chirlaque, Maria-Dolores; Huerta, Jose-Maria] Murcia Reg Hlth Author, Dept Epidemiol, Murcia, Spain; [Navarro, Carmen; Amiano, Pilar; Dorronsoro, Miren; Chirlaque, Maria-Dolores; Huerta, Jose-Maria; Molina, Esther; Sanchez Perez, Maria-Jose; Ardanaz, Eva; Moreno-Iribas, Conchi] CIBER Epidemiol & Salud Publ CIBERESP, Barcelona, Spain; [Lagiou, Pagona; Naska, Androniki; Trichopoulou, Antonia] Univ Athens, Sch Med, Dept Hyg Epidemiol & Med Stat, WHO Collaborating Ctr Food & Nutr Policies, GR-11527 Athens, Greece; [Lagiou, Pagona; Trichopoulos, Dimitrios] Harvard Univ, Sch Publ Hlth, Dept Epidemiol, Boston, MA 02115 USA; [Lagiou, Pagona; Trichopoulos, Dimitrios] Acad Athens, Bur Epidemiol Res, Athens, Greece; [Demetriou, Christiana] Univ London Imperial Coll Sci Technol & Med, Sch Publ Hlth, Dept Epidemiol & Biostat, London, England; [Demetriou, Christiana] Cyprus Inst Neurol & Genet, Dept EM Mol Pathol, Nicosia, Cyprus; [Amiano, Pilar; Dorronsoro, Miren] IIS Inst BioDonostia, Publ Hlth Div Gipuzkoa, Basque Hlth Dept, Gipuzkoa, Spain; [Molina, Esther; Sanchez Perez, Maria-Jose] Cuesta Observ, Escuela Andaluza Salud Publ, Granada, Spain; [Ardanaz, Eva; Moreno-Iribas, Conchi] Navarra Publ Hlth Inst, Pamplona, Spain; [Ramon Quiros, J.] Hlth & Hlth Care Serv Council, Asturias, Spain; [Naska, Androniki; Trichopoulou, Antonia] Hellen Hlth Fdn, Athens, Greece; [Giurdanella, Maria Concetta; Tumino, Rosario] Canc Registry, Ragusa, Italy; [Agnoli, Claudia; Grioni, Sara] Fdn IRCCS Ist Nazl Tumori, Nutr Epidemiol Unit, Milan, Italy; [Panico, Salvatore; Mattiello, Amalia] Univ Naples Federico II, Deparment Clin & Expt Med, Naples, Italy; [Masala, Giovanna; Palli, Domenico] Canc Res & Prevent Inst ISPO, Mol & Nutr Epidemiol Unit, Florence, Italy; [Sacerdote, Carlotta] Ctr Canc Prevent CPO Piemonte, Turin, Italy; [Sacerdote, Carlotta; Polidoro, Silvia] Human Genet Fdn HuGeF, Turin, Italy Institut Catala d'Oncologia; Institut d'Investigacio Biomedica de Bellvitge (IDIBELL); Murcia Regional Health Council; CIBER - Centro de Investigacion Biomedica en Red; CIBERESP; Athens Medical School; National & Kapodistrian University of Athens; World Health Organization; Harvard University; Harvard T.H. Chan School of Public Health; Academy of Athens; Imperial College London; Cyprus Institute of Neurology & Genetics; Escuela Andaluza de Salud Publica; Public Health Institute of Navarra; Fondazione IRCCS Istituto Nazionale Tumori Milan; University of Naples Federico II; ISPRO Istituto per lo studio, la prevenzione e la rete oncologica

Buckland, G (corresponding author), Catalan Inst Oncol ICO, Canc Epidemiol Res Programme, Unit Nutr Environm & Canc, Avda Gran Via 199-203, Barcelona 08907, Spain. gbuckland@iconcologia.net
Chirlaque, Maria-Dolores/KCL-3322-2024; Masala, Giovanna/AAC-5474-2022; Sacerdote, Carlotta/K-3611-2018; Trichopoulos, Dimitrios/G-6825-2012; Polidoro, Silvia/GYJ-3517-2022; Demetriou, Christiana A./NIU-0005-2025; TRICHOPOULOU, ANTONIA/ABF-8727-2021; Panico, Salvatore/K-6506-2016; Mattiello, Amalia/K-5112-2016; Agnoli, Claudia/K-5916-2016; Molina-Montes, Esther/AFS-7568-2022; SANCHEZ-PEREZ, MARIA JOSE/D-1087-2011; Huerta, Jose Maria/N-8654-2015; Gonzalez, Carlos A/O-4651-2014; Agudo, Antonio/J-1805-2016; grioni, Sara/K-5320-2016 tumino, rosario/0000-0003-2666-414X; Mattiello, Amalia/0000-0003-3676-7353; Ardanaz, Eva/0000-0001-8434-2013; Agnoli, Claudia/0000-0003-4472-1179; Polidoro, Silvia/0000-0003-2968-0575; Molina-Montes, Esther/0000-0002-0428-2426; SANCHEZ-PEREZ, MARIA JOSE/0000-0003-4817-0757; Huerta, Jose Maria/0000-0002-9637-3869; Panico, Salvatore/0000-0002-5498-8312; Navarro, Carmen/0000-0001-8896-7483; Demetriou, Christiana A./0000-0003-4002-2880; Masala, Giovanna/0000-0002-5758-9069; Sacerdote, Carlotta/0000-0002-8008-5096; Gonzalez, Carlos A/0000-0003-2822-9715; Agudo, Antonio/0000-0001-9900-5677; PALLI, Domenico/0000-0002-5558-2437; Naska, Androniki/0000-0002-1610-1813; grioni, Sara/0000-0002-5891-8426; Buckland, Genevieve/0000-0003-2060-6598; Travier, Noemie/0000-0001-5228-7769
Spanish Ministry of Health [ISCIII RETICC RD06/0020]; Spanish Regional Governments of Andalusia, Asturias, Basque Country, Murcia [6236]; European Commission (DG-SANCO); Navarra; Catalan Institute of Oncology; Hellenic Health Foundation; Stavros Niarchos Foundation (Greece); Compagnia di san Paolo (Naples, Italy) Spanish Ministry of Health(Instituto de Salud Carlos IIISpanish Government); Spanish Regional Governments of Andalusia, Asturias, Basque Country, Murcia; European Commission (DG-SANCO)(European Union (EU)European Commission Joint Research Centre); Navarra; Catalan Institute of Oncology; Hellenic Health Foundation; Stavros Niarchos Foundation (Greece); Compagnia di san Paolo (Naples, Italy)(Compagnia di San Paolo) Grant sponsor: Spanish Ministry of Health; Grant number: ISCIII RETICC RD06/0020; Grant sponsor: Spanish Regional Governments of Andalusia, Asturias, Basque Country, Murcia; Grant number: 6236Grant sponsors: European Commission (DG-SANCO), Navarra, Catalan Institute of Oncology, Hellenic Health Foundation, Stavros Niarchos Foundation (Greece), Compagnia di san Paolo (Naples, Italy) 14 36 37 0 21 WILEY HOBOKEN 111 RIVER ST, HOBOKEN 07030-5774, NJ USA 0020-7136 1097-0215
INT J CANCER Int. J. Cancer NOV 15 2012 131 10 2465 2469 10.1002/ijc.27516
http://dx.doi.org/10.1002/ijc.27516 5
Oncology Science Citation Index Expanded (SCI-EXPANDED)
Oncology 011SM 22392404 2025-06-24
WOS:000309185300028
J Lakkis, NA; Osman, MH; Abdallah, RM
Lakkis, Najla A.; Osman, Mona H.; Abdallah, Reem M.
Cervix Uteri Cancer in Lebanon: Incidence, Temporal Trends, and Comparison to Countries From Different Regions in the World CANCER CONTROL English Article
cervical cancer; epidemiology; cancer screening; incidence; prevention; cancer detection; risk factors
EXTENDED MIDDLE-EAST; HUMAN-PAPILLOMAVIRUS; NORTH-AFRICA;

PREVALENCE; BEIRUT; CHLAMYDIA; COVERAGE; TOBACCO; WOMEN
Background Invasive cervix uteri cancer is the fourth most common malignancy in women globally. This study investigates the incidence and trends of cervix uteri cancer in Lebanon, a country in the Middle East, and compares these rates to regional and global ones. Methods Data on cervix uteri were obtained from the Lebanese national cancer registry for the currently available years 2005 to 2016. The calculated age-standardized incidence and age-specific rates were expressed as per 100,000 population. Results From 2005 to 2016, cervix uteri cancer was the tenth most common cancer among women. Its age-standardized incidence rate fluctuated narrowly between 3.5 and 5.7 per 100,000, with the lowest rate in 2013 and the highest rate in 2012. The age-specific incidence rate had 2 peaks, the highest peak at age group 70-74 years and the second at age group 50-59 years. The annual percent change (+.05%) showed a non-statistically significant trend of increase. The age-standardized incidence rate of cervix uteri cancer in Lebanon was comparable to that of the Western Asia region that has the lowest incidence rate worldwide. The rate was intermediate as compared to other countries in the Middle East and North Africa Region and relatively similar to the ones in Australia, North America, and some Western European countries. Conclusion The incidence rates of invasive cervix uteri are low in Lebanon. This could be attributed to the low prevalence of human papilloma virus infection and other sexually transmitted infections among Lebanese women, and the opportunistic screening practices. It is important to adopt a comprehensive approach to decrease the potential burden of cervix uteri, especially with the rising patterns of risky sexual behaviors. This includes improving awareness, enhancing access to preventive services, developing clinical guidelines, and training health care providers on these guidelines.[Lakkis, Najla A.; Osman, Mona H.] Amer Univ Beirut Med Ctr AUBMC, Dept Family Med, Beirut, Lebanon; [Abdallah, Reem M.] Amer Univ Beirut Med Ctr AUBMC, Dept Obstet & Gynecol, Beirut, Lebanon American University of Beirut; American University of Beirut Abdallah, RM (corresponding author), Amer Univ Beirut, Fac Med, Dept Obstet & Gynecol, Beirut 11072020, Lebanon.
ra102@aub.edu.lb Osman, Mona/LCD-1775-2024 Osman, Mona/0000-0002-6364-6541
61 1 2
0 1 SAGE PUBLICATIONS INC THOUSAND OAKS 2455 TELLER RD, THOUSAND OAKS, CA 91320 USA 1073-2748 1526-2359
CANCER CONTROL Cancer Control JAN 7 2022 29
10732748211068634
10.1177/10732748211068634
http://dx.doi.org/10.1177/10732748211068634 11
Oncology Science Citation Index Expanded (SCI-EXPANDED)
Oncology YL9QW 35012377 Green Published, gold
2025-06-24 WOS:000746221200001
J Karbasi, A; Borhani, N; Daliri, K; Kazemi, B; Manoochehri, M Karbasi, Ashraf; Borhani, Nasim; Daliri, Karim; Kazemi, Bahram; Manoochehri, Mehdi
Downregulation of external death receptor genes FAS and DR5 in colorectal cancer samples positive for human papillomavirus infection PATHOLOGY RESEARCH AND PRACTICE English
Article Colorectal cancer; Human papillomavirus; Apoptosis POLYMERASE-CHAIN-REACTION; MEDIATED APOPTOSIS; COLON-CANCER; DNA; ONCOPROTEINS; ASSOCIATION; VIRUS; ADENOCARCINOMA; PREVALENCE; CARCINOMAS Aim: Human

papillomaviruses (HPV) have frequently been detected in colorectal cancer tumor samples, and may play a role in the pathogenesis of colorectal cancer. This study was designed to investigate the presence of DNA and RNA for the high-risk HPV genotypes 16 and 18 in samples of colorectal cancer tumors and adjacent normal tissues. We also investigated the expression of proapoptotic genes in HPV-positive colorectal tumors compared to normal tissue samples. Methods: Samples of tumoral and adjacent normal tissues were fresh-frozen, and HPV DNA was identified by nested and semiquantitative PCR. Real time PCR was used to quantitatively compare the expression of HPV-18 E6 and nine proapoptotic genes in HPV-positive tumors and samples of adjacent normal tissue. Results: HPV-16 DNA was found in 10.5% of the tumor samples, and HPV-18 DNA was found in 23.6% of the samples. Real time PCR results showed lower expression of the E6 gene in HPV-positive tumors than in adjacent normal tissue. The expression of two proapoptotic genes, FAS and DR5, was significantly lower in tumor samples than in adjacent normal tissues. Conclusions: HPV infection, especially HPV-18, may play a role in colorectal cancer tumorigenesis by downregulating death receptor genes and interfering with the extrinsic pathway of apoptosis. (C) 2015 Elsevier GmbH. All rights reserved. [Karbasi, Ashraf] Baqyiatallah Res Ctr Gastroenterol & Liver Dis, Dept Gastroenterol & Hepatol, Tehran, Iran; [Borhani, Nasim; Kazemi, Bahram; Manoochehri, Mehdi] Shahid Beheshti Univ Med Sci, Cellular & Mol Biol Res Ctr, Tehran, Iran; [Kazemi, Bahram; Manoochehri, Mehdi] Shahid Beheshti Univ Med Sci, Sch Med, Dept Biotechnol, Tehran, Iran; [Daliri, Karim] Shiraz Univ Med Sci, Dept Med Genet, Shiraz, Iran Shahid Beheshti University Medical Sciences; Shahid Beheshti University Medical Sciences; Shiraz University of Medical Science

Manoochehri, M (corresponding author), Shahroud Univ Med Sci, Sch Med, Shahroud, Iran. m.manoochehri@shmu.ac.ir

Kazemi, Bahram/F-2785-2016 Kazemi, Bahram/0000-0002-3072-8831 Iran National Science Foundation (INSF) Iran National Science Foundation (INSF)(Iran National Science Foundation (INSF)) We thank Dr. Seyed Alireza Nadji, Virology Research Center, Masih Daneshvari Hospital, for providing the SiHa and HeLa DNA as positive controls for our PCR assays. We also thank K. Shashok (Author AID in the Eastern Mediterranean) for improving the use of English in the manuscript. This study was supported by the Iran National Science Foundation (INSF). 37 17 21 0

10 ELSEVIER GMBH, URBAN & FISCHER VERLAG JENA OFFICE JENA, P O BOX 100537, 07705 JENA, GERMANY 0344-0338

PATHOL RES PRACT Pathol. Res. Pract. 2015 211 6

444 448

10.1016/j.prp.2015.02.001

http://dx.doi.org/10.1016/j.prp.2015.02.001 5

Pathology Science Citation Index Expanded (SCI-EXPANDED)

Pathology CJ6XV 25795228 2025-06-24

WOS:000355639400004

J Broeks, A; Urbanus, JHM; de Knijff, P; Devilee, P; Nicke, M; Klöpper, K; Dörk, T; Floore, AN; van't Veer, LJ

Broeks, A; Urbanus, JHM; de Knijff, P; Devilee, P; Nicke, M; Klöpper, K; Dörk, T; Floore, AN; van't Veer, LJ

IVS10-6T>G, an ancient ATM germline mutation linked with breast cancer HUMAN MUTATION English Article

ATM; haplotype; breast cancer; germline mutation; founder; Dutch; German; ataxia-telangiectasia;

AT ATAXIA-TELANGIECTASIA LOCUS; PROTEIN TRUNCATION TEST; RAPID DETECTION; GENE-MUTATIONS; FAMILIES; HETEROZYGOSITY; IDENTIFICATION; POPULATIONS; HAPLOTYPES; DEFICIENCY Patients with autosomal recessive multisystemic disorder ataxia-telangiectasia are homozygous or compound heterozygous for mutations in the ataxia-telangiectasia mutated (ATM) gene. Heterozygous carriers of an ATM germline mutation have an increased susceptibility for breast cancer. The subject of this study is one particular germline mutation, the ATM exon 11 splice-site mutation IVS10-6T>G, that has been identified as being associated with an increased risk for breast cancer both in the general population and in high risk breast cancer families. We investigated the natural history of this mutation, i.e., whether it is frequently arising de novo in a population, or whether it can be traced back to a single ancient mutational event. Genotyping of a number of polymorphic markers (two extragenic and two intragenic. microsatellite loci, a single nucleotide insertion/deletion polymorphism, and a dinucleotide insertion/deletion polymorphism) was performed in 18 samples from different populations carrying the IVS10-6T>G mutation (17 unrelated breast cancer patients who were heterozygous carriers of this mutation and a single A-T patient who was homozygous for the IVS10-6T>G mutation). The same markers were also genotyped among 39 unrelated healthy individuals without this mutation. Haplotype analyses revealed one common ancestor in all mutation carriers. By means of a maximum likelihood method, we estimated the age of this mutation to be approximately 2,000 generations. We provide evidence that the IVS10-6T>G mutation occurred only once during human evolution, at least 50,000 years ago. Our results predict that this mutation could be widely distributed across Europe and, probably, the Middle East and Western Asia. (C) 2003 Wiley Liss, Inc. Netherlands Canc Inst, Dept Expt Therapy, NL-1066 CX Amsterdam, Netherlands; Leiden Univ, Med Ctr, Dept Human & Clin Genet, MGC,Forens Lab DNA Res, Leiden, Netherlands; Hannover Med Sch, Dept Radiat Oncol, D-3000 Hannover, Germany; Hannover Med Sch, Clin Obstet & Gynecol, D-3000 Hannover, Germany
Netherlands Cancer Institute; Leiden University; Leiden University Medical Center (LUMC); Leiden University - Excl LUMC; Hannover Medical School; Hannover Medical School Broeks, A (corresponding author), Netherlands Canc Inst, Dept Expt Therapy, Plesmanlaan 121, NL-1066 CX Amsterdam, Netherlands.
DEVILEE, PETER/ABR-2140-2022; de Knijff, Peter/Y-2519-2018; Dork, Thilo/J-8620-2012 de Knijff, Peter/0000-0002-0899-771X; Dork, Thilo/0000-0002-9458-0282 36 23
23 0 4 WILEY-LISS NEW YORK DIV JOHN WILEY & SONS INC, 605 THIRD AVE, NEW YORK, NY 10158-0012 USA 1059-7794
HUM MUTAT Hum. Mutat. MAY 2003 21 5
521 528 10.1002/humu.10204
http://dx.doi.org/10.1002/humu.10204 8
Genetics & Heredity Science Citation Index Expanded (SCI-EXPANDED) Genetics & Heredity 673AX 12673794 gold
2025-06-24 WOS:000182557500008
J Pasta, L; Suero, LAM; Filippazzo, MG; Farinella, EM; Gargano, C; Serravalle, D; D'Amico, N Pasta, Linda; Suero, Leonardo Antonio Mesa; Filippazzo, Maria Gabriella; Farinella, Enzo Massimo; Gargano, Cettina; Serravalle, Domenico; D'Amico, Nicolo The Health Problems at the Landing of the Migrants in Lampedusa from 2011 to June 2019: Analysis of the

Data and Revision of the Literature JOURNAL OF INTERNATIONAL MIGRATION AND INTEGRATION English Article
Abortion spontaneous; Carbon monoxide poisoning; Fracture bone; Human migration; Neurocysticercosis; Seizures; Suicide attempted; Tuberculosis; Rhabdomyolysis
TUBERCULOSIS; REFUGEES; JANUARY This is a study on the migrants hospitalized from 2011 to June 2019, coming from Lampedusa, a small isle in the Mediterranean Sea, called Europe's door, where they arrive along the Sicily Canal Central Route. The physicians of 118 emergency service required the hospitalization for 775 patients (440 men and 335 women) in 6 Sicilian hospitals, mainly in Palermo: 203 in 2011; 62 in 2012; 95 in 2013; 45 in 2014, 184 in 2015; 72 in 2016, 73 in 2017, 35 in 2018, and 6 from January to June 2019. The mean age was 25 years; underage children were about 10%. Their diseases were very similar over the years, irrespective of their country provenance; 227 patients were hospitalized for obstetrics-gynecology problems, 167 infectious diseases (21 TB), 125 orthopedic, 92 intensive care, 89 medicine-cardiology, and 75 for dermatology lesions and burns. In decreasing order, the more frequent provenances were West, Horn, and North Africa and Middle East. The knowledge of the health problems is very important to study of the best practices to activate for these patients. First, help the migrants while they are in the sea, to allow the reduction of the serious health problems, related to the hardships and duration of the long journey, main risk factors for their hospitalization; second, optimize the moment of landing of migrants, for disseminating information on the basic principles of health education. In relation to their young age, these migrants have the mission of perpetuating their ethnic groups, at risk of annihilation by the war violence. [Pasta, Linda; Gargano, Cettina] Casa Cura Candela, Dept Med, Via Valerio Villareale 54, I-90141 Palermo, Italy; [Suero, Leonardo Antonio Mesa; Filippazzo, Maria Gabriella] Fdn Hlth Emergency South World Vincenzo Cervello, Via Trabucco 180, I-90146 Palermo, Italy; [Farinella, Enzo Massimo] Azienda Osped Osped Riuniti Villa Sofia Cervello, Infect Dis Unit, Via Trabucco 180, I-90146 Palermo, Italy; [Serravalle, Domenico] Soc Italiana Sistema 118 Palermo, Trapani, Italy; [Serravalle, Domenico] ARNAS PO Civico, Piazza Nicola Leotta 4, I-90127 Palermo, Italy; [D'Amico, Nicolo] Univ Palermo, Dept Chem Engn, Management, Informat,Mech, I-90128 Palermo, Italy University of Palermo; University of Palermo Pasta, L (corresponding author), Casa Cura Candela, Dept Med, Via Valerio Villareale 54, I-90141 Palermo, Italy. lindpas@yahoo.it Pasta, Linda/0000-0002-8514-3873 20 3 3 0 4
SPRINGER HEIDELBERG HEIDELBERG TIERGARTENSTRASSE 17, D-69121 HEIDELBERG, GERMANY 1488-3473 1874-6365 J INT MIGR INTEGRJ. Int. Migr. Integr. DEC 2020 21 4
1295 1308 10.1007/s12134-019-00719-3
http://dx.doi.org/10.1007/s12134-019-00719-3 NOV 2019
14 Demography Emerging Sources Citation Index (ESCI)
Demography OE8CY 2025-06-24
WOS:000495935800001
J Krusinska, B; Hawrysz, I; Wadolowska, L; Slowinska, MA; Biernacki, M; Czerwinska, A; Golota, JJ
Krusinska, Beata; Hawrysz, Iwona; Wadolowska, Lidia; Slowinska, Malgorzata Anna; Biernacki, Maciej; Czerwinska, Anna; Golota, Janusz Jacek Associations of Mediterranean

Diet and a Posteriori Derived Dietary Patterns with Breast and Lung Cancer Risk: A Case-Control Study NUTRIENTS
English Article breast cancer; lung cancer; dietary pattern; Mediterranean diet; adults
INDEX; ADHERENCE; COHORT; WOMEN; FOOD Lung cancer in men and breast cancer in women are the most commonly diagnosed cancers in Poland and worldwide. Results of studies involving dietary patterns (DPs) and breast or lung cancer risk in European countries outside the Mediterranean Sea region are limited and inconclusive. This study aimed to develop a 'Polish-adapted Mediterranean Diet' ('Polish-aMED') score, and then study the associations between the 'Polish-aMED' score and a posteriori-derived dietary patterns with breast or lung cancer risk in adult Poles. This pooled analysis of two case-control studies involved 560 subjects (280 men, 280 women) aged 40-75 years from Northeastern Poland. Diagnoses of breast cancer in 140 women and lung cancer in 140 men were found. The food frequency consumption of 21 selected food groups was collected using a 62-item Food Frequency Questionnaire (FFQ)-6. The 'Polish-adapted Mediterranean Diet' score which included eight items-vegetables, fruit, whole grain, fish, legumes, nuts and seeds-as well as the ratio of vegetable oils to animal fat and red and processed meat was developed (range: 0-8 points). Three DPs were identified in a Principal Component Analysis: 'Prudent', 'Non-healthy', 'Dressings and sweetened-low-fat dairy'. In a multiple logistic regression analysis, two models were created: crude, and adjusted for age, sex, type of cancer, Body Mass Index (BMI), socioeconomic status (SES) index, overall physical activity, smoking status and alcohol abuse. The risk of breast or lung cancer was lower in the average (3-5 points) and high (6-8 points) levels of the 'Polish-aMED' score compared to the low (0-2 points) level by 51% (odds ratio (OR): 0.49; 95% confidence interval (CI): 0.30-0.80; $p < 0.01$; adjusted) and 63% (OR: 0.37; 95% CI: 0.21-0.64; $p < 0.001$; adjusted), respectively. In the middle and upper tertiles compared to the bottom tertile of the 'Prudent' DP, the risk of cancer was lower by 38-43% (crude) but was not significant after adjustment for confounders. In the upper compared to the bottom tertile of the 'Non-healthy' DP, the risk of cancer was higher by 65% (OR: 1.65; 95% CI: 1.05-2.59; $p < 0.05$; adjusted). In conclusion, the Polish adaptation of the Mediterranean diet could be considered for adults living in non-Mediterranean countries for the prevention of the breast or lung cancers. Future studies should explore the role of a traditional Mediterranean diet fitted to local dietary patterns of non-Mediterranean Europeans in cancer prevention.[Krusinska, Beata; Hawrysz, Iwona; Wadolowska, Lidia; Slowinska, Malgorzata Anna] Univ Warmia & Mazury, Dept Human Nutr, Sloneczna 45f, PL-10718 Olsztyn, Poland; [Biernacki, Maciej] Univ Warmia & Mazury, Dept Surg, PL-11041 Olsztyn, Poland; [Czerwinska, Anna] Independent Publ Complex TB & Lung Dis Olsztyn, PL-10357 Olsztyn, Poland; [Golota, Janusz Jacek] Med Ctr Ars Med, Clin Thorac Surg, PL-10513 Olsztyn, Poland University of Warmia & Mazury; University of Warmia & Mazury Krusinska, B (corresponding author), Univ Warmia & Mazury, Dept Human Nutr, Sloneczna 45f, PL-10718 Olsztyn, Poland.
beata.krusinska@uwm.edu.pl; iwona.hawrysz@uwm.edu.pl; lidia.wadolowska@uwm.edu.pl; malgorzata.slowinska@uwm.edu.pl; maciej.biernacki@uwm.edu.pl; aczerwinska@pulmonologia.olsztyn.pl; januszgolota@vp.pl Wądołowska, Lidia/L-1458-2019; Wadolowska,

Lidia/O-8463-2018	Slowinska, Malgorzata/0000-0003-3836-8858; Stasiewicz, Beata/0000-0003-0718-9101; Wadolowska, Lidia/0000-0001-8571-9935; , Iwona/0000-0002-3430-5721	Polish Ministry of Science and Higher EducationPolish Ministry of Science and Higher Education(Ministry of Science and Higher Education, Poland)
Thanks are expressed to the participants for their contribution to the study. Thanks are expressed to the Krystyna Solnicka from the Breast Prevention and Diagnosis Center in Olsztyn for cooperation. Research relating to this paper came from Department of Human Nutrition, Faculty of Food Science, University of Warmia and Mazury in Olsztyn and was funded by Polish Ministry of Science and Higher Education.	48	64	65	0	11
MDPI	BASEL	ST ALBAN-ANLAGE 66, CH-4052 BASEL, SWITZERLAND
2072-6643		NUTRIENTS	Nutrients	APR	2018	10
4						470
10.3390/nu10040470	http://dx.doi.org/10.3390/nu10040470
15	Nutrition & Dietetics	Science Citation Index Expanded (SCI-EXPANDED)	Nutrition & Dietetics	GJ3HN
29641468	Green Submitted, Green Published, gold
2025-06-24	WOS:000435182900086
J	Clifford, GM; Tenet, V; Georges, D; Alemany, L; Pavón, MA; Chen, ZG; Yeager, M; Cullen, M; Boland, JF; Bass, S; Steinberg, M; Raine-Bennett, T; Lorey, T; Wentzensen, N; Walker, J; Zuna, R; Schiffman, M; Mirabello, L			Clifford, Gary M.; Tenet, Vanessa; Georges, Damien; Alemany, Laia; Angel Pavon, Miquel; Chen, Zigui; Yeager, Meredith; Cullen, Michael; Boland, Joseph F.; Bass, Sara; Steinberg, Mia; Raine-Bennett, Tina; Lorey, Thomas; Wentzensen, Nicolas; Walker, Joan; Zuna, Rosemary; Schiffman, Mark; Mirabello, Lisa			Human papillomavirus 16 sub-lineage dispersal and cervical cancer risk worldwide: Whole viral genome sequences from 7116 HPV16-positive women
PAPILLOMAVIRUS RESEARCH			English	Article
HPV16; Cervical cancer; HPV carcinogenesis; HPV epidemiology; HPV genomics; Whole virus genome sequencing	TYPE-18 VARIANTS; NATURAL-HISTORY; NEOPLASIA; INFECTION; E6; ADENOCARCINOMA; CLASSIFICATION; POLYMORPHISMS; PERSISTENCE; PREVALENCE	Background: Human papillomavirus (HPV)16 can be separated into genetic sub-lineages (A1-4, B1-4, C1-4, D1-4) which may have differential cervical cancer risk. Methods: A next-generation sequencing assay was used to whole-genome sequence 7116 HPV16-positive cervical samples from well-characterised international epidemiological studies, including 2076 controls, 1878 squamous cell carcinoma (SCC) and 186 adenocarcinoma/adenosquamous cell carcinoma (ADC), and to assign HPV16 sub-lineage. Logistic regression was used to estimate region-stratified country-adjusted odds ratios (OR) and 95%CI. Results: A1 was the most globally widespread sub-lineage, with others showing stronger regional specificity (A3 and A4 for East Asia, B1-4 and C1-4 for Africa, D2 for the Americas, B4, C4 and D4 for North Africa). Increased cancer risks versus A1 were seen for A3, A4 and D (sub)lineages in regions where they were common: A3 in East Asia (OR = 2.2, 95%CI:1.0-4.7); A4 in East Asia (6.6, 3.1-14.1) and North America (3.8, 1.7-8.3); and D in North (6.2, 4.1-9.3) and South/Central America (2.2, 0.8-5.7), where D lineages were also more frequent in ADC than SCC (3.2, 1.5-6.5; 12.1, 5.7-25.6, respectively). Conclusions: HPV16 genetic variation can strongly influence cervical cancer risk. However, burden of cervical cancer attributable to different sub-lineages worldwide

is largely driven by historical HPV16 sub-lineage dispersal.

[Clifford, Gary M.; Tenet, Vanessa; Georges, Damien] Int Agcy Res Canc, 150 Cours Albert Thomas, F-69372 Lyon 08, France; [Alemany, Laia; Angel Pavon, Miquel] Bellvitge Inst Biomed Res, Catalan Inst Oncol, Canc Epidemiol Res Program, Infect & Canc Unit, Barcelona, Spain; [Alemany, Laia] CIBER Epidemiol & Salud Publ CIBERESP, Barcelona, Spain; [Angel Pavon, Miquel] CIBER Oncol CIBERONC, Barcelona, Spain; [Chen, Zigui] Chinese Univ Hong Kong, Fac Med, Dept Microbiol, Hong Kong, Peoples R China; [Yeager, Meredith; Cullen, Michael; Boland, Joseph F.; Bass, Sara; Steinberg, Mia; Wentzensen, Nicolas; Schiffman, Mark; Mirabello, Lisa] NCI, Div Canc Epidemiol & Genet, NIH, Rockville, MD USA; [Yeager, Meredith; Cullen, Michael; Boland, Joseph F.; Bass, Sara; Steinberg, Mia] Leidos Biomed Res Inc, Canc Genom Res Lab, Frederick, MD USA; [Raine-Bennett, Tina] Kaiser Permanente Northern Calif, Womens Hlth Res Inst, Div Res, Oakland, CA USA; [Lorey, Thomas] Kaiser Permanente Northern Calif, Reg Lab, Oakland, CA USA; [Walker, Joan; Zuna, Rosemary] Univ Oklahoma, Hlth Sci Ctr, Oklahoma City, OK USA   World Health Organization; International Agency for Research on Cancer (IARC); Institut d'Investigacio Biomedica de Bellvitge (IDIBELL); Institut Catala d'Oncologia; CIBER - Centro de Investigacion Biomedica en Red; CIBERESP; CIBER - Centro de Investigacion Biomedica en Red; CIBERONC; Chinese University of Hong Kong; National Institutes of Health (NIH) - USA; NIH National Cancer Institute (NCI); NIH National Cancer Institute- Division of Cancer Epidemiology & Genetics; National Institutes of Health (NIH) - USA; NIH National Cancer Institute (NCI); Frederick National Laboratory for Cancer Research; Kaiser Permanente; Kaiser Permanente; University of Oklahoma System; University of Oklahoma Health Sciences Center

Clifford, GM (corresponding author), Int Agcy Res Canc, 150 Cours Albert Thomas, F-69372 Lyon 08, France.

CliffordG@iarc.fr; TenetV@iarc.fr; GeorgesD@iarc.fr; lalemany@iconcologia.net; mpavon@iconcologia.net; zigui.chen@cuhk.edu.hk; yeagerm@mail.nih.gov; michael.cullen@nih.gov; bolandj2@mail.nih.gov; sara.bass2@nih.gov; mia.steinberg@nih.gov; Tina.R.Raine-Bennett@kp.org; thomas.lorey@kp.org; wentzenn@mail.nih.gov; joan-walker@ouhsc.edu; rosemary-zuna@ouhsc.edu; schiffmm@exchange.nih.gov; mirabellol@mail.nih.gov   Wentzensen, Nicolas/AAG-8522-2019; Yeager, Meredith/AAA-5322-2020; Alemany, Laia/G-9643-2015; Mirabello, Lisa/H-5594-2018; Schiffman, Mark/B-9766-2015; CHEN, Zigui/E-8490-2017; Pavon, Miguel Angel/N-3538-2014 CHEN, Zigui/0000-0002-8577-1298; Damien, Georges/0000-0003-2425-7591; Pavon, Miguel Angel/0000-0003-3677-5329; Mirabello, Lisa/0000-0001-8485-0106   Institut National Du Cancer, France (HPV Genomics) [2016-127]   Institut National Du Cancer, France (HPV Genomics)   The work at IARC was supported by the Institut National Du Cancer, France (HPV Genomics; grant number 2016-127). The funder had no role in study design, data collection, analysis and interpretation, in the writing of the report or in the decision to submit the work for publication.   42   62   66
0   3   ELSEVIER SCIENCE BV   AMSTERDAM   PO BOX 211, 1000 AE AMSTERDAM, NETHERLANDS   2405-8521
PAPILLOMAVIRUS RES   Papillomavirus Res.   JUN   2019   7
67   74
10.1016/j.pvr.2019.02.001
http://dx.doi.org/10.1016/j.pvr.2019.02.001   8

Virology Emerging Sources Citation Index (ESCI) Virology IA8HU 30738204 Green Published, gold 2025-06-24 WOS:000469800500010
J Fung, TT; Hu, FB; McCullough, ML; Newby, PK; Willett, WC; Holmes, MD Fung, TT; Hu, FB; McCullough, ML; Newby, PK; Willett, WC; Holmes, MD Diet quality is associated with the risk of estrogen receptor-negative breast cancer in postmenopausal women. JOURNAL OF NUTRITION English Article breast cancer; diet; nutrition; estrogen receptor CORONARY-HEART-DISEASE; MAJOR CHRONIC DISEASE; POOLED ANALYSIS; COHORT; QUESTIONNAIRE; CONSUMPTION; VEGETABLES; MORTALITY; PATTERNS; FRUITS Emerging evidence suggests that diet quality indices may serve as prognostic indicators of disease. However, the ability of these indices to predict breast cancer risk has not been evaluated previously. We assessed the association between several diet quality scores and the risk of breast cancer in postmenopausal women. The indices we used were the Healthy Eating Index (HEI), Alternate Healthy Eating Index (AHEI), Diet Quality Index-Revised (DQI-R), Recommended Food Score (RFS), and the alternate Mediterranean Diet Score (aMed). We calculated diet quality indices from dietary information collected in FFQ administered 5 times between 1984 and 1998 among women in the Nurses' Health Study cohort. Relative risks (RR) were computed using Cox proportional hazards models and adjusted for known risk factors for breast cancer. Separate analyses were conducted for estrogen receptor positive (ER+) and negative (ER-) tumors. Between 1984 and 2002, we documented 3580 cases of breast cancer, of which 2367 were ER+, and 575 were ER-. We did not observe any association between the diet quality indices and total or ER+ breast cancer risk. However, for ER- breast cancer, after adjusting for potential confounders, the FIR comparing highest to lowest quintiles were 0.78 (95% Cl = 0.59-1.04, P for trend = 0.01) for the AHEI, 0.69 (95% Cl = 0.51-0.94, P for trend = 0.003) for the RFS, and 0.79 (95% Cl = 0.60-1.03, P for trend = 0.03) for the aMed. These observations appeared to be the result of an inverse association (P for trend = 0.01) with the vegetable component of the scores. We conclude that women who scored high in AHEI, RFS, and aMed had a lower risk of ER- breast cancer. The HEI and DQI-R appeared to be of limited value in predicting breast cancer risk. Simmons Coll, Dept Nutr, Boston, MA 02115 USA; Harvard Univ, Sch Publ Hlth, Dept Nutr, Boston, MA 02115 USA; Amer Canc Soc, Atlanta, GA 30329 USA; Tufts Univ, Jean Mayer US Dept Agr, Human Nutr Res Ctr Aging, Boston, MA 02111 USA; Harvard Univ, Sch Publ Hlth, Dept Epidemiol, Boston, MA 02115 USA; Harvard Univ, Sch Med, Brigham & Womens Hosp, Channing Lab,Dept Med, Boston, MA 02115 USA Simmons University; Harvard University; Harvard T.H. Chan School of Public Health; American Cancer Society; United States Department of Agriculture (USDA); Tufts University; Harvard University; Harvard T.H. Chan School of Public Health; Harvard University; Harvard University Medical Affiliates; Brigham & Women's Hospital; Harvard Medical School Fung, TT (corresponding author), Simmons Coll, Dept Nutr, Boston, MA 02115 USA. fung@simmons.edu Hu, Frank/C-1919-2013; Willett, Walter/E-2352-2013 McCullough, Marjorie/0000-0003-3025-6341 NCI NIH HHS [CA095589, CA87969] Funding Source: Medline NCI NIH HHS(United States Department of Health & Human ServicesNational Institutes of Health (NIH) - USANIH National Cancer Institute

(NCI)) 31 248 277 0 19 AMER SOCIETY NUTRITIONAL SCIENCE BETHESDA 9650 ROCKVILLE PIKE, RM L-2407A, BETHESDA, MD 20814 USA 0022-3166 J NUTR J. Nutr. FEB 2006 136 2 466 472 10.1093/jn/136.2.466 http://dx.doi.org/10.1093/jn/136.2.466 7 Nutrition & Dietetics Science Citation Index Expanded (SCI-EXPANDED) Nutrition & Dietetics 006JU 16424129 Bronze 2025-06-24 WOS:000234894600021
J El Hilali, H; El Hilali, F; Porter, SEG; Ghali, SA; Meyls, HM; Ouazzani, N; Laziri, F; Barber, A El Hilali, Hajar; El Hilali, Fatiha; Porter, Sarah E. G.; Ghali, Sarah A.; Meyls, Hannah M.; Ouazzani, Noureddine; Laziri, Fatiha; Barber, Amorette Olive oil varieties cultivated in Morocco reduce reactive oxygen species and cell viability of human cervical cancer cells MEDITERRANEAN JOURNAL OF NUTRITION AND METABOLISM English Article Antioxidant; cervical cancer; gene expression; olive oil VIRGIN OLIVE; OXIDATIVE STRESS; DIETARY LIPIDS; PHENOLS; ANTIOXIDANT; EXPRESSION; CARCINOMA; LIVER; ACID BACKGROUND: The Moroccan diet incorporates olive oil as the primary source of fat and may reduce cancer risk. However, different olive oil varieties often have varying levels of anti-cancer polyphenols and thus have unique biologic effects. OBJECTIVE: The anti-cancer activity of five varieties of extra virgin Moroccan-cultivated olive oil on human cervical cancer cells was assessed in vitro. METHODS: The presence of phenolic compounds in five olive oil varieties cultivated in Morocco was analyzed using HPLC. Human cervical cancer cell lines (HeLa, SKG-II, and HCS-2) were incubated with the olive oils and cell viability was measured by MTT assay, reactive oxygen species were measured using the CellRox assay, and gene expression was measured by RT-PCR. RESULTS: Each of the five Moroccan-cultivated olive oil varieties had a unique composition of phenolic compounds. Incubation with the olive oils reduced cell viability and reactive oxygen species in human cervical cancer cells. The expression of genes involved in cervical cancer carcinogenesis and cell cycle were also altered. All five olive oil varieties decreased expression of E6, E7, p16, p63, and NRP2 and increased expression of IVL and miR 331-3p. CONCLUSIONS: Use of Moroccan-cultivated olive oils could be a promising anti-cancer agent for cervical cancer. [El Hilali, Hajar; El Hilali, Fatiha; Laziri, Fatiha] Moulay Ismail Univ, Dept Biol, Meknes, Morocco; [Porter, Sarah E. G.; Ghali, Sarah A.; Meyls, Hannah M.] Longwood Univ, Dept Chem & Phys, Farmville, VA 23909 USA; [Ouazzani, Noureddine] Natl Sch Agr, Agropole Olivier, Meknes, Morocco; [Barber, Amorette] Longwood Univ, Dept Biol & Environm Sci, 201 High St, Farmville, VA 23909 USA Moulay Ismail University of Meknes; Longwood University; Moulay Ismail University of Meknes; Longwood University Barber, A (corresponding author), Longwood Univ, Dept Biol & Environm Sci, 201 High St, Farmville, VA 23909 USA. barberar@longwood.edu Longwood University's Faculty Research Grants; Department of Biological and Environmental Sciences Longwood University's Faculty Research Grants; Department of Biological and Environmental Sciences This work was supported by in part by Longwood University's Faculty Research Grants and the Department of Biological and Environmental Sciences. We are thankful to Dr. Nacer Bellaloui for reviewing and editing the manuscript. 44 2 2 2 13 IOS

PRESS AMSTERDAM NIEUWE HEMWEG 6B, 1013 BG AMSTERDAM, NETHERLANDS
1973-798X 1973-7998 MEDITERR J NUTR META Mediterr. J. Nutr. Metab. 2020 13 1 89
100 10.3233/MNM-190390
http://dx.doi.org/10.3233/MNM-190390 12
Medicine, General & Internal; Nutrition & Dietetics
Emerging Sources Citation Index (ESCI) General & Internal Medicine; Nutrition & Dietetics KU4SU
2025-06-24 WOS:000519701300008
J Hirko, KA; Soliman, AS; Banerjee, M; Ruterbusch, J; Harford, JB; Chamberlain, RM; Graff, JJ; Merajver, SD; Schwartz, K
Hirko, Kelly A.; Soliman, Amr S.; Banerjee, Mousumi; Ruterbusch, Julie; Harford, Joe B.; Chamberlain, Robert M.; Graff, John J.; Merajver, Sofia D.; Schwartz, Kendra
Characterizing inflammatory breast cancer among Arab Americans in the California, Detroit and New Jersey Surveillance, Epidemiology and End Results (SEER) registries (1988-2008)
SPRINGERPLUS English Article
Inflammatory breast cancer; Arab; Race; Hierarchical logistic regression MENOPAUSAL STATUS; RISK-FACTORS; WOMEN; CARCINOMA; SURVIVAL Introduction: Inflammatory breast cancer (IBC) is characterized by an apparent geographical distribution in incidence, being more common in North Africa than other parts of the world. Despite the rapid growth of immigrants to the United States from Arab nations, little is known about disease patterns among Arab Americans because a racial category is rarely considered for this group. The aim of this study was to advance our understanding of the burden of IBC in Arab ethnic populations by describing the proportion of IBC among different racial groups, including Arab Americans from the Detroit, New Jersey and California Surveillance, Epidemiology and End Results (SEER) registries. Methods: We utilized a validated Arab surname algorithm to identify women of Arab descent from the SEER registries. Differences in the proportion of IBC out of all breast cancer and IBC characteristics by race and menopausal status were evaluated using chi-square tests for categorical variables, t-tests and ANOVA tests for continuous variables, and log-rank tests for survival data. We modeled the association between race and IBC among all women with breast cancer using hierarchical logistic regression models, adjusting for individual and census tract-level variables. Results: Statistically significant differences in the proportion of IBC out of all breast cancers by race were evident. In a hierarchical model, adjusting for age, estrogen and progesterone receptor, human epidermal growth receptor 2, registry and census-tract level education, Arab-Americans (OR = 1.5, 95% CI = 1.2,1.9), Hispanics (OR= 1.2, 95% CI = 1.1,1.3), Non-Hispanic Blacks (OR = 1.3, 95% CI = 1.2, 1.4), and American Indians/Alaskans (OR= 1.9, 95% CI = 1.1, 3.4) had increased odds of IBC, while Asians (OR = 0.6, 95% CI = 0.6, 0.7) had decreased odds of IBC as compared to Non-Hispanic Whites. Conclusions: IBC may be more common among certain minority groups, including Arab American women. Understanding the descriptive epidemiology of IBC by race may generate hypotheses about risk factors for this aggressive disease. Future research should focus on etiologic factors that may explain these differences. [Hirko, Kelly A.; Chamberlain, Robert M.; Merajver, Sofia D.] Univ Michigan, Sch Publ Hlth, Dept Epidemiol, Ann Arbor, MI 48109 USA; [Hirko, Kelly A.; Merajver, Sofia D.] Univ Michigan, Ctr Global Hlth, Ann Arbor,

MI 48104 USA; [Soliman, Amr S.] Univ Nebraska Med Ctr, Dept Epidemiol, Omaha, NE 68198 USA; [Banerjee, Mousumi] Univ Michigan, Sch Publ Hlth, Dept Biostat, Ann Arbor, MI 48109 USA; [Ruterbusch, Julie] Wayne State Univ, Sch Med, Dept Oncol, Detroit, MI USA; [Harford, Joe B.] NCI, Dept Hlth & Human Serv, Ctr Global Hlth, NIH, Bethesda, MD 20892 USA; [Chamberlain, Robert M.] Univ Texas MD Anderson Canc Ctr, Dept Epidemiol, Houston, TX 77030 USA; [Graff, John J.] Univ Med & Dent New Jersey, Robert Wood Johnson Med Sch, Canc Inst New Jersey, Dept Radiat Oncol, New Brunswick, NJ 08901 USA; [Merajver, Sofia D.] Univ Michigan, Sch Med, Dept Internal Med, Ann Arbor, MI 48109 USA; [Schwartz, Kendra] Wayne State Univ, Sch Med, Dept Family Med & Publ Hlth Sci, Detroit, MI USA University of Michigan System; University of Michigan; University of Michigan System; University of Michigan; University of Nebraska System; University of Nebraska Medical Center; University of Michigan System; University of Michigan; Wayne State University; National Institutes of Health (NIH) - USA; NIH National Cancer Institute (NCI); University of Texas System; UTMD Anderson Cancer Center; Rutgers University System; Rutgers University New Brunswick; Rutgers University Biomedical & Health Sciences; Rutgers Cancer Institute of New Jersey; University of Michigan System; University of Michigan; Wayne State University
Hirko, KA (corresponding author), Univ Michigan, Sch Publ Hlth, Dept Epidemiol, Ann Arbor, MI 48109 USA.
kellyannelamb@gmail.com Hirko, Kelly/AFN-8256-2022; Chamberlain, Robert/AAR-4675-2020 Harford, Joe Bryan/0000-0002-6681-6315; hirko, kelly/0000-0002-0050-655X Avon Foundation; Breast Cancer Research Foundation; University of Michigan Center for Global Health and Rackham School of Graduate studies; Cancer Epidemiology Education in Special Populations Program of the University of Michigan [CA R25 112383] Avon Foundation; Breast Cancer Research Foundation; University of Michigan Center for Global Health and Rackham School of Graduate studies; Cancer Epidemiology Education in Special Populations Program of the University of Michigan(University of Michigan System) We would like to thank Xiaoling Niu from the New Jersey SEER Cancer Registry and Allyn Fernandez-Ami and Dr. Cyllene Morris from the California Cancer Registry for their efforts in compiling the datasets for our analysis. The authors also would like to thank Kirsten Herold for her review and comments on the manuscript. This work supported in part by the Avon Foundation (AS,SDM), the Breast Cancer Research Foundation (SDM), The University of Michigan Center for Global Health and Rackham School of Graduate studies (KH) and the Cancer Epidemiology Education in Special Populations Program of the University of Michigan (CA R25 112383). 41
13 15 0 2 SPRINGER INT PUBL AG CHAM
GEWERBESTRASSE 11, CHAM, CH-6330, SWITZERLAND 2193-1801
SPRINGERPLUS SpringerPlus 2013 2
3 10.1186/2193-1801-2-3 http://dx.doi.org/10.1186/2193-1801-2-3 8
Multidisciplinary Sciences Science Citation Index Expanded (SCI-EXPANDED) Science & Technology - Other Topics V40EM
23420611 Green Published, gold 2025-06-24
WOS:000209461900003
J Sina, M; Ghorbanoghli, Z; Abedrabbo, A; Al-Mulla, F; Ben Sghaier, R; Buisine, MP; Cortas, G; Goshayeshi, L; Hadjisavvas, A; Hammoudeh, W; Hamoudi, W; Jabari, C; Loizidou, MA; Majidzadeh-A, K; Marafie, MJ; Muslumov, G; Rifai, L; Seir, RA; Talaat, SM;

Tunca, B; Ziada-Bouchaar, H; Velthuizen, ME; Sharara, AI; Ahadova, A; Georgiou, D; Vasen, HFA Sina, Mohammad; Ghorbanoghli, Zeinab; Abedrabbo, Amal; Al-Mulla, Fahd; Ben Sghaier, Rihab; Buisine, Marie-Pierre; Cortas, George; Goshayeshi, Ladan; Hadjisavvas, Andreas; Hammoudeh, Wail; Hamoudi, Waseem; Jabari, Carol; Loizidou, Maria A.; Majidzadeh-A, Keivan; Marafie, Makia J.; Muslumov, Gurbankhan; Rifai, Laila; Seir, Rania Abu; Talaat, Suzan M.; Tunca, Berrin; Ziada-Bouchaar, Hadia; Velthuizen, Mary E.; Sharara, Ala I.; Ahadova, Aysel; Georgiou, Demetra; Vasen, Hans F. A. Middle East Netw Identification and management of Lynch syndrome in the Middle East and North African countries: outcome of a survey in 12 countries FAMILIAL CANCER English Article Colorectal cancer; Lynch syndrome; Middle Eastern countries; North African countries COLORECTAL-CANCER; HEREDITARY; PREVALENCE; MORTALITY; FAMILIES Background Lynch syndrome (LS), the most common inherited form of colorectal cancer (CRC), is responsible for 3% of all cases of CRC. LS is caused by a mismatch repair gene defect and is characterized by a high risk for CRC, endometrial cancer and several other cancers. Identification of LS is of utmost importance because colonoscopic surveillance substantially improves a patient's prognosis. Recently, a network of physicians in Middle Eastern and North African (ME/NA) countries was established to improve the identification and management of LS families. The aim of the present survey was to evaluate current healthcare for families with LS in this region. Methods A questionnaire was developed that addressed the following issues: availability of clinical management guidelines for LS; attention paid to family history of cancer; availability of genetic services for identification and diagnosis of LS; and assessment of knowledge of LS surveillance. Members of the network and authors of recent papers on LS from ME/NA and neighbouring countries were invited to participate in the survey and complete the online questionnaire. Results A total of 55 individuals were invited and 19 respondents from twelve countries including Algeria, Azerbaijan, Cyprus, Egypt, Iran, Jordan, Kuwait, Lebanon, Morocco, Palestine, Tunisia, and Turkey completed the questionnaire. The results showed that family history of CRC is considered in less than half of the surveyed countries. Guidelines for the management of LS are available in three out of twelve countries. The identification and selection of families for genetic testing were based on clinical criteria (Amsterdam criteria II or Revised Bethesda criteria) in most countries, and only one country performed universal screening. In most of the surveyed countries genetic services were available in few hospitals or only in a research setting. However, surveillance of LS families was offered in the majority of countries and most frequently consisted of regular colonoscopy. Conclusion The identification and management of LS in ME/NA countries are suboptimal and as a result most LS families in the region remain undetected. Future efforts should focus on increasing awareness of LS amongst both the general population and doctors, and on the improvement of the infrastructure in these countries. [Sina, Mohammad; Majidzadeh-A, Keivan] ACECR, Motamed Canc Inst, Breast Canc Res Ctr, Dept Genet, Tehran, Iran; [Sina, Mohammad] Univ Brescia, Dept Mol & Translat Med, A Nocivelli Inst Mol Med, I-25123 Brescia, Italy; [Ghorbanoghli, Zeinab; Vasen, Hans F. A.] Leiden Univ, Med Ctr, Dept Gastroenterol & Hepatol, Albinusdreef

2, NL-2333 ZA Leiden, Netherlands; [Ghorbanoghli, Zeinab; Vasen, Hans F. A.] Dutch Hereditary Canc Registry, Leiden, Netherlands; [Abedrabbo, Amal] Makassed Islamic Charitable Hosp, Dept Pediat, Jerusalem, Palestine; [Al-Mulla, Fahd] Dasman Diabet Inst, Dept Genet & Bioinformat, POB 1180, Kuwait 15462, Kuwait; [Ben Sghaier, Rihab] HACHED Hosp, Cytogenet Mol Genet & Human Reprod Biol Farhat, Sousse, Tunisia; [Buisine, Marie-Pierre] Lille Univ Hosp, Inst Biochem & Mol Biol, Unit Mol Oncol & Genet, Lille, France; [Cortas, George] Univ Balamand, Sch Med, St George Hosp, Dept Gastroenterol,Med Ctr, Beirut, Lebanon; [Goshayeshi, Ladan] Mashhad Univ Med Sci, Fac Med, Dept Gastroenterol & Hepatol, Mashhad, Razavi Khorasan, Iran; [Hadjisavvas, Andreas; Loizidou, Maria A.] Cyprus Inst Neurol & Genet, Dept Electron Microscopy Mol Pathol, Nicosia, Cyprus; [Hammoudeh, Wail] Arabcare Hosp, Dept Internal Med, Ramallah, Palestine; [Hamoudi, Waseem] Royal Hosp, Dept Gastroenterol, Amman, Jordan; [Jabari, Carol] Patients Friends Soc, Jerusalem, Palestine; [Jabari, Carol] Hebron Univ, Hebron, Palestine; [Marafie, Makia J.] Matern Hosp, Kuwait Med Genet Ctr, Safat 13059, Kuwait; [Muslumov, Gurbankhan] Surg Sci Ctr, Dept Colorectal Surg, Baku, Azerbaijan; [Rifai, Laila] Inst Natl DOncol Sidi Mohamed Ben Abdellah, Rabat Inst, Ctr Hosp Univ IBN SINA, BP 6213, Rabat, Morocco; [Seir, Rania Abu] Al Quds Univ, Abu Dis, Palestine; [Talaat, Suzan M.] Ahmed Maher Teaching Hosp, Cairo, Egypt; [Tunca, Berrin] Uludag Univ, Fac Med, Dept Med Biol, Bursa, Turkey; [Ziada-Bouchaar, Hadia] Univ 3 Rabah Bitat, Fac Med, Lab Biol & Mol Genet, Constantine, Constantine, Algeria; [Velthuizen, Mary E.] Univ Med Ctr Utrecht Locat WKZ, Dept Genet, Utrecht, Netherlands; [Sharara, Ala I.] Amer Univ Beirut, Med Ctr, Div Gastroenterol, Beirut, Lebanon; [Ahadova, Aysel] Univ Hosp Heidelberg, Inst Pathol, Dept Appl Tumour Biol, Heidelberg, Germany; [Ahadova, Aysel] German Canc Res Ctr, Cooperat Unit Appl Tumour Biol, Heidelberg, Germany; [Georgiou, Demetra] London North West Univ Healthcare, Dept Clin Genet, London, England Academic Center for Education, Culture & Research (ACECR); University of Brescia; Leiden University; Leiden University Medical Center (LUMC); Leiden University - Excl LUMC; Dasman Diabetes Institute (DDI); Universite de Lille; CHU Lille; University Balamand; Mashhad University of Medical Sciences; Cyprus Institute of Neurology & Genetics; Mohammed V University in Rabat; Ibn sina University Hospital Center of Rabat; Al-Quds University; Egyptian Knowledge Bank (EKB); General Organization of Teaching Hospitals & Institutes (GOTHI); Uludag University; American University of Beirut; Ruprecht Karls University Heidelberg; Helmholtz Association; German Cancer Research Center (DKFZ) Ghorbanoghli, Z (corresponding author), Leiden Univ, Med Ctr, Dept Gastroenterol & Hepatol, Albinusdreef 2, NL-2333 ZA Leiden, Netherlands.; Ghorbanoghli, Z (corresponding author), Dutch Hereditary Canc Registry, Leiden, Netherlands.   z.ghorbanoghli@gmail.com   Abu Seir, Rania/AAB-2992-2019; goshayeshi, ladan/AAQ-2715-2020; Loizidou, Maria/C-6607-2009; Ahadova, Aysel/AES-0478-2022; Ghorban Oghli, Zeinab/IAN-2731-2023; Tunca, Berrin/ABI-6078-2020; Ghorbanoghli, Zeinab/A-7348-2017; Al-Mulla, Fahd/E-2068-2015; Hamoudi, Waseem/I-3818-2015 muslumov, gurbankhan/0000-0002-5540-901X; Ghorbanoghli, Zeinab/0000-0001-6311-226X; Majidzadeh-A, Keivan/0000-0002-8811-0997; Abu Seir, Rania/0000-0002-4625-9742; Al-Mulla, Fahd/0000-0001-5409-3829; Sina, Mohammad/0000-0002-2086-5402; Hamoudi, Waseem/0000-0002-7973-3156
16   6   6   4   7   SPRINGER   DORDRECHT   VAN

GODEWIJCKSTRAAT 30, 3311 GZ DORDRECHT, NETHERLANDS 1389-9600
1573-7292 FAM CANCER Fam. Cancer JUL 2021 20 3
215 221 10.1007/s10689-020-
00211-3 http://dx.doi.org/10.1007/s10689-020-00211-3 OCT
2020 7 Oncology; Genetics & Heredity Science Citation
Index Expanded (SCI-EXPANDED) Oncology; Genetics & Heredity
SV0NU 33098072 hybrid, Green Published
2025-06-24 WOS:000583430200001
J Zare, E; Roozbeh, N; Akbari, PA; Teshnizi, SH;
Ghazanfarpour, M; Abdi, F Zare, Elham; Roozbeh,
Nasibeh; Akbari, Pouran Akhavan; Teshnizi, Saeed Hosseini;
Ghazanfarpour, Masumeh; Abdi, Fatemeh HPV and its
high-risk genotypes in Middle Eastern countries: a meta-analysis
FUTURE VIROLOGY English Article
human papillomavirus; Middle East;
prevalence; women HUMAN-PAPILLOMAVIRUS INFECTION; NORMAL
CERVICAL CYTOLOGY; IRANIAN WOMEN; PAP-SMEAR; PREVALENCE;
EPIDEMIOLOGY; VACCINATION; TEHRAN; DNA Background:HPV is the main
cause of cervical cancer. Determining the geographic distribution
of HPV genotypes is a major step in the implementation of cervical
cancer screening. This study aimed to evaluate the prevalence of
HPV and its high-risk genotypes in Middle Eastern
countries.Materials & methods:MEDLINE, ISI Web of Science, PubMed,
EMBASE, Scopus and ProQuest were searched from 2005 to 2018. The
quality of the studies was determined by Strengthening the
Reporting of Observational Studies in Epidemiology checklist.
Fixed/random effects models were conducted to estimate the pooled
prevalence.Results:The pooled prevalence of HPV was 12.3 and 5.2%.
The prevalence of HPV in Africa (22%) was higher than that in
Eurasia (8.3%) and Asia (12.6%). The prevalence of HPV was 14.4,
8.3, 22 and 10.2% in Iran, Turkey, Egypt and Arab countries,
respectively. The prevalence of high-risk-HPV in the above-
mentioned countries was 6.5, 6.2, 6.5 and
3.7%.Conclusion:Prevalence of HPV was increased in the Middle
East. This highlights the need for public education, screening and
vaccination programs. [Zare, Elham] Shahid Beheshti Univ Med Sci,
Sch Nursing & Midwifery, Dept Midwifery & Reprod Hlth, Tehran,
Iran; [Roozbeh, Nasibeh; Teshnizi, Saeed Hosseini] Hormozgan Univ
Med Sci, Mother & Child Welf Res Ctr, Bandar Abbas, Iran; [Akbari,
Pouran Akhavan] Ardabil Univ Med Sci, Nursing & Midwifery Fac,
Dept Midwifery, Ardebil, Iran; [Ghazanfarpour, Masumeh] Kerman
Univ Med Sci, Student Res Comm, Kerman, Iran; [Abdi, Fatemeh]
Alborz Univ Med Sci, Noncommunicable Dis Res Ctr, Karaj, Iran
Shahid Beheshti University Medical Sciences; Ardabil
University of Medical Sciences; Kerman University of Medical
Sciences; Alborz University of Medical Sciences Abdi, F
(corresponding author), Alborz Univ Med Sci, Noncommunicable Dis
Res Ctr, Karaj, Iran. abdi@sbmu.ac.ir Roozbeh, Nasibeh/P-5665-
2017; akbari, pouran/D-4375-2019; Abdi, Fatemeh/KHX-1805-2024;
Ghazanfarpour, Masoumeh/AAA-5941-2021; Zare, Elham/ABF-5164-2021
akhavan Akbari, pouran/0000-0002-5579-7438; Abdi,
Fatemeh/0000-0001-8338-166X Shahid Beheshti University of Medical
Sciences Shahid Beheshti University of Medical Sciences(Shahid
Beheshti University Medical Sciences) This study was funded and
supported by Shahid Beheshti University of Medical Sciences. The
authors have no other relevant affiliations or financial
involvement with any organization or entity with a financial
interest in or financial conflict with the subject matter or

materials discussed in the manuscript apart from those disclosed.
63 2 2 1 7 FUTURE MEDICINE LTD
LONDON UNITEC HOUSE, 3RD FLOOR, 2 ALBERT PLACE, FINCHLEY CENTRAL, LONDON, N3 1QB, ENGLAND 1746-0794 1746-0808
FUTURE VIROL Future Virol. SEP 2020 15 9
595 607 10.2217/fvl-2019-0155
http://dx.doi.org/10.2217/fvl-2019-0155 SEP 2020 13
Virology Science Citation Index Expanded (SCI-EXPANDED)
Virology OI4FC 2025-06-24
WOS:000567250800001
J Natalucci, V; Marini, CF; Lucertini, F; Annibalini, G; Sisti, D; Vallorani, L; Saltarelli, R; Panico, AR; Imperio, M; Flori, M; Busacca, P; Villarini, A; Zeppa, SD; Agostini, D; Monaldi, S; Barocci, S; Catalano, V; Rocchi, MBL; Benelli, P; Stocchi, V; Barbieri, E; Emili, R Natalucci, Valentina; Marini, Carlo Ferri; Lucertini, Francesco; Annibalini, Giosue; Sisti, Davide; Vallorani, Luciana; Saltarelli, Roberta; Panico, Andrea Rocco; Imperio, Marta; Flori, Marco; Busacca, Paolo; Villarini, Anna; Zeppa, Sabrina Donati; Agostini, Deborah; Monaldi, Silvia; Barocci, Simone; Catalano, Vincenzo; Rocchi, Marco Bruno Luigi; Benelli, Piero; Stocchi, Vilberto; Barbieri, Elena; Emili, Rita Effect of a lifestyle intervention program's on breast cancer survivors' cardiometabolic health: Two-year follow-up HELIYON English
Article Breast cancer survivors; Home-based lifestyle intervention; Aerobic exercise; Mediterranean diet; COVID-19PHYSICAL-ACTIVITY; METABOLIC SYNDROME; EXERCISE; PROGNOSIS; OBESITY; GUIDELINES; ADHERENCE; INSULIN; GLUCOSE; RISK The purpose of this study is to assess the cardiometabolic responses of a lifestyle intervention (LI) conducted at home among breast cancer (BC) survivors during the two years of COVID-19 pandemic. A 3-month LI focused on diet and exercise was performed on thirty BC survivors (women; stages 0-II; non-metastatic; aged 53.6 +/- 7.6 years; non-physically active) with a risk factor related to metabolic/endocrine diseases. Anthropometrics, cardiorespiratory fitness (V O2max), physical activity level (PAL), adherence to the Mediterranean diet (MeDiet modified questionnaire), and several biomarkers (i.e., glycemia, insulin, insulin resistance [HOMA-IR] index, triglycerides, high-[HDL] and low-[LDL] density lipoproteins, total cholesterol, proges-terone, testosterone, and hs-troponin) were evaluated before and 3-, 6-, 12-, and 24-month after the LI. Beneficial effects of the LI were observed on several variables (i.e., body mass index, waist circumference, MeDiet, PAL, V O2max, glycemia, insulin, HOMA-IR index, LDL, total cholesterol, triglycerides, testosterone) after 3-month. The significant effect on Mediterranean diet adherence and V O2max persisted up to the 24-month follow-up. Decreases in HOMA-IR index and tri-glycerides were observed up to 12-month, however did not persist afterward. This study provides evidence on the positive association between LI and cardiometabolic health in BC survivors. [Natalucci, Valentina; Marini, Carlo Ferri; Lucertini, Francesco; Annibalini, Giosue; Sisti, Davide; Vallorani, Luciana; Saltarelli, Roberta; Panico, Andrea Rocco; Imperio, Marta; Zeppa, Sabrina Donati; Agostini, Deborah; Rocchi, Marco Bruno Luigi; Benelli, Piero; Barbieri, Elena] Univ Urbino Carlo Bo, Dept Biomol Sci, I-61029 Urbino, Italy; [Flori, Marco; Busacca, Paolo] Osped Santa Maria Misericordia, UOC Cardiol, UTIC, AST, I-61029 Urbino, Italy;

[Villarini, Anna] Univ Perugia, Dept Med & Surg, Piazzale Settimio Gambuli, I-06132 Perugia, Italy; [Monaldi, Silvia; Catalano, Vincenzo; Emili, Rita] Osped Santa Maria Misericordia, UOC Oncol Med, I-61029 Urbino, Italy; [Barocci, Simone] Osped Santa Maria Misericordia, UOC Patol Clin, AST, I-61029 Urbino, Italy; [Stocchi, Vilberto] Univ San Raffaele, Dept Human Sci Promot Qual Life, I-20132 Rome, Italy University of Urbino; Hospital Santa Maria della Misericordia; University of Perugia; Hospital Santa Maria della Misericordia; Hospital Santa Maria della Misericordia; Universita Telematica San Raffaele Imperio, M; Barbieri, E (corresponding author), Univ Urbino Carlo Bo, Dept Biomol Sci, I-61029 Urbino, Italy. m.imperio@campus.uniurb.it; elena.barbieri@uniurb.it Natalucci, Valentina/AAM-7882-2020; Donati Zeppa, Sabrina/AAO-9088-2020; SALTARELLI, Roberta/AAM-4291-2021; Imperio, Marta/MGT-5131-2025; Rocchi, Marco/AAK-5926-2021; Lucertini, Francesco/F-6603-2012; Barbieri, Elena/AGI-0430-2022; Ferri Marini, Carlo/GRY-3765-2022; Annibalini, Giosue/J-7701-2016 Lucertini, Francesco/0000-0003-3134-4511; Barbieri, Elena/0000-0002-3480-7983; SALTARELLI, Roberta/0000-0002-6375-127X; Ferri Marini, Carlo/0000-0002-6866-997X; Annibalini, Giosue/0000-0002-6914-4905; Natalucci, Valentina/0000-0002-0582-1575; Stocchi, Vilberto/0000-0003-3269-9410 Athenaeum of Urbino entitled: Promozione della salute e della sicurezza alimentare [226/2021] Athenaeum of Urbino entitled: Promozione della salute e della sicurezza alimentareThis research was supported in part by a grant from the Athenaeum of Urbino entitled: Promozione della salute e della sicurezza alimentare (DR. n. 226/2021 19/05/2021) . The funders had no role in study design, data collection and analysis, decision to publish, or preparation of the manuscript.
70 3 3 0 1 CELL PRESS CAMBRIDGE 50 HAMPSHIRE ST, FLOOR 5, CAMBRIDGE, MA 02139 USA 2405-8440
HELIYON Heliyon NOV 2023 9 11
e21761
10.1016/j.heliyon.2023.e21761
http://dx.doi.org/10.1016/j.heliyon.2023.e21761 NOV 2023 11 Multidisciplinary Sciences Science Citation Index Expanded (SCI-EXPANDED) Science & Technology - Other Topics
Y4XR1 38027927 Green Published, gold 2025-06-24 WOS:001105310300001

J Romanos-Nanclares, A; Guasch-Ferré, M; Willett, WC; Chen, WY; Holmes, MD; Rosner, BA; Martinez-Gonzalez, MA; Eliassen, AH
Romanos-Nanclares, Andrea; Guasch-Ferre, Marta; Willett, Walter C.; Chen, Wendy Y.; Holmes, Michelle D.; Rosner, Bernard A.; Martinez-Gonzalez, Miguel A.; Eliassen, A. Heather Consumption of olive oil and risk of breast cancer in US women: results from the Nurses' Health Studies
BRITISH JOURNAL OF CANCER English Article
MEDITERRANEAN DIET; CARDIOVASCULAR RISK; FATTY-ACIDS; METAANALYSIS BackgroundOlive oil consumption may reduce breast cancer risk, but it is unclear whether olive oil is beneficial for breast cancer prevention in populations outside of Mediterranean regions, namely in the U.S., where the average consumption of olive oil is low compared with Mediterranean populations. We examined whether olive oil intake was associated with breast cancer risk in two prospective cohorts of U.S. women.MethodsWe used multivariable-adjusted time-varying Cox proportional hazards models to estimate hazard ratios (HR) and 95% confidence interval (CI) for breast cancer among 71,330

(Nurses' Health Study, 1990-2016) and 93,295 women (Nurses' Health Study II, 1991-2017) who were free of cancer at baseline. Diet was assessed by a validated semi-quantitative food frequency questionnaire every 4 years.ResultsDuring 3,744,068 person-years of follow-up, 9,638 women developed invasive breast cancer. The multivariable-adjusted HR (95% CI) for breast cancer among women who had the highest consumption of olive oil (>1/2 tablespoon/d or >7 g/d) compared with those who never or rarely consumed olive oil, was 1.01 (0.93, 1.09). Higher olive oil consumption was not associated with any subtype of breast cancer.ConclusionWe did not observe an association between higher olive oil intake and breast cancer risk in two large prospective cohorts of U.S. women, whose average olive oil consumption was low. Prospective studies are needed to confirm these findings and to further investigate whether different varieties of olive oil (e.g., virgin and extra virgin olive oil) may play a role in breast cancer risk.[Romanos-Nanclares, Andrea; Guasch-Ferre, Marta; Chen, Wendy Y.; Holmes, Michelle D.; Rosner, Bernard A.; Eliassen, A. Heather] Brigham & Womens Hosp, Dept Med, Channing Div Network Med, Boston, MA 02115 USA; [Romanos-Nanclares, Andrea; Guasch-Ferre, Marta; Chen, Wendy Y.; Holmes, Michelle D.; Rosner, Bernard A.; Eliassen, A. Heather] Harvard Med Sch, Boston, MA 02115 USA; [Guasch-Ferre, Marta; Willett, Walter C.; Martinez-Gonzalez, Miguel A.; Eliassen, A. Heather] Harvard TH Chan Sch Publ Hlth, Dept Nutr, Boston, MA USA; [Willett, Walter C.; Holmes, Michelle D.; Eliassen, A. Heather] Harvard TH Chan Sch Publ Hlth, Dept Epidemiol, Boston, MA USA; [Chen, Wendy Y.] Dana Farber Canc Inst, Dept Med Oncol, Boston, MA USA; [Rosner, Bernard A.] Harvard TH Chan Sch Publ Hlth, Dept Biostat, Boston, MA USA; [Martinez-Gonzalez, Miguel A.] Carlos III Hlth Inst, Biomed Res Network Ctr Pathophysiol Obes & Nutr C, Madrid, Spain; [Martinez-Gonzalez, Miguel A.] Univ Navarra, Dept Prevent Med & Publ Hlth, Pamplona, Spain   Harvard University; Harvard University Medical Affiliates; Brigham & Women's Hospital; Harvard University; Harvard Medical School; Harvard University; Harvard T.H. Chan School of Public Health; Harvard University; Harvard T.H. Chan School of Public Health; Harvard University; Harvard University Medical Affiliates; Dana-Farber Cancer Institute; Harvard University; Harvard T.H. Chan School of Public Health; University of Navarra   Romanos-Nanclares, A (corresponding author), Brigham & Womens Hosp, Dept Med, Channing Div Network Med, Boston, MA 02115 USA.; Romanos-Nanclares, A (corresponding author), Harvard Med Sch, Boston, MA 02115 USA.

nharo@channing.harvard.edu Holmes, Michelle/GLS-8692-2022; Guasch-Ferre, Marta/JJM-4619-2023; Martinez-Gonzalez, Miguel/AAE-7669-2019; Willett, Walter/E-2352-2013 Eliassen, A Heather/0000-0002-3961-6609; Willett, Walter/0000-0003-1458-7597; Romanos-Nanclares, Andrea/0000-0002-9694-7607; Guasch-Ferre, Marta/0000-0001-8525-1404   National Institutes of Health [UM1 CA186107, U01 CA176726, P01 CA87969, R01 CA50385]; Breast Cancer Research Foundation; Susan G Komen Foundation; Ramon Areces Foundation

National Institutes of Health(United States Department of Health & Human ServicesNational Institutes of Health (NIH) - USA); Breast Cancer Research Foundation; Susan G Komen Foundation(Susan G. Komen Breast Cancer Foundation); Ramon Areces Foundation   This study was supported by grants UM1 CA186107, U01 CA176726, P01 CA87969,and R01 CA50385 from the National Institutes of Health, the Breast Cancer Research Foundation and Susan G Komen Foundation and Ramon Areces Foundation. The funding sources did not

participate in the design or conduct of the study; collection, management, analysis, or interpretation of the data; or preparation, review, or approval of the manuscript 27 6 6 0 7 SPRINGERNATURE LONDON CAMPUS, 4 CRINAN ST, LONDON, N1 9XW, ENGLAND 0007-0920 1532-1827 BRIT J CANCER Br. J. Cancer AUG 24 2023 129 3 416 425 10.1038/s41416-023-02306-x http://dx.doi.org/10.1038/s41416-023-02306-x JUN 2023 10 Oncology Science Citation Index Expanded (SCI-EXPANDED) Oncology N8WK4 37311975 Green Published 2025-06-24 WOS:001008639400003

J Sánchez-Quesada, C; Gutiérrez-Santiago, F; Rodríguez-García, C; Gaforio, JJ Sanchez-Quesada, Cristina; Gutierrez-Santiago, Francisco; Rodriguez-Garcia, Carmen; Gaforio, Jose J. Synergistic Effect of Squalene and Hydroxytyrosol on Highly Invasive MDA-MB-231 Breast Cancer Cells NUTRIENTS English Article virgin olive oils; breast cancer; antitumor; proliferation; apoptosis; DNA damage; comet assay VIRGIN OLIVE OIL; OXIDATIVE DNA-DAMAGE; MEDITERRANEAN DIET; ANTIOXIDANT; PROLIFERATION; POTENTIATION; INHIBITION; PREVENTION; OLEUROPEIN; PROTECTS Several studies relate Mediterranean diet and virgin olive oil (VOO) intake with lower risk of several chronic diseases, including breast cancer. Many of them described antitumor properties of isolated minor compounds present in VOO, but beneficial properties of VOO arise from the effects of all its compounds acting together. The aim of the present study was to test the antitumor effects of two minor compounds from VOO (hydroxytyrosol (HT) and squalene (SQ)) on highly metastatic human breast tumor cells (MDA-MB-231) when acting in combination. Both isolated compounds were previously analyzed without showing any antitumoral effect on highly invasive MDA-MB-231 breast cancer cells, but the present results show that HT at 100 mu M, combined with different concentrations of SQ, could exert antitumor effects. When they are combined, HT and SQ are able to inhibit cell proliferation, promoting apoptosis and DNA damage in metastatic breast cancer cells. Therefore, our results suggest that the health-promoting properties of VOO may be due, at least in part, to the combined action of these two minor compounds.
[Sanchez-Quesada, Cristina; Gutierrez-Santiago, Francisco; Rodriguez-Garcia, Carmen; Gaforio, Jose J.] Univ Jaen, Fac Expt Sci, Dept Hlth Sci, Jaen 23071, Spain; [Sanchez-Quesada, Cristina; Rodriguez-Garcia, Carmen; Gaforio, Jose J.] Univ Jaen, Univ Inst Res Olive Groves & Olive Oils, Campus Lagunillas S-N, Jaen 23071, Spain; [Sanchez-Quesada, Cristina; Gaforio, Jose J.] Agrifood Campus Int Excellence CeiA3, Cordoba 14071, Spain; [Gaforio, Jose J.] Inst Salud Carlos III, CIBER Epidemiol & Salud Publ CIBER ESP, Madrid 28029, Spain Universidad de Jaen; Universidad de Jaen; CIBER - Centro de Investigacion Biomedica en Red; CIBERESP; Instituto de Salud Carlos III Gaforio, JJ (corresponding author), Univ Jaen, Fac Expt Sci, Dept Hlth Sci, Jaen 23071, Spain.; Gaforio, JJ (corresponding author), Univ Jaen, Univ Inst Res Olive Groves & Olive Oils, Campus Lagunillas S-N, Jaen 23071, Spain.; Gaforio, JJ (corresponding author), Agrifood Campus Int Excellence CeiA3, Cordoba 14071, Spain.; Gaforio, JJ (corresponding author), Inst Salud Carlos III, CIBER Epidemiol & Salud Publ CIBER ESP, Madrid 28029, Spain. csquesad@ujaen.es; fgutierr@ujaen.es; crgarcia@ujaen.es; jgaforio@ujaen.es Rodriguez

Garcia, Carmen/ACH-8755-2022; Sanchez, Cristina/ABC-5511-2021
Sanchez-Quesada, Cristina/0000-0003-2997-8515; Gutierrez, Francisco/0000-0002-4564-9271; Gaforio, Jose J/0000-0003-2996-9301; Rodriguez Garcia, Carmen/0000-0002-4531-3188 "Junta de Andalucia" (Proyecto de Excelencia) [PI10-AGR-6724] "Junta de Andalucia" (Proyecto de Excelencia)(Junta de Andalucia) This study was financially supported by the "Junta de Andalucia" (Proyecto de Excelencia PI10-AGR-6724). 45 13 13 1
4 MDPI BASEL MDPI AG, Grosspeteranlage 5, CH-4052 BASEL, SWITZERLAND 2072-6643 NUTRIENTS Nutrients JAN 2022
14 2 255
10.3390/nu14020255 http://dx.doi.org/10.3390/nu14020255
14 Nutrition & Dietetics Science Citation Index Expanded (SCI-EXPANDED) Nutrition & Dietetics 2T7WN
35057436 gold, Green Published 2025-06-24
WOS:000822680400001
J La Foresta, D; Buonocore, MN La Foresta, Daniela; Buonocore, Maria Nicola Mediterranean migrations: NGO as the voice of the voiceless?The role of NGOs in communicating Mediterranean migration effectively: challenges and opportunities CIVIL SZEMLE English Article
Migration flows; Mediterranean Sea; Search and Rescue Operations/(SAR operations); NGOs' strategies RESCUE; SEA The Mediterranean Sea has always represented a unique melting pot of cultures and peoples, holding a strategic position: since the mid-1990s it has been the preferred route for migrants to reach the European continent. Adverse weather conditions overcrowded and illegal routes, as well as the difficult management of the emergency by the EU, make the Mediterranean one of the most dangerous routes, with reports of migrant accidents being an everyday occurrence. Since 2014, approximately 29.000 migrants have gone missing or died in the Mediterranean, with the highest number of fatalities recorded in 2016 and the central route (UNHCR, 2022). The complexity of the situation indicates how it cannot be solved solely by the countries most affected by the phenomenon: indeed, cooperation and coordination with states, NGOs, and other maritime agencies are nec-essary. NGOs play a central role in the management of the situation, with the Council of Europe calling on states to cooperate constructively with NGOs to ensure the effective protection of human rights. Approximately 38 NGO ships conducted SAR operations in the Mediterranean Sea between 2016 and 2022 (FRA). This research aims to analyze the online communication of the pre-eminent NGOs that operate in the Mediterranean Sea. Greater importance is attributed to communication strategies: through them, organiza-tions can spread their values and promote their actions, while raising public awareness. Thus, the focus is on the core online communication tactics used to achieve their goals. [La Foresta, Daniela] Univ Naples Federico II, Dept Polit Sci, Naples, Italy; [Buonocore, Maria Nicola] Comenius Univ, Fac Social & Econ Sci, Bratislava, Slovakia University of Naples Federico II; Comenius University Bratislava La Foresta, D (corresponding author), Univ Naples Federico II, Dept Polit Sci, Naples, Italy. laforest@unina.it; buonocore1@uniba.sk
38 0 0 3 4 UJ MANDATUM KONYVKIADO BUDAPEST FRATER GYORGY TER 11, BUDAPEST, 1149, HUNGARY 1786-3341 CIV SZLE Civ. Szle.
2024 21 2

10.62560/csz.2024.02.08
http://dx.doi.org/10.62560/csz.2024.02.08 207
Public Administration Social Science Citation Index (SSCI)
Public Administration ZJ4L9 2025-06-
24 WOS:001274914100008
J Ganjavi, M; Faraji, B Ganjavi, Maryam;
Faraji, Bahram Late effect of the food consumption on
colorectal cancer rate INTERNATIONAL JOURNAL OF FOOD SCIENCES AND
NUTRITION English Article
Colorectal cancer; incidence; risk factor; food
availability DIETARY PATTERNS; MEAT CONSUMPTION; MEDITERRANEAN
DIET; RED MEAT; RISK; METAANALYSIS; ASSOCIATION; MORTALITY;
ADENOMAS; POULTRY Studies have suggested that higher meat
intake may increase colorectal cancer (CRC) risk while higher
vegetable intake may reduce this risk. There is a substantial lag
between the time of exposure to a risk factor (or protective
factor) and incidence of cancer. For CRC, in particular, the time
from formation of adenoma to occurrence of CRC takes from 10 to 15
years, or even more. This study correlates food disappearance data
per capita for vegetable and meat with future age-adjusted CRC
rates in USA. The lag weights, with a high confidence, showed that
there is a positive correlation between the red meat availability
and CRC age-adjusted incidence rates with a lag of at least 17
years and an Almon polynomial degree of 2. Conversely, there was a
negative correlation between vegetables availability and future
age-adjusted incidence rates of CRC. [Ganjavi, Maryam; Faraji,
Bahram] Morgan State Univ, Dept Publ Hlth, Nutr Sci Program,
Baltimore, MD 21239 USA Morgan State University Ganjavi,
M (corresponding author), Morgan State Univ, Dept Publ Hlth, Nutr
Sci Program, Baltimore, MD 21239 USA. maryam.ganjavi@morgan.edu
NIGMS/NIH [UL1 GM118973] NIGMS/NIH(United
States Department of Health & Human ServicesNational Institutes of
Health (NIH) - USANIH National Institute of General Medical
Sciences (NIGMS)) This work was supported in part by funding
from NIGMS/NIH, grant number [UL1 GM118973]. 50 4 4
2 17 TAYLOR & FRANCIS LTD ABINGDON 2-4 PARK
SQUARE, MILTON PARK, ABINGDON OR14 4RN, OXON, ENGLAND 0963-7486
1465-3478 INT J FOOD SCI NUTR Int. J. Food Sci.
Nutr. JAN 2 2019 70 1 98 106
10.1080/09637486.2018.1472747
http://dx.doi.org/10.1080/09637486.2018.1472747
9 Food Science & Technology; Nutrition & Dietetics
Science Citation Index Expanded (SCI-EXPANDED) Food
Science & Technology; Nutrition & Dietetics HF8RR 29768948
Green Accepted 2025-06-24 WOS:000454510400010
J Lopez-Ozuna, VM; Gupta, I; Kiow, RLC; Matanes, E; Yasmeen,
A; Vranic, S; Al Moustafa, AE Lopez-Ozuna,
Vanessa M.; Gupta, Ishita; Chen Kiow, Ryan Liu; Matanes, Emad;
Yasmeen, Amber; Vranic, Semir; Al Moustafa, Ala-Eddin
Exposure to water-pipe smoking dysregulates a set of genes
associated with breast cancer development and an unfavorable
outcome CLINICAL CANCER INVESTIGATION JOURNAL
English Article Breast
cancer; gene deregulation; mammary epithelial cells; smoking;
water-pipe EPITHELIAL-MESENCHYMAL TRANSITION; DIFFERENTIALLY
EXPRESSED GENES; CIGARETTE-SMOKING; TOBACCO SMOKING; POOR-
PROGNOSIS; OVARIAN-CANCER; STEM-CELLS; ACTIVATION; CHEMOKINE;
INVASION Background: Water-pipe smoking (WPS), a predominant

method of tobacco consumption, is common amongst young females in the Middle East. WPS smoke consists of toxins analogous to the ones that exist in cigarette smoke and frequently correlates with the onset of several types of human cancers including breast. However, the potential target genes and their underlying mechanisms in the initiation and/ or progression of human cancers, especially breast, due to WPS exposure are still unknown. Materials and Methods: In this investigation, we explored the effect of WPS chronic exposure on human normal mammary epithelial cells and analyzed alterations in the differentially ex-pressed gene (DEG) targets using the NanoString nCounter PanCancer Pathways Panel consisting of 770 gene transcripts and a quantitative real-time polymerase chain reaction (PCR) analysis. Results: Our NanoString analysis identified 13 genes dysregulated under the effect of WPS exposure involved in regulating signal transduction, cell cycle, cell motility, proliferation and migration/invasion as well as the inflammatory response. We further performed an in silico analysis to investigate the effect of the identified genes in the prognosis of breast cancer patients and reported those DEGs that directly correlated with smoking and were upregulated in breast cancer in comparison with normal tissue. Moreover, the Kaplan-Meier curve analysis showed a significant correlation be-tween WPS-dysregulated genes (MX1, CCL8, GNGT1 and MMP9) and relapse-free survival in breast cancer patients. Conclusions: Our data clearly suggest that exposure to WPS can alter the expression of key regulator genes involved in the pathogenesis of breast cancer, thereby affecting the breast cancer prognosis. [Lopez-Ozuna, Vanessa M.; Chen Kiow, Ryan Liu; Matanes, Emad; Yasmeen, Amber] McGill Univ, JGH, Lady Davis Inst Med Res, Segal Canc Ctr, Montreal, PQ, Canada; [Gupta, Ishita; Vranic, Semir; Al Moustafa, Ala-Eddin] Qatar Univ, Dept Basic Med Sci, Coll Med, QU Hlth,Coll Med, POB 2713, Doha, Qatar; [Gupta, Ishita; Al Moustafa, Ala-Eddin] Qatar Univ, Biomed & Pharmaceut Res Unit, QU Hlth, POB 2713, Doha, Qatar Lady Davis Institute; McGill University; Qatar University; Qatar University Gupta, I (corresponding author), Qatar Univ, Biomed & Pharmaceut Res Unit, QU Hlth, POB 2713, Doha, Qatar.
ishita.gupta@qu.edu.qa Vranic, Semir/J-4113-2012 Vranic, Semir/0000-0001-9743-7265 Qatar University [QUCG-CMED-20/21-2, QUHI-CMED-19/20-1] Qatar University(Qatar UniversityQatar National Research Fund (QNRF)) This work is supported by Qatar University, grant numbers: QUCG-CMED-20/21-2 and QUHI-CMED-19/20-1. The funders had no role in the design of the study; in the collection, analyses or interpretation of data; in the writing of the manuscript or in the decision to publish the results.
106 0 0 0 2 Middle Eastern Assoc Cancer Research Montreal 3240 Ave Lacombe, Montreal, Quebec, CANADA
2278-0513 CLIN CANCER INVESTIG Clin. Cancer Investig. J. NOV-DEC 2021 10 6
318 + 10.4103/ccij.ccij_109_21
http://dx.doi.org/10.4103/ccij.ccij_109_21 15
Oncology Emerging Sources Citation Index (ESCI) Oncology
XS2OW 2025-06-24 WOS:000732755600009
J Zhang, XX; Zeng, QL; Cai, WW; Ruan, WQ
Zhang, Xingxing; Zeng, Qingle; Cai, Wenwen; Ruan, Weiqing
Trends of cervical cancer at global, regional, and national level: data from the Global Burden of Disease study 2019 BMC PUBLIC HEALTH English Article

Cervical cancer; Global burden of disease; Quality-adjusted life years; Global Health; Health services HUMAN-PAPILLOMAVIRUS; MIDDLE-EAST; WOMEN; PREVENTION; STATISTICS; MORTALITY; PATTERNS; VACCINE; IMPACT; RISK

Background Cervical cancer is an important global health problem. In this study we aimed to analyze trends in cervical cancer at the global, regional, and national levels from 1990 to 2019, to inform health service decision-making. Methods Data on cervical cancer was extracted from the Global Burden of Disease study, 2019. Trends in cervical cancer burden were assessed based on estimated annual percentage change (EAPC) and age-standardized rate (ASR). Results Globally, decreasing trends were observed in incidence, death, and disability adjusted life years (DALYs) of cervical cancer from 1990 to 2019, with respective EAPCs of - 0.38 (95% confidence interval [CI]: - 0.41 to - 0.34), - 0.93 (95%CI: - 0.98 to - 0.88), and - 0.95 (95 CI%: - 1.00 to - 0.90). Meanwhile, decreasing trends were detected in most sociodemographic index (SDI) areas and geographic regions, particularly death and DALYs in Central Latin America, with respective EAPCs of - 2.61 (95% CI: - 2.76 to - 2.46) and - 2.48 (95% CI: - 2.63 to - 2.32); hhowever, a pronounced increasing trend in incidence occurred in East Asia (EAPC = 1.33; 95% CI: 1.12 to 1.55). At the national level, decreasing trends in cervical cancer were observed in most countries/territories, particularly DALYs in the Maldives (EAPC = - 5.06; 95% CI: - 5.40 to - 4.72), Whereas increasing trends were detected in Lesotho, Zimbabwe, and Bulgaria. Conclusions Slowly decreasing trends in cervical cancer were detected worldwide from 1990 to 2019. Cervical cancer remains a substantial health problem for women globally, requiring more effective prevention and control strategies. [Zhang, Xingxing; Cai, Wenwen; Ruan, Weiqing] Southern Med Univ, Nanfang Hosp, Huiqiao Med Ctr, Guangzhou, Guangdong, Peoples R China; [Zeng, Qingle] Southern Med Univ, Nanfang Hosp, Dept Intervent Radiol, Guangzhou, Peoples R China Southern Medical University - China; Southern Medical University - China Ruan, WQ (corresponding author), Southern Med Univ, Nanfang Hosp, Huiqiao Med Ctr, Guangzhou, Guangdong, Peoples R China. jamela@sina.com Zeng, Qingle/S-4308-2018

Guangdong Technology and Science Planning Fund [2020A1414040014] Guangdong Technology and Science Planning Fund This work was supported by the Guangdong Technology and Science Planning Fund (NO: 2020A1414040014). 34 106 109
3 54 BMC LONDON CAMPUS, 4 CRINAN ST, LONDON N1 9XW, ENGLAND 1471-2458 BMC PUBLIC HEALTH BMC Public Health MAY 12 2021 21 1
894 10.1186/s12889-021-10907-5
http://dx.doi.org/10.1186/s12889-021-10907-5 10
Public, Environmental & Occupational Health Science Citation Index Expanded (SCI-EXPANDED) Public, Environmental & Occupational Health SK4YP 33975583 gold, Green Published Y
N 2025-06-24 WOS:000656223200002

J Castelló, A; Rodríguez-Barranco, M; Lope, V; Guevara, M; Colorado-Yohar, S; Dorronsoro, A; Quirós, JR; Castro-Espin, C; Sayon-Orea, C; Santiuste, C; Amiano, P; Lasheras, C; Sanchez, MJ; Pollán, M Castello, Adela; Rodriguez-Barranco, Miguel; Lope, Virginia; Guevara, Marcela; Colorado-Yohar, Sandra; Dorronsoro, Ane; Quiros, Jose Ramon; Castro-Espin, Carlota; Sayon-Orea, Carmen; Santiuste, Carmen; Amiano, Pilar; Lasheras, Cristina; Sanchez, Maria-Jose; Pollan, Marina High

adherence to Western dietary pattern increases breast cancer risk (an EPIC-Spain study) MATURITAS English Article Dietary patterns; Diet; Western; Mediterranean; Breast neoplasms WOMEN; MENOPAUSE; INFLAMMATION; ASSOCIATION; PRUDENT; MODELS; AGE Objective: To explore the association between three previously identified and validated dietary patterns (Western, Prudent and Mediterranean) and breast cancer risk by tumour subtype and menopausal status.Methods: Data from the Spanish cohort of the European Prospective Investigation into Cancer and Nutrition study provided epidemiological information (including diet and cancer incidence) from 24,892 women (639 breast cancer cases) recruited between 1992 and 1996. The associations between adherence to the three dietary pat-terns and breast cancer risk (overall and by tumour subtype) were explored by fitting multivariate Cox proportional hazards regression models stratified by region, among other variables. A possible interaction with menopausal status (changing over time) was explored.Results: No clear association of the Prudent and Mediterranean dietary patterns with breast cancer risk was found. When compared with women with a level of adherence to the Western diet in the first quartile, women with a level of adherence in the third (hazard ratio (95 % confidence interval) (HR(95%CI)):1.37 (1.07;1.77)) and fourth quartiles (1.37 (1.03;1.83)); p for curvature of splines = 0.016) showed a non-linear increased risk, especially postmenopausal women (HR (95 % CI) 1.30 (0.98;1.72) in the third and 1.42 (1.04;1.94) in the fourth quartiles; p for curvature of splines = 0.081) and for estrogen or progesterone receptor positive with human epidermal growth factor receptor 2 negative tumours (HR (95 % CI) 1.62 (1.10;2.38) and 1.71 (1.11;2.63) for the third and fourth quartiles respectively; p for curvature of splines = 0.013).Conclusions: Intake of foods such as high-fat dairy products, red and processed meats, refined grains, sweets, caloric drinks, convenience food and sauces might be associated with a higher risk of breast cancer. [Castello, Adela; Lope, Virginia; Pollan, Marina] Carlos III Inst Hlth, Natl Ctr Epidemiol, Canc & Environm Epidemiol Unit, Madrid, Spain; [Castello, Adela; Rodriguez-Barranco, Miguel; Lope, Virginia; Guevara, Marcela; Colorado-Yohar, Sandra; Santiuste, Carmen; Amiano, Pilar; Sanchez, Maria-Jose; Pollan, Marina] Consortium Biomed Res Epidemiol & Publ Hlth CIBER, Madrid, Spain; [Rodriguez-Barranco, Miguel; Sanchez, Maria-Jose] Escuela Andaluza Salud Publ, Granada 18011, Spain; [Rodriguez-Barranco, Miguel; Sanchez, Maria-Jose] Inst Invest Biosanitaria Ibs GRANADA, Granada 18012, Spain; [Guevara, Marcela; Sayon-Orea, Carmen] Inst Salud Publ Navarra, Pamplona 31003, Spain; [Guevara, Marcela; Sayon-Orea, Carmen] Navarra Inst Hlth Res IdiSNA, Pamplona 31008, Spain; [Colorado-Yohar, Sandra; Santiuste, Carmen] Murcia Reg Hlth Council, Dept Epidemiol, IMIB, Murcia, Spain; [Colorado-Yohar, Sandra] Univ Antioquia, Natl Fac Publ Hlth, Res Grp Demog & Hlth, Medellin, Colombia; [Dorronsoro, Ane; Amiano, Pilar] Minist Hlth, Basque Govt, Sub Directorate Publ Hlth & Addict Gipuzkoa, San Sebastian 20013, Spain; [Dorronsoro, Ane; Amiano, Pilar] Biodonostia Hlth Res Inst, Epidemiol Chron & Communicable Dis Grp, San Sebastian 20014, Spain; [Quiros, Jose Ramon] Publ Hlth Directorate, Asturias, Spain; [Castro-Espin, Carlota] Catalan Inst Oncol ICO, Unit Nutr & Canc, Barcelona 08908, Spain; [Castro-Espin, Carlota] Bellvitge Biomed Res Inst IDIBELL, Epidemiol Publ Hlth Canc Prevent & Palliat Care Pr, Nutr & Canc Grp, Barcelona

08908, Spain; [Sayon-Orea, Carmen] Inst Salud Carlos III, Ctr Invest Biomed Red Fisiopatol Obesidad & Nutr C, Madrid, Spain; [Sayon-Orea, Carmen] Univ Navarra, Dept Prevent Med & Publ Hlth, Pamplona, Spain; [Lasheras, Cristina] Univ Oviedo, Funct Biol Dept, Oviedo 33006, Spain; [Sanchez, Maria-Jose] Univ Granada, Dept Prevent Med & Publ Hlth, Granada 18071, Spain; [Castello, Adela] Inst Salud Carlos III, Av Monforte Lemos 5, Madrid 28029, Spain; [Rodriguez-Barranco, Miguel] Escuela Andaluza Salud Publ, Cuesta Observ 4, Granada 18011, Spain Instituto de Salud Carlos III; CIBER - Centro de Investigacion Biomedica en Red; CIBERESP; Escuela Andaluza de Salud Publica; Instituto de Investigacion Biosanitaria IBS Granada; Public Health Institute of Navarra; University of Navarra; Murcia Regional Health Council; Universidad de Antioquia; Basque Government; Instituto de Investigacion Sanitaria Biogipuzkoa; Institut Catala d'Oncologia; Institut d'Investigacio Biomedica de Bellvitge (IDIBELL); Instituto de Salud Carlos III; University of Navarra; University of Oviedo; University of Granada; Instituto de Salud Carlos III; Escuela Andaluza de Salud Publica   Castelló, A (corresponding author), Inst Salud Carlos III, Av Monforte Lemos 5, Madrid 28029, Spain.; Rodríguez-Barranco, M (corresponding author), Escuela Andaluza Salud Publ, Cuesta Observ 4, Granada 18011, Spain.
acastello@isciii.es; miguel.rodriguez.barranco.easp@juntadeandalucia.es; vicarvajal@isciii.es; mp.guevara.eslava@navarra.es; a-dorronsoroerauskin@euskadi.eus; ccastro@idibell.cat; mc.sayon.orea@navarra.es; mcarmen.santiuste@carm.es; p-amiano@euskadi.eus; lasheras@uniovi.es; mariajose.sanchez.easp@juntadeandalucia.es   Martín-Sánchez, Marta/E-5831-2018; Castelló, Adela/C-3829-2014; LOPE, VIRGINIA/S-4774-2016; M, RODRIGUEZ-BARRANCO/H-4541-2012; Colorado-Yohar, Sandra/AAR-8824-2020; Guevara, Marcela/B-4473-2015; Sayon-Orea, Carmen/A-9828-2017   Colorado-Yohar, Sandra/0000-0002-6700-0780; Guevara, Marcela/0000-0001-9242-6364; Sayon-Orea, Carmen/0000-0002-4137-3263   Autonomous Community of Madrid [CM/JIN/2019-041]; Department of Epidemiology and Biostatistics, School of Public Health, Imperial College, London; Health Research Fund (FIS) - Instituto de Salud Carlos III (ISCIII); Alcala de Henares; International Agency for Research on Cancer (IARC); Regional Government of Andalucia; Regional Government of Asturias; Regional Government of Basque Country; Regional Government of Murcia; Regional Government of Navarra; Catalan Institute of Oncology-ICO (Spain)   Autonomous Community of Madrid; Department of Epidemiology and Biostatistics, School of Public Health, Imperial College, London; Health Research Fund (FIS) -Instituto de Salud Carlos III (ISCIII); Alcala de Henares; International Agency for Research on Cancer (IARC)(World Health Organization); Regional Government of Andalucia(Junta de Andalucia); Regional Government of Asturias(Principality of Asturias); Regional Government of Basque Country(Basque Government); Regional Government of Murcia; Regional Government of Navarra; Catalan Institute of Oncology-ICO (Spain)   This study was supported by Alcala de Henares and Autonomous Community of Madrid (CM/JIN/2019-041) . The coordination of EPIC is financially supported by International Agency for Research on Cancer (IARC) and also by the Department of Epidemiology and Biostatistics, School of Public Health, Imperial College, London, which has additional infrastructure support provided by the NIHR Imperial Biomedical Research Centre (BRC)

.The EPIC-Spain cohort is supported by the Health Research Fund (FIS) -Instituto de Salud Carlos III (ISCIII) , the Regional Governments of Andalucia, Asturias, Basque Country, Murcia and Navarra, and the Catalan Institute of Oncology-ICO (Spain) .
63 6 6 2 6 ELSEVIER IRELAND LTD CLARE
ELSEVIER HOUSE, BROOKVALE PLAZA, EAST PARK SHANNON, CO, CLARE, 00000, IRELAND 0378-5122 1873-4111 MATURITAS
Maturitas JAN 2024 179
107868 10.1016/j.maturitas.2023.107868
http://dx.doi.org/10.1016/j.maturitas.2023.107868 NOV 2023 14 Geriatrics & Gerontology; Obstetrics & Gynecology
Science Citation Index Expanded (SCI-EXPANDED)
Geriatrics & Gerontology; Obstetrics & Gynecology Y9IR0
37925868 2025-06-24 WOS:001108327800001
J Ergas, IJ; Cheng, RK; Roh, JM; Kresovich, JK; Iribarren, C; Nguyen-Huynh, M; Rana, JS; Rillamas-Sun, E; Laurent, CA; Lee, VS; Quesenberry, CP; Bhatt, A; Yao, S; Kushi, LH; Greenlee, H; Kwan, ML Ergas, Isaac J.; Cheng, Richard K.; Roh, Janise M.; Kresovich, Jacob K.; Iribarren, Carlos; Nguyen-Huynh, Mai; Rana, Jamal S.; Rillamas-Sun, Eileen; Laurent, Cecile A.; Lee, Valerie S.; Quesenberry, Charles P.; Bhatt, Ankeet; Yao, Song; Kushi, Lawrence H.; Greenlee, Heather; Kwan, Marilyn L.
Diet quality and cardiometabolic health in breast cancer survivors: the Pathways Study BREAST CANCER RESEARCH AND TREATMENT English Article
Breast cancer; Diet quality; Hypertension; Diabetes; Dyslipidemia CORONARY-HEART-DISEASE; FAT VEGAN DIET; SCIENTIFIC STATEMENT; COMPETING RISKS; BLOOD-PRESSURE; PREVENTION; MORTALITY; ASSOCIATION; MANAGEMENT; ADHERENCE Purpose Breast cancer (BC) survivors experience higher rates of cardiometabolic conditions, partly due to treatment. While healthy eating decreases the risk of these conditions in the general population, its association in BC survivors is unclear. Methods We included 3415 participants from the Pathways Study, a prospective cohort of women diagnosed with invasive BC between 2005 and 2013 and followed through 2021. Concordance of food intakes from food frequency questionnaires was estimated for five healthy eating patterns at BC diagnosis: Dietary Approaches to Stop Hypertension (DASH), healthy Plant-based Dietary Index (hPDI), 2020 Healthy Eating Index (HEI), American Cancer Society nutrition guidelines (ACS), and the alternate Mediterranean Diet Index (aMED). Incident hypertension, diabetes, and dyslipidemia were identified through electronic health records. Cumulative incidence rates (CIRs) were estimated accounting for the competing risk of death. Covariate-adjusted hazard ratios (HRs) and 95% confidence intervals (CIs) were calculated using Fine and Gray regression models, stratified by BC treatment status. Results Over an average 11.5 years (range = 0.3-16.3) of follow-up, 554 (16.2%) participants developed hypertension, 362 (10.6%) developed diabetes, and 652 (19.1%) developed dyslipidemia. CIRs for any cardiometabolic condition 15 years after BC diagnosis were 39.2% for women in the highest HEI quartile compared to 49.3% in the lowest. After adjustment, women in the highest HEI quartile had lower risks of any cardiometabolic condition (HR = 0.70, 95% CI 0.54-0.91, P-trend = 0.006), including hypertension (HR = 0.71, 95% CI 0.54-0.94, P-trend = 0.007), diabetes (HR = 0.57, 95% CI 0.41-0.79, P-trend < 0.001), and dyslipidemia (HR = 0.77, 95% CI 0.59-0.99, P-trend = 0.04). Similar associations were observed for DASH, hPDI, and ACS with

diabetes incidence. Conclusion Healthier diets at BC diagnosis, particularly those aligned with the HEI, were associated with lower cardiometabolic risks.[Ergas, Isaac J.; Roh, Janise M.; Iribarren, Carlos; Nguyen-Huynh, Mai; Rana, Jamal S.; Laurent, Cecile A.; Lee, Valerie S.; Quesenberry, Charles P.; Bhatt, Ankeet; Kushi, Lawrence H.; Kwan, Marilyn L.] Kaiser Permanente Northern Calif, Div Res, 4480 Hacienda Dr, Pleasanton, CA 94558 USA; [Cheng, Richard K.; Greenlee, Heather] Univ Washington, Sch Med, Seattle, WA USA; [Kresovich, Jacob K.] H Lee Moffitt Canc Ctr & Res Inst, Tampa, FL USA; [Rana, Jamal S.] Kaiser Permanente Northern Calif, Oakland Med Ctr, Oakland, CA USA; [Rillamas-Sun, Eileen; Greenlee, Heather] Fred Hutchinson Canc Ctr, Div Publ Hlth Sci, Seattle, WA USA; [Bhatt, Ankeet] Stanford Univ, Sch Med, Div Cardiovasc Med, Palo Alto, CA USA; [Yao, Song] Roswell Pk Comprehens Canc Ctr, Dept Canc Prevent & Control, Buffalo, NY USA
Kaiser Permanente; University of Washington; University of Washington Seattle; H Lee Moffitt Cancer Center & Research Institute; Kaiser Permanente; Fred Hutchinson Cancer Center; Stanford University; Roswell Park Comprehensive Cancer Center
Ergas, IJ (corresponding author), Kaiser Permanente Northern Calif, Div Res, 4480 Hacienda Dr, Pleasanton, CA 94558 USA.
isaac.j.ergas@kp.org Yao, Song/A-2534-2012; Kwan, Marilyn/AAH-4850-2021 National Cancer Institute National Cancer Institute(United States Department of Health & Human ServicesNational Institutes of Health (NIH) - USANIH National Cancer Institute (NCI)) We gratefully acknowledge the Pathways Study participants for their vital contributions to this research.
56 0 0 0 0 SPRINGER NEW YORK ONE NEW YORK PLAZA, SUITE 4600, NEW YORK, NY, UNITED STATES 0167-6806
1573-7217 BREAST CANCER RES TR Breast Cancer Res. Treat. MAY 2025 211 1 139 150
10.1007/s10549-025-07629-2
http://dx.doi.org/10.1007/s10549-025-07629-2 FEB 2025
12 Oncology Science Citation Index Expanded (SCI-EXPANDED) Oncology 0SG5P 39890673 2025-06-24 WOS:001412176100001
J Zhao, MM; Wu, QH; Hao, YH; Hu, JC; Gao, YX; Zhou, S; Han, LY
Zhao, Miaomiao; Wu, Qunhong; Hao, Yanhua; Hu, Jingcen; Gao, Yuexia; Zhou, Shan; Han, Liyuan
Global, regional, and national burden of cervical cancer for 195 countries and territories, 2007-2017: findings from the Global Burden of Disease Study 2017BMC WOMENS HEALTH
English Article Global Burden of Disease; Cervical cancer; Human papillomavirus; Incidence; Disability-adjusted life-years; Death SUB-SAHARAN AFRICA; HUMAN-PAPILLOMAVIRUS VACCINATION; SYSTEMATIC ANALYSIS; SEXUAL-BEHAVIOR; NORTH-AFRICA; MIDDLE-EAST; MORTALITY; WOMEN; EPIDEMIOLOGY; INFECTIONS Background Cervical cancer is one of the most common cancers among women worldwide. The formulation or evaluation on prevention strategies all require an accurate understanding of the burden for cervical cancer burden. We aimed to report the up-to-date estimates of cervical cancer burden at global, regional, and national levels. Methods Data were extracted from the Global Burden of Diseases, Injuries, and Risk Factors Study (GBD) 2017 study. The counts, age-standardized rates, and percentage changes of incidence, disability-adjusted life-years (DALYs), and death attributed to cervical cancer at the global, regional, and national levels in all 195 countries and territories

from 21 regions during 2007 to 2017 by age and by Socio-demographic Index (SDI) were measured. All estimates were reported with 95% uncertainty intervals (UIs). Results In 2017, 601,186 (95% UI 554,455 to 625,402) incident cases of cervical cancer were reported worldwide, which caused 8,061,667 (7,527,014 to 8,401,647) DALYs and 259,671 (241,128 to 269,214) deaths. The age-standardized rates for incidence, DALYs and death decreased by - 2.8% (- 7.8% to 0.6%), - 7.1% [- 11.8% to - 3.9%] and - 6.9% [- 11.5% to - 3.7%] from 2007 to 2017, respectively. The highest age-standardized incidence, DALYs and death rates in 2017 were observed in the low SDI quintile, Oceania, Central and Eastern Sub-Saharan Africa. During 2007 to 2017, only East Asia showed increase in these rates despite not significant. At the national level, the highest age-standardized rates for incidence, DALYs, and death in 2017 were observed in Kiribati, Somalia, Eritrea, and Central African Republic; and Georgia showed the largest increases in all these rates during 2007 to 2017. Conclusion Although the age-standardized rates for incidence, DALYs, and death of cervical cancer have decreased in most parts of the world from 2007 to 2017, cervical cancer remains a major public health concern in view of the absolute number of cervical cancer cases, DALYs, and deaths increased during this period. The challenge is more prone to in the low SDI quintile, Oceania, Central and Eastern Sub-Saharan Africa, East Asia, and some countries, suggesting an urgent to promote human papillomavirus vaccination in these regions. [Zhao, Miaomiao; Gao, Yuexia] Nantong Univ, Sch Publ Hlth, Dept Hlth Management, Nantong, Jiangsu, Peoples R China; [Wu, Qunhong; Hao, Yanhua] Harbin Med Univ, Sch Hlth Management, Dept Social Med, Harbin, Heilongjiang, Peoples R China; [Hu, Jingcen; Han, Liyuan] Univ Chinese Acad Sci, Hwa Mei Hosp, Dept Global Hlth, Ningbo, Zhejiang, Peoples R China; [Hu, Jingcen; Han, Liyuan] Univ Chinese Acad Sci, Ningbo Inst Life & Hlth Ind, Dept Global Hlth, Ningbo 315010, Zhejiang, Peoples R China; [Zhou, Shan] Univ Chinese Acad Sci, Hwa Mei Hosp, Dept Endocrinol, Ningbo, Zhejiang, Peoples R China Nantong University; Harbin Medical University; Chinese Academy of Sciences; University of Chinese Academy of Sciences, CAS; Chinese Academy of Sciences; University of Chinese Academy of Sciences, CAS; Chinese Academy of Sciences; University of Chinese Academy of Sciences, CAS Han, LY (corresponding author), Univ Chinese Acad Sci, Hwa Mei Hosp, Dept Global Hlth, Ningbo, Zhejiang, Peoples R China.; Zhou, S (corresponding author), Univ Chinese Acad Sci, Hwa Mei Hosp, Dept Endocrinol, Ningbo, Zhejiang, Peoples R China.

zhoushanningbo2020@126.com; hanqichunchen@126.com wu, wuqunhong/HNB-3313-2023; Zhou, Shan-Gui/B-3058-2008; hao, yan/HGB-0465-2022 Hwa Mei Research Fund of Hwa Mei Hospital, University of Chinese Academy of Sciences [2019HMKY32]; Innovative Talent Support Plan of the Medical and Health Technology Project in Zhejiang Province [2021422878]; National Social Science Foundation of China [19AZD013]; National Natural Science Foundation of China [82173648, 72004104] Hwa Mei Research Fund of Hwa Mei Hospital, University of Chinese Academy of Sciences; Innovative Talent Support Plan of the Medical and Health Technology Project in Zhejiang Province; National Social Science Foundation of China(National Office of Philosophy and Social Sciences); National Natural Science Foundation of China(National Natural Science Foundation of China (NSFC)) This research was funded by the Hwa Mei Research Fund of Hwa Mei Hospital,

University of Chinese Academy of Sciences (2019HMKY32), the Innovative Talent Support Plan of the Medical and Health Technology Project in Zhejiang Province (2021422878), National Social Science Foundation of China (19AZD013), and National Natural Science Foundation of China (82173648, 72004104). The funder had no role in the study design, data collection, data analysis, data interpretation, or preparation of the manuscript. All authors have full access to the study data and approve for the manuscript.	49	22	24	1	25	BMC	LONDON
	CAMPUS, 4 CRINAN ST, LONDON N1 9XW, ENGLAND	1472-6874
		BMC WOMENS HEALTH	BMC Womens Health	DEC 18
	2021	21	1				419
	10.1186/s12905-021-01571-3
	http://dx.doi.org/10.1186/s12905-021-01571-3		13
	Public, Environmental & Occupational Health; Obstetrics & Gynecology Science Citation Index Expanded (SCI-EXPANDED); Social Science Citation Index (SSCI)	Public, Environmental & Occupational Health; Obstetrics & Gynecology XQ2LS 34922503
	gold, Green Published		2025-06-24
	WOS:000731382700002
J	Odhiambo, P			Odhiambo, Paul
	Interregional mechanisms for protection of african migrants in the Gulf Region: What prospects for Africa?	OASIS-OBSERVATORIO DE ANALISIS DE LOS SISTEMAS INTERNACIONALES
	English	Article
	interregionalism; intra-African migration; multilateral forum; policy consultation and coordination	INTERNATIONAL MIGRATION	Migration is a common phenomenon in Africa as citizens migrate within the continent for several reasons including education, family, employment, trade, and investment. Significant numbers of African migrants travel to the Middle East in search of economic opportunities. With the intensification of globalisation and the advancement of technology, human mobility across international borders is expected to rise in the coming decades. Despite challenges of international migration, migrants play a significant role in destination countries as they boost working-age population; contribute to revenue generation through taxes; contribute to human capital development; and enrich technological progress. Countries of origin increasingly appreciate their Diaspora communities due to remittances and migrants' skills. The growing number of African migrants calls for a strategic partnership between Africa and the Middle East through a multilateral forum that will enhance policy consultation and coordination for the benefit for both sides. The paper analyses intra-African migration, migration patterns from Africa to the Middle East and Gulf Cooperation Council (GCC) countries, drivers of migrations, migrants' positive contributions and challenges; national and Africa's Regional Economic Communities (RECs) responses in protecting the migrants. Key recommendations including the strengthening of free movement policies is critical Africa in ensuring that rights of African migrants are protected within Africa; working towards improving migration data to inform policy; enhancing interregional cooperation between Africa's RECs to enhancing the potential of intra-African migration; and fast-tracking the establishment of an effective multilateral forum for Africa and the Middle East for enhancing policy consultation and coordination to guarantee their common interests.	[Odhiambo, Paul] Kenya Inst Publ Policy Res & Anal KIPPRA, Nairobi, Kenya;

[Odhiambo, Paul] Trade & Foreign Policy Dept KIPPRA, Nairobi, Kenya	Odhiambo, P (corresponding author), Kenya Inst Publ Policy Res & Anal KIPPRA, Nairobi, Kenya.; Odhiambo, P (corresponding author), Trade & Foreign Policy Dept KIPPRA, Nairobi, Kenya.	podhiambo@kippra.or.ke
	48	0	0	0	0	UNIV EXTERNADO COLOMBIA, DEPT PUBLICACIONES	BOGOTA	CALLE 12, NO 1-17 ESTE, BOGOTA, 00000, COLOMBIA	1657-7558	2346-2132		OASIS-OBS ANAL SIST	OASIS-Obs. Anal. Sist. Int.	JUL-DEC	2022	36
		181	202		10.18601/16577558.n36.11
	http://dx.doi.org/10.18601/16577558.n36.11		22
	International Relations	Emerging Sources Citation Index (ESCI)	International Relations	2H1VW	gold, Green Submitted		2025-06-24	WOS:000814086800011
J	Schairer, C; Soliman, AS; Omar, S; Khaled, H; Eissa, S; Ben Ayed, F; Khalafallah, S; Ben Ayoub, W; Kantor, ED; Merajver, S; Swain, SM; Gail, M; Brown, LM			Schairer, Catherine; Soliman, Amr S.; Omar, Sherif; Khaled, Hussein; Eissa, Saad; Ben Ayed, Farhat; Khalafallah, Samir; Ben Ayoub, Wided; Kantor, Elizabeth D.; Merajver, Sofia; Swain, Sandra M.; Gail, Mitchell; Brown, Linda Morris			Assessment of diagnosis of inflammatory breast cancer cases at two cancer centers in Egypt and TunisiaCANCER MEDICINE			English
	Article				Edema; Egypt; erythema; inflammatory breast cancer; peau d'orange; Tunisia
	The diagnosis of inflammatory breast cancer (IBC) is largely clinical and therefore inherently somewhat subjective. The objective of this study was to evaluate the diagnosis of IBC at two centers in North Africa where a higher proportion of breast cancer is diagnosed as IBC than in the United States (U.S.). Physicians prospectively enrolled suspected IBC cases at the National Cancer Institute (NCI) - Cairo, Egypt, and the Institut Salah Azaiz (ISA), Tunisia, recorded extent and duration of signs/symptoms of IBC on standardized forms, and took digital photographs of the breast. After second-level review at study hospitals, photographs and clinical information for confirmed IBC cases were reviewed by two U.S. oncologists. We calculated percent agreement between study hospital and U.S. oncologist diagnoses. Among cases confirmed by at least one U.S. oncologist, we calculated median extent and duration of signs and Spearman correlations. At least one U.S. oncologist confirmed the IBC diagnosis for 69% (39/50) of cases with photographs at the NCI-Cairo and 88% (21/24) of cases at the ISA. All confirmed cases had at least one sign of IBC (erythema, edema, peau d'orange) that covered at least one-third of the breast. The median duration of signs ranged from 1 to 3 months; extent and duration of signs were not statistically significantly correlated. From the above-mentioned outcomes, it can be concluded that the diagnosis of a substantial proportion of IBC cases is unambiguous, but a subset is difficult to distinguish from other types of locally advanced breast cancer. Among confirmed cases, the extent of signs was not related to delay in diagnosis.	[Schairer, Catherine; Gail, Mitchell; Brown, Linda Morris] NCI, Div Canc Epidemiol & Genet, Rockville, MD 20852 USA; [Soliman, Amr S.] Univ Nebraska, Med Ctr, Coll Publ Hlth, Dept Epidemiol, Omaha, NE 68198 USA; [Omar, Sherif] Cairo Univ, Natl Canc Inst, Dept Surg, Cairo 11796, Egypt; [Khaled, Hussein] Cairo Univ, Natl Canc Inst, Cairo 11796, Egypt; [Eissa, Saad] Cairo Univ, Natl Canc Inst, Dept Pathol, Cairo

11796, Egypt; [Ben Ayed, Farhat; Khalafallah, Samir; Ben Ayoub, Wided] Inst Salah Azaiz, Tunis 1006, Tunisia; [Kantor, Elizabeth D.] Univ Michigan, Sch Publ Hlth, Ann Arbor, MI 48109 USA; [Merajver, Sofia] Univ Michigan, Sch Med, Dept Internal Med, Ann Arbor, MI 48109 USA; [Swain, Sandra M.] Natl Naval Med Ctr, Med Oncol Branch, Bethesda, MD 20889 USA National Institutes of Health (NIH) - USA; NIH National Cancer Institute (NCI); NIH National Cancer Institute- Division of Cancer Epidemiology & Genetics; University of Nebraska System; University of Nebraska Medical Center; Egyptian Knowledge Bank (EKB); National Cancer Institute - Egypt; Cairo University; Egyptian Knowledge Bank (EKB); Cairo University; National Cancer Institute - Egypt; Egyptian Knowledge Bank (EKB); National Cancer Institute - Egypt; Cairo University; Universite de Tunis-El-Manar; Institut Salah Azaiez; University of Michigan System; University of Michigan; University of Michigan System; University of Michigan; Walter Reed National Military Medical Center Schairer, C (corresponding author), NCI, 6120 Execut Blvd,Rm 8026, Rockville, MD 20852 USA.
schairec@exchange.nih.gov Omar, Sherif/JDD-8433-2023
Brown, Linda/0000-0002-2181-9627; Swain, Sandra/0000-0002-1320-3830; Kantor, Elizabeth/0000-0002-9124-5323; Khalafallah, Shaaban/0000-0002-8593-9433 National Cancer Institute; National Cancer Institute [ZIACP010182] Funding Source: NIH RePORTER
National Cancer Institute(United States Department of Health & Human ServicesNational Institutes of Health (NIH) - USANIH National Cancer Institute (NCI)); National Cancer Institute(United States Department of Health & Human ServicesNational Institutes of Health (NIH) - USANIH National Cancer Institute (NCI)) This study was supported by intramural funds from the National Cancer Institute. 19 15 16 0 4 WILEY-BLACKWELL
HOBOKEN 111 RIVER ST, HOBOKEN 07030-5774, NJ USA
2045-7634 CANCER MED-US Cancer Med. APR 2013
2 2 178 184
10.1002/cam4.48 http://dx.doi.org/10.1002/cam4.48
7 Oncology Science Citation Index Expanded (SCI-EXPANDED) Oncology V36KI 23634285 Green Published, gold
2025-06-24 WOS:000209210600006
J Castello, A; Buijsse, B; Martín, M; Ruiz, A; Casas, AM; Baena-Cañada, JM; Pastor-Barriuso, R; Antolín, S; Ramos, M; Muñoz, M; Lluch, A; de Juan-Ferré, A; Jara, C; Lope, V; Jimeno, MA; Arriola-Arellano, E; Díaz, E; Guillem, V; Carrasco, E; Pérez-Gómez, B; Vioque, J; Pollán, M Castello, Adela; Buijsse, Brian; Martin, Miguel; Ruiz, Amparo; Casas, Ana M.; Baena-Canada, Jose M.; Pastor-Barriuso, Roberto; Antolin, Silvia; Ramos, Manuel; Munoz, Monserrat; Lluch, Ana; de Juan-Ferre, Ana; Jara, Carlos; Lope, Virginia; Jimeno, Maria A.; Arriola-Arellano, Esperanza; Diaz, Elena; Guillem, Vicente; Carrasco, Eva; Perez-Gomez, Beatriz; Vioque, Jesus; Pollan, Marina
GEICAM Researchers Evaluating the Applicability of Data-Driven Dietary Patterns to Independent Samples with a Focus on Measurement Tools for Pattern Similarity JOURNAL OF THE ACADEMY OF NUTRITION AND DIETETICS English
Article Dietary patterns; Pattern similarity; Congruence coefficient; Breast cancer; Mediterranean/Western patterns FOOD-FREQUENCY QUESTIONNAIRE; BREAST-CANCER RISK; PLASMA-CONCENTRATIONS; POSTMENOPAUSAL WOMEN; MULTIPLE IMPUTATION; RELATIVE VALIDITY; CHRONIC DISEASE; SWEDISH WOMEN; QUALITY; REPRODUCIBILITY Background Diet is a key

modifiable risk for many chronic diseases, but it remains unclear whether dietary patterns from one study sample are generalizable to other independent populations. Objective The primary objective of this study was to assess whether data-driven dietary patterns from one study sample are applicable to other populations. The secondary objective was to assess the validity of two criteria of pattern similarity. Methods Six dietary patterns-Western (n = 3), Mediterranean, Prudent, and Healthyfrom three published studies on breast cancer were reconstructed in a case-control study of 973 breast cancer patients and 973 controls. Three more internal patterns (Western, Prudent, and Mediterranean) were derived from this case-control study's own data. Statistical analysis Applicability was assessed by comparing the six reconstructed patterns with the three internal dietary patterns, using the congruence coefficient (CC) between pattern loadings. In cases where any pair met either of two commonly used criteria for declaring patterns similar (CC >= 0.85 or a statistically significant [P< 0.05] Pearson correlation), then the true similarity of those two dietary patterns was doublechecked by comparing their associations to risk for breast cancer, to assess whether those two criteria of similarity are actually reliable. Results Five of the six reconstructed dietary patterns showed high congruence (CC > 0.9) to their corresponding dietary pattern derived from the case-control study's data. Similar associations with risk for breast cancer were found in all pairs of dietary patterns that had high CC but not in all pairs of dietary patterns with statistically significant correlations. Conclusions Similar dietary patterns can be found in independent samples. The P value of a correlation coefficient is less reliable than the CC as a criterion for declaring two dietary patterns similar. This study shows that diet scores based on a particular study are generalizable to other populations. [Castello, Adela] Inst Salud Carlos III, Canc Epidemiol Unit, Natl Ctr Epidemiol, Ave Monforte Lemos 5, Madrid 28029, Spain; [Castello, Adela] Consortium Biomed Res Epidemiol & Publ Hlth, Madrid, Spain; [Castello, Adela; Buijsse, Brian] German Inst Human Nutr Potsdam Rehbrucke, Dept Epidemiol, Nuthetal, Germany; [Martin, Miguel] Hosp Clin Univ San Carlos, Med Oncol Unit, Med, Madrid, Spain; [Martin, Miguel] Gregorio Maranon Univ Complutense, Hlth Res Inst, Madrid, Spain; [Ruiz, Amparo; Guillem, Vicente] Inst Valenciano Oncol, Med Oncol Unit, Valencia, Spain; [Casas, Ana M.; Diaz, Elena] Hosp Virgen del Rocio, Med Oncol Unit, Seville, Spain; [Baena-Canada, Jose M.; Arriola-Arellano, Esperanza] Hosp Puerta del Mar, Med Oncol Unit, Cadiz, Spain; [Pastor-Barriuso, Roberto; Lope, Virginia; Perez-Gomez, Beatriz; Pollan, Marina] Inst Salud Carlos III, Canc Epidemiol Unit, Natl Ctr Epidemiol, Canc Epidemiol, Madrid, Spain; [Pastor-Barriuso, Roberto; Lope, Virginia; Perez-Gomez, Beatriz; Pollan, Marina] Inst Salud Carlos III, Consortium Biomed Res Epidemiol & Publ Hlth, Madrid, Spain; [Antolin, Silvia] Complejo Hosp Univ, Med Oncol Unit, La Coruna, Spain; [Ramos, Manuel] Ctr Oncol Galicia, Med Oncol Unit, La Coruna, Spain; [Munoz, Monserrat] Hosp Clin Barcelona, Med Oncol Unit, Barcelona, Spain; [Lluch, Ana] Univ Valencia, INCLIVA, Hosp Clin Univ Valencia, Med,Hematol Oncol Dept, Valencia, Spain; [de Juan-Ferre, Ana] Hosp Marques de Valdecilla, Med Oncol Unit, Santander, Spain; [Jara, Carlos] Univ Rey Juan Carlos, Hosp Univ Fdn Alcorcon, Med, Med Oncol Unit, Madrid, Spain; [Jimeno, Maria A.] Fdn GEICAM, Madrid, Spain; [Carrasco, Eva] GEICAM Spanish Grp

Breast Canc Res, Madrid, Spain; [Vioque, Jesus] Inst Salud Carlos III, Publ Hlth, Consortium Biomed Res Epidemiol & Publ Hlth, Madrid, Spain; [Vioque, Jesus] Univ Miguel Hernandez, Dept Publ Hlth, Sant Joan dAlacant, Spain Instituto de Salud Carlos III; CIBER - Centro de Investigacion Biomedica en Red; CIBERESP; Leibniz Association; Deutsches Institut fur Ernahrungsforschung Potsdam-Rehbrucke (DIfE); Instituto Valenciano De Oncologia; Virgen del Rocio University Hospital; Universidad de Cadiz; Hospital Universitario Puerta del Mar; Instituto de Salud Carlos III; CIBER - Centro de Investigacion Biomedica en Red; CIBERESP; Instituto de Salud Carlos III; University of Barcelona; Hospital Clinic de Barcelona; University of Valencia; Hospital Universitario Marques de Valdecilla (HUMV); Universidad Rey Juan Carlos; Alcorcon Foundation University Hospital; GEICAM; GEICAM; Instituto de Salud Carlos III; CIBER - Centro de Investigacion Biomedica en Red; CIBERESP; Universidad Miguel Hernandez de Elche
Castelló, A (corresponding author), Inst Salud Carlos III, Canc Epidemiol Unit, Natl Ctr Epidemiol, Ave Monforte Lemos 5, Madrid 28029, Spain. acastello@isciii.es Vioque, Jesus/A-1066-2008; Fernandez-Díaz, Elena/AAA-9148-2019; Lluch, Ana/R-5493-2019; Muñoz, Montserrat/AAO-9617-2020; Carrasco Bañuelos, Eva/HMP-7611-2023; Pollan, Marina/M-3259-2014; Perez-Gomez, Beatriz/C-4715-2012; Lope, Virginia/S-4774-2016; Pastor-Barriuso, Roberto/AAA-6746-2019; Castello, Adela/C-3829-2014 Pollan, Marina/0000-0002-4328-1565; Munoz Mateu, Montserrat/0000-0001-7772-1437; Perez-Gomez, Beatriz/0000-0002-4299-8214; Lluch, Ana/0000-0003-2766-407X; Lope, Virginia/0000-0002-6986-4021; Pastor-Barriuso, Roberto/0000-0002-7325-3960; Antolin Novoa, Silvia/0000-0002-0110-3349; Vioque, Jesus/0000-0002-2284-148X; Castello, Adela/0000-0002-1308-9927 Fundacion Cientifica Asociacion Espanola Contra el Cancer (Scientific Foundation of the Spanish Association Against Cancer); Spanish Ministry of Economy and Competitiveness [IJCI-2014-20900]; Fundacion Cerveza y Salud (Beer and Health Foundation); Sociedad Espanola de Oncologia Medica (Spanish Society of Medical Oncology); Federacion de Mujeres con Cancer de Mama (Association of Women with Breast Cancer) [EPY 1169-10]; Association of Women with Breast Cancer from Elche [EPY 1394/15]
Fundacion Cientifica Asociacion Espanola Contra el Cancer (Scientific Foundation of the Spanish Association Against Cancer); Spanish Ministry of Economy and Competitiveness(Spanish Government); Fundacion Cerveza y Salud (Beer and Health Foundation); Sociedad Espanola de Oncologia Medica (Spanish Society of Medical Oncology); Federacion de Mujeres con Cancer de Mama (Association of Women with Breast Cancer); Association of Women with Breast Cancer from Elche This study was funded by Fundacion Cientifica Asociacion Espanola Contra el Cancer (Scientific Foundation of the Spanish Association Against Cancer), the Spanish Ministry of Economy and Competitiveness (IJCI-2014-20900); Fundacion Cerveza y Salud 2005 (Beer and Health Foundation 2005), Sociedad Espanola de Oncologia Medica (Spanish Society of Medical Oncology), Federacion de Mujeres con Cancer de Mama (Association of Women with Breast Cancer) (EPY 1169-10 grant) and Association of Women with Breast Cancer from Elche (EPY 1394/15 grant). 43 24 24 0 11 ELSEVIER SCIENCE INC
NEW YORK 360 PARK AVE SOUTH, NEW YORK, NY 10010-1710 USA
2212-2672 2212-2680 J ACAD NUTR DIET J. Acad. Nutr. Diet. DEC 2016 116 12 1914 +
10.1016/j.jand.2016.05.008

http://dx.doi.org/10.1016/j.jand.2016.05.008 17
Nutrition & Dietetics Science Citation Index Expanded (SCI-EXPANDED) Nutrition & Dietetics ED9QY 27373727 Green Accepted 2025-06-24 WOS:000389208800006

J Derkaoui, T; Bakkach, J; Mansouri, M; Loudiyi, A; Fihri, M; Alaoui, FZ; Barakat, A; El Yemlahi, B; Bihri, H; Nourouti, NG; Mechita, MB Derkaoui, Touria; Bakkach, Joaira; Mansouri, Mohamed; Loudiyi, Ali; Fihri, Mohamed; Alaoui, Fatima Zahra; Barakat, Amina; El Yemlahi, Bouchra; Bihri, Hassan; Nourouti, Naima Ghailani; Mechita, Mohcine Bennani

Triple negative breast cancer in North of Morocco: clinicopathologic and prognostic features BMC WOMENS HEALTH English Article

Triple negative breast cancer; BRCA1; BRCA2; Prognostic; Clinicopathologic; Survival analysis ESTROGEN-RECEPTOR; WOMEN; SURVIVAL; BRCA1; PREVALENCE; RECURRENCE; MUTATIONS; PATTERNS; CARCINOMAS; RACE Background: Triple Negative Breast Cancer (TNBC) is defined by a lack of estrogen and progesterone receptor gene expression and by the absence of overexpression on HER2. It is associated to a poor prognosis. We propose to analyze the clinicopathologic and prognostic characteristics of this breast cancer subtype in a Mediterranean population originated or resident in the North of Morocco. Methods: We conducted a retrospective study of 279 patients diagnosed with breast cancer between January 2010 and January 2015. Clinicopathologic and prognostic features have been analyzed. Disease-Free Survival (DFS) and Overall Survival (OS) have been estimated. Results: Of all cases, forty-nine (17.6 %) were identified as having triple negative breast cancer with a median age of 46 years. The average tumor size was 3.6 cm. The majority of patients have had invasive ductal carcinoma (91.8 %) and 40.4 % of them were grade III SBR. Nodal metastasis was detected in 38.9 % of the patients and vascular invasion was found in 36.6 % of them. About half of the patients had an early disease (53.1 %) and 46.9 % were diagnosed at an advanced stage. Patients with operable tumors (61.2 %) underwent primary surgery and adjuvant chemotherapy. Patients with no operable tumors (26.5 %) received neoadjuvant chemotherapy followed by surgery, and patients with metastatic disease (12.2 %) were treated by palliative chemotherapy. DFS and OS at 5 years were respectively 83.7 and 71.4 %. Among 49, twelve had recurrences, found either when diagnosing them or after a follow-up. Local relapse was 6.1 %. Lung and liver metastases accounted consecutively for 8.2 and 10.2 %. Bone metastases were found in 4.1 % and brain metastases in 2.1 % of the cases. Conclusion: Our results are in accordance with literature data, particularly what concerning young age and poor prognosis among TNBC phenotype. Therefore, the identification of BRCA mutations in our population seems to be essential in order to better adapt management options for this aggressive form of breast cancer. [Derkaoui, Touria; Bakkach, Joaira; Mansouri, Mohamed; Alaoui, Fatima Zahra; Barakat, Amina; El Yemlahi, Bouchra; Bihri, Hassan; Nourouti, Naima Ghailani; Mechita, Mohcine Bennani] Univ Abdelmalek Essaadi, Human Genom Res Lab, Fac Sci & Tech Tangier, Tangier, Morocco; [Mansouri, Mohamed; Loudiyi, Ali] Oncol Clin Al Amal Tangier, Tangier, Morocco; [Fihri, Mohamed] Univ Abdelmalek Essaadi, Math & Applicat Lab, Fac Sci & Tech Tangier, Tangier, Morocco

Abdelmalek Essaadi University of Tetouan; Abdelmalek Essaadi University of Tetouan Derkaoui, T (corresponding author), Univ

Abdelmalek Essaadi, Human Genom Res Lab, Fac Sci & Tech Tangier, Tangier, Morocco. derkaoui.touria.22@gmail.comBakkach, Joaira/I-7022-2019; Zahra, Alaoui/AAQ-3086-2020; Fihri, Mohamed/M-8572-2017 BENNANI MECHITA, Mohcine/0000-0002-8137-0665; Fatima Zahra, Alaoui Ismaili/0000-0003-4691-0272; Fihri, Mohamed/0000-0001-6499-7258 39 8 8 0 3
BMC LONDON CAMPUS, 4 CRINAN ST, LONDON N1 9XW, ENGLAND
1472-6874 BMC WOMENS HEALTH BMC Womens Health OCT 22 2016 16
68 10.1186/s12905-016-0346-y
http://dx.doi.org/10.1186/s12905-016-0346-y 7
Public, Environmental & Occupational Health; Obstetrics & Gynecology Science Citation Index Expanded (SCI-EXPANDED); Social Science Citation Index (SSCI) Public, Environmental & Occupational Health; Obstetrics & Gynecology DZ7ZV 27770782
gold, Green Published 2025-06-24
WOS:000386088600001
J Jabeen, A; Sharma, A; Gupta, I; Kheraldine, H; Vranic, S; Al Moustafa, AE; Al Farsi, HF Jabeen, Ayesha; Sharma, Anju; Gupta, Ishita; Kheraldine, Hadeel; Vranic, Semir; Al Moustafa, Ala-Eddin; Al Farsi, Halema F. Elaeagnus angustifolia Plant Extract Inhibits Epithelial-Mesenchymal Transition and Induces Apoptosis via HER2 Inactivation and JNK Pathway in HER2-Positive Breast Cancer Cells MOLECULES
English Article Elaeagnus angustifolia; breast cancer; EMT; chemoprevention; apoptosis
TRAIL-INDUCED APOPTOSIS; C-JUN; SIGNAL-TRANSDUCTION; DEPENDENT DEGRADATION; LIVER-CANCER; BCL-2; PHOSPHORYLATION; ACTIVATION; L.; PREVENTION Elaeagnus angustifolia (EA) is a medicinal plant used for treating several human diseases in the Middle East. Meanwhile, the outcome of EA extract on HER2-positive breast cancer remains nascent. Thus, we herein investigated the effects of the aqueous EA extract obtained from the flowers of EA on two HER2-positive breast cancer cell lines, SKBR3 and ZR75-1. Our data revealed that EA extract inhibits cell proliferation and deregulates cell-cycle progression of these two cancer cell lines. EA extract also prevents the progression of epithelial-mesenchymal transition (EMT), an important event for cancer invasion and metastasis; this is accompanied by upregulations of E-cadherin and beta-catenin, in addition to downregulations of vimentin and fascin, which are major markers of EMT. Thus, EA extract causes a drastic decrease in cell invasion ability of SKBR3 and ZR75-1 cancer cells. Additionally, we found that EA extract inhibits colony formation of both cell lines in comparison with their matched control. The molecular pathway analysis of HER2 and JNK1/2/3 of EA extract exposed cells revealed that it can block HER2 and JNK1/2/3 activities, which could be the major molecular pathway behind these events. Our findings implicate that EA extract may possess chemo-preventive effects against HER2-positive breast cancer via HER2 inactivation and specifically JNK1/2/3 signaling pathways. [Jabeen, Ayesha; Sharma, Anju; Gupta, Ishita; Kheraldine, Hadeel; Vranic, Semir; Al Moustafa, Ala-Eddin; Al Farsi, Halema F.] Qatar Univ, Coll Med, QU Hlth, POB 2713, Doha, Qatar; [Jabeen, Ayesha; Gupta, Ishita; Kheraldine, Hadeel; Al Moustafa, Ala-Eddin] Qatar Univ, Biomed Res Ctr, POB 2713, Doha, Qatar; [Kheraldine, Hadeel] Qatar Univ, Coll Pharm, POB 2713, Doha, Qatar Qatar University; Qatar University; Qatar University Al Moustafa, AE; Al Farsi, HF (corresponding author),

Qatar Univ, Coll Med, QU Hlth, POB 2713, Doha, Qatar.; Al Moustafa, AE (corresponding author), Qatar Univ, Biomed Res Ctr, POB 2713, Doha, Qatar. jabeen@qu.edu.qa; anju.sharma7385@gmail.com; ishugupta28@gmail.com; hk1805332@student.qu.edu.qa; svranic@qu.edu.qa; aalmoustafa@qu.edu.qa; halfarsi@qu.edu.qa Vranic, Semir/J-4113-2012 Vranic, Semir/0000-0001-9743-7265; Gupta, Ishita/0000-0002-8627-3186; Sharma, Anju/0000-0001-7313-4286; Al Moustafa, Ala-Eddin/0000-0003-4452-3439; Kheraldine, Hadeel/0000-0002-9498-9387
Qatar University [QUCP-CMED-2019-1, QUHI-CMED-19/20-1, QUCG-CMED-20/21-2] Qatar University(Qatar UniversityQatar National Research Fund (QNRF)) Our lab is supported by grants from Qatar University: #QUCP-CMED-2019-1, QUHI-CMED-19/20-1, and QUCG-CMED-20/21-2. 81 23 24 2 12 MDPI BASEL ST ALBAN-ANLAGE 66, CH-4052 BASEL, SWITZERLAND 1420-3049
MOLECULES Molecules SEP 2020 25 18
4240 10.3390/molecules25184240
http://dx.doi.org/10.3390/molecules25184240 17
Biochemistry & Molecular Biology; Chemistry, Multidisciplinary Science Citation Index Expanded (SCI-EXPANDED) Biochemistry & Molecular Biology; Chemistry OF9MV
32947764 Green Published, gold 2025-06-24
WOS:000581522900001
J Carini, F; David, S; Tomasello, G; Mazzola, M; Damiani, P; Rappa, F; Battaglia, L; Cappello, F; Jurjus, A; Geagea, AG; Jurjus, R; Leone, A Carini, F.; David, S.; Tomasello, G.; Mazzola, M.; Damiani, P.; Rappa, F.; Battaglia, L.; Cappello, F.; Jurjus, A.; Geagea, A. Gerges; Jurjus, R.; Leone, A.
COLORECTAL CANCER: AN UPDATE ON THE EFFECTS OF LYCOPENE ON TUMOR PROGRESSION AND CELL PROLIFERATION JOURNAL OF BIOLOGICAL REGULATORS AND HOMEOSTATIC AGENTS
English Article
colorectal cancer; lycopene; oxidative stress; tomato; antioxidant; proliferation; progression MOUSE XENOGRAFT MODEL; COLON-CANCER; GROWTH; CAROTENOIDS; INHIBITION; SUPPRESSION; CONSUMPTION; PATHWAY Colorectal cancer (CRC) is one of the most common cancers worldwide. Various factors, including oxidative stress, where excessive productions of reactive oxygen species (ROS) and reactive nitrogen species (RNS) occur, contribute to its pathogenesis. Numerous studies have investigated the effect of antioxidant substances derived from food such as fruits and vegetables; however, data on Lycopene are still rare. Studies on HT-29 colorectal cancer cells and on animal models have shown that lycopene has effects on cell proliferation and on the progression of the CRC by interacting with various cellular signaling pathways. This analysis of the literature focused on the antioxidant effect of lycopene, a substance that is found in the tomato. [Carini, F.; David, S.; Tomasello, G.; Mazzola, M.; Rappa, F.; Cappello, F.; Geagea, A. Gerges] Univ Palermo, Sect Human Anat, BIONEC, Dept Expt Biomed & Clin Neurosci, Palermo, Italy; [Tomasello, G.; Mazzola, M.; Rappa, F.; Cappello, F.] Euro Mediterranean Inst Sci & Technol IEMEST Pale, Palermo, Italy; [Damiani, P.] Univ Palermo, Sch Med & Surg, AOUP P Giaccone, Palermo, Italy; [Battaglia, L.] Ist Zooprofilatt Sperimentale Sicilia, Palermo, Italy; [Jurjus, A.; Geagea, A. Gerges] Amer Univ Beirut, Dept Anat Cell Biol & Physiol Sci, Fac Med, Beirut, Lebanon; [Jurjus, R.] George Washington Sch Med & Hlth Sci, Dept Anat & Regenerat Biol, Washington, DC USA; [Leone, A.] Univ

Palermo, Sect Histol & Embryol, Dept Expt Biomed & Clin Neurosci, BIONEC, Palermo, Italy University of Palermo; University of Palermo; IZS Sicilia; American University of Beirut; George Washington University; University of Palermo Leone, A (corresponding author), Univ Palermo, Sch Med, Sect Histol & Embryol, BioNec, Via Vespro 129, I-90127 Palermo, Italy.
angelo.leone@unipa.it David, Sonia/AAU-6139-2020; Jurjus, Rosalyn/AAG-7853-2020; Francesca, Rappa/ADW-8431-2022; Cappello, Francesco/F-9153-2012 rappa, francesca/0000-0001-6610-5268; TOMASELLO, GIOVANNI/0000-0002-3071-822X; Cappello, Francesco/0000-0001-9288-1148; carini, francesco/0000-0001-9442-1671
23 25 27 0 6 BIOLIFE SAS SILVA MARINA (TE) VIA S STEFANO 39 BIS, 64029 SILVA MARINA (TE), ITALY
0393-974X 1724-6083 J BIOL REG HOMEOS AG J. Biol. Regul. Homeost. Agents JUL-SEP 2017 31 3
769 774 6
Endocrinology & Metabolism; Immunology; Medicine, Research & Experimental; Physiology Science Citation Index Expanded (SCI-EXPANDED) Endocrinology & Metabolism; Immunology; Research & Experimental Medicine; Physiology FJ4EE 28685524
2025-06-24 WOS:000412687500032
J Stasiewicz, B; Wadolowska, L; Biernacki, M; Slowinska, MA; Stachowska, E Stasiewicz, Beata; Wadolowska, Lidia; Biernacki, Maciej; Slowinska, Malgorzata Anna; Stachowska, Ewa Dietary Fat Intake: Associations with Dietary Patterns and Postmenopausal Breast Cancer-A Case-Control Study
CANCERS English Article
breast cancer; fat intake; dietary pattern; Mediterranean diet; women MEDITERRANEAN DIET; OLIVE OIL; RISK; HEALTH; MORTALITY; METAANALYSIS; HABITS; COHORT; ACIDS The aim of this study was to assess the associations of dietary fat intake with BC occurrence and dietary patterns. This case-control study involved 420 women aged 40-79 years from northeastern Poland, including 190 newly diagnosed BC cases. Dietary data were collected using a food frequency questionnaire (62-item FFQ-6 (R)). The Quick Food Scan of the National Cancer Institute and the Percentage Energy from Fat Screener scoring procedures were used to estimate the percentage energy from dietary fat (Pfat). The odds of BC occurrence was three times higher in the Pfat > 32%. The Pfat > 32% was positively associated with the 'Non-Healthy' DP and inversely associated with the Polish-aMED (R) score, 'Prudent' DP, and 'Margarine and Sweetened Dairy' DP. This case-control study suggests that a higher dietary fat intake (>32%) may contribute to an increased occurrence of peri- and postmenopausal breast cancer in women. Given the obtained results, an unhealthy dietary pattern characterized by the consumption of highly processed, high in sugar foods and animal fat foods should be avoided to reduce fat intake. Instead, the frequent consumption of low-processed plant foods, fish, and moderate consumption of low-fat dairy should be recommended since this pro-healthy diet is inversely associated with dietary fat intake. [Stasiewicz, Beata; Wadolowska, Lidia; Slowinska, Malgorzata Anna] Univ Warmia & Mazury, Fac Food Sci, Dept Human Nutr, Sloneczna 45f, PL-10718 Olsztyn, Poland; [Biernacki, Maciej] Univ Warmia & Mazury, Dept Surg, PL-11041 Olsztyn, Poland; [Stachowska, Ewa] Pomeranian Med Univ, Dept Human Nutr & Metabol, PL-71460 Szczecin, Poland
University of Warmia & Mazury; University of Warmia & Mazury; Pomeranian Medical University Stasiewicz, B

(corresponding author), Univ Warmia & Mazury, Fac Food Sci, Dept Human Nutr, Sloneczna 45f, PL-10718 Olsztyn, Poland.; Stachowska, E (corresponding author), Pomeranian Med Univ, Dept Human Nutr & Metabol, PL-71460 Szczecin, Poland.
beata.stasiewicz@uwm.edu.pl; lidia.wadolowska@uwm.edu.pl; maciej.biernacki@uwm.edu.pl; malgorzata.slowinska@uwm.edu.pl; ewa.stachowska@pum.edu.pl Wądołowska, Lidia/L-1458-2019; Stachowska, Ewa/G-9521-2017; Wadolowska, Lidia/O-8463-2018
Slowinska, Malgorzata/0000-0003-3836-8858; Stachowska, Ewa/0000-0002-4009-1977; Stasiewicz, Beata/0000-0003-0718-9101; Wadolowska, Lidia/0000-0001-8571-9935 program of the Minister of Science and Higher Education under the name "Regional Initiative of Excellence" in 2019-2022 Project [002/RID/2018/19] program of the Minister of Science and Higher Education under the name "Regional Initiative of Excellence" in 2019-2022 Project The project is financed from the program of the Minister of Science and Higher Education under the name "Regional Initiative of Excellence" in 2019-2022 Project Number 002/RID/2018/19 amount of financing 12,000,000 PLN. 69 3 3 0 9 MDPI
BASEL ST ALBAN-ANLAGE 66, CH-4052 BASEL, SWITZERLAND
2072-6694 CANCERS Cancers APR 2022 14 7
1724
10.3390/cancers14071724
http://dx.doi.org/10.3390/cancers14071724 19
Oncology Science Citation Index Expanded (SCI-EXPANDED)
Oncology 0L9OW 35406496 gold, Green Published
2025-06-24 WOS:000781796200001
J Hamdi, Y; Ben Rekaya, M; Shan, JX; Nagara, M; Messaoud, O; Elgaaied, AB; Mrad, R; Chouchane, L; Boubaker, MS; Abdelhak, S; Boussen, H; Romdhane, L Hamdi, Yosr; Ben Rekaya, Mariem; Shan Jingxuan; Nagara, Majdi; Messaoud, Olfa; Elgaaied, Amel Benammar; Mrad, Ridha; Chouchane, Lotfi; Boubaker, Mohamed Samir; Abdelhak, Sonia; Boussen, Hamouda; Romdhane, Lilia
A genome wide SNP genotyping study in the Tunisian population: specific reporting on a subset of common breast cancer risk loci BMC CANCER English
Article Breast cancer susceptibility; Haplotype analysis; Population genetics; Functional analysis SUSCEPTIBILITY LOCI; CONFER SUSCEPTIBILITY; HEREDITARY BREAST; GENETIC-STRUCTURE; AFRICAN-AMERICAN; R-PACKAGE; ASSOCIATION; MUTATIONS; VARIANTS; BRCA1 BackgroundBreast cancer is the most common cancer in women worldwide. Around 50% of breast cancer familial risk has been so far explained by known susceptibility alleles with variable levels of risk and prevalence. The vast majority of these breast cancer associated variations reported to date are from populations of European ancestry. In spite of its heterogeneity and genetic wealth, North-African populations have not been studied by the HapMap and the 1000Genomes projects. Thus, very little is known about the genetic architecture of these populations.MethodsThis study aimed to investigate a subset of common breast cancer loci in the general Tunisian population and to compare their genetic composition to those of other ethnic groups. We undertook a genome-wide haplotype study by genotyping 135 Tunisian subjects using the Affymetrix 6.0-Array. We compared Tunisian allele frequencies and linkage disequilibrium patterns to those of HapMap populations and we performed a comprehensive assessment of the functional effects of several selected variants.ResultsHaplotype analyses showed that at

risk haplotypes on 2p24, 4q21, 6q25, 9q31, 10q26, 11p15, 11q13 and 14q32 loci are considerably frequent in the Tunisian population (>20%). Allele frequency comparison showed that the frequency of rs13329835 is significantly different between Tunisian and all other HapMap populations. LD-blocks and Principle Component Analysis revealed that the genetic characteristics of breast cancer variants in the Tunisian, and so probably the North-African populations, are more similar to those of Europeans than Africans.Using eQTl analysis, we characterized rs9911630 as the most strongly expression-associated SNP that seems to affect the expression levels of BRCA1 and two long non coding RNAs (NBR2 and LINC008854). Additional in-silico analysis also suggested a potential functional significance of this variant.ConclusionsWe illustrated the utility of combining haplotype analysis in diverse ethnic groups with functional analysis to explore breast cancer genetic architecture in Tunisia. Results presented in this study provide the first report on a large number of common breast cancer genetic polymorphisms in the Tunisian population which may establish a baseline database to guide future association studies in North Africa. [Hamdi, Yosr; Ben Rekaya, Mariem; Nagara, Majdi; Messaoud, Olfa; Boubaker, Mohamed Samir; Abdelhak, Sonia; Romdhane, Lilia] Univ Tunis El Manar, Lab Biomed Genom & Oncogenet, Inst Pasteur Tunis, 13,Pl Pasteur BP 74, Tunis 1002, Belvedere, Tunisia; [Shan Jingxuan; Chouchane, Lotfi] Weill Cornell Med Coll Qatar, Dept Genet Med, Doha, Qatar; [Elgaaied, Amel Benammar] Univ Tunis El Manar, Fac Sci Tunis, Lab Genet Immunol & Human Pathol, Dept Biol, Tunis, Tunisia; [Mrad, Ridha] Charles Nicolle Hosp, Dept Human Genet, Tunis, Tunisia; [Boussen, Hamouda] Abderrahmen Mami Hosp, Med Oncol Dept, Ariana, Tunisia; [Romdhane, Lilia] Univ Tunis Carthage, Fac Sci Bizerte, Dept Biol, Tunis, Tunisia   Pasteur Network; Universite de Tunis-El-Manar; Institut Pasteur Tunis; Qatar Foundation (QF); Weill Cornell Medical College Qatar; Universite de Tunis-El-Manar; Faculte des Sciences de Tunis (FST); Universite de Tunis-El-Manar; Hopital Charles Nicolle; Universite de Tunis-El-Manar; Hopital Abderrahmene Mami; Universite de Carthage   Hamdi, Y (corresponding author), Univ Tunis El Manar, Lab Biomed Genom & Oncogenet, Inst Pasteur Tunis, 13,Pl Pasteur BP 74, Tunis 1002, Belvedere, Tunisia.   yosr.hamdi.82@gmail.com   Romdhane, Lilia/S-4691-2019; Chouchane, Lotfi/ABF-1360-2020; Messaoud, Olfa/I-8967-2019; Hamdi, Yosr/GLT-7117-2022; Elgaaied, Amel/AAL-1183-2020; Boussen, Hamouda/AAI-2796-2020; M'rad, Ridha/GLU-4806-2022; Abdelhak, Sonia/L-2831-2013 MESSAOUD, Olfa/0000-0003-4547-0180; Abdelhak, Sonia/0000-0001-8466-5525; Hamdi, Yosr/0000-0002-2815-1834; Romdhane, Lilia/0000-0001-5310-7272; Ben rekaya, Mariem/0000-0001-7176-5201 "Qatar National Research Foundation [NPRP 08-083-3-031]   "Qatar National Research Foundation(National Research Foundation of Korea) This work was supported by the "Qatar National Research Foundation" (NPRP 08-083-3-031) that contributed to the design of the study, collection, analysis, interpretation of data and in writing the manuscript.   80   11   12   0   6   BMC   LONDON
CAMPUS, 4 CRINAN ST, LONDON N1 9XW, ENGLAND   1471-2407
BMC CANCER BMC Cancer DEC 29   2018 18
1295 10.1186/s12885-018-5133-8
http://dx.doi.org/10.1186/s12885-018-5133-8   14
Oncology   Science Citation Index Expanded (SCI-EXPANDED)

Oncology HF9JZ 30594178 gold, Green Published 2025-06-24 WOS:000454559600007
J KrennHrubec, K; Mrad, K; Sriha, B; Ben Ayed, F; Bottalico, DM; Ostolaza, J; Smith, B; Tchaikovska, T; Soliman, AS; Burk, RD KrennHrubec, Keris; Mrad, Karima; Sriha, Badreddine; Ben Ayed, Farhat; Bottalico, Danielle M.; Ostolaza, Janae; Smith, Benjamin; Tchaikovska, Tatyana; Soliman, Amr S.; Burk, Robert D. HPV Types and Variants Among Cervical Cancer Tumors in Three Regions of Tunisia JOURNAL OF MEDICAL VIROLOGY English Article
North Africa; Cancer Registry; human papillomavirus (HPV); HPV variants; HPV16 HUMAN-PAPILLOMAVIRUS TYPE-16; INTRAEPITHELIAL NEOPLASIA; MOLECULAR VARIANTS; WOMEN; POPULATION; RISK; PREVALENCE; GENOTYPES; LINEAGES; LESIONS Cervical cancer is the second most common cancer among Tunisian women, and the incidence rates vary by region. Three Tunisian registries report age-standardized rates of 6.3/10(5) in the central region, 5.4/10(5) in the north, and 2.7/10(5) in the south. High-risk human papillomavirus (HPV) types and their variants differ in carcinogenic potential and geographic distribution. The HPV type and variant distribution could be a factor in the differing rates between regions of Tunisia. Tumor tissue was collected from 142 Tunisian cervical cancer patients. Demographic and reproductive characteristics of the patients were abstracted from cancer registry and hospital records. HPV type and variant analyses were performed using PCR-based Luminex and dot-blot hybridization assays. Eighty-three percent of tumors were infected with at least one HPV type. European variants of HPV16/18 were the most prevalent in tumors from all three regions, with all HPV18 infections and 64% of HPV16 infections being of European lineage. A higher frequency of HPV16 was present in Northern Tunisia (80%) than in Central (68%) or Southern Tunisia (50%) (P = 0.02). HPV18/45 was significantly more common in adenocarcinomas (50%) than in squamous cell carcinomas (11%) (P = 0.004). Frequent infection with European HPV variants most likely reflects the history of European migration to Tunisia. In addition to the importance of understanding the variants of HPV in Tunisia, behavioral and cultural attitudes towards screening and age-specific infection rates should be investigated to aid the development of future vaccination and HPV screening programs and policies. J. Med. Virol. 83:651-657, 2011. (C) 2011 Wiley-Liss, Inc. [KrennHrubec, Keris; Soliman, Amr S.] Univ Michigan, Sch Publ Hlth, Dept Epidemiol, Ann Arbor, MI 48109 USA; [Mrad, Karima] Salah Azaiez Canc Inst, Dept Anat & Pathol, Tunis, Tunisia; [Sriha, Badreddine] Farhat Hached Hosp, Dept Anat & Pathol, Sousse, Tunisia; [Ben Ayed, Farhat] Tunisian Assoc Canc Prevent & Control, Tunis, Tunisia; [Bottalico, Danielle M.; Ostolaza, Janae; Smith, Benjamin; Burk, Robert D.] Yeshiva Univ, Albert Einstein Coll Med, Dept Pediat, Bronx, NY USA; [Burk, Robert D.] Yeshiva Univ, Dept Microbiol, Albert Einstein Coll Med, Bronx, NY USA; [Burk, Robert D.] Yeshiva Univ, Albert Einstein Coll Med, Dept Immunol, Bronx, NY USA; [Burk, Robert D.] Yeshiva Univ, Albert Einstein Coll Med, Dept Epidemiol, Bronx, NY USA; [Burk, Robert D.] Yeshiva Univ, Albert Einstein Coll Med, Dept Populat Hlth, Bronx, NY USA; [Tchaikovska, Tatyana; Burk, Robert D.] Yeshiva Univ, Albert Einstein Coll Med, Dept Obstet Gynecol, Bronx, NY USA; [Tchaikovska, Tatyana; Burk, Robert D.] Yeshiva Univ, Albert Einstein Coll Med, Dept Womens Hlth, Bronx, NY USA University of

Michigan System; University of Michigan; Universite de Tunis-El-Manar; Institut Salah Azaiez; Universite de Sousse; Hopital Farhat Hached; Yeshiva University; Montefiore Medical Center; Albert Einstein College of Medicine; Montefiore Medical Center; Albert Einstein College of Medicine; Yeshiva University; Montefiore Medical Center; Albert Einstein College of Medicine; Yeshiva University; Montefiore Medical Center; Albert Einstein College of Medicine; Yeshiva University; Yeshiva University; Montefiore Medical Center; Albert Einstein College of Medicine; Montefiore Medical Center; Albert Einstein College of Medicine; Yeshiva University; Montefiore Medical Center; Albert Einstein College of Medicine; Yeshiva UniversityKrennHrubec, K (corresponding author), Univ Michigan, Sch Publ Hlth, Dept Epidemiol, 1415 Washington Hts,5626 Henry F Vaughn Publ Hlth, Ann Arbor, MI 48109 USA.
kkrennh@umich.edu University of Michigan
[R25 CA112383] University of Michigan(University of Michigan System) Grant sponsor: Cancer Epidemiology in Special Populations Program at the University of Michigan; Grant number: R25 CA112383. 32 11 13 0 5 WILEY-BLACKWELL
MALDEN COMMERCE PLACE, 350 MAIN ST, MALDEN 02148, MA USA
0146-6615 J MED VIROL J. Med. Virol. APR 2011
83 4 651 657
10.1002/jmv.22011 http://dx.doi.org/10.1002/jmv.22011
7 Virology Science Citation Index Expanded (SCI-EXPANDED) Virology 724IX 21328380 Green Accepted, Green Submitted 2025-06-24 WOS:000287570400013
J Vargas, AJ; Neuhouser, ML; George, SM; Thomson, CA; Ho, GYF; Rohan, TE; Kato, I; Nassir, R; Hou, LF; Manson, JE
Vargas, Ashley J.; Neuhouser, Marian L.; George, Stephanie M.; Thomson, Cynthia A.; Ho, Gloria Y. F.; Rohan, Thomas E.; Kato, Ikuko; Nassir, Rami; Hou, Lifang; Manson, JoAnn E. Diet Quality and Colorectal Cancer Risk in the Women's Health Initiative Observational Study AMERICAN JOURNAL OF EPIDEMIOLOGY English Article
Alternative Healthy Eating Index; Alternative Mediterranean Diet; colorectal cancer; diet; diet quality; Dietary Approaches to Stop Hypertension; dietary patterns; Healthy Eating Index
CORONARY-HEART-DISEASE; POSTMENOPAUSAL WOMEN; INFLAMMATORY INDEX; MORTALITY; PATTERNS; ASSOCIATION; SMOKING; STROKE; SCORES; FOOD Diet quality index scores on Healthy Eating Index 2010 (HEI-2010), Alternative HEI-2010, alternative Mediterranean Diet Index, and the Dietary Approaches to Stop Hypertension (DASH) index have been inversely associated with all-cause and cancer-specific death. This study assessed the association between these scores and colorectal cancer (CRC) incidence as well as CRC-specific mortality in the Women's Health Initiative Observational Study (1993-2012), a US study of postmenopausal women. During an average of 12.4 years of follow-up, there were 938 cases of CRC and 238 CRC-specific deaths. We estimated multivariate hazard ratios and 95% confidence intervals for relationships between quintiles of diet scores (from baseline food frequency questionnaires) and outcomes. HEI-2010 score (hazard ratios were 0.81, 0.77, and 0.73 with italic toggle="yes"P values of 0.04, 0.01, and < 0.01 for quintiles 3-5 vs. quintile 1, respectively) and DASH score (hazard ratios were 0.72, 0.74, and 0.78 with italic toggle="yes"P values of < 0.01, < 0.01, and 0.03 for quintiles 3-5 vs. quintile 1, respectively), but not other diet scores, were associated with a lower risk of CRC in adjusted models. No diet scores were

significantly associated with CRC-specific mortality. Closer adherence to HEI-2010 and DASH dietary recommendations was inversely associated with risk of CRC in this large cohort of postmenopausal women. [Vargas, Ashley J.; Neuhouser, Marian L.; George, Stephanie M.; Thomson, Cynthia A.; Ho, Gloria Y. F.; Rohan, Thomas E.; Kato, Ikuko; Nassir, Rami; Hou, Lifang; Manson, JoAnn E.] Harvard Med Sch, Brigham & Womens Hosp, Div Prevent Med, Dept Med, 900 Commonwealth Ave East,Third Floor, Boston, MA 02215 USA; [Vargas, Ashley J.] NCI, Canc Prevent Fellowship Program, Rockville, MD USA; [Vargas, Ashley J.] Harvard Univ, Dept Biostat, Harvard TH Chan Sch Publ Hlth, Boston, MA 02115 USA; [Neuhouser, Marian L.] Fred Hutchinson Canc Res Ctr, Canc Prevent Program, Seattle, WA USA; [George, Stephanie M.] NIH, Off Dis Prevent, Off Director, Rockville, MD USA; [Thomson, Cynthia A.] Univ Arizona, Canyon Ranch Ctr Prevent & Hlth Promot, Mel & Enid Zuckerman Coll Publ Hlth, Tucson, AZ USA; [Ho, Gloria Y. F.] Hofstra Northwell Sch Med, Dept Occupat Med Epidemiol & Prevent, Hempstead, NY USA; [Rohan, Thomas E.] Albert Einstein Coll Med, Dept Epidemiol & Populat Hlth, New York, NY USA; [Kato, Ikuko] Wayne State Univ, Sch Med, Dept Oncol, Detroit, MI USA; [Kato, Ikuko] Wayne State Univ, Sch Med, Dept Pathol, Detroit, MI 48201 USA; [Nassir, Rami] Univ Calif Davis, Dept Biochem & Mol Med, Sch Med, Davis, CA 95616 USA; [Hou, Lifang] Northwestern Univ, Dept Prevent Med, Feinberg Sch Med, Chicago, IL 60611 USA   Harvard University; Harvard Medical School; Harvard University Medical Affiliates; Brigham & Women's Hospital; National Institutes of Health (NIH) - USA; NIH National Cancer Institute (NCI); Harvard University; Harvard T.H. Chan School of Public Health; Fred Hutchinson Cancer Center; National Institutes of Health (NIH) - USA; University of Arizona; Northwell Health; Yeshiva University; Wayne State University; Wayne State University; University of California System; University of California Davis; Northwestern University; Feinberg School of Medicine   Manson, JE (corresponding author), Harvard Med Sch, Brigham & Womens Hosp, Div Prevent Med, Dept Med, 900 Commonwealth Ave East,Third Floor, Boston, MA 02215 USA.
jmanson@rics.bwh.harvard.eduManson, JoAnn/JOZ-3576-2023; Nassir, Rami/ADA-9013-2022 Hou, Lifang/0000-0003-4877-0031; Vargas, Ashley/0000-0002-4789-0675; Nassir, Rami/0000-0001-9914-7851 National Heart, Lung, and Blood Institute [HHSN268201100046C, HHSN268201100001C, HHSN268201100002C, HHSN268201100003C, HHSN268201100004C, HHSN271201100004C]; National Cancer Institute Cancer Prevention Fellowship Program   National Heart, Lung, and Blood Institute(United States Department of Health & Human ServicesNational Institutes of Health (NIH) - USANIH National Heart Lung & Blood Institute (NHLBI)); National Cancer Institute Cancer Prevention Fellowship Program(United States Department of Health & Human ServicesNational Institutes of Health (NIH) - USANIH National Cancer Institute (NCI)) The Women's Health Initiative (WHI) is funded by the National Heart, Lung, and Blood Institute through contracts HHSN268201100046C, HHSN268201100001C, HHSN268201100002C, HHSN268201100003C, HHSN268201100004C, and HHSN271201100004C. Additional support was provided by the National Cancer Institute Cancer Prevention Fellowship Program.   44   61   70   0   25   OXFORD UNIV PRESS INC   CARY JOURNALS DEPT, 2001 EVANS RD, CARY, NC 27513 USA 0002-9262 1476-6256   AM J EPIDEMIOL   Am. J. Epidemiol. JUL 1 2016 184   1   23   32
10.1093/aje/kwv304

http://dx.doi.org/10.1093/aje/kwv304 10
Public, Environmental & Occupational Health Science Citation Index Expanded (SCI-EXPANDED) Public, Environmental & Occupational Health DR2UV 27267948 Green Published, Bronze 2025-06-24 WOS:000379760300004
J Sukkarieh, O; Egede, LE; Bassil, M
Sukkarieh, Ola; Egede, Leonard E.; Bassil, Maya
Relationship between social determinants of health and quality of life in low income adults with diabetes in Lebanon
POSTGRADUATE MEDICINE English Article
Type 2 diabetes; social determinants of health; quality of life; MENA region; Lebanon ADVERSE CHILDHOOD EXPERIENCES; DEPRESSION; IMPACT; VALIDATION; OUTCOMES; ANXIETY; RISK; COMPLICATIONS; QUESTIONNAIRE; VALIDITY
Background & objectiveGlobal rates of type 2 diabetes (T2DM) are increasing, with the Middle East and North Africa (MENA) region having the second highest prevalence in the world. Populations from the MENA region, including Lebanon, are also witnessing massive waves of immigration to the western hemisphere. Limited data exist about how social determinants of health (SDOH) impact outcomes for T2DM in this population. Thus, the aim of this study was to assess the relationship between SDOH and quality of life (QoL) in Lebanese adults with T2DM.MethodsAdults with T2DM (n = 300) were recruited from primary healthcare centers in Lebanon. Demographic characteristics and WHO QoL domains (physical health, psychological wellbeing, social relationships, and environment) were assessed. SDOH included socioeconomic, neighborhood/built environment, and psychosocial variables. Partially and fully adjusted regression models were used to test for associations between SDOH and QoL domains.ResultsMean age of the participants was 60.3 years, 48% were women, 73% were married, and 64% had less than high-school education. Results from the fully adjusted regression models showed that psychosocial (i.e. adverse childhood experiences and depression), socioeconomic (i.e. employment, income, family size, insurance, financial status, and financial independence), and neighborhood/built environment (i.e. transportation, number of rooms in the household, and certain household items) variables were independent correlates of different QoL domains.ConclusionsThis study shows that psychosocial, socioeconomic, and neighborhood/built environment variables are differentially associated with different QoL domains, suggesting that SDOH factors are strongly associated with quality of life in low-income adults with T2DM in Lebanon.
[Sukkarieh, Ola] Lebanese Amer Univ, Alice Ramez Chagoury Sch Nursing, Byblos, Lebanon; [Egede, Leonard E.] Med Coll Wisconsin, Dept Med, Div Gen Internal Med, Milwaukee, WI USA; [Bassil, Maya] Qatar Univ, Coll Hlth Sci, Dept Human Nutr, QU Hlth, Doha, Qatar; [Bassil, Maya] Qatar Univ, Coll Hlth Sci, Dept Human Nutr, QU Hlth, POB 2713, Doha, Qatar Lebanese American University; Medical College of Wisconsin; Qatar University; Qatar University Bassil, M (corresponding author), Qatar Univ, Coll Hlth Sci, Dept Human Nutr, QU Hlth, POB 2713, Doha, Qatar.
bassil.maya@qu.edu.qa Egede, Leonard/0000-0003-1546-1515; Sukkarieh, Ola/0000-0001-9971-1563
53 4 4 1 5 TAYLOR & FRANCIS LTD ABINGDON
2-4 PARK SQUARE, MILTON PARK, ABINGDON OR14 4RN, OXON, ENGLAND 0032-5481 1941-9260 POSTGRAD MED Postgrad. Med. FEB 17 2023 135 2 169 178

10.1080/00325481.2023.2172283
http://dx.doi.org/10.1080/00325481.2023.2172283 FEB 2023 10 Medicine, General & InternalScience Citation Index Expanded (SCI-EXPANDED) General & Internal Medicine 9F6RP
36714928 Green Published, hybrid 2025-06-24 WOS:000923564300001
J Mas, S; Lafuente, MJ; Crescenti, A; Trias, M; Ballesta, A; Molina, R; Zheng, S; Wiencke, JK; Lafuente, A
Mas, Sergi; Lafuente, M. Jose; Crescenti, Anna; Trias, Manuel; Ballesta, Antonio; Molina, Rafael; Zheng, Shichun; Wiencke, John K.; Lafuente, Amalia Lower specific micronutrient intake in colorectal cancer patients with tumors presenting promoter hypermethylation in p16^{INK4a}, p14^{ARF} and hMLH1 ANTICANCER RESEARCH
English Article DNA hypermethylation; colorectal cancer; micronutrients; folate; vitamin CPG ISLAND METHYLATION; DNA METHYLATION; METHYLENETETRAHYDROFOLATE REDUCTASE; DEPENDENT RISK; FOLATE; GENE; HYPOMETHYLATION; SUSCEPTIBILITY; ASSOCIATION; EXPRESSION
Background: The diversity of the Mediterranean diet and the heterogeneity of acquired epigenetic alterations in colorectal cancer (CRC) led us to examine the possible association between dietary factors and promoter hypermethylation in genes implicated in the pathogenesis of these neoplasms (p16(INK4a), p14(ARF), hMLH1) and the interaction with methylene tetrahydrofolate reductase (MTHFR) C677T polymorphism. Patients and Methods: For the molecular study, 120 CRC patients were analyzed for hMLH1 promoter methylation status and MTHFR genotyping. Dietary patterns and molecular data on p16(INK4a) and p14(ARF) methylation were obtained from previous studies with this populations. Results: Patients with methylation in p16(INK4a) consumed significantly less folate (p=0.01), vitamin A (p=0.01), vitamin B1 (p=0.007), potassium (p=0.03) and iron (p=0.02) than controls. Patients with methylation in p14(ARF) or hMLH1 consumed significantly less vitamin A (p=0.001 and p=0.05, respectively). Conclusion: These results support that certain micronutrients protect against colorectal neoplasia and emphasize the importance of considering the different molecular forms of CRC as etiologically distinct diseases. Univ Barcelona, Sch Med, Dept Pharmacol, IDIBAPS, E-08036 Barcelona, Spain; Hosp Santa Creu & Sant Pau, Dept Surg, Barcelona 08025, Spain; Hosp Clin Barcelona, Dept Clin Chem, E-08036 Barcelona, Spain; Univ Calif San Francisco, Sch Med, Dept Epidemiol & Biostat, Lab Mol Epidemiol, San Francisco, CA 94143 USA University of Barcelona; Hospital Clinic de Barcelona; IDIBAPS; Hospital of Santa Creu i Sant Pau; University of Barcelona; Hospital Clinic de Barcelona; University of California System; University of California San Francisco Lafuente, A (corresponding author), Univ Barcelona, Fac Med, Dept Farmacol & Quim Terapeut, Casanova 143, E-08036 Barcelona, Spain.
amalialafuente@ub.edu Crescenti, Anna/AAA-7557-2019; Mas, Sergi/AAD-1996-2019 Crescenti, Anna/0000-0001-8581-0616; Mas, Sergi/0000-0003-3336-6298 NIEHS NIH HHS [ES 06717, ES 04704] Funding Source: Medline NIEHS NIH HHS(United States Department of Health & Human ServicesNational Institutes of Health (NIH) - USANIH National Institute of Environmental Health Sciences (NIEHS)) 36 18 20 0 3 INT INST ANTICANCER RESEARCH ATHENS EDITORIAL OFFICE 1ST KM KAPANDRITIOU-KALAMOU RD KAPANDRITI, PO BOX 22, ATHENS 19014,

GREECE 0250-7005 1791-7530 ANTICANCER RES
Anticancer Res. MAR-APR 2007 27 2
1151 1156 6 Oncology
Science Citation Index Expanded (SCI-EXPANDED) Oncology
155FJ 17465256 2025-06-24
WOS:000245563000065
J Mazzoccoli, G; Colangelo, T; Panza, A; Rubino, R; De Cata, A; Tiberio, C; Valvano, MR; Pazienza, V; Merla, G; Augello, B; Trombetta, D; Storlazzi, CT; Macchia, G; Gentile, A; Tavano, F; Vinciguerra, M; Bisceglia, G; Rosato, V; Colantuoni, V; Sabatino, L; Piepoli, A Mazzoccoli, Gianluigi; Colangelo, Tommaso; Panza, Anna; Rubino, Rosa; De Cata, Angelo; Tiberio, Cristiana; Valvano, Maria Rosa; Pazienza, Valerio; Merla, Giuseppe; Augello, Bartolomeo; Trombetta, Domenico; Storlazzi, Clelia Tiziana; Macchia, Gemma; Gentile, Annamaria; Tavano, Francesca; Vinciguerra, Manlio; Bisceglia, Giovanni; Rosato, Valeria; Colantuoni, Vittorio; Sabatino, Lina; Piepoli, Ada
Deregulated expression of cryptochrome genes in human colorectal cancer MOLECULAR CANCER English
Article
Clock gene; Cryptochrome; p53; Colorectal cancer; Chronotherapy; Circadian
CIRCADIAN TIMING SYSTEM; CLOCK GENES; CHEMOTHERAPY; SEX; RHYTHMS; ORGANIZATION; OXALIPLATIN; SENSITIVITY; DISRUPTION; MACHINERY
Background: Circadian disruption and deranged molecular clockworks are involved in carcinogenesis. The cryptochrome genes (CRY1 and CRY2) encode circadian proteins important for the functioning of biological oscillators. Their expression in human colorectal cancer (CRC) and in colon cancer cell lines has not been evaluated so far. Methods: We investigated CRY1 and CRY2 expression in fifty CRCs and in the CaCo2, HCT116, HT29, SW480 cell lines. Results: CRY1 ($p = 0.01$) and CRY2 ($p < 0.0001$) expression was significantly changed in tumour tissue, as confirmed in a large independent CRC dataset. In addition, lower CRY1 mRNA levels were observed in patients in the age range of 6274 years ($p = 0.018$), in female patients ($p = 0.003$) and in cancers located at the transverse colon ($p = 0.008$). Lower CRY2 levels were also associated with cancer location at the transverse colon ($p = 0.007$). CRC patients displaying CRY1 ($p = 0.042$) and CRY2 ($p = 0.043$) expression levels over the median were hallmarked by a poorer survival rate. Survey of selected colon cancer cell lines evidenced variable levels of cryptochrome genes expression and time-dependent changes in their mRNA levels. Moreover, they showed reduced apoptosis, increased proliferation and different response to 5-fluorouracil and oxaliplatin upon CRY1 and CRY2 ectopic expression. The relationship with p53 status came out as an additional layer of regulation: higher CRY1 and CRY2 protein levels coincided with a wild type p53 as in HCT116 cells and this condition only marginally affected the apoptotic and cell proliferation characteristics of the cells upon CRY ectopic expression. Conversely, lower CRY and CRY2 levels as in HT29 and SW480 cells coincided with a mutated p53 and a more robust apoptosis and proliferation upon CRY transfection. Besides, an heterogeneous pattern of ARNTL, WEE and c-MYC expression hallmarked the chosen colon cancer cell lines and likely influenced their phenotypic changes. Conclusion: Cryptochrome gene expression is altered in CRC, particularly in elderly subjects, female patients and cancers located at the transverse colon, affecting overall survival. Altered CRY1 and CRY2 expression

patterns and the interplay with the genetic landscape in colon cancer cells may underlie phenotypic divergence that could influence disease behavior as well as CRC patients survival and response to chemotherapy. [Mazzoccoli, Gianluigi; Rubino, Rosa; De Cata, Angelo; Tiberio, Cristiana; Vinciguerra, Manlio] IRCCS Sci Inst, Div Internal Med, San Giovanni Rotondo, FG, Italy; [Mazzoccoli, Gianluigi; Rubino, Rosa; De Cata, Angelo; Tiberio, Cristiana; Vinciguerra, Manlio] IRCCS Sci Inst, Chronobiol Unit, San Giovanni Rotondo, FG, Italy; [Mazzoccoli, Gianluigi; Panza, Anna; Rubino, Rosa; De Cata, Angelo; Tiberio, Cristiana; Valvano, Maria Rosa; Pazienza, Valerio; Merla, Giuseppe; Augello, Bartolomeo; Trombetta, Domenico; Gentile, Annamaria; Tavano, Francesca; Vinciguerra, Manlio; Bisceglia, Giovanni; Piepoli, Ada] Reg Gen Hosp Casa Sollievo della Sofferenza, San Giovanni Rotondo, FG, Italy; [Colangelo, Tommaso; Rosato, Valeria; Colantuoni, Vittorio; Sabatino, Lina] Univ Sannio, Dept Sci & Technol, Benevento, Italy; [Panza, Anna; Valvano, Maria Rosa; Pazienza, Valerio; Gentile, Annamaria; Tavano, Francesca] IRCCS Sci Inst, Div Gastroenterol, San Giovanni Rotondo, FG, Italy; [Panza, Anna; Valvano, Maria Rosa; Pazienza, Valerio; Gentile, Annamaria; Tavano, Francesca] IRCCS Sci Inst, Res Lab, San Giovanni Rotondo, FG, Italy; [Merla, Giuseppe; Augello, Bartolomeo] IRCCS Sci Inst, Med Genet, San Giovanni Rotondo, FG, Italy; [Trombetta, Domenico] IRCCS Sci Inst, Oncol Res Lab, San Giovanni Rotondo, FG, Italy; [Storlazzi, Clelia Tiziana; Macchia, Gemma] Univ Bari, Dept Biol, Bari, Italy; [Vinciguerra, Manlio] Euro Mediterranean Inst Sci & Technol IEMEST, Palermo, Italy; [Vinciguerra, Manlio] Nottingham Trent Univ, Sch Sci & Technol, Nottingham, England; [Vinciguerra, Manlio] UCL, Div Med, Inst Liver & Digest Hlth, London, England; [Bisceglia, Giovanni] IRCCS Sci Inst, Div Abdominal Surg, San Giovanni Rotondo, FG, Italy; [Piepoli, Ada] IRCCS Sci Inst, Div Epidemiol & Hlth Stat, San Giovanni Rotondo, FG, Italy; [Mazzoccoli, Gianluigi] IRCCS Sci Inst, Div Internal Med, Dept Med Sci, San Giovanni Rotondo, FG, Italy IRCCS Casa Sollievo Della Sofferenza; University of Sannio; Universita degli Studi di Bari Aldo Moro; Nottingham Trent University; University of London; University College London Mazzoccoli, G (corresponding author), IRCCS Sci Inst, Div Internal Med, San Giovanni Rotondo, FG, Italy. g.mazzoccoli@operapadrepio.it Sabatino, Lina/MFH-2120-2025; Pazienza, Valerio/K-5073-2012; merla, giuseppe/K-4627-2012; Piepoli, Ada/K-9299-2016; Vinciguerra, Manlio/N-1309-2015; Augello, Bartolomeo/B-1927-2017; Valvano, Maria Rosa/B-5118-2017; Mazzoccoli, Gianluigi Ubaldo/H-2447-2016; COLANGELO, TOMMASO/B-6119-2017; Panza, Anna/K-1989-2016; Tavano, Francesca/K-2203-2016; Trombetta, Domenico/K-6344-2016 Augello, Bartolomeo/0000-0001-7475-6976; Valvano, Maria Rosa/0000-0003-4704-8354; Mazzoccoli, Gianluigi Ubaldo/0000-0003-3535-7635; COLANGELO, TOMMASO/0000-0002-6927-403X; Panza, Anna/0000-0001-5840-0849; Pazienza, Valerio/0000-0002-3492-1153; Tavano, Francesca/0000-0002-8831-7349; Vinciguerra, Manlio/0000-0002-1768-3894; merla, giuseppe/0000-0001-5078-928X; Sabatino, Lina/0000-0001-5341-7430; Trombetta, Domenico/0000-0002-6156-8500; Storlazzi, Clelia Tiziana/0000-0002-1696-0028 "5x1000" voluntary contribution; Italian Ministry of Health through Department of Medical Sciences, Division of Gastroenterology [RC1203GA55, RC1203GA56]; Italian Ministry of Health through Division of Internal Medicine and Chronobiology Unit [RC1203ME46, RC1302ME31, RC1403ME50, RC1504ME53]; IRCCS Scientific Institute; Opera di Padre Pio da

Pietrelcina; San Giovanni Rotondo (FG), Italy; Italian Association on Cancer Research (AIRC) [MFAG 2012-13419, MFAG 2011-11405]; Regional General Hospital "Casa Sollievo della Sofferenza"
"5x1000" voluntary contribution; Italian Ministry of Health through Department of Medical Sciences, Division of Gastroenterology; Italian Ministry of Health through Division of Internal Medicine and Chronobiology Unit; IRCCS Scientific Institute; Opera di Padre Pio da Pietrelcina; San Giovanni Rotondo (FG), Italy; Italian Association on Cancer Research (AIRC)(Fondazione AIRC per la ricerca sul cancro); Regional General Hospital "Casa Sollievo della Sofferenza" We wish to express our gratitude and thank the colorectal cancer patients that each volunteered to participate in this study. We thank Angelo Andriulli for critical reading and Massimo Francavilla for support in computing. Financial support: the study was supported by the "5x1000" voluntary contribution, by a grant (AP) from the Italian Ministry of Health through Department of Medical Sciences, Division of Gastroenterology (RC1203GA55 and RC1203GA56), and (GM) through Division of Internal Medicine and Chronobiology Unit (RC1203ME46, RC1302ME31, RC1403ME50 and RC1504ME53), IRCCS Scientific Institute and Regional General Hospital "Casa Sollievo della Sofferenza", Opera di Padre Pio da Pietrelcina, San Giovanni Rotondo (FG), Italy, and by the Italian Association on Cancer Research (AIRC) (MFAG 2012-13419 to MV; MFAG 2011-11405 to CTS).
48 35 38 1 33 BMC LONDON CAMPUS, 4 CRINAN ST, LONDON N1 9XW, ENGLAND 1476-4598 MOL CANCER Mol. Cancer JAN 15 2016 15
6 10.1186/s12943-016-0492-8
http://dx.doi.org/10.1186/s12943-016-0492-8 20
Biochemistry & Molecular Biology; Oncology Science Citation Index Expanded (SCI-EXPANDED) Biochemistry & Molecular Biology; Oncology DA8QB 26768731 Green Published, gold
2025-06-24 WOS:000368069600002
J Partridge, AH; Niman, SM; Ruggeri, M; Peccatori, FA; Azim, HA ; Colleoni, M; Saura, C; Shimizu, C; Sætersdal, AB; Kroep, JR; Mailliez, A; Warner, E; Borges, VF; Amant, F; Gombos, A; Kataoka, A; Rousset-Jablonski, C; Borstnar, S; Takei, J; Lee, JE; Walshe, JM; Ruíz-Borrego, M; Moore, HCF; Saunders, C; Bjelic-Radisic, V; Susnjar, S; Cardoso, F; Smith, KL; Ferreiro, T; Ribi, K; Ruddy, K; Kammler, R; El-Abed, S; Viale, G; Piccart, M; Korde, LA; Goldhirsch, A; Gelber, RD; Pagani, O
Partridge, Ann H.; Niman, Samuel M.; Ruggeri, Monica; Peccatori, Fedro A.; Azim, Hatem A., Jr.; Colleoni, Marco; Saura, Cristina; Shimizu, Chikako; Saetersdal, Anna B.; Kroep, Judith R.; Mailliez, Audrey; Warner, Ellen; Borges, Virginia F.; Amant, Frederic; Gombos, Andrea; Kataoka, Akemi; Rousset-Jablonski, Christine; Borstnar, Simona; Takei, Junko; Lee, Jeong E.; Walshe, Janice M.; Ruiz-Borrego, Manuel; Moore, Halle C. F.; Saunders, Christobel; Bjelic-Radisic, Vesna; Susnjar, Snezana; Cardoso, Fatima; Smith, Karen L.; Ferreiro, Teresa; Ribi, Karin; Ruddy, Kathryn; Kammler, Roswitha; El-Abed, Sarra; Viale, Giuseppe; Piccart, Martine; Korde, Larissa A.; Goldhirsch, Aron; Gelber, Richard D.; Pagani, Olivia Int Breast Canc Study Grp POSITIVE Interrupting Endocrine Therapy to Attempt Pregnancy after Breast Cancer NEW ENGLAND JOURNAL OF MEDICINE
English Article
YOUNG-WOMEN; FERTILITY; PRESERVATION; ISSUES Background Prospective data on the risk of recurrence among women with

hormone receptor-positive early breast cancer who temporarily discontinue endocrine therapy to attempt pregnancy are lacking. Methods We conducted a single-group trial in which we evaluated the temporary interruption of adjuvant endocrine therapy to attempt pregnancy in young women with previous breast cancer. Eligible women were 42 years of age or younger; had had stage I, II, or III disease; had received adjuvant endocrine therapy for 18 to 30 months; and desired pregnancy. The primary end point was the number of breast cancer events (defined as local, regional, or distant recurrence of invasive breast cancer or new contralateral invasive breast cancer) during follow-up. The primary analysis was planned to be performed after 1600 patient-years of follow-up. The prespecified safety threshold was the occurrence of 46 breast cancer events during this period. Breast cancer outcomes in this treatment-interruption group were compared with those in an external control cohort consisting of women who would have met the entry criteria for the current trial. Results Among 516 women, the median age was 37 years, the median time from breast cancer diagnosis to enrollment was 29 months, and 93.4% had stage I or II disease. Among 497 women who were followed for pregnancy status, 368 (74.0%) had at least one pregnancy and 317 (63.8%) had at least one live birth. In total, 365 babies were born. At 1638 patient-years of follow-up (median follow-up, 41 months), 44 patients had a breast cancer event, a result that did not exceed the safety threshold. The 3-year incidence of breast cancer events was 8.9% (95% confidence interval [CI], 6.3 to 11.6) in the treatment-interruption group and 9.2% (95% CI, 7.6 to 10.8) in the control cohort. Conclusions Among select women with previous hormone receptor-positive early breast cancer, temporary interruption of endocrine therapy to attempt pregnancy did not confer a greater short-term risk of breast cancer events, including distant recurrence, than that in the external control cohort. Further follow-up is critical to inform longer-term safety. (Funded by ETOP IBCSG Partners Foundation and others; POSITIVE ClinicalTrials.gov number, NCT02308085.) Interrupting Therapy to Attempt Pregnancy after Breast Cancer In women with previous breast cancer who temporarily discontinued adjuvant endocrine therapy to attempt pregnancy, the frequency of breast cancer events was below the prespecified safety threshold and similar to that in controls.[Partridge, Ann H.; Gelber, Richard D.] Harvard Med Sch, Dept Med Oncol, Dana Farber Canc Inst, Boston, MA USA; [Niman, Samuel M.; Gelber, Richard D.] Dana Farber Canc Inst, Int Breast Canc Study Grp Stat Ctr, Boston, MA USA; [Niman, Samuel M.; Gelber, Richard D.] Dana Farber Canc Inst, Dept Data Sci, Div Biostat, Boston, MA USA; [Gelber, Richard D.] Frontier Sci & Technol Res Fdn Inc, Boston, MA USA; [Gelber, Richard D.] Harvard TH Chan Sch Publ Hlth, Boston, MA USA; [Ruggeri, Monica; Ribi, Karin; Kammler, Roswitha; Viale, Giuseppe; Goldhirsch, Aron] Int Breast Canc Study Grp, Bern, Switzerland; [Pagani, Olivia] Swiss Grp Clin Canc Res, Bern, Switzerland; [Pagani, Olivia] Interdisciplinary Canc Serv Hosp Riviera Chablais, Geneva, Switzerland; [Pagani, Olivia] Lugano Univ, Univ Hosp, Lugano, Switzerland; [Peccatori, Fedro A.; Colleoni, Marco; Viale, Giuseppe; Goldhirsch, Aron] Sci Inst Res Hospitalizat & Healthcare IRCCS, European Inst Oncol, Milan, Italy; [Azim, Hatem A., Jr.] Tecnol Monterrey, Breast Canc Ctr, Hosp Zambrano Hellion, Sch Med, San Pedro Garza Garcia, Mexico; [Saura, Cristina] Vall dHebron Univ Hosp, Vall dHebron Inst Oncol, Barcelona, Spain;

[Saura, Cristina] SOLTI Breast Canc Res Grp, Barcelona, Spain; [Ferreiro, Teresa] Soul Reconnect, Barcelona, Spain; [Ruiz-Borrego, Manuel] GEICAM Spanish Breast Canc Res Grp, Madrid, Spain; [Ruiz-Borrego, Manuel] Hosp Virgen del Rocio Sevilla, Seville, Spain; [Shimizu, Chikako] Natl Ctr Global Hlth & Med, Dept Breast & Med Oncol, Tokyo, Japan; [Kataoka, Akemi] Japanese Fdn Canc Res, Canc Inst Hosp, Breast Oncol Ctr, Tokyo, Japan; [Takei, Junko] St Lukes Int Hosp, Breast Ctr, Tokyo, Japan; [Saetersdal, Anna B.] Oslo Univ Hosp, Breast Canc Unit, Dept Oncol, Div Canc Med, Oslo, Norway; [Kroep, Judith R.] Leiden Univ, Med Ctr, Dept Med Oncol, Leiden, Netherlands; [Amant, Frederic] Antoni van Leeuwenhoek Netherlands Canc Inst, Amsterdam, Netherlands; [Mailliez, Audrey] Ctr Oscar Lambret, Dept Med Oncol, Lille, France; [Rousset-Jablonski, Christine] Ctr Leon Berard, Leon Berard Canc Ctr, Dept Surg, Lyon, France; [Warner, Ellen] Sunnybrook Hlth Sci Ctr, Odette Canc Ctr, Toronto, ON, Canada; [Borges, Virginia F.] Univ Colorado, Canc Ctr, Div Med Oncol, Dept Med, Aurora, CO USA; [Amant, Frederic] Katholieke Univ Leuven, Dept Oncol, Leuven, Belgium; [Amant, Frederic] Univ Hosp Leuven, Leuven Canc Inst, Leuven, Belgium; [Amant, Frederic] Univ Hosp Leuven, Dept Obstet & Gynecol, Leuven, Belgium; [Gombos, Andrea; Piccart, Martine] Inst Jules Bordet, Brussels, Belgium; [Gombos, Andrea; Piccart, Martine] Univ Libre Bruxelles, Brussels, Belgium; [El-Abed, Sarra] Breast Int Grp, Brussels, Belgium; [Borstnar, Simona] Inst Oncol, Div Med Oncol, Ljubljana, Slovenia; [Lee, Jeong E.] Sungkyunkwan Univ, Sch Med, Breast Div, Dept Surg,Samasung Med Ctr, Seoul, South Korea; [Lee, Jeong E.] Sungkyunkwan Univ, Dept Clin Res & Evaluat, Samsung Adv Inst Hlth Sci & Technol, Seoul, South Korea; [Walshe, Janice M.] St Vincents Univ Hosp, Canc Trials Ireland, Dept Med Oncol, Dublin, Ireland; [Walshe, Janice M.] Tallaght Univ Hosp, Dublin, Ireland; [Moore, Halle C. F.] Cleveland Clin, Taussig Canc Inst, Breast Oncol Program, Cleveland, Qld, Australia; [Saunders, Christobel] Univ Melbourne, Melbourne Med Sch, Ctr Canc Res, Dept Surg, Melbourne, Vic, Australia; [Saunders, Christobel] Univ Melbourne, Royal Melbourne Hosp, Melbourne, Vic, Australia; [Bjelic-Radisic, Vesna] Univ Witten Herdecke, Breast Unit, Helios Univ Hosp Wuppertal, Wuppertal, Germany; [Bjelic-Radisic, Vesna] Med Univ Graz, Dept Obstet & Gynecol, Graz, Austria; [Susnjar, Snezana] Inst Oncol & Radiol Serbia, Dept Med Oncol, Belgrade, Serbia; [Cardoso, Fatima] Champalimaud Fdn, Breast Unit, Chapalimaud Clin Ctr, Lisbon, Portugal; [Smith, Karen L.] Johns Hopkins Univ, Sidney Kimmel Comprehens Canc Ctr, Baltimore, MD USA; [Korde, Larissa A.] NCI, Breast Canc & Melanoma Therapeut, Canc Therapy Evaluat Program, Bethesda, MD USA; [Ruddy, Kathryn] Mayo Clin, Dept Oncol, Rochester, MN USA Harvard University; Harvard Medical School; Harvard University Medical Affiliates; Dana-Farber Cancer Institute; Harvard University; Harvard University Medical Affiliates; Dana-Farber Cancer Institute; Harvard University; Harvard University Medical Affiliates; Dana-Farber Cancer Institute; Frontier Science Foundation; Harvard University; Harvard T.H. Chan School of Public Health; International Breast Cancer Study Group; Swiss Group for Clinical Cancer Research (SAKK); Universita della Svizzera Italiana; IRCCS European Institute of Oncology (IEO); Tecnologico de Monterrey; Vall d'Hebron Institut d'Oncologia (VHIO); Hospital Universitari Vall d'Hebron; SOLTI Breast Cancer Research Group; GEICAM; Virgen del Rocio University Hospital; Japan Institute for Health Security

(JIHS); National Center for Global Health & Medicine - Japan; Japanese Foundation for Cancer Research; St. Luke's International Hospital; University of Oslo; Leiden University - Excl LUMC; Leiden University; Leiden University Medical Center (LUMC); Netherlands Cancer Institute; UNICANCER; Centre Oscar Lambret; UNICANCER; Centre Leon Berard; University of Toronto; Sunnybrook Health Science Center; Sunnybrook Research Institute; University of Colorado System; University of Colorado Anschutz Medical Campus; KU Leuven; KU Leuven; University Hospital Leuven; KU Leuven; University Hospital Leuven; Institut Jules Bordet; Universite Libre de Bruxelles; Breast International Group; Institute of Oncology - Slovenia; Sungkyunkwan University (SKKU); Sungkyunkwan University (SKKU); Samsung Medical Center; University College Dublin; Saint Vincent's University Hospital; Cleveland Clinic Foundation; University of Melbourne; University of Melbourne; Melbourne Health; Royal Melbourne Hospital; Witten Herdecke University; Medical University of Graz; Institute for Oncology & Radiology of Serbia (IORS); Fundacao Champalimaud; Johns Hopkins University; Johns Hopkins Medicine; National Institutes of Health (NIH) - USA; NIH National Cancer Institute (NCI); Mayo Clinic   Partridge, AH (corresponding author), Dana Farber Canc Inst, Dept Med Oncol, 450 Brookline Ave, Boston, MA 02215 USA. ann_partridge@dfci.harvard.edu   Pagani, Olivia/ABF-3164-2020; Saura, Carlos/AFM-9239-2022; Viale, Giuseppe/AAE-8921-2019; Rousset-Jablonski, Christine/AAE-1102-2020; Cardoso, Fatima/IWM-5523-2023; Saunders, Christobel/H-5779-2014; Amant, Frédéric/W-7436-2019; Lee, Jeeyun/I-7171-2015; peccatori, fedro/AAP-7058-2020   ferro, antonella/0000-0003-4109-6769; peccatori, fedro/0000-0001-8227-8740; Borrego, Maria Jose/0000-0002-8604-8800; Walshe, Janice/0009-0002-1938-8730; Ribi, Karin/0000-0003-4724-1197; El-abed, Sarra/0000-0002-8783-9671; Rousset-Jablonski, Christine/0000-0001-7087-5687   ETOP IBCSG Partners Foundation (globally); Alliance for Clinical Trials in Oncology (in North America); Breast International Group (BIG); BIG cooperative groups; National Clinical Trials Network of the National Cancer Institute; International Breast Cancer Study Group (IBCSG); Frontier Science and Technology Research Foundation - Switzerland; Southern Europe (Frontier Southern Europe) - Switzerland; Pink Ribbon Switzerland - Switzerland; Swiss Cancer League - Switzerland [KLS-3361-02]; Rising Tide Foundation for Clinical Cancer Research - Switzerland [CCR-15-120]; Swiss Group for Clinical Cancer Research - Switzerland; Clinical Cancer Research Foundation of Eastern Switzerland - Switzerland; Roche Diagnostics International - Switzerland; Swiss Cancer Foundation - Switzerland; Piajoh Fondazione di Famiglia - Switzerland; Gruppo Giovani Pazienti "Anna dai Capelli Corti" - Switzerland; Verein Barguf - Switzerland; Schweizer Frauenlauf Bern - Switzerland; BIG Against Breast Cancer, Belgium; Baillet Latour Fund, Belgium; Gateway for Cancer Research, United States [G-15-1900]; Breast Cancer Research Foundation - United States; C A, Germany; Dutch Cancer Society, the Netherlands; Norwegian Breast Cancer Society in Norway; Pink Ribbon - Norway; ELGC K.K. in Japan; Pink Ring in Japan; Korea Breast Cancer Foundation in South Korea; Mr. Yong Seop Lee in South Korea; National Cancer Institute of the National Institutes of Health (NIH) (Alliance for Clinical Trials in Oncology National Cancer Institute Community Oncology Research Program [NCORP] grant [UG1CA189823]; biorepository resource grant [U24CA196171]; ECOG-ACRIN NCORP grants [UG1CA189828, UG1CA233196];

NIH grants [UG1CA189974, U10CA180888, U10CA180868]; NCORP grant [UG1CA189867]; National Cancer Institute of the NIH [CA180863]; Canadian Cancer Society [707213]; Canada Foundation for Innovation; RETHINK Breast Cancer, Canada; Gilson Family Foundation, United States ETOP IBCSG Partners Foundation (globally); Alliance for Clinical Trials in Oncology (in North America); Breast International Group (BIG)(Breast International Group); BIG cooperative groups; National Clinical Trials Network of the National Cancer Institute; International Breast Cancer Study Group (IBCSG); Frontier Science and Technology Research Foundation - Switzerland; Southern Europe (Frontier Southern Europe) - Switzerland; Pink Ribbon Switzerland - Switzerland; Swiss Cancer League - Switzerland; Rising Tide Foundation for Clinical Cancer Research - Switzerland; Swiss Group for Clinical Cancer Research - Switzerland; Clinical Cancer Research Foundation of Eastern Switzerland - Switzerland; Roche Diagnostics International - Switzerland; Swiss Cancer Foundation - Switzerland; Piajoh Fondazione di Famiglia - Switzerland; Gruppo Giovani Pazienti "Anna dai Capelli Corti" - Switzerland; Verein Barguf - Switzerland; Schweizer Frauenlauf Bern - Switzerland; BIG Against Breast Cancer, Belgium; Baillet Latour Fund, Belgium; Gateway for Cancer Research, United States; Breast Cancer Research Foundation - United States; C A, Germany; Dutch Cancer Society, the Netherlands(KWF KankerbestrijdingNetherlands Government); Norwegian Breast Cancer Society in Norway; Pink Ribbon - Norway; ELGC K.K. in Japan; Pink Ring in Japan; Korea Breast Cancer Foundation in South Korea; Mr. Yong Seop Lee in South Korea; National Cancer Institute of the National Institutes of Health (NIH) (Alliance for Clinical Trials in Oncology National Cancer Institute Community Oncology Research Program [NCORP] grant; biorepository resource grant; ECOG-ACRIN NCORP grants; NIH grants(United States Department of Health & Human ServicesNational Institutes of Health (NIH) - USA); NCORP grant; National Cancer Institute of the NIH(United States Department of Health & Human ServicesNational Institutes of Health (NIH) - USANIH National Cancer Institute (NCI)); Canadian Cancer Society(Canadian Cancer Society (CCS)); Canada Foundation for Innovation(Canada Foundation for InnovationCGIARSpanish Government); RETHINK Breast Cancer, Canada; Gilson Family Foundation, United States Supported by the ETOP IBCSG Partners Foundation (globally) and by the Alliance for Clinical Trials in Oncology (in North America), in collaboration with the Breast International Group (BIG), the BIG cooperative groups, and the National Clinical Trials Network of the National Cancer Institute. Globally, the trial receives grant support for central or local trial conduct from the following: the International Breast Cancer Study Group (IBCSG); Frontier Science and Technology Research Foundation, Southern Europe (Frontier Southern Europe), Pink Ribbon Switzerland, Swiss Cancer League (KLS-3361-02), San Salvatore Foundation, Rising Tide Foundation for Clinical Cancer Research (CCR-15-120), Swiss Group for Clinical Cancer Research, Clinical Cancer Research Foundation of Eastern Switzerland, Roche Diagnostics International, Swiss Cancer Foundation, Piajoh Fondazione di Famiglia, Gruppo Giovani Pazienti "Anna dai Capelli Corti," Verein Barguf, and Schweizer Frauenlauf Bern - all in Switzerland; BIG Against Breast Cancer and the Baillet Latour Fund, Belgium; Gateway for Cancer Research (G-15-1900) and Breast Cancer Research Foundation -both in the United States; C & A, Germany; Dutch Cancer Society, the Netherlands;

Norwegian Breast Cancer Society and Pink Ribbon - both in Norway; ELGC K.K. and Pink Ring - both in Japan; Korea Breast Cancer Foundation and Mr. Yong Seop Lee - both in South Korea; and other private donors. In North America, the Alliance for Clinical Trials in Oncology receives support from the National Cancer Institute of the National Institutes of Health (NIH) (Alliance for Clinical Trials in Oncology National Cancer Institute Community Oncology Research Program [NCORP] grant UG1CA189823) and the biorepository resource grant U24CA196171; the Eastern Cooperative Oncology Group-American College of Radiology Imaging Network (ECOG-ACRIN) receives support under ECOG-ACRIN NCORP grants UG1CA189828 and UG1CA233196; Southwest Oncology Group Cancer Research Network receives support under NIH grants UG1CA189974 and U10CA180888; and NRG Oncology receives support under NIH grant U10CA180868 and NCORP grant UG1CA189867. Canadian Cancer Trials Group (CCTG) participation in the trial is supported through its grant from the National Cancer Institute of the NIH (CA180863). Additional programmatic funding support for the CCTG is provided by the Canadian Cancer Society (707213) and the Canada Foundation for Innovation. In addition, the trial receives support from RETHINK Breast Cancer, Canada, and the Gilson Family Foundation, United States. 27 113 118 4 35 MASSACHUSETTS MEDICAL SOC WALTHAM WALTHAM WOODS CENTER, 860 WINTER ST,, WALTHAM, MA 02451-1413 USA 0028-4793 1533-4406 NEW ENGL J MED N. Engl. J. Med. MAY 4 2023 388 18 1645 1656 10.1056/NEJMoa2212856
http://dx.doi.org/10.1056/NEJMoa2212856 12
Medicine, General & Internal Science Citation Index Expanded (SCI-EXPANDED) General & Internal Medicine M0OX1 37133584
Green Accepted, Green Published Y N 2025-06-24
WOS:001027210700008

J Guo, WM; Crossland, N; Crott, JW Guo, Weimin; Crossland, Nicholas; Crott, Jimmy W.

Mediterranean diet improves liver health but does not protect against azoxymethane-induced colon tumorigenesis compared to Western diet in A/J mice EXPERIMENTAL AND MOLECULAR PATHOLOGY English Article

Azoxymethane; Mediterranean diet; Colorectal cancer; Non-alcoholic fatty liver disease (NAFLD) COLORECTAL-CANCER; METABOLIC-ACTIVATION; CARCINOGENESIS; INFLAMMATION; CYP2E1; METHYLAZOXYMETHANOL; EXPRESSION; EXTRACTS; MODEL; RISK

Introduction: Abundant evidence indicates that the Mediterranean (MED) diet pattern is beneficial for health, especially cardiovascular health. Epidemiological evidence indicates that the MED diet also affords protection against colorectal cancer (CRC). To date, preclinical models have only evaluated specific MED diet components and therefore, although supportive, fall short of confirming the chemoprotective capacity of this complex dietary pattern. We sought to address this gap. Method: A/J mice were randomized to receive Western (WRN) or MED diets differing in their fat, protein, and carbohydrate sources. Azoxymethane (AOM) was used to initiate colon tumorigenesis and mice were maintained for 19 weeks after the final dose. Result: Unexpectedly high mortality was observed amongst male mice following the second AOM dose. At the end of the study hepatic Cyp2E1, an enzyme that metabolize AOM, was lower in males than females. Livers from MED diet mice were significantly lighter, had lower histologic Non-Alcoholic Fatty Liver Disease (NAFLD) scores,

and contained less triglycerides than WRN mice. Amongst females, serum alanine transaminase (ALT) was also lower in MED than WRN mice. Amongst male mice, those fed MED diet presented with significantly more colonic tumors than those on the WRN diet. Conclusion: In this study male mice displayed elevated sensitivity to AOM-induced hepatotoxicity and mortality than females. In agreement with human and preclinical data, livers of MED-diet-fed mice were healthier than those fed WRN diets. We could not confirm the chemoprotective capacity of the MED diet. Additional studies are required to evaluate the purported anticancer effect of the MED diet. [Guo, Weimin; Crossland, Nicholas; Crott, Jimmy W.] Boston Univ, Chobanian & Avedisian Sch Med, Dept Pathol & Lab Med, Boston, MA 02118 USA; [Crossland, Nicholas] Boston Univ, Comparat Pathol Lab, Natl Emerging Infect Dis Labs, Boston, MA USA; [Crossland, Nicholas] Boston Univ, Chobanian & Avedisian Sch Med, Dept Virol Immunol & Microbiol, Boston, MA USA Boston University; Boston University; Boston University Crott, JW (corresponding author), Boston Univ, Chobanian & Avedisian Sch Med, Dept Pathol & Lab Med, Boston, MA 02118 USA. jcrott@bu.edu USDA NIFA [2020-67017-37723]; NIH [S10OD030269] USDA NIFA(United States Department of Agriculture (USDA)); NIH(United States Department of Health & Human ServicesNational Institutes of Health (NIH) - USA) This study was funded in part by USDA NIFA grant #2020-67017-37723. This work utilized shared instrumentation funded by the NIH (S10OD030269) . 39 0 0 1 1 ACADEMIC PRESS INC ELSEVIER SCIENCE SAN DIEGO 525 B ST, STE 1900, SAN DIEGO, CA 92101-4495 USA 0014-4800 1096-0945 EXP MOL PATHOL Exp. Mol. Pathol. MAR 2025 141 104953 10.1016/j.yexmp.2025.104953 http://dx.doi.org/10.1016/j.yexmp.2025.104953 FEB 2025 7 Pathology Science Citation Index Expanded (SCI-EXPANDED) Pathology X4A9O 39919552 hybrid 2025-06-24 WOS:001424809400001

J Özçelik, B; Serin, IS; Gökahmetoglu, S; Basbug, M; Erez, R Özçelik, B; Serin, IS; Gökahmetoglu, S; Basbug, M; Erez, R Human papillomavirus frequency of women at-low risk of developing cervical cancer:: a preliminary study from a Turkish university hospital EUROPEAN JOURNAL OF GYNAECOLOGICAL ONCOLOGY English Article HPV frequency; low-risk women; Middle East Anatolia HYBRID CAPTURE; INFECTION; PREVALENCE Purpose: To investigate the frequency of human papillomavirus (HPV) infection among low-risk women for cervical cancer in our region. Methods: In one year period, 230 consecutive women at low risk of developing cervical cancer were enrolled to the study. HPV DNA testing was performed by Hybrid Capture-I System (HC-I) and groups were constituted by HPV-positive and HPV-negative women. A comparison of the groups according to age, obstetric history and age at the beginning of sexual intercourse was made. Statistical analysis was performed. Results: The frequency rate of HPV infection was demonstrated to be 6.1% (n = 14) in our study (5.9% in women less than or equal to 45 years and 7.7% in women > 45 years). Age-dependent differences were not observed between groups. There was no significant difference between HPV-positive and negative women regarding obstetric characteristics and mean age at first intercourse. Conclusion: This study provided significant information on the frequency of HPV infection of low-

risk women in our region. When considered with studies performed in other countries, our study may give some help on the natural history of HPV infection and cervical squamous lesions. Erciyes Univ, Tip Fak, Dept Obstet & Gynecol, TR-38039 Kayseri, Turkey; Erciyes Univ, Fac Med, Dept Microbiol, TR-38039 Kayseri, Turkey; Erciyes Univ, Fac Med, Dept Biostat, TR-38039 Kayseri, Turkey
Erciyes University; Erciyes University; Erciyes University
Özçelik, B (corresponding author), Erciyes Univ, Tip Fak, Dept Obstet & Gynecol, TR-38039 Kayseri, Turkey.
13 36 36 0 0 I R O G CANADA, INC MONTREAL 4900 COTE ST-LUC, APT#212, MONTREAL, QUEBEC H3W 2H3, CANADA 0392-2936 EUR J GYNAECOL ONCOL Eur. J. Gynaecol. Oncol. 2003 24 2
157 159 3 Oncology; Obstetrics & Gynecology Science Citation Index Expanded (SCI-EXPANDED) Oncology; Obstetrics & Gynecology 665CV 12701968
2025-06-24 WOS:000182102800013
J Saliba, W; Rennert, HS; Gronich, N; Gruber, SB; Rennert, G
Saliba, Walid; Rennert, Hedy S.; Gronich, Naomi; Gruber, Stephen B.; Rennert, Gad Red meat and processed meat intake and risk of colorectal cancer: a population-based case-control study EUROPEAN JOURNAL OF CANCER PREVENTION
English Article
beef; colorectal cancer; lamb; pork; processed meat; red meat CONSUMPTION; ASSOCIATIONS To examine the association between red meat subtypes intake and risk of colorectal cancer (CRC) among Jewish and Arabs populations in a unique Mediterranean environment. The Molecular Epidemiology of Colorectal Cancer study (n=10 026) is a prospective population-based case-control study in northern Israel. Participants were interviewed in-person about their dietary intake and lifestyle using a questionnaire that included a food-frequency questionnaire. Red meat consumption in Israel was found to be especially low in the Jewish population (1.29 +/- 1.45 servings/week), but higher in Arabs (3.0 +/- 1.98 servings/week) (P<0.001). Beef was the most commonly consumed red meat by Jews (1.15/1.29 servings/week, 89%) and proportionally less so by Arabs (2.00/3.00, 67%). Processed meat consumption (mostly pork free) was lower among Arabs (0.9 +/- 1.56 servings/week) compared with Jews (1.97 +/- 2.97 servings/week) (P<0.001). The adjusted odds of CRC per one serving/week of red meat were 1.05 (95% confidence interval: 1.01-1.08) in Jews and 0.94 (0.88-1.01) in Arabs. Compared with no consumption, beef consumption was associated with odds ratio (OR)=0.96 (0.86-1.07) in Jews and 0.94 (0.61-1.45) in Arabs, lamb consumption with OR=1.28 (1.10-1.5) and 1.01 (0.75-1.37), pork consumption with OR=1.44 (1.24-1.67) and 1.07 (0.73-1.56), and processed meat consumption with OR=1.22 (1.10-1.35) and 1.04 (0.82-1.33) in Jews and Arabs, respectively. Overall red meat consumption was associated weakly with CRC risk, significant only for lamb and pork, but not for beef, irrespective of tumor location. Processed meat was associated with mild CRC risk.[Saliba, Walid; Rennert, Hedy S.; Gronich, Naomi; Rennert, Gad] Technion Israel Inst Technol, Ruth & Bruce Rappaport Fac Med, Lady Davis Carmel Med Ctr, Dept Community Med & Epidemiol, Haifa, Israel; [Saliba, Walid; Rennert, Hedy S.; Gronich, Naomi; Rennert, Gad] Clalit Natl Canc Control Ctr, Haifa, Israel; [Gruber, Stephen B.] Univ Southern Calif, USC Norris Comprehens Canc Ctr, Los Angeles, CA USA; [Gruber, Stephen B.] Univ Southern Calif, Keck Sch Med, Dept

Prevent Med, Los Angeles, CA 90033 USA Clalit Health Services; Carmel Medical Center; Technion Israel Institute of Technology; Rappaport Faculty of Medicine; University of Southern California; University Southern California Hospital; University of Southern California Saliba, W (corresponding author), Carmel Hosp, Dept Community Med & Epidemiol, 7 Michal St, IL-34362 Haifa, Israel.
saliba_wa@clalit.org.il Rennert, Gad/0000-0002-8512-068X; Gronich, Naomi/0000-0003-4369-6813 NIH [R01 CA81488] NIH(United States Department of Health & Human ServicesNational Institutes of Health (NIH) - USA) This work was supported in part by NIH R01 CA81488. 21 15 16 0
44 LIPPINCOTT WILLIAMS & WILKINS PHILADELPHIA TWO COMMERCE SQ, 2001 MARKET ST, PHILADELPHIA, PA 19103 USA 0959-8278
1473-5709 EUR J CANCER PREV Eur. J. Cancer Prev.
JUL 2019 28 4 287 293
10.1097/CEJ.0000000000000451
http://dx.doi.org/10.1097/CEJ.0000000000000451
7 Oncology Science Citation Index Expanded (SCI-EXPANDED) Oncology II6AX 30640205 Green Accepted
2025-06-24 WOS:000475276600006
J Maliou, D; Belmadi, D; Saadi, W; Mahfouf, H; Benzidane, N; Bitam, A Maliou, Djamil; Belmadi, Dounia; Saadi, Wissem; Mahfouf, Hassen; Benzidane, Noureddine; Bitam, Arezki Effect of dairy products intake on breast cancer risk: A case-control study in Algeria NUTRITION CLINIQUE ET METABOLISME English Article
Breast cancer; Dairy products; Diet; Epidemiology
GROWTH-FACTOR-I; MILK-PRODUCTS; VITAMIN-D; MEDITERRANEAN DIET; CALCIUM INTAKE; FOOD; CONSUMPTION; NUTRITION; INSULIN; WOMEN
Objectives. The aim of this hospital-based case-control study is to evaluate the association between dairy products consumption and breast cancer risk. This relationship has not previously been studied in Algeria. Materials and methods. Our study was conducted on 184 breast cancer patients and equal number of age-matched controls. Data on sociodemographic characteristics, reproductive and menstrual history, medical history, lifestyle factors and anthropometric measurements were collected by interviewers. Dietary intake information was also obtained using a short food questionnaire. The Odds ratios [95% confidence interval (CI)] of breast cancer were estimated across categories of dairy intake using multivariable logistic regression. Results. We observed an increased breast cancer risk in women with higher consumption of milk [OR = 2.61, 95% CI: 1.32-5.16, P = 0.027] comparing with those in lower category of intake. An inverse association with breast cancer risk was observed for total cheese intake when comparing highest with lowest quartiles. The protective effect of cheese was confirmed only for fresh cheese. No significant association was found for other types of dairy foods. Conclusion. Our study demonstrates the differential effect of dairy products types on breast cancer risk. Future studies are warranted to confirm these results. (C) 2018 Association pour le developpement de la recherche en nutrition (ADREN). Published by Elsevier Masson SAS. All rights reserved. [Maliou, Djamil; Bitam, Arezki] Natl Higher Sch Agron ES06, Lab Human Nutr & Food Technol, Algiers 16131, Algeria; [Belmadi, Dounia] Univ Algiers, Fac Med, Mustapha Pacha Publ Hosp, Acad Med Periodontol Serv, Algiers 16014, Algeria; [Saadi, Wissem; Mahfouf, Hassen] Univ Algiers, Fac Med, Rouiba Publ Hosp, Acad Med Oncol Serv, Algiers

16012, Algeria; [Benzidane, Noureddine] Univ Algiers, Fac Med, Pierre & Marie Curie Ctr, Senol Serv, Algiers 16014, Algeria
Bitam, A (corresponding author), Natl Higher Sch Agron ES06, Lab Human Nutr & Food Technol, Algiers 16131, Algeria.
a.bitam@ensa.dz
Laboratory of Human Nutrition and food Technology of National Higher School of Agronomy (Algiers); Pierre and Marie Curie Center of Algiers; public hospital of Rouiba (Algiers); public hospital of Mustapha Pacha (Algiers) Laboratory of Human Nutrition and food Technology of National Higher School of Agronomy (Algiers); Pierre and Marie Curie Center of Algiers; public hospital of Rouiba (Algiers); public hospital of Mustapha Pacha (Algiers) This study was supported by: Laboratory of Human Nutrition and food Technology of National Higher School of Agronomy (Algiers), Pierre and Marie Curie Center of Algiers and public hospitals of Rouiba and Mustapha Pacha (Algiers). We would like to thank Pr. Malkia Meddad, Pr. Ferhat Zebboudj, Pr. Merzak Gharnaout, Dr Abdel Bassatketfi, Dr Nabil Bourahla and Dr. Mohammed Yehya Aissiou, for their assistance. We also thank Dr. Dounia Belmadi for her contribution to the data collection and all study participants.
54 4 4 0 16 MASSON EDITEUR
MOULINEAUX CEDEX 9 21 STREET CAMILLE DESMOULINS, ISSY, 92789 MOULINEAUX CEDEX 9, FRANCE 0985-0562 1768-3092 NUTR CLIN METAB Nutr. Clin. Metab. SEP 2018 32 3
187 194 10.1016/j.nupar.2018.04.001
http://dx.doi.org/10.1016/j.nupar.2018.04.001
8 Endocrinology & Metabolism; Nutrition & Dietetics
Science Citation Index Expanded (SCI-EXPANDED)
Endocrinology & Metabolism; Nutrition & Dietetics GX1MQ
2025-06-24 WOS:000447481800009
J Jacobs, S; Harmon, BE; Ollberding, NJ; Wilkens, LR; Monroe, KR; Kolonel, LN; Le Marchand, L; Boushey, CJ; Maskarinec, G
Jacobs, Simone; Harmon, Brook E.; Ollberding, Nicholas J.; Wilkens, Lynne R.; Monroe, Kristine R.; Kolonel, Laurence N.; Le Marchand, Loic; Boushey, Carol J.; Maskarinec, Gertraud Among 4 Diet Quality Indexes, Only the Alternate Mediterranean Diet Score Is Associated with Better Colorectal Cancer Survival and Only in African American Women in the Multiethnic Cohort JOURNAL OF NUTRITION English
Article colorectal cancer; nutrition; Healthy Eating Index; Alternative Healthy Eating Index; alternate Mediterranean Diet score; Dietary Approaches to Stop Hypertension index; dietary patterns; survival; Cox regression; Multiethnic Cohort COLON-CANCER; POSTMENOPAUSAL WOMEN; RISK; PATTERNS; MORTALITY; METAANALYSIS; ADHERENCE; DISEASE; WHITES; ANTIOXIDANTS Background: Colorectal cancer (CRC) is the second leading cause of cancer-related death in the United States, with a 5-y survival rate of similar to 65%. Therefore, the identification of modifiable health factors to improve CRC survival is crucial. Objective: We investigated the association of 4 prediagnostic a priori diet quality indexes with CRC-specific and all-cause mortality in the Multiethnic Cohort (MEC). Methods: The MEC included >215,000 African-American, Native Hawaiian, Japanese-American, Latino, and white adults living in Hawaii and California who completed a validated quantitative food-frequency questionnaire in 1993-1996. CRC cases and deaths were identified through linkages to cancer registries and to state and national vital registries. Sex specific HRs and 95% Cls were estimated for

the Healthy Eating Index (HEI) 2010, the Alternative HEI (AHEI) 2010, the alternate Mediterranean Diet (aMED) score, and the Dietary Approaches to Stop Hypertension (DASH) index with CRC-specific and overall mortality as the primary outcomes. Ethnicity-specific analyses were the secondary outcomes. Results: Among 4204 MEC participants diagnosed with invasive CRC through 2010, 1976 all-cause and 1095 CRC-specific deaths were identified. A higher aMED score was associated with lower CRC-specific mortality in women [HR continuous pattern score divided by its respective SD (HR1SD): 0.86; 95% CI: 0.77, 0.96] but not in men (HR1SD: 1.01; 95% CI: 0.92, 1.11). A higher aMED score was also associated with lower all-cause mortality in women (HR1SD: 0.88; 95% CI: 0.81, 0.96) but not in men (HR1SD: 1.00; 95% CI: 0.93, 1.07). The HEI-2010, AHEI-2010, and DASH index were not significantly associated with CRC-specific or with all-cause mortality. The inverse relation for the aMED score was limited to African Americans and to colon (compared with rectal) cancer. Conclusions: The aMED score was related to lower mortality only in African-American women (1 of 5 ethnic groups studied). The results should be interpreted with caution due to the small numbers of cases within ethnic groups and the issue of multiple testing. [Jacobs, Simone; Wilkens, Lynne R.; Kolonel, Laurence N.; Le Marchand, Loic; Boushey, Carol J.; Maskarinec, Gertraud] Univ Hawaii, Ctr Canc, Honolulu, HI 96822 USA; [Harmon, Brook E.] Univ Memphis, Sch Publ Hlth, Memphis, TN 38152 USA; [Ollberding, Nicholas J.] Cincinnati Childrens Hosp Med Ctr, Cincinnati, OH 45229 USA; [Monroe, Kristine R.] Univ Southern Calif, Hlth Sci Campus, Los Angeles, CA USA University of Hawaii System; Cancer Research Center of Hawaii; University of Memphis; Cincinnati Children's Hospital Medical Center; University of Southern California
Maskarinec, G (corresponding author), Univ Hawaii, Ctr Canc, Honolulu, HI 96822 USA. gertraud@cc.hawaii.edu Ollberding, Nicholas/N-8402-2015 Ollberding, Nicholas/0000-0001-9404-7368; Jacobs, Simone/0009-0008-3883-5202 National Cancer Institute (NCI) [U01CA164973]; German Research Foundation (DFG) [JA 2564/1-1]; NCI [N01 PC 35137, N01 PC 35139]; [R25CA90956] National Cancer Institute (NCI)(United States Department of Health & Human ServicesNational Institutes of Health (NIH) - USANIH National Cancer Institute (NCI)); German Research Foundation (DFG)(German Research Foundation (DFG)); NCI(United States Department of Health & Human ServicesNational Institutes of Health (NIH) - USANIH National Cancer Institute (NCI)); The Multiethnic Cohort Study is funded by grant U01CA164973 from the National Cancer Institute (NCI). BEH was supported by postdoctoral fellowships on grant R25CA90956. SJ was supported by a postdoctoral fellowship from the German Research Foundation (DFG, JA 2564/1-1). The tumor registries were supported by NCI contracts N01 PC 35137 and N01 PC 35139. 50 52 56 0 17 ELSEVIER SCIENCE INC NEW YORK STE 800, 230 PARK AVE, NEW YORK, NY 10169 USA
0022-3166 1541-6100 J NUTR J. Nutr. SEP 2016
146 9 1746 1755
10.3945/jn.116.234237
http://dx.doi.org/10.3945/jn.116.234237 10
Nutrition & Dietetics Science Citation Index Expanded (SCI-EXPANDED) Nutrition & Dietetics DU7VN 27511927 Green Published, Bronze 2025-06-24 WOS:000382422800017
J Allali, M; Boukhatem, N; Bouguenouch, L; Hardin, H; Boudouaya, HA; Cadenas, MB; Ouldim, K; Amzazi, S; Azcarate-Peril,

MA; Ghazal, H Allali, Mane; Boukhatem, Noureddine; Bouguenouch, Leila; Hardin, Hanaa; Abir Boudouaya, H.; Cadenas, M. Belen; Ouldim, Karim; Amzazi, Saaid; Azcarate-Peril, M. Andrea; Ghazal, Hassan Gut microbiome of Moroccan colorectal cancer patients MEDICAL MICROBIOLOGY AND IMMUNOLOGY English Article

Gut microbiome composition; Colorectal cancer; Bacterial community; 16S rRNA sequencing; Moroccan population

BACTEROIDES-FRAGILIS; PERIODONTAL-DISEASE; INTESTINAL MICROBIOTA; BACTERIAL METABOLITES; MEDITERRANEAN DIET; FECAL MICROBIOTA; LIFE-STYLE; COLON; FUSOBACTERIUM; RISK Although colorectal cancer is the third leading cause of death in Morocco, there are no studies of the microbiome changes associated with the disease in the Moroccan population. The aim of our study was to compare the stool microbiome of Moroccan cancer patients with healthy individuals. We analyzed the microbiome composition of samples from 11 CRC patients and 12 healthy individuals by 16S rRNA amplicon sequencing. Principal coordinate analysis of samples revealed defined cancer versus healthy clusters. Our findings showed that cancer samples had higher proportions of Firmicutes (T= 50.5%; N=28.4%; p = 0.04), specifically of Clostridia (T=48.3%; N= 19.0%; p = 0.002), and Fusobacteria (T=0.1%; N=0.0%; p= 0.02), especially of Fusobacteriia (T=0.1%; N=0.0%; p = 0.02), while Bacteroidetes were enriched in healthy samples (T=35.1%; N=62.8%; p = 0.06), particularly the class Bacteroidia (T=35.1%; N=62.6%; p = 0.06). Porphyromonas, Clostridium, Ruminococcus, Selenomonas, and Fusobacterium were significantly overrepresented in diseased patients, similarly to other studies. Predicted functional information showed that bacterial motility proteins, flagellar assembly, and fatty acid biosynthesis metabolism were significantly overrepresented in cancer patients, while amino acid metabolism and glycan biosynthesis were overrepresented in controls. This suggests that involvement of these functional metagenomes is similar and relevant in the carcinogenesis process, independent of the origin of the samples. Results from this study allowed identification of bacterial taxa relevant to the Moroccan population and encourages larger studies to facilitate population-directed therapeutic approaches. [Allali, Mane; Amzazi, Saaid] Mohammed V Univ Rabat, Fac Sci, Lab Biochem & Immunol, Rabat, Morocco; [Allali, Mane; Boukhatem, Noureddine; Hardin, Hanaa; Abir Boudouaya, H.; Ghazal, Hassan] Univ Mohammed Premier, Fac Sci Oujda, Lab Physiol Genet & Ethnopharmacol, Oujda, Morocco; [Ghazal, Hassan] Univ Mohammed Premier, Polydisciplinary Fac Nador, Nador, Morocco; [Bouguenouch, Leila; Ouldim, Karim] Univ Hosp Hassan II Fez, Dept Mol Genet, Fes, Morocco; [Allali, Mane; Cadenas, M. Belen; Azcarate-Peril, M. Andrea] Univ N Carolina, Sch Med, Dept Med, Chapel Hill, NC 27515 USA; [Allali, Mane; Cadenas, M. Belen; Azcarate-Peril, M. Andrea] Univ N Carolina, Sch Med, Microbiome Core Facil, Chapel Hill, NC 27515 USA; [Ghazal, Hassan] Natl Ctr Sci & Technolg Res, Rabat, Morocco Mohammed V University in Rabat; Mohammed First University of Oujda; Mohammed First University of Oujda; Sidi Mohamed Ben Abdellah University of Fez; Hassan II University Hospital Center of Fez; University of North Carolina; University of North Carolina Chapel Hill; University of North Carolina School of Medicine; University of North Carolina; University of North Carolina Chapel Hill; University of North Carolina School of Medicine Ghazal, H (corresponding author), Univ Mohammed Premier, Fac Sci Oujda, Lab Physiol Genet &

Ethnopharmacol, Oujda, Morocco.; Ghazal, H (corresponding author), Univ Mohammed Premier, Polydisciplinary Fac Nador, Nador, Morocco.; Ghazal, H (corresponding author), Natl Ctr Sci & Technolg Res, Rabat, Morocco. hassan.ghazal@fulbrightmail.org
Azcarate-Peril, M./ABH-5508-2020; Ghazal, Hassan/AAS-1090-2021 Ghazal, Hassan/0000-0002-0638-2180; bouguenouch, laila/0000-0002-4082-5432 NIH [U41HG006941] NIH(United States Department of Health & Human ServicesNational Institutes of Health (NIH) - USA) IA is a Fulbright scholar. This work was also supported by a Grant from the NIH for H3ABioNet/H3Africa to H.G (grant number U41HG006941). 122 53 60 0 21
SPRINGER NEW YORK ONE NEW YORK PLAZA, SUITE 4600, NEW YORK, NY, UNITED STATES 0300-8584 1432-1831 MED MICROBIOL IMMUN Med. Microbiol. Immunol. AUG 2018 207 3-4
211 225 10.1007/s00430-018-0542-5 http://dx.doi.org/10.1007/s00430-018-0542-5
15 Immunology; Microbiology Science Citation Index Expanded (SCI-EXPANDED) Immunology; Microbiology GO9HA
29687353 Green Published, hybrid 2025-06-24 WOS:000440415700006
J Ebeid, SA; Abd El Moneim, NA; Ghoneim, HEM; El-Benhawy, SA; Ismail, SE Ebeid, Samia A.; Abd El Moneim, Nadia A.; Ghoneim, Hossam El-Din M.; El-Benhawy, Sanaa A.; Ismail, Samah E. Combination of Doxorubicin and Berberine Generated Synergistic Anticancer Effect on Breast Cancer Cells Through Down-regulation of Nanog and miRNA-21 Gene Expression
MIDDLE EAST JOURNAL OF CANCER English
Article Breast cancer; Nanog; miRNA-21; Doxorubicin; Berberine SIDE POPULATION; MIR-21; PROLIFERATION; SENSITIVITY; DIMINISHES; RESISTANCE; CISPLATIN; APOPTOSIS Background: Our purpose was to investigate the effect of berberine (BER) and doxorubicin (DOX) on the expression of stem cell markers Nanog and microRNA-21 in MCF-7 cells. Methods: The study was an in vitro study employing the human breast cancer cell line MCF-7 that was divided into four groups: Group MCF-7 cell line maintained in drug-free environment as untreated control, Group 11: MCF-7 cell line treated with different concentrations of DOX, Group III: MCF-7 cell line treated with various concentrations of BER. Group IV: MCF-7 cell line treated with different concentrations of combined DOX and BER. MTT assay determined the metabolic activity and viability of MCF-7 cells for all groups. We further extracted total RNA from MCF-7 cells, and RT-PCR assayed the expression of Nanog and miRNA-21. Results: The results revealed that DOX and/or BER decreased the percentage of viable MCF-7 monolayer and mammospheres breast cancer cells in a concentration-dependent manner. Moreover, the combination of DOX and BER generated synergistic anticancer effect on MCF-7 monolayer cells and mammospheres. In addition, DOX alone, BER alone, and their combination significantly reduced Nanog and miRNA-21 gene expression in MCF-7 mammospheres compared with untreated mammospheres. Conclusions: BER may affect the viability of breast cancer cells through downregulation of Nanog and miRNA-21 gene expression, ultimately enhancing the sensitivity of breast cancer cell line to DOX. BER may be an effective chemotherapeutic agent against breast cancer where the combination of DOX and BER generates synergistic anticancer effects. [Ebeid, Samia A.; Ismail, Samah E.] Alexandria Univ, Med Res Inst, Dept Appl Med Chem, Alexandria, Egypt; [Abd El Moneim, Nadia A.] Alexandria

Univ, Med Res Inst, Dept Canc Management & Res, Alexandria, Egypt; [Ghoneim, Hossam El-Din M.] Alexandria Univ, Med Res Inst, Dept Immunol & Allergy, Alexandria, Egypt; [El-Benhawy, Sanaa A.] Alexandria Univ, Med Res Inst, Dept Radiat Sci, Alexandria, Egypt
Egyptian Knowledge Bank (EKB); Alexandria University; Egyptian Knowledge Bank (EKB); Alexandria University; Egyptian Knowledge Bank (EKB); Alexandria University; Egyptian Knowledge Bank (EKB); Alexandria University El-Benhawy, SA (corresponding author), 165 El Horreya Ave, Alexandria, Egypt.
dr_sanaa_ali13@yahoo.com
39 6 6 4 13 SHIRAZ UNIV MEDICAL SCIENCES
SHIRAZ NEMAZEE HOSPITAL, SHIRAZ, 71934, IRAN 2008-6709
2008-6687 MIDDLE EAST J CANCER Middle East J. Cancer
JUL 2020 11 3 273 285
10.30476/mejc.2019.81277.0
http://dx.doi.org/10.30476/mejc.2019.81277.0 13
Oncology Emerging Sources Citation Index (ESCI) Oncology
ML5EZ 2025-06-24 WOS:000549490400004
J Abdelaziz, LA; Harb, OA; Abdelbary, AM; Mohammed, AA; Elkalla, HMHR Abdelaziz, Lobna A.; Harb, Ola A.; Abdelbary, Abeer M.; Mohammed, Amrallah A.; Elkalla, Hend M. Hamdey Rashed The Prognostic Significance of ALDH-1 and SOX9 Expression in Early Breast Cancer MIDDLE EAST JOURNAL OF CANCER English Article
Egypt; Early breast cancer; Neoplastic stem cells; Aldehyde dehydrogenase 1; SOX9; Prognosis ALDEHYDE DEHYDROGENASE 1; POOR-PROGNOSIS; MARKER; PROLIFERATION; PREDICTOR; CARCINOMA; ESTROGEN; CELLS Background: Aldehyde dehydrogenase 1 (ALDH1) is an enzyme accountable for the detoxification of aldehydes. Sex-determining region Y-box 9 (SOX-9) plays a role in many biological and pathological processes. In this study, we aimed to evaluate the prognostic significance of ALDH1 and SOX9 expression in early breast cancer. Method: The expression of ALDH1 and SOX-9 was evaluated through immuno-histochemistry derived from 50 eligible patients with early breast cancer included in the current prospective cohort study. Results: Positive expression of ALDH1 and SOX-9 were detected in 29 (58%) and 34 (68%) patients, respectively. The positive expressions of both markers were statistically significant associated with increasing the stage, lymph nodes metastasis, high Ki67 labeling index, and molecular subtypes (P < 0.001), along with with the biological markers; estrogen receptors, progesterone receptors, and human epidermal growth factor receptor 2 over-expressions, and large tumor size (P = 0.039, P = 0.022, P = 0.024 and P = 0.003 for ALDH1 expression and P = 0.012, P = 0.007, P = 0.004 , and P = 0.002 for SOX-9 expression, respectively). There is a significant positive association between the expression of ALDH1 and SOX-9, r (correlation coefficient) = +0.806 (P < 0.001). Local recurrence was associated with the positive expression of ALDH1 only (P = 0.045) and the disease progression was statistically significant and associated with the positive expression of both ALDH1 and SOX-9 (P = 0.038, P = 0.023, respectively). There was significant association of positive expression of SOX-9 with reduced 3-y disease-free survival (P = 0.039). Conclusion: Positive expression of ALDH-1 and SOX9 were associated with aggressive histopathological features and poor outcome in early breast cancer and can be considered potential prognostic markers in this group of patients. [Abdelaziz, Lobna A.] Zagazig Univ, Fac Med, Dept

Clin Oncol, Zagazig, Egypt; [Harb, Ola A.; Abdelbary, Abeer M.] Zagazig Univ, Fac Med, Dept Pathol, Zagazig, Egypt; [Mohammed, Amrallah A.] Zagazig Univ, Fac Med, Dept Med Oncol, Zagazig, Egypt; [Elkalla, Hend M. Hamdey Rashed] Mansoura Univ, Dept Clin Oncol & Nucl Med, Fac Med, Mansoura, Egypt Egyptian Knowledge Bank (EKB); Zagazig University; Egyptian Knowledge Bank (EKB); Zagazig University; Egyptian Knowledge Bank (EKB); Zagazig University; Egyptian Knowledge Bank (EKB); Mansoura University
Abdelaziz, LA (corresponding author), Zagazig Univ, Fac Med, Dept Clin Oncol, Zagazig, Egypt. mmlobna90@gmail.com Harb, Ola/AAV-1316-2020; Elkalla, Hend/H-8326-2017 Elkalla, Hend/0000-0002-6124-7379 40 0 0 0 2
SHIRAZ UNIV MEDICAL SCIENCES SHIRAZ NEMAZEE HOSPITAL, SHIRAZ, 71934, IRAN 2008-6709 2008-6687 MIDDLE EAST J CANCER Middle East J. Cancer OCT 2022 13 4
581 592 10.1016/annonc/annonc267
http://dx.doi.org/10.1016/annonc/annonc267 12
Oncology Emerging Sources Citation Index (ESCI) Oncology
5E9NW 2025-06-24 WOS:000865949200003
J El Badawy, NA; El-Sheredy, HG; Fadali, GA; Kazem, AH
El Badawy, Nehal Ahmed; El-Sheredy, Heba Gaber; Fadali, Geylan Abd Elshafy; Kazem, Amani Hussein
Androgen Receptor Expression in Triple-negative Breast Cancer and its Relation with Epidermal Growth Factor Receptor, CD 105, and Clinicopathological Parameters MIDDLE EAST JOURNAL OF CANCER English Article
Breast cancer; Androgen receptors; Immunohistochemistry BASAL-LIKE; MONOCLONAL-ANTIBODY; MOLECULAR CHARACTERIZATION; MICROVESSEL DENSITY; PHASE-II; EGFR; CARCINOMAS; MARKERS; FEATURES; TUMORS Background: Triple-negative breast cancers (TNBC) are the tumors lacking expression of estrogen receptors, progesterone receptors, and human epidermal growth factor 2. The highest level of androgen receptors (AR) expression belongs to the Luminal androgen receptor subtype. AR is expressed in 70 to 90% of primary breast cancers. The biological role of AR in breast cancer continues to emerge. The overexpression of epidermal growth factor receptor (EGFR) has been previously studied in TNBC, where it was found to be associated with poor prognosis. In the evaluation of neovascularization, CD105 (endoglin) was found to be superior to CD34 and CD31 owing to its greater affinity for endothelial cells in tumor-related angiogenic tissue. We conducted the present work to assess the expression profile of androgen receptor in TNBC cases and its correlation with other clinicopathological parameters, EGFR and CD 105, in order to evaluate its clinical significance. Method: This retrospective study included 50 histologically confirmed breast cancer patients who were proven to be triple-negative based on immunohistochemical study. Formalin-fixed tissue blocks with tumor were chosen for immunohistochemical staining for AR, EGFR, CD105, and Ki 67. Results: Positive AR expression was associated with older age, postmenopausal status, negative nodes, and grade II tumors. AR was inversely correlated with EGFR, while there was no correlation between AR and both Endoglin and Ki 67. Conclusion: AR-positive TNBC may be a subtype of breast cancer with unique characteristics that could make it ideal for antiandrogen endocrine therapy. EGFR and Endoglin's distinct expression indicated that they might be unique biomarkers for targeted therapy and prognosis. [El Badawy, Nehal Ahmed; Fadali, Geylan Abd

Elshafy; Kazem, Amani Hussein] Alexandria Univ, Med Res Inst, Dept Pathol, Alexandria, Egypt; [El-Sheredy, Heba Gaber] Alexandria Univ, Med Res Inst, Dept Canc Management & Res, Alexandria, Egypt
Egyptian Knowledge Bank (EKB); Alexandria University; Egyptian Knowledge Bank (EKB); Alexandria University El-Sheredy, HG (corresponding author), Alexandria Univ, Med Res Inst, Dept Canc Management & Res, Alexandria, Egypt.
heba.gaber99@yahoo.com elsheredy, heba/Q-9339-2019
Elsheredy, Heba/0000-0001-8270-7330
45 1 1 0 1 SHIRAZ UNIV MEDICAL SCIENCES
SHIRAZ NEMAZEE HOSPITAL, SHIRAZ, 71934, IRAN 2008-6709
2008-6687 MIDDLE EAST J CANCER Middle East J. Cancer
JUL 2021 12 3 368 376
10.30476/mejc.2021.84138.1202
http://dx.doi.org/10.30476/mejc.2021.84138.1202
9 Oncology Emerging Sources Citation Index (ESCI)
Oncology UD5WW 2025-06-24
WOS:000687277900006

J Mehrabani-Khasraghi, S; Khalily, F; Ameli, M
Mehrabani-Khasraghi, Sahar; Khalily, Farzad; Ameli, Mitra
Analysis of Colorectal Cancer and Polyps for the Presence of Herpes Simplex Virus and Epstein - Barr virus DNA Sequences by Polymerase Chain Reaction MIDDLE EAST JOURNAL OF CANCER English Article
Colorectal cancer; Polyp; Herpes simplex virus; Epstein-Barr virus; Polymerase chain reaction
CYTOMEGALOVIRUS; ADENOCARCINOMA; CARCINOMA Background: Colorectal cancer is one of the most common malignancies worldwide with more than one million new cases diagnosed each year. The aim of this study is to investigate the prevalence of herpes simplex virus and Epstein-Barr virus in patients with colorectal carcinomas and polyps in comparison with healthy subjects by using the polymerase chain reaction technique. Methods: In this analytical case-control study, we selected 15 patients with colorectal cancer, 20 patients with colorectal polyps and 35 patients without malignancy as controls. Biopsy specimens were frozen under sterile conditions at -20 degrees C. After DNA extraction, analysis of polymerase chain reaction to detect herpes simplex virus and Epstein-Barr virus DNA in tissue samples was performed. Statistical analysis was performed with the chi 2 test. Results: We observed herpes simplex DNA in 33.3% of tumor samples (5 of 15) and 20% from the non-malignant control group (7 of 35). There was no herpes simplex DNA in the polyp tissues (0 of 20). Epstein-Barr DNA was found in 60% of tumor samples (9 of 15), 35% of polyp samples (7 of 20), and 40% of the non-malignant control group (14 of 35). Statistical analysis showed no significant association between the prevalence of herpes simplex and Epstein-Barr viruses and the incidence of colorectal cancer and polyps compared with the control group. Conclusion: The results demonstrate a lack of direct molecular evidence to support an association between herpes simplex and Epstein-Barr viruses with human colorectal malignancies. These results do not exclude a possible oncogenic role of these viruses to infect different colon cells. [Mehrabani-Khasraghi, Sahar] Islamic Azad Univ, Tonekabon Branch, Dept Microbiol, Mazandaran, Iran; [Khalily, Farzad] Karaj Univ Med Sci & Hlth Serv, Gastroenterol & Hepatol Res Ctr, Alborz, Iran; [Ameli, Mitra] Islamic Azad Univ, Tonekabon Branch, Dept Med, Mazandaran, Iran Islamic Azad University;

Islamic Azad University	Mehrabani-Khasraghi, S (corresponding author), Islamic Azad Univ, Tonekabon Branch, Dept Microbiol, Mazandaran, Iran.	saharmehrabani1@gmail.com
24	0	0	1	3	SHIRAZ UNIV MEDICAL SCIENCES SHIRAZ	NEMAZEE HOSPITAL, SHIRAZ, 71934, IRAN
2008-6709	2008-6687	MIDDLE EAST J CANCER	Middle East J. Cancer	OCT	2015	6	4	211
218	8	Oncology	Emerging Sources Citation Index (ESCI)	Oncology	DR0LS
2025-06-24	WOS:000379599600002
J	Arizpe, A; Navarro, S; Ochoa-Dominguez, CY; Rodriguez, C; Kim, SE; Farias, AJ	Arizpe, Angel; Navarro, Stephanie; Ochoa-Dominguez, Carol Y.; Rodriguez, Claudia; Kim, Sue E.; Farias, Albert J.	Nativity differences in socioeconomic barriers and healthcare delays among cancer survivors in the All of Us cohort	CANCER CAUSES & CONTROL
English	Article	SES barriers; Healthcare delays; Nativity; All of Us; Cancer survivors; Health literacy	LIFE-STYLE FACTORS; PHYSICAL-ACTIVITY; MEDITERRANEAN DIET; STAGE-III; QUESTIONNAIRE; VALIDITY; RISK; ASSOCIATION; RECURRENCE; PREVENTION	PurposeWe aimed to assess whether nativity differences in socioeconomic (SES) barriers and health literacy were associated with healthcare delays among US cancer survivors.Methods"All of Us" survey data were analyzed among adult participants ever diagnosed with cancer. A binary measure of healthcare delay (1+ delays versus no delays) was created. Health literacy was assessed using the Brief Health Literacy Screen. A composite measure of SES barriers (education, employment, housing, income, and insurance statuses) was created as 0, 1, 2, or 3+. Multivariable logistic regression model tested the associations of (1) SES barriers and health literacy with healthcare delays, and (2) whether nativity modified this relationship.ResultsMedian participant age was 64 years (n = 10,020), with 8% foreign-born and 18% ethnic minorities. Compared to survivors with no SES barriers, those with 3+ had higher likelihood of experiencing healthcare delays (OR 2.18, 95% CI 1.84, 2.58). For every additional barrier, the odds of healthcare delays were greater among foreign-born (1.72, 1.43, 2.08) than US-born (1.27, 1.21, 1.34). For every 1-unit increase in health literacy among US-born, the odds of healthcare delay decreased by 9% (0.91, 0.89, 0.94).ConclusionWe found that SES barriers to healthcare delays have a greater impact among foreign-born than US-born cancer survivors. Higher health literacy may mitigate healthcare delays among US cancer survivors. Healthcare providers, systems and policymakers should assess and address social determinants of health and promote health literacy as a way to minimize healthcare delays among both foreign- and US-born cancer survivors.	[Arizpe, Angel; Navarro, Stephanie; Kim, Sue E.; Farias, Albert J.] Univ Southern Calif, Keck Sch Med, 2001 N Soto St,Suite 318B, Los Angeles, CA 90032 USA; [Ochoa-Dominguez, Carol Y.] Univ Calif San Diego, San Diego, CA 92103 USA; [Rodriguez, Claudia] Univ Southern Calif, Los Angeles, CA 90007 USA
University of Southern California; University of California System; University of California San Diego; University of Southern California	Farias, AJ (corresponding author), Univ Southern Calif, Keck Sch Med, 2001 N Soto St,Suite 318B, Los Angeles, CA 90032 USA.	aarizpe@usc.edu; stephaan@usc.edu; cyochoa@health.ucsd.edu; cbr96380@usc.edu; sueekim@usc.edu;

albertfa@usc.edu Farias, Albert/N-8486-2019; Navarro, Stephanie/U-8234-2018; Solorzano, Claudia/G-8817-2011; Kim, Sue/LSK-7178-2024; Farias, Albert/C-3979-2017 Arizpe, Angel/0000-0002-4075-2675; Ochoa-Dominguez, Carol/0000-0002-6946-3287; Farias, Albert/0000-0002-6463-7831 National Cancer Institute National Cancer Institute(United States Department of Health & Human ServicesNational Institutes of Health (NIH) - USANIH National Cancer Institute (NCI)) The authors would like to thank all of the participants of the All of Us Research Program, Drs. Cecilia Patino-Sutton, and Elizabeth Burner. The All of Us Research Program is supported by the National Institutes of Health, Office of the Director: Regional Medical Centers: 1 OT2 OD026549; 1 OT2 OD026554; 1 OT2 OD026557; 1 OT2 OD026556; 1 OT2 OD026550; 1 OT2 OD 026552; 1 OT2 OD026553; 1 OT2 OD026548; 1 OT2 OD026551; 1 OT2 OD026555; IAA #: AOD 16037; Federally Qualified Health Centers: HHSN 263201600085U; Data and Research Center: 5 U2C OD023196; Biobank: 1 U24 OD023121; The Participant Center: U24 OD023176; Participant Technology Systems Center: 1 U24 OD023163; Communications and Engagement: 3 OT2 OD023205; 3 OT2 OD023206; and Community Partners: 1 OT2 OD025277; 3 OT2 OD025315; 1 OT2 OD025337; 1 OT2 OD025276. In addition, the All of Us Research Program would not be possible without the partnership of its participants. 49 5 5 0 2 SPRINGER DORDRECHT VAN GODEWIJCKSTRAAT 30, 3311 GZ DORDRECHT, NETHERLANDS 0957-5243 1573-7225 CANCER CAUSE CONTROL Cancer Causes Control FEB 2024 35 2 203 214 10.1007/s10552-023-01782-z http://dx.doi.org/10.1007/s10552-023-01782-z SEP 2023 12 Oncology; Public, Environmental & Occupational Health Science Citation Index Expanded (SCI-EXPANDED) Oncology; Public, Environmental & Occupational Health ES5Z6 37679534 hybrid 2025-06-24 WOS:001063944100002
J Maher, E; Gedawy, G; Fathy, W; Farouk, S; Abd El Maksoud, A; Guirgis, AA; Khalil, H Maher, Ehab; Gedawy, Gamalat; Fathy, Walid; Farouk, Sabah; Abd El Maksoud, Ahmed; Guirgis, Adel A.; Khalil, Hany Hsa-miR-21-mediated Cell Death and Tumor Metastases: A Potential Dual Response During Colorectal Cancer Development MIDDLE EAST JOURNAL OF CANCER English Article Colorectal cancer; Hsa-miR-21; Dual response EXPRESSION; RISK Background: Colorectal cancer (CRC), caused by abnormal cells growing in the colon or rectum, has a high mortality rate worldwide. On the other hand, microRNAs are small non-coding RNAs that contain approximately 22 nucleotides in length. They are upregulated in a wide range of human cancers such as CRC. MiRNA-21 post-transcriptionally regulates the expression of many tumor suppressor genes such as P53 gene. This indicates that miRNA-21 interacts like oncogenes and is required for CRC development. Method: The current original study was conducted in the National Liver Institute, Menofyia University, Egypt. We collected a total of 40 blood samples from CRC patients 40 samples from healthy individuals who served as controls. Quantitative real-time PCR detected the levels of miRNA-21 and the fold changes of phosphates-tensin homology (PTEN) gene expression, as a tumor suppressor gene, in blood samples. Results: The expression levels of miR-21 were upregulated in all obtained samples from patients with CRC in association with aging, gender, and tumor-node-metastasis staging. Furthermore, patients with poor and well-

differentiated CRC revealed reduced levels of PTEN gene expression. We observed a putative binding site of miR-21 in PTEN gene sequences. This indicates the direct cleavage between miR-21 and PTEN coding sequence. Prediction analysis for other potential targets identified several malignancy factors and tumor suppressor genes with putative seeding regions for miR-21 such as STAT3, transforming growth factor-beta, tumor necrosis factor-alpha (TNF-alpha), and programmed cell death CD4. Conclusion: The current data exhibited the potential dual role of hsa-miR-21 in regulating cancer progression and showed that hsa-miR-21 is an efficacious biomarker for CRC development and an attractive candidate for CRC treatment during early transformation. [Maher, Ehab; Farouk, Sabah; Guirgis, Adel A.; Khalil, Hany] Univ Sadat City, Genet Engn & Biotechnol Res Inst, Dept Mol Biol, Sadat City, Egypt; [Gedawy, Gamalat] Menofyia Univ, Natl Liver Inst, Dept Clin Biochem & Mol Diagnost, Sebin El Kom, Egypt; [Fathy, Walid] Menofyia Univ, Fac Med, Dept Clin Pathol, Sebin El Kom, Egypt; [Abd El Maksoud, Ahmed] Univ Sadat City, Genet Engn & Biotechnol Res Inst, Ind Biotechnol Dept, Sadat City, Egypt Egyptian Knowledge Bank (EKB); University of Sadat City; Egyptian Knowledge Bank (EKB); Menofia University; Egyptian Knowledge Bank (EKB); Menofia University; Egyptian Knowledge Bank (EKB); University of Sadat City Khalil, H (corresponding author), Univ Sadat City, Genet Engn & Biotechnol Res Inst, POB 32897, Sadat City, Egypt.
hkhalil74@gmail.com Abdelmaksoud, Ahmed/B-3651-2011; Guirgis, Adel/ABF-9899-2021; Khalil, Hany Hamed Esmail/J-7700-2017
Khalil, Hany Hamed Esmail/0000-0001-9738-4087; Guirgis, Adel A/0009-0001-9952-6920 29 10 10 0
4 SHIRAZ UNIV MEDICAL SCIENCESSHIRAZ NEMAZEE HOSPITAL, SHIRAZ, 71934, IRAN 2008-6709 2008-6687
MIDDLE EAST J CANCER Middle East J. Cancer FAL 2020 11
4 483 492
10.30476/mejc.2020.83146.1139
http://dx.doi.org/10.30476/mejc.2020.83146.1139
10 Oncology Emerging Sources Citation Index (ESCI)
Oncology OF0MM 2025-06-24
WOS:000580913500012
J Laso, N; Mas, S; Lafuente, MJ; Casterad, X; Trias, M; Ballesta, A; Molina, R; Salas, J; Ascaso, C; Zheng, SC; Wiencke, JK; Lafuente, A Laso, N; Mas, S; Lafuente, MJ; Casterad, X; Trias, M; Ballesta, A; Molina, R; Salas, J; Ascaso, C; Zheng, SC; Wiencke, JK; Lafuente, A Decrease in specific micronutrient intake in colorectal cancer patients with tumors presenting Ki-ras mutation ANTICANCER RESEARCH
English Article
colorectal cancer risk; Ki-ras; diet; vitamin A; thiamine; vitamin D; iron COLON-CANCER; PROGNOSTIC VALUE; GENE-MUTATIONS; DEPENDENT RISK; SEQUENCE; PROTOONCOGENE; EPIDEMIOLOGY; RETINOIDS; CARCINOMA; ADENOMAS Background: The diversity of the Mediterranean diet and the heterogeneity of acquired genetic alterations in colorectal cancer (CRC) led us to examine the possible association between dietary factors and mutations, such as Ki-ras mutations, in genes implicated in the pathogenesis of these neoplasms. Patients and Methods: The study was based on 246 cases and 296 controls. For the molecular study only 117 patients with Ki-ras tumor expression were included. Dietary patterns were assessed using a semi-quantitative food frequency questionnaire. Results: Patients with Ki-ras mutations in codon 12 (K12) consumed

significantly less vitamin A (p = 0.02), B1 (p = 0.01), D (p = 0.02) and iron (p = 0.03) than controls, whereas patients without these mutations had similar intakes of these nutrients to controls. The consumption of fiber, folate, vitamin E and potassium was lower in the two subgroups of patients (K12-positive or -negative) than in controls. Mutation in codon 13 was not associated with any nutrient deficit. Conclusion: These results support previous findings that certain micronutrients protect against colorectal neoplasia and emphasize the importance of considering the different molecular forms of CRC as etiologically distinct diseases. Univ Barcelona, Sch Med, Dept Pharmacol, IDIBAPS, Barcelona 08036, Spain; Univ Barcelona, Sch Med, Dept Stat, IDIBAPS, Barcelona 08036, Spain; Hosp Santa Creu & Sant Pau, Dept Surg, Barcelona 08025, Spain; Hosp Clin Barcelona, Clin Chem Dept, Barcelona 08036, Spain; Univ Rovira & Virgili, Sch Med, Nutr Unit, Reus, Spain; Univ Calif San Francisco, Sch Med, Dept Epidemiol & Biostat, Lab Mol Epidemiol, San Francisco, CA 94143 USA University of Barcelona; Hospital Clinic de Barcelona; IDIBAPS; University of Barcelona; Hospital Clinic de Barcelona; IDIBAPS; Hospital of Santa Creu i Sant Pau; University of Barcelona; Hospital Clinic de Barcelona; Universitat Rovira i Virgili; University of California System; University of California San Francisco Univ Barcelona, Sch Med, Dept Pharmacol, IDIBAPS, Casanova 143, Barcelona 08036, Spain. amalia@medicina.ub.es ; Salas-Salvado, Jordi/C-7229-2017; Mas, Sergi/AAD-1996-2019
Ascaso Terren, Carlos/0000-0002-4895-7616; Salas-Salvado, Jordi/0000-0003-2700-7459; Mas, Sergi/0000-0003-3336-6298
NIEHS NIH HHS [ES 06717, ES 04704] Funding Source: Medline
NIEHS NIH HHS(United States Department of Health & Human ServicesNational Institutes of Health (NIH) - USANIH National Institute of Environmental Health Sciences (NIEHS))
47 12 14 0 2 INT INST ANTICANCER RESEARCH ATHENS EDITORIAL OFFICE 1ST KM KAPANDRITIOU-KALAMOU RD KAPANDRITI, PO BOX 22, ATHENS 19014, GREECE 0250-7005 1791-7530 ANTICANCER RES Anticancer Res. MAY-JUN 2004 24 3B 2011 2020
10 Oncology Science Citation Index Expanded (SCI-EXPANDED) Oncology 839AZ 15274393 2025-06-24 WOS:000222757000031
J Kim, EHJ; Willett, WC; Fung, T; Rosner, B; Holmes, MD
Kim, Esther H. J.; Willett, Walter C.; Fung, Teresa; Rosner, Bernard; Holmes, Michelle D. Diet Quality Indices and Postmenopausal Breast Cancer Survival
NUTRITION AND CANCER-AN INTERNATIONAL JOURNAL
English Article
FOOD-FREQUENCY QUESTIONNAIRE; CORONARY-HEART-DISEASE; NATIONAL DEATH INDEX; LIFE-STYLE; MEDITERRANEAN DIET; PROSPECTIVE COHORT; WEIGHT-GAIN; WOMEN; DIAGNOSIS; MORTALITY Research on diet in breast cancer survival has been focused on single nutrients or foods, particularly dietary fat, fruits, vegetables, fiber, and alcohol. We hypothesized that diet quality indices decrease the risk of total and non-breast-cancer-related deaths in women diagnosed with breast cancer. We evaluated 4 dietary quality scores: Alternate Healthy Eating Index (AHEI), Diet Quality IndexRevised (DQIR), Recommended Food Score (RFS), and the alternate Mediterranean Diet Score (aMED), among 2,729 women from the Nurses' Health Study with invasive Stage 1-3 breast cancer diagnosed between 1978 and 1998 with follow-up through 2004. In

multivariate adjusted analyses, no association was found between diet quality indices and either total or non-breast-cancer-related deaths. However, a higher aMED score was associated with a lower risk of non-breast-cancer death in women with low physical activity; the RR comparing the highest to lowest tertile was 0.39 (95% CI, 0.20-0.75, P trend = 0.0004). Our results suggest that a higher-quality diet after breast cancer diagnosis does not considerably change the risk of death from breast cancer. However, healthy dietary choices may be important because women are at risk of death from non-breast-cancer-related causes affected by diet.
[Kim, Esther H. J.] Harvard Univ, Sch Publ Hlth, Dept Nutr, Boston, MA 02115 USA; [Willett, Walter C.; Rosner, Bernard; Holmes, Michelle D.] Brigham & Womens Hosp, Dept Med, Channing Lab, Boston, MA 02115 USA; [Willett, Walter C.; Rosner, Bernard; Holmes, Michelle D.] Harvard Univ, Sch Med, Boston, MA USA; [Fung, Teresa] Simmons Coll, Boston, MA 02115 USA Harvard University; Harvard T.H. Chan School of Public Health; Harvard University; Harvard University Medical Affiliates; Brigham & Women's Hospital; Harvard University; Harvard Medical School; Simmons University
Kim, EHJ (corresponding author), Harvard Univ, Sch Publ Hlth, Dept Nutr, 665 Huntington Ave, Boston, MA 02115 USA.
ehjkim@post.harvard.edu Holmes, Michelle/GLS-8692-2022; Willett, Walter/E-2352-2013
National Institutes of Health [CA 87969] National Institutes of Health(United States Department of Health & Human ServicesNational Institutes of Health (NIH) - USA) We wish to acknowledge the invaluable assistance of Dr. Diane Feskanich. This study was supported by the National Institutes of Health (CA 87969). 35 82 90 1 11
LAWRENCE ERLBAUM ASSOC INC-TAYLOR & FRANCIS PHILADELPHIA
325 CHESTNUT STREET, STE 800, PHILADELPHIA, PA 19106 USA
0163-5581 NUTR CANCER Nutr. Cancer 2011
63 3 381 388 PII 935845687
10.1080/01635581.2011.535963
http://dx.doi.org/10.1080/01635581.2011.535963
8 Oncology; Nutrition & Dietetics Science Citation Index Expanded (SCI-EXPANDED) Oncology; Nutrition & Dietetics
751RO 21462090 Green Accepted 2025-06-24
WOS:000289635500009
J Atmaca, H; Ilhan, S; Korkmaz, E; Zora, M
Atmaca, Harika; Ilhan, Suleyman; Korkmaz, Esra; Zora, Metin
Endoplasmic Reticulum Stress-Induced Apoptotic Effects of Novel 1-Pyrroline (3,4-Dihydro-2H-pyrrole) Derivatives on Breast Cancer Cells CHEMISTRY & BIODIVERSITY
English Article 1-pyrroline; apoptosis; ER stress; breast cancer
Heterocyclic compounds have emerged as promising and appealing scaffolds for developing effective antitumor agents. Here, the effects of synthesized 24 different 1-pyrroline derivatives (PDs) containing substituted aryl sulfide moiety were investigated on human breast cancer cell lines. The viability of cells was assessed via MTT assay. Reactive oxygen species (ROS) generation was analyzed via fluorescent dye CM-H2DCFDA. Apoptotic cells were determined via flow cytometry. Endoplasmic reticulum (ER) stress-associated protein levels were analyzed via western blot analysis. Four of the PDs (PD-12, -14, -16 and -17) had great cytotoxic selectivity against breast cancer cells. Apoptotic cell death was induced by PDs via the generation of ROS. PDs significantly increased the GRP78, p-PEAK, p-eIF2 alpha, and CHOP

protein levels indicating ER stress in breast cancer cells. These results imply that newly synthesized PDs may be potential anticancer agents as they selectively inhibit breast cancer cells.
[Atmaca, Harika; Ilhan, Suleyman] Celal Bayar Univ, Fac Sci & Letters, Dept Biol, TR-45140 Manisa, Turkey; [Korkmaz, Esra; Zora, Metin] Middle East Tech Univ, Dept Chem, TR-06800 Ankara, Turkey Celal Bayar University; Middle East Technical University Atmaca, H (corresponding author), Celal Bayar Univ, Fac Sci & Letters, Dept Biol, TR-45140 Manisa, Turkey.
harika.atmaca@cbu.edu.tr korkmaz, esra/AAE-7994-2022; Zora, Metin/ABB-7678-2020; ILHAN, Suleyman/AAZ-2000-2021; atmaca, harika/AAX-8164-2021 Zora, Metin/0000-0001-7764-2288; korkmaz, esra/0000-0002-6181-3254; ILHAN, Suleyman/0000-0002-6584-3979; atmaca, harika/0000-0002-8459-4373 Scientific and Technological Research Council of Turkey (TUBITAK) [118Z428]
Scientific and Technological Research Council of Turkey (TUBITAK)(Turkiye Bilimsel ve Teknolojik Arastirma Kurumu (TUBITAK)) We thank the Scientific and Technological Research Council of Turkey (TUBITAK, grant no. 118Z428) for financial support of the synthesis of 1-pyrroline compounds. 18 2
2 2 18 WILEY-V C H VERLAG GMBH WEINHEIM
POSTFACH 101161, 69451 WEINHEIM, GERMANY 1612-1872
1612-1880 CHEM BIODIVERS Chem. Biodivers. JUL 2022
19 7 e202200123
10.1002/cbdv.202200123
http://dx.doi.org/10.1002/cbdv.202200123 JUL 2022
9 Biochemistry & Molecular Biology; Chemistry, Multidisciplinary Science Citation Index Expanded (SCI-EXPANDED) Biochemistry & Molecular Biology; Chemistry 3D8DB
35785434 2025-06-24 WOS:000820398400001
J Mahdi, Y; Khmou, M; Souadka, A; Agouri, HE; Ech-charif, S; Mounjid, C; Khannoussi, BE Mahdi, Youssef; Khmou, Mouna; Souadka, Amine; Agouri, Hajar El; Ech-charif, Soumaya; Mounjid, Chaimaa; Khannoussi, Basma El
Correlation between KRAS and NRAS mutational status and clinicopathological features in 414 cases of metastatic colorectal cancer in Morocco: the largest North African case series BMC GASTROENTEROLOGY English Article
KRAS; NRAS; Colorectal neoplasms; Metastases; North Africa K-RAS; EGYPTIAN PATIENTS; BRAF MUTATIONS; COLON-CANCER; ASSOCIATION; GENE; P53 BackgroundAdvances in molecular biology have improved understanding of the molecular features of carcinogenesis and progression of colorectal cancer. It is clear that the efficacy of anti-EGFR depends upon the RAS mutational status, since any mutation in RAS is associated with resistance to anti-EGFR therapy. The aim of this study is to report the largest North African description of KRAS and NRAS status in metastatic colorectal cancer and to describe the association of these mutations with clinicopathological characteristics.MethodsThis is a prospective study of all consecutive unselected metastatic colorectal cancer samples, collected from the Laboratory of Pathology at the National Institute of Oncology of Rabat, Morocco, from January 1st 2020 to December 31st 2021. The molecular analysis was performed on the Idylla (TM) platform (fully automated real-time polymerase chain reaction-based assay) for KRAS and NRAS mutations in exons 2, 3 and 4. These mutations were correlated to gender, primary tumor site, histological type and degree of differentiation of tumor using adequate statistical

methods.ResultsFour hundred fourteen colorectal tumors were screened for KRAS and NRAS mutations. These mutations occurred in 51.7% of tumors for KRAS (mainly in exon 12) and in 3% of tumors for NRAS. There was a significant correlation between NRAS mutation and age of colorectal patients in this study. The low rate of invalid RAS tests (1.7% for KRAS and 3.1% for NRAS) was certainly obtained due to the strict respect of pre-analytical factors such as cold ischemia time and formalin fixation.ConclusionWe report the largest North African analysis of NRAS and KRAS status in colorectal metastatic patients. This study showed the ability in low middle income countries to perform a high rate of valid tests and the unusual trend towards older patients for NRAS mutations.[Mahdi, Youssef; Khmou, Mouna; Agouri, Hajar El; Ech-charif, Soumaya; Mounjid, Chaimaa; Khannoussi, Basma El] Natl Inst Oncol, Pathol Dept, Rabat, Morocco; [Mahdi, Youssef; Khmou, Mouna; Souadka, Amine; Agouri, Hajar El; Khannoussi, Basma El] Mohammed V Univ Rabat, Fac Med & Pharm, Rabat, Morocco; [Souadka, Amine] Natl Inst Oncol, Surg Oncol Dept, Rabat, Morocco; [Mounjid, Chaimaa] Mohammed V Univ Rabat, Lab Biol Human Pathol BioPath, Fac Sci, Rabat, Morocco Mohammed V University in Rabat; Ibn sina University Hospital Center of Rabat; Mohammed V University in Rabat; Ibn sina University Hospital Center of Rabat; Mohammed V University in Rabat; Ibn sina University Hospital Center of Rabat; Mohammed V University in Rabat Mahdi, Y (corresponding author), Natl Inst Oncol, Pathol Dept, Rabat, Morocco.; Mahdi, Y (corresponding author), Mohammed V Univ Rabat, Fac Med & Pharm, Rabat, Morocco. ysf.mahdi@gmail.com Amine, Souadka/ABB-1728-2020 41 6 6 0 2 BMC LONDON CAMPUS, 4 CRINAN ST, LONDON N1 9XW, ENGLAND 1471-230X BMC GASTROENTEROL BMC Gastroenterol. JUN 5 2023 23 1 193 10.1186/s12876-023-02694-7 http://dx.doi.org/10.1186/s12876-023-02694-7 7 Gastroenterology & Hepatology Science Citation Index Expanded (SCI-EXPANDED) Gastroenterology & Hepatology I0LJ0 37277698 Green Published, gold 2025-06-24 WOS:000999772600003
J Lendzion, K; Gornowicz, A; Strawa, JW; Bielawska, K; Czarnomysy, R; Poplawska, B; Bielawski, K; Tomczyk, M; Miltyk, W; Bielawska, A Lendzion, Karolina; Gornowicz, Agnieszka; Strawa, Jakub W.; Bielawska, Katarzyna; Czarnomysy, Robert; Poplawska, Bozena; Bielawski, Krzysztof; Tomczyk, Michal; Miltyk, Wojciech; Bielawska, Anna LC-PDA-MS and GC-MS Analysis of Scorzonera hispanica Seeds and Their Effects on Human Breast Cancer Cell Lines INTERNATIONAL JOURNAL OF MOLECULAR SCIENCES English Article Scorzonera; seeds; polyphenols; LC-PDA-MS; GC-MS; breast cancer; biological activity PHENOLIC-COMPOUNDS; ESTROGEN-RECEPTOR; MEDICINAL-PLANTS; APOPTOSIS; ACID; (-)-SYRINGARESINOL; SESQUITERPENOIDS; INTERLEUKIN-10; CONSTITUENTS; METABOLITES Scorzonera hispanica is an herbaceous perennial cultivated in Central and Southern Europe. This study aimed to qualitatively and quantitatively evaluate the composition of oil, extracts, and fractions (SH1-SH12) obtained from S. hispanica seeds. Furthermore, an evaluation of biological activities in breast cancer cell lines was also performed. GC-MS analysis revealed that the primary components of the seed oil (SH12) were fatty acids and beta-sitosterol. In the evaluation of extracts

(SH1-SH3, SH8-SH10) and fractions (SH4-SH7, SH11) composition, the presence of apigenin, derivatives of p-coumaric and caffeic acids, was reported. In the biological assays, methanolic extract (SH1), diethyl ether (SH4), and chloroform (SH11) fractions exhibited cytotoxicity toward cells. The highest activity was observed for fatty acids- and 3,4-dimethoxycinnamate-rich SH11 (IC50: 399.18 mu g/mL for MCF-7, 781.26 mu g/mL for MDA-MB-231). SH11 was also observed to induce apoptosis in MCF-7 cells (52.4%). SH1, SH4, and SH11 attenuate signaling pathways and affect the expression of apoptosis-, autophagy-, and inflammation-related proteins. SH12 was non-toxic toward either cancer or normal cell lines in concentrations up to 1 mg/mL. The results suggest that S. hispanica seeds exhibit a wide range of potential uses as a source of oil and bioactive compounds for complementary therapy of breast cancer. [Lendzion, Karolina; Gornowicz, Agnieszka; Poplawska, Bozena; Bielawska, Anna] Med Univ Bialystok, Fac Pharm, Dept Biotechnol, Ul Kilinskiego 1, PL-15089 Bialystok, Poland; [Strawa, Jakub W.; Tomczyk, Michal] Med Univ Bialystok, Fac Pharm, Dept Pharmacognosy, Ul Mickiewicza 2A, PL-15230 Bialystok, Poland; [Bielawska, Katarzyna; Miltyk, Wojciech] Med Univ Bialystok, Fac Pharm, Dept Pharmaceut & Biopharmaceut Anal, Ul Mickiewicza 2D, PL-15222 Bialystok, Poland; [Czarnomysy, Robert; Bielawski, Krzysztof] Med Univ Bialystok, Fac Pharm, Dept Synth & Technol Drugs, Ul Kilinskiego 1, PL-15089 Bialystok, Poland Medical University of Bialystok; Medical University of Bialystok; Medical University of Bialystok; Medical University of BialystokLendzion, K (corresponding author), Med Univ Bialystok, Fac Pharm, Dept Biotechnol, Ul Kilinskiego 1, PL-15089 Bialystok, Poland.
karolina.lendzion@umb.edu.plTomczyk, Michał/G-2161-2016; Gornowicz, Agnieszka/AAA-5918-2020; Czarnomysy, Robert/R-5773-2018; Bielawski, Krzysztof/KLZ-2862-2024; Strawa, Jakub Wladyslaw/O-9211-2018; Miltyk, Wojciech/P-6937-2018
Gornowicz, Agnieszka/0000-0002-0945-7870; Strawa, Jakub Wladyslaw/0000-0001-7133-1817; Lendzion, Karolina/0000-0003-3408-2289; Miltyk, Wojciech/0000-0001-5150-6093; Bielawski, Krzysztof/0000-0003-3187-4205; Tomczyk, Michal/0000-0002-4063-1048
Medical University of Bialystok [SUB/2/DN/22/001/2229, SUB/2/DN/22/002/2229] Medical University of Bialystok This work has been financed by the Medical University of Bialystok, grant numbers: SUB/2/DN/22/001/2229 and SUB/2/DN/22/002/2229. 85
6 6 4 15 MDPI BASEL MDPI AG, Grosspeteranlage 5, CH-4052 BASEL, SWITZERLAND 1661-6596 1422-0067 INT J MOL SCI Int. J. Mol. Sci. OCT 2022 23 19
11584 10.3390/ijms231911584
http://dx.doi.org/10.3390/ijms231911584 29
Biochemistry & Molecular Biology; Chemistry, Multidisciplinary Science Citation Index Expanded (SCI-EXPANDED) Biochemistry & Molecular Biology; Chemistry 5H6EY
36232888 Green Published, gold 2025-06-24
WOS:000867771700001
J Ilhan, S; Atmaca, H; Yilmaz, ES; Korkmaz, E; Zora, M
Ilhan, Suleyman; Atmaca, Harika; Yilmaz, Elif Serel; Korkmaz, Esra; Zora, Metin N-Propargylic β-enaminones in breast cancer cells: Cytotoxicity, apoptosis, and cell cycle analyses JOURNAL OF BIOCHEMICAL AND MOLECULAR TOXICOLOGY English Article
apoptosis; breast cancer; G0; G1 arrest; N-propargylic beta-enaminone; ROS level BIOLOGICAL-ACTIVITY; TRABECTEDIN

Breast cancer is one of the most common cancers worldwide and the discovery of new cytotoxic agents is needed. Enaminones are regarded to be a significant structural motif that is found in a variety of pharmacologically active compounds however the number of studies investigating the anticancer activities of N-propargylic beta-enaminones (NPEs) is limited. Herein we investigated the potential cytotoxic and apoptotic effects of 23 different NPEs (1-23) on human breast cancer cells. Cytotoxicity was evaluated via MTT assay. Apoptotic cell death and cell cycle distributions were investigated by flow cytometry. CM-H2DCFDA dye was used to evaluate cellular ROS levels. Expression levels of Bcl-2, Bax, p21, and Cyclin D1 were measured by quantitative real-time PCR. ADME properties were calculated using the ADMET 2.0 tool. NPEs 4, 9, 16, and 21 showed selective cytotoxic activity against breast cancer cells with SI values > 2. NPEs induced apoptosis and caused significant changes in Bcl-2 and Bax mRNA levels. The cell cycle was arrested at the G0/G1 phase and levels of p21 and Cyclin D1 were upregulated in both breast cancer cells. ROS levels were significantly increased by NPEs, suggesting that the cytotoxic and apoptotic effects of NPEs were mediated by ROS. ADME analysis revealed that NPEs showed favorable distributions in both breast cancer cell lines, meaning good lipophilicity values, low unfractionated values, and high bioavailability. Therefore, these potential anticancer compounds should be further validated by in vivo studies for their appropriate function in human health with a safety profile, and a comprehensive drug interaction study should be performed. [Ilhan, Suleyman; Atmaca, Harika] Celal Bayar Univ, Fac Sci & Letters, Dept Biol, Manisa, Turkiye; [Yilmaz, Elif Serel; Korkmaz, Esra; Zora, Metin] Middle East Tech Univ, Dept Chem, Ankara, Turkiye; [Ilhan, Suleyman] Celal Bayar Univ, Fac Sci & Letters, Dept Biol, TR-45140 Manisa, Turkiye
Celal Bayar University; Middle East Technical University; Celal Bayar University Ilhan, S (corresponding author), Celal Bayar Univ, Fac Sci & Letters, Dept Biol, TR-45140 Manisa, Turkiye. suleyman.ilhan@cbu.edu.tr korkmaz, esra/AAE-7994-2022; Yılmaz, Elif Serel/LPP-8610-2024; Zora, Metin/ABB-7678-2020; ILHAN, Suleyman/AAZ-2000-2021; atmaca, harika/AAX-8164-2021
ILHAN, Suleyman/0000-0002-6584-3979; atmaca, harika/0000-0002-8459-4373 Scientific and Technological Research Council of Turkey (TUBITAK) [110T113, 114Z811, 118Z428] Scientific and Technological Research Council of Turkey (TUBITAK)(Turkiye Bilimsel ve Teknolojik Arastirma Kurumu (TUBITAK)) ACKNOWLEDGMENTS We thank the Scientific and Technological Research Council of Turkey (TUBITAK, grant no. 110T113, 114Z811, and 118Z428) for financial support of the synthesis of NPEs. 35 6 6
0 10 WILEY HOBOKEN 111 RIVER ST, HOBOKEN 07030-5774, NJ USA 1095-6670 1099-0461 J BIOCHEM MOL TOXIC
J. Biochem. Mol. Toxicol. APR 2023 37 4
10.1002/jbt.23299
http://dx.doi.org/10.1002/jbt.23299 JAN 2023 13
Biochemistry & Molecular Biology; Toxicology Science Citation Index Expanded (SCI-EXPANDED) Biochemistry & Molecular Biology; Toxicology C9OH9 36647602 2025-06-24 WOS:000914472500001
J Brim, H; Reddy, CS; Chirumamilla, L; Oskrochi, G; Deverapalli, M; Rashid, R; Rashid, M; Nair, V; Morrison, N; Byer, D; Thompson, T; Yasin, B; Johnson, D; Snowden, A; Mammen, P; Carter, G; Jolly, V; Thompson, R; Abdulmoniem, R; Karodeh, N;

Gojela, Y; Ahmed, A; Saroya, S; Gibbs, T; Dawodu, D; Shayegh, N; Ahmed, AH; Zahedi, I; Aduli, F; Kibreab, A; Laiyemo, AO; Shokrani, B; Zafar, R; Nembhard, C; Carethers, JM; Ashktorab, H Brim, Hassan; Reddy, Challa Suryanarayana; Chirumamilla, Lakshmi; Oskrochi, Gholamreza; Deverapalli, Mrinalini; Rashid, Rumaisa; Rashid, Mudasir; Nair, Vaisakh; Morrison, Nicole; Byer, Danae; Thompson, Trae; Yasin, Belal; Johnson, David; Snowden, Alicia; Mammen, Priscilla; Carter, Gabriel; Jolly, Victor; Thompson, Rasheed; Abdulmoniem, Riad; Karodeh, Nima; Gojela, Yafiet; Ahmed, Ali; Saroya, Sabtain; Gibbs, Trinity; Dawodu, Dideolu; Shayegh, Nader; Ahmed, Ali H.; Zahedi, Iman; Aduli, Farshad; Kibreab, Angesom; Laiyemo, Adeyinka O.; Shokrani, Babak; Zafar, Rabia; Nembhard, Christine; Carethers, John M.; Ashktorab, Hassan Trends and Symptoms Among Increasing Proportion of African Americans with Early-Onset Colorectal Cancer over a 60-Year Period DIGESTIVE DISEASES AND SCIENCES English Article Early-onset; Late-onset; African Americans; Colorectal cancer AGE; DISPARITIES; STATISTICS; GUIDELINES; FEATURES; BIOLOGY; TIME Background The proportion of early onset colorectal cancer (EOCRC) is alarming in adults, including in African Americans (AA). Aim To investigate differences between EOCRC compared to late-onset colorectal cancer (LOCRC) among AA patients. Methods This retrospective study reviewed demographic, clinical presentations, colonoscopy, and pathology reports of patients at Howard University Hospital from 1959 to 2023. The study included 176 EOCRC cases (< 45 years) and 2034 LOCRC cases (> 45 years). Results Both EOCRC and LOCRC groups were predominantly AA (> 80%) with slightly more females (53%) than males. The mean age was 38 years for EOCRC and 66 years for LOCRC cases. EOCRC cases increased as a proportion of total detected CRC cases since 2010 (over 13%) after several decades of just above 6%. Family history of CRC in first degree relatives was higher among EOCRC (15.5% vs.3.4% in LOCRC patients, $p < 0.01$). Symptoms at presentation were prevalent in both EOCRC (93.8%) and LOCRC (92.6%). EOCRC patients exhibited higher incidence of abdominal pain (23.3% vs. 17.2%, $p = 0.05$) and changes in bowel habits (24.4% vs. 14%, $p < 0.01$) compared to LOCRC patients. Other symptoms such as melena, hematochezia, and weight loss were less prevalent in EOCRC patients. Comorbidities like hypertension (HTN), diabetes mellitus (DM), and inflammatory bowel disease (IBD) were less frequent among EOCRC patients. EOCRC was primarily observed in the sigmoid and rectosigmoid regions ($p = 0.02$). Metastasis at index colonoscopy was more prevalent with EOCRC compared to LOCRC ($p = 0.04$), with a higher proportion of patients at stage 3 cancer ($p < 0.05$). Significant differences were noted in the timeline for undergoing surgery after the diagnosis of colorectal cancer, with EOCRC patients taking longer than LOCRC patients ($p = 0.03$). Conclusion Presentation of EOCRC over LOCRC increased proportionally in our cohort since 2010 and is associated with family history, and symptoms such as abdominal pain and change in bowel habits. Likely because of age at presentation, there are less comorbidities among EOCRC patients who predominantly present in the outpatient setting, and more likely diagnosed with advanced stage lesions that are predominantly sigmoid or rectosigmoid. These findings are similar to observations seen in the general population with EOCRC, albeit African American patients have commonly had earlier age

presentation of CRC than White American patients. [Brim, Hassan; Reddy, Challa Suryanarayana; Chirumamilla, Lakshmi; Deverapalli, Mrinalini; Rashid, Rumaisa; Rashid, Mudasir; Nair, Vaisakh; Morrison, Nicole; Byer, Danae; Thompson, Trae; Yasin, Belal; Johnson, David; Snowden, Alicia; Mammen, Priscilla; Carter, Gabriel; Jolly, Victor; Thompson, Rasheed; Abdulmoniem, Riad; Karodeh, Nima; Gojela, Yafiet; Ahmed, Ali; Saroya, Sabtain; Gibbs, Trinity; Dawodu, Dideolu; Shayegh, Nader; Ahmed, Ali H.; Zahedi, Iman; Aduli, Farshad; Kibreab, Angesom; Laiyemo, Adeyinka O.; Shokrani, Babak; Zafar, Rabia; Nembhard, Christine; Ashktorab, Hassan] Howard Univ, Pathol & Canc Ctr, Dept Med, Coll Med, Washington 20059, DC USA; [Oskrochi, Gholamreza] Amer Univ Middle East, Coll Engn & Technol, Egaila, Kuwait; [Carethers, John M.] Univ Calif San Diego, Moores Canc Ctr, Wertheim Sch Publ Hlth & Human Longev, Dept Med, San Diego, CA USA Howard University; American University of the Middle East; University of California System; University of California San Diego Ashktorab, H (corresponding author), Howard Univ, Pathol & Canc Ctr, Dept Med, Coll Med, Washington 20059, DC USA. hashktorab@Howard.edu
Ashktorab, hassan/AAJ-2908-2020; Johnson, David/MXL-7780-2025 National Cancer Institute (National Institutes of Health) [R01CA258519] National Cancer Institute (National Institutes of Health)(United States Department of Health & Human ServicesNational Institutes of Health (NIH) - USANIH National Cancer Institute (NCI)) This work was supported by R01CA258519, grants from the National Cancer Institute (National Institutes of Health). 34 1 1 0 1 SPRINGER DORDRECHT VAN GODEWIJCKSTRAAT 30, 3311 GZ DORDRECHT, NETHERLANDS 0163-2116 1573-2568 DIGEST DIS SCI Dig. Dis. Sci. JAN 2025 70 1 168 176
10.1007/s10620-024-08739-5
http://dx.doi.org/10.1007/s10620-024-08739-5 NOV 2024
9 Gastroenterology & Hepatology Science Citation Index Expanded (SCI-EXPANDED) Gastroenterology & Hepatology
T5L2Z 39586927 2025-06-24
WOS:001363718600001
J Triantafillidis, JK; Kosmidis, PA; Papalois, A; Merikas, E; Cheracakis, P; Govosdis, V; Nicolakis, D; Panteris, V; Skourta, I; Tountas, I; Spanos, E; Karkanis, F; Golga, C
Triantafillidis, J. K.; Kosmidis, Paris A.; Papalois, A.; Merikas, E.; Cheracakis, P.; Govosdis, V.; Nicolakis, D.; Panteris, V.; Skourta, I.; Tountas, I.; Spanos, E.; Karkanis, F.; Golga, C. Screening programs for colorectal cancer in Greece: Results of two pilot studies conducted in March 2008 and 2009 ANNALS OF GASTROENTEROLOGY English Article
Colorectal cancer; screening; surveillance; fecal occult blood test; prevention
Background: Detection of occult blood in stools is an established method for the early detection of colorectal cancer in asymptomatic individuals of average risk. The aim of this study was to present the results derived from the application of the test in a cohort of Greek population of average risk. Subjects and Methods: We conducted two pilot studies in March 2008 and 2009 respectively. The first was conducted only in the greater area of Athens while the second one included two more major cities in the North and South part of the country (Salonika and Iraklion, Crete). In both campaigns all residents, aged between 55 and 72 years were asked through relevant television spots, articles in

newspapers, radio spots and press conferences to participate in both studies (March 2008 and March 2009), by submitting a stool sample in the nearest "Biomedicine" Laboratory. For the detection of haemoglobin in stools LINEAR immunochemical FOBT was applied. This test is a qualitative, lateral flow immunoassay for the detection of human hemoglobin in stools. In order to include in the statistical analysis more than 90% of all examined individuals, we divided the number of subjects participating in the 2009 trial into three groups aged 55-60, 61-66, and 67-72 years. Moreover, in order to be able to calculate the odds ratios, these groups were transformed from numerical to nominal ones. Statistical analysis was performed using Pearson chi square test. Results: 1st trial, March 2008: The total number of individuals examined was 4,010. The rate of positivity was 9.83% (394 out of 4,010 individuals examined). Among the positive samples 30.2% corresponded to subjects aged 5560 years, 35.3% to subjects aged 61-65 years and 34.5% to subjects aged 65-70 years. 2nd trial, March 2009: The total number of individuals tested was 7,079 with 3,131(44.2%) being men and 3948(55.8%) being women. The participation rate was: Athens 5,037 (71.1%), Salonika 1,407 (19.9%) and Iraklion 635 subjects (9.0%). The positivity rate was 11.1% (786 out of 7,079 subjects). The positivity rate among men (11.9%) (373/3,131) was higher compared to women (10.5%) (413/3,948) although this difference did not reach statistical significance (P=0.057). Mean age of the positive subjects was statistically significantly higher compared to negative ones (62.4 vs 61.9yrs, P=0.01). Significant differences existed between group 1 (55-60 years) vs group 3 (67-72 years). So, the probability of a positive test in the age of 67-72 was 1.36 times higher than at the age of 55-60 and the probability of a positive test in the age of 67-72 was 1.26 times higher than at the age of 61-66. Statistically significant difference in the positivity rate of the test between the three areas was noticed. So, residence was responsible for the variation in positive rate of FOBT by 6.9% (Eta statistics). Conclusion: The rate of positivity of iFOBT in Greece is in accordance with that reported in the relevant international literature and remains steady, at least during the years 2008 and 2009. However, it seems to be lower in areas adopting the so called Mediterranean diet (Iraklion, Crete). Taking into account the number of inhabitants of the country over the age of 55, we can assume that the compliance of the Greek population in colorectal cancer screening programs is relatively low. In the forthcoming years, screening programs in Greece must be adopted by the health authorities of the country in order to cover a larger part of the population. The experience derived from our studies could result in more successive and productive future programs. [Triantafillidis, J. K.; Kosmidis, Paris A.; Papalois, A.; Merikas, E.; Cheracakis, P.; Govosdis, V.; Nicolakis, D.; Panteris, V.] Hellen Soc Gastrointestinal Oncol, Athens, Greece; [Triantafillidis, J. K.; Nicolakis, D.; Panteris, V.] St Panteleimon Gen Hosp, Dept Gastroenterol, Nicea, Greece; [Skourta, I.] Non Profit Non Govt Org ATKAAIAZ, Athens, Greece; [Tountas, I.] Univ Athens, Ctr Hlth Serv Res, Med Sch, Athens, Greece; [Spanos, E.] BioIatriki Labs, Athens, Greece; [Karkanis, F.; Golga, C.] Roche Hellas Pharmaceut Co, Athens, Greece National & Kapodistrian University of Athens Triantafillidis, JK (corresponding author), Iera Odos 354, Haidari 12461, Greece. jkt@vodafone.net.gr 18

1 1 0 0 HELLENIC SOC GASTROENTEROLOGY ATHENS DEMOKRATIAS AVE 67, ATHENS, 15451, GREECE 1108-7471 1792-7463 ANN GASTROENTEROL Ann. Gastroenterol. 2010 23 1 42 47 6 Gastroenterology & Hepatology Emerging Sources Citation Index (ESCI) Gastroenterology & Hepatology V6B2K 2025-06-24 WOS:000420125700008

J Haslam, DE; John, EM; Knight, JA; Li, ZY; Buys, SS; Andrulis, IL; Daly, MB; Genkinger, JM; Terry, MB; Zhang, FF Haslam, Danielle E.; John, Esther M.; Knight, Julia A.; Li, Zhongyu; Buys, Saundra S.; Andrulis, Irene L.; Daly, Mary B.; Genkinger, Jeanine M.; Terry, Mary Beth; Zhang, Fang Fang Diet Quality and All-Cause Mortality in Women with Breast Cancer from the Breast Cancer Family Registry CANCER EPIDEMIOLOGY BIOMARKERS & PREVENTION English Article POSTMENOPAUSAL WOMEN; MULTIETHNIC COHORT; DISEASE MORTALITY; INDEXES; RISK; HYPERTENSION; ADHERENCE; SURVIVORS; PATTERNS; ASSOCIATION Background: The impact of diet on breast cancer survival remains inconclusive. We assessed associations of all-cause mor-tality with adherence to the four diet quality indices: Healthy Eating Index-2015 (HEI-2015), Alternative Healthy Eating Index (AHEI), Alternative Mediterranean Diet (aMED), and Dietary Approaches to Stop Hypertension (DASH).Methods: Dietary intake data were evaluated for 6,157 North American women enrolled in the Breast Cancer Family Registry who had been diagnosed with invasive breast cancer from 1993 to 2011 and were followed through 2018. Pre-diagnosis (n = 4,557) or post-diagnosis (n = 1,600) dietary intake was estimated through a food frequency questionnaire. During a median follow-up time of 11.3 years, 1,265 deaths occurred. Cox proportional hazards models were used to estimate multivariable-adjusted HR and 95% confi- dence intervals (CI).Results: Women in the highest versus lowest quartile of adher-ence to the HEI-2015, AHEI, aMED, and DASH indices had a lower risk of all-cause mortality. HR (95% CI) were 0.88 (0.74-1.04; Ptrend = 0.12) for HEI-2015; 0.82 (0.69-0.97; Ptrend = 0.02) for AHEI; 0.73 (0.59-0.92; Ptrend = 0.02) for aMED; and 0.78 (0.65-0.94; Ptrend = 0.006) for DASH. In subgroup analyses, the associations with higher adherence to the four indices were similar for pre-or post-diagnosis dietary intake and were confined to women with a body mass index <25 kg/m2 and women with hormone receptor positive tumors. Conclusions: Higher adherence to the HEI-2015, AHEI, aMED, and DASH indices was associated with lower mortality among women with breast cancer.Impact: Adherence to a healthy diet may improve survival of women with breast cancer.

[Haslam, Danielle E.; Li, Zhongyu; Zhang, Fang Fang] Tufts Univ, Friedman Sch Nutr Sci & Policy, Boston, MA USA; [John, Esther M.] Stanford Univ, Dept Epidemiol & Populat Hlth, Sch Med, Stanford, CA USA; [John, Esther M.] Stanford Univ, Dept Med Oncol, Sch Med, Stanford, CA USA; [John, Esther M.] Stanford Univ, Stanford Canc Inst, Sch Med, Stanford, CA USA; [Knight, Julia A.; Andrulis, Irene L.] Sinai Hlth Syst, Lunenfeld Tanenbaum Res Inst, Toronto, ON, Canada; [Knight, Julia A.] Univ Toronto, Dalla Lana Sch Publ Hlth, Toronto, ON, Canada; [Buys, Saundra S.] Univ Utah, Huntsman Canc Inst, Hlth Sci Ctr, Salt Lake City, UT USA; [Andrulis, Irene L.] Univ Toronto, Dept Mol Genet, Toronto, ON, Canada; [Daly, Mary B.] Fox Chase Canc Ctr, Clin Genet,

Philadelphia, PA USA; [Genkinger, Jeanine M.; Terry, Mary Beth] Columbia Univ, Mailman Sch Publ Hlth, New York, NY USA; [Genkinger, Jeanine M.; Terry, Mary Beth] Herbert Irving Comprehens Canc Ctr, New York, NY USA; [Zhang, Fang Fang] Tufts Univ, Nutr Epidemiol & Data Sci, 150 Harrison Ave, Boston, MA 02111 USA Tufts University; Stanford University; Stanford University; Stanford Cancer Institute; Stanford University; University of Toronto; Sinai Health System Toronto; Lunenfeld Tanenbaum Research Institute; University of Toronto; Utah System of Higher Education; University of Utah; Huntsman Cancer Institute; University of Toronto; Fox Chase Cancer Center; Columbia University; Tufts University Zhang, FF (corresponding author), Tufts Univ, Nutr Epidemiol & Data Sci, 150 Harrison Ave, Boston, MA 02111 USA. fang_fang.zhang@tufts.edu Knight, Julia/A-6843-2012; Andrulis, Irene/E-7267-2013; Li, Zhongyu/HDO-2794-2022; Haslam, Danielle/V-8846-2019; Zhang, Fangfang/D-6776-2013 Buys, Saundra/0000-0003-4120-5697; Zhang, Fang Fang/0000-0002-3130-0087; Haslam, Danielle/0000-0003-0144-3287; John, Esther/0000-0003-3259-8003; Genkinger, Jeanine/0000-0002-8438-0412
National Cancer Institute (NCI) [U01 CA164920]; NIH [5T32HL069772 - 15, 2T32CA009001 - 39] National Cancer Institute (NCI)(United States Department of Health & Human ServicesNational Institutes of Health (NIH) - USANIH National Cancer Institute (NCI)); NIH(United States Department of Health & Human ServicesNational Institutes of Health (NIH) - USA) The Breast Cancer Family Registry (BCFR) is supported by grant U01 CA164920 from the National Cancer Institute (NCI) . The content of this manuscript does not necessarily re fl ect the views or policies of the NCI or any of the collaborating centers in the BCFR, nor does mention of trade names, commercial products, or organizations imply endorsement by the U.S. Government or the BCFR. D.E. Haslam was partially supported by NIH 5T32HL069772 - 15 and NIH 2T32CA009001 - 39. 36 5 6 0 1 AMER ASSOC CANCER RESEARCH PHILADELPHIA 615 CHESTNUT ST, 17TH FLOOR, PHILADELPHIA, PA 19106-4404 USA 1055-9965 1538-7755
CANCER EPIDEM BIOMAR Cancer Epidemiol. Biomarkers Prev.
MAY 2023 32 5 678 686
10.1158/1055-9965.EPI-22-1198
http://dx.doi.org/10.1158/1055-9965.EPI-22-1198
9 Oncology; Public, Environmental & Occupational Health
Science Citation Index Expanded (SCI-EXPANDED) Oncology; Public, Environmental & Occupational Health F9OS3 36857773
Green Submitted, Green Accepted 2025-06-24
WOS:000985574700001
J Carini, F; Tomasello, G; Jurjus, A; Geagea, A; Al Kattar, S; Damiani, P; Sinagra, E; Rappa, F; David, S; Cappello, F; Mazzola, M; Leone, A Carini, F.; Tomasello, G.; Jurjus, A.; Geagea, A.; Al Kattar, S.; Damiani, P.; Sinagra, E.; Rappa, F.; David, S.; Cappello, F.; Mazzola, M.; Leone, A.
COLORECTAL CANCER AND INFLAMMATORY BOWEL DISEASES: EFFECTS OF DIET AND ANTIOXIDANTS JOURNAL OF BIOLOGICAL REGULATORS AND HOMEOSTATIC AGENTS English Article
inflammatory bowel diseases; IBD; colorectal cancer; antioxidant; anti-inflammatory activity of tea
COLON-CANCER; GREEN TEA; CELLS; POLYPHENOLS; INHIBITION; INDUCTION; GROWTH It is well established that oxidative stress is common in inflammatory bowel diseases (IBDs). Accordingly, antioxidants are recommended for treatment. The aim

of this study is to compare the effects of antioxidants contained in the various types of tea on symptoms and evolution of IBD and colorectal cancer (CRC). Analysis of the literature revealed that the theaflavin-3, 30-digallate (TFDG) contained in black tea, and epigallocatechin-3-O-gallate (EGCG) contained in green tea have protective effects against oxidative stress. Moreover, these substances are involved in many biochemical processes responsible for inflammation and proliferation of cancer cells. It is documented that both TFDG and EGCG are able to reduce inflammatory phenomena and symptoms associated with IBD, as well as to reduce the proliferation of CRC cells. Most studies are performed in vitro or in experimental animal models. It is, therefore, advisable to formulate studies that could be carried out on humans or human samples, in order to develop the appropriate therapeutic strategies.[Carini, F.; Tomasello, G.; Geagea, A.; Rappa, F.; David, S.; Cappello, F.; Mazzola, M.; Leone, A.] Univ Palermo, Dept Expt Biomed & Clin Neurosci, Palermo, Italy; [Jurjus, A.; Geagea, A.; Al Kattar, S.] Amer Univ Beirut, Dept Anat Cell Biol & Physiol Sci, Beirut, Lebanon; [Damiani, P.] Univ Palermo, Sch Med & Surg, AOUP P Giaccone, Palermo, Italy; [Sinagra, E.; Mazzola, M.] Euro Mediterranean Inst Sci & Techonolgy IEMEST P, Palermo, Italy; [Sinagra, E.] Fdn Ist S Raffaele G Giglio, Gastroenterol & Endoscopy Unit, Cefalu, Italy University of Palermo; American University of Beirut; University of Palermo Leone, A (corresponding author), Univ Palermo, Sch Med, Sect Histol & Embryol, BioNec, Via Vesrpo 129, I-90127 Palermo, Italy.
angelo.leone@unipa.it Francesca, Rappa/ADW-8431-2022; David, Sonia/AAU-6139-2020; Cappello, Francesco/F-9153-2012
TOMASELLO, GIOVANNI/0000-0002-3071-822X; Cappello, Francesco/0000-0001-9288-1148; rappa, francesca/0000-0001-6610-5268; carini, francesco/0000-0001-9442-1671
18 15 18 0 9 BIOLIFE SAS SILVA MARINA (TE)
VIA S STEFANO 39 BIS, 64029 SILVA MARINA (TE), ITALY
0393-974X 1724-6083 J BIOL REG HOMEOS AG J. Biol. Regul. Homeost. Agents JUL-SEP 2017 31 3
791 795 5
Endocrinology & Metabolism; Immunology; Medicine, Research & Experimental; Physiology Science Citation Index Expanded (SCI-EXPANDED) Endocrinology & Metabolism; Immunology; Research & Experimental Medicine; Physiology FJ4EE 28726358
2025-06-24 WOS:000412687500035
J Murtaugh, MA; Sweeney, C; Giuliano, AR; Herrick, JS; Hines, L; Byers, T; Baumgartner, KB; Slattery, ML
Murtaugh, Maureen A.; Sweeney, Carol; Giuliano, Anna R.; Herrick, Jennifer S.; Hines, Lisa; Byers, Tim; Baumgartner, Kathy B.; Slattery, Martha L. Diet patterns and breast cancer risk in Hispanic and non-Hispanic white women: the Four-Corners Breast Cancer Study AMERICAN JOURNAL OF CLINICAL NUTRITION English Article
PROSPECTIVE COHORT; OBESITY; OVERWEIGHT; HISTORY; WEIGHT; FAT Background: There is a lower incidence of breast cancer among Hispanic women than among non-Hispanic white women. Little is known about the role of diet in this difference. Objective: We examined the associations of dietary patterns (Western, Prudent, Native Mexican, Mediterranean, and Dieter) with risk for breast cancer in Hispanic women (757 cases, 867 controls) and non-Hispanic white women (1524 cases, 1598 controls) from the Four-Corners Breast Cancer Study. Design: Dietary intake, physical

activity, and other exposures were assessed by using interviews. Dietary patterns were defined via factor analysis. Risk was assessed by using logistic regression with adjustment for age, center, education, smoking, total activity, calories, dietary fiber, dietary calcium, height, parity, recent hormone exposure, family history of breast cancer, menopausal status, and body mass index X recent hormone exposure. Results: The Western (odds ratio for highest versus lowest quartile: 1.32; 95% CI: 1.04,168; P for trend < 0.01) and Prudent (1.42; 1. 1.4, 1.77; P for trend < 0.01) dietary patterns were associated with greater risk, and the Native Mexican (0.68; 0.55, 0.85; P for trend < 0.01) and Mediterranean (0.76; 0.63, 0.92; P for trend < 0.01) dietary patterns were associated with lower risk of breast cancer. Body mass index modified the associations of the Western diet and breast cancer among postmenopausal women and those of the Native Mexican diet among premenopausal women. Conclusions: Associations of dietary patterns with breast cancer risk varied by menopausal and body mass index status, but there was little difference in associations between non-Hispanic white and Hispanic women. [Murtaugh, Maureen A.; Sweeney, Carol; Herrick, Jennifer S.; Slattery, Martha L.] Univ Utah, Dept Internal Med, Div Epidemiol, Provo, UT USA; [Giuliano, Anna R.] Moffit Canc Ctr, Tampa, FL USA; [Hines, Lisa] Univ Colorado, Dept Biol, Denver, CO 80202 USA; [Byers, Tim] Univ Colorado, Sch Med, Dept Prevent Med & Biometr, Denver, CO USA; [Baumgartner, Kathy B.] Univ Louisville, Dept Epidemiol & Populat Hlth, Louisville, KY 40292 USA Utah System of Higher Education; University of Utah; University of Colorado System; University of Colorado Denver; University of Colorado System; University of Colorado Denver; University of Colorado Anschutz Medical Campus; University of Louisville Murtaugh, MA (corresponding author), AC 230 SOM,30N 1900 E St, Salt Lake City, UT 84132 USA.
maureen.murtaugh@hsc.utah.edu Murtaugh, Maureen/AAN-6225-2020 Sweeney, Carol/0000-0003-1113-7160; Murtaugh, Maureen/0000-0001-5281-0302; Herrick, Jennifer/0000-0001-9257-8843
NCI NIH HHS [R01 CA078682, CA 078762, CA 078682, N01-PC-67000, R01 CA078552, CA078802, R01 CA078802, CA078552, R01 CA078762] Funding Source: Medline NCI NIH HHS(United States Department of Health & Human ServicesNational Institutes of Health (NIH) - USANIH National Cancer Institute (NCI)) 21
119 136 0 8 OXFORD UNIV PRESS OXFORD
GREAT CLARENDON ST, OXFORD OX2 6DP, ENGLAND 0002-9165
1938-3207 AM J CLIN NUTR Am. J. Clin. Nutr. APR
2008 87 4 978 984
10.1093/ajcn/87.4.978
http://dx.doi.org/10.1093/ajcn/87.4.978 7
Nutrition & Dietetics Science Citation Index Expanded (SCI-EXPANDED) Nutrition & Dietetics 288UT 18400722 Bronze, Green Accepted 2025-06-24 WOS:000255012000025
J Shirkhani, M; Heydarheydari, S; Farshchian, N; Eivazi, MT; Haghparast, A Shirkhani, Mostafa; Heydarheydari, Sahel; Farshchian, Negin; Eivazi, Mohammad Taghi; Haghparast, Abbas Fetal Dose Estimation for Pregnant Breast Cancer Patients during Radiotherapy Using an In-house Phantom MIDDLE EAST JOURNAL OF CANCER
English Article Breast cancer; Radiation therapy; Fetal dose Background: Up to 3% of breast cancers may be diagnosed in pregnancy, during which period radiation therapy is not preferred, yet sometimes

inevitable. Due to fetal radiation sensitivity, the fetal radiation safety is of particular concern. The present study was performed to estimate fetal dose for pregnant breast cancer patients during radiotherapy using an in-house phantom. Method: The fetal dose was estimated through phantom measurement using an ion chamber dosimeter. The phantom measurement was performed by simulating treatment planning on an in-house anthropomorphic phantom which consisted of natural human bone, cork, and paraffin. The right breast and the right supraclavicular area of the phantom were irradiated under the four-field technique with 6 and 10 MV photon beams for un-wedged and wedged fields. Results: During the first trimester of pregnancy, the radiation dose delivered to the fetus was in the range of 0.11-0.14 Gy for a 50 Gy total tumor dose in 25 fractions. The fetal dose in the second and third trimester of pregnancy ranged from 0.14-0.19 Gy to 0.22-0.32 Gy, respectively. Conclusion: According to the results, the fetal dose is strongly dependent upon the energy beam, treatment procedure, and gestational stage. [Shirkhani, Mostafa; Heydarheydari, Sahel; Eivazi, Mohammad Taghi; Haghparast, Abbas] Kermanshah Univ Med Sci, Fac Med, Dept Med Phys, Kermanshah, Iran; [Farshchian, Negin] Kermanshah Univ Med Sci, Fac Med, Dept Radiat Oncol, Kermanshah, Iran; [Farshchian, Negin; Haghparast, Abbas] Imam Reza Hosp, Clin Res Dev Ctr, Kermanshah, Iran	Kermanshah University of Medical Sciences; Kermanshah University of Medical Sciences
Haghparast, A (corresponding author), Parastar Blvd,POB 6714415333, Kermanshah, Iran.	a.haghparast@kums.ac.ir
Heydarheydari, Sahel/N-2393-2014	Research Council of Kermanshah University of Medical Sciences; Clinical Research Development Center, Imam Reza Hospital	Research Council of Kermanshah University of Medical Sciences; Clinical Research Development Center, Imam Reza Hospital	The authors gratefully acknowledge the Research Council of Kermanshah University of Medical Sciences and Clinical Research Development Center, Imam Reza Hospital for their financial support and cooperation. This work (approved research plan No: 93156) was performed in partial fulfillment of the requirements for Med. Phys. D. of Mostafa Shirkhani, Faculty of Medicine, Kermanshah University of Medical Sciences, Kermanshah, Iran.	19	3	3	0	3
SHIRAZ UNIV MEDICAL SCIENCES	SHIRAZ	NEMAZEE HOSPITAL, SHIRAZ, 71934, IRAN	2008-6709	2008-6687	MIDDLE EAST J CANCER	Middle East J. Cancer	JAN	2020	11	1
99	104	6
Oncology	Emerging Sources Citation Index (ESCI)	Oncology
JZ1AF	2025-06-24	WOS:000504837000011
J	Bevanda, D; Racetin, A; Kelam, N; Filipovic, N; Bevanda, M; Dimlic, MR; Budimir, J; Glibo, DB; Bevanda, I; Ramljak, D; Vukojevic, K	Bevanda, Danijel; Racetin, Anita; Kelam, Nela; Filipovic, Natalija; Bevanda, Mateo; Rudan Dimlic, Marina; Budimir, Jelena; Bevanda Glibo, Daniela; Bevanda, Ivana; Ramljak, Danica; Vukojevic, Katarina
Expression Pattern of AIFM3, VGLL4, and WNT4 in Patients with Different Stages of Colorectal Cancer	CANCERS
English	Article
colorectal cancer; AIFM3; VGLL4; WNT4	MICROSATELLITE INSTABILITY	Background/Objectives: Colorectal cancer (CRC) remains a significant health burden, and its delayed diagnosis at advanced stages leads to poor survival outcome. Detection of known and novel prognostic markers is essential. In this study, the status

of likely prognostic markers-the apoptotic inducing factor (AIFM3), vestigial-like family member 4 (VGLL4), and WNT4-was evaluated. Methods: AIFM3, VGLL4, and WNT4 expression in CRC tissues across different stages (Dukes A-D) were analyzed using histological immunofluorescence staining and RNA sequencing analyses. Results: In advanced CRC stages, progressive loss of normal crypt architecture, reduction of goblet cells, and necrotic debris were detected along with differential expression patterns of AIFM3, VGLL4, and WNT4. AIFM3 exhibited high reactivity in the lamina propria of healthy tissue and Dukes A, but this was diminished in advanced CRC stages. VGLL4 expression, initially confined to the lamina propria, increased significantly in the epithelium of Dukes B and C, with a cytoplasmic localization pattern. WNT4 expression was elevated in the CRC epithelium across all stages, contrasting with a significant reduction in lamina propria reactivity. RNA sequencing corroborated these findings, showing significant downregulation of AIFM3 and WNT4 and upregulation of VGLL4 in CRC tissues compared to controls. Expression of AIFM3 and WNT4 showed no correlation with survival outcome, while low VGLL4 expression was correlated with better survival outcome. Conclusions: The results suggest distinct roles for AIFM3, VGLL4, and WNT4 in CRC progression, highlighting only VGLL4 as a potential prognostic marker. Further evaluation of VGLL4 and its specific role in CRC progression remains to be elucidated.[Bevanda, Danijel; Bevanda Glibo, Daniela] Univ Mostar, Univ Hosp Mostar, Sch Med, Dept Gastroenterol, Bijeli Brijeg Bb, Mostar 88000, Bosnia & Herceg; [Racetin, Anita; Kelam, Nela; Filipovic, Natalija; Vukojevic, Katarina] Univ Split, Dept Anat Histol & Embryol, Lab Early Human Dev, Sch Med, Soltanska 2A, Split 21000, Croatia; [Bevanda, Mateo] Univ Mostar, Univ Hosp Mostar, Sch Med, Dept Surg, Bijeli Brijeg Bb, Mostar 88000, Bosnia & Herceg; [Rudan Dimlic, Marina; Budimir, Jelena; Ramljak, Danica; Vukojevic, Katarina] Univ Split, Mediterranean Inst Life Sci MedILS, Mestrovicevo Setaliste 45, Split 21000, Croatia; [Bevanda, Ivana] Univ Mostar, Univ Hosp Mostar, Sch Med, Dept Endocrinol, Bijeli Brijeg Bb, Mostar 88000, Bosnia & Herceg; [Vukojevic, Katarina] Univ Split, Ctr Translat Res Biomed, Sch Med, Soltanska 2A, Split 21000, Croatia   University of Mostar; University of Split; University of Mostar; University of Split; University of Mostar; University of Split Vukojevic, K (corresponding author), Univ Split, Dept Anat Histol & Embryol, Lab Early Human Dev, Sch Med, Soltanska 2A, Split 21000, Croatia.; Vukojevic, K (corresponding author), Univ Split, Mediterranean Inst Life Sci MedILS, Mestrovicevo Setaliste 45, Split 21000, Croatia.; Vukojevic, K (corresponding author), Univ Split, Ctr Translat Res Biomed, Sch Med, Soltanska 2A, Split 21000, Croatia.

danijel.bevanda@gmail.com; anita.racetin@mefst.hr; nela.kelam@mefst.hr; natalija.filipovic@mefst.hr; bevanda333@gmail.com; mrdimlic@medils.unist.hr; jbudimir@medils.unist.hr; ela.bevanda@gmail.com; bjelanovic.ivanaaa@yahoo.com; dramljak@medils.unist.hr; katarina.vukojevic@mefst.hr Filipovic, Natalija/D-4964-2017; Vukojevic, Katarina/AAU-1906-2020; Kelam, Nela/KFR-9517-2024; Vukojevic, Katarina/D-5246-2017   Kelam, Nela/0000-0002-6529-5474; Budimir, Jelena/0000-0001-5411-5530; Vukojevic, Katarina/0000-0003-2182-2890; Filipovic, Natalija/0000-0002-8943-4109; Rudan Dimlic, Marina/0009-0004-8480-3616; Bevanda, Mateo/0000-0002-2121-4302 Croatian Science Foundation; [IP-2022-10-8720]   Croatian

Science Foundation; This research was funded by the Croatian Science Foundation, grant number IP-2022-10-8720. 32 1 1 0 0 MDPI BASEL ST ALBAN-ANLAGE 66, CH-4052 BASEL, SWITZERLAND 2072-6694 CANCERS Cancers JAN 2025 17 2 166 10.3390/cancers17020166 http://dx.doi.org/10.3390/cancers17020166 18 Oncology Science Citation Index Expanded (SCI-EXPANDED) Oncology T3B0I 39857952 gold, Green Published 2025-06-24 WOS:001403792500001

J Madani, SH; Payandeh, M; Sadeghi, M; Motamed, H; Sadeghi, E Madani, Seyed-Hamid; Payandeh, Mehrdad; Sadeghi, Masoud; Motamed, Hajar; Sadeghi, Edris The correlation between Ki-67 with other prognostic factors in breast cancer: A study in Iranian patients INDIAN JOURNAL OF MEDICAL AND PAEDIATRIC ONCOLOGY English Article Breast cancer; histological grade; Ki-67; P53 PROLIFERATION MARKERS; KI67; P53; ASSOCIATION; EXPRESSION; CARCINOMA; TUMOR; METAANALYSIS; FRACTION; INDEX

Context: Despite the fact that breast cancer (BC) is a major health issue, very few studies describe its characteristics in the Middle East. Aim: The aim of this study was to evaluate the use and value of Ki-67 as a prognostic marker in BC and associations between Ki-67, clinical, and histopathological parameters were evaluated. Subjects and Methods: In a retrospective study, 260 BC women and invasive ductal carcinoma were included to our study in Kermanshah city, Iran. Age, tumor size, lymph node involvement, histological grade, nuclear grade, and vascular invasion were other factors that determined in a lot of patients. Results: The mean age at diagnosis was 47.6 years (range, 24-84 years) with 100% female. Of 243 patients that tumor size was determined for them, 207 patients (85.2%) had tumor size >= 2 cm, and 36 patients (14.8%) had size <2 cm and also of 237 patients, 47 patients (19.8%), 140 (59.1%), and 50 (21.1%) had histological grades I, II, and III, respectively. There is significant correlation between Ki-67 with nuclear grade, human epidermal growth factor receptor 2 (HER2), and p53 (P < 0.05). Based on this result, more patients with Ki-67 >= 20% have higher nuclear grade, p53-positive, and HER2-positive. There was correlation between Ki-67 with type of tumor (P = 0.009). Conclusions: The higher Ki-67 has a direct significant correlation with higher nuclear grade, p53-positive, and HER2-positive. Furthermore, triple negative patients have higher Ki-67 compared to other subtypes. [Madani, Seyed-Hamid; Motamed, Hajar] Kermanshah Univ Med Sci, Imam Reza Hosp, Mol Pathol Res Ctr, Kermanshah, Iran; [Payandeh, Mehrdad] Kermanshah Univ Med Sci, Dept Hematol & Med Oncol, Kermanshah, Iran; [Sadeghi, Masoud; Sadeghi, Edris] Kermanshah Univ Med Sci, Med Biol Res Ctr, Kermanshah, Iran Kermanshah University of Medical Sciences; Kermanshah University of Medical Sciences; Kermanshah University of Medical Sciences Sadeghi, M (corresponding author), Kermanshah Univ Med Sci, Med Biol Res Ctr, Kermanshah, Iran. Sadeghi_mbrc@yahoo.com Sadeghi, Edris/O-2936-2015; Madani, Seyed/R-9322-2017; Motamed, Hajar/I-2011-2018; Sadeghi, Masoud/P-1546-2015; Payandeh, Mehrdad/N-1029-2017 Sadeghi, Masoud/0000-0002-3586-3012; Madani, Seyed Hamid/0000-0001-9435-4579; Sadeghi, Edris/0000-0001-7742-9372; Motamed, Hajar/0000-0002-4419-0497; Payandeh, Mehrdad/0000-0003-0074-7166 24 13 14 0 0

THIEME MEDICAL PUBL INC NEW YORK 333 SEVENTH AVE, NEW YORK, NY 10001 USA 0971-5851 0975-2129 INDIAN J MED PAEDIAT Indian J. Med. Paediatr. Oncol. APR-JUN 2016 37 2 95 99 10.4103/0971-5851.180136 http://dx.doi.org/10.4103/0971-5851.180136
5 Oncology Emerging Sources Citation Index (ESCI)
Oncology DX3ZW 27168707 Green Published, gold
2025-06-24 WOS:000384318100007
J Panagopoulou, M; Karaglani, M; Manolopoulos, VG; Iliopoulos, I; Tsamardinos, I; Chatzaki, E Panagopoulou, Maria; Karaglani, Makrina; Manolopoulos, Vangelis G.; Iliopoulos, Ioannis; Tsamardinos, Ioannis; Chatzaki, Ekaterini
Deciphering the Methylation Landscape in Breast Cancer: Diagnostic and Prognostic Biosignatures through Automated Machine Learning CANCERS English Article
breast cancer; methylation; machine learning; signature; predictive model; bioinformatics; pathway; transcription DNA METHYLATION; COMPREHENSIVE ANALYSIS; CELL-PROLIFERATION; TISSUE INHIBITOR; GROWTH; EXPRESSION; PREDICTION; SIGNATURE; GENE; AIM2
Simple Summary Breast cancer (BrCa) is characterized by aberrant DNA methylation. We leveraged high-throughput methylation data from BrCa and normal breast tissues and identified 11,176 to 27,786 differentially methylated genes (DMGs) against clinically relevant end-points. Innovative automated machine learning was employed to construct three highly performing signatures for (1) the discrimination of BrCa patients from healthy individuals, (2) the identification of BrCa metastatic disease and (3) the early diagnosis of BrCa. Furthermore, functional analysis revealed that most genes selected in the signatures showed associations to BrCa, with regulation of transcription being the main biological process, the nucleus being the main cellular component and transcription factor activity and sequence-specific DNA binding being the main molecular functions. Overall, revisiting methylome datasets led to three high-performance signatures that are readily available for improving BrCa precision management and significant knowledge mining related to disease pathophysiology. DNA methylation plays an important role in breast cancer (BrCa) pathogenesis and could contribute to driving its personalized management. We performed a complete bioinformatic analysis in BrCa whole methylome datasets, analyzed using the Illumina methylation 450 bead-chip array. Differential methylation analysis vs. clinical end-points resulted in 11,176 to 27,786 differentially methylated genes (DMGs). Innovative automated machine learning (AutoML) was employed to construct signatures with translational value. Three highly performing and low-feature-number signatures were built: (1) A 5-gene signature discriminating BrCa patients from healthy individuals (area under the curve (AUC): 0.994 (0.982-1.000)). (2) A 3-gene signature identifying BrCa metastatic disease (AUC: 0.986 (0.921-1.000)). (3) Six equivalent 5-gene signatures diagnosing early disease (AUC: 0.973 (0.920-1.000)). Validation in independent patient groups verified performance. Bioinformatic tools for functional analysis and protein interaction prediction were also employed. All protein encoding features included in the signatures were associated with BrCa-related pathways. Functional analysis of DMGs highlighted the regulation of transcription as the main biological process, the nucleus as the main cellular component and transcription factor activity and sequence-specific DNA binding as

the main molecular functions. Overall, three high-performance diagnostic/prognostic signatures were built and are readily available for improving BrCa precision management upon prospective clinical validation. Revisiting archived methylomes through novel bioinformatic approaches revealed significant clarifying knowledge for the contribution of gene methylation events in breast carcinogenesis. [Panagopoulou, Maria; Karaglani, Makrina; Manolopoulos, Vangelis G.; Chatzaki, Ekaterini] Democritus Univ Thrace, Med Sch, Lab Pharmacol, GR-68100 Alexandroupolis, Greece; [Iliopoulos, Ioannis] Univ Crete, Sch Med, Dept Basic Sci, GR-71003 Iraklion, Greece; [Tsamardinos, Ioannis] Gnosis Data Anal PC, JADBio, Sci & Technol Pk Crete, GR-70013 Iraklion, Greece; [Tsamardinos, Ioannis] Univ Crete, Dept Comp Sci, GR-70013 Iraklion, Greece; [Tsamardinos, Ioannis] Fdn Res & Technol Hellas, Inst Appl & Computat Math, GR-70013 Iraklion, Greece; [Chatzaki, Ekaterini] Hellenic Mediterranean Univ Res Ctr, Inst Agrifood & Life Sci, GR-71410 Iraklion, Greece Democritus University of Thrace; University of Crete; University of Crete; Foundation for Research & Technology - Hellas (FORTH) Chatzaki, E (corresponding author), Democritus Univ Thrace, Med Sch, Lab Pharmacol, GR-68100 Alexandroupolis, Greece.; Chatzaki, E (corresponding author), Hellenic Mediterranean Univ Res Ctr, Inst Agrifood & Life Sci, GR-71410 Iraklion, Greece. mpanagop@med.duth.gr; mkaragla@med.duth.gr; emanolop@med.duth.gr; iliopj@med.uoc.gr; tsamard@csd.uoc.gr; achatzak@med.duth.gr Chatzaki, Ekaterini/CAF-7645-2022; Karaglani, Makrina/JWO-3435-2024; Panagopoulou, Maria/KUD-6509-2024; Manolopoulos, Vangelis/J-3312-2015 Panagopoulou, Maria/0000-0002-7107-4774; Chatzaki, Ekaterini/0000-0002-5832-4257; Manolopoulos, Vangelis/0000-0003-0849-5469; Karaglani, Makrina/0000-0002-9853-3571 project "DNA methylation as a minimally-invasive biomarker: development and validation of classifiers with prognostic and/or predictive clinical value in breast cancer therapy" under the call for proposals EDULLL 103 [MIS 5049913]; European Union (European Social Fund-ESF) by the Operational Programme Human Resources Development, Education and Lifelong Learning 2014-2020 project "DNA methylation as a minimally-invasive biomarker: development and validation of classifiers with prognostic and/or predictive clinical value in breast cancer therapy" under the call for proposals EDULLL 103; European Union (European Social Fund-ESF) by the Operational Programme Human Resources Development, Education and Lifelong Learning 2014-2020 This research was carried out and funded in the context of the project "DNA methylation as a minimally-invasive biomarker: development and validation of classifiers with prognostic and/or predictive clinical value in breast cancer therapy" (MIS 5049913) under the call for proposals EDULLL 103. The project was co-financed by Greece and the European Union (European Social Fund-ESF) by the Operational Programme Human Resources Development, Education and Lifelong Learning 2014-2020. 77 25 25 0 7 MDPI BASEL MDPI AG, Grosspeteranlage 5, CH-4052 BASEL, SWITZERLAND 2072-6694 CANCERS Cancers APR 2021 13 7 1677 10.3390/cancers13071677 http://dx.doi.org/10.3390/cancers13071677 21 Oncology Science Citation Index Expanded (SCI-EXPANDED) Oncology RK5DY 33918195 gold, Green Published 2025-06-24 WOS:000638318000001

J	Mannoh, I; Turkson-Ocran, RA; Mensah, J; Mensah, D; Yi, SS; Michos, ED; Commodore-Mensah, Y	Mannoh, Ivy; Turkson-Ocran, Ruth-Alma; Mensah, Jasmine; Mensah, Danielle; Yi, Stella S.; Michos, Erin D.; Commodore-Mensah, Yvonne
Disparities in Awareness of Myocardial Infarction and Stroke Symptoms and Response Among United States- and Foreign-Born Adults in the National Health Interview Survey JOURNAL OF THE AMERICAN HEART ASSOCIATION	English	Article
cardiovascular disease; disparities; health education; heart attack; immigrants; stroke	HEART-ATTACK
Background Atherosclerotic cardiovascular disease, defined as nonfatal myocardial infarction (MI), coronary heart disease death, or fatal or nonfatal stroke, is the leading cause of death in the United States. MI and stroke symptom awareness and response reduce delays in hospitalization and mortality. Methods and Results We analyzed cross-sectional data from the 2014 and 2017 National Health Interview Surveys on US- and foreign-born adults from 9 regions of birth (Europe, South America, Mexico/Central America/Caribbean, Russia, Africa, Middle East, Indian subcontinent, Asia, and Southeast Asia). The outcomes were recommended MI and stroke knowledge, defined as knowing all 5 symptoms of MI or stroke, respectively, and choosing "call 9-1-1" as the best response. We included 63 059 participants, with a mean age 49.4 years; 54.1% were women, and 38.5% had a high school education or less. Recommended MI and stroke knowledge were highest in US-born people. In both 2014 and 2017, MI knowledge was lowest in individuals born in Asia (23.9%+/- 2.5% and 32.1%+/- 3.3%, respectively), and stroke knowledge lowest for the Indian subcontinent (44.4%+/- 2.4% and 46.0%+/- 3.2%, respectively). Among foreign-born adults, people from Russia and Europe had the highest prevalence of recommended MI knowledge in 2014 (37.4%+/- 5.4%) and 2017 (43.5%+/- 2.5%), respectively, and recommended stroke knowledge was highest in people from Europe (61.0%+/- 2.6% and 67.2%+/- 2.5%). Improvement in knowledge was not significant in all groups between 2014 and 2017. Conclusions These findings suggest a disparity in MI and stroke symptom awareness and response among immigrants in the United States. Culturally tailored public health education and health literacy initiatives are needed to help reduce these disparities in awareness.
[Mensah, Jasmine; Commodore-Mensah, Yvonne] Johns Hopkins Univ, Sch Nursing, 525 N Wolfe St, Baltimore, MD 21205 USA; [Mannoh, Ivy; Turkson-Ocran, Ruth-Alma; Michos, Erin D.] Johns Hopkins Univ, Sch Med, Baltimore, MD 21205 USA; [Mensah, Danielle] Drexel Univ, Coll Med, Philadelphia, PA 19104 USA; [Yi, Stella S.] NYU, Grossman Sch Med, New York, NY USA	Johns Hopkins University; Johns Hopkins University; Drexel University; New York University
Commodore-Mensah, Y (corresponding author), Johns Hopkins Univ, Sch Nursing, 525 N Wolfe St, Baltimore, MD 21205 USA.
ycommod1@jhu.edu	Commodore-Mensah, Yvonne/AAP-3381-2020; Turkson-Ocran, Ruth-Alma/L-3421-2017	Turkson-Ocran, Ruth-Alma/0000-0001-9932-052X; Commodore-Mensah, Yvonne/0000-0002-5054-3025; Michos, Erin/0000-0002-5547-5084
National Institutes of Health (NIH) National Institute on Minority Health and Health Disparities [U54MD000538]; National Heart, Lung and Blood Institute [R01HL141427]; National Heart Lung and Blood Institute [R01HL141427] Funding Source: NIH RePORTER; National Institute on Minority Health and Health Disparities [U54MD000538] Funding Source: NIH RePORTER	National Institutes of Health (NIH)

National Institute on Minority Health and Health Disparities; National Heart, Lung and Blood Institute(United States Department of Health & Human ServicesNational Institutes of Health (NIH) - USANIH National Heart Lung & Blood Institute (NHLBI)); National Heart Lung and Blood Institute(United States Department of Health & Human ServicesNational Institutes of Health (NIH) - USANIH National Heart Lung & Blood Institute (NHLBI)); National Institute on Minority Health and Health Disparities(United States Department of Health & Human ServicesNational Institutes of Health (NIH) - USANIH National Institute on Minority Health & Health Disparities (NIMHD)) Dr. Yi is supported by grants U54MD000538 from the National Institutes of Health (NIH) National Institute on Minority Health and Health Disparities, and R01HL141427 from the National Heart, Lung and Blood Institute. 30 6 6 0 3
WILEY HOBOKEN 111 RIVER ST, HOBOKEN 07030-5774, NJ USA
2047-9980 J AM HEART ASSOC J. Am. Heart Assoc.
DEC 7 2021 10 23
e020396 10.1161/JAHA.121.020396
http://dx.doi.org/10.1161/JAHA.121.020396 16
Cardiac & Cardiovascular Systems Science Citation Index Expanded (SCI-EXPANDED) Cardiovascular System & Cardiology
XK4BF 34845927 Green Published, gold 2025-06-24 WOS:000727412400047
J Fouzat, A; Hussein, OJ; Gupta, I; Al-Farsi, HF; Khalil, A; Al Moustafa, AE Fouzat, Arij; Hussein, Ola Jihad; Gupta, Ishita; Al-Farsi, Halema F.; Khalil, Ashraf; Al Moustafa, Ala-Eddin Elaeagnus angustifolia Plant Extract Induces Apoptosis via P53 and Signal Transducer and Activator of Transcription 3 Signaling Pathways in Triple-Negative Breast Cancer Cells FRONTIERS IN NUTRITION English
Article Elaeagnus angustifolia; triple-negative breast cancer; apoptosis; stat3; p53
NATURAL-PRODUCTS; STAT3; RESISTANCE; DISCOVERY; PATTERNS; TARGET; L. Elaeagnus angustifolia (EA) is used as an alternative medicine in the Middle East to manage numerous human diseases. We recently reported that EA flower extract inhibits cell proliferation and invasion of human oral and HER2-positive breast cancer cells. Nevertheless, the outcome of EA extract on triple-negative breast cancer (TNBC) cells has not been explored yet. We herein investigate the effect of the aqueous EA extract (100 and 200 mu l/ml) on two TNBC cell lines (MDA-MB-231 and MDA-MB-436) for 48 h and explore its underlying molecular pathways. Our data revealed that EA extract suppresses cell proliferation by approximately 50% and alters cell-cycle progression of these two cancer cell lines. Additionally, EA extract induces cell apoptosis by 40-50%, accompanied by the upregulation of pro-apoptotic markers (Bax and cleaved caspase-8) and downregulation of the anti-apoptotic marker, Bcl-2. Moreover, EA extract inhibits colony formation compared to their matched control. More significantly, the molecular pathway analysis of EA-treated cells revealed that EA extract enhances p53 expression, while inhibiting the expression of total and phosphorylated Signal Transducer and Activator Of Transcription 3 (STAT3) in both cell lines, suggesting p53 and STAT3 are the main key players behind the biological events provoked by the extract in TNBC cells. Our findings implicate that EA flower extract may possess an important potential as an anticancer drug against TNBC. [Fouzat, Arij; Hussein, Ola Jihad; Khalil, Ashraf] Qatar Univ, Coll Pharm, QU

Hlth, Doha, Qatar; [Gupta, Ishita; Al-Farsi, Halema F.; Al Moustafa, Ala-Eddin] Qatar Univ, Coll Med, QU Hlth, Doha, Qatar; [Al Moustafa, Ala-Eddin] Qatar Univ, Biomed Res Ctr, Doha, Qatar
Qatar University; Qatar University; Qatar University Al Moustafa, AE (corresponding author), Qatar Univ, Coll Med, QU Hlth, Doha, Qatar.; Al Moustafa, AE (corresponding author), Qatar Univ, Biomed Res Ctr, Doha, Qatar. ala-eddin.almoustafa@mcgill.ca hussein, ola/GQI-4091-2022 Hussein, Ola/0000-0001-7213-9077; Hassan, Arij/0000-0002-2012-4153
49 14 14 2 10 FRONTIERS MEDIA SA LAUSANNE AVENUE DU TRIBUNAL FEDERAL 34, LAUSANNE, CH-1015, SWITZERLAND 2296-861X FRONT NUTR Front. Nutr. MAR 18 2022 9 871667
10.3389/fnut.2022.871667
http://dx.doi.org/10.3389/fnut.2022.871667 11
Nutrition & Dietetics Science Citation Index Expanded (SCI-EXPANDED) Nutrition & Dietetics 0H2UY 35369073 gold, Green Published 2025-06-24 WOS:000778593600001
J Nouri, R; Schroeder, J; Champiri, BM; Akochakian, M
Nouri, Reza; Schroeder, Jan; Champiri, Bahareh Mahmudieh; Akochakian, Mahdieh Effect of Six-Week Resistance Training with Thera-Band and Combined Training on Static and Dynamic Balance in Breast Cancer Survivors: A Randomized Clinical Controlled Trial MIDDLE EAST JOURNAL OF CANCER English Article
Resistance training; Core stability training; Breast cancer EXERCISE PROGRAM; POSTURAL CONTROL; WOMEN; ADULTS; REHABILITATION; INSTABILITY; PREVENTION; INJURY; ERROR; MODEL
Background: Balance impairment is related to breast cancer treatments, such as radiation therapy and chemotherapy. The aim of the present study was to investigate the effect of six-week resistance training with Thera-Band and combined training (training with Thera-Band and core stability training) on static and dynamic balance in breast cancer survivors. Methods: In this randomized controlled trail, we divided 75 patients with breast cancer into three groups: 1) resistance training group (n=25, age= 46 +/- 5.82, BMI= 25.7 +/- 3.9), combined training group, (n=25, age= 48.9 +/- 7.06, BMI 24.6 +/- 3.5), and control group (n=25, age= 46 +/- 7.15, BMI= 25.17 +/- 3.7). Afterwards, we employed Single leg standing and Balance Error Scoring System (BESS) to measure the static balance; Four Square Step Test (FSST) and Timed Up and Go (TUG) were further used for dynamic balance in pre- and post-test. Exercise program was performed by two training groups for six weeks and three sessions per week. Resistance training with Thera-Band group performed 13 resistance trainings with Them-Band. In combined group, resistance training was similar to Thera-Band group, but combined group were performed seven core stability trainings. We analyzed the data by two-way repeated measurements ANOVA using the software package SPSS V.22. Results: Results showed that there were no significant interaction effects indicating an exercise specific development, depending on the type of exercise or control condition P>0.05). Furthermore, there were no significant deference among the resistance training with Thera-Band, the combined exercise, and the control groups (P>0.05). Conclusion: It can be concluded that the six-week resistance training with Thera-Band and combined training (training with Thera-Band and core stability training) has no positive effects on static and dynamic balance in breast cancer survivors. We

recommend that more studies be conducted in this regard.[Nouri, Reza; Champiri, Bahareh Mahmudieh; Akochakian, Mahdieh] Univ Tehran, Dept Sport Sci, Kish Int Campus,Mirmohana Blvd, Kish Island, Iran; [Schroeder, Jan] Univ Hamburg, Fac Psychol & Human Movement Sci, Dept Sports & Exercise Med, Hamburg, Germany
University of Hamburg Nouri, R (corresponding author), Univ Tehran, Dept Sport Sci, Kish Int Campus,Mirmohana Blvd, Kish Island, Iran. nuri_r7@ut.ac.ir Schroeder, Jan/Z-2082-2019; Akoochakian, Mahdieh/HKM-6688-2023 Akoochakian, Mahdieh/0000-0003-3214-5135; Schroeder, Jan/0000-0001-9484-1334
33 1 1 1 11 SHIRAZ UNIV MEDICAL SCIENCES SHIRAZ NEMAZEE HOSPITAL, SHIRAZ, 71934, IRAN
2008-6709 2008-6687 MIDDLE EAST J CANCER Middle East J. Cancer JUL 2020 11 3 343
350 10.30476/mejc.2020.81438.1007
http://dx.doi.org/10.30476/mejc.2020.81438.1007
8 Oncology Emerging Sources Citation Index (ESCI)
Oncology ML5EZ 2025-06-24
WOS:000549490400011
J Haghnavaz, N; Asghari, F; Shekari, N; Shanehbandi, D; Javadian, M; Mohammadi, A; Baradaran, B; Kazemi, T
Haghnavaz, Navideh; Asghari, Faezeh; Shekari, Najibeh; Shanehbandi, Dariush; Javadian, Mahsa; Mohammadi, Ali; Baradaran, Behzad; Kazemi, Tohid Paclitaxel May Inhibit Epithelial-Mesenchymal Transition Properties of Triple-negative Breast Cancer Cell Line via Altering the Expression of EMT-promoting and -inhibiting MicroRNAs MIDDLE EAST JOURNAL OF CANCER English Article
Breast cancer; MiR-199a-5p; MiR-10b; Paclitaxel; Vimentin; MMP-9 E-CADHERIN; METASTASIS; INVASION Background: Abnormal expressions of microRNAs are related to various cancers such as breast cancer for which paclitaxel is widely used as a chemotherapeutic agent. We aimed to investigate the effect of paclitaxel treatment on the expression level of miR-199a-5p and miR-10b, involved in epithelial-mesenchymal transition (EMT) process in breast cancer cell lines. Methods: Human breast cancer cell lines BT-474, SKBR-3, MDA-MB-231, and MCF-7 were cultured and MTT assay was used to determine IC50 of paclitaxel. RNA was extracted, cDNA was synthesized, and the expression level of miRNAs and genes was quantitatively determined using real-time PCR. Results: After treatment with paclitaxel, the expression level of miR-199a-5p significantly decreased in MCF-7 and SKBR-3 cell lines, while it increased in MDAMB-231 and BT-474. The expression level of miR-10b was also significantly reduced in MCF-7, MDA-MB-231, and SKBR-3 and increased in BT-474 cell lines following treatment with paclitaxel. Our results further indicated that paclitaxel reduced the expression level of vimentin and MMP-9 in MDA-MB-231 cell line. Conclusion: Our findings revealed the increased expression of EMT-inhibitor miR-199a-5p and the decreased expression of metastamir miR-10b after treatment of MDA-MB-231 metastatic breast cancer cell line. Reduced expressions of vimentin and MMP-9 were also observed, corroborating the inhibition of metastasis markers in this type of breast cancer. The therapeutic effect of paclitaxel may in part be due to the change in the balance of EMT-promoting and EMT-inhibiting miRNAs.
[Haghnavaz, Navideh] Tabriz Univ Med Sci, Drug Appl Res Ctr, Tabriz, Iran; [Haghnavaz, Navideh; Shekari, Najibeh; Shanehbandi, Dariush; Javadian, Mahsa; Mohammadi, Ali; Baradaran, Behzad;

Kazemi, Tohid] Tabriz Univ Med Sci, Immunol Res Ctr, Tabriz, Iran; [Haghnavaz, Navideh] Tabriz Univ Med Sci, Student Res Comm, Tabriz, Iran; [Asghari, Faezeh] Tabriz Univ Med Sci, Infect & Trop Dis Res Ctr, Tabriz, Iran	Tabriz University of Medical Science; Tabriz University of Medical Science; Tabriz University of Medical Science; Tabriz University of Medical Science	Kazemi, T (corresponding author), Tabriz Univ Med Sci, Fac Med, Dept Immunol, Tabriz, Iran.	kazemit@tbzmed.ac.ir	Shanehbandi, Dariush/H-2502-2013; Shekari, Najibeh/ABI-4798-2020; Kazemi, Tohid/AAF-2444-2019; Baradaran, Behzad/AAQ-5177-2020	Shekari, Najibeh/0000-0003-3139-1035; baradaran, behzad/0000-0002-8642-6795; Shanehbandi, Dariush/0000-0002-9449-0607	Ministry of Health and Medical Education, Islamic Republic of Iran	Ministry of Health and Medical Education, Islamic Republic of Iran	This work was supported by a grant from the Ministry of Health and Medical Education, Islamic Republic of Iran. The authors would like to thank Behzad Mansoori and Samira Goldar.	38	1	1	0	4	SHIRAZ UNIV MEDICAL SCIENCES	SHIRAZ	NEMAZEE HOSPITAL, SHIRAZ, 71934, IRAN	2008-6709	2008-6687		MIDDLE EAST J CANCER	Middle East J. Cancer	JAN	2020	11	1			42	49		8	Oncology	Emerging Sources Citation Index (ESCI)	Oncology	JZ1AF		2025-06-24	WOS:000504837000005
J	Ungvari, Z; Fekete, M; Fekete, JT; Grosso, G; Ungvari, A; Gyorffy, B		Ungvari, Zoltan; Fekete, Monika; Fekete, Janos Tibor; Grosso, Giuseppe; Ungvari, Anna; Gyorffy, Balazs		Adherence to the Mediterranean diet and its protective effects against colorectal cancer: a meta-analysis of 26 studies with 2,217,404 participants	GEROSCIENCE		English	Article; Early Access		Healthy aging; Dietary intervention; Malignant disease; Malignancies; Tumor; Unhealthy aging	CEREBROMICROVASCULAR ENDOTHELIAL-CELLS; CARDIOVASCULAR RISK-FACTORS; VASCULAR OXIDATIVE STRESS; CELLULAR SENESCENCE; SEX-DIFFERENCES; COENZYME Q(10); ELDERLY-MEN; OLIVE-OIL; QUALITY; CONSUMPTION	Colorectal cancer (CRC) is a major global health concern and represents a significant public health challenge in Hungary, where it exhibits some of the highest morbidity and mortality rates in the European Union. The Mediterranean diet has been suggested to reduce the incidence of CRC, but comprehensive evidence from diverse study designs is needed to substantiate this effect. A systematic literature search was conducted in PubMed, ClinicalTrials.gov, CENTRAL, and the Web of Science to identify randomized controlled trials and human clinical trials from 2008 to 2024 to identify relevant studies. Statistical analysis was performed using the https://metaanalysisonline.com web application using a random effects model to estimate the pooled hazard rates (HRs). Forest plots, funnel plots, and Z-score plots were utilized to visualize results. We identified 15 clinical trials and 9 case-control studies, encompassing a total of 2,217,404 subjects. The pooled analysis indicated that adherence to the Mediterranean diet significantly reduced the prevalence of CRC (HR = 0.84, 95% CI = 0.78-0.91, p < 0.01). This protective effect was consistent across sexes, with HRs of 0.85 (95% CI = 0.75-0.97, p = 0.01) for males and 0.88 (95% CI = 0.79-0.99, p = 0.03) for females. Case-control studies specifically showed a substantial effect (HR = 0.51, 95% CI = 0.38-0.68, p < 0.01). Notable heterogeneity was observed

across studies, yet the a priori information size was substantially below the cumulative sample size, ensuring sufficient data for reliable conclusions. The findings from this meta-analysis reinforce the protective role of the Mediterranean diet against CRC. The results of this meta-analysis will inform dietary interventions designed to mitigate CRC risk, which are conducted within the framework of the Semmelweis Study, an ongoing comprehensive cohort study at Semmelweis University, designed to explore the multifaceted causes of unhealthy aging in Hungary. These interventions aim to explore the practical application of Mediterranean dietary patterns in reducing CRC incidence among the Hungarian population. [Ungvari, Zoltan] Univ Oklahoma, Dept Neurosurg,Hlth Sci Ctr, Neurodegenerat & Hlth Brain Aging Program, Vasc Cognit Impairment, Oklahoma City, OK USA; [Ungvari, Zoltan] Univ Oklahoma, Stephenson Canc Ctr, Oklahoma City, OK USA; [Ungvari, Zoltan] Univ Oklahoma, Hlth Sci Ctr, Oklahoma Ctr Geroscience & Hlth Brain Aging, Oklahoma City, OK USA; [Ungvari, Zoltan] Univ Oklahoma, Coll Publ Hlth, Hlth Sci Ctr, Dept Hlth Promot Sci, Oklahoma City, OK USA; [Ungvari, Zoltan] Semmelweis Univ, Inst Prevent Med & Publ Hlth, Doctoral Coll, Int Training Program Geroscience, Budapest, Hungary; [Fekete, Monika; Ungvari, Anna] Semmelweis Univ, Inst Prevent Med & Publ Hlth, Budapest, Hungary; [Fekete, Janos Tibor; Gyorffy, Balazs] Semmelweis Univ, Dept Bioinformat, H-1094 Budapest, Hungary; [Fekete, Janos Tibor; Gyorffy, Balazs] HUN REN Res Ctr Nat Sci, Inst Mol Life Sci, Canc Biomarker Res Grp, H-1117 Budapest, Hungary; [Grosso, Giuseppe] Univ Catania, Dept Biomed & Biotechnol Sci, Catania, Italy; [Grosso, Giuseppe] Univ Catania, Ctr Human Nutr & Mediterranean Foods NUTREA, Catania, Italy; [Gyorffy, Balazs] Univ Pecs, Med Sch, Dept Biophys, H-7624 Pecs, Hungary University of Oklahoma System; University of Oklahoma Health Sciences Center; University of Oklahoma System; University of Oklahoma Health Sciences Center; University of Oklahoma System; University of Oklahoma Health Sciences Center; University of Oklahoma System; University of Oklahoma Health Sciences Center; Semmelweis University; Semmelweis University; Semmelweis University; HUN-REN; HUN-REN Research Centre for Natural Sciences; University of Catania; University of Catania; University of Pecs Ungvari, A (corresponding author), Semmelweis Univ, Inst Prevent Med & Publ Hlth, Budapest, Hungary.

Ungann2004@gmail.com Ungvari, Zoltan/GZK-8127-2022; monika, fekete/AAE-4135-2020; Gyorffy, Balazs/AAA-9135-2021; Grosso, Giuseppe/K-6730-2016; Fekete, János Tibor/J-4505-2018

Semmelweis University; National Institute on Aging [RF1AG072295, R01AG055395, R01AG068295, R01AG070915]; National Institute of Neurological Disorders and Stroke [R01NS100782]; National Cancer Institute [R01CA255840]; Ministry of Innovation and Technology of Hungary from the National Research, Development and Innovation Fund [TKP2021-NKTA-47]; National Cardiovascular Laboratory Program [RRF-2.3.1-21-2022-00003]; National Laboratory for Drug Research and Development (PharmaLab) by Ministry of Innovation and Technology of Hungary from the National Research, Development and Innovation Fund [RRF-2.3.1-21-2022-00015]; National Research, Development and Innovation Fund of Hungary [135784]; European University for Well-Being (EUniWell) program [101004093 / EUniWell/EAC-A02-2019 / EAC-A02-2019-1]

Semmelweis University; National Institute on Aging(United States Department of Health & Human ServicesNational Institutes of Health (NIH) - USANIH National Institute on Aging (NIA)); National

Institute of Neurological Disorders and Stroke(United States Department of Health & Human ServicesNational Institutes of Health (NIH) - USANIH National Institute of Neurological Disorders & Stroke (NINDS)); National Cancer Institute(United States Department of Health & Human ServicesNational Institutes of Health (NIH) - USANIH National Cancer Institute (NCI)); Ministry of Innovation and Technology of Hungary from the National Research, Development and Innovation Fund; National Cardiovascular Laboratory Program; National Laboratory for Drug Research and Development (PharmaLab) by Ministry of Innovation and Technology of Hungary from the National Research, Development and Innovation Fund; National Research, Development and Innovation Fund of Hungary; European University for Well-Being (EUniWell) program
Open access funding provided by Semmelweis University. This work was supported by grants from the National Institute on Aging (RF1AG072295, R01AG055395, R01AG068295; R01AG070915), the National Institute of Neurological Disorders and Stroke (R01NS100782), the National Cancer Institute (R01CA255840). AU was supported by TKP2021-NKTA-47, implemented with the support provided by the Ministry of Innovation and Technology of Hungary from the National Research, Development and Innovation Fund, financed under the TKP2021-NKTA funding scheme, by funding through the National Cardiovascular Laboratory Program (RRF-2.3.1-21-2022-00003) and by the National Laboratory for Drug Research and Development (PharmaLab, RRF-2.3.1-21-2022-00015) provided by the Ministry of Innovation and Technology of Hungary from the National Research, Development and Innovation Fund, Project no. 135784 implemented with the support provided from the National Research, Development and Innovation Fund of Hungary, financed under the K20 funding scheme and the European University for Well-Being (EUniWell) program (grant agreement number: 101004093 / EUniWell/EAC-A02-2019 / EAC-A02-2019-1). The 4.0 version of ChatGPT, developed by OpenAI, was used as a language tool to refine our writing, enhancing the clarity of our work. 144 12 12 3
7 SPRINGER DORDRECHT VAN GODEWIJCKSTRAAT 30, 3311 GZ DORDRECHT, NETHERLANDS 2509-2715 2509-2723 GEROSCIENCE
GeroScience 2024 AUG 1 2024
10.1007/s11357-024-01296-9
http://dx.doi.org/10.1007/s11357-024-01296-9 AUG 2024
17 Geriatrics & Gerontology Science Citation Index Expanded (SCI-EXPANDED) Geriatrics & Gerontology A5H7Y
39090501 hybrid 2025-06-24
WOS:001282846800001
J Mighri, N; Mejri, N; Boujemaa, M; Berrazega, Y; Rachdi, H; El Benna, H; Labidi, S; Benna, F; Boubaker, S; Boussen, H; Abdelhak, S; Hamdi, Y Mighri, Najah; Mejri, Nesrine; Boujemaa, Maroua; Berrazega, Yosra; Rachdi, Haifa; El Benna, Houda; Labidi, Soumaya; Benna, Farouk; Boubaker, Samir; Boussen, Hamouda; Abdelhak, Sonia; Hamdi, Yosr
Association between epidemiological and clinico-pathological features of breast cancer with prognosis, family history, Ki-67 proliferation index and survival in Tunisian breast cancer patients PLOS ONE English Article
YOUNG AGE; MOLECULAR SUBTYPES; WOMEN; MANAGEMENT; DIAGNOSIS; MARKER; IMPACT; COMORBIDITIES; CARCINOMA; COHORT Breast cancer has different epidemio-clinical characteristics in Middle East and North-African populations compared to those reported in the Western countries. The aim of

this study is to analyze the epidemiological and clinico-pathological features of breast cancer in Tunisia and to determine prognostic factors with special interest to family history, Ki-67 proliferation index and comorbidity. We retrospectively reviewed epidemiological and clinicopathological data from patients' medical records, treated in the Medical Oncology Department at Abderrahmane Mami Hospital, in the period 2011-2015. Data has been collected on 602 breast cancer patients and analyzed using SPSS software V.23.0. Our study showed high fractions of young breast cancer patients and cases with dense breasts. The most prevalent comorbidities observed in the studied cohort were cardiovascular diseases and diabetes. Familial breast cancer was found in 23.3% of cases and was associated with younger age at diagnosis (p<0.001) and advanced stage (p = 0.015). Ki-67 index >20% was significantly associated with early age at diagnosis, lymph node involvement (p = 0.002), advanced tumor grade (p<0.001) and high risk of relapse (p = 0.007). Ki-67 cut-off 30% predicted survival in luminal cases. Survival was worse in patients with triple negative breast cancer compared to non-triple negative breast cancer, inflammatory breast cancer compared to non-inflammatory breast cancer, moderately to poorly differentiated tumors compared to well-differentiated tumors and with positive lymph nodes compared to pN0 (p<0.05). Our study showed new insights into epidemiological and clinico-pathological characteristics of breast cancer that are not well explored in Tunisian population. Considering our findings along with the implementation of electronic health record system may improve patient health care quality and disease management. [Mighri, Najah; Mejri, Nesrine; Boujemaa, Maroua; El Benna, Houda; Labidi, Soumaya; Boubaker, Samir; Boussen, Hamouda; Abdelhak, Sonia; Hamdi, Yosr] Univ Tunis El Manar, Inst Pasteur Tunis, LR20IPT05, Lab Biomed Genom & Oncogenet, Tunis, Tunisia; [Mejri, Nesrine; Berrazega, Yosra; Rachdi, Haifa; El Benna, Houda; Labidi, Soumaya; Boussen, Hamouda] Univ Tunis El Manar, Fac Med Tunis, Abderrahman Mami Hosp, Dept Med Oncol, Tunis, Tunisia; [Benna, Farouk] Univ Tunis, Dept Radiat Oncol, Tunis, Tunisia; [Boubaker, Samir; Hamdi, Yosr] Inst Pasteur Tunis, Lab Human & Expt Pathol, Tunis, Tunisia   Pasteur Network; Universite de Tunis-El-Manar; Institut Pasteur Tunis; Universite de Tunis-El-Manar; Faculte de Medecine de Tunis (FMT); Hopital Abderrahmene Mami; Universite de Tunis; Pasteur Network; Universite de Tunis-El-Manar; Institut Pasteur Tunis   Hamdi, Y (corresponding author), Univ Tunis El Manar, Inst Pasteur Tunis, LR20IPT05, Lab Biomed Genom & Oncogenet, Tunis, Tunisia.; Hamdi, Y (corresponding author), Inst Pasteur Tunis, Lab Human & Expt Pathol, Tunis, Tunisia.   yosr.hamdi@pasteur.tn Hamdi, Yosr/GLT-7117-2022; Boujemaa, Maroua/JVY-7909-2024; Boussen, Hamouda/AAI-2796-2020; Abdelhak, Sonia/L-2831-2013 Abdelhak, Sonia/0000-0001-8466-5525; Hamdi, Yosr/0000-0002-2815-1834

Tunisian Ministry of Health [PEC-4-TUN]; Tunisian Ministry of Higher Education and Scientific Research [LR16IPT05, LR20IPT05]

Tunisian Ministry of Health(Ministry of Public Health in Tunisia); Tunisian Ministry of Higher Education and Scientific Research(Ministry of Higher Education & Scientific Research of Tunisia)   This study was supported by the Tunisian Ministry of Health (PEC-4-TUN) and the Tunisian Ministry of Higher Education and Scientific Research (LR16IPT05 and LR20IPT05). The funders had no role in study design, data collection and analysis, decision to publish, or preparation of the manuscript.   59   10   10

1 3 PUBLIC LIBRARY SCIENCE SAN FRANCISCO 1160 BATTERY STREET, STE 100, SAN FRANCISCO, CA 94111 USA
1932-6203 PLOS ONE PLoS One SEP 12 2022 17
9 e0269732
10.1371/journal.pone.0269732
http://dx.doi.org/10.1371/journal.pone.0269732
17 Multidisciplinary Sciences Science Citation Index Expanded (SCI-EXPANDED) Science & Technology - Other Topics
6R5XR 36094928 Green Published, gold 2025-06-24 WOS:000892376500006
J Guinter, MA; McLain, AC; Merchant, AT; Sandler, DP; Steck, SE Guinter, Mark A.; McLain, Alexander C.; Merchant, Anwar T.; Sandler, Dale P.; Steck, Susan E.
A dietary pattern based on estrogen metabolism is associated with breast cancer risk in a prospective cohort of postmenopausal women INTERNATIONAL JOURNAL OF CANCER English
Article breast cancer; dietary pattern; estrogen metabolism; reduced rank regression
ENDOGENOUS ESTROGENS; MEDITERRANEAN DIET; URINARY ESTROGENS; HEALTH; CONSUMPTION; MICROBIOME; NUTRITION Increased exposure to estrogen is a risk factor for postmenopausal breast cancer, and dietary factors can influence estrogen metabolism. However, studies of diet and breast cancer have been inconclusive. We developed a dietary pattern associated with levels of unconjugated estradiol and the ratio of 2- and 16-hydroxylated estrogen metabolites in a subsample of Prostate, Lung, Colorectal and Ovarian Screening Trial (PLCO) participants (n=653) using reduced rank regression, and examined its association with postmenopausal breast cancer prospectively in the larger PLCO cohort (n=27,488). The estrogen-related dietary pattern (ERDP) was comprised of foods with positively-weighted intakes (non-whole/refined grains, tomatoes, cruciferous vegetables, cheese, fish/shellfish high in -3 fatty acids, franks/luncheon meats) and negatively-weighted intakes (nuts/seeds, other vegetables, fish/shellfish low in -3 fatty acids, yogurt, coffee). A 1-unit increase in the ERDP score was associated with an increase in total (HR: 1.09, 95% CI: 1.01-1.18), invasive (HR: 1.13; 95% CI: 1.04-1.24) and estrogen receptor (ER)-positive (HR: 1.13, 95% CI: 1.02-1.24) breast cancer risk after adjustment for confounders. Associations were observed for the fourth quartile of ERDP compared with the first quartile for overall breast cancer (HR: 1.14; 95% CI: 0.98-1.32), invasive cases (HR: 1.20, 95% CI: 1.02-1.42) and ER-positive cases (HR: 1.19; 95% CI: 0.99-1.41). The increased risk associated with increasing ERDP score was more apparent in strata of some effect modifiers (postmenopausal hormone therapy non-users and non-obese participants) where the relative estrogen exposure due to that factor was lowest, although the p values for interaction were not statistically significant. Results suggest a dietary pattern based on estrogen metabolism is positively associated with postmenopausal breast cancer risk, possibly through an estrogenic influence. What's new? Serum levels of estrogen metabolites, which may be influenced by certain dietary factors, are associated with postmenopausal breast cancer risk. However, studies of diet and breast cancer have been inconclusive. In our study, an estrogen-related dietary pattern (ERDP) was developed based on data from dietary questionnaires and serum concentrations of estrogen metabolites in a subsample of control participants and cancer patients. The ERDP strongly correlated with the intake of refined

grains, cheese, lunch meats and yogurt. The ERDP was positively associated with postmenopausal breast cancer risk, with stronger effects in invasive and estrogen receptor-positive cases.
[Guinter, Mark A.] Amer Canc Soc, Behav & Epidemiol Res Grp, Atlanta, GA 30329 USA; [Guinter, Mark A.; McLain, Alexander C.; Merchant, Anwar T.; Steck, Susan E.] Univ South Carolina, Arnold Sch Publ Hlth, Dept Epidemiol & Biostat, Columbia, SC USA; [Sandler, Dale P.] NIEHS, Epidemiol Branch, NIH, Res Triangle Pk, NC 27709 USA; [Steck, Susan E.] Univ South Carolina, Canc Prevent & Control Program, Columbia, SC USA American Cancer Society; University of South Carolina System; University of South Carolina Columbia; National Institutes of Health (NIH) - USA; NIH National Institute of Environmental Health Sciences (NIEHS); University of South Carolina System; University of South Carolina Columbia
Steck, SE (corresponding author), 915 Greene St, Columbia, SC 29208 USA. ssteck@sc.edu Sandler, Dale/E-5110-2019; Steck, Susan/G-5736-2013; Merchant, Anwar/B-5233-2009; McLain, Alexander/AFD-8215-2022 McLain, Alexander/0000-0002-5475-0670
NIH-NIGMS [T32-GM081740]; Intramural Program of the NIH, National Institute of Environmental Health Sciences [Z01 ES-044005l] NIH-NIGMS(United States Department of Health & Human ServicesNational Institutes of Health (NIH) - USANIH National Institute of General Medical Sciences (NIGMS)); Intramural Program of the NIH, National Institute of Environmental Health Sciences
NIH-NIGMS; Grant number: T32-GM081740; Grant sponsor: Intramural Program of the NIH, National Institute of Environmental Health Sciences; Grant number: Z01 ES-044005l (DPS) 49
20 23 0 30 WILEY HOBOKEN 111 RIVER ST, HOBOKEN 07030-5774, NJ USA 0020-7136 1097-0215 INT J CANCER
Int. J. Cancer AUG 1 2018 143 3
580 590 10.1002/ijc.31387
http://dx.doi.org/10.1002/ijc.31387 11
Oncology Science Citation Index Expanded (SCI-EXPANDED)
Oncology GK4EY 29574860 Bronze, Green Accepted
2025-06-24 WOS:000436110100015
J Poyil, PK; Siraj, AK; Padmaja, D; Parvathareddy, SK; Alobaisi, K; Thangavel, S; Begum, R; Diaz, R; Al-Dayel, F; Al-Kuraya, KS Poyil, Pratheesh Kumar; Siraj, Abdul K.; Padmaja, Divya; Parvathareddy, Sandeep Kumar; Alobaisi, Khadija; Thangavel, Saravanan; Begum, Rafia; Diaz, Roxanne; Al-Dayel, Fouad; Al-Kuraya, Khawla S. Polo-like Kinase 1 Predicts Lymph Node Metastasis in Middle Eastern Colorectal Cancer Patients; Its Inhibition Reverses 5-Fu Resistance in Colorectal Cancer Cells CELLS English
Article PLK1; CRC; stemness; EMT; Zeb1 PLK1; PHOSPHORYLATION; APOPTOSIS; INVASION; MARKER; TARGET Polo-like kinase 1 (PLK1) is a serine/threonine-protein kinase essential for regulating multiple stages of cell cycle progression in mammals. Aberrant regulation of PLK1 has been observed in numerous human cancers and is linked to poor prognoses. However, its role in the pathogenesis of colorectal cancer (CRC) in the Middle East remains unexplored. PLK1 overexpression was noted in 60.3% (693/1149) of CRC cases and was significantly associated with aggressive clinico-pathological parameters and p-ERK1/2 overexpression. Intriguingly, multivariate logistic regression analysis identified PLK1 as an independent predictor of lymph node metastasis. Our in vitro experiments demonstrated that CRC cells with high PLK1 levels were resistant

to 5-Fu treatment, while those with low PLK1 expression were sensitive. To investigate PLK1 ' s role in chemoresistance, we used the specific inhibitor volasertib, which effectively reversed 5-Fu resistance. Interestingly, forced PLK1 expression activated the CRAF-MEK-ERK signaling cascade, while its inhibition suppressed this cascade. PLK1 knockdown reduced epithelial-to-mesenchymal transition (EMT) progression and stem cell-like traits in 5-Fu-resistant cells, implicating PLK1 in EMT induction and stemness in CRC. Moreover, silencing ERK1/2 significantly mitigated chemoresistance, EMT, and stemness properties in CRC cell lines that express PLK1. Furthermore, the knockdown of Zeb1 attenuated EMT and stemness, suggesting a possible link between EMT activation and the maintenance of stemness in CRC. Our findings underscore the pivotal role of PLK1 in mediating chemoresistance and suggest that PLK1 inhibition may represent a potential therapeutic strategy for the management of aggressive colorectal cancer subtypes. [Poyil, Pratheesh Kumar; Siraj, Abdul K.; Padmaja, Divya; Parvathareddy, Sandeep Kumar; Alobaisi, Khadija; Thangavel, Saravanan; Begum, Rafia; Diaz, Roxanne; Al-Kuraya, Khawla S.] King Faisal Specialist Hosp & Res Ctr, Human Canc Genom Res, POB 3354, Riyadh 11211, Saudi Arabia; [Al-Dayel, Fouad] King Faisal Specialist Hosp & Res Ctr, Dept Pathol, POB 3354, Riyadh 11211, Saudi Arabia King Faisal Specialist Hospital & Research Center; King Faisal Specialist Hospital & Research Center Al-Kuraya, KS (corresponding author), King Faisal Specialist Hosp & Res Ctr, Human Canc Genom Res, POB 3354, Riyadh 11211, Saudi Arabia. ppoyil@kfshrc.edu.sa; asiraj@kfshrc.edu.sa; dvsasidharan50@gmail.com; psandeepkumar@kfshrc.edu.sa; kalobaisi@kfshrc.edu.sa; tsaravanan97@kfshrc.edu.sa; brafia@kfshrc.edu.sa; rmelosantos87@kfshrc.edu.sa; dayelf@kfshrc.edu.sa; kkuraya@kfshrc.edu.sa Parvathareddy, Sandeep Kumar/JBI-9330-2023; Aldayel, Fouad/KHT-6847-2024; Siraj, Abdul/IQW-1179-2023; Thangavel, Saravanan/JLL-3433-2023; Diaz Caceres, Roxanne/MZR-0616-2025; Alkuraya, Khawla/AFQ-7946-2022
Alkuraya, Khawla/0000-0002-4126-3419
44 2 2 2 3 MDPI BASEL ST ALBAN-ANLAGE 66, CH-4052 BASEL, SWITZERLAND 2073-4409 CELLS-BASEL
Cells OCT 2024 13 20
1700 10.3390/cells13201700
http://dx.doi.org/10.3390/cells13201700 16 Cell Biology Science Citation Index Expanded (SCI-EXPANDED) Cell Biology K1Q7K 39451218 gold 2025-06-24
WOS:001341703200001
J Lattanzio, R; Iezzi, M; Sala, G; Tinari, N; Falasca, M; Alberti, S; Buglioni, S; Mottolese, M; Perracchio, L; Natali, PG; Piantelli, M Lattanzio, Rossano; Iezzi, Manuela; Sala, Gianluca; Tinari, Nicola; Falasca, Marco; Alberti, Saverio; Buglioni, Simonetta; Mottolese, Marcella; Perracchio, Letizia; Natali, Pier Giorgio; Piantelli, Mauro PLC-gamma-1 phosphorylation status is prognostic of metastatic risk in patients with early-stage Luminal-A and -B breast cancer subtypes
BMC CANCER English Article
Breast cancer; Phospholipase C gamma 1; Prognosis; Luminal subtypes; Menopausal status INTERNATIONAL EXPERT CONSENSUS; UNFOLDED PROTEIN RESPONSE; PHOSPHOLIPASE C-GAMMA-1; ESTROGEN-RECEPTOR; PRIMARY THERAPY; CYTOSKELETON; HIGHLIGHTS; SURVIVAL BackgroundPhospholipase C gamma 1 (PLC gamma 1) is highly expressed in human tumours. Our previous

studies reported that both stable and inducible PLC gamma 1 down-regulation can inhibit formation of breast-cancer-derived experimental lung metastasis. Further, high expression of PLC gamma 1 and its constitutively activated forms (i.e., PLC gamma 1-pY1253, PLC gamma 1-pY783) is associated with worse clinical outcome in terms of incidence of distant metastases, but not of local relapse in T1-T2, N0 breast cancer patients.MethodsIn the present retrospective study, we analysed the prognostic role of PLC gamma 1 in early breast cancer patients stratified according to the St. Gallen criteria and to their menopausal status. PLC gamma 1-pY1253 and PLC gamma 1-pY783 protein expression levels were determined by immunohistochemistry on tissue microarrays, and were correlated with patients' clinical data, using univariate and multivariate statistical analyses.ResultsIn our series, the prognostic value of PLC gamma 1 overexpression was restricted to Luminal type tumours. From multivariate analyses, pY1253-PLC gamma 1(High) was an independent prognostic factor only in postmenopausal patients with Luminal-B tumours (hazard ratio [HR], 2.4; 95% confidence interval [CI], 1.1-5.3; P=0.034). Conversely, PLC gamma 1-pY783(High) was a remarkably strong risk factor (HR, 20.1; 95% CI, 2.2-178.4; P=0.003) for pre/perimenopausal patients with Luminal-A tumours.ConclusionsPLC gamma 1 overexpression is a strong predictive surrogate marker of development of metastases in early Luminal-A and -B breast cancer patients, being able to discriminate patients with high and low risk of metastases. Therefore, targeting the PLC gamma 1 pathway can be considered of potential benefit for prevention of metastatic disease.

[Lattanzio, Rossano; Sala, Gianluca; Tinari, Nicola] G dAnnunzio Univ Chieti Pescara, Dept Med Oral & Biotechnol Sci, Chieti, Italy; [Lattanzio, Rossano; Iezzi, Manuela; Sala, Gianluca; Tinari, Nicola; Natali, Pier Giorgio; Piantelli, Mauro] G dAnnunzio Univ Chieti Pescara, CAST, Via Luigi Polacchi 11, I-66100 Chieti, Italy; [Iezzi, Manuela] G dAnnunzio Univ Chieti Pescara, Dept Med & Aging Sci, Chieti, Italy; [Falasca, Marco] Curtin Univ, Curtin Hlth Innovat Res Inst, Sch Pharm & Biomed Sci, Metab Signalling Grp, Perth, Australia; [Alberti, Saverio] Univ Messina, Dept Biotechnol BIOMORF, Via Consolare Valeria 1, I-98125 Messina, Italy; [Buglioni, Simonetta; Mottolese, Marcella; Perracchio, Letizia] Regina Elena Inst Canc Res, Dept Pathol, Via E Chianesi 53, I-00144 Rome, Italy G d'Annunzio University of Chieti-Pescara; G d'Annunzio University of Chieti-Pescara; G d'Annunzio University of Chieti-Pescara; Curtin University; University of Messina; IRCCS Istituti Fisioterapici Ospitalieri (IFO); IRCCS Regina Elena Lattanzio, R (corresponding author), G dAnnunzio Univ Chieti Pescara, Dept Med Oral & Biotechnol Sci, Chieti, Italy.; Lattanzio, R (corresponding author), G dAnnunzio Univ Chieti Pescara, CAST, Via Luigi Polacchi 11, I-66100 Chieti, Italy. rossano.lattanzio@unich.it Iezzi, Manuela/AAB-7939-2019; Lattanzio, Rossano/K-1927-2018; Sala, Gianluca/C-2056-2017; Alberti, Saverio/M-4511-2014Lattanzio, Rossano/0000-0001-9803-4476; Sala, Gianluca/0000-0002-4494-915X; Alberti, Saverio/0000-0002-4647-6042; Iezzi, Manuela/0000-0002-6296-6498; buglioni, simonetta/0000-0002-5066-1523 Associazione Italiana Ricerca sul Cancro (AIRC) [06/30/C/9]; Mediterranean Taskforce for Cancer Control Associazione Italiana Ricerca sul Cancro (AIRC)(Fondazione AIRC per la ricerca sul cancro); Mediterranean Taskforce for Cancer ControlThis research was supported by Associazione Italiana Ricerca sul Cancro (AIRC) (06/30/C/9) to

Pier Giorgio Natali, Mauro Piantelli, Marcella Mottolese, and Gianluca Sala, and by the Mediterranean Taskforce for Cancer Control (www.mtcc-prevention.net).The funding bodies did not have any role in the design of the study, the collection, analysis and interpretation of the data, and the writing of the manuscript.
31 16 17 0 1 BMC LONDON CAMPUS, 4 CRINAN ST, LONDON N1 9XW, ENGLAND 1471-2407 BMC CANCER BMC Cancer JUL 30 2019 19
747 10.1186/s12885-019-5949-x
http://dx.doi.org/10.1186/s12885-019-5949-x 10
Oncology Science Citation Index Expanded (SCI-EXPANDED)
Oncology IM4BB 31362705 Green Published, gold
2025-06-24 WOS:000477938600002
J Taghizadeh, A; Pourali, L; Joudi, M; Salehi, M; Eshghi, S; Torabian, F; Esmaeelpour, A Taghizadeh, Ali; Pourali, Leila; Joudi, Mona; Salehi, Maryam; Eshghi, Shohreh; Torabian, Farnaz; Esmaeelpour, Azin Assessment of Elevated Serum Tumor Markers Carcinoembryonic Antigen (CEA) and Cancer Antigen 15-3 (CA15- 3) among Patients with Different Subtypes of Metastatic Breast Cancer MIDDLE EAST JOURNAL OF CANCER English Article
Breast cancer; Metastasis; Cancer antigen 15-3 (CA15- 3); Carcinoembryonic antigen (CEA) CLINICOPATHOLOGICAL PARAMETERS; MOLECULAR SUBTYPES Background: Cancer antigen 15-3 and carcinoembryonic antigen are used in clinical and laboratory diagnosis of metastatic breast cancer. Previous studies have noted conflicting results about the association between carcinoembryonic antigen and cancer antigen 15-3 in metastatic breast cancer. The present study examined serum tumor marker levels of carcinoembryonic antigen and cancer antigen 15-3 among patients with different subtypes of metastatic breast cancer. Methods: In this cross-sectional study, we assessed metastatic breast cancer patients diagnosed between 2005 and 2012 who referred to academic Hospitals affiliated with Mashhad University of Medical Sciences. The patients were selected by systematic randomization sampling. Demographic, clinical, pathological, and therapeutic data were collected from patients' hospital records. Statistical analyses were performed by Statistical Package for the Social Sciences version 16.0 software. Results: A total of 298 eligible patients enrolled in the study. Patients' median age was 48.39 +/- 12.57 years. Elevated serum levels of carcinoembryonic antigen were identified in 65.17% of patients and cancer antigen 15-3 in 57.29% of patients. Based on molecular subtype categorization, 109 (39.5%) patients were human epidermal growth factor receptor 2 negative and 105 (38.0%) patients were in the luminal group. There was no significant correlation between serum carcinoembryonic antigen and cancer antigen 15-3 with subtypes of the tumor. The most common sites for metastasis were bones and liver, respectively. However, there was no significant correlation between serum carcinoembryonic antigen and cancer antigen 15-3 with the site of metastasis. There was a significant association between serum carcinoembryonic antigen level and stages IIA and IV. Conclusion: One of the most significant findings of the current study was the increased serum carcinoembryonic antigen and cancer antigen 15-3 levels in most metastatic breast cancer participants. We postulate that regular measurement of serum cancer antigen 15-3 and carcinoembryonic antigen could be useful for earlier detection and prediction of outcomes. [Taghizadeh,

Ali; Joudi, Mona] Mashhad Univ Med Sci, Surg Oncol Res Ctr, Mashhad, Iran; [Pourali, Leila] Mashhad Univ Med Sci, Dept Obstet & Gynecol, Fac Med, Mashhad, Iran; [Salehi, Maryam] Mashhad Univ Med Sci, Dept Sociomed, Fac Med, Mashhad, Iran; [Eshghi, Shohreh] Mashhad Univ Med Sci, Canc Res Ctr, Mashhad, Iran; [Torabian, Farnaz] Islamic Azad Univ, Mashhad Branch, Fac Med, Mashhad, Iran; [Esmaeelpour, Azin] Birjand Univ Med Sci, Student Res Comm, Birjand, Iran   Mashhad University of Medical Sciences; Mashhad University of Medical Sciences; Mashhad University of Medical Sciences; Mashhad University of Medical Sciences; Islamic Azad University; Birjand University of Medical Sciences Pourali, L (corresponding author), Mashhad Univ Med Sci, Dept Obstet & Gynecol, Fac Med, Mashhad, Iran. pouralil@mums.ac.ir
Esmaeelpour, Azin/IQU-8973-2023; Pourali, Leila/ABI-2011-2020; Taghizadeh, Mohsen/R-3733-2018; salehi, Maryam/K-8396-2016
Mashhad University of Medical Sciences [P8572]
Mashhad University of Medical Sciences(Mashhad University of Medical Sciences)   This article was derived from a student thesis (code no. P8572). The authors would like to express their appreciation to the respected Research Deputy of Mashhad University of Medical Sciences who financially supported this paper.   16   3   3   0   3   SHIRAZ UNIV MEDICAL SCIENCES   SHIRAZ   NEMAZEE HOSPITAL, SHIRAZ, 71934, IRAN
2008-6709   2008-6687   MIDDLE EAST J CANCER   Middle East J. Cancer   JAN   2019   10   1   17
22   6   Oncology   Emerging Sources Citation Index (ESCI)   Oncology   HI1LC
2025-06-24 WOS:000456205900003
J   Moudatsou, M; Vouyiouka, P; Karagianni-Hatziskou, E; Rovithis, M; Stavropoulou, A; Koukouli, S
Moudatsou, Maria; Vouyiouka, Panayiota; Karagianni-Hatziskou, Eleni; Rovithis, Michael; Stavropoulou, Areti; Koukouli, Sofia   Knowledge and Use of Cervical Cancer Prevention Services among Social Work and Nursing University Students   HEALTHCARE   English   Article
cervical cancer prevention services; knowledge; social capital; social work students; nursing students; health education HUMAN-PAPILLOMAVIRUS; BREAST; INFECTION; LATINAS
The present study examines: (a) the knowledge of healthcare students on cervical cancer (CC) issues and the use of related preventive services, as well as their association with the field of study and other sociodemographic characteristics; (b) the possible effect of social capital and its parameters. A cross-sectional study was conducted, using a convenience non-probability sampling technique. The final sample consisted of forty-nine social work and fifty-one nursing students. The two groups were similar regarding their sociodemographic characteristics and the knowledge and use of gynecological preventive services. However, the nursing students undertook a PAP smear check-up to a lesser extent (48.6%) compared to social work students (51.4%) (p = 0.026). The social capital scores were high for both groups, but social work students were significantly more 'Tolerant to diversity'. For the total sample, only the 'Family and friends connections' subscale correlated with knowledge about the existing gynecological preventive services. Among the main reasons explaining university students' avoidance of preventive testing were the feelings of fear and embarrassment associated with the PAP smear test. Given the significance of the future professional

roles of healthcare students as information sources and leaders in women's CC preventive behavior, understanding the individual factors contributing to their own adherence is essential. It is equally important to increase their scientific knowledge through the improvement of academic curricula regarding these issues.
[Moudatsou, Maria; Vouyiouka, Panayiota; Karagianni-Hatziskou, Eleni; Koukouli, Sofia] Hellen Mediterranean Univ, Sch Hlth Sci, Social Work Dept, GR-71410 Iraklion, Greece; [Moudatsou, Maria; Rovithis, Michael; Stavropoulou, Areti; Koukouli, Sofia] Hellen Mediterranean Univ, Lab Interdisciplinary Approaches Enhancement Qual, GR-71410 Iraklion, Greece; [Moudatsou, Maria; Rovithis, Michael; Koukouli, Sofia] Hellen Mediterranean Univ, Res Ctr, Inst Agrifood & Life Sci, GR-71410 Iraklion, Greece; [Rovithis, Michael] Hellen Mediterranean Univ, Sch Hlth Sci, Nursing Dept, GR-71410 Iraklion, Greece; [Stavropoulou, Areti] Univ West Attica, Sch Hlth & Care Sci, Nursing Dept, GR-12243 Athens, Greece Hellenic Mediterranean University; Hellenic Mediterranean University; Hellenic Mediterranean University; Hellenic Mediterranean University; University of West Attica
Moudatsou, M (corresponding author), Hellen Mediterranean Univ, Sch Hlth Sci, Social Work Dept, GR-71410 Iraklion, Greece.; Moudatsou, M (corresponding author), Hellen Mediterranean Univ, Lab Interdisciplinary Approaches Enhancement Qual, GR-71410 Iraklion, Greece.; Moudatsou, M (corresponding author), Hellen Mediterranean Univ, Res Ctr, Inst Agrifood & Life Sci, GR-71410 Iraklion, Greece. moudatsoum@hmu.gr; vgiota19@hotmail.com; elenaki_k21@hotmail.com; rovithis@hmu.gr; astavropoulou@uniwa.gr; koukouli@hmu.gr Stavropoulou, Areti/AAZ-3339-2021; KOUKOULI, SOFIA/AFC-3460-2022 Rovithis, Michael/0000-0003-0471-0117; KOUKOULI, SOFIA/0000-0003-0358-1405
52
4 4 0 5 MDPI BASEL MDPI AG, Grosspeteranlage 5, CH-4052 BASEL, SWITZERLAND 2227-9032
HEALTHCARE-BASEL Healthcare JUN 2022 10 6
1140 10.3390/healthcare10061140
http://dx.doi.org/10.3390/healthcare10061140 16
Health Care Sciences & Services; Health Policy & Services
Science Citation Index Expanded (SCI-EXPANDED); Social Science Citation Index (SSCI) Health Care Sciences & Services
2N5IT 35742191 gold, Green Published 2025-06-24 WOS:000818413100001
J Warleta, F; Campos, M; Allouche, Y; Sánchez-Quesada, C; Ruiz-Mora, J; Beltrán, G; Gaforio, JJ Warleta, Fernando; Campos, Maria; Allouche, Yosra; Sanchez-Quesada, Cristina; Ruiz-Mora, Jesus; Beltran, Gabriel; Gaforio, Jose J.
Squalene protects against oxidative DNA damage in MCF10A human mammary epithelial cells but not in MCF7 and MDA-MB-231 human breast cancer cells FOOD AND CHEMICAL TOXICOLOGY English Article
Squalene; Human breast cancer; Comet assay; Virgin olive oil; Cancer prevention VIRGIN OLIVE OIL; CHEMOPREVENTIVE AGENT; BONE-MARROW; IN-VITRO; CARCINOGENESIS; GROWTH; ASSAY; ANTIOXIDANTS; INHIBITION; PREVENTION Until now, very little has been known about the antioxidant capacity of squalene and its effect on human breast tumourigenesis. In the present work, we investigated squalene's scavenging properties and its effect on cell proliferation, cell cycle profile, apoptosis, reactive oxygen species (ROS) level and oxidative DNA damage, using human breast cell lines. Our results showed that squalene neither possesses

scavenging activity nor significantly alters cell proliferation rates, the cell cycle profile or cell apoptosis in human mammary epithelial cells (MCF10A), minimally invasive (MDA-MB-231) breast cancer cells, and highly invasive (MCF7) breast cancer cells. However, we found that squalene did exert the following effects on MCF10A epithelial cells in a dose-dependent manner: (a) it decreased intracellular ROS level, (b) it prevented H2O2-induced oxidative injury, and (c) it protected against oxidative DNA damage. Interestingly, squalene did not exert these effects on MCF7 and MDA-MB-231 cancer cells. Therefore, our data suggest that squalene, found in high amounts in virgin olive oils, could be partially responsible for the lower incidence of breast cancer in populations that consume the Mediterranean diet due to its protective activity against oxidative DNA damage in normal mammary cells. (C) 2010 Elsevier Ltd. All rights reserved. [Warleta, Fernando; Campos, Maria; Allouche, Yosra; Sanchez-Quesada, Cristina; Ruiz-Mora, Jesus; Gaforio, Jose J.] Univ Jaen, Div Immunol, Dept Hlth Sci, Fac Expt Sci, Jaen 23071, Spain; [Allouche, Yosra; Beltran, Gabriel] Ctr Venta Llano, Inst Andaluz Invest & Formac Agr Pesquera & Prod, Mengibar 23620, Spain
Universidad de Jaen Gaforio, JJ (corresponding author), Univ Jaen, Div Immunol, Dept Hlth Sci, Fac Expt Sci, Campus Lagunillas S-N, Jaen 23071, Spain. jgaforio@ujaen.es
Santos e Campos, MariaAparecida/C-4555-2019; Sanchez, Cristina/ABC-5511-2021; Beltran, Gabriel/D-1372-2016 Warleta, Fernando/0000-0002-7365-7367; Sanchez-Quesada, Cristina/0000-0003-2997-8515; Gaforio, Jose J/0000-0003-2996-9301 Ministerio de Ciencia e Innovacion [RTA2008-00066-0O3-03]; Instituto Andaluz de Blotecnologia [BIOANDALUS 08/22/L5.3]; Centro de Excelencia en Investigacion sobre Aceite de Oliva y Salud (CEAS) Ministerio de Ciencia e Innovacion(Spanish GovernmentInstituto de Salud Carlos III); Instituto Andaluz de Blotecnologia; Centro de Excelencia en Investigacion sobre Aceite de Oliva y Salud (CEAS) This study was supported by the "Ministerio de Ciencia e Innovacion" (RTA2008-00066-0O3-03); "Instituto Andaluz de Blotecnologia" (BIOANDALUS 08/22/L5.3); and; "Centro de Excelencia en Investigacion sobre Aceite de Oliva y Salud" (CEAS). 36 115 129 2 33
PERGAMON-ELSEVIER SCIENCE LTD OXFORD THE BOULEVARD, LANGFORD LANE, KIDLINGTON, OXFORD OX5 1GB, ENGLAND 0278-6915
1873-6351 FOOD CHEM TOXICOL Food Chem. Toxicol.
APR 2010 48 4 1092 1100
10.1016/j.fct.2010.01.031
http://dx.doi.org/10.1016/j.fct.2010.01.031 9
Food Science & Technology; Toxicology Science Citation Index Expanded (SCI-EXPANDED) Food Science & Technology; Toxicology 582JL 20138105 2025-06-24
WOS:000276593100014
J Ortashi, O; Shallal, M; Osman, N; Raheel, H
Ortashi, Osman; Shallal, Musa; Osman, Nawal; Raheel, Hina
Knowledge, Attitude and Practice of School Nurses in the United Arab Emirates about HPV Infection and Vaccine
ASIAN PACIFIC JOURNAL OF CANCER PREVENTION
English Article HPV vaccine; cervical cancer; school nurses; United Arab Emirates, Abu Dhabi HUMAN-PAPILLOMAVIRUS INFECTION; CERVICAL-CANCER; UNIVERSITY; WOMEN Background: In 2008, the Health Authority in Abu Dhabi (the capital of the United Arab Emirates) introduced HPV vaccine free of charge for high school girls entering grade 11, becoming the

first state in the Middle East to do so. The objectives of this study were to assess the knowledge, attitude and practice of school nurses in the Emirate of Abu Dhabi about HPV infection and the vaccine. Materials and Methods: A quantitative study was designed and conducted from June to August 2012 in Emirate of Abu Dhabi. Data were collected through direct face to face interviews. from one hundred and twenty five nurses. Results: Knowledge of HPV infection and HPV vaccine was almost universal among the school nurses (97%). The majority of the participants (71%) thought that the HPV vaccine was good. Cultural unacceptability (45%) and lack of women's concern about their own health (21%) were rated as the top barriers for the successful introduction of the vaccine in the UAE. More than half of the sampled nurses (58%) have either given this vaccine to school girls or taken it themselves. The majority (95%) did not come across any side effects from the vaccine. The level of qualification and the place of work did not significantly affect the correct knowledge of HPV infection or cervical cancer prevention methods. Conclusions: The knowledge and attitude of the sampled school nurses in Abu Dhabi State about HPV infection and vaccine is very good in both the public and private sectors. However, a knowledge gap in cervical cancer screening methods was identified. [Ortashi, Osman] United Arab Emirates Univ, Coll Med & Hlth Sci, Al Ain, U Arab Emirates; [Shallal, Musa] United Arab Emirates Univ, Fac Humanities & Social Sci, Al Ain, U Arab Emirates; [Osman, Nawal; Raheel, Hina] United Arab Emirates Univ, Fac Med & Hlth Sci, Al Ain, U Arab Emirates United Arab Emirates University; United Arab Emirates University; United Arab Emirates University Ortashi, O (corresponding author), United Arab Emirates Univ, Coll Med & Hlth Sci, Al Ain, U Arab Emirates.
Osman.ortashi@uaeu.ac.ae
15 15 16 0 6 ASIAN PACIFIC ORGANIZATION CANCER PREVENTION GYEONGGI-DO APJCP HEAD OFFICE, KOREAN NATL CANCER CENTER, 323 ILAN -RO, ILSANDONG-GU, GOYANG-SI, GYEONGGI-DO, 410-769, SOUTH KOREA 1513-7368 ASIAN PAC J CANCER P
Asian Pac. J. Cancer Prev. 2012 13 12
6481 6484 10.7314/APJCP.2012.13.12.6481
http://dx.doi.org/10.7314/APJCP.2012.13.12.6481
4 Oncology Science Citation Index Expanded (SCI-EXPANDED) Oncology 244RE 23464478 Green Submitted, gold
2025-06-24 WOS:000326406300098
J Nimee, F; Gioxari, A; Papandreou, P; Amerikanou, C; Karageorgopoulou, S; Kaliora, AC; Skouroliakou, M
Nimee, Frantzeska; Gioxari, Aristea; Papandreou, Panos; Amerikanou, Charalampia; Karageorgopoulou, Sofia; Kaliora, Andriana C.; Skouroliakou, Maria The Effect of Melatonin Supplementation on Cancer-Related Fatigue during Chemotherapy Treatment of Breast Cancer Patients: A Double-Blind, Randomized Controlled Study CANCERS English
Article melatonin; supplement; cancer-related fatigue; breast cancer; chemotherapy
MEDITERRANEAN DIET; SLEEP DISORDERS; TRIAL; DISTURBANCES; MANAGEMENT; QUALITY; WEIGHT; DRUGS; WOMEN Cancer-related fatigue (CRF) is a common distressing complaint of breast cancer (BC) patients treated with chemotherapy. Nutritional quality plays a pivotal role in CRF, while increased interest towards new pharmacological agents has been observed. Melatonin, an endogenous hormone that regulates the human sleep-wake cycle, could alleviate CRF. In the present randomized, placebo-controlled 3-month trial,

we investigated the effects of melatonin intake (i.e., 1 mg/day) vs. placebo in BC patients on CRF. In both arms, the Mediterranean diet (MD) was implemented. Medical history, anthropometry and blood withdrawal were performed. CRF was evaluated by the Functional Assessment of Chronic Illness Therapy-Fatigue questionnaire and MD adherence by the MedDietScore. In total, 49 BC women (median age 52 years) were recruited, namely N = 23 in the intervention arm and N = 26 in the placebo arm. At baseline, CRF was positively associated with body mass index (BMI), even when adjusted for age, waist circumference and blood indices related to disease prognosis (beta = -0.882, p = 0.003). At 3 months, both groups showed a BMI decrease (p < 0.05), but only the intervention group improved CRF compared to baseline (p = 0.003). No differences in CRF were observed between the groups. In conclusion, melatonin oral supplementation could ameliorate CRF in BC patients. [Nimee, Frantzeska; Amerikanou, Charalampia; Kaliora, Andriana C.; Skouroliakou, Maria] Harokopio Univ, Sch Hlth Sci & Educ, Dept Dietet & Nutr Sci, 70 El Venizelou Ave, Athens 17671, Greece; [Gioxari, Aristea] Sch Hlth Sci, Dept Nutr Sci & Dietet, Kalamata 24100, Greece; [Papandreou, Panos] IASO Hosp, Dept Nutr, 37 Chomatianou Str, Athens 15123, Greece; [Karageorgopoulou, Sofia] IASO Hosp, Dept Med Oncol 3, 37 Chomatianou Str, Athens 15123, Greece Harokopio University Athens Kaliora, AC (corresponding author), Harokopio Univ, Sch Hlth Sci & Educ, Dept Dietet & Nutr Sci, 70 El Venizelou Ave, Athens 17671, Greece. fnimee@hua.gr; a.gioxari@uop.gr; ppapandreou@cibusmed.com; amerikanou@windowslive.com; skarageorg@hotmail.com; akaliora@hua.gr; mskour@hua.gr Gioxari, Aristea/AAA-1163-2021; Skouroliakou, Maria/AAN-3002-2021; Kaliora, Andriana/AAM-2912-2021; Amerikanou, Charalampia/ABE-1634-2022
AMERIKANOU, CHARALAMPIA/0000-0002-2014-5392; Skouroliakou, Maria/0000-0002-0468-8397; Gioxari, Aristea/0000-0002-4869-6815; Papandreou, Panos/0000-0002-5629-0831 59
2 3 0 3 MDPI BASEL ST ALBAN-ANLAGE 66, CH-4052 BASEL, SWITZERLAND 2072-6694 CANCERS
Cancers FEB 2024 16 4
802 10.3390/cancers16040802
http://dx.doi.org/10.3390/cancers16040802 13
Oncology Science Citation Index Expanded (SCI-EXPANDED)
Oncology JR1N4 38398193 Green Published, gold
2025-06-24 WOS:001174802200001
J Junkins, K; Rodgers, M; Phelan, SA
Junkins, Katherine; Rodgers, Margaret; Phelan, Shelley A.
Oleuropein Induces Cytotoxicity and Peroxiredoxin Over-expression in MCF-7 Human Breast Cancer Cells ANTICANCER RESEARCH English Article
Breast cancer; MCF-7; oleuropein; olive leaf; peroxiredoxin Background/Aim: As a fundamental staple of the Mediterranean diet, olive oil has long been recognized for its health benefits, including its ability to reduce cardiovascular and neurological disease. Oleuropein is the primary phenolic chemical found in all parts of the olive tree, especially in the leaves and fruit. Oleuropein exhibits anti-inflammatory and antioxidant properties, and has been associated with cancer inhibition in various animal and cell models. We investigated the effects of oleuropein on the MCF-7 human breast cancer cell line, and compared it to the non-cancerous MCF-10A breast epithelial line. Materials and Methods: Both cell lines were treated with two

different concentrations of oleuropein for 48 and 72 hours. Cytotoxicity, apoptosis, and peroxiredoxin expression were measured. Results: Forty-eight hours of oleuropein treatment induced cytotoxicity in MCF-7 cells, whereas it had no effect on MCF-10A cells. Furthermore, oleuropein-induced cytotoxicity in MCF-7 cells involved a measurable increase in apoptosis. Oleuropein treatment of MCF-7 cells significantly and dramatically increased expression of all six peroxiredoxin mRNAs (Prdx1-Prdx6), whereas oleuropein treatment of MCF-10A cells resulted in only a small increase in Prdx1 and Prdx6 expression, with no change in the expression of the other peroxiredoxins. Together, these data demonstrate differential susceptibility to oleuropein-induced cell death between the two lines, and differential regulation of peroxiredoxins. Conclusion: Oleuropein-induced over -expression of peroxiredoxins in MCF-7 cells may either facilitate its cancer-specific cytotoxicity or, alternatively, is a consequence of an altered response of cancer cells. [Junkins, Katherine] Dana Farber Canc Inst, Dept Med Oncol, Boston, MA USA; [Rodgers, Margaret] Tufts Univ, Dept Cell & Mol Biol, Boston, MA USA; [Phelan, Shelley A.] Fairfield Univ, Dept Biol, Fairfield, CT 06824 USA Harvard University; Harvard University Medical Affiliates; Dana-Farber Cancer Institute; Tufts University; Fairfield University Phelan, SA (corresponding author), Fairfield Univ, Dept Biol, Fairfield, CT 06824 USA. sphelan@fairfield.edu Department of Biology; Science Institute at Fairfield University Department of Biology; Science Institute at Fairfield University This work was funded by the Department of Biology and the Science Institute at Fairfield University. 25 13 13 1 7 INT INST ANTICANCER RESEARCH ATHENS EDITORIAL OFFICE 1ST KM KAPANDRITIOU-KALAMOU RD KAPANDRITI, PO BOX 22, ATHENS 19014, GREECE 0250-7005 1791-7530 ANTICANCER RES
Anticancer Res. OCT 2023 43 10
4333 4339 10.21873/anticanres.16628
http://dx.doi.org/10.21873/anticanres.16628 7
Oncology Science Citation Index Expanded (SCI-EXPANDED)
Oncology W1IJ7 37772594 hybrid 2025-06-24 WOS:001089231200009
J Porciello, G; Coluccia, S; Vitale, S; Palumbo, E; Luongo, A; Grimaldi, M; Pica, R; Prete, M; Calabrese, I; Cubisino, S; Montagnese, C; Falzone, L; Martinuzzo, V; Poletto, L; Rotondo, E; Di Gennaro, P; De Laurentiis, M; D'Aiuto, M; Rinaldo, M; Thomas, G; Messina, F; Catalano, F; Ferrau, F; Montesarchio, V; Serraino, D; Crispo, A; Libra, M; Celentano, E; Augustin, LSA
Porciello, Giuseppe; Coluccia, Sergio; Vitale, Sara; Palumbo, Elvira; Luongo, Assunta; Grimaldi, Maria; Pica, Rosa; Prete, Melania; Calabrese, Ilaria; Cubisino, Serena; Montagnese, Concetta; Falzone, Luca; Martinuzzo, Valentina; Poletto, Luigina; Rotondo, Emanuela; Di Gennaro, Piergiacomo; De Laurentiis, Michelino; D'Aiuto, Massimiliano; Rinaldo, Massimo; Thomas, Guglielmo; Messina, Francesco; Catalano, Francesca; Ferrau, Francesco; Montesarchio, Vincenzo; Serraino, Diego; Crispo, Anna; Libra, Massimo; Celentano, Egidio; Augustin, Livia S. A.
DEDiCa Study Grp Baseline Association between Healthy Eating Index-2015 and Health-Related Quality of Life in Breast Cancer Patients Enrolled in a Randomized Trial CANCERS
English Article healthy eating index; diet quality; health-related quality of life; breast cancer survivors DIET QUALITY; EORTC QLQ-C30; SURVIVORS;

COMORBIDITIES; ONCOLOGISTS; PREVALENCE; DEPRESSION; EXERCISE; FATIGUE; PROGRAM Simple Summary Quality of life significantly affects health outcomes in cancer patients. However, evidence of an association between diet quality and quality of life in cancer survivors is sparse in Mediterranean countries. The aim of this study was to evaluate the associations between an a priori diet quality index, the Healthy Eating Index-2015 (HEI-2015), and quality of life, assessed through a validated questionnaire targeted at women with a breast cancer diagnosis. A higher HEI-2015 score was positively associated with summary quality of life score and inversely associated with symptom scores.Abstract Health-related quality of life (HRQoL) represents one of the most concerning aspects for cancer patients. The Healthy Eating Index (HEI) is an a priori diet quality index directly associated with health outcomes and HRQoL in cancer survivors in North American populations. We evaluated, in a Mediterranean population, the baseline associations between HEI-2015 and HRQoL in 492 women with breast cancer recruited in a DEDiCa lifestyle trial. Dietary data were obtained from 7-day food records; HRQoL was assessed through the European Organisation for Research and Treatment of Cancer Quality of Life Questionnaire Core 30 (EORTC QLQ C30) and the C30 Summary Score (SumSc). Analysis of variance and multivariable linear and log-gamma regression models were performed. Mean and standard deviation for HEI-2015 score was 68.8 +/- 11.2; SumSc was 81.5 +/- 12.9. Women with lower HEI-2015 score had higher BMI, were more frequently exposed to tobacco smoke and had fewer years of education. Patients with a HEI-2015 score greater than 68.7 (median value) showed a significant increase in SumSc of 4% (p = 0.02). HEI-2015 components also associated with SumSc were beans and greens (beta = 1.04; p = 0.02). Weak associations were found for total vegetables and saturated fats. Higher diet quality in breast cancer survivors was associated with higher overall HRQoL in this cross-sectional analysis. [Porciello, Giuseppe; Coluccia, Sergio; Vitale, Sara; Palumbo, Elvira; Luongo, Assunta; Grimaldi, Maria; Pica, Rosa; Prete, Melania; Rotondo, Emanuela; Crispo, Anna; Celentano, Egidio; Augustin, Livia S. A.] Ist Nazl Tumori IRCCS Fdn G Pascale, Epidemiol & Biostat Unit, I-80131 Naples, Italy; [Calabrese, Ilaria] A Cardarelli Hosp, Healthcare Direct, I-80131 Naples, Italy; [Cubisino, Serena] Human Ist Clin Catanese, I-95045 Misterbianco, Italy; [Montagnese, Concetta] CNR Italy, Inst Food Sci, I-83100 Avellino, Italy; [Falzone, Luca] Univ Catania, Dept Biomed & Biotechnol Sci, Oncol Clin & Gen Pathol Sect, I-95124 Catania, Italy; [Martinuzzo, Valentina; Poletto, Luigina; Serraino, Diego] Natl Canc Inst, Canc Epidemiol Unit, CRO, IRCCS, I-33081 Aviano, Italy; [Di Gennaro, Piergiacomo] Univ Campania Luigi Vanvitelli, Med Stat Unit, I-80138 Naples, Italy; [De Laurentiis, Michelino] Ist Nazl Tumori IRCCS Fdn G Pascale, Div Breast Med Oncol, Dept Breast & Thorac Oncol, I-80131 Naples, Italy; [D'Aiuto, Massimiliano; Rinaldo, Massimo] Clin Villa Fior, I-81031 Aversa, Italy; [Thomas, Guglielmo] Mediterranea Cardiocentro, I-80122 Naples, Italy; [Messina, Francesco] Osped Evangel Betania, Naples, Italy; [Catalano, Francesca] Cannizzaro Hosp, I-95126 Catania, Italy; [Ferrau, Francesco] Osped San Vincenzo, I-98039 Taormina, Italy; [Montesarchio, Vincenzo; Libra, Massimo] AORN Colli Monaldi Cotugno CTO, UOC Oncol, Naples, Italy

IRCCS Fondazione Pascale; Antonio Cardarelli Hospital; Consiglio Nazionale delle Ricerche (CNR); Istituto di Scienze dell' Alimentazione (ISA-CNR); University of Catania; IRCCS Aviano

(CRO); Universita della Campania Vanvitelli; IRCCS Fondazione Pascale   Porciello, G; Crispo, A (corresponding author), Ist Nazl Tumori IRCCS Fdn G Pascale, Epidemiol & Biostat Unit, I-80131 Naples, Italy.   g.porciello@istitutotumori.na.it; sergio.coluccia@istitutotumori.na.it; elvira.palumbo@istitutotumori.na.it; assunta.luongo@istitutotumori.na.it; m.grimaldi@istitutotumori.na.it; r.pica@istitutotumori.na.it; melania.prete@istitutotumori.na.it; ilariacalabrese@live.it; serena-cubisino@hotmail.it; concetta.montagnese@isa.cnr.it; luca.falzone@unict.it; nutrizionista.martinuzzo@gmail.com; poletto.gina08@gmail.com; e.rotondo@istitutotumori.na.it; piergiacomo.digennaro@unicampania.it; m.delaurentiis@istitutotumori.na.it; massimiliano.daiuto@gmail.com; massimo.rinaldo@tiscali.it; guglielmo.thomas@outlook.it; messina52@alice.it; fcatalano1968@tiscali.it; ferrau@oncologiataormina.it; vincenzo.montesarchio@ospedalideicolli.it; serrainod@cro.it; a.crispo@istitutotumori.na.it; m.libra@unict.it; e.celentano@istitutotumori.na.it; l.augustin@istitutotumori.na.it
Porciello, Giuseppe/AAA-7491-2020; Calabrese, Ilaria/AHE-4887-2022; Montagnese, Concetta/AAC-8706-2022; Messina, Francesco/AAC-1675-2019; De Laurentiis, Michelino/AAC-6321-2022; Celentano, Egidio/AAC-8984-2022; Coluccia, Sergio/AAC-6043-2022; Augustin, Livia/AAC-9009-2022; Falzone, Luca/AAC-2085-2019; Luongo, Assunta/IAR-4844-2023; Crispo, Anna/ABA-7435-2020; Libra, Massimo/L-8241-2018; Serraino, Diego/J-3915-2018; DE LAURENTIIS, Michelino/K-4934-2018 Porciello, Giuseppe/0000-0002-8731-2773; Libra, Massimo/0000-0002-7232-7737; Serraino, Diego/0000-0003-0565-8920; D'Aiuto, Massimiliano/0000-0002-4188-5226; Celentano, Egidio/0000-0002-3193-5699; Coluccia, Sergio/0000-0003-4044-1217; Augustin, Livia Silvia Adriana/0000-0002-6673-8281; Luongo, Assunta/0000-0002-1099-2686; Crispo, Anna/0000-0002-8455-3328; Falzone, Luca/0000-0001-7349-6826; DE LAURENTIIS, Michelino/0000-0001-9009-1572; Montagnese, Concetta/0000-0002-3662-2413; Calabrese, Ilaria/0000-0002-4222-4665
Italian Ministry of Health [PE-2013-02358099, L1/1]   Italian Ministry of Health(Ministry of Health, Italy)   This trial is funded by grants from the Italian Ministry of Health (Grant No. PE-2013-02358099; RC Project L1/1).
80   2   2   0   3   MDPI BASEL ST ALBAN-ANLAGE 66, CH-4052 BASEL, SWITZERLAND   2072-6694   CANCERS
Cancers   JUL   2024 16   14
2576 10.3390/cancers16142576
http://dx.doi.org/10.3390/cancers16142576   18
Oncology   Science Citation Index Expanded (SCI-EXPANDED)
Oncology   ZQ0V8 39061215   Green Published, gold
2025-06-24 WOS:001276652900001
J   Sinagra, E; Guarnotta, V; Raimondo, D; Mocciaro, F; Dolcimascolo, S; Rizzolo, CA; Puccia, F; Maltese, N; Citarrella, R; Messina, M; Spada, M; Tomasello, G; Cappello, F; Leone, A; Rossi, F; Di Mitri, R; Morreale, GC; Marasà, S; Midiri, M; Midiri, F; Sinagra, D   Sinagra, E.; Guarnotta, V.; Raimondo, D.; Mocciaro, F.; Dolcimascolo, S.; Rizzolo, C. A.; Puccia, F.; Maltese, N.; Citarrella, R.; Messina, M.; Spada, M.; Tomasello, G.; Cappello, F.; Leone, A.; Rossi, F.; Di Mitri, R.; Morreale, G. C.; Marasa, S.; Midiri, M.; Midiri, F.; Sinagra, D.
COLORECTAL CANCER IN PATIENTS WITH TYPE 2 DIABETES MELLITUS: A SINGLE-CENTER EXPERIENCE   JOURNAL OF

BIOLOGICAL REGULATORS AND HOMEOSTATIC AGENTS English Article type 2 diabetes; colorectal cancer; anti-diabetic drugs GROWTH-FACTOR; INSULIN; RISK; COHORT Type 2 diabetes mellitus (T2DM) is associated with an increased risk of colorectal cancer (CRC). The aim of the study is to evaluate the prevalence of CRC in a cohort of Caucasian patients with T2DM and the association with other variables previously known to be related with increased risk of CRC. We retrospectively evaluated the data of 741 consecutive Caucasian patients with T2DM who underwent colonoscopic screening in our tertiary referral center. A control cohort of 333 patients with thyroid disease was selected to evaluate the difference in the incidence of CRC. At a median follow-up of 132.5 months (range 33.3-175.7), 67 cases of cancer (prevalence 9%) occurred; among these, 14 cases of CRC were reported (prevalence 1.88%) among the diabetic patients, while only two case (one of these was a CRC) (overall prevalence 0.006%, prevalence of CRC 0.003%) occurred in the control group; the difference between the prevalence of CRC was statistically significant (chi-square 4.21, p = 0.04). The median duration of T2DM to CRC diagnosis was 168 months (range 12-768). At the univariate analysis, older age (p = 0.001, r 0.138) and diabetes duration (p = 0.001, r 0.138) were related to higher risk of cancer, while metformin seems to be protective towards cancer (p = 0.07, r - 0.098). In the subset of patients with CRC, the age (RR = 2.25; 95% CI: 0.30 - 17.31; p < 0.001), the diabetes duration (RR = 1.93; 95% CI: 0.25 - 14.77; p = 0.001) and the sulphonylureas treatment (RR = 2.33; 95% CI: 0.78 - 7.38; p = 0.007) were independently correlated with CRC. In our study, the prevalence of CRC in the cohort of patients with T2DM was higher compared to that from the National Tumor Register in 2010 (0.5%). Furthermore, we could speculate that sulphonylureas may play a role in CRC carcinogenesis impairing the physiological insulin secretion. [Sinagra, E.; Raimondo, D.; Rossi, F.] Fdn Ist G Giglio, Gastroenterol & Endoscopy Unit, Cefalu, Italy; [Sinagra, E.; Tomasello, G.; Cappello, F.] Euro Mediterranean Inst Sci & Technol IEMEST, Palermo, Italy; [Guarnotta, V.; Dolcimascolo, S.; Rizzolo, C. A.; Puccia, F.; Maltese, N.; Citarrella, R.; Sinagra, D.] Univ Palermo, Biomed Dept Internal & Specialist Med DIBIMIS, Sect Cardio Resp & Endocrine Metab Dis, Palermo, Italy; [Mocciaro, F.; Di Mitri, R.] ARNAS Civ Cristina Di Benfratelli Hosp, Gastroenterol & Endoscopy Unit, Palermo, Italy; [Messina, M.; Spada, M.] Fdn Ist G Giglio, Oncol Unit, Cefalu, Italy; [Tomasello, G.; Cappello, F.] Univ Palermo, Sect Human Anat, Dept Expt Biomed & Clin Neurosci, Palermo, Italy; [Leone, A.] Univ Palermo, Sect Histol, Dept Expt Biomed & Clin Neurosci, Palermo, Italy; [Morreale, G. C.] Osped Riuniti Villa Sofia Vincenzo Cervello, Unit Gastroenterol, Palermo, Italy; [Marasa, S.] Ctr Diagnost Marasa, Palermo, Italy; [Midiri, M.; Midiri, F.] Univ Palermo, Sect Radiol, DIBIMED, Palermo, Italy University of Palermo; A.R.N.A.S. Ospedali Civico Di Cristina Benfratelli; University of Palermo; University of Palermo; University of Palermo Sinagra, E (corresponding author), Fdn Ist Giuseppe Giglio, Gastroenterol & Endoscopy Unit, I-90015 Cefalu, Italy. emanuelesinagra83@googlemail.com Federico, Massimo/J-5984-2014; MESSINA, MARCO/LMP-3029-2024; Mocciaro, Filippo/AAC-7257-2022; Di Mitri, Roberto/AAC-7452-2022; Cappello, Francesco/F-9153-2012 SPADA, MASSIMILIANO/0000-0003-1025-0153; Cappello, Francesco/0000-0001-9288-1148; Mocciaro, Filippo/0000-0002-4389-

0643; TOMASELLO, GIOVANNI/0000-0002-3071-822X
17 4 5 0 4 BIOLIFE SAS SILVA MARINA (TE) VIA S STEFANO 39 BIS, 64029 SILVA MARINA (TE), ITALY
0393-974X 1724-6083 J BIOL REG HOMEOS AG J. Biol. Regul. Homeost. Agents OCT-DEC 2017 31 4
1101 1107 7
Endocrinology & Metabolism; Immunology; Medicine, Research & Experimental; Physiology Science Citation Index Expanded (SCI-EXPANDED) Endocrinology & Metabolism; Immunology; Research & Experimental Medicine; Physiology FX6HP 29254321
2025-06-24 WOS:000426184600036
J Notarnicola, M; Pisanti, S; Tutino, V; Bocale, D; Rotelli, MT; Gentile, A; Memeo, V; Bifulco, M; Perri, E; Caruso, MG
Notarnicola, Maria; Pisanti, Simona; Tutino, Valeria; Bocale, Domenica; Rotelli, Maria Teresa; Gentile, Antonio; Memeo, Vincenzo; Bifulco, Maurizio; Perri, Enzo; Caruso, Maria Gabriella Effects of olive oil polyphenols on fatty acid synthase gene expression and activity in human colorectal cancer cells GENES AND NUTRITION
English Article
Hydroxytyrosol; Oleuropein; Fatty acid synthase; Colorectal cancer MEDITERRANEAN DIET; ANTIOXIDANT; MODULATION; ACTIVATION; UNDERLIES; SURVIVAL; PHENOLS; GROWTH; RICH
Oleuropein (OL) and hydroxytyrosol (HT), the main olive oil polyphenols, possess anti-proliferative effects in vitro. Fatty acid synthase, a key anabolic enzyme of biosynthesis of fatty acids, plays an important role in colon carcinoma development. Our aim was to investigate whether gene expression of FAS, as well as its enzymatic activity, is regulated by HT and OL in two human colon cancer cell lines, as HT-29 and SW620. In addition, we investigated the effects of these polyphenols on growth and apoptosis in these cells. FAS gene expression and activity in treated HT-29 and SW620 cells were evaluated by real-time PCR and radiochemical assay, respectively. Cell growth and apoptosis, after polyphenols treatment, were measured by MTT test and flow cytometry, respectively. The inhibition of proliferation, detected after HT treatment, was mediated by an inhibition of FAS expression and its enzymatic activity in SW620 cells, while the anti-proliferative effect in HT-29 cells seems to be independent from FAS. OL exerted an anti-proliferative effect only on SW620 cells with a mechanism which excluded FAS. Olive oil polyphenols used were able to induce apoptosis in both cell lines studied. The increase of apoptosis in these cells was accompanied by the block of cell cycle in the S phase. This study demonstrates that HT and OL may induce anti-proliferative and pro-apoptotic effects only in certain human colorectal cancer cell types. These effects are FAS mediated only in SW620 cells after treatment with HT.
[Notarnicola, Maria; Tutino, Valeria; Gentile, Antonio; Caruso, Maria Gabriella] Natl Inst Digest Dis S de Bellis, Biochem Lab, I-70013 Bari, Italy; [Pisanti, Simona; Bifulco, Maurizio] Univ Salerno, Dept Pharmaceut Sci, I-84100 Salerno, Italy; [Bocale, Domenica; Rotelli, Maria Teresa; Memeo, Vincenzo] Univ Bari, DETO Div Surg, Bari, Italy; [Perri, Enzo] CRA Res Ctr Olive Growing & Olive Oil Ind, Arcavacata Di Rende, CS, Italy IRCCS Saverio de Bellis; University of Salerno; Universita degli Studi di Bari Aldo Moro Caruso, MG (corresponding author), Natl Inst Digest Dis S de Bellis, Biochem Lab, Via Resistenza, I-70013 Bari, Italy. gabriella.caruso@irccsdebellis.it Bifulco,

Maurizio/HOA-6546-2023; Pisanti, Simona/J-3521-2012; PERRI, ENZO/KAM-0129-2024 PERRI, ENZO/0000-0002-6314-7136; Bifulco, Maurizio/0000-0002-1771-4531 29 89 94 0 21 BMC LONDON CAMPUS, 4 CRINAN ST, LONDON N1 9XW, ENGLAND 1555-8932 1865-3499 GENES NUTR Genes Nutr. FEB 2011 6 1 63 69 10.1007/s12263-010-0177-7 http://dx.doi.org/10.1007/s12263-010-0177-7 7 Genetics & Heredity; Nutrition & Dietetics Science Citation Index Expanded (SCI-EXPANDED) Genetics & Heredity; Nutrition & Dietetics 723NT 21437031 Bronze, Green Published 2025-06-24 WOS:000287514200007

J Ajiboye, BO; Fatoki, TH; Akinnusi, PA; Ajuwon, OR; Oyinloye, BE; Jeje, TO; Owolabi, OV; Ogedengbe, OO; Genovese, C Ajiboye, Basiru Olaitan; Fatoki, Toluwase Hezekiah; Akinnusi, Precious Ayorinde; Ajuwon, Olawale Rasaq; Oyinloye, Babatunji Emmanuel; Jeje, Temitope Olawale; Owolabi, Olutunmise Victoria; Ogedengbe, Oluwatosin O.; Genovese, Claudia Molecular docking, MMGBSA, and ADMET studies of phytoconstituents of Ocimum gratissimum on multiple breast cancer targets NATURAL PRODUCT RESEARCH English Article; Early Access Ocimum gratissimum; breast cancer; EGFR; HER2; PI3K; binding affinity; pharmacokinetics APOPTOSIS; CARCINOMA; CELLS; INHIBITION; ISOVITEXIN; EXTRACT; GROWTH; ACID O. gratissimum is one of the most common medicinal plants in every community in Nigeria. This plant has been presumed to be useful in the management of diseases including breast cancer, which is one the commonest cancers affecting women globally. Hence, this study aimed to computationally investigate the phytochemicals present in O. gratissimum by elucidate their binding dynamics against five selected molecular targets of breast cancer and predict their pharmacokinetics properties. Molecular docking, MMGBSA calculation and ADMET prediction were used. The results showed that isovitexin has the highest binding affinity of -9.11 kcal/mol and -9.80 kcal/mol for Human Epidermal Growth Factor Receptor 2 (HER2) and Epidermal Growth Factor Receptor (EGFR) respectively. Rosmarinic acid has the highest binding affinity of -12.15 kcal/mol for Phosphatidylinositol 3-kinase (PI3K), Nepetoidin A has the highest binding affinity of -9.14 kcal/mol for oestrogen receptor (ER), and Vitexin has the highest binding affinity of -12.90 kcal/mol for Progesterone receptor (PR). MMGBSA provided total binding energy that confirmed the stability of the complexes under physiological conditions. The ADMET profiles showed that O. gratissimum top phytochemicals identified would be safe for oral administration with no hepatoxicity. Overall, this study identified isovitexin, vitexin, rosmarinic acid, nepetoidin A and luteolin among others, as compounds that exhibit strong anti-cancer properties against breast cancer cells. Graphical Abstract [Ajiboye, Basiru Olaitan] Fed Univ Oye Ekiti, Dept Biochem, Phytomedicine & Mol Toxicol Res Lab, Oye Ekiti, Nigeria; [Fatoki, Toluwase Hezekiah; Akinnusi, Precious Ayorinde] Fed Univ Oye Ekiti, Dept Biochem, Bioinformat & Enzymol Res Lab, Oye Ekiti, Nigeria; [Ajuwon, Olawale Rasaq] Fed Univ Oye Ekiti, Dept Biochem, Redox Biol Res Unit, Oye Ekiti, Nigeria; [Oyinloye, Babatunji Emmanuel] Afe Babalola Univ, Coll Sci, Dept Biochem, Phytomed Biochem Toxicol & Biotechnol Res Labs, Ado Ekiti, Nigeria; [Oyinloye, Babatunji Emmanuel] Univ Zululand, Dept Biochem &

Microbiol, Biotechnol & Struct Biol BSB Grp, Kwa Dlangezwa, South Africa; [Jeje, Temitope Olawale] Fed Univ Oye Ekiti, Dept Biochem, Biochem Immunol & Phytomedicine Lab, Oye Ekiti, Nigeria; [Owolabi, Olutunmise Victoria] Afe Babalola Univ, Coll Med & Hlth Sci, Med Biochem Unit, Ado Ekiti, Nigeria; [Ogedengbe, Oluwatosin O.] Fed Univ Oye Ekiti, Fac Basic Med Sci, Dept Anat, Oye Ekiti, Nigeria; [Genovese, Claudia] Inst Agr & Forestry Syst Mediterranean, Catania, Italy University of Zululand; Consiglio Nazionale delle Ricerche (CNR); Istituto per i Sistemi Agricoli e Forestali del Mediterraneo (ISAFoM-CNR) Ajiboye, BO (corresponding author), Fed Univ Oye Ekiti, Dept Biochem, Phytomedicine & Mol Toxicol Res Lab, Oye Ekiti, Nigeria.; Genovese, C (corresponding author), Inst Agr & Forestry Syst Mediterranean, Catania, Italy.
bash1428@yahoo.co.uk; claudia.genovese@cnr.it Fatoki, Toluwase/AAD-3789-2021; Ogedengbe, Oluwatosin/KSM-7173-2024; Genovese, Claudia/AAE-1588-2021; Oyinloye, Babatunji/J-1943-2017
GENOVESE, CLAUDIA/0000-0003-2202-468X
48 1 1 0 8 TAYLOR & FRANCIS LTD ABINGDON
2-4 PARK SQUARE, MILTON PARK, ABINGDON OR14 4RN, OXON, ENGLAND 1478-6419 1478-6427 NAT PROD RES Nat. Prod. Res. 2024 APR 17 2024
10.1080/14786419.2024.2344193
http://dx.doi.org/10.1080/14786419.2024.2344193 APR 2024 9 Chemistry, Applied; Chemistry, Medicinal Science Citation Index Expanded (SCI-EXPANDED) Chemistry; Pharmacology & Pharmacy OK1H9 38648537 Green Submitted 2025-06-24 WOS:001207068700001
J Ergas, IJ; Feliciano, EMC; Bradshaw, PT; Roh, JM; Kwan, ML; Cadenhead, J; Santiago-Torres, M; Troeschel, AN; Laraia, B; Madsen, K; Kushi, LH Ergas, Isaac J.; Feliciano, Elizabeth M. Cespedes; Bradshaw, Patrick T.; Roh, Janise M.; Kwan, Marilyn L.; Cadenhead, Jen; Santiago-Torres, Margarita; Troeschel, Alyssa N.; Laraia, Barbara; Madsen, Kristine; Kushi, Lawrence H. Diet Quality and Breast Cancer Recurrence and Survival: The Pathways Study JNCI CANCER SPECTRUM English Article
MEDITERRANEAN DIET; ADHERENCE; WOMEN; RISK; DIAGNOSIS; NUTRITION; MORTALITY; PATTERNS; THERAPY; DISEASE
Background: Prior research suggests a relationship between overall diet quality and breast cancer survival, although few studies have reported on this topic. We evaluated whether 4 dietary quality indices consistent with healthy eating recommendations around the time of breast cancer diagnosis were associated with risk of recurrence, cause-specific, and allcause mortality. Methods: A total of 3660 women diagnosed with invasive breast cancer were included. Diet was assessed an average of 2.3 (range = 0.7-18.7) months after diagnosis, from which 4 dietary quality indices were derived: the American Cancer Society guidelines (ACS), the alternate Mediterranean Diet Index (aMED), the Dietary Approaches to Stop Hypertension (DASH), and the 2015 Healthy Eating Index (HEI). Over 40 888 person-years of follow-up, 461 breast cancer recurrences, and 655 deaths were ascertained. Cox models were used to estimate hazards ratios (HRs) and 95% confidence intervals (CIs). Results: Adjusted comparisons between extreme quintiles showed all 4 dietary quality indices to be inversely associated with all-cause mortality, suggesting a 21%-27% lower risk (ACS HR = 0.73, 95% CI = 0.56 to 0.95; aMED HR = 0.79, 95% CI = 0.61 to 1.03; DASH HR = 0.76, 95% CI = 0.58 to

1.00; HEI HR = 0.77, 95% CI = 0.60 to 1.01). Similar patterns were noted for non-breast cancer mortality (ACS HR = 0.69, 95% CI = 0.48 to 0.98; aMED HR = 0.73, 95% CI = 0.50 to 1.05; DASH HR = 0.55, 95% CI = 0.38 to 0.79; HEI HR = 0.67, 95% CI = 0.48 to 0.94). None of the dietary quality indices were associated with recurrence or breast cancer-specific mortality. Conclusion: Food intake patterns concordant with dietary quality indices consistent with recommendations for healthy eating may be beneficial for women with breast cancer. [Ergas, Isaac J.; Feliciano, Elizabeth M. Cespedes; Roh, Janise M.; Kwan, Marilyn L.; Cadenhead, Jen; Kushi, Lawrence H.] Kaiser Permanente Northern Calif, Div Res, 2000 Broadway, Oakland, CA 94612 USA; [Bradshaw, Patrick T.; Laraia, Barbara] Univ Calif Berkeley, Sch Publ Hlth, Div Epidemiol & Biostat, Berkeley, CA 94720 USA; [Santiago-Torres, Margarita] Fred Hutchinson Canc Res Ctr, Div Publ Hlth Sci, Canc Prevent Program, 1124 Columbia St, Seattle, WA 98104 USA; [Troeschel, Alyssa N.] Emory Univ, Rollins Sch Publ Hlth, Dept Epidemiol, Atlanta, GA 30322 USA; [Laraia, Barbara; Madsen, Kristine] Univ Calif Berkeley, Sch Publ Hlth, Div Community Hlth Sci, Berkeley, CA 94720 USA Kaiser Permanente; University of California System; University of California Berkeley; Fred Hutchinson Cancer Center; Emory University; Rollins School Public Health; University of California System; University of California Berkeley Ergas, IJ (corresponding author), Kaiser Permanente Northern Calif, Div Res, 2000 Broadway, Oakland, CA 94612 USA. isaac.j.ergas@kp.org
Bradshaw, Patrick/AAJ-7529-2021; Feliciano, Elizabeth/AAD-4459-2020; Laraia, Barbara/GXG-1829-2022; Kwan, Marilyn/AAH-4850-2021 Santiago-Torres, Margarita/0000-0001-6051-3172; Cespedes Feliciano, Elizabeth/0000-0003-1192-4017; Kwan, Marilyn/0000-0001-8863-3950; Ergas, Isaac/0000-0002-3187-9754; Bradshaw, Patrick/0000-0001-7761-3129; Madsen, Kristine/0000-0002-1880-5363
American Institute for Cancer Research [632996]; National Cancer Institute at the National Institutes of Health [U01 CA195565, R01 CA105274, K01 CA226155] American Institute for Cancer Research; National Cancer Institute at the National Institutes of Health(United States Department of Health & Human ServicesNational Institutes of Health (NIH) - USANIH National Cancer Institute (NCI)) This work was supported by grants from the American Institute for Cancer Research (grant number 632996); and the National Cancer Institute at the National Institutes of Health (grant numbers U01 CA195565, R01 CA105274, K01 CA226155).
36 28 28 1 4 OXFORD UNIV PRESS
OXFORD GREAT CLARENDON ST, OXFORD OX2 6DP, ENGLAND
2515-5091 JNCI CANCER SPECT JNCI Cancer Spectr.
APR 2021 5 2
pkab019 10.1093/jncics/pkab019
http://dx.doi.org/10.1093/jncics/pkab019 MAR 2021
9 Oncology Emerging Sources Citation Index (ESCI)
Oncology SS1UR 33928215 Green Published, gold
2025-06-24 WOS:000661527500010
J Vecchio, A; Anzidei, M; Serpelloni, E
Vecchio, A.; Anzidei, M.; Serpelloni, E. Sea level rise projections up to 2150 in the northern Mediterranean coasts ENVIRONMENTAL RESEARCH LETTERS English
Article Mediterranean sea; sea level rise projections; vertical land movements LAND SUBSIDENCE; DEFORMATION; SCENARIOS; VELOCITY; MECHANISMS; DELTAS; RATES; FIELD Vertical land movements (VLM) play a crucial role

in affecting the sea level rise along the coasts. They need to be estimated and included in the analysis for more accurate Sea Level (SL) projections. Here we focus on the Mediterranean basin characterized by spatially variable rates of VLM that affect the future SL along the coasts. To estimate the VLM rates we used geodetic data from continuous global navigation satellite system stations with time series longer than 4.5 years in the 1996-2023 interval, belonging to Euro-Mediterranean networks and located within 5 km from the coast. Revised SL projections up to the year 2150 are provided at 265 points on a geographical grid and at the locations of 51 tide gauges of the Permanent Service for Mean Sea Level, by including the estimated VLM in the SL projections released by the Intergovernmental Panel on Climate Change (IPCC) in the AR6 Report. Results show that the IPCC projections underestimate future SL along the coasts of the Mediterranean Sea since the effects of tectonics and other local factors were not properly considered. Here we show that revised SL projections at 2100, when compared to the IPCC, show a maximum and minimum differences of 1094 +/- 103 mm and -773 +/- 106 mm, respectively, with an average value that exceeds by about 80 mm that of the IPCC in the reference Shared Socio-economic Pathways and different global warming levels. Finally, the projections indicate that about 19.000 km2 of the considered Mediterranean coasts will be more exposed to risk of inundation for the next decades, leading to enhanced impacts on the environment, human activities and infrastructures, thus suggesting the need for concrete actions to support vulnerable populations to adapt to the expected SL rise and coastal hazards by the end of this century. [Vecchio, A.] Radboud Univ Nijmegen, Dept Astrophys, Radboud Radio Lab, Nijmegen, Netherlands; [Vecchio, A.] Univ Paris, Univ PSL, Sorbonne Univ, CNRS,LESIA,Observ Paris, Meudon, France; [Vecchio, A.; Anzidei, M.] Ist Nazl Geofis & Vulcanol, Rome, Italy; [Serpelloni, E.] Ist Nazl Geofis & Vulcanol, Bologna, Italy
Radboud University Nijmegen; Centre National de la Recherche Scientifique (CNRS); Universite PSL; Observatoire de Paris; Universite Paris Cite; Sorbonne Universite; Istituto Nazionale Geofisica e Vulcanologia (INGV); Istituto Nazionale Geofisica e Vulcanologia (INGV) Vecchio, A (corresponding author), Radboud Univ Nijmegen, Dept Astrophys, Radboud Radio Lab, Nijmegen, Netherlands.; Vecchio, A (corresponding author), Univ Paris, Univ PSL, Sorbonne Univ, CNRS,LESIA,Observ Paris, Meudon, France.; Vecchio, A (corresponding author), Ist Nazl Geofis & Vulcanol, Rome, Italy. a.vecchio@astro.ru.nl Anzidei, Marco/A-3873-2017; Serpelloni, Enrico/KGK-5864-2024 Anzidei, Marco/0000-0003-1935-1049 EU [ECHO/SUB/2016/742473/PREV16, 874398]; Italian Ministry of Research [CUP D53J19000170001] EU(European Union (EU)); Italian Ministry of Research(Ministry of Education, Universities and Research (MIUR)) We acknowledge the two anonymous Reviewers for the useful comments. This research has been supported by the SAVEMEDCOASTS (Agreement number ECHO/SUB/2016/742473/PREV16 www. savemedcoasts.eu) and SAVEMEDCOASTS2 (Project Number 874398 www.savemedcoasts2.eu) projects, both funded by the EU under the umbrella of the DGECHO, and the 'Working Earth' project funded by the Italian Ministry of Research (CUP D53J19000170001). We thank the IPCC AR6 projection authors for developing and making the sea level rise projections available, multiple funding agencies for supporting the development of the projections, and the NASA Sea Level Change Team

for developing and hosting the IPCC AR6 Sea Level Projection Tool.
78 16 16 1 28 IOP Publishing Ltd
BRISTOL TEMPLE CIRCUS, TEMPLE WAY, BRISTOL BS1 6BE, ENGLAND 1748-9326 ENVIRON RES LETT Environ. Res. Lett. JAN 1 2024 19 1
014050 10.1088/1748-9326/ad127e
http://dx.doi.org/10.1088/1748-9326/ad127e 14
Environmental Sciences; Meteorology & Atmospheric Sciences
Science Citation Index Expanded (SCI-EXPANDED)
Environmental Sciences & Ecology; Meteorology & Atmospheric Sciences CS7N9 gold 2025-06-24
WOS:001127296700001
J Brown, R; Kerr, K; Haoudi, A; Darzi, A
Brown, Robert; Kerr, Karen; Haoudi, Abdelali; Darzi, Ara
Tackling cancer burden in the Middle East: Qatar as an example LANCET ONCOLOGY English Article
BREAST-CANCER; LOW-RESOURCE; MANAGEMENT; COUNTRIES; CONSENSUS; CARE Cancer prevalence is increasing in the Middle East, partly because of increased life expectancy and adoption of western lifestyle habits. Suboptimum delivery of health care also contributes to late diagnosis and poor survival of people with cancer. Public awareness of cancer risk is frequently low and misconceptions high, thereby preventing patients from seeking treatment early and constituting a substantial barrier to improvement of cancer outcomes. Screening programmes might have low uptake in Arab populations because of social and health beliefs about cancer. This review outlines the opportunities available to Middle Eastern countries and their emerging economies to learn from global experiences in cancer care, service provision, and research partnerships. The Middle East has begun to develop several health-care transformation programmes. Qatar, in particular, has published a National Health Strategy, in which cancer is one of the main commitments; this Strategy provides the focus of this review. The development of effective health-care strategies and evidence-based medicine directly linked to innovative cancer research is needed to improve cancer care. Although the full extent of the proposed solutions are not necessarily implementable in all Middle Eastern countries, wealthy states can lead derivation of population-specific approaches that could have effects throughout the region. Key challenges are outlined-namely, human capacity and training, subspecialisation of services, building on international cancer research initiatives, and the need for earlier diagnosis and awareness in the population. Countries in the Gulf Region (ie, countries bordering the Persian Gulf, including Iran, Iraq, Kuwait, Saudi Arabia, Bahrain, Qatar, UAE, and Oman) need to address these challenges to be at the forefront of integrated cancer care and research and ensure that the latest innovations and best possible care are delivered to their populations.
[Kerr, Karen; Darzi, Ara] Univ London Imperial Coll Sci Technol & Med, Div Surg, Dept Surg & Canc, St Marys Hosp, London W2 1NY, England; [Haoudi, Abdelali] Qatar Fdn, Doha, Qatar; [Brown, Robert] Univ London Imperial Coll Sci Technol & Med, Div Canc, Dept Surg & Canc, London W2 1NY, England Imperial College London; Qatar Foundation (QF); Imperial College London
Darzi, A (corresponding author), Univ London Imperial Coll Sci Technol & Med, Div Surg, Dept Surg & Canc, St Marys Hosp, London W2 1NY, England. a.darzi@imperial.ac.uk Brown,

Robert/0000-0001-7960-5755 41 41 44
0 23 ELSEVIER SCIENCE INC NEW YORK STE 800, 230
PARK AVE, NEW YORK, NY 10169 USA 1470-2045 1474-5488
LANCET ONCOL Lancet Oncol. NOV 2012 13 11
E501 E508 10.1016/S1470-
2045(12)70461-8 http://dx.doi.org/10.1016/S1470-2045(12)70461-8
8 Oncology Science Citation Index Expanded
(SCI-EXPANDED) Oncology 030KZ 23084766
2025-06-24 WOS:000310570900022
J Xie, W; Zuo, J; Ma, Z; Yu, W; Hu, Z; Yang, TS; Song, ZS
Xie, W.; Zuo, J.; Ma, Z.; Yu, W.; Hu, Z.; Yang,
Tingsong; Song, Zhenshun The Burden of Colorectal
Cancer Attributable to Diet Low in Fiber from 1990 to 2019: A
Global, Regional and National Analysis JOURNAL OF NUTRITION
HEALTH & AGING English Article
Colorectal cancer; death; diet low in fiber;
disability-adjusted life-year; global burden of disease study
UNITED-STATES; TRENDS Objectives The colorectal cancer (CRC)
burden is increasingly high. The aim of this study was to
investigate temporal and geographical trends in CRC deaths and
disability-adjusted life-years (DALYs) attributable to diet low in
fiber globally from 1990 to 2019. Design Cross-sectional study.
Setting The study based on the Global Burden of Disease Study
(GBD) 2019. Participants The population comprised individuals from
204 countries and territories who were diagnosed with CRC
attributable to diet low in fiber from 1990 to 2019. Measurements
Deaths, DALYs, age-standardized mortality rates (ASMR), and age-
standardized DALY rates (ASDR) for CRC attributable to diet low in
fiber were described, and estimated annual percentage change
(EAPC) was further calculated to assess the burden in different
regions, countries, sexes, and age groups. Additionally, we
explored the association between EAPC and ASMR/ASDR (in 1990) and
Human Development Index (HDI, in 2019). Results From 1990 to 2019,
global ASMR and ASDR for CRC attributable to diet low in fiber
decreased slightly, but the corresponding deaths and DALYs
increased by 63.37% and 51.36%, respectively. Those burden varied
considerably between regions and countries. The burden was higher
in high, high-middle and middle SDI regions, especially in Asia
and Western Europe, but when HDI > 0.7, an increasingly rapid
decline in ASMR and ASDR was revealed. Unexpectedly, many less
well-developed countries within the traditionally low deaths and
DALYs regions of Africa, Central Latin America, and Middle East
showed gradual increases in ASMR and ASDR. Conclusion The global
burden of CRC attributable to diet low in fiber has decreased over
the last 30 years, but remains at a high level. It is essential
for decision-makers to take targeted measures for improving
population awareness and intake of dietary fiber. [Xie, W.; Zuo,
J.; Yu, W.; Hu, Z.; Yang, Tingsong; Song, Zhenshun] Tongji Univ,
Sch Med, Shanghai Peoples Hosp 10, Dept Gen Surg, 301 Yanchang Rd,
Shanghai 200072, Peoples R China; [Ma, Z.] Shanghai Jiao Tong
Univ, Sch Med, Tongren Hosp, Dept Gen Surg, Shanghai, Peoples R
China; [Song, Zhenshun] Tongji Univ, Sch Med, Shanghai Peoples
Hosp 4, Dept Gen Surg, 1279 Sanmen Rd, Shanghai 200072, Peoples R
China Tongji University; Shanghai Jiao Tong University; Tongji
University Yang, TS; Song, ZS (corresponding author), Tongji
Univ, Sch Med, Shanghai Peoples Hosp 10, Dept Gen Surg, 301
Yanchang Rd, Shanghai 200072, Peoples R China.; Song, ZS
(corresponding author), Tongji Univ, Sch Med, Shanghai Peoples

Hosp 4, Dept Gen Surg, 1279 Sanmen Rd, Shanghai 200072, Peoples R
China.   tingsong.yang@tongji.edu.cn; zs_song@tongji.edu.cn
37   5   5   0   11
ELSEVIER SCIENCE INC   NEW YORK   STE 800, 230 PARK AVE, NEW
YORK, NY 10169 USA   1279-7707   1760-4788   J NUTR HEALTH
AGING J. Nutr. Health Aging   DEC   2022   26   12
1061   1069   10.1007/s12603-022-1865-x
http://dx.doi.org/10.1007/s12603-022-1865-x   NOV 2022
9   Geriatrics & Gerontology; Nutrition & Dietetics
Science Citation Index Expanded (SCI-EXPANDED)
Geriatrics & Gerontology; Nutrition & Dietetics   7C1KE
36519769   hybrid   2025-06-24
WOS:000883296100001
J   Blesa, JR; García, JA; Ochoa, E   Rafael
Blesa, Jose; Angel Garcia, Jose; Ochoa, Enrique
Frequency of Germ-Line BRCA1 Mutations among Spanish
Families from a Mediterranean Area   HUMAN MUTATION
English   Article   BRCA1;
breast cancer; CSGE; mutation screening   We have carried out a
study of breast cancer in Spanish families in which the entire
coding region of the BRCA1 gene have been analyzed. To identify
BRCA1 mutations, PTT and CSGE methods were used followed by direct
sequencing. We investigated 51 breast cancer women with a family
history. Among these we have identified 7 frameshifts mutations
(15%), 185delAG (4 times), 1623del5 and 3450del4 (2 times), and 3
missense mutations, Ser1613Gly, Met1652Ile and Ala1708Glu, which
are likely polymorphisms. These findings show that BRCA1 is
implicated in a fraction of Spanish familial breast cancer similar
to other countries. There was association between bilateral breast
cancer and BRCA1 mutations. The CSGE technique has been
demonstrated to be a highly reliable method for mutation screening
because of its sensitivity and high throughput. (C) 2000Wiley-
Liss, Inc. [Rafael Blesa, Jose; Angel Garcia, Jose; Ochoa,
Enrique] Hosp Gen Univ, Dept Anat Patol, Valencia, Spain
Blesa, JR (corresponding author), Inst Invest Citological
Caja Ahorros Valencia, Amadeo Saboya 4, Valencia 46010, Spain.
blesa@edunet.es   OCHOA, ENRIQUE/HKM-7935-2023; Blesa, Jose
R./P-4100-2014   Blesa, Jose R./0000-0003-1773-2278
Generalitat Valenciana, Spain [GV 3231/95]   Generalitat
Valenciana, Spain(Center for Forestry Research & Experimentation
(CIEF))   Contract grant sponsor: Generalitat Valenciana, Spain;
Contract grant number: GV 3231/95.   14   26   30   0
0   WILEY   HOBOKEN   111 RIVER ST, HOBOKEN 07030-5774, NJ
USA   1059-7794   1098-1004   HUM MUTAT   Hum. Mutat.   APR   2000
15   4   381   +
10.1002/(SICI)1098-1004(200004)15:4<381::AID-
HUMU14>3.0.CO;2-H   http://dx.doi.org/10.1002/(SICI)1098-
1004(200004)15:4<381::AID-HUMU14>3.0.CO;2-H   5
Genetics & Heredity   Science Citation Index Expanded (SCI-
EXPANDED)   Genetics & Heredity   V23XT   10737987   gold
2025-06-24   WOS:000208376200004
J   Sunar, V; Ates, Ö; Korcali Aslan, A; Karakas, Y; Altundag,
MK   Sunar, Veli; Ates, Ozturk; Korcali Aslan,
Alma; Karakas, Yusuf; Altundag, Mustafa Kadri   The
Prevalence of Systemic Rheumatic Diseases Among Breast Cancer
Patients and Its Relationship With Survival   ARCHIVES OF
RHEUMATOLOGY   English   Article
Breast cancer; prevalence; survival; systemic

rheumatic diseases	IN-SITU; CYTOKINES; ESTROGEN; TUMORS; RISK
Objectives: This study aims to investigate the prevalence of systemic rheumatic diseases (SRDs) among patients with breast cancer (BC) and to identify the clinicopathological characteristics of these patients. Patients and methods: A total of 3,744 female patients with BC (mean age 49 +/- 11.7 years; range, 18 to 92 years) followed in Hacettepe University Faculty of Medicine, Medical Oncology Department between January 2006 and December 2015 were retrospectively assessed. Patients with or without SRD were compared in terms of clinicopathological features including age, menopausal state, smoking status, body mass index (BMI), age of menarche, age at first labor, and number of children. The groups were also evaluated regarding tumor grade, stage, estrogen receptor and progesterone receptor expression, human epidermal growth factor receptor 2 overexpression, and survival. Results: Of the patients analyzed, 68 (1.81%) had concomitant SRD. Among these patients, 33 (48.6%) had rheumatoid arthritis, eight (11.8%) had familial Mediterranean fever, eight (11.8%) had Behcet's disease, four (5.8%) had Sjogren's syndrome, four (5.8%) had systemic lupus erythematosus, six (8.8%) had ankylosing spondylitis, three (4.4%) had systemic sclerosis, one (1.4%) had polymyositis, and one (1.4%) had temporal arteritis. The groups with or without SRDs were similar in terms of age, smoking status, BMI, menopausal state, breast feeding duration, age at menarche and first birth. Stage 1 and 2 BC was more prevalent in SRD patients (74.6% vs. 64.5%, p=0.018). The rate to receive chemotherapy was significantly lower in patients with SRD. However, there was no significant difference in five-year overall survival rates between patients with or without SRD. Conclusion: Among patients with BC, 1.81% had concomitant SRD. These patients were diagnosed at early stages and given chemotherapy less frequently. However, they had similar survival rates compared to those without SRDs.
[Sunar, Veli; Korcali Aslan, Alma] Hacettepe Univ, Dept Med Oncol, Med Fac, Ankara, Turkey; [Ates, Ozturk] Abdurrahman Yurtaslan Training & Res Hosp, Dept Med Oncol, Ankara, Turkey; [Karakas, Yusuf] Hakkari Devlet Hastanesi, Dept Med Oncol, Hakkari, Turkey; [Altundag, Mustafa Kadri] MKA Breast Canc Clin, Dept Med Oncol, Ankara, Turkey	Hacettepe University; Dr. Abdurrahman Yurtaslan Oncology Hospital; Hakkari State Hospital	Sunar, V (corresponding author), Hacettepe Univ, Tip Fak, Med Onkoloji Bilim Dali, TR-06230 Ankara, Turkey.
velisunar@gmail.com	SUNAR, Veli/AAG-3220-2021; karakas, yusuf/AAF-5441-2020; ates, ozturk/GYA-3954-2022	Sunar, Veli/0000-0003-4672-4621; ates, ozturk/0000-0003-0182-3933
27	2	2	0	1	TURKISH LEAGUE AGAINST RHEUMATISM	ANKARA	TALATPASA BULVARI DUMLUPINAR CAD 40 3 CEBECI DORTYOL, ANKARA, 06100, TURKEY	2148-5046	1309-0283
ARCH RHEUMATOL	Arch. Rheumatol.	JUN	2019	34	2
141	147
10.5606/ArchRheumatol.2019.6803
http://dx.doi.org/10.5606/ArchRheumatol.2019.6803
7	Rheumatology	Science Citation Index Expanded (SCI-EXPANDED)	Rheumatology	IA0TA	31497760	Green Submitted, gold, Green Published		2025-06-24	WOS:000469269200003
J	Ghosh, SK; Bera, T; Pal, S		Ghosh, Swapan Kumar; Bera, Tanmay; Pal, Sujoy		Antiproliferative, Apoptotic, and Antimigration Property of Ethyl Acetate Extract of Calocybe indica against HeLa and CaSki Cell Lines of Cervical

Cancer, and its Antioxidant and Mycochemistry Analysis MIDDLE EAST JOURNAL OF CANCER English Article
Mushroom; Cervical cancer; Cytotoxicity; Apoptosis; Metastasis; Bioactive compounds INHIBITS PROLIFERATION; MEDICINAL MUSHROOMS; HUMAN BREAST; CYCLE; GROWTH; ANTICANCER; ANTITUMOR; ARREST; SUBSTANCES; ACTIVATION

Background: The incidence rate of cervical cancer is increasing and its existing drugs are becoming more and more resistant. Therefore, we extracted the fruiting body of Calocybe indica edible mushroom in 90% ethyl acetate extract (EAE) and evaluated it as an anticancer property against HeLa and CaSki. Method: We performed cytotoxicity assay by MTT, cell morphological study by phase contrast microscope, and apoptosis study by nuclear morphology via DAPI staining under inverted microscopy; the expressions of proapoptotic and antiapoptotic genes and p53 were examined by Western blotting, cell cycle analysis, and cologenic and cell migration assay. Antioxidant content and activity assays were performed and for mycochemistry analysis of EAE, thin layer chromatography (TLC) was done. Results: EAE-treated HeLa and CaSki cells became round and showed condensed and fragmented nuclei. They inhibited the cell proliferation of both cancer cell lines in a dose-dependent manner. At maximum dose (1250 mu g/mL) after 24 h, the cell inhibition percentages of HeLa and CaSki cells were 97.12 +/- 10.01 and 98.52 +/- 10.08 (P<0.05), respectively. They upregulated the expression of p53, caspase 3, and caspase 9 while down-regulating BcL2 gene. Cell cycle became arrested at G2/M checkpoint of both cancer cell lines by EAE. EAE inhibited colony formation and cell migration. The antioxidant assay showed that EAE contained good amounts of phenolic compounds, flavonoids, and ascorbic acids and had good antioxidant activity. TLC supported the presence of bioactive components. Conclusion: The EAE of C. indica exerts very potent anticervical cancer effects. It is urgent that future studies analyze its bioactive compounds in detail and examine them in animal models. [Ghosh, Swapan Kumar; Bera, Tanmay; Pal, Sujoy] Ramakrishna Mission Vivekananda Centenary Coll Au, PG Dept Bot, Canc Res Unit, Kolkata, India

Ghosh, SK (corresponding author), Ramakrishna Mission Vivekananda Centenary Coll Au, PG Dept Bot, Canc Res Unit, Kolkata, India. gswapan582@gmail.com Ghosh, Swapan/ACP-9945-2022 DHESTBT, Gov. of West Bengal [840sanc/ST/P/ST/1G-11/2015] DHESTBT, Gov. of West BengalAuthors are grateful to Principal, RKMVC College, Rahara, and DHESTBT, Gov. of West Bengal for funding this research (Grant no.840sanc/ST/P/S&T/1G-11/2015).
61 10 11 0 5 SHIRAZ UNIV MEDICAL SCIENCES SHIRAZ NEMAZEE HOSPITAL, SHIRAZ, 71934, IRAN
2008-6709 2008-6687 MIDDLE EAST J CANCER Middle East J. Cancer FAL 2020 11 4 454
468 10.30476/mejc.2020.81870.1046
http://dx.doi.org/10.30476/mejc.2020.81870.1046
15 Oncology Emerging Sources Citation Index (ESCI)
Oncology OF0MM 2025-06-24
WOS:000580913500009

J Colomer, R; Lupu, R; Papadimitropoulou, A; Vellón, L; Vázquez-Martín, A; Brunet, J; Fernández-Gutíerrez, A; Segura-Carretero, A; Menéndez, JA Colomer, Ramon; Lupu, Ruth; Papadimitropoulou, Adriana; Vellon, Luciano; Vazquez-Martin, Alejandro; Brunet, Joan; Fernandez-Gutierrez, Alberto; Segura-Carretero, Antonio; Menendez, Javier A.

Giacomo!Castelvetro's salads.: Anti-HER2 oncogene nutraceuticals since the 17th century? CLINICAL & TRANSLATIONAL ONCOLOGY English Article

olive oil; HER2; Mediterranean diet; breast cancer; oleic acid; polyphenols BREAST-CANCER CELLS; MONOUNSATURATED FATTY-ACID; MEDITERRANEAN DIETARY TRADITIONS; VIRGIN OLIVE OIL; OLEIC-ACID; TRASTUZUMAB HERCEPTIN(TM); EXPRESSION; OVEREXPRESSION; AMPLIFICATION; NUTRIGENOMICSWe are accumulating evidence to suggest that 17th century Renaissance foodways-largely based on the old "Mediterranean dietary traditions"-may provide new nutraceutical management strategies against HER2-positive breast cancer disease in the 21st century. Epidemiological and experimental studies begin to support the notion that "The Sacred Law of Salads" (i.e., "raw vegetables. plenty of generous (olive) oil")-originally proposed in 1614 by Giacomo Castelvetro in its book The Fruit, Herbs & Vegetables of Italy -might be considered the first (unintended) example of customised diets for breast cancer prevention based on individual genetic make-up (i.e., nutraceuticals against human breast carcinomas bearing HER2 oncogene amplification/overexpression). First, the so-called salad vegetables dietary pattern (i.e., a high consumption of raw vegetables and olive oil) appears to exert a protective effect mostly confined to the HER2-positive breast cancer subtype, with no significant influence on the occurrence of HER2-negative breast cancers. Second, all the main olive oil constituents (i.e., the omega-9 monounsaturated fatty acid oleic acid and polyphenolic compounds such as the secoiridoid oleuropein or the lignan 1-[+]-acetoxypinoresinol] dramatically reduce HER2 expression and specifically induce apoptotic cell death in cultured HER2-positive breast cancer cells, with marginal effects against HER2-negative cells. Third, an olive oil-rich diet negatively influences experimental mammary tumorigenesis in rats likewise decreasing HER2 expression levels. If early 1600s Castelvetro's salads can be used as dietary protocols capable to protecting women against biologically aggressive HER2-positive breast cancer subtypes is an intriguing prospect that warrants to be evaluated in human pilot studies in the future. Here, at least, we would like to recognise Giacomo Castelvetro as the father of modern nutritional genomics in oncology. [Colomer, Ramon] MD Anderson Int Madrid, Madrid 28033, Spain; [Lupu, Ruth] Northwestern Univ, Feinberg Sch Med, Dept Med, Evanston, IL USA; [Papadimitropoulou, Adriana] Hellenic Pasteur Inst, Dept Biochem, Lab Mol Biol & Immunobiotechnol, Athens, Greece; [Vellon, Luciano] CIC bioGUNE, Spatial & Funct Cytogenet Grp, Bilbao, Spain; [Vazquez-Martin, Alejandro; Brunet, Joan; Menendez, Javier A.] Dr Josep Trueta Univ Hosp Girona, Girona Biomed Res Inst, Metab & Canc Lab, Hlth Serv Div Catalonia,ICO Girona, Girona 17007, Catalunya, Spain; [Fernandez-Gutierrez, Alberto; Segura-Carretero, Antonio] Univ Granada, Fac Sci, Dept Analyt Chem, Granada, Spain University of Texas System; UTMD Anderson Cancer Center; Northwestern University; Feinberg School of Medicine; CIC bioGUNE; Institut Catala d'Oncologia; Universitat de Girona; Girona University Hospital Dr. Josep Trueta; Institut d'Investigacio Biomedica de Girona (IDIBGI); University of Granada Colomer, R (corresponding author), MD Anderson Int Madrid, Arturo Soria 270, Madrid 28033, Spain. rcolomer@seom.org; jmenendez@ico.scs.es segura Carretero, Antonio/B-6867-2014; MENENDEZ MENENDEZ, JAVIER ABEL/C-6148-2016; Fernandez Gutierrez, Alberto/M-8512-2014; Brunet,

Joan/C-5292-2018; Colomer, Ramon/C-5142-2008 segura Carretero, Antonio/0000-0002-5564-5338; MENENDEZ MENENDEZ, JAVIER ABEL/0000-0001-8733-4561; Fernandez Gutierrez, Alberto/0000-0003-3647-2598; Brunet, Joan/0000-0003-1945-3512; Colomer, Ramon/0000-0002-6393-3444 34 16 18 0 6 SPRINGER INTERNATIONAL PUBLISHING AG CHAM GEWERBESTRASSE 11, CHAM, CH-6330, SWITZERLAND 1699-048X 1699-3055 CLIN TRANSL ONCOL Clin. Transl. Oncol. JAN 2008 10 1
30 34 10.1007/s12094-008-0151-7
http://dx.doi.org/10.1007/s12094-008-0151-7 5
Oncology Science Citation Index Expanded (SCI-EXPANDED)
Oncology 316JF 18208790 2025-06-24
WOS:000256944900006
J Barchitta, M; Maugeri, A; Quattrocchi, A; Agrifoglio, O; Scalisi, A; Agodi, A Barchitta, Martina; Maugeri, Andrea; Quattrocchi, Annalisa; Agrifoglio, Ottavia; Scalisi, Aurora; Agodi, Antonella The Association of Dietary Patterns with High-Risk Human Papillomavirus Infection and Cervical Cancer: A Cross-Sectional Study in Italy NUTRIENTS
English Article
cervical intraepithelial neoplasia; Mediterranean diet score; principal component analysis; Western diet; prudent diet
NATURAL-HISTORY; VITAMIN-A; PREVENTION; FOLATE; WOMEN; EPIDEMIOLOGY; EPIGENOME; NEOPLASIA Specific foods and nutrients help prevent the progression of persistent high-risk human papillomavirus (hrHPV) infection to cervical cancer (CC). The aim of this study was to investigate dietary patterns which may be associated with hrHPV status and the risk of high-grade cervical intraepithelial neoplasia (CIN2+). Overall, 539 eligible women, including 127 with CIN2+, were enrolled in a cross-sectional study, and tested for hrHPV infection. Food intake was estimated using a food frequency questionnaire. Logistic regression models were applied. Using the Mediterranean Diet Score, we demonstrated that, among 252 women with a normal cervical epithelium, medium adherence to the Mediterranean diet decreased the odds of hrHPV infection when compared to low adherence (adjOR = 0.40, 95%CI = 0.22-0.73). Using the principal component analysis, we also identified two dietary patterns which explained 14.31% of the variance in food groups intake. Women in the third and fourth quartiles of the "Western pattern" had higher odds of hrHPV infection when compared with first quartile (adjOR = 1.77, 95% CI = 1.04-3.54 and adjOR = 1.97, 95%CI = 1.14-4.18, respectively). Adjusting for hrHPV status and age, women in the third quartile of the "prudent pattern" had lower odds of CIN2+ when compared with those in the first quartile (OR = 0.50, 95%CI = 0.26-0.98). Our study is the first to demonstrate the association of dietary patterns with hrHPV infection and CC and discourages unhealthy habits in favour of a Mediterranean-like diet.
[Barchitta, Martina; Maugeri, Andrea; Quattrocchi, Annalisa; Agrifoglio, Ottavia; Agodi, Antonella] Univ Catania, Dept Med & Surg Sci & Adv Technol GF Ingrassia, Via S Sofia 87, I-95123 Catania, Italy; [Scalisi, Aurora] Azienda Sanit Prov Catania, Unita Operat Screening Ginecol, I-95126 Catania, Italy
University of Catania Agodi, A (corresponding author), Univ Catania, Dept Med & Surg Sci & Adv Technol GF Ingrassia, Via S Sofia 87, I-95123 Catania, Italy. martina.barchitta@unict.it; andreamaugeri88@gmail.com; annalisaquattrocchi@hotmail.com; ottavia.agrifoglio@gmail.com; aurora.scalisi@aspct.it;

agodia@unict.it Agodi, Antonella/B-3501-2011; Quattrocchi, Annalisa/AAU-5458-2020; Maugeri, Andrea/K-1018-2017; Barchitta, Martina/A-1362-2015 Maugeri, Andrea/0000-0003-2655-8574; Quattrocchi, Annalisa/0000-0002-5764-6721; Agrifoglio, Ottavia/0000-0002-3496-3097; Agodi, Antonella/0000-0002-4405-8162; Barchitta, Martina/0000-0002-0905-5003 Department of Medical and Surgical Sciences and Advanced Technologies "GF Ingrassia", University of Catania, ItalyDepartment of Medical and Surgical Sciences and Advanced Technologies "GF Ingrassia", University of Catania, Italy This paper has been prepared as part of the project "Non-communicable diseases risk: an integrated approach of nutritional and molecular epidemiology", led by A. A., funded by Department of Medical and Surgical Sciences and Advanced Technologies "GF Ingrassia", University of Catania, Italy. We are grateful to Bench Srl, University of Catania, Italy for assistance in data analysis. 39 75 79 0 7 MDPI BASEL ST ALBAN-ANLAGE 66, CH-4052 BASEL, SWITZERLAND 2072-6643 NUTRIENTS Nutrients APR 2018 10 4 469 10.3390/nu10040469 http://dx.doi.org/10.3390/nu10040469 12 Nutrition & Dietetics Science Citation Index Expanded (SCI-EXPANDED) Nutrition & Dietetics GJ3HN 29641467 Green Submitted, Green Published, gold 2025-06-24 WOS:000435182900085

J Kyro, C; Olsen, A; Landberg, R; Skeie, G; Loft, S; Åman, P; Leenders, M; Dik, VK; Siersema, PD; Pischon, T; Christensen, J; Overvad, K; Boutron-Ruault, MC; Fagherazzi, G; Cottet, V; Kühn, T; Chang-Claude, J; Boeing, H; Trichopoulou, A; Bamia, C; Trichopoulos, D; Palli, D; Krogh, V; Tumino, R; Vineis, P; Panico, S; Peeters, PH; Weiderpass, E; Bakken, T; Åsli, LA; Argüelles, M; Jakszyn, P; Sánchez, MJ; Amiano, P; Huerta, JM; Barricarte, A; Ljuslinder, I; Palmqvist, R; Khaw, KT; Wareham, N; Key, TJ; Travis, RC; Ferrari, P; Freisling, H; Jenab, M; Gunter, MJ; Murphy, N; Riboli, E; Tjonneland, A; Bueno-de-Mesquita, HB Kyro, Cecilie; Olsen, Anja; Landberg, Rikard; Skeie, Guri; Loft, Steffen; Aman, Per; Leenders, Max; Dik, Vincent K.; Siersema, Peter D.; Pischon, Tobias; Christensen, Jane; Overvad, Kim; Boutron-Ruault, Marie-Christine; Fagherazzi, Guy; Cottet, Vanessa; Kuehn, Tilman; Chang-Claude, Jenny; Boeing, Heiner; Trichopoulou, Antonia; Bamia, Christina; Trichopoulos, Dimitrios; Palli, Domenico; Krogh, Vittorio; Tumino, Rosario; Vineis, Paolo; Panico, Salvatore; Peeters, Petra H.; Weiderpass, Elisabete; Bakken, Toril; Asli, Lene Angell; Argueelles, Marcial; Jakszyn, Paula; Sanchez, Maria-Jose; Amiano, Pilar; Huerta, Jose Maria; Barricarte, Aurelio; Ljuslinder, Ingrid; Palmqvist, Richard; Khaw, Kay-Tee; Wareham, Nick; Key, Timothy J.; Travis, Ruth C.; Ferrari, Pietro; Freisling, Heinz; Jenab, Mazda; Gunter, Marc J.; Murphy, Neil; Riboli, Eilo; Tjonneland, Anne; Bueno-de-Mesquita, H. B(as) Plasma Alkylresorcinols, Biomarkers of Whole-Grain Wheat and Rye Intake, and Incidence of Colorectal Cancer JNCI-JOURNAL OF THE NATIONAL CANCER INSTITUTE English Article DIETARY FIBER; COLON-CANCER; RISK; REPRODUCIBILITY; COHORT; INTERVENTION; CONSUMPTION; VEGETABLES; PRODUCTS; HEALTH Background Few studies have investigated the association between whole-grain intake and colorectal cancer. Because whole-grain intake estimation might be prone to measurement errors, more objective measures (eg, biomarkers) could

assist in investigating such associations. Methods The association between alkylresorcinols, biomarkers of whole-grain rye and wheat intake, and colorectal cancer incidence were investigated using prediagnostic plasma samples from colorectal cancer case patients and matched control subjects nested within the European Prospective Investigation into Cancer and Nutrition. We included 1372 incident colorectal cancer case patients and 1372 individual matched control subjects and calculated the incidence rate ratios (IRRs) for overall and anatomical subsites of colorectal cancer using conditional logistic regression adjusted for potential confounders. Regional differences (Scandinavia, the Mediterranean, Central Europe) were also explored. Results High plasma total alkylresorcinol concentration was associated with lower incidence of distal colon cancer; the adjusted incidence rate ratio of distal colon cancer for the highest vs lowest quartile of plasma total alkylresorcinols was 0.48 (95% confidence interval [CI] = 0.28 to 0.83). An inverse association between plasma total alkylresorcinol concentrations and colon cancer was found for Scandinavian participants (IRR per doubling = 0.83; 95% CI = 0.70 to 0.98). However, plasma total alkylresorcinol concentrations were not associated with overall colorectal cancer, proximal colon cancer, or rectal cancer. Plasma alkylresorcinols concentrations were associated with colon and distal colon cancer only in Central Europe and Scandinavia (ie, areas where alkylresorcinol levels were higher). Conclusions High concentrations of plasma alkylresorcinols were associated with a lower incidence of distal colon cancer but not with overall colorectal cancer, proximal colon cancer, and rectal cancer. [Kyro, Cecilie; Olsen, Anja; Christensen, Jane; Trichopoulou, Antonia] Danish Canc Soc Res Ctr, Strandblvd 49, DK-2100 Copenhagen O, Denmark; [Landberg, Rikard; Aman, Per] Swedish Univ Agr Sci, Dept Food Sci, BioCtr, Uppsala, Sweden; [Skeie, Guri; Weiderpass, Elisabete; Bakken, Toril; Asli, Lene Angell] Univ Tromso, Dept Community Med, Tromso, Norway; [Loft, Steffen] Univ Copenhagen, Dept Publ Hlth, Environm Hlth Sect, Fac Hlth Sci, Copenhagen, Denmark; [Leenders, Max; Dik, Vincent K.; Siersema, Peter D.] Univ Med Ctr Utrecht, Dept Gastroenterol & Hepatol, Utrecht, Netherlands; [Peeters, Petra H.] Univ Med Ctr Utrecht, Dept Epidemiol, Julius Ctr Hlth Sci & Primary Care, Utrecht, Netherlands; [Pischon, Tobias] Max Delbruck Ctr Mol Med, Mol Epidemiol Grp, Berlin, Germany; [Overvad, Kim] Aarhus Univ, Epidemiol Sect, Dept Publ Hlth, Aarhus, Denmark; [Boutron-Ruault, Marie-Christine; Fagherazzi, Guy; Cottet, Vanessa] INSERM, Ctr Res Epidemiol & Populat Hlth CESP, U1018, Nutr Hormones & Womens Hlth team, F-94805 Villejuif, France; [Boutron-Ruault, Marie-Christine; Fagherazzi, Guy; Cottet, Vanessa] Univ Paris Sud, UMRS 1018, F-94805 Villejuif, France; [Boutron-Ruault, Marie-Christine; Fagherazzi, Guy; Cottet, Vanessa] IGR, F-94805 Villejuif, France; [Kuehn, Tilman; Chang-Claude, Jenny] German Canc Res Ctr, Div Canc Epidemiol, Heidelberg, Germany; [Boeing, Heiner] German Inst Human Nutr Potsdam Rehbrucke, Dept Epidemiol, Nuthetal, Germany; [Bamia, Christina; Trichopoulos, Dimitrios] Hellen Hlth Fdn, Athens, Greece; [Bamia, Christina] Univ Athens, Sch Med, WHO Collaborating Ctr Food & Nutr Policies, Dept Hyg Epidemiol & Med Stat, Athens, Greece; [Trichopoulos, Dimitrios] Harvard Univ, Sch Publ Hlth, Dept Epidemiol, Boston, MA 02115 USA; [Trichopoulos, Dimitrios] Acad Athens, Bur Epidemiol Res, Athens, Greece; [Palli, Domenico] Canc Res & Prevent Inst ISPO, Mol & Nutr Epidemiol Unit, Florence,

Italy; [Krogh, Vittorio] Fdn IRCCS Ist Nazl Tumori, Epidemiol & Prevent Unit, Milan, Italy; [Tumino, Rosario] ASP Ragusa, Civile MP Arezzo Hosp, Canc Registry & Histopathol Unit, Ragusa, Italy; [Vineis, Paolo] Univ London Imperial Coll Sci Technol & Med, MRC HPA Ctr Environm & Hlth, Sch Publ Hlth, London, England; [Gunter, Marc J.; Murphy, Neil; Riboli, Eilo] Imperial Coll London, Dept Epidemiol & Biostat, Sch Publ Hlth, London, England; [Panico, Salvatore] Univ Naples Federico II, Dipartimento Med Clin & Chirurg, Naples, Italy; [Weiderpass, Elisabete] Canc Registry Norway, Dept Res, Oslo, Norway; [Weiderpass, Elisabete] Karolinska Inst, Dept Med Epidemiol & Biostat, Stockholm, Sweden; [Weiderpass, Elisabete] Samfundet Folkhalsan, Helsinki, Finland; [Argueelles, Marcial] Publ Hlth Directorate, Asturias, Spain; [Jakszyn, Paula] Catalan Inst Oncol, Canc Epidemiol Res Programme, Unit Nutr Environm & Canc, Barcelona, Spain; [Sanchez, Maria-Jose] Andalusian Sch Publ Hlth, Granada, Spain; [Sanchez, Maria-Jose; Huerta, Jose Maria; Barricarte, Aurelio] CIBER Epidemiol & Salud Publ, Madrid, Spain; [Amiano, Pilar] Basque Reg Hlth Dept, Publ Hlth Div Guipuzkoa, San Sebastian, Spain; [Huerta, Jose Maria] Murcia Reg Hlth Council, Dept Epidemiol, Murcia, Spain; [Barricarte, Aurelio] Navarre Publ Hlth Inst, Pamplona, Spain; [Ljuslinder, Ingrid] Umea Univ, Dept Radiat Sci, Umea, Sweden; [Palmqvist, Richard] Umea Univ, Dept Med Biosci, Umea, Sweden; [Khaw, Kay-Tee; Wareham, Nick] Univ Cambridge, Dept Publ Hlth & Primary Care, Cambridge, England; [Wareham, Nick] Univ Cambridge, Med Res Council Epidemiol Unit, Cambridge, England; [Travis, Ruth C.] Univ Oxford, Canc Epidemiol Unit, Oxford, England; [Ferrari, Pietro; Freisling, Heinz; Jenab, Mazda] Int Agcy Res Canc, Lyon, France; [Bueno-de-Mesquita, H. B(as)] Natl Inst Publ Hlth & Environm, Bilthoven, Netherlands Danish Cancer Society; Swedish University of Agricultural Sciences; UiT The Arctic University of Tromso; University of Copenhagen; Utrecht University; Utrecht University Medical Center; Utrecht University; Utrecht University Medical Center; Helmholtz Association; Max Delbruck Center for Molecular Medicine; Aarhus University; Institut National de la Sante et de la Recherche Medicale (Inserm); Universite Paris Saclay; Institut National de la Sante et de la Recherche Medicale (Inserm); Universite Paris Saclay; UNICANCER; Gustave Roussy; Helmholtz Association; German Cancer Research Center (DKFZ); Leibniz Association; Deutsches Institut fur Ernahrungsforschung Potsdam-Rehbrucke (DIfE); World Health Organization; Athens Medical School; National & Kapodistrian University of Athens; Harvard University; Harvard T.H. Chan School of Public Health; Academy of Athens; ISPRO Istituto per lo studio, la prevenzione e la rete oncologica; Fondazione IRCCS Istituto Nazionale Tumori Milan; Civile M.P. Arezzo Hospital; Imperial College London; Imperial College London; University of Naples Federico II; University of Oslo; Karolinska Institutet; Samfundet Folkhalsan; Institut Catala d'Oncologia; Escuela Andaluza de Salud Publica; CIBER - Centro de Investigacion Biomedica en Red; CIBERESP; Murcia Regional Health Council; Public Health Institute of Navarra; Umea University; Umea University; University of Cambridge; University of Cambridge; UK Research & Innovation (UKRI); Medical Research Council UK (MRC); University of Oxford; World Health Organization; International Agency for Research on Cancer (IARC); Netherlands National Institute for Public Health & the Environment Kyro, C (corresponding author), Danish Canc Soc Res Ctr, Strandblvd 49, DK-2100 Copenhagen O, Denmark.   ceciliek@cancer.dk   Jenab,

Mehdi/L-2515-2019; Khaw, Kay-Tee/AAZ-3209-2021; Riboli, Elio/A-4357-2009; Tjonneland, Anne/AGU-0320-2022; Pischon, Tobias/HGE-8577-2022; Cottet, Vanessa/ABE-3236-2020; TRICHOPOULOU, ANTONIA/ABF-8727-2021; Panico, Salvatore/K-6506-2016; Gunter, Marc/AAP-8621-2020; Boutron-Ruault, Marie-Christine/H-3936-2014; Fagherazzi, Guy/ABB-2555-2020; Kühn, Tilman/JYP-5102-2024; Kyro, Cecilie/ACG-9913-2022; Sánchez, María/HOC-7747-2023; Trichopoulos, Dimitrios/G-6825-2012; Krogh, Vittorio/AAA-9171-2019; Siersema, Peter/V-1636-2019; Krogh, Vittorio/K-2628-2016; Weiderpass, Elisabete/M-4029-2016; Fagherazzi, Guy/P-3534-2017; SANCHEZ-PEREZ, MARIA JOSE/D-1087-2011; Jakszyn, Paula/K-1458-2016; Huerta, Jose Maria/N-8654-2015; Murphy, Neil/E-1189-2017 Loft, Steffen/0000-0001-9552-8518; Krogh, Vittorio/0000-0003-0122-8624; tumino, rosario/0000-0003-2666-414X; Jenab, Mazda/0000-0002-0573-1852; Gunter, Marc/0000-0001-5472-6761; Olsen, Anja/0000-0003-4788-503X; PALLI, Domenico/0000-0002-5558-2437; Riboli, Elio/0000-0001-6795-6080; Pischon, Tobias/0000-0003-1568-767X; Overvad, Kim/0000-0001-6429-7921; Weiderpass, Elisabete/0000-0003-2237-0128; Skeie, Guri/0000-0003-2476-4251; Kyro, Cecilie/0000-0002-9083-8960; Panico, Salvatore/0000-0002-5498-8312; Fagherazzi, Guy/0000-0001-5033-5966; SANCHEZ-PEREZ, MARIA JOSE/0000-0003-4817-0757; Jakszyn, Paula/0000-0003-0672-8847; Kuhn, Tilman/0000-0001-7702-317X; Freisling, Heinz/0000-0001-8648-4998; Huerta, Jose Maria/0000-0002-9637-3869; Murphy, Neil/0000-0003-3347-8249; Tjonneland, Anne/0000-0003-4385-2097
Wereld Kanker Onderzoek Fonds (WCRF NL) [2011/436]; WCRF International grant program; NordForsk (Centre of Excellence programme HELGA) [070015]; European Commission (DG-SANCO); International Agency for Research on Cancer; Danish Cancer Society (Denmark); Ligue Contre le Cancer, Institut Gustave Roussy (France); Mutuelle Generale de l'Education Nationale (France); Institut National de la Sante et de la Recherche Medicale (France); German Cancer Aid, German Cancer Research Center (Greece); Federal Ministry of Education and Research (Germany); Hellenic Health Foundation (Greece); Associazione Italiana per la Ricerca sul Cancro-AIRC-Milan, and National Research Council (Italy); Dutch Ministry of Public Health, Welfare and Sports, Netherlands Cancer Registry (The Netherlands); LK Research Funds, Dutch Prevention Funds (The Netherlands); Dutch Zorg Onderzoek Nederland; World Cancer Research Fund, Statistics Netherlands (The Netherlands) [ERC-2009-AdG 232997]; Norwegian Research Council (Norway); Extrastiftelsen Helse og Rehabiliering med Extra-midler (Norway); Health Research Fund, Regional Governments of Andalucia, Asturias, Basque Country, Murcia [6236]; Navarra, ISCIII RETIC [RD06/0020]; Swedish Cancer Society, Swedish Scientific Council; Regional Government of Skane and Vasterbotten (Sweden); Cancer Research UK; Medical Research Council (United Kingdom)   Wereld Kanker Onderzoek Fonds (WCRF NL); WCRF International grant program; NordForsk (Centre of Excellence programme HELGA)(NordForsk); European Commission (DG-SANCO)(European Union (EU)European Commission Joint Research Centre); International Agency for Research on Cancer; Danish Cancer Society (Denmark)(Danish Cancer Society); Ligue Contre le Cancer, Institut Gustave Roussy (France); Mutuelle Generale de l'Education Nationale (France); Institut National de la Sante et de la Recherche Medicale (France)(Institut National de la Sante et de la Recherche Medicale (Inserm)); German Cancer Aid, German Cancer Research Center (Greece); Federal Ministry of Education and Research (Germany)(Federal Ministry of Education & Research

(BMBF)); Hellenic Health Foundation (Greece); Associazione Italiana per la Ricerca sul Cancro-AIRC-Milan, and National Research Council (Italy)(Fondazione AIRC per la ricerca sul cancro); Dutch Ministry of Public Health, Welfare and Sports, Netherlands Cancer Registry (The Netherlands); LK Research Funds, Dutch Prevention Funds (The Netherlands); Dutch Zorg Onderzoek Nederland(Netherlands Organization for Scientific Research (NWO)); World Cancer Research Fund, Statistics Netherlands (The Netherlands); Norwegian Research Council (Norway)(Research Council of Norway); Extrastiftelsen Helse og Rehabiliering med Extra-midler (Norway); Health Research Fund, Regional Governments of Andalucia, Asturias, Basque Country, Murcia; Navarra, ISCIII RETIC; Swedish Cancer Society, Swedish Scientific Council; Regional Government of Skane and Vasterbotten (Sweden); Cancer Research UK(Cancer Research UK); Medical Research Council (United Kingdom)(UK Research & Innovation (UKRI)Medical Research Council UK (MRC)) This work was funded by Wereld Kanker Onderzoek Fonds (WCRF NL; grant 2011/436), as part of the WCRF International grant program, and by NordForsk (Centre of Excellence programme HELGA; 070015).r The coordination of EPIC is financially supported by the European Commission (DG-SANCO) and the International Agency for Research on Cancer. The national cohorts are supported by Danish Cancer Society (Denmark); Ligue Contre le Cancer, Institut Gustave Roussy, Mutuelle Generale de l'Education Nationale, Institut National de la Sante et de la Recherche Medicale (France); German Cancer Aid, German Cancer Research Center, Federal Ministry of Education and Research (Germany); the Hellenic Health Foundation (Greece); Associazione Italiana per la Ricerca sul Cancro-AIRC-Milan, and National Research Council (Italy); Dutch Ministry of Public Health, Welfare and Sports, Netherlands Cancer Registry, LK Research Funds, Dutch Prevention Funds, Dutch Zorg Onderzoek Nederland, World Cancer Research Fund, Statistics Netherlands (The Netherlands); ERC-2009-AdG 232997, the Norwegian Research Council, Extrastiftelsen Helse og Rehabiliering med Extra-midler (Norway); Health Research Fund, Regional Governments of Andalucia, Asturias, Basque Country, Murcia (no. 6236) and Navarra, ISCIII RETIC (RD06/0020) (Spain); Swedish Cancer Society, Swedish Scientific Council and Regional Government of Skane and Vasterbotten (Sweden); and Cancer Research UK, Medical Research Council (United Kingdom).	43	60	65	1	34	OXFORD UNIV PRESS INC	CARY	JOURNALS DEPT, 2001 EVANS RD, CARY, NC 27513 USA	0027-8874	1460-2105		JNCI-J NATL CANCER I	JNCI-J. Natl. Cancer Inst.	JAN	2014	106	1			djt352	10.1093/jnci/djt352	http://dx.doi.org/10.1093/jnci/djt352		9	Oncology	Science Citation Index Expanded (SCI-EXPANDED)	Oncology	AB6IV	24317181	Green Published, Green Accepted, hybrid, Green Submitted			2025-06-24	WOS:000331891900008
J	Busza, J; Shewamene, Z; Zimmerman, C; Erulkar, A; Hailu, E; Negeri, L; Anderson, E; Lo, YK			Busza, Joanna; Shewamene, Zewdneh; Zimmerman, Cathy; Erulkar, Annabel; Hailu, Eyasu; Negeri, Lemi; Anderson, Elizabeth; Lo, Yuki	Accidental traffickers: qualitative findings on labour recruitment in Ethiopia	GLOBALIZATION AND HEALTH	English	Article			Irregular migration; Trafficking; Ethiopia; Labour recruitment; Domestic workers; Middle east; Qualitative research	MIGRATION BROKERS;

DOMESTIC WORKERS; MODERN SLAVERY; SEX WORKERS; MIGRANTS; HEALTH; ECONOMY; RISK BackgroundThe growth of labour migration and associated risks of human trafficking and exploitation remain significant global human rights and health challenges. There is increasing policy interest in addressing structural determinants of adverse migration outcomes such as migrants' use of informal employment recruiters. In Ethiopia, "safe migration" policies have introduced regulations for registered private employment agencies and penalties for anyone else placing migrants into work overseas. Yet migrants continue to use informal facilitators who are often demonised as traffickers without evidence of their motivations, experiences or perceptions. We conducted qualitative interviews with 28 informal facilitators as part of a study into how recruitment practices shape risks for female migrants seeking domestic work in the Middle East and Gulf States. We present the realities of irregular recruitment on the ground, and how these practices are affected by policies that dichotomise recruiters into legal/safe and illegal/unsafe categories.ResultsWe identified four main themes. First, arranging migration from rural areas differs from in the capital, Addis Ababa, where laws and regulations originate. Outside Addis Ababa, registration was difficult for facilitators to arrange, with little incentive to do so due to its lack of importance to prospective migrants. Second, the ability to circumvent legal requirements was considered an advantage of informal facilitators because it reduced costs and expedited migrants' departure. Third, facilitators did not work alone but operated in long "chains" of diverse actors. This meant migrants' safety was not determined by any given individual, but spread across numerous people involved in sending a migrant abroad, some of whom might be registered and others not. And finally, facilitators did not believe they could realistically safeguard migrants once they were outside of Ethiopia and working under different laws and employers.ConclusionsFindings from this study add to a growing body of work demonstrating the diversity of people involved in the migration process, and consequent oversimplification of popular policy solutions. A more effective approach might be to constructively engage informal facilitators and identify ways they could assist with referring migrant workers to registered agencies and safe employment, rather than criminalising their participation. [Busza, Joanna; Shewamene, Zewdneh] London Sch Hyg & Trop Med, Fac Publ Hlth & Policy, Dept Publ Hlth Environm & Soc, 15-17 Tavistock Pl, London WC1H 9SH, England; [Zimmerman, Cathy] London Sch Hyg & Trop Med, Fac Publ Hlth & Policy, Dept Global Hlth & Dev, 15-17 Tavistock Pl, London WC1H 9SH, England; [Erulkar, Annabel; Hailu, Eyasu; Negeri, Lemi] Populat Council, Heritage Plaza,Bole Medhaneialem Rd, Addis Ababa 18609, Ethiopia; [Anderson, Elizabeth; Lo, Yuki] Freedom Fund, Lighterman House,30 Wharfdale Rd, London N1 9RY, England

University of London; London School of Hygiene & Tropical Medicine; University of London; London School of Hygiene & Tropical Medicine Busza, J (corresponding author), London Sch Hyg & Trop Med, Fac Publ Hlth & Policy, Dept Publ Hlth Environm & Soc, 15-17 Tavistock Pl, London WC1H 9SH, England.

Joanna.Busza@lshtm.ac.uk zimmerman, cathy/0000-0002-7815-4320 Office to Monitor and Combat Trafficking in Persons Office to Monitor and Combat Trafficking in Persons

We are grateful to all study participants who provided their time and voices to this research. We are also thankful to

Wondwosen Tesema, Gobeze Negash, Eyerusalem Girma, Helen Beshaw, Adanech Dutu, Tigist Solomon and Welela Tarekegn for their help with conducting interviews, transcription, and translation. We also thank Population Council staff based in Bahir Dar for their assistance in recruiting informal migration facilitators.
62 3 3 2 5 BMC LONDON CAMPUS, 4 CRINAN ST, LONDON N1 9XW, ENGLAND 1744-8603
GLOBALIZATION HEALTH Global. Health DEC 14 2023 19
1 102 10.1186/s12992-023-01005-9 http://dx.doi.org/10.1186/s12992-023-01005-9
13 Public, Environmental & Occupational Health Science Citation Index Expanded (SCI-EXPANDED); Social Science Citation Index (SSCI) Public, Environmental & Occupational Health
CO4F2 38098068 Green Published, Green Accepted, gold
2025-06-24 WOS:001126168500001
J Bulotta, S; Corradino, R; Celano, M; D'Agostino, M; Maiuolo, J; Oliverio, M; Procopio, A; Iannone, M; Rotiroti, D; Russo, D
Bulotta, Stefania; Corradino, Rosanna; Celano, Marilena; D'Agostino, Maria; Maiuolo, Jessica; Oliverio, Manuela; Procopio, Antonio; Iannone, Michelangelo; Rotiroti, Domenicantonio; Russo, Diego Antiproliferative and antioxidant effects on breast cancer cells of oleuropein and its semisynthetic peracetylated derivatives FOOD CHEMISTRY
English Article Olive leaves; Oleuropein; Green chemistry; Breast cancer OLIVE OIL; PHENOLIC-COMPOUNDS; MEDITERRANEAN DIET; HYDROXYTYROSOL; RISK; HL60; PROLIFERATION; INHIBITION; EXTRACTS Olive leaves extracts are a natural source of polyphenols, mainly oleuropein, widely considered to be potentially beneficial for health. This study focused on evaluation of the anti-tumoural activities of some oleuropein peracetylated derivatives, obtained with "green chemical" methodologies, against two human breast cancer cell lines. MCF-7 and T-47D cells were treated with oleuropein, peracetylated oleuropein, peracetylated aglycone and peracetylated hydroxytyrosol and the effects on growth and viability were investigated. Antioxidant effects were analysed after treatment with hydrogen peroxide. The peracetylated compounds exerted higher antiproliferative effects than oleuropein, by an arrest of cell cycle progression, associated with a strong antioxidant activity. Our results demonstrate that olive leaves, a by-product of olive manufacture, may provide a precious source of chemical derivatives, obtainable by peracetylation of oleuropein derivatives, which provide beneficial properties for human health.
(C) 2011 Elsevier Ltd. All rights reserved. [Bulotta, Stefania; Corradino, Rosanna; Celano, Marilena; D'Agostino, Maria; Maiuolo, Jessica; Oliverio, Manuela; Procopio, Antonio; Rotiroti, Domenicantonio; Russo, Diego] Magna Graecia Univ Catanzaro, Dept Pharmacobiol Sci, I-88100 Catanzaro, Italy; [Iannone, Michelangelo] ARPACal Environm Epidemiol Ctr, I-88021 Catanzaro, Italy; [Iannone, Michelangelo; Rotiroti, Domenicantonio] CNR ISN, I-88021 Catanzaro, Italy Magna Graecia University of Catanzaro; Consiglio Nazionale delle Ricerche (CNR); Istituto di Scienze Neurologiche (ISN-CNR) Russo, D (corresponding author), Magna Graecia Univ Catanzaro, Dept Pharmacobiol Sci, Viale Europa, I-88100 Catanzaro, Italy. d.russo@unicz.it Maiuolo, Jessica/AAU-2482-2020; Iannone, Michelangelo/L-4675-2013 Iannone, Michelangelo/0000-0002-0426-5191 Fondazione Umberto Di Mario; CNR - ISN, Roccelletta di Borgia, Catanzaro (Italy) [88021]

Fondazione Umberto Di Mario(Fondazione AIRC per la ricerca sul cancro); CNR - ISN, Roccelletta di Borgia, Catanzaro (Italy) This work was supported by Fondazione Umberto Di Mario (to D.R.) and CNR - ISN, 88021 Roccelletta di Borgia, Catanzaro (Italy). 35 88 97 0 28 ELSEVIER SCI LTD OXFORD THE BOULEVARD, LANGFORD LANE, KIDLINGTON, OXFORD OX5 1GB, OXON, ENGLAND 0308-8146 1873-7072 FOOD CHEM Food Chem. AUG 15 2011 127 4 1609 1614 10.1016/j.foodchem.2011.02.025 http://dx.doi.org/10.1016/j.foodchem.2011.02.025 6 Chemistry, Applied; Food Science & Technology; Nutrition & Dietetics Science Citation Index Expanded (SCI-EXPANDED) Chemistry; Food Science & Technology; Nutrition & Dietetics 754CP 2025-06-24 WOS:000289830000027

J Dela Cruz, R; Park, SY; Shvetsov, YB; Boushey, CJ; Monroe, KR; Le Marchand, L; Maskarinec, G Dela Cruz, Rica; Park, Song-Yi; Shvetsov, Yurii B.; Boushey, Carol J.; Monroe, Kristine R.; Le Marchand, Loic; Maskarinec, Gertraud Diet Quality and Breast Cancer Incidence in the Multiethnic Cohort EUROPEAN JOURNAL OF CLINICAL NUTRITION English Article PATTERNS; INDEXES; OBESITY; RISK This study investigated the relation of diet quality indexes (DQI) with breast cancer incidence among women from the Multiethnic Cohort (MEC). Participants completed a questionnaire with a validated food frequency questionnaire. Scores for Healthy Eating Index 2015 (HEI-2015), Alternate Healthy Eating Index 2010 (AHEI-2010), alternate Mediterranean diet score (aMED), and Dietary Approaches to Stop Hypertension (DASH) were divided into quintiles (Q1-Q5). Cox regression was applied to estimate hazard ratios (HRs) and 95% confidence intervals (CIs) for DQIs and breast cancer risk adjusted for known risk factors. The respective HRs for Q5 vs. Q1 were: 1.06 (95% CI, 0.98-1.14) for HEI-2015, 0.96 (95% CI, 0.90-1.04) for AHEI-2010, 1.01 (95% CI, 0.94-1.09) for aMED, and 0.95 (95% CI, 0.88-1.02) for DASH (p(trend) > 0.05 for all). However, overweight and obesity were significantly associated with breast cancer incidence. Despite the null association for DQIs, diet quality may lower breast cancer risk through its positive influence on weight status. [Dela Cruz, Rica; Park, Song-Yi; Shvetsov, Yurii B.; Boushey, Carol J.; Le Marchand, Loic; Maskarinec, Gertraud] Univ Hawaii, Ctr Canc, Honolulu, HI 96822 USA; [Monroe, Kristine R.] Univ Southern Calif, Dept Prevent Med, Los Angeles, CA 90007 USA Cancer Research Center of Hawaii; University of Hawaii System; University of Southern California Dela Cruz, R (corresponding author), Univ Hawaii, Ctr Canc, Honolulu, HI 96822 USA. ricadc@hawaii.edu Shvetsov, Yurii/ABA-3196-2020 Park, Song-Yi/0000-0001-7734-5320; Dela Cruz, Rica/0000-0001-9477-5971 National Cancer Institute [U01 CA164973, R03 CA223890, U54 CA143727] National Cancer Institute(United States Department of Health & Human ServicesNational Institutes of Health (NIH) - USANIH National Cancer Institute (NCI)) This work was supported by the following grants from the National Cancer Institute: U01 CA164973, R03 CA223890, and U54 CA143727 (fellowship for RDC). 12 18 21 0 5 SPRINGERNATURE LONDON CAMPUS, 4 CRINAN ST, LONDON, N1 9XW, ENGLAND 0954-3007 1476-5640 EUR J CLIN NUTR Eur. J. Clin. Nutr. DEC 2020 74 12

1743 1747 10.1038/s41430-020-0627-2 http://dx.doi.org/10.1038/s41430-020-0627-2 APR 2020 5 Nutrition & Dietetics Science Citation Index Expanded (SCI-EXPANDED) Nutrition & Dietetics PA1FQ 32286532 Green Accepted 2025-06-24 WOS:000526234700002
J Natalucci, V; Marini, CF; De Santi, M; Annibalini, G; Lucertini, F; Vallorani, L; Panico, AR; Sisti, D; Saltarelli, R; Zeppa, SD; Agostini, D; Gervasi, M; Baldelli, G; Grassi, E; Nart, A; Rossato, M; Biancalana, V; Piccoli, G; Benelli, P; Villarini, A; Somaini, M; Catalano, V; Guarino, S; Pietrelli, A; Monaldi, S; Sarti, D; Barocci, S; Flori, M; Rocchi, MBL; Brandi, G; Stocchi, V; Emili, R; Barbieri, E Natalucci, Valentina; Marini, Carlo Ferri; De Santi, Mauro; Annibalini, Giosue; Lucertini, Francesco; Vallorani, Luciana; Panico, Andrea Rocco; Sisti, Davide; Saltarelli, Roberta; Zeppa, Sabrina Donati; Agostini, Deborah; Gervasi, Marco; Baldelli, Giulia; Grassi, Eugenio; Nart, Alessandra; Rossato, Massimo; Biancalana, Vincenzo; Piccoli, Giovanni; Benelli, Piero; Villarini, Anna; Somaini, Matteo; Catalano, Vincenzo; Guarino, Stefania; Pietrelli, Alice; Monaldi, Silvia; Sarti, Donatella; Barocci, Simone; Flori, Marco; Rocchi, Marco Bruno Luigi; Brandi, Giorgio; Stocchi, Vilberto; Emili, Rita; Barbieri, Elena Movement and health beyond care, MoviS: study protocol for a randomized clinical trial on nutrition and exercise educational programs for breast cancer survivors TRIALS English Article
Breast cancer; Physical activity; Exercise; Mediterranean diet; Quality of life; Health-related parameters; Prevention QUALITY-OF-LIFE; LONG-TERM; GUIDELINES Background Breast cancer (BC) is the most common invasive cancer in women, and exercise can significantly improve the outcomes of BC survivors. MoviS (Movement and Health Beyond Care) is a randomized controlled trial aimed to evaluate the potential health benefits of exercise and proper nutritional habits. This study aims to assess the efficacy of aerobic exercise training in improving quality of life (QoL) and health-related factors in high-risk BC. Methods One hundred seventy-two BC survivor women, aged 30-70 years, non-metastatic, stage 0-III, non-physically active, 6-12 months post-surgery, and post chemo- or radiotherapy, will be recruited in this study. Women will be randomly allocated to the intervention arm (lifestyle recommendations and MoviS Training) or control arm (lifestyle recommendations). The MoviS training consists of 12 weeks of aerobic exercise training (2 days/week of supervised and 1 day/week of unsupervised exercise) with a progressive increase in exercise intensity (40-70% of heart rate reserve) and duration (20-60 min). Both arms will receive counseling on healthy lifestyle habits (nutrition and exercise) based on the World Cancer Research Fund International (WCRF) 2018 guidelines. The primary outcome is the improvement of the QoL. The secondary outcomes are improvement of health-related parameters such as Mediterranean diet adherence, physical activity level, flexibility, muscular fitness, fatigue, cardiorespiratory fitness (estimated maximal oxygen uptake), echocardiographic parameters, heart rate variability (average of the standard deviations of all 5 min normal to normal intervals (ASDNN/5 min) and 24 h very low and low frequency), and metabolic, endocrine, and inflammatory serum biomarkers (glycemia, insulin resistance, progesterone, testosterone, and high-sensitivity C-reactive protein). Discussion his trial aims to evaluate if supervised exercise may improve QoL

and health-related factors of BC survivors with a high risk of recurrence. Findings from this project could provide knowledge improvement in the field of exercise oncology through the participation of a multidisciplinary team that will provide a coordinated program of cancer care to improve healthcare quality, improve prognosis, increase survival times and QoL, and reduce the risk of BC recurrence. [Natalucci, Valentina; Marini, Carlo Ferri; De Santi, Mauro; Annibalini, Giosue; Lucertini, Francesco; Vallorani, Luciana; Panico, Andrea Rocco; Sisti, Davide; Saltarelli, Roberta; Zeppa, Sabrina Donati; Agostini, Deborah; Gervasi, Marco; Baldelli, Giulia; Grassi, Eugenio; Nart, Alessandra; Rossato, Massimo; Biancalana, Vincenzo; Piccoli, Giovanni; Benelli, Piero; Rocchi, Marco Bruno Luigi; Brandi, Giorgio; Barbieri, Elena] Univ Urbino Carlo Bo, Dept Biomol Sci, I-61029 Urbino, Italy; [Villarini, Anna] Univ Perugia, Dept Med & Surg, I-06129 Perugia, Italy; [Somaini, Matteo] Univ Milan, Sch Specializat Nutr Sci, Milan, Italy; [Catalano, Vincenzo; Guarino, Stefania; Pietrelli, Alice; Monaldi, Silvia; Sarti, Donatella; Emili, Rita] Hosp St Maria Misericordia Urbino, Med Oncol, I-61029 Urbino, Italy; [Barocci, Simone] Hosp St Maria Misericordia Urbino, Clin Pathol, I-61029 Urbino, Italy; [Flori, Marco] Hosp St Maria Misericordia Urbino, Cardiol, I-61029 Urbino, Italy; [Stocchi, Vilberto] Univ San Raffaele, Dept Human Sci Promot Qual Life, I-20132 Rome, Italy University of Urbino; University of Perugia; University of Milan; Universita Telematica San Raffaele

De Santi, M; Annibalini, G (corresponding author), Univ Urbino Carlo Bo, Dept Biomol Sci, I-61029 Urbino, Italy.

mauro.desanti@uniurb.it; giosue.annibalini@uniurb.it

Rocchi, Marco/AAK-5926-2021; ROSSATO, MASSIMO/JAX-1836-2023; Baldelli, Giulia/KGK-9219-2024; Natalucci, Valentina/AAM-7882-2020; Donati Zeppa, Sabrina/AAO-9088-2020; SALTARELLI, Roberta/AAM-4291-2021; De Santi, Mauro/F-5401-2013; Annibalini, Giosue/J-7701-2016; Ferri Marini, Carlo/GRY-3765-2022; Barbieri, Elena/AGI-0430-2022; Lucertini, Francesco/F-6603-2012 Rossato, Massimo/0000-0001-6781-9588; De Santi, Mauro/0000-0003-2983-8344; Annibalini, Giosue/0000-0002-6914-4905; Ferri Marini, Carlo/0000-0002-6866-997X; SALTARELLI, Roberta/0000-0002-6375-127X; Barbieri, Elena/0000-0002-3480-7983; Stocchi, Vilberto/0000-0003-3269-9410; Lucertini, Francesco/0000-0003-3134-4511 Ateneum project: "Promozione della salute e della sicurezza alimentare" [D.R. 446/2020]; Banca di Credito Cooperativo del Metauro for the contribution "Stile di vita attivo e microbiota nel tumore al seno" Ateneum project: "Promozione della salute e della sicurezza alimentare"; Banca di Credito Cooperativo del Metauro for the contribution "Stile di vita attivo e microbiota nel tumore al seno" This project is partially funded by the Ateneum project: "Promozione della salute e della sicurezza alimentare" (D.R. 446/2020). In addition, funding was provided by the Banca di Credito Cooperativo del Metauro for the contribution "Stile di vita attivo e microbiota nel tumore al seno" (autorizzazione Consiglio del Dipartimento del 13/7/2021 delibera n. 157).

47 6 6 1 8 BMC LONDON CAMPUS, 4 CRINAN ST, LONDON N1 9XW, ENGLAND 1745-6215 TRIALS

Trials FEB 22 2023 24 1

134 10.1186/s13063-023-07153-y

http://dx.doi.org/10.1186/s13063-023-07153-y 15

Medicine, Research & Experimental Science Citation Index Expanded (SCI-EXPANDED) Research & Experimental Medicine

9F1DS 36814313 gold, Green Published 2025-06-24 WOS:000937216000002
J Fung, TT; Kashambwa, R; Sato, K; Chiuve, SE; Fuchs, CS; Wu, KN; Giovannucci, E; Ogino, S; Hu, FB; Meyerhardt, JA
Fung, Teresa T.; Kashambwa, Rutendo; Sato, Kaori; Chiuve, Stephanie E.; Fuchs, Charles S.; Wu, Kana; Giovannucci, Edward; Ogino, Shuji; Hu, Frank B.; Meyerhardt, Jeffrey A.
Post Diagnosis Diet Quality and Colorectal Cancer Survival in Women PLOS ONE English Article
SYSTEMIC INFLAMMATORY RESPONSE; CORONARY-HEART-DISEASE; C-REACTIVE PROTEIN; MEDITERRANEAN DIET; PROSPECTIVE COHORT; COLON-CANCER; ALL-CAUSE; RISK; MORTALITY; PATTERNS Background: Dietary factors are known to influence colorectal cancer (CRC) risk, however, their association with CRC survival is unclear. Therefore, we prospectively examined the association between diet quality scores, dietary patterns and colorectal cancer (CRC) survival. Methods: 1201 women diagnosed with stage I-III CRC between 1986 and 2008, were followed through 2010. Diet was assessed via a food frequency questionnaire administered at least 6 months after diagnosis. We computed the Alternate Healthy Eating Index-2010 (AHEI-2010), alternate Mediterranean Diet score (aMED) and Dietary Approaches to Stop Hypertension score (DASH) and derived two dietary patterns, Western (unhealthy) and prudent (healthy), by principal component analysis for each woman. Results: During follow-up, we documented 435 deaths, including 162 from CRC. After adjusting for potential confounders, only a higher AHEI-2010 score was significantly associated with lower overall mortality (HR comparing extreme quintiles=0.71, 95% CI 0.52-0.98, p trend=0.01) as well as borderline significantly with lower risk of CRC mortality by the trend test (HR Q5 vs Q1=0.72, 95% CI=0.43-1.21, p trend=0.07). When AHEI-2010 components were examined separately, inverse associations for overall mortality were primarily accounted for by moderate alcohol intake (HR comparing abstainers vs 5-15 g/d=1.30, 95% CI=1.05-1.61) and lower intake of sugar sweetened beverages and fruit juices combined (HR for each additional serving=1.11, 95% CI=1.01-1.23). No other diet quality score or dietary pattern was associated with overall or CRC-specific mortality. Conclusion: Higher AHEI-2010 score may be associated with lower overall mortality, moderate alcohol consumption and lower consumption of sugar sweetened beverages and juices combined appeared to account for most of the observed associations. [Fung, Teresa T.] Simmons Coll, Dept Nutr, Boston, MA 02115 USA; [Kashambwa, Rutendo] Brigham & Womens Hosp, Dept Neurol, Boston, MA 02115 USA; [Chiuve, Stephanie E.; Wu, Kana; Giovannucci, Edward] Harvard Univ, Sch Publ Hlth, Dept Nutr, Boston, MA 02115 USA; [Ogino, Shuji] Harvard Univ, Brigham & Womens Hosp, Sch Med, Channing Div Network Med,Dept Surg, Boston, MA 02115 USA; [Fuchs, Charles S.; Hu, Frank B.] Harvard Univ, Sch Med, Brigham & Womens Hosp, Dept Med, Boston, MA 02115 USA; [Fuchs, Charles S.; Ogino, Shuji; Meyerhardt, Jeffrey A.] Dana Farber Canc Inst, Dept Med Oncol, Boston, MA 02115 USA Simmons University; Harvard University; Harvard University Medical Affiliates; Brigham & Women's Hospital; Harvard University; Harvard T.H. Chan School of Public Health; Harvard University; Harvard Medical School; Harvard University Medical Affiliates; Brigham & Women's Hospital; Harvard University; Harvard University Medical Affiliates; Brigham & Women's Hospital; Harvard Medical

School; Harvard University; Harvard University Medical Affiliates; Dana-Farber Cancer InstituteFung, TT (corresponding author), Simmons Coll, Dept Nutr, Boston, MA 02115 USA.
fung@simmons.edu Meyerhardt, Jeffrey/IAP-4528-2023; Hu, Frank/C-1919-2013; Giovannucci, Edward/ADE-8028-2022 Ogino, Shuji/0000-0002-3909-2323; Chiuve, Stephanie/0000-0002-3524-8917
National Institute of Health [CA87969, CA127003, CA149222, CA95589, CA151993, CA169141, CA 118553, UM1CA167552, U54CA155626, UM1 CA167552] National Institute of Health(United States Department of Health & Human ServicesNational Institutes of Health (NIH) - USA) This work is funded by the National Institute of Health research grants CA87969, CA127003, CA149222, CA95589, CA151993, CA169141, CA 118553, UM1CA167552, U54CA155626, UM1 CA167552. The funders had no role in study design, data collection and analysis, decision to publish, or preparation of the manuscript. 39 79 86 0 15 PUBLIC LIBRARY SCIENCE SAN FRANCISCO 1160 BATTERY STREET, STE 100, SAN FRANCISCO, CA 94111 USA 1932-6203 PLOS ONE PLoS One DEC 15 2014 9 12
e115377 10.1371/journal.pone.0115377
http://dx.doi.org/10.1371/journal.pone.0115377
13 Multidisciplinary Sciences Science Citation Index Expanded (SCI-EXPANDED) Science & Technology - Other Topics
AW9WY 25506700 gold, Green Published 2025-06-24 WOS:000346607100072
J Barbieri, A; Quagliariello, V; Del Vecchio, V; Falco, M; Luciano, A; Amruthraj, NJ; Nasti, G; Ottaiano, A; Berretta, M; Iaffaioli, RV; Arra, C Barbieri, Antonio; Quagliariello, Vincenzo; Del Vecchio, Vitale; Falco, Michela; Luciano, Antonio; Amruthraj, Nagoth Joseph; Nasti, Guglielmo; Ottaiano, Alessandro; Berretta, Massimiliano; Iaffaioli, Rosario Vincenzo; Arra, Claudio Anticancer and Anti-Inflammatory Properties of Ganoderma lucidum Extract Effects on Melanoma and Triple-Negative Breast Cancer Treatment NUTRIENTS English Article
Ganoderma lucidum; melanoma; breast cancer; inflammation; cytokines; curcumin; cell viability IN-VITRO; CELLS; INTERLEUKIN-6; TRITERPENOIDS; POLYSACCHARIDES; METASTASIS; INHIBITION; MECHANISMS; SURVIVAL; VIVO Among the most important traditional medicinal fungi, Ganoderma lucidum has been used as a therapeutic agent for the treatment of numerous diseases, including cancer, in Oriental countries. The aim of this study is to investigate the anti-inflammatory, anticancer and anti-metastatic activities of Ganoderma lucidum extracts in melanoma and triple-negative breast cancer cells. Ganoderma lucidum extracts were prepared by using common organic solvents; MDA-MB 231 and B16-F10 cell lines were adopted as cellular models for triple-negative breast cancer and melanoma and characterized for cell viability, wound-healing assay and measurement of cytokines secreted by cancer cells under pro-inflammatory conditions (incubation with lipopolysaccharide, LPS) and pretreatment with Ganoderma lucidum extract at different concentrations. Our study demonstrates, for the first time, how Ganoderma lucidum extracts can significantly inhibit the release of IL-8, IL-6, MMP-2 and MMP-9 in cancer cells under pro-inflammatory condition. Interestingly, Ganoderma lucidum extracts significantly also decrease the viability of both cancer cells in a time-and concentration-dependent manner, with abilities to reduce cell

migration over time, which is correlated with a lower release of matrix metalloproteases. Taken together, these results indicate the possible use of Ganoderma lucidum extract for the therapeutic management of melanoma and human triple-negative breast cancer.

[Barbieri, Antonio; Del Vecchio, Vitale; Falco, Michela; Luciano, Antonio; Arra, Claudio] Natl Canc Inst G Pascale, Dept Res, Anim Facil Unit, Via M Semmola, I-80131 Naples, Italy; [Quagliariello, Vincenzo; Nasti, Guglielmo; Ottaiano, Alessandro; Iaffaioli, Rosario Vincenzo] Natl Canc Inst G Pascale, Dept Abdominal Oncol, Via M Semmola, I-80131 Naples, Italy; [Quagliariello, Vincenzo; Nasti, Guglielmo; Ottaiano, Alessandro; Berretta, Massimiliano; Iaffaioli, Rosario Vincenzo] ASMO Assoc Multidisciplinary Studies Oncol, Mediterranean Diet, Piazza Nicola Amore 6, I-80138 Naples, Italy; [Amruthraj, Nagoth Joseph] Univ Study Campania Luigi Vanvitelli, Dept Cardio Vasc Med, Clin Expt & Med Sci Chair Nephrol, I-81100 Caserta, Italy; [Berretta, Massimiliano] Natl Canc Inst, Dept Med Oncol, I-33081 Aviano, Italy IRCCS Fondazione Pascale; IRCCS Fondazione Pascale; Universita della Campania Vanvitelli Barbieri, A (corresponding author), Natl Canc Inst G Pascale, Dept Res, Anim Facil Unit, Via M Semmola, I-80131 Naples, Italy. a.barbieri@istitutotumori.na.it; quagliariello.enzo@gmail.com; vitale_84@hotmail.it; michelafalco_89@libero.it; a.luciano@istitutotumori.na.it; amruthjon@gmail.com; guglielmo.nasti@libero.it; ale.otto@libero.it; mberretta@cro.it; rv.iaffaioli@gmail.com; c.arra@istitutotumori.na.it Del+Vecchio, Vitale/ACN-3410-2022; Barbieri, Antonio/AAG-8961-2019; Quagliariello, Vincenzo/GLT-0763-2022; Berretta, Massimiliano/S-5853-2019; Quagliariello, Vincenzo/A-8537-2019 Lafranconi, Alessandra/0000-0002-4586-9610; Falco, Michela/0000-0003-4513-8639; Berretta, Massimiliano/0000-0002-9837-9148; Nagoth, Joseph Amruthraj/0000-0002-8230-2365; Luciano, Antonio/0000-0003-0183-6574; Barbieri, Antonio/0000-0002-7788-6167; Quagliariello, Vincenzo/0000-0002-4557-5401; DEL VECCHIO, VITALE/0000-0002-7323-4444; Ottaiano, Alessandro/0000-0002-2901-3855 25 94 97 1 50

MDPI AG BASEL ST ALBAN-ANLAGE 66, CH-4052 BASEL, SWITZERLAND 2072-6643 NUTRIENTS Nutrients MAR 2017
9 3 210
10.3390/nu9030210 http://dx.doi.org/10.3390/nu9030210
9 Nutrition & Dietetics Science Citation Index Expanded (SCI-EXPANDED) Nutrition & Dietetics EO9QQ
28264501 Green Published, gold, Green Submitted
2025-06-24 WOS:000397023600029

J Walkden, GJ; Anderson, EL; Vink, MP; Tilling, K; Howe, LD; Ben-Shlomo, Y Walkden, G. J.; Anderson, E. L.; Vink, M. P.; Tilling, K.; Howe, L. D.; Ben-Shlomo, Y.

Frailty in older-age European migrants: Cross-sectional and longitudinal analyses of the Survey of Health, Aging and Retirement in Europe (SHARE) SOCIAL SCIENCE & MEDICINE

English Article

Migration; Frailty; Trajectories; Healthcare policy; Acculturation COUNTRIES; DETERMINANTS; IMMIGRATION; MIGRATION; MORTALITY; POLICIES; PEOPLE Frailty correlates with morbidity and is superior to chronological age in predicting mortality. Frailty of older migrants has important implications for the demands placed on healthcare systems. Examining 95,635 Europeans in the Survey of Health, Aging and Retirement in Europe, we investigated cross-sectional and longitudinal associations between migration

and frailty at ages > 50 years. We examined whether associations differed by countries' level of healthcare coverage and access for migrants and tested mediation by home-ownership and citizenship. Cross-sectionally, first-generation migrants > 50 years old were, on average, 16.4% (95% confidence interval [CI]: 14.6, 18.2%) frailer than non-migrants after confounder-adjustment. This decreased to 12.1% (95% CI: 10.1, 14.1%) after adjustment for citizenship. The strength of association between migrant status and frailty was greater in migrants from low-or-middle-income countries, compared with migrants from high-income countries. Migrants into Northern, Western and Eastern Europe were 37.3% (95% CI: 33.2, 41.5%), 12.2% (95% CI: 10.0, 14.6%) and 5.0% (95% CI: 0.5, 9.6%) frailer than non-migrants, respectively, but migrants into Southern Europe were no frailer than non-migrants. The strength of association between migrant status and frailty was greater in countries with lower healthcare coverage and access for migrants. However, citizenship attenuated this difference. Longitudinally, migrants were frailer than non-migrants at 50 years old and trajectories converged over time until migrants and non-migrants were equally frail by 80-90 years. Our work finds no evidence of the 'healthy migrant effect' outside of Southern Europe in older migrants and suggests that acculturation is a key determinant of migrant health. [Walkden, G. J.; Tilling, K.; Ben-Shlomo, Y.] Univ Bristol, Populat Hlth Sci Inst, Canynge Hall,39 Whatley Rd, Bristol BS8 2PS, Avon, England; [Anderson, E. L.; Howe, L. D.] Univ Bristol, MRC, Integrat Epidemiol Unit, Sch Social & Community Med, Oakfield House, Bristol BS8 2BN, Avon, England; [Vink, M. P.] Maastricht Univ, Fac Arts & Social Sci, Dept Polit Sci, Grote Gracht 90-92,POB 616, NL-6200 MD Maastricht, Netherlands University of Bristol; University of Bristol; Maastricht University Walkden, GJ (corresponding author), Univ Bristol, Populat Hlth Sci Inst, Canynge Hall,39 Whatley Rd, Bristol BS8 2PS, Avon, England. g.walkden@bristol.ac.uk Ben-Shlomo, Yoav/ABD-2004-2021; Tilling, Kate/AAY-1578-2021; Vink, Maarten/B-6632-2015 Howe, Laura/0000-0003-3357-2796; Tilling, Kate/0000-0002-1010-8926; Ben-Shlomo, Yoav/0000-0001-6648-3007; Vink, Maarten/0000-0001-7143-4859; Anderson, Emma/0000-0002-1508-0598 European Commission through FP5 [QLK6-CT-2001-00360]; European Commission through FP6 [SHARE-I3: RII-CT-2006-062193, COMPARE: CIT5-CT-2005-028857, SHARELIFE: CIT4-CT-2006-028812]; European Commission through FP7 (SHARE-PREP) [211909]; European Commission through FP7 (SHARE-LEAP) [227822]; European Commission through FP7 (SHARE M4) [261982]; German Ministry of Education and Research; U.S. National Institute on Aging [U01_AG09740-13S2, P01_AG005842, P01_AG08291, P30_AG12815, R21_AG025169, Y1-AG-4553-01, IAG_BSR06-11, OGHA_04-064]; National Institute on Aging of the National Institutes of Health [R01AG048835]; UK Economic and Social Research Council [ES/M010317/1]; UK Medical Research Council [MR/M020894/1, MC_UU_12013/9]; University of Bristol; European Research Council [682626]; ESRC [ES/M010317/1] Funding Source: UKRI; MRC [MR/M020894/1, MR/P014437/1, MC_UU_12013/9] Funding Source: UKRI; European Research Council (ERC) [682626] Funding Source: European Research Council (ERC) European Commission through FP5; European Commission through FP6(European Union (EU)Marie Curie Actions); European Commission through FP7 (SHARE-PREP); European Commission through FP7 (SHARE-LEAP); European Commission through FP7 (SHARE M4); German Ministry of Education and Research(Federal Ministry of Education & Research

(BMBF)); U.S. National Institute on Aging(United States Department of Health & Human ServicesNational Institutes of Health (NIH) - USANIH National Institute on Aging (NIA)); National Institute on Aging of the National Institutes of Health(United States Department of Health & Human ServicesNational Institutes of Health (NIH) - USANIH National Institute on Aging (NIA)); UK Economic and Social Research Council(UK Research & Innovation (UKRI)Economic & Social Research Council (ESRC)); UK Medical Research Council(UK Research & Innovation (UKRI)Medical Research Council UK (MRC)); University of Bristol; European Research Council(European Research Council (ERC)); ESRC(UK Research & Innovation (UKRI)Economic & Social Research Council (ESRC)); MRC(UK Research & Innovation (UKRI)Medical Research Council UK (MRC)); European Research Council (ERC)(European Research Council (ERC)) The SHARE data collection has been primarily funded by the European Commission through FP5 (QLK6-CT-2001-00360), FP6 (SHARE-I3: RII-CT-2006-062193, COMPARE: CIT5-CT-2005-028857, SHARELIFE: CIT4-CT-2006-028812) and FP7 (SHARE-PREP: No211909, SHARE-LEAP: No227822, SHARE M4: No261982). Additional funding from the German Ministry of Education and Research, the U.S. National Institute on Aging (U01_AG09740-13S2, P01_AG005842, P01_AG08291, P30_AG12815, R21_AG025169, Y1-AG-4553-01, IAG_BSR06-11, OGHA_04-064) and from various national funding sources is gratefully acknowledged (see www.share-project.org). Research reported in this publication was supported by the National Institute on Aging of the National Institutes of Health under Award Number R01AG048835. The content is solely the responsibility of the authors and does not necessarily represent the official views of the National Institutes of Health. This work was supported by a grant from the UK Economic and Social Research Council [ES/M010317/1]. LDH is supported by a fellowship from the UK Medical Research Council [MR/M020894/1]. ELA, KT and LDH work in a unit that receives funding from the University of Bristol and the UK Medical Research Council [MC_UU_12013/9]. Maarten Vink acknowledges funding by the European Research Council (grant agreement No 682626). This project was stimulated by the World Universities Network Health Outcomes of Migration Events (HOME) group (see http://www.wun.ac.uk/wun/research/view/home-health-outcomes-of-migration-events for membership). 49 24 25 0 20
PERGAMON-ELSEVIER SCIENCE LTD OXFORD THE BOULEVARD, LANGFORD LANE, KIDLINGTON, OXFORD OX5 1GB, ENGLAND 0277-9536
SOC SCI MED Soc. Sci. Med. SEP 2018 213
1 11
10.1016/j.socscimed.2018.07.033
http://dx.doi.org/10.1016/j.socscimed.2018.07.033
11 Public, Environmental & Occupational Health; Social Sciences, Biomedical Science Citation Index Expanded (SCI-EXPANDED); Social Science Citation Index (SSCI) Public, Environmental & Occupational Health; Biomedical Social Sciences
GV3FW 30053619 Green Published, hybrid
2025-06-24 WOS:000445980800001
J Natalucci, V; Marini, CF; Flori, M; Pietropaolo, F; Lucertini, F; Annibalini, G; Vallorani, L; Sisti, D; Saltarelli, R; Villarini, A; Monaldi, S; Barocci, S; Catalano, V; Rocchi, MBL; Benelli, P; Stocchi, V; Barbieri, E; Emili, R
Natalucci, Valentina; Marini, Carlo Ferri; Flori, Marco; Pietropaolo, Francesca; Lucertini, Francesco; Annibalini, Giosue; Vallorani, Luciana; Sisti, Davide; Saltarelli, Roberta; Villarini,

Anna; Monaldi, Silvia; Barocci, Simone; Catalano, Vincenzo; Rocchi, Marco Bruno Luigi; Benelli, Piero; Stocchi, Vilberto; Barbieri, Elena; Emili, Rita Effects of a Home-Based Lifestyle Intervention Program on Cardiometabolic Health in Breast Cancer Survivors during the COVID-19 Lockdown JOURNAL OF CLINICAL MEDICINE English Article COVID-19; breast cancer survivors; home-based lifestyle intervention; Mediterranean diet; exercise; cardiotoxicity; cardiovascular fitness; heart rate variability; secondary and tertiary prevention HEART-RATE-VARIABILITY; PHYSICAL-ACTIVITY QUESTIONNAIRE; C-REACTIVE PROTEIN; QUALITY-OF-LIFE; METABOLIC SYNDROME; ADJUVANT CHEMOTHERAPY; CARDIOVASCULAR RESERVE; EXERCISE GUIDELINES; MEDITERRANEAN DIET; INSULIN-RESISTANCE This study aimed to evaluate the cardiometabolic effects of a home-based lifestyle intervention (LI) in breast cancer survivors (BCSs) during the COVID-19 lockdown. In total, 30 BCSs (women; stages 0-II; non-metastatic; aged 53.5 +/- 7.6 years; non-physically active; normal left ventricular systolic function) with a risk factor for recurrence underwent a 3-month LI based on nutrition and exercise. Anthropometrics, Mediterranean diet adherence, physical activity level (PAL), cardiorespiratory fitness (VO2max), echocardiographic parameters, heart rate variability (average standard deviation of NN intervals (ASDNN/5 min) and 24 h very- (24 hVLF) and low-frequency (24 hLF)), and metabolic, endocrine, and inflammatory serum biomarkers (glycemia, insulin resistance, progesterone, testosterone, and high-sensitivity C-reactive protein (hs-CRP)) were evaluated before (T0) and after (T1) the LI. After the LI, there were improvements in: body mass index (kg/m(2): T0 = 26.0 +/- 5.0, T1 = 25.5 +/- 4.7; p = 0.035); diet (Mediet score: T0 = 6.9 +/- 2.3, T1 = 8.8 +/- 2.2; p < 0.001); PAL (MET-min/week: T0 = 647 +/- 547, T1 = 1043 +/- 564; p < 0.001); VO2max (mL center dot min(-1)center dot kg(-1): T0 = 30.5 +/- 5.8, T1 = 33.4 +/- 6.8; p < 0.001); signs of diastolic dysfunction (participants: T0 = 15, T1 = 10; p = 0.007); AS-DNN/5 min (ms: T0 = 50.6 +/- 14.4, T1 = 55.3 +/- 16.7; p = 0.032); 24 hLF (ms(2): T0 = 589 +/- 391, T1 = 732 +/- 542; p = 0.014); glycemia (mg/dL: T0 = 100.8 +/- 11.4, T1 = 91.7 +/- 11.0; p < 0.001); insulin resistance (HOMA-IR score: T0 = 2.07 +/- 1.54, T1 = 1.53 +/- 1.11; p = 0.005); testosterone (ng/mL: T0 = 0.34 +/- 0.27, T1 = 0.24 +/- 0.20; p = 0.003); hs-CRP (mg/L: T0 = 2.18 +/- 2.14, T1 = 1.75 +/- 1.74; p = 0.027). The other parameters did not change. Despite the home-confinement, LI based on exercise and nutrition improved cardiometabolic health in BCSs. [Natalucci, Valentina; Marini, Carlo Ferri; Lucertini, Francesco; Annibalini, Giosue; Vallorani, Luciana; Sisti, Davide; Saltarelli, Roberta; Rocchi, Marco Bruno Luigi; Benelli, Piero; Barbieri, Elena] Univ Urbino Carlo Bo, Dept Biomol Sci, I-61029 Urbino, Italy; [Flori, Marco; Pietropaolo, Francesca] Osped S Maria Misericordia, UOC Cardiol UTIC, Area Vasta 1, I-61029 Urbino, Italy; [Villarini, Anna] Fdn IRCCS Ist Nazl Tumori, Dept Res, Epidemiol Unit, I-20133 Milan, Italy; [Monaldi, Silvia; Catalano, Vincenzo; Emili, Rita] Osped Santa Maria della Misericordia Urbino, ASUR Area Vasta 1, UOC Oncol Med, I-61029 Urbino, Italy; [Barocci, Simone] Osped S Maria Misericordia, UOC Patol Clin, Area Vasta 1, I-61029 Urbino, Italy; [Stocchi, Vilberto] Univ San Raffaele, Dept Human Sci Promot Qual Life, I-20132 Rome, Italy University of Urbino; Hospital Santa Maria della Misericordia; Fondazione IRCCS Istituto Nazionale Tumori Milan; Hospital Santa Maria della Misericordia;

Universita Telematica San Raffaele	Barbieri, E (corresponding author), Univ Urbino Carlo Bo, Dept Biomol Sci, I-61029 Urbino, Italy.	valentina.natalucci@uniurb.it; carlo.ferrimarini@uniurb.it; marco.flori@sanita.marche.it; francesca.pietropaolo@sanita.marche.it; francesco.lucertini@uniurb.it; giosue.annibalini@uniurb.it; luciana.vallorani@uniurb.it; davide.sisti@uniurb.it; roberta.saltarelli@uniurb.it; a.villarini@istitutotumori.mi.it; silvia.monaldi@sanita.marche.it; simone.barocci@sanita.marche.it; vincenzo.catalano@sanita.marche.it; marco.rocchi@uniurb.it; piero.benelli@uniurb.it; vilberto.stocchi@uniroma5.it; elena.barbieri@uniurb.it; rita.emili@sanita.marche.it
SALTARELLI, Roberta/AAM-4291-2021; Villarini, Anna/K-2838-2017; Rocchi, Mariano/B-8451-2011; Natalucci, Valentina/AAM-7882-2020; Marini, Carlo/AAA-9281-2019; Barbieri, Elena/AGI-0430-2022; Ferri Marini, Carlo/GRY-3765-2022; Lucertini, Francesco/F-6603-2012; Annibalini, Giosue/J-7701-2016	Natalucci, Valentina/0000-0002-0582-1575; Barbieri, Elena/0000-0002-3480-7983; Rocchi, marco/0000-0002-0056-5795; SALTARELLI, Roberta/0000-0002-6375-127X; Ferri Marini, Carlo/0000-0002-6866-997X; Stocchi, Vilberto/0000-0003-3269-9410; Lucertini, Francesco/0000-0003-3134-4511; davide, sisti/0000-0002-7925-7495; vallorani, luciana/0000-0002-0858-6275; Annibalini, Giosue/0000-0002-6914-4905
Department of Biomolecular Sciences, University of Urbino Carlo Bo, Urbino, Italy [38/2019]; Associazione Culturale "Golden Brain" ETS; Associazione "Le Contrade di Urbino"; Fondazione Cassa di Risparmio di Pesaro	Department of Biomolecular Sciences, University of Urbino Carlo Bo, Urbino, Italy; Associazione Culturale "Golden Brain" ETS; Associazione "Le Contrade di Urbino"; Fondazione Cassa di Risparmio di Pesaro	Natalucci was supported in this research by the co-founded Postdoctoral Fellowship (Delibera n. 38/2019 27/02/2019 Department of Biomolecular Sciences, University of Urbino Carlo Bo, Urbino, Italy; Associazione Culturale "Golden Brain" ETS; Associazione "Le Contrade di Urbino" and "Fondazione Cassa di Risparmio di Pesaro"). The authors thank all the participants of the study and extend their gratitude to Eugenio Grassi and Donatella Sarti for their valuable technical assistance during data acquisition.
107	28	29	1	12	MDPI	BASEL	ST ALBAN-ANLAGE 66, CH-4052 BASEL, SWITZERLAND		2077-0383		J CLIN MED	J. Clin. Med.	JUN	2021	10	12
2678	10.3390/jcm10122678
http://dx.doi.org/10.3390/jcm10122678			16
Medicine, General & Internal	Science Citation Index Expanded (SCI-EXPANDED)	General & Internal Medicine	TA7UY	34204528
gold, Green Published			2025-06-24
WOS:000667452700001
J	Nani, S; Hassoune, S; Benallal, M; Kissi, D; Maaroufi, A
Nani, Samira; Hassoune, Samira; Benallal, Mohamed; Kissi, Dounia; Maaroufi, Abderrahmane
Knowledge by physicians concerning cervical cancer and testing: Benimellal province, Morocco	EASTERN MEDITERRANEAN HEALTH JOURNAL		French	Article
GENERAL-PRACTITIONERS; PRIMARY-CARE; ATTITUDES; PREVENTION; DIAGNOSIS; REGION; IMPACT; SMEAR
Background: Cervical cancer is the second most common cancer among women after skin cancer. Aims: This study aimed to evaluate the knowledge of general practitioners with regard to cervical

cancer in the province of Benimellal, Morocco. Methods: A cross-sectional study using a self-reported questionnaire given to 71 general practitioners operating in public and private healthcare centres in the province of Benimellal. Results: Almost half of general practitioners (49.3%) were not aware of a national plan for the prevention and control of cancer in Morocco, and only 18.2% gave a correct response on the incidence rate for cervical cancer. The human papilloma virus as the principle risk factor was identified by 21% of general practitioners. Conclusions: The knowledge of general practitioners was deficient and therefore initial and continuing training on cervical cancer is required.
[Nani, Samira; Hassoune, Samira; Maaroufi, Abderrahmane] Fac Med & Pharm, Lab Epidemiol, Casablanca, Morocco; [Kissi, Dounia] Direct Reg Sante Grand Casablanca, Programme Canc Serv Sante Publ & Surveillance Epi, Casablanca, Morocco Nani, S (corresponding author), Fac Med & Pharm, Lab Epidemiol, Casablanca, Morocco. nanisamira@hotmail.com Maaroufi, Abderrahmane/0009-0003-6300-1588
27 2
2 0 2 WHO EASTERN MEDITERRANEAN REGIONAL OFFICE
NASR CITY, CAIRO P. O. BOX 7608, NASR CITY, CAIRO, EGYPT
1020-3397 1687-1634 E MEDITERR HEALTH J East Mediterr. Health J. DEC 2018 24 12
1135 1145 10.26719/2018.24.12.1135
http://dx.doi.org/10.26719/2018.24.12.1135 11
Health Care Sciences & Services; Health Policy & Services; Public, Environmental & Occupational Health Science Citation Index Expanded (SCI-EXPANDED); Social Science Citation Index (SSCI) Health Care Sciences & Services; Public, Environmental & Occupational Health HL7DU 30799553 Bronze
2025-06-24 WOS:000458899400004
J KEYDAR, I; SELZER, G; CHAITCHIK, S; HAREUVENI, M; KARBY, S; HIZI, A KEYDAR, I; SELZER, G; CHAITCHIK, S; HAREUVENI, M; KARBY, S; HIZI, A A VIRAL-ANTIGEN AS A MARKER FOR THE PROGNOSIS OF HUMAN-BREAST CANCER EUROPEAN JOURNAL OF CANCER & CLINICAL ONCOLOGY English
Article
An antigen present in human breast tumor cells, and which is immunologically related to the envelope protein (gp52) of murine mammary tumor virus, was used as a marker for the detection of breast cancer in an Israeli population. The antigen was detectable in 128 of 204 breast carcinoma tested (62.7%). The immunological reaction was not detected in normal breast tissue, benign breast tumors, ductal hyperplasia or in primary malignancies in other organs. A significantly higher percentage of cases with demonstrable antigen was found in Israeli women born in North Africa (78%) compared to women of European origin (60.6%). The frequency of detection of the antigen was higher in stage IV (80%) compared to state I (15%), suggesting that the gp52 cross-reacting antigen is a marker for the severity of the disease. A retrospective study of 97 cases of stage II breast cancer shows that if the antigen is detected at the time of mastectomy, an unfavorable prognosis can usually be predicted. Survival data analysis indicates that patients without detectable antigen survived significantly longer than those with a detectable antigen.
TEL HASHOMER HOSP, SHEBA MED CTR, DEPT PATHOL, TEL AVIV, ISRAEL; TEL AVIV UNIV, ROKACH HOSP, SCH MED, DEPT RADIOISOTOPES, TEL AVIV, ISRAEL; TEL AVIV UNIV, SACKLER SCH MED, DEPT HISTOL & CELL BIOL, TEL AVIV, ISRAEL Tel Aviv University;

Chaim Sheba Medical Center; Tel Aviv University; Tel Aviv University; Sackler Faculty of Medicine TEL AVIV UNIV, DR GEORGE S WISE CTR LIFE SCI, DEPT MICROBIOL, TEL AVIV, ISRAEL. 13 26 27 0 0
PERGAMON-ELSEVIER SCIENCE LTD OXFORD THE BOULEVARD, LANGFORD LANE, KIDLINGTON, OXFORD OX5 1GB, ENGLAND 0277-5379
EUR J CANCER CLIN ON 1982 18 12
1321 1328 10.1016/0277-5379(82)90136-5 http://dx.doi.org/10.1016/0277-5379(82)90136-5
8 Oncology Science Citation Index Expanded (SCI-EXPANDED) Oncology PY316 6299750
2025-06-24 WOS:A1982PY31600014
J CENTONZE, S; BOEING, H; LEOCI, C; GUERRA, V; MISCIAGNA, G
CENTONZE, S; BOEING, H; LEOCI, C; GUERRA, V; MISCIAGNA, G DIETARY HABITS AND COLORECTAL-CANCER IN A LOW-RISK AREA - RESULTS FROM A POPULATION-BASED CASE-CONTROL STUDY IN SOUTHERN ITALY NUTRITION AND CANCER-AN INTERNATIONAL JOURNAL English Article
COLON Many epidemiological studies have focused on the relationships between diet and colorectal cancer, but only a few have been conducted in the Mediterranean area. A population-based case-control study was carried out from July 1987 to June 1989 in a low-risk area in Southern Italy. By means of an ''ad hoc'' tumor registry, 132 diagnosed colorectal cancers were detected during the two years of study. One hundred nineteen of these 132 colorectal cancer cases were interviewed about their personal dietary habits with use of a questionnaire concerning the frequency of consumption of 70 foods or beverages. An equal number of controls was randomly selected from the lists of general practitioners of the area during the same period and interviewed with the same food frequency questionnaire. In a multivariate analysis, the relative risks (RRs) of developing colorectal cancer were estimated according to the different levels of consumption of food groups and selected food items. All RRs were adjusted for age, sex, education, smoking status, and modifications in diet in the previous 10 years. The risk of colorectal cancer increased nearly threefold for the highest level of consumption of foods with a high content of refined sugar [RR = 2.75, 95% confidence interval (CI) 1.26-5.97] and for the consumption of wine (>1 1/day) (RR = 3.22, 95% CI 1.05-9.88). An inverse relationship was revealed for the highest consumption of raw and cooked vegetables (RR = 0.51, 95% CI 0.25-1.04) and diary products (RR = 0.46, 95% CI 0.22-0.98) and for the consumption of more than two cups of coffee per day (RR = 0.38, 95% CI 0.16-0.89). In this Mediterranean area, the main source of calories, cereals, did not show a significant relationship with colorectal cancer. These findings support the hypothesis that the local Mediterranean dietary pattern could explain the low risk of colorectal cancer.
GERMAN INST HUMAN NUTR,MED EPIDEMIOL UNIT,POTSDAM,GERMANY
Leibniz Association; Deutsches Institut fur Ernahrungsforschung Potsdam-Rehbrucke (DIfE) CENTONZE, S (corresponding author), OSPED GASTROENTEROL,IRCCS S DE BELLIS,EPIDEMIOL & BIOSTAT LAB,VIA VALENTE 4,I-70013 CASTELLANA GROTTE,ITALY. 24 68
70 0 7 LAWRENCE ERLBAUM ASSOC INC MAHWAH 10 INDUSTRIAL AVE, MAHWAH, NJ 07430-2262 0163-5581 NUTR CANCER Nutr. Cancer 1994 21 3
233 246 10.1080/01635589409514322

http://dx.doi.org/10.1080/01635589409514322 14
Oncology; Nutrition & Dietetics Science Citation Index Expanded (SCI-EXPANDED) Oncology; Nutrition & Dietetics
NN828 8072877 2025-06-24
WOS:A1994NN82800005
J Zheng, XB; Hur, J; Nguyen, LH; Liu, J; Song, MY; Wu, KN; Smith-Warner, SA; Ogino, S; Willett, WC; Chan, AT; Giovannucci, E; Cao, Y Zheng, Xiaobin; Hur, Jinhee; Long H Nguyen; Liu, Jie; Song, Mingyang; Wu, Kana; Smith-Warner, Stephanie A.; Ogino, Shuji; Willett, Walter C.; Chan, Andrew T.; Giovannucci, Edward; Cao, Yin Comprehensive Assessment of Diet Quality and Risk of Precursors of Early-Onset Colorectal Cancer JNCI-JOURNAL OF THE NATIONAL CANCER INSTITUTE English Article
PATTERNS; METABOLITES; ADENOMA Background: The role of poor diet quality in the rising incidence of colorectal cancer (CRC) diagnosed younger than age 50 years has not been explored. Based on molecular features of early-onset CRC, early-onset adenomas are emerging surrogate endpoints. Methods: In a prospective cohort study (Nurses' Health Study II), we evaluated 2 empirical dietary patterns (Western and prudent) and 3 recommendation-based indexes (Dietary Approaches to Stop Hypertension [DASH], Alternative Mediterranean Diet [AMED], and Alternative Healthy Eating Index [AHEI]-2010) with risk of early-onset adenoma overall and by malignant potential (high-risk: >= 1 cm, tubulovillous or villous histology, high-grade dysplasia, or >= 3 adenomas), among 29 474 women with 1 or more lower endoscopy before age 50 years (1991-2011). Multivariable logistic regressions were used to estimate odds ratios (ORs) and 95% confidence intervals (CIs). Results: We documented 1157 early-onset adenomas with 375 at high risk. Western diet was positively associated, whereas prudent diet, DASH, AMED, and AHEI-2010 were inversely associated with risk of early-onset adenoma. The associations were largely confined to high-risk adenomas (the highest vs lowest quintile: Western, OR = 1.67, 95% CI = 1.18 to 2.37; prudent, OR = 0.69, 95% CI = 0.48 to 0.98; DASH, OR = 0.65, 95% CI = 0.45 to 0.93; AMED, OR = 0.55, 95% CI = 0.38 to 0.79; AHEI-2010, OR = 0.71, 95% CI = 0.51 to 1.01; all P-trend <=.03), driven by those identified in the distal colon and rectum (all P-trend <=.04, except AMED: P-trend =.14). Conclusion: Poor diet quality was associated with an increased risk of early-onset distal and rectal adenomas of high malignant potential. These findings provide preliminary but strong support to the role of diet in early-onset CRC. [Zheng, Xiaobin; Liu, Jie; Cao, Yin] Washington Univ, Dept Surg, Div Publ Hlth Sci, Sch Med, 660 S Euclid Ave,Campus Box 8100, St Louis, MO 63110 USA; [Zheng, Xiaobin] Sun Yat Sen Univ, Affiliated Hosp 6, Dept Colorectal Surg, Guangzhou, Peoples R China; [Zheng, Xiaobin] Sun Yat Sen Univ, Affiliated Hosp 6, Guangdong Prov Key Lab Colorectal & Pelv Floor Di, Guangzhou, Peoples R China; [Hur, Jinhee; Song, Mingyang; Wu, Kana; Smith-Warner, Stephanie A.; Willett, Walter C.; Giovannucci, Edward] Harvard TH Chan Sch Publ Hlth, Dept Nutr, Boston, MA USA; [Long H Nguyen; Song, Mingyang; Chan, Andrew T.] Massachusetts Gen Hosp, Clin & Translat Epidemiol Unit, Boston, MA 02114 USA; [Long H Nguyen; Song, Mingyang; Ogino, Shuji; Willett, Walter C.; Chan, Andrew T.; Giovannucci, Edward] Harvard Med Sch, Boston, MA 02114 USA; [Long H Nguyen; Song, Mingyang; Chan, Andrew T.] Massachusetts Gen Hosp, Div Gastroenterol, Boston, MA 02114

USA; [Liu, Jie] Washington Univ, Brown Sch, St Louis, MO 63110 USA; [Song, Mingyang; Smith-Warner, Stephanie A.; Ogino, Shuji; Willett, Walter C.; Giovannucci, Edward] Harvard TH Chan Sch Publ Hlth, Dept Epidemiol, Boston, MA USA; [Ogino, Shuji] Brigham & Womens Hosp, Dept Pathol, Program MPE Mol Pathol Epidemiol, 75 Francis St, Boston, MA 02115 USA; [Ogino, Shuji; Chan, Andrew T.] MIT & Harvard Univ, Eli & Edythe L Broad Inst, Cambridge, MA USA; [Willett, Walter C.; Chan, Andrew T.; Giovannucci, Edward] Brigham & Womens Hosp, Dept Med, Channing Div Network Med, 75 Francis St, Boston, MA 02115 USA; [Chan, Andrew T.] Harvard TH Chan Sch Publ Hlth, Dept Immunol & Infect Dis, Boston, MA USA; [Cao, Yin] Washington Univ, Sch Med, Alvin J Siteman Canc Ctr, St Louis, MO USA; [Cao, Yin] Washington Univ, Sch Med, Dept Med, Div Gastroenterol, St Louis, MO 63110 USA Washington University (WUSTL); Sun Yat Sen University; Sun Yat Sen University; Harvard University; Harvard T.H. Chan School of Public Health; Harvard University; Harvard University Medical Affiliates; Massachusetts General Hospital; Harvard University; Harvard Medical School; Harvard University; Harvard University Medical Affiliates; Massachusetts General Hospital; Washington University (WUSTL); Harvard University; Harvard T.H. Chan School of Public Health; Harvard University; Harvard University Medical Affiliates; Brigham & Women's Hospital; Harvard University; Massachusetts Institute of Technology (MIT); Broad Institute; Harvard University; Harvard University Medical Affiliates; Brigham & Women's Hospital; Harvard University; Harvard T.H. Chan School of Public Health; Washington University (WUSTL); Siteman Cancer Center; Washington University (WUSTL) Cao, Y (corresponding author), Washington Univ, Dept Surg, Div Publ Hlth Sci, Sch Med, 660 S Euclid Ave,Campus Box 8100, St Louis, MO 63110 USA. yin.cao@wustl.edu nguyen, long/KHV-1588-2024; Willett, Walter/E-2352-2013; Giovannucci, Edward/ADE-8028-2022; Cao, Yin/ABE-2332-2021; Chan, Andrew/ADM-9271-2022; Song, Mingyang/M-6701-2013 Nguyen, Long/0000-0002-5436-4219; Hur, Jinhee/0000-0003-0968-208X; Cao, Yin/0000-0001-9835-7662; Willett, Walter/0000-0003-1458-7597; Liu, Jie/0000-0001-6365-1888; Ogino, Shuji/0000-0002-3909-2323 National Institutes of Health (NIH) [U01 CA176726, R00 CA215314, R03 CA197879, R21 CA222940, R21 CA230873, R01 CA151993, R35 CA197735, K24 DK098311, R37 CA246175, K07 CA218377]; International Program for PhD Candidates, Sun Yat-Sen University; NIH Loan Repayment Program Scholarship; Crohn's and Colitis Foundation Research Fellowship Award; Mentored Research Scholar Grant in Applied and Clinical Research from the American Cancer Society [MRSG-17-220-01-NEC]; American Institute for Cancer Research; Dana-Farber Harvard Cancer Center; Project P Fund for Colorectal Cancer Research; Friends of the Dana-Farber Cancer Institute; Bennett Family Fund; Entertainment Industry Foundation through National Colorectal Cancer Research Alliance; American Association for Cancer Research (Stand Up to Cancer Colorectal Cancer Dream Team Translational Research Grant); National Cancer Institute [U01CA176726, K07CA218377] Funding Source: NIH RePORTER National Institutes of Health (NIH)(United States Department of Health & Human ServicesNational Institutes of Health (NIH) - USA); International Program for PhD Candidates, Sun Yat-Sen University; NIH Loan Repayment Program Scholarship(United States Department of Health & Human ServicesNational Institutes of Health (NIH) - USAOffice of the Administrator (NIH)); Crohn's and Colitis Foundation Research Fellowship Award; Mentored Research Scholar

Grant in Applied and Clinical Research from the American Cancer Society; American Institute for Cancer Research; Dana-Farber Harvard Cancer Center; Project P Fund for Colorectal Cancer Research; Friends of the Dana-Farber Cancer Institute; Bennett Family Fund; Entertainment Industry Foundation through National Colorectal Cancer Research Alliance; American Association for Cancer Research (Stand Up to Cancer Colorectal Cancer Dream Team Translational Research Grant); National Cancer Institute(United States Department of Health & Human ServicesNational Institutes of Health (NIH) - USANIH National Cancer Institute (NCI)) The content is solely the responsibility of the authors and does not necessarily represent the official views of the National Institut This work was supported by the National Institutes of Health (NIH; NHSII cohort infrastructure grant of U01 CA176726, R00 CA215314 to MS; R03 CA197879 and R21 CA222940 to KW; R21 CA230873 to KWand SO; R01 CA151993 and R35 CA197735 to SO; K24 DK098311 to ATC; R37 CA246175 and K07 CA218377 to YC). The content is solely the responsibility of the authors and does not necessarily represent the official views of the National Institutes of Health. XZ was supported by International Program for PhD Candidates, Sun Yat-Sen University. LHN is supported by an NIH Loan Repayment Program Scholarship and a Crohn's and Colitis Foundation Research Fellowship Award. MS is supported by a Mentored Research Scholar Grant in Applied and Clinical Research, MRSG-17-220-01-NEC, from the American Cancer Society. KW is supported by an Investigator Initiated Grant from the American Institute for Cancer Research. SO is supported by Nodal Award from the Dana-Farber Harvard Cancer Center and by grants from the Project P Fund for Colorectal Cancer Research, the Friends of the Dana-Farber Cancer Institute, Bennett Family Fund, the Entertainment Industry Foundation through National Colorectal Cancer Research Alliance and American Association for Cancer Research (Stand Up to Cancer Colorectal Cancer Dream Team Translational Research Grant). ATC is a Stuart and Suzanne Steele MGH Research Scholar.	68	90	97
	2	32	OXFORD UNIV PRESS INC	CARY	JOURNALS DEPT, 2001 EVANS RD, CARY, NC 27513 USA	0027-8874	1460-2105		JNCI-J NATL CANCER I	JNCI-J. Natl. Cancer Inst.	MAY	2021	113	5
			543	552		10.1093/jnci/djaa164
	http://dx.doi.org/10.1093/jnci/djaa164		JAN 2021	10
	Oncology	Science Citation Index Expanded (SCI-EXPANDED)
	Oncology	SI2FG	33136160	Green Published
	2025-06-24	WOS:000654641000008
J	Díaz-Gay, M; dos Santos, W; Moody, S; Kazachkova, M; Abbasi, A; Steele, CD; Vangara, R; Senkin, S; Wang, JW; Fitzgerald, S; Bergstrom, EN; Khandekar, A; Otlu, B; Abedi-Ardekani, B; de Carvalho, AC; Cattiaux, T; Penha, RCC; Gaborieau, V; Chopard, P; Carreira, C; Cheema, S; Latimer, C; Teague, JW; Mukeriya, A; Zaridze, D; Cox, R; Albert, M; Phouthavongsy, L; Gallinger, S; Malekzadeh, R; Niavarani, A; Miladinov, M; Eric, K; Milosavljevic, S; Sangrajrang, S; Curado, MP; Aguiar, S; Reis, RM; Reis, MT; Romagnolo, LG; Guimaraes, DP; Holcatova, I; Kalvach, J; Vaccaro, CA; Piñero, TA; Swiatkowska, B; Lissowska, J; Roszkowska-Purska, K; Huertas-Salgado, A; Shibata, T; Shiba, S; Sangkhathat, S; Chitapanarux, T; Roshandel, G; Ashton-Prolla, P; Damin, DC; de Oliveira, FH; Humphreys, L; Lawley, TD; Perdomo, S; Stratton, MR; Brennan, P; Alexandrov, LB			Diaz-Gay, Marcos; dos Santos, Wellington; Moody, Sarah; Kazachkova, Mariya; Abbasi, Ammal; Steele, Christopher D.; Vangara, Raviteja; Senkin, Sergey;

Wang, Jingwei; Fitzgerald, Stephen; Bergstrom, Erik N.; Khandekar, Azhar; Otlu, Burcak; Abedi-Ardekani, Behnoush; de Carvalho, Ana Carolina; Cattiaux, Thomas; Penha, Ricardo Cortez Cardoso; Gaborieau, Valerie; Chopard, Priscilia; Carreira, Christine; Cheema, Saamin; Latimer, Calli; Teague, Jon W.; Mukeriya, Anush; Zaridze, David; Cox, Riley; Albert, Monique; Phouthavongsy, Larry; Gallinger, Steven; Malekzadeh, Reza; Niavarani, Ahmadreza; Miladinov, Marko; Eric, Katarina; Milosavljevic, Sasa; Sangrajrang, Suleeporn; Curado, Maria Paula; Aguiar, Samuel; Reis, Rui Manuel; Reis, Monise Tadin; Romagnolo, Luis Gustavo; Guimaraes, Denise Peixoto; Holcatova, Ivana; Kalvach, Jaroslav; Vaccaro, Carlos Alberto; Pinero, Tamara Alejandra; Swiatkowska, Beata; Lissowska, Jolanta; Roszkowska-Purska, Katarzyna; Huertas-Salgado, Antonio; Shibata, Tatsuhiro; Shiba, Satoshi; Sangkhathat, Surasak; Chitapanarux, Taned; Roshandel, Gholamreza; Ashton-Prolla, Patricia; Damin, Daniel C.; de Oliveira, Francine Hehn; Humphreys, Laura; Lawley, Trevor D.; Perdomo, Sandra; Stratton, Michael R.; Brennan, Paul; Alexandrov, Ludmil B.

Geographic and age variations in mutational processes in colorectal cancer NATURE English Article; Early Access GENOMIC LANDSCAPE; READ ALIGNMENT; SIGNATURES; EPIDEMIOLOGY; BIOLOGY

Incidence rates of colorectal cancer vary geographically and have changed over time1. Notably, in the past two decades, the incidence of early-onset colorectal cancer, which affects individuals below 50 years of age, has doubled in many countries2, 3, 4-5. The reasons for this increase are unknown. Here we investigate whether mutational processes contribute to geographic and age-related differences by examining 981 colorectal cancer genomes from 11 countries. No major differences were found in microsatellite-unstable cancers, but variations in mutation burden and signatures were observed in the 802 microsatellite-stable cases. Multiple signatures, most with unknown aetiologies, exhibited varying prevalence in Argentina, Brazil, Colombia, Russia and Thailand, indicating geographically diverse levels of mutagenic exposure. Signatures SBS88 and ID18, caused by the bacteria-produced mutagen colibactin6,7, had higher mutation loads in countries with higher colorectal cancer incidence rates. SBS88 and ID18 were also enriched in early-onset colorectal cancers, being 3.3 times more common in individuals who were diagnosed before 40 years of age than in those over 70 years of age, and were imprinted early during colorectal cancer development. Colibactin exposure was further linked to APC driver mutations, with ID18 being responsible for about 25% of APC driver indels in colibactin-positive cases. This study reveals geographic and age-related variations in colorectal cancer mutational processes, and suggests that mutagenic exposure to colibactin-producing bacteria in early life may contribute to the increasing incidence of early-onset colorectal cancer. [Diaz-Gay, Marcos; Kazachkova, Mariya; Abbasi, Ammal; Steele, Christopher D.; Vangara, Raviteja; Bergstrom, Erik N.; Khandekar, Azhar; Otlu, Burcak; Alexandrov, Ludmil B.] Univ Calif San Diego, Dept Cellular & Mol Med, La Jolla, CA 92093 USA; [Diaz-Gay, Marcos; Abbasi, Ammal; Steele, Christopher D.; Vangara, Raviteja; Bergstrom, Erik N.; Khandekar, Azhar; Otlu, Burcak; Alexandrov, Ludmil B.] Univ Calif San Diego, Dept Bioengn, La Jolla, CA 92093 USA; [Diaz-Gay, Marcos; Kazachkova, Mariya; Abbasi, Ammal; Steele, Christopher D.; Vangara, Raviteja; Bergstrom, Erik N.; Khandekar, Azhar; Otlu,

Burcak; Alexandrov, Ludmil B.] Univ Calif San Diego, Moores Canc Ctr, La Jolla, CA 92093 USA; [Diaz-Gay, Marcos] Spanish Natl Canc Res Ctr CNIO, Struct Biol Program, Digital Genom Grp, Madrid, Spain; [dos Santos, Wellington; Senkin, Sergey; Abedi-Ardekani, Behnoush; de Carvalho, Ana Carolina; Cattiaux, Thomas; Penha, Ricardo Cortez Cardoso; Gaborieau, Valerie; Chopard, Priscilia; Perdomo, Sandra; Brennan, Paul] Int Agcy Res Canc IARC WHO, Genom Epidemiol Branch, Lyon, France; [Moody, Sarah; Wang, Jingwei; Fitzgerald, Stephen; Cheema, Saamin; Latimer, Calli; Teague, Jon W.; Humphreys, Laura; Stratton, Michael R.] Wellcome Sanger Inst, Canc Ageing & Somat Mutat, Cambridge, England; [Kazachkova, Mariya] Univ Calif San Diego, Biomed Sci Grad Program, La Jolla, CA USA; [Khandekar, Azhar] NCI, Div Canc Epidemiol & Genet, Bethesda, MD USA; [Otlu, Burcak] Middle East Tech Univ, Grad Sch Informat, Dept Hlth Informat, Ankara, Turkiye; [Carreira, Christine] Int Agcy Res Canc IARC, Evidence Synth & Classificat Branch, WHO, Lyon, France; [Mukeriya, Anush; Zaridze, David] N N Blokhin Natl Med Res Ctr Oncol, Moscow 115478, Russia; [Cox, Riley; Albert, Monique; Phouthavongsy, Larry] Ontario Inst Canc Res, Ontario Tumour Bank, Toronto, ON, Canada; [Albert, Monique] Univ Guelph, Ctr Biodivers Genom, Guelph, ON, Canada; [Gallinger, Steven] Sinai Hlth Syst, Lunenfeld Tanenbaum Res Inst, Toronto, ON, Canada; [Malekzadeh, Reza; Niavarani, Ahmadreza] Univ Tehran Med Sci, Digest Dis Res Inst, Digest Oncol Res Ctr, Tehran 1411713135, Iran; [Miladinov, Marko] Univ Clin Ctr Serbia, Clin Digest Surg, Surg Clin 1, Belgrade, Serbia; [Eric, Katarina] Univ Clin Ctr Serbia, Dept Pathol, Belgrade, Serbia; [Milosavljevic, Sasa] Int Org Canc Prevent & Res, Belgrade, Serbia; [Sangrajrang, Suleeporn] NCI, Bangkok, Thailand; [Curado, Maria Paula] AC Camargo Canc Ctr, Dept Epidemiol, Sao Paulo, Brazil; [Aguiar, Samuel] AC Camargo Canc Ctr, Colorectal Canc Reference Ctr, Sao Paulo, Brazil; [Reis, Rui Manuel] Barretos Canc Hosp, Mol Oncol Res Ctr, Barretos, Brazil; [Reis, Rui Manuel] Minho Univ, Life & Hlth Sci Res Inst ICVS, Sch Med, Braga, Portugal; [Reis, Monise Tadin] Barretos Canc Hosp, Dept Pathol, Barretos, Brazil; [Romagnolo, Luis Gustavo] Barretos Canc Hosp, Dept Colorectal Oncol Surg, Barretos, Brazil; [Guimaraes, Denise Peixoto] Barretos Canc Hosp, Dept Endoscopy, Barretos, Brazil; [Holcatova, Ivana] Charles Univ Prague, Univ Hosp Motol, Fac Med 2, Dept Paediat Haematol & Oncol, Prague, Czech Republic; [Holcatova, Ivana] Charles Univ Prague, Fac Med 1, Inst Hyg & Epidemiol, Prague, Czech Republic; [Kalvach, Jaroslav] Charles Univ Prague, Fac Med 2, Surg Dept, Prague, Czech Republic; [Kalvach, Jaroslav] Cent Mil Hosp, Prague, Czech Republic; [Kalvach, Jaroslav] Charles Univ Prague, Motol Univ Hosp, Fac Med 2, Prague, Czech Republic; [Kalvach, Jaroslav] Motol Univ Hosp, Prague, Czech Republic; [Kalvach, Jaroslav] Czech Acad Sci, Inst Anim Physiol & Genet, Libechov, Czech Republic; [Kalvach, Jaroslav] ISCARE Clin Ctr, Prague, Czech Republic; [Vaccaro, Carlos Alberto; Pinero, Tamara Alejandra] Univ Hosp Italiano Buenos Aires UHIBA, Inst Med Traslac Ingn Biomed IMTIB, CONICET, Buenos Aires, Argentina; [Vaccaro, Carlos Alberto; Pinero, Tamara Alejandra] Hosp Italiano Buenos Aires HIBA, Buenos Aires, Argentina; [Swiatkowska, Beata] Nofer Inst Occupat Med, Dept Environm Epidemiol, Lodz, Poland; [Lissowska, Jolanta] Maria Sklodowska Cure Natl Res Inst Oncol, Warsaw, Poland; [Roszkowska-Purska, Katarzyna] Med Univ Warsaw, Dept Anat Pathol, Warsaw, Poland; [Huertas-Salgado, Antonio] Inst Nacl Cancerol, Dept Gynecol Oncol, Bogota, Colombia; [Shibata,

Tatsuhiro] Univ Tokyo, Inst Med Sci, Mol Med Lab, Minato Ku, Tokyo 1088639, Japan; [Shibata, Tatsuhiro; Shiba, Satoshi] Natl Canc Ctr, Res Inst, Div Canc Genom, Chuo Ku, Tokyo, Japan; [Sangkhathat, Surasak] Prince Songkla Univ, Fac Med, Translat Med Res Ctr, Hat Yai, Thailand; [Sangkhathat, Surasak] Prince Songkla Univ, Fac Med, Dept Biomed Sci & Biomed Engn, Hat Yai, Thailand; [Sangkhathat, Surasak] Prince Songkla Univ, Fac Med, Dept Surg, Hat Yai, Thailand; [Chitapanarux, Taned] Chiang Mai Univ, Fac Med, Dept Internal Med, Chiang Mai, Thailand; [Roshandel, Gholamreza] Golestan Univ Med Sci, Golestan Res Ctr Gastroenterol & Hepatol, Gorgan, Iran; [Ashton-Prolla, Patricia] Fed Univ Rio Grande UFRGS, Dept Physiol, Porto Alegre, Brazil; [Ashton-Prolla, Patricia] Hosp Clin Porto Alegre HCPA, Med Genet Serv, Porto Alegre, Brazil; [Damin, Daniel C.] Hosp Clin Porto Alegre HCPA, Dept Surg, Div Colorectal Surg, Porto Alegre, Brazil; [de Oliveira, Francine Hehn] Hosp Clin Porto Alegre HCPA, Dept Pathol Anat Pathol, Porto Alegre, Brazil; [Lawley, Trevor D.] Wellcome Sanger Inst, Parasites & Microbes, Cambridge, England; [Alexandrov, Ludmil B.] Univ Calif San Diego, Sanford Stem Cell Inst, La Jolla, CA USA

University of California System; University of California San Diego; University of California System; University of California San Diego; University of California System; University of California San Diego; Centro Nacional de Investigaciones Oncologicas (CNIO); World Health Organization; International Agency for Research on Cancer (IARC); Wellcome Trust Sanger Institute; University of California System; University of California San Diego; National Institutes of Health (NIH) - USA; NIH National Cancer Institute (NCI); NIH National Cancer Institute- Division of Cancer Epidemiology & Genetics; Middle East Technical University; World Health Organization; International Agency for Research on Cancer (IARC); University of Toronto; Ontario Institute for Cancer Research; University of Guelph; University of Toronto; Sinai Health System Toronto; Lunenfeld Tanenbaum Research Institute; Tehran University of Medical Sciences; Clinical Centre of Serbia; Clinical Centre of Serbia; A.C.Camargo Cancer Center; A.C.Camargo Cancer Center; Hospital de Cancer de Barretos; Universidade do Minho; Hospital de Cancer de Barretos; Hospital de Cancer de Barretos; Hospital de Cancer de Barretos; Motol University Hospital; Charles University Prague; Charles University Prague; Charles University Prague; Motol University Hospital; Military University Hospital Prague; Charles University Prague; Motol University Hospital; Motol University Hospital; Czech Academy of Sciences; Institute of Animal Physiology & Genetics of the Czech Academy of Sciences; Consejo Nacional de Investigaciones Cientificas y Tecnicas (CONICET); Nofer Institute of Occupational Medicine; Medical University of Warsaw; University of Tokyo; National Cancer Center - Japan; Prince of Songkla University; Prince of Songkla University; Prince of Songkla University; Chiang Mai University; Golestan University of Medical Sciences; Universidade Federal do Rio Grande do Sul; Hospital de Clinicas de Porto Alegre; Hospital de Clinicas de Porto Alegre; Hospital de Clinicas de Porto Alegre; Wellcome Trust Sanger Institute; University of California System; University of California San Diego Alexandrov, LB (corresponding author), Univ Calif San Diego, Dept Cellular & Mol Med, La Jolla, CA 92093 USA.; Alexandrov, LB (corresponding author), Univ Calif San Diego, Dept Bioengn, La Jolla, CA 92093 USA.; Alexandrov, LB (corresponding author), Univ Calif San Diego, Moores Canc Ctr, La Jolla, CA 92093

USA. L2alexandrov@health.ucsd.eduSwiatkowska, Beata/A-4175-2011; Curado, Maria/M-6200-2013; Zaridze, David/K-5605-2013; GUIMARÃES, DENISE/G-4892-2012; Sangkhathat, Surasak/F-6397-2010; Niavarani, Ahmadreza/O-1814-2019; Alexandrov, Ludmil/AAS-1454-2021; Shiba, Satoshi/AAJ-2157-2021; mansournia, Mohammad/AFA-8899-2022; Otlu, Burçak/LIC-1641-2024; Diaz-Gay, Marcos/G-4864-2017; Reis, Rui Manuel/B-1744-2018   Diaz-Gay, Marcos/0000-0003-0658-0467; Reis, Rui Manuel/0000-0002-9639-7940   Cancer Research UK [C98/A24032]; US National Institute of Health (NIH) [R01ES032547-01, R01CA269919-01, 1U01CA290479-01]; Packard Fellowship for Science and Engineering; IARC Fellowship Award; Mark Foundation for Cancer Research; Wellcome Trust [206194, 220540/Z/20/A]; Barretos Cancer Hospital, in Brazil; Public Ministry of Labor Campinas (Research, Prevention, and Education of Occupational Cancer) [C005/24-ED CV1]; European Union NextGenerationEU funds, through PRTR; Practical Research for Innovative Cancer Control from the Japan Agency for Medical Research and Development (AMED) [JP 24ck0106800h0002]; National Cancer Center Research and Development Fund [2023-A-05]; NIH [U01CA167551]   Cancer Research UK(Cancer Research UK); US National Institute of Health (NIH)(United States Department of Health & Human ServicesNational Institutes of Health (NIH) - USA); Packard Fellowship for Science and Engineering; IARC Fellowship Award; Mark Foundation for Cancer Research; Wellcome Trust(Wellcome Trust); Barretos Cancer Hospital, in Brazil; Public Ministry of Labor Campinas (Research, Prevention, and Education of Occupational Cancer); European Union NextGenerationEU funds, through PRTR(European Union (EU)Marie Curie Actions); Practical Research for Innovative Cancer Control from the Japan Agency for Medical Research and Development (AMED)(Japan Agency for Medical Research and Development (AMED)); National Cancer Center Research and Development Fund(National Cancer Center - Japan); NIH(United States Department of Health & Human ServicesNational Institutes of Health (NIH) - USA)   The authors thank the IARC General Services, including the Laboratory Services and Biobank team led by Z. Kozlakidis and the Section of Support to Research overseen by C. Mehta under IARC regular budget funding for the support provided; L. O'Neill, K. Roberts, K. Smith, S. Austin-Guest and the staff of Sequencing Operations at the Wellcome Sanger Institute for their contribution; L. Rodriguez Porras for her help in designing and reviewing the figures; the work of all other collaborators in the Mutographs project who participated in the recruitment of patients in all centres; and all the patients involved in this study and their families. The computational analyses reported in this manuscript have utilized the Triton Shared Computing Cluster at the San Diego Supercomputer Center of UC San Diego. Where authors are identified as personnel of the International Agency for Research on Cancer/World Health Organization, the authors alone are responsible for the views expressed in this article and they do not necessarily represent the decisions, policy or views of the International Agency for Research on Cancer/World Health Organization. This work was delivered as part of the Mutographs team supported by the Cancer Grand Challenges partnership funded by Cancer Research UK (C98/A24032). Work at UC San Diego was also supported by the US National Institute of Health (NIH) grants R01ES032547-01, R01CA269919-01 and 1U01CA290479-01 to L.B.A., a Packard Fellowship for Science and Engineering to L.B.A. The research performed in the laboratory of L.B.A. was further supported by UC San Diego

Sanford Stem Cell Institute. This work was supported in part by an IARC Fellowship Award to W.d.S. through The Mark Foundation for Cancer Research. Work at the IARC/WHO was also supported by regular budget funding. Work at the Wellcome Sanger Institute was also supported by the Wellcome Trust (grants 206194 and 220540/Z/20/A). Porto Alegre center in Brazil received support from Hospital de Clinicas de Porto Alegre and Fundac & atilde;o Medica do Rio Grande do Sul. Barretos Cancer Hospital, in Brazil, was also supported by the Public Ministry of Labor Campinas (Research, Prevention, and Education of Occupational Cancer). M.D.-G. fellowship within the "Generacion D" initiative, Red.es, Ministerio para la Transformacion Digital y de la Funcion Publica, for talent attraction (C005/24-ED CV1), is funded by the European Union NextGenerationEU funds, through PRTR. This work was supported by grants from Practical Research for Innovative Cancer Control from the Japan Agency for Medical Research and Development (AMED) (JP 24ck0106800h0002 to T.S.) and the National Cancer Center Research and Development Fund (2023-A-05 to T.S.). Work at Sinai Health System, Toronto, Canada received support from the NIH (grant U01CA167551). The designations employed and the presentation of the material in this publication, in particular in Figs. 1 and 2, do not imply the expression of any opinion whatsoever on the part of the authors or their institutions concerning the legal status of any country, territory, city or area or of its authorities, or concerning the delimitation of its frontiers or boundaries. The funders had no roles in study design, data collection and analysis, decision to publish, or preparation of the manuscript. 78 0 0 12 12 NATURE PORTFOLIO BERLIN HEIDELBERGER PLATZ 3, BERLIN, 14197, GERMANY 0028-0836 1476-4687 NATURE Nature 2025 APR 23 2025
10.1038/s41586-025-09025-8
http://dx.doi.org/10.1038/s41586-025-09025-8 APR 2025
34 Multidisciplinary Sciences Science Citation Index Expanded (SCI-EXPANDED) Science & Technology - Other Topics
3EB6H 40267983 2025-06-24
WOS:001498133700001

J Arslan, ZÇ; Yalçin, YD; Külah, H Arslan, Zeynep Caglayan; Yalcin, Yagmur Demircan; Kulah, Haluk

Label-free enrichment of MCF7 breast cancer cells from leukocytes using continuous flow dielectrophoresis ELECTROPHORESIS English Article

circulating tumor cell; CTC enrichment; dielectrophoresis; leukocyte; MCF7 breast cancer cell CIRCULATING TUMOR-CELLS; MULTIDRUG-RESISTANCE; PREDICT SURVIVAL; SEPARATION; CAPTURE; BLOOD; PLATFORM; EPCAM; MICROFLUIDICS; IMMUNOCAPTURE

Circulating tumor cells (CTCs) present in the bloodstream are strongly linked to the invasive behavior of cancer; therefore, their detection holds great significance for monitoring disease progression. Currently available CTC isolation tools are often based on tumor-specific antigen or cell size approaches. However, these techniques are limited due to the lack of a unique and universal marker for CTCs, and the overlapping size between CTCs and regular blood cells. Dielectrophoresis (DEP), governed by the intrinsic dielectric properties of the particles, is a promising marker-free, accurate, fast, and low-cost technique that enables the isolation of CTCs from blood cells. This study presents a continuous flow, antibody-free DEP-based microfluidic device to

concentrate MCF7 breast cancer cells, a well-established CTC model, in the presence of leukocytes extracted from human blood samples. The enrichment strategy was determined according to the DEP responses of the corresponding cells, obtained in our previously reported DEP spectrum study. It was based on the positive-DEP integrated with hydrodynamic focusing under continuous flow. In the proposed device, the parylene microchannel with two inlets and outlets was built on top of rectangular and equally spaced isolated planar electrodes rotated certain degree relative to the main flow (13 degrees). The recovery of MCF7 cells mixed with leukocytes was 74%-98% at a frequency of 1 MHz and a magnitude of 10-12 V-pp. Overall, the results revealed that the presented system successfully concentrates MCF7 cancer cells from leukocytes, ultimately verifying our DEP spectrum study, in which the enrichment frequency and separation strategy of the microfluidic system were determined. [Arslan, Zeynep Caglayan; Yalcin, Yagmur Demircan; Kulah, Haluk] Middle East Tech Univ METU, Dept Elect & Elect Engn, Dept DZ 05,Univ Mah,Dumlupmar Blv, TR-06800 Ankara, Turkey; [Arslan, Zeynep Caglayan; Kulah, Haluk] METU MEMS Ctr, Ankara, Turkey Zorlu Holding; Middle East Technical University Külah, H (corresponding author), Middle East Tech Univ METU, Dept Elect & Elect Engn, Dept DZ 05,Univ Mah,Dumlupmar Blv, TR-06800 Ankara, Turkey. kulah@metu.edu.tr Külah, Haluk/AAN-2377-2021; Çağlayan Arslan, Zeynep/G-3254-2019 kulah, haluk/0000-0003-1331-4474; Caglayan Arslan, Zeynep/0000-0001-7893-4266 Scientific and Technological Research Council of Turkey (TUBITAK) [213E024]; Republic of Turkey Ministry of Development [BAP-2016K121290] Scientific and Technological Research Council of Turkey (TUBITAK)(Turkiye Bilimsel ve Teknolojik Arastirma Kurumu (TUBITAK)); Republic of Turkey Ministry of Development(Turkiye Cumhuriyeti Kalkinma Bakanligi) The Scientific and Technological Research Council of Turkey (TUBITAK), Grant/Award Number: 213E024; Republic of Turkey Ministry of Development, Grant/Award Number: BAP-2016K121290 85 13 13 7 56 WILEY HOBOKEN 111 RIVER ST, HOBOKEN 07030-5774, NJ USA 0173-0835 1522-2683 ELECTROPHORESIS Electrophoresis JUL 2022 43 13-14 1531 1544 10.1002/elps.202100318 http://dx.doi.org/10.1002/elps.202100318 APR 2022 14 Biochemical Research Methods; Chemistry, Analytical Science Citation Index Expanded (SCI-EXPANDED) Biochemistry & Molecular Biology; Chemistry 2W7BL 35318696 2025-06-24 WOS:000787281700001

J Shively, CA; Register, TC; Appt, SE; Clarkson, TB; Uberseder, B; Clear, KYJ; Wilson, AS; Chiba, A; Tooze, JA; Cook, KL Shively, Carol A.; Register, Thomas C.; Appt, Susan E.; Clarkson, Thomas B.; Uberseder, Beth; Clear, Kenysha Y. J.; Wilson, Adam S.; Chiba, Akiko; Tooze, Janet A.; Cook, Katherine L. Consumption of Mediterranean versus Western Diet Leads to Distinct Mammary Gland Microbiome Populations CELL REPORTS English Article BREAST-CANCER RISK; CARDIOVASCULAR RISK; INFECTIOUS MASTITIS; ACID METABOLISM; BILE-ACIDS; ESTRADIOL; MILK; PROGESTERONE; PATTERNS; MODULATE Recent identification of a mammary gland-specific microbiome led to studies investigating bacteria populations in breast cancer. Malignant breast tumors have lower Lactobacillus abundance compared with benign lesions, implicating Lactobacillus as a

negative regulator of breast cancer. Diet is a main determinant of gut microbial diversity. Whether diet affects breast microbiome populations is unknown. In a non-human primate model, we found that consumption of a Western or Mediterranean diet modulated mammary gland microbiota and metabolite profiles. Mediterranean diet consumption led to increased mammary gland Lactobacillus abundance compared with Western diet-fed monkeys. Moreover, mammary glands from Mediterranean diet-fed monkeys had higher levels of bile acid metabolites and increased bacterial-processed bioactive compounds. These data suggest that diet directly influences microbiome populations outside the intestinal tract in distal sites such as the mammary gland. Our study demonstrates that diet affects the mammary gland microbiome, establishing an alternative mechanistic pathway for breast cancer prevention.

[Shively, Carol A.; Register, Thomas C.; Appt, Susan E.; Clarkson, Thomas B.; Uberseder, Beth] Wake Forest Sch Med, Dept Pathol, Sect Comparat Med, Winston Salem, NC 27157 USA; [Clear, Kenysha Y. J.; Wilson, Adam S.; Chiba, Akiko; Cook, Katherine L.] Wake Forest Sch Med, Dept Surg, Winston Salem, NC 27157 USA; [Appt, Susan E.; Chiba, Akiko; Cook, Katherine L.] Wake Forest Sch Med, Comprehens Canc Ctr, Winston Salem, NC 27157 USA; [Tooze, Janet A.] Wake Forest Sch Med, Dept Biostat Sci, Winston Salem, NC 27157 USA; [Cook, Katherine L.] Wake Forest Sch Med, Dept Canc Biol, Winston Salem, NC 27157 USA Wake Forest University; Wake Forest University; Wake Forest University; Wake Forest University; Wake Forest University Cook, KL (corresponding author), Wake Forest Sch Med, Dept Surg, Winston Salem, NC 27157 USA.; Cook, KL (corresponding author), Wake Forest Sch Med, Comprehens Canc Ctr, Winston Salem, NC 27157 USA.; Cook, KL (corresponding author), Wake Forest Sch Med, Dept Canc Biol, Winston Salem, NC 27157 USA.

klcook@wakehealth.edu Shively, Carol/L-2921-2019; Register, Thomas/KLY-9188-2024 Register, Thomas/0000-0002-4078-0166; Cook, Katherine/0000-0001-6241-0214 Chronic Disease Research Fund; American Cancer Society Research Scholar Grant [RSG-16-204-01-NEC]; Career Catalyst Grant from the Susan G. Komen foundation [CCR18547795]; Prevent Cancer Foundation; NIH [HL-087103]; Wake Forest Baptist Comprehensive Cancer Center's NCI Cancer Center Support Grant [P30CA012197] Chronic Disease Research Fund; American Cancer Society Research Scholar Grant(American Cancer Society); Career Catalyst Grant from the Susan G. Komen foundation; Prevent Cancer Foundation; NIH(United States Department of Health & Human ServicesNational Institutes of Health (NIH) - USA); Wake Forest Baptist Comprehensive Cancer Center's NCI Cancer Center Support Grant This research was supported by the Chronic Disease Research Fund (to K.L.C.), American Cancer Society Research Scholar Grant RSG-16-204-01-NEC (to K.L.C.), Career Catalyst Grant from the Susan G. Komen foundation CCR18547795 (to K.L.C.), a grant from the Prevent Cancer Foundation (to K.L.C.), and NIH grant HL-087103 (to C.A.S.). Shared resource services were provided by the Wake Forest Baptist Comprehensive Cancer Center's NCI Cancer Center Support Grant (P30CA012197). We also acknowledge the editorial assistance of Karen Klein, MA, at the Wake Forest Clinical and Translational Science Institute (UL1 TR001420; McClain, principal investigator [PI]). 56 118 135 1 33 CELL PRESS CAMBRIDGE

50 HAMPSHIRE ST, FLOOR 5, CAMBRIDGE, MA 02139 USA 2211-1247

CELL REP Cell Reports OCT 2 2018 25 1

47 +

10.1016/j.celrep.2018.08.078
http://dx.doi.org/10.1016/j.celrep.2018.08.078
13 Cell Biology Science Citation Index Expanded (SCI-EXPANDED) Cell Biology GV4WP 30282037 gold, Green Accepted 2025-06-24 WOS:000446102400006
J Link, LB; Canchola, AJ; Bernstein, L; Clarke, CA; Stram, DO; Ursin, G; Horn-Ross, PL Link, Lilli B.; Canchola, Alison J.; Bernstein, Leslie; Clarke, Christina A.; Stram, Daniel O.; Ursin, Giske; Horn-Ross, Pamela L.
Dietary patterns and breast cancer risk in the California Teachers Study cohort AMERICAN JOURNAL OF CLINICAL NUTRITION English Article
HORMONE-THERAPY USE; ALCOHOL-CONSUMPTION; MEDITERRANEAN DIET; WOMEN Background: Evidence that diet is associated with breast cancer risk is inconsistent. Most studies have examined risks associated with specific foods and nutrients, rather than measures of overall diet. Objective: This study aimed to evaluate dietary patterns and their relation to breast cancer risk in a large cohort of women. Design: Data from 91,779 women in the California Teachers Study cohort were analyzed, including data from 4140 women with a diagnosis of invasive breast cancer made between 1995 and 2009. Five predominant dietary patterns were identified by using principal components factor analysis: a plant-based diet, high in fruit and vegetables; a high-protein, high-fat diet, high in meats, eggs, fried foods, and high-fat condiments; a high-carbohydrate diet, high in convenience foods, pasta, and bread products; an ethnic diet, high in legumes, soy-based foods, rice, and dark-green leafy vegetables; and a salad and wine diet, high in lettuce, fish, wine, low-fat salad dressing, and coffee and tea. Results: The plant-based pattern was associated with a reduction in breast cancer risk (RR: 0.85; 95% CI: 0.76, 0.95 for the highest compared with the lowest consumption quintile; P-trend = 0.003); risk reduction was greater for estrogen receptor negative progesterone receptor negative (ER PR) tumors (RR: 0.66; 95% CI: 0.48, 0.91; P-trend = 0.03). The salad and wine pattern was associated with an increased risk of estrogen receptor positive progesterone receptor positive tumors (RR: 1.29; 95% CI: 1.12, 1.49); this effect was only slightly attenuated after adjustment for alcohol consumption. Conclusion: The finding that greater consumption of a plant-based dietary pattern is associated with a reduced breast cancer risk, particularly for ER PR tumors, offers a potential avenue for prevention. [Link, Lilli B.] Columbia Univ, Mailman Sch Publ Hlth, New York, NY USA; [Canchola, Alison J.; Clarke, Christina A.; Horn-Ross, Pamela L.] Canc Prevent Inst Calif, Fremont, CA USA; [Clarke, Christina A.; Horn-Ross, Pamela L.] Stanford Univ, Dept Hlth Res & Policy, Sch Med, Stanford, CA 94305 USA; [Bernstein, Leslie] City Hope Natl Med Ctr, Div Canc Etiol, Dept Populat Sci, Beckman Res Inst, Duarte, CA USA; [Stram, Daniel O.; Ursin, Giske] Univ So Calif, Keck Sch Med, Dept Prevent Med, Los Angeles, CA 90033 USA; [Ursin, Giske] Canc Registry Norway, Oslo, Norway; [Ursin, Giske] Univ Oslo, Dept Nutr, Oslo, Norway Columbia University; Cancer Prevention Institute of California; Stanford University; City of Hope; Beckman Research Institute of City of Hope; University of Southern California; University of Oslo; University of Oslo Horn-Ross, PL (corresponding author), Canc Prevent Inst Calif, 2201 Walnut Ave,Suite 300, Fremont, CA USA. pam@cpic.org Ursin, Giske/U-6637-2017 Ursin, Giske/0000-0002-0835-9507 National Cancer

Institute [R01 CA77398, R25 CA094061]; California Breast Cancer Research Fund [97-10500]; California Department of Health Services (CDHS) [103885]; NCI's Surveillance, Epidemiology and End Results Program [N01-PC-35136, N01-PC-35139, N02-PC-15105]; CDC National Program of Cancer Registries [U55/CCR921930-02]   National Cancer Institute(United States Department of Health & Human ServicesNational Institutes of Health (NIH) - USANIH National Cancer Institute (NCI)); California Breast Cancer Research Fund; California Department of Health Services (CDHS); NCI's Surveillance, Epidemiology and End Results Program(United States Department of Health & Human ServicesNational Institutes of Health (NIH) - USANIH National Cancer Institute (NCI)); CDC National Program of Cancer Registries(United States Department of Health & Human ServicesCenters for Disease Control & Prevention - USA)
Supported in part by grants R01 CA77398 and R25 CA094061 from the National Cancer Institute and by contract 97-10500 from the California Breast Cancer Research Fund. The collection of cancer incidence data used in this study was supported by the California Department of Health Services (CDHS) as part of the statewide cancer reporting program mandated by California Health and Safety Code Section 103885; the NCI's Surveillance, Epidemiology and End Results Program under contract N01-PC-35136 awarded to the Northern California Cancer Center, contract N01-PC-35139 awarded to the University of Southern California, and contract N02-PC-15105 awarded to the Public Health Institute (PHI); and the CDC National Program of Cancer Registries under agreement U55/CCR921930-02 awarded to the PHI.   52   90
101   0   38   OXFORD UNIV PRESS   OXFORD   GREAT CLARENDON ST, OXFORD OX2 6DP, ENGLAND   0002-9165   1938-3207
AM J CLIN NUTR   Am. J. Clin. Nutr.   DEC   2013   98   6
1524   1532
10.3945/ajcn.113.061184
http://dx.doi.org/10.3945/ajcn.113.061184   9
Nutrition & Dietetics   Science Citation Index Expanded (SCI-EXPANDED)   Nutrition & Dietetics   266DG   24108781   Bronze, Green Published   2025-06-24   WOS:000328002000020
J   Venkataswamy, M; Karunakaran, RS; Islam, MS; Meriga, B
Venkataswamy, Mallepogu; Karunakaran, Reddy Sankaran; Islam, Md. Shahidul; Meriga, Balaji
Capparis zeylanica L. root extract promotes apoptosis and cell cycle arrest, inhibits epithelial-to-mesenchymal transition and triggers E-cadherin expression in breast cancer cell lines
3 BIOTECH   English   Article
Apoptosis; Breast cancer; Capparis zeylanica; Cell cycle; Cytotoxicity; EMT markers   TOCOPHEROL COMPOSITION; SPINOSA; ANTIOXIDANT; SNAIL
Capparis zeylanica L. is a climbing shrub distributed in Indian subcontinent and Mediterranean region. Almost all parts of the plant are used in folk medicine and traditional practices to treat several human ailments. The present study was aimed to investigate the role of C. zeylanica L. root extract in preventing cancerous cells growth and proliferation, as well as promoting apoptosis and cell cycle arrest in MDA-MB-231 and MCF-7 breast cancer cells. Methanolic extract of C. zeylanica L. (MECz) was prepared and characterized by LC-ESI-MS/MS analysis. In vitro cytotoxicity and anti-proliferative activity of MECz was evaluated by MTT assay, while cell viability, apoptosis and cell cycle progression by Muse Cell analyzer. Furthermore, the mRNA and protein expressions of EMT markers were assessed using qRT-PCR and

western blotting techniques, respectively. The MECz was found to be rich in phenolic compounds including chlorogenic acid, 6-gingerol, and certain triterpenes like ursolic acid etc. The apparent anti-metastasis activity of MECz was evident from IC50 value of 19.12 and 24.22 mu g/mL, respectively, on MDA-MB-231 and MCF-7 cells in MTT assay. An absolute decrease in cell viability (78.1-53.4% and 89.9-49.0%), augmented apoptosis (90.98-48.25% and 88.25-47.70%) and S phase, G(2)/M phase cell cycle arrest was found by MECz treatment on MDA-MB-231 and MCF-7 cells. The gene expression studies revealed that MECz could significantly (p < 0.001) regulate the expression of EMT markers such as snail, slug, zeb-1, twist-1, fibronectin, vimentin and E-cadherin at molecular level. These findings demonstrate that C. zeylanica L. root extract inhibits breast cancer cells growth and proliferation through regulating the expression of key EMT marker genes and proteins. Thus, MECz may be suggested as a potential anti-metastasis agent in the treatment of breast cancer.
[Venkataswamy, Mallepogu; Karunakaran, Reddy Sankaran; Meriga, Balaji] Sri Venkateswara Univ, Dept Biochem, Tirupati 517502, Andhra Pradesh, India; [Islam, Md. Shahidul] Univ KwaZulu Natal, Sch Life Sci, Dept Biochem, Westville Campus, ZA-4000 Durban, South Africa Sri Venkateswara University; University of Kwazulu Natal Meriga, B (corresponding author), Sri Venkateswara Univ, Dept Biochem, Tirupati 517502, Andhra Pradesh, India. balaji.meriga@gmail.com Meriga, Balaji/ACP-0407-2022; Islam, Md Shahidul/AAT-3881-2021; Reddy-Sankaran, Karunakaran/HTM-1959-2023 Reddy-Sankaran, Karunakaran/0000-0003-4559-0107; balaji, meriga/0000-0001-9238-5523 Indian Council of Medical Research (ICMR), New Delhi, India [45/09/2020/TRM/BMS]
Indian Council of Medical Research (ICMR), New Delhi, India(Indian Council of Medical Research (ICMR)) The author VM is highly debited to the Indian Council of Medical Research (ICMR), New Delhi, India for providing financial support in the form of Research Associate fellowship (45/09/2020/TRM/BMS).
54 6 6 0 1 SPRINGER HEIDELBERG
HEIDELBERG TIERGARTENSTRASSE 17, D-69121 HEIDELBERG, GERMANY
2190-572X 2190-5738 3 BIOTECH 3 Biotech FEB 2023
13 2 41
10.1007/s13205-023-03461-x
http://dx.doi.org/10.1007/s13205-023-03461-x 17
Biotechnology & Applied Microbiology Science Citation Index Expanded (SCI-EXPANDED) Biotechnology & Applied Microbiology 7U2WO 36643403 Green Published
2025-06-24 WOS:000911996100001
J Gnagnarella, P; Dragà, D; Misotti, AM; Sieri, S; Spaggiari, L; Cassano, E; Baldini, F; Soldati, L; Maisonneuve, P
Gnagnarella, P.; Draga, D.; Misotti, A. M.; Sieri, S.; Spaggiari, L.; Cassano, E.; Baldini, F.; Soldati, L.; Maisonneuve, P. Validation of a short questionnaire to record adherence to the Mediterranean diet: An Italian experience
NUTRITION METABOLISM AND CARDIOVASCULAR DISEASES
English Article
Mediterranean diet; Validation study; Questionnaire FOOD PATTERN; DISEASE; RISK; REPRODUCIBILITY; PREVENTION; VALIDITY; INDEXES; QUALITY; OBESITY; HABITS Background and aims: A greater adherence to the Mediterranean diet has been associated with a reduced risk of major chronic diseases and cancer. The aim of the study was to assess the validity of a new short self-administered

15-item questionnaire (QueMD) to measure adherence to the Mediterranean diet in Italy. Methods and results: Four-hundred and eighty three participants to cancer-screening programmes at the European Institute of Oncology, Milan (Italy) were invited to join this study. Those interested compiled the QueMD and a validated Food Frequency Questionnaire (FFQ) reporting their usual food consumption during the previous six months. We derived the alternate Mediterranean score (aMED) from both questionnaires with values ranging from 0 (minimal adherence) to 9 (maximal adherence). Complete dietary data were available for 343 individuals (participation rates 71.0%). Spearman correlation coefficient between the responses to the 15 questions of the QueMD and corresponding food intake derived from the FFQ ranged from 0.15 to 0.84. A moderate correlation was found between the aMED scores calculated from the QueMD and the FFQ (intraclass correlation coefficient 0.50; 95% CI, 0.42-0.58), while agreement between the two instruments was only poor to fair for 7 of the 9 single items composing the aMED score, with values ranging from 53.0% for wholegrain products to 79.5% for fruits. Conclusion: This new self-administered 15-item questionnaire could be a useful tool to assess adherence to the Mediterranean diet in the Italian population. (C) 2018 The Italian Society of Diabetology, the Italian Society for the Study of Atherosclerosis, the Italian Society of Human Nutrition, and the Department of Clinical Medicine and Surgery, Federico II University. Published by Elsevier B.V. All rights reserved. [Gnagnarella, P.; Draga, D.; Maisonneuve, P.] European Inst Oncol, Div Epidemiol & Biostat, Milan, Italy; [Misotti, A. M.] ASST Melegnano & Martesana, UOSD Dietol & Nutr Clin, Vizzolo Predabiss, Italy; [Sieri, S.] Fdn IRCSS Ist Nazl Tumori, Dept Res, Epidemiol & Prevent Unit, Milan, Italy; [Spaggiari, L.] European Inst Oncol, Dept Thorac Surg, Milan, Italy; [Spaggiari, L.] Univ Milan, Dept Oncol & Ematooncol, Milan, Italy; [Cassano, E.] European Inst Oncol, Breast Imaging Div, Milan, Italy; [Baldini, F.] European Inst Oncol, Div Melanoma Sarcoma & Rare Canc, Milan, Italy; [Soldati, L.] Univ Milan, Dept Hlth Sci, Milan, Italy IRCCS European Institute of Oncology (IEO); Fondazione IRCCS Istituto Nazionale Tumori Milan; IRCCS European Institute of Oncology (IEO); University of Milan; IRCCS European Institute of Oncology (IEO); IRCCS European Institute of Oncology (IEO); University of Milan Gnagnarella, P (corresponding author), Ist Europeo Oncolo, Div Epidemiol & Biostat, Via Ripamonti 435, I-20141 Milan, Italy. patrizia.gnagnarella@ieo.it Spaggiari, Lorenzo/G-7915-2012; Misotti, Alessandro/AAD-2886-2022; gnagnarella, patrizia/AAA-9331-2019; Sieri, Sabina/K-4667-2016; Maisonneuve, Patrick/U-9789-2018 Gnagnarella, Patrizia/0000-0002-0560-4706; Sieri, Sabina/0000-0001-5201-172X; Misotti, Alessandro/0000-0002-3215-179X; Maisonneuve, Patrick/0000-0002-5309-4704; Spaggiari, Lorenzo/0000-0002-1068-3541
41 39 39 2 9 ELSEVIER SCI LTD OXFORD
THE BOULEVARD, LANGFORD LANE, KIDLINGTON, OXFORD OX5 1GB, OXON, ENGLAND 0939-4753 1590-3729 NUTR METAB CARDIOVAS
Nutr. Metab. Carbiovasc. Dis. NOV 2018 28 11
1140 1147
10.1016/j.numecd.2018.06.006
http://dx.doi.org/10.1016/j.numecd.2018.06.006
8 Cardiac & Cardiovascular Systems; Endocrinology & Metabolism; Nutrition & Dietetics Science Citation Index Expanded (SCI-EXPANDED) Cardiovascular System & Cardiology; Endocrinology

& Metabolism; Nutrition & Dietetics GZ6RT 30077491
2025-06-24 WOS:000449570400008
J Mourouti, N; Michou, M; Lionis, C; Kalagia, P; Ioannidis, AG; Kaloidas, M; Costarelli, V Mourouti, Niki; Michou, Maria; Lionis, Christos; Kalagia, Panagiota; Ioannidis, Angelos G.; Kaloidas, Michalis; Costarelli, Vassiliki
An educational intervention to improve health and nutrition literacy in hypertensive patients in Greece JOURNAL OF EDUCATION AND HEALTH PROMOTION English Article
Eating behavior; educational program; Greece; health literacy; hypertension nutrition literacy GLOBAL MEASURE; VALIDATION; BEHAVIOR BACKGROUND: Suboptimal control of blood pressure (BP) is a public health challenge in Greece. This educational intervention attempts to improve health literacy (HL) and nutrition literacy (NL) in primary healthcare (PHC) patients with hypertension (HTN). MATERIALS AND METHODS: This was a proof-of-concept (PoC) educational intervention. Twenty-four patients with HTN and low or medium levels of HL agreed to participate in this 6-week educational intervention. The program was delivered by a general practitioner (GP) in group A and by a qualified nurse in group B. Group C was the control group. The levels of HL of NL, adherence to the Mediterranean diet, and perceived stress were assessed using the European Health Literacy Survey Questionnaire 16 (HLS_EU_Q16) and part B (reading a medication label) of the High Blood Pressure-Health Literacy Scale (HBP-HLS), the Nutrition Literacy Scale-Greek (NLS-Gr), the Mediterranean Diet Adherence Screener (MEDAS), and the Perceived Stress Scale (PSS-14), respectively, together with certain socioeconomic, dietary, and lifestyle characteristics. The Kruskal-Wallis and Wilcoxon nonparametric, the Chi-square, and the McNemar-Bowker tests were used. The analysis was done with the STATA software, version 14 (MP & Associates, Sparta, Greece). RESULTS: No statistically significant differences were found in the total score of HL and NL before and after the intervention; however, the results indicate a slight improvement in HL and NL levels in the two intervention groups, together with small improvements in knowledge and behaviors related to HTN and dietary salt. A trend of improvement in the MEDAS levels in the GP's group and the HBP-HLS levels in the nurse's group was also reported. CONCLUSION: A slight improvement in HL and NL levels in the two intervention groups has been reported, and the changes, however, were not statistically significant. [Mourouti, Niki; Michou, Maria; Costarelli, Vassiliki] Harokopio Univ, Dept Econ & Sustainable Dev, Human Ecol Lab, Athens, Greece; [Lionis, Christos] Univ Crete, Sch Med, Clin Social & Family Med, Iraklion 71003, Greece; [Kalagia, Panagiota; Ioannidis, Angelos G.; Kaloidas, Michalis] Hlth Ctr, Athens, Greece Harokopio University Athens; University of Crete Costarelli, V (corresponding author), Harokopio Univ Athens, Dept Econ & Sustainable Dev, 70 El Venizelou Ave, Athens 17671, Greece. costarv@hua.gr
Costarelli, Vassiliki/AAM-1210-2020; Lionis, Christos/MBV-1499-2025 European Union (European Social Fund-ESF) [5049028] European Union (European Social Fund-ESF)(European Union (EU)European Social Fund (ESF)) This research was co-financed by Greece and the European Union (European Social Fund-ESF) through the Operational Programme "Human Resources Development, Education and Lifelong Learning 2014-2020" in the context of the project "Development and Pilot Implementation of an

Educational Intervention Program for Health Literacy and Nutrition Literacy in Hypertension Patients: A Randomized Control Trial" (MIS: 5049028). 26 1 1 3 10 WOLTERS KLUWER MEDKNOW PUBLICATIONS MUMBAI WOLTERS KLUWER INDIA PVT LTD , A-202, 2ND FLR, QUBE, C T S NO 1498A-2 VILLAGE MAROL, ANDHERI EAST, MUMBAI, Maharashtra, INDIA 2277-9531 2319-6440 J EDUC HEALTH PROMOT J. Educ. Health Promot. JAN-DEC 2023 12 1
10.4103/jehp.jehp_14_23
http://dx.doi.org/10.4103/jehp.jehp_14_23 9
Education, Scientific Disciplines; Public, Environmental & Occupational Health Emerging Sources Citation Index (ESCI)
Education & Educational Research; Public, Environmental & Occupational Health T7KH4 37727406 gold, Green Published
2025-06-24 WOS:001079730200012
J She, QB; Gruvberger-Saal, SK; Maurer, M; Chen, YL; Jumppanen, M; Su, T; Dendy, M; Lau, YKI; Memeo, L; Horlings, HM; van de Vijver, MJ; Isola, J; Hibshoosh, H; Rosen, N; Parsons, R; Saal, LH She, Qing-Bai; Gruvberger-Saal, Sofia K.; Maurer, Matthew; Chen, Yilun; Jumppanen, Mervi; Su, Tao; Dendy, Meaghan; Lau, Ying-Ka Ingar; Memeo, Lorenzo; Horlings, Hugo M.; van de Vijver, Marc J.; Isola, Jorma; Hibshoosh, Hanina; Rosen, Neal; Parsons, Ramon; Saal, Lao H.
Integrated molecular pathway analysis informs a synergistic combination therapy targeting PTEN/PI3K and EGFR pathways for basal-like breast cancer BMC CANCER English
Article Basal-like; Breast cancer; EGFR; PTEN; Combination therapy GENE-EXPRESSION SIGNATURE; GROWTH-FACTOR RECEPTOR; PIK3CA MUTATIONS; TUMOR-CELLS; PHOSPHATIDYLINOSITOL 3-KINASE; TRASTUZUMAB RESISTANCE; KINASE INHIBITORS; IN-SITU; PTEN; ACTIVATION Background: The basal-like breast cancer (BLBC) subtype is characterized by positive staining for basal mammary epithelial cytokeratin markers, lack of hormone receptor and HER2 expression, and poor prognosis with currently no approved molecularly-targeted therapies. The oncogenic signaling pathways driving basal-like tumorigenesis are not fully elucidated. Methods: One hundred sixteen unselected breast tumors were subjected to integrated analysis of phosphoinositide 3-kinase (PI3K) pathway related molecular aberrations by immunohistochemistry, mutation analysis, and gene expression profiling. Incidence and relationships between molecular biomarkers were characterized. Findings for select biomarkers were validated in an independent series. Synergistic cell killing in vitro and in vivo tumor therapy was investigated in breast cancer cell lines and mouse xenograft models, respectively. Results: Sixty-four % of cases had an oncogenic alteration to PIK3CA, PTEN, or INPP4B; when including upstream kinases HER2 and EGFR, 75 % of cases had one or more aberration including 97 % of estrogen receptor (ER)-negative tumors. PTEN-loss was significantly associated to stathmin and EGFR overexpression, positivity for the BLBC markers cytokeratin 5/14, and the BLBC molecular subtype by gene expression profiling, informing a potential therapeutic combination targeting these pathways in BLBC. Combination treatment of BLBC cell lines with the EGFR-inhibitor gefitinib plus the PI3K pathway inhibitor LY294002 was synergistic, and correspondingly, in an in vivo BLBC xenograft mouse model, gefitinib plus PI3K-inhibitor PWT-458 was more effective than either monotherapy and caused tumor regression. Conclusions: Our

study emphasizes the importance of PI3K/PTEN pathway activity in ER-negative and basal-like breast cancer and supports the future clinical evaluation of combining EGFR and PI3K pathway inhibitors for the treatment of BLBC. [She, Qing-Bai; Rosen, Neal] Mem Sloan Kettering Canc Ctr, Program Mol Pharmacol & Chem, 1275 York Ave, New York, NY 10021 USA; [She, Qing-Bai; Rosen, Neal] Mem Sloan Kettering Canc Ctr, Dept Med, 1275 York Ave, New York, NY 10021 USA; [She, Qing-Bai] Univ Kentucky, Coll Med, Markey Canc Ctr, Lexington, KY USA; [She, Qing-Bai] Univ Kentucky, Coll Med, Dept Pharmacol & Nutr Sci, Lexington, KY USA; [Gruvberger-Saal, Sofia K.; Chen, Yilun; Saal, Lao H.] Lund Univ, Div Oncol & Pathol, Clin Sci, Lund, Sweden; [Gruvberger-Saal, Sofia K.; Dendy, Meaghan; Parsons, Ramon; Saal, Lao H.] Columbia Univ, Med Ctr, Inst Canc Genet, New York, NY USA; [Maurer, Matthew; Su, Tao; Lau, Ying-Ka Ingar; Hibshoosh, Hanina] Columbia Univ, Herbert Irving Comprehens Canc Ctr, New York, NY USA; [Maurer, Matthew; Parsons, Ramon] Columbia Univ, Dept Med, New York, NY USA; [Jumppanen, Mervi] Seinajoki Cent Hosp, Dept Pathol, Seinajoki, Finland; [Memeo, Lorenzo] Mediterranean Inst Oncol, Dept Expt Oncol, Catania, Italy; [Horlings, Hugo M.] Netherlands Canc Inst, Dept Pathol, Amsterdam, Netherlands; [van de Vijver, Marc J.] Acad Med Ctr, Dept Pathol, Amsterdam, Netherlands; [Isola, Jorma] Univ Tampere, Inst Med Technol, Tampere, Finland; [Hibshoosh, Hanina; Parsons, Ramon] Columbia Univ, Dept Pathol, New York, NY USA; [Parsons, Ramon] Icahn Sch Med Mt Sinai, Dept Oncol Sci, New York, NY 10029 USA; [Parsons, Ramon] Icahn Sch Med Mt Sinai, Tisch Canc Inst, New York, NY 10029 USA; [Saal, Lao H.] Lund Univ, Div Oncol & Pathol, Translat Oncogen Unit, Ctr Canc, Medicon Village 404-B2, SE-22381 Lund, Sweden   Memorial Sloan Kettering Cancer Center; Memorial Sloan Kettering Cancer Center; University of Kentucky; University of Kentucky; Lund University; Columbia University; Columbia University; Columbia University; Seinajoki Central Hospital; Mediterranean Institute of Oncology; Netherlands Cancer Institute; University of Amsterdam; Academic Medical Center Amsterdam; Tampere University; Columbia University; Icahn School of Medicine at Mount Sinai; Icahn School of Medicine at Mount Sinai; Lund University Saal, LH (corresponding author), Lund Univ, Div Oncol & Pathol, Translat Oncogen Unit, Ctr Canc, Medicon Village 404-B2, SE-22381 Lund, Sweden. lao.saal@med.lu.se   chen, yilun/JYP-4798-2024; Rosen, Neal/ABF-2677-2020; Su, Tao/T-4411-2019; Parsons, Ramon/KFT-2922-2024; Saal, Lao/A-2919-2011   van de Vijver, Marc/0000-0002-0385-4787; Memeo, Lorenzo/0000-0003-4251-7203; Saal, Lao/0000-0002-0815-1896; She, Qing-Bai/0000-0002-7207-0599; Parsons, Ramon/0000-0002-6656-3514   U.S. National Institutes of Health [5 T32 GM07367-29, R01 CA175105, P01 CA097403, R01 CA082783, P01 CA094060]; Stand Up To Cancer Dream Team; Avon Foundation; Swedish Cancer Society; Swedish Research Council; Governmental Funding of Clinical Research within National Health Service; Mrs. Berta Kamprad Foundation; Skane University Hospital Foundation; King Gustav V's Jubilee Foundation; Krapperup Foundation; Gunnar Nilsson Cancer Foundation; Crafoord Foundation

U.S. National Institutes of Health(United States Department of Health & Human ServicesNational Institutes of Health (NIH) - USA); Stand Up To Cancer Dream Team; Avon Foundation; Swedish Cancer Society(Swedish Cancer Society); Swedish Research Council(Swedish Research Council); Governmental Funding of Clinical Research within National Health Service; Mrs. Berta Kamprad Foundation; Skane University Hospital Foundation; King

Gustav V's Jubilee Foundation; Krapperup Foundation; Gunnar Nilsson Cancer Foundation; Crafoord Foundation This study was funded by the U.S. National Institutes of Health (Medical Scientist Training Grant 5 T32 GM07367-29 [LHS], R01 CA175105 [Q-BS], P01 CA097403 and R01 CA082783 [RP], P01 CA094060 [NR]), Stand Up To Cancer Dream Team (MM, NR, RP), the Avon Foundation (HH and RP), and the Swedish Cancer Society, Swedish Research Council, Governmental Funding of Clinical Research within National Health Service, Mrs. Berta Kamprad Foundation, Skane University Hospital Foundation, King Gustav V's Jubilee Foundation, Krapperup Foundation, Gunnar Nilsson Cancer Foundation, and Crafoord Foundation (all to LHS). The funders had no role in the study design, data gathering, data analysis, data interpretation, decision to publish, or writing of the report. 56 22 23 0 9 BIOMED CENTRAL LTD LONDON 236 GRAYS INN RD, FLOOR 6, LONDON WC1X 8HL, ENGLAND 1471-2407 BMC CANCER BMC Cancer AUG 2 2016 16 587 10.1186/s12885-016-2609-2 http://dx.doi.org/10.1186/s12885-016-2609-2 16 Oncology Science Citation Index Expanded (SCI-EXPANDED) Oncology DT1BL 27484095 Green Published, gold 2025-06-24 WOS:000381216600021

J Brinbaum, Y Brinbaum, Yael

Incorporation of Immigrants and Second Generations into the French Labour Market: Changes between Generations and the Role of Human Capital and Origins SOCIAL INCLUSION English Article discrimination; employment; France; human capital; immigrants; labour market; returns to higher education; second generation; skilled workers SEGMENTED ASSIMILATION; INEQUALITIES This article analyses the labour market incorporation of migrants and second-generation minorities in France. Using the 2013-2017 French Labour Surveys and the 2014 adhoc module, we focus on labour market outcomes-activity, employment, occupation and subjective overqualification-and measure the gaps between ethnic minorities and the majority group by origins, generation and by gender. In order to elucidate the mechanisms behind these gaps and explain ethnic disadvantages for immigrants, we take into account different factors, such as education, and factors linked to migration-duration of stay in France, language skills, foreign qualifications, nationality-with additional controls for family, socioeconomic and contextual characteristics. We also investigate the returns to higher education among second-generation minority members compared to the majority population. We show large differences by country of origins, generation and gender. Across generations, most minority members have made clear progress in terms of access to employment and skilled jobs, but ethnic penalties remain for the descendants of North-Africa, Sub-Saharan Africa and Turkey. In contrast, Asian second-generation men and women encounter slight advantages in attaining highly-skilled positions. Controlling for tertiary degrees even increases the gap with majority members mostly in access to highly-skills jobs. [Brinbaum, Yael] CNAM, Lab Interdisciplinaire Sociol Econ LISE UMR CNRS, F-75003 Paris, France; [Brinbaum, Yael] CNAM, CEET, F-75003 Paris, France heSam Universite; Conservatoire National Arts & Metiers (CNAM); heSam Universite; Conservatoire National Arts & Metiers (CNAM) Brinbaum, Y (corresponding author), CNAM, Lab Interdisciplinaire Sociol Econ LISE UMR CNRS, F-75003 Paris,

France.; Brinbaum, Y (corresponding author), CNAM, CEET, F-75003 Paris, France. yael.brinbaum@lecnam.net European Commission [H2020 649255] European Commission(European Union (EU)European Commission Joint Research Centre) This thematic issue has been supported by funding from the European Commission (Grant number H2020 649255). 39 6 6 2 24
COGITATIO PRESS LISBON RUA FIALHO ALMEIDA 14, 2 ESQ, LISBON, 1070-129, PORTUGAL 2183-2803 SOC INCL Soc. Incl. 2018 6 3 104 118
10.17645/si.v6i3.1453
http://dx.doi.org/10.17645/si.v6i3.1453 15
Social Issues; Social Sciences, Interdisciplinary Social Science Citation Index (SSCI) Social Issues; Social Sciences - Other Topics GO7BQ Green Published, gold
2025-06-24 WOS:000440211000007
J Armenta-Guirado, BI; Mérida-Ortega, A; López-Carrillo, L; Denova-Gutiérrez, E Armenta-Guirado, Brianda Ioanna; Merida-Ortega, Angel; Lopez-Carrillo, Lizbeth; Denova-Gutierrez, Edgar Diet quality indices are associated with breast cancer by molecular subtypes in Mexican women
EUROPEAN JOURNAL OF NUTRITION English
Article Diet; Quality indices; Breast cancer; Molecular subtypes; Mexico MEDITERRANEAN DIET; RISK; ADHERENCE; CONSUMPTION; PATTERNS; ENERGY; COHORT
BackgroundInconclusive epidemiological evidence suggests that diet quality indices may influence breast cancer (BC) risk; however, the evidence does not consider the molecular expression of this cancer.PurposeWe aimed to evaluate if diet quality is related to molecular subtypes of BC, in women residing in Northern Mexico.MethodsThis is a secondary analysis of 1,045 incident cases and 1,030 population controls from a previous case-control study, conducted between 2007 and 2011 in Northern Mexico. Information about the expression of estrogen receptor (ER), progesterone receptor (PR), and human epidermal growth factor 2 (HER2) was obtained from medical records to classify BC as luminal (ER + and/or PR+/HER2-), HER2+ (ER+/-and/or PR+/-/HER2+), or triple-negative (TN) (ER- and PR-/HER2-) cases. Food consumption was assessed with a semi-quantitative food frequency questionnaire. Diet quality was evaluated using the Mexican Diet Quality Index (MxDQI) and the Mexican Alternative Healthy Eating Index (MxAHEI). We used unconditional logistic regression models to estimate the association between Mexican diet quality indices and BC molecular subtypes.ResultsThe MxDQI was related to lower odds of BC (ORT3vsT1=0.24; 95%CI: 0.18, 0.31). Similarly, MxAHEI was negatively associated with BC (ORT3vsT1=0.43; 95%CI: 0.34, 0.54). The associations of both indices remained significant in the ER + and ER- tumors, and in the BC luminal and HER2 + molecular subtypes, except in the TN molecular subtype for MxAHEI, which was not statistically significant.ConclusionsOur findings showed that MxDQI and MxAHEI were negatively associated with BC risk regardless of its molecular subtype. [Armenta-Guirado, Brianda Ioanna] Univ Sonora, Dept Hlth Sci, Blvd Bordo Nuevo S-N,Blvd Antiguo Ejido, Cajeme 85010, Sonora, Mexico; [Merida-Ortega, Angel; Lopez-Carrillo, Lizbeth] Natl Inst Publ Hlth, Ctr Populat Hlth Res, Ave Univ 655,Santa Maria Ahuacatitlan, Cuernavaca 62100, Mexico; [Denova-Gutierrez, Edgar] Natl Inst Publ Hlth, Ctr Nutr & Hlth Res, Ave Univ 655,Col Santa Maria Ahuacatitlan, Cuernavaca, Mexico Universidad de Sonora; Instituto Nacional de Salud

Publica; Instituto Nacional de Salud Publica Denova-Gutiérrez, E (corresponding author), Natl Inst Publ Hlth, Ctr Nutr & Hlth Res, Ave Univ 655,Col Santa Maria Ahuacatitlan, Cuernavaca, Mexico.
brianda.armenta@unison.mx; angel.merida@insp.edu.mx; lizbeth@insp.mx; edgar.denova@insp.mx Edgar, Denova-Gutiérrez/HLW-7955-2023; Armenta-Guirado, Brianda Ioanna/AAP-9856-2021; DENOVA-GUTIERREZ, EDGAR/E-4600-2011 Armenta-Guirado, Brianda Ioanna/0000-0003-2373-4501; DENOVA-GUTIERREZ, EDGAR/0000-0001-9671-9682 National Council for Science and Technology (CONACyT by its acronym in Spanish)-Sector Fund for Research in Health and Social Security (FOSISS) [SALUD- 2005-C02-14373]
National Council for Science and Technology (CONACyT by its acronym in Spanish)-Sector Fund for Research in Health and Social Security (FOSISS) This work was supported by the National Council for Science and Technology (CONACyT by its acronym in Spanish)-Sector Fund for Research in Health and Social Security (FOSISS) (SALUD- 2005-C02-14373). 76 1 1 0 0
SPRINGER HEIDELBERG HEIDELBERG TIERGARTENSTRASSE 17, D-69121 HEIDELBERG, GERMANY 1436-6207 1436-6215 EUR J NUTR Eur. J. Nutr. DEC 2024 63 8
3223 3233 10.1007/s00394-024-03502-y
http://dx.doi.org/10.1007/s00394-024-03502-y SEP 2024
11 Nutrition & Dietetics Science Citation Index Expanded (SCI-EXPANDED) Nutrition & Dietetics K7E7Q 39325098
2025-06-24 WOS:001324541900003
J Couto, E; Boffetta, P; Lagiou, P; Ferrari, P; Buckland, G; Overvad, K; Dahm, CC; Tjonneland, A; Olsen, A; Clavel-Chapelon, F; Boutron-Ruault, MC; Cottet, V; Trichopoulos, D; Naska, A; Benetou, V; Kaaks, R; Rohrmann, S; Boeing, H; von Ruesten, A; Panico, S; Pala, V; Vineis, P; Palli, D; Tumino, R; May, A; Peeters, PH; Bueno-de-Mesquita, HB; Büchner, FL; Lund, E; Skeie, G; Engeset, D; Gonzalez, CA; Navarro, C; Rodríguez, L; Sánchez, MJ; Amiano, P; Barricarte, A; Hallmans, G; Johansson, I; Manjer, J; Wirfärt, E; Allen, NE; Crowe, F; Khaw, KT; Wareham, N; Moskal, A; Slimani, N; Jenab, M; Romaguera, D; Mouw, T; Norat, T; Riboli, E; Trichopoulou, A Couto, E.; Boffetta, P.; Lagiou, P.; Ferrari, P.; Buckland, G.; Overvad, K.; Dahm, C. C.; Tjonneland, A.; Olsen, A.; Clavel-Chapelon, F.; Boutron-Ruault, M-C; Cottet, V.; Trichopoulos, D.; Naska, A.; Benetou, V.; Kaaks, R.; Rohrmann, S.; Boeing, H.; von Ruesten, A.; Panico, S.; Pala, V.; Vineis, P.; Palli, D.; Tumino, R.; May, A.; Peeters, P. H.; Bueno-de-Mesquita, H. B.; Buchner, F. L.; Lund, E.; Skeie, G.; Engeset, D.; Gonzalez, C. A.; Navarro, C.; Rodriguez, L.; Sanchez, M-J; Amiano, P.; Barricarte, A.; Hallmans, G.; Johansson, I.; Manjer, J.; Wirfart, E.; Allen, N. E.; Crowe, F.; Khaw, K-T; Wareham, N.; Moskal, A.; Slimani, N.; Jenab, M.; Romaguera, D.; Mouw, T.; Norat, T.; Riboli, E.; Trichopoulou, A.
Mediterranean dietary pattern and cancer risk in the EPIC cohort BRITISH JOURNAL OF CANCER English
Article Mediterranean diet; dietary patterns; cancer risk; epidemiology BREAST-CANCER; ADHERENCE; MORTALITY; SURVIVAL; POPULATION; CONFORMITY; ALCOHOL
BACKGROUND: Although several studies have investigated the association of the Mediterranean diet with overall mortality or risk of specific cancers, data on overall cancer risk are sparse. METHODS: We examined the association between adherence to Mediterranean dietary pattern and overall cancer risk using data from the European Prospective Investigation Into Cancer and

nutrition, a multi-centre prospective cohort study including 142 605 men and 335 873. Adherence to Mediterranean diet was examined using a score (range: 0-9) considering the combined intake of fruits and nuts, vegetables, legumes, cereals, lipids, fish, dairy products, meat products, and alcohol. Association with cancer incidence was assessed through Cox regression modelling, controlling for potential confounders. RESULTS: In all, 9669 incident cancers in men and 21 062 in women were identified. A lower overall cancer risk was found among individuals with greater adherence to Mediterranean diet (hazard ratio = 0.96, 95% CI 0.95-0.98) for a two-point increment of the Mediterranean diet score. The apparent inverse association was stronger for smoking-related cancers than for cancers not known to be related to tobacco (P (heterogeneity) = 0.008). In all, 4.7% of cancers among men and 2.4% in women would be avoided in this population if study subjects had a greater adherence to Mediterranean dietary pattern. CONCLUSION: Greater adherence to a Mediterranean dietary pattern could reduce overall cancer risk. British Journal of Cancer (2011) 104, 1493-1499. doi:10.1038/bjc.2011.106 www.bjcancer.com Published online 5 April 2011 (C) 2011 Cancer Research UK

[Couto, E.; Boffetta, P.; Moskal, A.; Slimani, N.; Jenab, M.] Int Agcy Res Canc, F-69372 Lyon, France; [Couto, E.] Univ Oslo, Dept Nutr, Oslo, Norway; [Boffetta, P.] Mt Sinai Sch Med, Tisch Canc Inst, New York, NY 10029 USA; [Boffetta, P.; Trichopoulos, D.; Trichopoulou, A.] Hellen Hlth Fdn, Athens, Greece; [Lagiou, P.; Naska, A.; Benetou, V.; Trichopoulou, A.] Univ Athens, Sch Med, Dept Hyg Epidemiol & med Stat, WHO Collaborating Ctr Food & Nutr Policies, GR-11527 Athens, Greece; [Ferrari, P.] European Food Safety, Data Collect & Exposure Unit, Parma, Italy; [Buckland, G.; Gonzalez, C. A.] Catalan Inst Oncol IDIBELL, Canc Epidemiol Res Programme, Unit Nutr Environm & Canc, Barcelona, Spain; [Overvad, K.] Aarhus Univ, Sch Publ Hlth, Dept Epidemiol, Aarhus, Denmark; [Dahm, C. C.] Aarhus Univ Hosp, Dept Clin Epidemiol, Aalborg, Denmark; [Tjonneland, A.; Olsen, A.] Danish Canc Soc, Inst Canc Epidemiol, Dept Diet Canc & Hlth, Copenhagen, Denmark; [Clavel-Chapelon, F.; Boutron-Ruault, M-C; Cottet, V.] Inst Gustave Roussy, INSERM, U1018, Ctr Res Epidemiol & Populat Hlth, F-94805 Villejuif, France; [Clavel-Chapelon, F.; Boutron-Ruault, M-C; Cottet, V.] Paris S Univ, UMRS 1018, F-94805 Villejuif, France; [Trichopoulos, D.] Harvard Univ, Sch Publ Hlth, Dept Epidemiol, Boston, MA 02115 USA; [Trichopoulos, D.] Acad Athens, Bur Epidemiol Res, Athens, Greece; [Kaaks, R.; Rohrmann, S.] German Canc Res Ctr, Div Canc Epidemiol, D-6900 Heidelberg, Germany; [Boeing, H.; von Ruesten, A.] German Inst Human Nutr, Dept Epidemiol, Potsdam, Germany; [Panico, S.] Univ Naples Federico II, Dept Clin & Expt Med, Naples, Italy; [Pala, V.] Fdn IRCSS Ist Nazl Tumori, Nutr Epidemiol Unit, Milan, Italy; [Vineis, P.; Romaguera, D.; Mouw, T.; Norat, T.; Riboli, E.] Univ London Imperial Coll Sci Technol & Med, Sch Publ Hlth, Dept Epidemiol & Biostat, London, England; [Vineis, P.] Inst Sci Interchange Fdn, Dept Epidemiol & Life Sci, Turin, Italy; [Palli, D.] Canc Res & Prevent Inst ISPO, Mol & Nutr Epidemiol Unit, Florence, Italy; [Tumino, R.] ASP 7, Canc Registry, Ragusa, Italy; [Tumino, R.] ASP 7, Histopathol Unit, Ragusa, Italy; [May, A.; Peeters, P. H.] Univ Med Ctr, Julius Ctr Hlth Sci & Primary Care, Utrecht, Netherlands; [Bueno-de-Mesquita, H. B.; Buchner, F. L.] Natl Inst Publ Hlth & Environm RIVM, Bilthoven, Netherlands; [Bueno-de-Mesquita, H. B.] Univ Med Ctr Utrecht, Dept Gastroenterol & Hepatol, Utrecht,

Netherlands; [Buchner, F. L.] Radboud Univ Nijmegen, Med Ctr, Dept Epidemiol Biostat & HTA, NL-6525 ED Nijmegen, Netherlands; [Lund, E.; Skeie, G.; Engeset, D.] Univ Tromso, Dept Community Med, Tromso, Norway; [Navarro, C.; Sanchez, M-J; Amiano, P.; Barricarte, A.] CIBER Epidemiol & Salud Publ, Consortium Biomed Res Epidemiol & Publ Hlth, Barcelona, Spain; [Navarro, C.] Murcia Reg Hlth Council, Dept Epidemiol, Murcia, Spain; [Rodriguez, L.] Hlth & Hlth Care Serv Council, Publ Hlth & Participat Directorate, Asturias, Spain; [Sanchez, M-J] Andalusian Sch Publ Hlth, Granada Canc Registry, Granada, Spain; [Amiano, P.] Basque Govt, Publ Hlth Div Gipuzkoa, San Sebastian, Spain; [Barricarte, A.] Publ Hlth Inst Navarra, Serv Epidemiol Prevent & Hlth Promot, Pamplona, Spain; [Hallmans, G.] Umea Univ, Dept Publ Hlth & Clin Med, Umea, Sweden; [Johansson, I.] Umea Univ, Dept Odontol, Umea, Sweden; [Manjer, J.] Lund Univ, Skane Univ Hosp, Dept Surg, Malmo, Sweden; [Wirfart, E.] Lund Univ, Dept Clin Sci Malmo Nutr Epidemiol, Malmo, Sweden; [Allen, N. E.; Crowe, F.] Univ Oxford, Nuffield Dept Clin Med, Canc Epidemiol Unit, Oxford, England; [Khaw, K-T; Wareham, N.] Univ Cambridge, Dept Publ Hlth & Primary Care, Cambridge, England   World Health Organization; International Agency for Research on Cancer (IARC); University of Oslo; Icahn School of Medicine at Mount Sinai; Athens Medical School; National & Kapodistrian University of Athens; World Health Organization; European Food Safety Authority; Institut Catala d'Oncologia; Institut d'Investigacio Biomedica de Bellvitge (IDIBELL); Aarhus University; Aarhus University; Danish Cancer Society; Institut National de la Sante et de la Recherche Medicale (Inserm); Universite Paris Saclay; UNICANCER; Gustave Roussy; Universite Paris Saclay; Institut National de la Sante et de la Recherche Medicale (Inserm); Harvard University; Harvard T.H. Chan School of Public Health; Academy of Athens; Helmholtz Association; German Cancer Research Center (DKFZ); Leibniz Association; Deutsches Institut fur Ernahrungsforschung Potsdam-Rehbrucke (DIfE); University of Naples Federico II; Fondazione IRCCS Istituto Nazionale Tumori Milan; Imperial College London; ISPRO Istituto per lo studio, la prevenzione e la rete oncologica; Utrecht University; Utrecht University Medical Center; Netherlands National Institute for Public Health & the Environment; Utrecht University; Utrecht University Medical Center; Radboud University Nijmegen; UiT The Arctic University of Tromso; CIBER - Centro de Investigacion Biomedica en Red; CIBERESP; Murcia Regional Health Council; Escuela Andaluza de Salud Publica; Basque Government; Public Health Institute of Navarra; Umea University; Umea University; Lund University; Skane University Hospital; Lund University; University of Oxford; University of Cambridge

Boffetta, P (corresponding author), Int Agcy Res Canc, 150 Cours Albert Thomas, F-69372 Lyon, France.

paolo.boffetta@mssm.edu   Tjonneland, Anne/AGU-0320-2022; TRICHOPOULOU, ANTONIA/ABF-8727-2021; Jenab, Mehdi/L-2515-2019; Engeset, Dagrun/AFW-5378-2022; Boffetta, Paolo/AAI-7767-2021; Romaguera, Dora/AAB-2852-2020; Panico, Salvatore/K-6506-2016; Benetou, Vassiliki/H-7977-2019; Clavel-Chapelon, Francoise/G-6733-2014; Rohrmann, Sabine/D-2113-2012; Boutron-Ruault, Marie-Christine/H-3936-2014; Sánchez, María/HOC-7747-2023; May, Anne/G-9183-2011; Khaw, Kay-Tee/AAZ-3209-2021; Cottet, Vanessa/ABE-3236-2020; Riboli, Elio/A-4357-2009; Trichopoulos, Dimitrios/G-6825-2012; Gonzalez, Carlos A/O-4651-2014; Pala, Valeria/K-4738-2016; Dahm, Christina/G-9787-2014; SANCHEZ-PEREZ, MARIA JOSE/D-1087-2011

Gonzalez, Carlos A/0000-0003-2822-9715; Pala, Valeria/0000-0001-5438-970X; Naska, Androniki/0000-0002-1610-1813; Tjonneland, Anne/0000-0003-4385-2097; Overvad, Kim/0000-0001-6429-7921; Jenab, Mazda/0000-0002-0573-1852; Buchner, Frederike/0000-0001-8977-5344; Engeset, Dagrun/0000-0001-6946-2949; Rohrmann, Sabine/0000-0002-2215-1200; Olsen, Anja/0000-0003-4788-503X; Lund, Eiliv/0000-0002-8071-8711; Panico, Salvatore/0000-0002-5498-8312; moskal, aurelie/0000-0003-4127-4334; Crowe, Francesca/0000-0003-4026-1726; Romaguera, Dora/0000-0002-5762-8558; Navarro, Carmen/0000-0001-8896-7483; Riboli, Elio/0000-0001-6795-6080; Buckland, Genevieve/0000-0003-2060-6598; Dahm, Christina/0000-0003-0481-2893; Benetou, Vassiliki/0000-0002-0415-0386; tumino, rosario/0000-0003-2666-414X; PALLI, Domenico/0000-0002-5558-2437; Skeie, Guri/0000-0003-2476-4251; SANCHEZ-PEREZ, MARIA JOSE/0000-0003-4817-0757 European Commission (DG-SANCO); International Agency for Research on Cancer; Danish Cancer Society (Denmark); Ligue contre le Cancer, 3M, Mutuelle Generale de l'Education Nationale, Institut National de la Sante et de la Recherche Medicale (France); Deutsche Krebshilfe, Deutsches Krebsforschungszentrum and Federal Ministry of Education and Research (Germany); Ministry of Health and Social Solidarity; Stavros Niarchos Foundation; Hellenic Health Foundation (Greece); Italian Association for Research on Cancer; National Research Council (Italy); Dutch Ministry of Public Health, Welfare and Sports (VWS); Netherlands Cancer Registry (NKR); LK Research Funds; Dutch Prevention Funds; Dutch ZON (Zorg Onderzoek Nederland); World Cancer Research Fund (The Netherlands); NordForsk (Norway); Health Research Fund (FIS), Regional Governments of Andalucia, Asturias, Basque Country, Murcia and Navarra, ISCIII RETIC [RD06/0020]; Swedish Cancer Society; Swedish Scientific Council; Regional Government of Skane and Vasterbotten (Sweden); Cancer Research UK; Medical Research Council; Stroke Association; British Heart Foundation; Department of Health; Food Standards Agency; Wellcome Trust (UK) European Commission (DG-SANCO)(European Union (EU)European Commission Joint Research Centre); International Agency for Research on Cancer; Danish Cancer Society (Denmark)(Danish Cancer Society); Ligue contre le Cancer, 3M, Mutuelle Generale de l'Education Nationale, Institut National de la Sante et de la Recherche Medicale (France); Deutsche Krebshilfe, Deutsches Krebsforschungszentrum and Federal Ministry of Education and Research (Germany)(Deutsche Krebshilfe); Ministry of Health and Social Solidarity; Stavros Niarchos Foundation; Hellenic Health Foundation (Greece); Italian Association for Research on Cancer(Fondazione AIRC per la ricerca sul cancro); National Research Council (Italy)(Consiglio Nazionale delle Ricerche (CNR)); Dutch Ministry of Public Health, Welfare and Sports (VWS); Netherlands Cancer Registry (NKR); LK Research Funds; Dutch Prevention Funds; Dutch ZON (Zorg Onderzoek Nederland)(Netherlands Organization for Scientific Research (NWO)); World Cancer Research Fund (The Netherlands)(Netherlands Government); NordForsk (Norway)(NordForsk); Health Research Fund (FIS), Regional Governments of Andalucia, Asturias, Basque Country, Murcia and Navarra, ISCIII RETIC(Instituto de Salud Carlos III); Swedish Cancer Society(Swedish Cancer Society); Swedish Scientific Council; Regional Government of Skane and Vasterbotten (Sweden); Cancer Research UK(Cancer Research UK); Medical Research Council(UK Research & Innovation (UKRI)Medical Research Council UK (MRC)); Stroke Association; British Heart

Foundation(British Heart Foundation); Department of Health; Food Standards Agency; Wellcome Trust (UK)(Wellcome Trust) The coordination of EPIC is financially supported by the European Commission (DG-SANCO) and the International Agency for Research on Cancer. The national cohorts are supported by Danish Cancer Society (Denmark); Ligue contre le Cancer, 3M, Mutuelle Generale de l'Education Nationale, Institut National de la Sante et de la Recherche Medicale (France); Deutsche Krebshilfe, Deutsches Krebsforschungszentrum and Federal Ministry of Education and Research (Germany); Ministry of Health and Social Solidarity, Stavros Niarchos Foundation and Hellenic Health Foundation (Greece); Italian Association for Research on Cancer and National Research Council (Italy); Dutch Ministry of Public Health, Welfare and Sports (VWS), Netherlands Cancer Registry (NKR), LK Research Funds, Dutch Prevention Funds, Dutch ZON (Zorg Onderzoek Nederland), World Cancer Research Fund (The Netherlands); NordForsk (Norway); Health Research Fund (FIS), Regional Governments of Andalucia, Asturias, Basque Country, Murcia and Navarra, ISCIII RETIC (RD06/0020; Spain); Swedish Cancer Society, Swedish Scientific Council and Regional Government of Skane and Vasterbotten (Sweden); Cancer Research UK, Medical Research Council, Stroke Association, British Heart Foundation, Department of Health, Food Standards Agency, and Wellcome Trust (UK). The contribution of Bertrand Hemon and Mathieu Boniol, IARC, is gratefully acknowledged. 32 208 222 1 31
SPRINGERNATURE LONDON CAMPUS, 4 CRINAN ST, LONDON, N1 9XW, ENGLAND 0007-0920 1532-1827 BRIT J CANCER Br. J. Cancer APR 26 2011 104 9 1493 1499 10.1038/bjc.2011.106
http://dx.doi.org/10.1038/bjc.2011.106 7
Oncology Science Citation Index Expanded (SCI-EXPANDED)
Oncology 755ME 21468044 hybrid, Green Published
2025-06-24 WOS:000289934900018
J Bowers, LW; Doerstling, SS; Shamsunder, MG; Lineberger, CG; Rossi, EL; Montgomery, SA; Coleman, MF; Gong, WD; Parker, JS; Howell, A; Harvie, M; Hursting, SD Bowers, Laura W.; Doerstling, Steven S.; Shamsunder, Meghana G.; Lineberger, Claire G.; Rossi, Emily L.; Montgomery, Stephanie A.; Coleman, Michael F.; Gong, Weida; Parker, Joel S.; Howell, Anthony; Harvie, Michelle; Hursting, Stephen D.
Reversing the Genomic, Epigenetic, and Triple-Negative Breast Cancer-Enhancing Effects of Obesity CANCER PREVENTION RESEARCH English Article
BODY-MASS INDEX; INTERMITTENT CALORIE RESTRICTION; MAMMARY-TUMOR INCIDENCE; DIETARY-FAT REDUCTION; DISEASE RISK MARKERS; WEIGHT CHANGE; ENERGY RESTRICTION; ADIPOSE-TISSUE; DIAGNOSIS; SURVIVAL The reversibility of the procancer effects of obesity was interrogated in formerly obese C57BL/6 mice that lost weight via a nonrestricted low-fat diet (LFD) or 3 distinct calorie -restricted (CR) regimens (low-fat CR, Mediterranean-style CR, or intermittent CR). These mice, along with continu-ously obese mice and lean control mice, were orthotopically injected with E0771 cells, a mouse model of triple-negative breast cancer. Tumor weight, systemic cytokines, and inci-dence of lung metastases were elevated in the continuously obese and nonrestricted LFD mice relative to the 3 CR groups. Gene expression differed between the obese and all CR groups, but not the nonrestricted LFD group, for numerous tumoral genes associated

with epithelial-to-mesenchymal transition as well as several genes in the normal mammary tissue associated with hypoxia, reactive oxygen species production, and p53 signaling. A high degree of concordance existed between differentially exp-ressed mammary tissue genes from obese versus all CR mice and a microarray dataset from overweight/obese women randomized to either no intervention or a CR diet. Assessment of differentially methylated regions in mouse mammary tissues revealed that obesity, relative to the 4 weight loss groups, was associated with significant DNA hypermethylation. However, the anticancer effects of the CR interventions were independent of their ability to reverse obesity-associated mammary epigenetic repro-gramming. Taken together, these preclinical data showing that the procancer effects of obesity are reversible by various forms of CR diets strongly support translational exploration of restricted dietary patterns for reducing the burden of obesity-associated cancers. Prevention Relevance: Obesity is an established risk and progression factor for triple-negative breast cancer (TNBC). Given rising global rates of obesity and TNBC, strategies to reduce the burden of obesity-driven TNBC are urgently need-ed. We report the genomic, epigenetic, and procancer effects of obesity are reversible by various calorie restriction regimens. [Bowers, Laura W.; Gong, Weida; Parker, Joel S.; Hursting, Stephen D.] Univ N Carolina, Lineberger Comprehens Canc Ctr, Chapel Hill, NC USA; [Doerstling, Steven S.] Duke Univ, Sch Med, Durham, NC USA; [Shamsunder, Meghana G.; Lineberger, Claire G.; Coleman, Michael F.; Hursting, Stephen D.] Univ N Carolina, Dept Nutr, Chapel Hill, NC USA; [Rossi, Emily L.] NCI, Ctr Canc Res, Lab Human Carcinogenesis, Bethesda, MD USA; [Montgomery, Stephanie A.] Univ N Carolina, Dept Pathol & Lab Med, Chapel Hill, NC USA; [Howell, Anthony; Harvie, Michelle] Manchester Univ NHS Fdn Trust, Nightingale Ctr, Prevent Breast Canc Res Unit, Manchester, England; [Howell, Anthony; Harvie, Michelle] Univ Manchester, Div Canc Sci, Manchester, England; [Hursting, Stephen D.] Univ N Carolina, Nutr Res Inst, Kannapolis, NC USA; [Hursting, Stephen D.] Univ N Carolina, 135 Dauer Dr,CB 7461, Chapel Hill, NC 27599 USA University of North Carolina; University of North Carolina Chapel Hill; Duke University; University of North Carolina; University of North Carolina Chapel Hill; National Institutes of Health (NIH) - USA; NIH National Cancer Institute (NCI); University of North Carolina; University of North Carolina Chapel Hill; Manchester University NHS Foundation Trust; University of Manchester; University of North Carolina; University of North Carolina; University of North Carolina Chapel Hill Hursting, SD (corresponding author), Univ N Carolina, 135 Dauer Dr,CB 7461, Chapel Hill, NC 27599 USA. hursting@email.unc.edu Coleman, Michael/AAM-4725-2021 Bowers, Laura W/0000-0002-2124-3007; Shamsunder, Meghana/0000-0003-1860-936X; harvie, michelle/0000-0001-9761-3089; Howell, Anthony/0000-0002-6233-719X; Coleman, Michael/0000-0002-6914-848X Breast Cancer Research Foundation [R35 CA197627]; National Cancer Institute [R25CA057726]; National Cancer Institute [R35CA197627] Funding Source: NIH RePORTER

Breast Cancer Research Foundation; National Cancer Institute(United States Department of Health & Human ServicesNational Institutes of Health (NIH) - USANIH National Cancer Institute (NCI)); National Cancer Institute(United States Department of Health & Human ServicesNational Institutes of Health (NIH) - USANIH National Cancer Institute (NCI)) Acknowledgments

We would like to acknowledge the contributions of Andrew H. Sims, who passed away in 2021, to this project. His assistance with data analysis was much appreciated. This study was supported by a grant from the Breast Cancer Research Foundation and R35 CA197627 (to S.D. Hursting) . L.W. Bowers and E.L. Rossi were supported by a grant from the National Cancer Institute (R25CA057726) . 50
13 15 0 8 AMER ASSOC CANCER RESEARCH
PHILADELPHIA 615 CHESTNUT ST, 17TH FLOOR, PHILADELPHIA, PA 19106-4404 USA 1940-6207 1940-6215 CANCER PREV RES
Cancer Prev. Res. SEP 2022 15 9
581 594 10.1158/1940-6207.CAPR-22-0113
http://dx.doi.org/10.1158/1940-6207.CAPR-22-0113
14 Oncology Science Citation Index Expanded (SCI-EXPANDED) Oncology 4N6NU 35696725 Green Submitted, Green Accepted, Bronze 2025-06-24 WOS:000854136000001
J Shahryarhesami, S; Heida, M; Heidari, M; Sadighi, N
Shahryarhesami, Soroosh; Heida, Mansour; Heidari, Masoud; Sadighi, Nahid Human Homeobox TGIFLX Regulates CDX1, CDX2, and OCT1 Genes Expression in Colorectal Cancer Cell Lines MIDDLE EAST JOURNAL OF CANCER English
Article Colorectal neoplasms; Homeobox genes; RNA; Small interfering; Mutation INTESTINAL METAPLASIA; HOMEOTIC GENES; HOX GENES; ROLES; MODEL
Background: Homeodomain transcriptional regulatory proteins, which are encoded by Homeobox (HOX) genes, play critical roles in both normal development and carcinogenesis. Previous studies have shown that the expression of HOX genes is deregulated in numerous tumors and this expression is specific to each cancer based on the arising embryonic origin tissue and the site of tumor. Method: In this in vitro study, the expression levels of HOXA10, CDX1, CDX2, TGIFLX, TGIFLY, and OCT1 genes were compared across 10 different human colorectal cancer cell lines with different differentiation stages. Subsequently, the effect of TGIFLX siRNA-mediated knockdown on the expression levels of CDX1, CDX2, and OCT1 genes was analyzed in SW948 cell line. Results: The obtained results revealed that these homeobox genes were differentially expressed in different colorectal cancer cell lines. Furthermore, the siRNA-mediated knockdown of TGIFLX led to higher levels of CDX1, CDX2, and OCT1 expression. Conclusion: Our data suggested that TGIFLX plays an important role in the upstream regulation of CDX1, CDX2, and OCT1 genes. [Shahryarhesami, Soroosh; Heida, Mansour] Univ Tehran Med Sci, Dept Med Genet, Tehran, Iran; [Heidari, Masoud] Tabriz Univ, Fac Nat Sci, Dept Anim Biol, Tabriz, Iran; [Sadighi, Nahid] Univ Tehran Med Sci, Canc Res Ctr, Canc Inst Iran, Tehran, Iran Tehran University of Medical Sciences; University of Tabriz; Tehran University of Medical Sciences Sadighi, N (corresponding author), Univ Tehran Med Sci, Canc Res Ctr, Canc Inst Iran, Tehran, Iran. sadighii@yahoo.com
40 0 0 0 1 SHIRAZ UNIV MEDICAL SCIENCES SHIRAZ NEMAZEE HOSPITAL, SHIRAZ, 71934, IRAN
2008-6709 2008-6687 MIDDLE EAST J CANCER Middle East J. Cancer APR 2022 13 2 216
225 10.30476/mejc.2021.86467.1345
http://dx.doi.org/10.30476/mejc.2021.86467.1345
10 Oncology Emerging Sources Citation Index (ESCI)
Oncology 1K3XS 2025-06-24
WOS:000798538500002

J	WALKER, ARP; WALKER, BF; STELMA, S
	WALKER, ARP; WALKER, BF; STELMA, S		IS
BREAST-CANCER AVOIDABLE - COULD DIETARY-CHANGES HELP
	INTERNATIONAL JOURNAL OF FOOD SCIENCES AND NUTRITION
		English	Article
	CORONARY HEART-DISEASE; RURAL BLACK-WOMEN; SOUTH-AFRICA; RISK-FACTORS; FOLLOW-UP; FAT; POPULATION; ASSOCIATION; PROSPECTS; PATTERNS	In the US, the Life time odds of developing breast cancer has reached one in eight, with an incidence rate of 85 per 100 000 world population. The rate is half or less in women in some Mediterranean countries. At the extreme are rural African women whose rate is approximately 5-10 per 100 000. In African, compared with White women, protective factors include (1) reproductive behaviour, namely, slower growth before and after puberty, later age at menarche, high teenage pregnancy, high parity and long periods of lactation and amenorrhoea and (2) a diet of relatively low energy intake and of low-fat and high-fibre contents. In the Mediterranean setting, major dietary protective factors include a relatively low intake of saturated fat and high intakes of monounsaturated fat and of vegetables and fruit. Among White women, a reversion to protective reproductive behaviour is out of the question. Only in respect of diet, could significant avoiding action be taken. Adoption of an African type diet is wholly impracticable. Moreover, even conformation to a former Mediterranean diet, which is already changing, would be difficult, requiring reorientation of fat composition and large rises in intakes of vegetable and fruit. However, with resolution, were such changes made, at least by the very vulnerable, they would help, additionally, to protect against other diet-related cancers, especially colon cancer and against coronary heart disease.	S AFRICAN INST MED RES, JOHANNESBURG 2000, SOUTH AFRICA	UNIV WITWATERSRAND, SCH PATHOL, DEPT TROP DIS, HUMAN BIOCHEM RES UNIT, POB 1038, JOHANNESBURG 2000, SOUTH AFRICA.	Walker, Alejandro/AAF-2627-2021		78	6
	6	0	7	TAYLOR & FRANCIS LTD	ABINGDON	2-4 PARK SQUARE, MILTON PARK, ABINGDON OR14 4RN, OXON, ENGLAND	0963-7486
	1465-3478	INT J FOOD SCI NUTR	Int. J. Food Sci. Nutr.	NOV	1995	46	4		373	381
	10.3109/09637489509012569
	http://dx.doi.org/10.3109/09637489509012569		9
	Food Science & Technology; Nutrition & Dietetics	Science Citation Index Expanded (SCI-EXPANDED)	Food Science & Technology; Nutrition & Dietetics	TD612	8574864		2025-06-24	WOS:A1995TD61200008
J	Ali, SHM; Al-Alwan, NAS; Al-Alwany, SHM		Ali, S. H. M.; Al-Alwan, N. A. S.; Al-Alwany, S. H. M.
	Detection and genotyping of human papillomavirus in breast cancer tissues from Iraqi patients	EASTERN MEDITERRANEAN HEALTH JOURNAL		English	Article
		CERVICAL-CANCER; HPV DNA; VIRUS; SEQUENCES; CARCINOMAS; SUBSET; WOMEN; RISK	Studies have suggested a possible link between breast cancer pathogenesis and human papillomavirus (HPV) infection. This study in Iraq used in situ hybridization to detect the frequency and genotyping of HPV in tissue specimens from 129 patients diagnosed with malignant breast cancer, 24 with benign breast tumours and 20 healthy controls. In the breast cancer group, cocktail HPV genotypes were detected in 60 (46.5%) archived tissue blocks. Of these, genotypes 16 (55.5%),

18 (58.4%), 31 (65.0%) and 33 (26.6%) were detected. Mixed HPV genotypes 16 + 18, 16 + 18 + 31, 16 + 18 + 33, 18 + 33, 16 + 31 and 18 + 31 were found in 5.0%, 25.0%, 8.3%, 7.7%, 10.0% and 13.3% of cancer cases respectively. Only 3 benign breast tumour tissues (12.5%) and none of the healthy breast tissue specimens were HPV-DNA-positive. The detection of high-oncogenic HPV genotypes in patients with breast cancer supports the hypothesis of an etiologic role for the virus in breast cancer development.

[Ali, S. H. M.; Al-Alwany, S. H. M.] Baghdad Med Coll, Communicable Dis Res Unit, Baghdad, Iraq; [Al-Alwan, N. A. S.] Univ Baghdad, Iraqi Natl Canc Res Ctr, Baghdad, Iraq

University of Baghdad Al-Alwan, NAS (corresponding author), Univ Baghdad, Iraqi Natl Canc Res Ctr, Baghdad, Iraq.

nadalwan@yahoo.com Alwan, Nada/AAS-8284-2020 HASAN MOHAMMED ALI, SAAD/0000-0001-6474-3442; Alwan, Nada A S/0000-0002-2157-8632 24 23 30 0 1 WHO EASTERN MEDITERRANEAN REGIONAL OFFICE NASR CITY, CAIRO P. O. BOX 7608, NASR CITY, CAIRO, EGYPT 1020-3397 1687-1634 E MEDITERR HEALTH J East Mediterr. Health J. JUN 2014 20

6 372 377

10.26719/2014.20.6.372

http://dx.doi.org/10.26719/2014.20.6.372 6

Health Care Sciences & Services; Health Policy & Services; Public, Environmental & Occupational Health Science Citation Index Expanded (SCI-EXPANDED); Social Science Citation Index (SSCI) Health Care Sciences & Services; Public, Environmental & Occupational Health AK2EQ 24960513 Bronze

2025-06-24 WOS:000338232300004

J Eng, A; McCormack, V; dos-Santos-Silva, I

Eng, Amanda; McCormack, Valerie; dos-Santos-Silva, Isabel

Receptor-Defined Subtypes of Breast Cancer in Indigenous Populations in Africa: A Systematic Review and Meta-Analysis PLOS MEDICINE English Article

ALDEHYDE DEHYDROGENASE 1; MOLECULAR SUBTYPES; EGYPTIAN WOMEN; ESTROGEN-RECEPTOR; HORMONE-RECEPTOR; RISK-FACTORS; OVER-EXPRESSION; MESSENGER-RNA; PREVALENCE; AGE Background Breast cancer is the most common female cancer in Africa. Receptor-defined subtypes are a major determinant of treatment options and disease outcomes but there is considerable uncertainty regarding the frequency of poor prognosis estrogen receptor (ER) negative subtypes in Africa. We systematically reviewed publications reporting on the frequency of breast cancer receptor-defined subtypes in indigenous populations in Africa. Methods and Findings Medline, Embase, and Global Health were searched for studies published between 1st January 1980 and 15th April 2014. Reported proportions of ER positive (ER+), progesterone receptor positive (PR+), and human epidermal growth factor receptor-2 positive (HER2+) disease were extracted and 95% CI calculated. Random effects meta-analyses were used to pool estimates. Fifty-four studies from North Africa (n=12,284 women with breast cancer) and 26 from sub-Saharan Africa (n=4,737) were eligible. There was marked between-study heterogeneity in the ER+ estimates in both regions (I-2>90%), with the majority reporting proportions between 0.40 and 0.80 in North Africa and between 0.20 and 0.70 in sub-Saharan Africa. Similarly, large between-study heterogeneity was observed for PR+ and HER2+ estimates (I-2>80%, in all instances). Meta-regression analyses showed that the proportion of ER+ disease was 10% (4%-17%) lower for studies based

on archived tumor blocks rather than prospectively collected specimens, and 9% (2%-17%) lower for those with 40% versus those with <40% grade 3 tumors. For prospectively collected samples, the pooled proportions for ER+ and triple negative tumors were 0.59 (0.56-0.62) and 0.21 (0.17-0.25), respectively, regardless of region. Limitations of the study include the lack of standardized procedures across the various studies; the low methodological quality of many studies in terms of the representativeness of their case series and the quality of the procedures for collection, fixation, and receptor testing; and the possibility that women with breast cancer may have contributed to more than one study. Conclusions The published data from the more appropriate prospectively measured specimens are consistent with the majority of breast cancers in Africa being ER+. As no single subtype dominates in the continent availability of receptor testing should be a priority, especially for young women with early stage disease where appropriate receptor-specific treatment modalities offer the greatest potential for reducing years of life lost. [Eng, Amanda; dos-Santos-Silva, Isabel] London Sch Hyg & Trop Med, Dept Noncommunicable Dis Epidemiol, London WC1, England; [Eng, Amanda] Massey Univ, Ctr Publ Hlth Res, Wellington, New Zealand; [McCormack, Valerie] Int Agcy Res Canc, Sect Environm & Radiat, F-69372 Lyon, FranceUniversity of London; London School of Hygiene & Tropical Medicine; Massey University; World Health Organization; International Agency for Research on Cancer (IARC)
Eng, A (corresponding author), London Sch Hyg & Trop Med, Dept Noncommunicable Dis Epidemiol, London WC1, England.
isabel.silva@lshtm.ac.uk McCormack, Valerie/NAZ-3472-2025
dos Santos Silva, Isabel/0000-0002-6596-8798; McCormack, Valerie/0000-0001-7397-3442 International Agency for Research on Cancer (IARC) International Agency for Research on Cancer (IARC)(World Health Organization) No specific funding was received for this study. IdSS contribution was partly funded by a Senior Visiting Scientist Award by the International Agency for Research on Cancer (IARC). This organisation had no role in study design, data collection and analysis, decision to publish, or preparation of the manuscript. 105 84 87 0 6 PUBLIC LIBRARY SCIENCE SAN FRANCISCO 1160 BATTERY STREET, STE 100, SAN FRANCISCO, CA 94111 USA 1549-1277 1549-1676 PLOS MED
PLos Med. SEP 2014 11 9
e1001720 10.1371/journal.pmed.1001720
http://dx.doi.org/10.1371/journal.pmed.1001720
28 Medicine, General & InternalScience Citation Index Expanded (SCI-EXPANDED) General & Internal Medicine AQ6FO
25202974 Green Published, Green Submitted, gold
2025-06-24 WOS:000342905700021
J Cervantes-Amat, M; López-Abente, G; Aragonés, N; Pollán, M; Pastor-Barriuso, R; Pérez-Gómez, B
Cervantes-Amat, Marta; Lopez-Abente, Gonzalo; Aragones, Nuria; Pollan, Marina; Pastor-Barriuso, Roberto; Perez-Gomez, Beatriz The end of the decline in cervical cancer mortality in Spain: trends across the period 1981-2012 BMC CANCER English Article
Uterine cervical neoplasms; Mortality rate; Spain; Trends HUMAN-PAPILLOMAVIRUS; BURDEN; COHORT; AGE; PREVENTION; REGRESSION Background: In Spain, cervical cancer prevention is based on opportunistic screening, due to the disease's traditionally low incidence and mortality rates. Changes in sexual

behaviour, tourism and migration have, however, modified the probability of exposure to human papilloma virus among Spaniards. This study thus sought to evaluate recent cervical cancer mortality trends in Spain. Methods: We used annual female population figures and individual records of deaths certified as cancer of cervix, reclassifying deaths recorded as unspecified uterine cancer to correct coding quality problems. Joinpoint models were fitted to estimate change points in trends, as well as the annual (APC) and average annual percentage change. Log- linear Poisson models were also used to study age-period-cohort effects on mortality trends and their change points. Results: 1981 marked the beginning of a decline in cervical cancer mortality (APC(1981-2003): -3.2; 95% CI:-3.4;-3.0) that ended in 2003, with rates reaching a plateau in the last decade (APC(2003-2012): 0.1; 95% CI:-0.9; 1.2). This trend, which was observable among women aged 45-46 years (APC(2003-2012): 1.4; 95% CI:-0.1; 2.9) and over 65 years (APC(2003-2012): -0.1; 95% CI:-1.9; 1.7), was clearest in Spain's Mediterranean and Southern regions. Conclusions: The positive influence of opportunistic screening is not strong enough to further reduce cervical cancer mortality rates in the country. Our results suggest that the Spanish Health Authorities should reform current prevention programmes and surveillance strategies in order to confront the challenges posed by cervical cancer.
[Cervantes-Amat, Marta; Lopez-Abente, Gonzalo; Aragones, Nuria; Pollan, Marina; Pastor-Barriuso, Roberto; Perez-Gomez, Beatriz] CIBERESP, Consortium Biomed Res Epidemiol & Publ Hlth, Madrid 28029, Spain; [Cervantes-Amat, Marta; Lopez-Abente, Gonzalo; Aragones, Nuria; Pollan, Marina; Pastor-Barriuso, Roberto; Perez-Gomez, Beatriz] Carlos III Inst Hlth, Natl Ctr Epidemiol, Canc & Environm Epidemiol Unit, Madrid 28029, Spain; [Lopez-Abente, Gonzalo; Aragones, Nuria; Pollan, Marina; Pastor-Barriuso, Roberto; Perez-Gomez, Beatriz] Puerta Hierro Biomed Res Inst, Majadahonda 28222, Spain CIBER - Centro de Investigacion Biomedica en Red; CIBERESP; Instituto de Salud Carlos III
Pérez-Gómez, B (corresponding author), CIBERESP, Consortium Biomed Res Epidemiol & Publ Hlth, Avda Monforte Lemos 5, Madrid 28029, Spain. bperez@isciii.es Lopez-Abente, Gonzalo/E-5221-2010; Perez-Gomez, Beatriz/C-4715-2012; Pollan, Marina/M-3259-2014; Aragones, Nuria/O-5962-2015; Pastor-Barriuso, Roberto/AAA-6746-2019 Perez-Gomez, Beatriz/0000-0002-4299-8214; Pollan, Marina/0000-0002-4328-1565; Aragones, Nuria/0000-0003-0983-2156; Pastor-Barriuso, Roberto/0000-0002-7325-3960 Spanish Health Research Fund [FIS PI11/00871] Spanish Health Research Fund(Instituto de Salud Carlos III) This study was supported by a research grant from the Spanish Health Research Fund [FIS PI11/00871]. Mortality data were furnished by the Spanish National Statistics Institute under the terms of a specific confidentially protocol. 36 12 13 0 11 BMC LONDON
CAMPUS, 4 CRINAN ST, LONDON N1 9XW, ENGLAND 1471-2407
BMC CANCER BMC Cancer APR 15 2015 15
287 10.1186/s12885-015-1306-x
http://dx.doi.org/10.1186/s12885-015-1306-x 9
Oncology Science Citation Index Expanded (SCI-EXPANDED)
Oncology CG7AR 25886170 Green Published, gold
2025-06-24 WOS:000353454800001
J Garcia-Guasch, M; Medrano, M; Costa, I; Vela, E; Grau, M; Escrich, E; Moral, R Garcia-Guasch, Maite; Medrano, Mireia; Costa, Irmgard; Vela, Elena; Grau, Marta;

Escrich, Eduard; Moral, Raquel Extra-Virgin Olive Oil and Its Minor Compounds Influence Apoptosis in Experimental Mammary Tumors and Human Breast Cancer Cell Lines CANCERS English Article

breast cancer; experimental mammary tumors; high-fat diets; apoptosis; cell death; olive oil; hydroxytyrosol; oleuropein; luteolin CORN-OIL; MEDITERRANEAN DIET; LIPID INFLUENCE; RISK; RAT; METABOLISM; MECHANISMS; CARCINOMA; LUTEOLIN; GROWTH Simple Summary Breast cancer is a disease influenced by dietetic factors, such as the type and amount of lipids in a diet. In this work, we aimed to elucidate the different effects of two high-fat diets on the histopathological and molecular characteristics of mammary tumors in an experimental model. Animals fed with a diet high in extra-virgin olive oil (EVOO), compared to those fed with a diet high in seed oil, developed tumors with less aggressiveness and proliferation. Tumor molecular analyses of several cell death pathways also suggested an effect of EVOO in this process. In vitro experiments indicated the role of EVOO minor compounds on the effects of this oil. Obtaining insights into the influence and the mechanisms of action of dietary compounds are necessary to understand the relevance that dietetic habits from childhood may have on health and the risk of disease. Breast cancer is the most common malignancy among women worldwide. Modifiable factors such as nutrition have a role in its etiology. In experimental tumors, we have observed the differential influence of high-fat diets in metabolic pathways, suggesting a different balance in proliferation/apoptosis. In this work, we analyzed the effects of a diet high in n-6 polyunsaturated fatty acids (PUFA) and a diet high in extra-virgin olive oil (EVOO) on the histopathological features and different cell death pathways in the dimethylbenz(a)anthracene-induced breast cancer model. The diet high in n-6 PUFA had a stimulating effect on the morphological aggressiveness of tumors and their proliferation, while no significant differences were found in groups fed the EVOO-enriched diet in comparison to a low-fat control group. The high-EVOO diet induced modifications in proteins involved in several cell death pathways. In vitro analysis in different human breast cancer cell lines showed an effect of EVOO minor compounds (especially hydroxytyrosol), but not of fatty acids, decreasing viability while increasing apoptosis. The results suggest an effect of dietary lipids on tumor molecular contexts that result in the modulation of different pathways, highlighting the importance of apoptosis in the interplay of survival processes and how dietary habits may have an impact on breast cancer risk.

[Garcia-Guasch, Maite; Medrano, Mireia; Vela, Elena; Grau, Marta; Escrich, Eduard; Moral, Raquel] Univ Autonoma Barcelona, Fac Med, Dept Cell Biol Physiol & Immunol, Barcelona 08193, Spain; [Costa, Irmgard] Corp Parc Tauli UDIAT, Dept Pathol, Sabadell 08208, Spain

Autonomous University of Barcelona; University of Barcelona; Autonomous University of Barcelona; Parc Tauli Hospital Universitari Moral, R (corresponding author), Univ Autonoma Barcelona, Fac Med, Dept Cell Biol Physiol & Immunol, Barcelona 08193, Spain. Mireia.Medrano@uab.cat; Mireia.Medrano@uab.cat; icosta@tauli.cat; Elena.Vela@uab.cat; Mireia.Medrano@uab.cat; Eduard.Escrich@uab.cat; Mireia.Medrano@uab.cat Grau, Marta/ABE-5817-2021; Moral, Raquel/H-2122-2015; Medrano, Mireia/AFP-3427-2022 Grau, Marta/0000-0003-3779-0387; Garcia, Maria Teresa/0000-0002-8375-5657; Moral, Raquel/0000-0002-2738-

6121; Medrano, Mireia/0000-0002-8707-5501 Plan Nacional de I + D + I [AGL2006-07691]; Fundacion Patrimonio Comunal Olivarero (FPCO) [FPCO2008-165.396, FPCO2013-CF611.084]; Agencia para el Aceite de Oliva del Ministerio de Agricultura, Alimentacion y Medio Ambiente [AAO2008-165.471]; Organizacion Interprofesional del Aceite de Oliva Espanol (OIAOE) [OIP2009-CD165.646]; Departaments de Salut i d'Agricultura, Alimentacio i Accio Rural de la Generalitat de Catalunya [GC2010-165.000]; FPCO; OIAOE [FPCO-OIP2016-CF614.087] Plan Nacional de I + D + I(Spanish Government); Fundacion Patrimonio Comunal Olivarero (FPCO); Agencia para el Aceite de Oliva del Ministerio de Agricultura, Alimentacion y Medio Ambiente; Organizacion Interprofesional del Aceite de Oliva Espanol (OIAOE); Departaments de Salut i d'Agricultura, Alimentacio i Accio Rural de la Generalitat de Catalunya; FPCO; OIAOE This research was funded by "Plan Nacional de I + D + I" (AGL2006-07691); "Fundacion Patrimonio Comunal Olivarero (FPCO)" (FPCO2008-165.396; FPCO2013-CF611.084); "Agencia para el Aceite de Oliva del Ministerio de Agricultura, Alimentacion y Medio Ambiente" (AAO2008-165.471); "Organizacion Interprofesional del Aceite de Oliva Espanol (OIAOE)" (OIP2009-CD165.646); "Departaments de Salut i d'Agricultura, Alimentacio i Accio Rural de la Generalitat de Catalunya" (GC2010-165.000); and FPCO and OIAOE (FPCO-OIP2016-CF614.087). 52 9 9 0 6 MDPI BASEL ST ALBAN-ANLAGE 66, CH-4052 BASEL, SWITZERLAND 2072-6694 CANCERS Cancers FEB 2022 14 4 905 10.3390/cancers14040905 http://dx.doi.org/10.3390/cancers14040905 20 Oncology Science Citation Index Expanded (SCI-EXPANDED) Oncology ZV9SJ 35205652 Green Published, gold 2025-06-24 WOS:000770863200001

J Luke, C; Nguyen, AM; Heard, A; Kenny, B; Shorne, L; Roder, D Luke, Colin; Nguyen, Anh-Minh; Heard, Adrian; Kenny, Bernadette; Shorne, Lesley; Roder, David Benchmarking epidemiological characteristics of cervical cancer in advance of change in screening practice and commencement of vaccination AUSTRALIAN AND NEW ZEALAND JOURNAL OF PUBLIC HEALTH English Article cervix cancer; histology; time trends; screening INDIGENOUS AUSTRALIANS; HUMAN-PAPILLOMAVIRUS; QUALITY ASSURANCE; SURVIVAL; TRENDS; RATES Objectives: To investigate trends in cervical cancer incidence, mortality and survival by histology for benchmarking purposes ahead of practice change and the introduction of Human Papilloma Virus (HPV) vaccine. Methods: Using data from the South Australian Cancer Registry, age-standardised rates are presented for four-year periods from 1977 to 2004. Socio-demographic and secular predictors of glandular as opposed to squamous cancers are investigated, using multivariable logistic regression. Disease-specific survivals are analysed using Kaplan-Meier product-limit estimates and Cox proportional hazards regression. Results: Incidence and mortality rates reduced by 55.1% and 59.3% respectively between 1977-80 and 2001-04, with larger reductions for squamous than glandular cancers. The ratio of squamous to glandular cancer incidence reduced from 5.41 in 1977-88 to 2.81 in 1993-2004, with a corresponding reduction from 5.21 to 3.01 for mortality. Compared with squamous cancers, glandular lesions were more common in patients from higher socioeconomic areas, but less common in those over 70 years of

age, Aboriginal patients, and those born in Southern Europe. Conclusion: The proportion of cancers comprising glandular lesions has increased, possibly reflecting prevention of squamous cancers through treatment of screen-detected preinvasive lesions. Additional mortality reductions from screening may be limited where the proportion of glandular lesions is high, with vaccination offering the best prospects for gains in the long term. Priority should be given to Aboriginal and Torres Strait Islander women in vaccination programs in view of their high death rate from cervical cancer. Dept Hlth, Epidemiol Branch, Adelaide, SA 5001, Australia Luke, C (corresponding author), Dept Hlth, Epidemiol Branch, POB 6,Rundle Mall, Adelaide, SA 5001, Australia. Colin.Luke@health.sa.gov.au Roder, David/B-7038-2013
Roder, David/0000-0001-6442-4409 30
8 8 0 1 PUBLIC HEALTH ASSOC AUSTRALIA INC
CURTIN PO BOX 319, CURTIN, ACT 2600, AUSTRALIA 1326-0200
AUST NZ J PUBL HEAL Aust. N. Z. Publ. Health
APR 2007 31 2 149 154
10.1111/j.1753-6405.2007.00033.x
http://dx.doi.org/10.1111/j.1753-6405.2007.00033.x
6 Public, Environmental & Occupational Health Science Citation Index Expanded (SCI-EXPANDED); Social Science Citation Index (SSCI) Public, Environmental & Occupational Health
163FH 17461006 Bronze 2025-06-24
WOS:000246143700010
J Benson, OG; Siciliano, A Gonzalez Benson, Odessa; Siciliano, Antonio A Rights-Based Framework in Global Social Work Education and International Development Work: Insights from a Global Independent Study in Tunisia JOURNAL OF HUMAN RIGHTS AND SOCIAL WORK
English Article Human rights framework; Social work field education; Activism; Inequality; Capacity building; Global social work FIELD; GLOBALIZATION; CHALLENGES In this reflective analysis, we use a rights-based framework from international development studies to examine a global independent study (GIS) course as social work field education. Our GIS course entailed fieldwork with a Tunisia-based migrant-led organization that advocates for missing migrants who have gone missing en route from Tunisia to Italy via the Mediterranean Sea. We use our case study to illustrate the five elements to a human rights framework. Rights and obligations, as two elements, set the terms of the engagement for fieldwork. Capacity building moves beyond service delivery and teaches empowerment. Inequality and poverty focus on structural aspects: policies, institutions, and sociopolitical contexts related to fieldwork. Activism and advocacy facilitate the student's participation in affecting change. The prioritizing of action in development work is thus applicable for teaching human rights in social work education "in the field," which requires going from theory to practice. [Gonzalez Benson, Odessa] Univ Michigan, Detroit Sch Urban Studies, Sch Social Work, Ann Arbor, MI 48109 USA; [Siciliano, Antonio] Univ Michigan, Sch Social Work, Ann Arbor, MI 48109 USA University of Michigan System; University of Michigan; University of Michigan System; University of Michigan
Benson, OG (corresponding author), Univ Michigan, Detroit Sch Urban Studies, Sch Social Work, Ann Arbor, MI 48109 USA.
odessagb@umich.edu 47
2 2 0 5 SPRINGER INT PUBL AG CHAM

GEWERBESTRASSE 11, CHAM, CH-6330, SWITZERLAND   2365-1792
J HUM RIGHTS SOC WOR   J. Hum. Rights Soc. Work
SEP   2021 6   3   183   192
10.1007/s41134-020-00158-6
http://dx.doi.org/10.1007/s41134-020-00158-6   MAR 2021
10   Social WorkEmerging Sources Citation Index (ESCI)
Social WorkUN7ZB   2025-06-24
WOS:000635060600001

J   Allahqoli, L; Mazidimoradi, A; Momenimovahed, Z; Rahmani, A; Hakimi, S; Tiznobaik, A; Gharacheh, M; Salehiniya, H; Babaey, F; Alkatout, I   Allahqoli, Leila; Mazidimoradi, Afrooz; Momenimovahed, Zohre; Rahmani, Azam; Hakimi, Sevil; Tiznobaik, Azita; Gharacheh, Maryam; Salehiniya, Hamid; Babaey, Farah; Alkatout, Ibrahim   The Global Incidence, Mortality, and Burden of Breast Cancer in 2019: Correlation With Smoking, Drinking, and Drug Use   FRONTIERS IN ONCOLOGY
English   Article   global; incidence; mortality; burden; breast cancer; smoking; drinking; drug use   HUMAN-DEVELOPMENT INDEX; RISK-FACTORS; WOMEN; SURVIVAL; TRENDS BackgroundFemale breast cancer (FBC) is the most common type of cancer and is associated with a considerable disease burden as well as significant mortality rates. The present study aimed to provide an update on the incidence, mortality, and burden of FBC in 2019, based on the Global Burden of Disease (GBD) Study. MaterialsThe incidence, death rate, disability-adjusted life years (DALYs), years of life lost (YLLs), years lived with disability (YLDs), the age-standardized rates (ASR) of FBC in 204 countries, and a variety of classifications, were retrieved from the Global Burden of Disease Study. Data on tobacco use, alcohol consumption, and drug use were collected. The incidence, mortality, and burden of FBC were registered and compared between regions. Associations between age-standardized incidence rates and age-standardized mortality rates of FBC with smoking, drinking, and drug use were determined. ResultsThe highest incidence of FBC was observed in countries with a high socioeconomic status such as those of the European continent. Despite the lower incidence of FBC in countries with a low socio-demographic index (SDI), mortality rates secondary to FBC are higher in these countries than in high-income countries. The highest age-standardized mortality rate has been reported in the Eastern Mediterranean Region (EMRO), followed by the African Region (AFRO). The highest age-standardized rates of DALY and YLL per 100,000 population in 2019 were observed in lower-income countries, while the highest ASR of YLD per 100,000 population was reported in high-income countries. ConclusionThe present GBD-based study provides a comprehensive review of the incidence, mortality, and burden of FBC in 2019. The incidence of FBC is higher in regions with a higher socioeconomic status, whereas mortality rates and DALYs are higher in poorly developed regions. We suggest better screening measures and early detection programs for the latter regions.
[Allahqoli, Leila] Minist Hlth & Med Educ, Midwifery Dept, Tehran, Iran; [Mazidimoradi, Afrooz] Shiraz Univ Med Sci, Student Res Comm, Shiraz, Iran; [Momenimovahed, Zohre] Qom Univ Med Sci, Fac Nursing & Midwifery, Dept Midwifery & Reprod Hlth, Qom, Iran; [Rahmani, Azam] Univ Tehran Med Sci, Nursing & Midwifery Care Res Ctr, Sch Nursing & Midwifery, Tehran, Iran; [Hakimi, Sevil] Tabriz Univ Med Sci, Sch Nursing & Midwifery, Tabriz, Iran; [Tiznobaik, Azita] Hamadan Univ Med Sci, Mother & Child Care Res Ctr, Sch

Nursing & Midwifery, Dept Midwifery & Reprod Hlth, Hamadan, Iran; [Gharacheh, Maryam] Iran Univ Med Sci, Nursing Care Res Ctr, Sch Nursing & Midwifery, Tehran, Iran; [Salehiniya, Hamid] Birjand Univ Med Sci, Social Determinants Hlth Res Ctr, Birjand, Iran; [Babaey, Farah] Minist Hlth & Med Educ, Midwifery Dept, Tehran, Iran; [Alkatout, Ibrahim] Univ Hosp Schleswig Holstein, Kiel Sch Gynaecol Endoscopy, Kiel, Germany Ministry of Health & Medical Education (MOHME); Shiraz University of Medical Science; Tehran University of Medical Sciences; Tabriz University of Medical Science; Hamadan University of Medical Sciences; Iran University of Medical Sciences; Birjand University of Medical Sciences; Ministry of Health & Medical Education (MOHME); University of Kiel; Schleswig Holstein University Hospital Alkatout, I (corresponding author), Univ Hosp Schleswig Holstein, Kiel Sch Gynaecol Endoscopy, Kiel, Germany. Ibrahim.Alkatout@uksh.de
Hakimi, sevil/AAB-9561-2022; Alkatout, Ibrahim/AAG-6077-2021; Gharacheh, Maryam/J-2577-2018; momenimovahed, zohre/GOH-0803-2022; Tiznobaik, Azita/AAI-5733-2021; Allahqoli, Leila/O-7376-2018; Salehiniya, Hamid/X-6214-2019 momenimovahed, zohre/0000-0001-7747-6080; Allahqoli, Leila/0000-0002-9851-6771
50 37 37 1 21 FRONTIERS MEDIA SA LAUSANNE AVENUE DU TRIBUNAL FEDERAL 34, LAUSANNE, CH-1015, SWITZERLAND 2234-943X FRONT ONCOL Front. Oncol. JUL 27 2022 12
921015 10.3389/fonc.2022.921015
http://dx.doi.org/10.3389/fonc.2022.921015 9
Oncology Science Citation Index Expanded (SCI-EXPANDED)
Oncology 3Z6KM 35965518 Green Published, gold
2025-06-24 WOS:000844526400001
J Van Blarigan, EL; Zhang, S; Ou, FS; Venlo, A; Ng, K; Atreya, C; Van Loon, K; Niedzwiecki, D; Giovannucci, E; Wolfe, EG; Lenz, HJ; Innocenti, F; O'Neil, BH; Shaw, JE; Polite, BN; Hochster, HS; Atkins, JN; Goldberg, RM; Mayer, RJ; Blanke, CD; O'Reilly, EM; Fuchs, CS; Meyerhardt, JA Van Blarigan, Erin L.; Zhang, Sui; Ou, Fang-Shu; Venlo, Alan; Ng, Kimmie; Atreya, Chloe; Van Loon, Katherine; Niedzwiecki, Donna; Giovannucci, Edward; Wolfe, Eric G.; Lenz, Heinz-Josef; Innocenti, Federico; O'Neil, Bert H.; Shaw, James E.; Polite, Blase N.; Hochster, Howard S.; Atkins, James N.; Goldberg, Richard M.; Mayer, Robert J.; Blanke, Charles D.; O'Reilly, Eileen M.; Fuchs, Charles S.; Meyerhardt, Jeffrey A. Association of Diet Quality With Survival Among People With Metastatic Colorectal Cancer in the Cancer and Leukemia B and Southwest Oncology Group 80405 Trial
JAMA NETWORK OPEN English Article
QUESTIONNAIRE; REPRODUCIBILITY; REGRESSION; VALIDITY; VALIDATION; RECURRENCE; PATTERNS
Importance Diet has been associated with survival in patients with stage I to III colorectal cancer, but data on patients with metastatic colorectal cancer are limited. Objective To examine the association between diet quality and overall survival among individuals with metastatic colorectal cancer. Design, Setting, and Participants This was a prospective cohort study of patients with metastatic colorectal cancer who were enrolled in the Cancer and Leukemia Group B (Alliance) and Southwest Oncology Group 80405 trial between October 27, 2005, and February 29, 2012, and followed up through January 2018. Exposures Participants completed a validated food frequency questionnaire within 4 weeks after initiation of first-line treatment for

metastatic colorectal cancer. Diets were categorized according to the Alternative Healthy Eating Index (AHEI), Alternate Mediterranean Diet (AMED) score, Dietary Approaches to Stop Hypertension (DASH) score, and Western and prudent dietary patterns derived using principal component analysis. Participants were categorized into sex-specific quintiles. Main Outcomes and Measures Multivariable hazard ratios (HRs) and 95% CIs for overall survival. Results In this cohort study of 1284 individuals with metastatic colorectal cancer, the median age was 59 (interquartile range [IQR]: 51-68) years, median body mass index was 27.2 (IQR, 24.1-31.4), 521 (41%) were female, and 1102 (86%) were White. There were 1100 deaths during a median follow-up of 73 months (IQR, 64-87 months). We observed an inverse association between the AMED score and risk of death (HR quintile 5 vs quintile 1, 0.83; 95% CI, 0.67-1.04; P = .04 for trend), but the point estimates were not statistically significant. None of the other diet scores or patterns were associated with overall survival. Conclusions and Relevance In this prospective analysis of patients with metastatic colorectal cancer, diet quality assessed at initiation of first-line treatment for metastatic disease was not associated with overall survival. This cohort study of patients with metastatic colorectal cancer examines associations between diet at initiation of first-line treatment and overall survival. Question Is diet quality at initiation of first-line treatment for metastatic colorectal cancer associated with overall survival? Findings In this cohort study including 1284 people with metastatic colorectal cancer enrolled between 2005 and 2012, diet quality (assessed by the Alternative Healthy Eating Index, Alternate Mediterranean Diet score, Dietary Approaches to Stop Hypertension diet score, a Western dietary pattern, and a prudent dietary pattern) was not associated with overall survival after a median follow-up of 73 months. Meaning The results of this study suggest that overall diet quality assessed at initiation of first-line treatment for metastatic colorectal cancer was not associated with overall survival. [Van Blarigan, Erin L.] Univ Calif San Francisco, Dept Epidemiol & Biostat, San Francisco, CA USA; [Zhang, Sui; Ng, Kimmie; Mayer, Robert J.; Meyerhardt, Jeffrey A.] Dana Farber Partners CancerCare, Boston, MA USA; [Ou, Fang-Shu; Wolfe, Eric G.] Mayo Clin, Alliance Stat & Data Management Ctr, Rochester, MN USA; [Venlo, Alan; Atreya, Chloe; Van Loon, Katherine] Univ Calif San Francisco, Dept Med, San Francisco, CA 94143 USA; [Niedzwiecki, Donna] Duke Univ, Alliance Stat & Data Ctr, Durham, NC USA; [Giovannucci, Edward] Harvard TH Chan Sch Publ Hlth, Dept Nutr, Boston, MA USA; [Giovannucci, Edward] Harvard TH Chan Sch Publ Hlth, Dept Epidemiol, Boston, MA USA; [Lenz, Heinz-Josef] Univ Southern Calif, USC Norris Comprehens Canc Ctr, Los Angeles, CA 90007 USA; [Innocenti, Federico] Univ N Carolina, UNC Eshelman Sch Pharm, Div Pharmacotherapy & Expt Therapeut, Chapel Hill, NC 27515 USA; [Innocenti, Federico] Univ N Carolina, Dept Med Hematol, Chapel Hill, NC 27515 USA; [O'Neil, Bert H.] Indiana Univ Sch Med, Simon Canc Ctr, Indianapolis, IN 46202 USA; [Shaw, James E.] Virginia Commonwealth Univ, Richmond, VA 23284 USA; [Polite, Blase N.] Univ Chicago, Pritzker Sch Med, Chicago, IL 60637 USA; [Hochster, Howard S.] Yale Univ, Sch Med, Dept Med Oncol, New Haven, CT USA; [Atkins, James N.] Southeast Clin Oncol Res Consortium, Winston Salem, NC USA; [Goldberg, Richard M.] West Virginia Univ, Canc Inst, Morgantown, WV 26506 USA; [Blanke, Charles D.] Oregon Hlth & Sci Univ, SWOG Grp Chairs

Off, Knight Canc Inst, Portland, OR 97201 USA; [O'Reilly, Eileen M.] Mem Sloan Kettering Canc Ctr, 1275 York Ave, New York, NY 10021 USA; [Fuchs, Charles S.] Yale Sch Med, Yale Canc Ctr, New Haven, CT USA   University of California System; University of California San Francisco; Harvard University; Harvard University Medical Affiliates; Dana-Farber Cancer Institute; Mayo Clinic; University of California System; University of California San Francisco; Duke University; Harvard University; Harvard T.H. Chan School of Public Health; Harvard University; Harvard T.H. Chan School of Public Health; University of Southern California; University Southern California Hospital; University of North Carolina; University of North Carolina Chapel Hill; University of North Carolina; University of North Carolina Chapel Hill; Indiana University System; Indiana University Bloomington; Virginia Commonwealth University; University of Chicago; Yale University; West Virginia University; Southwest Oncology Group; Oregon Health & Science University; Memorial Sloan Kettering Cancer Center; Yale University; Yale New Haven Hospital   Van Blarigan, EL (corresponding author), Univ Calif San Francisco, POB 0560,550 16th St,Second Floor, San Francisco, CA 94158 USA.
erin.vanblarigan@ucsf.edu   O'Reilly, Eileen/LTD-4381-2024; Innocenti, Federico/G-3206-2018; Meyerhardt, Jeffrey/IAP-4528-2023; Giovannucci, Edward/ADE-8028-2022 Niedzwiecki, Donna/0000-0002-3566-0450   National Cancer Institute   National Cancer Institute(United States Department of Health & Human ServicesNational Institutes of Health (NIH) - USANIH National Cancer Institute (NCI))   The National Cancer Institute contributed to the design of the study and review of the manuscript. Nonfederal sponsors had no role in the design and conduct of the study; collection, management, analysis, and interpretation of the data; preparation, review, or approval of the manuscript; and decision to submit the manuscript for publication.   29   14   14   0   4   AMER MEDICAL ASSOC CHICAGO   330 N WABASH AVE, STE 39300, CHICAGO, IL 60611-5885 USA   2574-3805   JAMA NETW OPEN   JAMA Netw. Open
OCT 30   2020 3   10
e2023500   10.1001/jamanetworkopen.2020.23500
http://dx.doi.org/10.1001/jamanetworkopen.2020.23500
9   Medicine, General & Internal Science Citation Index Expanded (SCI-EXPANDED)   General & Internal Medicine
OP5FY 33125497   gold, Green Published   2025-06-24   WOS:000588111100006
J   Parma, DAL; Reynolds, GL; Muñoz, E; Ramirez, AG
Parma, Dorothy A. Long; Reynolds, Grace L.; Munoz, Edgar; Ramirez, Amelie G.   Effect of an anti-inflammatory dietary intervention on quality of life among breast cancer survivors SUPPORTIVE CARE IN CANCER   English
Article   Health-related quality of life; Mediterranean diet; Perceived Stress Scale
FUNCTIONAL ASSESSMENT; STRESS; TRIAL; COMORBIDITIES; DEPRESSION; MANAGEMENT; PROGRAM; HEALTH; SCALE; CARE   Purpose Behavioral interventions have been used with breast cancer survivors (BCS) in cancer pain management and post-treatment quality of life (QOL) studies. We studied the effects of an anti-inflammatory dietary intervention on QOL in BCS. Methods One hundred fifty-three overweight and obese (body mass index [BMI] >= 25 kg/m(2)), early stage (0-III), English-speaking BCS who had completed all cancer treatment 2 or more months prior to

enrollment were recruited into a two-arm randomized controlled trial with a 2 (group) by 3 (time) repeated measures design. Intervention components included six monthly food-preparation workshops and twelve motivational interviewing telephone calls. Endpoints included the Perceived Stress Scale (PSS), the Functional Assessment of Cancer Therapy-General (FACT-G) and Breast Cancer (FACT-B), and the Center for Epidemiologic Studies Depression Scale (CES-D). Repeated measures analysis using PROC MIXED in SAS version 9.4 was used. Results On repeated measures analysis (intent to treat), there were no differences between groups on any of the QOL outcomes except the PSS total scores. These were significantly different in the intervention group (IG; n = 76) compared to control group (CG; n = 77), showing a main effect of assignment but no effect of time and no interaction effects. Conclusion There was an impact on QOL as measured by the PSS between groups. The intervention reduced perceived stress at 6-month follow-up, but the effects dissipated by 12 months. Sources and stress and stress reduction should be a focus of future studies. Future research should also identify appropriate QOL measures that are sensitive to changes brought about by behavioral interventions. [Parma, Dorothy A. Long; Munoz, Edgar; Ramirez, Amelie G.] UT Hlth San Antonio, Inst Hlth Promot Res, Dept Populat Hlth Sci, 7411 John Smith Dr Suite 1000, San Antonio, TX 78229 USA; [Reynolds, Grace L.] Calif State Univ Long Beach, Ctr Behav Res & Serv, Dept Hlth Care Adm, Long Beach, CA 90840 USA
University of Texas System; University of Texas Health Science Center at San Antonio; California State University System; California State University Long Beach Parma, DAL (corresponding author), UT Hlth San Antonio, Inst Hlth Promot Res, Dept Populat Hlth Sci, 7411 John Smith Dr Suite 1000, San Antonio, TX 78229 USA. longparma@uthscsa.edu Munoz, Edgar/HTT-4853-2023; Reynolds, Grace/AAR-7815-2021 Munoz, Edgar/0000-0002-1555-8408; Reynolds, Grace L./0000-0003-3324-0337; Ramirez, Amelie/0000-0002-5310-1337
Susan G. Komen for the Cure [SAB08-0005]; National Cancer Institute [P20 CA165589]; Mays Cancer Center at UT Health San Antonio, through the NCI Cancer Center Support Grant [P30 CA054174] Susan G. Komen for the Cure(Susan G. Komen Breast Cancer Foundation); National Cancer Institute(United States Department of Health & Human ServicesNational Institutes of Health (NIH) - USANIH National Cancer Institute (NCI)); Mays Cancer Center at UT Health San Antonio, through the NCI Cancer Center Support Grant This research was supported by Susan G. Komen for the Cure (SAB08-0005); the National Cancer Institute (P20 CA165589); and the Mays Cancer Center at UT Health San Antonio, through the NCI Cancer Center Support Grant (P30 CA054174).
48 13 13 1 7 SPRINGER NEW YORK ONE NEW YORK PLAZA, SUITE 4600, NEW YORK, NY, UNITED STATES 0941-4355
1433-7339 SUPPORT CARE CANCER Support. Care Cancer
JUL 2022 30 7 5903 5910
10.1007/s00520-022-07023-4
http://dx.doi.org/10.1007/s00520-022-07023-4 APR 2022
8 Oncology; Health Care Sciences & Services; Rehabilitation Science Citation Index Expanded (SCI-EXPANDED)
Oncology; Health Care Sciences & Services; Rehabilitation
1U7OO 35380268 Green Accepted 2025-06-24
WOS:000779023200001
J Barpanda, A; Tuckley, C; Ray, A; Banerjee, A; Duttagupta, SP; Kantharia, C; Srivastava, S Barpanda,

Abhilash; Tuckley, Chaitanya; Ray, Arka; Banerjee, Arghya; Duttagupta, Siddhartha P.; Kantharia, Chetan; Srivastava, Sanjeeva
A protein microarray-based serum proteomic investigation reveals distinct autoantibody signature in colorectal cancer PROTEOMICS CLINICAL APPLICATIONS
English Article
autoantibody; biological pathway analysis; colorectal cancer; parallel reaction monitoring; protein microarray; protein-protein interaction network; proteomics; targeted proteomics
HUMAN COLON; TUMOR; MIGRATION; CHANNEL; BREAST
PurposeColorectal cancer (CRC) has been reported as the second leading cause of cancer death worldwide. The 5-year annual survival is around 50%, mainly due to late diagnosis, striking necessity for early detection. This study aims to identify autoantibody in patients' sera for early screening of cancer. Experimental DesignThe study used a high-density human proteome array with approximately 17,000 recombinant proteins. Screening of sera from healthy individuals, CRC from Indian origin, and CRC from middle-east Asia origin were performed. Bio-statistical analysis was performed to identify significant autoantibodies altered. Pathway analysis was performed to explore the underlying mechanism of the disease. ResultsThe comprehensive proteomic analysis revealed dysregulation of 15 panels of proteins including CORO7, KCNAB1, WRAP53, NDUFS6, KRT30, and COLGALT2. Further biological pathway analysis for the top dysregulated autoantigenic proteins revealed perturbation in important biological pathways such as ECM degradation and cytoskeletal remodeling etc. Conclusions and Clinical RelevanceThe generation of an autoimmune response against cancer-linked pathways could be linked to the screening of the disease. The process of immune surveillance can be detected at an early stage of cancer. Moreover, AAbs can be easily extracted from blood serum through the least invasive test for disease screening. [Barpanda, Abhilash; Tuckley, Chaitanya; Ray, Arka; Duttagupta, Siddhartha P.; Srivastava, Sanjeeva] Indian Inst Technol, Ctr Res Nanotechnol & Sci CRNTS, Mumbai, India; [Barpanda, Abhilash; Banerjee, Arghya; Srivastava, Sanjeeva] Indian Inst Technol, Dept Biosci & Bioengn, Mumbai, India; [Kantharia, Chetan] Dept Surg gastroenterol King Edward Mem Hosp, Seth G S Med Coll, Mumbai, India; [Srivastava, Sanjeeva] Indian Inst Technol, Dept Biosci & Bioengn, Mumbai 400076, India
Indian Institute of Technology System (IIT System); Indian Institute of Technology (IIT) - Bombay; Indian Institute of Technology System (IIT System); Indian Institute of Technology (IIT) - Bombay; Seth Gordhandas Sunderdas Medical College & King Edward Memorial Hospital; Indian Institute of Technology System (IIT System); Indian Institute of Technology (IIT) - Bombay
Srivastava, S (corresponding author), Indian Inst Technol, Dept Biosci & Bioengn, Mumbai 400076, India. sanjeeva@iitb.ac.in
Srivastava, Sanjeeva/0000-0001-5159-6834 DBT India [BT/PR41020/COT/142/14/2020]; CSIR fellowship, India; IIT Bombay; Merck; [BT/PR13114/INF/22/206/2015]; [15IRAWD010] DBT India(Department of Biotechnology (DBT) India); CSIR fellowship, India; IIT Bombay; Merck(Merck & Company); ; The authors would like to acknowledge CDI LABS for providing the high-density HuProt (TM) protein array, MASSFIIT Facility, IIT Bombay supported by DBT India (BT/PR13114/INF/22/206/2015). Seed fund to SS (IIT Bombay-15IRAWD010) and a Center of Excellence supported by Merck (DO/2021-MLSP) is acknowledged and (BT/PR41020/COT/142/14/2020) to

S.S., A.B. is supported by CSIR fellowship, India for Ph.D.
69 8 8 0 9 WILEY-V C H VERLAG GMBH
WEINHEIM POSTFACH 101161, 69451 WEINHEIM, GERMANY
1862-8346 1862-8354 PROTEOM CLIN APPL Proteom. Clin. Appl. MAR 2023 17 2
10.1002/prca.202200062
http://dx.doi.org/10.1002/prca.202200062 NOV 2022
15 Biochemical Research Methods; Biochemistry & Molecular Biology Science Citation Index Expanded (SCI-EXPANDED)
Biochemistry & Molecular Biology X6OV5 36408811
2025-06-24 WOS:000892022200001
J Assidicky, R; Tokat, UM; Tarman, IO; Saatci, O; Ersan, PG; Raza, U; Ogul, H; Riazalhosseini, Y; Can, T; Sahin, O
Assidicky, Ridho; Tokat, Unal Metin; Tarman, Ibrahim Oguzhan; Saatci, Ozge; Ersan, Pelin Gulizar; Raza, Umar; Ogul, Hasan; Riazalhosseini, Yasser; Can, Tolga; Sahin, Ozgur
Targeting HIF1-alpha/miR-326/ITGA5 axis potentiates chemotherapy response in triple-negative breast cancer BREAST CANCER RESEARCH AND TREATMENT English Article
TNBC; miRNA-mRNA network; Chemoresistance; miR-326; Fibronectin; Integrin; Hypoxia; ECM stiffness CELL LUNG-CANCER; HYPOXIA; RESISTANCE; CARCINOMA; INVASION; PROLIFERATION; DOXORUBICIN; SURVIVAL; SEQUENCE; TUMORS
Purpose Triple-negative breast cancer (TNBC) is the most aggressive subtype of breast cancer that is frequently treated with chemotherapy. However, many patients exhibit either de novo chemoresistance or ultimately develop resistance to chemotherapy, leading to significantly high mortality rates. Therefore, increasing the efficacy of chemotherapy has potential to improve patient outcomes. Methods Here, we performed whole transcriptome sequencing (both RNA and small RNA-sequencing), coupled with network simulations and patient survival data analyses to build a novel miRNA-mRNA interaction network governing chemoresistance in TNBC. We performed cell proliferation assay, Western blotting, RNAi/miRNA mimic experiments, FN coating, 3D cultures, and ChIP assays to validate the interactions in the network, and their functional roles in chemoresistance. We developed xenograft models to test the therapeutic potential of the identified key miRNA/proteins in potentiating chemoresponse in vivo. We also analyzed several patient datasets to evaluate the clinical relevance of our findings. Results We identified fibronectin (FN1) as a central chemoresistance driver gene. Overexpressing miR-326 reversed FN1-driven chemoresistance by targeting FN1 receptor, ITGA5. miR-326 was downregulated by increased hypoxia/HIF1A and ECM stiffness in chemoresistant tumors, leading to upregulation of ITGA5 and activation of the downstream FAK/Src signaling pathways. Overexpression of miR-326 or inhibition of ITGA5 overcame FN1-driven chemotherapy resistance in vitro by inhibiting FAK/Src pathway and potentiated the efficacy of chemotherapy in vivo. Importantly, lower expression of miR-326 or higher levels of predicted miR-326 target genes was significantly associated with worse overall survival in chemotherapy-treated TNBC patients. Conclusion FN1 is central in chemoresistance. In chemoresistant tumors, hypoxia and resulting ECM stiffness repress the expression of the tumor suppressor miRNA, miR-326. Hence, re-expression of miR-326 or inhibition of its target ITGA5 reverses FN1-driven chemoresistance making them attractive therapeutic approaches to enhance chemotherapy response in TNBCs.
[Assidicky, Ridho; Tokat,

Unal Metin; Tarman, Ibrahim Oguzhan; Ersan, Pelin Gulizar; Raza, Umar] Bilkent Univ, Fac Sci, Dept Mol Biol & Genet, TR-06800 Ankara, Turkey; [Saatci, Ozge; Sahin, Ozgur] Univ South Carolina, Dept Drug Discovery & Biomed Sci, Columbia, SC 29208 USA; [Ogul, Hasan] Ostfold Univ Coll, Fac Comp Sci, NO-1757 Halden, Norway; [Riazalhosseini, Yasser] McGill Univ, Genome Ctr, Montreal, PQ H3A 0G1, Canada; [Riazalhosseini, Yasser] McGill Univ, Dept Human Genet, Montreal, PQ H3A 1B1, Canada; [Can, Tolga] Middle East Tech Univ, Dept Comp Engn, TR-06800 Ankara, Turkey Ihsan Dogramaci Bilkent University; University of South Carolina System; University of South Carolina Columbia; Ostfold University College; McGill University; McGill University; Middle East Technical University Sahin, O (corresponding author), Univ South Carolina, Dept Drug Discovery & Biomed Sci, Columbia, SC 29208 USA.
sahinozgur@gmail.com Ersan, Pelin/CAJ-0477-2022; Saatci, Ozge/MAI-4907-2025; Tokat, Ünal/ITT-0453-2023; Can, Tolga/D-1395-2010; Raza, Umar/Q-4978-2019; Sahin, Ozgur/F-4403-2014 Raza, Umar/0000-0002-0081-6996; Tokat, Unal Metin/0000-0003-0026-368X; Sahin, Ozgur/0000-0002-8033-7089; Ersan, Pelin/0000-0003-3637-4090
European Commission FP7 Marie Curie Career Integration Grant [PCIG14-GA-2013-631149]; American Cancer Society Institutional Research Grant [IRG17-179-04, R01-CA267101, 2P20GM109091-06]; Higher Education Commission of Pakistan; TUBITAK Domestic PhD Scholarship Program (UMT) [2211/A]; Susan G. Komen Interdisciplinary Graduate Training to Eliminate Cancer Disparities [(IGniTE-CD) GTDR17500160] European Commission FP7 Marie Curie Career Integration Grant; American Cancer Society Institutional Research Grant(American Cancer Society); Higher Education Commission of Pakistan(Higher Education Commission of Pakistan); TUBITAK Domestic PhD Scholarship Program (UMT)(Turkiye Bilimsel ve Teknolojik Arastirma Kurumu (TUBITAK)); Susan G. Komen Interdisciplinary Graduate Training to Eliminate Cancer Disparities(Susan G. Komen Breast Cancer Foundation) This work was supported by European Commission FP7 Marie Curie Career Integration Grant PCIG14-GA-2013-631149 (OS), the American Cancer Society Institutional Research Grant IRG17-179-04 (OS), NIH Research Project Grants R01-CA267101 (OS) and 2P20GM109091-06 (OS), the scholarship from Higher Education Commission of Pakistan (UR), the scholarship from 2211/A TUBITAK Domestic PhD Scholarship Program (UMT), and Susan G. Komen Interdisciplinary Graduate Training to Eliminate Cancer Disparities (IGniTE-CD) GTDR17500160 (OzgeS). YR is a research scholar of Fonds de recherche du Quebec-Sante (FRQS). 63 35 35 2 9 SPRINGER NEW YORK ONE NEW YORK PLAZA, SUITE 4600, NEW YORK, NY, UNITED STATES
0167-6806 1573-7217 BREAST CANCER RES TR Breast Cancer Res. Treat. JUN 2022 193 2
331 348 10.1007/s10549-022-06569-5
http://dx.doi.org/10.1007/s10549-022-06569-5 MAR 2022
18 Oncology Science Citation Index Expanded (SCI-EXPANDED) Oncology 1C6RB 35338412 Green Accepted
2025-06-24 WOS:000783078500003
J Pandurangan, AK; Ganapasam, S
Pandurangan, Ashok Kumar; Ganapasam, Sudhandiran
Cytotoxic Effect of Luteolin on Human Colorectal Cancer Cell Line (HCT-15): Crucial Involvement of Reactive Oxygen Species
MIDDLE EAST JOURNAL OF CANCER English
Article Colon cancer; Luteolin; ROS; HCT-15 INDUCED APOPTOSIS; NITRIC-OXIDE;

INHIBITION; INDUCTION; KINASE Background: Colorectal cancer, a major health concern worldwide, is the third most common form of cancer and second leading cause of cancer-related deaths. The flavonoids are naturally occurring diphenylpropanoids ubiquitous in plant foods and important components of the human diet. Luteolin, a bioflavonoid, possesses many beneficial effects including antioxidant, anti-inflammatory, anti-allergic activities. Methods: We used the HCT-15 colon adenocarcinoma cell line in this study. Cells were treated with luteolin (100 mu M). Results: Membrane damage markers such as alkaline phosphatase and lactate dehydrogenase were analyzed in a time-dependent manner. Luteolin increased reactive oxygen species in a time-dependent manner. DNA damage, a hallmark of apoptosis, was induced by luteolin as analyzed by agarose gel electrophoresis. Conclusion: Luteolin acts as a potential cytotoxic agent that can be used to treat colorectal cancer. [Pandurangan, Ashok Kumar; Ganapasam, Sudhandiran] Univ Madras, Dept Biochem, Madras, Tamil Nadu, India University of Madras Pandurangan, AK (corresponding author), Univ Madras, Dept Biochem, Sardar Patel Rd, Madras 600025, Tamil Nadu, India. panduashokkumar@gmail.com Ganapasam, Sudhandiran/AAD-4264-2021; Pandurangan, Ashok/M-7335-2013 28 5 5 0 1 SHIRAZ UNIV MEDICAL SCIENCES SHIRAZ NEMAZEE HOSPITAL, SHIRAZ, 71934, IRAN 2008-6709 2008-6687 MIDDLE EAST J CANCER Middle East J. Cancer OCT 2013 4 4 175 180 6 Oncology Emerging Sources Citation Index (ESCI) Oncology V34EK 2025-06-24 WOS:000215816100004

J Branca, JJV; Pacini, S; Ruggiero, M Branca, Jacopo J. V.; Pacini, Stefania; Ruggiero, Marco Effects of Pre-surgical Vitamin D Supplementation and Ketogenic Diet in a Patient with Recurrent Breast Cancer ANTICANCER RESEARCH English Article Vitamin D-3; ketogenic diet; breast cancer MONOUNSATURATED FATTY-ACID; SERUM 25-HYDROXYVITAMIN D; PROTEIN-KINASE-C; OLEIC-ACID; MEDITERRANEAN DIET; OLIVE OIL; CELLS; COMPLEXES; RISK; METAANALYSIS Background: A woman, mother of one at the age of 19 years, was diagnosed with mammary adenocarcinoma in the right breast in 1985 at the age of 37 years. The patient underwent surgery (quadrantectomy), lymphadenectomy and radiotherapy. In 1999, an adenocarcinoma was diagnosed in the left breast, followed by adequate resection, radiotherapy and anti-oestrogen receptor treatment for 6 years. In March 2014, an infiltrating adenocarcinoma was diagnosed in the remaining part of the right breast that had been operated on and irradiated in 1985. Case Report: The pre-surgical biopsy, showed weak positivity for progesterone receptor (PgR) (<1%), high positivity for oestrogen receptor (ER) (90%), high positivity for human epidermal growth factor receptor (HER2) (>10%, score 2+), and high positivity for the nuclear protein Ki67 (30%). In the three weeks between diagnosis and operation, when no other treatment had been planned, the patient decided to self-administer high doses of oral vitamin D-3 (10,000 IU/day), and to follow a strict ketogenic diet. Results: Following right mastectomy, analysis of the surgical specimen showed no positivity for HER2 expression (negative, score 0), and significant increase in positivity of PgR (20%). Positivity for ER and Ki67 were unaltered. Conclusion: This observation indicates that a combination of high-dose vitamin D-3

and ketogenic diet leads to changes in some biological markers of breast cancer, i.e. negativization of HER2 expression and increased expression of PgR.[Branca, Jacopo J. V.; Pacini, Stefania] Univ Florence, Dept Expt & Clin Med, I-50134 Florence, Italy; [Ruggiero, Marco] Univ Florence, Dept Expt & Clin Biomed Sci, I-50134 Florence, ItalyUniversity of Florence; University of Florence	Branca, JJV (corresponding author), Univ Florence, Dept Expt & Clin Med, Largo Brambilla 3, I-50134 Florence, Italy.
	jacopo.branca@libero.it	Branca, Jacopo Junio Valerio/AAJ-1593-2020	Branca, Jacopo Junio Valerio/0000-0003-3179-0706	33	30	32	0	24	INT INST ANTICANCER RESEARCH	ATHENS	EDITORIAL OFFICE 1ST KM KAPANDRITIOU-KALAMOU RD KAPANDRITI, PO BOX 22, ATHENS 19014, GREECE	0250-7005	1791-7530	ANTICANCER RES
	Anticancer Res.	OCT	2015	35	10
	5525	5532	8	Oncology
	Science Citation Index Expanded (SCI-EXPANDED)	Oncology
	CS1KM	26408720	2025-06-24
	WOS:000361823200048
J	Jandu, HK; Veal, CD; Fachal, L; Luccarini, C; Aguado-Barrera, ME; Altabas, M; Azria, D; Baten, A; Bourgier, C; Bultijnck, R; Colciago, RR; Farcy-Jacquet, MP; Chang-Claude, J; Choudhury, A; Dunning, A; Elliott, RM; Green, S; Gutiérrez-Enríquez, S; Herskind, C; Lambrecht, M; Monten, C; Rancati, T; Reyes, V; Rosenstein, BS; De Ruysscher, D; De Santis, MC; Seibold, P; Sperk, E; Veldwijk, M; Symonds, RP; Stobart, H; Taboada-Valladares, B; Vega, A; Veldeman, L; Webb, AJ; Weltens, C; West, CM; Rattay, T; Talbot, CJ	Jandu, Harkeran K.; Veal, Colin D.; Fachal, Laura; Luccarini, Craig; Aguado-Barrera, Miguel E.; Altabas, Manuel; Azria, David; Baten, Adinda; Bourgier, Celine; Bultijnck, Renee; Colciago, Riccardo R.; Farcy-Jacquet, Marie-Pierre; Chang-Claude, Jenny; Choudhury, Ananya; Dunning, Alison; Elliott, Rebecca M.; Green, Sheryl; Gutierrez-Enriquez, Sara; Herskind, Carsten; Lambrecht, Maarten; Monten, Christel; Rancati, Tiziana; Reyes, Victoria; Rosenstein, Barry S.; De Ruysscher, Dirk; De Santis, Maria Carmen; Seibold, Petra; Sperk, Elena; Veldwijk, Marlon; Symonds, R. Paul; Stobart, Hilary; Taboada-Valladares, Begona; Vega, Ana; Veldeman, Liv; Webb, Adam J.; Weltens, Caroline; West, Catharine M.; Rattay, Tim; Talbot, Christopher J.	REQUITE Consortium	Genome-wide association study of treatment-related toxicity two years following radiotherapy for breast cancer	RADIOTHERAPY AND ONCOLOGY	English	Article
	Radiogenomics; Genome-wide association study; Chronic toxicity; Radiotherapy side effects; Radiotherapy; Breast Cancer
	RADIATION; POLYMORPHISMS; DERMATITIS; GENES; RISK; GWAS
	Background and purpose: Up to a quarter of breast cancer patients treated by surgery and radiotherapy experience clinically significant toxicity. If patients at high risk of adverse effects could be identified at diagnosis, their treatment could be tailored accordingly. This study was designed to identify common single nucleotide polymorphisms (SNPs) associated with toxicity two years following whole breast radiotherapy. Materials and Methods: A genome-wide association study (GWAS) was performed in 1,640 breast cancer patients with complete SNP, clinical, treatment and toxicity data, recruited across 18 European and US centres into the prospective REQUITE cohort study. Toxicity data (CTCAE v4.0) were collected at baseline, end of radiotherapy, and

annual follow-up. A total of 7,097,340 SNPs were tested for association with the residuals of toxicity endpoints, adjusted for clinical, treatment co-variates and population substructure. Results: Quantile-quantile plots showed more associations with toxicity above the p 5 x 10-5 level than expected by chance. Eight SNPs reached genome-wide significance. Nipple retraction grade 2 was associated with the rs188287402 variant (p = 2.80 x 10-8), breastoedema grade > 2 with rs12657177 (p = 1. 12 x 10-10), rs75912034 (p = 1.12 x 10-10), rs145328458 (p = 1.06 x 10-9) and rs61966612 (p = 1.23 x 10- 9), induration grade > 2 with rs77311050 (p = 2.54 x 10-8) and rs34063419 (p = 1.21 x 10-8), and arm lymphoedema grade > 1 with rs643644 (p = 3.54 x 10-8). Heritability estimates across significant endpoints ranged from 25% to 39%. Our study did not replicate previously reported SNPs associated with breast radiation toxicity at the pre-specified significance level. Conclusions: This GWAS for long-term breast radiation toxicity provides further evidence for significant association of common SNPs with distinct toxicity endpoints. (c) 2023 The Authors. Published by Elsevier B.V. Radiotherapy and Oncology 187 (2023) 1-10 This is an open access article under the CC BY license (http://creativecommons.org/licenses/by/4.0/).

[Jandu, Harkeran K.; Veal, Colin D.; Webb, Adam J.; Talbot, Christopher J.] Univ Leicester, Dept Genet & Genome Biol, Leicester, England; [Fachal, Laura] Wellcome Sanger Inst, Wellcome Genome Campus, Hinxton, England; [Luccarini, Craig; Dunning, Alison; Vega, Ana] Univ Cambridge, Strangeways Res Lab, Ctr Canc Genet Epidemiol, Cambridge, England; [Aguado-Barrera, Miguel E.; Taboada-Valladares, Begona; Vega, Ana] Inst Invest Sanitaria Santiago De Compostela IDIS, Santiago De Compostela, Spain; [Aguado-Barrera, Miguel E.] Fdn Publ Galega Med Xenom, Santiago De Compostela, Spain; [Altabas, Manuel; Reyes, Victoria] Vall dHebron Hosp Univ, Vall dHebron Barcelona Hosp Campus, Dept Radiat Oncol, Barcelona, Spain; [Azria, David; Bourgier, Celine] Univ Montpellier, Univ Federat Radiat Oncol Mediterranean Occitanie, Inst Rech Cancerol Montpellier, INSERM U1194 IRCM, Montpellier, France; [Baten, Adinda; Lambrecht, Maarten; Weltens, Caroline] UZ Leuven, Radiat Oncol, Leuven, Belgium; [Bultijnck, Renee; Veldeman, Liv] Univ Ghent, Dept Human Struct & Repair, Ghent, Belgium; [Colciago, Riccardo R.; De Santis, Maria Carmen] Fdn IRCCS Ist Nazl Tumori, Unit Radiat Oncol, Milan, Italy; [Farcy-Jacquet, Marie-Pierre] Univ Federat Radiat Oncol Mediterranean Occitanie, Inst Cancerol Gard, CHU Caremeau, Nimes, France; [Chang-Claude, Jenny; Seibold, Petra] German Canc Res Ctr, Div Canc Epidemiol, Heidelberg, Germany; [Chang-Claude, Jenny] Univ Med Ctr Hamburg Eppendorf, Univ Canc Ctr Hamburg, Canc Epidemiol Grp, Hamburg, Germany; [Choudhury, Ananya; Elliott, Rebecca M.; West, Catharine M.] Univ Manchester, Manchester Acad, Christie Hosp, Translat Radiobiol Grp,Div Canc Sci,Hlth Sci Ctr, Manchester, England; [Green, Sheryl; Rosenstein, Barry S.] Icahn Sch Med Mt Sinai, Dept Radiat Oncol, New York, NY USA; [Gutierrez-Enriquez, Sara] Vall dHebron Barcelona Hosp Campus, Vall dHebron Inst Oncol VHIO, Hereditary Canc Genet Grp, Barcelona, Spain; [Herskind, Carsten; Sperk, Elena; Veldwijk, Marlon] Univ Med Mannheim, Univ Heidelberg, Med Fac Mannheim, Dept Radiat Oncol, Mannheim, Germany; [Monten, Christel; Veldeman, Liv] Ghent Univ Hosp, Dept Radiat Oncol, Ghent, Belgium; [Rancati, Tiziana] Fdn IRCCS Ist Nazl Tumori, Unit Data Sci, Milan, Italy; [De Ruysscher, Dirk] Maastricht Univ, GROW Sch Oncol & Dev Biol, MAASTRO Clin,

Med Ctr, Maastricht, Netherlands; [Symonds, R. Paul; Rattay, Tim] Univ Leicester, Leicester Canc Res Ctr, Leicester, England; [Taboada-Valladares, Begona] Complexo Hosp Univ Santiago, Dept Radiat Oncol, SERGAS, Santiago De Compostela, Spain
University of Leicester; Wellcome Trust Sanger Institute; University of Cambridge; Institut National de la Sante et de la Recherche Medicale (Inserm); Universite de Montpellier; KU Leuven; University Hospital Leuven; Ghent University; Fondazione IRCCS Istituto Nazionale Tumori Milan; Universite de Montpellier; CHU de Nimes; Helmholtz Association; German Cancer Research Center (DKFZ); University of Hamburg; University Medical Center Hamburg-Eppendorf; Christie NHS Foundation Trust; Christie Hospital; University of Manchester; Icahn School of Medicine at Mount Sinai; Vall d'Hebron Institut d'Oncologia (VHIO); Ruprecht Karls University Heidelberg; Ghent University; Ghent University Hospital; Fondazione IRCCS Istituto Nazionale Tumori Milan; Maastricht University; Maastricht University Medical Centre (MUMC); University of Leicester; Complexo Hospitalario Universitario de Santiago de Compostela Rattay, T (corresponding author), Univ Leicester, Leicester Canc Res Ctr, Leicester Royal Infirm, Clin Sci Bldg, Leicester LE2 7LX, England. tr104@le.ac.uk
Choudhury, Ananya/JKH-6416-2023; De Santis, Maria/E-8649-2017; Webb, Adam/A-1478-2009; Lambrecht, Maarten/H-1624-2011; Gutierrez, Sara/GXE-9832-2022; Vega, Ana/R-4758-2019; Rancati, Tiziana/K-7921-2016; Sperk, Elena/KHD-6997-2024; Colciago, Riccardo/LRU-7354-2024; West, Catharine/J-4152-2012; Bultijnck, Renee/J-2445-2016; Aguado-Barrera, Miguel E/J-3688-2017
Bultijnck, Renee/0000-0003-4122-2323; REYES LOPEZ, VICTORIA/0009-0000-1124-5028; Lambrecht, Maarten/0000-0002-8746-2691; Aguado-Barrera, Miguel E/0000-0002-7822-6726; Colciago, Riccardo Ray/0000-0002-3867-2844; Monten, Chris/0000-0003-2599-7313; Vega, Ana/0000-0002-7416-5137; Sperk, Elena/0000-0002-8771-8124 27 4 4 0 3 ELSEVIER IRELAND LTD CLARE ELSEVIER HOUSE, BROOKVALE PLAZA, EAST PARK SHANNON, CO, CLARE, 00000, IRELAND 0167-8140 1879-0887
RADIOTHER ONCOL Radiother. Oncol. OCT 2023 187
109806
10.1016/j.radonc.2023.109806
http://dx.doi.org/10.1016/j.radonc.2023.109806 AUG 2023 10 Oncology; Radiology, Nuclear Medicine & Medical Imaging Science Citation Index Expanded (SCI-EXPANDED)
Oncology; Radiology, Nuclear Medicine & Medical Imaging
S1DM0 37437607 Green Published, hybrid
2025-06-24 WOS:001068641900001
J Fitzmaurice, C; Alsharif, U; El Bcheraoui, C; Khalil, I; Charara, R; Moradi-Lakeh, M; Afshin, A; Collison, M; Chew, A; Krohn, KJ; Daoud, F; Dicker, D; Foreman, KJ; Frostad, J; Kassebaum, NJ; Kutz, M; Wang, HD; Abyu, GY; Adedeji, IA; Kiadaliri, AA; Ahmed, MB; Al-Eyadhy, A; Alam, K; Alasfoor, D; Ali, R; Alizadeh-Navaei, R; Al-Raddadi, R; Altirkawi, KA; Alvis-Guzman, N; Amini, E; Anber, N; Anwari, P; Artaman, A; Asgedom, SW; Atey, TM; Awasthi, A; Saleem, HOB; Bacha, U; Barac, A; Bedi, N; Bhutta, ZA; Butt, ZA; Castañeda-Orjuela, CA; Chitheer, AA; Danawi, H; das Neves, J; Davitoiu, DV; Dey, S; Dharmaratne, SD; Djalalinia, S; Do, HP; Dubey, M; Ebrahimi, H; Ekwueme, DU; Endries, AY; Eshrati, B; Esteghamati, A; Farvid, MS; Fereshtehnejad, SM; Fischer, F; Gebrehiwot, TT; Gopalani, SV; Hafezi-Nejad, N; Hamadeh, RR; Hamidi, S; Hareri, HA; Hay, RJ; Horita, N; Hsairi, M; Jakovljevic,

MB; Jonas, JB; Kasaeian, A; Kassaw, NA; Khader, YS; Khan, EA; Khan, G; Kim, D; Kinfu, Y; Larson, HJ; Latif, AA; Linn, S; Lunevicius, R; Abd El Razek, HM; Abd El Razek, MM; Majeed, A; Malekzadeh, R; Malta, DC; Markos, D; Memiah, P; Memish, ZA; Mendoza, W; Meretoja, TJ; Miller, TR; Mohammed, S; Nangia, V; Nguyen, QL; Nguyen, TH; Ogbo, FA; Mahesh, PA; Park, EK; Patel, T; Pereira, DM; Pishgar, F; Pourmalek, F; Qorbani, M; Radfar, A; Rafay, A; Rahimi-Movaghar, V; Rai, RK; Rana, SM; Rawaf, S; Renzaho, AMN; Rezaei, S; Roba, KT; Roshandel, G; Safdarian, M; Safi, S; Safiri, S; Salamati, P; Samy, AM; Sanabria, JR; Milicevic, MMS; Sartorius, B; Sepanlou, SG; Shaikh, MA; Shrime, MG; Stathopoulou, V; Sufiyan, MB; Abdulkader, RS; Tabarés-Seisdedos, R; Tehrani-Banihashemi, A; Tekelab, T; Temsah, MH; Tran, BX; Ukwaja, KN; Uthman, OA; Vlassov, VV; Vollset, SE; Wakayo, T; Weiderpass, E; Werdecker, A; Yaghoubi, M; Yaseri, M; Yimam, HH; Yonemoto, N; Zaki, ME; Zein, B; Jumaan, AO; Vos, T; Hay, SI; Naghavi, M; Murray, CJL; Mokdad, AH

Fitzmaurice, Christina; Alsharif, Ubai; El Bcheraoui, Charbel; Khalil, Ibrahim; Charara, Raghid; Moradi-Lakeh, Maziar; Afshin, Ashkan; Collison, Michael; Chew, Adrienne; Krohn, Kristopher J.; Daoud, Farah; Dicker, Daniel; Foreman, Kyle J.; Frostad, Joseph; Kassebaum, Nicholas J.; Kutz, Michael; Wang, Haidong; Abyu, Gebre Yitayih; Adedeji, Isaac Akinkunmi; Kiadaliri, Aliasghar Ahmad; Ahmed, Muktar Beshir; Al-Eyadhy, Ayman; Alam, Khurshid; Alasfoor, Deena; Ali, Raghib; Alizadeh-Navaei, Reza; Al-Raddadi, Rajaa; Altirkawi, Khalid A.; Alvis-Guzman, Nelson; Amini, Erfan; Anber, Nahla; Anwari, Palwasha; Artaman, Al; Asgedom, Solomon Weldegebreal; Atey, Tesfay Mehari; Awasthi, Ashish; Saleem, Huda Omer Ba; Bacha, Umar; Barac, Aleksandra; Bedi, Neeraj; Bhutta, Zulfiqar A.; Butt, Zahid A.; Castaneda-Orjuela, Carlos A.; Chitheer, Abdulaal A.; Danawi, Hadi; das Neves, Jose; Davitoiu, Dragos V.; Dey, Subhojit; Dharmaratne, Samath D.; Djalalinia, Shirin; Huyen Phuc Do; Dubey, Manisha; Ebrahimi, Hedyeh; Ekwueme, Donatus U.; Endries, Aman Yesuf; Eshrati, Babak; Esteghamati, Alireza; Farvid, Maryam S.; Fereshtehnejad, Seyed-Mohammad; Fischer, Florian; Gebrehiwot, Tsegaye Tewelde; Gopalani, Sameer Vali; Hafezi-Nejad, Nima; Hamadeh, Randah Ribhi; Hamidi, Samer; Hareri, Habtamu Abera; Hay, Roderick J.; Horita, Nobuyuki; Hsairi, Mohamed; Jakovljevic, Mihajlo B.; Jonas, Jost B.; Kasaeian, Amir; Kassaw, Nigussie Assefa; Khader, Yousef Saleh; Khan, Ejaz Ahmad; Khan, Gulfaraz; Kim, Daniel; Kinfu, Yohannes; Larson, Heidi J.; Latif, Asma Abdul; Linn, Shai; Lunevicius, Raimundas; Abd El Razek, Hassan Magdy; Abd El Razek, Mohammed Magdy; Majeed, Azeem; Malekzadeh, Reza; Malta, Deborah Carvalho; Markos, Desalegn; Memiah, Peter; Memish, Ziad A.; Mendoza, Walter; Meretoja, Tuomo J.; Miller, Ted R.; Mohammed, Shafiu; Nangia, Vinay; Quyen Le Nguyen; Trang Huyen Nguyen; Ogbo, Felix Akpojene; Mahesh, P. A.; Park, Eun-Kee; Patel, Tejas; Pereira, David M.; Pishgar, Farhad; Pourmalek, Farshad; Qorbani, Mostafa; Radfar, Amir; Rafay, Anwar; Rahimi-Movaghar, Vafa; Rai, Rajesh Kumar; Rana, Saleem M.; Rawaf, Salman; Renzaho, Andre M. N.; Rezaei, Satar; Roba, Kedir Teji; Roshandel, Gholamreza; Safdarian, Mahdi; Safi, Sare; Safiri, Saeid; Salamati, Payman; Samy, Abdallah M.; Sanabria, Juan Ramon; Milicevic, Milena M. Santric; Sartorius, Benn; Sepanlou, Sadaf G.; Shaikh, Masood Ali; Shrime, Mark G.; Stathopoulou, Vasiliki; Sufiyan, Muawiyyah Babale; Abdulkader, Rizwan Suliankatchi; Tabares-Seisdedos, Rafael; Tehrani-Banihashemi, Arash; Tekelab, Tesfalidet; Temsah, Mohamad-Hani;

Tran, Bach Xuan; Ukwaja, Kingsley Nnanna; Uthman, Olalekan A.; Vlassov, Vasiliy Victorovich; Vollset, Stein Emil; Wakayo, Tolassa; Weiderpass, Elisabete; Werdecker, Andrea; Yaghoubi, Mohsen; Yaseri, Mehdi; Yimam, Hassen Hamid; Yonemoto, Naohiro; Zaki, Maysaa El Sayed; Zein, Bassel; Jumaan, Aisha O.; Vos, Theo; Hay, Simon I.; Naghavi, Mohsen; Murray, Christopher J. L.; Mokdad, Ali H. GBD 2015 Eastern Mediterranean Reg Burden of cancer in the Eastern Mediterranean Region, 2005-2015: findings from the Global Burden of Disease 2015 Study INTERNATIONAL JOURNAL OF PUBLIC HEALTH English Article
Eastern Mediterranean Region; Cancer; Mortality; Incidence; Disability-adjusted life years DEATH To estimate incidence, mortality, and disability-adjusted life years (DALYs) caused by cancer in the Eastern Mediterranean Region (EMR) between 2005 and 2015. Vital registration system and cancer registry data from the EMR region were analyzed for 29 cancer groups in 22 EMR countries using the Global Burden of Disease Study 2015 methodology. In 2015, cancer was responsible for 9.4% of all deaths and 5.1% of all DALYs. It accounted for 722,646 new cases, 379,093 deaths, and 11.7 million DALYs. Between 2005 and 2015, incident cases increased by 46%, deaths by 33%, and DALYs by 31%. The increase in cancer incidence was largely driven by population growth and population aging. Breast cancer, lung cancer, and leukemia were the most common cancers, while lung, breast, and stomach cancers caused most cancer deaths. Cancer is responsible for a substantial disease burden in the EMR, which is increasing. There is an urgent need to expand cancer prevention, screening, and awareness programs in EMR countries as well as to improve diagnosis, treatment, and palliative care services. [GBD 2015 Eastern Mediterranean Reg] Inst Hlth Metr & Evaluat, Div Hematol, Dept Med, 2301 5th Ave,Suite 600,UW Campus,Mailbox 358210, Seattle, WA 98121 USA; [Fitzmaurice, Christina; El Bcheraoui, Charbel; Khalil, Ibrahim; Charara, Raghid; Afshin, Ashkan; Collison, Michael; Chew, Adrienne; Krohn, Kristopher J.; Daoud, Farah; Dicker, Daniel; Foreman, Kyle J.; Frostad, Joseph; Kassebaum, Nicholas J.; Kutz, Michael; Wang, Haidong; Larson, Heidi J.; Vollset, Stein Emil; Vos, Theo; Hay, Simon I.; Naghavi, Mohsen; Murray, Christopher J. L.; Mokdad, Ali H.] Univ Washington, Inst Hlth Metr & Evaluat, Seattle, WA 98195 USA; [Alsharif, Ubai] Charite, Berlin, Germany; [Charara, Raghid] Amer Univ Beirut, Beirut, Lebanon; [Moradi-Lakeh, Maziar] Iran Univ Med Sci, Dept Community Med, Prevent Med & Publ Hlth Res Ctr, Gastrointestinal & Liver Dis Res Ctr GILDRC, Tehran, Iran; [Foreman, Kyle J.; Rawaf, Salman] Imperial Coll London, London, England; [Kassebaum, Nicholas J.] Dept Anesthesiol & Pain Med, Seattle, WA USA; [Kassebaum, Nicholas J.] Seattle Childrens Hosp, Seattle, WA USA; [Abyu, Gebre Yitayih; Asgedom, Solomon Weldegebreal; Atey, Tesfay Mehari] Mekelle Univ, Mekelle, Ethiopia; [Adedeji, Isaac Akinkunmi] Olabisi Onabanjo Univ, Ago Iwoye, Nigeria; [Kiadaliri, Aliasghar Ahmad] Lund Univ, Dept Clin Sci Lund, Clin Epidemiol Unit, Lund, Sweden; [Ahmed, Muktar Beshir] Jimma Univ, Coll Hlth Sci, Dept Epidemiol, Jimma, Ethiopia; [Al-Eyadhy, Ayman; Altirkawi, Khalid A.; Temsah, Mohamad-Hani] King Saud Univ, Riyadh, Saudi Arabia; [Alam, Khurshid] Univ Melbourne, Murdoch Childrens Res Inst, Parkville, Vic, Australia; [Alam, Khurshid] Univ Melbourne, Melbourne, Vic, Australia; [Alam, Khurshid] Univ Sydney, Sydney, NSW, Australia; [Alasfoor, Deena] Minist Hlth, Al Khuwair, Oman; [Ali, Raghib]

Univ Oxford, Oxford, England; [Alizadeh-Navaei, Reza] Mazandaran Univ Med Sci, Gastrointestinal Canc Res Ctr, Sari, Iran; [Al-Raddadi, Rajaa] Joint Program Family & Community Med, Jeddah, Saudi Arabia; [Alvis-Guzman, Nelson] Univ Cartagena, Cartagena De Indias, Colombia; [Amini, Erfan] Univ Tehran Med Sci, Urooncol Res Ctr, Tehran, Iran; [Amini, Erfan] Univ Tehran Med Sci, Endocrinol & Metab Res Inst, Noncommunicable Dis Res Ctr, Tehran, Iran; [Anber, Nahla] Mansoura Univ, Mansoura, Egypt; [Artaman, Al] Univ Manitoba, Winnipeg, MB, Canada; [Awasthi, Ashish] Sanjay Gandhi Postgrad Inst Med Sci, Lucknow, Uttar Pradesh, India; [Saleem, Huda Omer Ba] Aden Univ, Fac Med & Hlth Sci, Aden, Yemen; [Bacha, Umar] Univ Management & Technol, Sch Hlth Sci, Lahore, Pakistan; [Barac, Aleksandra] Univ Belgrade, Fac Med, Belgrade, Serbia; [Bedi, Neeraj] Coll Publ Hlth & Trop Med, Jazan, Saudi Arabia; [Bhutta, Zulfiqar A.] Aga Khan Univ, Ctr Excellence Women & Child Hlth, Karachi, Pakistan; [Bhutta, Zulfiqar A.] Hosp Sick Children, Ctr Global Child Hlth, Toronto, ON, Canada; [Butt, Zahid A.] Al Shifa Trust Eye Hosp, Rawalpindi, Pakistan; [Castaneda-Orjuela, Carlos A.] Colombian Natl Hlth Observ, Inst Nacl Salud, Bogota, Colombia; [Castaneda-Orjuela, Carlos A.] Univ Nacl Colombia, Publ Hlth Dept, Epidemiol & Publ Hlth Evaluat Grp, Bogota, Colombia; [Chitheer, Abdulaal A.] Minist Hlth, Baghdad, Iraq; [Danawi, Hadi] Walden Univ, Minneapolis, MN USA; [das Neves, Jose] Univ Porto, I3S, Porto, Portugal; [das Neves, Jose] Univ Porto, INEB Inst Engn Biomed, Porto, Portugal; [Davitoiu, Dragos V.] Univ Med & Pharm Bucharest, Bucharest, Romania; [Dey, Subhojit] Publ Hlth Fdn India, Indian Inst Publ Hlth Delhi, Gurgaon, India; [Dharmaratne, Samath D.] Univ Peradeniya, Fac Med, Dept Community Med, Peradeniya, Sri Lanka; [Djalalinia, Shirin] Minist Hlth & Med Educ, Undersecretary Res & Technol, Tehran, Iran; [Huyen Phuc Do] Duy Tan Univ, Inst Global Hlth Innovat, Da Nang, Vietnam; [Dubey, Manisha] Int Inst Populat Sci, Bombay, Maharashtra, India; [Amini, Erfan; Pereira, David M.] Univ Tehran Med Sci, Noncommunicable Dis Res Ctr, Tehran, Iran; [Ebrahimi, Hedyeh] Univ Tehran Med Sci, Digest Dis Res Inst, Shariati Hosp, Liver & Pancreaticobiliary Dis Res Ctr, Tehran, Iran; [Ekwueme, Donatus U.] Ctr Dis Control & Prevent, Atlanta, GA USA; [Endries, Aman Yesuf] Arba Minch Univ, Arba Minch, Ethiopia; [Eshrati, Babak] Minist Hlth & Med Educ, Tehran, Iran; [Eshrati, Babak] Arak Univ Med Sci, Arak, Iran; [Esteghamati, Alireza] Univ Tehran Med Sci, Endocrinol & Metab Res Ctr, Tehran, Iran; [Farvid, Maryam S.] Harvard Univ, Harvard TH Chan Sch Publ Hlth, Dept Nutr, Boston, MA USA; [Farvid, Maryam S.] Massachusetts Gen Hosp, Mongan Inst Hlth Policy, Harvard MGH Ctr Genom Vulnerable Populat & Hlth D, Boston, MA 02114 USA; [Fereshtehnejad, Seyed-Mohammad] Karolinska Inst, Dept Neurobiol Care Sci & Soc NVS, Stockholm, Sweden; [Fischer, Florian] Univ Bielefeld, Sch Publ Hlth, Bielefeld, Germany; [Gebrehiwot, Tsegaye Tewelde; Wakayo, Tolassa] Jimma Univ, Jimma, Ethiopia; [Gopalani, Sameer Vali] Govt Federated States Micronesia, Dept Hlth & Social Affairs, Palikir, Micronesia; [Hafezi-Nejad, Nima] Univ Tehran Med Sci, Endocrinol & Metab Res Ctr, Tehran, Iran; [Hamadeh, Randah Ribhi] Arabian Gulf Univ, Manama, Bahrain; [Hamidi, Samer] Hamdan Bin Mohammed Smart Univ, Dubai, U Arab Emirates; [Hareri, Habtamu Abera; Kassaw, Nigussie Assefa] Addis Ababa Univ, Addis Ababa, Ethiopia; [Hay, Roderick J.] Int Fdn Dermatol, London, England; [Hay, Roderick J.] Kings Coll London, London, England; [Horita, Nobuyuki] Yokohama City Univ, Dept Pulmonol, Grad Sch Med, Yokohama, Kanagawa, Japan; [Hsairi, Mohamed] Salah Azaiz Inst,

Dept Epidemiol, Tunis, Tunisia; [Jakovljevic, Mihajlo B.] Univ Kragujevac, Fac Med Sci, Kragujevac, Serbia; [Jakovljevic, Mihajlo B.] Univ Washington, Inst Hlth Metr & Evaluat, Ctr Hlth Trends & Forecasts, Seattle, WA 98195 USA; [Jonas, Jost B.] Heidelberg Univ, Med Fac Mannheim, Dept Ophthalmol, Mannheim, Germany; [Kasaeian, Amir] Univ Tehran Med Sci, Hematol Oncol & Stem Cell Transplantat Res Ctr, Tehran, Iran; [Kasaeian, Amir] Univ Tehran Med Sci, Endocrinol & Metab Populat Sci Inst, Tehran, Iran; [Khader, Yousef Saleh] Jordan Univ Sci & Technol, Dept Community Med Publ Hlth & Family Med, Irbid, Jordan; [Khan, Ejaz Ahmad] Hlth Serv Acad, Islamabad, Pakistan; [Khan, Gulfaraz] United Arab Emirates Univ, Dept Microbiol & Immunol, Coll Med & Hlth Sci, Al Ain, U Arab Emirates; [Kim, Daniel] Northeastern Univ, Dept Hlth Sci, Boston, MA 02115 USA; [Kinfu, Yohannes] Univ Canberra, Ctr Res & Action Publ Hlth, Canberra, ACT, Australia; [Larson, Heidi J.] London Sch Hyg & Trop Med, Dept Infect Dis Epidemiol, London, England; [Latif, Asma Abdul] Women Univ, Dept Zool, Lahore Coll, Lahore, Pakistan; [Linn, Shai] Univ Haifa, Haifa, Israel; [Lunevicius, Raimundas] Aintree Univ Hosp Natl Hlth Serv Fdn Trust, Liverpool, Merseyside, England; [Lunevicius, Raimundas] Univ Liverpool, Sch Med, Liverpool, Merseyside, England; [Abd El Razek, Hassan Magdy] Mansoura Fac Med, Mansoura, Egypt; [Abd El Razek, Mohammed Magdy] Aswan Univ Hosp, Aswan Fac Med, Aswan, Egypt; [Majeed, Azeem] Imperial Coll London, Dept Primary Care & Publ Hlth, London, England; [Malekzadeh, Reza; Roshandel, Gholamreza; Sepanlou, Sadaf G.] Univ Tehran Med Sci, Digest Dis Res Inst, Tehran, Iran; [Malta, Deborah Carvalho] Univ Fed Minas Gerais, Belo Horizonte, MG, Brazil; [Markos, Desalegn] Madda Walabu Univ, Robe, Ethiopia; [Memiah, Peter] Univ West Florida, Pensacola, FL USA; [Memish, Ziad A.] Saudi Minist Hlth, Riyadh, Saudi Arabia; [Memish, Ziad A.] Alfaisal Univ, Coll Med, Riyadh, Saudi Arabia; [Mendoza, Walter] United Nations Populat Fund, Lima, Peru; [Meretoja, Tuomo J.] Helsinki Univ Hosp, Breast Surg Unit, Comprehens Canc Ctr, Helsinki, Finland; [Meretoja, Tuomo J.] Univ Helsinki, Helsinki, Finland; [Miller, Ted R.] Pacific Inst Res & Evaluat, Calverton, MD USA; [Miller, Ted R.] Curtin Univ, Ctr Populat Hlth, Perth, WA, Australia; [Mohammed, Shafiu] Ahmadu Bello Univ, Hlth Syst & Policy Res Unit, Zaria, Nigeria; [Mohammed, Shafiu] Heidelberg Univ, Inst Publ Hlth, Heidelberg, Germany; [Nangia, Vinay] Suraj Eye Inst, Nagpur, Maharashtra, India; [Quyen Le Nguyen; Trang Huyen Nguyen] Duy Tan Univ, Inst Global Hlth Innovat, Da Nang, Vietnam; [Ogbo, Felix Akpojene] Western Sydney Univ, Ctr Hlth Res, Sydney, NSW, Australia; [Mahesh, P. A.] JSS Univ, JSS Med Coll, Mysore, Karnataka, India; [Park, Eun-Kee] Kosin Univ, Dept Med Human & Social Med, Coll Med, Busan, South Korea; [Patel, Tejas] Mt Sinai Hlth Syst, New York, NY USA; [Pereira, David M.] Univ Porto, Fac Farm, Dept Quim, Lab Farmacognosia,REQUIMTE LAQV, Porto, Portugal; [Pishgar, Farhad] Univ Tehran Med Sci, Noncommunicable Dis Res Ctr, Tehran, Iran; [Pishgar, Farhad] Univ Tehran Med Sci, Urooncol Res Ctr, Tehran, Iran; [Pourmalek, Farshad] Univ British Columbia, Vancouver, BC, Canada; [Qorbani, Mostafa] Alborz Univ Med Sci, Noncommunicable Dis Res Ctr, Karaj, Iran; [Radfar, Amir] AT Still Univ, Kirksville, MO USA; [Rafay, Anwar; Rana, Saleem M.] Contech Int Hlth Consultants, Lahore, Pakistan; [Rafay, Anwar; Rana, Saleem M.] Contech Sch Publ Hlth, Lahore, Pakistan; [Rahimi-Movaghar, Vafa; Safdarian, Mahdi; Salamati, Payman] Univ Tehran Med Sci, Sina Trauma & Surg Res Ctr, Tehran, Iran; [Rai, Rajesh Kumar] Soc

Hlth & Demog Surveillance, Suri, India; [Renzaho, Andre M. N.] Univ Western Sydney, Penrith, NSW, Australia; [Rezaei, Satar] Kermanshah Univ Med Sci, Sch Publ Hlth, Kermanshah, Iran; [Roba, Kedir Teji] Haramaya Univ, Harar, Ethiopia; [Roshandel, Gholamreza] Golestan Univ Med Sci, Golestan Res Ctr Gastroenterol & Hepatol, Gorgan, Iran; [Safi, Sare] Shahid Beheshti Univ Med Sci, Ophthalm Epidemiol Res Ctr, Tehran, Iran; [Safiri, Saeid] Maragheh Univ Med Sci, Sch Nursing & Midwifery, Dept Publ Hlth, Managerial Epidemiol Res Ctr, Maragheh, Iran; [Samy, Abdallah M.] Ain Shams Univ, Cairo, Egypt; [Sanabria, Juan Ramon] Marshall Univ, J Edwards Sch Med, Huntington, WV USA; [Sanabria, Juan Ramon] Case Western Reserve Univ, Cleveland, OH 44106 USA; Univ Belgrade, Inst Social Med, Fac Med, Belgrade, Serbia; [Milicevic, Milena M. Santric] Univ Belgrade, Ctr Sch Publ Hlth & Hlth Management, Fac Med, Belgrade, Serbia; [Sartorius, Benn] Univ KwaZulu Natal, Sch Nursing & Publ Hlth, Publ Hlth Med, Durban, South Africa; [Sartorius, Benn] SAMRC, UKZN Gastrointestinal Canc Res Ctr, Durban, South Africa; [Shrime, Mark G.] Harvard Med Sch, Boston, MA USA; [Stathopoulou, Vasiliki] Attikon Univ Hosp, Athens, Greece; [Sufiyan, Muawiyyah Babale] Ahmadu Bello Univ, Zaria, Nigeria; [Abdulkader, Rizwan Suliankatchi] Minist Hlth, Riyadh, Saudi Arabia; [Tabares-Seisdedos, Rafael] Univ Valencia, INCLIVA Hlth Res Inst, Dept Med, Valencia, Spain; [Tabares-Seisdedos, Rafael] CIBERSAM, Valencia, Spain; [Tehrani-Banihashemi, Arash] Iran Univ Med Sci, Prevent Med & Publ Hlth Res Ctr, Tehran, Iran; [Tekelab, Tesfalidet] Wollega Univ, Nekemte, Ethiopia; [Tekelab, Tesfalidet] Univ Newcastle, Newcastle, NSW, Australia; [Tran, Bach Xuan] Johns Hopkins Univ, Baltimore, MD USA; [Tran, Bach Xuan] Hanoi Med Univ, Hanoi, Vietnam; [Ukwaja, Kingsley Nnanna] Fed Teaching Hosp, Dept Internal Med, Abakaliki, Ebonyi State, Nigeria; [Uthman, Olalekan A.] Univ Warwick, Warwick Med Sch, Coventry, W Midlands, England; [Vlassov, Vasiliy Victorovich] Natl Res Univ, Higher Sch Econ, Moscow, Russia; [Vollset, Stein Emil] Norwegian Inst Publ Hlth, Ctr Dis Burden, Bergen, Norway; [Vollset, Stein Emil] Univ Bergen, Dept Global Publ Hlth & Primary Care, Bergen, Norway; [Weiderpass, Elisabete] Karolinska Inst, Dept Med Epidemiol & Biostat, Stockholm, Sweden; [Weiderpass, Elisabete] Canc Registry Norway, Inst Populat Based Canc Res, Dept Res, Oslo, Norway; [Weiderpass, Elisabete] Univ Tromso, Arctic Univ Norway, Dept Community Med, Fac Hlth Sci, Tromso, Norway; [Weiderpass, Elisabete] Folkhalsan Res Ctr, Genet Epidemiol Grp, Helsinki, Finland; [Werdecker, Andrea] German Natl Cohort, Fed Inst Populat Res, Competence Ctr Mortal Follow Up, Wiesbaden, Germany; [Yaghoubi, Mohsen] Univ Saskatchewan, Sch Publ Hlth, Saskatoon, SK, Canada; [Yaseri, Mehdi] Univ Tehran Med Sci, Tehran, Iran; [Yaseri, Mehdi] Shahid Beheshti Univ Med Sci, Ophthalm Res Ctr, Tehran, Iran; [Yimam, Hassen Hamid] Mizan Tepi Univ, Mizan Teferi, Ethiopia; [Yonemoto, Naohiro] Kyoto Univ, Sch Publ Hlth, Dept Biostat, Kyoto, Japan; [Zaki, Maysaa El Sayed] Mansoura Univ, Fac Med, Mansoura, Egypt; [Zein, Bassel] Georgetown Univ, Dept Neurosci, Washington, DC USA; [Hay, Simon I.] Univ Oxford, Oxford Big Data Inst, Li Ka Shing Ctr Hlth Informat & Discovery, Oxford, England Institute for Health Metrics & Evaluation; University of Washington; University of Washington Seattle; Institute for Health Metrics & Evaluation; Berlin Institute of Health; Free University of Berlin; Humboldt University of Berlin; Charite Universitatsmedizin Berlin; American University of Beirut; Iran University of Medical Sciences;

Imperial College London; Seattle Children's Hospital; Mekelle University; Lund University; Jimma University; King Saud University; Murdoch Children's Research Institute; University of Melbourne; University of Melbourne; University of Sydney; University of Oxford; Mazandaran University of Medical Sciences; Universidad de Cartagena; Tehran University of Medical Sciences; Tehran University of Medical Sciences; Egyptian Knowledge Bank (EKB); Mansoura University; University of Manitoba; Sanjay Gandhi Postgraduate Institute of Medical Sciences; University of Management & Technology (UMT); University of Belgrade; Aga Khan University; University of Toronto; Hospital for Sick Children (SickKids); Instituto Nacional de Salud (Colombia); Universidad Nacional de Colombia; Ministry of Health Iraq; Walden University; Universidade do Porto; i3S - Instituto de Investigacao e Inovacao em Saude, Universidade do Porto; Universidade do Porto; Carol Davila University of Medicine & Pharmacy; Public Health Foundation of India; University of Peradeniya; Ministry of Health & Medical Education (MOHME); Duy Tan University; International Institute for Population Sciences; Tehran University of Medical Sciences; Tehran University of Medical Sciences; Centers for Disease Control & Prevention - USA; Arba Minch University; Ministry of Health & Medical Education (MOHME); Tehran University of Medical Sciences; Harvard University; Harvard T.H. Chan School of Public Health; Harvard University; Harvard University Medical Affiliates; Massachusetts General Hospital; Karolinska Institutet; University of Bielefeld; Jimma University; Tehran University of Medical Sciences; Arabian Gulf University; Addis Ababa University; University of London; King's College London; Yokohama City University; Universite de Tunis-El-Manar; Institut Salah Azaiez; University of Kragujevac; Institute for Health Metrics & Evaluation; University of Washington; University of Washington Seattle; Ruprecht Karls University Heidelberg; Tehran University of Medical Sciences; Tehran University of Medical Sciences; Jordan University of Science & Technology; Health Services Academy; United Arab Emirates University; Northeastern University; University of Canberra; University of London; London School of Hygiene & Tropical Medicine; University of Haifa; Aintree University Hospitals NHS Foundation Trust; University of Liverpool; Egyptian Knowledge Bank (EKB); Mansoura University; Egyptian Knowledge Bank (EKB); Aswan University; Imperial College London; Tehran University of Medical Sciences; Universidade Federal de Minas Gerais; State University System of Florida; University of West Florida; Alfaisal University; United Nations Population Fund; University of Helsinki; Helsinki University Central Hospital; University of Helsinki; Pacific Institute for Research & Evaluation (PIRE); Curtin University; Ahmadu Bello University; Ruprecht Karls University Heidelberg; Suraj Eye Institute; Duy Tan University; Western Sydney University; JSS Academy of Higher Education & Research; JSS Medical College, Mysuru; Icahn School of Medicine at Mount Sinai; Universidade do Porto; Tehran University of Medical Sciences; Tehran University of Medical Sciences; University of British Columbia; Alborz University of Medical Sciences; A.T. Still University of Health Sciences; Tehran University of Medical Sciences; Western Sydney University; Kermanshah University of Medical Sciences; Haramaya University; Golestan University of Medical Sciences; Shahid Beheshti University Medical Sciences; Egyptian Knowledge Bank (EKB); Ain Shams University; Marshall University; University

System of Ohio; Case Western Reserve University; University of Belgrade; University of Belgrade; University of Kwazulu Natal; Harvard University; Harvard Medical School; University Hospital Attikon; Ahmadu Bello University; Ministry of Health - Saudi Arabia; University of Valencia; CIBER - Centro de Investigacion Biomedica en Red; CIBERSAM; Iran University of Medical Sciences; University of Newcastle; Johns Hopkins University; Hanoi Medical University; University of Warwick; HSE University (National Research University Higher School of Economics); Norwegian Institute of Public Health (NIPH); University of Bergen; Karolinska Institutet; University of Oslo; UiT The Arctic University of Tromso; Folkhalsan Research Center; University of Saskatchewan; Tehran University of Medical Sciences; Shahid Beheshti University Medical Sciences; Kyoto University; Egyptian Knowledge Bank (EKB); Mansoura University; Georgetown University; University of Oxford Fitzmaurice, C (corresponding author), Univ Washington, Inst Hlth Metr & Evaluat, Seattle, WA 98195 USA.

cf11@uw.edu Abegaz, Kedir/AAE-7080-2021; Al-Aly, Ziyad/S-4439-2016; Yaghoubi, Mohsen/T-6630-2017; Vlassov, Valentin/F-4720-2013; Moradi-Lakeh, Maziar/ABC-9793-2021; Mendes, J/AAU-6390-2021; Amini, Erfan/X-8366-2019; Renzaho, Andre/AAH-7679-2021; Samy, Abdallah/B-4375-2010; Guadie, Habtamu/IWD-8924-2023; Atey, Tesfay/T-3382-2019; Malekzadeh, Reza/U-1382-2017; Anber, Nahla/AAM-9830-2021; Safi, Sare/AAW-4651-2020; Temsah, Mohamad-Hani/AAJ-9703-2020; Demissie, Dr.Dereje/AAN-3556-2021; Sisay, Eskinder/AAB-8251-2022; Kinfu, Yohannes/AAC-8054-2020; Sultana, Saima/GZB-0493-2022; Alvis-Guzman, Nelson/D-4913-2013; Mahesh, P/R-1184-2019; Kumar, Rajesh/ABA-6489-2020; Rezaei, Satar/ADA-3531-2022; Bacha, Umar/M-2055-2015; Phuc, Huyen/I-5061-2019; Barac, Aleksandra/JBS-0092-2023; Bedi, Neeraj/H-2838-2019; Khalil, Ibrahim/M-1520-2016; Rahimi-Movaghar, Vafa/L-6339-2019; Ebrahimi, Hedyeh/AAR-2899-2021; Jonas, Jost/AEP-3841-2022; Alsharif, Ubai/AAK-2088-2021; Endries, Aman/AAN-6871-2021; Nguyen, Hoang/HJI-3940-2023; Radfar, Amir/I-8057-2019; Safiri, Saeid/A-1678-2017; Elsayed, Omar/O-7012-2018; Khan, Gulfaraz/AAC-2859-2022; Nguyễn, Nhung/KHV-4764-2024; Qorbani, Mostafa/M-8171-2017; Roba, Kedir/LKK-6206-2024; Al-Eyadhy, Ayman/AAP-8396-2021; Motta, Jorge/ABI-6006-2020; Roshandel, Gholamreza/AAJ-9562-2021; Beyene, Tesfalidet/H-4090-2019; Fereshtehnejad, Seyed-Mohammad/ABF-6020-2020; Al-Raddadi, Rajaa/F-8337-2010; Tabares-Seisdedos, Rafael/H-6432-2013; Basaleem, Huda/AAX-9878-2020; Naohiro, Yonemoto/Y-3761-2019; Butt, Zahid/W-4292-2017; Vos, Theo/HLH-2955-2023; Uthman, Olalekan/N-5584-2019; Sufiyan, Muawiyyah Babale/GWN-2609-2022; Hamadeh, Randah/AAE-9720-2020; Dimbuene, Zacharie/H-3029-2019; Alasfoor, Deena/HJO-9139-2023; Memish, Ziad/AEJ-9424-2022; Awasthi, Ashish/Y-5915-2019; Kim, Daniel/A-8016-2019; Murray, Christopher/JZD-3782-2024; Violante, Francesco/A-6934-2009; Areri, Habtamu Abera/AAE-9882-2020; Memiah, Peter/P-3115-2017; Bhutta, Zulfiqar/ADZ-0156-2022; f, m/AAM-2063-2021; Tehrani-Banihashemi, Arash/L-5964-2018; Linn, Shai/N-3079-2019; Kasaeian, Amir/C-8290-2017; Davitoiu, Dragos/P-9703-2017; Fischer, Florian/F-9003-2016; /B-3002-2014; das Neves, Jose/J-8369-2013; Samy, Abdallah/I-1415-2014; Majeed, Azeem/KHC-7311-2024; Pereira, David/M-9286-2013; Bedi, Neeraj/AGR-0606-2022; Rana, Saleem/Y-9791-2018; Pourmalek, Farshad/A-8188-2010; khader, yousef/AAE-9620-2019; Bhutta, Zulfiqar/L-7822-2015; Rahimi-Movaghar, Vafa/T-7816-2017; Larson, Heidi J./N-1018-2017; Mokdad, Ali/AAD-1232-2022; Yaseri, Mehdi/I-1645-2018; Hassen, Hamid/A-1105-2019; Sepanlou, Sadaf/H-9343-2016;

Awasthi, Ashish/P-3966-2018; Castaneda-Orjuela, Carlos/N-2601-2017; Roshandel, Gholamreza/N-2260-2016; Alizadeh-Navaei, Reza/B-2207-2017; Santric Milicevic, Milena/H-6423-2018; Mohammed, Shafiu/P-2016-2014; El Sayed Zaki, Maysaa/C-1522-2013; Carvalho Malta, Deborah/H-7880-2012; Alsharif, Ubai/C-6527-2017; Weiderpass, Elisabete/M-4029-2016; Altirkawi, Khalid/D-7302-2017; S A, Rizwan/F-5289-2014; Ukwaja, Kingsley N./A-7794-2013; Lunevicius, Raimundas/B-2528-2018; Hay, Simon/F-8967-2015; Amini, Erfan/A-2063-2009; Ahmed, Muktar/G-6184-2019; Uthman, Olalekan/P-7916-2014; Khan, Ejaz/B-9340-2016; Vlassov, Vasiliy/B-4036-2014

Sartorius, Benn/0000-0001-6761-2325; Linn, Shai/0000-0002-0867-2958; Kasaeian, Amir/0000-0003-2018-9368; Davitoiu, Dragos/0000-0002-4493-6754; Sufiyan, Muawiyyah Babale/0000-0003-3630-6995; Fischer, Florian/0000-0002-4388-1245; /0000-0002-9160-6846; Pishgar, Farhad/0000-0003-0703-8442; das Neves, Jose/0000-0002-2317-2759; Tabares-Seisdedos, Rafael/0000-0002-1089-2204; Khan, Gulfaraz/0000-0001-6836-1783; Miller, Ted/0000-0002-0958-2639; Samy, Abdallah/0000-0003-3978-1134; Alam, Khurshid/0000-0002-7402-7519; Majeed, Azeem/0000-0002-2357-9858; Pereira, David/0000-0003-0384-7592; Bedi, Neeraj/0000-0003-4709-8423; Rana, Saleem/0000-0001-6479-9235; Pourmalek, Farshad/0000-0002-2134-0771; Al-Eyadhy, Ayman/0000-0002-6051-9125; Kiadaliri, Ali/0000-0002-4254-9099; khader, yousef/0000-0002-7830-6857; Rezaei, Satar/0000-0002-6194-6057; Bhutta, Zulfiqar/0000-0003-0637-599X; Farvid, Maryam/0000-0003-1783-4186; Al-Raddadi, Rajaa/0000-0002-8921-9628; Rahimi-Movaghar, Vafa/0000-0001-7347-8767; Larson, Heidi J./0000-0002-8477-7583; Ebrahimi, Hedyeh/0000-0003-3647-7356; Mokdad, Ali/0000-0002-4994-3339; Yaseri, Mehdi/0000-0002-4066-873X; Hassen, Hamid/0000-0001-6485-4193; Sepanlou, Sadaf/0000-0002-3669-5129; Moradi-Lakeh, Maziar/0000-0001-7381-5305; Awasthi, Ashish/0000-0002-9308-9782; Castaneda-Orjuela, Carlos/0000-0002-8735-6223; Dubey, Manisha/0000-0003-2879-903X; Tehrani Banihashemi, Seyed Arash/0000-0001-6911-9817; Endries, Aman Yesuf/0000-0002-4565-5364; Roshandel, Gholamreza/0000-0002-5494-0722; Tran, Bach/0000-0002-2191-3947; Hamidi, Samer/0000-0002-6766-3728; Alizadeh-Navaei, Reza/0000-0003-0580-000X; Beyene, Dr Tesfalidet/0000-0003-0774-7708; Temsah, Mohamad-Hani/0000-0002-4389-9322; Santric Milicevic, Milena/0000-0002-0684-359X; Mohammed, Shafiu/0000-0001-5715-966X; El Sayed Zaki, Maysaa/0000-0001-5431-0248; Carvalho Malta, Deborah/0000-0002-8214-5734; Esteghamati, Alireza/0000-0001-5114-3982; RENZAHO, ANDRE/0000-0002-6844-0833; Magdy Abd El Razek, Hassan/0000-0002-8857-0408; Alsharif, Ubai/0000-0002-4024-3950; Kim, Daniel/0000-0001-8907-6420; Shifti, Desalegn Markos/0000-0001-6638-6194; Alvis-Guzman, Nelson/0000-0001-9458-864X; Weiderpass, Elisabete/0000-0003-2237-0128; Rai, Rajesh Kumar/0000-0002-5249-9937; Altirkawi, Khalid/0000-0002-7331-4196; Kassaw, Nigussie Assefa/0000-0001-5420-0671; S A, Rizwan/0000-0002-3140-2614; Ukwaja, Kingsley N./0000-0002-1974-8735; Yaghoubi, Mohsen/0000-0002-6912-7267; Lunevicius, Raimundas/0000-0003-3295-0142; Hay, Simon/0000-0002-0611-7272; Amini, Erfan/0000-0001-9647-0047; Ahmed, Muktar/0000-0002-9524-7027; Uthman, Olalekan/0000-0002-8567-3081; Khan, Ejaz/0000-0002-7072-8035; Anber, Nahla/0000-0001-5684-9298; Rawaf, Salman/0000-0001-7191-2355; Mahesh, Padukudru Anand/0000-0003-1632-5945; Al Asfoor, Deena/0000-0002-7973-1935; Wasif, Muhammad/0009-0004-1374-302X; Qorbani, Mostafa/0000-0001-9465-7588; Barac, Aleksandra/0000-0002-0132-2277; Gopalani, Sameer/0000-0003-0611-305X; Kinfu, Yohannes/0000-0001-9607-6891;

Vlassov, Vasiliy/0000-0001-5203-549X; Chitheer, Abdulaal/0000-0003-4899-6196 Bill AMP; Melinda Gates Foundation Bill AMP; Melinda Gates Foundation(CGIAR) The funding source played no role in the design of the study, the analysis and interpretation of data, and the writing of the paper. GBD 2015 is funded by Bill & Melinda Gates Foundation. 34 45 46 1 22
FRONTIERS MEDIA SA LAUSANNE AVENUE DU TRIBUNAL FEDERAL 34, LAUSANNE, CH-1015, SWITZERLAND 1661-8556 1661-8564
INT J PUBLIC HEALTH Int. J. Public Health MAY 2018 63
1 151 164 10.1007/s00038-017-0999-9 http://dx.doi.org/10.1007/s00038-017-0999-9
14 Public, Environmental & Occupational Health Science Citation Index Expanded (SCI-EXPANDED); Social Science Citation Index (SSCI) Public, Environmental & Occupational Health
GH6AA 28776254 hybrid, Green Accepted, Green Submitted, Green Published 2025-06-24 WOS:000433519400015
J Benassi, F; Crisci, M; Matthews, SA; Rimoldi, SML
Benassi, Federico; Crisci, Massimiliano; Matthews, Stephen A.; Rimoldi, Stefania M. L. Migrants' Population, Residential Segregation, and Metropolitan Spaces - Insights from the Italian Experience over the Last 20 Years
MIGRATION LETTERS English Article
Foreign population; immigration; residential segregation; metropolitan space; Italy NON-EUROPEAN MIGRANTS; NEIGHBORHOOD CONCENTRATION; FAMILY; REPRESENTATION; MIGRATION; PATTERNS Southern European studies of migrants' spatial distribution within metropolitan cities (MCs) are increasingly relevant to understanding residential segregation and marginalisation, particularly of foreign nationals. This paper leverages original and partially unpublished data to examine overall and foreign national specific segregation over two decades in Rome MC and Milan MC, the two largest Italian MCs. We introduce a 5-class concentric ring typology to describe and uncover geographical patterns within the Mu and focus on the spatial and temporal distribution of four selected foreign nationalities: Romanian, Bangladeshi, Chinese, and Filipino. Results reveal heterogeneity in overall and foreign national specific distributions over time both within and between Rome MC and Milan MC. Comparing across groups and MCs we identify similarities but also unique patterns. These results shed light on the peculiarity of the urban demographic Italian landscape and raise questions regarding recent theories about residential segregation in the urban contexts of Southern Europe. [Benassi, Federico] Italian Natl Inst Stat ISTAT Pzza Guglielmo Marco, Rome, Italy; [Crisci, Massimiliano] Inst Res Populat & Social Policies CNR IRPPS, Italian Natl Res Council, Rome, Italy; [Matthews, Stephen A.] Penn State Univ, Dept Sociol & Criminol, University Pk, PA 16802 USA; [Matthews, Stephen A.] Penn State Univ, Dept Anthropol, University Pk, PA 16802 USA; [Rimoldi, Stefania M. L.] Univ Milano Bicocca, Dept Stat & Quantitat Methods, Via Bicocca Arcimboldi, Milan, Italy Consiglio Nazionale delle Ricerche (CNR); Istituto di Ricerche sulla Popolazione e le Politiche Sociali (IRPPS-CNR); Pennsylvania Commonwealth System of Higher Education (PCSHE); Pennsylvania State University; Pennsylvania State University - University Park; Penn State Behrend; Pennsylvania Commonwealth System of Higher Education (PCSHE); Pennsylvania State University; Penn State Behrend; Pennsylvania State University - University Park; University of Milano-Bicocca

Rimoldi, SML (corresponding author), Univ Milano Bicocca, Dept Stat & Quantitat Methods, Via Bicocca Arcimboldi, Milan, Italy. benassi@istat.it; m.crisci@irpps.cnr.it; sxm27@psu.edu; stefania.rimoldi@unimib.it Matthews, Stephen/GRR-7264-2022; Benassi, Federico/LTD-8323-2024; Crisci, Massimiliano/AAW-9406-2020; Rimoldi, Stefania/AAU-2692-2020; Matthews, Stephen/P-6906-2017 Matthews, Stephen/0000-0002-1645-4854; Benassi, Federico/0000-0002-8861-9996; RIMOLDI, STEFANIA MARIA LORENZA/0000-0001-9693-9642 Italian Ministry for Universities and Research; European Union' Horizon 2020 Programme; Population Research Institute (PRI); Eunice Kennedy Shriver National Institute of Child Health and Human Development [P2CHD041025]; Pennsylvania State University and its Social Science Research Institute Italian Ministry for Universities and Research(Ministry of Education, Universities and Research (MIUR)); European Union' Horizon 2020 Programme; Population Research Institute (PRI); Eunice Kennedy Shriver National Institute of Child Health and Human Development(United States Department of Health & Human ServicesNational Institutes of Health (NIH) - USANIH Eunice Kennedy Shriver National Institute of Child Health & Human Development (NICHD)); Pennsylvania State University and its Social Science Research Institute The analyses here presented are part of the following projects: "Immigration, integration, settlement. Italian-Style", PRIN 2017 (Italian Research Projects of National Relevance), financed by the Italian Ministry for Universities and Research; "Future Migration Scenarios for Europe" (FUME), European Union' Horizon 2020 Programme. Matthews would like to acknowledge the support of the Population Research Institute (PRI). PRI is supported by a grant from the Eunice Kennedy Shriver National Institute of Child Health and Human Development (P2CHD041025) and by the Pennsylvania State University and its Social Science Research Institute. 61 8 8 0 5 TRANSNATIONAL PRESS LONDON LONDON 12 RIDGEWAY GARDENS, LONDON, N6 5XR, ENGLAND 1741-8984 1741-8992 MIGR LETT Migr. Lett. MAY 2022 19 3 287 301 10.33182/ml.v19i3.1795 http://dx.doi.org/10.33182/ml.v19i3.1795 15 Demography Emerging Sources Citation Index (ESCI) Demography 1H5IZ 2025-06-24 WOS:000796578900004

J Dey, S; Hablas, A; Seifeldin, IA; Ismail, K; Ramadan, M; El-Hamzawy, H; Wilson, ML; Banerjee, M; Boffetta, P; Harford, J; Merajver, SD; Soliman, AS Dey, S.; Hablas, A.; Seifeldin, I. A.; Ismail, K.; Ramadan, M.; El-Hamzawy, H.; Wilson, M. L.; Banerjee, M.; Boffetta, P.; Harford, J.; Merajver, S. D.; Soliman, A. S. Urban-rural differences of gynaecological malignancies in Egypt (1999-2002) BJOG-AN INTERNATIONAL JOURNAL OF OBSTETRICS AND GYNAECOLOGY English Article Egypt; gynaecologic cancers; urban-rural; xenoestrogens PERSISTENT ORGANIC POLLUTANTS; ENDOMETRIAL CANCER RISK; BISPHENOL-A; OVARIAN-CANCER; BREAST-CANCER; EPIDEMIOLOGY; PESTICIDES; RECEPTOR; HISTORY; UTERINE Objective In previous studies, we have shown a three to four times higher urban incidence of breast cancer and estrogen receptor-positive breast cancers in the Gharbiah Province of Egypt. We investigated the urban-rural incidence differences of gynaecologic malignancies (uterine, ovarian and cervical cancers) to explore if they show the same trend that we found for breast

cancer. Design Cancer registry-based incidence comparison. Setting Gharbiah population-based cancer registry (GPCR), Tanta, Egypt. Sample All patients with uterine, ovarian and cervical cancer in GPCR from 1999 to 2002. Methods We calculated uterine, ovarian and cervical cancer incidence from 1999 to 2002. For each of the three cancers, we calculated the overall and age-specific rates for the province as a whole, and by urban-rural status, as well as for the eight districts of the province. Results Incidence of all three cancer sites was higher in urban than in rural areas. Uterine cancer showed the highest urban-rural incidence rate ratio (IRR = 6.07, 95% CI = 4.17, 8.85). Uterine cancer also showed the highest urban incidence in the oldest age group (70+ age category, IRR = 14.39, 95% CI = 4.24, 48.87) and in developed districts (Tanta, IRR = 4.14, 95% CI = 0.41, 42.04). Incidence rates by groups of cancer sites showed an increasing gradient of urban incidence for cancers related to hormonal aetiology, mainly of the breast and uterus (IRR = 4.96, 95% CI = 2.86, 8.61). Conclusions The higher urban incidence of uterine cancer, coupled with our previous findings of higher incidence of breast cancer and estrogen receptor positive breast cancer in urban areas in this region, may be suggestive of possible higher exposure to environmental estrogenic compounds, such as xenoestrogens, in urban areas.
[Dey, S.; Wilson, M. L.; Soliman, A. S.] Univ Michigan, Sch Publ Hlth, Dept Epidemiol, Ann Arbor, MI 48109 USA; [Hablas, A.; Ismail, K.] Gharbiah Canc Soc, Tanta, Gharbiah, Egypt; [Seifeldin, I. A.; Ramadan, M.; El-Hamzawy, H.] Tanta Canc Ctr, Tanta, Gharbiah, Egypt; [Banerjee, M.] Univ Michigan, Sch Publ Hlth, Dept Biostat, Ann Arbor, MI 48109 USA; [Boffetta, P.] Int Agcy Res Canc, F-69372 Lyon, France; [Harford, J.] NCI, Off Int Affairs, Bethesda, MD 20892 USA; [Merajver, S. D.] Univ Michigan, Ctr Comprehens Canc, Ann Arbor, MI 48109 USA University of Michigan System; University of Michigan; Egyptian Knowledge Bank (EKB); Tanta University; University of Michigan System; University of Michigan; World Health Organization; International Agency for Research on Cancer (IARC); National Institutes of Health (NIH) - USA; NIH National Cancer Institute (NCI); University of Michigan System; University of Michigan Soliman, AS (corresponding author), Univ Michigan, Sch Publ Hlth, Dept Epidemiol, 109 Observ St, Ann Arbor, MI 48109 USA.asoliman@umich.edu Boffetta, Paolo/AAI-7767-2021; Hablas, Ahmed/AFK-5458-2022 Harford, Joe Bryan/0000-0002-6681-6315 Middle East Cancer Consortium, National Cancer Institute, Bethesda [R25 CA112383, R03 CA117350, 5 P30 CA46592]; Burroughs Wellcome Fund; Breast Cancer Research Foundation; Block Grant of the Department of Epidemiology, University of Michigan School of Public Health; Travel Grant of the Rackham Graduate School of the University of Michigan; National Cancer Institute [P30CA046592] Funding Source: NIH RePORTER Middle East Cancer Consortium, National Cancer Institute, Bethesda(United States Department of Health & Human ServicesNational Institutes of Health (NIH) - USANIH National Cancer Institute (NCI)); Burroughs Wellcome Fund(Burroughs Wellcome Fund); Breast Cancer Research Foundation; Block Grant of the Department of Epidemiology, University of Michigan School of Public Health(University of Michigan System); Travel Grant of the Rackham Graduate School of the University of Michigan(University of Michigan System); National Cancer Institute(United States Department of Health & Human ServicesNational Institutes of Health (NIH) - USANIH National Cancer Institute (NCI)) This work was

supported by the Middle East Cancer Consortium, National Cancer Institute, Bethesda [R25 CA112383, R03 CA117350, 5 P30 CA46592], the Burroughs Wellcome Fund (SDM) and the Breast Cancer Research Foundation (SDM), a Block Grant of the Department of Epidemiology, University of Michigan School of Public Health and the Travel Grant of the Rackham Graduate School of the University of Michigan (S Dey). 29 30 30 0 4 WILEY HOBOKEN 111 RIVER ST, HOBOKEN 07030-5774, NJ USA 1470-0328 1471-0528 BJOG-INT J OBSTET GY BJOG FEB 2010 117 3 348 355 10.1111/j.1471-0528.2009.02447.x http://dx.doi.org/10.1111/j.1471-0528.2009.02447.x 8 Obstetrics & Gynecology Science Citation Index Expanded (SCI-EXPANDED) Obstetrics & Gynecology 543CJ 20015310 Green Accepted, Green Submitted 2025-06-24 WOS:000273547200014

J Neirich, L; Yahiaoui-Doktor, M; Lammert, J; Basrai, M; Seethaler, B; Berling-Ernst, A; Ramser, J; Quante, AS; Schmidt, T; Niederberger, U; Rhiem, K; Schmutzler, R; Engel, C; Bischoff, SC; Halle, M; Kiechle, M; Grill, S Neirich, Leonie; Yahiaoui-Doktor, Maryam; Lammert, Jacqueline; Basrai, Maryam; Seethaler, Benjamin; Berling-Ernst, Anika; Ramser, Juliane; Quante, Anne S.; Schmidt, Thorsten; Niederberger, Uwe; Rhiem, Kerstin; Schmutzler, Rita; Engel, Christoph; Bischoff, Stephan C.; Halle, Martin; Kiechle, Marion; Grill, Sabine Physical activity and Mediterranean diet as potential modulators of osteoprotegerin and soluble RANKL in gBRCA1/2 mutation carriers: results of the lifestyle intervention pilot study LIBRE-1 BREAST CANCER RESEARCH AND TREATMENT English Article BRCA1; 2 mutation carriers; Breast cancer; OPG; RANKL; Lifestyle intervention; Physical activity; Mediterranean diet; Fatty acids N-3 FATTY-ACIDS; KAPPA-B LIGAND; BREAST-CANCER RISK; RECEPTOR ACTIVATOR; SERUM OSTEOPROTEGERIN; DOCOSAHEXAENOIC ACID; RESISTANCE EXERCISE; AEROBIC EXERCISE; BRCA1 MUTATION; STEM-CELLS Purpose Emerging evidence suggests that the progesterone-mediated receptor activator of nuclear factor kappa B (RANK)/soluble RANK ligand (sRANKL)/osteoprotegerin (OPG) pathway plays an important role in mammary carcinogenesis and is hyperactivated in germline (g)BRCA1/2 mutation carriers. We analyzed the effects of a 3-month intensive lifestyle intervention within the LIBRE-1 study on the serum levels of OPG and sRANKL and hypothesized that the intervention program provides a beneficial impact on the biomarkers by increasing OPG and reducing sRANKL serum concentrations. Methods Serum levels of OPG and sRANKL of 49 gBRCA1/2 mutation carriers were quantified using enzyme-linked immunosorbent assays. We used previously collected blood samples from participants of the prospective LIBRE-1 study, who were randomized into an intervention group (IG), increasing physical activity and adherence to the Mediterranean diet (MedD) through supervised sessions from study entry to the first study visit after 3 months and a usual-care control group (CG). Differences in biomarker levels before and after the 3-month intervention were tested within and between study groups. Results The lifestyle intervention resulted in a significant increase in OPG for participants in both the IG ($q = 0.022$) and CG ($q = 0.002$). sRANKL decreased significantly in the IG ($q = 0.0464$) and seemed to decrease in the CG ($q = 0.5584$). An increase in the intake of Omega-3 polyunsaturated fatty acids was significantly associated

with an increase in OPG (r = 0.579, q = 0.045). Baseline serum levels of sRANKL were a strong predictor for the change of sRANKL in the course of the intervention (ss-estimate = - 0.70; q = 0.0018). Baseline physical fitness (assessed as VO(2)peak) might predict the change of OPG in the course of the intervention program (ss-estimate = 0.133 pg/ml/ml/min/kg; p = 0.0319; q = 0.2871). Conclusion Findings from this pilot study seem to confirm our hypothesis by showing an increase in OPG and decrease in sRANKL over a 3-month lifestyle intervention and suggest that increased physical activity and adherence to the MedD are potent modulators of the biomarkers OPG and potentially sRANKL.[Neirich, Leonie; Lammert, Jacqueline; Ramser, Juliane; Quante, Anne S.; Kiechle, Marion; Grill, Sabine] Tech Univ Munich TUM, Dept Gynecol, Univ Hosp Rechts Isar, Munich, Germany; [Neirich, Leonie; Lammert, Jacqueline; Ramser, Juliane; Quante, Anne S.; Kiechle, Marion; Grill, Sabine] Tech Univ Munich TUM, Univ Hosp Rechts Isar, Ctr Hereditary Breast & Ovarian Canc, Munich, Germany; [Yahiaoui-Doktor, Maryam; Engel, Christoph] Univ Leipzig, Inst Med Informat Stat & Epidemiol IMISE, Leipzig, Germany; [Basrai, Maryam; Seethaler, Benjamin] Univ Hohenheim, Inst Nutr Med, Stuttgart, Germany; [Berling-Ernst, Anika] Tech Univ Munich TUM, Univ Hosp Rechts Isar, Fac Med, Dept Prevent Rehabil & Sports Med, Munich, Germany; [Schmidt, Thorsten] Univ Hosp Schleswig Holstein, Ctr Comprehens Canc, Kiel, Germany; [Niederberger, Uwe] Univ Hosp Schleswig Holstein, Inst Med Psychol & Med Sociol, Kiel, Germany; [Rhiem, Kerstin; Schmutzler, Rita] Univ Hosp Cologne, Ctr Hereditary Breast & Ovarian Canc, Cologne, Germany; [Halle, Martin] DZHK German Ctr Cardiovasc Res, Partner Site Munich Heart Alliance, Munich, Germany; [Quante, Anne S.] Univ Freiburg, Inst Human Genet, Med Ctr Univ Freiburg, Fac Med, Freiburg, Germany
Technical University of Munich; Technical University of Munich; Leipzig University; University Hohenheim; Technical University of Munich; University of Kiel; Schleswig Holstein University Hospital; University of Kiel; Schleswig Holstein University Hospital; University of Cologne; German Centre for Cardiovascular Research; Munich Heart Alliance; University of Freiburg   Neirich, L (corresponding author), Tech Univ Munich TUM, Dept Gynecol, Univ Hosp Rechts Isar, Munich, Germany.; Neirich, L (corresponding author), Tech Univ Munich TUM, Univ Hosp Rechts Isar, Ctr Hereditary Breast & Ovarian Canc, Munich, Germany.   leonie.neirich@mri.tum.de   Yahiaoui-Doktor, Maryam/I-5859-2019; Niederberger, Uwe/A-8256-2010; Grill, Sabine/AAM-3978-2020 Yahiaoui-Doktor, Maryam/0000-0002-3321-1598; Niederberger, Uwe/0000-0003-3099-0183; Lammert, Jacqueline/0000-0002-0380-574X; Neirich, Leonie/0000-0003-3703-8726; Engel, Christoph/0000-0002-7247-282X German Cancer Aid (Deutsche Krebshilfe) within the Priority Program "Primary Prevention of Cancer" [110013]; Projekt DEAL German Cancer Aid (Deutsche Krebshilfe) within the Priority Program "Primary Prevention of Cancer"; Projekt DEAL   Open Access funding enabled and organized by Projekt DEAL. The LIBRE-1 study is funded by the German Cancer Aid (Deutsche Krebshilfe, http://www.krebs hilfe.de) within the Priority Program "Primary Prevention of Cancer" (Grant No. 110013). The funder has no authority and is not involved in the following activities: study design; collection, management, analysis, and interpretation of data; writing of the report; and the decision to submit the report for publication.   78   1   2   0   6   SPRINGER   NEW YORK ONE NEW YORK PLAZA, SUITE 4600, NEW YORK, NY, UNITED STATES

0167-6806 1573-7217 BREAST CANCER RES TR Breast Cancer Res. Treat. DEC 2021 190 3
463 475 10.1007/s10549-021-06400-7
http://dx.doi.org/10.1007/s10549-021-06400-7 SEP 2021
13 Oncology Science Citation Index Expanded (SCI-EXPANDED) Oncology WP4VR 34570303 Green Published, hybrid
2025-06-24 WOS:000701008000002
J Park, SY; Boushey, CJ; Wilkens, LR; Haiman, CA; Le Marchand, L Park, Song-Yi; Boushey, Carol J.; Wilkens, Lynne R.; Haiman, Christopher A.; Le Marchand, Loic
High-Quality Diets Associate With Reduced Risk of Colorectal Cancer: Analyses of Diet Quality Indexes in the Multiethnic Cohort
GASTROENTEROLOGY English Article
DASH; Food; Nutrition; Colon Cancer
MAJOR CHRONIC DISEASE; HEALTHY EATING INDEX; GUIDELINES-FOR-AMERICANS; PATTERNS; MORTALITY; ADHERENCE; WOMEN; SURVIVAL; HAWAII; FIBER
BACKGROUND & AIMS: Healthy eating patterns assessed by diet quality indexes (DQIs) have been related to lower risk of colorectal cancer-mostly among whites. We investigated the associations between 4 DQI scores (the Healthy Eating Index 2010 [HEI-2010], the Alternative Healthy Eating Index 2010 [AHEI-2010], the alternate Mediterranean diet score [aMED], and the Dietary Approaches to Stop Hypertension score) and colorectal cancer risk in the Multiethnic Cohort. METHODS: We analyzed data from 190,949 African American, Native Hawaiian, Japanese American, Latino, and white individuals, 45 to 75 years old, who entered the Multiethnic Cohort study from 1993 through 1996. During an average 16 years of follow-up, 4770 invasive colorectal cancer cases were identified. RESULTS: Scores from all 4 DQIs associated inversely with colorectal cancer risk; higher scores associated with decreasing colorectal cancer risk (all P's for trend <= .003). Associations were not significant for AHEI-2010 and aMED scores in women after adjustment for covariates: for the highest vs lowest quintiles, the hazard ratio for the HEI-2010 score in men was 0.69 (95% confidence interval [CI], 0.59-0.80) and in women was 0.82 (95% CI, 0.70-0.96); for the AHEI-2010 score the hazard ratio in men was 0.75 (95% CI, 0.65-0.85) and in women was 0.90 (95% CI, 0.78-1.04); for the aMED score the hazard ratio in men was 0.84 (95% CI, 0.73-0.97) and in women was 0.96 (95% CI, 0.82-1.13); for the Dietary Approaches to Stop Hypertension score the hazard ratio in men was 0.75 (95% CI, 0.66-0.86) and in women was 0.86 (95% CI, 0.75-1.00). Associations were limited to the left colon and rectum for all indexes. The inverse associations were less strong in African American individuals than in the other 4 racial/ethnic groups. CONCLUSIONS: Based on an analysis of data from the Multiethnic Cohort Study, high-quality diets are associated with a lower risk of colorectal cancer in most racial/ethnic subgroups.
[Park, Song-Yi; Boushey, Carol J.; Wilkens, Lynne R.; Le Marchand, Loic] Univ Hawaii, Ctr Canc, Canc Epidemiol Program, 701 Ilalo St, Honolulu, HI 96813 USA; [Haiman, Christopher A.] Univ Southern Calif, Keck Sch Med, Dept Prevent Med, Los Angeles, CA USA University of Hawaii System; Cancer Research Center of Hawaii; University of Southern California Park, SY (corresponding author), Univ Hawaii, Ctr Canc, Canc Epidemiol Program, 701 Ilalo St, Honolulu, HI 96813 USA.
spark@cc.hawaii.edu Haiman, Christopher/HGC-5586-2022
National Cancer Institute at the National Institutes of Health [U01 CA164973, NCI HHSN261201200423P, P30 CA071789]

National Cancer Institute at the National Institutes of Health(United States Department of Health & Human ServicesNational Institutes of Health (NIH) - USANIH National Cancer Institute (NCI)) Supported by the National Cancer Institute at the National Institutes of Health (U01 CA164973, NCI HHSN261201200423P, P30 CA071789). The sponsor had no other roles in the study. 42 97 106 0 20 W B SAUNDERS CO-ELSEVIER INC PHILADELPHIA 1600 JOHN F KENNEDY BOULEVARD, STE 1800, PHILADELPHIA, PA 19103-2899 USA 0016-5085 1528-0012 GASTROENTEROLOGY Gastroenterology AUG 2017 153 2 386 +
10.1053/j.gastro.2017.04.004
http://dx.doi.org/10.1053/j.gastro.2017.04.004
11 Gastroenterology & Hepatology Science Citation Index Expanded (SCI-EXPANDED) Gastroenterology & Hepatology
FB3KE 28428143 Green Accepted 2025-06-24
WOS:000406040600018
J Rolic, T; Mandic, S; Lukic, I; Banjari, I
Rolic, Tara; Mandic, Sanja; Lukic, Iva; Banjari, Ines
Can Dietary Iron Bioavailability Influence Colorectal Cancer Risk and Prognosis? MIDDLE EAST JOURNAL OF CANCER
English Article
Colorectal neoplasms; Dietary iron; Biological availability; Hepcidins; Projections and predictions DOSE-RESPONSE METAANALYSIS; ONE-CARBON METABOLISM; PHYTIC ACID; VITAMIN-B12; ABSORPTION; ANEMIA; ASSOCIATION; PREVENTION; PREDICTION; HEPCIDIN
Colorectal cancer (CRC) stands apart from other malignancies due to its pronounced association with dietary patterns. Approximately 70% of all CRC cases arise sporadically, and suboptimal dietary and lifestyle choices can override certain predisposing factors, including a family history of the disease. Hitherto, the most compelling evidence linking CRC risk has been attributed to heme iron, predominantly found in red and processed meats, although this form of iron constitutes a mere 20% of total dietary iron. The human organism maintains a remarkably intricate and tightly regulated iron homeostasis system owing to the deleterious consequences of both excessive and deficient serum iron levels. Dietary sources remain the sole means to replenish iron losses. Despite the abundant presence of iron in various food sources, its absorption, commonly referred to as bioavailability, is notably restricted due to an array of dietary inhibitors and homeostatic mechanisms. Consequently, a substantial 80% of ingested dietary iron is excreted in fecal matter, resulting in fecal iron concentrations that surpass those found in most body tissues by a tenfold margin. Prolonged exposure of the colorectum to excessive fecal iron, combined with concurrent physiological alterations, can instigate oncogenic processes leading to CRC. Notably, despite their recognized significance in CRC pathology, dietary habits, and lifestyle factors have been sporadically integrated into predictive models, primarily concerning CRC recurrence. Nonetheless, these models exhibit disparities in the dietary components, rendering them non -universally applicable. In light of these disparities, postulating that incorporating bioavailable iron, in conjunction with hepcidin levels, may offer superior predictive value for CRC risk assessment, and herein, elucidates the scientific foundation supporting this hypothesis.
[Rolic, Tara; Mandic, Sanja; Lukic, Iva] Osijek Univ Hosp Ctr, Inst Clin Lab Diagnost, Osijek, Croatia; [Mandic, Sanja;

Lukic, Iva] Univ Osijek, Fac Med, Dept Chem Biochem & Clin Chem, Osijek, Croatia; [Banjari, Ines] Univ Osijek, Fac Food Technol Osijek, Dept Food & Nutr Res, Osijek, Croatia University of JJ Strossmayer Osijek; University of JJ Strossmayer Osijek
Banjari, I (corresponding author), Univ Osijek, Fac Food Technol Osijek, Dept Food & Nutr Res, Osijek, Croatia.
ibanjari@ptfos.hr Rolić, Tara/MHR-3375-2025; Banjari, Ines/AFV-2245-2022 107 0 0
1 3 SHIRAZ UNIV MEDICAL SCIENCES SHIRAZ NEMAZEE HOSPITAL, SHIRAZ, 71934, IRAN 2008-6709 2008-6687
MIDDLE EAST J CANCER Middle East J. Cancer JUL 2024 15
3 163 175
10.30476/mejc.2023.99357.1939
http://dx.doi.org/10.30476/mejc.2023.99357.1939
13 Oncology Emerging Sources Citation Index (ESCI)
Oncology WG5M5 2025-06-24
WOS:001253729900002
J Ergas, IJ; Cheng, RK; Roh, JM; Kushi, LH; Kresovich, JK; Iribarren, C; Nguyen-Huynh, M; Rana, JS; Rillamas-Sun, E; Laurent, CA; Lee, VS; Quesenberry, CP; Greenlee, H; Kwan, ML
Ergas, Isaac J.; Cheng, Richard K.; Roh, Janise M.; Kushi, Lawrence H.; Kresovich, Jacob K.; Iribarren, Carlos; Nguyen-Huynh, Mai; Rana, Jamal S.; Rillamas-Sun, Eileen; Laurent, Cecile A.; Lee, Valerie S.; Quesenberry, Charles P.; Greenlee, Heather; Kwan, Marilyn L. Diet quality and cardiovascular disease risk among breast cancer survivors in the Pathways Study JNCI CANCER SPECTRUM English
Article CORONARY-HEART-DISEASE; MORTALITY; ADHERENCE; PATTERNS Background Women with breast cancer are at higher risk of cardiovascular disease (CVD) compared with women without breast cancer. Whether higher diet quality at breast cancer diagnosis lowers this risk remains unknown. We set out to determine if higher diet quality at breast cancer diagnosis was related to lower risk of CVD and CVD-related death.Methods This analysis included 3415 participants from the Pathway Study, a prospective cohort of women diagnosed with invasive breast cancer at Kaiser Permanente Northern California between 2005 and 2013 and followed through December 31, 2021. Scores from 5 diet quality indices consistent with healthy eating were obtained at the time of breast cancer diagnosis. Scores were categorized into ascending quartiles of concordance for each diet quality index, and multivariable adjusted hazard ratios (HRs) and 95% confidence intervals (CIs) were estimated. P values were 2-sided.Results The Dietary Approaches to Stop Hypertension diet quality index was associated with lower risk of heart failure (HR = 0.53, 95% CI = 0.33 to 0.87; Ptrend = .03), arrhythmia (HR = 0.77, 95% CI = 0.62 to 0.94; Ptrend = .008), cardiac arrest (HR = 0.77, 95% CI = 0.61 to 0.96; Ptrend = .02), valvular heart disease (HR = 0.79, 95% CI = 0.64 to 0.98; Ptrend = .046), venous thromboembolic disease (HR = 0.75, 95% CI = 0.60 to 0.93; Ptrend = .01), and CVD-related death (HR = 0.70, 95% CI = 0.50 to 0.99; Ptrend = .04), when comparing the highest with lowest quartiles. Inverse associations were also found between the healthy plant-based dietary index and heart failure (HR = 0.60, 95% CI = 0.39 to 0.94; Ptrend = .02), as well as the alternate Mediterranean dietary index and arrhythmia (HR = 0.74, 95% CI = 0.60 to 0.93; Ptrend = .02).Conclusion Among newly diagnosed breast cancer patients, higher diet quality at diagnosis was associated with

lower risk of CVD events and death. [Ergas, Isaac J.; Roh, Janise M.; Kushi, Lawrence H.; Iribarren, Carlos; Nguyen-Huynh, Mai; Rana, Jamal S.; Laurent, Cecile A.; Lee, Valerie S.; Quesenberry, Charles P.; Kwan, Marilyn L.] Kaiser Permanente Northern Calif, Div Res, 2000 Broadway, Oakland, CA 94612 USA; [Cheng, Richard K.; Greenlee, Heather] Univ Washington, Med Ctr, Seattle, WA USA; [Kresovich, Jacob K.] H Lee Moffitt Canc Ctr & Res Inst, Tampa, FL USA; [Rana, Jamal S.] Oakland Med Ctr, Oakland, CA USA; [Greenlee, Heather] Fred Hutchinson Canc Ctr, Div Publ Hlth Sci, Seattle, WA USA Kaiser Permanente; University of Washington; University of Washington Seattle; H Lee Moffitt Cancer Center & Research Institute; Fred Hutchinson Cancer Center
Ergas, IJ (corresponding author), Kaiser Permanente Northern Calif, Div Res, 2000 Broadway, Oakland, CA 94612 USA.
isaac.j.ergas@kp.org Kwan, Marilyn/AAH-4850-2021 Ergas, Isaac/0000-0002-3187-9754 National Cancer Institute [R01CA214057, R01CA105274, U01CA195565] National Cancer Institute(United States Department of Health & Human ServicesNational Institutes of Health (NIH) - USANIH National Cancer Institute (NCI)) This work was supported by the National Cancer Institute (R01CA214057, R01CA105274 and U01CA195565). 31 4 4 0 2 OXFORD UNIV PRESS OXFORD GREAT CLARENDON ST, OXFORD OX2 6DP, ENGLAND
2515-5091 JNCI CANCER SPECT JNCI Cancer Spectr.
FEB 29 2024 8 2
pkae013 10.1093/jncics/pkae013
http://dx.doi.org/10.1093/jncics/pkae013 12
Oncology Emerging Sources Citation Index (ESCI) Oncology
NV6J1 38627946 gold 2025-06-24
WOS:001203267300001
J Alshuail, N; Alehaideb, Z; Alghamdi, S; Suliman, R; Al-Eidi, H; Ali, R; Barhoumi, T; Almutairi, M; Alwhibi, M; Alghanem, B; Alamro, A; Alghamdi, A; Matou-Nasri, S Alshuail, Nora; Alehaideb, Zeyad; Alghamdi, Sahar; Suliman, Rasha; Al-Eidi, Hamad; Ali, Rizwan; Barhoumi, Tlili; Almutairi, Mansour; Alwhibi, Mona; Alghanem, Bandar; Alamro, Abir; Alghamdi, Amani; Matou-Nasri, Sabine Achillea fragrantissima (Forssk.) Sch.Bip Flower Dichloromethane Extract Exerts Anti-Proliferative and Pro-Apoptotic Properties in Human Triple-Negative Breast Cancer (MDA-MB-231) Cells: In Vitro and In Silico Studies
PHARMACEUTICALS English Article
Achillea fragrantissima; carbonic anhydrase; caspase activation; mitochondrial apoptosis pathway; triple-negative breast cancer CARBONIC-ANHYDRASE IX; NATURAL-PRODUCTS; CONSTITUENTS; EXPRESSION; INHIBITION; QUERCETIN; DISCOVERY; TUBULIN; BINDING; PLANTS The aggressive triple-negative breast cancer (TNBC) is a challenging disease due to the absence of tailored therapy. The search for new therapies involves intensive research focusing on natural sources. Achillea fragrantissima (A. fragrantissima) is a traditional medicine from the Middle East region. Various solvent extracts from different A. fragrantissima plant parts, including flowers, leaves, and roots, were tested on TNBC MDA-MB-231 cells. Using liquid chromatography, the fingerprinting revealed rich and diverse compositions for A. fragrantissima plant parts using polar to non-polar solvent extracts indicating possible differences in bioactivities. Using the CellTiter-Glo (TM) viability assay, the half-maximal inhibitory concentration (IC50) values were determined for each

extract and ranged from 32.4 to 161.7 mu g/mL. The A. fragrantissima flower dichloromethane extract had the lowest mean IC50 value and was chosen for further investigation. Upon treatment with increasing A. fragrantissima flower dichloromethane extract concentrations, the MDA-MB-231 cells displayed, in a dose-dependent manner, enhanced morphological and biochemical hallmarks of apoptosis, including cell shrinkage, phosphatidylserine exposure, caspase activity, and mitochondrial outer membrane permeabilization, assessed using phase-contrast microscopy, fluorescence-activated single-cell sorting analysis, Image-iT (TM) live caspase, and mitochondrial transition pore opening activity, respectively. Anticancer target prediction and molecular docking studies revealed the inhibitory activity of a few A. fragrantissima flower dichloromethane extract-derived metabolites against carbonic anhydrase IX, an enzyme reported for its anti-apoptotic properties. In conclusion, these findings suggest promising therapeutic values of the A. fragrantissima flower dichloromethane extract against TNBC development. [Alshuail, Nora; Alamro, Abir; Alghamdi, Amani] King Saud Univ, Coll Sci, Biochem Dept, Riyadh 11495, Saudi Arabia; [Alshuail, Nora; Alehaideb, Zeyad; Alghamdi, Sahar; Suliman, Rasha; Ali, Rizwan; Barhoumi, Tlili; Alghanem, Bandar] King Saud bin Abdulaziz Univ Hlth Sci KSAU HS, King Abdullah Int Med Res Ctr KAIMRC, Minist Natl Guard Hlth Affairs MNGHA, Med Res Core Facil & Platforms, Riyadh 11481, Saudi Arabia; [Alghamdi, Sahar; Suliman, Rasha] King Saud bin Abdulaziz Univ Hlth Sci KSAU HS, Coll Pharm, Pharmaceut Sci Dept, Riyadh 11481, Saudi Arabia; [Al-Eidi, Hamad; Matou-Nasri, Sabine] King Saud bin Abdulaziz Univ Hlth Sci KSAU HS, King Abdullah Int Med Res Ctr KAIMRC, Med Genom Res Dept,Cell & Gene Therapy Grp, Minist Natl Guard Hlth Affairs MNGHA, Riyadh 11481, Saudi Arabia; [Almutairi, Mansour] King Saud bin Abdulaziz Univ Hlth Sci KSAU HS, King Abdullah Int Med Res Ctr KAIMRC, Dev Med Dept, Minist Natl GuardHlth Affairs MNGHA, Riyadh 11481, Saudi Arabia; [Alwhibi, Mona] King Saud Univ, Coll Sci, Bot & Microbiol Dept, Riyadh 11495, Saudi Arabia; [Matou-Nasri, Sabine] King Saud bin Abdulaziz Univ Hlth Sci KSAU HS, King Abdullah Int Med Res Ctr KAIMRC, Cellular Therapy & Canc Res Dept, Minist Natl Guard Hlth Affairs MNGHA, Riyadh 11481, Saudi Arabia   King Saud University; King Saud Bin Abdulaziz University for Health Sciences; King Abdullah International Medical Research Center (KAIMRC); Ministry of National Guard - Health Affairs; King Saud Bin Abdulaziz University for Health Sciences; King Saud Bin Abdulaziz University for Health Sciences; Ministry of National Guard - Health Affairs; King Abdullah International Medical Research Center (KAIMRC); King Saud Bin Abdulaziz University for Health Sciences; King Abdullah International Medical Research Center (KAIMRC); King Saud University; King Saud Bin Abdulaziz University for Health Sciences; Ministry of National Guard - Health Affairs; King Abdullah International Medical Research Center (KAIMRC) Matou-Nasri, S (corresponding author), King Saud bin Abdulaziz Univ Hlth Sci KSAU HS, King Abdullah Int Med Res Ctr KAIMRC, Med Genom Res Dept,Cell & Gene Therapy Grp, Minist Natl Guard Hlth Affairs MNGHA, Riyadh 11481, Saudi Arabia.; Matou-Nasri, S (corresponding author), King Saud bin Abdulaziz Univ Hlth Sci KSAU HS, King Abdullah Int Med Res Ctr KAIMRC, Cellular Therapy & Canc Res Dept, Minist Natl Guard Hlth Affairs MNGHA, Riyadh 11481, Saudi Arabia.
matouepnasrisa@mngha.med.sa Alghamdi, Sahar/GSN-5377-2022; Suliman, Rasha/MAH-7364-2025; Alwahaibi, Mona/M-7398-2019; Ali,

Rizwan/P-3566-2017; Suliman, Rasha S./AAW-9005-2021; Alghamdi, Amani/HIR-6153-2022; Alwhibi, Mona/E-1927-2019 Ali, Rizwan/0000-0002-2475-0258; Suliman, Rasha S./0000-0002-6323-0781; Alghamdi, Amani/0000-0002-7251-1972; Alwhibi, Mona/0000-0002-3420-1164; Alshauil, Nora/0000-0003-4276-583X; Alghamdi, Sahar/0000-0002-2770-218X; Matou-Nasri, Sabine/0000-0003-4372-2903; Alghanem, Bandar/0000-0002-3414-6580; Al-eidi, Hamad/0000-0002-8495-7111; Alehaideb, Zeyad/0000-0002-7185-2820 King Abdullah International Medical Research Center (KAIMRC) (Riyadh, Saudi Arabia) [RC16/175/R] King Abdullah International Medical Research Center (KAIMRC) (Riyadh, Saudi Arabia) This study was fully funded by King Abdullah International Medical Research Center (KAIMRC) (Riyadh, Saudi Arabia) under grant number: RC16/175/R. The funder had no role in study design, data collection and analysis, preparation of the manuscript or decision to publish. 69 14 14 0 2 MDPI BASEL ST ALBAN-ANLAGE 66, CH-4052 BASEL, SWITZERLAND 1424-8247
PHARMACEUTICALS-BASE Pharmaceuticals SEP 2022 15 9
1060 10.3390/ph15091060
http://dx.doi.org/10.3390/ph15091060 30
Chemistry, Medicinal; Pharmacology & Pharmacy Science Citation Index Expanded (SCI-EXPANDED) Pharmacology & Pharmacy
4R3TH 36145281 gold, Green Published 2025-06-24 WOS:000856690100001
J Sidahmed, E; Cornellier, ML; Ren, J; Askew, LM; Li, Y; Talaat, N; Rapai, MS; Ruffin, MT; Turgeon, DK; Brenner, D; Sen, A; Djuric, Z Sidahmed, E.; Cornellier, M. L.; Ren, J.; Askew, L. M.; Li, Y.; Talaat, N.; Rapai, M. S.; Ruffin, M. T.; Turgeon, D. K.; Brenner, D.; Sen, A.; Djuric, Z.
Development of exchange lists for Mediterranean and Healthy Eating Diets: implementation in an intervention trial JOURNAL OF HUMAN NUTRITION AND DIETETICS English Article
Mediterranean diet; modified exchange lists; overweight; telephone counselling COLORECTAL-CANCER; CARDIOVASCULAR RISK; RANDOMIZED-TRIAL; VEGETABLE INTAKE; FATTY-ACIDS; COLON CARCINOGENESIS; METABOLIC SYNDROME; BREAST-CANCER; BODY-WEIGHT; STYLE DIET Background: There has been little research published on the adaptation of diabetic exchange list diet approaches for the design of intervention diets in health research despite their clinical utility. The exchange list approach can provide clear and precise guidance on multiple dietary changes simultaneously. The present study aimed to develop exchange list diets for Mediterranean and Healthy Eating, and to evaluate adherence, dietary intakes and markers of health risks with each counselling approach in 120 subjects at increased risk for developing colon cancer. Methods: A randomised clinical trial was implemented in the USA involving telephone counselling. The Mediterranean diet had 10 dietary goals targeting increases in mono-unsaturated fats, n-3 fats, whole grains and the amount and variety of fruits and vegetables. The Healthy Eating diet had five dietary goals that were based on the US Healthy People 2010 recommendations. Results: Dietary compliance was similar in both diet arms, with 82-88% of goals being met at 6 months, although subjects took more time to achieve the Mediterranean goals than the Healthy Eating goals. The relatively modest fruit and vegetable goals in the Healthy Eating arm were exceeded, resulting in fruit and vegetable intakes of approximately eight servings per day in each arm after 6 months. A significant ($P < 0.05$) weight

loss and a decrease in serum C-reactive protein concentrations
were observed in the overweight/obese subgroup of subjects in the
Mediterranean arm in the absence of weight loss goals.
Conclusions: Counselling for the Mediterranean diet may be useful
for both improving diet quality and for achieving a modest weight
loss in overweight or obese individuals. [Sidahmed, E.;
Djuric, Z.] Univ Michigan, Dept Environm Hlth Sci, Ann Arbor, MI
48109 USA; [Cornellier, M. L.; Ren, J.; Askew, L. M.; Li, Y.;
Rapai, M. S.; Ruffin, M. T.; Sen, A.; Djuric, Z.] Univ Michigan,
Dept Family Med, Ann Arbor, MI 48109 USA; [Talaat, N.] Oakwood
Hosp & Med Ctr, Dept Internal Med, Dearborn, MI USA; [Turgeon, D.
K.; Brenner, D.] Univ Michigan, Dept Internal Med, Ann Arbor, MI
48109 USA; [Sen, A.] Univ Michigan, Dept Biostat, Ann Arbor, MI
48109 USA University of Michigan System; University of Michigan;
University of Michigan System; University of Michigan; Beaumont
Health; University of Michigan System; University of Michigan;
University of Michigan System; University of Michigan Djuric, Z
(corresponding author), Univ Michigan, Ctr Canc, 1500 E Med Ctr
Dr,Room 2150, Ann Arbor, MI 48109 USA. zoralong@umich.edu
Anand, Amit/D-4232-2013; Djuric, Zora/H-5147-2013
Cornellier, Maria L./0000-0003-2145-9884; Djuric, Zora/0000-
0002-8886-8853; Ruffin, Mack/0000-0001-8336-478X; Turgeon, D.
Kim/0000-0003-1010-819X NIH [RO1 CA120381, P30 CA130810 S1,
5P60 DK20572, P30 CA046592]; Clinical Translational Science Award,
NIH [UL1RR024986]; National Cancer Institute [P30CA046592] Funding
Source: NIH RePORTER; National Institute of Diabetes and Digestive
and Kidney Diseases [P30DK089503, P30DK020572] Funding Source: NIH
RePORTER NIH(United States Department of Health & Human
ServicesNational Institutes of Health (NIH) - USA); Clinical
Translational Science Award, NIH; National Cancer Institute(United
States Department of Health & Human ServicesNational Institutes of
Health (NIH) - USANIH National Cancer Institute (NCI)); National
Institute of Diabetes and Digestive and Kidney Diseases(United
States Department of Health & Human ServicesNational Institutes of
Health (NIH) - USANIH National Institute of Diabetes & Digestive &
Kidney Diseases (NIDDK)) This study was supported by NIH grants
RO1 CA120381, P30 CA130810 S1 and Cancer Center Support Grant P30
CA046592. The study used core resources supported by a Clinical
Translational Science Award, NIH grant UL1RR024986 (the Michigan
Clinical Research Unit) and the Michigan Diabetes Research and
Training Center funded by NIH grant 5P60 DK20572 (Chemistry
Laboratory). 58 23 25 0 15 WILEY-BLACKWELL
HOBOKEN 111 RIVER ST, HOBOKEN 07030-5774, NJ USA
0952-3871 1365-277X J HUM NUTR DIET J. Hum. Nutr.
Diet. OCT 2014 27 5 413 425
10.1111/jhn.12158 http://dx.doi.org/10.1111/jhn.12158
13 Nutrition & Dietetics Science Citation
Index Expanded (SCI-EXPANDED) Nutrition & Dietetics AU5DZ
24112099 Green Accepted, Green Submitted
2025-06-24 WOS:000345628700002
J Celep, AGS; Yilmaz, S; Coruh, N Celep,
Adviye Gulcin Sagdicoglu; Yilmaz, Sukran; Coruh, Nursen
Antioxidant Capacity and Cytotoxicity of Aesculus
hippocastanum on Breast Cancer MCF-7 Cells JOURNAL OF FOOD AND
DRUG ANALYSIS English Article
Aesculus hippocastanum L.; antioxidant capacity;
cytotoxicity; MCF-7 CHRONIC VENOUS INSUFFICIENCY; RETRACTED
ARTICLE. SEE; HORSE CHESTNUT EXTRACT; BETA-ESCIN; ENDOTHELIAL-

CELLS; PROLIFERATION; AESCIN; APOPTOSIS; FLAVONOIDS; ASSAY

Aesculus hippocastanum L. is a native tree of Asia. Its leaves, seeds and flowers have long been used in folk medicine and in traditional food ingredients. In this study, the bark, seeds, leaves and flowers as aerial parts of A. hippocastanum were extracted in ethanol. The antioxidant capacity of each part was determined for its 1,1-diphenyl-2-picrylhydrazyl radical (DPPH) scavenging capacity, microsomal lipid peroxidation inhibition capacity and total phenolic content. Among all the parts examined, the bark extract of A. hippocastanum revealed the highest antioxidant capacity with an IC50 value of 0.025 mg/mL and 0.014 mg/mL for the inhibition of lipid peroxidation and for the scavenging of DPPH radical, respectively. The bark extract was further examined for its cytotoxic effect on human breast cancer cells (MCF-7) and on healthy cells (3T3) using the MTT method. Cell viability was reduced to 30% upon the addition of 0.5 mg/mL bark extract for both cell lines. [Coruh, Nursen] Middle E Tech Univ, Dept Chem, TR-06531 Ankara, Turkey; [Celep, Adviye Gulcin Sagdicoglu] Middle E Tech Univ, Dept Biochem, Grad Sch Nat & Appl Sci, TR-06531 Ankara, Turkey; [Celep, Adviye Gulcin Sagdicoglu] Gazi Univ, Ind Arts Educ Fac, Ankara, Turkey; [Yilmaz, Sukran] Inst Foot & Mouth Dis, Dept Cell & Virus Bank, Ankara, Turkey

Middle East Technical University; Middle East Technical University; Gazi University; Ministry of Agriculture & Forestry - Turkey Coruh, N (corresponding author), Middle E Tech Univ, Dept Chem, TR-06531 Ankara, Turkey. ncoruh@metu.edu.tr

Yılmaz, Şükran/ABZ-6448-2022; Sagdicoglu Celep, Prof. Dr. Adviye Gulcin/JCE-2215-2023 Yilmaz, Sukran/0000-0002-7945-1124; Sagdicoglu Celep, Adviye Gulcin/0000-0002-4598-5814 State Planning Organization of Turkey (DPT) [DPT-2003-K120920-16]; Scientific Research Grants of the Middle East Technical University [AFP-2001-07-02-00-50]; Institute of Foot and Mouth Diseases, Ankara, Turkey State Planning Organization of Turkey (DPT); Scientific Research Grants of the Middle East Technical University(Middle East Technical University); Institute of Foot and Mouth Diseases, Ankara, Turkey(Gida Tarim Ve Hayvancilik Bakanligi) This study was supported by the State Planning Organization of Turkey (DPT) (Grant No. DPT-2003-K120920-16) and the Scientific Research Grants of the Middle East Technical University (Grant No. AFP-2001-07-02-00-50). We would also like to acknowledge the Institute of Foot and Mouth Diseases, Ankara, Turkey for their support in this study. 43 18 21 0

34 FOOD & DRUG ADMINSTRATION TAIPEI 161-2 KUNYANG STREET, NANGANG, TAIPEI, 00000, TAIWAN 1021-9498 J FOOD DRUG ANAL J. Food Drug Anal. SEP 2012 20 3 692 698 10.6227/jfda.2012200318

http://dx.doi.org/10.6227/jfda.2012200318 7

Food Science & Technology; Pharmacology & Pharmacy Science Citation Index Expanded (SCI-EXPANDED) Food Science & Technology; Pharmacology & Pharmacy 028IB 2025-06-24 WOS:000310414900018

J Costarelli, V; Michou, M Costarelli, Vassiliki; Michou, Maria Predictors of COVID-19 vaccine hesitancy and prevention practice in Greece

INTERNATIONAL JOURNAL OF HEALTH PROMOTION AND EDUCATION English Article

COVID-19 vaccination; prevention; health literacy; Greece

HEALTH LITERACY; LIFE SATISFACTION; PERCEIVED STRESS; GLOBAL

MEASURE; ADULTS Achieving high COVID-19 vaccination rates globally is a key public health challenge. The study aims to investigate factors determining COVID-19 vaccine hesitancy and adherence to prevention measures, in Greek adults. This cross-sectional study was conducted in Greece, from the 21st of February to the 3rd of April 2021, during the strict lockdown period of the 3rd COVID-19 wave. A total of 2029 adults (75.3% women, median age 38 years) participated in an online survey. Participants completed a questionnaire assessing sociodemographic and anthropometric characteristics, adherence to COVID-19 prevention measures and willingness to vaccinate. Health Literacy (HL), perceived stress, life satisfaction and adherence to Mediterranean Diet (MD) were also assessed. Multiple linear and logistic regression analyses were employed. The median score of COVID-19 adherence to the prevention measures tool was 23.08 (interquartile range: 3.83; range: 1 to 35). A total of 57.5% of the participants were willing to vaccinate, 12.1% were unwilling and 30.4% were undecided. Linear regression revealed that women, obese participants, those with a chronic disease and those willing to vaccinate reported higher adherence to prevention measures. Older age, higher levels of HL, life satisfaction and adherence to MD were also positively associated with higher adherence. Logistic regression revealed that being a woman decreases the odds of having the willingness to vaccinate, whereas older age, higher education and adherence to measures increase the odds of willingness to vaccinate. The results could be used by practitioners, researchers and policy makers working in the field of prevention and management of COVID-19. [Costarelli, Vassiliki; Michou, Maria] Harokopio Univ, Dept Econ & Sustainable Dev, Human Ecol Lab, Kallithea, Greece
Harokopio University Athens Costarelli, V (corresponding author), Harokopio Univ Athens, Dept Econ & Sustainable Dev, 70 El Venizelou Ave, Athens 17671, Greece. costarv@hua.gr
Costarelli, Vassiliki/AAM-1210-2020
57 1 1 2 10 ROUTLEDGE JOURNALS, TAYLOR & FRANCIS LTD ABINGDON 2-4 PARK SQUARE, MILTON PARK, ABINGDON OX14 4RN, OXON, ENGLAND 1463-5240 2164-9545 INT J HEALTH PROMOT Int. J. Health Promot. Educ.MAR 3 2024 62 2
98 113
10.1080/14635240.2022.2073554
http://dx.doi.org/10.1080/14635240.2022.2073554 MAY 2022 16 Education, Scientific Disciplines Emerging Sources Citation Index (ESCI) Education & Educational Research RG6P8
2025-06-24 WOS:000794902700001
J Jauhiainen, JS; Vorobeva, E Jauhiainen, Jussi S.; Vorobeva, Ekaterina Syrian refugees and other Syrian forced migrants in Jordan: forms of capital approach
BRITISH JOURNAL OF MIDDLE EASTERN STUDIES
English Article
SOCIAL IDENTITY; IMMIGRANT The article explored human, social, and economic capital of Syrian refugees and other Syrian forced migrants in Jordan. Interrelations of their human, social, and economic forms of capital affected their everyday lives in Jordan as well as their future migration aspirations. The data consisted of a semi-structured survey among 408 Syrian refugees and other forced Syrian migrants in Jordan. Varied combinations of these forms of capital significantly impacted the well-being of these Syrians in the host state. Those who consolidated human capital and possessed dispersed and open social networks created

coping strategies through education and employment that were more likely to be effective, and they considered immigrating to countries that are more developed. Syrians who lacked capital stocks in Jordan often found themselves at the risk of poverty, immobility or forced return to their ruined livelihoods in Syria. The article debunked the myth about powerlessness and passiveness of refugees and other forced migrants and drew attention to their agency and assets. Their levels of vulnerability in the host country significantly varied among individuals with different capital stocks. The authors provided policy recommendations on empowerment of these increasingly vulnerable migrants.
[Jauhiainen, Jussi S.] Univ Turku, Dept Geog & Geol, FL-20014 Turku, Finland; [Jauhiainen, Jussi S.] Univ Tartu, Inst Ecol & Earth Sci, Tartu, Estonia; [Vorobeva, Ekaterina] Univ Bremen, Res Ctr East European Studies, Bremen, Germany University of Turku; University of Tartu; Tartu University Institute of Ecology & Earth Sciences; University of Bremen Jauhiainen, JS (corresponding author), Univ Turku, Dept Geog & Geol, FL-20014 Turku, Finland. jusaja@utu.fi Vorobeva, Ekaterina/HRD-6982-2023 Vorobeva, Ekaterina/0000-0003-4474-6384
Strategic Research Council at the Academy of Finland [303167] Strategic Research Council at the Academy of Finland This work was supported by the Strategic Research Council at the Academy of Finland [303167].
54 1 1 1 5 ROUTLEDGE JOURNALS, TAYLOR & FRANCIS LTD ABINGDON 2-4 PARK SQUARE, MILTON PARK, ABINGDON OX14 4RN, OXON, ENGLAND 1353-0194 1469-3542 BRIT J MIDDLE E STUD Br. J. Middle East. Stud. MAR 15 2023 50 2 281 299
10.1080/13530194.2021.1964068
http://dx.doi.org/10.1080/13530194.2021.1964068 SEP 2021 19 Area Studies; History Social Science Citation Index (SSCI); Arts & Humanities Citation Index (A&HCI) Area Studies; History G5ZK9 hybrid 2025-06-24
WOS:000694585400001
J Qusa, MH; Siddique, A; Nazzal, S; El Sayed, KA
Qusa, Mohammed H.; Siddique, Abu Bakar; Nazzal, Sami; El Sayed, Khalid A.
Novel olive oil phenolic (-)-oleocanthal (+)-xylitol-based solid dispersion formulations with potent oral anti-breast cancer activities
INTERNATIONAL JOURNAL OF PHARMACEUTICS English Article
Extra-virgin olive oil; c-MET receptor tyrosine kinase; (-)-Oleocanthal; Solid dispersion; Taste masking; (+)-Xylitol ELECTRONIC TONGUE; IN-VITRO; STATE CHARACTERIZATION; MEDITERRANEAN DIET; DISSOLUTION RATE; C-MET; XYLITOL; OLEOCANTHAL; SOLUBILITY; EXPRESSION
Epidemiological studies have compellingly documented the ability of the Mediterranean diet rich in extra-virgin olive oil to reduce the incidence of certain malignancies, and cardiovascular diseases, and slow the Alzheimer's disease progression. S-(-)-Oleocanthal (OC) was identified as the most bioactive olive oil phenolic with documented anti-inflammatory, anticancer, and anti-Alzheimer's activities. OC consumption causes irritating sensation at the oropharynx via activation of TRPA1. Accordingly, a taste-masked formulation of OC is needed for its future use as a nutraceutical while maintaining its bioactivity and unique chemistry. Therefore, the goal of this study was to prepare a taste-masked OC solid formulation with improved dissolution and pharmacodynamic profiles, by using (+)-xylitol as an inert carrier. Xylitol was hypothesized to serve as an ideal

vehicle for the preparation of OC solid dispersions due to its low melting point and sweetness. The optimized OC-(+)-xylitol solid dispersion was physically and chemically characterized and showed effective taste masking and enhanced dissolution properties. Furthermore, OC-(+)-xylitol solid dispersion maintained potent in vivo anti-breast cancer activity. It effectively suppressed the human triple negative breast cancer development, growth, and recurrence after primary tumor surgical excision in nude mice orthotopic xenograft models. Collectively, these results suggest the OC-(+)-xylitol solid dispersion formulation as a potential nutraceutical for effective control and prevention of human triple negative breast cancer. [Qusa, Mohammed H.; Siddique, Abu Bakar; El Sayed, Khalid A.] Univ Louisiana Monroe, Sch Basic Pharmaceut & Toxicol Sci, Coll Pharm, 1800 Bienville Dr, Monroe, LA 71201 USA; [Nazzal, Sami] Texas Tech Univ, Dept Pharmaceut Sci, Jerry H Hodge Sch Pharm, Hlth Sci Ctr, 5920 Forest Pk Rd, Dallas, TX 75235 USA University of Louisiana System; University of Louisiana Monroe; Texas Tech University System; Texas Tech University El Sayed, KA (corresponding author), Univ Louisiana Monroe, Sch Basic Pharmaceut & Toxicol Sci, Coll Pharm, 1800 Bienville Dr, Monroe, LA 71201 USA. elsayed@ulm.edu , Abu Bakar Siddique/W-8476-2019 El Sayed, Khalid/0000-0002-1456-4064; Siddique, Abu Bakar/0000-0001-5424-9475 Louisiana Board of Regents [LEQSF (2017-20)-RD-B-07]; National Cancer Institute of the National Institutes of Health [R15CA167475] Louisiana Board of Regents; National Cancer Institute of the National Institutes of Health(United States Department of Health & Human ServicesNational Institutes of Health (NIH) - USANIH National Cancer Institute (NCI)) This work was supported by the Louisiana Board of Regents [grant numbers LEQSF (2017-20)-RD-B-07]; the National Cancer Institute of the National Institutes of Health under [grant number R15CA167475]. 61 30 32 0 20 ELSEVIER AMSTERDAM RADARWEG 29, 1043 NX AMSTERDAM, NETHERLANDS 0378-5173 1873-3476 INT J PHARMACEUT Int. J. Pharm. OCT 5 2019 569 118596 10.1016/j.ijpharm.2019.118596 http://dx.doi.org/10.1016/j.ijpharm.2019.118596 13 Pharmacology & Pharmacy Science Citation Index Expanded (SCI-EXPANDED) Pharmacology & Pharmacy JA8TJ 31394181 Green Accepted 2025-06-24 WOS:000488123900025

J CORRENTI, M; CAVAZZA, ME; GUEDEZ, N; HERRERA, O; SUAREZCHACON, NR CORRENTI, M; CAVAZZA, ME; GUEDEZ, N; HERRERA, O; SUAREZCHACON, NR EXPRESSION OF THE MULTIDRUG-RESISTANCE (MDR) GENE IN BREAST-CANCER JOURNAL OF CHEMOTHERAPY English Article; Proceedings Paper 9th Mediterranean Congress of Chemotherapy JUN, 1994 MILAN, ITALY BREAST CANCER; ONCOGENE; MULTIPLE DRUG RESISTANCE Of the approximately 18,000 new cases of cancer in Venezuela each year, only half can be treated with surgery and radiation. The remainder must be treated systematically using chemotherapy or biological response modifiers. It has become evident that any drug resistant human tumors express the MDR1 gene, since MDR1 RNA levels are elevated in many cancers that do not respond to chemotherapy. Human mammary carcinomas have multiple oncogene alterations, the most frequently reported being overexpression of the oncogenes c-myc, int-2, neu and C-myb. Thirteen specimens of mammary cancer were obtained by

biopsy of untreated patients in stage IIIB. All these patients received three cycles of FAC or CMF-L + GM-CSF after biopsy. In the slot blot analysis of RNA from invasive carcinomas, MDR1 and c-myc transcripts were detectable at a high level in 30% of tumors. Two patients with increased levels of MDR1 before chemotherapy did not respond to the treatment and distant metastasis and death occurred in these patients. Another patient, MDR1-negative before therapy, did not respond to CMF-I + GM-CSF and showed high levels of MDR1 transcripts in a second biopsy which was obtained during surgery. CORRENTI, M (corresponding author), INST ONCOL & HEMATOL,GENET MOLEC LAB,MSAS,APARTADO POSTAL 40025,CARACAS 1020A,VENEZUELA.
cavazza, Maria/0000-0002-9605-8295
0 7 9 0 2 E I F T SRL FLORENCE VIA XX SETTEMBRE 102, 50129 FLORENCE, ITALY 1120-009X J CHEMOTHERAPY J. Chemother. OCT 1995 7 5
449 451 10.1179/joc.1995.7.5.449
http://dx.doi.org/10.1179/joc.1995.7.5.449 3
Oncology; Infectious Diseases; Pathology; Pharmacology & Pharmacy Conference Proceedings Citation Index - Science (CPCI-S); Science Citation Index Expanded (SCI-EXPANDED) Oncology; Infectious Diseases; Pathology; Pharmacology & Pharmacy TB403
8596130 2025-06-24 WOS:A1995TB40300011
J Piscitelli, P; Barba, M; Crespi, M; Di Maio, M; Santoriello, A; D'Aiuto, M; Fucito, A; Losco, A; Pentimalli, F; Maranta, P; Chitano, G; Argentiero, A; Neglia, C; Distante, A; Di Tanna, GL; Brandi, ML; Mazza, A; Marino, IR; Giordano, A
Piscitelli, Prisco; Barba, Maddalena; Crespi, Massimo; Di Maio, Massimo; Santoriello, Antonio; D'Aiuto, Massiliamo; Fucito, Alfredo; Losco, Arturo; Pentimalli, Francesca; Maranta, Pasquale; Chitano, Giovanna; Argentiero, Alberto; Neglia, Cosimo; Distante, Alessandro; Di Tanna, Gian Luca; Brandi, Maria Luisa; Mazza, Alfredo; Marino, Ignazio R.; Giordano, Antonio Human Hlth Fdn Study Grp The burden of breast cancer in Italy: mastectomies and quadrantectomies performed between 2001 and 2008 based on nationwide hospital discharge records JOURNAL OF EXPERIMENTAL & CLINICAL CANCER RESEARCH English
Article Hospital discharge records; Breast cancer; Mastectomies; Quadrantectomies; Cancer surveillance MEDICARE CLAIMS; REGISTRY DATA; DISEASE
Background: Where population coverage is limited, the exclusive use of Cancer Registries might limit ascertainment of incident cancer cases. We explored the potentials of Nationwide hospital discharge records (NHDRs) to capture incident breast cancer cases in Italy. Methods: We analyzed NHDRs for mastectomies and quadrantectomies performed between 2001 and 2008. The average annual percentage change (AAPC) and related 95% Confidence Interval (CI) in the actual number of mastectomies and quadrantectomies performed during the study period were computed for the full sample and for subgroups defined by age, surgical procedure, macro-area and singular Region. Re-admissions of the same patients were separately presented. Results: The overall number of mastectomies decreased, with an AAPC of -2.1% (-2.3 -1.8). This result was largely driven by the values observed for women in the 45 to 64 and 65 to 74 age subgroups (-3.0%, -3.4 -3.6 and -3.3%, -3.8 -2.8, respectively). We observed no significant reduction in mastectomies for women in the remaining age groups. Quadrantectomies showed an overall +4.7 AAPC (95%CI:4.5-4.9), with

no substantial differences by age. Analyses by geographical area showed a remarkable decrease in mastectomies, with inter-regional discrepancies possibly depending upon variability in mammography screening coverage and adherence. Quadrantectomies significantly increased, with Southern Regions presenting the highest average rates. Data on repeat admissions within a year revealed a total number of 46,610 major breast surgeries between 2001 and 2008, with an overall +3.2% AAPC (95%CI:2.8-3.6). Conclusions: In Italy, NHDRs might represent a valuable supplemental data source to integrate Cancer Registries in cancer surveillance.
[Piscitelli, Prisco; Brandi, Maria Luisa] Univ Florence, Dept Internal Med, I-50139 Florence, Italy; [Barba, Maddalena] Regina Elena Inst Canc Res, Sci Direct, I-00144 Rome, Italy; [Di Maio, Massimo] Local Hlth Author Naples ASL NA1, I-80143 Naples, Italy; [Santoriello, Antonio] Univ Naples 2, Dept Gen Surg, I-80131 Naples, Italy; [D'Aiuto, Massiliamo; Fucito, Alfredo] G Pascale Fdn, Natl Canc Inst, Dept Senol, I-80131 Naples, Italy; [Fucito, Alfredo; Maranta, Pasquale; Mazza, Alfredo; Giordano, Antonio] Temple Univ, Coll Sci & Technol, Sbarro Inst Canc Res & Mol Med, Philadelphia, PA 19122 USA; [Fucito, Alfredo; Maranta, Pasquale; Mazza, Alfredo; Giordano, Antonio] Temple Univ, Coll Sci & Technol, Ctr Biotechnol, Philadelphia, PA 19122 USA; [Losco, Arturo] Local Hlth Author Salerno, I-84124 Salerno, Italy; [Pentimalli, Francesca; Giordano, Antonio] G Pascale Fdn, Natl Canc Inst, Canc Res Ctr, INT CROM, I-83013 Avellino, Italy; [Maranta, Pasquale; Giordano, Antonio] Human Hlth Fdn, I-06049 Spoleto Perugia, Italy; [Chitano, Giovanna; Argentiero, Alberto; Neglia, Cosimo; Distante, Alessandro] Furo Mediterranean Biomed Inst ISBEM Res Ctr, I-72023 Brindisi, Mesagne, Italy; [Di Tanna, Gian Luca] Univ Roma La Sapienza, Dept Publ Hlth & Infect Dis, I-00185 Rome, Italy; [Marino, Ignazio R.] Thomas Jefferson Univ, Jefferson Med Coll, Dept Surg, Philadelphia, PA 19107 USA; [Giordano, Antonio] Univ Siena, Dept Human Pathol & Oncol, I-53100 Siena, Italy University of Florence; IRCCS Istituti Fisioterapici Ospitalieri (IFO); IRCCS Regina Elena; Universita della Campania Vanvitelli; IRCCS Fondazione Pascale; Pennsylvania Commonwealth System of Higher Education (PCSHE); Temple University; Pennsylvania Commonwealth System of Higher Education (PCSHE); Temple University; IRCCS Fondazione Pascale; Sapienza University Rome; Thomas Jefferson University; University of Siena

Giordano, A (corresponding author), Temple Univ, Coll Sci & Technol, Sbarro Inst Canc Res & Mol Med, BioLife Sci Bldg,Suite 431,1900 N 12th St, Philadelphia, PA 19122 USA.

giordano@temple.edu Marino, Ignazio/ABB-3729-2020; Di Tanna, Gian/AAF-2352-2019; Argentiero, Alberto/ACW-5026-2022; Piscitelli, Prisco/L-1033-2016; Giordano, Antonio/F-1927-2010; Barba, Maddalena/K-7277-2018; Pentimalli, Francesca/K-4936-2014; DI MAIO, Massimo/K-9990-2016Di Tanna, Gian Luca/0000-0002-5470-3567; ARGENTIERO, ALBERTO/0000-0002-6597-5461; Piscitelli, Prisco/0000-0003-4556-6182; Giordano, Antonio/0000-0002-5959-016X; Barba, Maddalena/0000-0001-9050-2917; Neglia, Cosimo/0000-0002-9863-7178; DISTANTE, Alessandro/0000-0003-2776-0192; Marino, Ignazio/0000-0002-1519-8385; Pentimalli, Francesca/0000-0003-4740-6801; DI MAIO, Massimo/0000-0001-8906-3785 Human Health Foundation, Spoleto (PG), Italy; Sbarro Health Research Organization, Philadelphia, PA; DoD, Army Research and Development; DoH Commonwealth of Pennsylvania Human Health Foundation, Spoleto (PG), Italy; Sbarro Health Research

Organization, Philadelphia, PA; DoD, Army Research and Development(United States Department of Defense); DoH Commonwealth of Pennsylvania This work was supported by the Human Health Foundation, Spoleto (PG), Italy (http://www.hhfonlus.org), the Sbarro Health Research Organization, Philadelphia, PA (http://www.shro.org), the DoD, Army Research and Development, and the DoH Commonwealth of Pennsylvania. Authors are also grateful to the Euro Mediterranean Scientific Institute (ISBEM, Brindisi), for data management and analysis. 23 9 10 0 4
BMC LONDON CAMPUS, 4 CRINAN ST, LONDON N1 9XW, ENGLAND
1756-9966 J EXP CLIN CANC RES J. Exp. Clin. Cancer Res. NOV 20 2012 31
96 10.1186/1756-9966-31-96
http://dx.doi.org/10.1186/1756-9966-31-96 9
Oncology Science Citation Index Expanded (SCI-EXPANDED)
Oncology 075NB 23168067 gold, Green Published
2025-06-24 WOS:000313892500001
J Jo, EH; Hong, HD; Ahn, NC; Jung, JW; Yang, SR; Park, JS; Kim, SH; Lee, YS; Kang, KS Jo, EH; Hong, HD; Ahn, NC; Jung, JW; Yang, SR; Park, JS; Kim, SH; Lee, YS; Kang, KS
Modulations of the Bcl-2/Bax family were involved in the chemopreventive effects of licorice root (Glycyrrhiza uralensis fisch) in MCF-7 human breast cancer cell JOURNAL OF AGRICULTURAL AND FOOD CHEMISTRY English Article
licorice; MCF-7 cell; Bcl-2; Bax; PARP; apoptosis CYCLE ARREST; APOPTOSIS; PHOSPHORYLATION; CONSTITUENTS; CAPACITY; LDL Recently, cancer chemoprevention with strategies using foods and medicinal herbs has been regarded as one of the most visible fields for cancer control. Genistein in soy, American ginseng, and resveratrol are well-known to have antiproliferative properties in human breast cancer. Licorice root is a botanical, a shrub native to southern Europe and Asia, which primarily has desirable qualities in sweetening and herbal medicine. In this study, licorice (Glycyrrhiza uralensis Fisch) root also inhibits cell proliferation in human breast cancer cell. The cell proliferation study demonstrated that licorice root reduced the proliferation of MCF-7 cells in a dose- and time-dependent manner. The extracts were fractionated in CHCL3, EtOAc, C6H14, and CH3OH-H2O (70:30), and these extracts of licorice root (50 mug/mL) induced DNA fragmentation demonstrated by Hoechst 33258 staining. Apoptosis also determined the sub-G1 accumulation by flow cytometry analysis. These results were consistent with specific cleavage of PARP and antiapoptotic protein Bcl-2 and up-regulation of proapoptotic protein Bax demonstrated by Western blotting. Our findings suggest that licorice root may have chemopreventive effects against human breast cancer through the modulation of the expression of the Bcl-2/ Bax family of apoptotic regulatory factors. Seoul Natl Univ, Coll Vet Med, Dept Vet Publ Hlth, Lab Stem Cell & Tumor Biol, Seoul 151742, South Korea; Kyung Hee Univ, Grad Sch EW Med Sci, Yongin, South Korea; Korea Food Res Inst, Sungnam, South Korea Seoul National University (SNU); Kyung Hee University; Korea Food Research Institute (KFRI)
Seoul Natl Univ, Coll Vet Med, Dept Vet Publ Hlth, Lab Stem Cell & Tumor Biol, Seoul 151742, South Korea.
kangpub@snu.ac.kr LEE, SOOYEON/KAM-6204-2024; Kang, Kyung-Sun/G-6205-2013 Kang, Kyung-Sun/0000-0002-9322-741X
22 63 72 1 16 AMER CHEMICAL SOC WASHINGTON 1155 16TH ST, NW, WASHINGTON, DC 20036 USA

0021-8561 1520-5118 J AGR FOOD CHEM J. Agric. Food Chem. MAR 24 2004 52 6 1715 1719
10.1021/jf035012t
http://dx.doi.org/10.1021/jf035012t 5
Agriculture, Multidisciplinary; Chemistry, Applied; Food Science & Technology Science Citation Index Expanded (SCI-EXPANDED) Agriculture; Chemistry; Food Science & Technology
804FW 15030235 2025-06-24
WOS:000220285600048
J Shivappa, N; Hébert, JR; Steck, SE; Hofseth, LJ; Shehadah, I; Bani-Hani, KE; Al-Jaberi, T; Al-Nusairr, M; Heath, D; Tayyem, R
Shivappa, Nitin; Hebert, James R.; Steck, Susan E.; Hofseth, Lorne J.; Shehadah, Ihab; Bani-Hani, Kamal E.; Al-Jaberi, Tareq; Al-Nusairr, Majed; Heath, Dennis; Tayyem, Reema
Dietary inflammatory index and odds of colorectal cancer in a case-control study from Jordan APPLIED PHYSIOLOGY NUTRITION AND METABOLISM English Article
diet; cytokines; nutrition; inflammation; epidemiology; dietary inflammatory index SOCIAL DESIRABILITY BIAS; SELF-REPORT; ENERGY-INTAKE; RISK; ASSOCIATION; VALIDATION; COMPROMISE; BIOMARKERS; VALIDITY; NSAIDS
Dietary components that promote inflammation of the colon have been suggested to be risk factors in the development of colorectal cancer (CRC). The possible link between inflammatory potential of diet and CRC has been investigated in several developed or Western countries. Despite the fact that dietary choices in the Middle East differ markedly from those in the West, results have not been reported from any study conducted in a Middle-Eastern population. We examined the association between dietary inflammatory index (DII) scores and CRC in a case-control study conducted in Jordan. This study included 153 histopathologically confirmed CRC cases and 202 disease-free control subjects' frequency matched on age, sex, and occupation. Data were collected between January 2010 and December 2012, using interviewer-administered questionnaires. DII scores were computed from dietary data reported using a food frequency questionnaire. Logistic regression models were used to estimate odds ratios (ORs) and 95% confidence intervals (CIs) adjusted for age, sex, education, physical activity, body mass index, smoking, and family history of CRC. Subjects with higher DII scores were at increased odds of CRC, with the DII being used both as a continuous variable (ORcontinuous = 1.45, 95% CI: 1.13-1.85; 1-unit increase corresponding to approximate to 20% of its range in the current study) and as a categorical variable (ORtertile 3 vs tertile 1 = 2.13, 95% CI: 1.23-3.72). Our results, based on a Jordanian population, add to the growing literature indicating that a pro-inflammatory diet is associated with increased odds of CRC. [Shivappa, Nitin; Hebert, James R.] Univ South Carolina, Canc Prevent & Control Program, Columbia, SC 29208 USA; [Shivappa, Nitin; Hebert, James R.; Steck, Susan E.] Univ South Carolina, Arnold Sch Publ Hlth, Dept Epidemiol & Biostat, Columbia, SC 29208 USA; [Shivappa, Nitin; Hebert, James R.] Connecting Hlth Innovat LLC, Columbia, SC 29201 USA; [Hofseth, Lorne J.] Univ South Carolina, South Carolina Coll Pharm, Columbia, SC 29208 USA; [Shehadah, Ihab] King Hussein Canc Ctr, Gastroenterol Div, POB 1269, Amman 11941, Jordan; [Bani-Hani, Kamal E.] Hashemite Univ, Fac Med, POB 330127, Zarqa 13133, Jordan; [Al-Jaberi, Tareq] Jordan Univ Sci & Technol, Dept Surg, POB 3030, Irbid 22110, Jordan; [Al-Nusairr, Majed] Prince Hamza

Hosp, Div Gastroenterol, POB 86, Amman 11118, Jordan; [Heath, Dennis] Univ Calif San Diego, Moores Canc Ctr, Canc Prevent & Control Program, La Jolla, CA 92093 USA; [Tayyem, Reema] Univ Jordan, Fac Agr, Dept Nutr & Food Technol, POB 2920, Amman 11941, Jordan University of South Carolina System; University of South Carolina Columbia; University of South Carolina System; University of South Carolina Columbia; Connecting Health Innovations LLC; University of South Carolina System; University of South Carolina Columbia; Medical University of South Carolina; King Hussein Cancer Center; Hashemite University; Jordan University of Science & Technology; University of California System; University of California San Diego; University of Jordan

Shivappa, N (corresponding author), Univ South Carolina, Canc Prevent & Control Program, Columbia, SC 29208 USA.; Shivappa, N (corresponding author), Univ South Carolina, Arnold Sch Publ Hlth, Dept Epidemiol & Biostat, Columbia, SC 29208 USA.; Shivappa, N (corresponding author), Connecting Hlth Innovat LLC, Columbia, SC 29201 USA. shivappa@mailbox.sc.edu Hebert, James/IUO-5628-2023; Shivappa, Nitin/X-2215-2018; Steck, Susan/G-5736-2013; Tayyem, Reema/AAA-5326-2019; Tayyem, Reema/I-1217-2016 Tayyem, Reema/0000-0003-1640-0511 United States National Institute of Diabetes and Digestive and Kidney Diseases [R44DK103377]; Higher Council of Science and Technology United States National Institute of Diabetes and Digestive and Kidney Diseases(United States Department of Health & Human ServicesNational Institutes of Health (NIH) - USANIH National Institute of Diabetes & Digestive & Kidney Diseases (NIDDK)); Higher Council of Science and Technology N. Shivappa and J.R. Hebert were supported by grant number R44DK103377 from the United States National Institute of Diabetes and Digestive and Kidney Diseases. The authors would like to thank the Higher Council of Science and Technology for sponsoring the research projects. 45 23 24 1 16 CANADIAN SCIENCE PUBLISHING OTTAWA 65 AURIGA DR, SUITE 203, OTTAWA, ON K2E 7W6, CANADA 1715-5312 1715-5320 APPL PHYSIOL NUTR ME Appl. Physiol. Nutr. Metab. JUL 2017 42 7 744 749 10.1139/apnm-2017-0035

http://dx.doi.org/10.1139/apnm-2017-0035 6

Nutrition & Dietetics; Physiology; Sport Sciences Science Citation Index Expanded (SCI-EXPANDED) Nutrition & Dietetics; Physiology; Sport Sciences EZ1GW 28226219

2025-06-24 WOS:000404459400009

J Bassatne, A; Harb, H; Jaafar, B; Romanos, J; Ammar, W; Fuleihan, GEH Bassatne, A.; Harb, H.; Jaafar, B.; Romanos, J.; Ammar, W.; Fuleihan, G. El-Hajj

Disease burden of osteoporosis and other non-communicable diseases in Lebanon OSTEOPOROSIS INTERNATIONAL

English Article Hip fractures; Osteoporosis; Major osteoporotic fractures; Middle East; Non-communicable diseases POSTMENOPAUSAL WOMEN; VERTEBRAL FRACTURES; PREVALENCE; OLDER; DIAGNOSIS; CANCER; MANAGEMENT; MORTALITY; DENSITY; IMPACT Osteoporosis is more common than most feared non-communicable diseases in the Middle East. This justifies the need to place osteoporosis as a health priority in the region. Introduction Osteoporosis is a common disease associated with severe debilitating consequences. The objective of this study is To evaluate and compare disease burden from osteoporosis and other non-communicable diseases (NCDs) in Lebanon. Methods We assessed the prevalence of osteoporosis and

other NCDs, such as obesity, diabetes, hypertension, dyslipidemia, and cardiovascular diseases, based on a published population-based study of Lebanese >= 65 years. We compared incidence rates of hip fractures and major osteoporotic fractures (MOF) (spine, hip, humerus, and forearm) to the five commonest cancers in women >= 50 years. Rates were based on the national hip fracture and cancer registry data, provided by the Lebanese Ministry of Public Health. MOF incidence rates were derived from national hip fracture incidence rates and MOF/hip fractures incidence rate ratios from the literature. Results Over 70% of elderly Lebanese had osteoporosis defined by densitometric criteria or prevalent morphometric vertebral fractures. This by far exceeded the prevalence of other NCDs, such as hypertension (53%), diabetes (21%), dyslipidemia (31%), and cardiovascular diseases (30%). Morphometric vertebral fractures (grades 2 and 3) were present in 19% of women and 12% of men. The incidence rates for MOF were 1.6 times greater than those for breast cancer, and 7.4-9.9 folds higher than those for the next commonest cancers of the lungs, colon, and ovaries. Hip fracture incidence rates were lower than those of breast cancer but were 2.1-2.8 folds higher than those of the above-mentioned cancers. Conclusion This first of its kind study in the Middle East demonstrates that osteoporosis is a common disease, more common than most feared NCDs. Our findings are comparable to those in western populations and justify placing osteoporosis on the top of NCDs' priority list in our country and possibly the region. [Bassatne, A.; Jaafar, B.; Fuleihan, G. El-Hajj] Amer Univ Beirut, Dept Internal Med, Div Endocrinol & Metab, Calcium Metab & Osteoporosis Program,Med Ctr, Beirut, Lebanon; [Harb, H.; Romanos, J.; Ammar, W.] Minist Publ Hlth, Beirut, Lebanon American University of Beirut Fuleihan, GEH (corresponding author), Amer Univ Beirut, Dept Internal Med, Div Endocrinol & Metab, Calcium Metab & Osteoporosis Program,Med Ctr, Beirut, Lebanon. gf01@aub.edu.lb Amini, Erfan/X-8366-2019 El-Hajj Fuleihan, Ghada/0000-0002-2076-5858; Bassatne, Aya/0000-0002-7841-3986 Office of Dietary Supplements of the National Institutes of Health [D43 TW009118] Office of Dietary Supplements of the National Institutes of Health(United States Department of Health & Human ServicesNational Institutes of Health (NIH) - USA) AB received training under the Scholars in HeAlth Research Program (SHARP), Fogarty International Center and Office of Dietary Supplements of the National Institutes of Health under Award Number D43 TW009118. The content is solely the responsibility of the authors and does not necessarily represent the official views of the National Institutes of Health. The authors would like to thank Mr. Ali Hammoudi for his assistance in the figures' preparation. 48 6 6 0 3 SPRINGER LONDON LTD LONDON 236 GRAYS INN RD, 6TH FLOOR, LONDON WC1X 8HL, ENGLAND 0937-941X 1433-2965 OSTEOPOROSIS INT Osteoporosis Int. SEP 2020 31 9 1769 1777 10.1007/s00198-020-05433-w http://dx.doi.org/10.1007/s00198-020-05433-w MAY 2020 9 Endocrinology & Metabolism Science Citation Index Expanded (SCI-EXPANDED) Endocrinology & Metabolism MZ6PM 32377809 2025-06-24 WOS:000530801200004

J Ly, TTG; Yun, J; Lee, DH; Chung, JS; Kwon, SM

Ly, Thanh Truong Giang; Yun, Jisoo; Lee, Dong-Hyung; Chung, Joo-Seop; Kwon, Sang-Mo Protective Effects and Benefits of Olive Oil and Its Extracts on Women's Health

NUTRIENTS English Article
olive oil; mediterranean diet; oleuropein; hydroxytyrosol; breast cancer; gynecologic cancer; osteoporosis; postmenopausal disorders CANCER CELL-PROLIFERATION; FATTY-ACID SYNTHASE; BREAST-CANCER; MEDITERRANEAN DIET; PHENOLIC-COMPOUNDS; BONE LOSS; ENDOTHELIAL FUNCTION; OVARIAN-CANCER; TRANSCRIPTION FACTORS; MOLECULAR-MECHANISMS Women and men share similar diseases; however, women have unique issues, including gynecologic diseases and diseases related to menstruation, menopause, and post menopause. In recent decades, scientists paid more attention to natural products and their derivatives because of their good tolerability and effectiveness in disease prevention and treatment. Olive oil is an essential component in the Mediterranean diet, a diet well known for its protective impact on human well-being. Investigation of the active components in olive oil, such as oleuropein and hydroxytyrosol, showed positive effects in various diseases. Their effects have been clarified in many suggested mechanisms and have shown promising results in animal and human studies, especially in breast cancer, ovarian cancer, postmenopausal osteoporosis, and other disorders. This review summarizes the current evidence of the role of olives and olive polyphenols in women's health issues and their potential implications in the treatment and prevention of health problems in women. [Ly, Thanh Truong Giang; Yun, Jisoo; Kwon, Sang-Mo] Pusan Natl Univ, Med Res Inst, Lab Vasc Med & Stem Cell Biol, Dept Physiol,Sch Med, Yangsan 50612, South Korea; [Ly, Thanh Truong Giang; Yun, Jisoo; Kwon, Sang-Mo] Pusan Natl Univ, Convergence Stem Cell Res Ctr, Yangsan 50612, South Korea; [Lee, Dong-Hyung] Pusan Natl Univ Yangsan Hosp, Dept Obstet & Gynecol, Yangsan 50612, South Korea; [Chung, Joo-Seop] Pusan Natl Univ Hosp, Med Res Inst, Dept Hematol Oncol, Busan 49241, South Korea Pusan National University; Pusan National University; Pusan National University; Pusan National University Hospital; Pusan National University; Pusan National University Hospital Kwon, SM (corresponding author), Pusan Natl Univ, Med Res Inst, Lab Vasc Med & Stem Cell Biol, Dept Physiol,Sch Med, Yangsan 50612, South Korea.; Kwon, SM (corresponding author), Pusan Natl Univ, Convergence Stem Cell Res Ctr, Yangsan 50612, South Korea.; Chung, JS (corresponding author), Pusan Natl Univ Hosp, Med Res Inst, Dept Hematol Oncol, Busan 49241, South Korea.
lythanhtruonggiang@gmail.com; jsyun14@hanmail.net; ldh0707@hanmail.net; hemon@pusan.ac.kr; smkwon323@pusan.ac.kr
National Research Foundation of Korea [2021R1I1A3055686, NRF-2020R1A2C2101297, NRF-2015R1A5A2009656]; Korean Health Technology R&D Project, Ministry of Health and Welfare, Republic of Korea [HI18C2459, HI18C2458] National Research Foundation of Korea(National Research Foundation of Korea); Korean Health Technology R&D Project, Ministry of Health and Welfare, Republic of Korea FundingThis work was supported by the National Research Foundation of Korea (grant nos. 2021R1I1A3055686, NRF-2020R1A2C2101297, and NRF-2015R1A5A2009656) and the Korean Health Technology R&D Project, Ministry of Health and Welfare, Republic of Korea (grant nos. HI18C2459 and HI18C2458). 174 9 9 1 14 MDPI BASEL MDPI AG, Grosspeteranlage 5, CH-4052 BASEL, SWITZERLAND 2072-6643
NUTRIENTS Nutrients DEC 2021 13 12
4279 10.3390/nu13124279
http://dx.doi.org/10.3390/nu13124279 21

Nutrition & Dietetics Science Citation Index Expanded (SCI-EXPANDED) Nutrition & Dietetics XY3OW 34959830 gold, Green Published 2025-06-24 WOS:000736887100001

J del Saz-Lara, A; Boughanem, H; de las Hazas, MCL; Crespo, C; Saz-Lara, A; Visioli, F; Macias-González, M; Dávalos, A

del Saz-Lara, Andrea; Boughanem, Hatim; Lopez de las Hazas, Maria-Carmen; Crespo, Carmen; Saz-Lara, Alicia; Visioli, Francesco; Macias-Gonzalez, Manuel; Davalos, Alberto

Hydroxytyrosol decreases EDNRA expression through epigenetic modification in colorectal cancer cells PHARMACOLOGICAL RESEARCH English Article

Hydroxytyrosol; DNA methylation; EDNRA; CpGs; Extra virgin olive oil; Caco-2 cells; Cancer VIRGIN OLIVE OIL; COLON; DIET

The Mediterranean diet (MD) is one of the healthiest ones and is associated with a lower incidence of cardio-vascular and cerebrovascular diseases as well as cancer. Extra virgin olive oil (EVOO) is probably the most idiosyncratic component of this diet. EVOO has been attributed with many healthful effects, which may be due to its phenolic components, e.g. including hydroxytyrosol (HT). Recent studies suggest that EVOO and HT have molecular targets in human tissues and modulate epigenetic mechanisms. DNA methylation is one of the most studied epigenetic mechanisms and consists of the addition of a methyl group to the cytosines of the DNA chain. Given the purported health effects of EVOO (poly)phenols, we analyzed the changes induced by HT in DNA methylation, in a colorectal cancer cell line. Caco-2 cells were treated with HT for one week or with the demethylating agent 5'-azacytidine for 48 h. Global DNA methylation was assessed by ELISA. DNA bisulfitation was performed and Infinium Methylation EPIC BeadChips were used to analyze the specific methylation of CpG sites. We show an increase in global DNA methylation in Caco-2 cells after HT treatment, with a total of 32,141 differentially methylated (CpGs DMCpGs). Interestingly, our analyses revealed the endothelin receptor type A gene (EDNRA) as a possible molecular target of HT. In summary, we demonstrate that cellular supplementation with HT results in a specific methylome map and propose a potential gene target for HT.[del Saz-Lara, Andrea; Lopez de las Hazas, Maria-Carmen; Davalos, Alberto] CEI UAM CSIC, Madrid Inst Adv Studies IMDEA Food, Lab Epigenet Lipid Metab, Madrid 28049, Spain; [del Saz-Lara, Andrea; Crespo, Carmen; Visioli, Francesco] CEI UAM CSIC, Madrid Inst Adv Studies IMDEA Food, Lab Funct Foods, Madrid 28049, Spain; [Boughanem, Hatim; Macias-Gonzalez, Manuel] Univ Malaga, Virgen Victoria Univ Hosp, Inst Biomed Res Malaga IBIMA, Dept Endocrinol & Nutr, Malaga, Spain; [Boughanem, Hatim; Macias-Gonzalez, Manuel] Inst Salud Carlos III, Ctr Invest Biome Red Fisiopatol Obes & Nutr, Madrid, Spain; [Saz-Lara, Alicia] Univ Castilla La Mancha, Hlth & Social Res Ctr, Cuenca 16171, Spain; [Visioli, Francesco] Univ Padua, Dept Mol Med, Padua, Italy Consejo Superior de Investigaciones Cientificas (CSIC); IMDEA Food Institute; Consejo Superior de Investigaciones Cientificas (CSIC); IMDEA Food Institute; Universidad de Malaga; Instituto de Investigacion Biomedica de Malaga y Plataforma en Nanomedicina (IBIMA); Instituto de Salud Carlos III; Universidad de Castilla-La Mancha; University of Padua

Dávalos, A (corresponding author), CEI UAM CSIC, Madrid Inst Adv Studies IMDEA Food, Lab Epigenet Lipid Metab, Madrid 28049, Spain.; Visioli, F (corresponding author), Univ Padua, Dept Mol Med, Padua, Italy.; Macias-González, M (corresponding author),

Hosp Virgen Victoria, Unidad Gest Clin Endocrinol & Nutr, Inst Invest Biomed Malaga IBIMA, Malaga 29010, Spain.
francesco.visioli@imdea.org López-Gil, José Francisco/AAN-5618-2020; del Carmen Crespo, María/P-3925-2015; Macias-Gonzalez, Manuel/E-7584-2016; Davalos, Alberto/AAA-9949-2021; Boughanem, Hatim/AAB-4890-2020; Visioli, Francesco/J-9356-2013; Lopez de las Hazas, Maria Carmen/L-3033-2017 Visioli, Francesco/0000-0002-1756-1723; Lopez de las Hazas, Maria Carmen/0000-0001-8199-6724; del Saz Lara, Andrea/0000-0002-2264-6027; Boughanem, Hatim/0000-0001-7743-311X; Crespo, Maria del Carmen/0000-0003-0546-2143
Spanish Agencia Estatal de Investigacion; Ministry of Science and Innovation, Spain; Consejeria de innovacion junta de Andalucia [PID2019-109369RB-I00, MCIN/AEI/10.13039/501100011033]; instituto de Salud Carlos III-Fondo de investigacion Sanitaria [PAIDI PY20-01270]; Juan de la Cierva [PI21/00633]; International Olive Council [IJC2020-044353-/MCIN/AEI/10.13039/501100011033/EU/PRTR]; Investigacion Biomedica en Red Fisiopatologia de la Obesidad y Nutricion [2021-01-PhD GRANT]; European Regional Development Fund; ISCIII [CB06/03]; Servicio Andaluz de Salud, Junta de Andalucia, Spain [PI18/01399, Predoc20_002]; [RC-0001-2018]; [C-0029-2014] Spanish Agencia Estatal de Investigacion(Spanish Government); Ministry of Science and Innovation, Spain(Spanish Government); Consejeria de innovacion junta de Andalucia(Junta de Andalucia); instituto de Salud Carlos III-Fondo de investigacion Sanitaria(Instituto de Salud Carlos IIISpanish Government); Juan de la Cierva(Instituto de Salud Carlos IIISpanish Government); International Olive Council; Investigacion Biomedica en Red Fisiopatologia de la Obesidad y Nutricion; European Regional Development Fund(European Union (EU)); ISCIII(Instituto de Salud Carlos IIISpanish Government); Servicio Andaluz de Salud, Junta de Andalucia, Spain(Junta de Andalucia); ; This work was supported by grants from the Spanish Agencia Estatal de Investigacion and European FEDER Funds to AD (PID2019-109369RB-I00) from the Ministry of Science and Innovation (MCIN/AEI/10.13039/501100011033, Spain) ; This work was also supported by Consejeria de innovacion junta de Andalucia (PAIDI PY20-01270) and by instituto de Salud Carlos III-Fondo de investigacion Sanitaria (PI21/00633) . MCLH is a recipient of a Juan de la Cierva Grant IJC2020-044353-/MCIN/AEI/10.13039/501100011033/EU/PRTR. AS-L is supported by a predoctoral fellowship (N? 2021-01-PhD GRANT) from the International Olive Council. Also supported by Centro de Investigacion Biomedica en Red Fisiopatologia de la Obesidad y Nutricion, which is an initiative of the Instituto de Salud Carlos III (ISCIII) of Spain, financed by the European Regional Development Fund,?A way to make Europe?/?Investing in your future? (CB06/03) and a grant from ISCIII (PI18/01399) . H.B. is supported by a predoctoral fellowship (?Plan Propio IBIMA 2020 A.1 Contratos predoctorales,? no. Predoc20_002) . M.M.-G. is the recipient of the Nicolas Monardes Pro-gram funding from the "Servicio Andaluz de Salud, Junta de Andalucia," Spain (grants RC-0001-2018 and C-0029-2014) . 49 9 9 5 11 ACADEMIC PRESS LTD- ELSEVIER SCIENCE LTD LONDON 24-28 OVAL RD, LONDON NW1 7DX, ENGLAND 1043-6618 1096-1186 PHARMACOL RES
Pharmacol. Res. JAN 2023 187
106612 10.1016/j.phrs.2022.106612
http://dx.doi.org/10.1016/j.phrs.2022.106612 DEC 2022

8 Pharmacology & Pharmacy Science Citation Index Expanded (SCI-EXPANDED) Pharmacology & Pharmacy 7Z4DW
36528246 hybrid 2025-06-24
WOS:000915512600001
J Zaki, TA; Ziogas, A; Chang, JY; Murphy, CC; Anton-Culver, H
Zaki, Timothy A.; Ziogas, Argyrios; Chang, Jenny; Murphy, Caitlin C.; Anton-Culver, Hoda
Survival of Middle Eastern and North African Individuals Diagnosed with Colorectal Cancer: A Population-Based Study in California CANCER EPIDEMIOLOGY BIOMARKERS & PREVENTION
English Article
SOCIOECONOMIC-STATUS; RACIAL DISPARITIES; MEDITERRANEAN DIET; UNITED-STATES; MORTALITY; HEALTH; ARAB; RACE; CARE; ASSOCIATION Background: Literature on colorectal cancer outcomes in indi-viduals of Middle Eastern and North African (MENA) descent limited. To address this gap, we estimated five-year colorectal cancer-specific survival by race and ethnicity, including MENA individuals, in a diverse, population-based sample in California. Methods: We identified adults (ages 18-79 years) diagnosed with a first or only colorectal cancer in 2004 to 2017 using the California Cancer Registry (CCR), including non-Hispanic White, non-Hispanic Black, non-Hispanic Asian, Hispanic, and MENA indi-viduals. For each racial/ethnic group, we calculated five-year colo-rectal cancer-specific survival and used Cox proportional hazards regression models to examine the association of race/ethnicity and survival, adjusting for clinical and socio demographic factors. Results: Of 110,192 persons diagnosed with colorectal cancer, five-year colorectal cancer-specific survival was lowest in Black (61.0%) and highest in MENA (73.2%) individuals. Asian (72.2%) individuals had higher survival than White (70.0%) and Hispanic (68.2%) individuals. In adjusted analysis, MENA [adjusted HR (aHR), 0.82; 95% confidence interval (CI), 0.76-0.89], Asian (aHR, 0.86; 95% CI, 0.83-0.90), and Hispanic (aHR, 0.94; 95% CI, 0.91-0.97) race/ethnicity were associated with higher, and Black (aHR, 1.13; 95% CI, 1.09-1.18) race/ethnicity was associated with lower survival compared with non-Hispanic White race/ethnicity. Conclusions: To our knowledge, this is the first study to report colorectal cancer survival in MENA individuals in the United States. We observed higher survival of MENA individuals compared with other racial/ethnic groups, adjusting for sociodemographic and clinical factors. Impact: Future studies are needed to identify factors contributing to cancer outcomes in this unique population. [Zaki, Timothy A.] Univ Texas Southwestern Med Ctr, Dept Internal Med, Dallas, TX USA; [Ziogas, Argyrios; Chang, Jenny; Anton-Culver, Hoda] Univ Calif Irvine, Sch Med, Dept Med, Irvine, CA 92697 USA; [Murphy, Caitlin C.] Univ Texas Hlth Sci Ctr Houston UT Hlth Houston, Sch Publ Hlth, Houston, TX USA; [Anton-Culver, Hoda] Univ Calif Irvine, Dept Med, Irvine, CA 92697 USA University of Texas System; University of Texas Southwestern Medical Center; University of California System; University of California Irvine; University of Texas System; University of Texas Health Science Center Houston; University of Texas School Public Health; University of California System; University of California Irvine
Anton-Culver, H (corresponding author), Univ Calif Irvine, Dept Med, Irvine, CA 92697 USA. hantoncu@uci.edu Murphy, Caitlin/0000-0001-9365-0691; Ziogas, Argyrios/0000-0003-4529-3727; Zaki, Timothy/0000-0003-0299-5171 NCI at the NIH [R01CA242558];

California Department of Public Health pursuant; Centers for Disease Control and Prevention's (CDC) National Program of Cancer Registries [1NU58DP007156]; National Cancer Institute's Surveillance, Epidemiology and End Results Program [HHSN261201800032I, HHSN261201800015I, HHSN261201800009I]; National Cancer Institute [P30CA062203] Funding Source: NIH RePORTER NCI at the NIH; California Department of Public Health pursuant; Centers for Disease Control and Prevention's (CDC) National Program of Cancer Registries; National Cancer Institute's Surveillance, Epidemiology and End Results Program(United States Department of Health & Human ServicesNational Institutes of Health (NIH) - USANIH National Cancer Institute (NCI)); National Cancer Institute(United States Department of Health & Human ServicesNational Institutes of Health (NIH) - USANIH National Cancer Institute (NCI)) C. Murphy is supported by the NCI at the NIH under award number R01CA242558. The collection of cancer incidence data used in this study was supported by the California Department of Public Health pursuant to California Health and Safety Code Section 103885; Centers for Disease Control and Prevention's (CDC) National Program of Cancer Registries, under cooperative agreement 1NU58DP007156; the National Cancer Institute's Surveillance, Epidemiology and End Results Program under contract HHSN261201800032I awarded to the Univer-sity of California, San Francisco, contract HHSN261201800015I awarded to the University of Southern California, and contract HHSN261201800009I awarded to the Public Health Institute. The ideas and opinions expressed herein are those of the author (s) and do not necessarily reflect the opinions of the State of California, Department of Public Health, the National Cancer Institute, and the Centers for Disease Control and Prevention or their Contractors and Subcontractors. The publication costs of this article were defrayed in part by the payment of publication fees. Therefore, and solely to indicate this fact, this article is hereby marked "advertisement" in accordance with 18 USC section 1734. 63 1 1 0 1 AMER ASSOC CANCER RESEARCH PHILADELPHIA 615 CHESTNUT ST, 17TH FLOOR, PHILADELPHIA, PA 19106-4404 USA 1055-9965 1538-7755 CANCER EPIDEM BIOMAR Cancer Epidemiol. Biomarkers Prev. JUN 1 2023 32 6 795 801 10.1158/1055-9965.EPI-22-1326 http://dx.doi.org/10.1158/1055-9965.EPI-22-1326 7 Oncology; Public, Environmental & Occupational Health Science Citation Index Expanded (SCI-EXPANDED) Oncology; Public, Environmental & Occupational Health J2UI7 37012208 Green Accepted, Green Submitted 2025-06-24 WOS:001008208600001

J Pot, GK; Stephen, AM; Dahm, CC; Key, TJ; Cairns, BJ; Burley, VJ; Cade, JE; Greenwood, DC; Keogh, RH; Bhaniani, A; McTaggart, A; Lentjes, MAH; Mishra, G; Brunner, EJ; Khaw, KT

Pot, G. K.; Stephen, A. M.; Dahm, C. C.; Key, T. J.; Cairns, B. J.; Burley, V. J.; Cade, J. E.; Greenwood, D. C.; Keogh, R. H.; Bhaniani, A.; McTaggart, A.; Lentjes, M. A. H.; Mishra, G.; Brunner, E. J.; Khaw, K. T. Dietary patterns derived with multiple methods from food diaries and breast cancer risk in the UK Dietary Cohort Consortium EUROPEAN JOURNAL OF CLINICAL NUTRITION English Article

FAT; QUALITY; EATERS; INDEX

BACKGROUND/OBJECTIVES: In spite of several studies relating dietary patterns to breast cancer risk, evidence so far remains

inconsistent. This study aimed to investigate associations of dietary patterns derived with three different methods with breast cancer risk. SUBJECTS/METHODS: The Mediterranean Diet Score (MDS), principal components analyses (PCA) and reduced rank regression (RRR) were used to derive dietary patterns in a case-control study of 610 breast cancer cases and 1891 matched controls within four UK cohort studies. Dietary intakes were collected prospectively using 4-to 7-day food diaries and resulting food consumption data were grouped into 42 food groups. Conditional logistic regression models were used to estimate odds ratios (ORs) for associations between pattern scores and breast cancer risk adjusting for relevant covariates. A separate model was fitted for post-menopausal women only. RESULTS: The MDS was not associated with breast cancer risk (OR comparing first tertile with third 1.20 (95% CI 0.92; 1.56)), nor the first PCA-derived dietary pattern, explaining 2.7% of variation of diet and characterized by cheese, crisps and savoury snacks, legumes, nuts and seeds (OR 1.18 (95% CI 0.91; 1.53)). The first RRR-derived pattern, a ` high-alcohol' pattern, was associated with a higher risk of breast cancer (OR 1.27; 95% CI 1.00; 1.62), which was most pronounced in post-menopausal women (OR 1.46 (95% CI 1.08; 1.98)). CONCLUSIONS: A ` high-alcohol' dietary pattern derived with RRR was associated with an increased breast cancer risk; no evidence of associations of other dietary patterns with breast cancer risk was observed in this study.[Pot, G. K.] Kings Coll London, London SE1 9NH, England; [Pot, G. K.; Stephen, A. M.] MRC Human Nutr Res, Cambridge, England; [Dahm, C. C.; Keogh, R. H.; Bhaniani, A.; McTaggart, A.; Lentjes, M. A. H.; Khaw, K. T.] Univ Cambridge, Dept Publ Hlth & Primary Care, Cambridge, England; [Dahm, C. C.] Aarhus Univ, Dept Publ Hlth, Epidemiol Sect, Aarhus, Denmark; [Key, T. J.] Univ Oxford, Canc Epidemiol Unit, Oxford, England; [Burley, V. J.; Cade, J. E.] Univ Leeds, Sch Food Sci & Nutr, Nutr Epidemiol Grp, Leeds, W Yorkshire, England; [Greenwood, D. C.] Univ Leeds, Ctr Epidemiol & Biostat, Leeds, W Yorkshire, England; [Keogh, R. H.] Inst Publ Hlth, MRC, Biostat Unit, Cambridge, England; [Keogh, R. H.] London Sch Hyg & Trop Med, Dept Med Stat, London WC1, England; [Mishra, G.] MRC, Unit Lifelong Hlth & Ageing, London, England; [Brunner, E. J.] UCL, Dept Epidemiol & Publ Hlth, London WC1E 6BT, England   University of London; King's College London; UK Research & Innovation (UKRI); Medical Research Council UK (MRC); MRC Human Nutrition Research; University of Cambridge; Aarhus University; University of Oxford; University of Leeds; University of Leeds; University of Cambridge; MRC Biostatistics Unit; University of London; London School of Hygiene & Tropical Medicine; University of London; University College London   Pot, GK (corresponding author), Kings Coll London, Dept Nutr & Dietet, Franklin Wilkins Bldg,150 Stamford St, London SE1 9NH, England.   Gerda.Pot@kcl.ac.uk   Khaw, Kay-Tee/AAZ-3209-2021; Cairns, Benjamin/D-2748-2011; Greenwood, Darren/C-3220-2008; Brunner, Eric/H-2114-2011; Cade, Janet/G-4250-2016; Dahm, Christina/G-9787-2014 Burley, Victoria/0000-0003-0282-2932; Cade, Janet/0000-0003-3421-0121; Brunner, Eric/0000-0002-0595-4474; Dahm, Christina/0000-0003-0481-2893; Lentjes, Marleen/0000-0003-4713-907X; Keogh, Ruth/0000-0001-6504-3253; Cairns, Benjamin/0000-0001-7994-8213   ESRC [ES/J023299/1] Funding Source: UKRI; MRC [MC_U105960384, G0500300, G0800603, MC_U123092726, MR/K013351/1, MC_U123092725] Funding Source: UKRI; Cancer Research UK [16491, 14136] Funding Source: Medline; Medical

Research Council [MR/K013351/1, G0800603, MC_U123092726, G1000143, G0401527, G0500300, MC_U105960384, MC_U123092725] Funding Source: Medline ESRC(UK Research & Innovation (UKRI)Economic & Social Research Council (ESRC)); MRC(UK Research & Innovation (UKRI)Medical Research Council UK (MRC)); Cancer Research UK(Cancer Research UK); Medical Research Council(UK Research & Innovation (UKRI)Medical Research Council UK (MRC))
36 30 34 0 16 SPRINGERNATURE LONDON CAMPUS, 4 CRINAN ST, LONDON, N1 9XW, ENGLAND 0954-3007 1476-5640 EUR J CLIN NUTR Eur. J. Clin. Nutr. DEC 2014 68 12 1353 1358 10.1038/ejcn.2014.135 http://dx.doi.org/10.1038/ejcn.2014.135 6
Nutrition & Dietetics Science Citation Index Expanded (SCI-EXPANDED) Nutrition & Dietetics AW0DJ 25052230 Bronze, Green Accepted 2025-06-24 WOS:000345960200014
J Yiannakou, I; Singer, MR; Jacques, PF; Xanthakis, V; Ellison, RC; Moore, LL Yiannakou, Ioanna; Singer, Martha R.; Jacques, Paul F.; Xanthakis, Vanessa; Ellison, R. Curtis; Moore, Lynn L. Adherence to a Mediterranean-Style Dietary Pattern and Cancer Risk in a Prospective Cohort Study NUTRIENTS English Article
cancer; Mediterranean diet; diet patterns; cohort study; epidemiology BREAST-CANCER; COLORECTAL-CANCER; CARDIOVASCULAR RISK; METABOLIC SYNDROME; POOLED ANALYSIS; REPRODUCIBILITY; VALIDITY; IMPACT; WOMEN A Mediterranean-style diet is a healthy eating pattern that may benefit cancer risk, but evidence among Americans is scarce. We examined the prospective association between adherence to such a diet pattern and total cancer risk. A Mediterranean-style dietary pattern (MSDP) score was derived from a semi-quantitative food frequency questionnaire at exam 5 (1991-1995). Subjects included 2966 participants of the Framingham Offspring Study who were free of prevalent cancer. Cox proportional hazards regression models were used to estimate hazard ratios (HRs) and 95% confidence intervals (CIs), adjusting for demographic, lifestyle, and anthropometric measures. Cox-models were also used to examine effect modification by lifestyle and anthropometric measures. During 18 years of median follow-up, 259 women and 352 men were diagnosed with cancer. Women with moderate or higher adherence to the MSDP had & GE;25% lower risks of cancer than women with the lowest MSDP (HR (moderate vs. lowest): 0.71, 95% CI: 0.52-0.97 and HR (highest vs. lowest): 0.74; 95% CI: 0.55-0.99). The association between MSDP score and cancer risk in men was weaker except in non-smokers. Beneficial effects of the MSDP in women were stronger among those who were not overweight. In this study, higher adherence to MSDP was associated with lower cancer risk, especially among women.
[Yiannakou, Ioanna; Singer, Martha R.; Xanthakis, Vanessa; Ellison, R. Curtis; Moore, Lynn L.] Boston Univ, Sch Med, Dept Med Prevent Med & Epidemiol, Boston, MA 02118 USA; [Yiannakou, Ioanna; Moore, Lynn L.] Boston Univ, Sch Med, Grad Program Nutr, Boston, MA 02118 USA; [Yiannakou, Ioanna; Moore, Lynn L.] Boston Univ, Sch Med, Grad Program Metab, Boston, MA 02118 USA; [Jacques, Paul F.] Tufts Univ, Jean Mayer USDA Human Nutr Res Ctr Aging, Nutr Epidemiol, Boston, MA 02111 USA; [Xanthakis, Vanessa] Boston Univ, Sch Publ Hlth, Dept Biostat, Boston, MA 02118 USA Boston University; Boston University; Boston University; United States Department of Agriculture (USDA); Tufts University; Boston

University Moore, LL (corresponding author), Boston Univ, Sch Med, Dept Med Prevent Med & Epidemiol, Boston, MA 02118 USA.; Moore, LL (corresponding author), Boston Univ, Sch Med, Grad Program Nutr, Boston, MA 02118 USA.; Moore, LL (corresponding author), Boston Univ, Sch Med, Grad Program Metab, Boston, MA 02118 USA. ioannay@bu.edu; msinger@bu.edu; paul.jacques@tufts.edu; Vanessax@bu.edu; ellison@bu.edu; llmoore@bu.edu Yiannakou, Ioanna/0000-0003-4910-0013; Moore, Lynn/0000-0002-1028-919X; Jacques, Paul/0000-0001-5567-3147
National Heart, Lung, and Blood Institute [N01-HC-25195, HHSN268201500001I] National Heart, Lung, and Blood Institute(United States Department of Health & Human ServicesNational Institutes of Health (NIH) - USANIH National Heart Lung & Blood Institute (NHLBI)) These data were originally collected with funding from the National Heart, Lung, and Blood Institute (Framingham Study contract N01-HC-25195 and HHSN268201500001I). 45 13 13 0 6 MDPI
BASEL ST ALBAN-ANLAGE 66, CH-4052 BASEL, SWITZERLAND
2072-6643 NUTRIENTS Nutrients NOV 2021 13 11
4064 10.3390/nu13114064
http://dx.doi.org/10.3390/nu13114064 14
Nutrition & Dietetics Science Citation Index Expanded (SCI-EXPANDED) Nutrition & Dietetics XF2WI 34836319 gold, Green Published 2025-06-24 WOS:000723936000001
J Siddique, A; Ayoub, NM; Tajmim, A; Meyer, SA; Hill, RA; El Sayed, KA Siddique, Abu Bakar; Ayoub, Nehad M.; Tajmim, Afsana; Meyer, Sharon A.; Hill, Ronald A.; El Sayed, Khalid A. (-)-Oleocanthal Prevents Breast Cancer Locoregional Recurrence After Primary Tumor Surgical Excision and Neoadjuvant Targeted Therapy in Orthotopic Nude Mouse Models
CANCERS English Article
breast cancer; extra-virgin olive oil; HER2; lapatinib; MET; neoadjuvant; oleocanthal; surgical excision; recurrence VIRGIN OLIVE OIL; IN-VITRO; MEDITERRANEAN DIET; MET EXPRESSION; OLEOCANTHAL; RECEPTOR; GROWTH; METASTASIS; BIOMARKERS; PATTERNS Breast cancer (BC) recurrence represents a challenge for survivors who have had their primary tumors surgically excised, and/or have completed radiation, neoadjuvant, or adjuvant therapeutic regimens. Current BC treatments mostly lack the ability to reduce the risk of disease recurrence. About 70% of BC patients will subsequently suffer disease relapse, manifesting as local, regional, or distant tumor recurrence, which clearly underscores the urgent need to discover novel recurrence inhibitors. (-)-Oleocanthal (OC) is a natural phenolic, found so far exclusively in extra-virgin olive oil (EVOO). OC exerts documented bioactivities against diverse cancer types, inflammation, and neurodegenerative diseases. Herein we report the novel activity of daily oral treatment with OC (10 mg/kg) in preventing BC locoregional recurrence in a nude mouse xenograft model generated by orthotopic inoculation with BT-474 cells as a luminal type B model. We further report inhibition of tumor recurrence by OC after completion of a lapatinib neoadjuvant regimen. However, in a recurrence model of triple-negative breast cancer (TNBC), OC treatment (10 mg/kg) did not effectively prevent tumor recurrence, but rather, was seen to significantly reduce the growth of recurrent tumors as compared to vehicle control-treated animals. Inhibition of tumor recurrence was associated with significant serum level reductions of the human BC recurrence

marker CA 15-3 at the study end in animals treated with OC. OC treatment upregulated the expression of the epithelial marker E-cadherin and downregulated the levels of the mesenchymal marker vimentin in recurrent tumors vs. untreated control animals. OC treatment also reduced the activation of MET and HER2 receptors, as indicated by reduced phosphorylation levels of these proteins in recurrent tumors vs. controls. Collectively, the results of our studies provide the first evidence for suppression of BC tumor recurrence by oral OC treatment in an animal model for such recurrence, and furthermore, highlight favorable prospects for this natural product to emerge as a first-in-class BC recurrence inhibitor. [Siddique, Abu Bakar; Tajmim, Afsana; Meyer, Sharon A.; Hill, Ronald A.; El Sayed, Khalid A.] Univ Louisiana Monroe, Coll Pharm, Sch Basic Pharmaceut & Toxicol Sci, 1800 Bienville Dr, Monroe, LA 71201 USA; [Ayoub, Nehad M.] Jordan Univ Sci & Technol, Fac Pharm, Dept Clin Pharm, Irbid 22110, Jordan University of Louisiana System; University of Louisiana Monroe; Jordan University of Science & Technology El Sayed, KA (corresponding author), Univ Louisiana Monroe, Coll Pharm, Sch Basic Pharmaceut & Toxicol Sci, 1800 Bienville Dr, Monroe, LA 71201 USA. siddiqab@warhawks.ulm.edu; nmayoub@just.edu.jo; tajmima@warhawks.ulm.edu; meyer@ulm.edu; rhill@ulm.edu; elsayed@ulm.edu , Abu Bakar Siddique/W-8476-2019 Siddique, Abu Bakar/0000-0001-5424-9475; Ayoub, Nehad M./0000-0003-2284-4370; Tajmim, Afsana/0000-0003-4717-1141; El Sayed, Khalid/0000-0002-1456-4064; Meyer, Sharon/0000-0002-4720-5949; Hill, Ronald/0000-0003-4622-5996 Louisiana Board of Regents [LEQSF (2017-20)-RD-B-07]; National Cancer Institute of the National Institutes of Health [R15CA167475] Louisiana Board of Regents; National Cancer Institute of the National Institutes of Health(United States Department of Health & Human ServicesNational Institutes of Health (NIH) - USANIH National Cancer Institute (NCI)) Research reported in this publication was supported by the Louisiana Board of Regents, Award Number LEQSF (2017-20)-RD-B-07 and the National Cancer Institute of the National Institutes of Health under Award Number R15CA167475. The funders had no role in study design, data collection and analysis, decision to publish, or preparation of the study. 58 28 31 0 4 MDPI BASEL MDPI AG, Grosspeteranlage 5, CH-4052 BASEL, SWITZERLAND 2072-6694 CANCERS Cancers MAY 2019 11 5 637 10.3390/cancers11050637 http://dx.doi.org/10.3390/cancers11050637 19 Oncology Science Citation Index Expanded (SCI-EXPANDED) Oncology IF0AP 31072015 gold, Green Submitted, Green Published 2025-06-24 WOS:000472738300050
J Kryeziu, T; Bagci, U; Loshaj-Shala, A; Oral, A; Stefkov, G; Zimmer, A; Basholli-Salihu, M Kryeziu, T.; Bagci, U.; Loshaj-Shala, A.; Oral, A.; Stefkov, GJ.; Zimmer, A.; Basholli-Salihu, M. Cytotoxic activity of liposomal Thymus capitatus essential oil on HT-29 human colorectal cancer cell line PHARMAZIE English Article ANTIOXIDANT Multidrug resistance, severe side effects, and high cancer treatment costs are still well-known issues and remain an open challenge. These factors reduce the therapy's efficiency and safety, seriously affecting human health. Developing therapeutic approaches based on plant extracts, especially based on essential oils with cytotoxic and antioxidant properties, could be of efficacious strategies. This

work incorporated Thymus capitatus essential oil (TEO) in liposomes. Thymus capitatus is a plant native to the northern region of Albania and found specifically in the Mediterranean region. TEO has several biological activities and cytotoxic properties. Due to its volatility, poor solubility, and chemical instability, however, its applicability is restricted. Incorporation into liposomes enables its effective use because the exposure time to the active compounds can be extended, increasing its efficacy against colorectal cancer cell lines, as highlighted in in vitro studies. TEO demonstrated detectable cytotoxic action against HT -29 colorectal cancer cells, and this action could be enhanced by applying various formulations of TEO-loaded liposomes to this cell line. Among the tested nanosystems, TEO-Phospholipon 90H liposomes showed more significant cytotoxic effects than TEO-Lipoid S100 liposomes and TEO-Phospholipon 85G liposomes. TEO-Phospholipon 90 H liposomes also maintained its physicochemical stability for six months at 25 degrees C. This research suggests that TEO, particularly when encapsulated in TEO-Phospholipon 90 H liposomes, may offer a promising therapeutic approach. However, these findings are based on in vitro studies and further in vivo research is needed to validate the efficacy and safety of this approach in clinical settings. [Kryeziu, T.; Loshaj-Shala, A.; Basholli-Salihu, M.] Univ Prishtina, Fac Med, Dept Drug Anal & Pharmaceut Technol, Blvd & Deshmoreve, Pristina 1000, Kosovo; [Kryeziu, T.; Bagci, U.] Trakya Univ, Technol Res Dev Applicat & Res Ctr, Edirne, Turkiye; [Oral, A.] Canakkale Onsekiz Mart Univ, Fac Sci & Arts, Dept Chem, Canakkale, Turkiye; [Stefkov, GJ.] Ss Cyril & Methodius Univ, Inst Pharmacognosy, Fac Pharm, Skopje, North Macedonia; [Kryeziu, T.; Zimmer, A.] Karl Franzens Univ Graz, Inst Pharmaceut Sci, Dept Pharmaceut Technol, Graz, Austria
Universiteti i Prishtines; Trakya University; Canakkale Onsekiz Mart University; Saints Cyril & Methodius University of Skopje; University of Graz Basholli-Salihu, M (corresponding author), Univ Prishtina, Fac Med, Dept Drug Anal & Pharmaceut Technol, Blvd & Deshmoreve, Pristina 1000, Kosovo.
mimoza.basholli@uni-pr.edu Kryeziu, Toskë/AAS-2226-2021; Oral, Ayhan/J-6910-2012; Bagci, Ulas/A-4225-2012 Kryeziu, Toske/0000-0002-5591-2520 CEEPUS/OeAD mobility program [MPC-2021-02934]CEEPUS/OeAD mobility programThe authors are grateful to the CEEPUS/OeAD mobility program for providing financial support (scholarship) to Toske Kryeziu (Ref. no.: MPC-2021-02934).
57 2 2 3 6 AVOXA-MEDIENGRUPPE DEUTSCHER APOTHEKER GMBH ESCHBORN CARL-MANNICH-STR 26, ESCHBORN, GERMANY 0031-7144 PHARMAZIE Pharmazie
MAY 2024 79 3-5 49 56
10.1691/ph.2024.3037 http://dx.doi.org/10.1691/ph.2024.3037
8 Chemistry, Medicinal; Chemistry, Multidisciplinary; Pharmacology & Pharmacy Science Citation Index Expanded (SCI-EXPANDED) Pharmacology & Pharmacy; Chemistry WU3W4 38872271 2025-06-24
WOS:001257358400002
J Legrand, C; Duchateau, L; Sylvester, R; Janssen, P; van der Hage, JA; van de Velde, CJH; Therasse, P
Legrand, C; Duchateau, L; Sylvester, R; Janssen, P; van der Hage, JA; van de Velde, CJH; Therasse, P
Heterogeneity in disease free survival between centers: lessons learned from an EORTC breast cancer trial CLINICAL TRIALS
English Article

EUROPEAN ORGANIZATION; PERIOPERATIVE CHEMOTHERAPY; FRAILTY Background: Large phase III clinical trials convey a lot of important information besides the main analysis of the treatment effect. For example, the use of multicenter clinical trial data to identify prognostic indices is now common. In addition, the study of heterogeneity in patient outcome between centers has received considerable attention in recent years. In this paper, we explain and illustrate a method used to investigate such heterogeneity with data from an early breast cancer clinical trial. Methods: The inclusion of a random effect for center in a Cox proportional hazards model allows us to study the heterogeneity in time-to-event outcomes between centers. Such a model has the major advantage that it provides a measure of the spread of outcomes over centers. This technique is illustrated using data from EORTC trial 10854, a randomized phase III trial comparing perioperative chemotherapy with no perioperative chemotherapy for early breast cancer; 2793 patients were entered by 14 centers. Results: Substantial heterogeneity between centers was detected for disease-free survival. This can be explained by the geographical area in which the center is located, with better outcomes achieved in France as compared with southern Europe and South Africa. None of the prognostic factors considered could explain this heterogeneity. Conclusion: Although clinical trials are run with the objective of removing as much heterogeneity as possible, some heterogeneity in the outcome of patients between centers may remain, as was the case in our study. The use of a random effect for center within a Cox PH model is an excellent method to investigate this heterogeneity. Such types of analyses, although exploratory, provide further insight into possible factors which may have an impact on the patient's outcome.

EORTC Data Ctr, B-1200 Brussels, Belgium; Univ Ghent, Fac Vet Med, Dept Physiol Biochem & Biometr, B-9820 Merelbeke, Belgium; Hasselt Univ, Ctr Stat, B-3590 Diepenbeek, Belgium; Leiden Univ, Med Ctr, NL-2300 RC Leiden, Netherlands European Organisation for Research & Treatment of Cancer; Ghent University; Hasselt University; Leiden University - Excl LUMC; Leiden University; Leiden University Medical Center (LUMC) EORTC Data Ctr, Ave E Mounier 83,Bte 11, B-1200 Brussels, Belgium.

catherine.legrand@eortc.be Janssen, Paul/V-8575-2019; van de Velde, Cornelis/AAY-8360-2020; van der Hage, Jos/AAG-3541-2021

JANSSEN, PAUL/0000-0002-7308-3455; van der Hage, Jos/0000-0001-9040-1323 NCI NIH HHS [5U10CA11488-33] Funding Source: Medline NCI NIH HHS(United States Department of Health & Human ServicesNational Institutes of Health (NIH) - USANIH National Cancer Institute (NCI)) 18 10 10 0 4

SAGE PUBLICATIONS LTD LONDON 1 OLIVERS YARD, 55 CITY ROAD, LONDON EC1Y 1SP, ENGLAND 1740-7745 1740-7753 CLIN TRIALS Clin. Trials 2006 3 1

10 18 10.1191/1740774506cn132oa

http://dx.doi.org/10.1191/1740774506cn132oa 9

Medicine, Research & Experimental Science Citation Index Expanded (SCI-EXPANDED) Research & Experimental Medicine

017XJ 16539086 2025-06-24

WOS:000235726300002

J Péridy, N Peridy, Nicolas A GENERALIZED MODEL OF INTERNATIONAL MIGRATION DETERMINANTS. APPLICATION TO EUROPEAN IMMIGRATION FROM SOUTHERN MEDITERRANEAN COUNTRIES REVUE ECONOMIQUE French Article

POLITICAL-ECONOMY; TRADE; RETURNS; SKILL Based on new developments in migration theory, this paper develops a model which includes both traditional and new migration determinants. An empirical application is implemented for European immigration from Southern Mediterranean Countries (SMCS). Results show that migrations are not only explained by differences in income levels. They also depend on a set of many other variables, such as income inequality, differences in unemployment and costs of living, the education level of the migrants, human networks, differences in languages, border effects, migration policy as well as non economic determinants, including climate, environment as well as religion.
[Peridy, Nicolas] Univ Sud Toulon Var, LEAD, UFR Sci Econ, Ave Univ,BP 20132, F-83957 La Garde, France Péridy, N (corresponding author), Univ Sud Toulon Var, LEAD, UFR Sci Econ, Ave Univ,BP 20132, F-83957 La Garde, France. nicolas.peridy@univ-tln.fr 58 2 2 0
2 PRESSES FOND NAT SCI POLIT PARIS 07 27 RUE SAINT-GUILLAUME, 75341 PARIS 07, FRANCE 0035-2764 1950-6694 REV ECON-FR Rev. Econ. NOV 2010 61 6
981 1010 10.3917/reco.616.0981
http://dx.doi.org/10.3917/reco.616.0981 30
Economics Emerging Sources Citation Index (ESCI) Business & Economics VH6WE 2025-06-24
WOS:000453966300002
J El Kinany, K; Huybrechts, I; Kampman, E; Boudouaya, HA; Hatime, Z; Deoula, MMS; El Asri, A; Benslimane, A; Nejjari, C; Ibrahimi, SA; Mrabti, H; Abda, N; Alaoui, R; Gunter, MJ; El Rhazi, K El Kinany, Khaoula; Huybrechts, Inge; Kampman, Ellen; Boudouaya, Hanae Abir; Hatime, Zineb; Deoula, Meimouna Mint Sidi; El Asri, Achraf; Benslimane, Abdelilah; Nejjari, Chakib; Ibrahimi, Sidi Adil; Mrabti, Hind; Abda, Naima; Alaoui, Rhimou; Gunter, Marc J.; El Rhazi, Karima
Concordance with the World Cancer Research Fund/American Institute for Cancer Research recommendations for cancer prevention and colorectal cancer risk in Morocco: A large, population-based case-control study INTERNATIONAL JOURNAL OF CANCER English Article
WCRF; AICR recommendations; colorectal cancer; case-control study; FFQ; Morocco PHYSICAL-ACTIVITY; NUTRITION TRANSITION; RESEARCH GUIDELINES; MEDITERRANEAN DIET; ADHERENCE; MORTALITY; PATTERNS; OBESITYThe present study aimed to investigate associations between adherence to the recommendations on cancer prevention from the WCRF/AICR and colorectal cancer (CRC) risk in Morocco. Incident CRC cases (n = 1,516) and controls (n = 1,516) matched on age, sex and center, were recruited between September 2009 and February 2017 at five major hospitals located in Morocco. In-person interviews were conducted to assess habitual diet using a validated Food Frequency Questionnaire, physical activity and anthropometric measurements. Adherence to the WCRF/AIRC Recommendations was ranged from 0 (no adherence) to 6 (maximal adherence) and incorporating six WCRF/AICR components (food groups, physical activity and BMI). Multivariable odd ratios (ORA) and 95% confidence intervals (CI) were calculated using conditional multivariate logistic regression models, with low adherence as referent, adjusting for potential confounding factors. Compared to those with the lowest adherence score, individuals in the highest WCRF/AICR score category had a

statistically significant reduced risk for colon cancer (ORA = 0.63, 95% CI 0.53-0.76); rectal cancer (ORA = 0.52, 95% CI 0.43-0.63) and CRC overall (ORA = 0.58, 95% CI 0.51-0.66). For individual score components, when comparing the lowest with the highest adherence category, CRC risk was significantly lower in the highest adherence category for body fatness (ORA = 0.73; 95% CI 0.62-0.85), physical activity (ORA = 0.70; 95% CI 0.60-0.82), plant foods (ORA = 0.50; 95% CI 0.39-0.63) and red/processed meat (ORA = 0.81; 95% CI 0.71-0.92). Our analysis indicated that greater adherence to the WCRF/AICR recommendations for cancer prevention may lower CRC risk in Morocco. [El Kinany, Khaoula; Boudouaya, Hanae Abir; Hatime, Zineb; Deoula, Meimouna Mint Sidi; El Asri, Achraf; Benslimane, Abdelilah; Nejjari, Chakib; El Rhazi, Karima] Sidi Mohamed Ben Abdellah Univ, Fac Med & Pharm Fez, Dept Epidemiol & Publ Hlth, Km 2 200 Sidi Harazem Rd 1893, Fes, Morocco; [Huybrechts, Inge; Gunter, Marc J.] WHO, Int Agcy Res Canc, Sect Nutr & Metab, Lyon, France; [Kampman, Ellen] Wageningen Univ, Div Human Nutr, Wageningen, Netherlands; [Ibrahimi, Sidi Adil] Sidi Mohamed Ben Abdellah Univ, Hassan II Univ Hosp Ctr, Fac Med & Pharm, Dept Hepatogastroenterol,Team Dis Digest Syst, Fes, Morocco; [Mrabti, Hind] Natl Inst Oncol, Dept Med Oncol, Rabat, Morocco; [Abda, Naima] Mohammed First Univ, Dept Epidemiol Clin Res & Publ Hlth, Oujda, Morocco; [Alaoui, Rhimou] Ibn Rochd Univ Hosp Ctr, Dept Med B, Casablanca, Morocco   Sidi Mohamed Ben Abdellah University of Fez; World Health Organization; International Agency for Research on Cancer (IARC); Wageningen University & Research; Sidi Mohamed Ben Abdellah University of Fez; Hassan II University Hospital Center of Fez; Mohammed V University in Rabat; Ibn sina University Hospital Center of Rabat; Mohammed First University of Oujda; Hassan II University of Casablanca; Ibn Rochd University Hospital Center of Casablanca

El Kinany, K (corresponding author), Sidi Mohamed Ben Abdellah Univ, Fac Med & Pharm Fez, Dept Epidemiol & Publ Hlth, Km 2 200 Sidi Harazem Rd 1893, Fes, Morocco.

elkinanykhaoula@hotmail.fr Gunter, Marc/AAP-8621-2020; Huybrechts, Inge/ITT-7052-2023; nejjari, chakib/KBB-6065-2024; Benslimane, Abdelilah/AAC-6576-2019; EL RHAZI, Karima/ABY-6004-2022 BENSLIMANE, ABDELILAH/0000-0002-5923-4843; Gunter, Marc/0000-0001-5472-6761; Nejjari, Chakib/0000-0001-9610-8507; El Asri, Achraf/0000-0003-0458-9847; EL RHAZI, Karima/0000-0002-8135-9044; EL KINANY, khaoula/0000-0003-2861-7224; Hatime, Zineb/0000-0002-4823-2199; Abda, Naima/0000-0002-6702-4977 Moroccan Society of Diseases of the Digestive System (SMMAD); Lalla Salma Foundation, Prevention and Treatment of Cancers (FLSC) [06/AP2013]

Moroccan Society of Diseases of the Digestive System (SMMAD); Lalla Salma Foundation, Prevention and Treatment of Cancers (FLSC)   Grant sponsor: Moroccan Society of Diseases of the Digestive System (SMMAD); Grant sponsor: Lalla Salma Foundation, Prevention and Treatment of Cancers (FLSC); Grant number: No 06/AP2013   48   25   25   0   20   WILEY

HOBOKEN   111 RIVER ST, HOBOKEN 07030-5774, NJ USA

0020-7136 1097-0215   INT J CANCER   Int. J. Cancer

OCT 1 2019 145   7   1829 1837

10.1002/ijc.32263   http://dx.doi.org/10.1002/ijc.32263

9   Oncology   Science Citation Index Expanded (SCI-EXPANDED)   Oncology   IO4AB 30861106   Bronze

2025-06-24 WOS:000479320800012

J Gil, A; Ortega, RM; Maldonado, J Gil, Angel; Ortega, Rosa M.; Maldonado, Jose Wholegrain cereals and bread: a duet of the Mediterranean diet for the prevention of chronic diseases PUBLIC HEALTH NUTRITION English Article

Bread; Cereals; Chronic disease; Grains; Diet; Mediterranean

CORONARY-HEART-DISEASE; GRAIN FOOD-INTAKE; GLYCEMIC LOAD; CARDIOVASCULAR-DISEASE; RISK-FACTORS; POSTMENOPAUSAL WOMEN; DIABETES-MELLITUS; COLORECTAL-CANCER; FIBER INTAKE; FINNISH MEN

Objective: The promotion of healthy lifestyles is one of the major goals of governments and international agencies all over the world. Wholegrain cereals are rich in nutrients and many phytochemical compounds, with recognised benefits for health, including dietary fibre, a number of phenolic compounds, lignans, vitamins and minerals and other bioactive components. The aim of the present work is to review the fundamental studies that support the consumption of wholegrain cereals and bread to prevent chronic diseases. Design: Descriptive review considering human studies. Setting and subjects: Subjects included in randomised intervention trials and cohort studies from different countries published up to 2010. Results: Several studies show consistently that subjects who ingest three or more portions of foods per day based on wholegrain cereals have a 20-30% lower risk of CVD than subjects who ingest low quantities of cereals. This level of protection is not observed with the ingestion of refined cereals, these being even higher than with the intake of fruit and vegetables. Likewise, high intake of wholegrain cereals and their products, such as whole-wheat bread, is associated with a 20-30% reduction in the risk of type 2 diabetes. Finally, protection against the risk of colorectal cancer and polyps, other cancers of the digestive tract, cancers related to hormones and pancreatic cancer has been associated with the regular consumption of wholegrain cereals and derived products. Conclusions: The regular intake of wholegrain cereals can contribute to reduction of risk factors related to non-communicable chronic diseases. [Gil, Angel] Univ Granada, Inst Nutr & Food Technol, Ctr Biomed Res, Dept Biochem & Mol Biol 2, Granada 18100, Spain; [Ortega, Rosa M.] Univ Complutense Madrid, Sch Pharm, Dept Nutr, E-28040 Madrid, Spain; [Maldonado, Jose] Univ Granada, Sch Med, Dept Paediat, E-18071 Granada, Spain

University of Granada; Complutense University of Madrid; University of Granada Gil, A (corresponding author), Univ Granada, Inst Nutr & Food Technol, Ctr Biomed Res, Dept Biochem & Mol Biol 2, Avda Conocimiento S-N, Granada 18100, Spain.

agil@ugr.es Ortega, Rosa/S-5760-2016; Gil, Angel/L-2275-2014

ORTEGA ANTA, ROSA MARIA/0000-0003-3837-9450; Gil, Angel/0000-0001-7663-0939 Instituto de Salud Carlos III del Ministerio de Ciencia e Innovacion [RD08/0072] Instituto de Salud Carlos III del Ministerio de Ciencia e Innovacion(Instituto de Salud Carlos III) A.G. and J.M. are funded in part by the Instituto de Salud Carlos III del Ministerio de Ciencia e Innovacion. Red SAMID RETIC n. RD08/0072. The authors have no conflict of interest to declare related to the topic and content of the article. The authors thank the Mediterranean Diet Foundation for its valuable comments and suggestions. 60

107 120 1 60 CAMBRIDGE UNIV PRESS CAMBRIDGE

EDINBURGH BLDG, SHAFTESBURY RD, CB2 8RU CAMBRIDGE, ENGLAND

1368-9800 1475-2727 PUBLIC HEALTH NUTR Public Health Nutr. DEC 2011 14 12A SI 2316

2322 10.1017/S1368980011002576
http://dx.doi.org/10.1017/S1368980011002576 7
Public, Environmental & Occupational Health; Nutrition & Dietetics Science Citation Index Expanded (SCI-EXPANDED)
Public, Environmental & Occupational Health; Nutrition & Dietetics 871IF 22166190 Bronze 2025-06-24
WOS:000298730800008
J Calahorra, J; Martínez-Lara, E; De Dios, C; Siles, E
Calahorra, Jesus; Martinez-Lara, Esther; De Dios, Cristina; Siles, Eva Hypoxia modulates the antioxidant effect of hydroxytyrosol in MCF-7 breast cancer cells
PLOS ONE English Article
OLIVE OIL PHENOLICS; OXIDATIVE STRESS; THERAPEUTIC TARGET; HEME OXYGENASE-1; RISK; INVOLVEMENT; INDUCTION; PATHWAYS; PROTECTS; MUSCLE Although cancer is multifactorial, a strong correlation between this pathology and increased oxidative stress has long been stablished. Hypoxia, inherent to solid tumors, increases reactive oxygen species and should be taken into account when analyzing the response of tumor cells to antioxidants. The Mediterranean diet has been related to a lower incidence of cancer, and particularly of breast cancer. Given that hydroxytyrosol (HT) is largely responsible for the antioxidant properties of olive oil, we have performed a comprehensive and comparative study of its effect on the oxidative stress response of the human breast cancer cell line MCF-7 in hypoxia and normoxia. Our results demonstrate that the antioxidant action of HT is particularly effective in a hypoxic environment. Moreover, we have observed that this polyphenol modulates the transcription and translation of members of the PGC-1 alpha/ERR alpha and PGC-1 alpha/Nrf2 pathways. However, while the transcriptional effects of HT are similar in normoxic and hypoxic conditions, its translational action is less prominent and partially attenuated in hypoxia, and therefore cannot completely explain the antioxidant effect of HT. Consequently, our results underscore that the hypoxic environment of tumor cells should be considered when analyzing the effect of bioactive compounds. Besides, this study also points to the importance of assessing the regulatory role of HT at both mRNA and protein level to get a complete picture of its effects. [Calahorra, Jesus; Martinez-Lara, Esther; De Dios, Cristina; Siles, Eva] Univ Jaen, Dept Expt Biol, Campus Las Lagunillas S-N, Jaen, Spain; [De Dios, Cristina] Higher Council Sci Res IIBB CSIC, Biomed Res Inst Barcelona, Dept Cell Death & Proliferat, Barcelona, Spain Universidad de Jaen; Consejo Superior de Investigaciones Cientificas (CSIC); CSIC - Instituto de Investigaciones Biomedicas de Barcelona (IIBB); Barcelona Institute of Science & Technology; Institute for Research in Biomedicine - IRB Barcelona Siles, E (corresponding author), Univ Jaen, Dept Expt Biol, Campus Las Lagunillas S-N, Jaen, Spain. esiles@ujaen.es Calahorra, Jesus/NJS-6120-2025; Martínez-Lara, Esther/ABG-3428-2020; de Dios, Cristina/AAA-7602-2022 de Dios, Cristina/0000-0003-4512-7817; Calahorra, Jesus/0000-0002-6399-751X; Siles Rivas, Eva/0000-0003-4192-7008; Martinez Lara, Esther del Pilar/0000-0002-4962-3634
48 23 23 0 7 PUBLIC LIBRARY SCIENCE SAN FRANCISCO 1160 BATTERY STREET, STE 100, SAN FRANCISCO, CA 94111 USA 1932-6203 PLOS ONE PLoS One SEP 20 2018 13 9
e0203892 10.1371/journal.pone.0203892

http://dx.doi.org/10.1371/journal.pone.0203892 16 Multidisciplinary Sciences Science Citation Index Expanded (SCI-EXPANDED) Science & Technology - Other Topics GU8XH 30235254 Green Published, gold, Green Submitted 2025-06-24 WOS:000445626400039

J Farràs, M; Almanza-Aguilera, E; Hernáez, A; Agustí, N; Julve, J; Fitó, M; Castañer, O Farras, Marta; Almanza-Aguilera, Enrique; Hernaez, Alvaro; Agusti, Nuria; Julve, Josep; Fito, Montserrat; Castaner, Olga Beneficial effects of olive oil and Mediterranean diet on cancer physio-pathology and incidence SEMINARS IN CANCER BIOLOGY English Article Cancer; Fatty acids; Mediterranean diet; Olive oil; Phenolic compounds GAMMA-LINOLENIC ACID; ESTROGEN-RECEPTOR EXPRESSION; MONOUNSATURATED FATTY-ACID; ACTIVATED PROTEIN-KINASE; CELL-CYCLE ARREST; BREAST-CANCER; OLEIC-ACID; PHENOLIC-COMPOUNDS; COLORECTAL-CANCER; LUNG-CANCER Virgin olive oil is a characteristic component and the main source of fat of the Mediterranean diet. It is a mix of high-value health compounds, including monounsaturated fatty acids (mainly oleic acid), simple phenols (such as hydroxytyrosol and tyrosol), secoiridoids (such as oleuropein, oleocanthal), flavonoids, and terpenoids (such as squalene). Olive oil consumption has been shown to improve different aspects of human health and has been associated with a lower risk of cancer. However, the underlying cellular mechanisms involved in such effects are still poorly defined, but seem to be related to a promotion of apoptosis, modulation of epigenetic patterns, blockade of cell cycle, and angiogenesis regulation. The aim of this review is to update the current associations of cancer risk with the Mediterranean diet, olive oil consumption and its main components. In addition, the identification of key olive oil components involved in anticarcinogenic mechanisms and pathways according to experimental models is also addressed. [Farras, Marta; Julve, Josep] Inst Recerca Hosp Santa Creu & St Pau, Inst Invest Biomed IIB St Pau, C St Quinti 77, Barcelona 08041, Spain; [Farras, Marta; Julve, Josep] Inst Salud Carlos III, CIBER Diabet & Enfermedades Metab Asociadas CIBER, Madrid 28029, Spain; [Almanza-Aguilera, Enrique; Fito, Montserrat; Castaner, Olga] Hosp Del Mar Res Inst IMIM, Cardiovasc Risk & Nutr Res Grp CARIN, Barcelona 08003, Spain; [Almanza-Aguilera, Enrique] Inst Salud Carlos III, Ctr Invest Biomed Red Fragilidad & Envejecimiento, Madrid 28029, Spain; [Almanza-Aguilera, Enrique] Univ Barcelona, Inst Nutr & Food Safety INSA UB, Santa Coloma De Gramenet 08921, Spain; [Hernaez, Alvaro; Fito, Montserrat; Castaner, Olga] Inst Salud Carlos III, Consorcio CIBER, MP Fisiopatol Obesidad & Nutr CIBEROBN, Madrid 28029, Spain; [Hernaez, Alvaro; Agusti, Nuria] August Pi i Sunyer Biomed Res Inst IDIBAPS, Barcelona 08036, Spain; [Agusti, Nuria] Univ Barcelona, Inst Clin Gynecol Obstet & Neonatol, Hosp Clin, Barcelona 08036, Spain Hospital of Santa Creu i Sant Pau; Instituto de Salud Carlos III; CIBER - Centro de Investigacion Biomedica en Red; CIBERES; Hospital del Mar Research Institute; Hospital del Mar; CIBER - Centro de Investigacion Biomedica en Red; CIBERFES; Instituto de Salud Carlos III; University of Barcelona; Instituto de Salud Carlos III; University of Barcelona; Hospital Clinic de Barcelona; IDIBAPS; University of Barcelona; Hospital Clinic de Barcelona Farràs, M (corresponding author), Inst Recerca Hosp Santa Creu & St Pau, Inst Invest Biomed IIB St Pau, C St Quinti 77, Barcelona 08041, Spain.; Castañer, O

(corresponding author), Hosp del Mar Res Inst IMIM, PRBB, Cardiovasc Risk & Nutr Res Grp CARIN, C Doctor Aiguader 88, Barcelona 08003, Spain. mfarras@santpau.cat; ealmanzaa@outlook.com; alvaro.hernaez1@gmail.com; nagusti@clinic.cat; jjulve@santpau.cat; mfito@imim.es; ocastaner@imim.es Castaner, Olga/F-1533-2013; Farràs, Marta/AAA-9418-2021; Almanza Aguilera, Enrique/AAA-2853-2020; Hernaez, Alvaro/GXF-3337-2022; Fito Colomer, Montse/C-1822-2012; Julve, Josep/I-1003-2017 Castaner Nino, Olga/0000-0003-3169-997X; Hernaez, Alvaro/0000-0001-8593-1477; Almanza Aguilera, Enrique/0000-0002-4805-0774; Fito Colomer, Montse/0000-0002-1817-483X; Julve, Josep/0000-0002-6531-2246; Agusti, Nuria/0000-0002-1165-0661 Instituto de Salud Carlos III; FEDER "Una manera de hacer Europa" (Spain) [PI18/00020, PI17/00214, PI1700232, CD17/00233, CD17/00122, JR17/00022, CPII18/00004]; CONACYT (Mexico) postdoctoral fellowship [2018-000022-01EXTV-00459]; Agencia de Gestio d'Ajuts Universitaris i de Recerca (Spain) [2017 SGR 222]; CIBER of the Physiopathology of Obesity and Nutrition (CIBEROBN) (Spain); CIBER de Diabetes y Enfermedades Metabolicas Asociadas (CIBERDEM) (Spain); CIBER de Red Fragilidad y Envejecimiento Saludable (CIBERFES) (Spain); Red de Investigacion en "Enfermedades Metabolicas y Cancer" [RED2018-102799-T]; Ministerio de Economia y Competitividad (MINECO) (Spain) Instituto de Salud Carlos III(Instituto de Salud Carlos IIISpanish Government); FEDER "Una manera de hacer Europa" (Spain); CONACYT (Mexico) postdoctoral fellowship; Agencia de Gestio d'Ajuts Universitaris i de Recerca (Spain); CIBER of the Physiopathology of Obesity and Nutrition (CIBEROBN) (Spain); CIBER de Diabetes y Enfermedades Metabolicas Asociadas (CIBERDEM) (Spain); CIBER de Red Fragilidad y Envejecimiento Saludable (CIBERFES) (Spain); Red de Investigacion en "Enfermedades Metabolicas y Cancer"; Ministerio de Economia y Competitividad (MINECO) (Spain)(Spanish Government) All sources of funding should also be acknowledged and you should declare any involvement of study sponsors in the study design; collection, analysis and interpretation of data; the writing of the manuscript; the decision to submit the manuscript for publication. If the study sponsors had no such involvement, this should be stated.This work was funded by the Instituto de Salud Carlos III and FEDER "Una manera de hacer Europa" (Spain) , grant numbers: PI18/00020 (to M.F.-IMIM) ,PI17/00214 (to O.C.) PI1700232 (to J.J.) , Sara Borrell contracts CD17/00233 (to M.F.-St.Pau) and CD17/00122 (to A.H.) , Joan Rodes contract (JR17/00022) (to O.C.) , and Miguel Servet Type 2 contract (CPII18/00004) (to J.J.) . CONACYT (Mexico) postdoctoral fellowship (2018-000022-01EXTV-00459) and Age`ncia de Gestio d'Ajuts Universitaris i de Recerca (2017 SGR 222) (Spain) . CIBER of the Physiopathology of Obesity and Nutrition (CIBEROBN) (Spain) , CIBER de Diabetes y Enfermedades Metabolicas Asociadas (CIBERDEM) (Spain) , and CIBER de Red Fragilidad y Envejecimiento Saludable (CIBERFES) (Spain) . Red de Investigacion en "Enfermedades Metabolicas y Cancer" (RED2018102799T) , Ministerio de Economia y Competitividad (MINECO) (Spain) . 190 39 42 4 36 ACADEMIC PRESS LTD- ELSEVIER SCIENCE LTD LONDON 24-28 OVAL RD, LONDON NW1 7DX, ENGLAND 1044-579X 1096-3650 SEMIN CANCER BIOL Semin. Cancer Biol. AUG 2021 73 SI 178 195 10.1016/j.semcancer.2020.11.011 http://dx.doi.org/10.1016/j.semcancer.2020.11.011 JUN

2021 18 Oncology Science Citation Index Expanded (SCI-EXPANDED) Oncology SR7TI 33249203 Green Accepted
2025-06-24 WOS:000661248600014
J Elkharashy, MS; Mohamed, NGR; Hanafi, NF; Orief, YI; El Sabaa, BM Elkharashy, Mona; Mohamed, Nagwa; Hanafi, Nesrine; Orief, Yasser; El Sabaa, Bassma
Prevalence of high risk human papillomavirus types 16/18 in cytologically abnormal cervical smears in Alexandria, Egypt. A cytological molecular study MIDDLE EAST FERTILITY SOCIETY JOURNAL
English Article
Human papillomavirus; Polymerase chain reaction; High-risk human papilloma-virus 16 and 18; Cervical cancer; Cervical smear
Introduction: In Egypt, cervical cancer ranks as the second most frequent cancer after breast cancer, among women between 15 and 44 years of age. High-risk human papillomavirus (HPV) 16 and 18 detection holds the potential to be used as a tool to detect women, at risk for consequent development of cervical cancer because of their predominance and potentially greater oncogenic nature than other high risk HPV subtypes. Objective: To determine the prevalence of high-risk HPV 16/18 DNA in women with abnormal cervical cytology. Subjects and methods: 45 cases were collected from Egyptian women seeking routine gynecologic care. Ten cytologically normal cervical smear cell samples were included in the study as a control to be tested for the presence of HPV 16/18 DNA and were collected from asymptomatic patients having cystorectocele or coming for loop insertion or removal. The 45 specimens were subjected to real-time polymerase chain reaction, using multiplex HPV 16 and 18 PCR kit. Results: 45 cervical smears were collected in the present study. Cytopathological examination revealed that 5 (11.1%) were ASCUS, 8 (17.8) were LSIL, 5 (11.1%) were HSIL, 1 (2.2%) was squamous cell carcinoma (SCC), 1 (2.2%) was adenocarcinoma and 25 (55.6%) were benign (inflammatory). 20 patients with abnormal cervical cytology and 10 controls were included in the present study. In patients with abnormal cervical cytology, 5 (25%) were ASCUS, 8 (40%) were LSIL, 5 (25%) were HSIL, and 1 (5%) was SCC and 1 (5%) was adenocarcinoma. Statistical analysis revealed a significant difference between patient and control groups as regards regularity of menstruation where irregular menstruation and higher prevalence of menopausal women, abnormal vaginal bleeding, menorrhagia, vaginal infection, and abnormal cervical appearance were encountered in patients. A statistically significant higher prevalence of married women was found in the control group. There was no significant difference in the distribution of patients and control as regards HPV 16 or HPV 18 in which 20% of patients were HPV 16 positive and 10% of patients were HPV 18 positive compared with none in the control group. 6 were positive either for HPV 16 or 18, while 39 were negative. The HPV 16/18 positive patients had significantly higher age and marital duration when compared with HPV 16/18 negative group. Significantly, most of the HPV 16/18 positive patients were menopause. A significantly higher prevalence of women with cervicitis, contraceptive users and married women was in the HPV 16/18 negative group. Conclusion: The study generates epidemiological data of prevalence of HPV 16/18 in cytologically abnormal cervical smears in women seeking routine gynecologic care at the outpatient clinics of the Obstetrics and Gynecology Department at El Shatby University. High-risk HPV DNA testing by PCR of cervical samples diagnosed according to the Bethesda 2001

guidelines may benefit the management of patients with abnormal cervical smears, especially among women aged 46 years and older, in menopausal women and in women complaining of PMB. Therefore, HPV DNA testing should be made use of as an adjunct to cervical smears. (C) 2013 Production and hosting by Elsevier B.V. on behalf of Middle East Fertility Society. [Elkharashy, Mona; Mohamed, Nagwa; Hanafi, Nesrine] Univ Alexandria, Fac Med, Dept Med Microbiol & Immunol, Alexandria, Egypt; [Orief, Yasser] Univ Alexandria, Fac Med, Dept Obstet & Gynecol, Alexandria, Egypt; [El Sabaa, Bassma] Univ Alexandria, Fac Med, Dept Pathol, Alexandria, Egypt Egyptian Knowledge Bank (EKB); Alexandria University; Egyptian Knowledge Bank (EKB); Alexandria University; Egyptian Knowledge Bank (EKB); Alexandria University Orief, YI (corresponding author), Univ Alexandria, Fac Med, Dept Obstet & Gynecol, Alexandria, Egypt. yaserorief@yahoo.com orief, yasser/0000-0001-7715-1948 82 2 2 0 1 ELSEVIER SCIENCE BV AMSTERDAM PO BOX 211, 1000 AE AMSTERDAM, NETHERLANDS 1110-5690 MIDDLE EAST FERTIL S Middle East Fertil. Soc. J. DEC 2013 18 4 253 267 10.1016/j.mefs.2013.01.002 http://dx.doi.org/10.1016/j.mefs.2013.01.002 15 Reproductive Biology Emerging Sources Citation Index (ESCI) Reproductive Biology V57AU gold 2025-06-24 WOS:000210601500006

J Acheampong, AO; Opoku, EEO Acheampong, Alex O.; Opoku, Eric Evans Osei Democracy, Human Capital, and Foreign Direct Investment: Evidence From Developing Economies ECONOMICS & POLITICS English Article; Early Access democracy; developing economies; foreign direct investment; human capital; location theory; multinational companies DEVELOPING-COUNTRIES; NATURAL-RESOURCES; INSTITUTIONS; INFLOWS; GROWTH; FDI; DETERMINANTS; GOVERNANCE; REVERSAL; ORIGINS This paper examines the role of human capital in democracy and foreign direct investment (FDI) relationship in 129 developing countries from 1980 to 2022. Democracy is viewed as a multidimensional concept with five measures: electoral, liberal, egalitarian, participatory, and participation democracy. Employing the dynamic generalized method of moments, the findings demonstrate that democracy indices and human capital have direct, positive, and significant impact on FDI. Additionally, we observe that democracy indices increase FDI inflows when the human capital index is above a certain threshold, while democracy variables decrease FDI inflows below this threshold. The paper also reveals that democracy reduces FDI inflows to low-income economies, sub-Saharan Africa, and Middle East and North Africa countries. However, it increases FDI inflows to middle-income economies and other developing regions. These findings are robust to alternative econometric techniques and model specifications. The findings underscore the importance of enhancing democratization and human capital development to attract FDI to developing economies.[Acheampong, Alex O.] Bond Univ, Bond Business Sch, Gold Coast, Australia; [Acheampong, Alex O.] Bond Univ, Ctr Data Analyt, Gold Coast, Australia; [Opoku, Eric Evans Osei] Univ Nottingham Ningbo China, Nottingham Univ, Business Sch China, Ningbo, Peoples R China Bond University; Bond University; University of Nottingham Ningbo China Acheampong, AO (corresponding author), Bond Univ, Bond Business Sch, Gold Coast,

Australia.; Acheampong, AO (corresponding author), Bond Univ, Ctr Data Analyt, Gold Coast, Australia. aacheamp@bond.edu.au
No Statement Available 65 0 0
0 0 WILEY PERIODICALS, INC SAN FRANCISCO ONE MONTGOMERY ST, SUITE 1200, SAN FRANCISCO, CA 94104 USA 0954-1985
1468-0343 ECON POLIT-OXFORD Econ. Polit. 2025 JUN 19 2025
10.1111/ecpo.70004 http://dx.doi.org/10.1111/ecpo.70004
JUN 2025 22 Economics; Political ScienceSocial Science Citation Index (SSCI) Business & Economics; Government & Law 3YE1K 2025-06-24 WOS:001511813200001
J Kars, MD; Iseri, ÖD; Ural, AU; Avcu, F; Beyzadeoglu, M; Dirican, B; Gündüz, U Kars, Meltem Demirel; Iseri, Ozlem Darcansoy; Ural, Ali Ugur; Avcu, Ferit; Beyzadeoglu, Murat; Dirican, Bahar; Gunduz, Ufuk Development of radioresistance in drug resistant human MCF-7 breast cancer cells
JOURNAL OF RADIOTHERAPY IN PRACTICE English
Article MDR1; MCF-7; radioresistance; Real-time PCR Background and purpose: Radiotherapy is used for the treatment of malignant tumours, and may be used as the primary therapy. It is also common to combine radiotherapy with surgery, chemotherapy, hormone therapy or some combination of them. Even if the tumour is treated intensively, women diagnosed with breast cancer may develop a recurrence. Most recurrences may be in the form of distant metastases, development of multi-drug resistance phenotype or both together. This study demonstrated that some of the multi-drug resistant cancer cells may also become radioresistant. Materials and Methods: Chemoresistance in paclitaxel (MCF-7/Pac), docetaxel (MCF-7/Doc), vincristine (MCF-7/Vinc), doxorubicin (MCF-7/Dox) and zoledronic acid (MCF-7/Zol) resistant MCF-7 cells were demonstrated by XTT assay. MDR1 gene expression was detected by real-time PCR in human MCF-7 breast cancer cells. Drug resistant and sensitive cells were exposed to g-radiation and development of radioresistance was investigated. Results: Results have indicated that paclitaxel, docetaxel, vincristine, doxorubicin and zoledronic acid-selected cells gained varying degrees of resistance to their selective drugs when compared with original MCF-7/S. MCF-7/Pac, MCF-7/Doc, MCF-7/Vinc and MCF-7/Dox cells have all acquired MDR1 expression. Among the resistant sub-lines, MCF-7/Pac and MCF-7/Doc cells were significantly cross-resistant to irradiation compared to the sensitive cells. Conclusion: MCF-7/Pac and MCF-7/Doc cell lines were found radioresistant to g-radiation. On the contrary, doxorubicin, vincristine and zoledronic acid resistant cancer cells were still sensitive to radiation. [Kars, Meltem Demirel; Iseri, Ozlem Darcansoy; Gunduz, Ufuk] Middle East Tech Univ, Dept Biol Sci, TR-06531 Ankara, Turkey; [Ural, Ali Ugur; Avcu, Ferit] Gulhane Mil Acad, Sch Med, Dept Hematol, Ankara, Turkey; [Beyzadeoglu, Murat; Dirican, Bahar; Gunduz, Ufuk] Gulhane Mil Acad, Sch Med, Dept Radiat Oncol, Ankara, Turkey Middle East Technical University; Gulhane Military Medical Academy; Gulhane Military Medical Academy Gündüz, U (corresponding author), Middle East Tech Univ, Dept Biol Sci, TR-06531 Ankara, Turkey. ufukg@metu.edu.tr Kars, Meltem/A-7591-2019; işeri, özlem/AGE-6072-2022 TUBITAK, Turkey [SBAG 3297] TUBITAK, Turkey(Turkiye Bilimsel ve Teknolojik Arastirma Kurumu (TUBITAK))
Grants: This study was supported by TUBITAK (SBAG 3297, BIDEB), Turkey. 30 7 8 0 0 CAMBRIDGE UNIV

PRESS CAMBRIDGE EDINBURGH BLDG, SHAFTESBURY RD, CB2 8RU CAMBRIDGE, ENGLAND 1460-3969 1467-1131 J RADIOTHER PRACT J. Radiother. Pract. DEC 2009 8 4 207 213 10.1017/S1460396909990070 http://dx.doi.org/10.1017/S1460396909990070 7 Radiology, Nuclear Medicine & Medical Imaging Emerging Sources Citation Index (ESCI) Radiology, Nuclear Medicine & Medical Imaging V81KZ 2025-06-24 WOS:000212250400005

J Atmaca, H; Ilhan, S; Yilmaz, ES; Zora, M
Atmaca, Harika; Ilhan, Suleyman; Yilmaz, Elif Serel; Zora, Metin 4-Propargyl-substituted 1H-pyrroles induce apoptosis and autophagy via extracellular signal-regulated signaling pathway in breast cancer ARCHIV DER PHARMAZIE English Article
apoptosis; autophagy; cytotoxicity; ERK1; 2; pyrrole derivatives BIOLOGICAL-ACTIVITY; CELL-DEATH; PYRROLE; DERIVATIVES; MECHANISMS; ERK Novel pyrrole derivatives (PDs) with propargyl units (1-7) were investigated for their anticancer activity on breast cancer cells. The MTT assay was used to assess the cell viability. Morphological changes in human breast cancer cells were visualized under a phase-contrast microscope. Apoptosis and autophagy were detected using the DNA fragmentation assay and staining by autophagic vacuoles, respectively. The levels of apoptosis- and autophagy-related proteins such as cytochrome c, Bcl-2, LC3-I/II were investigated by Western blot analysis. The effect of PDs on the ERK1/2 signaling pathway was investigated using specific inhibitors. All the tested PDs were found to be active in the range of 36.7 +/- 0.2 to 459.7 +/- 4.2 mu M. Compounds 3 and 4 showed cytotoxic activity in breast cancer cells, but were found to be safer with lower cytotoxicity on human nontumorigenic epithelial breast cells. Compound 4 induced apoptosis, whereas compound 3 induced autophagy. Both compounds inhibited the ERK signaling pathway in breast cancer cells. The present study revealed that both synthesized PDs induced different programmed cell death types by inhibiting the ERK signaling pathway in two genotypically different breast cancer cells. Therefore, novel PDs might be promising anticancer agents for breast cancer therapy and further structural modifications of PDs may yield promising anticancer agents. [Atmaca, Harika; Ilhan, Suleyman] Manisa Celal Bayar Univ, Fac Sci & Letters, Dept Biol, TR-45140 Manisa, Turkey; [Yilmaz, Elif Serel; Zora, Metin] Middle East Tech Univ, Dept Chem, Ankara, Turkey Celal Bayar University; Middle East Technical University Atmaca, H (corresponding author), Manisa Celal Bayar Univ, Fac Sci & Letters, Dept Biol, TR-45140 Manisa, Turkey.
harika.atmaca@cbu.edu.tr Yılmaz, Elif Serel/LPP-8610-2024; Zora, Metin/ABB-7678-2020; atmaca, harika/AAX-8164-2021; ILHAN, Suleyman/AAZ-2000-2021 Zora, Metin/0000-0001-7764-2288; Yilmaz, Elif Serel/0000-0001-7541-1507; atmaca, harika/0000-0002-8459-4373; ILHAN, Suleyman/0000-0002-6584-3979
34 2 2 0 6 WILEY-V C H VERLAG GMBH WEINHEIM POSTFACH 101161, 69451 WEINHEIM, GERMANY 0365-6233 1521-4184 ARCH PHARM Arch. Pharm. OCT 2021 354 10 e2100170 10.1002/ardp.202100170 http://dx.doi.org/10.1002/ardp.202100170 JUN 2021 9 Chemistry, Medicinal; Chemistry, Multidisciplinary;

Pharmacology & Pharmacy	Science Citation Index Expanded (SCI-EXPANDED)	Pharmacology & Pharmacy; Chemistry	UZ8SZ	34165807
2025-06-24	WOS:000665104600001
J	Ozdal, T; Sari-Kaplan, G; Mutlu-Altundag, E; Boyacioglu, D; Capanoglu, E		Ozdal, Tugba; Sari-Kaplan, Gulce; Mutlu-Altundag, Ergul; Boyacioglu, Dilek; Capanoglu, Esra
Evaluation of Turkish propolis for its chemical composition, antioxidant capacity, anti-proliferative effect on several human breast cancer cell lines and proliferative effect on fibroblasts and mouse mesenchymal stem cell line	JOURNAL OF APICULTURAL RESEARCH		English	Article
propolis; antioxidant capacity; phenolic profile; HPLC-PDA; breast cancer cell line; mesenchymal stem cell line	RADICAL-SCAVENGING ACTIVITY; BRAZILIAN RED PROPOLIS; POPLAR-TYPE PROPOLIS; PHENOLIC-COMPOUNDS; IN-VITRO; CONSTITUENTS; APOPTOSIS; EXTRACTS; REGIONS; ASSAY	Propolis is an extremely complex resinous natural compound collected by honey bees from various plant sources and exhibits pharmacological and biological properties attributed to the presence of polyphenols. This study examined the total phenolic and flavonoid contents as well as the total antioxidant capacity using 2,2 '-azino-bis(3-ethylbenzothiazoline-6-sulphonic acid (ABTS), 2,2-diphenyl-1-picrylhydrazyl (DPPH), cupric-reducing antioxidant capacity (CUPRAC), and ferric-reducing antioxidant power (FRAP) methods. Turkish propolis has very high total phenolic (314.36 +/- 3.65 mg GAE/g propolis) and total flavonoid contents (522.71 +/- 11.45 mg QE/g propolis). The highest antioxidant capacity value was obtained by the CUPRAC method (1184.94 +/- 63.27 mg TE/g propolis). Phenolic profile of Turkish propolis was also determined by high performance liquid chromatography with photodiode array detection (HPLC-PDA) method. The main phenolic compounds identified in Turkish propolis was flavonoids including pinocembrin > chrysin > galangin > pinobanksin > pinostrobin and phenolic acids including caffeic acid > p-coumaric acid > ferrulic acid > t-cinnamic acid. In the present work, anti-proliferative and proliferative effects of propolis extracts were also investigated on two different breast cancer cell lines; MDA-MB-231, UACC-3199 and on two normal cell lines; fibroblasts and mouse mesenchymal stem cell lines. According to the XTT results, Turkish propolis sample showed significant anti-proliferative effect on MDA-MB-231 and UACC breast cancer cell lines. Interestingly, Turkish propolis sample had proliferative effect on both fibroblasts and mouse mesenchymal stem cells. These results suggest that Turkish propolis can be considered as a potent agent on breast cancer treatment for further investigations.	[Ozdal, Tugba] Okan Univ, Fac Engn & Architecture, Dept Food Engn, Istanbul, Turkey; [Sari-Kaplan, Gulce] Okan Univ, Dept Genet & Bioengn, Fac Engn & Architecture, Istanbul, Turkey; [Mutlu-Altundag, Ergul] Eastern Mediterranean Univ, Fac Med, Dept Biochem, Famagusta, Turkey; [Boyacioglu, Dilek; Capanoglu, Esra] Istanbul Tech Univ, Fac Chem & Met Engn, Dept Food Engn, Istanbul, Turkey	Okan University; Okan University; Eastern Mediterranean University; Istanbul Technical University
Capanoglu, E (corresponding author), Istanbul Tech Univ, Fac Chem & Met Engn, Dept Food Engn, Istanbul, Turkey.
capanogl@itu.edu.tr	Capanoglu, Esra/A-4455-2018; Mutlu Altundağ, Ergul/AFK-4360-2022; SARI, Gulce/ISS-7005-2023; Ozdal, Tugba/B-5799-2018; Boyacioglu, Dilek/AAX-8482-2020 SARI,

Gulce/0000-0002-8585-5889; Capanoglu, Esra/0000-0003-0335-9433; mutlu Altundag, ergul/0000-0002-2377-4047; BOYACIOGLU, DILEK/0000-0002-8160-0619 92 23 25 0 42
TAYLOR & FRANCIS LTD ABINGDON 2-4 PARK SQUARE, MILTON PARK, ABINGDON OR14 4RN, OXON, ENGLAND 0021-8839 2078-6913
J APICULT RES J. Apic. Res. OCT 20 2018 57 5 627 638
10.1080/00218839.2018.1494888
http://dx.doi.org/10.1080/00218839.2018.1494888
12 Entomology Science Citation Index Expanded (SCI-EXPANDED) Entomology GY3EA 2025-06-24
WOS:000448429100005
J Bahr, S; Bzieh, R; El Hayek, GY; Adib, S
Bahr, Sarah; Bzieh, Rania; El Hayek, Ghinwa Y.; Adib, Salim
Cost-benefit analysis of a projected national human papilloma virus vaccination programme in Lebanon EASTERN MEDITERRANEAN HEALTH JOURNAL English Article
HPV vaccination; cost benefit analysis; cervical cancer; Lebanon EXTENDED MIDDLE-EAST; CERVICAL-CANCER; HPV VACCINE; IMPACT; QUADRIVALENT; PREVENTION; INFECTION; EFFICACY; BIVALENT; HEALTH Background: The adoption of a population-based human papilloma virus (HPV) vaccination programme is debated in Lebanon on epidemiological, sociocultural, logistical and economic grounds. Aims: This cost-benefit analysis contributes to generating quantitative evidence necessary for a decision regarding costs through locally available data. Methods: The 2 sides of the cost-benefit analysis equation are: estimation of the cost of HPV vaccination campaigns targeting 11 year-old girls, using the cheapest vaccine in 2016 and estimation of the management cost for treatment of a yearly average case-load for cervical cancer. Results: A Cervarix (R) only campaign would cost US$ 5 407 790 to vaccinate 38 08311-year-old girls. The estimated cost of managing a mean annual mixed case-load of 100 incident cervical cancer cases would cost US$ 1 591 336. The nearest break-even point may occur 5 years after this current analysis. Conclusion: This cost-benefit analysis using limited available data indicates that massive HPV vaccination would not be cost-beneficial under the circumstances existing in 2016. Nevertheless, some indications point to the need for a re-assessment around 2020. This finding will inform public health decision-makers in Lebanon and similar neighbouring countries. [Bahr, Sarah; Bzieh, Rania; El Hayek, Ghinwa Y.; Adib, Salim] Amer Univ Beirut, Fac Hlth Sci, Dept Epidemiol & Populat Hlth, Beirut, Lebanon American University of Beirut El Hayek, GY (corresponding author), Amer Univ Beirut, Fac Hlth Sci, Dept Epidemiol & Populat Hlth, Beirut, Lebanon. gyeo1@mail.aub.edu
37 5 5 0 7 WHO EASTERN MEDITERRANEAN REGIONAL OFFICE NASR CITY, CAIRO P. O. BOX 7608, NASR CITY, CAIRO, EGYPT 1020-3397 1687-1634 E MEDITERR HEALTH J
East Mediterr. Health J. OCT 2019 25 10
715 721 10.26719/2019.25.10.715
http://dx.doi.org/10.26719/2019.25.10.715 7
Health Care Sciences & Services; Health Policy & Services; Public, Environmental & Occupational Health Science Citation Index Expanded (SCI-EXPANDED); Social Science Citation Index (SSCI) Health Care Sciences & Services; Public, Environmental & Occupational Health JK6GQ 31774137 Bronze
2025-06-24 WOS:000494940100007

J Pérez-Jiménez, J; Díaz-Rubio, ME; Saura-Calixto, F
Perez-Jimenez, Jara; Elena Diaz-Rubio, M.; Saura-Calixto, Fulgencio Contribution of Macromolecular Antioxidants to Dietary Antioxidant Capacity: A Study in the Spanish Mediterranean Diet PLANT FOODS FOR HUMAN NUTRITION
English Article
Dietary antioxidants; Dietary antioxidant capacity; Food antioxidant capacity; Macromolecular antioxidants; Non-extractable polyphenols; Spanish Mediterranean diet NONEXTRACTABLE POLYPHENOLS; CARDIOVASCULAR-DISEASE; MOLECULAR-MECHANISMS; PLANT FOODS; FIBER; CANCER; FRUITS; ASSAY; MICE Epidemiological and clinical studies show that diets with a high antioxidant capacity, such us those rich in plant food and beverages, are associated with significant decreases in the overall risk of cardiovascular disease or colorectal cancer. Current studies on dietary antioxidants and dietary antioxidant capacity focus exclusively on low molecular weight or soluble antioxidants (vitamins C and E, phenolic compounds and carotenoids), ignoring macromolecular antioxidants. These are polymeric phenolic compounds or polyphenols and carotenoids linked to plant food macromolecules that yield bioavailable metabolites by the action of the microbiota with significant effects either local and/or systemic after absorption. This study determined the antioxidant capacity of the Spanish Mediterranean diet including for the first time both soluble and macromolecular antioxidants. Antioxidant capacity and consumption data of the 54 most consumed plant foods and beverages were used. Results showed that macromolecular antioxidants are the major dietary antioxidants, contributing a 61 % to the diet antioxidant capacity (8000 mu mol Trolox, determined by ABTS method). The antioxidant capacity data for foods and beverages provided here may be used to estimate the dietary antioxidant capacity in different populations, where similar contributions of macromolecular antioxidants may be expected, and also to design antioxidant-rich diets. Including macromolecular antioxidants in mechanistic, intervention and observational studies on dietary antioxidants may contribute to a better understanding of the role of antioxidants in nutrition and health.
[Perez-Jimenez, Jara; Elena Diaz-Rubio, M.; Saura-Calixto, Fulgencio] Inst Food Sci Technol & Nutr ICTAN CSIC, Dept Metab & Nutr, Madrid, Spain Consejo Superior de Investigaciones Cientificas (CSIC); CSIC - Instituto de Ciencia y Tecnologia de Alimentos y Nutricion (ICTAN) Saura-Calixto, F (corresponding author), Inst Food Sci Technol & Nutr ICTAN CSIC, Dept Metab & Nutr, Madrid, Spain. jara.perez@ictan.csic.es Diaz, Maria Elena/AAF-2138-2021; Perez-Jimenez, Jara/B-3989-2009 Perez-Jimenez, Jara/0000-0002-2811-4558 Spanish Ministry of Economy and Competitiveness [AGL2011-27,741]; CSIC; MINNECO Spanish Ministry of Economy and Competitiveness(Spanish Government); CSIC; MINNECO This research was supported by the Spanish Ministry of Economy and Competitiveness (AGL2011-27,741). M.E. D-R. and J. P-J. acknowledge the CSIC and the MINNECO for JAE-Doc and Juan de la Cierva postdoctoral contracts, respectively. 34 52 53
9 190 SPRINGER DORDRECHT VAN GODEWIJCKSTRAAT 30, 3311 GZ DORDRECHT, NETHERLANDS 0921-9668 1573-9104
PLANT FOOD HUM NUTR Plant Food Hum. Nutr. DEC 2015 70
4 365 370 10.1007/s11130-015-0513-6 http://dx.doi.org/10.1007/s11130-015-0513-6
6 Plant Sciences; Chemistry, Applied; Food Science &

Technology; Nutrition & Dietetics Science Citation Index Expanded (SCI-EXPANDED) Plant Sciences; Chemistry; Food Science & Technology; Nutrition & Dietetics CX2FI 26482738
2025-06-24 WOS:000365510900002
J Bartmann, C; Raman, SRJ; Floter, J; Schulze, A; Bahlke, K; Willingstorfer, J; Strunz, M; Wockel, A; Klement, RJ; Kapp, M; Djuzenova, CS; Otto, C; Kammerer, U Bartmann, Catharina; Raman, Sudha R. Janaki; Floeter, Jessica; Schulze, Almut; Bahlke, Katrin; Willingstorfer, Jana; Strunz, Maria; Woeckel, Achim; Klement, Rainer J.; Kapp, Michaela; Djuzenova, Cholpon S.; Otto, Christoph; Kaemmerer, Ulrike
Beta-hydroxybutyrate (3-OHB) can influence the energetic phenotype of breast cancer cells, but does not impact their proliferation and the response to chemotherapy or radiation
CANCER & METABOLISM English Article
Ketogenic diet; beta-Hydroxybutyrate; Ketone bodies; Breast cancer; Seahorse; Metabolic profile; Chemotherapy; Ionizing radiation KETOGENIC MEDITERRANEAN DIET; KETONE-BODIES; BODY-COMPOSITION; MONOCARBOXYLATE TRANSPORTERS; ADJUVANT CHEMOTHERAPY; TUMOR METABOLISM; LACTATE INCREASE; GLYCEMIC INDEX; VITAMIN-D; GROWTH Background: Ketogenic diets (KDs) or short-term fasting are popular trends amongst supportive approaches for cancer patients. Beta-hydroxybutyrate (3-OHB) is the main physiological ketone body, whose concentration can reach plasma levels of 2-6 mM during KDs or fasting. The impact of 3-OHB on the biology of tumor cells described so far is contradictory. Therefore, we investigated the effect of a physiological concentration of 3 mM 3-OHB on metabolism, proliferation, and viability of breast cancer (BC) cells in vitro. Methods: Seven different human BC cell lines (BT20, BT474, HBL100, MCF-7, MDA-MB 231, MDA-MB 468, and T47D) were cultured in medium with 5 mM glucose in the presence of 3 mM 3-OHB at mild hypoxia (5% oxygen) or normoxia (21% oxygen). Metabolic profiling was performed by quantification of the turnover of glucose, lactate, and 3-OHB and by Seahorse metabolic flux analysis. Expression of key enzymes of ketolysis as well as the main monocarboxylic acid transporter MCT2 and the glucose-transporter GLUT1 was analyzed by RT-qPCR and Western blotting. The effect of 3-OHB on short- and long-term cell proliferation as well as chemo-and radiosensitivity were also analyzed. Results: 3-OHB significantly changed the oxygen consumption rate (OCR) and extracellular acidification rate (ECAR) in BT20 cells resulting in a more oxidative energetic phenotype. MCF-7 and MDA-MB 468 cells had increased ECAR only in response to 3-OHB, while the other three cell types remained uninfluenced. All cells expressed MCT2 and GLUT1, thus being able to uptake the metabolites. The consumption of 3-OHB was not strongly linked to mRNA overexpression of key enzymes of ketolysis and did not correlate with lactate production and glucose consumption. Neither 3-OHB nor acetoacetate did interfere with proliferation. Further, 3-OHB incubation did not modify the response of the tested BC cell lines to chemotherapy or radiation. Conclusions: We found that a physiological level of 3-OHB can change the energetic profile of some BC cell lines. However, 3-OHB failed to influence different biologic processes in these cells, e.g., cell proliferation and the response to common breast cancer chemotherapy and radiotherapy. Thus, we have no evidence that 3-OHB generally influences the biology of breast cancer cells in vitro.
[Bartmann, Catharina; Bahlke, Katrin; Willingstorfer, Jana;

Strunz, Maria; Woeckel, Achim; Kapp, Michaela; Kaemmerer, Ulrike] Univ Hosp Wurzburg, Dept Obstet & Gynaecol, Josef Schneider Str 4, D-97080 Wurzburg, Germany; [Raman, Sudha R. Janaki; Floeter, Jessica; Schulze, Almut] Univ Wurzburg, Bioctr, Theodor Boveri Inst, Dept Biochem & Mol Biol, D-97070 Wurzburg, Germany; [Klement, Rainer J.] Leopoldina Hosp, Dept Radiotherapy & Radiat Oncol, D-97422 Schweinfurt, Germany; [Djuzenova, Cholpon S.] Univ Hosp Wurzburg, Dept Radiotherapy, D-97080 Wurzburg, Germany; [Otto, Christoph] Univ Hosp Wurzburg, Dept Gen Visceral Vasc & Pediat Surg, Expt Surg, D-97080 Wurzburg, Germany University of Wurzburg; University of Wurzburg; University of Wurzburg; University of Wurzburg Kammerer, U (corresponding author), Univ Hosp Wurzburg, Dept Obstet & Gynaecol, Josef Schneider Str 4, D-97080 Wurzburg, Germany. frak057@mail.uni-wuerzburg.de
Klement, Rainer/H-3484-2019; Schulze, Almut/AAX-8257-2020
Interdisciplinary Centre for Clinical Research (IZKF) University Hospital of Wurzburg [Z-2/66] Interdisciplinary Centre for Clinical Research (IZKF) University Hospital of Wurzburg Part of this work was supported by a grant of the Interdisciplinary Centre for Clinical Research (IZKF) University Hospital of Wurzburg (Z-2/66 to C.B.). 113 36 39 0
17 BMC LONDON CAMPUS, 4 CRINAN ST, LONDON N1 9XW, ENGLAND 2049-3002 CANCER METAB Cancer Metab.
JUN 11 2018 6
8 10.1186/s40170-018-0180-9
http://dx.doi.org/10.1186/s40170-018-0180-9 19
Oncology; Cell Biology Science Citation Index Expanded (SCI-EXPANDED) Oncology; Cell Biology GJ2AD 29942509 gold, Green Published 2025-06-24 WOS:000435068100001
J Eghbali, SS; Amirinejad, R; Obeidi, N; Mosadeghzadeh, S; Vahdat, K; Azizi, F; Pazoki, R; Sanjdideh, Z; Amiri, Z; Nabipour, I; Zandi, K Eghbali, Seyed Sajjad; Amirinejad, Roya; Obeidi, Narges; Mosadeghzadeh, Shiva; Vahdat, Katayoun; Azizi, Fatemeh; Pazoki, Raha; Sanjdideh, Zahra; Amiri, Zahra; Nabipour, Iraj; Zandi, Keivan Oncogenic human papillomavirus genital infection in southern Iranian women: population-based study versus clinic-based data VIROLOGY JOURNAL English Article
Human papilloma virus; Bushehr; Cervical cancer; Iran; PCR CERVICAL-CANCER; HPV TYPES; PREVALENCE; CYTOLOGY; WORLDWIDE; SMEAR Background: Epidemiological studies on genital human papilloma viruses infection (HPVs) in general population are crucial for the implementation of health policy guidelines for developing the strategies to prevent the primary and secondary cervical cancer. In different parts of Iran, there is a lack of population-based studies to determine the prevalence of HPV in the general population. The aim of this population-based study is to compare the prevalence rate of genital HPV infection among reproductive women with our previous clinic-based data, which showed a prevalence rate of 5% in women in southern Iran. Results: Using general primers for all genotypes of HPV, of 799 randomly selected women, five (0.63%, 95% CI 0.23-1.55%) tested positive for HPV DNA. Overall, seven different HPV genotypes were detected: six types (16, 18, 31, 33, 51 and 56) were carcinogenic, or "high risk genotypes" and one genotype (HPV-66) was "probably carcinogenic." Conclusions: In a population-based study, the prevalence of HPV infection among southern Iranian women was lower than that observed worldwide. However, our gynaecological clinic-

based study on the prevalence of HPV infection showed results comparable with other studies in the Middle East and Persian Gulf countries. Since gynaecological clinic-based data may generally overestimate HPV prevalence, estimates of prevalence according to clinic-based data should be adjusted downward by the population-based survey estimates. [Eghbali, Seyed Sajjad; Amirinejad, Roya; Obeidi, Narges; Mosadeghzadeh, Shiva; Vahdat, Katayoun; Azizi, Fatemeh; Pazoki, Raha; Sanjdideh, Zahra; Amiri, Zahra; Zandi, Keivan] Bushehr Univ Med Sci, Persian Gulf Trop Med Res Ctr, Dept Virol, Bushehr, Iran; [Zandi, Keivan] Univ Malaya, Fac Med Trop & Infect Dis, Res & Educ Ctr, Dept Med Microbiol, Kuala Lumpur, Malaysia; [Eghbali, Seyed Sajjad] Bushehr Univ Med Sci, Sch Med, Dept Pathol, Bushehr, Iran; [Nabipour, Iraj] Bushehr Univ Med Sci, Persian Gulf Marine Biotechnol Res Ctr, Dept Biochem, Bushehr, Iran Universiti Malaya Zandi, K (corresponding author), Bushehr Univ Med Sci, Persian Gulf Trop Med Res Ctr, Dept Virol, Bushehr, Iran. keivanzandi@yahoo.com ZANDI, KEIVAN/B-8762-2010; Vahdat, Katayoun/D-8911-2017; Obeidi, Narges/D-4656-2017; nabipour, Iraj/D-8924-2017 Pazoki, Raha/0000-0002-5142-2348; Vahdat, Katayoun/0000-0001-9782-2067; Zandi, Keivan/0000-0001-5107-8754; Obeidi, Narges/0000-0001-8087-2850; Eghbali, Seyed Sajad/0000-0002-2524-8243; nabipour, Iraj/0000-0002-1785-0883; Zandi, Keivan/0000-0001-9728-5038 Bushehr Province Technology and Research Committee; Research Deputy of Bushehr University of Medical Science Bushehr Province Technology and Research Committee; Research Deputy of Bushehr University of Medical Science This study was supported in part by a grant from the Bushehr Province Technology and Research Committee and the Research Deputy of Bushehr University of Medical Science.
33 17 18 0 6 BMC LONDON CAMPUS, 4 CRINAN ST, LONDON N1 9XW, ENGLAND 1743-422X VIROL J
Virol. J. SEP 12 2012 9
194 10.1186/1743-422X-9-194
http://dx.doi.org/10.1186/1743-422X-9-194 6
Virology Science Citation Index Expanded (SCI-EXPANDED)
Virology 015RP 22967396 Green Published, gold
2025-06-24 WOS:000309464000001
J Pourianazar, NT; Gunduz, U Pourianazar, Negar Taghavi; Gunduz, Ufuk Changes in apoptosis-related gene expression and cytokine release in breast cancer cells treated with CpG-loaded magnetic PAMAM nanoparticles
INTERNATIONAL JOURNAL OF PHARMACEUTICS English
Article CpG-ODN; Apoptosis-related genes; Cytokine release; Dendrimeric nanoparticles; Magnetic nanoparticles TOLL-LIKE RECEPTORS; CELLULAR INVASION; OVARIAN-CANCER; BCL-2 FAMILY; SURVIVIN; PROLIFERATION; INTERLEUKIN-6; DELIVERY; BAX; OLIGODEOXYNUCLEOTIDES CpG-oligodeoxynucleotide (CpG-ODN) can function as an immune adjuvant. Previously, we showed that stimulation of breast cancer cells with CpG-ODN conjugated with PAMAM dendrimer-coated magnetic nanoparticles (DcMNPs) has induced apoptosis. The aim of the current study was to evaluate the expression levels of some apoptosis-regulating genes in several human breast cancer cells treated with CpG/DcMNPs. Treated MDA-MB231 cells showed an increase in Noxa and Bax gene expression levels, whereas the expression level of Survivin decreased. Similarly, Noxa gene was overexpressed in treated MCF7 cells. In treated SKBR3 cells, a decline in the c-Flip mRNA level was determined. Furthermore,

release of cytokines, IL-6, IL-10, and TNF-alpha, was determined in cell culture supernatants. CpG/DcMNP treatment leads to an increase in the release of IL-6 in MDA-MB231 and SKBR3 cells, whereas release of IL-10 and TNF-alpha did not change significantly. It is indicated that CpG-ODN may show its cytotoxic effect by regulating the expression of apoptosis-related genes and the release of cytokine in breast cancer cells. (C) 2016 Elsevier B.V. All rights reserved. [Pourianazar, Negar Taghavi; Gunduz, Ufuk] Middle East Tech Univ, Dept Biotechnol, TR-06800 Ankara, Turkey; [Gunduz, Ufuk] Middle East Tech Univ, Dept Biol Sci, TR-06800 Ankara, Turkey Middle East Technical University; Middle East Technical University Pourianazar, NT (corresponding author), Middle East Tech Univ, Dept Biol Sci, TR-06800 Ankara, Turkey. Negar_taghavi22@yahoo.com Taghavi Pourianazar, Negar/IZP-9751-2023 Scientific and Technical Research Council of Turkey (TUBITAK) [2215, 1002] Scientific and Technical Research Council of Turkey (TUBITAK)(Turkiye Bilimsel ve Teknolojik Arastirma Kurumu (TUBITAK)) This work is supported by the Scientific and Technical Research Council of Turkey (TUBITAK-grant 2215 and 1002). 59 11 11 1 45 ELSEVIER SCIENCE BV AMSTERDAM PO BOX 211, 1000 AE AMSTERDAM, NETHERLANDS
0378-5173 1873-3476 INT J PHARMACEUT Int. J. Pharm.
DEC 30 2016 515 1-2 11 19
10.1016/j.ijpharm.2016.10.007
http://dx.doi.org/10.1016/j.ijpharm.2016.10.007
9 Pharmacology & Pharmacy Science Citation Index Expanded (SCI-EXPANDED) Pharmacology & Pharmacy ED8WD
27717915 2025-06-24 WOS:000389150700002
J Menendez, JA; Vazquez-Martin, A; Oliveras-Ferraros, C; Garcia-Villalba, R; Carrasco-Pancorbo, A; Fernandez-Gutierrez, A; Segura-Carreter, A Menendez, Javier A.; Vazquez-Martin, Alejandro; Oliveras-Ferraros, Cristina; Garcia-Villalba, Rocio; Carrasco-Pancorbo, Alegria; Fernandez-Gutierrez, Alberto; Segura-Carreter, Antonio Extra-virgin olive oil polyphenols inhibit HER2 (erbB-2)-induced malignant transformation in human breast epithelial cells: Relationship between the chemical structures of extra-virgin olive oil secoiridoids and lignans and their inhibitory activities on the tyrosine kinase activity of HER2 INTERNATIONAL JOURNAL OF ONCOLOGY English Article
olive oil; Mediterranean diet; phenolics; breast cancer; HER2; tyrosine kinases MONOUNSATURATED FATTY-ACID; APIGENIN INDUCES APOPTOSIS; C-HA-RAS; OLEIC-ACID; PHENOLIC-COMPOUNDS; CANCER-CELLS; TRASTUZUMAB HERCEPTIN(TM); CAPILLARY-ELECTROPHORESIS; BIOLOGICAL-ACTIVITIES; MOLECULAR-MECHANISMS
Depending on their structure, Some polyphenols (e.g. flavonoids) abundantly found in plant-derived beverages Such as green tea can efficiently inhibit tyrosine kinase and serine/threonine kinase activities. Extra-virgin olive oil (EVOO - the juice of the olive obtained solely by pressing and consumed without any further refining process) is unique among other vegetable oils because of the high level of naturally occurring phenolic compounds, We explored the ability of EVOO polyphenols to modulate HER2 tyrosine kinase receptor-induced in vitro transformed phenotype in human breast epithelial cells. Using MCF10A normal breast epithelial cells retrovirally engineered to overexpress the wild-type sequence of human HER2, we further determined the relationship between chemical structures of EVOO-

derived phenolics and their inhibitory activities on the tyrosine kinase activity of the HER2 oncoprotein. When the activation (phosphorylation) status of HER2 was semi-quantitatively measured the secoiridoids blocked HER2 signaling by rapidly reducing the activation status of the 1248 tyrosine residue (Y1248), the main autophosphorylation site of HER2. EVOO-derived single phenols tyrosol and hydroxytyrosol and the phenolic acid elenolic acid failed to significantly decrease HER2 tyrosine kinase activity. The anti-HER2 tyrosine kinase activity IC50 values were up to 5-times lower in the presence of EVOO-derived lignans and secoiridoids than in the presence of EVOO-derived single phenols and phenolic acids. EVOO polyphenols induced strong tumoricidal effects by selectively triggering high levels of apoptotic cell death in HER2-positive MCF10A/HER2 cells but not in MCF10A/pBABE matched control cells. EVOO lignans and secoiridoids prevented HER2-induced in vitro transformed phenotype as they inhibited colony formation of MCF10A/HER2 cells in soft-agar. Our current findings not only molecularly support recent epidemiological evidence revealing that EVOO-related anti-breast cancer effects primarily affect the occurrence of breast tumors over-expressing the type I receptor tyrosine kinase HER2 but further suggest that the stereochemistry of EVOO-derived lignans and secoiridoids might provide an excellent and safe platform for the design of new HER2 targeted anti-breast cancer drugs. [Menendez, Javier A.; Vazquez-Martin, Alejandro; Oliveras-Ferraros, Cristina] Dr Josep Trueta Univ Hosp Girona, ICO, Hlth Serv Div Catalonia, Girona Biomed Res Inst IdIBGi, E-17007 Girona, Catalonia, Spain; [Garcia-Villalba, Rocio; Carrasco-Pancorbo, Alegria; Fernandez-Gutierrez, Alberto; Segura-Carreter, Antonio] Univ Granada, Dept Analyt Chem, Fac Sci, E-18071 Granada, Spain Institut Catala d'Oncologia; Universitat de Girona; Girona University Hospital Dr. Josep Trueta; Institut d'Investigacio Biomedica de Girona (IDIBGI); University of Granada Menendez, JA (corresponding author), Dr Josep Trueta Univ Hosp Girona, ICO, Hlth Serv Div Catalonia, Girona Biomed Res Inst IdIBGi, Ave Francia S-N, E-17007 Girona, Catalonia, Spain. jmenendez@ico.scs.es; ansegura@ugr.es

Carretero, Antonio/B-6867-2014; Villalba, Rocio/G-7217-2015; Simal-Gandara, Jesus/A-9533-2009; MENENDEZ MENENDEZ, JAVIER ABEL/C-6148-2016; Fernandez Gutierrez, Alberto/M-8512-2014

Simal-Gandara, Jesus/0000-0001-9215-9737; MENENDEZ MENENDEZ, JAVIER ABEL/0000-0001-8733-4561; Fernandez Gutierrez, Alberto/0000-0003-3647-2598; Carrasco Pancorbo, Alegria/0000-0001-8856-4676; Garcia Villalba, Rocio/0000-0003-1883-1673 Susan G. Komen Breast Cancer Foundation (TX, USA) [BCTR0600894]; Instituto de Salud Carlos III (Ministerio de Sanidad y Consumo, Fondo de Investigacion Sanitaria -FIS-, Spain [CP05-00090, P106-0778, RD06-0020-0028]; Fundacion Cientifica de la Asociacion Espanola Contra el Cancer (AECC, Spain); Ministerio de Educacion y Ciencia [CTQ2005-01914/BQU]; Junta de Andalucia [AGR-02619] Susan G. Komen Breast Cancer Foundation (TX, USA)(Susan G. Komen Breast Cancer Foundation); Instituto de Salud Carlos III (Ministerio de Sanidad y Consumo, Fondo de Investigacion Sanitaria -FIS-, Spain(Instituto de Salud Carlos III); Fundacion Cientifica de la Asociacion Espanola Contra el Cancer (AECC, Spain); Ministerio de Educacion y Ciencia(Spanish Government); Junta de Andalucia(Junta de Andalucia) Javier A. Menendez is the recipient of a Basic, Clinical and Translational Research Award (BCTR0600894) from the Susan G. Komen Breast Cancer Foundation (TX, USA). This study was

supported in part by Instituto de Salud Carlos III (Ministerio de Sanidad y Consumo, Fondo de Investigacion Sanitaria -FIS-, Spain, Grants CP05-00090, P106-0778 and RD06-0020-0028 to Javier A. Menendez). Javier A. Menendez and Antonio Segura-Carretero are also supported by a Grant from the Fundacion Cientifica de la Asociacion Espanola Contra el Cancer (AECC, Spain) and by the Ministerio de Educacion y Ciencia CTQ2005-01914/BQU and Junta de Andalucia (Proyecto de Excelencia AGR-02619). 45 61 68 0 22 SPANDIDOS PUBL LTD ATHENS POB 18179, ATHENS, 116 10, GREECE 1019-6439 1791-2423 INT J ONCOL Int. J. Oncol. JAN 2009 34 1 43 51 10.3892/ijo_00000127 http://dx.doi.org/10.3892/ijo_00000127 9 Oncology Science Citation Index Expanded (SCI-EXPANDED) Oncology 389WT 19082476 Bronze 2025-06-24 WOS:000262127000005

J Manguoglu, AE; Lüleci, G; Özçelik, T; Çolak, T; Schayek, H; Akaydin, M; Friedman, E Manguoglu, A. Esra; Luleci, Guven; Ozcelik, Tayfun; Colak, Taner; Schayek, Hagit; Akaydin, Mustafa; Friedman, Eitan Germline Mutations in the BRCA1 and BRCA2 Genes in Turkish Breast/Ovarian Cancer Patients HUMAN MUTATION English Article BRCA1; BRCA2; DGGE; PTT; Turkish Population; high risk for breast cancer In this study we genotyped Turkish breast/ovarian cancer patients for BRCA1/BRCA2 mutations: protein truncation test (PTT) for exon 11 BRCA1 of and, multiplex PCR and denaturing gradient gel electrophoresis (DGGE) for BRCA2, complemented by DNA sequencing. In addition, a modified restriction assay was used for analysis of the predominant Jewish mutations: 185delAG, 5382InsC, Tyr978X (BRCA1) and 6174delT (BRCA2). Eighty three breast/ovarian cancer patients were screened: twenty three had a positive family history of breast/ovarian cancer, ten were males with breast cancer at any age, in eighteen the disease was diagnosed under 40 years of age, one patient had ovarian cancer in addition to breast cancer and one patient had ovarian cancer. All the rest (n=30) were considered sporadic breast cancer cases. Overall, 3 pathogenic mutations (3/53-5.7%) were detected, all in high risk individuals (3/23 - 13%): a novel (2990insA) and a previously described mutation (R1203X) in BRCA1, and a novel mutation (9255delT) in BRCA2. In addition, three missense mutations [two novel (T42S, N2742S) and a previously published one (S384F)] and two neutral polymorphisms (P9P, P2532P) were detected in BRCA2. Notably none of the male breast cancer patients harbored any mutation, and none of the tested individuals carried any of the Jewish mutations. Our findings suggest that there are no predominant mutations within exon 11 of the BRCA1 and in BRCA2 gene in Turkish high risk families. (c) 2003 Wiley-Liss, Inc. [Manguoglu, A. Esra; Luleci, Guven] Akdeniz Univ, Fac Med, Dept Med Biol & Genet, Antalya, Turkey; [Manguoglu, A. Esra; Schayek, Hagit; Friedman, Eitan] Chaim Sheba Med Ctr, Danek Gertner Inst Genet, Susanne Levy Gertner Oncogenet Unit, IL-52621 Tel Hashomer, Israel; [Ozcelik, Tayfun] Tel Aviv Univ, Sackler Sch Med, Ramat Aviv, Israel; [Colak, Taner; Akaydin, Mustafa] Bilkent Univ, Dept Mol Biol & Genet, TR-06533 Ankara, Turkey; Akdeniz Univ, Fac Med, Dept Surg, TR-07070 Antalya, Turkey Akdeniz University; Chaim Sheba Medical Center; Tel Aviv University; Sackler Faculty of Medicine; Ihsan Dogramaci Bilkent University; Akdeniz University Friedman,

E (corresponding author), Chaim Sheba Med Ctr, Danek Gertner Inst Genet, Susanne Levy Gertner Oncogenet Unit, IL-52621 Tel Hashomer, Israel.   eitan211@netvision.net.il   Middle East Cancer Consortium (MECC); Haifa Rotary Club; Moshe Greidinger Scholarship Fund; Aspendos Rotary Club Middle East Cancer Consortium (MECC); Haifa Rotary Club; Moshe Greidinger Scholarship Fund; Aspendos Rotary Club Grant sponsor: Middle East Cancer Consortium (MECC) to Eitan Friedman; Grant sponsors: Aspendos and Haifa Rotary Clubs; Moshe Greidinger Scholarship Fund.   34
21   21   0   1   WILEY HOBOKEN   111 RIVER ST, HOBOKEN 07030-5774, NJ USA   1059-7794   1098-1004   HUM MUTAT   Hum. Mutat.   APR   2003   21   4
590   10.1002/humu.9119
http://dx.doi.org/10.1002/humu.9119   7
Genetics & Heredity   Science Citation Index Expanded (SCI-EXPANDED)   Genetics & Heredity   VH8XX
2025-06-24 WOS:000457435600007
J   Oddo, VM; Mabli, J   Oddo, Vanessa M.; Mabli, James   Association of Participation in the Supplemental Nutrition Assistance Program and Psychological Distress   AMERICAN JOURNAL OF PUBLIC HEALTH   English
Article   SEASONAL AFFECTIVE-DISORDER; SERIOUS MENTAL-ILLNESS; MEDITERRANEAN DIETARY PATTERN; POISSON REGRESSION APPROACH; FOOD INSECURITY; DEPRESSIVE SYMPTOMS; WELFARE RECIPIENTS; GENERAL-POPULATION; FINANCIAL STRAIN; UNITED-STATES Objectives. We assessed whether households' participation in the Supplemental Nutrition Assistance Program (SNAP) was associated with improvements in well-being, as indicated by lower rates of psychological distress. Methods. We used longitudinal data for 3146 households in 30 states, collected between October 2011 and September 2012 for the SNAP Food Security survey, the largest longitudinal national survey of SNAP participants to date. Analyses compared households within days of program entry to the same households approximately 6 months later. We measured psychological distress in the past 30 days on a 6-item Kessler screening scale and used multivariable regression to estimate associations between SNAP participation and psychological distress. Results. A smaller percentage of household heads exhibited psychological distress after 6 months of participation in SNAP than at baseline (15.3% vs 23.2%; difference=-7.9%). In adjusted models, SNAP participation was associated with a decrease in psychological distress (adjusted relative risk = 0.72; 95% confidence interval=0.66, 0.78). Conclusions. Continuing support for federal nutrition programs, such as SNAP, may reduce the public health burden of mental illness, thus improving well-being among vulnerable populations.   [Oddo, Vanessa M.; Mabli, James] Math Policy Res, Cambridge, MA USA; [Oddo, Vanessa M.] Johns Hopkins Bloomberg Sch Publ Hlth, Dept Int Hlth, Program Human Nutr, Baltimore, MD 21205 USA   Mathematica; Johns Hopkins University; Johns Hopkins Bloomberg School of Public Health
Oddo, VM (corresponding author), Johns Hopkins Bloomberg Sch Publ Hlth, Dept Int Hlth, 615 N Wolfe St,Room W2501, Baltimore, MD 21205 USA. voddo1@jhu.edu   Mathematica Policy Research [GS-10F-0050L]   Mathematica Policy Research This article uses data from the SNAP Food Security (SNAPFS) study. The SNAPFS study was designed by the Food and Nutrition Service (FNS), US Department of Agriculture (USDA), and was conducted by Mathematica Policy Research under Contract number GS-10F-0050L

with FNS. The Harry D. Kruse Publication Award in Human Nutrition is gratefully acknowledged. 49 53 57 1 11 AMER PUBLIC HEALTH ASSOC INC WASHINGTON 800 I STREET, NW, WASHINGTON, DC 20001-3710 USA 0090-0036 1541-0048 AM J PUBLIC HEALTH Am. J. Public Health JUN 2015 105 6 E30 E35 10.2105/AJPH.2014.302480 http://dx.doi.org/10.2105/AJPH.2014.302480 6 Public, Environmental & Occupational Health Science Citation Index Expanded (SCI-EXPANDED); Social Science Citation Index (SSCI) Public, Environmental & Occupational Health CQ2XW 25880949 Green Published 2025-06-24 WOS:000360466800012

J Siraj, AK; Bu, R; Iqbal, K; Siraj, N; Al-Haqawi, W; Al-Badawi, IA; Parvathareddy, SK; Masoodi, T; Tulbah, A; Al-Dayel, F; Al-Kuraya, KS Siraj, Abdul K.; Bu, Rong; Iqbal, Kaleem; Siraj, Nabil; Al-Haqawi, Wael; Al-Badawi, Ismail A.; Parvathareddy, Sandeep Kumar; Masoodi, Tariq; Tulbah, Asma; Al-Dayel, Fouad; Al-Kuraya, Khawla S. Prevalence, spectrum, and founder effect of BRCA1 and BRCA2 mutations in epithelial ovarian cancer from the Middle East HUMAN MUTATION English Article

BRCA1; BRCA2; epithelial; mutation; ovarian cancer BREAST-CANCER; WOMEN; RISK; CARRIERS; SURGERY; ACCOUNT Germline mutations in breast cancer susceptibility gene 1 and 2 have previously been estimated to contribute to 13-18% of all epithelial ovarian cancer (EOC). To characterize the prevalence and effect of BRCA1 and BRCA2 mutations in Middle Eastern EOC patients, BRCA mutation screening was performed in 407 unselected ovarian cancer patients using targeted capture and/or Sanger sequencing. A total of 19 different pathogenic variants (PVs) were identified in 50 (12.3%) women. Nine PVs were recurrent accounting for 80% of cases with PVs (40/50) in the entire cohort. Founder mutation analysis revealed only two mutations (c.4136_4137delCT and c.1140dupG) sharing the same haplotypes thus representing founder mutations in the Middle Eastern population. Identification of the mutation spectrum, prevalence, and founder effect in Middle Eastern population facilitates genetic counseling, risk assessment, and development of a cost-effective screening strategy. [Siraj, Abdul K.; Bu, Rong; Iqbal, Kaleem; Siraj, Nabil; Al-Haqawi, Wael; Parvathareddy, Sandeep Kumar; Masoodi, Tariq; Al-Kuraya, Khawla S.] King Faisal Specialist Hosp & Res Ctr, Res Ctr, Human Canc Genom Res, Riyadh, Saudi Arabia; [Al-Badawi, Ismail A.] King Faisal Specialist Hosp & Res Ctr, Dept Obstet & Gynecol, Riyadh, Saudi Arabia; [Tulbah, Asma] King Faisal Specialist Hosp & Res Ctr, Dept Pathol & Lab Med, Riyadh, Saudi Arabia; [Al-Dayel, Fouad] King Faisal Specialist Hosp & Res Ctr, Dept Pathol, Riyadh, Saudi Arabia King Faisal Specialist Hospital & Research Center; King Faisal Specialist Hospital & Research Center; King Faisal Specialist Hospital & Research Center; King Faisal Specialist Hospital & Research Center Al-Kuraya, KS (corresponding author), King Faisal Specialist Hosp & Res Canc, Human Canc Genom Res, Res Ctr, MBC 98-16,POB 3354, Riyadh 11211, Saudi Arabia. kkuraya@kfshrc.edu.sa Siraj, Abdul/IQW-1179-2023; Bu, Rong/JBI-9735-2023; Siraj, Nabil/IQU-4189-2023; Parvathareddy, Sandeep Kumar/JBI-9330-2023; Masoodi, Tariq/C-9843-2012; Iqbal, Kaleem/GXH-7291-2022; Alkuraya, Khawla/AFQ-7946-2022 Masoodi, Tariq/0000-0002-9186-6349; Al Dayel, Fouad/0000-0001-6175-9051; , Nabil/0000-0002-9412-5909; Alkuraya,

Khawla/0000-0002-4126-3419; Iqbal, Muhammad Kaleem/0000-0001-5634-5030 34 13 14 0 2 WILEY HOBOKEN 111 RIVER ST, HOBOKEN 07030-5774, NJ USA 1059-7794 1098-1004 HUM MUTAT Hum. Mutat. JUN 2019 40 6 729 733 10.1002/humu.23736 http://dx.doi.org/10.1002/humu.23736 5 Genetics & Heredity Science Citation Index Expanded (SCI-EXPANDED) Genetics & Heredity HZ1RI 30825404 gold 2025-06-24 WOS:000468625200007

J Djuric, Z; Vanloon, G; Radakovich, K; Dilaura, NM; Heilbrun, LK; Sen, A Djuric, Zora; Vanloon, Glee; Radakovich, Katherine; Dilaura, Nora M.; Heilbrun, Lance K.; Sen, Ananda Design of a Mediterranean Exchange List Diet Implemented by Telephone Counseling JOURNAL OF THE AMERICAN DIETETIC ASSOCIATION English Article CARDIOVASCULAR RISK; RANDOMIZED-TRIAL; BODY-WEIGHT; GREEK DIET; OLIVE OIL; CANCER; INTERVENTION; SURVIVAL; FRUIT; CAROTENOIDS A Greek-Mediterranean dietary pattern has two distinct aspects that differ relative to average intakes in the United States: a high intake of monounsaturated fats and a high intake of fruit and vegetables. The purpose of the study was to develop and test an exchange list Greek-Mediterranean diet that could be used in future clinical trials of breast cancer prevention. A total of 69 women, ages 25 to 59 years, were randomized to either continue their own usual diet or follow an intervention diet for 6 months during 2004 through 2005. Intervention goals were to decrease usual fat intakes by about half and to replace those fats with olive oil and other high-monounsaturated fatty acid foods; increase fruit and vegetable intakes to 7 to 9 servings/day, depending on energy intake; and consume at least one serving per day each of culinary herbs and album vegetables. Registered dietitians provided exchange goals and individualized telephone counseling, and diets were self-selected using a Mediterranean exchange list developed specifically for this study. Changes in diet were assessed by 7-day food records. Results demonstrated that counseling using the Mediterranean exchange list was effective for large dietary changes relative to the nonintervention group. Repeated measures analysis of variance indicated a statistically significant 48% increase in dietary monounsaturated fat with no appreciable change in total fat intake, and a significant increase in fruit and vegetable intake from 4.0 to 8.6 servings/day ($P < 0.05$).

[Djuric, Zora; Vanloon, Glee; Sen, Ananda] Univ Michigan, Dept Family Med, Ann Arbor, MI 48109 USA; [Radakovich, Katherine; Dilaura, Nora M.; Heilbrun, Lance K.] Karmanos Canc Inst, Detroit, MI USA University of Michigan System; University of Michigan; Barbara Ann Karmanos Cancer Institute Djuric, Z (corresponding author), Univ Michigan, Dept Family Med, 1500 E Hosp Dr,Room 2150 Canc Ctr, Ann Arbor, MI 48109 USA. zoralong@umich.edu

Anand, Amit/D-4232-2013; Djuric, Zora/H-5147-2013 Djuric, Zora/0000-0002-8886-8853 American Institute for Cancer Research [03B043]; National Institutes of Health (NIH) [CA-22453, CA-46592]; Chemistry Laboratory of the Michigan Diabetes Research and Training Center (MDRTC); General Clinical Research Center (GCRC) at the University of Michigan; National Institute of Diabetes and Digestive and Kidney Diseases [NIH5P60 DK20572]; National Center for Research Resources (NCRR) [M01-RR000042]; National Cancer Institute [P30CA046592] Funding Source: NIH RePORTER; National

Institute of Diabetes and Digestive and Kidney Diseases [P30DK020572] Funding Source: NIH RePORTER American Institute for Cancer Research; National Institutes of Health (NIH)(United States Department of Health & Human ServicesNational Institutes of Health (NIH) - USA); Chemistry Laboratory of the Michigan Diabetes Research and Training Center (MDRTC); General Clinical Research Center (GCRC) at the University of Michigan(United States Department of Health & Human ServicesNational Institutes of Health (NIH) - USANIH National Center for Research Resources (NCRR)); National Institute of Diabetes and Digestive and Kidney Diseases(United States Department of Health & Human ServicesNational Institutes of Health (NIH) - USANIH National Institute of Diabetes & Digestive & Kidney Diseases (NIDDK)); National Center for Research Resources (NCRR)(United States Department of Health & Human ServicesNational Institutes of Health (NIH) - USANIH National Center for Research Resources (NCRR)); National Cancer Institute(United States Department of Health & Human ServicesNational Institutes of Health (NIH) - USANIH National Cancer Institute (NCI)); National Institute of Diabetes and Digestive and Kidney Diseases(United States Department of Health & Human ServicesNational Institutes of Health (NIH) - USANIH National Institute of Diabetes & Digestive & Kidney Diseases (NIDDK)) This work was funded by the American Institute for Cancer Research, grant number 03B043. Additional support was obtained from National Institutes of Health (NIH) Cancer Center Support Grants CA-22453 and CA-46592, the Chemistry Laboratory of the Michigan Diabetes Research and Training Center (MDRTC) and the General Clinical Research Center (GCRC) at the University of Michigan. The Michigan Diabetes Research and Training Center is funded by NIH5P60 DK20572 from the National Institute of Diabetes and Digestive and Kidney Diseases. The GCRC is funded by grant M01-RR000042 from the National Center for Research Resources (NCRR), a component of the NIH. The contents of this article are solely the responsibility of the authors and do not necessarily represent the official views of NCRR or NIH.We thank the women who volunteered to participate in the Mediterranean Eating Study. Barbara Poore helped prepare this manuscript and Jason Blythe provided data and sample management for the study. Heather Sirko, Kristen Mar, and Adrienne Wagener provided technical assistance as part of the Undergraduate Research Opportunity Program. 30 22 26 0 7 AMER DIETETIC ASSOC CHICAGO 120 S RIVERSIDE PLZ, STE 2000, CHICAGO, IL 60606-6995 USA 0002-8223 J AM DIET ASSOC J. Am. Diet. Assoc. DEC 2008 108 12 2059 2065 10.1016/j.jada.2008.09.006 http://dx.doi.org/10.1016/j.jada.2008.09.006 7 Nutrition & Dietetics Science Citation Index Expanded (SCI-EXPANDED) Nutrition & Dietetics 381ES 19027409 Green Accepted 2025-06-24 WOS:000261519000026

J Adamo, V; Ricciardi, GRR; Giuffrida, D; Scandurra, G; Russo, A; Blasi, L; Spadaro, P; Iacono, C; Parra, HJS; Savarino, A; Ferraú, F; Zerilli, F; Verderame, F; Butera, A; Santangelo, C; Franchina, V; Caruso, M Adamo, Vincenzo; Ricciardi, Giuseppina Rosaria Rita; Giuffrida, Dario; Scandurra, Giuseppa; Russo, Antonio; Blasi, Livio; Spadaro, Pietro; Iacono, Carmelo; Soto Parra, Hector J.; Savarino, Antonino; Ferrau, Francesco; Zerilli, Filippo; Verderame, Francesco; Butera, Alfredo; Santangelo, Carlo; Franchina, Veronica; Caruso, Michele

Eribulin mesylate use as third-line therapy in patients with metastatic breast cancer (VESPRY): a prospective, multicentre, observational study THERAPEUTIC ADVANCES IN MEDICAL ONCOLOGY English Article

eribulin; metastatic breast cancer; multicentre; prospective; real world; third line ANTHRACYCLINE; EFFICACY; WOMEN; TAXANE; SITE Background: In real-world practice, eribulin mesylate provides significant survival benefit, with a manageable safety profile in heavily pretreated patients with metastatic breast cancer (MBC). Methods: In this prospective, open-label, multicentre, observational study we evaluated the effectiveness and tolerability of eribulin as third-line treatment in a homogeneous population. The primary endpoints were the safety profile and response in metastatic sites; secondary endpoints included the response in different subtypes, overall response rate (ORR), progression-free survival (PFS) and overall survival (OS). Results: From 2013 to 2016, 118 women were treated in 21 Sicilian institutions; the median age was 58 years (range 29-79), with 69% of patients under 65. The median cycles of eribulin were 5.5 (range 1-26). The most common adverse event was neutropenia (9.3%, 3 cases of grade 3, 4 of grade 4); only 1 case of QT prolongation was reported. Eribulin was effective in controlling metastatic disease in all sites, and it achieved the highest ORR in brain (16%) and liver (14.9%). Median OS was 31.8 months (95% CI 27.9-34.4) and median PFS 5.5 months (95% CI 4.2-6.6). PFS was 5.2 months (95% CI 2.8-8.4) in patients with triple-negative subtype. Median PFS was longer in patients over 65 years (6.1 months, 95% CI 4.4-8.3). In patients who had visceral metastases PFS was 5.5 months (95% CI 95% 3.5-6.6) and OS 33.9 months (95% CI 29.8-40.8). Conclusions: Eribulin as third-line treatment shows an acceptable safety profile and a substantial antitumour activity in the treatment of MBC, even in elderly patients and in those with visceral disease. [Adamo, Vincenzo; Ricciardi, Giuseppina Rosaria Rita; Franchina, Veronica] Univ Messina, Med Oncol Unit AO Papardo, Messina, Italy; [Adamo, Vincenzo; Ricciardi, Giuseppina Rosaria Rita; Franchina, Veronica] Univ Messina, Dept Human Pathol, Messina, Italy; [Adamo, Vincenzo] Univ Messina, Med Oncol, Messina, Italy; [Adamo, Vincenzo] Papardo Hosp, Oncol Hematol Dept, I-98158 Messina, Italy; [Adamo, Vincenzo] Papardo Hosp, Med Oncol Unit, I-98158 Messina, Italy; [Giuffrida, Dario] Mediterranean Inst Oncol, Dept Med Oncol, Viagrande, CT, Italy; [Scandurra, Giuseppa] Osped Emergenze Cannizzaro, Oncol Med, Catania, Italy; [Russo, Antonio] Univ Palermo, Sect Med Oncol, Dept Surg Oncol & Oral Sci, Palermo, Italy; [Blasi, Livio] ARNAS Civ, UOC Oncol Med, Piazza Nicola Leotta, Palermo, Italy; [Spadaro, Pietro] Casa Cura Villa Salus, UO Oncol & Ematol, Messina, Italy; [Iacono, Carmelo] Osped Maria Paterno Arezzo, Dept Med Oncol, Ragusa, Italy; [Soto Parra, Hector J.] Univ Hosp Policlin Vittorio Emanuele, Dept Med Oncol, Catania, Italy; [Savarino, Antonino] Osped Barone Lombardo Canicatti, Unita Operat Oncol, Canicatti, AG, Italy; [Ferrau, Francesco] Osped S Vincenzo, Dept Med Oncol, Taormina, ME, Italy; [Zerilli, Filippo] San Antonio Abate Hosp, Med Oncol, Trapani, Italy; [Verderame, Francesco] AO Osped Riuniti Villa Sofia Cervello, Palermo, Italy; [Butera, Alfredo] Hosp Agrigento, Med Oncol, Agrigento, Italy; [Santangelo, Carlo] PO Umberto I, Contrada Ferrante, Enna, Italy; [Caruso, Michele] Humanitas Ctr Catanese Oncol, Catania, Italy

University of Messina; University of Messina; University of

Messina; Mediterranean Institute of Oncology; University of Palermo; A.R.N.A.S. Ospedali Civico Di Cristina Benfratelli; Civile M.P. Arezzo Hospital; University of Catania; University Catania Hospital; Azienda Ospedaliera Universitaria Policlinico Vittorio Emanuele Presidio Ferraotto   Adamo, V (corresponding author), Univ Messina, Med Oncol Unit AO Papardo, Messina, Italy.; Adamo, V (corresponding author), Univ Messina, Dept Human Pathol, Messina, Italy.; Adamo, V (corresponding author), Univ Messina, Med Oncol, Messina, Italy.; Adamo, V (corresponding author), Papardo Hosp, Oncol Hematol Dept, I-98158 Messina, Italy.; Adamo, V (corresponding author), Papardo Hosp, Med Oncol Unit, I-98158 Messina, Italy. vadamo@unime.it Scandurra, Giuseppa/ABA-1407-2020; Giuffrida, Dario/AAT-2484-2020; Russo, Antonio/JBJ-3134-2023; de Rosa, Francesco/AAC-5935-2022; SOTO PARRA, HECTOR/IZE-2180-2023 Scandurra, Giuseppa/0000-0001-8025-581X; SOTO PARRA, HECTOR/0000-0001-7966-4472 Eisai Eisai(Eisai Co Ltd)   The author(s) disclosed receipt of the following financial support for the research, authorship, and publication of this article: Medical writing assistance was funded by Eisai.   20   10   11   0
4   SAGE PUBLICATIONS LTD LONDON   1 OLIVERS YARD, 55 CITY ROAD, LONDON EC1Y 1SP, ENGLAND   1758-8340 1758-8359
THER ADV MED ONCOL   Ther. Adv. Med. Oncol. DEC   2019 11
1758835919895755
10.1177/1758835919895755
http://dx.doi.org/10.1177/1758835919895755   7
Oncology   Science Citation Index Expanded (SCI-EXPANDED)
Oncology   JX9XP 31903098   Green Published, gold
2025-06-24 WOS:000504080200001
J   Jalili, F; Tehrani, GA; Mirzaahamdi, S
Jalili, Farzaneh; Tehrani, Golnaz Asaadi; Mirzaahamdi, Sina
Evaluation of CAMD1 Gene Promoter Hypermethylation in Human Papillomavirus Positive Cytological Samples of Cervical Cancer MIDDLE EAST JOURNAL OF CANCER
English   Article
Human papillomavirus; Uterine cervical neoplasms; Hypermthylation; EpigenomicsMETHYLATION; EXPRESSION
Background: Cervical cancer (CC) is the second most common type of cancer among women. A key factor in developing the disease is the human papillomavirus (HPV) infection. Aberrant DNA hypermethylation in gene promoter regions is one of the most well-fined epigenetic alterations in tumors. This study aimed to investigate cell adhesion molecule 1 (CADM1), promoter methylation in HPV serological positive samples in the Iranian population.Method: Genomic DNA was extracted from cervical smears from patients and healthy patients acting as a control group for this analytical observational case-control investigation. Reverse dot blotting was performed to first identify the presence of HPV DNA infection. After that, each sample was transformed by being treated with sodium bisulfite, and MSP was then used to determine the CADM1 gene's level of methylation.Results: Among total number of positive HPV samples (n = 52), 63.5% methylated, 23% hemi-methylated, and 13.5% were unmethylated. On the contrary, in the control group (n=38), 11% methylated, 71% hemi-methylated, and 18% were unmethylated ($P < 0.0001$). Furthermore, comparing the methylation status among high, low and high/low patients indicated that methylation was (66.66, 24.24, and 9.09%), (25, 66.66, and 8.33%), and (14.28, 57.14%, and 28.57%), respectively ($P < 0.0001$).Conclusion: The present study confirmed that the

methylation status of CADM1 gene was significantly higher in HPV-positive patients than in patients negative for HPV DNA. Moreover, the CADM1 pattern of the gene was associated with the highrisk subtypes of HPV, not with the low-risk ones. Therefore, CADM1 methylation appeared to be a promising biomarker for future studies. [Jalili, Farzaneh; Tehrani, Golnaz Asaadi; Mirzaahamdi, Sina] Islamic Azad Univ, Dept Genet, Zanjan Branch, Zanjan, Iran Islamic Azad University Tehrani, GA (corresponding author), Islamic Azad Univ, Dept Genet, Zanjan Branch, Zanjan, Iran. golnaz_asaadi@yahoo.com
31 0 0 0 0 SHIRAZ UNIV MEDICAL SCIENCES SHIRAZ NEMAZEE HOSPITAL, SHIRAZ, 71934, IRAN
2008-6709 2008-6687 MIDDLE EAST J CANCER Middle East J. Cancer APR 2023 14 2 250
258 10.30476/mejc.2022.93587.1691
http://dx.doi.org/10.30476/mejc.2022.93587.1691
9 Oncology Emerging Sources Citation Index (ESCI)
Oncology E4ES6 2025-06-24
WOS:000975097200006
J Graziani, V; Scognamiglio, M; Belli, V; Esposito, A; D'Abrosca, B; Chambery, A; Russo, R; Panella, M; Russo, A; Ciardiello, F; Troiani, T; Potenza, N; Fiorentino, A
Graziani, Vittoria; Scognamiglio, Monica; Belli, Valentina; Esposito, Assunta; D'Abrosca, Brigida; Chambery, Angela; Russo, Rosita; Panella, Marta; Russo, Aniello; Ciardiello, Fortunato; Troiani, Teresa; Potenza, Nicoletta; Fiorentino, Antonio Metabolomic approach for a rapid identification of natural products with cytotoxic activity against human colorectal cancer cells SCIENTIFIC REPORTS
English Article
INHIBITS TUMOR-GROWTH; COLON-CANCER; ASTRAGALUS-MEMBRANACEUS; ACQUIRED-RESISTANCE; SAPONINS; CHEMOTHERAPY; CETUXIMAB; L.; DEREPLICATION; METABOLITES The discovery of bioactive compounds from natural sources entails an extremely lengthy process due to the timescale and complexity of traditional methodologies. In our study, we used a rapid NMR based metabolomic approach as tool to identify secondary metabolites with anti-proliferative activity against a panel of human colorectal cancer cell lines with different mutation profiles. For this purpose, fourteen Fabaceae species of Mediterranean vegetation were investigated using a double screening method: H-1 NMR profiling enabled the identification of the main compounds present in the mixtures, whilst parallel biological assays allowed the selection of two plant extracts based on their strong anti-proliferative properties. Using high-resolution 2D NMR spectroscopy, putative active constituents were identified in the mixture and isolated by performing a bio-guided fractionation of the selected plant extracts. As a result, we found two active principles: a cycloartane glycoside and protodioscin derivative. Interestingly, these metabolites displayed a preferential anti-proliferative effect on colon cancer cell lines with an intrinsic resistance to anti-EGFR therapies. Our work provides an NMR-based metabolomic approach as a powerful and efficient tool to discover natural products with anticancer activities circumventing time-consuming procedures.[Graziani, Vittoria; Scognamiglio, Monica; Esposito, Assunta; D'Abrosca, Brigida; Chambery, Angela; Russo, Rosita; Panella, Marta; Russo, Aniello; Potenza, Nicoletta; Fiorentino, Antonio] Univ Campania Luigi Vanvitelli, Dipartimento Sci & Tecnol

Ambientali Biol & Farma, Via Vivaldi 43, I-81100 Caserta, Italy; [Scognamiglio, Monica] Max Planck Inst Chem Ecol Beutenberg Campus, Hans Knoll Str 8, D-07745 Jena, Germany; [Belli, Valentina; Ciardiello, Fortunato; Troiani, Teresa] Univ Campania Luigi Vanvitelli, Dipartimento Internist Clin & Sperimentale Flavia, Via Pansini 5, I-80131 Naples, Italy Universita della Campania Vanvitelli; Universita della Campania Vanvitelli
Potenza, N; Fiorentino, A (corresponding author), Univ Campania Luigi Vanvitelli, Dipartimento Sci & Tecnol Ambientali Biol & Farma, Via Vivaldi 43, I-81100 Caserta, Italy.; Troiani, T (corresponding author), Univ Campania Luigi Vanvitelli, Dipartimento Internist Clin & Sperimentale Flavia, Via Pansini 5, I-80131 Naples, Italy. teresa.troiani@unicampania.it; nicoletta.potenza@unicampania.it; antonio.fiorentino@unicampania.it Scognamiglio, Monica/U-1116-2017; D'Abrosca, Brigida/ABG-7435-2020; Graziani, Vittoria/AAO-2436-2020; Fiorentino, Antonio/W-3531-2018 Scognamiglio, Monica/0000-0001-9614-0929; Graziani, Vittoria/0000-0002-8391-2763; Fiorentino, Antonio/0000-0002-9439-7074; Russo, Rosita/0000-0002-2235-6302; Potenza, Nicoletta/0000-0002-9736-792X; Esposito, Assunta/0000-0003-2937-7373; belli, valentina/0000-0002-7774-3764; Russo, Aniello/0000-0001-5421-3552; D'ABROSCA, Brigida/0000-0002-6556-5462 56 33 33 0 12
NATURE PORTFOLIO BERLIN HEIDELBERGER PLATZ 3, BERLIN, 14197, GERMANY 2045-2322 SCI REP-UK Sci Rep MAR 28 2018 8 5309
10.1038/s41598-018-23704-9
http://dx.doi.org/10.1038/s41598-018-23704-9 11
Multidisciplinary Sciences Science Citation Index Expanded (SCI-EXPANDED) Science & Technology - Other Topics GA7LX
29593231 gold, Green Published 2025-06-24
WOS:000428518900006
J Berc, G Berc, Gordana
Croatian Experience with the Refugee Crisis on the Balkan Route and Possible Implications for Social Work Practice and Education JOURNAL OF HUMAN RIGHTS AND SOCIAL WORK
English Article
Migration; Legislation; Human rights; Social services; Psychosocial support; Social work MENTAL-HEALTH The purpose of this review article is to describe some aspects of the migration crises experience that Croatia as a Member State on the European Union external border went through as a transit country on the Balkan route 2015-2016. In that period of time, migrant movement was the most intensive, when Croatia received more than 600,000 refugees and migrants who migrated mainly from Syria, Afghanistan, Pakistan, and other Middle East countries, escaping from the war devastation. The international and national legal frameworks on refugees' rights' protection are presented as well as EU funds for the refugee crisis and improving migration management. The main focus is on elaboration of organized social services and psychosocial support in Croatian reception centers. Interplay between public and civil sector in providing needed services for the different migrants' needs is explained. Since Croatia in 1990s experienced a strong war devastation and refugee crisis, comparison on similarities and differences basis is described. Migrants' reception-recovery process and aspects of the role of the social work profession are highlighted, as well as possible improvement in social work practice and education based on the

recent migrant crisis experience. [Berc, Gordana] Univ Zagreb, Fac Law, Dept Social Work, Nazorova 51, Zagreb 10000, Croatia
University of Zagreb Berc, G (corresponding author), Univ Zagreb, Fac Law, Dept Social Work, Nazorova 51, Zagreb 10000, Croatia. gordana.berc@gmail.com
56 2 2 1 11 SPRINGER INTERNATIONAL PUBLISHING AG CHAM GEWERBESTRASSE 11, CHAM, CH-6330, SWITZERLAND 2365-1792 J HUM RIGHTS SOC WOR J. Hum. Rights Soc. Work MAR 2019 4 1 SI 63 73 10.1007/s41134-018-0079-y
http://dx.doi.org/10.1007/s41134-018-0079-y 11
Social Work Emerging Sources Citation Index (ESCI) Social Work HS2XH 2025-06-24 WOS:000463725800008
J Caruso, A; Accattatis, FM; Giordano, C; Gelsomino, L; Del Console, P; Fiorita, MF; Gyorffy, B; Bianchi, L; Carleo, A; De Salvo, R; Simoes, BM; Clarke, RB; Memeo, L; Colarossi, C; Aiello, E; Conforti, F; Fuqua, SAW; Todaro, M; Stassi, G; Morelli, C; Sisci, D; Bonofiglio, D; Catalano, S; Andò, S; Barone, I
Caruso, Amanda; Accattatis, Felice Maria; Giordano, Cinzia; Gelsomino, Luca; Del Console, Piercarlo; Fiorita, Maria Francesca; Gyorffy, Balazs; Bianchi, Laura; Carleo, Alfonso; De Salvo, Rossana; Simoes, Bruno M.; Clarke, Robert B.; Memeo, Lorenzo; Colarossi, Cristina; Aiello, Eleonora; Conforti, Francesco; Fuqua, Suzanne A. W.; Todaro, Matilde; Stassi, Giorgio; Morelli, Catia; Sisci, Diego; Bonofiglio, Daniela; Catalano, Stefania; Ando, Sebastiano; Barone, Ines
Adipocyte/Tumor cell crosstalk via IGF-1/TXNIP axis promotes malignancy and endocrine resistance in breast cancer CELL COMMUNICATION AND SIGNALING English Article
Breast Cancer; Endocrine resistance; Adipocytes; Tumor microenvironment; Obesity; IGF-1; TXNIP
ESTROGEN-RECEPTOR-ALPHA; THIOREDOXIN-INTERACTING PROTEIN; GROWTH-FACTOR; TAMOXIFEN RESISTANCE; EXPRESSION; TXNIP; METASTASIS; SURVIVAL Background Despite significant improvements in the outcome of Estrogen Receptor (ER) alpha-positive breast cancer (BC) following the use of endocrine therapies, resistance remains a major challenge. Clinical studies proved that obesity, in addition to promote BC progression, is associated with a reduced efficacy to these treatments, but mechanisms remain unclear. Methods We used co-culture systems followed by validation through an 'ex vivo' model of human mammary obese (Ob) adipocytes and obese endocrine-resistant metastatic Patient-Derived Organoids (PDOs). Transcriptomics with MixOmics-MINT and MetaCore Functional Tools along with lentiviral and pharmacological approaches provide insights into mechanisms. Clinical relevance was investigated using public datasets, transcriptome-based (n = 375), and immunohistochemistry-based (n = 65) evaluations. Results In a model of co-culture, we demonstrated that conditioned media (CM) released by 3T3-L1A adipocytes reduced the sensitivity of parental MCF-7 BC cells to the inhibitory effects of Tamoxifen (Tam) on growth, motility and invasion and significantly increased the proliferative, motile and invasive phenotype of Tam-resistant (TR) BC cells. Transcriptomics identified TXNIP (Thioredoxin-interacting protein), a known tumor suppressor gene, as a network central hub, that was significantly down-regulated in CM-treated MCF-7 and TR cells. Accordingly, TXNIP expression was negatively correlated with Body Mass Index (BMI) in BC patients. Lentiviral TXNIP overexpression and pharmacological induction of TXNIP (i.e.

SAHA) or the blockade of insulin-like growth factor-I (IGF-1) signaling, an obesity hallmark able to affect TXNIP expression, reversed CM-mediated effects. TXNIP down-regulation, proliferation and motility in TR cells were exacerbated by CM derived from Ob 3T3-L1A, and combination of an IGF-1 inhibitor and SAHA abrogated Ob-CM activities. Results were also validated in aromatase inhibitor-resistant BC cells. The effectiveness of IGF-1/TXNIP axis inhibition was confirmed using an 'ex vivo' model of human mammary obese adipocytes and PDO models. Finally, retrospective analyses demonstrated that an IGF-1(high)/TXNIPlow signature was correlated with poorer survival in endocrine-treated BC patients. Conclusions In conclusion, our study sheds new light on adipocyte/BC cell crosstalk, underscoring the potential of targeting IGF-1/TXNIP axis to block this harmful connection, especially in the context of obesity. [Caruso, Amanda; Accattatis, Felice Maria; Giordano, Cinzia; Gelsomino, Luca; Del Console, Piercarlo; Fiorita, Maria Francesca; Morelli, Catia; Sisci, Diego; Bonofiglio, Daniela; Catalano, Stefania; Ando, Sebastiano; Barone, Ines] Univ Calabria, Dept Pharm Hlth & Nutr Sci, Via P Bucci, I-87036 Cosenza, Italy; [Giordano, Cinzia; Gelsomino, Luca; Del Console, Piercarlo; Fiorita, Maria Francesca; Morelli, Catia; Sisci, Diego; Bonofiglio, Daniela; Catalano, Stefania; Ando, Sebastiano; Barone, Ines] Univ Calabria, Ctr Sanitario, Via P Bucci, I-87036 Cosenza, Italy; [Giordano, Cinzia; Catalano, Stefania] AO Annunziata, Clin Lab Unit, I-87100 Cosenza, Italy; [Gyorffy, Balazs] Semmelweis Univ, Dept Bioinformat, H-1094 Budapest, Hungary; [Gyorffy, Balazs] Univ Pecs, Med Sch, Dept Biophys, H-7624 Pecs, Hungary; [Gyorffy, Balazs] HUN REN Res Ctr Nat Sci, Inst Mol Life Sci, Canc Biomarker Res Grp, H-1117 Budapest, Hungary; [Bianchi, Laura; De Salvo, Rossana] Univ Siena, Dept Life Sci, Lab Funct Prote, Siena, Italy; [Carleo, Alfonso] Univ Salerno, Scuola Med Salernitana, Dept Med Surg & Dent, Lab Mol Med & Genom, Baronissi, Italy; [Simoes, Bruno M.; Clarke, Robert B.] Univ Manchester, Fac Biol Med & Hlth, Manchester Acad Hlth Sci Ctr, Manchester Breast Ctr,Div Canc Sci,Sch Med Sci, Manchester, England; [Memeo, Lorenzo; Colarossi, Cristina; Aiello, Eleonora] Mediterranean Inst Oncol, Dept Expt Oncol, Pathol Unit, Catania, Italy; [Conforti, Francesco] Annunziata Hosp, Div Pathol Anat, Cosenza, Italy; [Fuqua, Suzanne A. W.] Baylor Coll Med, Lester & Sue Smith Breast Ctr, 1 Baylor Plaza, Houston, TX USA; [Todaro, Matilde] Univ Palermo, Dept Hlth Promot Sci Internal Med & Med Specialtie, Palermo, Italy; [Stassi, Giorgio] Univ Palermo, Dept Precis Med Med Surg & Crit Care, Palermo, Italy

University of Calabria; University of Calabria; Semmelweis University; University of Pecs; HUN-REN; HUN-REN Research Centre for Natural Sciences; University of Siena; University of Salerno; University of Manchester; Mediterranean Institute of Oncology; Baylor College of Medicine; University of Palermo; University of Palermo Catalano, S; Barone, I (corresponding author), Univ Calabria, Dept Pharm Hlth & Nutr Sci, Via P Bucci, I-87036 Cosenza, Italy.; Catalano, S; Barone, I (corresponding author), Univ Calabria, Ctr Sanitario, Via P Bucci, I-87036 Cosenza, Italy.; Catalano, S (corresponding author), AO Annunziata, Clin Lab Unit, I-87100 Cosenza, Italy. stefania.catalano@unical.it; ines.barone@unical.it Simoes, Bruno/AAB-2582-2019; Bonofiglio, Daniela/AAV-8371-2020; Györffy, Balázs/H-1620-2017; Del Console, Piercarlo/MAI-0694-2025; Accattatis, Felice Maria/JPW-7881-2023

BANDO PRIN 2017 [2017WNKSLR]; PRIN 2022-European

Union's NextGenerationEU initiative under the Italian Ministry of University and Research [202239N8PR, CUP H53D23006360006]; PRIN PNRR 2022-European Union's NextGenerationEU initiative under the Italian Ministry of University and Research, PNRR [P2022YAKJY, CUP H53D23010120001]; European Union's NextGenerationEU initiative under the Italian Ministry of University and Research [2022AA4FTJ, CUP H53D23006420006, PE00000019, M4C2-I1.3, CUP B83D22001050004]; AIRC Investigator Grant (IG) [21414, 30782, 26246]; National Research, Development and Innovation Office, Hungary (PharmaLab) [RRF-2.3.1-21-2022-00015]; NCI [R01CA072038, BCRF-23-055]; NIHR Manchester Biomedical Research Centre [NIHR203308] BANDO PRIN 2017; PRIN 2022-European Union's NextGenerationEU initiative under the Italian Ministry of University and Research; PRIN PNRR 2022-European Union's NextGenerationEU initiative under the Italian Ministry of University and Research, PNRR; European Union's NextGenerationEU initiative under the Italian Ministry of University and Research; AIRC Investigator Grant (IG)(Fondazione AIRC per la ricerca sul cancro); National Research, Development and Innovation Office, Hungary (PharmaLab); NCI(United States Department of Health & Human ServicesNational Institutes of Health (NIH) - USANIH National Cancer Institute (NCI)); NIHR Manchester Biomedical Research Centre(National Institutes of Health Research (NIHR))   This research was funded by BANDO PRIN 2017 #2017WNKSLR, PRIN 2022-European Union's NextGenerationEU initiative under the Italian Ministry of University and Research - M4C2-I1.1 (#202239N8PR, CUP H53D23006360006), and PRIN PNRR 2022-European Union's NextGenerationEU initiative under the Italian Ministry of University and Research as part of the PNRR - M4C2-I1.1 (#P2022YAKJY, CUP H53D23010120001) to I. Barone; PRIN 2022, European Union's NextGenerationEU initiative under the Italian Ministry of University and Research - M4C2-I1.1 (#2022AA4FTJ, CUP H53D23006420006) to Giordano C; AIRC Investigator Grant (IG) #21414 and #30782 to S. Catalano; AIRC Investigator Grant (IG) #26246 to S. Ando; National Research, Development and Innovation Office, Hungary (PharmaLab, RRF-2.3.1-21-2022-00015) to BG; NCI R01CA072038 and BCRF-23-055 to S. Fuqua; European Union's NextGenerationEU initiative under the Italian Ministry of University and Research as part of the PNRR - M4C2-I1.3 Project PE00000019 'HEAL ITALIA' to L. Memeo (CUP B83D22001050004). BM Sim & otilde;es was funded by the NIHR Manchester Biomedical Research Centre (NIHR203308). The views expressed are those of the author and not necessarily those of the NIHR or the Department of Health and Social Care.   69   0   0   0   0   BMC   LONDON
CAMPUS, 4 CRINAN ST, LONDON N1 9XW, ENGLAND   1478-811X
CELL COMMUN SIGNAL   Cell Commun. Signal.   JUN 3 2025
23   1   262
10.1186/s12964-025-02262-4
http://dx.doi.org/10.1186/s12964-025-02262-4   20
Cell Biology   Science Citation Index Expanded (SCI-EXPANDED)   Cell Biology   3JW0S 40462107
2025-06-24 WOS:001502081800001
J   Bavi, PP; Abubaker, JA; Jehan, ZD; Al-Jomah, NA; Siraj, AK; Al-Harbi, SR; Atizado, VL; Abduljabbar, AS; Alhomoud, S; Ashari, LH; Al-Dayel, FH; Uddin, S; Al-Kuraya, KS; Alsanea, NA
Bavi, Prashant P.; Abubaker, Jehad A.; Jehan, Zeenath D.; Al-Jomah, Naif A.; Siraj, Abdul K.; Al-Harbi, Sayer R.; Atizado, Valerie L.; Abduljabbar, Alaa S.; Alhomoud, SamarJ; Ashari, Luai H.; Al-Dayel, Fouad H.; Uddin, Shahab; Al-Kuraya,

Khawla S.; Alsanea, Nasser A.		Colorectal carcinomas from Middle East - Molecular and tissue microarray analysis of genomic instability pathwaysSAUDI MEDICAL JOURNAL
English	Article
LEVEL MICROSATELLITE INSTABILITY; REVISED BETHESDA GUIDELINES; MISMATCH REPAIR; LYNCH-SYNDROME; CANCER; EXPRESSION; FREQUENCY; MUTATION; PROTEIN; HNPCC	Objectives: To evaluate the overall incidence of microsatellite instability (MSI), hereditary non polyposis colorectal cancer, and tumor supressor gene (TP53) mutations in Saudi colorectal carcinomas. Methods: We studied the MSI pathway in Saudi colorectal cancers (CRC) from 179 unselected patients using 2 methods: MSI by polymerase chain reaction, and immunohistochemistry detection of mutL homologs 1 and mutS homologs 2 proteins. The TP53 mutations were studied by sequencing exons 5, 6, 7, and 8. Results: Of the 150 colorectal carcinomas analyzed for MSI, 16% of the tumors showed high level instability (MSI-H), 19.3% had low-level instability (MSI-L) and the remaining 64% tumors were stable. Survival of the MSI-H group was better as compared to the MSI-L or microsatellite stable group (p=0.0217). In the MSI-H group, 48% were familial MSI tumors, which could be attributable to the high incidence of consanguinity in the Saudi population. The TP53 mutations were found in 24% of the cases studied. Conclusions: A high proportion of familial MSI cases and a lower incidence of TP53 mutations are some of the hallmarks of the Saudi colorectal carcinomas, which need to be explored further.	[Bavi, Prashant P.; Abubaker, Jehad A.; Jehan, Zeenath D.; Al-Jomah, Naif A.; Siraj, Abdul K.; Al-Harbi, Sayer R.; Atizado, Valerie L.; Uddin, Shahab; Al-Kuraya, Khawla S.] King Faisal Specialist Hosp & Res Ctr, King Fahad Natl Ctr Children Canc& Res, Res Ctr, Dept Human Canc Genom Res, Riyadh 11211, Saudi Arabia; [Abduljabbar, Alaa S.; Alhomoud, SamarJ; Ashari, Luai H.; Alsanea, Nasser A.] King Faisal Specialist Hosp & Res Ctr, Res Ctr, Colorectal Unit, Riyadh 11211, Saudi Arabia; [Al-Dayel, Fouad H.] King Faisal Specialist Hosp & Res Ctr, Dept Surg, Riyadh 11211, Saudi Arabia	King Faisal Specialist Hospital & Research Center; King Faisal Specialist Hospital & Research Center; King Faisal Specialist Hospital & Research Center	Al-Kuraya, KS (corresponding author), King Faisal Specialist Hosp & Res Ctr, King Fahad Natl Ctr Children Canc& Res, Res Ctr, Dept Human Canc Genom Res, MBC 98-16,POB 3354, Riyadh 11211, Saudi Arabia.	kkuraya@kfshrc.edu.sa	Aldayel, Fouad/KHT-6847-2024; Siraj, Abdul/IQW-1179-2023; Binjumah, Naif/JCE-8161-2023; Alsanea, Nasser/J-1565-2016; Alkuraya, Khawla/AFQ-7946-2022; Uddin, Shahab/AFH-8541-2022; BAVI, PRASHANT/B-2123-2009	Alsanea, Nasser/0000-0002-6336-5942; Al Dayel, Fouad/0000-0001-6175-9051; Alkuraya, Khawla/0000-0002-4126-3419; Uddin, Shahab/0000-0003-1886-6710; BAVI, PRASHANT/0000-0002-7711-2911; Abubaker, Jehad/0000-0003-0681-7305	31	9	10
0	2	SAUDI MED J	RIYADH	ARMED FORCES HOSPITAL, PO BOX 7897,, RIYADH 11159, SAUDI ARABIA	0379-5284
SAUDI MED J	Saudi Med. J.	JAN	2008	29	1
75	80		6
Medicine, General & Internal	Science Citation Index Expanded (SCI-EXPANDED)	General & Internal Medicine	257IE	18176677
2025-06-24	WOS:000252791600012
J	Hillyer, LM; Hucik, B; Baracuhy, EM; Lin, Z; Muller, WJ; Robinson, LE; Ma, DWL		Hillyer, Lyn M.; Hucik, Barbora; Baracuhy, Enzo M.; Lin, Zhen; Muller, William J.;

Robinson, Lindsay E.; Ma, David W. L. Her-2 Breast Cancer Outcomes Are Mitigated by Consuming n-3 Polyunsaturated, Saturated, and Monounsaturated Fatty Acids Compared to n-6 Polyunsaturated Fatty Acids NUTRIENTS English Article fatty acid; breast cancer; n-3 PUFA; n-6 PUFA; MUFA; SFA MAMMARY-GLAND DEVELOPMENT; DOCOSAHEXAENOIC ACID; CARDIOVASCULAR-DISEASE; MEDITERRANEAN DIET; LIFE-STYLE; OLEIC-ACID; OLIVE OIL; FISH-OIL; RISK; CELLS Lifestyle habits, such as the consumption of a healthy diet, may prevent up to 30-50% of breast cancer (BC) cases. Dietary fats are of specific interest, as research provides strong evidence regarding the association of dietary fats and BC. However, there is limited research on the role of different types of fats including polyunsaturated (PUFA), monounsaturated (MUFA), and saturated fatty acids (SFA). The objective of this study was to determine the effects of lifelong exposure to various dietary fats on mammary tumour development over a 20-week period. Female heterozygous MMTV-neu (ndl) YD5 mouse models were fed five maternal diets containing (1) 10% safflower oil (n-6 PUFA, control), (2) 3% menhaden oil + 7% safflower oil (marine n-3 PUFA, control), (3) 3% flaxseed + 7% safflower oil (plant-based n-3 PUFA), (4) 10% olive oil (MUFA), or (5) 10% lard (SFA). The primary measures, tumour latency, volume, and multiplicity differed by diet treatment in the following general order, n-6 PUFA > plant n-3 PUFA, SFA, MUFA > marine n-3 PUFA. Overall, these findings show that the quality of the diet plays a significant role influencing mammary tumour outcomes. [Hillyer, Lyn M.; Hucik, Barbora; Baracuhy, Enzo M.; Lin, Zhen; Robinson, Lindsay E.; Ma, David W. L.] Univ Guelph, Dept Human Hlth & Nutr Sci, Guelph, ON N1G 2W1, Canada; [Muller, William J.] McGill Univ, Rosalind & Morris Goodman Canc Ctr, Dept Biochem, Montreal, PQ H3A 1A3, Canada University of Guelph; McGill University Robinson, LE; Ma, DWL (corresponding author), Univ Guelph, Dept Human Hlth & Nutr Sci, Guelph, ON N1G 2W1, Canada. lhillyer@uoguelph.ca; bhucik@uoguelph.ca; ebaracuh@uoguelph.ca; zlin01@mail.uoguelph.ca; william.muller@mcgill.ca; lrobinso@uoguelph.ca; davidma@uoguelph.ca Ma, David/0000-0002-1165-5972 Cancer Research Society Cancer Research Society This research was funded by a grant from the Cancer Research Society. 63 6 8 0 8 MDPI BASEL ST ALBAN-ANLAGE 66, CH-4052 BASEL, SWITZERLAND 2072-6643 NUTRIENTS Nutrients DEC 2020 12 12 3901 10.3390/nu12123901 http://dx.doi.org/10.3390/nu12123901 15 Nutrition & Dietetics Science Citation Index Expanded (SCI-EXPANDED) Nutrition & Dietetics PK3VV 33419361 Green Published, gold 2025-06-24 WOS:000602377900001

J Aulisa, G; Binda, C; Padua, E; Pratesi, A; Bellia, A; Bellia, C; Lombardo, M Aulisa, Giovanni; Binda, Claudio; Padua, Elvira; Pratesi, Antonio; Bellia, Alfonso; Bellia, Chiara; Lombardo, Mauro If we eat soy, do we keep the beneficial effects of the Mediterranean diet? NUTRITION & FOOD SCIENCE English Article Soy; Diet; Mediterranean; Soya; Health BREAST-CANCER; HEALTHY-ADULTS; CONSUMPTION; PROTEIN; FOODS; RISK; SUPPLEMENTATION; ADOLESCENCE; ISOFLAVONES; DISORDERS Purpose This study aims to evaluate if soya consumption can compromise or positively influence the effects of the Mediterranean diet (MD).

Design/methodology/approach A full literature review has been conducted as part of a proposal of a new point of view on the consumption of soya and its derivatives in areas where until a few decades ago this type of food did not exist at all. Findings There does not seem to be any contraindications for soy systematic use, therefore, excluding historical-geographical reasons, soya could be included in an MD without altering the benefits associated with it. Practical implications - Soya is not advised as a typical food in the MD, but promoting its use could probably contribute to increase the variety of the diet and likely consolidates the positive health benefits characteristic of MD. Originality/value To the best of the authors' knowledge, this review is one of the first to evaluate soybean consumption within the MD. [Aulisa, Giovanni; Binda, Claudio; Padua, Elvira; Bellia, Alfonso; Lombardo, Mauro] San Raffaele Open Univ, Dept Human Sci & Promot Qual Life, Rome, Italy; [Pratesi, Antonio] ULSS 2 Marca Trevigiana, MMG Dept, Treviso, Italy; [Bellia, Alfonso] Univ Roma Tor Vergata, Dept Syst Med, Rome, Italy; [Bellia, Chiara] Univ Palermo, Dept Biomed Neurosci & Adv Diagnost, Palermo, Italy ULSS 2 Marca TV; Ospedale Ca' Foncello Treviso; University of Rome Tor Vergata; University of Palermo Lombardo, M (corresponding author), San Raffaele Open Univ, Dept Human Sci & Promot Qual Life, Rome, Italy. aulisadietista@gmail.com; c.binda@libero.it; elvira.padua@uniroma5.it; pratesiantonio@gmail.com; bellia@med.uniroma2.it; chiara.bellia@unipa.it; mauro.lombardo@uniroma5.it Padua, Elvira/AAO-3237-2020; Bellia, Chiara/AAO-3725-2020; bellia, alfonso/AIA-9860-2022; Lombardo, Mauro/F-6133-2019 Lombardo, Mauro/0000-0001-7509-5487 48 5 5 0 3 EMERALD GROUP PUBLISHING LTDBINGLEY HOWARD HOUSE, WAGON LANE, BINGLEY BD16 1WA, W YORKSHIRE, ENGLAND 0034-6659 1758-6917 NUTR FOOD SCI Nutr. Food Sci. OCT 27 2020 50 6 1099 1108 10.1108/NFS-10-2019-0322 http://dx.doi.org/10.1108/NFS-10-2019-0322 FEB 2020 10 Food Science & Technology Emerging Sources Citation Index (ESCI) Food Science & Technology OM9MS 2025-06-24 WOS:000515186800001
J Ioannidis, GS; Goumenakis, M; Stefanis, I; Karantanas, A; Marias, K Ioannidis, Georgios S.; Goumenakis, Michalis; Stefanis, Ioannis; Karantanas, Apostolos; Marias, Kostas Quantification and Classification of Contrast Enhanced Ultrasound Breast Cancer Data: A Preliminary Study DIAGNOSTICS English Article perfusion; models; breast carcinoma; contrast enhanced ultrasonography; prognostic factors; quantitative analysis ULTRASONOGRAPHY; DIFFERENTIATION; DIAGNOSIS; FEATURES; CRITERIA; LESIONS; BENIGN; WOMEN; US This study aimed to investigate which of the two frequently adopted perfusion models better describes the contrast enhanced ultrasound (CEUS) perfusion signal in order to produce meaningful imaging markers with the goal of developing a machine-learning model that can classify perfusion curves as benign or malignant in breast cancer data. Twenty-five patients with high suspicion of breast cancer were analyzed with exponentially modified Gaussian (EMG) and gamma variate functions (GVF). The adjusted R-2 metric was the criterion for assessing model performance. Various classifiers were trained on the quantified perfusion curves in order to classify the curves

as benign or malignant on a voxel basis. Sensitivity, specificity, geometric mean, and AUROC were the validation metrics. The best quantification model was EMG with an adjusted R-2 of 0.60 +/- 0.26 compared to 0.56 +/- 0.25 for GVF. Logistic regression was the classifier with the highest performance (sensitivity, specificity, G(mean), and AUROC = 89.2 +/- 10.7, 70.0 +/- 18.5, 77.1 +/- 8.6, and 91.0 +/- 6.6, respectively). This classification method obtained similar results that are consistent with the current literature. Breast cancer patients can benefit from early detection and characterization prior to biopsy. [Ioannidis, Georgios S.; Goumenakis, Michalis; Stefanis, Ioannis; Karantanas, Apostolos; Marias, Kostas] Fdn Res & Technol Hellas FORTH, Computat BioMed Lab CBML, Iraklion 70013, Greece; [Goumenakis, Michalis; Karantanas, Apostolos] Univ Crete, Med Sch, Dept Radiol, Iraklion 71003, Greece; [Stefanis, Ioannis; Marias, Kostas] Hellen Mediterranean Univ, Dept Elect & Comp Engn, Iraklion 71410, Greece; [Karantanas, Apostolos] Univ Hosp, Dept Med Imaging, Iraklion 71003, Greece University of Crete; Hellenic Mediterranean University; University of Patras Ioannidis, GS (corresponding author), Fdn Res & Technol Hellas FORTH, Computat BioMed Lab CBML, Iraklion 70013, Greece. grs.ioannidis@gmail.com; mgoumenakis@gmail.com; stefanis@ics.forth.gr; karantanas@med.uoc.gr; kmarias@ics.forth.gr Marias, Kostas/AAM-2330-2021 Ioannidis, Georgios S./0000-0002-8139-5790; Goumenakis, Michail/0000-0001-6001-7865; Marias, Kostas/0000-0003-3783-5223 Stavros Niarchos Foundation Stavros Niarchos Foundation G.S.I. and I.S. acknowledge the support by the Stavros Niarchos Foundation within the framework of the project ARCHERS ("Advancing Young Researchers' Human Capital in Cutting Edge Technologies in the Preservation of Cultural Heritage and the Tackling of Societal Challenges"). 49 7 7 1 11 MDPI BASEL ST ALBAN-ANLAGE 66, CH-4052 BASEL, SWITZERLAND 2075-4418 DIAGNOSTICS Diagnostics FEB 2022 12 2 425
10.3390/diagnostics12020425
http://dx.doi.org/10.3390/diagnostics12020425
11 Medicine, General & Internal Science Citation Index Expanded (SCI-EXPANDED) General & Internal Medicine ZM2UO
35204514 Green Published, gold 2025-06-24
WOS:000764219100001
J Whalen, KA; Judd, S; McCullough, ML; Flanders, WD; Hartman, TJ; Bostick, RM Whalen, Kristine A.; Judd, Suzanne; McCullough, Marjorie L.; Flanders, W. Dana; Hartman, Terryl J.; Bostick, Robord M. Paleolithic and Mediterranean Diet Pattern Scores Are Inversely Associated with All-Cause and Cause-Specific Mortality in Adults JOURNAL OF NUTRITION English Article
Paleolithic diet; Mediterranean diet; diet patterns; cohort study; mortality CARDIOVASCULAR RISK-FACTORS; CORONARY-HEART-DISEASE; CANCER-RISK; RACIAL-DIFFERENCES; MYOCARDIAL-INFARCTION; COLORECTAL-CANCER; PHYSICAL-ACTIVITY; MEAT CONSUMPTION; NUT CONSUMPTION; BREAST-CANCER Background: Poor diet quality is associated with a higher risk of many chronic diseases that are among the leading causes of death in the United States. It has been hypothesized that evolutionary discordance may account for some of the higher incidence and mortality from these diseases. Objective: We investigated associations of 2 diet pattern scores, the Paleolithic and the Mediterranean, with all-

cause and cause-specific mortality in the REGARDS (REasons for Geographic and Racial Differences in Stroke) study, a longitudinal cohort of black and white men and women >= 45 y of age. Methods: Participants completed questionnaires, including a Block food-frequency questionnaire (FFQ), at baseline and were contacted every 6 mo to determine their health status. Of the analytic cohort (n = 21,423), a total of 2513 participants died during a median follow-up of 6.25 y. We created diet scores from FFQ responses and assessed their associations with mortality using multivariable Cox proportional hazards regression models adjusting for major risk factors. Results: For those in the highest relative to the lowest quintiles of the Paleolithic and Mediterranean diet scores, the multivariable adjusted HRs for all-cause mortality were, respectively, 0.77 (95% CI: 0.67, 0.89; P-trend < 0.01) and 0.63 (95% CI: 0.54, 0.73; P-trend < 0.01). The corresponding HRs for all-cancer mortality were 0.72 (95% CI: 0.55, 0.95; P-trend = 0.03) and 0.64 (95% CI: 0.48, 0.84; P-trend = 0.01), and for all-cardiovascular disease mortality they were 0.78 (95% CI: 0.61, 1.00; P-trend = 0.06) and HR: 0.68 (95% CI: 0.53, 0.88; P-trend = 0.01). Conclusions: Findings from this biracial prospective study suggest that diets closer to Paleolithic or Mediterranean diet patterns may be inversely associated with all-cause and cause-specific mortality. [Whalen, Kristine A.; Flanders, W. Dana; Hartman, Terryl J.; Bostick, Roberd M.] Emory Univ, Rollins Sch Publ Hlth, Dept Epidemiol, Atlanta, GA 30322 USA; [Flanders, W. Dana] Emory Univ, Rollins Sch Publ Hlth, Dept Biostat & Bioinformat, Atlanta, GA 30322 USA; [Judd, Suzanne] Univ Alabama Birmingham, Dept Biostat, Birmingham, AL 35294 USA; [McCullough, Marjorie L.] Amer Canc Soc, Epidemiol Res Program, Atlanta, GA 30329 USA; [Flanders, W. Dana; Hartman, Terryl J.; Bostick, Roberd M.] Emory Univ, Winship Canc Inst, Atlanta, GA 30322 USAEmory University; Rollins School Public Health; Emory University; Rollins School Public Health; University of Alabama System; University of Alabama Birmingham; American Cancer Society; Emory University Bostick, RM (corresponding author), Emory Univ, Rollins Sch Publ Hlth, Dept Epidemiol, Atlanta, GA 30322 USA.; Bostick, RM (corresponding author), Emory Univ, Winship Canc Inst, Atlanta, GA 30322 USA. rmbosti@emory.edu McCullough, Marjorie/0000-0003-3025-6341; Whalen, Kristine/0000-0003-4418-6233

National Institute of Neurological Disorders and Stroke, NIH [U01 NS041588]; Department of Health and Human Services; Franklin Foundation National Institute of Neurological Disorders and Stroke, NIH(United States Department of Health & Human ServicesNational Institutes of Health (NIH) - USANIH National Institute of Neurological Disorders & Stroke (NINDS)); Department of Health and Human Services; Franklin Foundation Supported by cooperative agreement U01 NS041588 from the National Institute of Neurological Disorders and Stroke, NIH, and Department of Health and Human Services. Additional support was provided by the Franklin Foundation. 78 65 68 0 31 OXFORD UNIV PRESS OXFORD GREAT CLARENDON ST, OXFORD OX2 6DP, ENGLAND

0022-3166 1541-6100 J NUTR J. Nutr. APR 2017

147 4 612 620

10.3945/jn.116.241919

http://dx.doi.org/10.3945/jn.116.241919 9

Nutrition & Dietetics Science Citation Index Expanded (SCI-EXPANDED) Nutrition & Dietetics EV7VV 28179490 Bronze, Green Published 2025-06-24 WOS:000401990600018

J	Hadjisavvas, A; Charalambous, E; Adamou, A; Christodoulou, CG; Kyriacou, K			Hadjisavvas, Andreas; Charalambous, Elpida; Adamou, Adamos; Christodoulou, Christina G.; Kyriacou, Kyriacos		BRCA2 Germline Mutations in Cypriot Patients With Familial Breast/Ovarian Cancer	HUMAN MUTATION		English	Article
	BRCA2; germline mutations; breast cancer; Cyprus
Germline mutations in the BRCA2 gene have been shown to be associated with familial female and male breast cancer. Mutations occur throughout the entire coding region of the gene, and there is considerable ethnic and geographical diversity in the deleterious mutations detected in different populations. No data exist on the role of the BRCA2 gene in the Cypriot population. In this study we present the results of characterizing mutations in the BRCA2 gene, in 26 Cypriot families with multiple cases of breast / ovarian cancer. The entire coding region, including splice sites, of BRCA2 were sequenced using cycle sequencing. In total 29 BRCA2 variants were detected which include 3 truncating mutations, 8 missense mutations, 6 polymorphisms and 12 intronic variants. The 3 truncating mutations are frameshift mutation 8984delG (exon 22), and two nonsense mutations, namely C1913X (exon 11) which is a novel mutation, and K3326X (exon 27). It is of interest that frameshift mutation 8984delG was the most frequent, since it was detected in 5 patients from three different families. Among the 6 polymorphisms detected, polymorphism T77T is novel and similarly 4 of the 12 intronic variants were also novel, namely IVS1+8G>A, IVS1-96insA, IVS4+36A>G and IVS11-51G>T. These results show that deleterious BRCA2 mutations, occur at the same frequency, about 20%, in Cypriot families, as that recorded in other European populations. We conclude that the BRCA2 gene plays a significant role in the familial breast cancer phenotype in the Cypriot population. (C) 2003 Wiley-Liss, Inc.	[Hadjisavvas, Andreas; Charalambous, Elpida; Kyriacou, Kyriacos] Cyprus Inst Neurol & Genet, Dept Electron Microscopy & Mol Pathol, POB 23462, CY-1683 Nicosia, Cyprus; [Adamou, Adamos] Bank Cyprus Oncol Ctr, Nicosia, Cyprus; [Christodoulou, Christina G.] Cyprus Inst Neurol & Genet, Dept Mol Virol, Nicosia, Cyprus	Cyprus Institute of Neurology & Genetics; Cyprus Institute of Neurology & Genetics
Kyriacou, K (corresponding author), Cyprus Inst Neurol & Genet, Dept Electron Microscopy & Mol Pathol, POB 23462, CY-1683 Nicosia, Cyprus. kyriacos@.cing.ac.cy	Cyprus TELETHON; Middle East Cancer Consortium [MECC, 970014]; United Nations Development Programme; USA through a UNOPS grant	Cyprus TELETHON; Middle East Cancer Consortium; United Nations Development Programme; USA through a UNOPS grant	Grant sponsor: Cyprus TELETHON; Grant sponsor: the Middle East Cancer Consortium; Grant number: MECC, 970014; Grant sponsor: the United Nations Development Programme and the people of the USA for sponsoring this study, through a UNOPS grant	19	16	17	0	0
WILEY-BLACKWELL	HOBOKEN	111 RIVER ST, HOBOKEN 07030-5774, NJ USA	1059-7794	1098-1004		HUM MUTAT	Hum. Mutat.	FEB	2003	21	2
	10.1002/humu.9110
http://dx.doi.org/10.1002/humu.9110		5
Genetics & Heredity	Science Citation Index Expanded (SCI-EXPANDED)	Genetics & Heredity	V45NU	12552570	gold
2025-06-24	WOS:000209824100004

J Hann, M; Sibbald, B; Young, R Hann, Mark; Sibbald, Bonnie; Young, Ruth Workforce participation among international medical graduates in the National Health Service of England: a retrospective longitudinal study HUMAN RESOURCES FOR HEALTH English Article MIGRATION; PHYSICIANS

Background: Balancing medical workforce supply with demand requires good information about factors affecting retention. Overseas qualified doctors comprise 30% of the National Health Service (NHS) workforce in England yet little is known about the impact of country of qualification on length of stay. We aimed to address this need. Methods: Using NHS annual census data, we calculated the duration of 'episodes of work' for doctors entering the workforce between 1992 and 2003. Survival analysis was used to examine variations in retention by country of qualification. The extent to which differences in retention could be explained by differences in doctors' age, sex and medical specialty was examined by logistic regression. Results: Countries supplying doctors to the NHS could be divided into those with better or worse long-term retention than domestically trained doctors. Countries in the former category were generally located in the Middle East, non-European Economic Area Europe, Northern Africa and Asia, and tended to be poorer with fewer doctors per head of population, but stronger economic growth. A doctor's age and medical specialty, but not sex, influenced patterns of retention. Conclusion: Adjusting workforce participation by country of qualification can improve estimates of the number of medical school places needed to balance supply with demand. Developing countries undergoing strong economic growth are likely to be the most important suppliers of long stay medical migrants. [Hann, Mark; Sibbald, Bonnie] Univ Manchester, Natl Primary Care Res & Dev Ctr, Manchester M13 9PL, Lancs, England; [Young, Ruth] Kings Coll London, Sch Nursing & Midwifery, London SE1 9NH, England

University of Manchester; University of London; King's College London Hann, M (corresponding author), Univ Manchester, Natl Primary Care Res & Dev Ctr, 5th Floor Williamson Bldg,Oxford Rd, Manchester M13 9PL, Lancs, England.

mark.hann@manchester.ac.uk; bonnie.sibbald@manchester.ac.uk; ruth.young@kcl.ac.uk 13 10 11 0 7 BMC LONDON CAMPUS, 4 CRINAN ST, LONDON N1 9XW, ENGLAND 1478-4491 HUM RESOUR HEALTH Hum. Resour. Health MAY 30 2008 6 9 10.1186/1478-4491-6-9 http://dx.doi.org/10.1186/1478-4491-6-9 7 Health Policy & Services; Industrial Relations & Labor Social Science Citation Index (SSCI) Health Care Sciences & Services; Business & Economics 432FN 18513401 Green Published, gold 2025-06-24 WOS:000265119000001

J Atmaca, H; Özkan, AN; Zora, M Atmaca, Harika; Ozkan, Ayse Nur; Zora, Metin Novel ferrocenyl pyrazoles inhibit breast cancer cell viability via induction of apoptosis and inhibition of PI3K/Akt and ERK1/2 signaling CHEMICO-BIOLOGICAL INTERACTIONS English Article Ferrocene; Pyrazole; Cell viability; Apoptosis; Necrosis; PI3K/Akt; ERK1/2 BIOLOGICAL EVALUATION; DERIVATIVES; POTENT; STATISTICS; LIGANDS; SERIES; A549 Despite the advances in early detection and targeted therapies, chemotherapy is still of vital importance in

breast cancer treatment. However, development of drug resistance and serious side effects limits their usage. Thus, there is an urgent need for safer and more effective agents against breast cancer. We have previously described the synthesis of a number of pyrazole derivatives, and in the current study, we have investigated the effects of two different ferrocenyl pyrazole (FP) derivates, 5-ferrocenyl-1-phenyl-1H-pyrazole (FP-Ph) and 5-ferrocenyl-IH-pyrazole (FP-H), on breast cancer cells. First, we investigated the effects of both FPs on cell viability and induction of cell death in breast cancer cells and benign MCF-10A cells by XTT and DNA fragmentation assays, respectively. Morphological changes in human breast cancer cells after FPs treatment were detected by both phase contrast microscope and atomic force microscopy (AFM). Then, we tested whether FPs exert their cytotoxic effect through inhibiting PI3K/Akt and/or ERK1/ 2 signaling pathways by using specific inhibitors. Both FPs induced cytotoxicity in a time and concentration-dependent manner in breast cancer cells; however, MCF-10A benign breast epithelial cells were much less susceptible to the cytotoxic effect of both FPs. FPs inhibited both PI3K/Akt and ERK 1/2 signaling pathways in breast cancer cells. The ultra structure images of MCF-7 cells by AFM showed that the cell surface was smooth in untreated cells, but it was rough with protrusions in treated cells. Both FPs induced apoptotic cell death in MDA-MB-231 cells; however, necrotic cell death was induced in caspase-3 lack MCF-7 cells, which implies that the synthesized FPs may induce apoptosis through caspase-3 dependent mechanism. In summary, these results suggest that FPs might be promising agents for the breast cancer therapy. (C) 2016 Elsevier Ireland Ltd. All rights reserved.
[Atmaca, Harika] Celal Bayar Univ, Fac Sci & Letters, Mol Biol Sect, Dept Biol, TR-45140 Muradiye, Manisa, Turkey; [Ozkan, Ayse Nur] Celal Bayar Univ, Appl Sci Res Ctr, TR-45140 Manisa, Turkey; [Zora, Metin] Middle East Tech Univ, Dept Chem, TR-06800 Ankara, Turkey Celal Bayar University; Celal Bayar University; Middle East Technical University Atmaca, H (corresponding author), Celal Bayar Univ, Fac Sci & Letters, Mol Biol Sect, Dept Biol, TR-45140 Muradiye, Manisa, Turkey.
harika.atmaca@cbu.edu.tr; aysenur.ozkan@cbu.edu.tr; zora@metu.edu.tr Zora, Metin/ABB-7678-2020; atmaca, harika/AAX-8164-2021 Zora, Metin/0000-0001-7764-2288; atmaca, harika/0000-0002-8459-4373; Ozkan, Ayse Nur/0000-0002-9391-288X
39 29 30 1 26 ELSEVIER IRELAND LTD
CLARE ELSEVIER HOUSE, BROOKVALE PLAZA, EAST PARK SHANNON, CO, CLARE, 00000, IRELAND 0009-2797 1872-7786 CHEM-BIOL INTERACT Chem.-Biol. Interact. FEB 1 2017 263
28 35 10.1016/j.cbi.2016.12.010
http://dx.doi.org/10.1016/j.cbi.2016.12.010 8
Biochemistry & Molecular Biology; Pharmacology & Pharmacy; Toxicology Science Citation Index Expanded (SCI-EXPANDED)
Biochemistry & Molecular Biology; Pharmacology & Pharmacy; Toxicology EK6XV 27989600 2025-06-24
WOS:000394070500004
J Sabzalizadeh-Ardabili, S; Alizadeh-Navaei, R; Hedaytizadeh-Omran, A; Janbabaei, G Sabzalizadeh-Ardabili, Saeid; Alizadeh-Navaei, Reza; Hedaytizadeh-Omran, Akbar; Janbabaei, Ghasem Cancer Incidence and Mortality Pattern in Eastern Mediterranean Regional Office Countries and its Association with the Human Development Index CLINICAL CANCER

INVESTIGATION JOURNAL English Article
Cancer; Eastern Mediterranean Regional Office; Human Development Index; incidence; mortality TO-INCIDENCE RATIOS Purpose: Cancer is one of the main causes of death in the Eastern Mediterranean Regional Office (EMRO) region. The aim of this study was to determine the correlation between cancer incidence and mortality with the Human Development Index (HDI) in the EMRO region. Materials and Methods: The incidence and mortality rates of all cancers were obtained from the GLOBOCAN cancer project, and the data about the HDI were obtained from the United Nations Development Program database. The correlation between incidence, mortality rates, and the HDI parameters was analyzed by SPSS software. Results: The highest age-standardized incidence and mortality rates of cancers in the EMRO region were shown for colorectal cancer (10.19 +/- 5.30) and lung cancer (8.92 +/- 4.63), respectively, and the lowest was Kaposi's sarcoma in the incidence (0.20 +/- 0.14) and mortality rate (0.10 +/- 0.09), respectively. The lowest and highest incidence of all cancers in the region pertained to Yemen (80.40) and Lebanon (197.40), respectively. In addition, the lowest and highest mortality from all cancers were in Saudi Arabia (53.90) and Egypt (103.40), respectively. In examining the components of the HDI, gross national income per capita was related to mortality rate of all cancers (P = 0.02). The HDI was related between the incidence rates of colorectal, gallbladder, kidney, lip and oral cavity, multiple myeloma, esophagus, pancreas, and thyroid cancer and the mortality rates of lip and oral cavity, skin melanoma, multiple myeloma, nasopharynx, esophagus, larynx, and pancreas cancers (P < 0.05). Conclusion: The findings of the present study showed that the incidence and mortality rates of some cancers were related to HDI, and the highest correlation was found between the incidence of kidney cancer and the mortality of pancreatic cancer with HDI.

[Sabzalizadeh-Ardabili, Saeid] Mazandaran Univ Med Sci, Student Res Comm, Sari, Iran; [Alizadeh-Navaei, Reza; Hedaytizadeh-Omran, Akbar; Janbabaei, Ghasem] Mazandaran Univ Med Sci, Gastrointestinal Canc Res Ctr, Sari, Iran Mazandaran University of Medical Sciences; Mazandaran University of Medical Sciences Alizadeh-Navaei, R (corresponding author), Mazandaran Univ Med Sci, Gastrointestinal Canc Res Ctr, Sari, Iran.
reza_nava@yahoo.com hedayatizadeh-Omran, Akbar/E-2273-2017; ardabili, Saeid/P-6652-2018; Alizadeh-Navaei, Reza/B-2207-2017 Alizadeh-Navaei, Reza/0000-0003-0580-000X

18 0 0 0 3 Middle Eastern Assoc Cancer Research Montreal 3240 Ave Lacombe, Montreal, Quebec, CANADA 2278-0513 CLIN CANCER INVESTIG Clin. Cancer Investig. J. JAN-FEB 2019 8 1
15 20 10.4103/ccij.ccij_90_18
http://dx.doi.org/10.4103/ccij.ccij_90_18 6
Oncology Emerging Sources Citation Index (ESCI) Oncology
HR9YG 2025-06-24 WOS:000463516900003

J Männistö, S; Harald, K; Härkänen, T; Maukonen, M; Eriksson, JG; Heikkinen, S; Jousilahti, P; Kaartinen, NE; Kanerva, N; Knekt, P; Koskinen, S; Laaksonen, MA; Malila, N; Rissanen, H; Pitkäniemi, J Mannisto, Satu; Harald, Kennet; Harkanen, Tommi; Maukonen, Mirkka; Eriksson, Johan G.; Heikkinen, Sanna; Jousilahti, Pekka; Kaartinen, Niina E.; Kanerva, Noora; Knekt, Paul; Koskinen, Seppo; Laaksonen, Maarit A.; Malila, Nea; Rissanen, Harri; Pitkaniemi, Janne Association

between overall diet quality and postmenopausal breast cancer risk in five Finnish cohort studies SCIENTIFIC REPORTS
English Article
BALTIC SEA DIET; FOOD FREQUENCY QUESTIONNAIRE; HEALTHY NORDIC DIET; MEDITERRANEAN DIET; ADHERENCE; DISEASE; METAANALYSIS; VALIDITY; WOMEN; INDEX There is limited evidence for any dietary factor, except alcohol, in breast cancer (BC) risk. Therefore, studies on a whole diet, using diet quality indices, can broaden our insight. We examined associations of the Nordic Diet (mNDI), Mediterranean diet (mMEDI) and Alternative Healthy Eating Index (mAHEI) with postmenopausal BC risk. Five Finnish cohorts were combined including 6374 postmenopausal women with dietary information. In all, 8-9 dietary components were aggregated in each index, higher total score indicating higher adherence to a healthy diet. Cox proportional hazards regression was used to estimate the combined hazard ratio (HR) and 95% confidence interval (CI) for BC risk. During an average 10-year follow-up period, 274 incident postmenopausal BC cases were diagnosed. In multivariable models, the HR for highest vs. lowest quintile of index was 0.67 (95 %CI 0.48-1.01) for mNDI, 0.88 (0.59-1.30) for mMEDI and 0.89 (0.60-1.32) for mAHEI. In this combined dataset, a borderline preventive finding of high adherence to mNDI on postmenopausal BC risk was found. Of the indices, mNDI was more based on the local food culture than the others. Although a healthy diet has beneficially been related to several chronic diseases, the link with the etiology of postmenopausal BC does not seem to be that obvious. [Mannisto, Satu; Harald, Kennet; Harkanen, Tommi; Maukonen, Mirkka; Eriksson, Johan G.; Jousilahti, Pekka; Kaartinen, Niina E.; Knekt, Paul; Koskinen, Seppo; Laaksonen, Maarit A.; Rissanen, Harri] Finnish Inst Hlth & Welf, POB 30, Helsinki 00271, Finland; [Eriksson, Johan G.] Univ Helsinki, Dept Gen Practice & Primary Hlth Care, Helsinki, Finland; [Eriksson, Johan G.] Helsinki Univ Hosp, Helsinki, Finland; [Eriksson, Johan G.] Folkhalsan Res Ctr, Helsinki, Finland; [Eriksson, Johan G.] Agcy Sci Technol & Res, Singapore Inst Clin Sci, Singapore, Singapore; [Eriksson, Johan G.] Natl Univ Singapore, Yong Loo Lin Sch Med, Dept Obstet & Gynaecol, Human Potential Translat Res Programme, Singapore, Singapore; [Heikkinen, Sanna; Malila, Nea; Pitkaniemi, Janne] Inst Stat & Epidemiol Canc Res, Finnish Canc Registry, Helsinki, Finland; [Laaksonen, Maarit A.] Univ New South Wales, Sch Math & Stat, Sydney, NSW, Australia; [Kanerva, Noora; Pitkaniemi, Janne] Univ Helsinki, Dept Publ Hlth, Helsinki, Finland; [Pitkaniemi, Janne] Univ Tampere, Sch Hlth Sci, Tampere, Finland University of Helsinki; University of Helsinki; Helsinki University Central Hospital; Folkhalsan Research Center; Agency for Science Technology & Research (A*STAR); A*STAR - Singapore Institute for Clinical Sciences (SICS); National University of Singapore; Finnish Cancer Registry; University of New South Wales Sydney; University of Helsinki; Tampere University Männistö, S (corresponding author), Finnish Inst Hlth & Welf, POB 30, Helsinki 00271, Finland. satu.mannisto@thl.fi Maukonen, Mirkka/G-9230-2018; Laaksonen, Maarit/O-3467-2016; Kaartinen, Niina/AAZ-8585-2020; Harkanen, Tommi/G-4866-2019 Kanerva, Noora/0000-0001-6776-9357; Heikkinen, Sanna/0000-0001-9995-5605; Harkanen, Tommi/0000-0002-4577-1808; RISSANEN, HARRI/0009-0007-4203-200X Cancer Foundation Finland; Cancer Institute New South Wales Career Development Fellowship [2019/CDF1022] Cancer Foundation Finland;

Cancer Institute New South Wales Career Development Fellowship
J.P. and S. M. have received research grants from the Cancer Foundation Finland. MAL was supported by the Cancer Institute New South Wales Career Development Fellowship (2019/CDF1022).
45 10 10 0 5 NATURE PORTFOLIO BERLIN
HEIDELBERGER PLATZ 3, BERLIN, 14197, GERMANY 2045-2322
SCI REP-UK Sci Rep AUG 18 2021 11 1
16718 10.1038/s41598-021-95773-2
http://dx.doi.org/10.1038/s41598-021-95773-2 9
Multidisciplinary Sciences Science Citation Index Expanded (SCI-EXPANDED) Science & Technology - Other Topics UC7MM
34408173 Green Published, gold 2025-06-24
WOS:000686708000070
J Souiai, O; Sallemi, A Souiai, Oussama; Sallemi, Ameni Retrospective Phylodynamic and Phylogeographic Analysis of the Human Papillomavirus 16 E6 Gene in the Mediterranean Region BIOINFORMATICS AND BIOLOGY INSIGHTS
English Article
HPV16; E6; phylogeography; evolution; substitution rate
INFECTION; RISK Human papillomavirus 16 (HPV16) is considered to be strongly correlated with the development of cervical cancer. Among the 8 HPV16 genes, the E6 constitutes a remarkable marker to follow the evolutionary history and spatial phylodynamics of HPV16 in the Mediterranean basin. Thus, this work aims to decipher the major evolutionary events and crosstalks in the Mediterranean basin with a focus on Tunisian strains regarding the E6 oncogene. In this study, we first extracted the available and annotated Mediterranean strains of HPV16 E6 gene sequences (n = 155) from the NCBI nucleotide database. These sequences were aligned, edited, and used for the downstream phylogenetic analyses. Finally, a Bayesian Markov Chain Monte Carlo approach was applied to reconstruct the evolutionary history of HPV16 migration. Our results showed that the HPV circulating in Tunisia derived from a Croatian ancestor around the year 1987. This starting point spreads to most European countries to reach northern Africa through the Moroccan gateway in 2004. [Souiai, Oussama; Sallemi, Ameni] Inst Pasteur Tunis, Lab Bioinformat Biomath & Biostat BIMS, Tunis, Tunisia; [Sallemi, Ameni] Inst Super Biotechnol Sidi Thabet, Ariana, Tunisia; [Souiai, Oussama] Inst Pasteur Tunis, Lab Bioinformat Biomath & Biostat BIMS, Tunis 1002, Tunisia Pasteur Network; Universite de Tunis-El-Manar; Institut Pasteur Tunis; Pasteur Network; Universite de Tunis-El-Manar; Institut Pasteur Tunis Souiai, O (corresponding author), Inst Pasteur Tunis, Lab Bioinformat Biomath & Biostat BIMS, Tunis 1002, Tunisia. souiai@gmail.com souiai, oussama/AAD-4314-2022 oussama, souiai/0000-0003-2443-114X
25 0 0 1 1 SAGE PUBLICATIONS LTD
LONDON 1 OLIVERS YARD, 55 CITY ROAD, LONDON EC1Y 1SP, ENGLAND 1177-9322 BIOINFORM BIOL INSIG
Bioinform. Biol. Insights JUN 2023 17
11779322231178598
10.1177/11779322231178598
http://dx.doi.org/10.1177/11779322231178598 5
Biochemical Research MethodsEmerging Sources Citation Index (ESCI) Biochemistry & Molecular Biology I6IG0 37313033
gold, Green Published 2025-06-24
WOS:001003794700001

J Piccitto, G; Avola, M; Panichella, N
Piccitto, Giorgio; Avola, Maurizio; Panichella, Nazareno
Migration, social stratification, and labor market attainment: An analysis of the ethnic penalty in 12 Western European countries INTERNATIONAL JOURNAL OF COMPARATIVE SOCIOLOGY English Article
Inequality; labor market attainment; migration; occupation; stratification OCCUPATIONAL INTEGRATION; ECONOMIC INCORPORATION; RECENT IMMIGRANTS; MOBILITY; SOCIETIES; EDUCATION; MIGRANTS; ORIGINS This article presents a comprehensive investigation into the socioeconomic integration of migrants across 12 Western European countries, considering their likelihood of employment and socioeconomic status. Using the data from the European Social Survey, the study employs linear regression and probit models to achieve two aims: (a) to quantify the penalty for male and female migrants in terms of employment and socioeconomic status attainment; (b) to assess how the ethnic penalty for men and women changes based on their education and social background of origin. Results reveal that male and female migrants face a penalty in most countries under consideration, albeit with varying degrees of magnitude and characteristics. Migrants in Southern European countries exhibit a trade-off between employment and socioeconomic status attainment, while those in Central-Northern Europe experience a double penalty on both outcomes. Moreover, it emerges that the ethnic penalty in labor market attainment is more heterogeneous across migrants with different educational levels than with different social classes of origin: migrants' social background of origin affects to a lesser extent their labor market outcomes, if compared with their human capital. Migrants with high education and social origin suffer the largest penalty, due to hurdles in leveraging their educational qualifications and social position. This pattern is particularly evident in Southern Europe, where the socioeconomic integration of migrant workers is characterized by a leveling-down process, pushing them into the lowest strata of the occupational hierarchy regardless of their education and social background. [Piccitto, Giorgio] Univ Milano Bicocca, Bicocca, Italy; [Avola, Maurizio] Univ Catania, Catania, Italy; [Panichella, Nazareno] Univ Milan, Milan, Italy; [Piccitto, Giorgio] Univ Milano Bicocca, Dept Sociol & Social Res, Piazza Dellateneo Nuovo 1, I-20126 Milan, Italy University of Milano-Bicocca; University of Catania; University of Milan; University of Milano-Bicocca Piccitto, G (corresponding author), Univ Milano Bicocca, Dept Sociol & Social Res, Piazza Dellateneo Nuovo 1, I-20126 Milan, Italy. giorgio.piccitto1@gmail.com Piccitto, Giorgio/ABE-8227-2020; PANICHELLA, NAZARENO/E-7230-2017 AVOLA, Maurizio/0000-0002-0534-6110; PANICHELLA, NAZARENO/0000-0002-7326-6817 67 1 1 10 18 SAGE PUBLICATIONS INC THOUSAND OAKS 2455 TELLER RD, THOUSAND OAKS, CA 91320 USA 0020-7152 1745-2554 INT J COMP SOCIOL
Int. J. Comp. Sociol. APR 2025 66 2
121 139 10.1177/00207152241246166
http://dx.doi.org/10.1177/00207152241246166 APR 2024
19 Sociology Social Science Citation Index (SSCI)
Sociology 1CY3V 2025-06-24
WOS:001206724800001
J Adigüzel, FI; Adigüzel, C; Seyfettinoglu, S; Hürriyetoglu, S; Kazgan, H; Yilmaz, ESS; Yücel, O; Baser, E
Adiguzel, Fikriye Isil; Adiguzel, Cevdet; Seyfettinoglu,

Sevtap; Hurriyetoglu, Serif; Kazgan, Halil; Yilmaz, Esra Selver Saygili; Yucel, Oguz; Baser, Eralp HPV awareness and HPV vaccine acceptance among women who apply to the gynecology outpatient clinics at a tertiary referral hospital in the south Mediterranean region of Turkey MEDICAL JOURNAL OF BAKIRKOY Turkish Article

Human papillomavirus (HPV); HPV vaccine; cervical cancer

HUMAN-PAPILLOMAVIRUS INFECTION; CERVICAL-CANCER; ACCEPTABILITY; PREVENTION; PREDICTORS Objective: To investigate the awareness about human papillomavirus (HPV) and acceptance of HPV vaccine among women who present to gynecology outpatient clinics at a tertiary referral hospital in South Mediterranean region of Turkey. Materials and Methods: A total of 426 women aging between 18 and 65 who applied to gynecology outpatient clinics between January and April 2015 were included in analysis in order to evaluate the awareness and knowledge about HPV infection and vaccination. In the present study, "Participant Knowledge Form" and "HPV Knowledge Evaluation Questionnaire" were used, which were developed by authors after analyzing the relevant literature. Study data were analyzed with SPSS 21 computer software. Descriptive statistics were presented as mean +/- standard deviation, and number (percentage). Results: Among 426 participants that were included in the study, mean age was 32.3 +/- 10.1 years. Two hundred and fifty-eight (60.6%) stated that they have not heard about HPV, whereas 168 (39.4%) stated otherwise. On the other hand, 141 participants (33.1%) heard about HPV vaccine. HPV vaccination was accepted by 182 (42.7%) participants, whereas 244 (57.3%) women did not accept vaccination. Conclusion: In the present study, it was concluded that women who present to gynecology outpatient clinics did not have adequate knowledge about HPV infection and HPV vaccination. For the purposes of public health education, written, verbal and visual educational programs should be implemented at schools, hospitals and internet. [Adiguzel, Fikriye Isil] Adana Kadin Dogum & Cocuk Hastanesi, Kadin Hastaliklari & Dogum Klin, Adana, Turkey; [Adiguzel, Cevdet; Seyfettinoglu, Sevtap; Hurriyetoglu, Serif; Kazgan, Halil; Yilmaz, Esra Selver Saygili; Yucel, Oguz; Baser, Eralp] Adana Numune Egitim & Arastirma Hastanesi, Kadin Hastaliklari & Dogum Klin, Adana, Turkey Adana Numune Training & Research Hospital; Ankara Numune Training & Research Hospital

Adigüzel, FI (corresponding author), Adana Kadin Dogum & Cocuk Hastanesi, Kadin Hastaliklari & Dogum Klin, Adana, Turkey.

aze_isil@hotmail.com Seyfettinoglu, Sevtap/ITU-2752-2023; Adiguzel, Isil/LTE-8228-2024; Adıgüzel, Cevdet/HOF-3274-2023

Adiguzel, Fikriye Isil/0000-0001-6849-2193; adiguzel, cevdet/0000-0002-3003-4573; seyfettinoglu, sevtap/0000-0001-8607-6628 18 2 2 0 4 YERKURE TANITIM & YAYINCILIK HIZMETLERI A S ISTANB CUMHURYET CAD 48-3B, HARBIYE, ISTANB, 34367, TURKEY 1305-9319 1305-9327

MED J BAKIRKOY Med. J. Bakirkoy 2016 12 3 136 139 10.5350/BTDMJB201612306

http://dx.doi.org/10.5350/BTDMJB201612306 4

Medicine, General & InternalEmerging Sources Citation Index (ESCI) General & Internal Medicine EJ4EB

2025-06-24 WOS:000393168200006

J Kenfield, SA; Dupre, N; Richman, EL; Stampfer, MJ; Chan, JM; Giovannucci, EL Kenfield, Stacey A.; Dupre, Natalie; Richman, Erin L.; Stampfer, Meir J.; Chan, June M.;

Giovannucci, Edward L.		Mediterranean Diet and Prostate Cancer Risk and Mortality in the Health Professionals Follow-up Study EUROPEAN UROLOGY		English	Article
				Prostate cancer; Risk; Mortality; Mediterranean diet; Epidemiology	NIH-AARP DIET; EUROPEAN COUNTRIES; BREAST-CANCER; ADHERENCE; PATTERN; CONSUMPTION; DIAGNOSIS; PROGRESSION; CONFORMITY; SURVIVAL Background: Prostate cancer (PCa) mortality rates are lower in the Mediterranean countries compared with northern Europe. Although specific components of the Mediterranean diet (Med-Diet) may influence PCa risk, few studies have assessed the traditional Med-Diet pattern with the risk of incident advanced or lethal PCa or with disease progression among men diagnosed with nonmetastatic PCa. Objective: To determine whether the traditional Med-Diet pattern is associated with risk of incident advanced or lethal PCa and with PCa-specific and overall mortality among men with PCa. Design, setting, and participants: We prospectively followed 47 867 men in the Health Professionals Follow-up Study followed from 1986 to 2010. The case-only analysis included 4538 men diagnosed with nonmetastatic PCa, followed from diagnosis to lethal outcome or to January 2010. Outcome measurements and statistical analysis: We used Cox proportional hazards models to examine traditional and alternative Med-Diet scores in relation to PCa incidence outcomes (advanced and lethal disease). In a case-only survival analysis, we examined postdiagnostic Med-Diet and risk of lethal (metastases or PCa death) and fatal PCa as well as overall mortality among men diagnosed with nonmetastatic disease. Results and limitations: Between 1986 and 2010, 6220 PCa cases were confirmed. The Med-Diet was not associated with risk of advanced or lethal PCa. In the case-only analysis, there was no association between the Med-Diet after diagnosis and risk of lethal or fatal PCa. However, there was a 22% lower risk of overall mortality (hazard ratio: 0.78; 95% confidence interval, 0.67-0.90; p(trend) = 0.0007) among men with greater adherence to the Med-Diet after PCa diagnosis. We found similar associations for the alternative score. Conclusions: A higher Med-Diet score was not associated with risk of advanced PCa or disease progression. Greater adherence to the Med-Diet after diagnosis of nonmetastatic PCa was associated with lower overall mortality. (C) 2013 European Association of Urology. Published by Elsevier B. V. All rights reserved.	[Kenfield, Stacey A.; Chan, June M.] Univ Calif San Francisco, Dept Urol, San Francisco, CA USA; [Kenfield, Stacey A.; Dupre, Natalie; Stampfer, Meir J.; Giovannucci, Edward L.] Harvard Univ, Sch Publ Hlth, Dept Epidemiol, Boston, MA 02115 USA; [Richman, Erin L.; Chan, June M.] Univ Calif San Francisco, Dept Epidemiol & Biostat, San Francisco, CA 94143 USA; [Stampfer, Meir J.; Giovannucci, Edward L.] Brigham & Womens Hosp, Dept Med, Channing Div Network Med, Boston, MA 02115 USA; [Stampfer, Meir J.; Giovannucci, Edward L.] Harvard Univ, Sch Med, Boston, MA USA; [Giovannucci, Edward L.] Harvard Univ, Sch Publ Hlth, Dept Nutr, Boston, MA 02115 USA
	University of California System; University of California San Francisco; Harvard University; Harvard T.H. Chan School of Public Health; University of California System; University of California San Francisco; Harvard University; Harvard University Medical Affiliates; Brigham & Women's Hospital; Harvard University; Harvard Medical School; Harvard University; Harvard T.H. Chan School of Public Health Kenfield, SA (corresponding author), Univ Calif San Francisco, Helen Diller Family Canc Res

Bldg,Room HD389,1450, San Francisco, CA 94158 USA.
KenfieldS@urology.ucsf.edu Giovannucci, Edward/ADE-8028-2022 Dupre, Natalie/0000-0003-4587-0467; Chan, June/0000-0002-7620-569X National Institutes of Health/National Cancer Institute [UM1 CA167552, T32CA009001, R25CA098566, R01CA141298, R01CA133891]; Prostate Cancer Foundation National Institutes of Health/National Cancer Institute(United States Department of Health & Human ServicesNational Institutes of Health (NIH) - USANIH National Cancer Institute (NCI)); Prostate Cancer Foundation This work was supported by grants from the National Institutes of Health/National Cancer Institute (UM1 CA167552, T32CA009001, R25CA098566, R01CA141298, R01CA133891) and the Prostate Cancer Foundation. 33 105 113 0 21
ELSEVIER AMSTERDAM RADARWEG 29, 1043 NX AMSTERDAM, NETHERLANDS 0302-2838 1873-7560 EUR UROL Eur. Urol. MAY
2014 65 5 887 894
10.1016/j.eururo.2013.08.009
http://dx.doi.org/10.1016/j.eururo.2013.08.009
8 Urology & Nephrology Science Citation Index Expanded (SCI-EXPANDED) Urology & Nephrology AD2JE 23962747 Green Accepted, Green Published 2025-06-24
WOS:000333060200011
J Sukkarieh, O; Egede, LE; Bassil, M
Sukkarieh, Ola; Egede, Leonard E.; Bassil, Maya
Relationship between material needs security and clinical outcomes in adults with type 2 diabetes in Lebanon DIABETES RESEARCH AND CLINICAL PRACTICE English Article
Type 2 diabetes; Material Needs Security; Glycemic control; Hemoglobin A1c; Blood pressure; Lebanon SOCIOECONOMIC-STATUS; SOCIAL DETERMINANTS; SELF-EFFICACY; HEALTH; CARE; ASSOCIATION Background: Despite their documented significance in type 2 diabetes (T2DM) management, social determinants of health (SDOHs) including material needs security are poorly studied in the Middle East and North Africa (MENA) region. This study aims to assess the relation between material needs security and clinical outcomes in Lebanese adults with T2DM. Methods: Subjects with T2DM (n = 300) were recruited; demographic and material need variables were collected using self-reported questionnaires. Measured clinical outcomes included Hemoglobin A1c (A1C), systolic (SBP) and diastolic blood pressure (DBP). Regression models were used to examine associations between material needs security and clinical outcomes, controlling for relevant confounding variables. Results: Most of the participants were men, married and with lower education levels. Having higher material needs security and being employed predicted better A1C levels (p < 0.05), whereas male sex was associated with poorer A1C(ss = 0.52, p = 0.03) and DBP(ss = 3.06, p = 0.05). Other predictors of DBP included older age and lack of confidence in filling out medical forms, reflecting lower health literacy. Conclusions: Our study highlights the importance of material needs in achieving optimal T2DM outcomes T2DM in the MENA region. Further research is needed to understand potential pathways/mechanisms and options for effective interventions.
[Sukkarieh, Ola] Lebanese Amer Univ, AliceRamez Chagoury Sch Nursing, Byblos, Lebanon; [Egede, Leonard E.] Med Coll Wisconsin, Dept Med, Div Gen Internal Med, Milwaukee, WI 53226 USA; [Bassil, Maya] Lebanese Amer Univ, Sch Arts & Sci, Dept Nat Sci, Beirut, Lebanon; [Bassil, Maya] Qatar Univ, Coll Hlth Sci, Dept Human

Nutr, QU Hlth, POB 2713, Doha, Qatar Lebanese American University; Medical College of Wisconsin; Lebanese American University; Qatar UniversityBassil, M (corresponding author), Qatar Univ, Coll Hlth Sci, Dept Human Nutr, QU Hlth, POB 2713, Doha, Qatar. bassil.maya@qu.edu.qa Bassil, Maya/AAG-2846-2019 Sukkarieh, Ola/0000-0001-9971-1563
18 3 3 0 1 ELSEVIER IRELAND LTD CLARE ELSEVIER HOUSE, BROOKVALE PLAZA, EAST PARK SHANNON, CO, CLARE, 00000, IRELAND 0168-8227 1872-8227 DIABETES RES CLIN PR Diabetes Res. Clin. Pract. APR 2022 186
109818
10.1016/j.diabres.2022.109818
http://dx.doi.org/10.1016/j.diabres.2022.109818 MAR 2022 4 Endocrinology & Metabolism Science Citation Index Expanded (SCI-EXPANDED) Endocrinology & Metabolism 1S2JP
35247523 2025-06-24 WOS:000803883000014
J Page, KR; Genovese, E; Franchi, M; Cella, S; Fiorini, G; Tlili, R; Salazar, S; Duvoisin, A; Cailhol, J; Jackson, Y
Page, Kathleen R.; Genovese, Eleonora; Franchi, Matteo; Cella, Silvano; Fiorini, Gianfrancesco; Tlili, Rim; Salazar, Sebastian; Duvoisin, Aline; Cailhol, Johann; Jackson, Yves COVID-19 vaccine hesitancy among undocumented migrants during the early phase of the vaccination campaign: a multicentric cross-sectional study BMJ OPEN
English Article COVID-19; Health policy; GENERAL MEDICINE (see Internal Medicine) UNITED-STATES; DEATHS; DISPARITIES; IMMIGRANTS; ETHNICITY; HEALTH; CARE; RACE; AGE Study objectives The marginalisation of undocumented migrants raises concerns about equitable access to COVID-19 vaccination. This study aims to describe migrants' hesitancy about the COVID-19 vaccination during the early phase of the vaccination campaign. Setting This multicentric cross-sectional survey was conducted in health facilities providing care to undocumented migrants in the USA, Switzerland, Italy and France in February-May 2021. Participants Eligibility criteria included age >16 years, being of foreign origin and living without valid residency permit in the country of recruitment. A convenience sample of minimum 100 patients per study site was targeted. Primary and secondary outcome measures Data were collected using an anonymous structured questionnaire. The main outcomes were perceived access to the local COVID-19 vaccination programme and demand for vaccination. Results Altogether, 812 undocumented migrants participated (54.3% Geneva, 17.5% Baltimore, 15.5% Milano and 12.7% Paris). Most (60.9%) were women. The median age was 39 years (interquartile range 1). Participants originated from the Americas (55.9%), Africa (12.7%), Western Pacific (11.2%) Eastern Mediterranean (7.9%), Europe (7.6%) and South-East Asia (4.7%). Overall, 14.1% and 26.2% of participants, respectively, reported prior COVID-19 infection and fear of developing severe COVID-19 infection. Risk factors for severe infection were frequently reported (29.5%). Self-perceived accessibility of COVID-19 vaccination was high (86.4%), yet demand was low (41.1%) correlating with age, comorbidity and views on vaccination which were better for vaccination in general (77.3%) than vaccination against COVID-19 (56.5%). Participants mainly searched for information about vaccination in the traditional and social media. Conclusions We found a mismatch between perceived accessibility and demand for the COVID-19 vaccination. Public health interventions using

different communication modes should build on trust about vaccination in general to tackle undocumented migrants' hesitancy for COVID-19 vaccination with a specific attention to men, younger migrants and those at low clinical risk of severe infection.
[Page, Kathleen R.] Johns Hopkins Univ, Med, Baltimore, MD USA; [Genovese, Eleonora] Univ Milano Bicocca, Dept Stat & Quantitat Methods, Lab Healthcare Res & Pharmacoepidemiol, Milan, Italy; [Franchi, Matteo] Univ Milano Bicocca, Med Stat & Quantitat Methods, Milan, Italy; [Cella, Silvano] Univ Milan, Dept Clin Sci & Community Hlth, Lab Clin Pharmacol & Pharmacoepidemiol, Milan, Italy; [Fiorini, Gianfrancesco] Zucchi Clin Inst, Milan, Italy; [Tlili, Rim; Cailhol, Johann] Hop Avicenne, Infect & Trop Dis Dept, Bobigny, France; [Salazar, Sebastian] Johns Hopkins Univ, Sch Med, Baltimore, MD USA; [Duvoisin, Aline] Univ Geneva, Ctr Interdisciplinary Study Gerontol & Vulnerabil, Geneva, Switzerland; [Cailhol, Johann] Univ Paris 13 Nord, Lab Educ & Prat Sante, Bobigny, France; [Jackson, Yves] Univ Geneva, Dept Community Hlth & Med, Geneva, Switzerland; [Jackson, Yves] Geneva Univ Hosp, Div Primary Care Med, Geneva, Switzerland Johns Hopkins University; University of Milano-Bicocca; University of Milano-Bicocca; University of Milan; Assistance Publique Hopitaux Paris (APHP); Hopital Universitaire Avicenne - APHP; Universite Paris 13; Johns Hopkins University; University of Geneva; University of Geneva; University of Geneva Jackson, Y (corresponding author), Univ Geneva, Dept Community Hlth & Med, Geneva, Switzerland.; Jackson, Y (corresponding author), Geneva Univ Hosp, Div Primary Care Med, Geneva, Switzerland.
yves.jackson@hcuge.ch Fiorini, Gianfrancesco/G-5280-2018; Franchi, Matteo/AAN-5991-2020; Cailhol, Johann/AGL-9501-2022; Jackson, Yves/HMD-4215-2023; GENOVESE, ELEONORA/C-8581-2019
FRANCHI, MATTEO/0000-0001-9620-8057; GENOVESE, ELEONORA/0000-0001-5215-5280; Jackson, Yves/0000-0001-5619-333X
National Institute of Health RADx-UP initiative [R01 DA045556-04S1]; Ministry of Education, University and Research in Italy ('PRIN' 2017) [2017728JPK]; National Institute on Drug Abuse [R01DA045556] Funding Source: NIH RePORTER National Institute of Health RADx-UP initiative; Ministry of Education, University and Research in Italy ('PRIN' 2017); National Institute on Drug Abuse(United States Department of Health & Human ServicesNational Institutes of Health (NIH) - USANIH National Institute on Drug Abuse (NIDA)) This work was in part supported by the National Institute of Health RADx-UP initiative (grant R01 DA045556-04S1) for the activities conducted in the USA and the Ministry of Education, University and Research in Italy ('PRIN' 2017, project 2017728JPK). 54 34 34 1 13 BMJ PUBLISHING GROUP LONDON BRITISH MED ASSOC HOUSE, TAVISTOCK SQUARE, LONDON WC1H 9JR, ENGLAND 2044-6055 BMJ OPEN BMJ Open
MAR 2022 12 3
e056591 10.1136/bmjopen-2021-056591
http://dx.doi.org/10.1136/bmjopen-2021-056591
12 Medicine, General & InternalScience Citation Index Expanded (SCI-EXPANDED) General & Internal Medicine ZW2ND
35301211 Green Published, gold 2025-06-24
WOS:000771053700006
J Hidalgo-Liberona, N; Meroño, T; Zamora-Ros, R; Rabassa, M; Semba, R; Tanaka, T; Bandinelli, S; Ferrucci, L; Andres-Lacueva, C; Cherubini, A Hidalgo-Liberona, Nicole; Merono, Tomas; Zamora-Ros, Raul; Rabassa, Montserrat; Semba,

Richard; Tanaka, Toshiko; Bandinelli, Stefania; Ferrucci, Luigi; Andres-Lacueva, Cristina; Cherubini, Antonio Adherence to the Mediterranean diet assessed by a novel dietary biomarker score and mortality in older adults: the InCHIANTI cohort study BMC MEDICINE English Article Dietary biomarkers; Older adults; Mediterranean diet; Mortality; Polyphenols; Carotenoids; Dietary questionnaires PLASMA CAROTENOIDS; TOCOPHEROL CONCENTRATIONS; MUSCLE STRENGTH; NUTRIENT INTAKE; BREAST-CANCER; FATTY-ACIDS; NUTRITION; ASSOCIATIONS; POLYPHENOLS; RISK Background: Dietary biomarkers may complement dietary intake assessment made by dietary questionnaires. We developed an a-posteriori dietary biomarkers score based on Mediterranean diet food groups and evaluated its association with mortality. Methods: 642 participants (56% female), aged >= 65 years, with complete data on dietary biomarkers were followed during 20 years in the InCHIANTI cohort study (Tuscany, Italy). The main outcomes were all-cause, cardiovascular, and cancer mortality. Dietary biomarkers were selected from literature and from correlation analyses with dietary intakes of Mediterranean diet food groups in the study. The baseline levels of the following dietary biomarkers were chosen: urinary total polyphenols and resveratrol metabolites, and plasma carotenoids, selenium, vitamin B12, linolenic, eicosapentaenoic and docosahexaenoic acids, and the mono-unsaturated/saturated fatty acid ratio. Associations of the Mediterranean diet score using dietary biomarkers and a validated food frequency questionnaire (FFQ) (as tertiles) with mortality were assessed through Cox regression. Results: During the 20-year follow-up [median (Q1-Q3), 14 (8-18) years], and 435 deaths occurred (139 from cardiovascular diseases and 89 from cancer-related causes). In the fully adjusted models, the dietary biomarker-Mediterranean diet score was inversely associated with all-cause (HRT3vs.T1 0.72; 95%CI 056-0.91) and cardiovascular (HRT3vs.T1 0.60; 95%CI 0.38-0.93), but not with cancer mortality. Associations between the FFQ-Mediterranean diet score and mortality were not statistically significant. Conclusions: A greater adherence at baseline to a Mediterranean diet assessed by a dietary biomarker score was associated with a lower risk of mortality in older adults during a 20-year follow-up. The measurement of dietary biomarkers may contribute to guide individualized dietary counseling to older people. [Hidalgo-Liberona, Nicole; Merono, Tomas; Zamora-Ros, Raul; Rabassa, Montserrat; Andres-Lacueva, Cristina] Univ Barcelona, Fac Pharm & Food Sci, Dept Nutr Food Sci & Gastron, Biomarkers & Nutrimetabol Lab, Barcelona, Spain; [Hidalgo-Liberona, Nicole; Merono, Tomas; Andres-Lacueva, Cristina] Inst Salud Carlos III, Ctr Invest Biomed Red Fragilidad & Envejecimiento, Madrid, Spain; [Zamora-Ros, Raul] Catalan Inst Oncol ICO, Bellvitge Biomed Res Inst IDIBELL, Unit Nutr & Canc, Canc Epidemiol Res Programme, Barcelona, Spain; [Semba, Richard] Johns Hopkins Univ, Sch Med, Wilmer Eye Inst, Baltimore, MD 21205 USA; [Tanaka, Toshiko] NIA, Translat Gerontol Branch, NIH, Baltimore, MD 21224 USA; [Bandinelli, Stefania] ASL Toscana Ctr, Geriatr Unit, Florence, Italy; [Ferrucci, Luigi] NIA, Clin Res Branch, NIH, Baltimore, MD USA; [Cherubini, Antonio] IRCCS INRCA, Accettaz Geriatr, Geriatria, Ancona, Italy; [Cherubini, Antonio] IRCCS INRCA, Ctr Ric Invecchiamento, Ancona, Italy University of Barcelona; CIBER - Centro de Investigacion Biomedica en Red; CIBERFES; Instituto de Salud Carlos III;

Institut d'Investigacio Biomedica de Bellvitge (IDIBELL); Institut Catala d'Oncologia; Johns Hopkins University; Johns Hopkins Medicine; National Institutes of Health (NIH) - USA; NIH National Institute on Aging (NIA); National Institutes of Health (NIH) - USA; NIH National Institute on Aging (NIA); IRCCS INRCA; IRCCS INRCA Zamora-Ros, R; Andres-Lacueva, C (corresponding author), Univ Barcelona, Fac Pharm & Food Sci, Dept Nutr Food Sci & Gastron, Biomarkers & Nutrimetabol Lab, Barcelona, Spain.; Andres-Lacueva, C (corresponding author), Inst Salud Carlos III, Ctr Invest Biomed Red Fragilidad & Envejecimiento, Madrid, Spain.; Zamora-Ros, R (corresponding author), Catalan Inst Oncol ICO, Bellvitge Biomed Res Inst IDIBELL, Unit Nutr & Canc, Canc Epidemiol Res Programme, Barcelona, Spain. rzamora@idibell.cat; candres@ub.edu Hidalgo Liberona, Nicole/HMP-7141-2023; Rabassa, Montse/AAA-6723-2020; Ferrucci, Luigi/AED-9724-2022; Tanaka, Toshiko/HKW-0340-2023; bandinelli, stefania/AAL-4570-2020; Zamora, Raul/HNC-1515-2023; Merono, Tomas/T-4060-2017; Andres-Lacueva, Cristina/J-3377-2012 Rabassa Bonet, Montserrat/0000-0002-0276-6726; Hidalgo Liberona, Nicole/0000-0003-0860-9657; Zamora-Ros, Raul/0000-0002-6236-6804; Bandinelli, Stefania/0000-0002-6491-0850; Merono, Tomas/0000-0002-2673-3494; Ferrucci, Luigi/0000-0002-6273-1613; Andres-Lacueva, Cristina/0000-0002-8494-4978
Italian Ministry of Health [ICS110.1/RF97.71, PE-201102350413]; U.S. National Institute on Aging [263 MD 9164, 263 MD 821336, N.1-AG-1-1, N.1-AG-1-2111, N01-AG-5-0002]; Intramural Research Program of the National Institute on Aging, National Institutes of Health, Baltimore, Maryland; CIBERFES - Instituto de Salud Carlos III [AC19/00096]; European Regional Development Fund "A way to make Europe"; Generalitat de Catalunya's Agency AGAUR [2017SGR1546]; ICREA Academia 2018; Carlos III Institute of Health [CD16/00157, CPII20/00009]; European Social Fund (ESF); MINECO [IJCI-2017-32534] Italian Ministry of Health(Ministry of Health, Italy); U.S. National Institute on Aging(United States Department of Health & Human ServicesNational Institutes of Health (NIH) - USANIH National Institute on Aging (NIA)); Intramural Research Program of the National Institute on Aging, National Institutes of Health, Baltimore, Maryland; CIBERFES - Instituto de Salud Carlos III; European Regional Development Fund "A way to make Europe"; Generalitat de Catalunya's Agency AGAUR; ICREA Academia 2018; Carlos III Institute of Health(Instituto de Salud Carlos III); European Social Fund (ESF)(European Social Fund (ESF)); MINECO(Spanish Government) The InCHIANTI study baseline (1998-2000) was supported as a "targeted project" (ICS110.1/RF97.71) by the Italian Ministry of Health (PE-201102350413) and in part by the U.S. National Institute on Aging (Contracts: 263 MD 9164 and 263 MD 821336), the InCHIANTI Follow-up 1 (2001-2003) was funded by the U.S. National Institute on Aging (Contracts: N.1-AG-1-1 and N.1-AG-1-2111), the InCHIANTI follow-up 2 and 3 studies (2004-2010) were financed by the U.S. National Institute on Aging (Contract: N01-AG-5-0002), and supported in part by the Intramural Research Program of the National Institute on Aging, National Institutes of Health, Baltimore, Maryland. This study was further supported by CIBERFES, AC19/00096 funded by Instituto de Salud Carlos III and co-funded by the European Regional Development Fund "A way to make Europe" and the award of the Generalitat de Catalunya's Agency AGAUR [2017SGR1546] and ICREA Academia 2018. MR and RZ-R would like to thank the "Sara Borrell" (CD16/00157) and "Miguel Servet"

(CPII20/00009) research contracts, respectively, from the Carlos III Institute of Health and the European Social Fund (ESF). TM would like to thank the "Juan de la Cierva" program from MINECO (IJCI-2017-32534). 59 15 15 1 16 BMC LONDON CAMPUS, 4 CRINAN ST, LONDON N1 9XW, ENGLAND 1741-7015 BMC MED BMC Med. NOV 24 2021 19 1 280 10.1186/s12916-021-02154-7 http://dx.doi.org/10.1186/s12916-021-02154-7 13 Medicine, General & Internal Science Citation Index Expanded (SCI-EXPANDED); Social Science Citation Index (SSCI) General & Internal Medicine XC2UI 34814922 gold, Green Published 2025-06-24 WOS:000721874000001

J Menna, M; Aiello, A; D'Aniello, F; Imperatore, C; Luciano, P; Vitalone, R; Irace, C; Santamaria, R Menna, Marialuisa; Aiello, Anna; D'Aniello, Filomena; Imperatore, Concetta; Luciano, Paolo; Vitalone, Rocco; Irace, Carlo; Santamaria, Rita Conithiaquinones A and B, Tetracyclic Cytotoxic Meroterpenes from the Mediterranean Ascidian Aplidium conicum EUROPEAN JOURNAL OF ORGANIC CHEMISTRY English Article Natural products; Terpenoids; Structure elucidation; Medicinal chemistry QUINONES; STEREOCHEMISTRY Chemical investigation of the Mediterranean ascidian A. conicum resulted in the isolation of two new meroterpenes, the conithiaquinones A (1) and B (2), in addition to two previously reported chromenols (3 and 4) and conicaquinones (5 and 6). The structures of conithiaquinones A and B were determined by interpretation of spectroscopic data, and regiochemical and stereochemical assignments were achieved with the aid of computational methods, including the recently developed DP4 NMR spectral prediction method. Both conithiaquinones A and B showed significant effects on the growth and viability of cells, with 1 showing interesting cytotoxicity against human breast cancer cells. [Menna, Marialuisa; Aiello, Anna; D'Aniello, Filomena; Imperatore, Concetta; Luciano, Paolo; Vitalone, Rocco] Univ Naples Federico II, Dipartimento Farm, NeaNat Grp, I-80131 Naples, Italy; [Irace, Carlo; Santamaria, Rita] Univ Naples Federico II, Dipartimento Farm, I-80131 Naples, Italy University of Naples Federico II; University of Naples Federico II Menna, M (corresponding author), Univ Naples Federico II, Dipartimento Farm, NeaNat Grp, Via D Montesano 49, I-80131 Naples, Italy. mlmenna@unina.it Irace, Carlo/ADC-8772-2022; Menna, Marialuisa/AAJ-3878-2021; Imperatore, Concetta/AAV-6692-2021 MENNA, Marialuisa/0000-0002-4515-3014; Irace, Carlo/0000-0002-5172-4573 European Commission [229893, 311848]; Italian Ministero dell'Universita e della Ricerca (MIUR) European Commission(European Union (EU)European Commission Joint Research Centre); Italian Ministero dell'Universita e della Ricerca (MIUR)(Ministry of Education, Universities and Research (MIUR)) This work was supported by the European Commission through the FP7 Projects 229893 (NatPharma) and 311848 (BlueGenics), and by the Italian Ministero dell'Universita e della Ricerca (MIUR) (PRIN2009: Sostanze naturali ed analoghi sintetici in grado di interferire con target biologici coinvolti nel controllo della crescita tumorale). All data concerning the reported compounds are included in LIBIOMOL, the chemical library of natural and synthetic bioactive molecules, accessible at the website http://www.libiomol.unina.it. 24 31 31 0 23

WILEY-V C H VERLAG GMBH WEINHEIM POSTFACH 101161, 69451 WEINHEIM, GERMANY 1434-193X 1099-0690 EUR J ORG CHEM Eur. J. Org. Chem. JUN 2013 2013 16
3241 3246 10.1002/ejoc.201300260
http://dx.doi.org/10.1002/ejoc.201300260 6
Chemistry, Organic Science Citation Index Expanded (SCI-EXPANDED); Index Chemicus (IC) Chemistry 161EQ
2025-06-24 WOS:000320175200006
J Jessri, M; Rashidkhani, B; Hajizadeh, B; Jacques, PF
Jessri, Mahsa; Rashidkhani, Bahram; Hajizadeh, Bahareh; Jacques, Paul F. Adherence to Mediterranean-Style Dietary Pattern and Risk of Esophageal Squamous Cell Carcinoma: A Case-Control Study in Iran JOURNAL OF THE AMERICAN COLLEGE OF NUTRITION English
Article esophageal squamous cell carcinoma; Iran; Mediterranean-Style Dietary Pattern Score (MSDPS) BREAST-CANCER RISK; AERODIGESTIVE TRACT CANCER; GUIDELINES-FOR-AMERICANS; MAJOR CHRONIC DISEASE; FOOD GROUP INTAKE; PHYSICAL-ACTIVITIES; RELATIVE VALIDITY; SELF-REPORT; POPULATION; SURVIVAL Objective: The benefit of adherence to a Mediterranean-style dietary pattern in relation to the risk of esophageal squamous cell carcinoma (ESCC) has not been investigated among non-Mediterranean high-risk populations. The objective of the present study was to examine the association of compliance with the Mediterranean dietary pattern as measured by Mediterranean-Style Dietary Pattern Score (MSDPS) and the risk of ESCC in Iranian population. Methods: This case-control study was conducted on 47 ESCC cases and 96 hospital controls aged 40-75 years. Participants were interviewed using validated questionnaires, and dietary patterns were characterized using the MSDPS. Results: Generally, the mean MSDPS in this population was low (30.84 +/- 8.58). MSDPS showed content validity through having expected positive associations with several lifestyle characteristics and dietary intakes. Being in the highest quartile category of MSDPS, compared to the lowest, was independently associated with 37% reduction in risk of ESCC. Two-unit and 3-unit increases in the MSDPS resulted in 41% and 47% reduction in risk of ESCC, respectively. Higher intakes of olive oil (odds ratio [OR] = 0.15, 95% CI: 0.01-0.49), fish and other seafood (OR = 0.48, 95% CI: 0.23-0.98), whole grain (OR = 0.57, 95% CI: 0.28-0.76), and fruits (OR = 0.77, 95% CI: 0.38-0.86) were significantly associated with reduced ESCC risk. In contrast, higher sweet (OR = 1.86, 95% CI: 1.04-2.12) and meat intakes (OR = 1.61, 95% CI: 1.25-2.49) were associated with higher ESCC risk. Conclusion: Consuming a diet in concordance with the principles of the Mediterranean dietary pattern may protect against ESCC. Preventive strategies to reduce ESCC risk in high-risk countries should focus on overall dietary pattern and dietary habits to be effective. [Jessri, Mahsa] Univ Alberta Edmonton, Div Human Nutr, Dept Agr Food & Nutr Sci, Edmonton Clin Hlth Acad, Edmonton, AB, Canada; [Jessri, Mahsa] Univ Alberta Edmonton, Alberta Inst Human Nutr, Edmonton Clin Hlth Acad, Edmonton, AB, Canada; [Rashidkhani, Bahram] Shahid Beheshti Univ Med Sci, Community Nutr Dept, Fac Nutr Sci & Food Technol, Natl Nutr & Food Technol Res Inst,WHO Collaborati, Tehran, Iran; [Hajizadeh, Bahareh] Kurdistan Univ Med Sci Kurdistan, Dept Oncol, Kurdistan, Iran; [Jacques, Paul F.] Tufts Univ, Jean Mayer USDA Human Nutr Res Ctr Aging, Boston, MA 02111 USA Shahid Beheshti University Medical Sciences; Tufts

University; United States Department of Agriculture (USDA)
Rashidkhani, B (corresponding author), Shahid Beheshti Univ Med Sci, Community Nutr Dept, Fac Nutr Sci & Food Technol, Natl Nutr & Food Technol Res Inst, Tehran, Iran.
b_rashidkhani@sbmu.ac.ir rashidkhani, bahram/ABG-9304-2021 Jessri, Mahsa/0000-0001-5197-4962 National Nutrition and Food Technology Research Institute (WHO Collaborating Center), Shahid Beheshti University of Medical Sciences, Tehran, Iran [4030] National Nutrition and Food Technology Research Institute (WHO Collaborating Center), Shahid Beheshti University of Medical Sciences, Tehran, Iran We are grateful to all filed investigators, staff, and participants of the present study. This study was supported by grant no. 4030 from the National Nutrition and Food Technology Research Institute (WHO Collaborating Center), Shahid Beheshti University of Medical Sciences, Tehran, Iran.
88 19 22 0 4 ROUTLEDGE JOURNALS, TAYLOR & FRANCIS LTD ABINGDON 2-4 PARK SQUARE, MILTON PARK, ABINGDON OX14 4RN, OXON, ENGLAND 0731-5724 1541-1087 J AM COLL NUTR
J. Am. Coll. Nutr. OCT 2012 31 5
338 351 10.1080/07315724.2012.10720437
http://dx.doi.org/10.1080/07315724.2012.10720437
14 Nutrition & Dietetics Science Citation Index Expanded (SCI-EXPANDED) Nutrition & Dietetics 120BN 23529991
2025-06-24 WOS:000317144600005
J Sarkin, J Sarkin, Jeremy
Respecting and protecting the lives of migrants and refugees: the need for a human rights approach to save lives and find missing persons INTERNATIONAL JOURNAL OF HUMAN RIGHTS
English Article
International law; refugees; migrants; missing persons; human rights; Europe IDENTIFICATION; MIGRATION; CONFLICT; POLITICS; VICTIMS; BORDERS; DEATHS; RESCUE; SEA The world is in a migration crisis. Thousands of people are dying annually trying to get across the Mediterranean. However, this is not a problem unique to Europe. It has remained a hidden global problem for a long time. What is specifically unknown are the numbers of people who have gone missing while migrating. This article therefore focuses on such missing people. It examines the numbers of people that are known to have died, and argues that there is a general dearth of information about both people who have died, as well as those who have gone missing. The article reviews who missing people are and argues that the term missing is only found to any large measure in the laws of armed conflict, and most other situations that cause people to go missing, those who are subject to enforced disappearances, are not covered. The article argues that more research ought to be done on these issues and more data ought to be collected and analysed. It argues that people on migration routes are vulnerable and further argues that more needs to be done to provide them with protection and assistance. The laws dealing with the missing are analysed. The article argues that a human rights approach is needed to deal with the problem and that states need to play much more of a compassionate and humane role concerning migration matters and missing people generally and specifically. The article also argues for partnerships and coordination, that the families need to be given more support, and that more public education is needed to deal with the negative perceptions and misconceptions that exist in many societies to which those migrating seek to move to, as this

will give greater positive impetus to states to deal more appropriately with those who migrate for whatever reason.

[Sarkin, Jeremy] Univ South Africa UNISA Pretoria, Dept Publ Constitut & Int Law, Pretoria, South Africa; [Sarkin, Jeremy] NOVA Univ Lisbon, Sch Law, Lisbon, Portugal University of South Africa; Universidade Nova de Lisboa Sarkin, J (corresponding author), Univ South Africa UNISA Pretoria, Dept Publ Constitut & Int Law, Pretoria, South Africa.; Sarkin, J (corresponding author), NOVA Univ Lisbon, Sch Law, Lisbon, Portugal.
JSarkin@post.harvard.edu Sarkin, Jeremy/W-2410-2019
Sarkin, Jeremy/0000-0002-9424-6874

136 10 10 0 13 ROUTLEDGE JOURNALS, TAYLOR & FRANCIS LTD ABINGDON 2-4 PARK SQUARE, MILTON PARK, ABINGDON OX14 4RN, OXON, ENGLAND 1364-2987 1744-053X INT J HUM RIGHTS Int. J. Hum. Rights 2018 22 2
207 236 10.1080/13642987.2017.1354572
http://dx.doi.org/10.1080/13642987.2017.1354572
30 Law Social Science Citation Index (SSCI)
Government & Law GE2GJ Green Published
2025-06-24 WOS:000431033300005

J Sungu, M; Isik, M; Güler, Ü; Eylem, CC; Eskizengin, H; Nemutlu, E; Salih, B; Derkus, B Sungu, Mustafa; Isik, Melis; Guler, Ulku; Eylem, Cemil Can; Eskizengin, Hakan; Nemutlu, Emirhan; Salih, Bekir; Derkus, Burak

Manipulating macrophage polarization with nanoparticles to control metastatic behavior in heterotypic breast cancer micro-tissues via exosome signaling NANOSCALE English
Article ACTIVATING MACROPHAGES; IMMUNITY; PATHWAY; OXIDE This study aimed to investigate the effects of nanoparticles on macrophage polarization and their subsequent influence on post-tumorigenic behavior. Initially, seven different nanoparticles were applied to macrophages, and Zn-Ni-FeO (100 nm) and palladium nanoparticles (PdNPs, similar to 25 nm) were found to induce M1-polarization in macrophages. A co-culture experiment was then conducted to examine the effects of macrophages on MCF-7 breast cancer micro-tissues. The M2-macrophages promoted tumor proliferation, while M1- and PdNPs-induced macrophages showed anti-tumor effects by suppressing cell proliferation. To reveal the mechanisms of effect, exosomes isolated from M1 (M1-Exo), M0 (M0-Exo), M2 (M2-Exo), and PdNPs-induced (PdNPs-Exo) macrophages were applied to the heterotypic tumor micro-tissues including MCF-7, human umbilical vein endothelial cells (HUVECs), and primary human dermal fibroblasts (phDFs). M2-Exo was seen to promote the migration of cancer cells and induce epithelial-mesenchymal transition (EMT), while M1-Exo suppressed these behaviors. PdNPs-Exo was effective in suppressing the aggressive nature of breast cancer cells similar to M1-Exo, moreover, the efficacy of 5-fluorouracil (5-FU) was increased in combination with PdNPs-Exo in both MCF-7 and heterotypic micro-tissues. In conclusion, PdNPs-Exo has potential anti-tumor effects, can be used as a combination therapy to enhance the efficacy of anti-cancer drugs, as well as innovative implants for breast cancer treatment. [Sungu, Mustafa; Isik, Melis; Derkus, Burak] Ankara Univ, Fac Sci, Dept Chem, Stem Cell Res Lab, Ankara, Turkiye; [Guler, Ulku; Salih, Bekir] Hacettepe Univ, Fac Sci, Dept Chem, TR-06800 Ankara, Turkiye; [Eylem, Cemil Can; Nemutlu, Emirhan] Hacettepe Univ, Fac Pharm, Analyt Chem Div, TR-06230 Ankara, Turkiye; [Eskizengin, Hakan] Ankara Univ, Fac Sci, Dept

Biol, TR-06560 Ankara, Turkiye; [Derkus, Burak] Ankara Univ, Fac Dent, Dept Dentomaxillofacial Radiol, TR-06560 Ankara, Turkiye
Ankara University; Hacettepe University; Hacettepe University; Ankara University; Ankara University Derkus, B (corresponding author), Ankara Univ, Fac Sci, Dept Chem, Stem Cell Res Lab, Ankara, Turkiye.; Derkus, B (corresponding author), Ankara Univ, Fac Dent, Dept Dentomaxillofacial Radiol, TR-06560 Ankara, Turkiye. bderkus@ankara.edu.tr Eskizengin, Hakan/AAH-9305-2020; Eylem, Cemil/AAR-6624-2020; Derkus, Burak/AAA-8727-2020; GÜLER, ÜLKÜ/G-9292-2013; Salih, Bekir/IWD-6549-2023; Nemutlu, Emirhan/D-3218-2013Isik, Melis/0000-0003-1101-7548; Nemutlu, Emirhan/0000-0002-7337-6215; Derkus, Burak/0000-0001-5558-0995 Turkish Academy of Science (TUBA) Turkish Academy of Science (TUBA)(Turkish Academy of Sciences) This paper has been produced with the data presented in M. Sungu's Master's Thesis. B. Derkus and B. Salih acknowledge the Turkish Academy of Science (TUBA) for their support. We thank the Fatih Inci and Nedim Haciosmanoglu (Bilkent University, Turkey) for their kind help in NTA analysis. We also thank the Prof. Orhan Adal & imath; and Dr Merve Akkulak (Middle East Technical University, Turkey) for their helps in ultracentrifugation. 69 3 3 5 21
ROYAL SOC CHEMISTRY CAMBRIDGE THOMAS GRAHAM HOUSE, SCIENCE PARK, MILTON RD, CAMBRIDGE CB4 0WF, CAMBS, ENGLAND
2040-3364 2040-3372 NANOSCALE Nanoscale DEC 21
2023 16 1 394 410
10.1039/d3nr04980a http://dx.doi.org/10.1039/d3nr04980a
DEC 2023 17 Chemistry, Multidisciplinary; Nanoscience & Nanotechnology; Materials Science, Multidisciplinary; Physics, Applied Science Citation Index Expanded (SCI-EXPANDED) Chemistry; Science & Technology - Other Topics; Materials Science; Physics CR0K4 38073471
2025-06-24 WOS:001122807500001
J Hermansyah, D; Pricilia, G; Simamora, Y; Siregar, DR
Hermansyah, Dedy; Pricilia, Gracia; Simamora, Yolanda; Siregar, Denny Rifsal Clinicopathological Characteristics of Patients with BRCA Mutation Breast Cancer in North Sumatera: Case Report MIDDLE EAST JOURNAL OF CANCER
English Article
Breast neoplasms; Genes; BRCA1; BRCA2; Immunohistochemistry
The most common cancer in women is breast cancer (BC) with an incidence of 24.2%. BC in younger patients will in general be more forceful, prompting more awful results and a requirement for more forceful treatment which may bring about a higher probability of long-haul treatment-related harmfulness and novel psychosocial issues. Furthermore, family inclination to BC as BRCA1 and BRCA2 mutations is more prevalent in this age group. There were a total of five ladies who had tumor pathology testing with negative results. All intrusive BC examples were regularly assessed for estrogen receptor, progesterone receptor, and human epidermal growth factor receptor-2 (HER2)/neu status utilizing immunohistochemistry. Cases with HER2/neu staining of 1+, 2+ or 3+ on immunohistochemistry examination were additionally assessed by fluorescent in situ hybridization for the enhancement of the HER2/neu quality. In this examination, we distinguished clinicopathological attributes of patients with BC. We partitioned into two gatherings, BRCA positive change and BRCA negative transformation. Roughly 5%-10% instances of BC have a positive family ancestry and about 20%-40% BC development were in acquired

variations. Our study revealed that 20% of cases included individuals who had a family history of BRCA mutation. Male relatives with BC, earlier age at onset, a greater prevalence of reciprocal breast disease, and a connection to various malignancies in the ovary, colon, prostate, pancreas, and endometrial are only a few of the clear clinical characteristics of BRCA1/2-related BC. [Hermansyah, Dedy; Pricilia, Gracia; Simamora, Yolanda; Siregar, Denny Rifsal] Univ Sumatera Utara, Fac Med, Dept Surg, Medan, Indonesia University of North Sumatra
Hermansyah, D (corresponding author), Univ Sumatera Utara, Fac Med, Dept Surg, Medan, Indonesia. escape744@gmail.com
Hermansyah, Dedy/AAC-7849-2021
15 0 0 0 0 SHIRAZ UNIV MEDICAL SCIENCES
SHIRAZ NEMAZEE HOSPITAL, SHIRAZ, 71934, IRAN 2008-6709
2008-6687 MIDDLE EAST J CANCER Middle East J. Cancer
APR 2023 14 2 332 337
10.30476/mejc.2022.92503.1659
http://dx.doi.org/10.30476/mejc.2022.92503.1659
6 Oncology Emerging Sources Citation Index (ESCI)
Oncology E4ES6 2025-06-24
WOS:000975097200017
J Galbete, C; Kröger, J; Jannasch, F; Iqbal, K; Schwingshackl, L; Schwedhelm, C; Weikert, C; Boeing, H; Schulze, MB
Galbete, Cecilia; Kroeger, Janine; Jannasch, Franziska; Iqbal, Khalid; Schwingshackl, Lukas; Schwedhelm, Carolina; Weikert, Cornelia; Boeing, Heiner; Schulze, Matthias B.
Nordic diet, Mediterranean diet, and the risk of chronic diseases: the EPIC-Potsdam study BMC MEDICINE
English Article
Mediterranean diet; Nordic diet; regional diets; chronic diseases; diabetes; myocardial infarction; stroke; cancer; EPIC-Potsdam study; longitudinal analysis FOOD-FREQUENCY QUESTIONNAIRE; BALTIC SEA DIET; BREAST-CANCER RISK; CARDIOVASCULAR-DISEASE; COLORECTAL-CANCER; NO ASSOCIATION; ADHERENCE; INDEX; WOMEN; METAANALYSIS Background: The Mediterranean Diet (MedDiet) has been acknowledged as a healthy diet. However, its relation with risk of major chronic diseases in non-Mediterranean countries is inconclusive. The Nordic diet is proposed as an alternative across Northern Europe, although its associations with the risk of chronic diseases remain controversial. We aimed to investigate the association between the Nordic diet and the MedDiet with the risk of chronic disease (type 2 diabetes (T2D), myocardial infarction (MI), stroke, and cancer) in the EPIC-Potsdam cohort. Methods: The EPIC-Potsdam cohort recruited 27,548 participants between 1994 and 1998. After exclusion of prevalent cases, we evaluated baseline adherence to a score reflecting the Nordic diet and two MedDiet scores (tMDS, reflecting the traditional MedDiet score, and the MedPyr score, reflecting the MedDiet Pyramid). Cox regression models were applied to examine the association between the diet scores and the incidence of major chronic diseases. Results: During a follow-up of 10.6 years, 1376 cases of T2D, 312 of MI, 321 of stroke, and 1618 of cancer were identified. The Nordic diet showed a statistically non-significant inverse association with incidence of MI in the overall population and of stroke in men. Adherence to the MedDiet was associated with lower incidence of T2D (HR per 1 SD 0.93, 95% CI 0.88-0.98 for the tMDS score and 0.92, 0.87-0.97 for the MedPyr score). In women, the MedPyr score was also

inversely associated with MI. No association was observed for any of the scores with cancer. Conclusions: In the EPIC-Potsdam cohort, the Nordic diet showed a possible beneficial effect on MI in the overall population and for stroke in men, while both scores reflecting the MedDiet conferred lower risk of T2D in the overall population and of MI in women. [Galbete, Cecilia; Kroeger, Janine; Jannasch, Franziska; Schulze, Matthias B.] German Inst Human Nutr Potsdam Rehbruecke DIfE, Dept Mol Epidemiol, Nuthetal, Germany; [Iqbal, Khalid; Schwingshackl, Lukas; Schwedhelm, Carolina; Boeing, Heiner] German Inst Human Nutr Potsdam Rehbrucke, Dept Epidemiol, Nuthetal, Germany; [Weikert, Cornelia] Fed Inst Risk Assessment, Dept Food Safety, Berlin, Germany; [Schulze, Matthias B.] Univ Potsdam, Inst Nutr Sci, Nuthetal, Germany; [Schulze, Matthias B.] German Ctr Diabet Res DZD, Munich, Germany; [Schulze, Matthias B.] DZHK German Ctr Cardiovasc Res, Partner Site Berlin, Berlin, Germany; [Galbete, Cecilia; Iqbal, Khalid; Schwingshackl, Lukas; Schwedhelm, Carolina; Weikert, Cornelia; Boeing, Heiner; Schulze, Matthias B.] NutriAct Competence Cluster Nutr Res Berlin Potsd, Nuthetal, Germany
Leibniz Association; Deutsches Institut fur Ernahrungsforschung Potsdam-Rehbrucke (DIfE); Leibniz Association; Deutsches Institut fur Ernahrungsforschung Potsdam-Rehbrucke (DIfE); Federal Institute for Risk Assessment; University of Potsdam; German Center for Diabetes Research (DZD); German Centre for Cardiovascular Research Schulze, MB (corresponding author), German Inst Human Nutr Potsdam Rehbruecke DIfE, Dept Mol Epidemiol, Nuthetal, Germany.; Schulze, MB (corresponding author), Univ Potsdam, Inst Nutr Sci, Nuthetal, Germany.; Schulze, MB (corresponding author), German Ctr Diabet Res DZD, Munich, Germany.; Schulze, MB (corresponding author), DZHK German Ctr Cardiovasc Res, Partner Site Berlin, Berlin, Germany.; Schulze, MB (corresponding author), NutriAct Competence Cluster Nutr Res Berlin Potsd, Nuthetal, Germany. mschulze@dife.de Schwingshackl, Lukas/AAC-4119-2019; Iqbal, Khalid/AAD-7112-2022; Schwedhelm, Carolina/AAE-6756-2022; Schwingshackl, Lukas/B-9220-2013; Schulze, Matthias B./AAH-6906-2021; Galbete, Cecilia/M-1276-2013
Schwingshackl, Lukas/0000-0003-3407-7594; Schulze, Matthias B./0000-0002-0830-5277; Galbete, Cecilia/0000-0002-2497-6791; Iqbal, Khalid/0000-0002-3312-4259 NutriAct - Competence Cluster Nutrition Research Berlin-Potsdam - German Federal Ministry of Education and Research [FKZ: 01EA1408A-G] NutriAct - Competence Cluster Nutrition Research Berlin-Potsdam - German Federal Ministry of Education and Research This work was supported by NutriAct - Competence Cluster Nutrition Research Berlin-Potsdam funded by the German Federal Ministry of Education and Research (FKZ: 01EA1408A-G). The funders had no role in study design, data collection and analysis, decision to publish, or preparation of the manuscript. 53 90 92 1 20 BMC LONDON
CAMPUS, 4 CRINAN ST, LONDON N1 9XW, ENGLAND 1741-7015
BMC MED BMC Med. JUN 27 2018 16
99 10.1186/s12916-018-1082-y
http://dx.doi.org/10.1186/s12916-018-1082-y 13
Medicine, General & Internal Science Citation Index Expanded (SCI-EXPANDED) General & Internal Medicine GL3KX 29945632
Green Published, gold 2025-06-24
WOS:000437034600001

J Abou Khouzam, R; Chouaib, S; Iqbal, MA Abou Khouzam, Raefa; Chouaib, Salem; Iqbal, Mohammad Askandar

Integrative systems-level analysis reveals a contextual crosstalk between hypoxia and global metabolism in human breast tumors MOLECULAR ONCOLOGY English Article breast cancer; cancer metabolism; hypoxia; systems biology; Warburg effect AEROBIC GLYCOLYSIS; CANCER CELLS; REQUIREMENTS; THERAPY; GROWTH Hypoxia is known to induce reprogramming of glucose metabolism in cancer. However, the impact of hypoxia on global metabolism remains poorly understood. Here, using the systems approach, we evaluated the potential crosstalk between hypoxia and global metabolism using data from > 2000 breast tumors. Tumor samples were scored for hypoxia and 90 metabolic pathways, and these metrics were subjected to an analysis pipeline. Hypoxia showed a very strong association with metabolic aggression and an overall contextual relationship with metabolism. Out of three (M1, M2, and M3) metabolic types in breast cancer, M3 exhibited the strongest relationship with hypoxia; that is, high hypoxic tumors were also metabolically deregulated. Further, the overall correlation pattern between hypoxia and metabolic pathway scores was specific to each type, with M1 showing maximal sensitivity to hypoxia, followed by M2 and then M3. Experimental validation using metabolic inhibitors on cell lines with high or low hypoxia scores further confirmed the metabolic type-dependence of hypoxia. In addition, evaluation of the impact of hypoxia on cancer pathways other than metabolic ones revealed a potential role of hypoxia in immune evasive characteristic of M3 tumors. Overall, the results suggest a complex interplay between hypoxia and metabolism in the context of human breast tumors, with potential implications for both basic cancer biology and breast cancer therapy. [Abou Khouzam, Raefa; Chouaib, Salem; Iqbal, Mohammad Askandar] Gulf Med Univ, Thumbay Res Inst Precis Med, Ajman, U Arab Emirates; [Abou Khouzam, Raefa; Chouaib, Salem; Iqbal, Mohammad Askandar] Gulf Med Univ, Coll Med, Ajman, U Arab Emirates; [Chouaib, Salem] Univ Paris Saclay, EPHE, INSERM UMR 1186, Gustave Roussy,Fac Med, F-94805 Villejuif, France Institut National de la Sante et de la Recherche Medicale (Inserm); Universite Paris Saclay; UNICANCER; Gustave Roussy; Universite PSL; Ecole Pratique des Hautes Etudes (EPHE) Iqbal, MA (corresponding author), Gulf Med Univ, Thumbay Res Inst Precis Med, Ajman, U Arab Emirates. dr.askandar@gmu.ac.ae Abou Khouzam, Raefa/GLS-8588-2022 Iqbal, Mohammad Askandar/0000-0003-3485-3530 Gulf Medical University; L'Oreal-UNESCO For Women in Science Middle East Regional Young Talents program grant Gulf Medical University; L'Oreal-UNESCO For Women in Science Middle East Regional Young Talents program grant Authors are grateful to Miss Kosar Hajnajafi for the assistance in analysis. Authors further acknowledge funding support from Gulf Medical University. RAK acknowledges support received from the L'Oreal-UNESCO For Women in Science Middle East Regional Young Talents program grant 2022. 47 0 0 0 0 WILEY HOBOKEN 111 RIVER ST, HOBOKEN 07030-5774, NJ USA 1574-7891 1878-0261 MOL ONCOL Mol. Oncol. JUN 2025 19 6 1725 1736 10.1002/1878-0261.13762 http://dx.doi.org/10.1002/1878-0261.13762 DEC 2024 12 Oncology Science Citation Index Expanded (SCI-EXPANDED) Oncology 3SY6N 39729399 gold 2025-06-24 WOS:001385549800001

J Genovese, C; Garozzo, A; D'Angeli, F; Malfa, GA; Bellia, F; Tomasello, B; Nicolosi, D; Malaguarnera, R; Ronsisvalle, S; Guadagni, F; Acquaviva, R Genovese, Carlo; Garozzo, Adriana; D'Angeli, Floriana; Malfa, Giuseppe Antonio; Bellia, Francesco; Tomasello, Barbara; Nicolosi, Daria; Malaguarnera, Roberta; Ronsisvalle, Simone; Guadagni, Fiorella; Acquaviva, Rosaria Orobanche crenata Forssk. Extract Affects Human Breast Cancer Cell MCF-7 Survival and Viral Replication CELLS English Article Orobanche crenata extract; MCF-7 cells; MDA-MB-231 cells; Herpes simplex virus; Coxsackievirus; parasitic plant CYCLE ARREST; IN-VITRO; ANTIVIRAL ACTIVITY; LUTEOLIN; ANTIOXIDANT; SALIDROSIDE; APOPTOSIS; APIGENIN; THERAPY; L. Background: Breast cancer (BC) is the leading cause of death worldwide. The severity of BC strictly depends on the molecular subtype. The less aggressive hormone-positive subtype is treated with adjuvant endocrine therapy (AET), which causes both physical and psychological side effects. This condition strongly impacts the adherence and persistence of AET among oncologic patients. Moreover, viral infections also constitute a serious problem for public health. Despite their efficacy, antiviral agents present several therapeutic limits. Accordingly, in the present work, we investigated the antitumor and antiviral activities of Orobanche crenata Forssk. (O. crenata), a parasitic plant, endemic to the Mediterranean basin, traditionally known for its beneficial properties for human health. Methods: The MTT assay was carried out to evaluate the cytotoxic effect of O. crenata leaf extract (OCLE) on human breast cancer cells (MCF-7 and MDA-MB-231) and the primary HFF-1 cell line. The lactic dehydrogenase (LDH) assay was performed on MCF-7 cells to analyze necrotic cell death. The antioxidant effect of OCLE was evaluated by intracellular determination of the reactive oxygen species and thiol groups, by DPPH and ABTS assays. The antiviral activity of OCLE was determined against Poliovirus 1, Echovirus 9, Human respiratory syncytial virus, Adenovirus type 2 and type 5, Coxsackievirus B1 (CoxB1) and B3 (CoxB3), Herpes simplex type 1 (HSV-1) and type 2 (HSV-2), and beta-Coronavirus by the plaque reduction assay. Results: The extract, after 24 h of incubation, did not affect MDA-MB-231 and HFF-1 cell viability. However, at the same time point, it showed a dose-dependent inhibitory effect on MCF-7 cells, with an increase in LDH release. OCLE exhibited free radical scavenging activity and significantly increased non-protein thiol levels in MCF-7 cells. OCLE effectively inhibited HSV-1, HSV-2, CoxB1, and CoxB3 replication. Conclusions: The overall results showed an interesting inhibitory effect of OCLE on both MCF-7 cell survival and viral replication. [Genovese, Carlo; Malaguarnera, Roberta] Kore Univ Enna, Fac Med & Surg, I-94100 Enna, Italy; [Genovese, Carlo; Nicolosi, Daria; Ronsisvalle, Simone; Acquaviva, Rosaria] Spin Off Univ Catania, Nacture Srl, I-95123 Catania, Italy; [Garozzo, Adriana] Univ Catania, Dept Biomed & Biotechnol Sci, Microbiol Sect, I-95123 Catania, Italy; [D'Angeli, Floriana; Guadagni, Fiorella] San Raffaele Roma Open Univ, Dept Human Sci & Qual Life Promot, I-00166 Rome, Italy; [Malfa, Giuseppe Antonio; Tomasello, Barbara; Acquaviva, Rosaria] Univ Catania, Dept Drug & Hlth Sci, Biochem Sect, I-95125 Catania, Italy; [Malfa, Giuseppe Antonio; Tomasello, Barbara; Acquaviva, Rosaria] Univ Catania, Res Ctr Nutraceut & Hlth Prod CERNUT, I-95125 Catania, Italy; [Bellia, Francesco] Natl Res Council CNR,

Inst Crystallog, I-95126 Catania, Italy; [Nicolosi, Daria] Univ Catania, Dept Drug & Hlth Sci, Microbiol Sect, I-95125 Catania, Italy; [Ronsisvalle, Simone] Univ Catania, Dept Drug & Hlth Sci, Med Chem Sect, I-95125 Catania, Italy; [Guadagni, Fiorella] IRCCS San Raffaele Pisana, BioBIM Interinst Multidisciplinary Biobank, I-00166 Rome, Italy Universita Kore di ENNA; University of Catania; University of Catania; University of Catania; Consiglio Nazionale delle Ricerche (CNR); Istituto Di Cristallografia (IC-CNR); University of Catania; University of Catania; IRCCS San Raffaele Pisana D'Angeli, F (corresponding author), San Raffaele Roma Open Univ, Dept Human Sci & Qual Life Promot, I-00166 Rome, Italy. carlo.genovese@unikore.it; agar@unict.it; floriana.dangeli@uniroma5.it; g.malfa@unict.it; francesco.bellia@cnr.it; btomase@unict.it; dnicolosi@unict.it; roberta.malaguarnera@unikore.it; s.ronsisvalle@unict.it; fiorella.guadagni@sanraffaele.it; racquavi@unict.it
MALAGUARNERA, ROBERTA/K-8838-2016; Acquaviva, Rosaria/A-6750-2018; Ronsisvalle, Simone/AAD-7995-2019; D'Angeli, Floriana/AAU-5570-2020; NICOLOSI, DARIA/AAR-9902-2020; Malfa, Giuseppe/AAG-4234-2020; Bellia, Francesco/H-2812-2013; GENOVESE, Carlo/D-7837-2012 MALAGUARNERA, Roberta/0000-0003-4149-9488; Acquaviva, Rosaria/0000-0002-3139-1177; Malfa, Giuseppe/0000-0002-6733-0587; Bellia, Francesco/0000-0002-9273-5949; GENOVESE, Carlo/0000-0003-2463-9047 European Social Fund [CUP G88I18000710007] European Social Fund(European Social Fund (ESF))
This work was partially supported by Grants from the European Social Fund PO FESR 2014-2020 Sicily Region Action 1.1.5, NUVACAL BC (CUP G88I18000710007). 116 6 6 0 3
MDPI BASEL ST ALBAN-ANLAGE 66, CH-4052 BASEL, SWITZERLAND
2073-4409 CELLS-BASEL Cells MAY 2022 11 10
1696 10.3390/cells11101696
http://dx.doi.org/10.3390/cells11101696 19 Cell Biology Science Citation Index Expanded (SCI-EXPANDED) Cell Biology 1Q1YY 35626733 Green Published, gold
2025-06-24 WOS:000802493200001
J Mehra, A; Mehrkar, R; Fakhri, A; Fard, PJ; Asgharzadeh, MR; Agdam, MHG; Sharifi, R Mehra, Amin; Mehrkar, Reza; Fakhri, Amir; Fard, Pedram Jabbari; Asgharzadeh, Mohammad Reza; Agdam, Mohammad Hossein Ghaffari; Sharifi, Rasoul
A Mimicry of the Tumor Microenvironment's Impact on SLC4A7 (NBCn1) and Caspase-3 Gene Expression in Breast Cancer, along with in Silico Traits of NBCn1 MIDDLE EAST JOURNAL OF CANCER
English Article
Tumor microenvironment; Caspase-3; Breast neoplasms; In silico COTRANSPORTER NBCN1; NA+/H+ EXCHANGER; NHE1
Background: This study investigates the relative expression of the Na + , HCO 3- cotransport gene NBCn1, and caspase-3 within the tumor microenvironment of human breast cancer, considering the in vivo microenvironment. Method: In this experimental study, breast cancer MDA-MB-231 cells were cultured under normoxia/hypoxia conditions for 24, 48, and 72 hours with varying glucose concentrations (5.5, 11, and 25 mM). The mRNA expression of NBCn1 and caspase-3 was evaluated using real -time polymerase chain reaction. The stability and binding pocket of NBCn1 were assessed using DispHred and the Computed Atlas of Surface Topography of proteins (CASTp) servers, respectively. The location prediction of the protein was determined using the Transmembrane Helices; Hidden Markov Model (TMHMM) server. Results: Normoxia led

to an increase in NBCn1 expression during all three time periods, displaying heterogeneity. The expression was particularly elevated at glucose concentrations of 25 and 5.5 mM. In hypoxic conditions, gene expression was reduced; however, an increase in glucose concentration enhanced SLC4A7 expression. Specifically, a glucose concentration of 25 mM led to decreased caspase-3 expression under hypoxic conditions. In silico studies revealed that SLC4A7 becomes disordered when the pH falls below 7, with most amino acids in the binding pocket being nonpolar. Conclusion: The heightened risk of breast cancer metastasis may be linked to the upregulation of SLC4A7 and downregulation of caspase-3 expression, underscoring their fundamental roles in cancer treatment and prevention. SLC4A7 is a transmembrane protein, and its folding is pH-dependent.
[Mehra, Amin] Tabriz Univ Med Sci, Fac Pharm, Tabriz, Iran; [Mehrkar, Reza] Tabriz Univ Med Sci, Fac Paramed, Dept Med Lab Sci, Tabriz, Iran; [Fakhri, Amir; Asgharzadeh, Mohammad Reza] Islamic Azad Univ, Dept Biol, Urmia Branch, Orumiyeh, Iran; [Fard, Pedram Jabbari; Agdam, Mohammad Hossein Ghaffari] Islamic Azad Univ, Fac Basic Sci, Dept Microbiol, Sci & Res Branch, Tehran, Iran; [Sharifi, Rasoul] Islamic Azad Univ, Dept Biol, Ahar Branch, Ahar, Iran; [Sharifi, Rasoul] Islamic Azad Univ, Dept Biol, Tabriz Branch, Tabriz, Iran Tabriz University of Medical Science; Tabriz University of Medical Science; Islamic Azad University; Islamic Azad University; Islamic Azad University; Islamic Azad University Sharifi, R (corresponding author), Islamic Azad Univ, Dept Biol, Ahar Branch, Ahar, Iran.; Sharifi, R (corresponding author), Islamic Azad Univ, Dept Biol, Tabriz Branch, Tabriz, Iran. rasoulsharifi.sci@gmail.com sharifi, rasoul/ABA-7390-2021; Fakhri, Amir/HKF-3741-2023; Asgharzadeh, Mohmmad/AAN-4889-2021
sharifi, rasoul/0000-0001-5079-5655; Mehra, Amin/0009-0000-4139-5326
38 0 0 0 1
SHIRAZ UNIV MEDICAL SCIENCES SHIRAZ NEMAZEE HOSPITAL, SHIRAZ, 71934, IRAN 2008-6709 2008-6687 MIDDLE EAST J CANCER Middle East J. Cancer JUL 2024 15 3
176 188 10.30476/mejc.2023.99391.1942
http://dx.doi.org/10.30476/mejc.2023.99391.1942
13 Oncology Emerging Sources Citation Index (ESCI)
Oncology WG5M5 2025-06-24
WOS:001253729900003
J Daoud, N; Osman, A; Hart, TA; Berry, EM; Adler, B
Daoud, Nihaya; Osman, Amira; Hart, Trevor A.; Berry, Elliott M.; Adler, Bella Self-care management among patients with type 2 diabetes in East Jerusalem HEALTH EDUCATION JOURNAL English Article
Health Belief Model; low-income populations; Middle East; self-care management; type 2 diabetes
HEALTH; POPULATION; BEHAVIORS; MELLITUS; BARRIERS; BELIEFS
Objective: Little research exists on diabetes self-care management (DSCM) in Arab populations. We examined the contribution of health belief constructs, socioeconomic position (SEP) and clinical factors (glycated haemoglobin [HbA1C] level, type of diabetes treatments, and receiving professional guidance) to DSCM among Arab patients in East Jerusalem with type 2 diabetes. Method: Using a structured questionnaire, we conducted face-to-face interviews with a random sample of 230 patients with type 2 diabetes in a large diabetes clinic. DSCM included engagement in any of the following in the last week: physical activity, consumption of low-fat and low-sugar diet, self-

monitoring of blood glucose, medication uptake and foot care. We obtained HbA1C levels from the clinic's patient registry. We used linear regression to examine the contribution of health beliefs, SEP and clinical factors to explaining DSCM. Results: Adherence to DSCM was low. Most patients (84.8%) were physically inactive, 64.3% did not consume a low-fat or low-sugar diet (46.5%) and 51% did not self-monitor blood glucose. However, medication adherence (95.7%) and foot care were high (77.4%). About 71% of participants had high HbA1C (>7.0%). In the multivariate analysis, total DSCM scores were higher among patients with low financial barriers, high perception of the benefits of DSCM and higher self-efficacy. Patients using oral medication (vs insulin) had significantly lower DSCM scores. Conclusion: Among Arab patients with diabetes, more interventions are needed to encourage DSCM, specifically in areas of lifestyle (diet and physical activity). Patients' financial barriers, benefits of DSCM and patient self-efficacy should be emphasised. [Daoud, Nihaya] Ben Gurion Univ Negev, Fac Hlth Sci, Dept Publ Hlth, IL-84015 Beer Sheva, Israel; [Osman, Amira; Berry, Elliott M.; Adler, Bella] Hebrew Univ Jerusalem Hadassah Hosp & Med Sch, Braun Sch Publ Hlth & Community Med, Jerusalem, Israel; [Hart, Trevor A.] Ryerson Univ, Dept Psychol, Toronto, ON, Canada; [Berry, Elliott M.] Hebrew Univ Jerusalem Hadassah Hosp & Med Sch, Dept Human Nutr & Metab, Jerusalem, Israel	Ben-Gurion University of the Negev; Hebrew University of Jerusalem; Hadassah University Hospital; Hadassah University Medical Center; Toronto Metropolitan University; Hebrew University of Jerusalem; Hadassah University Hospital; Hadassah University Medical Center	Daoud, N (corresponding author), Ben Gurion Univ Negev, Fac Hlth Sci, Dept Publ Hlth, POB 653, IL-84015 Beer Sheva, Israel.	daoud@bgu.ac.il	; Daoud, Nihaya/AAE-6602-2020
Hart, Trevor/0000-0001-5107-7452; Daoud, Nihaya/0000-0003-4542-8978	Israel National Institute for Health Policy and Health Services Research (NIHP); Canadian Institutes of Health Research
Israel National Institute for Health Policy and Health Services Research (NIHP); Canadian Institutes of Health Research(Canadian Institutes of Health Research (CIHR))	This research received no specific grant from any funding agency in the public, commercial, or not-for-profit sectors. Amira Osman was supported by The Israel National Institute for Health Policy and Health Services Research (NIHP). Trevor A Hart was supported by a New Investigator Salary Award from the Canadian Institutes of Health Research.	30	4	6	2	20	SAGE PUBLICATIONS LTD LONDON	1 OLIVERS YARD, 55 CITY ROAD, LONDON EC1Y 1SP, ENGLAND	0017-8969	1748-8176		HEALTH EDUC J
Health Educ. J.	SEP	2015	74	5
603	615		10.1177/0017896914555038
http://dx.doi.org/10.1177/0017896914555038		13
Education & Educational Research; Public, Environmental & Occupational Health	Social Science Citation Index (SSCI)
Education & Educational Research; Public, Environmental & Occupational Health	CP7PN			2025-06-24
WOS:000360080300009
J	Barragán, R; Coltell, O; Asensio, EM; Francés, F; Sorlí, JV; Estruch, R; Salas-Huetos, A; Ordovas, JM; Corella, D
Barragan, Rocio; Coltell, Oscar; Asensio, Eva M.; Frances, Francesc; Sorli, Jose V.; Estruch, Ramon; Salas-Huetos, Albert; Ordovas, Jose M.; Corella, Dolores		MicroRNAs and Drinking: Association between the Pre-miR-27a rs895819

Polymorphism and Alcohol Consumption in a Mediterranean Population
INTERNATIONAL JOURNAL OF MOLECULAR SCIENCES
English Article
microRNAs; alcohol; miR27a; Mediterranean SINGLE NUCLEOTIDE POLYMORPHISMS; GASTRIC-CANCER SUSCEPTIBILITY; FUNCTIONAL POLYMORPHISM; MENDELIAN RANDOMIZATION; COLORECTAL-CANCER; GENETIC VARIANT; RISK; EXPRESSION; MIR-27A; DIETRecently, microRNAs (miRNA) have been proposed as regulators in the different processes involved in alcohol intake, and differences have been found in the miRNA expression profile in alcoholics. However, no study has focused on analyzing polymorphisms in genes encoding miRNAs and daily alcohol consumption at the population level. Our aim was to investigate the association between a functional polymorphism in the pre-miR-27a (rs895819 A>G) gene and alcohol consumption in an elderly population. We undertook a cross-sectional study of PREvencion con DIeta MEDiterranea (PREDIMED)-Valencia participants (n = 1007, including men and women aged 67 +/- 7 years) and measured their alcohol consumption (total and alcoholic beverages) through a validated questionnaire. We found a strong association between the pre-miR-27a polymorphism and total alcohol intake, this being higher in GG subjects (5.2 +/- 0.4 in AA, 5.9 +/- 0.5 in AG and 9.1 +/- 1.8 g/day in GG; p(adjusted) = 0.019). We also found a statistically-significant association of the pre-miR-27a polymorphism with the risk of having a high alcohol intake (> 2 drinks/day in men and > 1 in women): 5.9% in AA versus 17.5% in GG; p(adjusted) < 0.001. In the sensitivity analysis, this association was homogeneous for sex, obesity and Mediterranean diet adherence. In conclusion, we report for the first time a significant association between a miRNA polymorphism (rs895819) and daily alcohol consumption.
[Barragan, Rocio; Asensio, Eva M.; Frances, Francesc; Sorli, Jose V.; Corella, Dolores] Univ Valencia, Sch Med, Dept Prevent Med & Publ Hlth, Valencia 46010, Spain; [Barragan, Rocio; Coltell, Oscar; Asensio, Eva M.; Frances, Francesc; Sorli, Jose V.; Estruch, Ramon; Salas-Huetos, Albert; Corella, Dolores] Inst Salud Carlos III, CIBER Fisiopatol Obesidad & Nutr, Madrid 28029, Spain; [Coltell, Oscar] Univ Jaume 1, Sch Technol & Expt Sci, Dept Comp Languages & Syst, Castellon de La Plana 12071, Spain; [Estruch, Ramon] Hosp Clin Barcelona, IDIBAPS, Dept Internal Med, Barcelona 08036, Spain; [Salas-Huetos, Albert] Univ Rovira & Virgili, IISPV, Dept Biochem & Biotechnol, Human Nutr Unit, Reus 43003, Spain; [Ordovas, Jose M.] Ctr Nacl Invest Cardiovasc CNIC, Dept Cardiovasc Epidemiol & Populat Genet, Madrid 28029, Spain; [Ordovas, Jose M.] IMDEA Alimentac, Madrid 28049, Spain; [Ordovas, Jose M.] Tufts Univ, Nutr & Genom Lab, JM USDA Human Nutr Res Ctr Aging, Boston, MA 02111 USA University of Valencia; CIBER - Centro de Investigacion Biomedica en Red; CIBEROBN; Instituto de Salud Carlos III; Universitat Jaume I; University of Barcelona; Hospital Clinic de Barcelona; IDIBAPS; Universitat Rovira i Virgili; Institut d'Investigacio Sanitaria Pere Virgili (IISPV); Centro Nacional de Investigaciones Cardiovasculares (CNIC); United States Department of Agriculture (USDA); Tufts University Corella, D (corresponding author), Univ Valencia, Sch Med, Dept Prevent Med & Publ Hlth, Valencia 46010, Spain.; Corella, D (corresponding author), Inst Salud Carlos III, CIBER Fisiopatol Obesidad & Nutr, Madrid 28029, Spain. rocio.barragan@uv.es; oscar.coltell@uji.es; eva.m.asensio@uv.es; francesc.frances@uv.es; jose.sorli@uv.es; RESTRUCH@clinic.cat; albert.salas@uab.cat; jose.ordovas@tufts.edu;

dolores.corella@uv.es Estruch, Ramon/AAZ-3723-2020; Coltell, Oscar/AAA-9936-2019; Sorlí, José/L-8758-2014; Salas-Huetos, Albert/A-8509-2011; Corella, Dolores/L-9888-2014; Coltell, Oscar/L-8549-2014 BARRAGAN-ARNAL, ROCIO/0000-0003-0917-7251; Estruch, Ramon/0000-0003-1260-4445; Salas-Huetos, Albert/0000-0001-5914-6862; Corella, Dolores/0000-0002-2366-4104; Sorli, Jose V/0000-0002-0130-2006; Coltell, Oscar/0000-0002-4518-8495
Spanish Ministry of Health (Instituto de Salud Carlos III); Ministerio de Economia y Competitividad-Fondo Europeo de Desarrollo Regional [CNIC-06/2007, RTIC G03/140, CIBER 06/03, PI06-1326, PI07-0954, PI11/02505, SAF2009-12304, AGL2010-22319-C03-03, PRX14/00527]; lUniversity Jaume I [P1-1B2013-54, 53-K06-5-10, 58-1950-9-001]; U.S. Department of Agriculture Research Service, USA; Generalitat Valenciana [ACOMP2010-181, AP111/10, AP-042/11, ACOM2011/145, ACOMP/2012/190, ACOMP/2013/159, ACOMP/213/165]; Colegio Complutense at Harvard University, Cambridge. MA, USA; Conselleria d'Educacio, Investigacio, Cultura i Esport. Generalitat Valenciana, Spain [ACIF/2013/168] Spanish Ministry of Health (Instituto de Salud Carlos III)(Instituto de Salud Carlos IIISpanish Government); Ministerio de Economia y Competitividad-Fondo Europeo de Desarrollo Regional(Spanish Government); lUniversity Jaume I; U.S. Department of Agriculture Research Service, USA(United States Department of Agriculture (USDA)); Generalitat Valenciana(Center for Forestry Research & Experimentation (CIEF)); Colegio Complutense at Harvard University, Cambridge. MA, USA; Conselleria d'Educacio, Investigacio, Cultura i Esport. Generalitat Valenciana, Spain This study was funded, by the Spanish Ministry of Health (Instituto de Salud Carlos III) and the Ministerio de Economia y Competitividad-Fondo Europeo de Desarrollo Regional (Projects CNIC-06/2007, RTIC G03/140, CIBER 06/03, PI06-1326, PI07-0954, PI11/02505, SAF2009-12304, AGL2010-22319-C03-03 and PRX14/00527), by the lUniversity Jaume I (Project P1-1B2013-54), by Contracts 53-K06-5-10 and 58-1950-9-001 from the U.S. Department of Agriculture Research Service, USA, by the Generalitat Valenciana (ACOMP2010-181, AP111/10, AP-042/11, ACOM2011/145, ACOMP/2012/190, ACOMP/2013/159 and ACOMP/213/165), and with the collaboration of the Real Colegio Complutense at Harvard University, Cambridge. MA, USA. Rocio Barragon's contract is funded by the Ayudas para la contratacion de personal investigador en formacion de caracter predoctoral, Programa "VALencia Investigacion mas Desarrollo" (VALi+d). Conselleria d'Educacio, Investigacio, Cultura i Esport. Generalitat Valenciana, Spain (ACIF/2013/168). 56 8 10 0 15 MDPI BASEL ST ALBAN-ANLAGE 66, CH-4052 BASEL, SWITZERLAND 1422-0067 INT J MOL SCI Int. J. Mol. Sci. AUG 2016 17 8 1338 10.3390/ijms17081338 http://dx.doi.org/10.3390/ijms17081338 18 Biochemistry & Molecular Biology; Chemistry, Multidisciplinary Science Citation Index Expanded (SCI-EXPANDED) Biochemistry & Molecular Biology; Chemistry DU6PU 27537871 Green Published, gold, Green Submitted 2025-06-24 WOS:000382337900150
J Shlapeko, EA; Stepanova, SV Shlapeko, Ekaterina A.; Stepanova, Svetlana V. CULTURAL INTEGRATION: POSITIVE AND NEGATIVE PERCEPTIONS (CASE OF TORNIO, FINLAND) GEOGRAPHIA POLONICA English Article cultural integration; migration;

newcomer; asylum seeker; host community; Tornio IMMIGRANT INCORPORATION; ACCULTURATION; ASSIMILATION; MIGRATION Increase in the migration flows has become a challenge in the world today. In Finland there is considerable shift in the number of migrants from Africa and the Middle East countries. The paper is based on the sociological research conducted in August-September, 2016 in Tornio (Finland). The empirical observations were gained from the interviews with 12 migrants and the questionnaires on the city Tornio attractiveness (73 locals) and the human mobility challenges (89 locals). The obtained results highlight the significant role of the communication activities with the joint participation of locals and newcomers in understanding each others' culture, decreasing negative perceptions and reactions in the integration process. [Shlapeko, Ekaterina A.; Stepanova, Svetlana V.] Russian Acad Sci, Karelian Res Ctr, 50 A Nevskogo St, Petrozavodsk 185030, Russia Russian Academy of Sciences; Karelian Research Centre of the Russian Academy of Sciences Shlapeko, EA (corresponding author), Russian Acad Sci, Karelian Res Ctr, 50 A Nevskogo St, Petrozavodsk 185030, Russia. shlapeko_kate@mail.ru; svkorka@mail.ru Kondrateva, Svetlana/H-3097-2015; Shlapeko, Ekaterina/L-8234-2017 Shlapeko, Ekaterina/0000-0003-3518-4543; Kondrateva (Stepanova), Svetlana/0000-0001-8832-9182Ministry of Education and Culture of Finland; European Cultural Foundation Ministry of Education and Culture of Finland; European Cultural Foundation We express our gratitude towards Maria Dyakonova (Institute of Economics KarRC of RAS), Marika Kurth (Finnish Red Cross, Tornio Reception center for asylum seekers), Malla Alatalo (Art Promotion Center Finland) for the fruitful collaboration as well as to the Finnish Red Cross volunteers and storytellers who helped us during the research activities in 2016. We also thank Juha Franti (Tornio City Social Department) for his statistical advice, Helena Junes (Tornio City Cultural Office) and Elena Kaukonen (Tornio Finnish-Russian Society) for the help in conducting sociological surveys. Special thanks to Stephen G F Hall, PhD candidate at University College London (UCL) and John Dawton (LVC, London School of English) for thorough language review of the text. This publication is a part of the research works conducted at the Institute of Economics of the Karelian Research Center of the Russian Academy of Sciences that was supported by the Ministry of Education and Culture of Finland as well as European Cultural Foundation. 50 3 3 0 10 POLISH ACAD SCIENCES, INST GEOGRAPHY & SPATIAL ORGANIZATION WARSAW TWARDA 51-55, WARSAW, 00818, POLAND 0016-7282 2300-7362 GEOGR POL Geogr. Pol. 2018 91 3 301 315 10.7163/GPol.0122 http://dx.doi.org/10.7163/GPol.0122 15 Geography Emerging Sources Citation Index (ESCI) Geography HA7IJ 2025-06-24 WOS:000450454400003

J Stone, RAT; Waring, ME; Cutrona, SL; Kiefe, CI; Allison, J; Doubeni, CA Stone, Rosalie A. Torres; Waring, Molly E.; Cutrona, Sarah L.; Kiefe, Catarina I.; Allison, Jeroan; Doubeni, Chyke A. The association of dietary quality with colorectal cancer among normal weight, overweight and obese men and women: a prospective longitudinal study in the USA BMJ OPEN English Article FOOD-FREQUENCY QUESTIONNAIRE; HEALTH-AMERICAN-ASSOCIATION; RETIRED-PERSONS DIET; NIH-AARP DIET;

MEDITERRANEAN DIET; UNITED-STATES; NATIONAL-INSTITUTES; RECTAL-CANCER; RISK-FACTORS; ALL-CAUSE Objective Lower body mass index (BMI) and higher dietary quality reduce the risk of colorectal cancer (CRC). A full understanding of how these associations vary by sex and weight is lacking. Methods We used data from the National Institutes of Health - American Association of Retired Persons (NIH)-AARP) Diet and Health Study for 398 458 persons who were 50-71 years old in 1995-1996 and followed through 2006. Exposures were dietary quality as reflected by the Mediterranean Diet, the Healthy Eating Index-2010 and the Dietary Approaches to Stop Hypertension score, stratified by BMI category. The outcome was CRC diagnosis from cancer registry data. Cox regression models were adjusted for disease risk factors. Results Over a mean duration of 123 months of follow-up, there were 6515 new diagnoses of CRC (1953 among the normal weight, 2924 among the overweight and 1638 among the obese; 4483 among men and 2032 among women). For normal weight and overweight men, we found a strong dose-response pattern for the association of increasing quintile of dietary quality with decreasing risk of CRC; this pattern was observed for obese men as well, but less consistently across the three measures of dietary quality. The findings were of smaller magnitude and less consistent for women but still suggesting associations of similar direction. Conclusion We observed that increased dietary quality was associated with lower risk of incident CRC up to 10 years later for men regardless of baseline weight category. [Stone, Rosalie A. Torres] Clark Univ, Dept Sociol, Worcester, MA 01610 USA; [Stone, Rosalie A. Torres] Univ Massachusetts, Med Sch, Syst & Psychosocial Adv Res Ctr, Dept Psychiat, Shrewsbury, MA 01545 USA; [Waring, Molly E.; Kiefe, Catarina I.; Allison, Jeroan] Univ Massachusetts, Med Sch, Dept Quantitat Hlth Sci, Worcester, MA USA; [Cutrona, Sarah L.] Univ Massachusetts, Med Sch, Dept Med, Worcester, MA USA; [Doubeni, Chyke A.] Univ Penn, Perelman Sch Med, Dept Family Med & Community Hlth, Philadelphia, PA 19104 USA; [Doubeni, Chyke A.] Univ Penn, Perelman Sch Med, Ctr Clin Epidemiol & Biostat, Philadelphia, PA 19104 USA Clark University; University of Massachusetts System; University of Massachusetts System; University of Massachusetts Worcester; University of Massachusetts System; University of Massachusetts Worcester; University of Pennsylvania; University of Pennsylvania   Stone, RAT (corresponding author), Clark Univ, Dept Sociol, Worcester, MA 01610 USA.; Stone, RAT (corresponding author), Univ Massachusetts, Med Sch, Syst & Psychosocial Adv Res Ctr, Dept Psychiat, Shrewsbury, MA 01545 USA.
rtorresstone@clarku.edu   Doubeni, Chyke/W-6749-2019
Torres Stone, Rosalie/0000-0002-1939-2046 National Cancer Institute at the National Institutes of Health [U01-CA1517361]; National Institute of Minority Health; Health Disparities of National Institutes of Health [P60MD006912]; NIH [KL2TR000160, U01HL105268]; National Center for Advancing Translational Sciences of National Institutes of Health [KL2TR000160]   National Cancer Institute at the National Institutes of Health(United States Department of Health & Human ServicesNational Institutes of Health (NIH) - USANIH National Cancer Institute (NCI)); National Institute of Minority Health; Health Disparities of National Institutes of Health; NIH(United States Department of Health & Human ServicesNational Institutes of Health (NIH) - USA); National Center for Advancing Translational Sciences of National Institutes of Health The content of this manuscript was developed with

funding from the National Cancer Institute at the National Institutes of Health (U01-CA1517361, PI: Doubeni). The contents of this manuscript do not necessarily reflect the views of the funding agencies and you should not assume endorsement by the Federal Government. Research reported in this publication was supported by the National Institute of Minority Health and Health Disparities of the National Institutes of Health under Award Number P60MD006912 (PI: Allison). The content is solely the responsibility of the authors and does not necessarily represent the official views of the National Institutes of Health. Partial support for Dr Waring provided by NIH grants KL2TR000160 and U01HL105268. Dr Cutrona was supported by the National Center for Advancing Translational Sciences of the National Institutes of Health under award number KL2TR000160. The content is solely the responsibility of the authors and does not necessarily represent the official views of the NIH. 51 43 44 1 6
BMJ PUBLISHING GROUP LONDON BRITISH MED ASSOC HOUSE, TAVISTOCK SQUARE, LONDON WC1H 9JR, ENGLAND 2044-6055
BMJ OPEN BMJ Open JUN 2017 7 6
e015619 10.1136/bmjopen-2016-015619
http://dx.doi.org/10.1136/bmjopen-2016-015619
11 Medicine, General & InternalScience Citation Index Expanded (SCI-EXPANDED); Social Science Citation Index (SSCI)
General & Internal Medicine FB8LT 28679675 Green Published, Green Submitted, gold 2025-06-24
WOS:000406391200173
J Molina-Montes, E; Sánchez, MJ; Buckland, G; Bueno-de-Mesquita, HB; Weiderpass, E; Amiano, P; Wark, PA; Kühn, T; Katzke, V; Huerta, JM; Ardanaz, E; Quirós, JR; Affret, A; His, M; Boutron-Ruault, MC; Peeters, PH; Ye, WM; Sund, M; Boeing, H; Iqbal, K; Ohlsson, B; Sonestedt, E; Tjonneland, A; Petersen, KEN; Travis, RC; Skeie, G; Agnoli, C; Panico, S; Palli, D; Tumino, R; Sacerdote, C; Freisling, H; Huybrechts, I; Overvad, K; Trichopoulou, A; Bamia, C; Vasilopoulou, E; Wareham, N; Khaw, KT; Cross, AJ; Ward, HA; Riboli, E; Duell, EJ
Molina-Montes, Esther; Sanchez, Maria-Jose; Buckland, Genevieve; Bueno-de-Mesquita, H. B(as); Weiderpass, Elisabete; Amiano, Pilar; Wark, Petra A.; Kuehn, Tilman; Katzke, Verena; Maria Huerta, Jose; Ardanaz, Eva; Ramon Quiros, Jose; Affret, Aurelie; His, Mathilde; Boutron-Ruault, Marie-Christine; Peeters, Petra H.; Ye, Weimin; Sund, Malin; Boeing, Heiner; Iqbal, Khalid; Ohlsson, Bodil; Sonestedt, Emily; Tjonneland, Anne; Petersen, Kristina E. N.; Travis, Ruth C.; Skeie, Guri; Agnoli, Claudia; Panico, Salvatore; Palli, Domenico; Tumino, Rosario; Sacerdote, Carlotta; Freisling, Heinz; Huybrechts, Inge; Overvad, Kim; Trichopoulou, Antonia; Bamia, Christina; Vasilopoulou, Effie; Wareham, Nick; Khaw, Kay-Tee; Cross, Amanda J.; Ward, Heather A.; Riboli, Elio; Duell, Eric J. Mediterranean diet and risk of pancreatic cancer in the European Prospective Investigation into Cancer and Nutrition cohort BRITISH JOURNAL OF CANCER English Article
Mediterranean diet; pancreatic cancer; cohort study
BREAST-CANCER; ADHERENCE; PATTERNS; METAANALYSIS; CONSUMPTION; COUNTRIES; MORTALITY; DEATHS; SCORE; MEAT
Background: The Mediterranean diet (MD) has been proposed as a means for cancer prevention, but little evidence has been accrued regarding its potential to prevent pancreatic cancer. We investigated the association between the adherence to the MD and

pancreatic cancer risk within the European Prospective Investigation into Cancer and Nutrition (EPIC) cohort. Methods: Over half a million participants from 10 European countries were followed up for over 11 years, after which 865 newly diagnosed exocrine pancreatic cancer cases were identified. Adherence to the MD was estimated through an adapted score without the alcohol component (arMED) to discount alcohol-related harmful effects. Cox proportional hazards regression models, stratified by age, sex and centre, and adjusted for energy intake, body mass index, smoking status, alcohol intake and diabetes status at recruitment, were used to estimate hazard ratios (HRs) associated with pancreatic cancer and their corresponding 95% confidence intervals (CIs). Results: Adherence to the arMED score was not associated with risk of pancreatic cancer (HR high vs low adherence = 0.99; 95% CI: 0.77-1.26, and HR per increments of two units in adherence to arMED = 1.00; 95% CI: 0.94-1.06). There was no convincing evidence for heterogeneity by smoking status, body mass index, diabetes or European region. There was also no evidence of significant associations in analyses involving microscopically confirmed cases, plausible reporters of energy intake or other definitions of the MD pattern. Conclusions: A high adherence to the MD is not associated with pancreatic cancer risk in the EPIC study.

[Molina-Montes, Esther] Spanish Natl Canc Res Ctr CNIO, Genet & Mol Epidemiol Grp, Madrid, Spain; [Molina-Montes, Esther; Sanchez, Maria-Jose] Univ Granada, Andalusian Sch Publ Hlth, Hosp Univ Granada, Inst Invest Biosanitaria Ibs GRANADA, Granada, Spain; [Molina-Montes, Esther; Sanchez, Maria-Jose] CIBER Epidemiol Salud Publ, CIBERESP, Madrid, Spain; [Buckland, Genevieve; Duell, Eric J.] Catalan Inst Oncol ICO IDIBELL, Unit Nutr & Canc, Canc Epidemiol Res Programme, Barcelona, Spain; [Bueno-de-Mesquita, H. B(as)] Natl Inst Publ Hlth & Environm RIVM, Dept Determinants Chron Dis DCD, Bilthoven, Netherlands; [Bueno-de-Mesquita, H. B(as)] Univ Med Ctr, Dept Gastroenterol & Hepatol, Utrecht, Netherlands; [Bueno-de-Mesquita, H. B(as); Peeters, Petra H.; Cross, Amanda J.; Ward, Heather A.; Riboli, Elio] Imperial Coll London, Sch Publ Hlth, Dept Epidemiol & Biostat, London, England; [Bueno-de-Mesquita, H. B(as)] Univ Malaya, Dept Social Prevent Med, Fac Med, Kuala Lumpur, Malaysia; [Weiderpass, Elisabete] Univ Tromso, Arctic Univ Norway, Dept Community Med, Fac Hlth Sci, Tromso, Norway; [Weiderpass, Elisabete] Canc Registry Norway, Inst Populat Based Canc Res, Dept Res, Oslo, Norway; [Weiderpass, Elisabete] Karolinska Inst, Dept Med Epidemiol & Biostat, Stockholm, Sweden; [Weiderpass, Elisabete] Folkhalsan Res Ctr, Genet Epidemiol Grp, Helsinki, Finland; [Amiano, Pilar] BioDonostia Res Inst, Publ Hlth Div Gipuzkoa, San Sebastian, Spain; [Wark, Petra A.] Imperial Coll London, Sch Publ Hlth, Dept Primary Care & Publ Hlth, Global eHealth Unit, London, England; [Kuehn, Tilman; Katzke, Verena] German Canc Res Ctr DFKZ, Div Canc Epidemiol, Heidelberg, Germany; [Maria Huerta, Jose] Dept Epidemiol, Murcia Reg Hlth Council, IMIB Arrixaca, Murcia, Spain; [Ardanaz, Eva] Navarra Publ Hlth Inst, Pamplona, Spain; [Ardanaz, Eva] Navarra Inst Hlth Res, IdiSNA, Pamplona, Spain; [Maria Huerta, Jose] Publ Hlth Directorate, Asturias, Spain; [Affret, Aurelie] Univ Paris Sud, Univ Paris Saclay, UVSQ, CESP Generat & Hlth Team,INSERM, Villejuif, France; [Affret, Aurelie; His, Mathilde; Boutron-Ruault, Marie-Christine] Gustave Roussy, F-94805 Villejuif, France; [Affret, Aurelie; His, Mathilde; Boutron-Ruault, Marie-Christine] Univ Med Ctr Utrecht, Julius Ctr Hlth Sci

& Primary Care, Utrecht, Netherlands; [Ye, Weimin; Sund, Malin] Karolinska Inst, Dept Med Epidemiol & Biostat, Stockholm, Sweden; [Sund, Malin] Umea Univ, Med Biobank, Umea, Sweden; [Boeing, Heiner; Iqbal, Khalid] German Inst Human Nutr Potsdam Rehbrucke, Dept Epidemiol, Nuthetal, Germany; [Ohlsson, Bodil] Skane Univ Hosp, Dept Internal Med, Malmo, Sweden; [Ohlsson, Bodil; Sonestedt, Emily] Lund Univ, Dept Clin Sci, Malmo, Sweden; [Tjonneland, Anne; Petersen, Kristina E. N.] Danish Canc Soc Res Ctr, Unit Diet Genes & Environm, Copenhagen, Denmark; [Travis, Ruth C.] Univ Oxford, Canc Epidemiol Unit, Nuffield Dept Populat Hlth, Oxford, England; [Agnoli, Claudia] Fdn IRCCS Ist Nazl Tumori, Epidemiol & Prevent Unit, Milan, Italy; [Panico, Salvatore] Univ Naples Federico II, Dipartimento Med Clin & Chirurg, Naples, Italy; [Palli, Domenico] Canc Res & Prevent Inst ISPO, Mol & Nutr Epidemiol Unit, Florence, Italy; [Tumino, Rosario] ASP Ragusa, Civic MP Arezzo Hosp, Canc Registry & Histopathol Unit, Ragusa, Italy; [Sacerdote, Carlotta] Univ Turin, Citta Salute & Sci Hosp, Unit Canc Epidemiol, Turin, Italy; [Sacerdote, Carlotta] Ctr Canc Prevent CPO, Turin, Italy; [Huybrechts, Inge] IARC WHO, Sect Nutr & Metab, Lyon, France; [Overvad, Kim] Aarhus Univ, Epidemiol Sect, Dept Publ Hlth, Aarhus, Denmark; [Trichopoulou, Antonia; Bamia, Christina] Hellenic Hlth Fdn, Athens, Greece; [Trichopoulou, Antonia; Bamia, Christina; Vasilopoulou, Effie] Univ Athens, Dept Hyg Epidemiol & Med Stat, WHO Collaborating Ctr Nutr & Hlth, Unit Nutr Epidemiol & Nutr Publ Hlth,Sch Med, Athens, Greece; [Wareham, Nick] Med Res Council MCR, Epidemiol Unit, Cambridge, England; [Khaw, Kay-Tee] Univ Cambridge, Sch Clin Med, Cambridge, England Centro Nacional de Investigaciones Oncologicas (CNIO); University of Granada; Instituto de Investigacion Biosanitaria IBS Granada; Escuela Andaluza de Salud Publica; CIBER - Centro de Investigacion Biomedica en Red; CIBERESP; Institut Catala d'Oncologia; Institut d'Investigacio Biomedica de Bellvitge (IDIBELL); Netherlands National Institute for Public Health & the Environment; Utrecht University; Utrecht University Medical Center; Imperial College London; Universiti Malaya; UiT The Arctic University of Tromso; University of Oslo; Karolinska Institutet; Folkhalsan Research Center; Imperial College London; Helmholtz Association; German Cancer Research Center (DKFZ); Murcia Regional Health Council; Hospital Clinico Universitario Virgen de la Arrixaca; Public Health Institute of Navarra; University of Navarra; Institut National de la Sante et de la Recherche Medicale (Inserm); Universite Paris Saclay; UNICANCER; Gustave Roussy; Utrecht University; Utrecht University Medical Center; Karolinska Institutet; Umea University; Leibniz Association; Deutsches Institut fur Ernahrungsforschung Potsdam-Rehbrucke (DIfE); Lund University; Skane University Hospital; Lund University; Danish Cancer Society; University of Oxford; Fondazione IRCCS Istituto Nazionale Tumori Milan; University of Naples Federico II; ISPRO Istituto per lo studio, la prevenzione e la rete oncologica; Civile M.P. Arezzo Hospital; University of Turin; A.O.U. Citta della Salute e della Scienza di Torino; World Health Organization; International Agency for Research on Cancer (IARC); Aarhus University; National & Kapodistrian University of Athens; Athens Medical School; University of Cambridge Sánchez, MJ (corresponding author), Univ Granada, Andalusian Sch Publ Hlth, Hosp Univ Granada, Inst Invest Biosanitaria Ibs GRANADA, Granada, Spain.; Sánchez, MJ (corresponding author), CIBER Epidemiol Salud Publ,

CIBERESP, Madrid, Spain.
mariajose.sanchez.easp@juntadeandalucia.es Huybrechts, Inge/ITT-7052-2023; Sacerdote, Carlotta/K-3611-2018; Affret, Aurélie/T-1297-2019; Tjonneland, Anne/AGU-0320-2022; Iqbal, Khalid/AAD-7112-2022; Khaw, Kay-Tee/AAZ-3209-2021; Kühn, Tilman/JYP-5102-2024; TRICHOPOULOU, ANTONIA/ABF-8727-2021; Sonestedt, Emily/I-3814-2016; Boutron-Ruault, Marie-Christine/H-3936-2014; Panico, Salvatore/K-6506-2016; Ye, Weimin/A-5939-2008; Riboli, Elio/A-4357-2009; Huerta, Jose Maria/N-8654-2015; Molina-Montes, Esther/AFS-7568-2022; Malats, Nuria/H-7041-2015; Weiderpass, Elisabete/M-4029-2016; Agnoli, Claudia/K-5916-2016; Wark, Petra/C-1752-2016; SANCHEZ-PEREZ, MARIA JOSE/D-1087-2011
Huerta, Jose Maria/0000-0002-9637-3869; Ward, Heather/0000-0002-2721-2404; Molina-Montes, Esther/0000-0002-0428-2426; Freisling, Heinz/0000-0001-8648-4998; Malats, Nuria/0000-0003-2538-3784; Weiderpass, Elisabete/0000-0003-2237-0128; Ardanaz, Eva/0000-0001-8434-2013; Overvad, Kim/0000-0001-6429-7921; Iqbal, Khalid/0000-0002-3312-4259; Kuhn, Tilman/0000-0001-7702-317X; Riboli, Elio/0000-0001-6795-6080; tumino, rosario/0000-0003-2666-414X; Buckland, Genevieve/0000-0003-2060-6598; Agnoli, Claudia/0000-0003-4472-1179; Wark, Petra/0000-0003-1020-4640; His, Mathilde/0000-0003-3850-8291; SANCHEZ-PEREZ, MARIA JOSE/0000-0003-4817-0757; Sacerdote, Carlotta/0000-0002-8008-5096; Skeie, Guri/0000-0003-2476-4251; Tjonneland, Anne/0000-0003-4385-2097
European Commission (DG-SANCO); International Agency for Research on Cancer; Danish Cancer Society (Denmark); Ligue Contre le Cancer; Institut Gustave Roussy; Mutuelle Generale de l'Education Nationale; Institut National de la Sante et de la Recherche Medicale (INSERM) (France); German Cancer Aid; German Cancer Research Center (DKFZ); Federal Ministry of Education and Research (BMBF); Cancer Research Center (DKFZ); Deutsche Krebshilfe; Deutsches Krebsforschungszentrum; Federal Ministry of Education and Research (Germany); Hellenic Health Foundation (Greece); Associazione Italiana per la Ricerca sul Cancro-AIRC-Italy; National Research Council (Italy); Dutch Ministry of Public Health, Welfare and Sports (VWS); Netherlands Cancer Registry (NKR); LK Research Funds; Dutch Prevention Funds; Dutch ZON (Zorg Onderzoek Nederland); World Cancer Research Fund (WCRF); Statistics Netherlands (the Netherlands); Nordforsk, Nordic Centre of Excellence programme on Food, Nutrition and Health (Norway) [ERC-2009-AdG 232997]; Health Research Fund (FIS) - ERDF [PI12/00002, PI13/00061, PI13/01162]; Regional Governments of Andalucia; Asturias; Basque Country; Murcia; Navarra; ISCIII RETIC (Spain) [RD06/0020]; WCR [15-0391]; Swedish Cancer Society; Swedish Research Council; County Councils of Skane and Vasterbotten (Sweden); Cancer Research UK [14136, C570/A16491, C8221/A19170]; Medical Research Council (UK) [1000143, MR/M012190/1]; MRC [MR/N003284/1] Funding Source: UKRI European Commission (DG-SANCO)(European Union (EU)European Commission Joint Research Centre); International Agency for Research on Cancer; Danish Cancer Society (Denmark)(Danish Cancer Society); Ligue Contre le Cancer(Ligue nationale contre le cancer); Institut Gustave Roussy; Mutuelle Generale de l'Education Nationale; Institut National de la Sante et de la Recherche Medicale (INSERM) (France)(Institut National de la Sante et de la Recherche Medicale (Inserm)); German Cancer Aid(Deutsche Krebshilfe); German Cancer Research Center (DKFZ)(Helmholtz Association); Federal Ministry of Education and Research (BMBF)(Federal Ministry of Education &

Research (BMBF)); Cancer Research Center (DKFZ); Deutsche Krebshilfe(Deutsche Krebshilfe); Deutsches Krebsforschungszentrum; Federal Ministry of Education and Research (Germany)(Federal Ministry of Education & Research (BMBF)); Hellenic Health Foundation (Greece); Associazione Italiana per la Ricerca sul Cancro-AIRC-Italy(Fondazione AIRC per la ricerca sul cancro); National Research Council (Italy)(Consiglio Nazionale delle Ricerche (CNR)); Dutch Ministry of Public Health, Welfare and Sports (VWS); Netherlands Cancer Registry (NKR); LK Research Funds; Dutch Prevention Funds; Dutch ZON (Zorg Onderzoek Nederland)(Netherlands Organization for Scientific Research (NWO)); World Cancer Research Fund (WCRF)(World Cancer Research Fund International (WCRF)); Statistics Netherlands (the Netherlands)(Netherlands Government); Nordforsk, Nordic Centre of Excellence programme on Food, Nutrition and Health (Norway)(NordForsk); Health Research Fund (FIS) - ERDF; Regional Governments of Andalucia; Asturias(Principality of Asturias); Basque Country(Basque Government); Murcia; Navarra; ISCIII RETIC (Spain)(Instituto de Salud Carlos III); WCR; Swedish Cancer Society(Swedish Cancer Society); Swedish Research Council(Swedish Research Council); County Councils of Skane and Vasterbotten (Sweden); Cancer Research UK(Cancer Research UK); Medical Research Council (UK)(UK Research & Innovation (UKRI)Medical Research Council UK (MRC)); MRC(UK Research & Innovation (UKRI)Medical Research Council UK (MRC)) EM is thankful to Dr Nuria Malats of the Spanish National Cancer Research Center (CNIO) for her critical comments on this paper. The coordination of EPIC is financially supported by the European Commission (DG-SANCO) and the International Agency for Research on Cancer. The national cohorts are supported by Danish Cancer Society (Denmark); Ligue Contre le Cancer, Institut Gustave Roussy, Mutuelle Generale de l'Education Nationale, Institut National de la Sante et de la Recherche Medicale (INSERM) (France); German Cancer Aid, German Cancer Research Center (DKFZ), Federal Ministry of Education and Research (BMBF), Deutsche Krebshilfe, Deutsches Krebsforschungszentrum and Federal Ministry of Education and Research (Germany); The Hellenic Health Foundation (Greece); Associazione Italiana per la Ricerca sul Cancro-AIRC-Italy and National Research Council (Italy); Dutch Ministry of Public Health, Welfare and Sports (VWS), Netherlands Cancer Registry (NKR), LK Research Funds, Dutch Prevention Funds, Dutch ZON (Zorg Onderzoek Nederland), World Cancer Research Fund (WCRF), Statistics Netherlands (the Netherlands); ERC-2009-AdG 232997 and Nordforsk, Nordic Centre of Excellence programme on Food, Nutrition and Health (Norway); Health Research Fund (FIS), PI12/00002 co-funded by ERDF, PI13/00061 to Granada, PI13/01162 to Murcia, Regional Governments of Andalucia, Asturias, Basque Country, Murcia and Navarra, ISCIII RETIC (RD06/0020; Spain); WCR (15-0391); Swedish Cancer Society, Swedish Research Council and County Councils of Skane and Vasterbotten (Sweden); Cancer Research UK (14136 to EPIC-Norfolk; C570/A16491 and C8221/A19170 to EPIC-Oxford), Medical Research Council (1000143 to EPIC-Norfolk, MR/M012190/1 to EPIC-Oxford; UK).   40   25   27
      0   15   NATURE PUBLISHING GROUP   LONDON   MACMILLAN BUILDING, 4 CRINAN ST, LONDON N1 9XW, ENGLAND   0007-0920
      1532-1827   BRIT J CANCER   Br. J. Cancer   MAR 16
      2017 116   6   811   820
      10.1038/bjc.2017.14   http://dx.doi.org/10.1038/bjc.2017.14

10 Oncology Science Citation Index Expanded (SCI-EXPANDED) Oncology EP5RK 28170373 hybrid, Green Published 2025-06-24 WOS:000397436100017

J Godos, J; Bella, F; Sciacca, S; Galvano, F; Grosso, G Godos, J.; Bella, F.; Sciacca, S.; Galvano, F.; Grosso, G. Vegetarianism and breast, colorectal and prostate cancer risk: an overview and meta-analysis of cohort studies JOURNAL OF HUMAN NUTRITION AND DIETETICS English Article breast cancer; colorectal cancer; epidemiology; meta-analysis; prostate cancer; vegetarian diet; vegetarianism LOW MEAT CONSUMPTION; POPULATION-BASED COHORT; DIETARY PATTERNS; MEDITERRANEAN DIET; LIFE-STYLE; HEALTH; DYSLIPIDEMIA; PREVENTION; VEGETABLES; HUMANS

BackgroundVegetarian diets may be associated with certain benefits toward human health, although current evidence is scarce and contrasting. In the present study, a systematic review and meta-analysis of prospective cohort studies was performed with respect to the association between vegetarian diets and breast, colorectal and prostate cancer risk. MethodsStudies were systematically searched in Pubmed and EMBASE electronic databases. Eligible studies had a prospective design and compared vegetarian, semi- and pesco-vegetarian diets with a non-vegetarian diet. Random-effects models were applied to calculate relative risks (RRs) of cancer between diets. Statistical heterogeneity and publication bias were explored. ResultsA total of nine studies were included in the meta-analysis. Studies were conducted on six cohorts accounting for 686 629 individuals, and 3441, 4062 and 1935 cases of breast, colorectal and prostate cancer, respectively. None of the analyses showed a significant association of vegetarian diet and a lower risk of either breast, colorectal, and prostate cancer compared to a non-vegetarian diet. By contrast, a lower risk of colorectal cancer was associated with a semi-vegetarian diet (RR = 0.86, 95% confidence interval = 0.79-0.94; I-2 = 0%, P-heterogeneity = 0.82) and a pesco-vegetarian diet (RR = 0.67, 95% confidence interval = 0.53, 0.83; I-2 = 0%, P-heterogeneity = 0.46) compared to a non-vegetarian diet. The subgroup analysis by cancer localisation showed no differences in summary risk estimates between colon and rectal cancer. ConclusionsA summary of the existing evidence from cohort studies on vegetarian diets showed that complete exclusion of any source of protein from the diet is not associated with further benefits for human health.

[Godos, J.; Bella, F.; Sciacca, S.; Grosso, G.] Azienda Osped Univ Policlin Vittorio Emanuele, Integrated Canc Registry Catania Messina Siracusa, Catania, Italy; [Galvano, F.] Univ Catania, Dept Biomed & Biotechnol Sci, Catania, Italy Azienda Ospedaliera Universitaria Policlinico Vittorio Emanuele Presidio Ferraotto; University of Catania; University Catania Hospital; University of Catania Grosso, G (corresponding author), Integrated Canc Registry Catania Messina Siracusa, Via S Sofia 85, I-95123 Catania, Italy. giuseppe.grosso@studium.unict.it Galvano, Fabio/JSL-7451-2023; Godos, Justyna/AAC-1302-2019; Sciacca, Salvatore/B-2778-2011; Grosso, Giuseppe/K-6730-2016; Galvano, Fabio/F-8122-2010 Sciacca, Salvatore/0000-0002-8613-6296; Godos, Justyna/0000-0002-5809-5706; Grosso, Giuseppe/0000-0003-3930-5285; Galvano, Fabio/0000-0003-0644-0755

48 71 73 0 80 WILEY HOBOKEN 111 RIVER ST, HOBOKEN 07030-5774, NJ USA 0952-3871 1365-277X J HUM NUTR DIET J. Hum. Nutr. Diet. JUN 2017 30 3

349 359 10.1111/jhn.12426
http://dx.doi.org/10.1111/jhn.12426 11
Nutrition & Dietetics Science Citation Index Expanded (SCI-EXPANDED) Nutrition & Dietetics ET9ED 27709695
2025-06-24 WOS:000400608800010
J Clemens, MA Clemens, Michael A.
Global Skill Partnerships: a proposal for technical training in a mobile world IZA JOURNAL OF LABOR POLICY
English Article Brain drain; Development; Education; Finance; Health; Nurse; Eldercare; Mobility; Aging; Migration BRAIN-DRAIN; PHYSICIAN ASSISTANTS; NURSE-PRACTITIONERS; HEALTH-CARE; WORKFORCE Skilled workers emigrate from developing countries in rising numbers, raising fears of a drain on the human and financial resources of the countries they leave. This paper critiques existing policy proposals to address the development effects of skilled migration. It then proposes a new kind of ex ante public-private agreement to link skill formation and skilled migration for the mutual benefit of origin countries, destination countries, and migrants: 'Global Skill Partnerships'. The paper describes how such an agreement might work in one profession (nursing) and one region (North Africa), and offers design lessons from related initiatives around the world. [Clemens, Michael A.] Ctr Global Dev, 2055 L St NW,5th Floor, Washington, DC 20036 USA; [Clemens, Michael A.] IZA Inst Study Labor, D-53113 Bonn, Germany IZA Institute Labor Economics Clemens, MA (corresponding author), Ctr Global Dev, 2055 L St NW,5th Floor, Washington, DC 20036 USA.
mclemens@cgdev.org World Bank ILM program at the Center for Mediterranean Integration; John D. and Catherine T. MacArthur Foundation; William and Flora Hewlett Foundation; Nordic Trust Fund for Human Rights; Good Ventures World Bank ILM program at the Center for Mediterranean Integration; John D. and Catherine T. MacArthur Foundation; William and Flora Hewlett Foundation; Nordic Trust Fund for Human Rights; Good Ventures This research was supported by the World Bank ILM program at the Center for Mediterranean Integration under the overall supervision of Manjula Luthria, and received additional support from the John D. and Catherine T. MacArthur Foundation, the William and Flora Hewlett Foundation, the Nordic Trust Fund for Human Rights, and Good Ventures. Marla Spivack, Tejaswi Velayudhan, Christian Meyer, and Nabil Hashmi provided excellent research assistance. Helpful ideas also came from Steffen Angenendt, Ottilie Balz, Stefan Bethe, Nancy Birdsall, Satish Chand, Stephan Hillebrand, Stephen Howes, Natasha Iskander, Benjamin Leo, Akiko Maeda, Francisco Marmolejo, Aaditya Mattoo, Matthias Mayer, David McKenzie, Meiko Merda, Christal Morehouse, Mujobu Moyo, Caglar Ozden, Yann Pouget, Lant Pritchett, Andrea Riester, Kaye Schofield, Madeleine Sumption, Erwin Tiongson, Anna Wittenborg, and Astrid Ziebarth, as well as with participants in seminars at AusAID, the Bosch Foundation, the German Ministry for Economic Cooperation and Development, Rutgers University, and the Universite Internationale de Rabat. But the views in this paper and any errors are solely the responsibility of the author; they do not necessarily reflect the opinions of the Center for Global Development or the World Bank, their boards, or their funders. The author thanks the anonymous referee. 50 24 39 0 1 SCIENDO
WARSAW BOGUMILA ZUGA 32A, WARSAW, MAZOVIA, POLAND
2193-9004 IZA J LABOR POLICY IZA J. Labor

Policy		2015	4
	2	10.1186/s40173-014-0028-z
	http://dx.doi.org/10.1186/s40173-014-0028-z		18
	Industrial Relations & LaborEmerging Sources Citation Index (ESCI)	Business & Economics	VF3OY	Green Published, hybrid			2025-06-24	WOS:000442673800002
J	Assi, N; Moskal, A; Slimani, N; Viallon, V; Chajes, V; Freisling, H; Monni, S; Knueppel, S; Förster, J; Weiderpass, E; Lujan-Barroso, L; Amiano, P; Ardanaz, E; Molina-Montes, E; Salmerón, D; Quiórs, JR; Olsen, A; Tjonneland, A; Dahm, CC; Overvad, K; Dossus, L; Fournier, A; Baglietto, L; Fortner, RT; Kaaks, R; Trichopoulou, A; Bamia, C; Orfanos, P; De Magistris, MS; Masala, G; Agnoli, C; Ricceri, F; Tumino, R; de Mesquita, HBB; Bakker, MF; Peeters, PHM; Skeie, G; Braaten, T; Winkvist, A; Johansson, I; Khaw, KT; Wareham, NJ; Key, T; Travis, R; Schmidt, JA; Merritt, MA; Riboli, E; Romieu, I; Ferrari, P
	Assi, Nada; Moskal, Aurelie; Slimani, Nadia; Viallon, Vivian; Chajes, Veronique; Freisling, Heinz; Monni, Stefano; Knueppel, Sven; Foerster, Jana; Weiderpass, Elisabete; Lujan-Barroso, Leila; Amiano, Pilar; Ardanaz, Eva; Molina-Montes, Esther; Salmeron, Diego; Ramon Quiros, Jose; Olsen, Anja; Tjonneland, Anne; Dahm, Christina C.; Overvad, Kim; Dossus, Laure; Fournier, Agnes; Baglietto, Laura; Fortner, Renee Turzanski; Kaaks, Rudolf; Trichopoulou, Antonia; Bamia, Christina; Orfanos, Philippos; De Magistris, Maria Santucci; Masala, Giovanna; Agnoli, Claudia; Ricceri, Fulvio; Tumino, Rosario; de Mesquita, H. Bas Bueno; Bakker, Marije F.; Peeters, Petra H. M.; Skeie, Guri; Braaten, Tonje; Winkvist, Anna; Johansson, Ingegerd; Khaw, Kay-Tee; Wareham, Nicholas J.; Key, Tim; Travis, Ruth; Schmidt, Julie A.; Merritt, Melissa A.; Riboli, Elio; Romieu, Isabelle; Ferrari, Pietro			A treelet transform analysis to relate nutrient patterns to the risk of hormonal receptor-defined breast cancer in the European Prospective Investigation into Cancer and Nutrition (EPIC) PUBLIC HEALTH NUTRITION			English
	Article				Nutrient patterns; Treelet transform; Breast cancer; European Prospective Investigationinto Cancer and Nutrition; Principal component analysis	TOTAL-ENERGY INTAKE; DIETARY PATTERNS; ALCOHOL-CONSUMPTION; MEDITERRANEAN DIET; PROSPECTIVE COHORT; ASSOCIATION; DISEASE; PROJECT; WOMEN; LIFE	Objective Pattern analysis has emerged as a tool to depict the role of multiple nutrients/foods in relation to health outcomes. The present study aimed at extracting nutrient patterns with respect to breast cancer (BC) aetiology. Design Nutrient patterns were derived with treelet transform (TT) and related to BC risk. TT was applied to twenty-three log-transformed nutrient densities from dietary questionnaires. Hazard ratios (HR) and 95 % confidence intervals computed using Cox proportional hazards models quantified the association between quintiles of nutrient pattern scores and risk of overall BC, and by hormonal receptor and menopausal status. Principal component analysis was applied for comparison. Setting The European Prospective Investigation into Cancer and Nutrition (EPIC). Subjects Women (n 334 850) from the EPIC study. Results The first TT component (TC1) highlighted a pattern rich in nutrients found in animal foods loading on cholesterol, protein, retinol, vitamins B-12 and D, while the second TT component (TC2) reflected a diet rich in -carotene, riboflavin, thiamin, vitamins C and B-6, fibre, Fe, Ca, K, Mg, P and folate. While TC1 was not

associated with BC risk, TC2 was inversely associated with BC risk overall (HRQ5 v. Q1=089, 95 % CI 083, 095, P-trend<001) and showed a significantly lower risk in oestrogen receptor-positive (HRQ5 v. Q1=089, 95 % CI 081, 098, P-trend=002) and progesterone receptor-positive tumours (HRQ5 v. Q1=087, 95 % CI 077, 098, P-trend<001). Conclusions TT produces readily interpretable sparse components explaining similar amounts of variation as principal component analysis. Our results suggest that participants with a nutrient pattern high in micronutrients found in vegetables, fruits and cereals had a lower risk of BC. [Assi, Nada; Moskal, Aurelie; Slimani, Nadia; Chajes, Veronique; Freisling, Heinz; Romieu, Isabelle; Ferrari, Pietro] Int Agcy Res Canc, 150 Cours Albert Thomas, F-69372 Lyon 08, France; [Assi, Nada] Univ Lyon 1, F-69622 Villeurbanne, France; [Viallon, Vivian] Univ Lyon, Lyon, France; [Viallon, Vivian] Univ Lyon 1, UMRESTTE, F-69365 Lyon, France; [Viallon, Vivian] IFSTTAR, UMRESTTE, Bron, France; [Monni, Stefano; Fortner, Renee Turzanski; Kaaks, Rudolf] German Canc Res Ctr, Div Canc Epidemiol, Heidelberg, Germany; [Knueppel, Sven; Foerster, Jana] German Inst Human Nutr Potsdam Rehbrucke, Dept Epidemiol, Nuthetal, Germany; [Weiderpass, Elisabete; Skeie, Guri; Braaten, Tonje] Univ Tromso, Arctic Univ Norway, Fac Hlth Sci, Dept Community Med, Tromso, Norway; [Weiderpass, Elisabete] Karolinska Inst, Dept Med Epidemiol & Biostat, Stockholm, Sweden; [Weiderpass, Elisabete] Canc Registry Norway, Oslo, Norway; [Weiderpass, Elisabete] Folkhalsan Res Ctr, Dept Genet Epidemiol, Helsinki, Finland; [Lujan-Barroso, Leila] Bellvitge Biomed Res Inst IDIBELL, Catalan Inst Oncol, Canc Epidemiol Res Program, Unit Nutr Environm & Canc, Barcelona, Spain; [Amiano, Pilar; Ardanaz, Eva; Molina-Montes, Esther; Salmeron, Diego] CIBER Epidemiol & Salud Publ CIBERESP, Madrid, Spain; [Amiano, Pilar] BioDonostia Res Inst, Dept Hlth, Publ Hlth Div Gipuzkoa, San Sebastian, Spain; [Ardanaz, Eva] Navarre Publ Hlth Inst, Pamplona, Spain; [Molina-Montes, Esther] Inst Invest Biosanitaria Granada Granada Ibs, Escuela Andaluza Salud Publ, Granada, Spain; [Salmeron, Diego] Murcia Reg Hlth Council, Dept Epidemiol, Murcia, Spain; [Salmeron, Diego] Univ Murcia, Dept Hlth & Social Sci, Murcia, Spain; [Ramon Quiros, Jose] Publ Hlth Directorate, Oviedo, Spain; [Olsen, Anja; Tjonneland, Anne] Danish Canc Soc, Res Ctr, Copenhagen, Denmark; [Dahm, Christina C.; Overvad, Kim] Aarhus Univ, Dept Publ Hlth, Epidemiol Sect, Aarhus, Denmark; [Dossus, Laure; Fournier, Agnes] INSERM, Ctr Res Epidemiol & Populat Hlth CESP, Nutr Hormones & Womens Hlth Team, Villejuif, France; [Dossus, Laure; Fournier, Agnes] Univ Paris 11, UMRS, Villejuif, France; [Dossus, Laure; Fournier, Agnes] IGR, Villejuif, France; [Baglietto, Laura] Canc Council Victoria, Canc Epidemiol Ctr, Melbourne, Vic, Australia; [Baglietto, Laura] Univ Melbourne, Sch Populat & Global Hlth, Ctr Biostat & Epidemiol, Melbourne, Vic, Australia; [Trichopoulou, Antonia] Hellen Hlth Fdn, Athens, Greece; [Trichopoulou, Antonia] Acad Athens, Bur Epidemiol Res, Athens, Greece; [Bamia, Christina; Orfanos, Philippos] Univ Athens, Sch Med, Dept Hyg Epidemiol & Med Stat, GR-11527 Athens, Greece; [De Magistris, Maria Santucci] Azienda Osped Univ, Federico 2, Naples, Italy; [Masala, Giovanna] Canc Res & Prevent Inst ISPO, Mol & Nutr Epidemiol Unit, Florence, Italy; [Agnoli, Claudia] Ist Nazl Tumori, Fdn IRCCS, Epidemiol & Prevent Unit, Via Venezian 1, I-20133 Milan, Italy; [Ricceri, Fulvio] Univ Turin, Dept Med Sci, Unit Canc Epidemiol CERMS, Turin, Italy; [Ricceri, Fulvio] Citta Salute & Sci Hosp, Turin, Italy; [Tumino, Rosario] Civile MP Arezzo Hosp, Canc Registry,

Ragusa, Italy; [Tumino, Rosario] Civile MP Arezzo Hosp, Histopathol Unit, Ragusa, Italy; [de Mesquita, H. Bas Bueno] Natl Inst Publ Hlth & Environm RIVM, Dept Determinants Chron Dis DCD, Bilthoven, Netherlands; [de Mesquita, H. Bas Bueno] Univ Med Ctr, Dept Gastroenterol & Hepatol, Utrecht, Netherlands; [de Mesquita, H. Bas Bueno; Merritt, Melissa A.; Riboli, Elio] Univ London Imperial Coll Sci Technol & Med, Sch Publ Hlth, Dept Epidemiol & Biostat, London, England; [Bakker, Marije F.; Peeters, Petra H. M.] Univ Med Ctr Utrecht, Julius Ctr Hlth Sci & Primary Care, Dept Epidemiol, Utrecht, Netherlands; [Winkvist, Anna] Sahlgrens Acad, Dept Internal Med & Clin Nutr, Gothenburg, Sweden; [Johansson, Ingegerd] Umea Univ, Dept Odontol, Umea, Sweden; [Khaw, Kay-Tee] Univ Cambridge, Sch Clin Med, Dept Publ Hlth & Primary Care, Cambridge, England; [Wareham, Nicholas J.] Univ Cambridge, Sch Clin Med, MRC Epidemiol Unit, Cambridge, England; [Key, Tim; Travis, Ruth; Schmidt, Julie A.] Univ Oxford, Nuffield Dept Populat Hlth, Canc Epidemiol Unit, Oxford, England World Health Organization; International Agency for Research on Cancer (IARC); Universite Claude Bernard Lyon 1; Universite Claude Bernard Lyon 1; Universite Gustave-Eiffel; Universite Gustave-Eiffel; Helmholtz Association; German Cancer Research Center (DKFZ); Leibniz Association; Deutsches Institut fur Ernahrungsforschung Potsdam-Rehbrucke (DIfE); UiT The Arctic University of Tromso; Karolinska Institutet; University of Oslo; Folkhalsan Research Center; Institut d'Investigacio Biomedica de Bellvitge (IDIBELL); Institut Catala d'Oncologia; CIBER - Centro de Investigacion Biomedica en Red; CIBERESP; Public Health Institute of Navarra; Escuela Andaluza de Salud Publica; Murcia Regional Health Council; University of Murcia; Danish Cancer Society; Aarhus University; Institut National de la Sante et de la Recherche Medicale (Inserm); Universite Paris Saclay; Universite Paris Saclay; Institut National de la Sante et de la Recherche Medicale (Inserm); UNICANCER; Gustave Roussy; Cancer Council Victoria; University of Melbourne; Academy of Athens; National & Kapodistrian University of Athens; Athens Medical School; ISPRO Istituto per lo studio, la prevenzione e la rete oncologica; Fondazione IRCCS Istituto Nazionale Tumori Milan; University of Turin; A.O.U. Citta della Salute e della Scienza di Torino; Civile M.P. Arezzo Hospital; Civile M.P. Arezzo Hospital; Netherlands National Institute for Public Health & the Environment; Utrecht University; Utrecht University Medical Center; Imperial College London; Utrecht University; Utrecht University Medical Center; University of Gothenburg; Umea University; University of Cambridge; University of Cambridge; University of OxfordFerrari, P (corresponding author), Int Agcy Res Canc, 150 Cours Albert Thomas, F-69372 Lyon 08, France. ferrarip@iarc.fr Lujan-Barroso, Leila/AAD-3166-2021; Masala, Giovanna/AAC-5474-2022; TRICHOPOULOU, ANTONIA/ABF-8727-2021; Salmerón Martínez, Diego/AAA-3403-2019; Dossus, Laure/AAW-9097-2021; Orfanos, Philippos/AAL-2606-2021; Merritt, Melissa/LQJ-8182-2024; Khaw, Kay-Tee/AAZ-3209-2021; Riboli, Elio/A-4357-2009; Turzanski Fortner, Renée/KMX-9538-2024; Winkvist, Anna/AAC-6178-2021; Baglietto, Laura/AAB-9051-2019; Timpson, Nicholas/O-7548-2015; Foerster, Jana/F-4847-2013; Tjonneland, Anne/AGU-0320-2022; Knüppel, Sven/AAI-7195-2020; Dahm, Christina/G-9787-2014; RICCERI, FULVIO/I-9910-2018; Agnoli, Claudia/K-5916-2016; Molina-Montes, Esther/AFS-7568-2022; Weiderpass, Elisabete/M-4029-2016; Fournier, Agnes/H-4615-2018

Merritt, Melissa A./0000-0002-5067-6119; Fortner,

Renee/0000-0002-1426-8505; Dahm, Christina/0000-0003-0481-2893; Winkvist, Anna/0000-0001-9122-7240; Olsen, Anja/0000-0003-4788-503X; Orfanos, Philippos/0000-0002-9949-3137; Lujan-Barroso, Leila/0000-0001-6224-1764; RICCERI, FULVIO/0000-0001-8749-9737; Agnoli, Claudia/0000-0003-4472-1179; Baglietto, Laura/0000-0002-8193-7529; Tjonneland, Anne/0000-0003-4385-2097; Freisling, Heinz/0000-0001-8648-4998; Molina-Montes, Esther/0000-0002-0428-2426; Weiderpass, Elisabete/0000-0003-2237-0128; Chajes, Veronique/0000-0003-1297-3064; Overvad, Kim/0000-0001-6429-7921; Fournier, Agnes/0000-0001-8380-3439; Dossus, Laure/0000-0003-2716-5748; Riboli, Elio/0000-0001-6795-6080; moskal, aurelie/0000-0003-4127-4334; Salmeron Martinez, Diego/0000-0002-4574-6880; tumino, rosario/0000-0003-2666-414X; Skeie, Guri/0000-0003-2476-4251; Ardanaz, Eva/0000-0001-8434-2013; Masala, Giovanna/0000-0002-5758-9069 European Commission (Directorate General for Health and Consumer Affairs); International Agency for Research on Cancer (IARC); Health Research Fund (FIS) of the Spanish Ministry of Health RTICC 'Red Tematica de Investigacion Cooperativa en Cancer [Rd06/0020/0091, Rd12/0036/0018]; Regional Government of Andalucia; Regional Government of Asturias; Regional Government of Basque Country; Regional Government of Murcia [6236]; Regional Government of Navarra; Instituto de Salud Carlos III, Redes de Investigacion Cooperativa (Spain) [RD06/0020]; Danish Cancer Society (Denmark); Ligue Contre le Cancer; Institut Gustave Roussy, Mutuelle Generale de l'Education Nationale; Institut National de la Sante et de la Recherche Medicale (France); Deutsche Krebshilfe; Deutsches Krebsforschungszentrum; Federal Ministry of Education and Research (Germany); Hellenic Health Foundation; Stavros Niarchos Foundation; Hellenic Ministry of Health and Social Solidarity (Greece); Italian Association for Research on Cancer (AIRC); National Research Council (Italy); Dutch Ministry of Public Health, Welfare and Sports; Netherlands Cancer Registry; LK Research Funds; Dutch Prevention Funds; Dutch Zorg Onderzoek Nederland; World Cancer Research Fund; Statistics Netherlands (Netherlands); European Research Council [2009-AdG 232997]; Nordforsk; Nordic Centre of Excellence programme on Food, Nutrition and Health (Norway); Swedish Cancer Society; Swedish Research Council; Regional Government of Skane; Regional Government of Vasterbotten (Sweden); Cancer Research UK; Medical Research Council; Stroke Association; British Heart Foundation; Department of Health; Food Standards Agency; Wellcome Trust (UK); Universite de Lyon doctoral grant (EDISS doctoral school); MRC [MC_UU_12015/1] Funding Source: UKRI European Commission (Directorate General for Health and Consumer Affairs)(European Union (EU)European Commission Joint Research Centre); International Agency for Research on Cancer (IARC)(World Health Organization); Health Research Fund (FIS) of the Spanish Ministry of Health RTICC 'Red Tematica de Investigacion Cooperativa en Cancer; Regional Government of Andalucia(Junta de Andalucia); Regional Government of Asturias(Principality of Asturias); Regional Government of Basque Country(Basque Government); Regional Government of Murcia; Regional Government of Navarra; Instituto de Salud Carlos III, Redes de Investigacion Cooperativa (Spain)(Instituto de Salud Carlos III); Danish Cancer Society (Denmark)(Danish Cancer Society); Ligue Contre le Cancer(Ligue nationale contre le cancer); Institut Gustave Roussy, Mutuelle Generale de l'Education Nationale; Institut National de la Sante et de la Recherche Medicale (France)(Institut National de la Sante

et de la Recherche Medicale (Inserm)); Deutsche Krebshilfe(Deutsche Krebshilfe); Deutsches Krebsforschungszentrum; Federal Ministry of Education and Research (Germany)(Federal Ministry of Education & Research (BMBF)); Hellenic Health Foundation; Stavros Niarchos Foundation; Hellenic Ministry of Health and Social Solidarity (Greece); Italian Association for Research on Cancer (AIRC)(Fondazione AIRC per la ricerca sul cancro); National Research Council (Italy)(Consiglio Nazionale delle Ricerche (CNR)); Dutch Ministry of Public Health, Welfare and Sports; Netherlands Cancer Registry; LK Research Funds; Dutch Prevention Funds; Dutch Zorg Onderzoek Nederland(Netherlands Organization for Scientific Research (NWO)); World Cancer Research Fund(World Cancer Research Fund International (WCRF)); Statistics Netherlands (Netherlands)(Netherlands Government); European Research Council(European Research Council (ERC)); Nordforsk(NordForsk); Nordic Centre of Excellence programme on Food, Nutrition and Health (Norway); Swedish Cancer Society(Swedish Cancer Society); Swedish Research Council(Swedish Research Council); Regional Government of Skane; Regional Government of Vasterbotten (Sweden); Cancer Research UK(Cancer Research UK); Medical Research Council(UK Research & Innovation (UKRI)Medical Research Council UK (MRC)); Stroke Association; British Heart Foundation(British Heart Foundation); Department of Health; Food Standards Agency; Wellcome Trust (UK)(Wellcome Trust); Universite de Lyon doctoral grant (EDISS doctoral school); MRC(UK Research & Innovation (UKRI)Medical Research Council UK (MRC)) Financial support: The coordination of the EPIC study is financially supported by the European Commission (Directorate General for Health and Consumer Affairs) and the International Agency for Research on Cancer (IARC). The national cohorts are supported by: the Health Research Fund (FIS) of the Spanish Ministry of Health RTICC 'Red Tematica de Investigacion Cooperativa en Cancer (grant numbers Rd06/0020/0091 and Rd12/0036/0018), the Regional Governments of Andalucia, Asturias, Basque Country, Murcia (project 6236) and Navarra, and the Instituto de Salud Carlos III, Redes de Investigacion Cooperativa (RD06/0020) (Spain); the Danish Cancer Society (Denmark); the Ligue Contre le Cancer, the Institut Gustave Roussy, Mutuelle Generale de l'Education Nationale and the Institut National de la Sante et de la Recherche Medicale (France); the Deutsche Krebshilfe, the Deutsches Krebsforschungszentrum and the Federal Ministry of Education and Research (Germany); the Hellenic Health Foundation, the Stavros Niarchos Foundation and the Hellenic Ministry of Health and Social Solidarity (Greece); the Italian Association for Research on Cancer (AIRC) and the National Research Council (Italy); the Dutch Ministry of Public Health, Welfare and Sports, the Netherlands Cancer Registry, LK Research Funds, Dutch Prevention Funds, Dutch Zorg Onderzoek Nederland, the World Cancer Research Fund and Statistics Netherlands (Netherlands); the European Research Council (2009-AdG 232997) and the Nordforsk, Nordic Centre of Excellence programme on Food, Nutrition and Health (Norway); the Swedish Cancer Society, the Swedish Research Council and the Regional Governments of Skane and Vasterbotten (Sweden); Cancer Research UK, the Medical Research Council, the Stroke Association, the British Heart Foundation, the Department of Health, the Food Standards Agency and the Wellcome Trust (UK). The work undertaken by N.A. was supported by a Universite de Lyon doctoral grant (EDISS doctoral school).

81 31 34 0 28 CAMBRIDGE UNIV PRESS CAMBRIDGE EDINBURGH BLDG, SHAFTESBURY RD, CB2 8RU CAMBRIDGE, ENGLAND 1368-9800 1475-2727 PUBLIC HEALTH NUTR Public Health Nutr. FEB 2016 19 2 242 254 10.1017/S1368980015000294 http://dx.doi.org/10.1017/S1368980015000294 13 Public, Environmental & Occupational Health; Nutrition & Dietetics Science Citation Index Expanded (SCI-EXPANDED) Public, Environmental & Occupational Health; Nutrition & Dietetics DH2TR 25702596 Green Submitted, Bronze, Green Published 2025-06-24 WOS:000372639900006
J Ozsoy, M; Keles, C; Kahya, M; Keles, G
Ozsoy, Mustafa; Keles, Celalettin; Kahya, Mumtaz; Keles, Gonul Primary echinococcal cyst in the axillary region JOURNAL OF INFECTION IN DEVELOPING COUNTRIES English Article Hydatid Cyst; Breast Cancer; Axilla NEEDLE-ASPIRATION BIOPSY; PRIMARY HYDATID CYST; THYROID-GLAND; DISEASE Introduction: Human hydatid disease is a parasitic infection caused by the larval form of Echinococcus granulosus. It has worldwide distribution and is endemic in many countries, especially the Mediterranean region. It most commonly affects the liver and lungs although multi-organ involvement has been observed in 20-30% of patients. Case report: A 45-year-old woman presented to a gynaecologist because of a mass in the axillary region. Her mother and her two sisters were undergoing treatment for breast cancer. In her examination, a hard, semi-mobile, painless mass was found that was approximately 3 cm in diameter. Axillary ultrasonography showed lymphadenopathy. No abnormality was found in mammographic examination of either breast, or in abdominal ultrasonography and chest X-ray. Occult breast cancer was suspected but when the mass was excised for pathological examination the biopsy showed a hydatid cyst with germinative membranes. Subsequent lung, abdomen and brain tomography scans, whole body bone scintigraphy and hydatid serology, including indirect haemagglutination and enzyme-linked immunosorbent assay, were negative. For these reasons an isolated axillary hydatid cyst was diagnosed. Conclusion: Parasitic cysts should be considered in endemic areas in patients presenting with a soft tissue mass in the axillary region. Imaging methods should be planned to include this possibility. [Ozsoy, Mustafa; Keles, Celalettin; Kahya, Mumtaz] Manisa State Hosp, Dept Gen Surg, Manisa, Turkey; [Keles, Gonul] Celal Bayar Univ, Fac Med, Dept Anaesthesiol, Manisa, TurkeyManisa Merkez Efendi State Hospital; Manisa State Hospital; Celal Bayar University Ozsoy, M (corresponding author), 1819-11 Sok 16-10 Istasyonalti Mahallesi Cigli, TR-35630 Izmir, Turkey. dr.mustafaozsoy@gmail.com 20 10 13 0 3 J INFECTION DEVELOPING COUNTRIES TRAMANIGLIO JIDC CENT OFF PORTO CONTE RICERCHE RES CTR, S P 55, PORTO CONTE CAPO CACCIA KM 8.400 LOC, TRAMANIGLIO, 07041, ITALY 1972-2680 J INFECT DEV COUNTR J. Infect. Dev. Ctries. NOV 2011 5 11 825 827 10.3855/jidc.1589 http://dx.doi.org/10.3855/jidc.1589 3 Infectious Diseases Science Citation Index Expanded (SCI-EXPANDED) Infectious Diseases 905JW 22112739 gold, Green Submitted 2025-06-24 WOS:000301269500013
J Roshanizadeh, Z; Haghshenas, MR; Ramezani, A; Shajari, N; Tahmasebi, S; Akrami, M; Ghaderi, A

Roshanizadeh, Zahra; Haghshenas, Mohammad Reza; Ramezani, Amin; Shajari, Neda; Tahmasebi, Sedigheh; Akrami, Majid; Ghaderi, Abbas Importance of Nectin2, NUF2, and Nectin4 Gene Expression in the Pathogenesis of Different Subtypes of Breast Cancer MIDDLE EAST JOURNAL OF CANCER English Article Triple negative breast neoplasms; Biomarker; Estrogen receptor; Progesterone receptor Background: The targeted therapy using breast cancer (BC)-associated biomarkers has significantly minimized the side-effects of BC treatment. This study aims to elucidate the role of Nectin2, NUF2, and Nectin4 gene expression in the pathogenesis of BC. Method: In this case-control study, the expression of Nectin2, Nectin4, and NUF2 genes was investigated through real-time polymerase chain reaction assay in 46 tumor tissues from BC patients and 46 adjacent non-tumorous tissues as a control group. Data were analyzed using SPSS-21 software, employing independent t-tests and one-way ANOVA. A P-value of <0.05 was considered statistically significant. Results: The results demonstrated a significant increase in the expression of the NUF2 gene in tumor tissues compared with adjacent normal tissues (P = 0.005, fold change = 3.7). No statistically significant difference was observed in the expression of Nectin2 and Nectin4 between the tumor and adjacent tissues. However, higher expression of Nectin2 was noted in the early stages of the disease, particularly in subtypes with estrogen receptor-positive (ER+), progesterone receptor positive (PR+), and human epidermal growth factor receptor 2 negative (HER2-). Furthermore, the expression of NUF2 and Nectin4 was elevated in advanced stages and triple-negative BC subtypes. Notably, the expression of these three genes was higher in patients aged <= 45 years. Conclusion: The findings suggest that the expression levels of NUF2, Nectin2, and Nectin4 genes may influence the initiation, progression, and pathogenesis of BC subtypes.[Roshanizadeh, Zahra; Shajari, Neda; Ghaderi, Abbas] Shiraz Univ Med Sci, Sch Med, Dept Immunol, Shiraz, Iran; [Roshanizadeh, Zahra; Haghshenas, Mohammad Reza; Ramezani, Amin; Shajari, Neda; Ghaderi, Abbas] Shiraz Univ Med Sci, Shiraz Inst Canc Res, Sch Med, Shiraz, Iran; [Ramezani, Amin; Ghaderi, Abbas] Shiraz Univ Med Sci, Sch Adv Med Sci & Technol, Dept Med Biotechnol, Shiraz, Iran; [Tahmasebi, Sedigheh] Shiraz Univ Med Sci, Lymphedema Res Ctr, Dept Gen Surg, Shiraz, Iran; [Akrami, Majid] Shiraz Univ Med Sci, Breast Dis Res Canc, Shiraz, Iran Shiraz University of Medical Science; Shiraz University of Medical Science; Shiraz University of Medical Science; Shiraz University of Medical Science; Shiraz University of Medical Science Ghaderi, A (corresponding author), Shiraz Univ Med Sci, Shiraz Inst Canc Res, Sch Med, Shiraz, Iran.
Ghaderia@sums.ac.ir Ramezani, Amin/S-1035-2017; Haghshenas, Mohammad/N-1314-2018; tahmasebi, sedigheh/D-5433-2018; Ghaderi, Abbas/B-3874-2012; Akrami, Majid/AAG-2773-2021
Shiraz University of Medical Sciences, Shiraz, Iran [23215]; Shiraz Institute for Cancer Research [ICR-100-503] Shiraz University of Medical Sciences, Shiraz, Iran(Shiraz University of Medical ScienceGolestan University of Medical Sciences); Shiraz Institute for Cancer Research The project received financial support from Shiraz University of Medical Sciences, Shiraz, Iran (Grant No. 23215) and the Shiraz Institute for Cancer Research (ICR-100-503) . 19 0 0 0 0 SHIRAZ UNIV MEDICAL SCIENCES SHIRAZ NEMAZEE HOSPITAL, SHIRAZ, 71934, IRAN

2008-6709 2008-6687 MIDDLE EAST J CANCER Middle East J. Cancer OCT 2024 15 4 281 288 10.30476/mejc.2024.100701.1997 http://dx.doi.org/10.30476/mejc.2024.100701.1997 8 Oncology Emerging Sources Citation Index (ESCI) Oncology J2E7Q 2025-06-24 WOS:001335258100003
J Peiris, D; Ghosh, A; Manne-Goehler, J; Jaacks, LM; Theilmann, M; Marcus, ME; Zhumadilov, Z; Tsabedze, L; Supiyev, A; Silver, BK; Sibai, AM; Norov, B; Mayige, MT; Martins, JS; Lunet, N; Labadarios, D; Jorgensen, JMA; Houehanou, C; Guwatudde, D; Gurung, MS; Damasceno, A; Aryal, KK; Andall-Brereton, G; Agoudavi, K; McKenzie, B; Webster, J; Atun, R; Bärnighausen, T; Vollmer, S; Davies, JI; Geldsetzer, P Peiris, David; Ghosh, Arpita; Manne-Goehler, Jennifer; Jaacks, Lindsay M.; Theilmann, Michaela; Marcus, Maja E.; Zhumadilov, Zhaxybay; Tsabedze, Lindiwe; Supiyev, Adil; Silver, Bahendeka K.; Sibai, Abla M.; Norov, Bolormaa; Mayige, Mary T.; Martins, Joao S.; Lunet, Nuno; Labadarios, Demetre; Jorgensen, Jutta M. A.; Houehanou, Corine; Guwatudde, David; Gurung, Mongal S.; Damasceno, Albertino; Aryal, Krishna K.; Andall-Brereton, Glennis; Agoudavi, Kokou; McKenzie, Briar; Webster, Jacqui; Atun, Rifat; Baernighausen, Till; Vollmer, Sebastian; Davies, Justine I.; Geldsetzer, Pascal
Cardiovascular disease risk profile and management practices in 45 low-income and middle-income countries: A cross-sectional study of nationally representative individual-level survey data
PLOS MEDICINE English Article BLOOD-PRESSURE; STRATEGIES
Author summary Why was this study done? CVD burden in low-income and middle-income countries (LMICs) is high and rising. CVD risk estimation using validated risk prediction equations is recommended in most guidelines; however, there are few population-representative analyses of CVD risk and its association with socio-demographic characteristics. Despite guidelines recommending using CVD risk estimates as an essential first step in guiding management practices, the extent to which risk-based approaches are being implemented in LMICs is not well characterised. What did the researchers do and find? We analysed population-representative survey data from 45 LMICs to determine country-specific levels of CVD risk, associations between socio-demographic factors and levels of CVD risk, and adherence to WHO guidelines on use of blood pressure medication. We found high variation in CVD risk profiles, with higher levels of risk in the Europe and the Eastern Mediterranean region and lower levels of risk in sub-Saharan Africa, as well as an inverse association between CVD risk and higher education and employment in most countries. We found an underuse of medicines in people at elevated CVD risk across all countries (only 24.2% of males and 41.6% of females at high CVD risk are taking guideline-recommended BP medication) and an overuse of medicines in people at lower levels of CVD risk, with 47% of all BP medication being used by people at low CVD risk without a guideline indication for use. What do these findings mean? There is large variation in CVD risk across LMICs, and an inverse association between CVD risk and higher education and employment in most countries. There is an overuse of medicines in people at lower levels of CVD risk and an underuse of medicines in people at elevated CVD risk across all countries. The large heterogeneity of the findings in this study reflects varying

country contexts. Country-specific targeted policies are needed to improve the identification and management of those at highest CVD risk. Background Global cardiovascular disease (CVD) burden is high and rising, especially in low-income and middle-income countries (LMICs). Focussing on 45 LMICs, we aimed to determine (1) the adult population's median 10-year predicted CVD risk, including its variation within countries by socio-demographic characteristics, and (2) the prevalence of self-reported blood pressure (BP) medication use among those with and without an indication for such medication as per World Health Organization (WHO) guidelines. Methods and findings We conducted a cross-sectional analysis of nationally representative household surveys from 45 LMICs carried out between 2005 and 2017, with 32 surveys being WHO Stepwise Approach to Surveillance (STEPS) surveys. Country-specific median 10-year CVD risk was calculated using the 2019 WHO CVD Risk Chart Working Group non-laboratory-based equations. BP medication indications were based on the WHO Package of Essential Noncommunicable Disease Interventions guidelines. Regression models examined associations between CVD risk, BP medication use, and socio-demographic characteristics. Our complete case analysis included 600,484 adults from 45 countries. Median 10-year CVD risk (interquartile range [IQR]) for males and females was 2.7% (2.3%-4.2%) and 1.6% (1.3%-2.1%), respectively, with estimates indicating the lowest risk in sub-Saharan Africa and highest in Europe and the Eastern Mediterranean. Higher educational attainment and current employment were associated with lower CVD risk in most countries. Of those indicated for BP medication, the median (IQR) percentage taking medication was 24.2% (15.4%-37.2%) for males and 41.6% (23.9%-53.8%) for females. Conversely, a median (IQR) 47.1% (36.1%-58.6%) of all people taking a BP medication were not indicated for such based on CVD risk status. There was no association between BP medication use and socio-demographic characteristics in most of the 45 study countries. Study limitations include variation in country survey methods, most notably the sample age range and year of data collection, insufficient data to use the laboratory-based CVD risk equations, and an inability to determine past history of a CVD diagnosis. Conclusions This study found underuse of guideline-indicated BP medication in people with elevated CVD risk and overuse by people with lower CVD risk. Country-specific targeted policies are needed to help improve the identification and management of those at highest CVD risk.

[Peiris, David; Ghosh, Arpita; McKenzie, Briar; Webster, Jacqui] UNSW Sydney, George Inst Global Hlth, Sydney, NSW, Australia; [Ghosh, Arpita] Manipal Acad Higher Educ, Manipal, India; [Manne-Goehler, Jennifer; Theilmann, Michaela; Baernighausen, Till; Geldsetzer, Pascal] Heidelberg Univ, Med Fac, Heidelberg Inst Global Hlth, Heidelberg, Germany; [Manne-Goehler, Jennifer; Theilmann, Michaela; Baernighausen, Till; Geldsetzer, Pascal] Heidelberg Univ, Univ Hosp, Heidelberg, Germany; [Jaacks, Lindsay M.; Atun, Rifat] Harvard TH Chan Sch Publ Hlth, Dept Global Hlth & Populat, Boston, MA USA; [Marcus, Maja E.; Vollmer, Sebastian] Univ Goettingen, Dept Econ, Gottingen, Germany; [Marcus, Maja E.; Vollmer, Sebastian] Univ Goettingen, Ctr Modern Indian Studies, Gottingen, Germany; [Zhumadilov, Zhaxybay] Nazarbayev Univ, Sch Med, Nur Sultan, Kazakhstan; [Tsabedze, Lindiwe] Eswatini Minist Hlth, Mbabane, Eswatini; [Supiyev, Adil] Nazarbayev Univ, Natl Lab Astana, Ctr Life Sci, Lab Epidemiol & Publ Hlth, Astana,

Kazakhstan; [Silver, Bahendeka K.] St Francis Hosp Nsambya, Kampala, Uganda; [Sibai, Abla M.] Amer Univ Beirut, Fac Hlth Sci, Dept Epidemiol & Populat Hlth, Beirut, Lebanon; [Norov, Bolormaa] Natl Ctr Publ Hlth, Ulan Bator, Mongolia; [Mayige, Mary T.] Natl Inst Med Res, Dar Es Salaam, Tanzania; [Martins, Joao S.] Univ Nacl Timor Lorosae, Fac Med & Hlth Sci, Dili, Timor-Leste; [Lunet, Nuno] Univ Porto, Dept Ciencias Saude Publ & Forenses & Educ Med, Fac Med, Porto, Portugal; [Labadarios, Demetre] Stellenbosch Univ, Fac Med & Hlth Sci, Stellenbosch, South Africa; [Jorgensen, Jutta M. A.] Univ Copenhagen, Dept Publ Hlth, Copenhagen, Denmark; [Houehanou, Corine] Univ Abomey Calavi, Fac Hlth Sci, Lab Epidemiol Chron & Neurol Dis, Cotonou, Benin; [Guwatudde, David] Makerere Univ, Sch Publ Hlth, Dept Epidemiol & Biostat, Kampala, Uganda; [Gurung, Mongal S.] Minist Hlth, Hlth Res & Epidemiol Unit, Thimphu, Bhutan; [Damasceno, Albertino] Eduardo Mondlane Univ, Fac Med, Maputo, Mozambique; [Aryal, Krishna K.] Abt Associates Inc, Monitoring Evaluat & Operat Res Project, Kathmandu, Nepal; [Andall-Brereton, Glennis] Caribbean Publ Hlth Agcy, Noncommun Dis, Port Of Spain, Trinidad Tobago; [Agoudavi, Kokou] Togo Minist Hlth, Lome, Togo; [Davies, Justine I.] Univ Birmingham, Inst Appl Hlth Res, Birmingham, England; [Davies, Justine I.] Stellenbosch Univ, Ctr Global Surg, Dept Global Hlth, Cape Town, South Africa; [Davies, Justine I.] Univ Witwatersrand, Wits Univ, Rural Publ Hlth & Hlth Transit Res Unit, Fac Hlth Sci,Sch Publ Hlth,Med Res Council, Johannesburg, South Africa; [Geldsetzer, Pascal] Stanford Univ, Dept Med, Div Primary Care & Populat Hlth, Stanford, CA 94305 USA   University of New South Wales Sydney; George Institute for Global Health; Manipal Academy of Higher Education (MAHE); Ruprecht Karls University Heidelberg; Ruprecht Karls University Heidelberg; Harvard University; Harvard T.H. Chan School of Public Health; University of Gottingen; University of Gottingen; Nazarbayev University; Nazarbayev University; American University of Beirut; Ministry of Health Mongolia; National Institute of Medical Research; Universidade do Porto; Stellenbosch University; University of Copenhagen; University of Abomey Calavi; Makerere University; Eduardo Mondlane University; University of Birmingham; Stellenbosch University; South African Medical Research Council; University of Witwatersrand; Stanford University   Peiris, D (corresponding author), UNSW Sydney, George Inst Global Hlth, Sydney, NSW, Australia. dpeiris@georgeinstitute.org Aryal, Krishna/AFN-8677-2022; Supiyev, Adil/D-6455-2012; McKenzie, Briar/AAA-8495-2022; Zhumadilov, Kassym/O-7356-2014; Crump, John/AAZ-6412-2020; Barnighausen, Till/Y-2388-2019; Atun, Rifat/AAH-5537-2021; Marcus, Maja/ABZ-0474-2022; Gurung, Mongal Singh/J-9785-2016; Vollmer, Sebastian/U-6496-2017; Mayige, Mary/L-5342-2016   Gurung, Mongal Singh/0000-0001-6481-6242; Zhumadilov, Zhaxybay/0009-0001-0433-8290; Webster, Jacqui/0000-0003-3513-3340; Vollmer, Sebastian/0000-0002-7863-0462; Mayige, Mary/0000-0003-4861-7870; Silver, Bahendeka/0000-0001-8080-7872; Theilmann, Michaela/0000-0003-3413-232X; Agoudavi, Kokou/0000-0002-3139-9777; Ghosh, Arpita/0000-0003-0036-4376; Sibai, Abla/0000-0002-1851-5606; Marcus, Maja E./0000-0003-4904-2087; Jaacks, Lindsay/0000-0002-7791-5167; McKenzie, Briar/0000-0001-6972-6617; Lunet, Nuno/0000-0003-1870-1430; Peiris, David/0000-0002-6898-3870 Harvard McLennan Family Fund; National Health and Medical Research Council of Australia [1143904]; Heart Foundation of Australia [101890]; National Center for Advancing Translational Sciences of the

National Institutes of Health [KL2TR003143]; National Health and Medical Research Council of Australia [1143904] Funding Source: NHMRC Harvard McLennan Family Fund; National Health and Medical Research Council of Australia(National Health & Medical Research Council (NHMRC) of Australia); Heart Foundation of Australia(National Heart Foundation of Australia); National Center for Advancing Translational Sciences of the National Institutes of Health(United States Department of Health & Human ServicesNational Institutes of Health (NIH) - USANIH National Center for Advancing Translational Sciences (NCATS)); National Health and Medical Research Council of Australia(National Health & Medical Research Council (NHMRC) of Australia) This study was supported with funding from the Harvard McLennan Family Fund. DP is supported by fellowships from the National Health and Medical Research Council of Australia (1143904) and the Heart Foundation of Australia (101890). PG was supported by the National Center for Advancing Translational Sciences of the National Institutes of Health under Award Number KL2TR003143. The funding sources for this study had no involvement in the design, collection, analysis and interpretation of the data. The academic investigators participated in the design and oversight of the project. They had full access to all the data and had final responsibility for the decision to submit for publication. All authors gave approval to submit for publication. 30 35 35 1 12
PUBLIC LIBRARY SCIENCE SAN FRANCISCO 1160 BATTERY STREET, STE 100, SAN FRANCISCO, CA 94111 USA 1549-1277 1549-1676
PLOS MED PLos Med. MAR 2021 18 3
e1003485 10.1371/journal.pmed.1003485
http://dx.doi.org/10.1371/journal.pmed.1003485
19 Medicine, General & Internal Science Citation Index Expanded (SCI-EXPANDED) General & Internal Medicine QT5BH
33661979 Green Published, gold 2025-06-24
WOS:000626602400005

J Sanchez-Martin, V; Schneider, DA; Ortiz-Gonzalez, M; Soriano-Lerma, A; Linde-Rodriguez, A; Perez-Carrasco, V; Gutierrez-Fernandez, J; Cuadros, M; González, C; Soriano, M; Garcia-Salcedo, JA Sanchez-Martin, Victoria; Schneider, David A.; Ortiz-Gonzalez, Matilde; Soriano-Lerma, Ana; Linde-Rodriguez, Angel; Perez-Carrasco, Virginia; Gutierrez-Fernandez, Jose; Cuadros, Marta; Gonzalez, Carlos; Soriano, Miguel; Garcia-Salcedo, Jose A. Targeting ribosomal G-quadruplexes with naphthalene-diimides as RNA polymerase I inhibitors for colorectal cancer treatment CELL CHEMICAL BIOLOGY
English Article
TRANSCRIPTION; BINDING; VISUALIZATION; TRANSPORTERS; VITRO Guanine quadruplexes (G4s) are non-canonical nucleic acid structures commonly found in regulatory genomic regions. G4 targeting has emerged as a therapeutic approach in cancer. We have screened naphthalene-diimides (NDIs), a class of G4 ligands, in a cellular model of colorectal cancer (CRC). Here, we identify the leading compound T5 with a potent and selective inhibition of cell growth by high-affinity binding to G4s in ribosomal DNA, impairing RNA polymerase I (Pol I) elongation. Consequently, T5 induces a rapid inhibition of Pol I transcription, nucleolus disruption, proteasome-dependent Pol I catalytic subunit A degradation and autophagy. Moreover, we attribute the higher selectivity of carbohydrate-conjugated T5 for tumoral cells to its preferential uptake through the overexpressed glucose transporter 1. Finally,

we succinctly demonstrate that T5 could be explored as a
therapeutic agent in a patient cohort with CRC. Therefore, we
report a mode of action for these NDIs involving ribosomal G4
targeting. [Sanchez-Martin, Victoria; Ortiz-Gonzalez, Matilde;
Soriano-Lerma, Ana; Linde-Rodriguez, Angel; Perez-Carrasco,
Virginia; Cuadros, Marta; Soriano, Miguel; Garcia-Salcedo, Jose
A.] Univ Granada, Pfizer, Andalusian Reg Govt, GENYO Ctr Genom &
Oncol Res, Granada 18016, Spain; [Sanchez-Martin, Victoria; Linde-
Rodriguez, Angel; Perez-Carrasco, Virginia; Gutierrez-Fernandez,
Jose; Garcia-Salcedo, Jose A.] Univ Hosp Virgen de las Nieves,
Biosanitary Res Inst IBS Granada, Microbiol Unit, Granada 18014,
Spain; [Sanchez-Martin, Victoria; Cuadros, Marta] Univ Granada,
Dept Biochem Mol Biol & Immunol 3, Granada 18016, Spain;
[Schneider, David A.] Univ Alabama Birmingham, Dept Biochem & Mol
Genet, Birmingham, AL 35294 USA; [Ortiz-Gonzalez, Matilde;
Soriano, Miguel] Univ Almeria, Ctr Intens Mediterranean Agrosyst &
Agrifood Biot, Almeria 04001, Spain; [Soriano-Lerma, Ana] Univ
Granada, Dept Physiol, Granada 18011, Spain; [Gutierrez-Fernandez,
Jose] Univ Granada, Dept Microbiol, Granada 18011, Spain;
[Gonzalez, Carlos] CSIC, Inst Quim Fis Rocasolano, Madrid 28006,
Spain University of Granada; Pfizer; Pfizer Spain; Hospital
Universitario Virgen de las Nieves; University of Granada;
University of Alabama System; University of Alabama Birmingham;
Universidad de Almeria; University of Granada; University of
Granada; Consejo Superior de Investigaciones Cientificas (CSIC);
CSIC - Instituto de Quimica Fisica Blas Cabrera (IQF-CSIC)

Garcia-Salcedo, JA (corresponding author), Univ Granada,
Pfizer, Andalusian Reg Govt, GENYO Ctr Genom & Oncol Res, Granada
18016, Spain.; Garcia-Salcedo, JA (corresponding author), Univ
Hosp Virgen de las Nieves, Biosanitary Res Inst IBS Granada,
Microbiol Unit, Granada 18014, Spain. jags@genyo.es Cuadros,
Marta/K-1576-2014; Lerma, Ana/AAA-8272-2019; Garcia-Salcedo,
Jose/AAA-9434-2019; Soriano, Miguel/L-9532-2014; Martín,
Victoria/AAR-5251-2020; Ortiz-Gonzalez, Matilde/AFR-6424-2022;
Gonzalez, Carlos/A-4734-2013; GUTIERREZ-FERNANDEZ, JOSE/H-9985-
2014; Morales, Juan Carlos/B-7853-2008 GUTIERREZ-FERNANDEZ,
JOSE/0000-0001-6146-9740; Morales, Juan Carlos/0000-0003-2400-405X

European Commission; National Institutes of Health
[GM084946]; Government of Spain [FPU16/05822, FPU17/05413];
University of Almeria European Commission(European Union
(EU)European Commission Joint Research Centre); National
Institutes of Health(United States Department of Health & Human
ServicesNational Institutes of Health (NIH) - USA); Government of
Spain(Spanish Government); University of Almeria We thank Juan
Carlos Morales' laboratory (IPLN-CSIC, Granada, Spain) for kindly
supplying GLUT inhibitors and NDI derivatives. We also thank Maria
Jose Serrano's laboratory (Genyo, Granada, Spain) for supplying
HCT116 and HT29 cell lines, and Pedro Real's group (Genyo,
Granada, Spain) for providing us with MG132 reagent. We thank
``Manuel Rico'' NMR laboratory (LMR), a node of the Spanish Large-
Scale National Facility (ICTS R-LRB) for performing NMR
experiments. Finally, we gratefully acknowledge Javier Oliver
(IPLN-CSIC, Granada, Spain) for supplying LC3 antibody and his
valuable assistance. This work was supported by the European
Commission (TARBRAINFECT to J.A.G.-S.) and the National Institutes
of Health (GM084946 to D.A.S.). The Government of Spain granted
with PhD fellowships FPU16/05822 to V.S.-M. and FPU17/05413 to
A.S.-L. The University of Almeria granted with PhD fellowship to

M.O.-G. Funding for open access charge: European Commission.
50 14 14 1 14 CELL PRESS CAMBRIDGE 50 HAMPSHIRE ST, FLOOR 5, CAMBRIDGE, MA 02139 USA 2451-9448
CELL CHEM BIOL Cell Chem. Biol. NOV 18 2021 28
11 1590 +
10.1016/j.chembiol.2021.05.021
http://dx.doi.org/10.1016/j.chembiol.2021.05.021
17 Biochemistry & Molecular Biology Science Citation Index Expanded (SCI-EXPANDED) Biochemistry & Molecular Biology
XD3WB Green Published 2025-06-24
WOS:000722642500006
J Luparello, C; Ragona, D; Asaro, DML; Lazzara, V; Affranchi, F; Celi, M; Arizza, V; Vazzana, M Luparello, Claudio; Ragona, Debora; Asaro, Dalia Maria Lucia; Lazzara, Valentina; Affranchi, Federica; Celi, Monica; Arizza, Vincenzo; Vazzana, Mirella Cytotoxic Potential of the Coelomic Fluid Extracted from the Sea Cucumber Holothuria tubulosa against Triple-Negative MDA-MB231 Breast Cancer Cells BIOLOGY-BASEL
English Article
Holothuria tubulosa; coelomic fluid; breast cancer; cell viability; cell cycle; mitochondrial function; autophagy IN-VITRO; AUTOPHAGY; ANTICANCER; LINES; INHIBITOR; APOPTOSIS; CAPACITY; BECLIN-1; KINASE; DEATH Growing evidence has demonstrated that the extracts of different holothurian species exert beneficial effects on human health. Triple negative breast cancers (TNBC) are highly malignant tumors that present a poor prognosis due to the lack of effective targeted therapies. In the attempt to identify novel compounds that might counteract TNBC cell growth, we studied the effect of the exposure of the TNBC cell line MDA-MB231 to total and filtered aqueous extracts of the coelomic fluid obtained from the sea cucumber Holoturia tubulosa, a widespread species in the Mediterranean Sea. In particular, we examined cell viability and proliferative behaviour, cell cycle distribution, apoptosis, autophagy, and mitochondrial metabolic/cell redox state. The results obtained indicate that both total and fractionated extracts are potent inhibitors of TNBC cell viability and growth, acting through both an impairment of cell cycle progression and mitochondrial transmembrane potential and a stimulation of cellular autophagy, as demonstrated by the increase of the acidic vesicular organelles and of the intracellular protein markers beclin-1, and total LC3 and LC3-II upon early exposure to the preparations. Identification of the water-soluble bioactive component(s) present in the extract merit further investigation aiming to develop novel prevention and/or treatment agents efficacious against highly metastatic breast carcinomas.
[Luparello, Claudio; Ragona, Debora; Asaro, Dalia Maria Lucia; Lazzara, Valentina; Affranchi, Federica; Celi, Monica; Arizza, Vincenzo; Vazzana, Mirella] Univ Palermo, Dipartimento Sci & Tecnol Biol Chim & Farmaceut S, Viale Sci, I-90128 Palermo, Italy University of Palermo Luparello, C (corresponding author), Univ Palermo, Dipartimento Sci & Tecnol Biol Chim & Farmaceut S, Viale Sci, I-90128 Palermo, Italy.
claudio.luparello@unipa.it; debora0410ale@gmail.com; daliaasaro@libero.it; valentina.lazzara@community.unipa.it; federica.affranchi@community.unipa.it; monica.celi@unipa.it; vincenzo.arizza@unipa.it; mirella.vazzana@unipa.it Lazzara, Valentina/AEN-9546-2022; Arizza, Vincenzo/I-3477-2012 Vazzana, Mirella/0000-0001-6437-9409; Luparello, Claudio/0000-0001-9821-

5891; Arizza, Vincenzo/0000-0002-8772-7143 University of Palermo (Italy), grant Fondo Finalizzato alla Ricerca (FFR) 2018
University of Palermo (Italy), grant Fondo Finalizzato alla Ricerca (FFR) 2018 This research was funded by The University of Palermo (Italy), grant Fondo Finalizzato alla Ricerca (FFR) 2018 to C.L. and M.V. 54 27 29 0 3 MDPI
BASEL MDPI AG, Grosspeteranlage 5, CH-4052 BASEL, SWITZERLAND 2079-7737 BIOLOGY-BASEL Biology-Basel
DEC 2019 8 4 76
10.3390/biology8040076
http://dx.doi.org/10.3390/biology8040076 14
Biology Science Citation Index Expanded (SCI-EXPANDED)
Life Sciences & Biomedicine - Other Topics KA0VB 31600896
gold, Green Published 2025-06-24
WOS:000505517600008

J Illescas, O; Ferrero, G; Bel, A; Pardini, B; Tarallo, S; Ciniselli, CM; Noci, S; Daveri, E; Signoroni, S; Cattaneo, L; Mancini, A; Morelli, D; Milione, M; Cordero, F; Rivoltini, L; Verderio, P; Pasanisi, P; Vitellaro, M; Naccarati, A; Gariboldi, M Illescas, Oscar; Ferrero, Giulio; Bel, Antonino; Pardini, Barbara; Tarallo, Sonia; Ciniselli, Chiara M.; Noci, Sara; Daveri, Elena; Signoroni, Stefano; Cattaneo, Laura; Mancini, Andrea; Morelli, Daniele; Milione, Massimo; Cordero, Francesca; Rivoltini, Licia; Verderio, Paolo; Pasanisi, Patrizia; Vitellaro, Marco; Naccarati, Alessio; Gariboldi, Manuela
Modulation of faecal miRNAs highlights the preventive effects of a Mediterranean low-inflammatory dietary intervention
CLINICAL NUTRITION English Article
Familial Adenomatous Polyposis (FAP); Mediterranean low-inflammatory; intervention diet; Stool microRNA profiles; Intestinal tissue gene expression; Small RNA sequencing
COLORECTAL-CANCER; EXPRESSION; MICRORNA; MUCOSA; SAMPLE; PILOT; FAP Background: Dietary interventions have been proposed as therapeutic approaches for several diseases, including cancer. A low-inflammatory Mediterranean dietary intervention, conducted as a pilot study in subjects with Familial Adenomatous Polyposis (FAP), reduced markers of local and systemic inflammation. We aim to determine whether this diet may modulate faecal microRNA (miRNA) and gene expression in the gut. Methods: Changes in the faecal miRNome were evaluated by small RNA sequencing at baseline (T0), after the three-month intervention (T1), and after an additional three months (T2). Changes in the transcriptome of healthy rectal mucosa and adenomas were evaluated by RNA sequencing at T0 and T2. The identification of validated miRNA-gene interactions and functional analysis of miRNA targets were performed using in silico approaches. Results: Twenty-seven subjects were included in this study. It was observed that the diet modulated 29 faecal miRNAs ($p < 0.01$; |log2 Fold Change|>1), and this modulation persisted for three months after the intervention. Levels of miR-3612-3p and miR-941 correlated with the adherence to the diet, miR-3670 and miR-4252-5p with faecal calprotectin, and miR-3670 and miR-6867 with serum calprotectin. Seventy genes were differentially expressed between adenoma and normal tissue, and most were different before the dietary intervention but reached similar levels after the diet. Functional enrichment analysis identified the proinflammatory ERK1/2, cell cycle regulation, and nutrient response pathways as commonly regulated by the modulated miRNAs and genes. Conclusions: Faecal

miRNAs modulated by the dietary intervention target genes that participate in inflammation. Changes in levels of miRNAs and genes with oncogenic and tumour suppressor functions further support the potential cancer-preventive effect of the low-inflammatory Mediterranean diet. Clinical trial number registration: NCT04552405, Registered in ClinicalTrials.gov. (c) 2024 The Authors. Published by Elsevier Ltd. This is an open access article under the CC BY-NC-ND license (http://creativecommons.org/licenses/by-nc-nd/4.0/).

[Illescas, Oscar; Noci, Sara; Gariboldi, Manuela] Fdn IRCCS Ist Nazl Tumori, Mol Epigen Unit, Milan, Italy; [Ferrero, Giulio; Cordero, Francesca] Univ Turin, Dept Clin & Biol Sci, Turin, Italy; [Ferrero, Giulio; Cordero, Francesca] Univ Turin, Dept Comp Sci, Turin, Italy; [Bel, Antonino; Cattaneo, Laura; Milione, Massimo] Fdn IRCCS Ist Nazl Tumori, Pathol Div 1, Milan, Italy; [Pardini, Barbara; Tarallo, Sonia; Naccarati, Alessio] IRCCS Candiolo, Italian Inst Genom Med IIGM, Turin, Italy; [Pardini, Barbara; Tarallo, Sonia; Naccarati, Alessio] FPO IRCCS, Candiolo Canc Inst, Turin, Italy; [Ciniselli, Chiara M.; Verderio, Paolo] Fdn IRCCS Ist Nazl Tumori, Bioinformat & Biostat Unit, Milan, Italy; [Daveri, Elena; Rivoltini, Licia] Fdn IRCCS Ist Nazl Tumori, Unit Immunotherapy Human Tumors, Milan, Italy; [Signoroni, Stefano; Vitellaro, Marco] Fdn IRCCS Ist Nazl Tumori, Hereditary Digest Tract Tumors Unit, Milan, Italy; [Mancini, Andrea] Fdn IRCCS Ist Nazl Tumori, Diagnost & Therapeut Endoscopy Unit, Milan, Italy; [Morelli, Daniele] Fdn IRCCS Ist Nazl Tumori, Lab Med Div, Milan, Italy; [Pasanisi, Patrizia] Fdn IRCCS Ist Nazl Tumori, Unit Epidemiol & Prevent, Milan, Italy; [Gariboldi, Manuela] Fdn IRCCS Ist Nazl Tumori Milano, AmadeoLab, Via Amadeo 42, I-20133 Milan, Italy Fondazione IRCCS Istituto Nazionale Tumori Milan; University of Turin; University of Turin; Fondazione IRCCS Istituto Nazionale Tumori Milan; Italian Institute for Genomic Medicine (IIGM); IRCCS Fondazione del Piemonte per l'Oncologia; IRCCS Fondazione del Piemonte per l'Oncologia; Fondazione IRCCS Istituto Nazionale Tumori Milan; Fondazione IRCCS Istituto Nazionale Tumori Milan; Fondazione IRCCS Istituto Nazionale Tumori Milan; Fondazione IRCCS Istituto Nazionale Tumori Milan; Fondazione IRCCS Istituto Nazionale Tumori Milan; Fondazione IRCCS Istituto Nazionale Tumori Milan; Fondazione IRCCS Istituto Nazionale Tumori Milan

Gariboldi, M (corresponding author), Fdn IRCCS Ist Nazl Tumori Milano, AmadeoLab, Via Amadeo 42, I-20133 Milan, Italy.

manuela.gariboldi@istitutotumori.mi.it Belfiore, Antonino/B-4652-2011; Vitellaro, Marco/B-8278-2017; Pasanisi, Patrizia/F-4908-2017; Ciniselli, Chiara/C-4518-2017; Ferrero, Giulio/K-9508-2019; Pardini, Barbara/ABI-2295-2020; Daveri, Elena/C-6910-2019; Tarallo, Sonia/AAB-8656-2022; Cattaneo, Laura/ABI-6609-2020; Illescas Pomposo, Oscar/AFR-7445-2022; Signoroni, Stefano/D-6311-2017; Ferrero, Giulio/B-9838-2014; Pardini, Barbara/K-9451-2016; Noci, Sara/E-5425-2017 Ferrero, Giulio/0000-0002-4580-0680; Pardini, Barbara/0000-0001-9571-4257; Tarallo, Sonia/0000-0003-0887-2607; Noci, Sara/0000-0002-6227-3907; Illescas, Oscar/0000-0002-7326-5486 European Union [825410]; Fondazione Umberto Veronesi (FUV); Pezcoller Foundation; Italian Institute for Genomic Medicine (IIGM); Compagnia di San Paolo Torino, Italy; The 5 per 1000 Funds-MIUR-2014 European Union(European Union (EU)); Fondazione Umberto Veronesi (FUV)(Fondazione Umberto Veronesi); Pezcoller Foundation(ACEV Foundation); Italian Institute for Genomic Medicine (IIGM); Compagnia di San Paolo Torino,

Italy(Compagnia di San Paolo); The 5 per 1000 Funds-MIUR-2014 MG and MV are supported by funds obtained through an Italian law that allows taxpayers to allocate 0.5 percent of their income tax to a research institution of their choice (5 per 1000 Funds-MIUR-2014) . MG and AN are funded by the European Union's Horizon 2020 research and innovation program, grant agreement No 825410 (ONCOBIOME project) . OI is recipient of the Fondazione Umberto Veronesi (FUV) Post-doctoral fellowship 2021, 2022 and 2023. ED was supported by Pezcoller Foundation in 2021, and by FUV as Post-doctoral fellow in 2022-2023. BP, AN and ST were supported by the Italian Institute for Genomic Medicine (IIGM) and Compagnia di San Paolo Torino, Italy. 50 6 6 1 3 CHURCHILL LIVINGSTONE EDINBURGH JOURNAL PRODUCTION DEPT, ROBERT STEVENSON HOUSE, 1-3 BAXTERS PLACE, LEITH WALK, EDINBURGH EH1 3AF, MIDLOTHIAN, SCOTLAND 0261-5614 1532-1983 CLIN NUTR
Clin. Nutr. APR 2024 43 4 951
959 10.1016/j.clnu.2024.02.023
http://dx.doi.org/10.1016/j.clnu.2024.02.023 FEB 2024
9 Nutrition & Dietetics Science Citation Index Expanded (SCI-EXPANDED) Nutrition & Dietetics NU0K3 38422953 hybrid
2025-06-24 WOS:001202848600001
J Fu, SP; Arráez-Roman, D; Segura-Carretero, A; Menéndez, JA; Menéndez-Gutiérrez, MP; Micol, V; Fernández-Gutiérrez, A
Fu, Shaoping; Arraez-Roman, David; Segura-Carretero, Antonio; Menendez, Javier A.; Menendez-Gutierrez, Maria P.; Micol, Vicente; Fernandez-Gutierrez, Alberto Qualitative screening of phenolic compounds in olive leaf extracts by hyphenated liquid chromatography and preliminary evaluation of cytotoxic activity against human breast cancer cells
ANALYTICAL AND BIOANALYTICAL CHEMISTRY English
Article; Proceedings Paper 36th Colloquium Spectroscopicum Internationale AUG 30-SEP 03, 2009 Eotvos Lorand Univ, Budapest, HUNGARY Eotvos Lorand Univ Olive leaf; Phenolic compounds; High-performance liquid chromatography; Time-of-flight mass spectrometry; Ion trap multiple-stage tandem mass spectrometry; Breast cancer TANDEM MASS-SPECTROMETRY; OLEA-EUROPAEA L.; TIME-OF-FLIGHT; MEDITERRANEAN DIET; OLEUROPEIN AGLYCON; IDENTIFICATION; LEAVES; OIL; POLYPHENOLS; PRESSURE In this work, high-performance liquid chromatography (HPLC) coupled to electrospray time-of-flight mass spectrometry (ESI-TOF-MS) and electrospray ion trap multiple-stage tandem mass spectrometry (ESI-IT-MS(2)) has been applied to screen phenolic compounds in olive leaf extracts. The use of a small particle size C18 column (1.8 mu m) provided great resolution and made separation of a lot of isomers possible. The structural characterization was based on accurate mass data obtained by ESI-TOF-MS, and the nature of fragmentation ions were further confirmed by ESI-IT-MS(2) when possible. In addition, we employed tetrazolium salt (MTT)-based assays to assess the effects of olive leaf extracts on the growth of human tumor-derived cells. Upon this approach, we achieved an accurate profile of olive leaf phenolics along with the identification of several important isomers of secoiridoids and flavonoids. This will allow a better understanding of the complete composition of olive-leaf-bioactive compounds as well as their involvement in Olea europaea L. biochemical pathways. Importantly, olive leaf extracts exhibited dose-dependent inhibitory effects on the metabolic status (cell viability) of three breast cancer models in vitro. Since the tumoricidal activity of the extracts

should be mainly attributed to the identified olive leaf phenolics, these findings warrant further investigation at the structure-function molecular level to definitely establish the anticancer value of these phytochemicals. [Fu, Shaoping; Arraez-Roman, David; Segura-Carretero, Antonio; Fernandez-Gutierrez, Alberto] Univ Granada, Fac Sci, Dept Analyt Chem, E-18071 Granada, Spain; [Fu, Shaoping] Dalian Polytech Univ, Sch Biol & Food Engn, Inst Chem & Applicat Plant Resources, Dalian 116034, Peoples R China; [Micol, Vicente] Univ Miguel Hernandez, Inst Mol & Cell Biol, Alicante 03202, Spain; [Menendez, Javier A.] ICO Girona, IdIBGi, Girona 17007, Spain University of Granada; Dalian Polytechnic University; Universidad Miguel Hernandez de Elche; Universitat de Girona; Girona University Hospital Dr. Josep Trueta; Institut d'Investigacio Biomedica de Girona (IDIBGI); Institut Catala d'Oncologia Segura-Carretero, A (corresponding author), Univ Granada, Fac Sci, Dept Analyt Chem, C Fuentenueva, E-18071 Granada, Spain. ansegura@ugr.es Fu, Shaoping/H-9797-2012; Micol, Vicente/K-6841-2014; segura Carretero, Antonio/B-6867-2014; Fernandez Gutierrez, Alberto/M-8512-2014; Arraez-Roman, David/M-8256-2014; MENENDEZ MENENDEZ, JAVIER ABEL/C-6148-2016
segura Carretero, Antonio/0000-0002-5564-5338; Fernandez Gutierrez, Alberto/0000-0003-3647-2598; Micol, Vicente/0000-0001-8089-0696; Fu, Shaoping/0000-0001-6005-8835; Arraez-Roman, David/0000-0003-1267-6676; MENENDEZ MENENDEZ, JAVIER ABEL/0000-0001-8733-4561
38 120 130 2 58
SPRINGER HEIDELBERG HEIDELBERG TIERGARTENSTRASSE 17, D-69121 HEIDELBERG, GERMANY 1618-2642 ANAL BIOANAL CHEM Anal. Bioanal. Chem. MAY 2010 397 2
643 654 10.1007/s00216-010-3604-0
http://dx.doi.org/10.1007/s00216-010-3604-0 12
Biochemical Research Methods; Chemistry, Analytical
Science Citation Index Expanded (SCI-EXPANDED); Conference Proceedings Citation Index - Science (CPCI-S) Biochemistry & Molecular Biology; Chemistry 584QW 20238105
2025-06-24 WOS:000276768400027
J Kennedy, SA; Jarboui, MA; Srihari, S; Raso, C; Bryan, K; Dernayka, L; Charitou, T; Bernal-Llinares, M; Herrera-Montavez, C; Krstic, A; Matallanas, D; Kotlyar, M; Jurisica, I; Curak, J; Wong, V; Stagljar, I; LeBihan, T; Imrie, L; Pillai, P; Lynn, MA; Fasterius, E; Al-Khalili Szigyarto, C; Breen, J; Kiel, C; Serrano, L; Rauch, N; Rukhlenko, O; Kholodenko, BN; Iglesias-Martinez, LF; Ryan, CJ; Pilkington, R; Cammareri, P; Sansom, O; Shave, S; Auer, M; Horn, N; Klose, F; Ueffing, M; Boldt, K; Lynn, DJ; Kolch, W
Kennedy, Susan A.; Jarboui, Mohamed-Ali; Srihari, Sriganesh; Raso, Cinzia; Bryan, Kenneth; Dernayka, Layal; Charitou, Theodosia; Bernal-Llinares, Manuel; Herrera-Montavez, Carlos; Krstic, Aleksandar; Matallanas, David; Kotlyar, Max; Jurisica, Igor; Curak, Jasna; Wong, Victoria; Stagljar, Igor; LeBihan, Thierry; Imrie, Lisa; Pillai, Priyanka; Lynn, Miriam A.; Fasterius, Erik; Al-Khalili Szigyarto, Cristina; Breen, James; Kiel, Christina; Serrano, Luis; Rauch, Nora; Rukhlenko, Oleksii; Kholodenko, Boris N.; Iglesias-Martinez, Luis F.; Ryan, Colm J.; Pilkington, Ruth; Cammareri, Patrizia; Sansom, Owen; Shave, Steven; Auer, Manfred; Horn, Nicola; Klose, Franziska; Ueffing, Marius; Boldt, Karsten; Lynn, David J.; Kolch, Walter
Extensive rewiring of the EGFR network in colorectal cancer cells expressing transforming levels of KRAS^{G13D}
NATURE COMMUNICATIONS English Article

HUMAN INTERACTOME; INFORMATION-FLOW; COLON-CANCER; K-RAS; PROTEIN; MUTATIONS; KRAS; IDENTIFICATION; VISUALIZATION; SELECTION Protein-protein-interaction networks (PPINs) organize fundamental biological processes, but how oncogenic mutations impact these interactions and their functions at a network-level scale is poorly understood. Here, we analyze how a common oncogenic KRAS mutation (KRAS(G13D)) affects PPIN structure and function of the Epidermal Growth Factor Receptor (EGFR) network in colorectal cancer (CRC) cells. Mapping >6000 PPIs shows that this network is extensively rewired in cells expressing transforming levels of KRAS(G13D) (mtKRAS). The factors driving PPIN rewiring are multifactorial including changes in protein expression and phosphorylation. Mathematical modelling also suggests that the binding dynamics of low and high affinity KRAS interactors contribute to rewiring. PPIN rewiring substantially alters the composition of protein complexes, signal flow, transcriptional regulation, and cellular phenotype. These changes are validated by targeted and global experimental analysis. Importantly, genetic alterations in the most extensively rewired PPIN nodes occur frequently in CRC and are prognostic of poor patient outcomes. [Kennedy, Susan A.; Raso, Cinzia; Charitou, Theodosia; Herrera-Montavez, Carlos; Krstic, Aleksandar; Matallanas, David; Kiel, Christina; Rauch, Nora; Rukhlenko, Oleksii; Kholodenko, Boris N.; Iglesias-Martinez, Luis F.; Ryan, Colm J.; Pilkington, Ruth; Kolch, Walter] Univ Coll Dublin, Syst Biol Ireland, Dublin, Ireland; [Jarboui, Mohamed-Ali; Dernayka, Layal; Horn, Nicola; Klose, Franziska; Ueffing, Marius; Boldt, Karsten] Univ Tubingen, Inst Ophthalm Res, Tubingen, Germany; [Jarboui, Mohamed-Ali] Univ Tubingen, Werner Siemens Imaging Ctr, Tubingen, Germany; [Srihari, Sriganesh; Bryan, Kenneth; Charitou, Theodosia; Bernal-Llinares, Manuel; Pillai, Priyanka; Lynn, Miriam A.; Lynn, David J.] South Australian Hlth & Med Res Inst, EMBL Australia Grp, Adelaide, SA 5000, Australia; [Srihari, Sriganesh] QIMR Berghofer Med Res Inst, Brisbane, Qld 4006, Australia; [Kotlyar, Max; Jurisica, Igor] Univ Hlth Network, Krembil Res Inst, Toronto, ON, Canada; [Jurisica, Igor] Univ Toronto, Dept Med Biophys, Toronto, ON, Canada; [Jurisica, Igor] Univ Toronto, Dept Comp Sci, Toronto, ON, Canada; [Jurisica, Igor] Slovak Acad Sci, Inst Neuroimmunol, Bratislava, Slovakia; [Curak, Jasna; Wong, Victoria; Stagljar, Igor] Univ Toronto, Donnelly Ctr, Toronto, ON, Canada; [Curak, Jasna; Wong, Victoria; Stagljar, Igor] Univ Toronto, Dept Biochem, Toronto, ON, Canada; [Curak, Jasna; Wong, Victoria; Stagljar, Igor] Univ Toronto, Dept Mol Genet, Toronto, ON, Canada; [Stagljar, Igor] Mediterranean Inst Life Sci, Split, Croatia; [LeBihan, Thierry; Imrie, Lisa] Univ Edinburgh, Synthet & Syst Biol, Edinburgh, Midlothian, Scotland; [Fasterius, Erik; Al-Khalili Szigyarto, Cristina] KTH Royal Inst Technol, Sch Biotechnol, Stockholm, Sweden; [Al-Khalili Szigyarto, Cristina] KTH Royal Inst Technol, Sci Life Lab, Stockholm, Sweden; [Breen, James] Univ Adelaide, Bioinformat Hub, Sch Biol Sci, Adelaide, SA, Australia; [Breen, James] South Australian Hlth & Med Res Inst, Computat & Syst Biol Program, Adelaide, SA, Australia; [Kiel, Christina; Serrano, Luis] Barcelona Inst Sci & Technol, Ctr Genom Regulat, Barcelona, Spain; [Kiel, Christina; Kholodenko, Boris N.; Kolch, Walter] Univ Coll Dublin, Conway Inst, Dublin, Ireland; [Kholodenko, Boris N.] Yale Univ, Sch Med, Dept Pharmacol, New Haven, CT 06510 USA; [Ryan, Colm J.] Univ Coll Dublin, Sch Comp Sci, Dublin, Ireland; [Cammareri, Patrizia; Sansom, Owen] Canc Res

UK Beatson Inst, Glasgow, Lanark, Scotland; [Sansom, Owen] Glasgow Univ, Inst Canc Studies, Glasgow, Lanark, Scotland; [Shave, Steven; Auer, Manfred] Univ Edinburgh, Sch Biol Sci, Edinburgh, Midlothian, Scotland; [Shave, Steven; Auer, Manfred] Univ Edinburgh, Sch Biomed Sci, Edinburgh, Midlothian, Scotland; [Lynn, David J.] Flinders Univ S Australia, Coll Med & Publ Hlth, Bedford Pk, SA 5042, Australia; [Kolch, Walter] Univ Coll Dublin, Sch Med, Dublin, Ireland University College Dublin; Eberhard Karls University of Tubingen; Eberhard Karls University Hospital; Siemens AG; Eberhard Karls University of Tubingen; South Australian Health & Medical Research Institute (SAHMRI); QIMR Berghofer Medical Research Institute; Krembil Research Institute; University of Toronto; University Health Network Toronto; University of Toronto; University of Toronto; Slovak Academy of Sciences; Institute of Neuroimmunology, SAS; University of Toronto; University of Toronto; University of Toronto; University of Edinburgh; Royal Institute of Technology; Royal Institute of Technology; University of Adelaide; South Australian Health & Medical Research Institute (SAHMRI); Barcelona Institute of Science & Technology; Pompeu Fabra University; Centre de Regulacio Genomica (CRG); University College Dublin; Yale University; University College Dublin; Beatson Institute; University of Glasgow; University of Edinburgh; University of Edinburgh; Flinders University South Australia; University College Dublin

Boldt, K (corresponding author), Univ Tubingen, Inst Ophthalm Res, Tubingen, Germany.; Lynn, DJ (corresponding author), South Australian Hlth & Med Res Inst, EMBL Australia Grp, Adelaide, SA 5000, Australia.; Kolch, W (corresponding author), Univ Coll Dublin, Conway Inst, Dublin, Ireland.; Lynn, DJ (corresponding author), Flinders Univ S Australia, Coll Med & Publ Hlth, Bedford Pk, SA 5042, Australia.; Kolch, W (corresponding author), Univ Coll Dublin, Sch Med, Dublin, Ireland.

karsten.boldt@uni-tuebingen.de; david.lynn@sahmri.com; walter.kolch@ucd.ie   Srihari, Sriganesh/H-1740-2013; Serrano-Macin, Luis/M-1157-2015; Breen, Jimmy/HHZ-1361-2022; Szigyarto, Cristina/ADC-7830-2022; Lynn, Miriam/NJT-2858-2025; Kolch, Walter/ABF-2102-2021; Le Bihan, Thierry/AAN-2534-2020; Krstic, Aleksandar/KBA-7391-2024; Lynn, David/JZE-6811-2024; gomez, david/U-9465-2019; Admin, SBI/HGB-2738-2022; Jarboui, Mohamed Ali/C-8496-2013; Rukhlenko, Oleksii/G-6893-2014   Sansom, Owen J./0000-0001-9540-3010; Kennedy, Susan/0009-0003-3592-4967; Al-Khalili Szigyarto, Cristina/0000-0001-6990-1905; Iglesias-Martinez, Luis F./0000-0002-9110-2189; Lynn, David/0000-0003-4664-1404; Rauch, Nora/0000-0001-6009-5177; Jurisica, Igor/0000-0002-2507-946X; Krstic, Aleksandar/0000-0002-7128-5707; Ryan, Colm/0000-0003-2750-9854; Cammareri, Patrizia/0000-0002-5888-6903; Breen, James/0000-0001-6184-0925; Lynn, Miriam Anne/0000-0003-4300-1380; Kiel, Christina/0000-0001-8454-6008; Kholodenko, Boris/0000-0002-9483-4975; Imrie, Lisa/0000-0003-1115-1720; Jarboui, Mohamed Ali/0000-0002-5203-235X; Herrera Montavez, Carlos/0000-0001-8586-7778; Matallanas, David/0000-0002-2360-3141; Rukhlenko, Oleksii/0000-0003-1863-4987; Bernal Llinares, Manuel/0000-0002-7368-180X European Union FP7 Grant [278568]; Science Foundation Ireland [14/IA/2395]; SmartNanoTox [686098]; NanoCommons [731032]; MSCA-IF-2016 SAMNets [750688]; Science Foundation Ireland Career Development award [15-CDA-3495]; Canada Research Chair Program (CRC) [225404]; Krembil Foundation; Ontario Research Fund [GL2-01-030, 34876, ORF/DIG-501411, RE08-009];

Natural Sciences Research Council (NSERC) [203475]; Canada Foundation for Innovation (CFI) [225404, 30865]; IBM; EMBL Australia; ERC investigator Award [ColonCan 311301]; CRUK; Canadian Cancer Society Research Institute [703889]; Genome Canada via Ontario Genomics [9427, 9428]; Consortium Quebecois sur la Decouverte du Medicament (CQDM Quantum Leap); Brain Canada (Quantum Leap); CQDM Explore; OCE [23929]; Teagasc Walsh Fellowship; Tistou & Charlotte Kerstan Stiftung; eResearch SA; National Collaborative Research Infrastructure Strategy; Marie Curie Actions (MSCA) [750688] Funding Source: Marie Curie Actions (MSCA) European Union FP7 Grant; Science Foundation Ireland(Science Foundation Ireland (SFI)); SmartNanoTox; NanoCommons; MSCA-IF-2016 SAMNets; Science Foundation Ireland Career Development award(Science Foundation Ireland (SFI)); Canada Research Chair Program (CRC)(Canada Research Chairs); Krembil Foundation; Ontario Research Fund; Natural Sciences Research Council (NSERC)(Natural Sciences and Engineering Research Council of Canada (NSERC)); Canada Foundation for Innovation (CFI)(Canada Foundation for Innovation); IBM(International Business Machines (IBM)); EMBL Australia; ERC investigator Award(European Research Council (ERC)); CRUK(Cancer Research UK); Canadian Cancer Society Research Institute(Canadian Cancer Society (CCS)); Genome Canada via Ontario Genomics; Consortium Quebecois sur la Decouverte du Medicament (CQDM Quantum Leap); Brain Canada (Quantum Leap); CQDM Explore; OCE; Teagasc Walsh Fellowship; Tistou & Charlotte Kerstan Stiftung; eResearch SA; National Collaborative Research Infrastructure Strategy(Australian GovernmentDepartment of Industry, Innovation and Science); Marie Curie Actions (MSCA)(Marie Curie Actions) This work was supported by European Union FP7 Grant No. 278568 "PRIMES" and Science Foundation Ireland Investigator Program Grant 14/IA/2395 to W.K. B.K. is supported by SmartNanoTox (Grant no. 686098), NanoCommons (Grant no. 731032), O.R. by MSCA-IF-2016 SAMNets (Grant no. 750688). D.M. is supported by Science Foundation Ireland Career Development award 15-CDA-3495. I.J. is supported by the Canada Research Chair Program (CRC #225404), Krembil Foundation, Ontario Research Fund (GL2-01-030 and #34876), Natural Sciences Research Council (NSERC #203475), Canada Foundation for Innovation (CFI #225404, #30865), and IBM. D.J.L. is supported by EMBL Australia. O.S. is supported by ERC investigator Award ColonCan 311301 and CRUK. I.S. is supported by the Canadian Cancer Society Research Institute (#703889), Genome Canada via Ontario Genomics (#9427 & #9428), Ontario Research fund (ORF/DIG-501411 & RE08-009), Consortium Quebecois sur la Decouverte du Medicament (CQDM Quantum Leap) & Brain Canada (Quantum Leap), and CQDM Explore and OCE (#23929). T.C. was supported by a Teagasc Walsh Fellowship. MU and KB are supported by the Tistou & Charlotte Kerstan Stiftung. We thank Prof M. Uhlen for discussions and critical review of the HKE3 and HCT116 genome analysis. PRIMESDB (primesdb.eu) is supported by use of the NeCTAR Research Cloud and by eResearch SA. The NeCTAR Research Cloud is a collaborative Australian research platform supported by the National Collaborative Research Infrastructure Strategy. 68
46 46 1 11 NATURE PUBLISHING GROUP LONDON
MACMILLAN BUILDING, 4 CRINAN ST, LONDON N1 9XW, ENGLAND
2041-1723 NAT COMMUN Nat. Commun. JAN 24
2020 11 1 499
10.1038/s41467-019-14224-9
http://dx.doi.org/10.1038/s41467-019-14224-9 14

Multidisciplinary Sciences Science Citation Index Expanded (SCI-EXPANDED) Science & Technology - Other Topics MD4US
31980649 Green Published, gold 2025-06-24
WOS:000543967700005

J Khoshroo, M; Yazdanpanah, MJ; Yasrebi, S
Khoshroo, Mohammad; Yazdanpanah, Mohammad Javad; Yasrebi, Samira Serum Interleukin-24 Levels in Gastric and Breast Cancers and Non-cancerous Inflammations MIDDLE EAST JOURNAL OF CANCER English Article
Interleukin- 24; Breast cancer; Helicobacter pylori; Gastric cancer DIFFERENTIATION-ASSOCIATED GENE; HUMAN-MELANOMA DIFFERENTIATION; IL-24 EXPRESSION; RECEPTORS; CYTOKINE; CELLS; MDA-7/IL-24; GROWTH; MDA-7 Background: Human interleukin-24 (IL-24) is a cytokine belonging to the Interleukin-10 (IL-10) family of cytokines, also known as melanoma differentiation-associated gene 7, due to its discovery as a tumor-suppressing protein. A tumor-suppressing protein, IL-24 is produced by a variety of cells, including cancerous and non-cancerous healthy cells. The aim of the present study was to evaluate serum IL-24 concentrations in different cancers and compare them with non-cancerous inflammations. Method: In this case-control study, we divided a total of 200 subjects into five groups of 40 control subjects without cancer and without Helicobacter Pylori (H. Pylori) infection, patients with gastric cancer and H. Pylori infection, patients with H. Pylori infection without cancer, and patients with breast cancer and without H. Pylori infection. We measured the serum IL-24 level using specific enzyme-linked immunosorbent assay (ELISA) kit; we analysed the data with SPSS software. Results: The level of IL-24 was significantly higher in breast cancer group (160.65 +/--55pg/mL) (mean= SD) followed by gastric cancer with (76.2 +/--16.27 pg/mL) (mean +/- SD) and without (72.5 +/- 17.84 pg/mL) (mean +/- SD) H. Pylori infection groups. The level of IL-24 in H. Pylori infected patients and controls were (32.78 +/- 12.96 pg/mL) (mean +/- SD) and (27.4 +/- 8.5 pg/mL (mean +/- SD)), respectively. Conclusion: The mechanisms by which IL-24 is produced may be different between immune and cancer cells and serum IL-24 is more likely generated by immune cells than tumor cells. In breast cancer patients, estrogen or other sex hormones may provoke IL-24 production.
[Khoshroo, Mohammad; Yasrebi, Samira] Islamic Azad Univ, Fac Med, Dept Med, Qom Branch, Qom, Iran; [Yazdanpanah, Mohammad Javad] Islamic Azad Univ, Dept Microbiol, Naein Branch, Esfahan, Iran Islamic Azad University; Islamic Azad University Khoshroo, M (corresponding author), Islamic Azad Univ, Fac Med, Dept Med, Qom Branch, Qom, Iran. mohammadkhoshroo@yahoo.com
29 1 1 0 3 SHIRAZ UNIV MEDICAL SCIENCES SHIRAZ NEMAZEE HOSPITAL, SHIRAZ, 71934, IRAN
2008-6709 2008-6687 MIDDLE EAST J CANCER Middle East J. Cancer APR 2021 12 2 183
189 10.30476/mejc.2020.82945.1122
http://dx.doi.org/10.30476/mejc.2020.82945.1122
7 Oncology Emerging Sources Citation Index (ESCI)
Oncology SM4OA 2025-06-24
WOS:000657585700003

J Kalantari, T; Mohseni-Aghdam, B; Nasri, F; Tamaddon, G; Kalantari, M Kalantari, Tahereh; Mohseni-Aghdam, Bahram; Nasri, Fatemeh; Tamaddon, Gholamhossein; Kalantari, Mohsen The Efficacy of siRNA Specific

MDM2 in the Induction of Apoptosis in MCF-7 Breast Cancer Cell Line	MIDDLE EAST JOURNAL OF CANCER		English
Article		Transfected MCF-7 cells; Breast neoplasms; Bcl-2-associated X protein; BH3 interacting domain; BCL-2; p53	GASTRIC-CANCER; TUMOR SIZE; PROGNOSTIC INDICATOR; RISK-FACTORS; CARCINOMA; SURVIVAL

Background: A high number of human breast cancers overexpress the murine double minute (MDM2) gene which blocks the p53 protein which plays an important role in arresting the cell growth. The present study aimed to investigate the efficacy of siRNA specific MDM2 in knocking down MDM2 and its subsequent effects on p53 to exert antiproliferative effects on Michigan Cancer Foundation-7 (MCF-7) breast cancer cells. Method: In this in vitro study, we used the specific siRNA of the MDM2 gene to knock down the expression of the MDM2 protein in the MCF-7 cell line. The expression of MDM2, BCL2-associated X (BAX), BH3 interacting-domain death agonist (BID), and B cell lymphoma 2 (BCL2) genes was evaluated using the real-time polymerase chain reaction (PCR) technique. The apoptosis level was also assessed using the flow cytometry technique by the Annexin V test. Results: The results showed that the entry of MDM2 siRNA into MCF-7 cells significantly reduced the mRNA expression of MDM2 gene (P-value < 0.05). Besides, the expression of the antiapoptotic gene of BCL2 significantly decreased (P-value < 0.05) in transfected MCF-7 cells, while that of BAX and BID genes increased (P-value < 0.05). Conclusion: Based on the results, MDM2 inhibition is conducive to prevent cancer metastasis by the induction of cancer cell apoptosis. Moreover, it can be considered in cancer therapy along with chemotherapy.

[Kalantari, Tahereh; Mohseni-Aghdam, Bahram; Tamaddon, Gholamhossein] Shiraz Univ Med Sci, Sch Paramed Sci, Dept Med Lab Sci, Div Med Biotechnol, Shiraz, Iran; [Kalantari, Tahereh; Nasri, Fatemeh; Tamaddon, Gholamhossein] Shiraz Univ Med Sci, Sch Paramed Sci, Diagnost Lab Sci, Shiraz, Iran; [Kalantari, Tahereh; Nasri, Fatemeh; Tamaddon, Gholamhossein] Shiraz Univ Med Sci, Technol Res Ctr, Sch Paramed Sci, Shiraz, Iran; [Kalantari, Mohsen] Shiraz Univ, Sch Vet Med, Dept Clin Sci, Shiraz, Iran; [Kalantari, Tahereh] Shiraz Univ Med Sci, Sch Paramed Sci, Dept Med Lab Sci, Shiraz, Iran	Shiraz University of Medical Science; Shiraz University of Medical Science; Shiraz University of Medical Science; Shiraz University; Shiraz University of Medical Science	Kalantari, T (corresponding author), Shiraz Univ Med Sci, Sch Paramed Sci, Dept Med Lab Sci, Shiraz, Iran.	kalantari_t@sums.ac.ir	nasri, fatemeh/JES-8750-2023; tamaddon, gholamhossein/Q-9141-2017; kalantari, tahereh/I-2349-2015

Shiraz University of Medical Sciences, Shiraz, Iran [95-01-10-12383]	Shiraz University of Medical Sciences, Shiraz, Iran(Shiraz University of Medical Science) This study was financially supported by Shiraz University of Medical Sciences, Shiraz, Iran(Grant no: 95-01-10-12383).	21	0	0	0	0	SHIRAZ UNIV MEDICAL SCIENCES	SHIRAZ	NEMAZEE HOSPITAL, SHIRAZ, 71934, IRAN
2008-6709	2008-6687	MIDDLE EAST J CANCER	Middle East J. Cancer	OCT	2022	13	4		657
664	10.30476/mejc.2022.89113.150
http://dx.doi.org/10.30476/mejc.2022.89113.150
8	Oncology	Emerging Sources Citation Index (ESCI)
Oncology	5E9NW		2025-06-24
WOS:000865949200010

J Kalantari, T; Mohseni-Aghdam, B; Nasri, F; Tamaddon, G; Kalantari, M Kalantari, Tahereh; Mohseni-Aghdam, Bahram; Nasri, Fatemeh; Tamaddon, Gholamhossein; Kalantari, Mohsen The Efficacy of siRNA Specific MDM2 in the Induction of Apoptosis in MCF-7 Breast Cancer Cell Line MIDDLE EAST JOURNAL OF CANCER English Article Transfected MCF-7 cells; Breast neoplasms; Bcl-2-associated X protein; BH3 interacting domain; BCL-2; p53 FEEDBACK LOOP; P53; EXPRESSION; GENE; PROTEIN; RESISTANCE; CISPLATIN; ONCOGENE; BCL-2

Background: A high number of human breast cancers overexpress the murine double minute (MDM2) gene which blocks the p53 protein which plays an important role in arresting the cell growth. The present study aimed to investigate the efficacy of siRNA specific MDM2 in knocking down MDM2 and its subsequent effects on p53 to exert antiproliferative effects on Michigan Cancer Foundation-7 (MCF-7) breast cancer cells.Method: In this in vitro study, we used the specific siRNA of the MDM2 gene to knock down the expression of the MDM2 protein in the MCF-7 cell line. The expression of MDM2, BCL2-associated X (BAX), BH3 interacting-domain death agonist (BID), and B cell lymphoma 2 (BCL2) genes was evaluated using the real-time polymerase chain reaction (PCR) technique. The apoptosis level was also assessed using the flow cytometry technique by the Annexin V test.Results: The results showed that the entry of MDM2 siRNA into MCF-7 cells significantly reduced the mRNA expression of MDM2 gene (P-value < 0.05). Besides, the expression of the antiapoptotic gene of BCL2 significantly decreased (P-value < 0.05) in transfected MCF-7 cells, while that of BAX and BID genes increased (P -value < 0.05).Conclusion: Based on the results, MDM2 inhibition is conducive to prevent cancer metastasis by the induction of cancer cell apoptosis. Moreover, it can be considered in cancer therapy along with chemotherapy. [Kalantari, Tahereh; Mohseni-Aghdam, Bahram; Tamaddon, Gholamhossein] Shiraz Univ Med Sci, Sch Paramed Sci, Dept Med Lab Sci, Div Med Biotechnol, Shiraz, Iran; [Kalantari, Tahereh; Nasri, Fatemeh; Tamaddon, Gholamhossein] Shiraz Univ Med Sci, Sch Paramed Sci, Diagnost Lab Sci, Shiraz, Iran; [Kalantari, Tahereh; Nasri, Fatemeh; Tamaddon, Gholamhossein] Shiraz Univ Med Sci, Technol Res Ctr, Sch Paramed Sci, Shiraz, Iran; [Kalantari, Mohsen] Shiraz Univ, Sch Vet Med, Dept Clin Sci, Shiraz, Iran; [Kalantari, Tahereh] Shiraz Univ Med Sci, Sch Paramed Sci, Dept Med Lab Sci, Shiraz, Iran Shiraz University of Medical Science; Shiraz University of Medical Science; Shiraz University of Medical Science; Shiraz University; Shiraz University of Medical Science Kalantari, T (corresponding author), Shiraz Univ Med Sci, Sch Paramed Sci, Dept Med Lab Sci, Shiraz, Iran. kalantari_t@sums.ac.ir tamaddon, gholamhossein/Q-9141-2017; nasri, fatemeh/JES-8750-2023; kalantari, tahereh/I-2349-2015 Nasri, Fatemeh/0000-0002-4182-3930 Shiraz University of Medical Sciences, Shiraz, Iran [:95-01-10-12383] Shiraz University of Medical Sciences, Shiraz, Iran(Shiraz University of Medical Science) Funding This study was financially supported by Shiraz University of Medical Sciences, Shiraz, Iran (Grant no:95-01-10-12383) . 34 1 1 0 0 SHIRAZ UNIV MEDICAL SCIENCESSHIRAZ NEMAZEE HOSPITAL, SHIRAZ, 71934, IRAN 2008-6709 2008-6687 MIDDLE EAST J CANCER Middle East J. Cancer OCT 2022 13 4 565 572

10.30476/mejc.2021.89554.1532
http://dx.doi.org/10.30476/mejc.2021.89554.1532
8 Oncology Emerging Sources Citation Index (ESCI)
Oncology 5E9NW 2025-06-24
WOS:000865949200001
J Gutíerrez-Díaz, I; Fernández-Navarro, T; Salazar, N; Bartolome, B; Moreno-Arribas, MV; de Andres-Galiana, EJ; Fernández-Martinez, JL; Reyes-Gavilan, CGD; Gueirnonde, M; González, S Gutierrez-Diaz, Isabel; Fernandez-Navarro, Tania; Salazar, Nuria; Bartolome, Begona; Victoria Moreno-Arribas, M.; Juan de Andres-Galiana, Enrique; Luis Femandez-Martinez, Juan; de los Reyes-Gavilan, Clara G.; Gueirnonde, Miguel; Gonzalez, Sonia Adherence to a Mediterranean Diet Influences the Fecal Metabolic Profile of Microbial-Derived Phenolics in a Spanish Cohort of Middle-Age and Older People JOURNAL OF AGRICULTURAL AND FOOD CHEMISTRY
English Article
Mediterranean dietary pattern; phenolic compounds; fiber; fecal metabolites excretion; physical activity RED WINE/GRAPE JUICE; MAJOR FOOD SOURCES; HUMAN FECES; INTESTINAL MICROBIOTA; COLORECTAL-CANCER; BLACK TEA; POLYPHENOLS; WINE; ANTHOCYANINS; PATTERNS Despite the evidence regarding the influence of certain polyphenol food sources on the metabolic profile in feces, the association between the different phenolics provided by the diet and the fecal phenolic profile has not been elucidated. In this study, the composition of phenolic metabolites in fecal solutions was analyzed by UPLC-ESI-MS/MS in 74 volunteers. This fecal phenolic profile showed a high interindividual variation of the different compounds analyzed, phenylacetic and phenylpropionic acids being the major classes of phenolic metabolites excreted in feces. Subjects with higher adherence to a Mediterranean dietary pattern presented greater fecal concentrations of benzoic and 3-hydroxyphenylacetic acids, positively correlated with the intake of the principal classes and subclasses of polyphenols and fibers, and higher levels of Clostridium cluster XVIa and Faecalibacterium prausnitzii. These results provide a link among the Mediterranean dietary pattern, the bioactive compounds of the diet, and the fecal metabolic phenolic profile. [Gutierrez-Diaz, Isabel; Fernandez-Navarro, Tania; Gonzalez, Sonia] Univ Oviedo, Dept Funct Biol, C Julian Claveria S-N, Oviedo 33006, Asturias, Spain; [Gutierrez-Diaz, Isabel; Fernandez-Navarro, Tania; Salazar, Nuria; de los Reyes-Gavilan, Clara G.; Gueirnonde, Miguel] IPLA CSIC, Dept Microbiol & Biochem Dairy Prod, Paseo Rio Linares S-N, Villaviciosa 33300, Asturias, Spain; [Bartolome, Begona; Victoria Moreno-Arribas, M.] CEI UAM CSIC, CSIC UAM, Inst Food Sci Res CIAL, C Nicolas Cabrera 9, Madrid 28049, Spain; [Juan de Andres-Galiana, Enrique; Luis Femandez-Martinez, Juan] Univ Oviedo, Dept Appl Math, Asturias 33007, Spain University of Oviedo; Consejo Superior de Investigaciones Cientificas (CSIC); CSIC - Instituto de Productos Lacteos de Asturias (IPLA); Consejo Superior de Investigaciones Cientificas (CSIC); CSIC-UAM - Instituto de Investigacion en Ciencias de la Alimentacion (CIAL); University of Oviedo González, S (corresponding author), Univ Oviedo, Dept Funct Biol, C Julian Claveria S-N, Oviedo 33006, Asturias, Spain.
soniagsolares@uniovi.es deAndrés-Galiana, Enrique/AAH-4797-2019; Gonzalez-Solares, Sonia/P-6720-2015; Fernandez-Martinez, Juan Luis/M-1446-2017; Moreno-Arribas, M.Victoria/H-3841-2012; Salazar, Nuria/D-2189-2015; Bartolome, Begona/H-4857-

2012; G. de los Reyes-Gavilan, Clara/E-5443-2011 Gutierrez-Diaz, Isabel/0000-0003-0388-8431; deAndres-Galiana, Enrique J/0000-0001-8555-3832; Fernandez-Navarro, Tania/0000-0001-8695-6174; Gonzalez-Solares, Sonia/0000-0003-2602-7036; Fernandez-Martinez, Juan Luis/0000-0002-4758-2832; Moreno-Arribas, M.Victoria/0000-0002-4136-595X; Salazar, Nuria/0000-0003-1435-7628; Bartolome, Begona/0000-0002-4470-251X; G. de los Reyes-Gavilan, Clara/0000-0001-9396-6311 "Plan Regional de Investigation del Principado de Asturias", Spain [GRUPIN14-043]; Biopolis SL. within Spanish Ministry of Science and Innovation; Spanish MINECO [AGL2015-64.522-C2-R]; Clarin regional grant - Marie Curie CoFund European Program [ACB 14-08] "Plan Regional de Investigation del Principado de Asturias", Spain; Biopolis SL. within Spanish Ministry of Science and Innovation; Spanish MINECO(Spanish Government); Clarin regional grant - Marie Curie CoFund European Program This work was funded through the Grant GRUPIN14-043 "Microbiota Humana, Alimentacion y Salud" from "Plan Regional de Investigation del Principado de Asturias", Spain, and by a grant from Biopolis SL. within the framework of the e-CENIT Project SENIFOOD from the Spanish Ministry of Science and Innovation, and the Project AGL2015-64.522-C2-R from the Spanish MINECO. N.S. was the recipient of a postdoctoral contract supported by a Clarin regional grant (ACB 14-08) cofinanced by the Marie Curie CoFund European Program. 62 63 66 7 62 AMER CHEMICAL SOC WASHINGTON 1155 16TH ST, NW, WASHINGTON, DC 20036 USA 0021-8561 1520-5118 J AGR FOOD CHEM J. Agric. Food Chem. JAN 25 2017 65 3 586 595 10.1021/acs.jafc.6b04408 http://dx.doi.org/10.1021/acs.jafc.6b04408 10 Agriculture, Multidisciplinary; Chemistry, Applied; Food Science & Technology Science Citation Index Expanded (SCI-EXPANDED) Agriculture; Chemistry; Food Science & Technology EJ0PB 28029051 2025-06-24 WOS:000392910700007

J Wong, MCS; Huang, JJ; Lok, V; Wang, JX; Fung, F; Ding, HY; Zheng, ZJ Wong, Martin C. S.; Huang, Junjie; Lok, Veeleah; Wang, Jingxuan; Fung, Franklin; Ding, Hanyue; Zheng, Zhi-Jie Differences in Incidence and Mortality Trends of Colorectal Cancer Worldwide Based on Sex, Age, and Anatomic Location CLINICAL GASTROENTEROLOGY AND HEPATOLOGY English Article SEER; NORDCAN; Global; Economic Factor TEMPORAL PATTERNS; EUROPE BACKGROUND & AIMS: We studied incidence and mortality trends of colorectal cancer (CRC) in 39 countries according to age, sex, and anatomic location (colon vs rectum). METHODS: We retrieved incidence data from registries from 36 countries. The registries included the following: Cancer Incidence in 5 Continents volumes I to XI; the Surveillance, Epidemiology, and End Results Program of the National Cancer Institute; and the Nordic Cancer Registries from Europe. We obtained mortality data from 39 countries of the World Health Organization database. We evaluated average annual percentage changes in CRC incidence and mortality in the past decade using joinpoint regression analysis. RESULTS: From 2007 to 2016, 2006 to 2015, or 2005 to 2014, depending on the availability of the data, the incidence of colon cancer increased in 10 of 36 countries analyzed (all in Asia or Europe); India had the greatest increase, followed by Poland. All 10 of these countries have medium to high Human Development Index

(HDI) scores. Six countries had a decrease in colon cancer incidence; these countries had the highest HDI scores; the United States had the greatest decrease, followed by Israel. Seven countries (including all countries from Northern America) had a decrease in incidence among persons older than 50 years. Eight countries had an increase in colon cancer incidence among persons younger than 50 years, including the United Kingdom and India. Countries with a decreased or stable incidence among persons 50 years or older but a significant increase in persons younger than 50 years, included Germany, Australia, the United States, Sweden, Canada, and the United Kingdom. Only Italy had a decrease in CRC incidence among persons younger than 50 years. Among women, 12 of 36 countries (all from Asia and Europe) had an increase in colon cancer incidence and 7 countries had a decrease; India had the greatest increase followed by Slovenia. Five of 36 countries had an increase in incidence of rectal cancer and 8 countries had a decrease; Ecuador and Thailand had the greatest increases in incidence. The incidence of rectal cancer among persons younger than 50 years increased significantly in Finland, Australia, Canada, the United States, and The Netherlands. Four countries had an increase in the incidence of rectal cancer in women; Ecuador had the greatest increase followed by Thailand. The incidence of rectal cancer in women decreased in 8 countries. Among women younger than 50 years, rectal cancer incidence increased, despite a decrease in women older than 50 years, in Costa Rica, Slovenia, Japan, Slovakia, Canada, and the United States there was an increase in incidence, although their elder population had a stable or decreased incidence. Twenty-four countries reported a reduction in CRC mortality, including North America, Oceania, and most European countries. Nevertheless, some countries from Asia, Latin America, and Southern Europe had significant increases in CRC mortality. CONCLUSIONS: In an analysis of incidence and mortality databases from 39 countries, we found that the incidence of colon and rectal cancers has continued to increase in countries with medium to high HDI and in younger populations. Preventive strategies are needed for countries with increasing CRC and rectal cancer incidence and mortality. [Wong, Martin C. S.; Huang, Junjie; Lok, Veeleah; Wang, Jingxuan; Fung, Franklin; Ding, Hanyue] Chinese Univ Hong Kong, Fac Med, JC Sch Publ Hlth & Primary Care, Hong Kong, Peoples R China; [Zheng, Zhi-Jie] Peking Univ, Sch Publ Hlth, Dept Global Hlth, Beijing, Peoples R China
Chinese University of Hong Kong; Peking University Zheng, ZJ (corresponding author), Dept Global Hlth, 38 Xue Yuan Rd, Beijing, Peoples R China. zhengzj@bjmu.edu.cn Huang, Junjie/GOV-3795-2022; Wong, Martin/L-4568-2014 Ding, Hanyue/0000-0001-9977-0985; Zheng, Zhijie/0000-0002-0233-5010; Huang, Junjie/0000-0003-2382-4443; Wong, Martin/0000-0001-7706-9370
35 268 280 4 76 ELSEVIER SCIENCE INC NEW YORK STE 800, 230 PARK AVE, NEW YORK, NY 10169 USA 1542-3565 1542-7714 CLIN GASTROENTEROL H Clin. Gastroenterol. Hepatol. MAR 2021 19 5 955 + 10.1016/j.cgh.2020.02.026
http://dx.doi.org/10.1016/j.cgh.2020.02.026 APR 2021 73 Gastroenterology & Hepatology Science Citation Index Expanded (SCI-EXPANDED) Gastroenterology & Hepatology RN3ID 32088300 hybrid Y N 2025-06-24
WOS:000640244200014

J Tahmasebi, S; Shahin, B; Johari, MG; Akrami, M; Zanguri, V; Talei, A; Keumarsi, Z; Karimaghaei, N
Tahmasebi, Sedigheh; Shahin, Baharak; Johari, Masoumeh Ghoddusi; Akrami, Majid; Zanguri, Vahid; Talei, Abdolrasoul; Keumarsi, Zahra; Karimaghaei, Nazanin Does Existence of Ductal Carcinoma In Situ Accompanying Invasive Ductal Carcinoma Lead to Different Clinicopathological Features and Clinical Outcome? Report of a Breast Cancer Registry MIDDLE EAST JOURNAL OF CANCER English Article
Ductal carcinoma in situ; Carcinoma; Breast; Prognosis; Survival LOCAL RECURRENCE; GENOMIC DIFFERENCES; CONSERVING SURGERY; AMERICAN SOCIETY; STAGE-I; COMPONENT; MARKERS; RISK; IRRADIATION; EXPRESSION Background: Ductal carcinoma in situ (DCIS) is widely recognized as the precursor of invasive ductal carcinoma (IDC). We aimed to compare clinicopathological characteristics and prognosis between IDC with and without coexisting DCIS stratified by biological subtypes to evaluate the clinical outcome of these two groups. Method: Data from 5814 patients with IDC (32.4) and IDC/DCIS (67.6%), who underwent surgery from December 1993 through December 2019, were retrospectively assessed. We evaluated the prognosis of IDC with coexisting DCIS in different molecular subtypes. Results: IDC/DCIS patients were younger (P < 0.001). They also presented with a low tumor grade and had less lymph node involvement compared with the pure IDC patients. Compared with the patients with IDC, luminal B subtype was more frequent in those with IDC/DCIS, with 19.4% versus 13.2 %; human epidermal growth factor receptor-2 enriched subtype was also more frequently observed, with 12.2 vs. 8.7%. The 5-year disease-free survival (DFS) was higher in the IDC/DCIS patients (P = 0.036). The survival outcomes significantly improved in the cases with a higher amount of DCIS. The presence of coexisting DCIS (P 0.038), tumor size (P < 0.001), lymph node status (P = 0.005), lymph vascular invasion (P = 0.02), and molecular subtypes (P < 0.001) were found to be DFS-associated independent prognostic factors. Conclusion: DCIS along with IDC were associated with improved prognosis. The presence of DCIS may be a marker of lower aggressiveness, and could be noticed as a prognostic factor in future treatment algorithms. [Tahmasebi, Sedigheh; Shahin, Baharak; Johari, Masoumeh Ghoddusi; Akrami, Majid; Talei, Abdolrasoul; Keumarsi, Zahra] Shiraz Univ Med Sci, Breast Dis Res Ctr, Shiraz, Iran; [Zanguri, Vahid] Shiraz Univ Med Sci, Div Surg Oncol, Dept Surg, Shiraz, Iran; [Karimaghaei, Nazanin] Northumbria Healthcare NHS Fdn Trust, Core Med Trainee, Newcastle Upon Tyne, Tyne & Wear, England Shiraz University of Medical Science; Shiraz University of Medical Science Shahin, B (corresponding author), Shiraz Univ Med Sci, Breast Dis Res Ctr, Shiraz, Iran. baharakshahin@gmail.com Akrami, Majid/AAG-2773-2021; johari, Masoumeh/ABA-7112-2020; tahmasebi, sedigheh/D-5433-2018 Shiraz Breast Cancer Registry, Breast Diseases Research Center Shiraz Breast Cancer Registry, Breast Diseases Research Center This work was funded by Shiraz Breast Cancer Registry, Breast Diseases Research Center. 33 0 0
0 1 SHIRAZ UNIV MEDICAL SCIENCES SHIRAZ NEMAZEE HOSPITAL, SHIRAZ, 71934, IRAN 2008-6709 2008-6687
MIDDLE EAST J CANCER Middle East J. Cancer JUL 2022 13
3 472 482
10.30476/mejc.2022.86985.1384
http://dx.doi.org/10.30476/mejc.2022.86985.1384

11 Oncology Emerging Sources Citation Index (ESCI) Oncology 2U3AY 2025-06-24 WOS:000823032500005
J Huebner, K; Erlenbach-Wuensch, K; Prochazka, J; Sheraj, I; Hampel, C; Mrazkova, B; Michalcikova, T; Tureckova, J; Iatsiuk, V; Weissmann, A; Ferrazzi, F; Kunze, P; Nalli, E; Sammer, E; Gehring, A; Cheema, MM; Eckstein, M; Paap, EM; Soederberg, A; Fischer, C; Paul, S; Mahadevan, V; Ndreshkjana, B; Meier, MA; Muehlich, S; Geppert, C; Merkel, S; Grutzmann, R; Roehe, A; Banerjee, S; Hartmann, A; Sedlacek, R; Schneider-Stock, R
Huebner, Kerstin; Erlenbach-Wuensch, Katharina; Prochazka, Jan; Sheraj, Ilir; Hampel, Chuanpit; Mrazkova, Blanka; Michalcikova, Tereza; Tureckova, Jolana; Iatsiuk, Veronika; Weissmann, Anne; Ferrazzi, Fulvia; Kunze, Philipp; Nalli, Enise; Sammer, Elisabeth; Gehring, Annemarie; Cheema, Marie M.; Eckstein, Markus; Paap, Eva-Maria; Soederberg, Agnes; Fischer, Corinna; Paul, Sushmita; Mahadevan, Vijayalakshmi; Ndreshkjana, Benardina; Meier, Melanie A.; Muehlich, Susanne; Geppert, Carol, I; Merkel, Susanne; Grutzmann, Robert; Roehe, Adriana; Banerjee, Sreeparna; Hartmann, Arndt; Sedlacek, Radislav; Schneider-Stock, Regine
ATF2 loss promotes tumor invasion in colorectal cancer cells via upregulation of cancer driver TROP2 CELLULAR AND MOLECULAR LIFE SCIENCES English Article
De-adhesion; Migration; Intratumoral heterogeneity; Liver metastasis; EMT; CAM model ACTIVATING TRANSCRIPTION FACTOR-2; PROSTATE-CANCER; METASTASIS; SENSITIVITY; MUTATIONS; BIOMARKER; SURVIVAL; JUN In cancer, the activating transcription factor 2 (ATF2) has pleiotropic functions in cellular responses to growth stimuli, damage, or inflammation. Due to only limited studies, the significance of ATF2 in colorectal cancer (CRC) is not well understood. We report that low ATF2 levels correlated with worse prognosis and tumor aggressiveness in CRC patients. NanoString gene expression and ChIP analysis confirmed trophoblast cell surface antigen 2 (TROP2) as a novel inhibitory ATF2 target gene. This inverse correlation was further observed in primary human tumor tissues. Immunostainings revealed that high intratumoral heterogeneity for ATF2 and TROP2 expression was sustained also in liver metastasis. Mechanistically, our in vitro data of CRISPR/Cas9-generated ATF2 knockout (KO) clones revealed that high TROP2 levels were critical for cell de-adhesion and increased cell migration without triggering EMT. TROP2 was enriched in filopodia and displaced Paxillin from adherens junctions. In vivo imaging, micro-computer tomography, and immunostainings verified that an ATF2(KO)/TROP2(high) status triggered tumor invasiveness in in vivo mouse and chicken xenograft models. In silico analysis provided direct support that ATF2(low)/TROP2(high) expression status defined high-risk CRC patients. Finally, our data demonstrate that ATF2 acts as a tumor suppressor by inhibiting the cancer driver TROP2. Therapeutic TROP2 targeting might prevent particularly the first steps in metastasis, i.e., the de-adhesion and invasion of colon cancer cells. [Huebner, Kerstin; Hampel, Chuanpit; Weissmann, Anne; Kunze, Philipp; Nalli, Enise; Sammer, Elisabeth; Gehring, Annemarie; Paap, Eva-Maria; Soederberg, Agnes; Fischer, Corinna; Ndreshkjana, Benardina; Schneider-Stock, Regine] Friedrich Alexander Univ Erlangen Nurnberg FAU, Univ Hosp Erlangen, Inst Pathol, Expt Tumor Pathol, Univ Str 22, D-91054 Erlangen, Germany; [Erlenbach-Wuensch, Katharina; Ferrazzi, Fulvia; Eckstein, Markus;

Geppert, Carol, I; Hartmann, Arndt] Friedrich Alexander Univ Erlangen Nurnberg FAU, Univ Hosp Erlangen, Inst Pathol, D-91054 Erlangen, Germany; [Prochazka, Jan; Mrazkova, Blanka; Michalcikova, Tereza; Tureckova, Jolana; Iatsiuk, Veronika; Cheema, Marie M.; Sedlacek, Radislav] Inst Mol Genet ASCR, Czech Ctr Phenogen, Vvi, Prague 14220, Czech Republic; [Sheraj, Ilir; Banerjee, Sreeparna] Middle East Tech Univ, Dept Biol Sci, TR-06800 Ankara, Turkey; [Ferrazzi, Fulvia] Friedrich Alexander Univ Erlangen Nurnberg, Univ Hosp Erlangen, Inst Pathol, Dept Nephropathol, D-91054 Erlangen, Germany; [Paul, Sushmita] Indian Inst Technol Jodhpur, Dept Biosci & Bioengn, Jodhpur 342037, Rajasthan, India; [Mahadevan, Vijayalakshmi] Inst Bioinformat & Appl Biotechnol IBAB, Bangalore 560100, Karnataka, India; [Meier, Melanie A.; Muehlich, Susanne] Friedrich Alexander Univ Erlangen Nurnberg, Dept Chem & Pharm Mol & Clin Pharm, D-91058 Erlangen, Germany; [Merkel, Susanne; Grutzmann, Robert] Friedrich Alexander Univ Erlangen Nurnberg, Univ Hosp Erlangen, Dept Surg, D-91054 Erlangen, Germany; [Grutzmann, Robert; Hartmann, Arndt; Schneider-Stock, Regine] Friedrich Alexander Univ Erlangen Nurnberg, Univ Hosp Erlangen, Comprehens Canc Ctr Erlangen EMN CCC ER EMN, D-91054 Erlangen, Germany; [Roehe, Adriana] Fed Univ Hlth Sci Porto Alegre, Dept Pathol & Legal Med, BR-90050170 Porto Alegre, RS, Brazil   University of Erlangen Nuremberg; University of Erlangen Nuremberg; Czech Academy of Sciences; Institute of Molecular Genetics of the Czech Academy of Sciences; Middle East Technical University; University of Erlangen Nuremberg; Indian Institute of Technology System (IIT System); Indian Institute of Technology (IIT) - Jodhpur; University of Erlangen Nuremberg; University of Erlangen Nuremberg; University of Erlangen Nuremberg

Schneider-Stock, R (corresponding author), Friedrich Alexander Univ Erlangen Nurnberg FAU, Univ Hosp Erlangen, Inst Pathol, Expt Tumor Pathol, Univ Str 22, D-91054 Erlangen, Germany.; Schneider-Stock, R (corresponding author), Friedrich Alexander Univ Erlangen Nurnberg, Univ Hosp Erlangen, Comprehens Canc Ctr Erlangen EMN CCC ER EMN, D-91054 Erlangen, Germany.

regine.schneider-stock@uk-erlangen.de   Banerjee, Sreeparna/AAZ-6742-2020; Ferrazzi, Fulvia/C-1161-2008; Eckstein, Markus/KPB-0180-2024; Sheraj, Ilir/LUZ-8650-2024; Sedlacek, Radislav/G-4408-2014; Prochazka, Jan/HQZ-4419-2023; Mrazkova, Blanka/V-7146-2017   Muehlich, Susanne/0000-0003-2001-5755; Huebner, Kerstin/0009-0009-8791-0317; Eckstein, Markus/0000-0001-5418-3349; Sheraj, Ilir/0000-0001-6020-0256; Mrazkova, Blanka/0000-0003-1985-2361; Mahadevan, Vijayalakshmi/0000-0003-1102-9979 Projekt DEAL; European Cooperation in Science and Technology (COST) [CA17118]; Bavarian-Czech University Agency [BTHAAP-2018-9, BTHA-JC-2019-1]; Manfred-Stolte-Stiftung (Bayreuth, Germany); Deutsche Forschungsgemeinschaft (DFG) [SCHN477/18-1, SFB TRR 305-Z01]; German Academic Exchange Service (DAAD); Bavarian Equal Opportunities Sponsorship for Realization Equal Opportunities for Women in Research and Teaching; Academy of Sciences of the Czech Republic [RVO 68378050]; Czech Center for Phenogenomics by the Ministry of Education, Youth and Sports [LM2015040]; Ministry of Education, Youth and Sports and Education Research and Development Foundation [CZ.02.1.01/0.0/0.0/16_013/0001789, CZ.1.05/2.1.00/19.0395]; Biotechnology and Biomedicine Center of the Academy of Sciences and Charles University in Vestec [Z.1.05/1.1.00/02.0109] Projekt DEAL; European Cooperation in Science and Technology (COST)(European

Cooperation in Science and Technology (COST)); Bavarian-Czech University Agency; Manfred-Stolte-Stiftung (Bayreuth, Germany); Deutsche Forschungsgemeinschaft (DFG)(German Research Foundation (DFG)); German Academic Exchange Service (DAAD)(Deutscher Akademischer Austausch Dienst (DAAD)); Bavarian Equal Opportunities Sponsorship for Realization Equal Opportunities for Women in Research and Teaching; Academy of Sciences of the Czech Republic(Czech Academy of Sciences); Czech Center for Phenogenomics by the Ministry of Education, Youth and Sports; Ministry of Education, Youth and Sports and Education Research and Development Foundation; Biotechnology and Biomedicine Center of the Academy of Sciences and Charles University in VestecOpen Access funding enabled and organized by Projekt DEAL. This article is partly based on work from COST Action CA17118, supported by the European Cooperation in Science and Technology (COST; www.cost.eu).K.H., J. P. and R.S.S. were supported by grants from the Bavarian-Czech University Agency (BTHAAP-2018-9; BTHA-JC-2019-1), the Manfred-Stolte-Stiftung (Bayreuth, Germany) and the Deutsche Forschungsgemeinschaft (DFG) SCHN477/18-1. K.H. was sponsored by a short-term grant from the German Academic Exchange Service (DAAD) and by a Bavarian Equal Opportunities Sponsorship for Realization Equal Opportunities for Women in Research and Teaching. J.P., T.M., M.K., and R.S. were supported by RVO 68378050 from the Academy of Sciences of the Czech Republic; LM2015040 Czech Center for Phenogenomics by the Ministry of Education, Youth and Sports; CZ.02.1.01/0.0/0.0/16_ 013/0001789 Upgrade of the Czech Center for Phenogenomics: developing toward translation research by the Ministry of Education, Youth and Sports and Education Research and Development Foundation; Z.1.05/1.1.00/02.0109 Biotechnology and Biomedicine Center of the Academy of Sciences and Charles University in Vestec; and CZ. 1.05/2.1.00/19.0395 Higher quality and capacity for transgenic models by the Ministry of Education, Youth and Sports and Education Research and Development Foundation. F.F. was supported by the Deutsche Forschungsgemeinschaft (DFG)-SFB TRR 305-Z01.
45 18 18 2 16 SPRINGER BASEL AG BASEL
PICASSOPLATZ 4, BASEL, 4052, SWITZERLAND 1420-682X
1420-9071 CELL MOL LIFE SCI Cell. Mol. Life Sci.
AUG 2022 79 8 423
10.1007/s00018-022-04445-5
http://dx.doi.org/10.1007/s00018-022-04445-5 17
Biochemistry & Molecular Biology; Cell Biology Science Citation Index Expanded (SCI-EXPANDED) Biochemistry & Molecular Biology; Cell Biology 2Y6QC 35838828 hybrid, Green Published
2025-06-24 WOS:000826018000001
J Gigic, B; Boeing, H; Toth, R; Böhm, J; Habermann, N; Scherer, D; Schrotz-King, P; Abbenhardt-Martin, C; Skender, S; Brenner, H; Chang-Claude, J; Hoffmeister, M; Syrjala, K; Jacobsen, PB; Schneider, M; Ulrich, A; Ulrich, CM Gigic, Biljana; Boeing, Heiner; Toth, Reka; Bohm, Jurgen; Habermann, Nina; Scherer, Dominique; Schrotz-King, Petra; Abbenhardt-Martin, Clare; Skender, Stephanie; Brenner, Hermann; Chang-Claude, Jenny; Hoffmeister, Michael; Syrjala, Karen; Jacobsen, Paul B.; Schneider, Martin; Ulrich, Alexis; Ulrich, Cornelia M.
Associations Between Dietary Patterns and Longitudinal Quality of Life Changes in Colorectal Cancer Patients: The ColoCare Study NUTRITION AND CANCER-AN INTERNATIONAL JOURNAL
English Article

EUROPEAN-ORGANIZATION; MEDITERRANEAN DIET; FECAL INCONTINENCE; STYLE FACTORS; POPULATION; NUTRITION; DIAGNOSIS; SURVIVAL; FIBER; IMPACT   Quality of life (QoL) is an important clinical outcome in cancer patients. We investigated associations between dietary patterns and QoL changes in colorectal cancer (CRC) patients. The study included 192 CRC patients with available EORTC QLQ-C30 data before and 12 months post-surgery and food frequency questionnaire data at 12 months post-surgery. Principal component analysis was used to identify dietary patterns. Multivariate regression models assessed associations between dietary patterns and QoL changes over time. We identified four major dietary patterns: "Western" dietary pattern characterized by high consumption of potatoes, red and processed meat, poultry, and cakes, "fruit& vegetable" pattern: high intake of vegetables, fruits, vegetable oils, and soy products, "bread& butter" pattern: high intake of bread, butter and margarine, and "high-carb" pattern: high consumption of pasta, grains, nonalcoholic beverages, sauces and condiments. Patients following a "Western" diet had lower chances to improve in physical functioning (OR = 0.45 [0.21-0.99]), constipation (OR = 0.30 [0.13-0.72]) and diarrhea (OR: 0.44 [0.20-0.98]) over time. Patients following a "fruit& vegetable" diet showed improving diarrhea scores (OR: 2.52 [1.21-5.34]. A "Western" dietary pattern after surgery is inversely associated with QoL in CRC patients, whereas a diet rich in fruits and vegetables may be beneficial for patients' QoL over time. [Gigic, Biljana; Schneider, Martin; Ulrich, Alexis] Univ Clin Heidelberg, Dept Surg, Heidelberg, Germany; [Gigic, Biljana; Schrotz-King, Petra; Abbenhardt-Martin, Clare; Skender, Stephanie; Brenner, Hermann; Ulrich, Cornelia M.] Natl Ctr Tumor Dis, Div Prevent Oncol, Heidelberg, Germany; [Gigic, Biljana; Schrotz-King, Petra; Abbenhardt-Martin, Clare; Skender, Stephanie; Brenner, Hermann; Ulrich, Cornelia M.] German Canc Res Ctr, Heidelberg, Germany; [Gigic, Biljana; Brenner, Hermann] German Canc Res Ctr, German Canc Consortium DKTK, Heidelberg, Germany; [Boeing, Heiner] German Inst Human Nutr, Dept Epidemiol, Potsdam, Germany; [Toth, Reka] German Canc Res Ctr, Div Epigen & Canc Risk Factors, Heidelberg, Germany; [Bohm, Jurgen; Ulrich, Cornelia M.] Huntsman Canc Inst, Populat Sci, Salt Lake City, UT 84112 USA; [Habermann, Nina] European Mol Biol Lab, Genome Biol Unit, Heidelberg, Germany; [Scherer, Dominique] Heidelberg Univ, Inst Med Biometry & Informat, Heidelberg, Germany; [Brenner, Hermann; Hoffmeister, Michael] German Canc Res Ctr, Div Clin Epidemiol & Aging Res, Heidelberg, Germany; [Chang-Claude, Jenny] German Canc Res Ctr, Div Canc Epidemiol, Heidelberg, Germany; [Syrjala, Karen] Fred Hutchinson Canc Res Ctr, Div Clin Res, 1124 Columbia St, Seattle, WA 98104 USA; [Jacobsen, Paul B.] H Lee Moffitt Canc Ctr & Res Inst, Dept Hlth Outcomes & Behav, Tampa, FL USA; [Ulrich, Cornelia M.] Fred Hutchinson Canc Res Ctr, Canc Prevent Program, 1124 Columbia St, Seattle, WA 98104 USA   Ruprecht Karls University Heidelberg; Helmholtz Association; German Cancer Research Center (DKFZ); Ruprecht Karls University Heidelberg; National Center for Tumor Diseases; Helmholtz Association; German Cancer Research Center (DKFZ); Helmholtz Association; German Cancer Research Center (DKFZ); Leibniz Association; Deutsches Institut fur Ernahrungsforschung Potsdam-Rehbrucke (DIfE); Helmholtz Association; German Cancer Research Center (DKFZ); Utah System of Higher Education; University of Utah; Huntsman Cancer Institute; European Molecular Biology Laboratory (EMBL); Ruprecht Karls

University Heidelberg; Helmholtz Association; German Cancer Research Center (DKFZ); Helmholtz Association; German Cancer Research Center (DKFZ); Fred Hutchinson Cancer Center; H Lee Moffitt Cancer Center & Research Institute; Fred Hutchinson Cancer Center Ulrich, CM (corresponding author), Huntsman Canc Inst, 2000 Circle Hope,Rm 4725, Salt Lake City, UT 84112 USA.
neli.ulrich@hci.utah.edu Hoffmeister, Michael/T-7187-2019; Toth, Reka/AAB-1695-2020; Schneider, Martin/H-3745-2011; Brenner, Hermann/ABE-6383-2020 Brenner, Hermann/0000-0002-6129-1572; Toth, Reka/0000-0002-6096-1052; Jacobsen, Paul/0000-0002-4427-8800; Hoffmeister, Michael/0000-0002-8307-3197 Matthias Lackas Foundation; ERA-NET on Translational Cancer Research (TRANSCAN) project [01KT1503]; National Cancer Institute [NIH R01 CA 189184, NIH U01 CA 206110, R01CA189184]; Huntsman Cancer Foundation; NIH [R01 CA207371]; German Consortium of Translational Cancer Research (DKTK); Transcan-ERANET grant [01KT1503]; German Cancer Research Center (DKFZ), Heidelberg, Germany Matthias Lackas Foundation; ERA-NET on Translational Cancer Research (TRANSCAN) project; National Cancer Institute(United States Department of Health & Human ServicesNational Institutes of Health (NIH) - USANIH National Cancer Institute (NCI)); Huntsman Cancer Foundation; NIH(United States Department of Health & Human ServicesNational Institutes of Health (NIH) - USA); German Consortium of Translational Cancer Research (DKTK); Transcan-ERANET grant; German Cancer Research Center (DKFZ), Heidelberg, Germany Biljana Gigic was funded by the Matthias Lackas Foundation, the ERA-NET on Translational Cancer Research (TRANSCAN) project 01KT1503, and the National Cancer Institute project R01CA189184. Dr. Cornelia M Ulrich and Dr. Jurgen Bohm were funded by the National Cancer Institute projects NIH R01 CA 189184 and NIH U01 CA 206110 and the Huntsman Cancer Foundation. Dr. Ulrich was further funded by NIH R01 CA207371. The Heidelberg ColoCare study was supported by the Matthias Lackas Foundation, the German Consortium of Translational Cancer Research (DKTK) and the Transcan-ERANET grant 01KT1503 (FOCUS consortium) and institutional funding from the German Cancer Research Center (DKFZ), Heidelberg, Germany to the Division of Preventive Oncology (Dr. Cornelia M. Ulrich, Dr. Hermann Brenner) at the National Center for Tumor Diseases (NCT), Heidelberg, Germany. 46 45 47 0 17 ROUTLEDGE JOURNALS, TAYLOR & FRANCIS LTD ABINGDON 2-4 PARK SQUARE, MILTON PARK, ABINGDON OX14 4RN, OXON, ENGLAND 0163-5581 1532-7914 NUTR CANCER Nutr. Cancer 2018 70 1 51 60
10.1080/01635581.2018.1397707
http://dx.doi.org/10.1080/01635581.2018.1397707
10 Oncology; Nutrition & Dietetics Science Citation Index Expanded (SCI-EXPANDED); Social Science Citation Index (SSCI) Oncology; Nutrition & Dietetics GB7MK 29244538
Green Accepted 2025-06-24 WOS:000429258500007
J Battino, M; Forbes-Hernández, TY; Gasparrini, M; Afrin, S; Cianciosi, D; Zhang, JJ; Manna, PP; Reboredo-Rodríguez, P; Lopez, AV; Quiles, JL; Mezzetti, B; Bompadre, S; Xiao, JB; Giampieri, F Battino, Maurizio; Forbes-Hernandez, Tamara Y.; Gasparrini, Massimiliano; Afrin, Sadia; Cianciosi, Danila; Zhang, Jiaojiao; Manna, Piera P.; Reboredo-Rodriguez, Patricia; Varela Lopez, Alfonso; Quiles, Jose L.; Mezzetti, Bruno; Bompadre, Stefano; Xiao, Jianbo; Giampieri, Francesca Relevance of functional foods in the Mediterranean diet: the role of olive

oil, berries and honey in the prevention of cancer and cardiovascular diseases CRITICAL REVIEWS IN FOOD SCIENCE AND NUTRITION English Article; Proceedings Paper 3rd International Symposium on Phytochemicals in Medicine and Food (ISPMF) AUG 25-30, 2018 Kunming, PEOPLES R CHINA Phytochem Soc Europe, Int Soc Chinese Med, Physiol Soc Japan, Phytochem Soc Asia Mediterranean diet; olive oil; berries; honey; cardiovascular diseases; cancer BLACK-RASPBERRY EXTRACT; RUBUS-ALCEIFOLIUS POIR; XENOBIOTIC-METABOLIZING ENZYMES; COLONIC ADENOMA DEVELOPMENT; RANDOMIZED CONTROLLED-TRIAL; TO-MESENCHYMAL TRANSITION; CELLS IN-VITRO; VIRGIN OLIVE; BREAST-CANCER; BLOOD-PRESSURE The traditional Mediterranean diet (MedDiet) is a well-known dietary pattern associated with longevity and improvement of life quality as it reduces the risk of the most common chronic pathologies, such as cancer and cardiovascular diseases (CVDs), that represent the principal cause of death worldwide. One of the most characteristic foods of MedDiet is olive oil, a very complex matrix, which constitutes the main source of fats and is used in the preparation of foods, both raw as an ingredient in recipes, and in cooking. Similarly, strawberries and raspberries are tasty and powerful foods which are commonly consumed in the Mediterranean area in fresh and processed forms and have attracted the scientific and consumer attention worldwide for their beneficial properties for human health. Besides olive oil and berries, honey has lately been introduced in the MedDiet thanks to its relevant nutritional, phytochemical and antioxidant profile. It is a sweet substance that has recently been classified as a functional food. The aim of this review is to present and discuss the recent evidence, obtained from in vitro, in vivo and epidemiological studies, on the potential roles exerted by these foods in the prevention and progression of different types of cancer and CVDs. [Battino, Maurizio; Forbes-Hernandez, Tamara Y.; Gasparrini, Massimiliano; Afrin, Sadia; Cianciosi, Danila; Zhang, Jiaojiao; Manna, Piera P.; Reboredo-Rodriguez, Patricia; Varela Lopez, Alfonso; Giampieri, Francesca] Univ Politecn Marche, Dept Clin Sci, Fac Med, Ancona, Italy; [Reboredo-Rodriguez, Patricia] Univ Vigo, Dept Analyt & Food Chem, Nutr & Bromatol Grp, Fac Sci, Ourense Campus, Orense, Spain; [Varela Lopez, Alfonso; Quiles, Jose L.] Univ Granada, Inst Nutr & Food Technol Jos Mataix, Biomed Res Ctr, Dept Physiol, Granada, Spain; [Mezzetti, Bruno] Univ Politecn Marche, Dipartimento Sci Agr Alimentari & Ambientali, Ancona, Italy; [Bompadre, Stefano] Univ Politecn Marche, Dipartimento Sci Biomed & Sanita Pubbl, Ancona, Italy; [Xiao, Jianbo] Univ Macau, Inst Chinese Med Sci, Taipa, Macau, Peoples R China; [Battino, Maurizio; Forbes-Hernandez, Tamara Y.; Giampieri, Francesca] Univ Vigo, CACTI, CITACA, Nutr & Food Sci Grp,Dept Analyt & Food Chem, Vigo Campus, Vigo, Spain Marche Polytechnic University; Universidade de Vigo; University of Granada; Marche Polytechnic University; Marche Polytechnic University; University of Macau; Universidade de Vigo Battino, M; Giampieri, F (corresponding author), Univ Politecn Marche, Fac Med, Dipartimento Sci Clin Specialist & Odontosmatol D, Sez Biochim, Via Ranieri 65, I-60131 Ancona, Italy.; Battino, M; Giampieri, F (corresponding author), Univ Vigo, CACTI, CITACA, Nutr & Food Sci Grp,Dept Analyt & Food Chem, Vigo Campus, Vigo, Spain. m.a.battino@univpm.it; f.giampieri@univpm.it jian, xiao/AAH-2773-2020; Zhang, Jiaojiao/AAN-1038-2020; Reboredo-Rodríguez, Patricia/AAH-2388-

2019; Mezzetti, Bruno/AAB-8500-2019; Afrin, Sadia/AAQ-5030-2020; Cianciosi, Danila/H-7405-2019; Battino, Maurizio/E-6103-2012; Varela-Lopez, Alfonso/F-8055-2016; Forbes Hernandez, Tamara/AAB-1872-2021; Quiles, Jose L./C-6911-2013; Xiao, Jianbo/C-7323-2012; Zhang, Jiaojiao/ADL-2406-2022; Giampieri, Francesca/I-1911-2015
Battino, Maurizio/0000-0002-7250-1782; Mezzetti, Bruno/0000-0001-9307-812X; Varela-Lopez, Alfonso/0000-0002-0504-5086; Forbes Hernandez, Tamara/0000-0001-7021-9276; Quiles, Jose L./0000-0002-9048-9086; Cianciosi, Danila/0000-0002-8781-3535; Afrin, Sadia/0000-0001-5063-9900; Xiao, Jianbo/0000-0003-3311-770X; Reboredo Rodriguez, Patricia/0000-0001-8440-6347; Zhang, Jiaojiao/0000-0002-6084-5502; Giampieri, Francesca/0000-0002-8151-9132 Alfonso Martin Escudero Foundation Alfonso Martin Escudero Foundation Authors are indebted with Ms. M. Glebocki for extensive editing of the manuscript. Patricia Reboredo-Rodriguez acknowledges Xunta de Galicia for her post-doctoral contract. Alfonso Varela-Lopez is supported by a fellowship from Alfonso Martin Escudero Foundation. 200 132 133 1
163 TAYLOR & FRANCIS INC PHILADELPHIA 530 WALNUT STREET, STE 850, PHILADELPHIA, PA 19106 USA 1040-8398 1549-7852
CRIT REV FOOD SCI Crit. Rev. Food Sci. Nutr. MAR 26 2019 59 6 SI 893 920
10.1080/10408398.2018.1526165
http://dx.doi.org/10.1080/10408398.2018.1526165
28 Food Science & Technology; Nutrition & Dietetics
Science Citation Index Expanded (SCI-EXPANDED); Conference Proceedings Citation Index - Science (CPCI-S) Food Science & Technology; Nutrition & Dietetics HX9OX 30421983 Green Published
2025-06-24 WOS:000467738800006
J Trivizakis, E; Ioannidis, GS; Souglakos, I; Karantanas, AH; Tzardi, M; Marias, K Trivizakis, Eleftherios; Ioannidis, Georgios S.; Souglakos, Ioannis; Karantanas, Apostolos H.; Tzardi, Maria; Marias, Kostas A neural pathomics framework for classifying colorectal cancer histopathology images based on wavelet multi-scale texture analysis SCIENTIFIC REPORTS English Article
RADIOMICS; FEATURES; SCALE Colorectal cancer (CRC) constitutes the third most commonly diagnosed cancer in males and the second in females. Precise histopathological classification of CRC tissue pathology is the cornerstone not only for diagnosis but also for patients' management decision making. An automated system able to accurately classify different CRC tissue regions may increase diagnostic precision and alleviate clinical workload. However, tissue classification is a challenging task due to the variability in morphological and textural characteristics present in histopathology images. In this study, an artificial neural network was trained to classify between eight classes of CRC tissue image patches derived from a public dataset with 5000 CRC histopathology image tiles. A total of 532 multi-level pathomics features examined at different scales were extracted by visual descriptors such as local binary patterns, wavelet transforms and Gabor filters. An exhaustive evaluation involving a variety of wavelet families and parameters was performed in order to shed light on the impact of scale on pathomics based CRC tissue differentiation. Our model achieved a performance accuracy of 95.3% with tenfold cross validation demonstrating superior performance compared to 87.4% reported in recent studies. Furthermore, we experimentally showed that the

first and the second levels of the wavelet approximations can be used without compromising classification performance.

[Trivizakis, Eleftherios; Tzardi, Maria] Univ Crete, Med Sch, Iraklion 71003, Greece; [Trivizakis, Eleftherios; Ioannidis, Georgios S.; Karantanas, Apostolos H.; Marias, Kostas] Fdn Res & Technol Hellas FORTH, Computat Biomed Lab CBML, Iraklion 70013, Greece; [Souglakos, Ioannis] Univ Crete, Med Sch, Lab Translat Oncol, Iraklion 71003, Greece; [Souglakos, Ioannis] Univ Hosp Heraklion, Dept Med Oncol, Iraklion 71500, Greece; [Karantanas, Apostolos H.] Univ Crete, Med Sch, Dept Radiol, Iraklion 71003, Greece; [Marias, Kostas] Hellen Mediterranean Univ, Elect & Comp Engn, Iraklion 71410, GreeceUniversity of Crete; University of Crete; University Hospital of Heraklion; University of Crete; Hellenic Mediterranean University Trivizakis, E (corresponding author), Univ Crete, Med Sch, Iraklion 71003, Greece.; Trivizakis, E (corresponding author), Fdn Res & Technol Hellas FORTH, Computat Biomed Lab CBML, Iraklion 70013, Greece.

trivizakis@ics.forth.gr Marias, Kostas/AAM-2330-2021; Trivizakis, Eleftherios/KHZ-5260-2024 Ioannidis, Georgios S./0000-0002-8139-5790; Trivizakis, Eleftherios/0000-0003-3988-6809; Marias, Kostas/0000-0003-3783-5223 Stavros Niarchos Foundation within project ARCHERS ('Advancing Young Researchers' Human Capital in Cutting Edge Technologies in the Preservation of Cultural Heritage and the Tackling of Societal Challenges')

Stavros Niarchos Foundation within project ARCHERS ('Advancing Young Researchers' Human Capital in Cutting Edge Technologies in the Preservation of Cultural Heritage and the Tackling of Societal Challenges') This study was financially supported by the Stavros Niarchos Foundation within the framework of the project ARCHERS (`Advancing Young Researchers' Human Capital in Cutting Edge Technologies in the Preservation of Cultural Heritage and the Tackling of Societal Challenges').

27 28 29 1 10 NATURE PORTFOLIO BERLIN HEIDELBERGER PLATZ 3, BERLIN, 14197, GERMANY 2045-2322 SCI REP-UK Sci Rep JUL 30 2021 11 1 15546 10.1038/s41598-021-94781-6 http://dx.doi.org/10.1038/s41598-021-94781-6 10

Multidisciplinary Sciences Science Citation Index Expanded (SCI-EXPANDED) Science & Technology - Other Topics TX8GF

34330946 Green Published, gold 2025-06-24

WOS:000683324400003

J Santella, ME; Hagedorn, RL; Wattick, RA; Barr, ML; Horacek, TM; Olfert, MD Santella, Madison E.; Hagedorn, Rebecca L.; Wattick, Rachel A.; Barr, Makenzie L.; Horacek, Tanya M.; Olfert, Melissa D. Learn first, practice second approach to increase health professionals' nutrition-related knowledge, attitudes and self-efficacy INTERNATIONAL JOURNAL OF FOOD SCIENCES AND NUTRITION English Article

Mediterranean Diet; nutrition education; self-efficacy; attitude; experiential learning

EDUCATION INTERVENTION; MEDITERRANEAN DIET; LOW-INCOME; STUDENTS; QUALITY; MODEL Health professionals generally have positive attitudes towards the role of nutrition in medicine, but limited knowledge and low self-efficacy for incorporating it into routine care. To assess the effectiveness of a "learn first, practice second" intervention on the nutrition-related knowledge, attitudes, and self-efficacy of multidisciplinary health professionals, the present approach consisted of 16weeks of online

education and 2weeks of cultural immersion in Tuscany, Italy. Data was captured via online surveys at Baseline, Post-education, Post-immersion, and Follow Up. Repeated measures ANOVA with irregular spacing was used, followed by Dunnett's or Cochran-Mantel-Haenszel testing. Results indicate significantly improved participant nutrition knowledge (nonzero correlation p=.0136, means score p=.0075) and self-efficacy (T0-T1 p<.0001, T0-T2 p<.0001, T0-T3 p=.0002), with differences in attitude trending towards significance (p=.0764). Findings from this study suggest that a combination of online education and hands on learning experiences can be beneficial for increasing health professionals' nutrition knowledge, confidence, and potentially attitude. [Santella, Madison E.; Hagedorn, Rebecca L.; Wattick, Rachel A.; Barr, Makenzie L.; Olfert, Melissa D.] West Virginia Univ, Davis Coll Agr Nat Resources & Design, Div Anim & Nutr Sci, G25 Agr Sci Bldg,333 Evansdale Dr, Morgantown, WV 26506 USA; [Horacek, Tanya M.] Syracuse Univ, Dept Publ Hlth Food Studies & Nutr, David B Falk Coll Sport & Human Dynam, Syracuse, NY USA West Virginia University; Syracuse University Olfert, MD (corresponding author), West Virginia Univ, Davis Coll Agr Nat Resources & Design, Div Anim & Nutr Sci, G25 Agr Sci Bldg,333 Evansdale Dr, Morgantown, WV 26506 USA. Melissa.offert@mail.wvu.edu Hagedorn, Rebecca/P-6995-2019; Barr, Makenzie/P-9902-2019; Olfert, Melissa/NFS-7300-2025; Horacek, Tanya/D-6355-2013 Wattick, Rachel/0000-0002-0837-028X; Olfert, Melissa D/0000-0002-6686-3891; Santella, Madison/0000-0001-9736-6516; Barr-Porter, Makenzie/0000-0002-6332-215X; Hagedorn-Hatfield, Rebecca/0000-0002-5046-4757
National Institute of General Medical Sciences T32 grant [GM081741]; West Virginia Agricultural and Forestry Experiment Station [WVA00689, WVA00721]; West Virginia University Office of Global Affairs; WVU General International Grant National Institute of General Medical Sciences T32 grant; West Virginia Agricultural and Forestry Experiment Station; West Virginia University Office of Global Affairs; WVU General International Grant Partial funding for materials used for abroad data collection through WVU General International Grant. RLH was supported by National Institute of General Medical Sciences T32 grant (GM081741). Additional support by the West Virginia Agricultural and Forestry Experiment Station (WVA00689 and WVA00721) and West Virginia University Office of Global Affairs.
31 16 18 2 17 TAYLOR & FRANCIS LTD
ABINGDON 2-4 PARK SQUARE, MILTON PARK, ABINGDON OR14 4RN, OXON, ENGLAND 0963-7486 1465-3478 INT J FOOD SCI NUTR
Int. J. Food Sci. Nutr. APR 2 2020 71 3
370 377 10.1080/09637486.2019.1661977
http://dx.doi.org/10.1080/09637486.2019.1661977 NOV 2019 8 Food Science & Technology; Nutrition & Dietetics
Science Citation Index Expanded (SCI-EXPANDED) Food Science & Technology; Nutrition & Dietetics LE8WO 31724444
Green Accepted 2025-06-24 WOS:000496370200001
J Elorabi, K; Ishak, S; Alhasnawi, MY; Dapo, OS; Maher, M
Elorabi, Khaled; Ishak, Suryati; Alhasnawi, Mushtaq Yousif; Somod Dapo, Olohunlana; Maher, Mohamed
Relationship between international remittances, political stability and income inequality: evidence from MENA countries
JOURNAL OF ECONOMIC AND ADMINISTRATIVE SCIENCES
English Article; Early Access
Income inequality; Remittances; MENA; Political stability;

Pooled mean group; Granger non-causality; D31; O53; P16; C33; F24
ECONOMIC-GROWTH; TRADE LIBERALIZATION; MIGRATION; POVERTY; CHALLENGES; LINKAGES; IMPACT; PANEL PurposeDespite the significant role of remittances in the Middle East and North Africa (MENA) region, their impact on income inequality has received limited attention in related research, especially in times of political instability. Therefore, this paper explores the moderating effect of political stability on the strengthening of the relationship between remittance inflows and income inequality in MENA countries over the period 1996-2022.Design/methodology/approachThis paper utilizes the pooled mean group (PMG) approach. For robustness, Dumitrescu and Hurlin's (2012) panel Granger non-causality test has been conducted, revealing the absence of reverse causality.FindingsThis paper demonstrates that remittance inflows increase income inequality in MENA recipient countries, indicating that most individuals receiving remittances in the MENA region are predominantly from well-off families. In addition, political stability reduces income inequality in MENA recipient countries, while remittance inflows increase income inequality when the polity becomes less stable.Practical implicationsWe recommend addressing obstacles faced by low-income groups seeking migration, especially in countries with high unemployment rates, such as deficiencies in education, skills, information asymmetry and financial constraints. Additionally, supplementary policies, such as directing resources toward pro-poor projects, particularly those that improve education, health and infrastructure, should be implemented to mitigate the adverse effects of remittances on income distribution. Finally, priority must be given to policies that promote political stability to create a business environment conducive to human and physical investments.Originality/valueThe paper addresses the impact of remittances on income inequality in the MENA region, which has received limited attention in previous research. [Elorabi, Khaled; Maher, Mohamed] Mansoura Univ, Dept Econ, Mansoura, Egypt; [Ishak, Suryati; Somod Dapo, Olohunlana] Univ Putra Malaysia, Dept Econ, Serdang, Malaysia; [Alhasnawi, Mushtaq Yousif] Univ Thi Qar, Dept Accounting, Nassiriya, Iraq
Egyptian Knowledge Bank (EKB); Mansoura University; Universiti Putra Malaysia; University of Thi-Qar Alhasnawi, MY (corresponding author), Univ Thi Qar, Dept Accounting, Nassiriya, Iraq. kh_elorabi@mans.edu.eg; suryatiis@upm.edu.my; Alhasnawi78@utq.edu.iq; osomd@yahoo.com; mohamed_maher16961737@mans.edu.eg Maher, Mohamed/V-7760-2018; Alhasnawi, Mushtaq/KYP-5591-2024 Yousif Alhasnawi, Mushtaq/0009-0009-2346-9681; Maher, Mohamed/0000-0002-9825-5276
73 0 0 0 0 EMERALD GROUP PUBLISHING LTD Leeds Floor 5, Northspring 21-23 Wellington Street, Leeds, W YORKSHIRE, ENGLAND 1026-4116 2054-6246 J ECONOM ADM SCI J. Econom. Adm. Sci. 2025 APR 23 2025
10.1108/JEAS-10-2024-0448
http://dx.doi.org/10.1108/JEAS-10-2024-0448 APR 2025
21 Economics Emerging Sources Citation Index (ESCI)
Business & Economics 1PY1I 2025-06-24 WOS:001470869800001
J Shokrzadeh, M; Modanloo, M; Samadi, S; Kunter, I; Haghighi, H Shokrzadeh, Mohammad; Modanloo, Mona; Samadi, Sara; Kunter, Imge; Haghighi, Hosna The Effects of Valproic Acid on Cell Viability of Breast Cancer and

Genotoxicity of Human Lymphocytes Blood Cell Line JOURNAL OF RESEARCH IN MEDICAL AND DENTAL SCIENCE English Article Valproic acid; Mcf-7; Genotoxicity; MTT assay; Micronucleus assay Introduction: Breast cancer is a complicated and multifactor disease in which so many genetic and environment agents play important roles. Nowadays different ways are available for cancer therapy such as surgery, chemotherapy and radiotherapy that are very costly and have various side effects. Valproic acid is an inhibitor of histone deacetylase enzyme that is effective on apoptosis of some cancerous cells. Aim: The aim of this research is to evaluate the toxic effects of this drug on both normal and cancerous cells, alongside with causing genetic disorder on peripheral blood lymphocytes as a model to evaluate genetic disorder. Material and methods: Cells were incubated with different concentrations of valproic acid and cisplatin. This incubation lead to cytotoxicity and Geno toxicity and then the viability of cells were measured. Cellular viability as well as Geno toxicity was assessed by MTT assay and micronucleus assay. Results: Results shows that valproic acid has an ability to significantly reduce the viability of breast cancer cells. Also toxic doses of valproic acid can affect BEAS-2B cells and can detract the viability of cells. Conclusion: This research proves the efficacy of valproic acid on cellular activity of Mcf-7 cell line and BEAS-2B cell line. This effect is probably associated with the ability of valproic acid to inhibit histone deacetylase enzyme and stop cells from growing and specialization and cause death in tumor cells, and the advantage of these drug class is that they have minimum effect on normal cells so they kill them rarely. [Shokrzadeh, Mohammad; Modanloo, Mona] Mazandaran Univ Med Sci, Dept Toxicol, Sari, Iran; [Samadi, Sara; Kunter, Imge; Haghighi, Hosna] Eastern Mediterranean Univ, Dept Pharm, Famagusta, Turkey Mazandaran University of Medical Sciences; Eastern Mediterranean University Shokrzadeh, M (corresponding author), Mazandaran Univ Med Sci, Dept Toxicol, Sari, Iran.mslamuki@gmail.com
5 0 0 0 1 AMBER PUBLICATION GUJARAT
402, PARIJAT APT, WALKESHWARI NAGAR, JAMNAGAR, GUJARAT, 361 008, INDIA 2347-2545 2347-2367 J RES MED DENT SCI J. Res. Med. Dent. Sci. 2021 9 12
341 345 5 Medicine, Research & Experimental Emerging Sources Citation Index (ESCI)
Research & Experimental Medicine YD8SX
2025-06-24 WOS:000740706400020
J Kühn, B; Brat, C; Fettel, J; Hellmuth, N; Maucher, IV; Bulut, U; Hock, KJ; Grimmer, J; Manolikakes, G; Rühl, M; Kühn, A; Zacharowski, K; Matrone, C; Urbschat, A; Roos, J; Steinhilber, D; Maier, TJ Kuehn, Benjamin; Brat, Camilla; Fettel, Jasmin; Hellmuth, Nadine; Maucher, Isabelle V.; Bulut, Ufuk; Hock, Katharina J.; Grimmer, Jennifer; Manolikakes, Georg; Ruehl, Michael; Kuehn, Alessa; Zacharowski, Kai; Matrone, Carmela; Urbschat, Anja; Roos, Jessica; Steinhilber, Dieter; Maier, Thorsten J. Anti-inflammatory nitro-fatty acids suppress tumor growth by triggering mitochondrial dysfunction and activation of the intrinsic apoptotic pathway in colorectal cancer cells BIOCHEMICAL PHARMACOLOGY English Article Nitroalkene; Nitrooleate; Apoptosis; Cancer; ROS; Michael acceptor BOVINE SERUM-ALBUMIN; NITROLINOLEIC ACID; MEDITERRANEAN DIET; OXIDATIVE STRESS; COLON-

CANCER; ANTITUMOR-ACTIVITY; MURINE MODEL; DNA-DAMAGE; PPAR-GAMMA; COMPLEX II Nitro-fatty acids (NFAs) are endogenously occurring lipid mediators exerting strong anti-inflammatory effects and acting as anti-oxidants in a number of animal models of inflammation. These NFA effects are mediated by targeting important regulatory proteins involved in inflammatory processes, such as 5-lipoxygenase, soluble epoxide hydrolase, or NF-kappa B. In the present study, we investigated the anti-tumorigenic effects of NFAs on colorectal cancer (CRC) cells in cell culture-based experiments and in a murine xenograft model of human CRC. We could show that 9-NOA suppresses the viability of CRC cells (HCT-116 and HT-29) by inducing a caspase-dependent apoptosis via the intrinsic apoptotic pathway. Co-treatment with the pan-caspase inhibitor Q-VD-OPH counteracted the NFA-mediated apoptosis in both cell lines. Furthermore, NFAs affected the cell cycle transition and reduced the oxygen consumption rate (OCR) immediately. On the contrary to their well-known anti-oxidative properties, NFAs mediated the generation of mitochondrial oxidative stress in human CRC cells. Additionally, similar to the cytostatic drug mitomycin, 9-NOA significantly reduced tumor growth in a murine xenograft model of human colorectal cancer. In contrast to the established cytostatic drug, 9-NOA treatment was well tolerated by mice. This study delivers a novel mechanistic approach for nitro-fatty acid-induced inhibition of CRC cell growth by targeting mitochondrial functions such as the mitochondria] membrane potential and mitochondria] respiration. We suggest these naturally occurring lipid mediators as a new class of well tolerated chemotherapeutic drug candidates for treatment of CRC or potentially other inflammation-driven cancer types. [Kuehn, Benjamin; Fettel, Jasmin; Maucher, Isabelle V.; Ruehl, Michael; Steinhilber, Dieter] Goethe Univ, Inst Pharmaceut Chem, Max von Laue Str 9, D-60438 Frankfurt, Germany; [Brat, Camilla; Hellmuth, Nadine; Bulut, Ufuk; Zacharowski, Kai; Roos, Jessica; Maier, Thorsten J.] Goethe Univ, Dept Anesthesiol Intens Care Med & Pain Therapy, Univ Hosp Frankfurt, Theodor Stern Kai 7, D-60590 Frankfurt, Germany; [Hock, Katharina J.; Grimmer, Jennifer; Manolikakes, Georg] Goethe Univ, Inst Organ Chem & Chem Biol, Max von Laue Str 7, D-60438 Frankfurt, Germany; [Hock, Katharina J.] Rhein Westfal TH Aachen, Inst Organ Chem, Landoltweg 1, D-52074 Aachen, Germany; [Grimmer, Jennifer; Manolikakes, Georg] TU Kaiserslautern, Dept Chem, Erwin Schrodinger Str 54, D-67663 Kaiserslautern, Germany; [Kuehn, Alessa] Goethe Univ, Inst Pharmaceut Biol, Max von Laue Str 9, D-60438 Frankfurt, Germany; [Matrone, Carmela; Urbschat, Anja; Maier, Thorsten J.] Aarhus Univ, Dept Biomed, Bartholins Alle 6, DK-8000 Aarhus C, Denmark Goethe University Frankfurt; Goethe University Frankfurt; Goethe University Frankfurt Hospital; Goethe University Frankfurt; RWTH Aachen University; University of Kaiserslautern; Goethe University Frankfurt; Aarhus University

Roos, J (corresponding author), Goethe Univ, Dept Anesthesiol Intens Care Med & Pain Therapy, Univ Hosp Frankfurt, Theodor Stern Kai 7, D-60590 Frankfurt, Germany.

Jessica.Roos@kgu.de Matrone, Carmela/AAC-2966-2019; Thomsen, Martin/AAU-5014-2021; Steinhilber, Dieter/J-3221-2012; Manolikakes, Georg/J-6408-2016 Steinhilber, Dieter/0000-0001-8905-5208; Manolikakes, Georg/0000-0002-4013-5757; Zacharowski, Kai/0000-0002-0212-9110; Matrone, Carmela/0000-0002-6719-0107 Else Kroner-Fresenius-Foundation (EKFS); Graduate School TRIP (Translational Research Innovation-Pharma); German Research

Foundation (DFG) [MA-5825/1-1]; DFG [Sonderforschungsbereich SFB-1039]; Aarhus University Research Foundation (AUFF); German Research Foundation [DFG-MA-5825/2-1] Else Kroner-Fresenius-Foundation (EKFS); Graduate School TRIP (Translational Research Innovation-Pharma); German Research Foundation (DFG)(German Research Foundation (DFG)); DFG(German Research Foundation (DFG)); Aarhus University Research Foundation (AUFF); German Research Foundation(German Research Foundation (DFG)) This work was funded by the Else Kroner-Fresenius-Foundation (EKFS) as well as the Graduate School TRIP (Translational Research Innovation-Pharma), the German Research Foundation (DFG project MA-5825/1-1), the DFG Sonderforschungsbereich SFB-1039 and the Aarhus University Research Foundation (AUFF). Thorsten Jurgen Maier was recipient of a Heisenberg fellowship from the German Research Foundation (DFG-MA-5825/2-1). 66 19 20 1 18 PERGAMON-ELSEVIER SCIENCE LTD OXFORD THE BOULEVARD, LANGFORD LANE, KIDLINGTON, OXFORD OX5 1GB, ENGLAND 0006-2952 1873-2968
BIOCHEM PHARMACOL Biochem. Pharmacol. SEP 2018 155
48 60
10.1016/j.bcp.2018.06.014
http://dx.doi.org/10.1016/j.bcp.2018.06.014 13
Pharmacology & Pharmacy Science Citation Index Expanded (SCI-EXPANDED) Pharmacology & Pharmacy GU5JZ 29909078
2025-06-24 WOS:000445323100005
J Elamin, MH; Elmahi, AB; Daghestani, MH; Al-Olayan, EM; Al-Ajmi, RA; Alkhuriji, AF; Hamed, SS; Elkhadragy, MF
Elamin, Maha H.; Elmahi, Abdelsalam B.; Daghestani, Maha H.; Al-Olayan, Ebtesam M.; Al-Ajmi, Reem A.; Alkhuriji, Afrah F.; Hamed, Sherifa S.; Elkhadragy, Manal F. Synergistic Anti-Breast-Cancer Effects of Combined Treatment With Oleuropein and Doxorubicin In Vivo ALTERNATIVE THERAPIES IN HEALTH AND MEDICINE English Article
NF-KAPPA-B; MEDITERRANEAN DIET; CYCLIN D1; OLIVE OIL; PATHWAY; ACTIVATION; APOPTOSIS; HYDROXYTYROSOL; CYTOSKELETON; ANTIOXIDANTContext. Breast cancer is a leading cause of cancer fatalities among women worldwide. Of the more than 80% of patients who receive adjuvant chemotherapy, approximately 40% relapse. The majority of these patients die of disseminated metastatic disease, which emphasizes the need for new therapeutic strategies Objective. The study intended to investigate the anticancer effects of oleuropcin (OL) and doxorubicin (DOX) individually and in combination on breast tumor xenografts and also to evaluate the molecular pathways involved. Design. The research team designed in vivo (animal) and in vitro (cell culture) studies. Setting: The study was performed in the College of Science of King Saud University in the University Center for Women Students (Riyadh, Saudi Arabia). Animals. The study involved 40 female, nude mice (BALB/c OlaHsd-foxn1). Intervention. The mice were injected subcutaneously with MDA- MB- 231 human breast cancer cells. After the growth of tumors, the animals were randomly divided into 4 groups to receive intraperitoneal injections: (1) group 1 (control group)-dimethyl sulfoxide, (2) group 2 (intervention group)-50 mg/kg of OL, (3) group 3 (intervention group)-2.5 mg/kg of DOX, and (4) group 4 (intervention group)-1.5 mg/kg of DOX, immediately followed by 50 mg/kg of OL. The OL was extracted from Manzanillo olive trees (Olea europaea) grown in Tabouk, Saudi Arabia. Outcome Measures. The measures included the isolation and primary culture of the tumor xenografts, apoptosis analysis by annexin V, cellular

lysate preparation, and immunoblotting. Results: The volume of the tumor increased aggressively, reaching 173 mm 3 in the control animals in a time-dependent manner. On the other hand, a sharp drop, to 48.7 mm 3 , in the volume of the tumor was observed with the 2 drugs combined, a more than 3-fold decrease. The effect was mediated through the induction of apoptosis via the mitochondria] pathway. The combined treatment downregulated the antiapoptosis and proproliferation protein, nuclear factor-kappa B, and its main oncogcnic target cyclin DI. Furthermore, it inhibited the expression of BCL-2 and survivin. This inhibition could explain the cooperative suppression of the proliferation of breast tumor xenografts and the induction of apoptosis by the combined effect of the compounds used. Conclusions. The key findings clearly indicate the synergistic efficacy of DOX with natural and nontoxic OL against breast tumor xenografts. [Elamin, Maha H.; Daghestani, Maha H.; Al-Olayan, Ebtesam M.; Al-Ajmi, Reem A.; Alkhuriji, Afrah F.; Hamed, Sherifa S.; Elkhadragy, Manal F.] King Saud Univ, Univ Ctr Women Students, Coll Sci, Zool Dept, In Riyadh, Saudi Arabia; [Elmahi, Abdelsalam B.] Univ Khartoum, Fac Vet Med, Pathol Dept, Khartoum, Sudan; [Al-Olayan, Ebtesam M.] King Saud Univ, Fac Sci, Zool Dept, Chair Vaccines Infect Dis, Riyadh, Saudi Arabia; [Hamed, Sherifa S.] Alexandria Univ, Fac Sci, Zool Dept, Alexandria, Egypt; [Elkhadragy, Manal F.] Helwan Univ, Fac Sci, Zool & Entomol Dept, Cairo, Egypt King Saud University; University of Khartoum; King Saud University; Egyptian Knowledge Bank (EKB); Alexandria University; Egyptian Knowledge Bank (EKB); Helwan University Elamin, MH (corresponding author), King Saud Univ, Univ Ctr Women Students, Coll Sci, Zool Dept, In Riyadh, Saudi Arabia. mahaalamin@yahoo.com Alolayan, Ebtesam/LLL-7698-2024 National Science, Technology, and Innovation Plan's strategic technologies program in the Kingdom of Saudi Arabia [10-ENV993-02] National Science, Technology, and Innovation Plan's strategic technologies program in the Kingdom of Saudi Arabia This project was supported by National Science, Technology, and Innovation Plan's strategic technologies program (No. 10-ENV993-02) in the Kingdom of Saudi Arabia. 52 31 31 0 8 INNOVISION COMMUNICATIONS ALISO VIEJO 101 COLUMBIA, ALISO VIEJO, CA 92656 USA 1078-6791 ALTERN THER HEALTH M Altern. Ther. Health Med. MAY-JUN 2019 25 3 17 24 8 Integrative & Complementary Medicine Science Citation Index Expanded (SCI-EXPANDED) Integrative & Complementary Medicine KG7VU 28646810 2025-06-24 WOS:000510157400002

J Menendez, JA; Papadimitropoulou, A; Vellon, L; Lupu, R Menendez, Javier A.; Papadimitropoulou, Adriana; Vellon, Luciano; Lupu, Ruth A genomic explanation connecting "Mediterranean diet", olive oil and cancer:: Oleic acid, the main monounsaturated fatty acid of olive oil, induces formation of inhibitory "PEA3 transcription factor-PEA3 DNA binding site" complexes at the Her-2/neu (erbB-2) oncogene promoter in breast, ovarian and stomach cancer cells EUROPEAN JOURNAL OF CANCER English Article her-2/neu; erbB-2; PEA3; oleic acid; olive oil; Mediterranean diet; cancer GROWTH-FACTOR RECEPTOR; METASTATIC BREAST; MONOCLONAL-ANTIBODY; MAMMARY-CARCINOMA; NEU ONCOGENE; OVEREXPRESSION; RISK; TRASTUZUMAB; THERAPY; EXPRESSION
Olive oil is an integral ingredient of the "Mediterranean

diet" and accumulating evidence suggests that it may have a potential role in lowering risk of several cancers. We recently hypothesized that the anti-cancer actions of olive oil may relate to its monounsaturated fatty acid (MUFA) oleic acid (OA; 18:1n - 9) content to specifically regulate oncogenes. In this study, transient transfection experiments with human Her-2/neu promoter-driven luciferase gene established the ability of OA to specifically repress the transcriptional activity of Her-2/neu gene. Gene repression was seen in tumour-derived cell lines with Her-2/neu gene ;amplification and overexpression, including SK-Br3 (<= 56% reduction), SK-OV3 (<= 75% reduction) and NCI-N87 (55 reduction) breast, ovarian and stomach cancer cell lines, respectively. Also marginal decreases in promoter activity were observed in cancer cells expressing physiological levels of Her-2/neu (<= 20% reduction in MCF-7 breast cancer cells). Remarkably, OA treatment in Her-2/neu-overexpressing cancer cells was found to induce up-regulation of the Ets protein polyomavirus enhancer activator 3 (PEAS), a transcriptional repressor of Her-2/neu promoter. Also, an intact PEAS DNA-binding-site at endogenous Her-2/neu gene promoter was essential for OA-induced repression of this gene. Moreover, OA treatment failed to decrease Her-2/neu protein levels in MCF-7/Her2-18 transfectants, which stably express full-length human Her-2/neu cDNA controlled by a SV40 viral promoter. OA-induced transcriptional repression of Her-2/neu through the action of PEAS protein at the promoter level may represent a novel mechanism linking "Mediterranean diet" and cancer. (c) 2005 Elsevier Ltd. All rights reserved. Evanston Northwestern Healthcare Res Inst, Dept Med, Evanston, IL 60201 USA; Northwestern Univ Feinberg, Sch Med, Dept Med, Chicago, IL USA; Northwestern Univ, Robert H Lurie Comprehens Canc Ctr, Chicago, IL USA Northwestern University; Feinberg School of Medicine; Robert H. Lurie Comprehensive Cancer Center; Northwestern University; Ann & Robert H. Lurie Children's Hospital of Chicago Lupu, R (corresponding author), Evanston Northwestern Healthcare Res Inst, Dept Med, 1001 Univ Pl, Evanston, IL 60201 USA. r-lupu@northwestern.edu MENENDEZ MENENDEZ, JAVIER ABEL/C-6148-2016 MENENDEZ MENENDEZ, JAVIER ABEL/0000-0001-8733-4561 NCI NIH HHS [P50CA89018-03] Funding Source: Medline NCI NIH HHS(United States Department of Health & Human ServicesNational Institutes of Health (NIH) - USANIH National Cancer Institute (NCI)) 48 92 103 0 20
ELSEVIER SCI LTD OXFORD THE BOULEVARD, LANGFORD LANE, KIDLINGTON, OXFORD OX5 1GB, OXON, ENGLAND 0959-8049 1879-0852
EUR J CANCER Eur. J. Cancer OCT 2006 42 15
2425 2432
10.1016/j.ejca.2005.10.016
http://dx.doi.org/10.1016/j.ejca.2005.10.016 8
Oncology Science Citation Index Expanded (SCI-EXPANDED)
Oncology 103ZS 16406575 2025-06-24
WOS:000241926300012
J Nejatollahi, F; Asgharpour, M; Jaberipour, M
Nejatollahi, Foroogh; Asgharpour, Mahdi; Jaberipour, Mansooreh Down-regulation of vascular endothelial growth factor expression by anti-her2/neu single chain antibodies
MEDICAL ONCOLOGY English Article
Single-chain antibody; HER2/neu; VEGF; Angiogenesis; Breast cancer BREAST-CANCER; 1ST-LINE TREATMENT; MONOCLONAL-ANTIBODIES; TUMOR ANGIOGENESIS; FACTOR VEGF; IN-VITRO;

THERAPY; CELLS; HER2; TRASTUZUMAB HER-2/neu is overexpressed in 25-30% of breast tumors. Signaling through HER-2/neu leads to an increase in the production of vascular endothelial growth factor (VEGF) and enhances angiogenesis. We evaluated the effects of three specific anti-HER2/neu single chain-Fv (scFv) antibodies on the expression level of VEGF in HER2/neu-expressing breast cancer cell lines. A nonimmunized human scFv library was panned against three epitopes of HER2/neu. BT-474 human breast cancer cell line was treated with three specific anti-HER2/neu scFv antibodies and the amount of VEGF gene transcript was determined by quantitative real-time PCR. The expression of VEGF protein was analyzed by western blot. All three scFv antibodies along with their combination inhibited VEGF expression at both the gene and protein levels. Our results show that anti-HER2/neu recombinant antibodies can be considered as anti-angiogenic agents in HER2/neu-positive breast cancers. [Jaberipour, Mansooreh] Shiraz Univ Med Sci, Gene Therapy Lab, Inst Canc Res, Shiraz, Iran; [Nejatollahi, Foroogh; Asgharpour, Mahdi] Shiraz Univ Med Sci, Recombinant Antibody Lab, Dept Immunol, Grad Sch Adv Biomed Sci, Shiraz, Iran; [Nejatollahi, Foroogh] Shiraz Univ Med Sci, AIDS Res Ctr, Shiraz, IranShiraz University of Medical Science; Shiraz University of Medical Science; Shiraz University of Medical Science Jaberipour, M (corresponding author), Shiraz Univ Med Sci, Gene Therapy Lab, Inst Canc Res, POB 71345-3119, Shiraz, Iran. jaberim@sums.ac.ir
Nejatollahi, Foroogh/V-6434-2017 Nejatollahi, Foroogh/0000-0002-8928-8519 Shiraz University of Medical Sciences Shiraz University of Medical Sciences(Shiraz University of Medical Science) The authors acknowledge Shiraz University of Medical Sciences for financial support and K. Shashok (Author AID in the Eastern Mediterranean) for improving the use of English in the manuscript. 43 12 12 0 5 HUMANA PRESS INC
TOTOWA 999 RIVERVIEW DRIVE SUITE 208, TOTOWA, NJ 07512
USA 1357-0560 1559-131X MED ONCOL Med. Oncol. MAR 2012
29 1 378 383
10.1007/s12032-010-9796-5
http://dx.doi.org/10.1007/s12032-010-9796-5 6
Oncology Science Citation Index Expanded (SCI-EXPANDED)
Oncology 892WV 21267676 2025-06-24
WOS:000300317200057
J Hamaï, A; Duperrier-Amouriaux, K; Pignon, P; Raimbaud, I; Memeo, L; Colarossi, C; Canzonieri, V; Perin, T; Classe, JM; Campone, M; Jézéquel, P; Campion, L; Ayyoub, M; Valmori, D
Hamai, Ahmed; Duperrier-Amouriaux, Karine; Pignon, Pascale; Raimbaud, Isabelle; Memeo, Lorenzo; Colarossi, Cristina; Canzonieri, Vincenzo; Perin, Tiziana; Classe, Jean-Marc; Campone, Mario; Jezequel, Pascal; Campion, Loic; Ayyoub, Maha; Valmori, Danila Antibody Responses to NY-ESO-1 in Primary Breast Cancer Identify a Subtype Target for Immunotherapy
PLOS ONE English Article
TESTIS ANTIGEN NY-ESO-1; IMMUNE-RESPONSES; EXPRESSION; MELANOMA; EPITOPES; CELLS The highly immunogenic human tumor antigen NY-ESO-1 (ESO) is a target of choice for anti-cancer immune therapy. In this study, we assessed spontaneous antibody (Ab) responses to ESO in a large cohort of patients with primary breast cancer (BC) and addressed the correlation between the presence of anti-ESO Ab, the expression of ESO in the tumors and their characteristics. We found detectable Ab responses to ESO in 1% of the patients. Tumors from patients with circulating Ab to

ESO exhibited common characteristics, being mainly hormone receptor (HR)(-) invasive ductal carcinomas of high grade, including both HER2(-) and HER2(+) tumors. In line with these results, we detected ESO expression in 20% of primary HR(-) BC, including both ESO Ab(+) and Ab(-) patients, but not in HR(+) BC. Interestingly, whereas expression levels in ESO(+) BC were not significantly different between ESO Ab(+) and Ab(-) patients, the former had, in average, significantly higher numbers of tumor-infiltrated lymph nodes, indicating that lymph node invasion may be required for the development of spontaneous anti-tumor immune responses. Thus, the presence of ESO Ab identifies a tumor subtype of HR(-) (HER2(-) or HER2(+)) primary BC with frequent ESO expression and, together with the assessment of antigen expression in the tumor, may be instrumental for the selection of patients for whom ESO-based immunotherapy may complement standard therapy.

[Hamai, Ahmed; Duperrier-Amouriaux, Karine; Pignon, Pascale; Raimbaud, Isabelle; Ayyoub, Maha; Valmori, Danila] CLCC Rene Gauducheau, INSERM, U892, St Herblain, France; [Memeo, Lorenzo; Colarossi, Cristina] Mediterranean Inst Oncol, Pathol Unit, Catania, Italy; [Canzonieri, Vincenzo; Perin, Tiziana] IRCCS, Ctr Riferimento Oncol, Aviano, Pordenone, Italy; [Classe, Jean-Marc] CLCC Rene Gauducheau, Dept Surg, St Herblain, France; [Campone, Mario] CLCC Rene Gauducheau, Dept Med Oncol, St Herblain, France; [Jezequel, Pascal] CLCC Rene Gauducheau, Dept Oncobiol, St Herblain, France; [Campion, Loic] CLCC Rene Gauducheau, Dept Biostat, St Herblain, France; [Campone, Mario] IRT UN, INSERM, U892, Nantes, France; [Valmori, Danila] Univ Nantes, Fac Med, Nantes, France UNICANCER; Institut de Cancerologie de l'Ouest (ICO); Institut National de la Sante et de la Recherche Medicale (Inserm); Mediterranean Institute of Oncology; IRCCS Aviano (CRO); UNICANCER; Institut de Cancerologie de l'Ouest (ICO); UNICANCER; Institut de Cancerologie de l'Ouest (ICO); UNICANCER; Institut de Cancerologie de l'Ouest (ICO); UNICANCER; Institut de Cancerologie de l'Ouest (ICO); Institut National de la Sante et de la Recherche Medicale (Inserm); Nantes Universite Hamaï, A (corresponding author), CLCC Rene Gauducheau, INSERM, U892, St Herblain, France.

Maha.Ayyoub@univ-nantes.fr; Danila.Valmori@univ-nantes.fr

Jézéquel, Pascal/K-8206-2015; Valmori, Danila/K-2439-2015; campone, mario/L-4880-2015; Canzonieri, Vincenzo/AAA-7951-2019; Ayyoub, Maha/A-2074-2017; Campion, Loic/K-4726-2015; Canzonieri, Vincenzo/K-3141-2018; Perin, Tiziana/AAC-2403-2020 Campion, Loic/0000-0003-4903-0908; Canzonieri, Vincenzo/0000-0001-6010-0976; Perin, Tiziana/0000-0002-9823-0167; Hamai, Ahmed/0000-0002-7921-4014; Memeo, Lorenzo/0000-0003-4251-7203; Ayyoub, Maha/0000-0003-2022-0898; Colarossi, Cristina/0000-0001-5395-8608 Cancer Research Institute; Ludwig Institute for Cancer Research; Institut National du Cancer; Canceropole Grand Ouest Cancer Research Institute; Ludwig Institute for Cancer Research; Institut National du Cancer(Institut National du Cancer (INCA) France); Canceropole Grand Ouest This study was supported by the Cancer Research Institute (http://www.cancerresearch.org) and the Ludwig Institute for Cancer Research (http://www.licr.org). The funders had no role in study design, data collection and analysis, decision to publish, or preparation of the manuscript. Sera and surgical tumor specimens from breast cancer patients were obtained from the tumor bank of the Institut Regional du Cancer Nantes-Atlantique, supported by the Institut National du Cancer and the Canceropole Grand Ouest. We are grateful to Drs. G. Ritter and L.J. Old for

providing the recombinant proteins and for helpful advice, and to Dr. Maryam Mehrpour for providing the BC lines. We would like to thank Mrs. Nicole Andrieux for assistance with clinical data analysis, and Mr. Sandro Barbuscia for IHC staining. 21
19 23 0 4 PUBLIC LIBRARY SCIENCE SAN FRANCISCO
185 BERRY ST, STE 1300, SAN FRANCISCO, CA 94107 USA
1932-6203 PLOS ONE PLoS One JUN 17 2011
6 6 e21129
10.1371/journal.pone.0021129
http://dx.doi.org/10.1371/journal.pone.0021129
7 Multidisciplinary Sciences Science Citation Index Expanded (SCI-EXPANDED) Science & Technology - Other Topics
778VR 21747904 Green Submitted, gold, Green Published
2025-06-24 WOS:000291737600045
J Vidua, RK; Dubey, N; Pramanik, P; Mattoo, SK; Jakher, N
Vidua, Raghvendra K.; Dubey, Nisha; Pramanik, Parthsarthi; Mattoo, Sanjay K.; Jakher, Naresh A Representative Study of Deaths of North Indian Migrants and Its Medical Certification Across the World JOURNAL OF IMMIGRANT AND MINORITY HEALTH English Article
International migrant; Manner of death; Mode of death; Ambiguous with unspecified etiology; Medical certification of death; 2nd autopsy Many Indians is moving to other nations of the world in the search of employment, education or other reasons. The process of globalization along with the faster mode of traveling and communication has facilitated this movement in the hope of getting more opportunities and earning of easy money abroad, than in India. Unfortunately, sometimes they meet a tragic end and their families in India get only their dead bodies back from abroad. This study focuses on these kind of unfortunate tragic events faced by Indians in different countries and thereby raise a concern on their safety abroad and necessitate the need of relooking in to the quality of medical certification of death and medico legal investigations to find out the real reasons of deaths to avoid any kind of doubt in mind. Data was collected from medical death certificates and passports of the deceased Indians. There was a total of 711 dead bodies/human remains received in 2012 at IGI airport New Delhi. The Middle East countries together contributed a total of 398 (55.98 %) cases. The distribution as per manner of death shows that in maximum number of cases (269), the manner of death was ambiguous with unspecified aetiology. The study therefore recommends for upgradation of the level of medico legal investigations in form of 2nd autopsy in recipient country and proper medical death certification. [Vidua, Raghvendra K.] AIIMS, Forens Med & Toxicol, Bhopal 462020, MP, India; [Pramanik, Parthsarthi] Govt Jama, Kingston, Jamaica; [Mattoo, Sanjay K.; Jakher, Naresh] Airport Hlth Org, New Delhi 110037, India All India Institute of Medical Sciences (AIIMS) Bhopal Vidua, RK (corresponding author), AIIMS, Forens Med & Toxicol, Bhopal 462020, MP, India. raghvendra.fmt@aiimsbhopal.edu.in; drnishadubey22@gmail.com; drbubay@rediffmail.com; sanjaykmattoo@gmail.com; nareshjakher2003@gmail.com Vidua, Dr Raghvendra Kumar/IUP-4438-2023; PRAMANIK, PARTHASARATHI/H-1731-2013 PRAMANIK, PARTHASARATHI/0000-0001-8820-9009
27 0 0 0 4 SPRINGER NEW YORK 233 SPRING ST, NEW YORK, NY 10013 USA 1557-1912 1557-1920 J IMMIGR MINOR HEALT J. Immigr. Minor. Health FEB 2018 20
1 73 82 10.1007/s10903-

016-0504-1 http://dx.doi.org/10.1007/s10903-016-0504-1
10 Public, Environmental & Occupational Health Social Science Citation Index (SSCI) Public, Environmental & Occupational Health FT0FE 27669719 2025-06-24 WOS:000422796300012
J Atmaca, H; Ilhan, S; Dundar, BA; Zora, M
Atmaca, Harika; Ilhan, Suleyman; Dundar, Buse Aysen; Zora, Metin Bioevaluation of Spiro N-Propargylic β-Enaminones as Anti-Breast Cancer Agents: In Vitro and Molecular Docking Studies CHEMISTRY & BIODIVERSITY English
Article spiro N-propargylic beta-enaminones; cytotoxicity; apoptosis; cell cycle; ADMET; docking FLUORINE; RECEPTOR The study aimed to investigate the in vitro inhibitory activities of spiro N-propargylic beta-enaminones, SPEs 1-31, against BCa cells, to perform in silico molecular docking studies to understand the nature of the interaction between the compounds and the ER alpha, PR, EGFR, and Her2, and to determine the ADMET and drug-likeness properties. Cytotoxic activity was investigated via MTT assay. DNA fragmentation was evaluated via ELISA assay. Cell cycle distributions were investigated by flow cytometry. Expression levels of Bcl-2, Bax, p21 and Cyclin D1 were measured by qRT-PCR and western blot analysis. Molecular docking was done using Autodock/vina software. ADMET analysis was calculated using the ADMETlab 2.0 tool. SPEs 1, 22, and 28 showed selective cytotoxic activity against all BCa cells with SI values >2. SPEs induced apoptosis and caused significant changes in Bcl-2 and Bax levels. The cell cycle was arrested at the S phase and levels of p21 and Cyclin D1 were induced in all BCa cells. Molecular docking analysis revealed that SPE1, SPE22, and SPE28 showed high binding affinities with ER alpha, PR, EGFR, and Her2. ADMET analysis revealed that SPEs are drug-like compounds as they obey the five rules of Lipinsky and are not toxic. Therefore, these potential anticancer compounds should be further validated by in vivo studies for their appropriate function in human health with a safety profile, and a comprehensive drug interaction study should be performed. [Atmaca, Harika; Ilhan, Suleyman] Manisa Celal Bayar Univ, Fac Sci & Letters, Dept Biol, TR-45140 Manisa, Turkiye; [Dundar, Buse Aysen; Zora, Metin] Middle East Tech Univ, Dept Chem, TR-06800 Ankara, Turkiye Celal Bayar University; Middle East Technical University Atmaca, H (corresponding author), Manisa Celal Bayar Univ, Fac Sci & Letters, Dept Biol, TR-45140 Manisa, Turkiye. harika.atmaca@cbu.edu.tr Dundar, Buse/AAW-1553-2021; Zora, Metin/ABB-7678-2020; ILHAN, Suleyman/AAZ-2000-2021; atmaca, harika/AAX-8164-2021 atmaca, harika/0000-0002-8459-4373 We thank the Scientific and Technological Research Council of Turkey [TUBITAK, Grant No. 114Z811] and the Research Fund of Middle East Technical University [METU, Grant No. GAP-103-2018-2770] for financial support of the synthesis of spiro NNN</ITALIC>-propargylic beta-enaminones (SPEs). 38 3 3 0 4 WILEY-V C H VERLAG GMBH WEINHEIM POSTFACH 101161, 69451 WEINHEIM, GERMANY
1612-1872 1612-1880 CHEM BIODIVERS Chem. Biodivers. NOV 2023 20 11
10.1002/cbdv.202301228
http://dx.doi.org/10.1002/cbdv.202301228 NOV 2023
16 Biochemistry & Molecular Biology; Chemistry, Multidisciplinary Science Citation Index Expanded (SCI-

EXPANDED) Biochemistry & Molecular Biology; Chemistry AH5O0
37837366 Bronze 2025-06-24
WOS:001101416100001
J Ali, A; Ali, A; Warsi, MH; Ahmad, W; Tahir, A
Ali, Abuzer; Ali, Amena; Warsi, Musarrat Husain; Ahmad, Wasim; Tahir, Abu Chemical characterization, antidiabetic and anticancer activities of Santolina chamaecyparissus SAUDI JOURNAL OF BIOLOGICAL SCIENCES
English Article Santolina chamaecyparissus; Diabetes; alpha-glucosidase; EGFR; Human breast cancer; GC-MS MEDICINAL-PLANTS; NATURAL-PRODUCTS; ESSENTIAL OIL; DRUGS Santolina chamaecyparissus is an important medicinal plant growing in the Mediterranean region and has been reported as a potent anti-inflammatory, antibacterial, antioxidant, and antifungal agent. The purpose of the current research is to identify the chemical constituents in ethyl acetate extract (EAE) from the leaves of S. chamaecyparissus, and to evaluate antidiabetic, and anticancer activity. Chemical constituents of EAE were identified by GC-MS, and the antidiabetic activity was evaluated by aglucosidase inhibition assay. The anticancer activity was assessed by Epidermal Growth Factor Receptor (EGFR) expression in human breast cancer cell line (MCF7) by using quantitative RT-PCR method. GC-MS analysis of EAE of S. chamaecyparissus yielded 44 compounds. Tetrapentacontane (27.15%), eicosyl acetate (8.40%), 2-methylhexacosane (6.87%), and n-pentadecanol (5.44%) were found as major chemical constituents. The EAE of S. chamaecyparissus showed concentration dependant inhibition of alpha-glucosidase enzyme and the IC50 value (IC50 110 +/- 4.25 mu g/mL) was found comparable with standard acarbose (IC50 105 +/- 3.74 mu g/mL). The real-time qRT-PCR results showed that the EGFR protein (bcl-2) in human breast cancer cell line (MCF7) was negatively expressed with a value of -0.69297105 after treatment with EAE (100 mu g/mL). The study results are suggesting the possible use of S. chamaecyparissus in the management of diabetes, and human breast cancer. (C) 2021 The Author(s). Published by Elsevier B.V. on behalf of King Saud University.
[Ali, Abuzer] Taif Univ, Dept Pharmacognosy, Coll Pharm, POB 11099, At Taif 21944, Saudi Arabia; [Ali, Amena] Taif Univ, Dept Pharmaceut Chem, Coll Pharm, POB 11099, At Taif 21944, Saudi Arabia; [Warsi, Musarrat Husain] Taif Univ, Dept Pharmaceut & Ind Pharm, Coll Pharm, POB 11099, At Taif 21944, Saudi Arabia; [Ahmad, Wasim] Mohammed Al Mana Coll Med Sci, Dept Pharm, Dammam 34222, Saudi Arabia; [Tahir, Abu] Raghukul Coll Pharm, Dept Pharmacol, Bhopal, India Taif University; Taif University; Taif University; Mohammed Al-Mana College for Medical Sciences Ali, A (corresponding author), Taif Univ, Dept Pharmacognosy, Coll Pharm, POB 11099, At Taif 21944, Saudi Arabia.
abuali@tu.edu.sa Warsi, Musarrat/AAN-6095-2021; AHMAD, WASIM/AAM-6289-2020; Ali, Abuzer/GRF-4916-2022 Taif University, Taif, Saudi Arabia [TURSP-2020/124] Taif University, Taif, Saudi Arabia(Taif University) Dr Abuzer Ali is thankful to Taif University Researchers Supporting Project Number (TURSP-2020/124), Taif University, Taif, Saudi Arabia. Authors are thankful to Trichy Research Institute of Biotechnology Pvt. Ltd., Trichy, Tamil Nadu, India for conducting anticancer cell line study. 34 27 29 0 6 ELSEVIER
AMSTERDAM RADARWEG 29, 1043 NX AMSTERDAM, NETHERLANDS
1319-562X 2213-7106 SAUDI J BIOL SCI Saudi J. Biol.

Sci. AUG 2021 28 8 4575 4580
10.1016/j.sjbs.2021.04.060
http://dx.doi.org/10.1016/j.sjbs.2021.04.060 JUL 2021
6 Biology Science Citation Index Expanded (SCI-EXPANDED) Life Sciences & Biomedicine - Other Topics TQ6FB
34354443 hybrid, Green Published 2025-06-24 WOS:000678372600028
J Abbade, Y; Kisla, MM; Hassan, MA; Celik, I; Dogan, TS; Mutlu, P; Ates-Alagoz, Z Abbade, Yemna; Kisla, Mehmet Murat; Hassan, Mohammed Al-Kassim; Celik, Ismail; Dogan, Tugba Somay; Mutlu, Pelin; Ates-Alagoz, Zeynep
Synthesis, Anticancer Activity, and In Silico Modeling of Alkylsulfonyl Benzimidazole Derivatives: Unveiling Potent Bcl-2 Inhibitors for Breast CancerACS OMEGA English
Article DRUG ABSORPTION; FAMILY PROTEINS; DESIGN; OPTIMIZATION; METABOLISM; EXPRESSION; PREDICTION; APOPTOSIS; DOCKING; CELLS A series of alkylsulfonyl 1H-benzo[d]imidazole derivatives were synthesized and evaluated for anticancer activity against human breast cancer cells, MCF-7 in vitro. The cytotoxic potential was determined using the xCELLigence real-time cell analysis, and expression levels of genes related to microtubule organization, tumor suppression, apoptosis, cell cycle, and proliferation were examined by quantitative real-time polymerase chain reaction. Molecular docking against Bcl-2 was carried out using AutoDock Vina, while ADME studies were performed to predict the physicochemical and drug-likeness properties of the synthesized compounds. The results revealed that compounds 23 and 27 were the most potent cytotoxic derivatives against MCF-7 cells. Gene expression analysis showed that BCL-2 was the most prominent gene studied. Treatment of MCF-7 cells with compounds 23 and 27 resulted in significant downregulation of the BCL-2 gene, with fold changes of 128 and 256, respectively. Docking analysis predicted a strong interaction between the compounds and the target protein. Interestingly, all of the compounds exhibit a higher binding affinity toward Bcl-2 than the standard drug (compound 27 vina score = -9.6 kcal/mol, vincristine = -6.7 kcal/mol). Molecular dynamics simulations of compounds 23 and 27 showed a permanent stabilization in the binding site of Bcl-2 for 200 ns. Based on Lipinski and Veber's filters, all synthesized compounds displayed drug-like characteristics. These findings suggest that compounds 23 and 27 were the most promising cytotoxic compounds and downregulated the expression of the BCL-2 gene. These derivatives could be further explored as potential candidates for the treatment of breast cancer. [Abbade, Yemna; Kisla, Mehmet Murat; Hassan, Mohammed Al-Kassim; Ates-Alagoz, Zeynep] Ankara Univ, Fac Pharm, Dept Pharmaceut Chem, TR-06100 Ankara, Turkiye; [Abbade, Yemna; Kisla, Mehmet Murat; Hassan, Mohammed Al-Kassim] Ankara Univ, Grad Sch Hlth Sci, TR-06110 Ankara, Turkiye; [Hassan, Mohammed Al-Kassim] Bayero Univ, Fac Pharmaceut Sci, Dept Pharmaceut & Med Chem, PMB 3011, Kano, Nigeria; [Celik, Ismail] Erciyes Univ, Fac Pharm, Dept Pharmaceut Chem, TR-38039 Kayseri, Turkiye; [Dogan, Tugba Somay] Middle East Tech Univ, Mol Biol & Biotechnol R&D Ctr, Cent Lab, TR-06800 Ankara, Turkiye; [Mutlu, Pelin] Ankara Univ, Biotechnol Inst, Dept Biotechnol, TR-06135 Ankara, Turkiye Ankara University; Ankara University; Bayero University; Erciyes University; Middle East Technical University; Ankara UniversityAtes-Alagoz, Z

(corresponding author), Ankara Univ, Fac Pharm, Dept Pharmaceut Chem, TR-06100 Ankara, Turkiye.   zates@pharmacy.ankara.edu.tr
Doğan, Tuğba/AAZ-6712-2021; Mohammed, Al-Kassim/ABC-2855-2020; KISLA, Mehmet/AAH-4202-2020; Ates-Alagoz, Zeynep/AAG-5278-2019; Mutlu, Pelin/AAZ-6710-2021; celik, ismail/ABE-5813-2021
KISLA, Mehmet Murat/0000-0001-6209-0361; celik, ismail/0000-0002-8146-1663; ATES ALAGOZ, ZEYNEP/0000-0002-5898-9988; Hassan Mohammed, Al-Kassim/0000-0002-5423-4633Central Laboratory of the Faculty of Pharmacy, Ankara University Central Laboratory of the Faculty of Pharmacy, Ankara University The authors thank the Central Laboratory of the Faculty of Pharmacy, Ankara University, for providing support in the acquisition of the NMR, mass spectrometry, and elemental analysis instruments used in this study. The numerical calculations reported in this paper were partially performed at TUBITAK ULAKBIM, High Performance and Grid Computing Center (TRUBA resources).   69   9   9   2
15   AMER CHEMICAL SOC   WASHINGTON 1155 16TH ST, NW, WASHINGTON, DC 20036 USA   2470-1343   ACS OMEGA   ACS Omega FEB 14   2024   9   8   9547   9563
10.1021/acsomega.3c09411
http://dx.doi.org/10.1021/acsomega.3c09411   FEB 2024
17   Chemistry, MultidisciplinaryScience Citation Index Expanded (SCI-EXPANDED)   Chemistry   IW2U7 38434899   Green Published, gold   2025-06-24   WOS:001164671500001
J   Ranieri, J; Guerra, F; Cilli, E; Brancati, F; Di Giacomo, D
Ranieri, Jessica; Guerra, Federica; Cilli, Eleonora; Brancati, Francesco; Di Giacomo, Dina   BRCA genetic result disclosure for women with Breast Cancer: influence of +/predisposition genetic mutation   MEDITERRANEAN JOURNAL OF CLINICAL PSYCHOLOGY   English   Article
BRCA+/-; Psychological distress; Personality traits; Genetic testing; Psychological counseling; Breast cancer; Oncological clinical practice; Personalized medicine; Clinical psychology   ITALIAN VERSION; PSYCHOMETRIC PROPERTIES; EMOTION-REGULATION; IMPACT; RISK; DISTRESS; OUTCOMES; SURGERY; STRESS Background: Albeit the genetic testing/counseling is increasing progressively in oncological clinical practice, psychological impact of BRCA genetic testing has been an under-researched area in oncological population; few studies have examined a wide range of possible predictive individual factors for psychological adaptation after genetic testing for hereditary cancer. Aim of the study was to examine the implication of clinical psychological in BRCA genetic result post- disclosure dealing with the emotional health of patients undergoing genetic testing depending to the personal resources. Methods: Participants were composed of n = 32 female patients in range age 30 - 55 years, who have a BC diagnosis and who underwent BRCA mutation testing. Psychological battery was applied after genetic testing. Results: Our finding highlighted the psychological influence of genetic testing on wellbeing of BC patients, and more drawing clinical perspective for positive/negative disclosure regarding the predictors for psychological distress. Conclusion: Genetic testing needs to be integrated by psychological counseling to manage better the impact of result disclosure (whatever the outcome is) in order to manage better the physical and mental health of patients into efficient personalized medicine toward to the improvement of patient compliance and adherence into well-being perspective and Quality of Life maintaining. Sample size and

lack of longitudinal data could be limits of the study. [Ranieri, Jessica; Guerra, Federica; Cilli, Eleonora; Brancati, Francesco; Di Giacomo, Dina] Univ Aquila, Life Publ Hlth & Envornm Sci Dept, Laquila, Italy; [Brancati, Francesco] IRCCS, San Raffaele Roma, Human Funct Genom Lab, Rome, Italy University of L'Aquila
Ranieri, J (corresponding author), Univ Aquila, Life Publ Hlth & Envornm Sci Dept, Laquila, Italy.
jessica.ranieri@univaq.it Ranieri, Jessica/ADN-7258-2022; guerra, federica/HMO-5772-2023; Fava, Francesca/GPC-5378-2022; Di Giacomo, Dina/K-1983-2016 Cilli, Eleonora/0000-0002-0787-1277; Di Giacomo, Dina/0000-0001-8189-2052 40
1 1 2 4 UNIV STUDI MESSINA MESSINA VIA DEI VERDI, MESSINA, 98122, ITALY 2282-1619 MEDITERR J CLIN PSYC Mediterr. J. Clin. Psychol. 2023 11 2
10.13129/2282-1619/mjcp-3805 http://dx.doi.org/10.13129/2282-1619/mjcp-3805
17 Psychology, Clinical Emerging Sources Citation Index (ESCI) Psychology W8LG0 2025-06-24
WOS:001094082000020
J Varghese, B; Tamimi, M; Qureshi, A; Dayoub, N
Varghese, Bessy; Tamimi, Mariam; Qureshi, Abida; Dayoub, Nawal Randomly Selected Smear Test Screening Outcome BAHRAIN MEDICAL BULLETIN English
Article INVASIVE CERVICAL-CANCER; HUMAN-PAPILLOMAVIRUS; RISK; INFECTION; PARITY
Background: The cervical cancer screening coverage in Bahrain was 43.1% in 2018. Even with the presence of a screening program, most screening in the Middle East usually take place at random. Objective: To evaluate the risk factors and outcome of randomly selected smear tests. Design: A Retrospective Study. Setting: Bahrain Defence Force Hospital, Bahrain. Method: All Pap smear tests performed from January 2017 to December 2017 were included in the study. The smear results were divided into four groups: normal, borderline, premalignant changes and malignant changes. Risk factors such as age, parity, contraceptive/medical history, smoking, and human papillomavirus (HPV) positive screening were documented. The outcome of care were divided into four categories: repeat smear test, colposcopy and cervical biopsy, hysteroscopy/dilatation and curettage, and hysterectomy. Result: A total of 2,626 smear tests were included in the study; 2,246 (85.5%) were negative smears. Two hundred forty (9%) were borderline and 135 (5%) had pre-cancerous lesions. Five (0.2%) cases were malignant; the older the patient, the higher the risk of malignant changes. Twenty-nine (1.1%) of the previous pre-cancerous smear became negative on the successive smear and 72 (2.7%) patients had repeat smear test. Some of the negative and borderline patients had a hysterectomy. Conclusion: A uniform cervical screening policy must be initiated and cost-effective clear protocols must he laid down to improve the quality of women's health in Bahrain. [Varghese, Bessy; Tamimi, Mariam; Qureshi, Abida; Dayoub, Nawal] Bahrain Def Force Hosp, Royal Med Serv, Dept Obstet & Gynecol, Riffa, Bahrain Varghese, B (corresponding author), Bahrain Def Force Hosp, Royal Med Serv, Dept Obstet & Gynecol, Riffa, Bahrain. bessymiriam@hotmail.com
dayoub, nawal/W-9643-2018 dayoub, nawal/0000-0003-2454-3686 23 0 0 0 1 BAHRAIN MEDICAL BULLETIN KINGDOM BAHRAIN PO BOX 30880, KINGDOM BAHRAIN, 00000, BAHRAIN 1012-8298 BAHRAIN MED B Bahrain

Med. Bull. MAR 2020 42 1 31 34
4 Medicine, General &
Internal Emerging Sources Citation Index (ESCI) General &
Internal Medicine KN4WM 2025-06-24
WOS:000514839300006
J Cano-Ibáñez, N; Gea, A; Ruiz-Canela, M; Corella, D; Salas-Salvadó, J; Schröder, H; Navarrete-Muñoz, EM; Romaguera, D; Martínez, JA; Barón-López, FJ; López-Miranda, J; Estruch, R; Riquelme-Gallego, B; Alonso-Gómez, A; Tur, JA; Tinahones, FJ; Serra-Majem, L; Martín, V; Lapetra, J; Vázquez, C; Pintó, X; Vidal, J; Daimiel, L; Gaforio, JJ; Matía, P; Ros, E; Fernández-Carrión, R; Díaz-López, A; Zomeño, MD; Candela, I; Konieczna, J; Abete, I; Buil-Cosiales, P; Basora, J; Fitó, M; Martínez-González, MA; Bueno-Cavanillas, A Cano-Ibanez, Naomi; Gea, Alfredo; Ruiz-Canela, Miguel; Corella, Dolores; Salas-Salvado, Jordi; Schroder, Helmut; Navarrete-Munoz, Eva Ma; Romaguera, Dora; Alfredo Martinez, J.; Javier Baron-Lopez, F.; Lopez-Miranda, Jose; Estruch, Ramon; Riquelme-Gallego, Blanca; Alonso-Gomez, Angel; Tur, Josep A.; Tinahones, Francisco J.; Serra-Majem, Lluis; Martin, Vicente; Lapetra, Jose; Vazquez, Clotilde; Pinto, Xavier; Vidal, Josep; Daimiel, Lidia; Juan Gaforio, Jose; Matia, Pilar; Ros, Emilio; Fernandez-Carrion, Rebeca; Diaz-Lopez, Andres; Dolors Zomeno, M.; Candela, Inmaculada; Konieczna, Jadwiga; Abete, Itziar; Buil-Cosiales, Pilar; Basora, Josep; Fito, Montserrat; Martinez-Gonzalez, Miguel A.; Bueno-Cavanillas, Aurora Diet quality and nutrient density in subjects with metabolic syndrome: Influence of socioeconomic status and lifestyle factors. A cross-sectional assessment in the PREDIMED-Plus study CLINICAL NUTRITION
English Article Diet quality; Nutrient density; Mediterranean diet; Socioeconomic factors; Lifestyle factors; Metabolic syndrome PHYSICAL-ACTIVITY QUESTIONNAIRE; DWELLING OLDER-ADULTS; MEDITERRANEAN DIET; ENERGY-INTAKE; HEALTH; POPULATION; VALIDATION; PREVENTION; PATTERNS; ADEQUACY Background: Socioeconomic disparities and lifestyle factors are likely to determine the overall quality of the diet. In addition, overeating is compatible with inadequate micronutrient intake and it can lead to adverse health outcomes. Objective: To assess adequacy of dietary nutrient intake and to investigate the influence of socioeconomic and lifestyle factors on nutrient density in a large primary cardiovascular prevention trial conducted in healthy participants with metabolic syndrome (MetS) to assess the cardiovascular effects of an energy-restricted Mediterranean diet (PREDIMED-Plus). Methods: Baseline cross-sectional analysis of the PREDIMED-Plus trial with 6646 Spanish participants (aged 55-75 years in men and 60-75 years in women) with overweight/obesity and MetS. Energy and nutrient intake (for 10 nutrients) were calculated using a validated 143-item Food Frequency Questionnaire (FFQ) and nutrient density was estimated dividing the absolute nutrient intake by total energy intake. The prevalence of inadequate intake was estimated according to dietary reference intakes. Multivariable linear regression models were fitted to examine associations between socioeconomic status or lifestyle factors and nutrient density. Results: A considerable proportion of the screened participants showed a deficient intake of vitamins A, D, E, B-9, calcium, magnesium and dietary fibre. Inadequate intake of four or more of the ten nutrients considered was present in 17% of participants. A

higher nutrient density was directly and significantly associated with female sex, higher educational level and a better adherence to the Mediterranean diet. Lifestyle factors such as non-smoking and avoidance of sedentary lifestyles were also independently associated with better nutrient density. Conclusions: Patients with MetS, despite being overweight, exhibited suboptimal nutrient intake, especially among men. Low nutrient density diet can be largely explained by differences in socioeconomic and lifestyle factors. These results highlight the importance of focussing on nutritional education in vulnerable populations, taking into account nutrient requirements. (C) 2019 Elsevier Ltd and European Society for Clinical Nutrition and Metabolism. All rights reserved. [Cano-Ibanez, Naomi; Riquelme-Gallego, Blanca; Bueno-Cavanillas, Aurora] Univ Granada, Dept Prevent Med & Publ Hlth, Avda Invest 11, Granada 18016, Spain; [Cano-Ibanez, Naomi; Schroder, Helmut; Navarrete-Munoz, Eva Ma; Martin, Vicente; Juan Gaforio, Jose; Candela, Inmaculada; Bueno-Cavanillas, Aurora] ISCIII, CIBER Epidemiol & Salud Publ CIBERESP, Madrid, Spain; [Cano-Ibanez, Naomi; Bueno-Cavanillas, Aurora] IBS GRANADA, Inst Invest Biosanitaria Granada, Granada, Spain; [Gea, Alfredo; Ruiz-Canela, Miguel; Buil-Cosiales, Pilar; Martinez-Gonzalez, Miguel A.] Univ Navarra, Med Sch, Dept Prevent Med & Publ Hlth, Pamplona, Spain; [Gea, Alfredo; Ruiz-Canela, Miguel; Alfredo Martinez, J.; Abete, Itziar; Martinez-Gonzalez, Miguel A.] Navarra Inst Hlth Res IdisNa, Pamplona, Spain; [Gea, Alfredo; Ruiz-Canela, Miguel; Corella, Dolores; Salas-Salvado, Jordi; Romaguera, Dora; Alfredo Martinez, J.; Javier Baron-Lopez, F.; Lopez-Miranda, Jose; Estruch, Ramon; Alonso-Gomez, Angel; Tur, Josep A.; Tinahones, Francisco J.; Serra-Majem, Lluis; Lapetra, Jose; Vazquez, Clotilde; Pinto, Xavier; Ros, Emilio; Fernandez-Carrion, Rebeca; Diaz-Lopez, Andres; Konieczna, Jadwiga; Abete, Itziar; Buil-Cosiales, Pilar; Basora, Josep; Fito, Montserrat; Martinez-Gonzalez, Miguel A.] ISCIII, CIBER Fisiopatol Obesidad & Nutric CIBEROBN, Madrid, Spain; [Corella, Dolores; Fernandez-Carrion, Rebeca] Univ Valencia, Dept Prevent Med, Valencia, Spain; [Salas-Salvado, Jordi; Diaz-Lopez, Andres; Basora, Josep] Univ Rovira & Virgili, Dept Bioquim & Biotecnol, Unitat Nutr, Reus, Spain; [Salas-Salvado, Jordi; Diaz-Lopez, Andres; Basora, Josep] IISPV, Reus, Spain; [Salas-Salvado, Jordi; Diaz-Lopez, Andres; Basora, Josep] Univ Hosp St Joan Reus, Nutr Unit, Reus, Spain; [Schroder, Helmut; Dolors Zomeno, M.; Fito, Montserrat] Inst Hosp Mar Invest Med Municipal Invest Med IMI, Unit Cardiovasc Risk & Nutr, Barcelona, Spain; [Navarrete-Munoz, Eva Ma; Candela, Inmaculada] Miguel Hernandez Univ, Nutr Epidemiol Unit, ISABIAL FISABIO, Alicante, Spain; [Romaguera, Dora; Konieczna, Jadwiga] Hlth Res Inst Balearic Isl IdISBa, Palma De Mallorca, Spain; [Alfredo Martinez, J.; Abete, Itziar] Univ Navarra, Ctr Nutr Res, Dept Nutr Food Sci & Physiol, Pamplona, Spain; [Alfredo Martinez, J.; Daimiel, Lidia] CEI UAM CSIC, IMDEA Food, Nutr Genom & Epigen Grp, Madrid, Spain; [Javier Baron-Lopez, F.] Univ Malaga IBIMA, Dept Publ Hlth, Malaga, Spain; [Lopez-Miranda, Jose] Univ Cordoba, Reina Sofia Univ Hosp, Maimonides Biomed Res Inst Cordoba IMIBIC, Lipids & Atherosclerosis Unit,Dept Internal Med, Cordoba, Spain; [Estruch, Ramon] Univ Barcelona, Hosp Clin, Inst Invest Biomed August Pi & Sunyer IDIBAPS, Dept Internal Med, Barcelona, Spain; [Alonso-Gomez, Angel] Univ Basque Country, Univ Hosp Araba, Dept Cardiol, OSI ARABA,UPV EHU, Vitoria, Spain; [Tur, Josep A.] Univ Balearic Isl, Res Grp Community Nutr & Oxidat Stress, Palma De

Mallorca, Spain; [Tinahones, Francisco J.] Univ Malaga, Virgen Victoria Hosp, Inst Invest Biomed Malaga IBIMA, Dept Endocrinol, Malaga, Spain; [Serra-Majem, Lluis] Univ Las Palmas Gran Canaria, Res Inst Biomed & Hlth Sci, Las Palmas Gran Canaria, Spain; [Martin, Vicente] Univ Leon, Inst Biomed IBIOMED, Leon, Spain; [Lapetra, Jose] Dist Sanitario Atenc Primaria Sevilla, Res Unit, Dept Family Med, Seville, Spain; [Vazquez, Clotilde] Fdn Jimenez Diaz, Dept Endocrinol, Madrid, Spain; [Pinto, Xavier] Hosp Univ Bellvitge, Lipids & Vasc Risk Unit, Internal Med, Lhospitalet De Llobregat, Spain; [Vidal, Josep; Ros, Emilio] Hosp Clin Barcelona, Endocrinol & Nutr Serv, IDIBAPS, Barcelona, Spain; [Vidal, Josep] ISCIII, CIBER Diabet & Enfermedades Metabol CIBERDEM, Madrid, Spain; [Juan Gaforio, Jose] Univ Jaen, Ctr Estudios Avanzados Olivar & Aceites Oliva, Jaen, Spain; [Matia, Pilar] Inst Invest Sanitaria Hosp Clin San Carlos IdISSC, Dept Endocrinol & Nutr, Madrid, Spain; [Dolors Zomeno, M.] Blanquerna Ramon Llull Univ, Human Nutr Unit, Barcelona, Spain; [Buil-Cosiales, Pilar] Serv Navarro Salud Osasunbidea, Atenc Primaria, Pamplona, Spain; [Martinez-Gonzalez, Miguel A.] Harvard TH Chan Sch Publ Hlth, Dept Nutr, Boston, MA USA University of Granada; Instituto de Salud Carlos III; CIBER - Centro de Investigacion Biomedica en Red; CIBERESP; Instituto de Investigacion Biosanitaria IBS Granada; University of Navarra; University of Navarra; Instituto de Salud Carlos III; CIBER - Centro de Investigacion Biomedica en Red; CIBEROBN; University of Valencia; Universitat Rovira i Virgili; Universitat Rovira i Virgili; Institut d'Investigacio Sanitaria Pere Virgili (IISPV); General University Hospital of Alicante; Universidad Miguel Hernandez de Elche; Universitat d'Alacant; Instituto de Investigacion Sanitaria y Biomedica de Alicante (ISABIAL); Institut Investigacio Sanitaria Illes Balears (IdISBa); University of Navarra; Consejo Superior de Investigaciones Cientificas (CSIC); IMDEA Food Institute; Instituto de Investigacion Biomedica de Malaga y Plataforma en Nanomedicina (IBIMA); Universidad de Cordoba; University of Barcelona; Hospital Clinic de Barcelona; IDIBAPS; University Hospital of Araba; University of Basque Country; Universitat de les Illes Balears; Universidad de Malaga; Instituto de Investigacion Biomedica de Malaga y Plataforma en Nanomedicina (IBIMA); Universidad de Las Palmas de Gran Canaria; Universidad de Leon; Institut d'Investigacio Biomedica de Bellvitge (IDIBELL); Bellvitge University Hospital; University of Barcelona; Hospital Clinic de Barcelona; IDIBAPS; Instituto de Salud Carlos III; CIBER - Centro de Investigacion Biomedica en Red; CIBERDEM; Universidad de Jaen; Universitat Ramon Llull; Servicio Navarro de Salud - Osasunbidea; Harvard University; Harvard T.H. Chan School of Public Health

Cano-Ibáñez, N (corresponding author), Univ Granada, Dept Prevent Med & Publ Hlth, Avda Invest 11, Granada 18016, Spain.

ncaiba@ugr.es   Abad-Gurumeta, Alfredo/M-2337-2019; Tinahones, Francisco/AAB-2882-2020; Tur, Josep/AAE-5748-2020; Fernandez-Carrion, Rebeca/AAA-5713-2019; López, Francisco/O-9249-2016; Vidal, Josep/MIK-6936-2025; Martinez-Gonzalez, Miguel/AAE-7669-2019; Konieczna, Jadwiga/AAB-2817-2020; Pintó, Xavier/AGI-4297-2022; Lapetra, Jose/F-2552-2015; Lopez-Miranda, Jose/Y-8306-2019; Ruiz-Canela, Miguel/JYP-1794-2024; Navarrete-Muñoz, Eva/F-1666-2011; ALONSO GOMEZ, ANGEL/HLG-2476-2023; Romaguera, Dora/ABE-7004-2020; RIQUELME GALLEGO, BLANCA/AAB-1710-2020; Estruch, Ramon/AAZ-3723-2020; Corella, Dolores/L-9888-2014; Martin, Vicente/A-1597-2008; Daimiel-Ruiz, Lidia Angeles/M-7779-2014;

Schroder, Helmut/G-2586-2015; Barabash, Ana/B-1612-2012; Salas-Huetos, Albert/A-8509-2011; Salas-Salvado, Jordi/C-7229-2017; Ruiz-Canela, Miguel/I-7738-2016; Fito Colomer, Montse/C-1822-2012; Bueno Cavanillas, Aurora/O-1513-2015 Compan Gabucio, Laura M/0000-0001-5324-1535; Martin, Vicente/0000-0003-0552-2804; Daimiel-Ruiz, Lidia Angeles/0000-0001-9898-6629; Buil-Cosiales, Pilar/0000-0002-8586-577X; Schroder, Helmut/0000-0003-2231-5081; Cano-Ibanez, Naomi/0000-0002-3640-5486; Gea, Alfredo/0000-0002-2229-4690; Barabash, Ana/0000-0003-2383-1563; Tinahones, Francisco J/0000-0001-6871-4403; Basora, Josep/0000-0003-0278-1149; Vidal Cortada, Josep/0000-0002-4564-4518; Salas-Huetos, Albert/0000-0001-5914-6862; Salas-Salvado, Jordi/0000-0003-2700-7459; Diaz-Lopez, Andres/0000-0002-7500-5629; Romaguera, Dora/0000-0002-5762-8558; Pinto Sala, Xavier/0000-0002-2216-2444; Zomeno, M. Dolores/0000-0003-1280-7680; Lopez-Miranda, Jose/0000-0002-8844-0718; Ruiz-Canela, Miguel/0000-0002-7684-2787; Fito Colomer, Montse/0000-0002-1817-483X; Vazquez, Clotilde/0000-0003-1629-2143; Riquelme Gallego, Blanca/0000-0003-3422-7310; Bueno Cavanillas, Aurora/0000-0002-0649-3016 European Research Council [340918]; official funding agency for biomedical research of the Spanish Government, ISCIII through the Fondo de Investigacion para la Salud (FIS); European Regional Development Fund [PI13/00673, PI13/00492, PI13/00272, PI13/01123, PI13/00462, PI13/00233, PI13/02184, PI13/00728, PI13/01090, PI13/01056, PI14/01722, PI14/00636, PI14/00618]; The Especial Action Project entitled: "Implementacion y Evaluaci on de una intervencion intensiva sobre la actividad fisica Cohorte PREDIMED-Plus"; Consejeria de Salud de la Junta de Andalucia [PI0458/2013, PS0358/2016]; Generalitat Valenciana [PROMETEO/2017/017]; SEMERGEN grant; CIBEROBN, ISCIII [CB06/03]; FEDER funds, ISCIII [CB06/03]; International Nut&Dried Fruit Council-FESNAD [201302]; "FOLIUM" program within the FUTURMed project; Ministry of Education of Spain [FPU14/03630]; Recercaixa grant [2013ACUP00194]; [PI14/00696]; [PI14/01206]; [PI14/01919]; [PI14/00853]; [PI14/01374]; [PI16/00473]; [PI16/00662]; [PI16/01873]; [PI16/01094]; [PI16/00501]; [PI16/00533]; [PI16/00381]; [PI16/00366]; [PI16/01522]; [PI16/01120]; [PI17/00764]; [PI17/01183]; [PI17/00855]; [PI17/01347]; [PI17/00525]; [PI17/01827]; [PI17/00532]; [PI17/00215]; [PI17/01441]; [PI17/00508]; [PI17/01732]; [PI17/00926] European Research Council(European Research Council (ERC)); official funding agency for biomedical research of the Spanish Government, ISCIII through the Fondo de Investigacion para la Salud (FIS); European Regional Development Fund(European Union (EU)); The Especial Action Project entitled: "Implementacion y Evaluaci on de una intervencion intensiva sobre la actividad fisica Cohorte PREDIMED-Plus"; Consejeria de Salud de la Junta de Andalucia(Junta de Andalucia); Generalitat Valenciana(Center for Forestry Research & Experimentation (CIEF)); SEMERGEN grant; CIBEROBN, ISCIII; FEDER funds, ISCIII; International Nut&Dried Fruit Council-FESNAD; "FOLIUM" program within the FUTURMed project; Ministry of Education of Spain(Spanish Government); Recercaixa grant; The PREDIMED-Plus trial was supported by the European Research Council (Advanced Research Grant 2013-2018; 340918) grant to Miguel Angel Martinez-Gonzalez and by the official funding agency for biomedical research of the Spanish Government, ISCIII through the Fondo de Investigacion para la Salud (FIS), which is co-funded by the European Regional Development Fund (four

coordinated FIS projects led by Jordi Salas-Salvad~o and Josep Vidal), including the following projects: PI13/00673, PI13/00492, PI13/00272, PI13/01123, PI13/00462, PI13/00233, PI13/02184, PI13/00728, PI13/01090, PI13/01056, PI14/01722, PI14/00636, PI14/00618, PI14/00696, PI14/01206, PI14/01919, PI14/00853, PI14/01374, PI16/00473, PI16/00662, PI16/01873, PI16/01094, PI16/00501, PI16/00533, PI16/00381, PI16/00366, PI16/01522, PI16/01120, PI17/00764, PI17/01183, PI17/00855, PI17/01347, PI17/00525, PI17/01827, PI17/00532, PI17/00215, PI17/01441, PI17/00508, PI17/01732, PI17/00926. The Especial Action Project entitled: "Implementacion y Evaluaci on de una intervencion intensiva sobre la actividad fisica Cohorte PREDIMED-Plus" grant to Jordi SalasSalvado, the Recercaixa grant to Jordi Salas-Salvado (2013ACUP00194), the grant from the Consejeria de Salud de la Junta de Andalucia (PI0458/2013; PS0358/2016), the PROMETEO/2017/017 grant from the Generalitat Valenciana, the SEMERGEN grant, and CIBEROBN and FEDER funds (CB06/03), ISCIII. International Nut&Dried Fruit Council-FESNAD No 201302: Miguel ~Angel Martinez-Gonzalez. J. K. is supported by the "FOLIUM" program within the FUTURMed project. Talent for the medicine within the future from the Fundacio Institut d'Investigacio Sanitaria Illes Balears. This call is co-financed at 50% with charge to the Operational Program FSE 2014-2020 of the Balearic Islands. None of the funding sources took part in the design, collection, analysis or interpretation of the data, or in the decision to submit the manuscript for publication. N.CeI holds a grant from the Ministry of Education of Spain (FPU14/03630). The corresponding author had full access to all the data in the study and had final responsibility to submit for publication. 75
31 33 0 38 CHURCHILL LIVINGSTONE EDINBURGH
JOURNAL PRODUCTION DEPT, ROBERT STEVENSON HOUSE, 1-3 BAXTERS PLACE, LEITH WALK, EDINBURGH EH1 3AF, MIDLOTHIAN, SCOTLAND
0261-5614 1532-1983 CLIN NUTR Clin. Nutr. APR 2020
39 4 1161 1173
10.1016/j.clnu.2019.04.032
http://dx.doi.org/10.1016/j.clnu.2019.04.032 13
Nutrition & Dietetics Science Citation Index Expanded (SCI-EXPANDED) Nutrition & Dietetics LG1EZ 31101439
2025-06-24 WOS:000527854300022
J Laantri, N; Jalbout, M; Khyatti, M; Ben Ayoub, W; Dahmoul, S; Ayad, M; Bedadra, W; Abdoun, M; Mesli, S; Kandil, M; Hamdi-Cherif, M; Boualga, K; Bouaouina, N; Chouchane, L; Benider, A; Ben-Ayed, F; Goldgar, D; Corbex, M Laantri, Nadia; Jalbout, Majida; Khyatti, Meriem; Ben Ayoub, Wided; Dahmoul, Sami; Ayad, Messaoud; Bedadra, Wided; Abdoun, Meriem; Mesli, Sarah; Kandil, Mostafa; Hamdi-Cherif, Mokhtar; Boualga, Kada; Bouaouina, Noureddine; Chouchane, Lotfi; Benider, Abdellatif; Ben-Ayed, Farhat; Goldgar, David; Corbex, Marilys
XRCC1 and hOGG1 Genes and Risk of Nasopharyngeal Carcinoma in North African Countries MOLECULAR CARCINOGENESIS
English Article
nasopharyngeal carcinoma; DNA repair; XRCC1; hOGG1; North Africa LUNG-CANCER RISK; DNA-REPAIR GENE; GENOME-WIDE ASSOCIATION; BREAST-CANCER; SER326CYS POLYMORPHISM; SUSCEPTIBILITY; POPULATION; METAANALYSIS
Although genetic susceptibility to nasopharyngeal carcinoma (NPC) has been recognized for a long time, little is known about the responsible genes. X-Ray repair cross-complementing protein 1 (XRCC1) and

human 8-oxo-guanine glycosylase 1 (hOGG1) genes are involved in deoxyribonucleic acid (DNA) repair and were found associated with NPC risk in three Asian case-control studies. The objective of the present study was to test these genes in a sample from North Africa, one of the major NPC endemic regions in the world. Three single nucleotide polymorphisms (SNPs) in the XRCC1 gene and one SNP in the hOGG1 gene were genotyped in 598 NPC cases from Morocco, Algeria, and Tunisia and 545 controls frequency matched by recruitment center, age, sex, and urban/rural household. The genotype and allelic distributions for the hOGG1 (326)Ser/Cys SNP and for the XRCC1 (399)Arg/Trp, (280)Arg/His, and (194)Arg/Trp SNPs did not differ significantly among NPC cases and controls. The XRCC1 (194)Trp allele frequency was significantly lower in the North African population than in Asian population (f = 0.04 vs. 0.31 in Cantonese Chinese and 0.21 Han Chinese). The hOGG1 (326)Ser allele frequency was significantly higher in the North African population (f = 0.73) than in Asian populations (f = 0.39 in Taiwanese). The results of the present study obtained from a large sample indicate that the XRCC1 and hOGG1 genes are unlikely to play a role in the susceptibility to NPC in North Africans. Our results do not corroborate those found in Asian population on smaller samples. (C) 2011 Wiley-Liss, Inc. [Laantri, Nadia; Khyatti, Meriem] Inst Pasteur Maroc, Lab Oncovirol, Casablanca 20360, Morocco; [Laantri, Nadia; Kandil, Mostafa] Doukkali Univ, Lab Anthropogenet & Physiopathol Chouaib, El Jadida, Morocco; [Jalbout, Majida] Informat Univ St Esprit Kaslik USEK, Fac Sci & Genie, Dept Chim & Sci Vie, Lebanon, NH USA; [Ben Ayoub, Wided; Ben-Ayed, Farhat] Assoc Tunisienne Lutte Contre Canc, Tunis, Tunisia; [Dahmoul, Sami; Bouaouina, Noureddine] CHU Farhat Hached, Serv Radiotherapie, Sousse, Tunisia; [Ayad, Messaoud; Mesli, Sarah; Boualga, Kada] Ctr Anticanc Blida, Serv Radiotherapie Oncol, Blida, Algeria; [Bedadra, Wided; Abdoun, Meriem; Hamdi-Cherif, Mokhtar] CHU Setif, Serv Epidemiol, Setif, Algeria; [Chouchane, Lotfi] Fac Med, Lab Immunooncol Mol, Monastir, Tunisia; [Benider, Abdellatif] Ctr Oncol Ibn Rochd, Serv Radiotherapie, Casablanca, Morocco; [Goldgar, David] Univ Utah, Sch Med, Dept Dermatol, Salt Lake City, UT USA; [Corbex, Marilys] Int Agcy Res Canc, F-69372 Lyon, France Chouaib Doukkali University of El Jadida; Universite de Sousse; Hopital Farhat Hached; Universite de Monastir; Hassan II University of Casablanca; Ibn Rochd University Hospital Center of Casablanca; Utah System of Higher Education; University of Utah; World Health Organization; International Agency for Research on Cancer (IARC)

Khyatti, M (corresponding author), Inst Pasteur Maroc, Lab Oncovirol, 1 Pl Louis Pasteur, Casablanca 20360, Morocco.

Chouchane, Lotfi/ABF-1360-2020; ABDOUN, Meriem/ABB-6769-2020

CORBEX, Marilys/0000-0001-5755-7326; ABDOUN, Meriem/0000-0003-4055-1571 Association for International Cancer Research [03-252] Association for International Cancer Research

Without the funding support from the Association for International Cancer Research (grant number 03-252), this project would not have been accomplished. We also thank our colleagues, Ms. Valerie Gaborieau for efficient data management, Ms. Colette Bonnardel for data entry and checking, Ms. Helene Renard and Mr. Othman Yaqoubi for database design, Dr. Dominique Jeannel and Dr. Annie Sasco for help in designing the questionnaire, and Dr. Mohamed Maamer for its efficient help to the study in Tunis.

33 33 40 0 7 WILEY-BLACKWELL MALDEN

COMMERCE PLACE, 350 MAIN ST, MALDEN 02148, MA USA 0899-1987
MOL CARCINOGEN Mol. Carcinog. SEP 2011 50
9 732 737
10.1002/mc.20754 http://dx.doi.org/10.1002/mc.20754
6 Biochemistry & Molecular Biology; Oncology
Science Citation Index Expanded (SCI-EXPANDED)
Biochemistry & Molecular Biology; Oncology 808CM 21520294
2025-06-24 WOS:000293952200008
J Grant, WB Grant, William B.
Vitamin D and health in the Mediterranean countries
HORMONES-INTERNATIONAL JOURNAL OF ENDOCRINOLOGY AND METABOLISM English Article; Proceedings Paper 1st Mediterranean Expert Meeting on Vitamin D in the Prevention of Health Disparities during Adult Life SEP 29, 2017
Thessaloniki, GREECE Vitamin D; Autism; Cancer; Cardiovascular disease; Dental caries; Respiratory tract infections; All-cause mortality; Pregnancy and birth outcomes
SERUM 25-HYDROXYVITAMIN D; D SUPPLEMENTATION; BREAST-CANCER; D DEFICIENCY; HYPOVITAMINOSIS D; MORTALITY-RATES; DENTAL-CARIES; HUMAN-SKIN; CALCIUM SUPPLEMENTATION; CARDIOVASCULAR-DISEASE
Vitamin D, traditionally well known for its role in maintaining optimal health through its contribution to calcium metabolism and skeletal health, has received increased attention over the past two decades, with considerable focus being placed on its nonskeletal benefits. This paper is a narrative review of the nonskeletal health benefits of vitamin D, of particular interest to inhabitants of Mediterranean countries, namely, autism, cancer, cardiovascular disease, chronic obstructive pulmonary disease, dental caries, diabetes mellitus, erectile dysfunction, hypertension, metabolic syndrome, respiratory tract infections, all-cause mortality, and pregnancy and birth outcomes, because of the relatively high incidence and/or prevalence of these disorders in this region. Currently, the best evidence is coming out of observational studies related to serum 25-hydroxyvitamin D [25(OH)D] concentrations. Vitamin D clinical trials have generally been poorly designed and conducted, usually being based on vitamin D dose rather than 25(OH)D concentration. The optimal 25(OH)D concentration is above 75nmol/l (30ng/ml), with even better health outcomes in the range of 100-150nmol/l. Achieving these concentrations with vitamin D-3 supplements will require 1000-4000IU/day of vitamin D-3. Sensible sun exposure should also be encouraged. Countries should also consider fortifying grain and dairy products with vitamin D-3. [Grant, William B.] Sunlight Nutr & Hlth Res Ctr, POB 641603, San Francisco, CA 94164 USA
Grant, WB (corresponding author), Sunlight Nutr & Hlth Res Ctr, POB 641603, San Francisco, CA 94164 USA.
wbgrant@infionline.net Grant, William/B-8311-2009
134 12 13 1 18 SPRINGER INTERNATIONAL PUBLISHING AG CHAM GEWERBESTRASSE 11, CHAM, CH-6330, SWITZERLAND 1109-3099 2520-8721 HORM-INT J ENDOCRINO Horm.-Int. J. Endocrinol. Metab. MAR 2019 18 1
23 35 10.1007/s42000-018-0059-8 http://dx.doi.org/10.1007/s42000-018-0059-8
13 Endocrinology & Metabolism Science Citation Index Expanded (SCI-EXPANDED); Conference Proceedings Citation Index - Science (CPCI-S) Endocrinology & Metabolism HS3ES 30209779
2025-06-24 WOS:000463746300005

J Siddique, A; Ebrahim, H; Mohyeldin, M; Qusa, M; Batarseh, Y; Fayyad, A; Tajmim, A; Nazzal, S; Kaddoumi, A; El Sayed, K
Siddique, Abu Bakar; Ebrahim, Hassan; Mohyeldin, Mohamed; Qusa, Mohammed; Batarseh, Yazan; Fayyad, Ahmed; Tajmim, Afsana; Nazzal, Sami; Kaddoumi, Amal; El Sayed, Khalid
Novel liquid-liquid extraction and self-emulsion methods for simplified isolation of extra-virgin olive oil phenolics with emphasis on (-)-oleocanthal and its oral anti-breast cancer activity PLOS ONE English Article
IN-VITRO; INHIBITS PROLIFERATION; OLEOCANTHAL; OLEACEIN; ACTIVATION; EXPRESSION; OLEUROPEIN; COMPOUND Epidemiological and clinical studies compellingly documented the ability of Mediterranean diet rich in extra-virgin olive oil (EVOO) to reduce breast and colon cancers incidence, cardiovascular diseases, and aging cognitive functions decline. (-)-Oleocanthal (OC) and other EVOO phenolics gain progressive research attention due to their documented biological effects against cancer, inflammations, and Alzheimer's disease. There is no simple, reliable, and cost-effective isolation protocol for EVOO phenolics, which hinder their therapeutic applications. This study develops novel methods to isolate OC and other EVOO phenolics. This includes the use of ultra-freezing to eliminate most EVOO fats and the successful water capacity to efficiently extract OC and EVOO phenolics as self-emulsified nano-emulsion. Subsequent resin entrapment and size exclusion chromatography afforded individual EVOO phenolics in high purity. OC in vitro and in vivo oral anti-breast cancer (BC) activities validated its lead candidacy. Effective isolation of EVOO phenolics provided in this study will facilitate future preclinical and clinical investigations and stimulate the therapeutic development of these important bioactive natural products. [Siddique, Abu Bakar; Ebrahim, Hassan; Mohyeldin, Mohamed; Qusa, Mohammed; Batarseh, Yazan; Fayyad, Ahmed; Tajmim, Afsana; Nazzal, Sami; El Sayed, Khalid] Univ Louisiana Monroe, Coll Pharm, Sch Basic Pharmaceut & Toxicol Sci, Monroe, LA 71209 USA; [Kaddoumi, Amal] Auburn Univ, Harrison Sch Pharm, Dept Drug Discovery & Dev, Auburn, AL 36849 USA University of Louisiana System; University of Louisiana Monroe; Auburn University System; Auburn University El Sayed, K (corresponding author), Univ Louisiana Monroe, Coll Pharm, Sch Basic Pharmaceut & Toxicol Sci, Monroe, LA 71209 USA.
elsayed@ulm.edu Ebrahim, Hassan/F-2050-2017; , Abu Bakar Siddique/W-8476-2019; Mohyeldin, Mohamed/K-5059-2017 El Sayed, Khalid/0000-0002-1456-4064; Mohyeldin, Mohamed/0000-0001-9052-7620; Ebrahim, Hassan Y./0000-0001-9057-4582; Siddique, Abu Bakar/0000-0001-5424-9475; Tajmim, Afsana/0000-0003-4717-1141
Louisiana Board of Regents [LEQSF(2017-20)-RD-B-07]; National Cancer Institute of the National Institutes of Health [R15CA167475] Louisiana Board of Regents; National Cancer Institute of the National Institutes of Health(United States Department of Health & Human ServicesNational Institutes of Health (NIH) - USANIH National Cancer Institute (NCI)) Research reported in this publication was supported by the Louisiana Board of Regents, Award Number LEQSF(2017-20)-RD-B-07 and the National Cancer Institute of the National Institutes of Health under Award Number R15CA167475. The funders had no role in study design, data collection and analysis, decision to publish, or preparation of the study. 35 37 39 0 23 PUBLIC LIBRARY SCIENCE SAN FRANCISCO 1160 BATTERY STREET, STE 100, SAN

FRANCISCO, CA 94111 USA 1932-6203 PLOS ONE PLoS One APR 9 2019 14 4
e0214798 10.1371/journal.pone.0214798
http://dx.doi.org/10.1371/journal.pone.0214798
20 Multidisciplinary Sciences Science Citation Index Expanded (SCI-EXPANDED) Science & Technology - Other Topics
HS4YI 30964898 Green Submitted, gold, Green Published
2025-06-24 WOS:000463876300030
J Cianciosi, D; Forbes-Hernández, TY; Ansary, J; Gil, E; Amici, A; Bompadre, S; Simal-Gandara, J; Giampieri, F; Battino, M Cianciosi, Danila; Forbes-Hernandez, Tamara Y.; Ansary, Johura; Gil, Emilio; Amici, Adolfo; Bompadre, Stefano; Simal-Gandara, Jesus; Giampieri, Francesca; Battino, Maurizio
Phenolic compounds from Mediterranean foods as nutraceutical tools for the prevention of cancer: The effect of honey polyphenols on colorectal cancer stem-like cells from spheroids FOOD CHEMISTRY English Article; Proceedings Paper 2nd Food Chemistry Conference on Shaping the Future of Food Quality, Safety, Nutrition and Health SEP 17-19, 2019 Seville, SPAIN 3D culture; Cancer stem cells; Chemoresistance; Manuka honey; Self-renewal; Tumorspheres; Spheroids SELF-RENEWAL CAPACITY; BREAST-CANCER; MODULATION; RESISTANCE; APOPTOSIS; PATHWAY; TARGET Chemoresistance and development of relapses are ascribable to a rare cell population of tumour mass: cancer stem cells (CSCs). Targeting CSCs could increase patients' survival rate and it is important to identify molecules that can act on the main pathways of these cells. Natural bioactive compounds, of which Manuka honey (MH) is rich, could be a good opportunity to target them. This work aims to evaluate the effect of MH on CSCs-like from human colorectal carcinoma (HCT-116 cell line) enriched through the in vitro sphere-forming assay. The results showed that MH reduced the volume of the entire culture of spheroids, affecting also their morphological parameters and induced apoptosis and ROS intracellular accumulation in CSCs-like. In addition, MH decreased the mRNA expression of one of ABC transporters (ABCG2) and affected self-renewal ability through the downregulation of the mRNA expression of one of the receptor membranes of Wnt/beta-catenin pathway (Frizzled 7). [Cianciosi, Danila; Ansary, Johura; Amici, Adolfo; Giampieri, Francesca; Battino, Maurizio] Univ Politecn Marche, Dipartimento Sci Clin Specialistiche & Odontostom, Via Ranieri 65, I-60130 Ancona, Italy; [Forbes-Hernandez, Tamara Y.; Giampieri, Francesca; Battino, Maurizio] Univ Vigo, Nutr & Food Sci Grp, Dept Analyt & Food Chem, CITACA,CACTI, Vigo Campus, Orense 32004, Spain; [Gil, Emilio] Univ Vigo, Fac Biol, Dept Biochem Genet & Immunol, Nutr & Food Sci Grp, Vigo, Spain; [Bompadre, Stefano] Univ Politecn Marche, Dipartimento Sci Biomed & Sanita Pubbl, Via Ranieri 65, I-60130 Ancona, Italy; [Simal-Gandara, Jesus] Univ Vigo, Fac Sci, Dept Analyt & Food Chem, Nutr & Bromatol Grp, Ourense Campus, E-32004 Orense, Spain; [Giampieri, Francesca] Northwest Univ, Coll Food Sci & Technol, Xian 710069, Shaanxi, Peoples R China; [Battino, Maurizio] Jiangsu Univ, Int Res Ctr Food Nutr & Safety, Zhenjiang 212013, Jiangsu, Peoples R China Marche Polytechnic University; Universidade de Vigo; Universidade de Vigo; Marche Polytechnic University; Universidade de Vigo; Northwest University Xi'an; Jiangsu University Giampieri, F; Battino, M (corresponding author), Univ Politecn Marche, Dipartimento Sci Clin

Specialistiche & Odontostom, Via Ranieri 65, I-60130 Ancona, Italy.	d.cianciosi@pm.univpm.it; tforbes@uvigo.es; egil@uvigo.es; a.amici@staff.univpm.it; s.bompadre@univpm.it; jsimal@uvigo.es; f.giampieri@univpm.it; m.a.battino@univpm.it
Giampieri, Francesca/I-1911-2015; Cianciosi, Danila/H-7405-2019; Ansary, J/AAL-2565-2021; Forbes Hernandez, Tamara/AAB-1872-2021; Amici, Adolfo/C-5342-2012; Battino, Maurizio/E-6103-2012; Simal-Gandara, Jesus/A-9533-2009	Forbes Hernandez, Tamara/0000-0001-7021-9276; Amici, Adolfo/0000-0002-1081-7749; Battino, Maurizio/0000-0002-7250-1782; Cianciosi, Danila/0000-0002-8781-3535; Ansary, Ph.D, Johura/0000-0003-3301-7215; Simal-Gandara, Jesus/0000-0001-9215-9737	Juan de la Cierva-Formacion	Juan de la Cierva-Formacion	Tamara Y. Forbes-Hernandez is supported by a "Juan de la Cierva-Formacion" post-doctoral contract.	39
55	56	5	57	ELSEVIER SCI LTD	OXFORD	THE BOULEVARD, LANGFORD LANE, KIDLINGTON, OXFORD OX5 1GB, OXON, ENGLAND	0308-8146	1873-7072		FOOD CHEM	Food Chem.	SEP 30	2020	325			126881
10.1016/j.foodchem.2020.126881
http://dx.doi.org/10.1016/j.foodchem.2020.126881
10	Chemistry, Applied; Food Science & Technology; Nutrition & Dietetics	Science Citation Index Expanded (SCI-EXPANDED); Conference Proceedings Citation Index - Science (CPCI-S)	Chemistry; Food Science & Technology; Nutrition & Dietetics
LR9ZL	32387951			2025-06-24
WOS:000536053400022
J	García-Villalba, R; Carrasco-Pancorbo, A; Oliveras-Ferraros, C; Menéndez, JA; Segura-Carretero, A; Fernández-Gutiérrez, A
Garcia-Villalba, Rocio; Carrasco-Pancorbo, Alegria; Oliveras-Ferraros, Cristina; Menendez, Javier A.; Segura-Carretero, Antonio; Fernandez-Gutierrez, Alberto
Uptake and metabolism of olive oil polyphenols in human breast cancer cells using nano-liquid chromatography coupled to electrospray ionization-time of flight-mass spectrometry	JOURNAL OF CHROMATOGRAPHY B-ANALYTICAL TECHNOLOGIES IN THE BIOMEDICAL AND LIFE SCIENCES			English	Article
Olive oil; Phenolic compounds; Cancer cells; Metabolites; Nano-liquid chromatography; Electrospray-time of flight-mass; spectrometry	PHENOLIC-COMPOUNDS; IN-VITRO; HYDROXYTYROSOL; PREVENTION; HEALTH; HL60	Polyphenols from extra virgin olive oil (EVOO), a main component of the Mediterranean diet, have demonstrated repeatedly anti-tumor activity in several in vitro and in vivo studies. However, little is known about the efficiency of the absorption process and metabolic conversion of these compounds at cellular level. In this study, a nano liquid chromatography-electrospray ionization-time of flight mass spectrometry (nanoLC-ESI-TOF MS) method was developed to study the cellular uptake and metabolism of olive oil phenols in JIMT-1 human breast cancer cells. After incubation for different time periods with EVOO-derived phenolic extracts, culture media, cytosolic fraction and solid particles fraction were separated and analyzed. Most of the free phenols, mainly hydroxytyrosol, its secoiridoid derivatives, and the flavonoid luteolin, disappeared in the culture media in different ways and at different times. Besides, several metabolites were detected in the culture media, fact that may indicate absorption and intracellular metabolism followed by rapid cellular export. Low intracellular accumulation was observed with only traces of some

compounds detected in the cytosolic and solid particles fractions. Methylated conjugates were the major metabolites detected, suggesting a catalytic action of catechol-O-methyl transferase (COMT) in cancer cells. (C) 2012 Elsevier B.V. All rights reserved. [Carrasco-Pancorbo, Alegria; Fernandez-Gutierrez, Alberto] Univ Granada, Fac Sci, Dept Analyt Chem, Res Grp FQM 297, E-18071 Granada, Spain; [Oliveras-Ferraros, Cristina; Menendez, Javier A.] Dr Josep Trueta Univ Hosp, Girona Biomed Res Inst, Catalan Inst Oncol, Girona, Spain University of Granada; Institut Catala d'Oncologia; Universitat de Girona; Girona University Hospital Dr. Josep Trueta; Institut d'Investigacio Biomedica de Girona (IDIBGI) Carrasco-Pancorbo, A (corresponding author), Univ Granada, Fac Sci, Dept Analyt Chem, Res Grp FQM 297, Ave Fuentenueva S-N, E-18071 Granada, Spain. alegriac@ugr.es; albertof@ugr.es Villalba, Rocio/G-7217-2015; segura Carretero, Antonio/B-6867-2014; Simal-Gandara, Jesus/A-9533-2009; MENENDEZ MENENDEZ, JAVIER ABEL/C-6148-2016; Fernandez Gutierrez, Alberto/M-8512-2014 segura Carretero, Antonio/0000-0002-5564-5338; Carrasco Pancorbo, Alegria/0000-0001-8856-4676; Garcia Villalba, Rocio/0000-0003-1883-1673; Simal-Gandara, Jesus/0000-0001-9215-9737; MENENDEZ MENENDEZ, JAVIER ABEL/0000-0001-8733-4561; Fernandez Gutierrez, Alberto/0000-0003-3647-2598 Instituto de Salud Carlos III (Ministerio de Sanidad y Consumo, Fondo de Investigacion Sanitaria - FIS, Spain) [CP05-00090, PI06-0778, RD06-0020-0028]; Fundacion Cientifica de la Asociacion Espanola Contra el Cancer (AECC, Spain); Ministerio de Ciencia e Innovacion [SAF2009-11579] Instituto de Salud Carlos III (Ministerio de Sanidad y Consumo, Fondo de Investigacion Sanitaria - FIS, Spain)(Instituto de Salud Carlos III); Fundacion Cientifica de la Asociacion Espanola Contra el Cancer (AECC, Spain); Ministerio de Ciencia e Innovacion(Spanish GovernmentInstituto de Salud Carlos III) The authors are very grateful to Ministry of Education and Science (FPU, AP2005-4356) and Junta de Andalucia (project P09-FQM-5469, project P07-AGR-02619 and AGL 2008-05108-CO3-03/ALI). Besides, this work was supported in part by Instituto de Salud Carlos III (Ministerio de Sanidad y Consumo, Fondo de Investigacion Sanitaria - FIS, Spain, Grants CP05-00090, PI06-0778 and RD06-0020-0028 to Javier A. Menendez). Javier A. Menendez was also supported by a Grant from the Fundacion Cientifica de la Asociacion Espanola Contra el Cancer (AECC, Spain) and by the Ministerio de Ciencia e Innovacion (SAF2009-11579, Plan Nacional de I + D + I, MICINN, Spain). 30 27 27 1 31
ELSEVIER SCIENCE BV AMSTERDAM PO BOX 211, 1000 AE AMSTERDAM, NETHERLANDS 1570-0232 1873-376X J CHROMATOGR B
J. Chromatogr. B JUN 1 2012 898
69 77 10.1016/j.jchromb.2012.04.021
http://dx.doi.org/10.1016/j.jchromb.2012.04.021
9 Biochemical Research Methods; Chemistry, Analytical
Science Citation Index Expanded (SCI-EXPANDED)
Biochemistry & Molecular Biology; Chemistry 956NZ 22608806
Green Accepted 2025-06-24 WOS:000305097700009
J Licciardi, M; Li Volsi, A; Mauro, N; Scialabba, C; Cavallaro, G; Giammona, G Licciardi, Mariano; Li Volsi, Anna; Mauro, Nicolo; Scialabba, Cinzia; Cavallaro, Gennara; Giammona, Gaetano Preparation and Characterization of Inulin Coated Gold Nanoparticles for Selective Delivery of Doxorubicin to Breast Cancer Cells JOURNAL OF NANOMATERIALS English Article

THERAPEUTICS; PACLITAXEL; HYDROGELS; TOOL

A novel folate-targeted gold-based nanosystem for achieving selectivity towards folate receptor (FR) positive cells is proposed, by virtue of the fact that the FR is a molecularly targeted entity overexpressed in a wide spectrum of solid tumors. A new inulin-folate derivative (INU-FA) has been synthesized to act as coating agent for 40 nm gold nanoparticles. The obtained polymer-coated gold nanoparticles (Au@INU-FA) were characterized in terms of hydrodynamic radius, shape, zeta potential, and aqueous stability and were loaded with doxorubicin (Au@INU-FA/Doxo). Its release capability was tested in different release media. The selectivity of Au@INU-FA/Doxo system towards FRs-positive cancer cells was proved by the differences in the quantitative uptake using human breast cancer MCF7 as FR-positive cells and 16HBE epithelial as noncancer cell line. Furthermore, the folate-mediated uptake mechanism was studied by FRs-blocking experiments. On the whole Au@INU-FA/Doxo was able to be preferentially internalized into MCF7 cells proving a folate-mediated endocytosis mechanism which allowed a higher and selective cytotoxic effect towards cancer cells. The cytotoxicity profile was evaluated on both cancer and noncancer cell lines, displaying that folate-mediated targeting implied advantageous therapeutic effects, such as amplified drug uptake and increased anticancer activity towards MCF7 cancer cells. [Licciardi, Mariano; Li Volsi, Anna; Mauro, Nicolo; Scialabba, Cinzia; Cavallaro, Gennara; Giammona, Gaetano] Univ Palermo, Dipartimento Sci & Tecnol Biol Chim & Farmaceut, STEBICEF, Via Archirafi 32, I-90123 Palermo, Italy; [Licciardi, Mariano; Giammona, Gaetano] Univ Palermo, Mediterranean Ctr Human Hlth Adv Biotechnol CHAB, ATeN Ctr, Palermo, Italy University of Palermo; University of Palermo Licciardi, M (corresponding author), Univ Palermo, Dipartimento Sci & Tecnol Biol Chim & Farmaceut, STEBICEF, Via Archirafi 32, I-90123 Palermo, Italy.; Licciardi, M (corresponding author), Univ Palermo, Mediterranean Ctr Human Hlth Adv Biotechnol CHAB, ATeN Ctr, Palermo, Italy. mariano.licciardi@unipa.it

Lazzara, Giuseppe/O-3356-2013 Mauro, Nicolo/0000-0003-0246-3474; LICCIARDI, Mariano/0000-0003-4539-9337; CAVALLARO, Gennara/0000-0003-0585-6564; SCIALABBA, Cinzia/0000-0002-5209-9319

MIUR; University of Palermo MIUR(Ministry of Education, Universities and Research (MIUR)); University of Palermo The authors thank the MIUR and the University of Palermo for funding.

34 25 25 2 24 HINDAWI LTD LONDON ADAM HOUSE, 3RD FLR, 1 FITZROY SQ, LONDON, W1T 5HF, ENGLAND 1687-4110 1687-4129 J NANOMATER J. Nanomater. 2016 2016 2078315

10.1155/2016/2078315 http://dx.doi.org/10.1155/2016/2078315

12 Nanoscience & Nanotechnology; Materials Science, Multidisciplinary Science Citation Index Expanded (SCI-EXPANDED) Science & Technology - Other Topics; Materials Science

DY5FU Green Submitted, gold, Green Published

2025-06-24 WOS:000385125400001

J Jumbri, IA; Ikeda, S; Managi, S Jumbri, Isma Addi; Ikeda, Shinya; Managi, Shunsuke

Heterogeneous global health stock and growth: quantitative evidence from 140 countries, 1990-2100 ARCHIVES OF PUBLIC HEALTH English Article

Sustainable development; Health stock; Health forecasting; Time series INCLUSIVE WEALTH; LIFE EXPECTANCY; FERTILITY; IMPACT;

INCOME	BackgroundIn the prevailing economic perspective, health is viewed as a type of capital stock that yields healthy days' in human society. However, evaluations of this health capital stock are still limited to specific contexts. The primary aim of this study is to measure and forecast the global health stocks in 140 countries from 1990 to 2100.MethodsThe health capital stock in each country from 1990 to 2015 was estimated using a capital approach. The future health stocks between 2016 and 2100 were forecast using a time-series model.ResultsBased on the health stocks from 1990 to 2015, low-income countries have much larger and more rapidly growing health stocks. In the long-term, to 2100, upper-middle income countries, particularly countries in the Middle East and North Africa, exhibit great growth that benefits from the peaks in their youth or working-age populations. Immigration also contributes to health stock growth, as do other factors, e.g., the fertility rate, population ageing, and working-age and youth populations.ConclusionsHealth stock is a vital component of global sustainable development that should be consistently included as a stock-based sustainability index in the evaluations of other capital to accurately measure national wealth and sustainability.	[Jumbri, Isma Addi] Tohoku Univ, Grad Sch Environm Studies, Sendai, Miyagi, Japan; [Ikeda, Shinya] Ibaraki Univ, Coll Agr Reg & Environm Sci, Inashiki, Ibaraki, Japan; [Managi, Shunsuke] Kyushu Univ, Dept Urban & Environm Engn, Fukuoka, Fukuoka, Japan; [Managi, Shunsuke] Kyushu Univ, Urban Inst, Fukuoka, Fukuoka, Japan	Tohoku University; Ibaraki University; Kyushu University; Kyushu University	Managi, S (corresponding author), Kyushu Univ, Dept Urban & Environm Engn, Fukuoka, Fukuoka, Japan.; Managi, S (corresponding author), Kyushu Univ, Urban Inst, Fukuoka, Fukuoka, Japan.	managi.s@gmail.com	Ikeda, Shinya/AAF-6878-2020; Managi, Shunsuke/G-1740-2013	Managi, Shunsuke/0000-0001-7883-1427; Ikeda, Shinya/0000-0001-8479-7958	Japan Society for the Promotion of Science [26000001] Japan Society for the Promotion of Science(Ministry of Education, Culture, Sports, Science and Technology, Japan (MEXT)Japan Society for the Promotion of Science)	This study is funded by the Grant-in-Aid for Specially Promoted Research (26000001) by the Japan Society for the Promotion of Science.	48	2	2	0	10	BMC	LONDON	CAMPUS, 4 CRINAN ST, LONDON N1 9XW, ENGLAND	0778-7367	2049-3258		ARCH PUBLIC HEALTH	Arch. PUblic Health	DEC 28	2018	76	81	10.1186/s13690-018-0327-8	http://dx.doi.org/10.1186/s13690-018-0327-8	14	Public, Environmental & Occupational Health	Science Citation Index Expanded (SCI-EXPANDED); Social Science Citation Index (SSCI)	Public, Environmental & Occupational Health	HF9JK	30607246	Green Published, gold		2025-06-24	WOS:000454558100001
J	Martin-Calvo, N; Chavarro, JE; Falbe, J; Hu, FB; Field, AE		Martin-Calvo, N.; Chavarro, J. E.; Falbe, J.; Hu, F. B.; Field, A. E.		Adherence to the Mediterranean dietary pattern and BMI change among US adolescents	INTERNATIONAL JOURNAL OF OBESITY		English	Article		SELF-REPORTED WEIGHT; METABOLIC SYNDROME; GREEK ADOLESCENTS; LIFE-STYLE; SAMPLE; CHILDREN; FOOD; COMPONENTS; QUALITY; PREVENTION	BACKGROUND: Among adults, the Mediterranean dietary pattern (MDP) is inversely related to body mass index (BMI). Data are lacking on adherence to

the MDP among youth in the United States and whether the MDP is related to weight change in that group. OBJECTIVE: To assess whether adherence to the MDP was associated with BMI change among adolescents. To examine temporality, we studied the association between baseline and 2-3-year changes in adherence to the MDP with concurrent changes in BMI, as well as subsequent changes in BMI over a 7-year period. METHODS: We prospectively followed 6002 females and 4916 males in the Growing Up Today Study II, aged 8-15 years in 2004, living across United States. Data were collected by questionnaire in 2004, 2006, 2008 and 2011. Dietary intake was assessed by the Youth/Adolescent Questionnaire. The KidMed Index was derived to measure the adherence to the MDP. We used generalized estimating equations with repeated measures within subjects to assess the association between MDP and BMI change. RESULTS: A two-point increment in the KidMed Index was independently associated with a lower gain in BMI (-0.04 kg m(-2); P = 0.001). A greater increase in adherence to the KidMed Index was independently related to a lower gain in BMI in both the concurrent (P-for-trend < 0.001) and the subsequent period (P-for-trend = 0.002). CONCLUSIONS: Adherence to MDP was inversely associated with change in BMI among adolescents. Two-year improvement in adherence to MDP was independently associated with less steep gain in the BMI in both the concurrent and the subsequent period. [Martin-Calvo, N.] Univ Navarra, Dept Prevent Med & Publ Hlth, IdiSNA Inst Invest Sanitaria Navarra, Pamplona, Spain; [Martin-Calvo, N.] Inst Hlth Carlos III, Ctr Invest Biomed Red Fisiopatol Obesidad & Nutr, Madrid, Spain; [Chavarro, J. E.; Hu, F. B.] Harvard TH Chan Sch Publ Hlth, Dept Nutr, Boston, MA USA; [Chavarro, J. E.; Hu, F. B.; Field, A. E.] Brigham & Womens Hosp, Dept Med, Channing Div Network Med, 75 Francis St, Boston, MA 02115 USA; [Chavarro, J. E.; Hu, F. B.; Field, A. E.] Harvard Med Sch, Boston, MA USA; [Falbe, J.] Univ Calif Berkeley, Div Community Hlth & Human Dev, Berkeley, CA 94720 USA; [Field, A. E.] Boston Childrens Hosp, Div Adolescent Med, Boston, MA USA; [Field, A. E.] Harvard TH Chan Sch Publ Hlth, Dept Epidemiol, Boston, MA USA; [Field, A. E.] Brown Univ, Sch Publ Hlth, Dept Epidemiol, 121 S Main St, Providence, RI 02912 USA
University of Navarra; CIBER - Centro de Investigacion Biomedica en Red; CIBEROBN; Harvard University; Harvard T.H. Chan School of Public Health; Harvard University; Harvard University Medical Affiliates; Brigham & Women's Hospital; Harvard University; Harvard Medical School; University of California System; University of California Berkeley; Harvard University; Harvard University Medical Affiliates; Boston Children's Hospital; Harvard University; Harvard T.H. Chan School of Public Health; Brown University Field, AE (corresponding author), Brown Univ, Sch Publ Hlth, Dept Epidemiol, 121 S Main St, Providence, RI 02912 USA. Alison_Field@brown.edu Hu, Frank/C-1919-2013; Field, Alison/AAA-4508-2021; Chavarro, Jorge/AAE-5665-2021; Martin-Calvo, Nerea/D-1727-2017 Falbe, Jennifer/0000-0002-8405-4326; Chavarro, Jorge/0000-0002-4436-9630; Martin-Calvo, Nerea/0000-0001-7549-1455 Breast Cancer Research Foundation [R01-DK084001, R01-HL096905]; National Institute of Health Breast Cancer Research Foundation; National Institute of Health(United States Department of Health & Human ServicesNational Institutes of Health (NIH) - USA) We thank the thousands of participants in the Growing Up Today Study II and their mothers. The Growing up Today Study II is supported by the Breast Cancer Research Foundation and

the grants R01-DK084001 and R01-HL096905 from the National Institute of Health. 34 36 36 0 9
SPRINGERNATURE LONDON CAMPUS, 4 CRINAN ST, LONDON, N1 9XW, ENGLAND 0307-0565 1476-5497 INT J OBESITY Int. J. Obes. JUL 2016 40 7 1103 1108
10.1038/ijo.2016.59
http://dx.doi.org/10.1038/ijo.2016.59 6
Endocrinology & Metabolism; Nutrition & Dietetics Science Citation Index Expanded (SCI-EXPANDED) Endocrinology & Metabolism; Nutrition & Dietetics DQ8YS 27102053 Bronze, Green Accepted 2025-06-24 WOS:000379498200009

J Mordukhovich, I; Rossner, P; Terry, MB; Santella, R; Zhang, YJ; Hibshoosh, H; Memeo, L; Mansukhani, M; Long, CM; Garbowski, G; Agrawal, M; Gaudet, MM; Steck, SE; Sagiv, SK; Eng, SM; Teitelbaum, SL; Neugut, AI; Conway-Dorsey, K; Gammon, MD
Mordukhovich, Irina; Rossner, Pavel, Jr.; Terry, Mary Beth; Santella, Regina; Zhang, Yu-Jing; Hibshoosh, Hanina; Memeo, Lorenzo; Mansukhani, Mahesh; Long, Chang-Min; Garbowski, Gail; Agrawal, Meenakshi; Gaudet, Mia M.; Steck, Susan E.; Sagiv, Sharon K.; Eng, Sybil M.; Teitelbaum, Susan L.; Neugut, Alfred I.; Conway-Dorsey, Kathleen; Gammon, Marilie D.
Associations between Polycyclic Aromatic Hydrocarbon-Related Exposures and p53 Mutations in Breast Tumors ENVIRONMENTAL HEALTH PERSPECTIVES English Article
breast cancer; p53 mutation; p53 overexpression; PAH; polycylic aromatic hydrocarbons CIGARETTE-SMOKING; RISK-FACTORS; CANCER RISK; DNA-ADDUCTS; LONG-ISLAND; TOBACCO-SMOKE; EXPRESSION; DAMAGE; MEAT; POLLUTION BACKGROUND: Previous studies have suggested that polycyclic aromatic hydrocarbons (PAHs) may be associated with breast cancer. However, the carcinogenicity of PAHs on the human breast remains unclear. Certain carcinogens may be associated with specific mutation patterns in the p53 tumor suppressor gene, thereby contributing information about disease etiology. OBJECTIVES: We hypothesized that associations of PAH-related exposures with breast cancer would differ according to tumor p53 mutation status, effect, type, and number. METHODS: We examined this possibility in a population-based case control study using polytomous logistic regression. As previously reported, 151 p53 mutations among 859 tumors were identified using Surveyor nuclease and confirmed by sequencing. RESULTS: We found that participants with p53 mutations were less likely to be exposed to PAHs (assessed by smoking status in 859 cases and 1,556 controls, grilled/smoked meat intake in 822 cases and 1,475 controls, and PAH-DNA adducts in peripheral mononuclear cells in 487 cases and 941 controls) than participants without p53 mutations. For example, active and passive smoking was associated with p53 mutation negative [odds ratio (OR) = 1.55; 95% confidence interval (CI), 1.11-2.15] but not p53 mutation positive (OR = 0.77; 95% CI, 0.43-1.38) cancer (ratio of the ORs = 0.50, p < 0.05). However, frameshift mutations, mutation number, G:C -> A:T transitions at CpG sites, and insertions/deletions were consistently elevated among exposed subjects. CONCLUSIONS: These findings suggest that PAHs may be associated with specific breast tumor p53 mutation subgroups rather than with overall p53 mutations and may also be related to breast cancer through mechanisms other than p53 mutation. [Mordukhovich, Irina; Gaudet, Mia M.; Conway-Dorsey, Kathleen; Gammon, Marilie D.] Univ N Carolina, Dept Epidemiol, Chapel Hill, NC 27599 USA; [Rossner,

Pavel, Jr.; Santella, Regina; Zhang, Yu-Jing; Long, Chang-Min; Garbowski, Gail; Neugut, Alfred I.] Columbia Univ, Dept Environm Hlth Sci, Mailman Sch Publ Hlth, New York, NY USA; [Rossner, Pavel, Jr.] Acad Sci Czech Republ, Inst Expt Med, Lab Genet Ecotoxicol, Prague, Czech Republic; [Terry, Mary Beth] Columbia Univ, Dept Epidemiol, Mailman Sch Publ Hlth, New York, NY USA; [Hibshoosh, Hanina; Memeo, Lorenzo; Mansukhani, Mahesh; Agrawal, Meenakshi] Columbia Univ, Coll Phys & Surg, Dept Pathol, New York, NY USA; [Memeo, Lorenzo] Mediterranean Inst Oncol, Pathol Unit, Catania, Italy; [Gaudet, Mia M.] Mem Sloan Kettering Canc Ctr, Dept Epidemiol, New York, NY 10021 USA; [Steck, Susan E.] Univ S Carolina, Dept Epidemiol & Biostat, Arnold Sch Publ Hlth, Columbia, SC 29208 USA; [Sagiv, Sharon K.] Harvard Univ, Sch Publ Hlth, Dept Environm Hlth Sci, Boston, MA 02115 USA; [Eng, Sybil M.] Pfizer Inc, Epidemiol Resources Safety Evaluat & Epidemiol, New York, NY USA; [Teitelbaum, Susan L.] Mt Sinai Sch Med, Dept Community & Prevent Med, New York, NY USA; [Neugut, Alfred I.] Columbia Univ, Dept Med, Coll Phys & Surg, New York, NY USA; [Conway-Dorsey, Kathleen] Univ N Carolina, Lineberger Comprehens Canc Ctr, Chapel Hill, NC 27599 USA University of North Carolina; University of North Carolina Chapel Hill; Columbia University; Czech Academy of Sciences; Institute of Experimental Medicine of the Czech Academy of Sciences; Columbia University; Columbia University; Mediterranean Institute of Oncology; Memorial Sloan Kettering Cancer Center; University of South Carolina System; University of South Carolina Columbia; Harvard University; Harvard T.H. Chan School of Public Health; Pfizer; Pfizer USA; Icahn School of Medicine at Mount Sinai; Columbia University; University of North Carolina; University of North Carolina Chapel Hill Mordukhovich, I (corresponding author), Univ N Carolina, Dept Epidemiol, CB 7435 McGavran Greenberg Hall, Chapel Hill, NC 27599 USA. irinam@email.unc.edu Zhang, Yujing/ABB-3590-2021; Steck, Susan/G-5736-2013; Rossner, Pavel/AAI-5789-2020 Terry, Mary Beth/0000-0002-4106-5033; Memeo, Lorenzo/0000-0003-4251-7203; Sagiv, Sharon/0000-0003-2245-1905; Rossner, Pavel/0000-0001-6921-5446 National Cancer Institute [U01 CA/ES66572, P30ES009089, P30ES10126, T32-CA09330]; National Institute of Environmental Health Sciences; U.S. Army [BC972772] National Cancer Institute(United States Department of Health & Human ServicesNational Institutes of Health (NIH) - USANIH National Cancer Institute (NCI)); National Institute of Environmental Health Sciences(United States Department of Health & Human ServicesNational Institutes of Health (NIH) - USANIH National Institute of Environmental Health Sciences (NIEHS)); U.S. Army(United States Department of DefenseUnited States Army) This work was supported in part by grants U01 CA/ES66572, P30ES009089, P30ES10126, and T32-CA09330 from the National Cancer Institute and the National Institute of Environmental Health Sciences; awards from the U.S. Army (BC972772). Breast Cancer Research Foundation and Women at Risk Program; and gifts from private citizens.
63 51 61 1 16 US DEPT HEALTH HUMAN SCIENCES PUBLIC HEALTH SCIENCE RES TRIANGLE PK NATL INST HEALTH, NATL INST ENVIRONMENTAL HEALTH SCIENCES, PO BOX 12233, RES TRIANGLE PK, NC 27709-2233 USA 0091-6765 1552-9924 ENVIRON HEALTH PERSP Environ. Health Perspect. APR 2010 118 4
511 518 10.1289/ehp.0901233
http://dx.doi.org/10.1289/ehp.0901233 8
Environmental Sciences; Public, Environmental & Occupational

Health; Toxicology   Science Citation Index Expanded (SCI-EXPANDED) Environmental Sciences & Ecology; Public, Environmental & Occupational Health; Toxicology   580NA 20064791   Green Published, Green Accepted, gold, Green Submitted   2025-06-24 WOS:000276454800024
J   Elbouzidi, A; Ouassou, H; Aherkou, M; Kharchoufa, L; Meskali, N; Baraich, A; Mechchate, H; Bouhrim, M; Idir, A; Hano, C; Zrouri, H; Addi, M   Elbouzidi, Amine; Ouassou, Hayat; Aherkou, Marouane; Kharchoufa, Loubna; Meskali, Nada; Baraich, Abdellah; Mechchate, Hamza; Bouhrim, Mohamed; Idir, Abderrazak; Hano, Christophe; Zrouri, Hassan; Addi, Mohamed   LC-MS/MS Phytochemical Profiling, Antioxidant Activity, and Cytotoxicity of the Ethanolic Extract of Atriplex halimus L. against Breast Cancer Cell Lines: Computational Studies and Experimental Validation PHARMACEUTICALS   English   Article   Atriplex halimus L.; antioxidant activity; cytotoxicity; breast cancer; computational study; ADMET analysis; toxicity prediction   CARBONIC-ANHYDRASE INHIBITORS; GALLIC ACID; METABOLIZING ENZYMES; ADENOSINE-DEAMINASE; MOLECULAR DOCKING; DRUG TRANSPORTERS; MEDICINAL-PLANTS; OXIDATIVE STRESS; AQUEOUS EXTRACT; IN-SILICO Atriplex halimus L., also known as Mediterranean saltbush, and locally as "Lgtef", an halophytic shrub, is used extensively to treat a wide variety of ailments in Morocco. The present study was undertaken to determine the antioxidant activity and cytotoxicity of the ethanolic extract of A. halimus leaves (AHEE). We first determined the phytochemical composition of AHEE using a liquid chromatography (LC)-tandem mass spectrometry (MS/MS) technique. The antioxidant activity was evaluated using different methods including DPPH scavenging capacity, beta-carotene bleaching assay, ABTS scavenging, iron chelation, and the total antioxidant capacity assays. Cytotoxicity was investigated against human cancer breast cells lines MCF-7 and MDA-MB-231. The results showed that the components of the extract are composed of phenolic acids and flavonoids. The DPPH test showed strong scavenging capacity for the leaf extract (IC50 of 0.36 +/- 0.05 mg/mL) in comparison to ascorbic acid (IC50 of 0.19 +/- 0.02 mg/mL). The beta-carotene test determined an IC50 of 2.91 +/- 0.14 mg/mL. The IC50 values of ABTS, iron chelation, and TAC tests were 44.10 +/- 2.92 TE mu mol/mL, 27.40 +/- 1.46 mg/mL, and 124 +/- 1.27 mu g AAE/mg, respectively. In vitro, the AHE extract showed significant inhibitory activity in all tested tumor cell lines, and the inhibition activity was found in a dose-dependent manner. Furthermore, computational techniques such as molecular docking and ADMET analysis were used in this work. Moreover, the physicochemical parameters related to the compounds' pharmacokinetic indicators were evaluated, including absorption, distribution, metabolism, excretion, and toxicity prediction (Pro-Tox II).   [Elbouzidi, Amine; Meskali, Nada; Addi, Mohamed] Univ Mohammed Premier, Fac Sci, Lab Ameliorat Prod Agr Biotechnol & Environm LAPA, Oujda 60000, Morocco; [Ouassou, Hayat; Kharchoufa, Loubna; Baraich, Abdellah; Bouhrim, Mohamed] Univ Mohammed First, Fac Sci, Blvd Mohamed VI,BP 717, Oujda 60000, Morocco; [Aherkou, Marouane; Idir, Abderrazak] Sultan Moulay Slimane Univ, Fac Sci & Technol, Team Expt Oncol & Nat Subst Cellular & Mol Immuno, Beni Mellal 23000, Morocco; [Mechchate, Hamza] Univ Sidi Mohamed Ben Abdellah USMBA, Fac Sci, BP 1796, Fes 30003, Morocco; [Hano, Christophe] Univ Orleans, Lab Biol Ligneux & Grandes Cultures, INRAE USC1328, F-45067 Orleans 2, France; [Zrouri, Hassan]

Mohammed First Univ, Lab Bioresources Biotechnol Ethnopharmacol & Hlth, Fac Sci, Blvd Mohamed VI,BP 717, Oujda 60000, Morocco
Mohammed First University of Oujda; Mohammed First University of Oujda; Sultan Moulay Slimane University of Beni Mellal; Sidi Mohamed Ben Abdellah University of Fez; Universite de Orleans; INRAE; Mohammed First University of Oujda Addi, M (corresponding author), Univ Mohammed Premier, Fac Sci, Lab Ameliorat Prod Agr Biotechnol & Environm LAPA, Oujda 60000, Morocco.; Hano, C (corresponding author), Univ Orleans, Lab Biol Ligneux & Grandes Cultures, INRAE USC1328, F-45067 Orleans 2, France. hano@univ-orleans.fr; m.addi@ump.ac.ma Meskali, Nada/GVU-0125-2022; bouhrim, mohamed/AAT-4657-2020; IDIR, Abderrazak/AFV-9389-2022; Elbouzidi, Amine/ABD-2051-2020; ADDI, Mohamed/ABE-1723-2021; Hano, Christophe/V-2630-2019 Loubna, Nefla/0009-0002-1945-6641; IDIR, Abderrazak/0000-0002-8258-9466; Elbouzidi, Amine/0000-0003-0314-8484; Baraich, Abdellah/0000-0002-7483-6738; ADDI, Mohamed/0000-0002-3973-6694; BOUHRIM, Mohamed/0000-0001-9944-811X; Hano, Christophe/0000-0001-9938-0151; Meskali, Nada/0000-0002-9377-3453 Moroccan Ministry of Higher Education, Scientific Research and Innovation (Mohamed Premier University, Faculty of Science, Oujda) - Conseil Departemental d'Eure et Loir; Conseil Regional Centre-Val de Loire (program Biomedicaments, EtopoCentre) Moroccan Ministry of Higher Education, Scientific Research and Innovation (Mohamed Premier University, Faculty of Science, Oujda) - Conseil Departemental d'Eure et Loir; Conseil Regional Centre-Val de Loire (program Biomedicaments, EtopoCentre) The main research fund was subsidized by the Moroccan Ministry of Higher Education, Scientific Research and Innovation (Mohamed Premier University, Faculty of Science, Oujda). Moreover, part of this research was fund by Conseil Departemental d'Eure et Loir and Conseil Regional Centre-Val de Loire (program Biomedicaments, EtopoCentre). 139 47 49 0 7 MDPI BASEL ST ALBAN-ANLAGE 66, CH-4052 BASEL, SWITZERLAND 1424-8247 PHARMACEUTICALS-BASE Pharmaceuticals SEP 2022 15 9 1156 10.3390/ph15091156 http://dx.doi.org/10.3390/ph15091156 25 Chemistry, Medicinal; Pharmacology & Pharmacy Science Citation Index Expanded (SCI-EXPANDED) Pharmacology & Pharmacy 4S6SM 36145377 Green Published, gold 2025-06-24 WOS:000857568300001
J Mackey, JR; Ramos-Vazquez, M; Lipatov, O; McCarthy, N; Krasnozhon, D; Semiglazov, V; Manikhas, A; Gelmon, KA; Konecny, GE; Webster, M; Hegg, R; Verma, S; Gorbunova, V; Gerges, DA; Thireau, F; Fung, H; Simms, L; Buyse, M; Ibrahim, A; Martin, M Mackey, John R.; Ramos-Vazquez, Manuel; Lipatov, Oleg; McCarthy, Nicole; Krasnozhon, Dmitriy; Semiglazov, Vladimir; Manikhas, Alexey; Gelmon, Karen A.; Konecny, Gottfried E.; Webster, Marc; Hegg, Roberto; Verma, Sunil; Gorbunova, Vera; Gerges, Dany Abi; Thireau, Francois; Fung, Helena; Simms, Lorinda; Buyse, Marc; Ibrahim, Ayman; Martin, Miguel Primary Results of ROSE/TRIO-12, a Randomized Placebo-Controlled Phase III Trial Evaluating the Addition of Ramucirumab to First-Line Docetaxel Chemotherapy in Metastatic Breast Cancer JOURNAL OF CLINICAL ONCOLOGY English Article ENDOTHELIAL GROWTH-FACTOR; CELL LUNG-CANCER; NEOADJUVANT CHEMOTHERAPY; DOUBLE-BLIND; BEVACIZUMAB; ANGIOGENESIS; THERAPY; VEGFR-1; OVARIAN Purpose Currently,

antiangiogenic strategies in metastatic breast cancer have demonstrated modest improvements in progression-free survival (PFS) but not improved quality or duration of survival, warranting evaluation of new agents in a placebo-controlled setting. Ramucirumab is a human immunoglobulin G1 antibody that binds vascular endothelial growth factor receptor-2 and blocks ligand-stimulated activation. The ROSE/TRIO-012 trial evaluated ramucirumab with docetaxel in unresectable, locally recurrent, or metastatic breast cancer. Patients and Methods In this double-blind, placebo-controlled, randomized, multinational phase III trial, 1,144 patients with human epidermal growth factor receptor 2 (HER2) -negative breast cancer who had not received cytotoxic chemotherapy in the advanced setting were randomly assigned at a two-to-one ratio to receive docetaxel 75 mg/m(2) plus ramucirumab 10 mg/kg or docetaxel 75 mg/antiangiogenic plus placebo once every 3 weeks. Treatment continued until disease progression, unacceptable toxicity, or other withdrawal criteria. Patients were stratified by previous taxane therapy, visceral metastasis, hormone receptor status, and geographic region. An independent data monitoring committee oversaw the trial. The primary end point was investigator-assessed PFS. Results Median PFS in patients treated with ramucirumab plus docetaxel was 9.5 months, compared with 8.2 months in patients who received placebo plus docetaxel (hazard ratio [HR], 0.88; P = .077). Median overall survival was 27.3 months in patients who received ramucirumab plus docetaxel, compared with 27.2 months in patients who received placebo plus docetaxel (HR, 1.01; P = .915). Toxicities seen at significantly higher rates in patients receiving ramucirumab included fatigue, hypertension, febrile neutropenia, palmar-plantar erythrodysesthesia syndrome, and stomatitis. Conclusion Addition of ramucirumab to docetaxel in HER2-negative advanced breast cancer did not meaningfully improve important clinical outcomes. (C) 2014 by American Society of Clinical Oncology [Mackey, John R.] Cross Canc Inst, Edmonton, AB T6G 1Z2, Canada; [Thireau, Francois; Fung, Helena] Translat Res Oncol, Edmonton, AB, Canada; [Buyse, Marc] Tom Baker Canc Clin, Calgary, AB, Canada; [Gelmon, Karen A.] British Columbia Canc Agcy, Vancouver, BC V5Z 4E6, Canada; [Verma, Sunil] Sunnybrook Hlth Sci Ctr, Toronto, ON M4N 3M5, Canada; [Simms, Lorinda] Eli Lilly, Toronto, ON, Canada; [Ramos-Vazquez, Manuel] Ctr Oncol Galicia Jose Antonio Quiroga & Pineiro, La Coruna, Spain; [Martin, Miguel] Univ Complutense, Inst Invest Sanitaria Gregorio Maranon, E-28040 Madrid, Spain; [Lipatov, Oleg] Bashkortostan Republ Minist Hlth, Republican Clin Oncol Dispensary, Ufa, Russia; [Krasnozhon, Dmitriy] Leningrad Reg Oncol Dispensary, St Petersburg, Russia; [Semiglazov, Vladimir] NN Petrov Oncol Res Inst, St Petersburg, Russia; [Manikhas, Alexey] City Clin Oncol Dispensary, St Petersburg, Russia; [Gorbunova, Vera] Russian Acad Med Sci, NN Blokhin Russian Canc Res Ctr, Moscow, Russia; [McCarthy, Nicole] ICON Canc Care Wesley, Brisbane, Qld, Australia; [Konecny, Gottfried E.] Univ Calif Los Angeles, Los Angeles, CA USA; [Hegg, Roberto] Hosp Perola Byigton, Ctr Referencia Saude Mulher, Sao Paulo, Brazil; [Gerges, Dany Abi] Middle East Inst Hlth, Bsalim, Lebanon; [Buyse, Marc] Int Inst Drug Dev, Louvain, Belgium; [Ibrahim, Ayman] ImClone Syst, Bridgewater, NJ USA   University of Alberta; Tom Baker Cancer Clinic; University of Calgary; British Columbia Cancer Agency; University of Toronto; Sunnybrook Research Institute; Sunnybrook Health Science Center; Eli Lilly; Complutense University of

Madrid; N.N. Petrov Research Institute of Oncology; Russian Academy of Medical Sciences; N.N. Blokhin Russian Cancer Research Center; ICON plc; University of California System; University of California Los Angeles; International Drug Development Institute; Eli Lilly; Imclone Systems Inc Mackey, JR (corresponding author), Univ Alberta, Dept Med Oncol, Cross Canc Inst, 11560 Univ Ave, Edmonton, AB T6G 1Z2, Canada.
john.mackey@trioncology.org Martin, Miguel/V-6589-2019; Buyse, Marc/J-4658-2013; Manikhas, Alexey/ABI-7193-2020; Mccarthy, Nicole/R-8527-2019; Lipatov, Oleg/J-3169-2017 Manikhas, Alexey/0000-0001-9616-6635; Hegg, Roberto/0000-0002-6453-8155; McCarthy, Nicole/0000-0001-7394-8909; MARTIN, MIGUEL/0000-0001-9237-3231 Eli Lilly/ImClone Systems Eli Lilly/ImClone Systems
Supported by Eli Lilly/ImClone Systems. 30 91 99
0 19 AMER SOC CLINICAL ONCOLOGY ALEXANDRIA 2318 MILL ROAD, STE 800, ALEXANDRIA, VA 22314 USA 0732-183X 1527-7755
J CLIN ONCOL J. Clin. Oncol. JAN 10 2015 33 2
141 U38
10.1200/JCO.2014.57.1513
http://dx.doi.org/10.1200/JCO.2014.57.1513 9
Oncology Science Citation Index Expanded (SCI-EXPANDED)
Oncology CF2ZX 25185099 Bronze, Green Published
2025-06-24 WOS:000352418100007
J Atilgan, AO; Tepeoglu, M; Haberal, AN; Durukan, E; Kuscu, E; Haberal, M Atilgan, Alev Ok; Tepeoglu, Merih; Haberal, A. Nihan; Durukan, Elif; Kuscu, Esra; Haberal, Mehmet
Papanicolaou Smear Findings in Solid-Organ Transplant Recipients Compared With Normal Subjects According to the Bethesda 2001 System EXPERIMENTAL AND CLINICAL TRANSPLANTATION English Article; Proceedings Paper
14th Congress of the Middle-East-Society-for-Organ-Transplantation / 5th Middle East Transplant Games SEP 10-13, 2014
Istanbul, TURKEY Cervical cancer; Immunosuppression; Kidney transplant; Liver transplant; Pap smear
CERVICAL INTRAEPITHELIAL NEOPLASIA; HUMAN-PAPILLOMAVIRUS; REGRESSION; INFECTION; WOMENObjectives: Solid-organ transplant recipients are at increased risk of developing cancer including cervical cancer compared with woman in the general population, mostly due to long-term immunosuppressive therapy. The Papanicolaou smear remains the primary method of screening cervical pathology including preinvasive and invasive lesions. The objective of this study was to evaluate Pap smear findings in solid-organ transplant recipients, determine the prevalence of abnormal smears, and compare these patients with the general population. Materials and Methods: We retrospectively examined 111 women patients who received liver or kidney transplant between January 1990 to December 2012 at Baskent University Ankara Hospital. Pap smear findings were compared with normal control patients matched for same age and technical procedure of cervical cytology. To selection of control patients, propensity score matching program was performed. All Pap smears were re-examined according to Bethesda 2001 criteria. Results: In 111 transplant patients, 2 patients (1.8%) had atypical squamous cells of undetermined significance, 8 patients (7.2%) had low-grade squamous intraepithelial lesion, 15 patients (13.5%) had Candida infection, 2 patients (1.8%) had Trichomonas vaginalis, 1 patient (0.9%) had herpes simplex infection, 13 patients (11.7%) had bacterial vaginosis, 15 patients (13.5%) had reactive changes due

to inflammation, and 18 patients (16.2%) had atrophy. When we compared our results with the control group, there were statistically significant differences (P <= .05) between the 2 groups in epithelial cell abnormalities (low-grade squamous intraepithelial lesion), Candida infection, bacterial vaginosis, and atrophy. Conclusions: Pap smear screening potentially may help recognize cervical preinvasive and invasive lesions. The risk of developing cervical intraepithelial neoplasia is greater in transplant recipients because of immunosuppressive therapy. The incidence of low-grade squamous intraepithelial lesion was significantly greater in transplant recipients than the general population. Intensive follow-up with Pap smear in transplant recipients is important in the early detection of these lesions.
[Atilgan, Alev Ok; Tepeoglu, Merih; Haberal, A. Nihan] Baskent Univ, Dept Pathol, Fac Med, TR-06490 Ankara, Turkey; [Durukan, Elif] Baskent Univ, Dept Publ Hlth, Fac Med, TR-06490 Ankara, Turkey; [Kuscu, Esra] Baskent Univ, Dept Obstet & Gynecol, Fac Med, TR-06490 Ankara, Turkey; [Haberal, Mehmet] Baskent Univ, Dept Transplantat Surg, Fac Med, TR-06490 Ankara, TurkeyBaskent University; Baskent University; Baskent University; Baskent University Atilgan, AO (corresponding author), Baskent Univ, Dept Pathol, 79 Sokak,7-4, TR-06490 Ankara, Turkey.
potkala@hotmail.com Atılgan, Alev/AAK-3333-2021; Kuşçu, Esra/AAI-8792-2021; durukan, elif/AAJ-8621-2021; Haberal, Mehmet/AAJ-8097-2021; Tepeoglu, Merih/AAK-5222-2021; HABERAL REYHAN, ASUMAN NIHAN/AAK-4587-2021 Ok Atilgan, Alev/0000-0001-8595-8880; durukan, elif/0000-0002-8579-5564; Haberal, Mehmet/0000-0002-3462-7632; Tepeoglu, Merih/0000-0002-9894-8005; HABERAL REYHAN, ASUMAN NIHAN/0000-0001-9852-9911
9 4 4 0 6 BASKENT UNIV ANKARA
TASKENT CADDESI NO 77, KAT 4, BAHCELIEVLER, ANKARA, 06490, TURKEY 1304-0855 EXP CLIN TRANSPLANT Exp. Clin. Transplant. APR 2015 13 1
219 222 10.6002/ect.mesot2014.P18
http://dx.doi.org/10.6002/ect.mesot2014.P18 4
Transplantation Science Citation Index Expanded (SCI-EXPANDED); Conference Proceedings Citation Index - Science (CPCI-S) Transplantation CI8YU 25894158 2025-06-24 WOS:000355058400042
J Pourianazar, NT; Gunduz, U Pourianazar, Negar Taghavi; Gunduz, Ufuk CpG oligodeoxynucleotide-loaded PAMAM dendrimer-coated magnetic nanoparticles promote apoptosis in breast cancer cells BIOMEDICINE & PHARMACOTHERAPY English Article
PAMAM dendrimer; Drug delivery; Flow cytometry; CpG-ODN; Cancer therapy; Targeted therapy TOLL-LIKE-RECEPTORS; HUMAN INTESTINAL EPITHELIUM; IRON-OXIDE NANOPARTICLES; NF-KAPPA-B; HELICOBACTER-PYLORI; DELIVERY; TLR9; EXPRESSION; DNA; OLIGONUCLEOTIDES One major application of nanotechnology in cancer treatment involves designing nanoparticles to deliver drugs, oligonucleotides, and genes to cancer cells. Nanoparticles should be engineered so that they could target and destroy tumor cells with minimal damage to healthy tissues. This research aims to develop an appropriate and efficient nanocarrier, having the ability of interacting with and delivering CpG-oligodeoxynucleotides (CpG-ODNs) to tumor cells. CpG-ODNs activate Toll-like receptor 9 (TLR9), which can generate a signal cascade for cell death. In our study, we utilized three-layer magnetic

nanoparticles composed of a Fe3O4 magnetic core, an aminosilane (APTS) interlayer and a cationic poly(amidoamine) (PAMAM) dendrimer. This will be a novel targeted delivery system to enhance the accumulation of CpG-ODN molecules in tumor cells. The validation of CpG-ODN binding to DcMNPs was performed using agarose gel electrophoresis, UV-spectrophotometer, XPS analyses. Cytotoxicity of conjugates was assessed in MDA-MB231 and SKBR3 cancer cells based on cell viability by XTT assay and flow cytometric analysis. Our results indicated that the synthesized DcMNPs having high positive charges on their surface could attach to CpG-ODN molecules via electrostatic means. These nanoparticles with the average sizes of 40 +/- 10 nm bind to CpG-ODN molecules efficiently and induce cell death in MDA-MB231 and SKBR3 tumor cells and could be considered a suitable targeted delivery system for CpG-ODN in biomedical applications. The magnetic core of these nanoparticles represents a promising option for selective drug targeting as they can be concentrated and held in position by means of an external magnetic field. (C) 2016 Elsevier Masson SAS. All rights reserved. [Pourianazar, Negar Taghavi; Gunduz, Ufuk] Middle E Tech Univ, Dept Biotechnol, TR-06800 Ankara, Turkey; [Gunduz, Ufuk] Middle E Tech Univ, Dept Biol Sci, TR-06800 Ankara, Turkey Middle East Technical University; Middle East Technical University Pourianazar, NT; Gunduz, U (corresponding author), Middle E Tech Univ, Dept Biol Sci, TR-06800 Ankara, Turkey. Negar_taghavi22@yahoo.com; ufukg@metu.edu.tr Taghavi Pourianazar, Negar/IZP-9751-2023 Scientific and Technical Research Council of Turkey (TUBITAK) [2215, 1002]; Middle East Technical University [BAP-07-02-2012-71] Scientific and Technical Research Council of Turkey (TUBITAK)(Turkiye Bilimsel ve Teknolojik Arastirma Kurumu (TUBITAK)); Middle East Technical University(Middle East Technical University) We appreciate Prof. Dr. Inci Eroglu for letting us to use her laboratory during some parts of the synthesis work. This work is supported by the Scientific and Technical Research Council of Turkey (TUBITAK-grant 2215 and 1002) and Middle East Technical University (BAP-07-02-2012-71). 49 29 31 0 70 ELSEVIER FRANCE-EDITIONS SCIENTIFIQUES MEDICALES ELSEVIER PARIS 23 RUE LINOIS, 75724 PARIS, FRANCE 0753-3322 1950-6007 BIOMED PHARMACOTHER Biomed. Pharmacother. MAR 2016 78
81 91
10.1016/j.biopha.2016.01.002
http://dx.doi.org/10.1016/j.biopha.2016.01.002
11 Medicine, Research & Experimental; Pharmacology & Pharmacy Science Citation Index Expanded (SCI-EXPANDED)
Research & Experimental Medicine; Pharmacology & Pharmacy
DE3MZ 26898428 2025-06-24
WOS:000370535300011
J Warleta, F; Quesada, CS; Campos, M; Allouche, Y; Beltrán, G; Gaforio, JJ Warleta, Fernando; Sanchez Quesada, Cristina; Campos, Maria; Allouche, Yosra; Beltran, Gabriel; Gaforio, Jose J. Hydroxytyrosol Protects against Oxidative DNA Damage in Human Breast Cells NUTRIENTS
English Article breast cancer; Mediterranean diet; olive oil minor compounds; hydroxytyrosol; tyrosol; phenols; oxidative stress; reactive oxygen species; DNA damage OLIVE OIL PHENOLICS; ANTIOXIDANT PROPERTIES; EPITHELIAL-CELLS; CANCER CELLS; CARCINOGENESIS; TYROSOL; STRESS; HEALTH; LINES; ASSAY Over recent years, several

studies have related olive oil ingestion to a low incidence of several diseases, including breast cancer. Hydroxytyrosol and tyrosol are two of the major phenols present in virgin olive oils. Despite the fact that they have been linked to cancer prevention, there is no evidence that clarifies their effect in human breast tumor and non-tumor cells. In the present work, we present hydroxytyrosol and tyrosol's effects in human breast cell lines. Our results show that hydroxytyrosol acts as a more efficient free radical scavenger than tyrosol, but both fail to affect cell proliferation rates, cell cycle profile or cell apoptosis in human mammary epithelial cells (MCF10A) or breast cancer cells (MDA-MB-231 and MCF7). We found that hydroxytyrosol decreases the intracellular reactive oxygen species (ROS) level in MCF10A cells but not in MCF7 or MDA-MB-231 cells while very high amounts of tyrosol is needed to decrease the ROS level in MCF10A cells. Interestingly, hydroxytyrosol prevents oxidative DNA damage in the three breast cell lines. Therefore, our data suggest that simple phenol hydroxytyrosol could contribute to a lower incidence of breast cancer in populations that consume virgin olive oil due to its antioxidant activity and its protection against oxidative DNA damage in mammary cells. [Warleta, Fernando; Sanchez Quesada, Cristina; Campos, Maria; Allouche, Yosra; Gaforio, Jose J.] Univ Jaen, Div Immunol, Dept Hlth Sci, Fac Expt Sci, Jaen 23071, Spain; [Allouche, Yosra; Beltran, Gabriel] Ctr Venta Llano, Pesquera Prod Ecol IFAPA, Inst Andaluz Invest & Formac Agr, Mengibar 23620, Spain Universidad de Jaen Gaforio, JJ (corresponding author), Univ Jaen, Div Immunol, Dept Hlth Sci, Fac Expt Sci, Campus Lagunillas S-N, Jaen 23071, Spain. fwarleta@ujaen.es; csquesad@ujaen.es; mcampos@ujaen.es; yosraallouche@yahoo.fr; gabriel.beltran@juntadeandalucia.es; jgaforio@ujaen.es Santos e Campos, MariaAparecida/C-4555-2019; Sanchez, Cristina/ABC-5511-2021; Beltran, Gabriel/D-1372-2016 Sanchez-Quesada, Cristina/0000-0003-2997-8515; Gaforio, Jose J/0000-0003-2996-9301; Warleta, Fernando/0000-0002-7365-7367 Instituto Andaluz de Biotecnologia [BIOANDALUS 08/22/L5.3]; Ministerio de Ciencia e Innovacion [RTA2008-00066-C03-03]; "Centro de Excelencia en Investigacion sobre Aceite de Oliva y Salud" (CEAS) Instituto Andaluz de Biotecnologia; Ministerio de Ciencia e Innovacion(Spanish GovernmentInstituto de Salud Carlos III); "Centro de Excelencia en Investigacion sobre Aceite de Oliva y Salud" (CEAS) This study was supported by the "Instituto Andaluz de Biotecnologia" (BIOANDALUS 08/22/L5.3); Ministerio de Ciencia e Innovacion-FEDER (RTA2008-00066-C03-03); and "Centro de Excelencia en Investigacion sobre Aceite de Oliva y Salud" (CEAS).
33 115 121 1 35 MDPI AG BASEL POSTFACH, CH-4005 BASEL, SWITZERLAND 2072-6643 NUTRIENTS
Nutrients OCT 2011 3 10 839
857 10.3390/nu3100839
http://dx.doi.org/10.3390/nu3100839 19
Nutrition & Dietetics Science Citation Index Expanded (SCI-EXPANDED) Nutrition & Dietetics 864OD 22254082 Green Published, Green Submitted, gold 2025-06-24
WOS:000298248600001
J Kleckner, AS; Reschke, JE; Kleckner, IR; Magnuson, A; Amitrano, AM; Culakova, E; Shayne, M; Netherby-Winslow, CS; Czap, S; Janelsins, MC; Mustian, KM; Peppone, LJ
Kleckner, Amber S.; Reschke, Jennifer E.; Kleckner, Ian R.; Magnuson, Allison; Amitrano, Andrea M.; Culakova, Eva; Shayne,

Michelle; Netherby-Winslow, Colleen S.; Czap, Susan; Janelsins, Michelle C.; Mustian, Karen M.; Peppone, Luke J. The Effects of a Mediterranean Diet Intervention on Cancer-Related Fatigue for Patients Undergoing Chemotherapy: A Pilot Randomized Controlled Trial CANCERS English Article oncology; nutrition; supportive care; integrative oncology; metabolism; mitochondria ANDROGEN DEPRIVATION THERAPY; QUALITY-OF-LIFE; PROSTATE-CANCER; GENE-EXPRESSION; NUTRITION; RISK; MEN; MITOCHONDRIA; PREVALENCE; OUTCOMES Simple Summary Cancer-related fatigue affects the majority of people undergoing chemotherapy for cancer. The Mediterranean Diet provides healthy macro- and micronutrients that promote energy production and counter known mechanisms that contribute to fatigue. Thus, we designed a Mediterranean Diet program specifically for patients undergoing chemotherapy that included food provision, education, a cookbook, and weekly telephone check-ins. In a two-arm randomized controlled trial (n = 33), we found that our program was safe and feasible; there was excellent adherence (>70%) to the Mediterranean Diet. The Mediterranean Diet program led to less fatigue to a small-moderate degree at weeks 4 and 8. For those with a lower Mediterranean Diet score before the program, the program had a larger effect. Mitochondria are the cellular organelles that produce ATP energy and, in circulating T cells, fatigue was associated with mitochondrial dysfunction. These data support larger studies testing how and how much a Mediterranean Diet during chemotherapy can alleviate fatigue. Cancer-related fatigue is a common, burdensome symptom of cancer and a side-effect of chemotherapy. While a Mediterranean Diet (MedDiet) promotes energy metabolism and overall health, its effects on cancer-related fatigue remain unknown. In a randomized controlled trial, we evaluated a rigorous MedDiet intervention for feasibility and safety as well as preliminary effects on cancer-related fatigue and metabolism compared to usual care. Participants had stage I-III cancer and at least six weeks of chemotherapy scheduled. After baseline assessments, randomization occurred 2:1, MedDiet:usual care. Measures were collected at baseline, week 4, and week 8 including MedDiet adherence (score 0-14), dietary intake, and blood-based metabolic measures. Mitochondrial respiration from freshly isolated T cells was measured at baseline and four weeks. Participants (n = 33) were 51.0 +/- 14.6 years old, 94% were female, and 91% were being treated for breast cancer. The study was feasible, with 100% completing the study and >70% increasing their MedDiet adherence at four and eight weeks compared to baseline. Overall, the MedDiet intervention vs. usual care had a small-moderate effect on change in fatigue at weeks 4 and 8 (ES = 0.31, 0.25, respectively). For those with a baseline MedDiet score <5 (n = 21), the MedDiet intervention had a moderate-large effect of 0.67 and 0.48 at weeks 4 and 8, respectively. The MedDiet did not affect blood-based lipids, though it had a beneficial effect on fructosamine (ES = -0.55). Fatigue was associated with mitochondrial dysfunction including lower basal respiration, maximal respiration, and spare capacity (p < 0.05 for FACIT-F fatigue subscale and BFI, usual fatigue). In conclusion, the MedDiet was feasible and attenuated cancer-related fatigue among patients undergoing chemotherapy, especially those with lower MedDiet scores at baseline. [Kleckner, Amber S.; Kleckner, Ian R.] Univ Maryland, Dept Pain & Translat Symptom Sci, Sch Nursing,

Baltimore, MD 21201 USA; [Kleckner, Amber S.; Kleckner, Ian R.] Univ Maryland, Greenebaum Comprehens Canc Ctr, Baltimore, MD 21201 USA; [Reschke, Jennifer E.; Culakova, Eva; Netherby-Winslow, Colleen S.; Janelsins, Michelle C.; Mustian, Karen M.; Peppone, Luke J.] Univ Rochester, Dept Surg, Div Support Care Canc, Med Ctr, Rochester, NY 14642 USA; [Reschke, Jennifer E.; Magnuson, Allison; Culakova, Eva; Shayne, Michelle; Czap, Susan; Janelsins, Michelle C.; Mustian, Karen M.; Peppone, Luke J.] Wilmot Canc Inst, Rochester, NY 14642 USA; [Magnuson, Allison; Shayne, Michelle] Univ Rochester, Dept Med, Med Ctr, Rochester, NY 14642 USA; [Amitrano, Andrea M.] Univ Rochester, Dept Pathol, Med Ctr, Rochester, NY 14642 USA University System of Maryland; University of Maryland Baltimore; University System of Maryland; University of Maryland Baltimore; University of Rochester; University of Rochester; University of Rochester; University of Rochester Kleckner, AS (corresponding author), Univ Maryland, Dept Pain & Translat Symptom Sci, Sch Nursing, Baltimore, MD 21201 USA.; Kleckner, AS (corresponding author), Univ Maryland, Greenebaum Comprehens Canc Ctr, Baltimore, MD 21201 USA.

amber.kleckner@umaryland.edu Amitrano, Andrea/0000-0002-1101-872X; Kleckner, Amber/0000-0002-5088-1139; Kleckner, Ian/0000-0002-9828-9986; Netherby, Colleen/0000-0001-5676-768X

National Institutes of Health (NIH) National Cancer Institute (NCI) [UG1CA189961, T32CA102618, K07CA221931]; Maryland Department of Health's Cigarette Restitution Fund Program; Transdisciplinary Research and Energetics in Cancer (TREC) [NIH NCI R25CA203650]; National Cancer Institute [UG1CA189961, K07CA221931] Funding Source: NIH RePORTER National Institutes of Health (NIH) National Cancer Institute (NCI)(United States Department of Health & Human ServicesNational Institutes of Health (NIH) - USANIH National Cancer Institute (NCI)); Maryland Department of Health's Cigarette Restitution Fund Program; Transdisciplinary Research and Energetics in Cancer (TREC); National Cancer Institute(United States Department of Health & Human ServicesNational Institutes of Health (NIH) - USANIH National Cancer Institute (NCI)) This work was supported by the National Institutes of Health (NIH) National Cancer Institute (NCI) under grant numbers UG1CA189961 to K.M.M. and Gary Morrow, T32CA102618 to M.C.J. and Gary Morrow, and K07CA221931 to I.R.K. Helpful discussions regarding the results of this project occurred at the Transdisciplinary Research and Energetics in Cancer (TREC) Training Workshop in June 2022 (NIH NCI R25CA203650 to Melinda Irwin). This publication was supported by funds through the Maryland Department of Health's Cigarette Restitution Fund Program. 72 21 22 0 15 MDPI BASEL ST ALBAN-ANLAGE 66, CH-4052 BASEL, SWITZERLAND 2072-6694

CANCERS Cancers SEP 2022 14 17

4202 10.3390/cancers14174202

http://dx.doi.org/10.3390/cancers14174202 18

Oncology Science Citation Index Expanded (SCI-EXPANDED)

Oncology 4J1DD 36077737 gold, Green Published, Green Submitted 2025-06-24 WOS:000851010100001

J Freij, MA; Saleh, HH; Farsakh, HAA; Khadra, MM; Ijmail, AA; Rahall, BO; Waldali, MH; Najeeb, NS; Tahtamouni, LH

Freij, M. A.; Saleh, H. H.; Farsakh, H. A. A.; Khadra, M. M.; Ijmail, A. A.; Rahall, B. O.; Waldali, M. H.; Najeeb, N. S.; Tahtamouni, L. H. Type-specific prevalence of human papillomavirus among women with abnormal cytology in Jordan

EUROPEAN JOURNAL OF GYNAECOLOGICAL ONCOLOGY
English Article HPV; Pap smear; Cervical cancer; Vaccination; Jordan CERVICAL INTRAEPITHELIAL NEOPLASIA; EXTENDED MIDDLE-EAST; MOLECULAR EPIDEMIOLOGY; MULTIPLE TYPES; CANCER; INFECTION; RISK; GENOTYPE; HPV; CARCINOMA Background: Infection with human papillomavirus (HPV) is the most common sexually transmitted infection in women worldwide. Infection with high-risk HPV (HR-HPV) is the main cause of cervical cancer. However, little is known about its prevalence in the female population in Jordan. The aim of this study was to discover HPV type-specific prevalence in women living in Jordan. Data regarding HPV type-specific prevalence for a particular country are crucial for providing baseline information to estimate the effectiveness of implementing HPV-based cervical cancer prevention. Materials and Methods: Between 2008 and 2014, cervical epithelium samples were collected by conventional Papanicolaou (Pap) smear; all abnormal smears had reflex HPV testing from apparently healthy and non-healthy women. HPV prevalence and its genotype distribution were examined in these smears. DNA samples were extracted and HPV genotyping was performed. The results were retrospectively analyzed. Results: A total of 209 abnormal Pap smears were identified and reflex HPV testing was performed on these cases; 153 (73.2%) of the tested cases were HPV positive. The mean age of the women included was 38.3 +/- 10.2 (range: 20-70) years and the mean age of HPV-positive women was 38.5 +/- 10.3 (range: 21-70) years. There was a significant increase in the incidence of HPV infection over the study period. HPV 6 and 11 infections were very common in the HPV positive women; 38.6% and 47%, respectively. HPV 16 was the single most common (57.5%) HPV infection detected. HPV 30's (31, 33, 34, 35, and 39) as a group were the most common HPV infections recorded at 59.5%. HPV 50's (51, 52, 53, 56, 58, and 59) were detected in 56.9% of the cases. HPV type 68 was recorded in one case (0.6%). Of notice, the vast majority of cases had co-infection of multiple genotypes (89%) and only 11% were single genotype infection. Conclusion: The results of the study showed a relatively high prevalence (73.2%) of HPV infection and a higher incidence of co-infection with multiple high risk genotypes (89%) in comparison with other studies from the present region. This study suggests that there is sufficient evidence to warrant further population-based studies and further interventions. HPV vaccination should be considered for Jordanian girls as a preventative measure. [Freij, M. A.; Khadra, M. M.; Rahall, B. O.] Univ Jordan, Dept Obstet & Gynecol, Fac Med, Queen Rania St, Amman 11942, Jordan; [Saleh, H. H.] Elite Moms Clin, Amman, Jordan; [Farsakh, H. A. A.; Ijmail, A. A.] First Med Labs, Amman, Jordan; [Waldali, M. H.] Specialty Hosp, Obstet & Gynecol Dept, Amman, Jordan; [Najeeb, N. S.; Tahtamouni, L. H.] Hashemite Univ, Fac Sci, Dept Biol & Biotechnol, Zarqa, Jordan
University of Jordan; Hashemite University Freij, MA (corresponding author), Univ Jordan, Dept Obstet & Gynecol, Fac Med, Queen Rania St, Amman 11942, Jordan. mazen2k@yahoo.com
Tahtamouni, Lubna/AAT-4121-2020; Khadra, Maysa/M-5399-2017
khadra, Maysa/0000-0003-3042-5686; ABDALLAH, NISSREEN/0000-0002-0765-8230 27 3 3 0 1
I R O G CANADA, INC MONTREAL 4900 COTE ST-LUC, APT#212, MONTREAL, QUEBEC H3W 2H3, CANADA 0392-2936 EUR J GYNAECOL ONCOL Eur. J. Gynaecol. Oncol. 2017 38 6
901 904

10.12892/ejgo3750.2017
http://dx.doi.org/10.12892/ejgo3750.2017 4
Oncology; Obstetrics & Gynecology Science Citation Index Expanded (SCI-EXPANDED) Oncology; Obstetrics & Gynecology
GF0LB 2025-06-24 WOS:000431620900015
J Gok, S; Kuzmenko, O; Babinskyi, A; Severcan, F
Gok, Seher; Kuzmenko, Oleksandr; Babinskyi, Andrii; Severcan, Feride Vitamin E Derivative with Modified Side Chain Induced Apoptosis by Modulating the Cellular Lipids and Membrane Dynamics in MCF7 Cells CELL BIOCHEMISTRY AND BIOPHYSICS English Article
Vitamin E derivative; Tocopherol; Lipid metabolism; Breast cancer; Spectroscopy ALPHA-TOCOPHERYL SUCCINATE; APOLIPOPROTEIN-E GENOTYPE; BREAST-CANCER CELLS; E ANALOGS; BIOCHEMICAL-COMPOSITION; TUMOR BURDEN; MODEL; METABOLISM; PROSTATE; FLUIDITY
The vitamin E derivative with side chain modification (TC6OAc) has been shown to possess anticancer activity in our earlier in vivo studies. It was hypothesized that, as Vitamin E (VE) and VE derivative are fat soluble lipophilic molecules, they exert their function by modulating the lipid metabolism and related pathways. This study aimed to evaluate the cellular impact of this VE derivative (2,5,7,8-Tetramethyl-2-(4 '-Methyl-3 '-Pentenyl)-6-Acetoxy Chromane-TC6OH), using alpha-tocopherol as a reference compound throughout the experiments. Their effects on the cellular metabolism, the biophysical properties of cellular lipids and the functional characteristics of cells were monitored in human estrogen receptor (ER) positive breast cancer cells. It has been documented that TC6OH treatment induces tumor cell apoptosis by dissipating the mitochondrial membrane potential, modulating the lipid, transportation and degradation as well as downregulating certain anti-apoptotic and growth factor related proteins. Due to resistance of ER positive cells to the established therapies, the findings of this study are of translational value. [Gok, Seher] Sci & Technol Res Council Turkey, Ankara, Turkey; [Kuzmenko, Oleksandr; Babinskyi, Andrii] Palladin Inst Biochem, Dept Vitamins & Coenzymes Biochem, Kiev, Ukraine; [Severcan, Feride] Altinbas Univ, Fac Med, Dept Biophys, Istanbul, Turkey; [Severcan, Feride] Middle East Tech Univ, Dept Biol Sci, Ankara, Turkey Turkiye Bilimsel ve Teknolojik Arastirma Kurumu (TUBITAK); National Academy of Sciences Ukraine; Palladin Institute of Biochemistry of NASU; Altinbas University; Middle East Technical University Severcan, F (corresponding author), Altinbas Univ, Fac Med, Dept Biophys, Istanbul, Turkey.; Severcan, F (corresponding author), Middle East Tech Univ, Dept Biol Sci, Ankara, Turkey. feride.severcan@altinbas.edu.tr
Kuzmenko, Oleksandr/P-6059-2016 Severcan, Feride/0000-0002-1717-2517 Scientific and Technological Research Council of Turkey (TUBITAK) [114Z424]; National Academy of Sciences of Ukraine Scientific and Technological Research Council of Turkey (TUBITAK)(Turkiye Bilimsel ve Teknolojik Arastirma Kurumu (TUBITAK)); National Academy of Sciences of Ukraine This work was supported by the Scientific and Technological Research Council of Turkey (TUBITAK) (Project No. 114Z424) and the National Academy of Sciences of Ukraine for this joint research project. 62
5 5 1 9 HUMANA PRESS INC TOTOWA 999 RIVERVIEW DRIVE SUITE 208, TOTOWA, NJ 07512 USA 1085-9195
1559-0283 CELL BIOCHEM BIOPHYS Cell Biochem. Biophys. JUN 2021 79 2 271 287

10.1007/s12013-020-00961-y
http://dx.doi.org/10.1007/s12013-020-00961-y JAN 2021
17 Biochemistry & Molecular Biology; Biophysics; Cell Biology Science Citation Index Expanded (SCI-EXPANDED)
Biochemistry & Molecular Biology; Biophysics; Cell Biology
SA1IJ 33442824 2025-06-24
WOS:000607489300002
J Shtaiwi, A; Adnan, R; Khairuddean, M; Khan, SU
Shtaiwi, Amneh; Adnan, Rohana; Khairuddean, Melati; Khan, Shafi Ullah Computational investigations of the binding mechanism of novel benzophenone imine inhibitors for the treatment of breast cancer RSC ADVANCES
English Article
ESTROGEN-RECEPTOR-ALPHA; MOLECULAR-DYNAMICS SIMULATIONS; SIDE-CHAIN; ACTIVATION; MODULATORS; LIGANDS; AFFINITY; DESIGN; ANTIESTROGENS; OPTIMIZATION 4-Hydroxytamoxifen (4-OHT), the most common hormone used for the treatment of breast cancer, is a selective estrogen receptor modulator (SERM) inhibitor that acts as an antagonist in breast tissue and a partial agonist in the endometrium. However, the detailed molecular mechanism of 4-OHT structure modification has not been well investigated to date. Herein, molecular docking, molecular dynamics simulations and free energy calculations were performed to explore the mechanisms of the molecular interactions between newly designed benzophenone imines (BIs) and the three forms apo, antagonist and agonist of the human estrogen receptor hER alpha. The proposed inhibitors were designed by replacing the triarylethylene estrogenic scaffold found in 4-OHT with Schiff base triarylimine derivatives. The antiestrogen scaffold i.e. the O-alkyl side chain in 4-OHT was developed by incorporating an alanine amino acid side chain functionality into the triarylimine scaffold. Docking results reveal that the newly designed BIs bind to the hydrophobic open pocket of the apo and antagonist hER alpha conformations with higher affinity as compared to the natural and synthetic estrogen estradiol (E2) and 4-OHT. The analysis of the molecular dynamics simulation results based on six different systems of the best docked BI (5c) with hER alpha receptors demonstrates stable interactions, and the complex undergoes fewer conformational fluctuations in the open apo/antagonist hER alpha receptors as compared to the case of the closed agonist. In addition, the calculated binding free energies indicate that the main factor that contributes to the stabilization of the receptor-inhibitor complexes is hydrophobic interactions. This study suggests that the development of these Schiff base derivatives may be worth exploring for the preparation of new 4-OHT analogues. [Shtaiwi, Amneh] Middle East Univ, Sch Pharm, Queen Alia Airport St, Amman 11118, Jordan; [Shtaiwi, Amneh; Adnan, Rohana; Khairuddean, Melati] Univ Sains Malaysia, Sch Chem Sci, George Town 11800, Malaysia; [Khan, Shafi Ullah] Monash Univ Malaysia, Sch Pharm, Jalan Lagoon Selatan, Bandar Sunway 47500, Subang Jaya, Malaysia
Middle East University; Universiti Sains Malaysia; Monash University; Monash University Malaysia Adnan, R (corresponding author), Univ Sains Malaysia, Sch Chem Sci, George Town 11800, Malaysia. ashtaiwi@meu.edu.jo; r_adnan@usm.my; shafiullahpharmD@gmail.com Khan, Shafi Ullah/AFU-4868-2022; Adnan, Rohana/E-5342-2012; Shtaiwi, Amneh/P-7844-2018 Shtaiwi, Amneh/0000-0001-6081-7440; Adnan, Rohana/0000-0002-7628-7656
Universiti Sains Malaysia through the USM Fellowship Scheme

under the Institute of Postgraduate Studies; Ministry of Higher Education through FRGS [203/PKIMIA/6711558] Universiti Sains Malaysia through the USM Fellowship Scheme under the Institute of Postgraduate Studies; Ministry of Higher Education through FRGS
The authors gratefully acknowledge the financial support received from the Universiti Sains Malaysia through the USM Fellowship Scheme under the Institute of Postgraduate Studies and Ministry of Higher Education through FRGS Grant No. 203/PKIMIA/6711558. Khan thanks OpenEye Scientific Software, Inc., for a free academic license of the OpenEye Toolkits. 69
15 15 1 4 ROYAL SOC CHEMISTRY CAMBRIDGE
THOMAS GRAHAM HOUSE, SCIENCE PARK, MILTON RD, CAMBRIDGE CB4 0WF, CAMBS, ENGLAND 2046-2069 RSC ADV RSC Adv.
NOV 3 2019 9 61 35401 35416
10.1039/c9ra04759j http://dx.doi.org/10.1039/c9ra04759j
16 Chemistry, MultidisciplinaryScience Citation Index Expanded (SCI-EXPANDED) Chemistry JQ3PM 35541022
gold, Green Published 2025-06-24
WOS:000498861100010
J Etteieb, S; Kawachi, A; Han, J; Tarhouni, J; Isoda, H
Etteieb, Selma; Kawachi, Atsushi; Han, Junkyu; Tarhouni, Jamila; Isoda, Hiroko Estrogenic Activity Assessment in Environmental Matrices by Means of In Vitro Cell-Based Bioassays Coupled With Molecular Investigation CLEAN-SOIL AIR WATER English Article
Biomarkers; Endocrine disrupting compounds; MCF-7 breast cancer cells; Modified E-screen assay WASTE-WATER TREATMENT; HUMAN BREAST-CANCER; C-JUN; TREATMENT-PLANT; ER-ALPHA; RIVER; RECEPTOR; OVEREXPRESSION; PROLIFERATION; ACTIVATION
Pollution of river systems in Tunisia by endocrine disrupting compounds (EDCs) is due to anthropogenic impacts through non-treated industrial wastewater and municipal-treated wastewater discharge. The aim of this study was to assess the estrogenic activity of environmental matrices involving raw industrial wastewater (WW), surface water (SW), and treated wastewater (TWW). A modified E-screen assay was used to evaluate estrogenic activity of the samples in MCF-7 breast cancer cells. Western blotting and real-time PCR molecular approaches were applied to investigate the mechanism of EDC-induced MCF-7 breast cancer cell proliferation which interferes with estrogen receptor alpha (ER alpha) and c-Jun. Our results revealed a dose-dependent estrogenic activity reaching an optimal level at a concentration of 0.01% for industrial WW and SW and 10% for TWW. Monitoring ER alpha protein and c-Jun gene expression levels after 1, 6, 24, and 48 h of treatment showed a correlation in the overexpression of ER alpha and c-Jun most of the time. In fact, ER alpha and c-Jun correlated up-regulation began after 1 h of treatment for industrial WW, TWW, and one SW sample. For the other SW samples, this correlated up-regulation started after 6-24 h of treatment. Results emphasized the estrogen-like effect of our contaminated samples on MCF-7 cells which facilitate a crosstalk between ER alpha and c-Jun factor, thereby directly regulating the expression of estrogen-induced genes to mediate breast cancer cell growth.
[Etteieb, Selma; Tarhouni, Jamila] Natl Agron Inst Tunisia INAT, Lab Water Sci & Technol LSTE, Tunis 1082, Tunisia; [Kawachi, Atsushi; Han, Junkyu; Isoda, Hiroko] Univ Tsukuba, Grad Sch Life & Environm Sci, Tsukuba, Ibaraki, Japan; [Kawachi, Atsushi; Han, Junkyu; Isoda, Hiroko] Univ Tsukuba, Alliance Res North Africa

ARENA, Tsukuba, Ibaraki, Japan   Universite de Carthage; University of Tsukuba; University of Tsukuba Etteieb, S (corresponding author), Natl Agron Inst Tunisia INAT, 43 Charles Nicolle St,Menzeh 1, Tunis 1082, Tunisia.   selmaetteieb@yahoo.fr
Isoda, Hiroko/0000-0002-1399-9541 DAAD; SATREPS project for Valorization of Bio-resources in Semi-arid and Arid Land for Regional Development   DAAD(Deutscher Akademischer Austausch Dienst (DAAD)); SATREPS project for Valorization of Bio-resources in Semi-arid and Arid Land for Regional Development   The authors would like to thank DAAD for their financial support to participate and present the current work in the symposium of "Emerging Pollutants in Irrigation Waters: Origins, Fate, Risks and Mitigation" in Tunisia. This research was supported by funds from the SATREPS project for Valorization of Bio-resources in Semi-arid and Arid Land for Regional Development.   57   0
0   0   13   WILEY HOBOKEN   111 RIVER ST, HOBOKEN 07030-5774, NJ USA   1863-0650   1863-0669   CLEAN-SOIL AIR WATER Clean-Soil Air Water   SEP   2015   43   9   SI
1279   1288   10.1002/clen.201400468
http://dx.doi.org/10.1002/clen.201400468   10
Green & Sustainable Science & Technology; Environmental Sciences; Marine & Freshwater Biology; Water Resources   Science Citation Index Expanded (SCI-EXPANDED)   Science & Technology - Other Topics; Environmental Sciences & Ecology; Marine & Freshwater Biology; Water Resources   CT5JB
2025-06-24   WOS:000362843800003
J   Harvey, BI; Youngblood, SM; Kleckner, AS
Harvey, Brianna I.; Youngblood, Shari M.; Kleckner, Amber S.
Barriers and Facilitators to Adherence to a Mediterranean Diet Intervention during Chemotherapy Treatment: A Qualitative Analysis   NUTRITION AND CANCER-AN INTERNATIONAL JOURNAL   English   Article
CANCER-RELATED FATIGUE; CONTROLLED-TRIAL; NUTRITION; BREAST; HEALTH; GUIDELINES; DIAGNOSIS; IMPACT Patients undergoing chemotherapy are at risk for malnutrition and a high symptom burden, and nutritional interventions can address clinical and supportive care outcomes. Herein, we identified barriers and facilitators to adhering to a Mediterranean Diet (MedDiet) intervention during chemotherapy. Patients with cancer (any type) who were undergoing chemotherapy were enrolled into a clinical trial testing the effects of an 8-week MedDiet intervention on cancer-related fatigue. Participants were randomized 2:1, MedDiet:control. The intervention entailed food provision, education, a cookbook, a session with a nutritionist, and weekly check-ins. Post-intervention, all participants completed semi-structured exit interviews. The interviews were transcribed and open coding was conducted to describe the facilitators and barriers to MedDiet adherence. Participants (n = 29, n = 21 in the intervention group) were 51.0 +/- 15.1 years old and 93.1% had breast cancer. Educational materials and convenient food delivery were the highest reported facilitators. Many patients offered that changing their diet gave a sense of control and empowerment. Barriers to adherence were that the frozen food was unappetizing, participants' (or their spouse/children's) food preferences did not align with the MedDiet, and chemotherapy-induced side effects that prevented food consumption (eg, mouth sores, lack of appetite). This project helps understand the patient experience within nutritional interventions to optimize dietary programs

during chemotherapy treatment. [Harvey, Brianna I.; Youngblood, Shari M.; Kleckner, Amber S.] Univ Maryland, Dept Pain & Translat Symptom Sci, Sch Nursing, Baltimore, MD USA; [Harvey, Brianna I.] Indiana Univ Sch Med, Terre Haute, IN USA; [Youngblood, Shari M.] Saybrook Univ, Pasadena, CA USA; [Kleckner, Amber S.] Greenebaum Comprehens Canc Ctr, Baltimore, MD USA; [Kleckner, Amber S.] Univ Maryland, Dept Pain & Translat Symptom Sci, Sch Nursing, Baltimore, MD 20742 USA University System of Maryland; University of Maryland Baltimore; Indiana University System; University System of Maryland; University of Maryland Baltimore
Kleckner, AS (corresponding author), Univ Maryland, Dept Pain & Translat Symptom Sci, Sch Nursing, Baltimore, MD 20742 USA.
amber.kleckner@umaryland.edu Kleckner, Amber/0000-0002-5088-1139; Youngblood, Shari/0000-0003-3037-7821; Harvey, Brianna/0000-0003-3460-2627 National Institutes of Health (NIH) National Cancer Institute (NCI) [UG1CA189961, T32CA102618]; National Institute of Diabetes and Digestive and Kidney Diseases (NIDDK) [T35DK095737]; Maryland Department of Health's Cigarette Restitution Fund Program [CH-649-CRF] National Institutes of Health (NIH) National Cancer Institute (NCI)(United States Department of Health & Human ServicesNational Institutes of Health (NIH) - USANIH National Cancer Institute (NCI)); National Institute of Diabetes and Digestive and Kidney Diseases (NIDDK)(United States Department of Health & Human ServicesNational Institutes of Health (NIH) - USANIH National Institute of Diabetes & Digestive & Kidney Diseases (NIDDK)); Maryland Department of Health's Cigarette Restitution Fund Program
AcknowledgmentsWe would like to thank Lisadine Cherubin for assisting with the transcription of the exit interviews and helpful discussions. We are also grateful for helpful discussions with Cheryl Knott regarding qualitative research methodology. We would like to thank the Summer Program in Obesity, Diabetes, and Nutrition Research Training (SPORT) at the University of Maryland School of Medicine for opportunities, resources, and mentorship for B.I.H. 50 10 11 5 16 ROUTLEDGE JOURNALS, TAYLOR & FRANCIS LTD ABINGDON 2-4 PARK SQUARE, MILTON PARK, ABINGDON OX14 4RN, OXON, ENGLAND 0163-5581 1532-7914 NUTR CANCER Nutr. Cancer MAY 28 2023 75 5
1349 1360 10.1080/01635581.2023.2192891
http://dx.doi.org/10.1080/01635581.2023.2192891 MAR 2023 12 Oncology; Nutrition & Dietetics Science Citation Index Expanded (SCI-EXPANDED) Oncology; Nutrition & Dietetics
G3FR2 36942399 Green Accepted 2025-06-24
WOS:000950974100001
J Cheema, S; Abraham, A; Maisonneuve, P; Jithesh, A; Chaabna, K; al Janahi, R; Sarker, S; Hussain, A; Rao, S; Lowenfels, AB; Mamtani, R Cheema, Sohaila; Abraham, Amit; Maisonneuve, Patrick; Jithesh, Anupama; Chaabna, Karima; al Janahi, Reem; Sarker, Shaunak; Hussain, Athba; Rao, Shrinidhi; Lowenfels, Albert B.; Mamtani, Ravinder HPV infection and vaccination: a cross-sectional study of knowledge, perception, and attitude to vaccine uptake among university students in Qatar
BMC PUBLIC HEALTH English Article
HPV infection; HPV vaccine; Cervical cancer; Qatar; University students HUMAN-PAPILLOMAVIRUS VACCINE; ABNORMAL CERVICAL CYTOLOGY; NORTH-AFRICA; MIDDLE-EAST; INTERVENTIONS; WOMEN; EPIDEMIOLOGY; COVERAGE; CANCER
BackgroundHuman Papilloma Virus (HPV) infection is a

significant public health concern in the Gulf Cooperation Council countries, being widely prevalent and the main risk factor for cervical cancer. We aimed to assess knowledge and perception towards HPV, acceptability of the HPV vaccine, and HPV vaccination rates among university students in Education City, Doha, Qatar.MethodsThis cross-sectional survey utilized proportional quota-sampling, with quotas based on university, sex, and nationality, to recruit students from seven universities between February and September 2022. The English language questionnaire requested socio-demographic information, knowledge, and attitudes about HPV infection and the vaccine. The chi-square test, Student t-test, Mann-Whitney-Wilcoxon tests and multivariable ordinal logistic regression were used to assess differences in proportion, mean, and median according to broad HPV knowledge categories.ResultsThree hundred and ninety-eight students were recruited (response rate = 82.3%), of whom 251 (63.1%) were female. Mean age was 21.7 years. Eighty-nine (22.4%, 95% CI 18.4-26.8%) students had poor knowledge about HPV, 220 (55.3%, 95% CI 50.2-60.2%) students had some awareness, and 89 (22.4%, 95% CI 18.4-26.8%) students were knowledgeable. Age, nationality, and field of study influenced the students' knowledge about HPV. Only 25 (6.3%) students had previously been vaccinated against HPV. However, 71% of the unvaccinated students reported being willing to get vaccinated if recommended by their healthcare provider.ConclusionsOverall, 77.7% of the student population had some-to-good levels of knowledge about HPV-related infection, cancer, and vaccination. There are gaps in the student population's understanding and knowledge about HPV. Increasing knowledge can be key toward shared decision-making for HPV vaccination among eligible populations. Targeted public health campaigns and integration into childhood vaccination programs should be critical first steps, especially as most of the surveyed students had a positive outlook on getting vaccinated. Healthcare professionals should be incentivized to increase their HPV knowledge and communication skills, while policymakers can work toward easing barriers in integrating HPV vaccinations in the immunization schedule and encouraging overall HPV vaccination uptake. [Cheema, Sohaila; Abraham, Amit; Jithesh, Anupama; Chaabna, Karima; Mamtani, Ravinder] Weill Cornell Med Qatar, Inst Populat Hlth, Doha, Qatar; [Maisonneuve, Patrick] IEO European Inst Oncol IRCCS, Div Epidemiol & Biostat, Milan, Italy; [al Janahi, Reem; Sarker, Shaunak; Hussain, Athba; Rao, Shrinidhi] Weill Cornell Med Qatar, Doha, Qatar; [Lowenfels, Albert B.] New York Med Coll, Dept Surg, Dept Family Med, Valhalla, NY USA
Qatar Foundation (QF); Weill Cornell Medical College Qatar; Qatar Foundation (QF); Weill Cornell Medical College Qatar; New York Medical College Cheema, S (corresponding author), Weill Cornell Med Qatar, Inst Populat Hlth, Doha, Qatar. soc2005@qatar-med.cornell.edu Maisonneuve, Patrick/U-9789-2018; Lowenfels, Albert/ABD-8388-2021; Chaabna, Karima/H-9986-2019 Al-Janahi, Reem/0009-0002-9440-0904 Qatar National Library Qatar National Library(Qatar National Research Fund (QNRF)) Open access funding provided by the Qatar National Library. 54 0 0 0 2 BMC LONDON CAMPUS, 4 CRINAN ST, LONDON N1 9XW, ENGLAND 1471-2458 BMC PUBLIC HEALTH BMC Public Health AUG 26 2024 24 1 2316 10.1186/s12889-024-19792-0
http://dx.doi.org/10.1186/s12889-024-19792-0 13

Public, Environmental & Occupational Health Science Citation Index Expanded (SCI-EXPANDED) Public, Environmental & Occupational Health E0K7B 39187821 gold 2025-06-24 WOS:001299986100010
J Djuric, Z; Ren, JW; Blythe, J; VanLoon, G; Sen, A
Djuric, Zora; Ren, Jianwei; Blythe, Jason; VanLoon, Glee; Sen, Ananda A Mediterranean dietary intervention in healthy American women changes plasma carotenoids and fatty acids in distinct clusters NUTRITION RESEARCH
English Article
Cancer prevention; Monounsaturated fat; Fruit; Vegetables; Olive oil; Human; Clinical trial; Carotenoids BREAST-CANCER; INSULIN-RESISTANCE; SERUM CAROTENOIDS; POOLED ANALYSIS; BODY-WEIGHT; RISK; BIOMARKERS; VEGETABLES; SURVIVAL; COHORT This study examined patterns of changes in plasma fatty acids and carotenoids when women were asked to follow a novel, Greek-Mediterranean exchange list diet. A total of 69 healthy, nonobese women ages 25 to 59 years were randomized either to continue their own usual diet or to follow a modified Mediterranean diet for 6 months. There were no significant changes in blood lipids, triacylglycerol, insulin, glucose, or C-reactive protein. Mean plasma carotenoids increased by 55%, which is consistent with a large increase in fruit and vegetable consumption. Likewise, changes in fat intakes were reflected in blood fatty acids, with a 25% increase in mean plasma monounsaturated fatty acids. Principal component analysis was conducted to examine the sources of interindividual variation for changes in carotenoid and fatty acid levels. Changes in the Mediterranean diet were clustered together in 4 components that accounted for 78% of the variance in plasma levels. Increases in plasma lutein, a-carotene, and beta-carotene clustered together in a "vegetable" pattern, and increases in carotenoids found in fruit, beta-cryptoxanthin and zeaxanthin also clustered together but accounted for less of the variance. Increases in plasma monounsaturated fatty acids were clustered with a decrease in plasma polyunsaturated fatty acids, consistent with substitution in the type of oils consumed. The only association of fatty acid levels with carotenoids was that of lycopene, which clustered together with an increase in saturated fatty acids. The changes in blood levels indicate the exchange list diet was effective for targeting Mediterranean nutrient intakes using foods available in the United States. (c) 2009 Elsevier Inc. All rights reserved. [Djuric, Zora] Univ Michigan, Canc & Geriatr Ctr, Dept Family Med, Ann Arbor, MI 48109 USA University of Michigan System; University of Michigan
Djuric, Z (corresponding author), Univ Michigan, Canc & Geriatr Ctr, Dept Family Med, Ann Arbor, MI 48109 USA.
zoralong@umich.edu Djuric, Zora/H-5147-2013 Djuric, Zora/0000-0002-8886-8853 American Institute for Cancer Research [03B043]; National Institutes of Health (NIH) Cancer Center [P30 CA46592]; National Institute of Diabetes and Digestive and Kidney Diseases [P60 DK20572]; National Center for Research Resources (NCRR), [M01-RR000042]; National Cancer Institute [P30CA046592] Funding Source: NIH RePORTERAmerican Institute for Cancer Research; National Institutes of Health (NIH) Cancer Center(United States Department of Health & Human ServicesNational Institutes of Health (NIH) - USA); National Institute of Diabetes and Digestive and Kidney Diseases(United States Department of Health & Human ServicesNational Institutes of Health (NIH) - USANIH National

Institute of Diabetes & Digestive & Kidney Diseases (NIDDK));
National Center for Research Resources (NCRR),(United States
Department of Health & Human ServicesNational Institutes of Health
(NIH) - USANIH National Center for Research Resources (NCRR));
National Cancer Institute(United States Department of Health &
Human ServicesNational Institutes of Health (NIH) - USANIH
National Cancer Institute (NCI)) We thank the women who
volunteered their time to participate in the Mediterranean Eating
Study. Hoffman LaRoche, Ltd (Basel, Switzerland) generously
provided tocol for the high-performance liquid chromatography
analyses. Katherine Radakovich and Nora DiLaura conducted some of
the dietary counseling for the study and helped design the
methods. This work was funded by the American Institute for Cancer
Research (grant no. 03B043). Additional support at the University
of Michigan was obtained from National Institutes of Health (NIH)
Cancer Center Support Grant P30 CA46592, the Chemistry Laboratory
of the Michigan Diabetes Research and Training Center (funded by
P60 DK20572 from the National Institute of Diabetes and Digestive
and Kidney Diseases) and the General Clinical Research Center
(funded by grant M01-RR000042 from the National Center for
Research Resources (NCRR), a component of the NTH). The contents
of this article are solely the responsibility of the authors and
do not necessarily represent the official views of NCRR or NIH.
50 48 53 0 13 PERGAMON-ELSEVIER SCIENCE
LTD OXFORD THE BOULEVARD, LANGFORD LANE, KIDLINGTON, OXFORD
OX5 1GB, ENGLAND 0271-5317 NUTR RES Nutr. Res. MAR
2009 29 3 156 163
10.1016/j.nutres.2009.03.001
http://dx.doi.org/10.1016/j.nutres.2009.03.001
8 Nutrition & Dietetics Science Citation Index Expanded
(SCI-EXPANDED) Nutrition & Dietetics 435SR 19358929 Green
Accepted 2025-06-24 WOS:000265364100003
J Ragusa, A; Romano, P; Lenucci, MS; Civino, E; Vergara, D;
Pitotti, E; Neglia, C; Distante, A; Romano, GD; Di Renzo, N;
Surico, G; Piscitelli, P; Maffia, M Ragusa,
Andrea; Romano, Pietrina; Lenucci, Marcello Salvatore; Civino,
Emanuela; Vergara, Daniele; Pitotti, Elena; Neglia, Cosimo;
Distante, Alessandro; Romano, Giampiero Diego; Di Renzo, Nicola;
Surico, Giammarco; Piscitelli, Prisco; Maffia, Michele
Differential Glycosylation Levels in Saliva from Patients
with Lung or Breast Cancer: A Preliminary Assessment for Early
Diagnostic Purposes METABOLITES English Article
HPEAC-PAD; glycoprotein;
glycomics; glycosylation; saliva; early diagnosis; lung cancer;
breast cancer; fucose; mannose; glucosamine CELL-WALL
POLYSACCHARIDES; EXCHANGE CHROMATOGRAPHY; N-GLYCOSYLATION; ORAL-
CANCER; BIOMARKERS; SERUM; DISCOVERY; FUCOSE Glycans play a
fundamental role in several biological processes, such as cell-
cell adhesion, signaling, and recognition. Similarly, abnormal
glycosylation is involved in many pathological processes, among
which include tumor growth and progression. Several highly
glycosylated proteins found in blood are currently used in
clinical practice as cancer biomarkers (e.g., CA125, PSA, and
CA19-9). The development of novel non-invasive diagnostic
procedures would greatly simplify the screening and discovery of
pathologies at an early stage, thus also allowing for simpler
treatment and a higher success rate. In this observational study
carried out on 68 subjects diagnosed with either breast or lung

cancer and 34 healthy volunteers, we hydrolyzed the glycoproteins in saliva and quantified the obtained free sugars (fucose, mannose, galactose, glucosamine, and galactosamine) by using high-performance anion-exchange chromatography with pulsed-amperometric detection (HPAEC-PAD). The glycosidic profiles were compared by using multivariate statistical analysis, showing differential glycosylation patterns among the three categories. Furthermore, Receiver Operating Characteristics (ROC) analysis allowed obtaining a reliable and minimally invasive protocol able to discriminate between healthy and pathological subjects. [Ragusa, Andrea; Lenucci, Marcello Salvatore; Civino, Emanuela; Vergara, Daniele; Maffia, Michele] Univ Salento, Dept Biol & Environm Sci & Technol, Campus Ecotekne,Via Monteroni, I-73100 Lecce, Italy; [Ragusa, Andrea; Maffia, Michele] CNR Nanotec, Inst Nanotechnol, Via Monteroni, I-73100 Lecce, Italy; [Romano, Pietrina; Romano, Giampiero Diego; Di Renzo, Nicola; Surico, Giammarco] Vito Fazzi Hosp, Med Oncol Unit, I-73100 Lecce, Italy; [Pitotti, Elena; Piscitelli, Prisco] Local Hlth Author ASL Lecce, I-73100 Lecce, Italy; [Neglia, Cosimo; Distante, Alessandro] Euro Mediterranean Sci Biomed Inst ISBEM, I-72100 Brindisi, Italy University of Salento; Consiglio Nazionale delle Ricerche (CNR); Istituto di Nanotecnologia (NANOTEC-CNR); Azienda Ospedaliera Vito Fazzi
Ragusa, A; Maffia, M (corresponding author), Univ Salento, Dept Biol & Environm Sci & Technol, Campus Ecotekne,Via Monteroni, I-73100 Lecce, Italy.; Ragusa, A; Maffia, M (corresponding author), CNR Nanotec, Inst Nanotechnol, Via Monteroni, I-73100 Lecce, Italy. andrea.ragusa@unisalento.it; info@isbem.it; marcello.lenucci@unisalento.it; emanuela.civino@unisalento.it; daniele.vergara@unisalento.it; proteomica.polecce@ausl.le.it; neglia@isbem.it; distante@isbem.it; oncologia.polecce@ausl.le.it; direnzo.ematolecce@gmail.com; repol@ausl.le.it; piscitelli@unescochairnapoli.it; michele.maffia@unisalento.it
MAFFIA, MICHELE/AAC-2943-2020; Piscitelli, Prisco/L-1033-2016; Ragusa, Andrea/A-5563-2008; Lenucci, Marcello Salvatore/N-7174-2015; Vergara, Daniele/K-3831-2014 Ragusa, Andrea/0000-0002-2198-6185; DISTANTE, Alessandro/0000-0003-2776-0192; MAFFIA, MICHELE/0000-0003-0665-4534; Lenucci, Marcello Salvatore/0000-0002-6493-9549; Vergara, Daniele/0000-0002-2396-7674 Puglia Technological Cluster Project [T7WGSJ3] Puglia Technological Cluster Project This research was funded by Puglia Technological Cluster Project cod. T7WGSJ3 (2015): "Sistema"-Development of new methodologies and innovative instruments for diagnosis and treatment of human epithelial tumors. 56 15 19 0
13 MDPI BASEL MDPI AG, Grosspeteranlage 5, CH-4052 BASEL, SWITZERLAND 2218-1989 METABOLITES Metabolites SEP 2021
11 9 566
10.3390/metabo11090566
http://dx.doi.org/10.3390/metabo11090566 16
Biochemistry & Molecular Biology Science Citation Index Expanded (SCI-EXPANDED) Biochemistry & Molecular Biology
UW4IA 34564382 gold, Green Published 2025-06-24 WOS:000700120500001
J Holguin, F; Moughrabieh, MA; Ojeda, V; Patel, SR; Peyrani, P; Pinedo, M; Celedón, JC; Douglas, IS; Upson, DJ; Roman, J
Holguin, Fernando; Anas Moughrabieh, M.; Ojeda, Victoria; Patel, Sanjay R.; Peyrani, Paula; Pinedo, Miguel; Celedon, Juan C.; Douglas, Ivor S.; Upson, Dona J.; Roman, Jesse
Respiratory Health in Migrant Populations: A

Crisis Overlooked   ANNALS OF THE AMERICAN THORACIC SOCIETY   English   Article

lung; asthma; deportation; sleep; refugees   POSTTRAUMATIC-STRESS-DISORDER; INJECTION-DRUG USERS; DEPORTATION EXPERIENCES; SLEEP DISTURBANCE; HIV-INFECTION; UNITED-STATES; RISK-FACTORS; TIJUANA; ASTHMA; ACCULTURATION   The crisis in the Middle East has raised awareness about the challenges encountered by migrant populations, in particular, health-care access and delivery. Similar challenges are encountered by migrant populations around the world, including those entering the United States as refugees and/or survivors of torture as well as Mexicans and other Latin Americans crossing the border. During the 2016 International American Thoracic Society Meeting held in San Francisco, California, a group of researchers and health-care providers discussed these challenges at a minisymposium devoted to the respiratory health of migrants. The discussion focused on the increased incidence of airway diseases among individuals migrating to more developed countries, the problems created by sleep disorders and their implications for cardiovascular and mental health, the challenges inherent in the control of infections in refugee populations, and the problems resulting from deportation. The group also discussed the potential impact of novel strategies made available by Internet-based technologies and how these strategies could be deployed to support worldwide efforts in assisting migrants and refugees, even in countries that find themselves in the direst circumstances. These presentations are summarized in this document, which is not meant to be exhaustive, but to improve awareness about the challenges confronted by migrants and their host nations regarding respiratory health-care access and delivery, and about the need for adequate investment of resources to better define these challenges through research and for the development of efficient strategies for intervention.

[Holguin, Fernando] Univ Colorado, Dept Med, Pulm Sci, Denver, CO 80262 USA; [Anas Moughrabieh, M.] Wayne State Univ, Dept Med, Div Pulm & Crit Care, Detroit, MI 48202 USA; [Ojeda, Victoria] Univ Calif San Diego, Dept Med, Div Global Publ Hlth, La Jolla, CA 92093 USA; [Patel, Sanjay R.] Univ Pittsburgh, Dept Med, Div Pulm Allergy & Crit Care Med, Pittsburgh, PA 15260 USA; [Peyrani, Paula] Univ Louisville, Hlth Sci Ctr, Dept Med, Div Infect Dis, Louisville, KY 40292 USA; [Pinedo, Miguel] Univ Calif Berkeley, Alcohol Res Grp, Berkeley, CA 94720 USA; [Celedon, Juan C.] Univ Pittsburgh, Dept Pediat, Div Pulm Med Allergy & Immunol, Pittsburgh, PA 15260 USA; [Douglas, Ivor S.] Univ Colorado, Denver Hlth Med Ctr, Dept Med, Div Pulm & Crit Care, Boulder, CO 80309 USA; [Douglas, Ivor S.] Anschutz Med Ctr, Denver, CO USA; [Upson, Dona J.] New Mexico Vet Affairs Hlth Care Serv, Dept Med, Div Pulm & Crit Care, Albuquerque, NM USA; [Roman, Jesse] Univ Louisville, Dept Med, Div Pulm Crit Care & Sleep Disorders, Louisville, KY 40292 USA; [Roman, Jesse] Univ Louisville, Dept Pharmacol, Louisville, KY 40292 USA; [Roman, Jesse] Robley Rex Vet Affairs Med Ctr, Louisville, KY USA University of Colorado System; University of Colorado Anschutz Medical Campus; University of Colorado Denver; Wayne State University; University of California System; University of California San Diego; Pennsylvania Commonwealth System of Higher Education (PCSHE); University of Pittsburgh; University of Louisville; University of California System; University of California Berkeley; Alcohol Research Group; Pennsylvania Commonwealth System of Higher Education (PCSHE);

University of Pittsburgh; University of Colorado System; University of Colorado Boulder; Denver Health Medical Center; University of Colorado System; University of Colorado Anschutz Medical Campus; University of Louisville; University of Louisville
Roman, J (corresponding author), Univ Louisville, Dept Med, 550 S Jackson St,ACB 3rd floor,Med Suite, Louisville, KY 40202 USA. j.roman@louisville.edu ; Douglas, Ivor/AAC-6245-2021
Patel, Sanjay/0000-0002-9142-5172; Douglas, Ivor/0000-0002-4541-1431 National Institute on Alcohol Abuse and Alcoholism [T32AA007240] Funding Source: NIH RePORTER; NHLBI NIH HHS [T32 HL007741, R01 HL117191] Funding Source: Medline; NIAAA NIH HHS [T32 AA007240, P50 AA005595] Funding Source: Medline National Institute on Alcohol Abuse and Alcoholism(United States Department of Health & Human ServicesNational Institutes of Health (NIH) - USANIH National Institute on Alcohol Abuse & Alcoholism (NIAAA)); NHLBI NIH HHS(United States Department of Health & Human ServicesNational Institutes of Health (NIH) - USANIH National Heart Lung & Blood Institute (NHLBI)); NIAAA NIH HHS(United States Department of Health & Human ServicesNational Institutes of Health (NIH) - USANIH National Institute on Alcohol Abuse & Alcoholism (NIAAA)) 72 13 15 0 13 AMER THORACIC SOC NEW YORK 25 BROADWAY, 18 FL, NEW YORK, NY 10004 USA
1546-3222 2325-6621 ANN AM THORAC SOC Ann. Am. Thoracic Society FEB 2017 14 2 153
159 10.1513/AnnalsATS.201608-592PS
http://dx.doi.org/10.1513/AnnalsATS.201608-592PS
7 Respiratory System Science Citation Index Expanded (SCI-EXPANDED); Social Science Citation Index (SSCI)
Respiratory System FR9ZX 28146384 Green Published
2025-06-24 WOS:000419434800001
J Sezaki, A; Imai, T; Miyamoto, K; Kawase, F; Shirai, Y; Abe, C; Sanada, M; Inden, A; Kato, T; Suzuki, N; Shimokata, H
Sezaki, Ayako; Imai, Tomoko; Miyamoto, Keiko; Kawase, Fumiya; Shirai, Yoshiro; Abe, Chisato; Sanada, Masayo; Inden, Ayaka; Kato, Takumi; Suzuki, Norie; Shimokata, Hiroshi
Global relationship between Mediterranean diet and the incidence and mortality of ischaemic heart disease EUROPEAN JOURNAL OF PUBLIC HEALTH English Article
BREAST-CANCER; RISK; ADHERENCE; INDEXES; HEALTH; SCORE Background: The purpose of this study was to clarify the global relationship between the Mediterranean diet score (MDS) and country-wise incidence and mortality of ischaemic heart disease (IHD) using an international database. Methods: We used population data from a global longitudinal database covering 137 countries with a population of over one million. MDS were evaluated based on the total score of the nine foods that comprise the Mediterranean diet. The incidence and mortality of IHD by country was derived from the Global Burden of Disease (GBD) database. Average food (g/day/capita) and energy supply (kcal/day/capita) by country, excluding loss between production and household, were obtained from the Food and Agriculture Organization of the United Nations Statistics Division database. Data from the GBD database were used for body mass index, current smoking rates, physical activity, years of education and percentage of the Muslim population. We identified the percentage of the population over 65 years of age (aging rate) and gross domestic product per capita (US$/capita) using the World Bank database. A linear mixed-effect model was used for evaluating the

effects of MDS on incidence and mortality of IHD controlled for socioeconomic and lifestyle variables. Results: Analysis showed that MDS was significantly associated with IHD incidence after controlling for covariates (-1.01 +/- 0.27, P< 0.001). Similarly, there was a significant association between MDS and IHD-related mortality after controlling for covariates (-0.73 +/- 0.34, P< 0.05). Conclusion: Analysis of 27 years of data suggests that a Mediterranean diet might have a preventive effect on IHD.
[Sezaki, Ayako; Sanada, Masayo; Inden, Ayaka; Shimokata, Hiroshi] Nagoya Univ Arts & Sci, Grad Sch Nutr Sci, Takenoyama 57,Iwasaki Cho, Nisshin, Aichi 4700196, Japan; [Sezaki, Ayako] Ryukoku Univ, Dept Food Sci & Human Nutr, Otsu, Shiga, Japan; [Imai, Tomoko] Doshisha Womens Coll Liberal Arts, Dept Food Sci & Nutr, Kyoto, Japan; [Miyamoto, Keiko] Nagoya Univ Arts & Sci, Dept Nursing, Nisshin, Aichi, Japan; [Kawase, Fumiya] Asuke Hosp Aichi Prefectural Welf Federat Agr Coo, Dept Nutr, Toyota, Aichi, Japan; [Shirai, Yoshiro] Kinjo Gakuin Univ, Dept Food & Nutr Environm, Nagoya, Aichi, Japan; [Abe, Chisato] Tsu City Coll, Dept Life & Environm Sci, Tsu, Mie, Japan; [Inden, Ayaka] Hamamatsu Univ Hosp, Dept Nutr, Shizuoka, Japan; [Kato, Takumi] Japanese Red Cross Nagoya Daini Hosp, Dept Nutr, Nagoya, Aichi, Japan; [Suzuki, Norie] Ochanomizu Univ, Fac Core Res, Tokyo, Japan Ryukoku University; Hamamatsu University School of Medicine; Japanese Red Cross Nagoya Daini Hospital; Ochanomizu University Sezaki, A (corresponding author), Nagoya Univ Arts & Sci, Grad Sch Nutr Sci, Takenoyama 57,Iwasaki Cho, Nisshin, Aichi 4700196, Japan.
20gn102@st.nuas.ac.jp Miyamoto, keiko/IYT-2910-2023; Kawase, Fumiya/JGM-8633-2023 Sezaki, Ayako/0000-0002-3671-5476; Shirai, Yoshiro/0000-0001-5471-5333; Kawase, Fumiya/0000-0001-6187-4543; Imai, Tomoko/0000-0002-6552-3158 19 4
4 1 7 OXFORD UNIV PRESS OXFORD GREAT CLARENDON ST, OXFORD OX2 6DP, ENGLAND 1101-1262 1464-360X
EUR J PUBLIC HEALTH Eur. J. Public Health JUN 2021 31
3 608 612
10.1093/eurpub/ckab008
http://dx.doi.org/10.1093/eurpub/ckab008 FEB 2021
6 Public, Environmental & Occupational Health Science Citation Index Expanded (SCI-EXPANDED); Social Science Citation Index (SSCI) Public, Environmental & Occupational Health
YS7RN 33674837 Bronze 2025-06-24
WOS:000750870100030
J Asik, E; Aslan, TN; Güray, NT; Volkan, M
Asik, Elif; Aslan, Tugba Nur; Guray, N. Tulin; Volkan, Murvet Cellular uptake and apoptotic potential of rhenium labeled magnetic protein cages in MDA-MB-231 cells
ENVIRONMENTAL TOXICOLOGY AND PHARMACOLOGY
English Article
Magnetoferritin; Cytotoxicity; Apoptosis; Breast cancer cell lines CANCER-CELLS; OXIDE NANOPARTICLES; CYTOTOXICITY; APOFERRITIN; TOXICITY; FERRITIN; STRESS Re-188-magnetoferritin nanoparticles (NPs) provide an attractive platform for localized radiation therapy due to their magnetic targeting capability while enhancing contrast in magnetic resonans imaging (MRI) signals. In this study, cellular uptake, in vitro cytotoxicity, apoptotic potential of a non-radioactive isotope of rhenium in the form of Re-187-magnetoferritin NPs were evaluated in both human normal mammary epithelial and breast metastatic adenocarcinoma cell lines. The results showed that, NP administration into the cells

is through receptor mediated endocytosis and cancer cells displayed significantly higher uptake and cytotoxicity compared to normal cells. IC50 values of nanoparticles were calculated as 0.96 mg/mL for cancer and 1.73 mg/mL for normal cells. Annexin V/ Propidium Iodide (PI) staining also showed that, NPs induced higher apoptotic rates in cancer cells compared to normal cells. Gene expression analyses confirming the results showed that, pro-apoptotic PUMA and BAX genes were significantly up-regulated while anti-apoptotic BCL-2 and SURVIVIN genes were down-regulated in cancer cells compared to normal cells. Overall, these in vitro results suggest that, Re-187-magnetoferritin NPs have a promising potential for cancer therapy and can be used for imaging and diagnostic purposes for breast cancer at concentrations lower than 0.96 mg/mL. At concentrations above 1 mg/mL, NPs induce apoptosis which can also be used for cancer treatments. [Asik, Elif; Guray, N. Tulin] Middle East Tech Univ, Dept Biotechnol, TR-06800 Ankara, Turkey; [Aslan, Tugba Nur; Volkan, Murvet] Middle East Tech Univ, Dept Chem, TR-06800 Ankara, Turkey; [Guray, N. Tulin] Middle East Tech Univ, Dept Biol Sci, TR-06800 Ankara, Turkey; [Aslan, Tugba Nur] Necmettin Erbakan Univ, Fac Sci, Dept Mol Biol & Genet, Konya, Turkey Middle East Technical University; Middle East Technical University; Middle East Technical University; Necmettin Erbakan UniversityVolkan, M (corresponding author), Middle East Tech Univ, Dept Chem, TR-06800 Ankara, Turkey.
murvet@metu.edu.tr Aslan, Tuğba/KCL-6092-2024; Asik, Elif/AAE-1841-2020; Volkan, Murvet/AAU-8639-2021; Guray, N.Tulin/AAZ-6727-2020 Guray, N.Tulin/0000-0003-4572-1429; ASLAN, TUGBA NUR/0000-0002-5516-3603; Volkan, Murvet/0000-0001-5112-9486
Research Fund of METU OYP Grant [BAP-08-11-DPT-2011K121010]
Research Fund of METU OYP Grant This study was funded by the Research Fund of METU OYP Grant No: BAP-08-11-DPT-2011K121010.
32 7 7 0 13 ELSEVIER SCIENCE BV
AMSTERDAM PO BOX 211, 1000 AE AMSTERDAM, NETHERLANDS
1382-6689 1872-7077 ENVIRON TOXICOL PHAR Environ. Toxicol. Pharmacol. OCT 2018 63
127 134 10.1016/j.etap.2018.08.014
http://dx.doi.org/10.1016/j.etap.2018.08.014 8
Environmental Sciences; Pharmacology & Pharmacy; Toxicology
Science Citation Index Expanded (SCI-EXPANDED)
Environmental Sciences & Ecology; Pharmacology & Pharmacy; Toxicology GX1ML 30223109 2025-06-24
WOS:000447481300017
J Hamed, Y; Ayadi, Y; Hadji, R; Ben Saad, A; Gentilucci, M; Elaloui, E Hamed, Younes; Ayadi, Yosra; Hadji, Rihab; Ben Saad, Amina; Gentilucci, Matteo; Elaloui, Elimame
Environmental Radioactivity, Ecotoxicology (²³⁸U, ²³²Th and ⁴⁰K) and Potentially Toxic Elements in Water and Sediments from North Africa Dams SUSTAINABILITY English Article
radioactivity; water quality; potentially toxic elements; health risks; potential toxicity
NATURAL RADIONUCLIDES; FERTILIZERS; SAMPLES; RIVER The natural radioactivity of U-238, Th-232 and K-40 was measured in water and sediment samples collected from Sidi Salem dam (Tunisia) and A & iuml;n Dalia dam (Algeria) in the Tuniso-Algerian transboundary basin. The samples were measured using a TERRA detector of gamma, beta, and alpha rays, and atomic absorption and gamma-ray spectrometry were used to analyze the levels of

radionuclides and toxic elements, respectively. Potentially toxic elements (Fe, Pb, Zn, Ni, Cr, Cu and Cd) and associated health risks in surface water and sediment of dams were investigated in this present study. The concentrations of Fe, Pb, Zn, Ni, Cr, Cu and Cd in surface water ranged from 5.430 to 9.700 mg<middle dot>L-1, 0.022 to 0.168, 0.018 to 0.142, 0.065 to 0.366, BDL to 0.0351, BDL to 0.071 and BDL to 0.048 mg<middle dot>L-1, respectively. In the sediments, the concentrations of Fe, Pb, Zn, Cu, Ni, Cd and Cr were of the order of 136.7, 3.41, 3.22, 0.213, 0.182, 0.15 mg<middle dot>L-1 and BDL, respectively. The mean radioactivity rates in the water samples were 1.72, 0.068 and 94.6 Bq<middle dot>L-1 for U-238, Th-232 and K-40, respectively (Tunisia dam), and were 1.9, 0.09 and 131.43 Bq<middle dot>L-1 for U-238, Th-232 and K-40, respectively (Algeria dam). The mean U-238, Th-232 and K-40 radioactivity concentrations measured in the sediment samples were 2.67, 0.18 and 197.87 Bq<middle dot>kg(-1), respectively (Tunisian dam), and were 4.34, 0.27 and 287.61 Bq<middle dot>kg(-1), respectively (Algeria dam). The activity concentration of K-40 was higher than that of U-238 and Th-232 for the water and sediment samples. The activity concentrations follow the order K-40 > U-238 > Th-232. The cumulative impact of these radio-geochemical elements can cause immediate serious problems in the ecosystem due to their high potential toxicity to the environment and human health in this study area and can be transposable to any other similar region. A good knowledge of monitoring quality and quantity for transboundary water resources and international collaborations are essential to safeguard human health (women's breast cancer, thyroid cancer, neurological impact) and avoid conflicts, especially during climatic upheavals of drought.[Hamed, Younes; Ayadi, Yosra; Ben Saad, Amina; Elaloui, Elimame] Univ Gafsa, Fac Sci Gafsa, Lab Applicat Mat Environm Water & Energy LAM3E, Gafsa 2112, Tunisia; [Hamed, Younes] Univ Houston, Dept Earth & Atmospher Sci, Sci & Res Bldg 1,3507 Cullen Blvd,Room 312, Houston, TX 77204 USA; [Hamed, Younes] TDS Co, Riyadh 11342, Saudi Arabia; [Hadji, Rihab] Set 1 Univ, Lab Appl Res Engn Geol Geotech Water Sci & Environ, Setif 19000, Algeria; [Gentilucci, Matteo] Univ Camerino, Sch Sci & Technol, Geol Div, I-62032 Camerino, Italy Universite de Gafsa; University of Houston System; University of Houston; Universite Ferhat Abbas Setif; University of Camerino Hamed, Y (corresponding author), Univ Gafsa, Fac Sci Gafsa, Lab Applicat Mat Environm Water & Energy LAM3E, Gafsa 2112, Tunisia.; Hamed, Y (corresponding author), Univ Houston, Dept Earth & Atmospher Sci, Sci & Res Bldg 1,3507 Cullen Blvd,Room 312, Houston, TX 77204 USA.; Hamed, Y (corresponding author), TDS Co, Riyadh 11342, Saudi Arabia. hamedhydro.tn@gmail.com; ayadiyosraa@yahoo.fr; hadjirihab@yahoo.fr; aminabensaad@yahoo.fr; matteo.gentilucci@unicam.it; limam_aloui@yahoo.fr Hamed, Younes/ABC-6660-2021; Elaloui, Elimame/HKF-6366-2023; Gentilucci, Matteo/M-3968-2018; HADJI, Riheb/P-7390-2016 Gentilucci, Matteo/0000-0002-5826-5031; HADJI, Riheb/0000-0002-9632-0812
52 11 11 5 12 MDPI BASEL ST ALBAN-ANLAGE 66, CH-4052 BASEL, SWITZERLAND 2071-1050
SUSTAINABILITY-BASEL Sustainability JAN 2024 16 2
490 10.3390/su16020490
http://dx.doi.org/10.3390/su16020490 20
Green & Sustainable Science & Technology; Environmental Sciences; Environmental Studies Science Citation Index Expanded

(SCI-EXPANDED); Social Science Citation Index (SSCI) Science & Technology - Other Topics; Environmental Sciences & Ecology
GM4B5 Green Submitted, gold 2025-06-24
WOS:001153060000001
J Gali-Muhtasib, H; Diab-Assaf, M; Boltze, C; Al-Hmaira, J; Hartig, R; Roessner, A; Schneider-Stock, R
Gali-Muhtasib, H; Diab-Assaf, M; Boltze, C; Al-Hmaira, J; Hartig, R; Roessner, A; Schneider-Stock, R
Thymoquinone extracted from black seed triggers apoptotic cell death in human colorectal cancer cells via a p53-dependent mechanism INTERNATIONAL JOURNAL OF ONCOLOGY English
Article anticancer; blackseed; cell cycle arrest; colon cancer; thymoquinone NIGELLA-SATIVA; CYTOCHROME-C; G(1) ARREST; P53; MITOCHONDRIA; INHIBITION; ACTIVATION; EXPRESSION; RELEASE; PROTEIN For centuries, the black seed (Nigella sativa) herb and oil have been used in Asia, Middle East and Africa to promote health and fight disease. Thymoquinone (TQ), the most abundant constituent present in black seed, is a promising dietary chemopreventive agent. We investigated the effects of thymoquinone (TQ) against HCT-116 human colon cancer cells and attempted to identify its potential molecular mechanisms of action. We report that TQ inhibits the growth of colon cancer cells which was correlated with G, phase arrest of the cell cycle. Furthermore, TUNEL staining and flow cytometry analysis indicate that TQ triggers apoptosis in a dose- and time-dependent manner. Apoptosis induction by TQ was associated with a 2.5-4.5-fold increase in mRNA expression of p53 and the downstream p53 target gene, p21(WAF1). Simultaneously, we found a marked increase in p53 and p21(WAF1) protein levels but a significant inhibition of anti-apoptotic Bcl-2 protein. Co-incubation with pifithrin-alpha. (PFT-alpha), a specific inhibitor of p53, restored Bcl-2, p53 and p21WAF1 levels to the untreated control and suppressed TQ-induced cell cycle arrest and apoptosis. p53-null HCT-116 cells were less sensitive to TQ-induced growth arrest and apoptosis. These results indicate that TQ is antineoplastic and pro-apoptotic against colon cancer cell line HCT116. The apoptotic effects of TQ are modulated by Bcl-2 protein and are linked to and dependent on p53. Our data support the potential for using the agent TQ for the treatment of colon cancer. Amer Univ Beirut, Dept Biol, Beirut, Lebanon; Otto Von Guericke Univ, Dept Pathol, Magdeburg, Germany; Otto Von Guericke Univ, Dept Immunol, Magdeburg, Germany American University of Beirut; Otto von Guericke University; Otto von Guericke University
Amer Univ Beirut, Dept Biol, Beirut, Lebanon.
regine.schneider-stock@medizin.uni-magdeburg.de
Schneider-Stock, Regine/H-8863-2012 Hartig, Roland/0000-0002-3706-7458 41 262 284 0 18
SPANDIDOS PUBL LTD ATHENS POB 18179, ATHENS, 116 10, GREECE 1019-6439 1791-2423 INT J ONCOL Int. J. Oncol.
OCT 2004 25 4 857 866
10 Oncology Science Citation Index Expanded (SCI-EXPANDED) Oncology 856VP 15375533
2025-06-24 WOS:000224073700007
J MciLvenny, S; Al Mahrouqi, F; Al Busaidi, T; Al Nabhani, A; Al Hikmani, F; Al Kharousi, Z; Al Mammari, S; Al Hoti, A; Al Shihi, A; Al Lawati, A; Al Kharousi, I
MciLvenny, S; Al Mahrouqi, F; Al Busaidi, T; Al Nabhani, A; Al Hikmani, F; Al Kharousi, Z; Al Mammari, S; Al Hoti, A; Al

Shihi, A; Al Lawati, A; Al Kharousi, I Rear seat belt use as an indicator of safe road behaviour in a rapidly developing country JOURNAL OF THE ROYAL SOCIETY FOR THE PROMOTION OF HEALTH English Article
developing countries; Middle East; Oman; road traffic accidents; seat belts SAUDI-ARABIA; INJURIES; EPIDEMIOLOGY
Injuries from road traffic accidents are set to become the second highest cause of disability-adjusted life years lost in developing countries by 2020. The number of injuries and deaths are disproportionately high in low income countries, which account for only 40% of all motor vehicles. Human behaviour is thought to be a major factor in most accidents. In Oman wearing a seat belt is compulsory in the front seats but not in the rear. Wearing a seat belt can reduce the severity of injuries when sitting in rear seats. This study examines the use of seat belts in cars entering a university and hospital campus in Oman to determine the degree of seat belt wearing in the rear. At peak times on a selected day, cars were stopped at the university's entrance barrier. The type of driver was identified - student, employee, hospital patient or visitor - and the degree of seat belt wearing among driver and passengers was noted. A total of 1,066 cars were stopped. Of this total, 90.1%. of drivers and 80.9% of front seat passengers were wearing a restraint complying with local traffic regulations. However, only 1.4% of back seat passengers wore a seat belt. Only 3.7% of children under the age of five were restrained in a child seat and only 16.7% of five- to 12-year-olds were strapped in. A third (34.6%) of under-fives were sitting in the front seat. In cars with child occupants, 40% of the time parents wore seat belts but the children did not. Occupants conformed to the law but behaviour indicated a tack of awareness of the dangers of not wearing seat belts, especially towards children. Traffic regulations need to be updated and the public educated about the need to wear seat belts. Health agencies could be more active in educating the public about road safety behaviour and should also be involved in the overall strategy to reduce injuries and deaths.
Sultan Qaboos Univ, Oman Med Specialty Board, Muscat 123, Oman Sultan Qaboos University MciLvenny, S (corresponding author), Sultan Qaboos Univ, Oman Med Specialty Board, POB 35, Muscat 123, Oman. 14
18 18 0 5 ROYAL SOC OF HEALTH LONDON 38A ST, GEORGES DR, LONDON SW1V 4BH, ENGLAND 1466-4240
J R SOC PROMO HEALTH J. R. Soc. Promot. Health NOV 2004
124 6 280 283
10.1177/146642400412400617
http://dx.doi.org/10.1177/146642400412400617 4
Public, Environmental & Occupational Health Social Science Citation Index (SSCI) Public, Environmental & Occupational Health
890KG 15602998 2025-06-24
WOS:000226509500015
J Wiens, KE; Lindstedt, PA; Blacker, BF; Johnson, KB; Baumann, MM; Schaeffer, LE; Abbastabar, H; Abd-Allah, F; Abdelalim, A; Abdollahpour, I; Abegaz, KH; Abejie, AN; Abreu, LG; Abrigo, MRM; Abualhasan, A; Accrombessi, MMK; Acharya, D; Adabi, M; Adamu, AA; Adebayo, OM; Adedoyin, RA; Adekanmbi, V; Adetokunboh, OO; Adhena, BM; Afarideh, M; Ahmad, S; Ahmadi, K; Ahmed, AE; Ahmed, MB; Ahmed, R; Akalu, TY; Alahdab, F; Al-Aly, Z; Alam, N; Alam, S; Alamene, GM; Alanzi, TM; Alcalde-Rabanal, JE; Ali, BA; Alijanzadeh, M; Alipour, V; Aljunid, SM; Almasi, A; Almasi-Hashiani, A; Al-

Mekhlafi, HM; Altirkawi, KA; Alvis-Guzman, N; Alvis-Zakzuk, NJ; Amini, S; Amit, AML; Andrei, CL; Anjomshoa, M; Anoushiravani, A; Ansari, F; Antonio, CAT; Antony, B; Antriyandarti, E; Arabloo, J; Aref, HMA; Aremu, O; Armoon, B; Arora, A; Aryal, KK; Arzani, A; Asadi-Aliabadi, M; Atalay, HT; Athari, SS; Athari, SM; Atre, SR; Ausloos, M; Awoke, N; Quintanilla, BPA; Ayano, G; Ayanore, MA; Aynalem, YA; Azari, S; Azzopardi, PS; Babaee, E; Babalola, TK; Badawi, A; Bairwa, M; Bakkannavar, SM; Balakrishnan, S; Bali, AG; Banach, M; Banoub, JAM; Barac, A; Barnighausen, TW; Basaleem, H; Basu, S; Bay, VD; Bayati, M; Baye, E; Bedi, N; Beheshti, M; Behzadifar, M; Behzadifar, M; Bekele, BB; Belayneh, YM; Bell, ML; Bennett, DA; Berbada, DA; Bernstein, RS; Bhat, AG; Bhattacharyya, K; Bhattarai, S; Bhaumik, S; Bhutta, ZA; Bijani, A; Bikbov, B; Birihane, BM; Biswas, RK; Bohlouli, S; Bojia, HA; Boufous, S; Brady, OJ; Bragazzi, NL; Briko, AN; Briko, NI; Britton, GB; Nagaraja, SB; Busse, R; Butt, ZA; Cámera, LA; Campos-Nonato, IR; Cano, J; Car, J; Cárdenas, R; Carvalho, F; Castaneda-Orjuela, CA; Castro, F; Chanie, WF; Chatterjee, P; Chattu, VK; Chichiabellu, TY; Chin, KL; Christopher, DJ; Chu, DT; Cormier, NM; Costa, VM; Culquichicon, C; Daba, MS; Damiani, G; Dandona, L; Dandona, R; Dang, AK; Darwesh, AM; Darwish, AH; Daryani, A; Das, JK; Das Gupta, R; Dash, AP; Davey, G; Dávila-Cervantes, CA; Davis, AC; Davitoiu, DV; De La Hoz, FP; Demis, AB; Demissie, DB; Demissie, GD; Demoz, GT; Demoz, GT; Denova-Gutiérrez, E; Deribe, K; Desalew, A; Deshpande, A; Dharmaratne, SD; Dhillon, P; Dhimal, M; Dhungana, GP; Diaz, D; Dipeolu, IO; Djalalinia, S; Doyle, KE; Dubljanin, E; Duko, B; Duraes, AR; Kalan, ME; Edinur, HA; Effiong, A; Eftekhari, A; El Nahas, N; El Sayed, I; Zaki, ME; El Tantawi, M; Elema, TB; Elhabashy, HR; El-Jaafary, SI; Elkout, H; Elsharkawy, A; Elyazar, IRF; Endalamaw, A; Endalew, DA; Eskandarieh, S; Esteghamati, A; Esteghamati, S; Etemadi, A; Ezekannagha, O; Fareed, M; Faridnia, R; Farzadfar, F; Fazlzadeh, M; Feigin, VL; Fereshtehnejad, SM; Fernandes, E; Filip, I; Fischer, F; Foigt, NA; Folayan, MO; Foroutan, M; Franklin, RC; Fukumoto, T; Gad, MM; Gayesa, RT; Gebre, T; Gebremedhin, KB; Gebremeskel, GG; Gesesew, HA; Gezae, KE; Ghadiri, K; Ghashghaee, A; Ghimire, PR; Gill, PS; Gill, TK; Ginindza, TG; Gomes, NGM; Gopalani, SV; Goulart, AC; Goulart, BNG; Grada, A; Gubari, MIM; Gugnani, HC; Guido, D; Guimaraes, RA; Guo, YM; Gupta, R; Hafezi-Nejad, N; Haile, DH; Hailu, GB; Haj-Mirzaian, A; Haj-Mirzaian, A; Hamadeh, RR; Hamidi, S; Handiso, DW; Haririan, H; Hariyani, N; Hasaballah, AI; Hasan, MM; Hasanpoor, E; Hasanzadeh, A; Hassankhani, H; Hassen, HY; Hegazy, MI; Heibati, B; Heidari, B; Hendrie, D; Henry, NJ; Herteliu, C; Heydarpour, F; de Hidru, HD; Hird, TR; Hoang, CL; Rad, EH; Hoogar, P; Hoseini, M; Hossain, N; Hosseini, M; Hosseinzadeh, M; Househ, M; Hsairi, M; Hu, GQ; Hussen, MM; Ibitoye, SE; Igumbor, EU; Ilesanmi, OS; Ilic, MD; Imani-Nasab, MH; Iqbal, U; Irvani, SSN; Islam, SMS; Iwu, CJ; Izadi, N; Jaca, A; Jahanmehr, N; Jakovljevic, M; Jalali, A; Jayatilleke, AU; Jha, RP; Jha, V; Ji, JS; Jonas, JB; Jozwiak, JJ; Kabir, A; Kabir, Z; Kahsay, A; Kalani, H; Kanchan, T; Matin, BK; Karch, A; Karim, MA; Karki, HKSS; Kasaeian, A; Kasahun, GG; Kasahun, YC; Kasaye, HK; Kassa, GG; Kassa, GM; Kayode, GA; Karyani, AK; Kebede, MM; Keiyoro, PN; Kelbore, AG; Kengne, AP; Ketema, DB; Khader, YS; Khafaie, MA; Khalid, N; Khalilov, R; Khan, EA; Khan, J; Khan, MN; Khan, MS; Khatab, K; Khater, AM; Khater, MM; Khayamzadeh, M; Khazaei, M; Khazaei, S; Khosravi, MH; Khubchandani, J; Kiadaliri, A; Kim, YJ; Kimokoti, RW; Kisa, A; Kisa, S; Kissoon, N; Shivakumar, KM; Kochhar, S; Kolola, T;

Komaki, H; Kosen, S; Koul, PA; Koyanagi, A; Kraemer, MUG; Krishan, K; Kugbey, N; Kumar, GA; Kumar, M; Kumar, P; Kusuma, VKD; La Vecchia, C; Lacey, B; Lad, SD; Lal, DK; Lam, F; Lami, FH; Lamichhane, P; Lansingh, VC; Lasrado, S; Laxmaiah, A; Lee, PH; LeGrand, KE; Leili, M; Lenjebo, TL; Leshargie, CT; Levine, AJ; Li, SS; Linn, S; Liu, SW; Liu, SM; Lodha, R; Longbottom, J; Lopez, JCF; Abd El Razek, HM; Abd El Razek, MM; Prasad, DRM; Mahasha, PW; Mahotra, NB; Majeed, A; Malekzadeh, R; Malta, DC; Mamun, AA; Manafi, N; Manda, AL; Manohar, NDD; Mansournia, MA; Mapoma, CC; Maravilla, JC; Martinez, G; Martini, S; Martins-Melo, FR; Masaka, A; Massenburg, BB; Mathur, MR; Mayala, BK; Mazidi, M; McAlinden, C; Meharie, BG; Mehndiratta, MM; Mehta, KM; Mekonnen, TC; Meles, GG; Memiah, PTN; Memish, ZA; Mendoza, W; Menezes, RG; Mereta, ST; Meretoja, TJ; Mestrovic, T; Miazgowski, B; Mihretie, KM; Miller, TR; Mini, GK; Mirrakhimov, EM; Moazen, B; Mohajer, B; Mohamadi-Bolbanabad, A; Mohammad, DK; Mohammad, KA; Mohammad, Y; Mezerji, NMG; Mohammadibakhsh, R; Mohammadifard, N; Mohammed, JA; Mohammed, S; Mohebi, F; Mokdad, AH; Molokhia, M; Monasta, L; Moodley, Y; Moore, CE; Moradi, G; Moradi, M; Moradi-Joo, M; Moradi-Lakeh, M; Moraga, P; Morales, L; Velásquez, IM; Mosapour, A; Mouodi, S; Mousavi, SM; Mozaffor, M; Muchie, KF; Mulaw, GF; Munro, SB; Muriithi, MK; Murray, CJL; Murthy, GVS; Musa, KI; Mustafa, G; Muthupandian, S; Nabhan, AF; Naderi, M; Nagarajan, AJ; Naidoo, KS; Naik, G; Najafi, F; Nangia, V; Nansseu, JR; Nascimento, BR; Nazari, J; Ndwandwe, DE; Negoi, I; Netsere, HB; Ngunjiri, JW; Nguyen, CT; Nguyen, HLT; Nguyen, TH; Nigatu, D; Nigatu, SG; Ningrum, DNA; Nnaji, CA; Nojomi, M; Nong, VM; Norheim, OF; Noubiap, JJ; Motlagh, SN; Oancea, B; Ogah, OS; Ogbo, FA; Oh, IH; Olagunju, AT; Olagunju, TO; Olusanya, BO; Olusanya, JO; Onwujekwe, OE; Oren, E; Ortega-Altamirano, DV; Osarenotor, O; Osei, FB; Owolabi, MO; Mahesh, PA; Padubidri, JR; Pakhale, S; Patel, SK; Paternina-Caicedo, AJ; Pathak, A; Patton, GC; Paudel, D; Paulos, K; Pepito, VCF; Pereira, A; Perico, N; Pervaiz, A; Pescarini, JM; Piroozi, B; Pirsaheb, M; Postma, MJ; Pourjafar, H; Pourmalek, F; Pourshams, A; Poustchi, H; Prada, SI; Prasad, N; Preotescu, L; Quintana, H; Rabiee, N; Radfar, A; Rafiei, A; Rahim, F; Rahimi-Movaghar, A; Rahimi-Movaghar, V; Rahman, MHU; Rahman, MA; Rahman, S; Rajati, F; Rana, SM; Ranabhat, CL; Rasella, D; Rawaf, DL; Rawaf, S; Rawal, L; Rawasia, WF; Renjith, V; Renzaho, AMN; Resnikoff, S; Reta, MA; Rezaei, N; Rezai, MS; Riahi, SM; Ribeiro, AI; Rickard, J; Rios-Blancas, M; Roever, L; Ronfani, L; Roro, EM; Ross, JM; Rubagotti, E; Rubino, S; Saad, AM; Sabde, YD; Sabour, S; Sadeghi, E; Safari, Y; Safari-Faramani, R; Sagar, R; Sahebkar, A; Sahraian, MA; Sajadi, SM; Salahshoor, MR; Salam, N; Salamati, P; Salem, H; Salem, MR; Salimi, Y; Salimzadeh, H; Samy, AM; Sanabria, J; Santric-Milicevic, MM; Jose, BPS; Saraswathy, SYI; Sarkar, K; Sarker, AR; Sarrafzadegan, N; Sartorius, B; Sathian, B; Sathish, T; Sawhney, M; Saxena, S; Schwebel, DC; Senbeta, AM; Senthilkumaran, S; Sepanlou, SG; Serván-Mori, E; Shabaninejad, H; Shafieesabet, A; Shaikh, MA; Shalash, AS; Shallo, SA; Shams-Beyranvand, M; Shamsi, M; Shamsizadeh, M; Shannawaz, M; Sharafi, K; Sharifi, H; Shehata, HS; Sheikh, A; Shetty, BSK; Shibuya, K; Shiferaw, WS; Shifti, DM; Shigematsu, M; Shin, JI; Shiri, R; Shirkoohi, R; Siabani, S; Siddiqi, TJ; Silva, DAS; Singh, A; Singh, JA; Singh, NP; Singh, V; Sisay, MM; Skiadaresi, E; Sobhiyeh, MR; Sokhan, A; Soltani, S; Somayaji, R; Soofi, M; Sorrie, MB; Soyiri, IN; Sreeramareddy, CT; Sudaryanto, A; Sufiyan, MB; Suleria, HAR; Sultana, M; Sunguya, BF; Sykes, BL; Tabarés-

Seisdedos, R; Tabuchi, T; Tadesse, DB; Tarigan, IU; Tasew, AA; Tefera, YM; Tekle, MG; Temsah, MH; Tesfay, BE; Tesfay, FH; Tessema, B; Tessema, ZT; Thankappan, KR; Thomas, N; Toma, A; Topor-Madry, R; Tovani-Palone, MR; Traini, E; Tran, BX; Tran, KB; Ullah, I; Unnikrishnan, B; Usman, MS; Uzochukwu, BSC; Valdez, PR; Varughese, S; Violante, FS; Vollmer, S; Hawariat, FGW; Waheed, Y; Wallin, MT; Wang, YF; Wang, YP; Weaver, M; Weji, BG; Weldesamuel, GT; Welgan, CA; Werdecker, A; Westerman, R; Wiangkham, T; Wiysonge, CS; Wolde, HF; Wondafrash, DZ; Wonde, TE; Worku, GT; Wu, AM; Xu, GL; Yadollahpour, A; Jabbari, SHY; Yamada, T; Yatsuya, H; Yeshaneh, A; Yilgwan, CS; Yilma, MT; Yip, P; Yisma, E; Yonemoto, N; Yoon, SJ; Younis, MZ; Yousefifard, M; Yousof, HASA; Yu, CH; Yusefzadeh, H; Zadey, S; Zaidi, Z; Bin Zaman, S; Zamani, M; Zandian, H; Zepro, NB; Zerfu, TA; Zhang, YQ; Zhao, XJG; Ziapour, A; Zodpey, S; Zuniga, YMH; Hay, SI; Reiner, RC

Wiens, Kirsten E.; Lindstedt, Paulina A.; Blacker, Brigette F.; Johnson, Kimberly B.; Baumann, Mathew M.; Schaeffer, Lauren E.; Abbastabar, Hedayat; Abd-Allah, Foad; Abdelalim, Ahmed; Abdollahpour, Ibrahim; Abegaz, Kedir Hussein; Abejie, Ayenew Negesse; Abreu, Lucas Guimaraes; Abrigo, Michael R. M.; Abualhasan, Ahmed; Accrombessi, Manfred Mario Kokou; Acharya, Dilaram; Adabi, Maryam; Adamu, Abdu A.; Adebayo, Oladimeji M.; Adedoyin, Rufus Adesoji; Adekanmbi, Victor; Adetokunboh, Olatunji O.; Adhena, Beyene Meressa; Afarideh, Mohsen; Ahmad, Sohail; Ahmadi, Keivan; Ahmed, Anwar E.; Ahmed, Muktar Beshir; Ahmed, Rushdia; Akalu, Temesgen Yihunie; Alahdab, Fares; Al-Aly, Ziyad; Alam, Noore; Alam, Samiah; Alamene, Genet Melak; Alanzi, Turki M.; Alcalde-Rabanal, Jacqueline Elizabeth; Ali, Beriwan Abdulqadir; Alijanzadeh, Mehran; Alipour, Vahid; Aljunid, Syed Mohamed; Almasi, Ali; Almasi-Hashiani, Amir; Al-Mekhlafi, Hesham M.; Altirkawi, Khalid A.; Alvis-Guzman, Nelson; Alvis-Zakzuk, Nelson J.; Amini, Saeed; Amit, Arianna Maever L.; Andrei, Catalina Liliana; Anjomshoa, Mina; Anoushiravani, Amir; Ansari, Fereshteh; Antonio, Carl Abelardo T.; Antony, Benny; Antriyandarti, Ernoiz; Arabloo, Jalal; Aref, Hany Mohamed Amin; Aremu, Olatunde; Armoon, Bahram; Arora, Amit; Aryal, Krishna K.; Arzani, Afsaneh; Asadi-Aliabadi, Mehran; Atalay, Hagos Tasew; Athari, Seyyed Shamsadin; Athari, Seyyede Masoume; Atre, Sachin R.; Ausloos, Marcel; Awoke, Nefsu; Quintanilla, Beatriz Paulina Ayala; Ayano, Getinet; Ayanore, Martin Amogre; Aynalem, Yared Asmare; Azari, Samad; Azzopardi, Peter S.; Babaee, Ebrahim; Babalola, Tesleem Kayode; Badawi, Alaa; Bairwa, Mohan; Bakkannavar, Shankar M.; Balakrishnan, Senthilkumar; Bali, Ayele Geleto; Banach, Maciej; Banoub, Joseph Adel Mattar; Barac, Aleksandra; Barnighausen, Till Winfried; Basaleem, Huda; Basu, Sanjay; Bay, Vo Dinh; Bayati, Mohsen; Baye, Estifanos; Bedi, Neeraj; Beheshti, Mahya; Behzadifar, Masoud; Behzadifar, Meysam; Bekele, Bayu Begashaw; Belayneh, Yaschilal Muche; Bell, Michelle L.; Bennett, Derrick A.; Berbada, Dessalegn Ajema; Bernstein, Robert S.; Bhat, Anusha Ganapati; Bhattacharyya, Krittika; Bhattarai, Suraj; Bhaumik, Soumyadeep; Bhutta, Zulfiqar A.; Bijani, Ali; Bikbov, Boris; Birihane, Binyam Minuye; Biswas, Raaj Kishore; Bohlouli, Somayeh; Bojia, Hunduma Amensisa; Boufous, Soufiane; Brady, Oliver J.; Bragazzi, Nicola Luigi; Briko, Andrey Nikolaevich; Briko, Nikolay Ivanovich; Britton, Gabrielle B.; Nagaraja, Sharath Burugina; Busse, Reinhard; Butt, Zahid A.; Camera, Luis Alberto; Campos-Nonato, Ismael R.; Cano, Jorge; Car, Josip; Cardenas, Rosario; Carvalho, Felix; Castaneda-Orjuela, Carlos A.; Castro, Franz;

Chanie, Wagaye Fentahun; Chatterjee, Pranab; Chattu, Vijay Kumar; Chichiabellu, Tesfaye Yitna; Chin, Ken Lee; Christopher, Devasahayam J.; Chu, Dinh-Toi; Cormier, Natalie Maria; Costa, Vera Marisa; Culquichicon, Carlos; Daba, Matiwos Soboka; Damiani, Giovanni; Dandona, Lalit; Dandona, Rakhi; Dang, Anh Kim; Darwesh, Aso Mohammad; Darwish, Amira Hamed; Daryani, Ahmad; Das, Jai K.; Das Gupta, Rajat; Dash, Aditya Prasad; Davey, Gail; Davila-Cervantes, Claudio Alberto; Davis, Adrian C.; Davitoiu, Dragos Virgil; De la Hoz, Fernando Pio; Demis, Asmamaw Bizuneh; Demissie, Dereje Bayissa; Demissie, Getu Debalkie; Demoz, Gebre Teklemariam; Demoz, Gebre Teklemariam; Denova-Gutierrez, Edgar; Deribe, Kebede; Desalew, Assefa; Deshpande, Aniruddha; Dharmaratne, Samath Dhamminda; Dhillon, Preeti; Dhimal, Meghnath; Dhungana, Govinda Prasad; Diaz, Daniel; Dipeolu, Isaac Oluwafemi; Djalalinia, Shirin; Doyle, Kerrie E.; Dubljanin, Eleonora; Duko, Bereket; Duraes, Andre Rodrigues; Kalan, Mohammad Ebrahimi; Edinur, Hisham Atan; Effiong, Andem; Eftekhari, Aziz; El Nahas, Nevine; El Sayed, Iman; Zaki, Maysaa El Sayed; El Tantawi, Maha; Elema, Teshome Bekele; Elhabashy, Hala Rashad; El-Jaafary, Shaimaa I.; Elkout, Hajer; Elsharkawy, Aisha; Elyazar, Iqbal R. F.; Endalamaw, Aklilu; Endalew, Daniel Adane; Eskandarieh, Sharareh; Esteghamati, Alireza; Esteghamati, Sadaf; Etemadi, Arash; Ezekannagha, Oluchi; Fareed, Mohammad; Faridnia, Roghiyeh; Farzadfar, Farshad; Fazlzadeh, Mehdi; Feigin, Valery L.; Fereshtehnejad, Seyed-Mohammad; Fernandes, Eduarda; Filip, Irina; Fischer, Florian; Foigt, Nataliya A.; Folayan, Morenike Oluwatoyin; Foroutan, Masoud; Franklin, Richard Charles; Fukumoto, Takeshi; Gad, Mohamed M.; Gayesa, Reta Tsegaye; Gebre, Teshome; Gebremedhin, Ketema Bizuwork; Gebremeskel, Gebreamlak Gebremedhn; Gesesew, Hailay Abrha; Gezae, Kebede Embaye; Ghadiri, Keyghobad; Ghashghaee, Ahmad; Ghimire, Pramesh Raj; Gill, Paramjit Singh; Gill, Tiffany K.; Ginindza, Themba G.; Gomes, Nelson G. M.; Gopalani, Sameer Vali; Goulart, Alessandra C.; Goulart, Barbara Niegia Garcia; Grada, Ayman; Gubari, Mohammed Ibrahim Mohialdeen; Gugnani, Harish Chander; Guido, Davide; Guimaraes, Rafael Alves; Guo, Yuming; Gupta, Rajeev; Hafezi-Nejad, Nima; Haile, Dessalegn H.; Hailu, Gessessew Bugssa; Haj-Mirzaian, Arvin; Haj-Mirzaian, Arya; Hamadeh, Randah R.; Hamidi, Samer; Handiso, Demelash Woldeyohannes; Haririan, Hamidreza; Hariyani, Ninuk; Hasaballah, Ahmed I.; Hasan, Md Mehedi; Hasanpoor, Edris; Hasanzadeh, Amir; Hassankhani, Hadi; Hassen, Hamid Yimam; Hegazy, Mohamed I.; Heibati, Behzad; Heidari, Behnam; Hendrie, Delia; Henry, Nathaniel J.; Herteliu, Claudiu; Heydarpour, Fatemeh; de Hidru, Hagos Degefa; Hird, Thomas R.; Hoang, Chi Linh; Rad, Enayatollah Homaie; Hoogar, Praveen; Hoseini, Mohammad; Hossain, Naznin; Hosseini, Mostafa; Hosseinzadeh, Mehdi; Househ, Mowafa; Hsairi, Mohamed; Hu, Guoqing; Hussen, Mohammedaman Mama; Ibitoye, Segun Emmanuel; Igumbor, Ehimario U.; Ilesanmi, Olayinka Stephen; Ilic, Milena D.; Imani-Nasab, Mohammad Hasan; Iqbal, Usman; Irvani, Seyed Sina Naghibi; Islam, Sheikh Mohammed Shariful; Iwu, Chinwe Juliana; Izadi, Neda; Jaca, Anelisa; Jahanmehr, Nader; Jakovljevic, Mihajlo; Jalali, Amir; Jayatilleke, Achala Upendra; Jha, Ravi Prakash; Jha, Vivekanand; Ji, John S.; Jonas, Jost B.; Jozwiak, Jacek Jerzy; Kabir, Ali; Kabir, Zubair; Kahsay, Amaha; Kalani, Hamed; Kanchan, Tanuj; Matin, Behzad Karami; Karch, Andre; Karim, Mohd Anisul; Karki, Hamidreza Karimi-Sari Surendra; Kasaeian, Amir; Kasahun, Gebremicheal Gebreslassie; Kasahun, Yawukal Chane; Kasaye, Habtamu Kebebe; Kassa, Gebrehiwot G.; Kassa, Getachew

Mullu; Kayode, Gbenga A.; Karyani, Ali Kazemi; Kebede, Mihiretu M.; Keiyoro, Peter Njenga; Kelbore, Abraham Getachew; Kengne, Andre Pascal; Ketema, Daniel Bekele; Khader, Yousef Saleh; Khafaie, Morteza Abdullatif; Khalid, Nauman; Khalilov, Rovshan; Khan, Ejaz Ahmad; Khan, Junaid; Khan, Md Nuruzzaman; Khan, Muhammad Shahzeb; Khatab, Khaled; Khater, Amir M.; Khater, Mona M.; Khayamzadeh, Maryam; Khazaei, Mohammad; Khazaei, Salman; Khosravi, Mohammad Hossein; Khubchandani, Jagdish; Kiadaliri, Ali; Kim, Yun Jin; Kimokoti, Ruth W.; Kisa, Adnan; Kisa, Sezer; Kissoon, Niranjan; Shivakumar, K. M.; Kochhar, Sonali; Kolola, Tufa; Komaki, Hamidreza; Kosen, Soewarta; Koul, Parvaiz A.; Koyanagi, Ai; Kraemer, Moritz U. G.; Krishan, Kewal; Kugbey, Nuworza; Kumar, G. Anil; Kumar, Manasi; Kumar, Pushpendra; Kusuma, Vivek Kumar Dian; La Vecchia, Carlo; Lacey, Ben; Lad, Sheetal D.; Lal, Dharmesh Kumar; Lam, Felix; Lami, Faris Hasan; Lamichhane, Prabhat; Lansingh, Van Charles; Lasrado, Savita; Laxmaiah, Avula; Lee, Paul H.; LeGrand, Kate E.; Leili, Mostafa; Lenjebo, Tsegaye Lolaso; Leshargie, Cheru Tesema; Levine, Aubrey J.; Li, Shanshan; Linn, Shai; Liu, Shiwei; Liu, Simin; Lodha, Rakesh; Longbottom, Joshua; Lopez, Jaifred Christian F.; Abd El Razek, Hassan Magdy; Abd El Razek, Muhammed Magdy; Prasad, D. R. Mahadeshwara; Mahasha, Phetole Walter; Mahotra, Narayan B.; Majeed, Azeem; Malekzadeh, Reza; Malta, Deborah Carvalho; Mamun, Abdullah A.; Manafi, Navid; Manda, Ana Laura; Manohar, Narendar Dawani Dawanu; Mansournia, Mohammad Ali; Mapoma, Chabila Christopher; Maravilla, Joemer C.; Martinez, Gabriel; Martini, Santi; Martins-Melo, Francisco Rogerlandio; Masaka, Anthony; Massenburg, Benjamin Ballard; Mathur, Manu Raj; Mayala, Benjamin K.; Mazidi, Mohsen; McAlinden, Colm; Meharie, Birhanu Geta; Mehndiratta, Man Mohan; Mehta, Kala M.; Mekonnen, Tefera Chane; Meles, Gebrekiros Gebremichael; Memiah, Peter T. N.; Memish, Ziad A.; Mendoza, Walter; Menezes, Ritesh G.; Mereta, Seid Tiku; Meretoja, Tuomo J.; Mestrovic, Tomislav; Miazgowski, Bartosz; Mihretie, Kebadnew Mulatu; Miller, Ted R.; Mini, G. K.; Mirrakhimov, Erkin M.; Moazen, Babak; Mohajer, Bahram; Mohamadi-Bolbanabad, Amjad; Mohammad, Dara K.; Mohammad, Karzan Abdulmuhsin; Mohammad, Yousef; Mezerji, Naser Mohammad Gholi; Mohammadibakhsh, Roghayeh; Mohammadifard, Noushin; Mohammed, Jemal Abdu; Mohammed, Shafiu; Mohebi, Farnam; Mokdad, Ali H.; Molokhia, Mariam; Monasta, Lorenzo; Moodley, Yoshan; Moore, Catrin E.; Moradi, Ghobad; Moradi, Masoud; Moradi-Joo, Mohammad; Moradi-Lakeh, Maziar; Moraga, Paula; Morales, Linda; Velasquez, Ilais Moreno; Mosapour, Abbas; Mouodi, Simin; Mousavi, Seyyed Meysam; Mozaffor, Miliva; Muchie, Kindie Fentahun; Mulaw, Getahun Fentaw; Munro, Sandra B.; Muriithi, Moses K.; Murray, Christoper J. L.; Murthy, G. V. S.; Musa, Kamarul Imran; Mustafa, Ghulam; Muthupandian, Saravanan; Nabhan, Ashraf F.; Naderi, Mehdi; Nagarajan, Ahamarshan Jayaraman; Naidoo, Kovin S.; Naik, Gurudatta; Najafi, Farid; Nangia, Vinay; Nansseu, Jobert Richie; Nascimento, Bruno Ramos; Nazari, Javad; Ndwandwe, Duduzile Edith; Negoi, Ionut; Netsere, Henok Biresaw; Ngunjiri, Josephine W.; Nguyen, Cuong Tat; Nguyen, Huong Lan Thi; Nguyen, Trang Huyen; Nigatu, Dabere; Nigatu, Solomon Gedlu; Ningrum, Dina Nur Anggraini; Nnaji, Chukwudi A.; Nojomi, Marzieh; Nong, Vuong Minh; Norheim, Ole F.; Noubiap, Jean Jacques; Motlagh, Soraya Nouraei; Oancea, Bogdan; Ogah, Okechukwu Samuel; Ogbo, Felix Akpojene; Oh, In-Hwan; Olagunju, Andrew T.; Olagunju, Tinuke O.; Olusanya, Bolajoko Olubukunola; Olusanya, Jacob Olusegun; Onwujekwe, Obinna E.; Oren, Eyal; Ortega-Altamirano, Doris V.; Osarenotor,

Osayomwanbo; Osei, Frank B.; Owolabi, Mayowa O.; Mahesh, P. A.; Padubidri, Jagadish Rao; Pakhale, Smita; Patel, Sangram Kishor; Paternina-Caicedo, Angel J.; Pathak, Ashish; Patton, George C.; Paudel, Deepak; Paulos, Kebreab; Pepito, Veincent Christian Filipino; Pereira, Alexandre; Perico, Norberto; Pervaiz, Aslam; Pescarini, Julia Moreira; Piroozi, Bakhtiar; Pirsaheb, Meghdad; Postma, Maarten J.; Pourjafar, Hadi; Pourmalek, Farshad; Pourshams, Akram; Poustchi, Hossein; Prada, Sergio I.; Prasad, Narayan; Preotescu, Liliana; Quintana, Hedley; Rabiee, Navid; Radfar, Amir; Rafiei, Alireza; Rahim, Fakher; Rahimi-Movaghar, Afarin; Rahimi-Movaghar, Vafa; Rahman, Mohammad Hifz Ur; Rahman, Muhammad Aziz; Rahman, Shafiur; Rajati, Fatemeh; Rana, Saleem Muhammad; Ranabhat, Chhabi Lal; Rasella, Davide; Rawaf, David Laith; Rawaf, Salman; Rawal, Lal; Rawasia, Wasiq Faraz; Renjith, Vishnu; Renzaho, Andre M. N.; Resnikoff, Serge; Reta, Melese Abate; Rezaei, Negar; Rezai, Mohammad Sadegh; Riahi, Seyed Mohammad; Ribeiro, Ana Isabel; Rickard, Jennifer; Rios-Blancas, Maria; Roever, Leonardo; Ronfani, Luca; Roro, Elias Merdassa; Ross, Jennifer M.; Rubagotti, Enrico; Rubino, Salvatore; Saad, Anas M.; Sabde, Yogesh Damodar; Sabour, Siamak; Sadeghi, Ehsan; Safari, Yahya; Safari-Faramani, Roya; Sagar, Rajesh; Sahebkar, Amirhossein; Sahraian, Mohammad Ali; Sajadi, S. Mohammad; Salahshoor, Mohammad Reza; Salam, Nasir; Salamati, Payman; Salem, Hosni; Salem, Marwa Rashad; Salimi, Yahya; Salimzadeh, Hamideh; Samy, Abdallah M.; Sanabria, Juan; Santric-Milicevic, Milena M.; Jose, Bruno Piassi Sao; Saraswathy, Sivan Yegnanarayana Iyer; Sarkar, Kaushik; Sarker, Abdur Razzaque; Sarrafzadegan, Nizal; Sartorius, Benn; Sathian, Brijesh; Sathish, Thirunavukkarasu; Sawhney, Monika; Saxena, Sonia; Schwebel, David C.; Senbeta, Anbissa Muleta; Senthilkumaran, Subramanian; Sepanlou, Sadaf G.; Servan-Mori, Edson; Shabaninejad, Hosein; Shafieesabet, Azadeh; Shaikh, Masood Ali; Shalash, Ali S.; Shallo, Seifadin Ahmed; Shams-Beyranvand, Mehran; Shamsi, MohammadBagher; Shamsizadeh, Morteza; Shannawaz, Mohammed; Sharafi, Kiomars; Sharifi, Hamid; Shehata, Hatem Samir; Sheikh, Aziz; Shetty, B. Suresh Kumar; Shibuya, Kenji; Shiferaw, Wondimeneh Shibabaw; Shifti, Desalegn Markos; Shigematsu, Mika; Shin, Jae Il; Shiri, Rahman; Shirkoohi, Reza; Siabani, Soraya; Siddiqi, Tariq Jamal; Silva, Diego Augusto Santos; Singh, Ambrish; Singh, Jasvinder A.; Singh, Narinder Pal; Singh, Virendra; Sisay, Malede Mequanent; Skiadaresi, Eirini; Sobhiyeh, Mohammad Reza; Sokhan, Anton; Soltani, Shahin; Somayaji, Ranjani; Soofi, Moslem; Sorrie, Muluken Bekele; Soyiri, Ireneous N.; Sreeramareddy, Chandrashekhar T.; Sudaryanto, Agus; Sufiyan, Mu'awiyyah Babale; Suleria, Hafiz Ansar Rasul; Sultana, Marufa; Sunguya, Bruno Fokas; Sykes, Bryan L.; Tabares-Seisdedos, Rafael; Tabuchi, Takahiro; Tadesse, Degena Bahrey; Tarigan, Ingan Ukur; Tasew, Aberash Abay; Tefera, Yonatal Mesfin; Tekle, Merhawi Gebremedhin; Temsah, Mohamad-Hani; Tesfay, Berhe Etsay; Tesfay, Fisaha Haile; Tessema, Belay; Tessema, Zemenu Tadesse; Thankappan, Kavumpurathu Raman; Thomas, Nihal; Toma, Alemayehu; Topor-Madry, Roman; Tovani-Palone, Marcos Roberto; Traini, Eugenio; Tran, Bach Xuan; Tran, Khanh Bao; Ullah, Irfan; Unnikrishnan, Bhaskaran; Usman, Muhammad Shariq; Uzochukwu, Benjamin S. Chudi; Valdez, Pascual R.; Varughese, Santosh; Violante, Francesco S.; Vollmer, Sebastian; Hawariat, Feleke Gebremeskel W.; Waheed, Yasir; Wallin, Mitchell Taylor; Wang, Yafeng; Wang, Yuan-Pang; Weaver, Marcia; Weji, Bedilu Girma; Weldesamuel, Girmay Teklay; Welgan, Catherine A.; Werdecker, Andrea; Westerman, Ronny; Wiangkham, Taweewat;

Wiysonge, Charles Shey; Wolde, Haileab Fekadu; Wondafrash, Dawit Zewdu; Wonde, Tewodros Eshete; Worku, Getasew Taddesse; Wu, Ai-Min; Xu, Gelin; Yadollahpour, Ali; Jabbari, Seyed Hossein Yahyazadeh; Yamada, Tomohide; Yatsuya, Hiroshi; Yeshaneh, Alex; Yilgwan, Christopher Sabo; Yilma, Mekdes Tigistu; Yip, Paul; Yisma, Engida; Yonemoto, Naohiro; Yoon, Seok-Jun; Younis, Mustafa Z.; Yousefifard, Mahmoud; Yousof, Hebat-Allah Salah A.; Yu, Chuanhua; Yusefzadeh, Hasan; Zadey, Siddhesh; Zaidi, Zoubida; Bin Zaman, Sojib; Zamani, Mohammad; Zandian, Hamed; Zepro, Nejimu Biza; Zerfu, Taddese Alemu; Zhang, Yunquan; Zhao, Xiu-Ju George; Ziapour, Arash; Zodpey, Sanjay; Zuniga, Yves Miel H.; Hay, Simon I.; Reiner, Robert C., Jr. Local Burden Dis Diarrhoea

Mapping geographical inequalities in oral rehydration therapy coverage in low-income and middle-income countries, 2000-17 LANCET GLOBAL HEALTH English Article

DIARRHEAL DISEASE; CHILDREN; CHOLERA; AFRICA; MORBIDITY; MORTALITY; DEATHS; ZINC

Background Oral rehydration solution (ORS) is a form of oral rehydration therapy (ORT) for diarrhoea that has the potential to drastically reduce child mortality; yet, according to UNICEF estimates, less than half of children younger than 5 years with diarrhoea in low-income and middle-income countries (LMICs) received ORS in 2016. A variety of recommended home fluids (RHF) exist as alternative forms of ORT; however, it is unclear whether RHF prevent child mortality. Previous studies have shown considerable variation between countries in ORS and RHF use, but subnational variation is unknown. This study aims to produce high-resolution geospatial estimates of relative and absolute coverage of ORS, RHF, and ORT (use of either ORS or RHF) in LMICs. Methods We used a Bayesian geostatistical model including 15 spatial covariates and data from 385 household surveys across 94 LMICs to estimate annual proportions of children younger than 5 years of age with diarrhoea who received ORS or RHF (or both) on continuous continent-wide surfaces in 2000-17, and aggregated results to policy-relevant administrative units. Additionally, we analysed geographical inequality in coverage across administrative units and estimated the number of diarrhoeal deaths averted by increased coverage over the study period. Uncertainty in the mean coverage estimates was calculated by taking 250 draws from the posterior joint distribution of the model and creating uncertainty intervals (UIs) with the 2 center dot 5th and 97 center dot 5th percentiles of those 250 draws. Findings While ORS use among children with diarrhoea increased in some countries from 2000 to 2017, coverage remained below 50% in the majority (62 center dot 6%; 12 417 of 19 823) of second administrative-level units and an estimated 6 519 000 children (95% UI 5 254 000-7 733 000) with diarrhoea were not treated with any form of ORT in 2017. Increases in ORS use corresponded with declines in RHF in many locations, resulting in relatively constant overall ORT coverage from 2000 to 2017. Although ORS was uniformly distributed subnationally in some countries, within-country geographical inequalities persisted in others; 11 countries had at least a 50% difference in one of their units compared with the country mean. Increases in ORS use over time were correlated with declines in RHF use and in diarrhoeal mortality in many locations, and an estimated 52 230 diarrhoeal deaths (36 910-68 860) were averted by scaling up of ORS coverage between 2000 and 2017. Finally, we identified key subnational areas in Colombia, Nigeria, and Sudan as examples of where

diarrhoeal mortality remains higher than average, while ORS coverage remains lower than average. Interpretation To our knowledge, this study is the first to produce and map subnational estimates of ORS, RHF, and ORT coverage and attributable child diarrhoeal deaths across LMICs from 2000 to 2017, allowing for tracking progress over time. Our novel results, combined with detailed subnational estimates of diarrhoeal morbidity and mortality, can support subnational needs assessments aimed at furthering policy makers' understanding of within-country disparities. Over 50 years after the discovery that led to this simple, cheap, and life-saving therapy, large gains in reducing mortality could still be made by reducing geographical inequalities in ORS coverage. Copyright (c) 2020 The Author(s). Published by Elsevier Ltd. This is an Open Access article under the CC BY 4.0 license.[Wiens, Kirsten E.; Lindstedt, Paulina A.; Blacker, Brigette F.; Johnson, Kimberly B.; Baumann, Mathew M.; Schaeffer, Lauren E.; Cormier, Natalie Maria; Dandona, Lalit; Dandona, Rakhi; Deshpande, Aniruddha; Dharmaratne, Samath Dhamminda; Feigin, Valery L.; Henry, Nathaniel J.; LeGrand, Kate E.; Levine, Aubrey J.; Mayala, Benjamin K.; Mokdad, Ali H.; Munro, Sandra B.; Murray, Christoper J. L.; Ross, Jennifer M.; Weaver, Marcia; Welgan, Catherine A.; Hay, Simon I.; Reiner, Robert C., Jr.] Univ Washington, Inst Hlth Metr & Evaluat, Seattle, WA 98195 USA; [Dandona, Rakhi; Dharmaratne, Samath Dhamminda; Mokdad, Ali H.; Murray, Christoper J. L.; Sartorius, Benn; Weaver, Marcia; Hay, Simon I.; Reiner, Robert C., Jr.] Univ Washington, Sch Med, Dept Hlth Metr Sci, Seattle, WA USA; [Kochhar, Sonali; Ross, Jennifer M.] Univ Washington, Dept Global Hlth, Seattle, WA 98195 USA; [Massenburg, Benjamin Ballard] Univ Washington, Div Plast & Reconstruct Surg, Seattle, WA 98195 USA; [Ross, Jennifer M.; Somayaji, Ranjani] Univ Washington, Dept Med, Seattle, WA USA; [Oren, Eyal] Univ Washington, Seattle, WA 98195 USA; [Abbastabar, Hedayat] Univ Tehran Med Sci, Adv Diagnost & Intervent Radiol Res Ctr, Tehran, Iran; [Afarideh, Mohsen; Esteghamati, Alireza; Esteghamati, Sadaf; Heidari, Behnam; Rezaei, Negar] Univ Tehran Med Sci, Endocrinol & Metab Res Ctr, Tehran, Iran; [Anoushiravani, Amir; Malekzadeh, Reza; Pourshams, Akram; Poustchi, Hossein; Salimzadeh, Hamideh; Sepanlou, Sadaf G.] Univ Tehran Med Sci, Digest Dis Res Inst, Tehran, Iran; [Eskandarieh, Sharareh; Mohajer, Bahram; Sahraian, Mohammad Ali] Univ Tehran Med Sci, Multiple Sclerosis Res Ctr, Tehran, Iran; [Farzadfar, Farshad; Mohajer, Bahram; Mohebi, Farnam; Rezaei, Negar] Univ Tehran Med Sci, Noncommunicable Dis Res Ctr, Tehran, Iran; [Fazlzadeh, Mehdi] Univ Tehran Med Sci, Dept Environm Hlth Engn, Tehran, Iran; [Hafezi-Nejad, Nima] Univ Tehran Med Sci, Sch Med, Tehran, Iran; [Haj-Mirzaian, Arvin] Univ Tehran Med Sci, Dept Pharmacol, Tehran, Iran; [Hasanzadeh, Amir] Univ Tehran Med Sci, Dept Microbiol, Tehran, Iran; [Hosseini, Mostafa; Mansournia, Mohammad Ali] Univ Tehran Med Sci, Dept Epidemiol & Biostat, Tehran, Iran; [Hosseini, Mostafa] Univ Tehran Med Sci, Pediat Chron Kidney Dis Res Ctr, Tehran, Iran; [Kasaeian, Amir] Univ Tehran Med Sci, Hematol Oncol & Stem Cell Transplantat Res Ctr, Tehran, Iran; [Mohebi, Farnam] Univ Tehran Med Sci, Natl Inst Hlth Res, Tehran, Iran; [Mousavi, Seyyed Meysam] Univ Tehran Med Sci, Dept Hlth Policy Management & Econ, Tehran, Iran; [Rahim, Fakher] Univ Tehran Med Sci, Metabol & Genom Res Ctr, Tehran, Iran; [Rahimi-Movaghar, Afarin] Univ Tehran Med Sci, Iranian Natl Ctr Addict Studies, Tehran, Iran; [Rahimi-Movaghar, Vafa; Salamati, Payman] Univ Tehran Med Sci, Sina Trauma

& Surg Res Ctr, Tehran, Iran; [Shirkoohi, Reza] Univ Tehran Med Sci, Canc Res Inst, Tehran, Iran; [Shirkoohi, Reza] Univ Tehran Med Sci, Canc Biol Res Ctr, Tehran, Iran; [Etemadi, Arash] Univ Tehran Med Sci, Tehran, Iran; [Abd-Allah, Foad; Abdelalim, Ahmed; Abualhasan, Ahmed; El-Jaafary, Shaimaa I.; Hegazy, Mohamed I.; Shehata, Hatem Samir] Cairo Univ, Dept Neurol, Cairo, Egypt; [Elhabashy, Hala Rashad] Cairo Univ, Neurophysiol Dept, Cairo, Egypt; [Elsharkawy, Aisha] Cairo Univ, Endem Med & Hepatogastroentrol Dept, Cairo, Egypt; [Khater, Amir M.] Cairo Univ, Natl Hepatol & Trop Med Res Inst, Cairo, Egypt; [Khater, Mona M.; Yousof, Hebat-Allah Salah A.] Cairo Univ, Dept Med Parasitol, Cairo, Egypt; [Salem, Hosni] Cairo Univ, Urol Dept, Cairo, Egypt; [Abdollahpour, Ibrahim] Isfahan Univ Med Sci, Neurosci Res Ctr, Esfahan, Iran; [Mohammadifard, Noushin; Sarrafzadegan, Nizal] Isfahan Univ Med Sci, Isfahan Cardiovasc Res Inst, Esfahan, Iran; [Abegaz, Kedir Hussein] Near East Univ, Dept Biostat, Nicosia, Cyprus; [Abegaz, Kedir Hussein] Madda Walabu Univ, Dept Biostat & Hlth Informat, Bale Robe, Ethiopia; [Alamene, Genet Melak] Madda Walabu Univ, Dept Publ Hlth, Bale Robe, Ethiopia; [Hussen, Mohammedaman Mama] Madda Walabu Univ, Dept Med Lab Sci, Bale Robe, Ethiopia; [Haile, Dessalegn H.] Debre Markos Univ, Dept Nursing, Debre Markos, Ethiopia; [Kassa, Getachew Mullu] Debre Markos Univ, Coll Hlth Sci, Debre Markos, Ethiopia; [Ketema, Daniel Bekele; Wonde, Tewodros Eshete] Debre Markos Univ, Dept Publ Hlth, Debre Markos, Ethiopia; [Abejie, Ayenew Negesse] Debre Markos Univ, Debre Markos, Ethiopia; [Abreu, Lucas Guimaraes] Univ Fed Minas Gerais, Dept Pediat Dent, Belo Horizonte, MG, Brazil; [Malta, Deborah Carvalho] Univ Fed Minas Gerais, Dept Maternal & Child Nursing & Publ Hlth, Belo Horizonte, MG, Brazil; [Nascimento, Bruno Ramos] Univ Fed Minas Gerais, Dept Clin Med, Belo Horizonte, MG, Brazil; [Nascimento, Bruno Ramos] Univ Fed Minas Gerais, Clin Hosp, Belo Horizonte, MG, Brazil; [Jose, Bruno Piassi Sao] Univ Fed Minas Gerais, Dept Infect Dis & Trop Med, Belo Horizonte, MG, Brazil; [Abrigo, Michael R. M.] Philippine Inst Dev Studies, Dept Res, Quezon City, Philippines; [Accrombessi, Manfred Mario Kokou; Cano, Jorge] London Sch Hyg & Trop Med, Dept Dis Control, London, England; [Brady, Oliver J.] London Sch Hyg & Trop Med, Dept Infect Dis Epidemiol, London, England; [Sartorius, Benn] London Sch Hyg & Trop Med, Fac Infect & Trop Dis, London, England; [Accrombessi, Manfred Mario Kokou] Fdn Sci Res, Clin Res & Operat, Cotonou, Benin; [Acharya, Dilaram] Dongguk Univ, Dept Prevent Med, Gyeongju, South Korea; [Acharya, Dilaram] Kathmandu Univ, Dept Community Med, Devdaha, Nepal; [Khazaei, Mohammad; Leili, Mostafa] Hamadan Univ Med Sci, Dept Environm Hlth Engn, Hamadan, Iran; [Khazaei, Salman] Hamadan Univ Med Sci, Dept Epidemiol, Hamadan, Iran; [Komaki, Hamidreza] Hamadan Univ Med Sci, Neurophysiol Res Ctr, Hamadan, Iran; [Mezerji, Naser Mohammad Gholi] Hamadan Univ Med Sci, Dept Biostat, Hamadan, Iran; [Adabi, Maryam; Mohammadibakhsh, Roghayeh] Hamadan Univ Med Sci, Hamadan, Iran; [Adamu, Abdu A.; Adetokunboh, Olatunji O.; Iwu, Chinwe Juliana] Stellenbosch Univ, Dept Global Hlth, Cape Town, South Africa; [Jaca, Anelisa] Stellenbosch Univ, Ctr Evidence Based Hlth Care, Cape Town, South Africa; [Adamu, Abdu A.; Ndwandwe, Duduzile Edith] South African Med Res Council, Cochrane South Africa, Cape Town, South Africa; [Mahasha, Phetole Walter] South African Med Res Council, Grants Innovat & Product Dev Unit, Cape Town, South Africa; [Iwu, Chinwe Juliana; Jaca, Anelisa; Nnaji, Chukwudi A.; Wiysonge, Charles Shey] South African

Med Res Council, Cape Town, South Africa; [Adebayo, Oladimeji M.] Univ Coll Hosp, Coll Med, Ibadan, Nigeria; [Ilesanmi, Olayinka Stephen] Univ Coll Hosp, Dept Community Med, Ibadan, Nigeria; [Owolabi, Mayowa O.] Univ Coll Hosp, Dept Med, Ibadan, Nigeria; [Adedoyin, Rufus Adesoji] Obafemi Awolowo Univ, Dept Med Rehabil, Ife, Nigeria; [Folayan, Morenike Oluwatoyin] Obafemi Awolowo Univ, Dept Child Dent Hlth, Ife, Nigeria; [Adekanmbi, Victor] Cardiff Univ, Sch Med, Cardiff, Wales; [Adetokunboh, Olatunji O.] Stellenbosch Univ, Ctr Excellence Epidemiol Modelling & Anal, Stellenbosch, South Africa; [Adhena, Beyene Meressa; Meles, Gebrekiros Gebremichael; Tesfay, Fisaha Haile] Mekelle Univ, Sch Publ Hlth, Mekelle, Ethiopia; [Gebremeskel, Gebreamlak Gebremedhn] Mekelle Univ, Dept Nursing, Mekelle, Ethiopia; [Gesesew, Hailay Abrha] Mekelle Univ, Dept Epidemiol, Mekelle, Ethiopia; [Gezae, Kebede Embaye; Tasew, Aberash Abay] Mekelle Univ, Dept Biostat, Mekelle, Ethiopia; [Hailu, Gessessew Bugssa] Mekelle Univ, Dept Med Parasitol & Entomol, Mekelle, Ethiopia; [Kahsay, Amaha] Mekelle Univ, Dept Nutr & Dietet, Mekelle, Ethiopia; [Muthupandian, Saravanan] Mekelle Univ, Dept Microbiol & Immunol, Mekelle, Ethiopia; [Wondafrash, Dawit Zewdu] Mekelle Univ, Dept Pharmacol & Toxicol, Mekelle, Ethiopia; [Afarideh, Mohsen] Mayo Clin, Dept Dermatol, Rochester, MN USA; [Ahmad, Sohail] Mahsa Univ, Fac Pharm, Kuala Langat, Malaysia; [Ahmadi, Keivan] Univ Nottingham, Lincoln Med Sch, Lincoln, England; [Ahmadi, Keivan] Univ Lincoln, Lincoln Med Sch, Lincoln, England; [Ahmed, Anwar E.] Univ Gezira, Econ & Rural Dev Dept, Wad Madani, Sudan; [Ahmed, Muktar Beshir] Jimma Univ, Dept Epidemiol, Jimma, Ethiopia; [Daba, Matiwos Soboka] Jimma Univ, Dept Psychiat, Jimma, Ethiopia; [Demis, Asmamaw Bizuneh] Jimma Univ, Sch Nursing, Jimma, Ethiopia; [Mereta, Seid Tiku] Jimma Univ, Dept Environm Hlth Sci & Technol, Jimma, Ethiopia; [Ahmed, Muktar Beshir] Univ South Australia, Australian Ctr Precis Hlth, Adelaide, SA, Australia; [Ahmed, Rushdia; Das Gupta, Rajat] BRAC Univ, James P Grant Sch Publ Hlth, Dhaka, Bangladesh; [Ahmed, Rushdia] Int Ctr Diarrhoeal Dis Res, Hlth Syst & Populat Studies Div, Dhaka, Bangladesh; [Sultana, Marufa] Int Ctr Diarrhoeal Dis Res, Nutr & Clin Serv Div, Dhaka, Bangladesh; [Bin Zaman, Sojib] Int Ctr Diarrhoeal Dis Res, Maternal & Child Hlth Div, Dhaka, Bangladesh; [Akalu, Temesgen Yihunie; Nigatu, Solomon Gedlu; Sisay, Malede Mequanent; Tessema, Zemenu Tadesse; Wolde, Haileab Fekadu] Univ Gondar, Dept Epidemiol & Biostat, Gondar, Ethiopia; [Chanie, Wagaye Fentahun; Demissie, Getu Debalkie] Univ Gondar, Inst Publ Hlth, Gondar, Ethiopia; [Netsere, Henok Biresaw] Univ Gondar, Sch Nursing, Gondar, Ethiopia; [Tessema, Belay] Univ Gondar, Dept Med Microbiol, Gondar, Ethiopia; [Alahdab, Fares] Mayo Clin Fdn Med Educ & Res, Mayo Evidence Based Practice Ctr, Rochester, MN USA; [Al-Aly, Ziyad] Washington Univ, John T Milliken Dept Internal Med, St Louis, MO 63110 USA; [Al-Aly, Ziyad] Dept Vet Affairs, Clin Epidemiol Ctr, St Louis, MO USA; [Alam, Noore] Queensland Hlth, Prevent Div, Brisbane, Qld, Australia; [Alam, Noore] Griffith Univ, Ctr Environm & Populat Hlth, Nathan, Qld, Australia; [Alam, Samiah] Dalhousie Univ, Community Hlth & Epidemiol, Halifax, NS, Canada; [Alanzi, Turki M.] Imam Abdulrahman Bin Faisal Univ, Hlth Informat Management & Technol Dept, Dammam, Saudi Arabia; [Menezes, Ritesh G.] Imam Abdulrahman Bin Faisal Univ, Forens Med Div, Dammam, Saudi Arabia; [Alcalde-Rabanal, Jacqueline Elizabeth] Natl Inst Publ Hlth, Ctr Hlth Syst Res, Cuernavaca, Morelos, Mexico; [Campos-Nonato, Ismael R.] Natl Inst Publ Hlth, Hlth &

Nutr Res Ctr, Cuernavaca, Morelos, Mexico; [Denova-Gutierrez, Edgar] Natl Inst Publ Hlth, Ctr Nutr & Hlth Res, Cuernavaca, Morelos, Mexico; [Morales, Linda] Natl Inst Publ Hlth, Ctr Populat Hlth Res, Cuernavaca, Morelos, Mexico; [Rios-Blancas, Maria; Servan-Mori, Edson] Natl Inst Publ Hlth, Ctr Hlth Syst Res, Cuernavaca, Morelos, Mexico; [Ortega-Altamirano, Doris V.] Natl Inst Publ Hlth, Hlth Syst Res Ctr, Cuernavaca, Morelos, Mexico; [Ali, Beriwan Abdulqadir] Erbil Polytech Univ, Erbil Tech Hlth Coll, Erbil, Iraq; [Ali, Beriwan Abdulqadir] Tishk Int Univ, Sch Pharm, Erbil, Iraq; [Mohammad, Karzan Abdulmuhsin] Tishk Int Univ, Erbil, Iraq; [Alijanzadeh, Mehran] Qazvin Univ Med Sci, Social Determinants Hlth Res Ctr, Qazvin, Iran; [Alipour, Vahid; Arabloo, Jalal; Azari, Samad; Ghashghaee, Ahmad] Iran Univ Med Sci, Hlth Management & Econ Res Ctr, Tehran, Iran; [Alipour, Vahid] Iran Univ Med Sci, Hlth Econ Dept, Tehran, Iran; [Asadi-Aliabadi, Mehran; Babaee, Ebrahim; Moradi-Lakeh, Maziar; Nojomi, Marzieh] Iran Univ Med Sci, Prevent Med & Publ Hlth Res Ctr, Tehran, Iran; [Ghashghaee, Ahmad] Iran Univ Med Sci, Student Res Comm, Tehran, Iran; [Kabir, Ali] Iran Univ Med Sci, Minimally Invas Surg Res Ctr, Tehran, Iran; [Kasaeian, Amir] Iran Univ Med Sci, Pars Adv & Minimally Invas Med Manners Res Ctr, Tehran, Iran; [Khosravi, Mohammad Hossein] Iran Univ Med Sci, Dept Neurosurg, Tehran, Iran; [Manafi, Navid] Iran Univ Med Sci, Dept Ophthalmol, Tehran, Iran; [Nojomi, Marzieh] Iran Univ Med Sci, Dept Community & Family Med, Tehran, Iran; [Shabaninejad, Hosein] Iran Univ Med Sci, Dept Hlth Serv Management, Tehran, Iran; [Yousefifard, Mahmoud] Iran Univ Med Sci, Physiol Res Ctr, Tehran, Iran; [Aljunid, Syed Mohamed] Kuwait Univ, Dept Hlth Policy & Management, Safat, Kuwait; [Aljunid, Syed Mohamed] Natl Univ Malaysia, Int Ctr Casemix & Clin Coding, Bandar Tun Razak, Malaysia; [Almasi, Ali] Kermanshah Univ Med Sci, Dept Environm Hlth Engn, Kermanshah, Iran; [Ghadiri, Keyghobad] Kermanshah Univ Med Sci, Infect Dis Res Ctr, Kermanshah, Iran; [Ghadiri, Keyghobad] Kermanshah Univ Med Sci, Pediat Dept, Kermanshah, Iran; [Heydarpour, Fatemeh] Kermanshah Univ Med Sci, Med Biol Res Ctr, Kermanshah, Iran; [Jalali, Amir] Kermanshah Univ Med Sci, Hlth Inst, Kermanshah, Iran; [Jalali, Amir] Kermanshah Univ Med Sci, Subst Abuse Prevent Res Ctr, Kermanshah, Iran; [Matin, Behzad Karami; Karyani, Ali Kazemi; Moradi, Masoud; Najafi, Farid; Pirsaheb, Meghdad; Rajati, Fatemeh; Sadeghi, Ehsan; Safari, Yahya; Sharafi, Kiomars; Soltani, Shahin] Kermanshah Univ Med Sci, Res Ctr Environm Determinants Hlth, Kermanshah, Iran; [Karyani, Ali Kazemi] Kermanshah Univ Med Sci, Dept Publ Hlth, Kermanshah, Iran; [Naderi, Mehdi] Kermanshah Univ Med Sci, Clin Res Dev Ctr, Kermanshah, Iran; [Najafi, Farid; Salimi, Yahya] Kermanshah Univ Med Sci, Dept Epidemiol & Biostat, Kermanshah, Iran; [Safari-Faramani, Roya] Kermanshah Univ Med Sci, Fac Publ Hlth, Kermanshah, Iran; [Salahshoor, Mohammad Reza] Kermanshah Univ Med Sci, Dept Anat Sci, Kermanshah, Iran; [Salimi, Yahya; Soofi, Moslem] Kermanshah Univ Med Sci, Social Dev & Hlth Promot Res Ctr, Kermanshah, Iran; [Shamsizadeh, Morteza] Kermanshah Univ Med Sci, Dept Sports Med & Rehabil, Kermanshah, Iran; [Siabani, Soraya; Ziapour, Arash] Kermanshah Univ Med Sci, Dept Hlth Educ & Hlth Promot, Kermanshah, Iran; [Sobhiyeh, Mohammad Reza] Kermanshah Univ Med Sci, Dept Vasc & Endovasc Surg, Kermanshah, Iran; [Almasi-Hashiani, Amir] Arak Univ Med Sci, Dept Epidemiol, Arak, Iran; [Amini, Saeed] Arak Univ Med Sci, Hlth Serv Management Dept, Arak, Iran; [Nazari, Javad] Arak Univ Med Sci, Dept Pediat, Arak, Iran; [Al-Mekhlafi, Hesham M.] Jazan Univ, Med

Res Ctr, Jazan, Saudi Arabia; [Bedi, Neeraj] Jazan Univ, Jazan, Saudi Arabia; [Al-Mekhlafi, Hesham M.] Sanaa Univ, Dept Parasitol, Sanaa, Yemen; [Altirkawi, Khalid A.; Temsah, Mohamad-Hani] King Saud Univ, Pediat Intens Care Unit, Riyadh, Saudi Arabia; [Mohammad, Yousef] King Saud Univ, Internal Med Dept, Riyadh, Saudi Arabia; [Alvis-Guzman, Nelson] Univ Cartagena, Res Grp Hlth Econ, Cartagena, Colombia; [Alvis-Guzman, Nelson] Univ Coast, Res Grp Hosp Management & Hlth Policies, Barranquilla, Colombia; [Alvis-Zakzuk, Nelson J.] Univ Coast, Dept Econ Sci, Barranquilla, Colombia; [Alvis-Zakzuk, Nelson J.] Natl Inst Hlth, Natl Hlth Observ, Bogota, Colombia; [Castaneda-Orjuela, Carlos A.] Natl Inst Hlth, Colombian Natl Hlth Observ, Bogota, Colombia; [Amit, Arianna Maever L.] Univ Philippines Manila, Dept Epidemiol & Biostat, Manila, Philippines; [Antonio, Carl Abelardo T.] Univ Philippines Manila, Dept Hlth Policy & Adm, Manila, Philippines; [Lopez, Jaifred Christian F.] Univ Philippines Manila, Dept Nutr, Manila, Philippines; [Amit, Arianna Maever L.] Johns Hopkins Univ, Sch Publ Hlth, Baltimore, MD USA; [Bairwa, Mohan] Johns Hopkins Univ, Dept Epidemiol, Baltimore, MD USA; [Hafezi-Nejad, Nima; Haj-Mirzaian, Arya] Johns Hopkins Univ, Dept Radiol & Radiol Sci, Baltimore, MD USA; [Atre, Sachin R.] Johns Hopkins Univ, Ctr Clin Global Hlth Educ, Baltimore, MD USA; [Andrei, Catalina Liliana] Carol Davila Univ Med & Pharm, Cardiol Dept, Bucharest, Romania; [Davitoiu, Dragos Virgil; Manda, Ana Laura; Negoi, Ionut] Carol Davila Univ Med & Pharm, Dept Gen Surg, Bucharest, Romania; [Preotescu, Liliana] Carol Davila Univ Med & Pharm, Dept Infect Dis, Bucharest, Romania; [Anjomshoa, Mina] Rafsanjan Univ Med Sci, Social Determinants Hlth Res Ctr, Rafsanjan, Iran; [Ansari, Fereshteh] Tabriz Univ Med Sci, Res Ctr Evidence Based Med, Tabriz, Iran; [Hassankhani, Hadi] Tabriz Univ Med Sci, Sch Nursing & Midwifery, Tabriz, Iran; [Haririan, Hamidreza] Tabriz Univ Med Sci, Tabriz, Iran; [Ansari, Fereshteh] Agr Res Educ & Extens Org, Razi Vaccine & Serum Res Inst, Tehran, Iran; [Antonio, Carl Abelardo T.] Hong Kong Polytech Univ, Dept Appl Social Sci, Hong Kong, Peoples R China; [Lee, Paul H.] Hong Kong Polytech Univ, Sch Nursing, Hong Kong, Peoples R China; [Antony, Benny; Singh, Ambrish] Univ Tasmania, Menzies Inst Med Res, Hobart, Tas, Australia; [Antriyandarti, Ernoiz] Sebelas Maret Univ, Agribusiness Study Program, Surakarta, Indonesia; [Aref, Hany Mohamed Amin; El Nahas, Nevine; Shalash, Ali S.] Ain Shams Univ, Neurol Dept, Cairo, Egypt; [Nabhan, Ashraf F.] Ain Shams Univ, Dept Obstet & Gynecol, Cairo, Egypt; [Samy, Abdallah M.] Ain Shams Univ, Dept Entomol, Cairo, Egypt; [Aremu, Olatunde] Birmingham City Univ, Dept Publ Hlth, Birmingham, W Midlands, England; [Armoon, Bahram] Saveh Univ Med Sci, Social Determinants Hlth Res Ctr, Saveh, Iran; [Armoon, Bahram] Yasuj Univ Med Sci, Social Determinants Hlth Res Ctr, Yasuj, Iran; [Arora, Amit] Western Sydney Univ, Sch Hlth Sci, Campbelltown, NSW, Australia; [Arora, Amit] Univ Sydney, Disciple Child & Adolescent Hlth, Westmead, NSW, Australia; [Aryal, Krishna K.] Abt Associates Nepal, Monitoring Evaluat & Operat Res Project, Lalitpur, Nepal; [Arzani, Afsaneh] Babol Univ Med Sci, Sch Nursing & Midwifery, Babol, Iran; [Bijani, Ali; Mouodi, Simin] Babol Univ Med Sci, Social Determinants Hlth Res Ctr, Babol, Iran; [Mosapour, Abbas] Babol Univ Med Sci, Dept Clin Biochem, Babol, Iran; [Zamani, Mohammad] Babol Univ Med Sci, Student Res Comm, Babol, Iran; [Arzani, Afsaneh] Babol Univ Med Sci, Babol, Iran; [Atalay, Hagos Tasew; Gebremeskel, Gebreamlak Gebremedhn; Tadesse, Degena Bahrey;

Weldesamuel, Girmay Teklay] Aksum Univ, Dept Nursing, Aksum, Ethiopia; [Demoz, Gebre Teklemariam; Kassa, Gebrehiwot G.] Aksum Univ, Sch Pharm, Aksum, Ethiopia; [Kassa, Gebrehiwot G.] Aksum Univ, Dept Biomed Sci, Aksum, Ethiopia; [Athari, Seyyed Shamsadin] Zanjan Univ Med Sci, Dept Immunol, Zanjan, Iran; [Athari, Seyyed Shamsadin] Maragheh Univ Med Sci, Dept Biol, Maragheh, Iran; [Eftekhari, Aziz] Maragheh Univ Med Sci, Dept Pharmacol & Toxicol, Maragheh, Iran; [Hasanpoor, Edris] Maragheh Univ Med Sci, Dept Healthcare Management, Maragheh, Iran; [Hasanzadeh, Amir] Maragheh Univ Med Sci, Dept Microbiol, Maragheh, Iran; [Pourjafar, Hadi] Maragheh Univ Med Sci, Dept Nutr & Food Sci, Maragheh, Iran; [Atre, Sachin R.] Dr DY Patil Vidyapeeth, Dr DY Patil Med Coll, Hosp & Res Ctr, Pune, Maharashtra, India; [Ausloos, Marcel] Univ Leicester, Sch Business, Leicester, Leics, England; [Ausloos, Marcel; Herteliu, Claudiu] Bucharest Univ Econ Studies, Dept Stat & Econometr, Bucharest, Romania; [Awoke, Nefsu; Chichiabellu, Tesfaye Yitna] Wolaita Sodo Univ, Dept Nursing, Wolaita Sodo, Ethiopia; [Kelbore, Abraham Getachew] Wolaita Sodo Univ, Dept Dermatol, Wolaita Sodo, Ethiopia; [Lenjebo, Tsegaye Lolaso] Wolaita Sodo Univ, Sch Publ Hlth, Wolaita Sodo, Ethiopia; [Paulos, Kebreab] Wolaita Sodo Univ, Dept Midwifery, Wolaita Sodo, Ethiopia; [Quintanilla, Beatriz Paulina Ayala] La Trobe Univ, Judith Lumley Ctr, Melbourne, Vic, Australia; [Rahman, Muhammad Aziz] La Trobe Univ, Sch Nursing & Midwifery, Melbourne, Vic, Australia; [Quintanilla, Beatriz Paulina Ayala] Peruvian Natl Inst Hlth, Gen Off Res & Technol Transfer, Lima, Peru; [Ayano, Getinet; Duko, Bereket; Hendrie, Delia; Miller, Ted R.] Curtin Univ, Sch Publ Hlth, Perth, WA, Australia; [Ayanore, Martin Amogre] Univ Hlth & Allied Sci, Dept Hlth Policy Planning & Management, Ho, Ghana; [Kugbey, Nuworza] Univ Hlth & Allied Sci, Dept Family & Community Hlth, Ho, Ghana; [Aynalem, Yared Asmare; Shiferaw, Wondimeneh Shibabaw] Debre Berhan Univ, Dept Nursing, Debre Berhan, Ethiopia; [Azzopardi, Peter S.] Burnet Inst, Global Adolescent Hlth Grp, Melbourne, Vic, Australia; [Azzopardi, Peter S.] South Australian Hlth & Med Res Inst, Wardliparingga Aboriginal Res Unit, Adelaide, SA, Australia; [Babalola, Tesleem Kayode] Univ KwaZulu Natal, Dept Publ Hlth Med, Durban, South Africa; [Ginindza, Themba G.] Univ KwaZulu Natal, Discipline Publ Hlth Med, Durban, South Africa; [Kugbey, Nuworza] Univ KwaZulu Natal, Dept Psychol & Hlth Promot, Durban, South Africa; [Naidoo, Kovin S.] Univ KwaZulu Natal, Discipline Optometry, Durban, South Africa; [Ginindza, Themba G.] Univ KwaZulu Natal, Durban, South Africa; [Babalola, Tesleem Kayode] Univ Lagos, Dept Community Hlth & Primary Care, Lagos, Nigeria; [Olagunju, Andrew T.] Univ Lagos, Dept Psychiat, Lagos, Nigeria; [Badawi, Alaa] Publ Hlth Agcy Canada, Publ Hlth Risk Sci Div, Toronto, ON, Canada; [Badawi, Alaa] Univ Toronto, Dept Nutr Sci, Toronto, ON, Canada; [Bhutta, Zulfiqar A.] Univ Toronto, Ctr Global Child Hlth, Toronto, ON, Canada; [Chattu, Vijay Kumar] Univ Toronto, Dept Med, Toronto, ON, Canada; [Bairwa, Mohan] All India Inst Med Sci, Ctr Community Med, New Delhi, India; [Lodha, Rakesh] All India Inst Med Sci, Dept Paediat, New Delhi, India; [Sagar, Rajesh] All India Inst Med Sci, Dept Psychiat, New Delhi, India; [Bakkannavar, Shankar M.] Manipal Acad Higher Educ, Dept Forens Med & Toxicol, Manipal, India; [Hoogar, Praveen] Manipal Acad Higher Educ, Ctr Bio Cultural Studies, Manipal, India; [Jha, Vivekanand] Manipal Acad Higher Educ, Manipal, India; [Balakrishnan, Senthilkumar] Haramaya Univ, Dept Med Microbiol, Harar, Ethiopia; [Bali, Ayele Geleto; Chanie,

Wagaye Fentahun; Tekle, Merhawi Gebremedhin] Haramaya Univ, Sch Publ Hlth, Harar, Ethiopia; [Bojia, Hunduma Amensisa] Haramaya Univ, Sch Pharm, Harar, Ethiopia; [Desalew, Assefa] Haramaya Univ, Sch Nursing & Midwifery, Harar, Ethiopia; [Banach, Maciej] Med Univ Lodz, Dept Hypertens, Lodz, Poland; [Banach, Maciej] Polish Mothers Mem Hosp, Res Inst, Lodz, Poland; [Banoub, Joseph Adel Mattar] Univ London, Dept Internal Med, London, England; [Banoub, Joseph Adel Mattar] Alexandria Univ, Dept Gen Med, Alexandria, Egypt; [El Sayed, Iman] Alexandria Univ, Biomed Informat & Med Stat Dept, Alexandria, Egypt; [El Tantawi, Maha] Alexandria Univ, Pediat Dent & Dent Publ Hlth Dept, Alexandria, Egypt; [Barac, Aleksandra] Clin Ctr Serbia, Clin Infect & Trop Dis, Belgrade, Serbia; [Barac, Aleksandra; Santric-Milicevic, Milena M.] Univ Belgrade, Fac Med, Belgrade, Serbia; [Dubljanin, Eleonora] Univ Belgrade, Inst Microbiol & Immunol, Belgrade, Serbia; [Santric-Milicevic, Milena M.] Univ Belgrade, Sch Publ Hlth & Hlth Management, Belgrade, Serbia; [Barnighausen, Till Winfried; Moazen, Babak; Mohammed, Shafiu] Heidelberg Univ, Heidelberg Inst Global Hlth, Heidelberg, Germany; [Barnighausen, Till Winfried] Harvard Univ, TH Chan Sch Publ Hlth, Boston, MA 02115 USA; [Basu, Sanjay] Harvard Univ, Ctr Primary Care, Boston, MA 02115 USA; [Kraemer, Moritz U. G.] Harvard Univ, Harvard Med Sch, Boston, MA 02115 USA; [Chattu, Vijay Kumar] Harvard Univ, Brigham & Womens Hosp, Boston, MA 02115 USA; [Norheim, Ole F.; Vollmer, Sebastian] Harvard Univ, Dept Global Hlth & Populat, Boston, MA 02115 USA; [Pereira, Alexandre] Harvard Univ, Dept Genet, Boston, MA 02115 USA; [Sheikh, Aziz] Harvard Univ, Div Gen Internal Med, Boston, MA 02115 USA; [Basaleem, Huda] Aden Coll, Sch Publ Hlth & Community Med, Aden, Yemen; [Basu, Sanjay; Saxena, Sonia] Imperial Coll London, Sch Publ Hlth, London, England; [Car, Josip; Majeed, Azeem; Rawaf, Salman] Imperial Coll London, Dept Primary Care & Publ Hlth, London, England; [Davis, Adrian C.] Imperial Coll London, Dept Surg & Canc, London, England; [Kusuma, Vivek Kumar Dian] Imperial Coll London, Imperial Coll Business Sch, London, England; [Rawaf, David Laith] Imperial Coll London, WHO Collaborating Ctr Publ Hlth Educ & Training, London, England; [Bay, Vo Dinh] Ho Chi Minh City Univ Technol, Informat Technol, Ho Chi Minh City, Vietnam; [Bayati, Mohsen] Shiraz Univ Med Sci, Hlth Human Resources Res Ctr, Shiraz, Iran; [Hoseini, Mohammad] Shiraz Univ Med Sci, Dept Environm Hlth, Shiraz, Iran; [Hoseini, Mohammad] Shiraz Univ Med Sci, Res Ctr Hlth Sci, Inst Hlth, Shiraz, Iran; [Malekzadeh, Reza; Sepanlou, Sadaf G.] Shiraz Univ Med Sci, Noncommunicable Dis Res Ctr, Shiraz, Iran; [Baye, Estifanos] Wollo Univ, Sch Publ Hlth, Dessie, Ethiopia; [Belayneh, Yaschilal Muche; Meharie, Birhanu Geta] Wollo Univ, Dept Pharm, Dessie, Ethiopia; [Mekonnen, Tefera Chane] Wollo Univ, Dept Publ Hlth, Dessie, Ethiopia; [Tefera, Yonatal Mesfin] Wollo Univ, Dept Environm Hlth, Dessie, Ethiopia; [Bedi, Neeraj] Gandhi Med Coll Bhopal, Dept Community Med, Bhopal, India; [Beheshti, Mahya] NYU, Dept Phys Med & Rehabil, New York, NY USA; [Behzadifar, Masoud] Lorestan Univ Med Sci, Social Determinants Hlth Res Ctr, Khorramabad, Iran; [Behzadifar, Meysam] Lorestan Univ Med Sci, Dept Epidemiol & Biostat, Khorramabad, Iran; [Imani-Nasab, Mohammad Hasan; Motlagh, Soraya Nouraei] Lorestan Univ Med Sci, Dept Publ Hlth, Khorramabad, Iran; [Bekele, Bayu Begashaw; Hassen, Hamid Yimam] Mizan Tepi Univ, Dept Publ Hlth, Mizan Teferi, Ethiopia; [Bekele, Bayu Begashaw] Univ Debrecen, Doctoral Sch Hlth Sci, Debrecen, Hungary; [Bell, Michelle L.] Yale Univ, Sch

Environm, New Haven, CT USA; [Bennett, Derrick A.; Karim, Mohd Anisul; Lacey, Ben] Univ Oxford, Nuffield Dept Populat Hlth, Oxford, England; [Kraemer, Moritz U. G.] Univ Oxford, Dept Zool, Oxford, England; [Moore, Catrin E.] Univ Oxford, Big Data Inst, Oxford, England; [Berbada, Dessalegn Ajema; Sorrie, Muluken Bekele; Hawariat, Feleke Gebremeskel W.] Arba Minch Univ, Dept Publ Hlth, Arba Minch, Ethiopia; [Worku, Getasew Taddesse] Arba Minch Univ, Fac Business & Econ, Arba Minch, Ethiopia; [Bernstein, Robert S.] Emory Univ, Hubert Dept Global Hlth, Atlanta, GA 30322 USA; [Shin, Jae Il] Emory Univ, Div Cardiol, Atlanta, GA 30322 USA; [Bernstein, Robert S.] Univ S Florida, Dept Global Hlth, Tampa, FL 33620 USA; [Bhat, Anusha Ganapati] Univ Massachusetts, Sch Med, Div Gen Internal Med, Springfield, MA USA; [Bhattacharyya, Krittika] Natl Inst Biomed Genom, Dept Stat & Computat Genom, Kalyani, W Bengal, India; [Bhattacharyya, Krittika] Univ Calcutta, Dept Stat, Kolkata, India; [Bhattarai, Suraj] Global Inst Interdisciplinary Studies, Dept Global Hlth, Kathmandu, Nepal; [Bhaumik, Soumyadeep] George Inst Global Hlth, Injury Div, New Delhi, India; [Jha, Vivekanand] George Inst Global Hlth, New Delhi, India; [Bhaumik, Soumyadeep] Univ New South Wales, George Inst Global Hlth, Sydney, NSW, Australia; [Biswas, Raaj Kishore; Boufous, Soufiane] Univ New South Wales, Transport & Rd Safety Res Ctr, Sydney, NSW, Australia; [Karki, Hamidreza Karimi-Sari Surendra] Univ New South Wales, Sch Publ Hlth & Community Med, Sydney, NSW, Australia; [Naidoo, Kovin S.; Resnikoff, Serge] Univ New South Wales, Sch Optometry & Vis Sci, Sydney, NSW, Australia; [Das, Jai K.] Aga Khan Univ, Div Women & Child Hlth, Karachi, Pakistan; [Bikbov, Boris] Mario Negri Inst Pharmacol Res, Ranica, Italy; [Biswas, Raaj Kishore] Swinburne Univ Technol, Sch Hlth Sci, Melbourne, Vic, Australia; [Bohlouli, Somayeh] Islamic Azad Univ, Dept Vet Med, Kermanshah, Iran; [Bragazzi, Nicola Luigi] Univ Genoa, Genoa, Italy; [Briko, Andrey Nikolaevich] Bauman Moscow State Tech Univ, Dept Biomed Technol, Moscow, Russia; [Briko, Nikolay Ivanovich] IM Sechenov First Moscow State Med Univ, Dept Epidemiol & Evidence Based Med, Moscow, Russia; [Jakovljevic, Mihajlo] IM Sechenov First Moscow State Med Univ, NA Semashko Dept Publ Hlth & Healthcare, Moscow, Russia; [Nagaraja, Sharath Burugina] Employee State Insurance Post Grad Inst Med Sci &, Dept Community Med, Bangalore, Karnataka, India; [Busse, Reinhard] Tech Univ Berlin, Dept Hlth Care Management, Berlin, Germany; [Butt, Zahid A.] Univ Waterloo, Sch Publ Hlth & Hlth Syst, Waterloo, ON, Canada; [Butt, Zahid A.] Al Shifa Trust Eye Hosp, Al Shifa Sch Publ Hlth, Rawalpindi, Pakistan; [Camera, Luis Alberto] Argentine Soc Med, Board Directors, Buenos Aires, DF, Argentina; [Valdez, Pascual R.] Argentine Soc Med, Buenos Aires, DF, Argentina; [Car, Josip] Nanyang Technol Univ, Ctr Populat Hlth Sci, Singapore, Singapore; [Gomes, Nelson G. M.] Univ Porto, Dept Chem, Porto, Portugal; [Ribeiro, Ana Isabel] Univ Porto, EPI Unit, Publ Hlth Inst, Porto, Portugal; [Castaneda-Orjuela, Carlos A.] Univ Nacl Colombia, Epidemiol & Publ Hlth Evaluat Grp, Bogota, Colombia; [De la Hoz, Fernando Pio] Univ Nacl Colombia, Dept Publ Hlth, Bogota, Colombia; [Chatterjee, Pranab] Indian Council Med Res, Div Epidemiol & Communicable Dis, New Delhi, India; [Dandona, Lalit] Indian Council Med Res, New Delhi, India; [Chin, Ken Lee] Univ Melbourne, Melbourne Med Sch, Parkville, Vic, Australia; [Christopher, Devasahayam J.] Christian Med Coll & Hosp, Dept Pulm Med, Vellore, Tamil Nadu, India; [Thomas, Nihal] Christian Med

Coll & Hosp, Dept Endocrinol Diabet & Metab, Vellore, Tamil Nadu, India; [Varughese, Santosh] Christian Med Coll & Hosp, Dept Nephrol, Vellore, Tamil Nadu, India; [Damiani, Giovanni] Univ Milan, Clin Dermatol, IRCCS Ist Ortopedico Galeazzi, Milan, Italy; [La Vecchia, Carlo] Univ Milan, Dept Clin Sci & Community Hlth, Milan, Italy; [Damiani, Giovanni] Case Western Reserve Univ, Dept Dermatol, Cleveland, OH 44106 USA; [Sanabria, Juan] Case Western Reserve Univ, Dept Nutr & Prevent Med, Cleveland, OH 44106 USA; [Mathur, Manu Raj] Publ Hlth Fdn India, Hlth Policy Res, Gurugram, India; [Zodpey, Sanjay] Publ Hlth Fdn India, Indian Inst Publ Hlth, Gurugram, India; [Dandona, Lalit; Dandona, Rakhi; Kumar, G. Anil; Lal, Dharmesh Kumar] Publ Hlth Fdn India, Gurugram, India; [Darwesh, Aso Mohammad] Univ Human Dev, Dept Informat Technol, Sulaymaniyah, Iraq; [Hosseinzadeh, Mehdi] Univ Human Dev, Dept Comp Sci, Sulaymaniyah, Iraq; [Darwish, Amira Hamed] Tanta Univ, Dept Pediat, Tanta, Egypt; [Daryani, Ahmad] Mazandaran Univ Med Sci, Toxoplasmosis Res Ctr, Sari, Iran; [Faridnia, Roghiyeh] Mazandaran Univ Med Sci, Dept Med Parasitol, Sari, Iran; [Rafiei, Alireza] Mazandaran Univ Med Sci, Dept Immunol, Sari, Iran; [Rafiei, Alireza] Mazandaran Univ Med Sci, Mol & Cell Biol Res Ctr, Sari, Iran; [Rezai, Mohammad Sadegh] Mazandaran Univ Med Sci, Pediat Infect Dis Res Ctr, Sari, Iran; [Davey, Gail] Brighton & Sussex Med Sch, Dept Global Hlth & Infect, Brighton, E Sussex, England; [Deribe, Kebede] Brighton & Sussex Med Sch, Wellcome Trust Brighton & Sussex Ctr Global Hlth, Brighton, E Sussex, England; [Elema, Teshome Bekele] Addis Ababa Univ, Ctr Food Sci & Nutr, Addis Ababa, Ethiopia; [Gebremedhin, Ketema Bizuwork] Addis Ababa Univ, Dept Nursing & Midwifery, Addis Ababa, Ethiopia; [Wondafrash, Dawit Zewdu] Addis Ababa Univ, Dept Pharmacol, Addis Ababa, Ethiopia; [Yisma, Engida] Addis Ababa Univ, Sch Allied Hlth Sci, Addis Ababa, Ethiopia; [Demoz, Gebre Teklemariam] Addis Ababa Univ, Addis Ababa, Ethiopia; [Davila-Cervantes, Claudio Alberto] Latin Amer Fac Social Sci Mexico, Dept Populat & Dev, Mexico City, DF, Mexico; [Davis, Adrian C.] UCL, Ear Inst, London, England; [Kumar, Manasi] UCL, Div Psychol & Language Sci, London, England; [Sarkar, Kaushik] UCL, Inst Child Hlth, London, England; [Reta, Melese Abate] Woldia Univ, Dept Med Lab Sci, Woldia, Ethiopia; [Demissie, Dereje Bayissa] St Pauls Hosp, Millennium Med Coll, Neonatal Nursing Dept, Addis Ababa, Ethiopia; [Weji, Bedilu Girma] St Pauls Hosp, Millennium Med Coll, Anesthesia Dept, Addis Ababa, Ethiopia; [Shifti, Desalegn Markos] St Pauls Hosp, Millennium Med Coll, Addis Ababa, Ethiopia; [Dhillon, Preeti] Int Inst Populat Sci, Dept Math Demog & Stat, Mumbai, Maharashtra, India; [Khan, Junaid] Int Inst Populat Sci, Dept Populat Studies, Mumbai, Maharashtra, India; [Dhungana, Govinda Prasad] Far Western Univ, Dept Microbiol, Mahendranagar, Nepal; [Diaz, Daniel] Univ Nacl Autonoma Mexico, Ctr Complex Sci, Mexico City, DF, Mexico; [Diaz, Daniel] Autonomous Univ Sinaloa, Fac Vet Med & Zootech, Culiacan Rosales, Mexico; [Ilesanmi, Olayinka Stephen] Univ Ibadan, Dept Community Med, Ibadan, Nigeria; [Ogah, Okechukwu Samuel; Owolabi, Mayowa O.] Univ Ibadan, Dept Med, Ibadan, Nigeria; [Djalalinia, Shirin] Minist Hlth & Med Educ, Dev Res & Technol Ctr, Tehran, Iran; [Ogbo, Felix Akpojene] Western Sydney Univ, Translat Hlth Res Inst, Sydney, NSW, Australia; [Toma, Alemayehu] Hawassa Univ, Dept Pharm, Hawassa, Ethiopia; [Duraes, Andre Rodrigues] Univ Fed Bahia, Sch Med, Salvador, BA, Brazil; [Rasella, Davide] Univ Fed Bahia, Inst Collect Hlth, Salvador, BA, Brazil; [Kalan, Mohammad Ebrahimi] Florida Int Univ, Dept Epidemiol, Miami, FL 33199 USA;

[Edinur, Hisham Atan] Univ Sains Malaysia, Sch Hlth Sci, Kubang Kerian, Kelantan, Malaysia; [Khan, Md Nuruzzaman] Univ Newcastle, Sch Med & Publ Hlth, Newcastle, NSW, Australia; [Eftekhari, Aziz] John Paul II Catholic Univ Lublin, Dept Pharmacol & Toxicol, Lublin, Poland; [Elkout, Hajer] WHO, Hlth Informat, Tripoli, Libya; [Mihretie, Kebadnew Mulatu; Muchie, Kindie Fentahun] Bahir Dar Univ, Dept Epidemiol & Biostat, Bahir Dar, Ethiopia; [Nigatu, Dabere] Bahir Dar Univ, Dept Reprod Hlth & Populat Studies, Bahir Dar, Ethiopia; [Endalew, Daniel Adane; Yeshaneh, Alex] Wolkite Univ, Dept Midwifery, Wolkite, Ethiopia; [Ezekannagha, Oluchi] Int Inst Trop Agr, Ibadan, Nigeria; [Fazlzadeh, Mehdi] Ardabil Univ Med Sci, Dept Environm Hlth Engn, Ardebil, Iran; [Feigin, Valery L.] Auckland Univ Technol, Natl Inst Stroke & Appl Neurosci, Auckland, New Zealand; [Feigin, Valery L.] Res Ctr Neurol, Moscow, Russia; [Fereshtehnejad, Seyed-Mohammad] Univ Ottawa, Div Neurol, Ottawa, ON, Canada; [Filip, Irina] Kaiser Permanente, Psychiat Dept, Fontana, CA USA; [Radfar, Amir] AT Still Univ, Coll Grad Hlth Sci, Mesa, AZ 85206 USA; [Fischer, Florian] Ravensburg Weingarten Univ Appl Sci, Inst Gerontol Hlth Serv & Nursing Res, Weingarten, Germany; [Foroutan, Masoud] Abadan Sch Med Sci, Abadan Fac Med Sci, Abadan, Iran; [Franklin, Richard Charles] James Cook Univ, Sch Publ Hlth Med & Vet Sci, Douglas, Qld, Australia; [Fukumoto, Takeshi] Kobe Univ, Dept Dermatol, Kobe, Hyogo, Japan; [Saad, Anas M.] Cleveland Clin, Inst Heart & Vasc, Cleveland, OH 44106 USA; [Gad, Mohamed M.] Univ N Carolina, Gillings Sch Global Publ Hlth, Chapel Hill, NC 27515 USA; [Yilma, Mekdes Tigistu] Wollega Univ, Dept Publ Hlth, Nekemte, Ethiopia; [Gesesew, Hailay Abrha] Flinders Univ S Australia, Coll Med & Publ Hlth, Adelaide, SA, Australia; [Tefera, Yonatal Mesfin] Univ Adelaide, Sch Publ Hlth, Adelaide, SA, Australia; [Gopalani, Sameer Vali] Univ Oklahoma, Hlth Sci Ctr, Hudson Coll Publ Hlth, Oklahoma City, OK USA; [Goulart, Alessandra C.; Goulart, Barbara Niegia Garcia] Univ Fed Rio Grande do Sul, Postgrad Program Epidemiol, Porto Alegre, RS, Brazil; [Gubari, Mohammed Ibrahim Mohialdeen] Univ Sulaimani, Dept Family & Community Med, Sulaimani, Iraq; [Gugnani, Harish Chander] St James Sch Med, Dept Epidemiol, The Valley, Anguilla; [Guimaraes, Rafael Alves] Univ Fed Goias, Inst Trop Pathol & Publ Hlth, Goiania, Go, Brazil; [Guo, Yuming] Binzhou Med Univ, Dept Epidemiol, Yantai, Peoples R China; [Gupta, Rajeev] Eternal Heart Care Ctr & Res Inst, Dept Prevent Cardiol, Jaipur, Rajasthan, India; [Gupta, Rajeev] Mahatma Gandhi Univ Med Sci, Dept Med, Jaipur, Rajasthan, India; [Jahanmehr, Nader] Shahid Beheshti Univ Med, Sch Management & Med Educ, Tehran, Iran; [Jahanmehr, Nader] Shahid Beheshti Univ Med, Safety Promot & Injury Prevent Res Ctr, Tehran, Iran; [Hamadeh, Randah R.] Arabian Gulf Univ, Dept Family & Community Med, Manama, Bahrain; [Hamidi, Samer] Hamdan Bin Mohammed Smart Univ, Sch Hlth & Environm Studies, Dubai, U Arab Emirates; [Martini, Santi] Airlangga Univ, Fac Publ Hlth, Surabaya, Indonesia; [Hasaballah, Ahmed I.] Al Azhar Univ, Dept Zool & Entomol, Cairo, Egypt; [Hasan, Md Mehedi; Mamun, Abdullah A.] Univ Queensland, Inst Social Sci Res, Indooroopilly, Qld, Australia; [Hasan, Md Mehedi] Univ Queensland, ARC Ctr Excellence Children & Families Life Cours, Indooroopilly, Qld, Australia; [Hassen, Hamid Yimam] Univ Hosp Antwerp, Dept Primary & Interdisciplinary Care, Antwerp, Belgium; [Herteliu, Claudiu] London South Bank Univ, Sch Business, London, England; [de Hidru, Hagos Degefa; Tesfay, Berhe Etsay] Adigrat Univ, Dept Publ Hlth, Adigrat, Ethiopia; [Hird, Thomas R.] Univ Bath, Dept Hlth, Bath,

Avon, England; [Moraga, Paula] Univ Bath, Dept Math Sci, Bath, Avon, England; [Hoang, Chi Linh; Nguyen, Trang Huyen] Nguyen Tat Thanh Univ, Ctr Excellence Behav Med, Ho Chi Minh City, Vietnam; [Hossain, Naznin] Bangladesh Ind Gases Ltd, Dept Pharmacol, Tangail, Bangladesh; [Househ, Mowafa] Hamad Bin Khalifa Univ, Div Informat & Comp Technol, Doha, Qatar; [Hu, Guoqing] Cent South Univ, Dept Epidemiol & Hlth Stat, Changsha, Peoples R China; [Igumbor, Ehimario U.] Univ Western Cape, Sch Publ Hlth, Cape Town, South Africa; [Iqbal, Usman] Taipei Med Univ, Coll Publ Hlth, Taipei, Taiwan; [Ningrum, Dina Nur Anggraini] Taipei Med Univ, Grad Inst Biomed Informat, Taipei, Taiwan; [Islam, Sheikh Mohammed Shariful] Deakin Univ, Inst Phys Act & Nutr, Burwood, Vic, Australia; [Islam, Sheikh Mohammed Shariful] Univ Sydney, Sydney Med Sch, Sydney, NSW, Australia; [Jayatilleke, Achala Upendra] Inst Violence & Injury Prevent, Fac Grad Studies, Colombo, Sri Lanka; [Jha, Ravi Prakash] Dr Baba Saheb Ambedkar Med Coll & Hosp, Dept Community Med, Delhi, India; [Jha, Ravi Prakash] Banaras Hindu Univ, Dept Community Med, Varanasi, Uttar Pradesh, India; [Ji, John S.] Duke Univ, Nicholas Sch Environm, Durham, NC 27708 USA; [Zadey, Siddhesh] Duke Univ, Duke Global Hlth Inst, Durham, NC 27708 USA; [Jonas, Jost B.] Beijing Tongren Hosp, Beijing Inst Ophthalmol, Beijing, Peoples R China; [Jozwiak, Jacek Jerzy] Univ Opole, Dept Family Med & Publ Hlth, Opole, Poland; [Kanchan, Tanuj] All India Inst Med Sci, Dept Forens Med & Toxicol, Jodhpur, Rajasthan, India; [Karch, Andre] Univ Munster, Inst Epidemiol & Social Med, Munster, Germany; [Karki, Hamidreza Karimi-Sari Surendra] Middle East Liver Dis Ctr, Dept Young Investigators, Tehran, Iran; [Traini, Eugenio] Univ Utrecht, Inst Risk Assessment Sci, Utrecht, Netherlands; [Kebede, Mihiretu M.] German Canc Res Ctr, Dept Canc Epidemiol, Heidelberg, Germany; [Kebede, Mihiretu M.] Leibniz Inst Prevent Res & Epidemiol, Dept Prevent & Evaluat, Bremen, Germany; [Kumar, Manasi] Univ Nairobi, Dept Psychiat, Nairobi, Kenya; [Muriithi, Moses K.] Univ Nairobi, Sch Econ, Nairobi, Kenya; [Kengne, Andre Pascal] Med Res Council South Africa, NonCommunicable Dis Res Unit, Cape Town, South Africa; [Kengne, Andre Pascal] Univ Cape Town, Dept Med, Cape Town, South Africa; [Nnaji, Chukwudi A.; Wiysonge, Charles Shey] Univ Cape Town, Sch Publ Hlth & Family Med, Cape Town, South Africa; [Khalid, Nauman] Univ Management & Technol, Sch Food & Agr Sci, Lahore, Pakistan; [Khalilov, Rovshan] Baku State Univ, Dept Biophys & Mol Biol, Baku, Azerbaijan; [Khalilov, Rovshan] Azerbaijan Natl Acad Sci, Inst Radiat Problems, Baku, Azerbaijan; [Khan, Muhammad Shahzeb; Usman, Muhammad Shariq] Dow Univ Hlth Sci, Dept Internal Med, Karachi, Pakistan; [Siddiqi, Tariq Jamal] Dow Univ Hlth Sci, Dept Med, Karachi, Pakistan; [Khatab, Khaled] Ohio Univ, Coll Arts & Sci, Zanesville, OH USA; [Khayamzadeh, Maryam] Iranian Acad Med Sci, Tehran, Iran; [Khubchandani, Jagdish] Ball State Univ, Dept Nutr & Hlth Sci, Muncie, IN 47306 USA; [Kim, Yun Jin] Xiamen Univ Malaysia, Sch Tradit Chinese Med, Sepang, Malaysia; [Kimokoti, Ruth W.] Simmons Univ, Dept Nutr, Boston, MA USA; [Kisa, Sezer] Oslo Metropolitan Univ, Dept Nursing & Hlth Promot, Oslo, Norway; [Shivakumar, K. M.] Deemed Univ, Krishna Inst Med Sci, Publ Hlth Dent Dept, Karad, India; [Koul, Parvaiz A.] Sheri Kashmir Inst Med Sci, Dept Internal & Pulm Med, Srinagar, India; [Koyanagi, Ai] San Juan Dios Sanit Pk, CIBERSAM, Sant Boi De Llobregat, Spain; [Koyanagi, Ai] Catalan Inst Res & Adv Studies, Barcelona, Spain; [Lacey, Ben] Oxford Biomed Res Ctr, Natl Inst Hlth Res, Oxford, England; [Lam, Felix] Clinton Hlth

Access Initiat, Dept Essential Medicines & Hlth Prod, Boston, MA USA; [Lami, Faris Hasan] Univ Baghdad, Dept Community & Family Med, Baghdad, Iraq; [Lamichhane, Prabhat] Deakin Univ, Sch Med, Geelong, Vic, Australia; [Lasrado, Savita] Father Muller Med Coll, Dept Otorhinolaryngol, Mangalore, India; [Laxmaiah, Avula] Indian Council Med Res, Natl Inst Nutr, Hyderabad, India; [Liu, Shiwei] Chinese Ctr Dis Control & Prevent, Beijing, Peoples R China; [Liu, Simin] Brown Univ, Dept Epidemiol, Providence, RI 02912 USA; [Lopez, Jaifred Christian F.] Alliance Improving Hlth Outcomes, Quezon City, Philippines; [Abd El Razek, Hassan Magdy] Mansoura Fac Med, Radiol Dept, Mansoura, Egypt; [Mahotra, Narayan B.] Tribhuvan Univ, Dept Clin Physiol, Kathmandu, Nepal; [Manafi, Navid] Univ Manitoba, Ophthalmol Dept, Winnipeg, MB, Canada; [Manda, Ana Laura] Emergency Univ Hosp Bucharest, Gen Surg Dept 1, Bucharest, Romania; [Martins-Melo, Francisco Rogerlandio] Fed Inst Educ Sci & Technol Ceara, Campus Caucaia, Caucaia, Brazil; [Masaka, Anthony] Botho Univ Botswana, Fac Hlth & Educ, Gaborone, Botswana; [Mathur, Manu Raj] Univ Liverpool, Inst Populat Hlth Sci, Liverpool, Merseyside, England; [Molokhia, Mariam] Kings Coll London, Fac Life Sci & Med, London, England; [Shibuya, Kenji] Kings Coll London, Inst Populat Hlth, London, England; [Mehta, Kala M.] Univ Calif San Francisco, Dept Epidemiol & Biostat, San Francisco, CA 94143 USA; [Mendoza, Walter] United Nations Populat Fund, Peru Country Off, Lima, Peru; [Meretoja, Tuomo J.] Helsinki Univ Hosp, Breast Surg Unit, Helsinki, Finland; [Mestrovic, Tomislav] Dr Zora Profoz Polyclin, Clin Microbiol & Parasitol Unit, Zagreb, Croatia; [Mini, G. K.] Womens Social & Hlth Studies Fdn, Womens Inst Social & Hlth Studies, Trivandrum, Kerala, India; [Mirrakhimov, Erkin M.] Kyrgyz State Med Acad, Internal Med Programme, Bishkek, Kyrgyzstan; [Moazen, Babak] Frankfurt Univ Appl Sci, Res Inst Addict, Frankfurt, Germany; [Moradi, Ghobad] Kurdistan Univ Med Sci, Dept Epidemiol & Biostat, Sanandaj, Iran; [Zepro, Nejimu Biza] Samara Univ, Dept Nursing, Semera, Ethiopia; [Moodley, Yoshan] Cent Univ Technol, Hlth & Environm Sci Dept, Bloemfontein, South Africa; [Mosapour, Abbas] Tarbiat Modares Univ, Dept Clin Biochem, Tehran, Iran; [Mozaffor, Miliva] Med Coll Women & Hosp, Dept Biochem, Dhaka, Bangladesh; [Mustafa, Ghulam] Inst Mother & Child Care, Dept Pediat & Pediat Pulmonol, Multan, Pakistan; [Schwebel, David C.] Univ Alabama Birmingham, Dept Psychol, Birmingham, AL USA; [Nansseu, Jobert Richie] Minist Publ Hlth, Dept Control Dis Epidem & Pandem, Yaounde, Cameroon; [Netsere, Henok Biresaw] Bahir Dar Univ, Sch Hlth Sci, Gondar, Ethiopia; [Oancea, Bogdan] Univ Bucharest, Adm & Econ Sci Dept, Bucharest, Romania; [Uzochukwu, Benjamin S. Chudi] Univ Nigeria Nsukka, Dept Community Med, Enugu, Nigeria; [Oren, Eyal] San Diego State Univ, Grad Sch Publ Hlth, San Diego, CA 92182 USA; [Osarenotor, Osayomwanbo] Univ Benin, Dept Environm Management & Toxicol, Benin, Nigeria; [Unnikrishnan, Bhaskaran] Manipal Acad Higher Educ, Kasturba Med Coll, Mangalore, India; [Pakhale, Smita] Ottawa Hosp Res Inst, Dept Med, Ottawa, ON, Canada; [Patel, Sangram Kishor] Indian Inst Hlth Management Res Univ, Jaipur, Rajasthan, India; [Paternina-Caicedo, Angel J.] Univ Sinu, Sch Med, Cartagena, Colombia; [Patton, George C.] Univ Melbourne, Dept Pediat, Melbourne, Vic, Australia; [Suleria, Hafiz Ansar Rasul] Univ Melbourne, Dept Agr & Food Syst, Melbourne, Vic, Australia; [Paudel, Deepak] Ludwig Maximilians Univ Munchen, Ctr Int Hlth, Munich, Germany; [Pescarini, Julia Moreira] Fundacao Oswaldo Cruz, Ctr Integrat Data & Hlth Knowledge, Salvador, BA, Brazil; [Postma,

Maarten J.] Univ Groningen, Univ Med Ctr Groningen, Groningen, Netherlands; [Prada, Sergio I.] Fdn Valle Lili, Clin Res Ctr, Cali, Colombia; [Prada, Sergio I.] ICESI Univ, Ctr Studies Social Protect & Hlth Econ, Cali, Colombia; [Rabiee, Navid] Sharif Univ Technol, Dept Chem, Tehran, Iran; [Radfar, Amir] Univ Cent Florida, Coll Med, Orlando, FL 32816 USA; [Rahman, Shafiur] Hamamatsu Univ Sch Med, Res Ctr Child Mental Dev, Hamamatsu, Shizuoka, Japan; [Rana, Saleem Muhammad] Univ Lahore, Univ Inst Publ Hlth, Lahore, Pakistan; [Ranabhat, Chhabi Lal] Policy Res Inst, Res Dept, Kathmandu, Nepal; [Rawaf, David Laith] Univ Coll London Hosp, London, England; [Renzaho, Andre M. N.] Western Sydney Univ, Sch Social Sci & Psychol, Penrith, NSW, Australia; [Reta, Melese Abate] Univ Pretoria, Dept Med Microbiol, Pretoria, South Africa; [Riahi, Seyed Mohammad] Birjand Univ Med Sci, Cardiovasc Dis Res Ctr, Birjand, Iran; [Rickard, Jennifer] Univ Teaching Hosp Kigali, Dept Surg, Kigali, Rwanda; [Rubagotti, Enrico] Southern Univ Sci & Technol, Dept Ocean Sci & Engn, Shenzhen, Peoples R China; [Rubino, Salvatore] Univ Sassari, Dept Biomed Sci, Sassari, Italy; [Sabde, Yogesh Damodar] Natl Inst Res Environm Hlth, Environm Epidemiol & Publ Hlth, Bhopal, India; [Sahebkar, Amirhossein] Food & Drug Adm Islamic Republ Iran, Halal Res Ctr IRI, Tehran, Iran; [Salam, Nasir] Cent Univ Punjab, Dept Microbiol, Bathinda, India; [Sanabria, Juan] Marshall Univ, Dept Surg, Huntington, WV USA; [Saraswathy, Sivan Yegnanarayana Iyer] PSG FAIMER South Asia Reg Inst, Coimbatore, Tamil Nadu, India; [Sathian, Brijesh] Hamad Med Corp, Dept Geriatr & Long Term Care, Doha, Qatar; [Sathian, Brijesh] Bournemouth Univ, Fac Hlth & Social Sci, Bournemouth, Dorset, England; [Sawhney, Monika] Univ N Carolina, Dept Publ Hlth Sci, Charlotte, NC USA; [Shabaninejad, Hosein] Newcastle Univ, Populat Hlth Sci Inst, Newcastle Upon Tyne, Tyne & Wear, England; [Shafieesabet, Azadeh] Charite Med Univ Berlin, Dept Cardiol, Berlin, Germany; [Shannawaz, Mohammed] BLDE Univ, Dept Community Med, Vijayapur, India; [Soyiri, Ireneous N.] Univ Edinburgh, Usher Inst Populat Hlth Sci & Informat, Edinburgh, Midlothian, Scotland; [Shigematsu, Mika] Natl Inst Infect Dis, Tokyo, Japan; [Siabani, Soraya] Univ Technol Sydney, Sch Hlth, Sydney, NSW, Australia; [Silva, Diego Augusto Santos] Univ Fed Santa Catarina, Dept Phys Educ, Florianopolis, SC, Brazil; [Singh, Jasvinder A.] US Dept Vet Affairs, Med Serv, Birmingham, AL USA; [Sokhan, Anton] Kharkiv Natl Med Univ, Dept Infect Dis, Kharkiv, Ukraine; [Somayaji, Ranjani] Univ Calgary, Dept Med, Calgary, AB, Canada; [Soyiri, Ireneous N.] Univ Hull, Hull York Med Sch, Kingston Upon Hull, N Humberside, England; [Sultana, Marufa] Deakin Univ, Hlth Econ, Melbourne, Vic, Australia; [Sunguya, Bruno Fokas] Muhimbili Univ Hlth & Allied Sci, Dept Community Hlth, Dar Es Salaam, Tanzania; [Sykes, Bryan L.] Univ Calif Irvine, Dept Criminol Law & Soc, Irvine, CA USA; [Tabares-Seisdedos, Rafael] Univ Valencia, Dept Med, Valencia, Spain; [Tabares-Seisdedos, Rafael] Biomed Res Networking Ctr, Mental Hlth Network, Carlos III Hlth Inst, Madrid, Spain; [Thankappan, Kavumpurathu Raman] Cent Univ Kerala, Dept Publ Hlth & Community Med, Kasaragod, India; [Topor-Madry, Roman] Jagiellonian Univ Med Coll, Inst Publ Hlth, Krakow, Poland; [Tovani-Palone, Marcos Roberto] Modestum LTD, London, England; [Tran, Bach Xuan] Hanoi Med Univ, Dept Hlth Econ, Hanoi, Vietnam; [Ullah, Irfan] Iqra Natl Univ, Dept Microbiol, Peshawar, Pakistan; [Violante, Francesco S.] Univ Bologna, Dept Med & Surg Sci, Bologna, Italy; [Waheed, Yasir] Fdn Univ Islamabad, Fdn Univ Med

Coll, Islamabad, Pakistan; [Wallin, Mitchell Taylor] George Washington Univ, Dept Neurol, Washington, DC USA; [Wiangkham, Taweewat] Naresuan Univ, Dept Phys Therapy, Phitsanulok, Thailand; [Worku, Getasew Taddesse] Addis Continental Inst Publ Hlth, Dept Hlth Econ, Addis Ababa, Ethiopia; [Wu, Ai-Min] Wenzhou Med Univ, Dept Orthopaed, Wenzhou, Peoples R China; [Xu, Gelin] Nanjing Univ, Sch Med, Nanjing, Peoples R China; [Yatsuya, Hiroshi] Nagoya Univ, Dept Publ Hlth & Hlth Syst, Nagoya, Aichi, Japan; [Yilgwan, Christopher Sabo] Univ Jos, Pediat Dept, Jos, Nigeria; [Yilgwan, Christopher Sabo] Jos Univ, Teaching Hosp, Dept Pediat, Jos, Nigeria; [Yip, Paul] Univ Hong Kong, Dept Social Work & Social Adm, Hong Kong, Peoples R China; [Yonemoto, Naohiro] Natl Ctr Neurol & Psychiat, Dept Neuropsychopharmaol, Kodaira, Tokyo, Japan; [Yonemoto, Naohiro] Juntendo Univ, Dept Publ Hlth, Tokyo, Japan; [Yoon, Seok-Jun] Korea Univ, Dept Prevent Med, Seoul, South Korea; [Younis, Mustafa Z.] Jackson State Univ, Dept Hlth Policy & Management, Jackson, MS USA; [Younis, Mustafa Z.] Tsinghua Univ, Sch Med, Beijing, Peoples R China; [Yusefzadeh, Hasan] Urmia Univ Med Sci, Dept Hlth Care Management & Econ, Orumiyeh, Iran; [Zaidi, Zoubida] Univ Ferhat Abbas Setif, Dept Med, Setif, Algeria; [Zepro, Nejimu Biza] Univ Basel, Dept Epidemiol & Publ Hlth, Basel, Switzerland; [Zerfu, Taddese Alemu] Dilla Univ, Coll Med & Hlth Sci, Dilla, Ethiopia; [Zhang, Yunquan] Wuhan Univ Sci & Technol, Sch Publ Hlth, Wuhan, Peoples R China; [Zhang, Yunquan] Wuhan Univ Sci & Technol, Hubei Prov Key Lab Occupat Hazard Identificat & C, Wuhan, Peoples R China; [Zhao, Xiu-Ju George] Wuhan Polytech Univ, Sch Biol & Pharmaceut Engn, Wuhan, Peoples R China; [Zuniga, Yves Miel H.] Dept Hlth Philippines, Hlth Technol Assessment Unit, Manila, Philippines; [Zuniga, Yves Miel H.] MentalHealthPH, Quezon City, Philippines	Institute for Health Metrics & Evaluation; University of Washington; University of Washington Seattle; University of Washington; University of Washington Seattle; University of Washington; University of Washington Seattle; University of Washington; University of Washington Seattle; University of Washington; University of Washington Seattle; University of Washington; University of Washington Seattle; Tehran University of Medical Sciences; Egyptian Knowledge Bank (EKB); Cairo University; Egyptian Knowledge Bank (EKB); Cairo University; Egyptian Knowledge Bank (EKB); Cairo University; Egyptian Knowledge Bank (EKB); Cairo University; National Hepatology & Tropical Medicine Research Institute (NHTMRI); Egyptian Knowledge Bank (EKB); Cairo University; Egyptian Knowledge Bank (EKB); Cairo University; Isfahan University of Medical Sciences; Isfahan University of Medical Sciences; Near East University; Universidade Federal de Minas Gerais; Universidade Federal de Minas Gerais; Universidade Federal de Minas Gerais; Universidade Federal de

Minas Gerais; Universidade Federal de Minas Gerais; University of London; London School of Hygiene & Tropical Medicine; University of London; London School of Hygiene & Tropical Medicine; University of London; London School of Hygiene & Tropical Medicine; Dongguk University; Hamadan University of Medical Sciences; Hamadan University of Medical Sciences; Hamadan University of Medical Sciences; Hamadan University of Medical Sciences; Hamadan University of Medical Sciences; Stellenbosch University; Stellenbosch University; South African Medical Research Council; South African Medical Research Council; South African Medical Research Council; University of Ibadan; University College Hospital, Ibadan; University of Ibadan; University College Hospital, Ibadan; University of Ibadan; University College Hospital, Ibadan; Obafemi Awolowo University; Obafemi Awolowo University; Cardiff University; Stellenbosch University; Mekelle University; Mekelle University; Mekelle University; Mekelle University; Mekelle University; Mekelle University; Mekelle University; Mekelle University; Mayo Clinic; Mahsa University; University of Nottingham; University of Lincoln; Jimma University; Jimma University; Jimma University; Jimma University; University of South Australia; Bangladesh Rural Advancement Committee BRAC; BRAC University; International Centre for Diarrhoeal Disease Research (ICDDR); International Centre for Diarrhoeal Disease Research (ICDDR); International Centre for Diarrhoeal Disease Research (ICDDR); University of Gondar; University of Gondar; University of Gondar; University of Gondar; Mayo Clinic; Washington University (WUSTL); Queensland Health; Griffith University; Dalhousie University; Imam Abdulrahman Bin Faisal University; Imam Abdulrahman Bin Faisal University; Instituto Nacional de Salud Publica; Instituto Nacional de Salud Publica; Instituto Nacional de Salud Publica; Instituto Nacional de Salud Publica; Instituto Nacional de Salud Publica; Instituto Nacional de Salud Publica; Erbil Polytechnic University; Tishk International University; Tishk International University; Qazvin University of Medical Sciences (QUMS); Iran University of Medical Sciences; Iran University of Medical Sciences; Iran University of Medical Sciences; Iran University of Medical Sciences; Iran University of Medical Sciences; Iran University of Medical Sciences; Iran University of Medical Sciences; Iran University of Medical Sciences; Iran University of Medical Sciences; Iran University of Medical Sciences; Iran University of Medical Sciences; Kuwait University; Kermanshah University of Medical Sciences; Kermanshah University of Medical Sciences; Kermanshah University of Medical Sciences; Kermanshah University of Medical Sciences; Kermanshah University of Medical Sciences; Kermanshah University of Medical Sciences; Kermanshah University of Medical Sciences; Kermanshah University of Medical Sciences; Kermanshah University of Medical Sciences; Kermanshah University of Medical Sciences; Kermanshah University of Medical Sciences; Kermanshah University of Medical Sciences; Kermanshah University of Medical Sciences; Kermanshah University of Medical Sciences; Kermanshah University of Medical Sciences; Kermanshah University of Medical Sciences; Jazan University; Jazan University; King Saud University; King Saud University; Universidad de Cartagena; Universidad de la Costa; Universidad de la Costa; University of the Philippines System; University of the Philippines Manila; University of the Philippines System; University of the Philippines Manila; University of the Philippines System;

University of the Philippines Manila; Johns Hopkins University; Johns Hopkins University; Johns Hopkins University; Johns Hopkins University; Carol Davila University of Medicine & Pharmacy; Carol Davila University of Medicine & Pharmacy; Carol Davila University of Medicine & Pharmacy; National Institute for Infectious Diseases "Matei Bals"; Tabriz University of Medical Science; Tabriz University of Medical Science; Tabriz University of Medical Science; Razi Vaccine & Serum Research Institute; Hong Kong Polytechnic University; Hong Kong Polytechnic University; University of Tasmania; Menzies Institute for Medical Research; Sebelas Maret University; Egyptian Knowledge Bank (EKB); Ain Shams University; Egyptian Knowledge Bank (EKB); Ain Shams University; Egyptian Knowledge Bank (EKB); Ain Shams University; Birmingham City University; Yasouj University; Western Sydney University; University of Sydney; University of Sydney; Babol University of Medical Sciences; Babol University of Medical Sciences; Babol University of Medical Sciences; Babol University of Medical Sciences; Babol University of Medical Sciences; Dr DY Patil Vidyapeeth Pune; Dr D Y Patil Medical College, Hospital & Research Centre; University of Leicester; Bucharest University of Economic Studies; La Trobe University; La Trobe University; Instituto Nacional de Salud - Peru; Curtin University; Burnet Institute; South Australian Health & Medical Research Institute (SAHMRI); University of Kwazulu Natal; University of Kwazulu Natal; University of Kwazulu Natal; University of Kwazulu Natal; University of Kwazulu Natal; University of Lagos; University of Lagos; Public Health Agency of Canada; University of Toronto; University of Toronto; University of Toronto; All India Institute of Medical Sciences (AIIMS) New Delhi; All India Institute of Medical Sciences (AIIMS) New Delhi; All India Institute of Medical Sciences (AIIMS) New Delhi; Manipal Academy of Higher Education (MAHE); Manipal Academy of Higher Education (MAHE); Manipal Academy of Higher Education (MAHE); Haramaya University; Haramaya University; Haramaya University; Haramaya University; Medical University Lodz; Polish Mother's Memorial Hospital - Research Institute; University of London; Egyptian Knowledge Bank (EKB); Alexandria University; Egyptian Knowledge Bank (EKB); Alexandria University; Egyptian Knowledge Bank (EKB); Alexandria University; Clinical Centre of Serbia; University of Belgrade; University of Belgrade; University of Belgrade; Ruprecht Karls University Heidelberg; Harvard University; Harvard T.H. Chan School of Public Health; Harvard University; Harvard University; Harvard Medical School; Harvard University; Harvard University Medical Affiliates; Brigham & Women's Hospital; Harvard University; Harvard University; Harvard University; Imperial College London; Imperial College London; Imperial College London; Imperial College London; Imperial College London; Vietnam National University Ho Chi Minh City (VNUHCM) System; VNU-HCM University of Technology (HCMUT); Shiraz University of Medical Science; Shiraz University of Medical Science; Shiraz University of Medical Science; Shiraz University of Medical Science; New York University; Lorestan University of Medical Sciences; Lorestan University of Medical Sciences; Lorestan University of Medical Sciences; University of Debrecen; Yale University; University of Oxford; University of Oxford; University of Oxford; Arba Minch University; Arba Minch University; Emory University; Emory University; State University System of Florida; University of South Florida; University of Massachusetts System; Department of Biotechnology (DBT) India;

National Institute of Biomedical Genomics (NIBMG); University of Calcutta; George Institute for Global Health; University of New South Wales Sydney; University of New South Wales Sydney; University of New South Wales Sydney; University of New South Wales Sydney; Aga Khan University; Istituto di Ricerche Farmacologiche Mario Negri IRCCS; Swinburne University of Technology; Islamic Azad University; University of Genoa; Bauman Moscow State Technical University; Sechenov First Moscow State Medical University; Sechenov First Moscow State Medical University; Technical University of Berlin; University of Waterloo; Nanyang Technological University; Universidade do Porto; Universidade do Porto; Universidad Nacional de Colombia; Universidad Nacional de Colombia; Indian Council of Medical Research (ICMR); Indian Council of Medical Research (ICMR); University of Melbourne; Christian Medical College & Hospital (CMCH) Vellore; Christian Medical College & Hospital (CMCH) Vellore; Christian Medical College & Hospital (CMCH) Vellore; University of Milan; IRCCS Istituto Ortopedico Galeazzi; University of Milan; University System of Ohio; Case Western Reserve University; University System of Ohio; Case Western Reserve University; Public Health Foundation of India; Public Health Foundation of India; Public Health Foundation of India; Egyptian Knowledge Bank (EKB); Tanta University; Mazandaran University of Medical Sciences; Mazandaran University of Medical Sciences; Mazandaran University of Medical Sciences; Mazandaran University of Medical Sciences; Mazandaran University of Medical Sciences; University of Sussex; University of Brighton; University of Sussex; University of Brighton; Addis Ababa University; Addis Ababa University; Addis Ababa University; Addis Ababa University; Addis Ababa University; University of London; University College London; University of London; University College London; University of London; University College London; International Institute for Population Sciences; International Institute for Population Sciences; Universidad Nacional Autonoma de Mexico; University of Ibadan; University of Ibadan; Ministry of Health & Medical Education (MOHME); Western Sydney University; Hawassa University; Universidade Federal da Bahia; Universidade Federal da Bahia; State University System of Florida; Florida International University; Universiti Sains Malaysia; University of Newcastle; Catholic University of Lublin; World Health Organization; Bahir Dar University; Bahir Dar University; CGIAR; International Institute of Tropical Agriculture (IITA); Ardabil University of Medical Sciences; Auckland University of Technology; Research Center of Neurology; University of Ottawa; Kaiser Permanente; A.T. Still University of Health Sciences; James Cook University; Kobe University; Cleveland Clinic Foundation; University of North Carolina; University of North Carolina Chapel Hill; Flinders University South Australia; University of Adelaide; University of Oklahoma System; University of Oklahoma Health Sciences Center; Universidade Federal do Rio Grande do Sul; University of Sulimanyah; Universidade Federal de Goias; Binzhou Medical University; Eternal Heart Care Centre & Research Institute; Shahid Beheshti University Medical Sciences; Shahid Beheshti University Medical Sciences; Arabian Gulf University; Airlangga University; Egyptian Knowledge Bank (EKB); Al Azhar University; University of Queensland; University of Queensland; ARC Centre of Excellence for Children & Families Over the Life Course; University of Antwerp; London South Bank University; University of Bath; University of

Bath; Nguyen Tat Thanh University (NTTU); Qatar Foundation (QF); Hamad Bin Khalifa University-Qatar; Central South University; University of the Western Cape; Taipei Medical University; Taipei Medical University; Deakin University; University of Sydney; Banaras Hindu University (BHU); Duke University; Duke University; Capital Medical University; University of Opole; All India Institute of Medical Sciences (AIIMS) Jodhpur; University of Munster; Utrecht University; Helmholtz Association; German Cancer Research Center (DKFZ); Leibniz Association; Leibniz Institute for Prevention Research & Epidemiology (BIPS); University of Nairobi; University of Nairobi; University of Cape Town; University of Cape Town; University of Management & Technology (UMT); Ministry of Education of Azerbaijan Republic; Baku State University; Azerbaijan National Academy of Sciences (ANAS); Institute of Radiation Problems of the Azerbaijan National Academy of Sciences; Dow University of Health Sciences; Dow University of Health Sciences; University System of Ohio; Ohio University; Ball State University; Xiamen University Malaysia Campus; Simmons University; Oslo Metropolitan University (OsloMet); Krishna Vishwa Vidyapeeth; Krishna Institute of Medical Sciences; Sher-i-Kashmir Institute of Medical Sciences; CIBER - Centro de Investigacion Biomedica en Red; CIBERSAM; ICREA; University of Oxford; University of Baghdad; Deakin University; Father Muller Medical College; Indian Council of Medical Research (ICMR); ICMR - National Institute of Nutrition (NIN); Chinese Center for Disease Control & Prevention; Brown University; Egyptian Knowledge Bank (EKB); Mansoura University; Tribhuvan University; University of Manitoba; Instituto Federal do Ceara (IFCE); University of Liverpool; University of London; King's College London; University of London; King's College London; University of California System; University of California San Francisco; United Nations Population Fund; University of Helsinki; Helsinki University Central Hospital; Ministry of Health - Kyrgyzstan; Kyrgyz State Medical Academy; Kurdistan University of Medical Sciences; Samara University; Central University of Technology; Tarbiat Modares University; University of Alabama System; University of Alabama Birmingham; Bahir Dar University; University of Bucharest; University of Nigeria; California State University System; San Diego State University; University of Benin; Manipal Academy of Higher Education (MAHE); Kasturba Medical College, Mangalore; University of Ottawa; Ottawa Hospital Research Institute; University of Melbourne; University of Melbourne; University of Munich; Fundacao Oswaldo Cruz; University of Groningen; Fundacion Valle del Lili; Universidad ICESI; Sharif University of Technology; State University System of Florida; University of Central Florida; Hamamatsu University School of Medicine; University of Lahore; University of London; University College London; University College London Hospitals NHS Foundation Trust; Western Sydney University; University of Pretoria; Birjand University of Medical Sciences; Southern University of Science & Technology; University of Sassari; Indian Council of Medical Research (ICMR); ICMR - National Institute for Research in Environmental Health (NIREH); Central University of Punjab; Marshall University; Hamad Medical Corporation; Bournemouth University; University of North Carolina; University of North Carolina Charlotte; Newcastle University - UK; Berlin Institute of Health; Free University of Berlin; Humboldt University of Berlin; Charite Universitatsmedizin Berlin; BLDE (Deemed to be University); University of Edinburgh; Japan Institute for Health

Security (JIHS); National Institute of Infectious Diseases (NIID); University of Technology Sydney; Universidade Federal de Santa Catarina (UFSC); US Department of Veterans Affairs; Kharkiv National Medical University; University of Calgary; University of York - UK; University of Hull; Deakin University; Muhimbili University of Health & Allied Sciences; University of California System; University of California Irvine; University of Valencia; Central University of Kerala; Jagiellonian University; Collegium Medicum Jagiellonian University; Hanoi Medical University; University of Bologna; Quaid I Azam University; George Washington University; Naresuan University; Addis Continental Institute of Public Health; Wenzhou Medical University; Nanjing University; Nagoya University; University of Jos; University of Hong Kong; National Center for Neurology & Psychiatry - Japan; Juntendo University; Korea University; Jackson State University; Tsinghua University; Urmia University of Medical Sciences; Universite Ferhat Abbas Setif; University of Basel; Dilla University; Wuhan University of Science & Technology; Wuhan University of Science & Technology; Wuhan Polytechnic University   Wiens, KE (corresponding author), Univ Washington, Inst Hlth Metr & Evaluat, Seattle, WA 98195 USA. bcreiner@uw.edu Kalani, Hamed/M-4087-2018; Kasahun, Yawukal/IWE-3195-2023; Patton, George/B-5246-2013; , Van Charles Lansingh/C-8672-2018; Gebremedhin, Ketema/JCN-7040-2023; Guadie, Habtamu/IWD-8924-2023; Rahimi-Movaghar, Afarin/E-9505-2011; Salahshoor, Mohammad/S-2241-2017; Mustafa, Ghulam/AET-4683-2022; Yeshaneh, Alex/AAD-1562-2021; Bhutta, Zulfiqar/ADZ-0156-2022; Amini, Saeed/K-8194-2018; Fareed, Mohammad/E-3701-2018; Violante, Francesco/A-6934-2009; Izadi, Neda/AIB-6552-2022; Sufiyan, Muawiyyah Babale/GWN-2609-2022; Khan, Nuruzzaman/K-8677-2019; Demissie, Dr.Dereje/AAN-3556-2021; Piroozi, Bakhtiar/G-1534-2017; Topor-Madry, Roman/ABF-5449-2020; Najafi, Farid/JSL-1581-2023; Mohebi, Farnam/AGB-7159-2022; Tefera, Yonatal Mesfin/GXV-7491-2022; Sharafi, Kiomars/CAH-6352-2022; Alipour, Vahid/K-5099-2019; yusefzadeh, hasan/F-9850-2017; Sen, Abhijit/AAD-6009-2022; Atre, Sachin/KYQ-8593-2024; Bhandari, Dinesh/H-3159-2017; Tran, Khanh/A-2437-2019; Kugbey, Nuworza/H-9264-2019; Ilesanmi, Olayinka/AAF-8628-2021; Shabaninejad, Hosein/KVZ-3669-2024; Tabuchi, Takahiro/KFQ-0737-2024; Rahimi-Movaghar, Vafa/L-6339-2019; magdy, mohamed/KYO-9748-2024; Feigin, Valery/AAF-2313-2019; Kissoon, Niranjan/AAC-6140-2021; Kalan, Mohammad/AAR-7187-2020; Padubidri, Jagadish Rao/AIC-5229-2022; Acharya, Dilaram/AAD-9825-2020; Prasad, Ravi/AAA-8084-2019; Motta, Jorge/ABI-6006-2020; Ahmed, Muktar/AAY-6783-2020; Jha, Vivek kumar/AHC-6454-2022; Mendes, J/AAU-6390-2021; Sagar, Rajesh/L-7775-2016; Ilic, Milena/W-1498-2019; KUMAR, ANIL/ACD-8340-2022; Davitoiu, Dragos/P-9703-2017; Singh, Jasvinder/R-6172-2019; MARTINI, SANTI/AAW-6554-2020; Bhattacharyya, Krittika/AAA-8258-2021; Abegaz, Kedir/AAE-7080-2021; Onwujekwe, Obinna/O-6232-2014; Butt, Zahid/W-4292-2017; Dhillon, Preeti/ABC-7955-2020; Gill, Tiffany/F-9042-2010; Shamsi, MohammadBagher/M-2601-2017; Ansari, Fereshteh/AAG-9873-2019; Rajati, Fatemeh/S-1041-2017; Jonas, Jost/AEP-3841-2022; , B/HZK-8896-2023; Kasaye, Habtamu/LRT-4361-2024; mansournia, Mohammad/AFA-8899-2022; Bairwa, Mohan/G-9463-2015; Lad, Sheetal/ABB-9395-2020; Balakrishnan, Senthilkumar/J-6908-2014; Shifti, Desalegn/KTH-7715-2024; Al-Aly, Ziyad/S-4439-2016; MULAW, Getahun/JGD-9049-2023; Darwish, Amira/Z-4625-2019; Mini, GK/GMX-3520-2022; Santos, Ana/KHY-6833-2024; Shallo, Seifadin/AEB-0659-2022; GUIMARAES, RAFAEL/IQU-1076-2023; Gebre, Teshome/F-2973-2011;

Yisma, Engida/ABA-4170-2020; NGUYEN, THI HUYEN TRANG/IWM-4126-2023; Jahanmehr, Nader/L-9998-2017; Deribe, Kebede/K-7055-2019; Oancea, Bogdan/C-4147-2011; Karami-Matin, Behzad/P-4845-2017; Suleria, Hafiz Ansar Rasul/D-3385-2013; Foroutan, Masoud/AAD-6837-2020; Mahesh, P/R-1184-2019; Hosseinzadeh, Mehdi/GWV-3822-2022; Kisa, Sezer/GPC-8087-2022; El-Jaafary, Shaimaa/ABI-5451-2020; Guimaraes, Andre/D-8122-2011; Tes, Bel/L-3858-2019; Rahman, Dr. Md. Obaidur/AAK-5762-2020; Muthupandian, Saravanan/F-3835-2012; Ghashghaee, Ahmad/HZJ-9700-2023; Debalkie, Getu/AAH-4043-2020; Nigatu, Solomon/MVY-7735-2025; Sayed, Iman/K-8926-2019; Renzaho, Andre/AAH-7679-2021; Fukumoto, Takeshi/AFN-9234-2022; Bojia, Hunduma/MTG-6230-2025; Elsayed, Omar/O-7012-2018; Hassanvand, Mohammad/J-6695-2014; Soltani, Shahin/CAE-8698-2022; Rawaf, David/JXL-7101-2024; Kengne, Andre/ABB-3696-2020; Memish, Ziad/AEJ-9424-2022; Ginindza, Prof. Themba/N-5762-2013; Safari, Yahya/L-7110-2017; Salimzadeh, Hamideh/AAL-6666-2020; Yatsuya, Hiroshi/L-4213-2016; Khalilov, Rovshan/D-5445-2019; Bell, Michelle/Y-4608-2018; Mihretie, Kebadnew/JAC-4589-2023; Househ, Mowafa/GPX-8430-2022; Hailu, Alemayehu/AAZ-5206-2020; Soshnikov, Sergey/KMY-2416-2024; Rezai, Mohammad/F-6274-2017; Hendrie, Delia/AGO-0265-2022; Asgari, Samaneh/JXW-5396-2024; Iwu, Chinwe/AAF-9662-2019; Lami, Faris/AAW-7666-2021; Maravilla, Joemer/AAE-6806-2019; Hamadeh, Randah/AAE-9720-2020; Naohiro, Yonemoto/Y-3761-2019; Kisa, Adnan/Q-2081-2019; Bayati, Mohsen/R-7729-2017; Fereshtehnejad, Seyed-Mohammad/ABF-6020-2020; Imani-Nasab, Mohammad/I-2283-2016; Kabir, A./M-5979-2016; Zaki, Maysaa/H-3049-2019; Khosravi, Mohammad Hossein/G-3767-2015; Jha, Ravi/ABC-6588-2020; Gesesew, Hailay/AAF-6486-2020; Gayesa, Reta/JOK-5315-2023; Ronfani, Luca/B-6668-2013; Rasella, Davide/ABG-4182-2020; Vardast, Mohammad/F-8329-2017; Leili, Mostafa/A-6329-2013; El Tantawi, Maha/IAN-4212-2023; Kabir, Ashad/AAY-8323-2020; Dubljanin, Eleonora/AAW-6278-2020; Badawi, Alaa/ABC-1300-2021; Brugha, Traolach/T-9326-2019; Almasi-Hashiani, Amir/P-3356-2018; Kamyari, Naser/AAP-1999-2021; Andrei, Catalina/KHU-8658-2024; Tovani-Palone, Marcos Roberto/J-6491-2014; Prada, Sergio/ABC-4648-2020; Abrigo, Michael R.M./GZH-2928-2022; Sharifi, Hamid/B-5493-2019; Ullah, Irfan/L-5150-2017; Heydarpour, Fatemeh/S-9380-2017; Rafiei, Alireza/A-2314-2009; Haj-Mirzaian, Arvin/W-6013-2019; Unnikrishnan, Bhaskaran/O-1025-2015; Alam, Noore/AAU-9616-2020; Sahebkar, Amirhossein/B-5124-2018; Mirzaei, Maryam/ABC-7674-2020; Wickramasinghe, Nuwan/I-3578-2019; Khazaei, Mohammad/I-6520-2016; Wiangkham, Taweewat/I-9818-2019; Oh, In/AAI-9070-2020; Mosapour, Abbas/Q-5418-2016; Varughese, Santosh/W-5989-2019; Manafi, Navid/AAM-7393-2020; Shirkoohi, Reza/ACG-1572-2022; Dhimal, Meghnath/AAD-7261-2021; Manda, Ana/AAD-3352-2019; Tarigan, Ingan/AFQ-5904-2022; Aremu, Olatunde/AAD-1995-2019; Carapetis, Jonathan/H-8933-2014; Mereta, Seid Tiku/HIA-0661-2022; Riahi, Seyed/Y-1531-2018; Hasanpoor, Edris/AAL-2953-2020; Safari-Faramani, Roya/C-7344-2018; Shiri, Rahman/ABB-1780-2021; Edgar, Denova-Gutiérrez/HLW-7955-2023; adabi, maryam/O-8028-2017; Merat, Shahin/A-5478-2009; Remuzzi, Giuseppe/V-9766-2017; Arabloo, Jalal/K-1829-2019; Tekle, Merhawi/ABA-3488-2021; kumar, Pushpendra/GRR-1736-2022; Desalew, Assefa/JCE-0300-2023; Tadesse, Zemenu/ABG-1690-2021; Goulart, Bárbara/N-9444-2018; Roro, Elias/HGU-5987-2022; Jayatilleke, Achala/H-1714-2017; Usman, Muhammad/AAU-8410-2021; Basaleem, Huda/AAX-9878-2020; Rahman, Shafiur/LFV-5206-2024; Peykari, Niloofar/L-4521-2016; McAlinden, Colm/AGZ-8185-2022; naik, Gurudatta/O-7284-2015; Sheikh, Aziz/D-

2818-2009; Moradi-Lakeh, Maziar/ABC-9793-2021; Athari, Seyyed/F-5963-2017; Brady, Oliver/P-9421-2019; Munro, Sandra/S-2611-2019; Almasi, Ali/R-9311-2017; Shafieesabet, Azadeh/AAE-6860-2019; Meressa, Beyene/ABA-8501-2021; Hussen, Mohammedaman Mama/GLT-3052-2022; Ezekannagha, Oluchi/JVZ-4584-2024; Daryani, Ahmad/E-2186-2017; Barnighausen, Till/Y-2388-2019; Mohajer, Bahram/I-9529-2019; Bedi, Neeraj/AGR-0606-2022; fazlzadeh, mehdi/J-6218-2017; Ndwandwe, Duduzile/L-2296-2013; Dimbuene, Zacharie/H-3029-2019; Tabares-Seisdedos, Rafael/H-6432-2013; Mohammadifard, Noushin/M-2244-2018; Duko, Bereket/P-8904-2019; Antriyandarti, Ernoiz/ACE-2361-2022; Tadesse, Degena/AAW-6490-2020; KANCHAN, TANUJ/L-8547-2015; Nnaji, Chukwudi/C-4019-2018; Rezaei, Negar/AAS-8147-2020; Bettencourt, Paulo/V-5793-2017; shiferaw, wondimeneh/ABB-2928-2020; Ausloos, Marcel/AAC-8812-2020; ahmadi, ali/JMC-5690-2023; Biswas, Raaj Kishore/K-4256-2018; Alvis-Guzman, Nelson/D-4913-2013; Kebede, Mihiretu/M-5800-2015; Malekzadeh, Reza/U-1382-2017; Khubchandani, Jagdish/D-1831-2014; Salimi, Yahya/V-2626-2017; Meles, Gebrekiros/AAX-8170-2020; Gebremeskel, GEBREAMLAK/ABB-8484-2020; Car, Josip/H-6755-2015; Rubagotti, Enrico/AAL-1807-2020; Barac, Aleksandra/JBS-0092-2023; Alanzi, Truki/O-2778-2019; Bhaumik, Soumyadeep/JOZ-4026-2023; Bekele, Bayu Begashaw/IVU-9935-2023; wang, yafeng/J-4829-2017; Kassa, Getachew/AFU-1253-2022; Christopher, DJ/R-1479-2019; Khayamzadeh, Maryam/AAD-8977-2019; Adebayo, Oladimeji/Y-5260-2019; Ayano, Getinet/ABD-2311-2020; Damiani, Giovanni/AAG-6507-2019; Gezae, Kebede Embaye/HPC-7086-2023; bohlouli, somayeh/AGA-4983-2022; Ahmadi, Keivan/AAU-5419-2020; Meharie, Birhanu/AAG-2261-2021; Mohamadi-Bolbanabad, Amjad/V-4137-2019; Foigt, Nataliya/AAU-5229-2020; Kochhar, Sonali/H-4036-2012; Rahman, Mohammad Hifz Ur/GVU-3673-2022; Siabani, Soraya/U-7739-2017; Campos, Luciana/IUO-4112-2023; Nagaraja, Sharath/ABD-7238-2021; Abolhassani, Hassan/B-3465-2014; Ding, Eric/K-1118-2019; Kim, Yun Jin/AAE-8281-2019; Bizuneh, Asmamaw/ABA-1868-2021; Soyiri, Dr. Ireneous/IQS-7439-2023; Henry, Nathaniel/ABD-4631-2020; Kelbore, Abraham/ABG-6835-2021; Lee, Paul/F-2549-2010; Sepanlou, Sadaf/H-9343-2016; Manohar, Narendar/AAD-9805-2020; Bakkannavar, Shankar/K-7634-2015; Aryal, Krishna/AFN-8677-2022; Minuye, Binyam/AAJ-9404-2020; Naidoo, Kovin/AAF-5914-2020; Behzadifar, Meysam/K-2781-2016; Saxena, Sonia/G-4821-2013; Rubino, Salvatore/AAC-5848-2022; Abreu, Lucas/I-7369-2016; Behzadifar, Masoud/H-1433-2016; Kassa, Gebrehiwot/AAZ-3135-2020; babaee, Ebrahim/O-4416-2019; Massenburg, Benjamin/AAB-9349-2019; Yousefifard, Mahmoud/P-1621-2018; La Vecchia, Carlo/Z-1710-2019; Gebreslassie, Gebremicheal/AAI-9900-2020; Ghadiri, Keyghobad/N-9733-2017; Yu, chuanhua/I-6711-2016; McGrath, John/G-5493-2010; Mahasha, Phetole/JOK-0316-2023; Moradi, Masoud/AAF-2174-2020; RIOS-BLANCAS, MARIA/HDM-1357-2022; Etemadi, Arash/Y-7082-2018; azzopardi, peter/J-7355-2012; Nojomi, Marzieh/C-7391-2018; Briko, Andrey/AAB-9340-2021; Khater, Mona/ACR-4854-2022; Chu, Dinh-Toi/I-7414-2019; Weetman, David/M-1261-2014; Amit, Arianna Maever/E-4482-2017; Radfar, Amir/I-8057-2019; Toma, Alemayehu/I-2560-2018; Temsah, Mohamad-Hani/AAJ-9703-2020; Karch, André/D-6973-2017; Yimer, Ebrahim/N-4597-2018; Iqbal, Usman/L-2467-2016; girma, bedilu/AFZ-0564-2022; Kumar, Manasi/AAI-9488-2020; Ayala Quintanilla, Beatriz Paulina/C-8325-2016; Ibitoye, Segun/AAV-4960-2020; Awoke, Nefsu/AFV-2432-2022; Armoon, Bahram/P-6089-2018; Memiah, Peter/P-3115-2017; kumar, vivek/JEO-7153-2023; Hasan, Mehedi/HHC-4236-2022; f, m/AAM-2063-2021; K M, Shivakumar/AAV-4508-2020; Aljunid, Syed Mohamed/J-6009-2014;

mohammadi, mokhtar/U-4872-2019; Kabir, Zubair/KQX-5433-2024; Thankappan, Kavumpurathu/ABC-9551-2021; Nazari, Javad/ABE-6348-2021; Samy, Abdallah/B-4375-2010; pirsaheb, meghdad/H-5415-2017; Afarideh, Mohsen/H-8945-2019; Mouodi, Simin/IUQ-0991-2023; Nacimento, Bruno/F-8066-2013; Moraga, Paula/MSW-7814-2025; Gubari, Mohammed/HLW-1535-2023; Antonio, Carl/AAP-4280-2020; Xu, Guofan/KCK-3569-2024; Saravi, Babak/AAV-9666-2020; Selvaraj, Siddharthan/KII-8130-2024; Mekonnen, Tefera/AAC-8002-2021; Karim, Mohd/ABD-6578-2020; Yousof, Hebat-Allah/JMQ-8237-2023; Jalali, Amir/JTT-7337-2023; Perico, Norberto/ABH-3222-2020; Zodpey, Sanjay/B-2820-2011; sadeghi, ehsan/AGR-8484-2022; Miao Jonasson, Junmei/MIN-9869-2025; Karyani, Ali/J-8602-2017; Saad, Anas/L-6672-2017; Li, Shanshan/HLH-7747-2023; Martins-Melo, Francisco/IUM-9000-2023; Siddiqi, Tariq/AAH-1034-2020; Safiri, Saeid/A-1678-2017; Ketema, Daniel/AAI-6801-2020; Lee, Shaun/E-9934-2014; Salem, Maher/ABD-5106-2020; Das, Jai K./JOZ-1359-2023; Hasanzadeh, Amir/AAI-8266-2020; Ziapour, Arash/AAJ-9043-2020; Banach, Maciej/A-1271-2009; Paternina-Caicedo, Angel/N-4496-2015; Naderi, Mehdi/R-4325-2017; Kasaeian, Amir/C-8290-2017; Soofi, Moslem/E-7665-2019; Zaman, Sojib Bin/MFH-9407-2025; Davila, Claudio/F-3722-2019; Liu, Simin/I-3689-2014; Moore, Catrin/K-1980-2017; kumar, vivek/J-4421-2016; Lopez, Jaifred Christian/A-3262-2017; shams-beyranvand, mehran/S-2695-2017; Adetokunboh, Olatunji/O-7224-2018; Hoogar, Praveen/C-4050-2017; Tesfay (PhD), Fisaha Haile/J-5522-2015; SHIN, JAE IL/J-6922-2017; Zaman, Shahaduz/JQI-0849-2023; Accrombessi, Manfred/W-1546-2017; Chattu, Vijay Kumar/C-2778-2014; Renjith, Vishnu/Q-8043-2016; Hasaballah, Ahmed I./G-6829-2019; ALVIS-ZAKZUK, NELSON J./HSH-0822-2023; Ahmad, Sohail/D-6272-2017; Hassen, Hamid/A-1105-2019; Santric Milicevic, Milena/H-6423-2018; Rawal, Lal/AEP-3205-2022; Eftekhari, Aziz/O-5120-2017; Salam, Nasir/C-8049-2015; Quintana, Hedley Knewjen/J-6562-2019; Soyiri, Ireneous/H-4594-2011; Koul, Parvaiz/B-1666-2017; Monasta, Lorenzo/B-1388-2012; Altirkawi, Khalid/D-7302-2017; Shamsizadeh, Morteza/C-6000-2014; Herteliu, Claudiu/K-4643-2014; Rodrigues Duraes, Andre/N-2593-2017; Rana, Saleem/Y-9791-2018; el nahas, nevine/W-1672-2019; Mokdad, Ali/AAD-1232-2022; Sokhan, Anton/Q-2373-2016; Majeed, Azeem/KHC-7311-2024; Castaneda-Orjuela, Carlos/N-2601-2017; Preotescu, Liliana/AFU-6281-2022; Nigatu, Dabere/Q-9332-2018; Igumbor, Ehimario/B-2652-2012; /B-3002-2014; Alahdab, Fares/N-6680-2013; Atan, Hisham Atan/F-3946-2014; Reta, Melese Abate/I-5817-2018; Sreeramareddy, Chandrashekhar/C-5433-2014; Ningrum, Dina Nur Anggraini/KFR-4997-2024; Bragazzi, Nicola/G-1672-2011; Bikbov, Boris/I-4594-2013; Mousavi, Seyyed Meysam/P-7797-2015; Aref, Hany/AAW-3366-2020; Khalid, Nauman/G-8134-2015; Rahim, Fakher/AAC-3905-2019; Fischer, Florian/F-9003-2016; Bhutta, Zulfiqar/L-7822-2015; Bairwa, Mohan/I-3335-2014; Zhang, Yunquan/M-9828-2017; Shannawaz, Mohd/AAF-9702-2020; Waheed, Yasir/F-6390-2015; Das Gupta, Rajat/E-5545-2018; Gomes, Nelson/S-5420-2016; Nabhan, Ashraf/A-1718-2008; Linn, Shai/N-3079-2019; Menezes, Ritesh/A-7480-2015; teklay, girmay/O-9966-2018; Norheim, Ole F./AAC-8771-2020; Balakrishnan, Senthilkumar/A-8552-2016; Rabiee, Navid/K-4407-2019; Zandian, Hamed/J-9351-2017; Heibati, Behzad/AFK-6612-2022; Wang, Yuan-Pang/A-4863-2008; Hariyani, Ninuk/AAI-7738-2021; Mamun, Abdullah/A-4673-2011; Diaz, Daniel/P-1916-2018; Samy, Abdallah/I-1415-2014; Khafaie, Morteza Abdullatif/J-5659-2016; Arzani, Afsaneh/H-4031-2016; Ji, John/AAT-4219-2021; Krishan, Kewal/I-3285-2014; Khan, Ejaz/B-9340-2016; Guo, Yuming/I-8353-2018; Davis, Adrian/E-6022-2015; Franklin,

Richard/H-1731-2012; Negoi, Ionut/A-9039-2012; Rahimi-Movaghar, Vafa/T-7816-2017; Folayan, Morenike Oluwatoyin/A-8632-2018; Yilgwan, Christopher/DJC-9032-2022; Singh, Ambrish/W-2163-2017; Rahman, Muhammad Aziz/B-3380-2009; Thirunavukkarasu, Sathish/ABM-9697-2022; Vollmer, Sebastian/U-6496-2017; Carvalho, Felix/D-4914-2013; khader, yousef/AAE-9620-2019; El Tantawi, Maha/K-4336-2014; Etemadi, Arash/C-1386-2016; Moradi, Ghobad/R-1267-2016; Silva, Diego Augusto Santos/AAB-9249-2020; Demoz, Gebre Teklemariam/K-6986-2019; Hay, Simon/F-8967-2015; Abbastabar, Hedayat/D-8779-2017; Soboka, Matiwos/AAF-2163-2020; Costa, Vera Marisa/D-6284-2013; Chatterjee, Pranab/P-6272-2017; Atalay, Hagos Tasew/O-2286-2016; Pepito, Veincent Christian/AAZ-7140-2020; Musa, Kamarul Imran/N-3198-2015; Naghibi Irvani, Seyed Sina/O-2413-2018; Pourmalek, Farshad/A-8188-2010; Sathian, Brijesh/G-7576-2014; yadollahpour, ali/K-9098-2015; Martins-Melo, Francisco Rogerlandio/I-4868-2012; Resnikoff, Serge/N-2355-2019; Zhao, Xiu-Ju/A-6529-2012; Islam, Sheikh Mohammed Shariful/B-1219-2011; Wiysonge, Charles/A-3843-2008; Mirrakhimov, Erkin/E-6900-2017; Hoseini, Mohammad/M-6971-2017; C Goulart, Alessandra/J-2845-2014; Bijani, Ali/B-1718-2017; Briko, Nikolay/U-4804-2017; Olagunju, Andrew/B-4746-2017; Homaie Rad, Enayatollah/G-5567-2015; Ahmed, Muktar/G-6184-2019; Grada, Ayman/N-3691-2013; Sudaryanto, Agus/G-5534-2014; Fernandes, Eduarda/D-4943-2013; Abdelalim, Ahmed/A-6051-2015; Mohammed, Shafiu/P-2016-2014; Al-Mekhlafi, Hesham/R-3568-2016; Olusanya, Bolajoko/F-4504-2012; Ortega-Altamirano, Doris V./ABA-8549-2020; Endalamaw, Aklilu/P-3617-2019

Paternina-Caicedo, Angel/0000-0002-6332-5174; Mena, Alemayehu Toma/0000-0003-1511-1531; Zepro, Nejimu Biza/0000-0001-8502-7605; Sharifi, Hamid/0000-0002-9008-7618; Naderi, Mehdi/0000-0002-5608-6582; Khan, Dr Nuruzzaman/0000-0002-4550-4363; Jha, Vivekanand/0000-0002-8015-9470; Esteghamati, Alireza/0000-0001-5114-3982; Asadi-Aliabadi, Mehran/0000-0003-1582-5489; Kasaeian, Amir/0000-0003-2018-9368; Soofi, Moslem/0000-0003-4922-8412; Zaman, Sojib Bin/0000-0002-3043-7954; Davila, Claudio/0000-0002-7656-3606; Liu, Simin/0000-0003-2098-3844; Yisma, Engida/0000-0002-7289-9946; , Dharmesh/0009-0000-5068-4955; Moore, Catrin/0000-0002-8639-9846; kumar, vivek/0000-0003-4379-5651; Gupta, Rajeev/0000-0002-8356-3137; Lopez, Jaifred Christian/0000-0002-8273-992X; shams-beyranvand, mehran/0000-0001-9474-0955; Massenburg, Benjamin/0000-0002-8570-5178; Gebreslassie, Gebremicheal/0000-0002-8981-1832; Meles, Gebrekiros/0000-0001-6563-0001; Dandona, Rakhi/0000-0003-0926-788X; Burugina Nagaraja, Sharath/0000-0002-6599-5753; Sufiyan, Muawiyyah Babale/0000-0003-3630-6995; Adetokunboh, Olatunji/0000-0002-4608-3951; Ketema, Daniel/0000-0002-7464-7814; Bohlouli, Somayeh/0000-0003-0854-1875; Wolde, Haileab Fekadu/0000-0001-6490-7048; Briko, Andrey/0000-0002-7284-5649; Sartorius, Benn/0000-0001-6761-2325; Hoogar, Praveen/0000-0003-2170-5643; Tesfay (PhD), Fisaha Haile/0000-0003-0399-1711; Dipeolu, Isaac Oluwafemi/0000-0002-7538-1050; MARTINEZ, GABRIEL/0000-0002-3692-3339; , Tariq Jamal Siddiqi/0000-0002-9672-6628; SHIN, JAE IL/0000-0003-2326-1820; Atre, Sachin/0000-0003-2148-465X; Ullah, Irfan/0000-0001-6992-6723; Zaman, Shahaduz/0000-0003-1340-1869; Accrombessi, Manfred/0000-0001-9550-9413; Li, Shanshan/0000-0002-9021-8470; , Jiansong/0000-0001-6975-620X; Kiadaliri, Ali/0000-0002-4254-9099; Chattu, Vijay Kumar/0000-0001-9840-8335; Renjith, Vishnu/0000-0002-3718-8399; ahmed, seifadin/0000-0001-6415-7515; shetty, suresh/0000-0002-8499-1942; Shalash, Ali/0000-0001-7012-5907; Ngunjiri, Josephine/0000-0003-

3952-0823; arabloo, Jalal/0000-0003-1223-4528; Sheikh, Aziz/0000-0001-7022-3056; Kassa, Getachew M/0000-0002-8095-7376; Khubchandani, Jagdish/0000-0002-9058-4278; Abegaz, Kedir/0000-0001-8194-1643; Hasaballah, Ahmed I./0000-0003-1596-1958; ALVIS-ZAKZUK, NELSON J./0000-0001-9382-214X; Werdecker, Andrea/0009-0002-2370-4409; Ghimire, Pramesh/0000-0002-7902-7294; Ahmad, Sohail/0000-0001-6458-7697; Komaki, Hamidreza/0000-0003-1830-9500; Tran, Bach/0000-0002-2191-3947; Ayala Quintanilla, Beatriz Paulina/0000-0002-2630-4569; Hassen, Hamid/0000-0001-6485-4193; Santric Milicevic, Milena/0000-0002-0684-359X; Rawal, Lal/0000-0003-1106-0108; Armani, Keivan/0000-0001-5674-2765; Eftekhari, Aziz/0000-0003-0274-4479; Salam, Nasir/0000-0001-9133-1304; Quintana, Hedley Knewjen/0000-0002-5929-8791; rezaei, negar/0000-0001-7047-7638; Martini, Santi/0000-0003-2424-1776; Olusanya, Jacob/0000-0002-1566-9554; Soyiri, Ireneous/0000-0003-4697-5156; Shabaninejad, Hosein/0000-0001-9512-1398; Tsegaye, Reta/0000-0003-4721-3647; Koul, Parvaiz/0000-0002-1700-9285; Shifti, Desalegn Markos/0000-0001-6638-6194; Monasta, Lorenzo/0000-0001-7774-548X; Altirkawi, Khalid/0000-0002-7331-4196; Shamsizadeh, Morteza/0000-0002-8153-5482; Hegazy, Mohamed/0000-0001-5012-3998; Demissie, Dr. Dereje Bayissa/0000-0003-1006-4318; Elema, Teshome Bekele/0000-0001-6791-7153; Herteliu, Claudiu/0000-0001-8860-9547; Rodrigues Duraes, Andre/0000-0002-1506-0327; Ranabhat, Chhabi L/0000-0002-4460-2121; Abualhasan, Ahmed/0000-0002-9949-667X; DE LA HOZ RESTREPO, FERNANDO/0000-0001-9436-7935; Magdy Abd El Razek, Hassan/0000-0002-8857-0408; Hamadeh, Randah/0000-0002-4205-1354; Rana, Saleem/0000-0001-6479-9235; Antriyandarti, Ernoiz/0000-0002-0948-4000; Dubljanin, Eleonora/0000-0002-6484-3192; Temsah, Mohamad-Hani/0000-0002-4389-9322; Kamyari, Naser/0000-0001-6245-5447; nazari, javad/0000-0002-8277-6819; Kasaye, Habtamu/0000-0003-3759-2604; Hasan, Md. Mehedi/0000-0001-7801-0506; Moradi, Masoud/0000-0003-2036-5333; el nahas, nevine/0000-0003-0430-6528; Mini, GK/0000-0003-2775-629X; Mokdad, Ali/0000-0002-4994-3339; Shafieesabet, Azadeh/0000-0001-8205-0981; Meressa, Beyene/0000-0002-1864-0765; Amit, Arianna Maever/0000-0003-4571-400X; Yousof, Hebat-Allah Salah A./0000-0001-7214-0991; Perico, Norberto/0000-0002-3147-4327; Welgan, Katie/0000-0002-4310-1632; Moreno Velasquez, Ilais/0000-0001-6058-8983; Wiens, Kirsten E./0000-0003-4093-4054; Alam, Noore/0000-0002-1150-7582; Dharmaratne, Samath/0000-0003-4144-2107; Adekanmbi, Victor/0000-0002-7394-1640; alcalde rabanal, jacqueline elizabeth/0000-0002-9172-2302; Sokhan, Anton/0000-0003-1860-3099; Foigt, Nataliya/0000-0002-3613-5965; Majeed, Azeem/0000-0002-2357-9858; Gubari, Mohammed Ibrahim Mohialdeen/0000-0002-2873-3981; Castaneda-Orjuela, Carlos/0000-0002-8735-6223; sajadi, s.mohammad/0000-0001-8284-5178; Preotescu, Liliana/0000-0002-3328-4789; Nigatu, Dabere/0000-0001-7303-6723; Igumbor, Ehimario/0000-0002-6313-6031; /0000-0002-9160-6846; Alahdab, Fares/0000-0001-5481-696X; Atan, Hisham Atan/0000-0002-3379-6063; Reta, Melese Abate/0000-0003-1301-4746; Sreeramareddy, Chandrashekhar/0000-0002-5693-7631; Ningrum, Dina Nur Anggraini/0000-0002-8557-2862; Manafi, Navid/0000-0002-4610-402X; Bragazzi, Nicola/0000-0001-8409-868X; Eathakkattu Antony, Benny Samuel/0000-0001-8704-6084; Ibitoye, Segun Emmanuel/0000-0002-5074-816X; Hird, Thomas Robert/0000-0003-3423-4617; Longbottom, Joshua/0000-0002-4151-9031; Chu, Dinh Toi/0000-0002-4596-2022; Das, Jai/0000-0002-2966-7162; Bikbov, Boris/0000-0002-1925-7506; Mousavi, Seyyed Meysam/0000-0002-6795-7224; Aref, Hany/0000-0003-1497-7915; Birhane, Binyam Minuye/0000-0002-9338-0334; /0000-0002-

0102-1722; Zuniga, Yves Miel/0000-0003-4015-3995; Wiangkham, Taweewat/0000-0003-4115-704X; Mohammad, Karzan/0000-0003-1631-5675; Khalid, Nauman/0000-0002-8045-199X; Lami, Faris/0000-0002-3673-3818; Rahim, Fakher/0000-0002-2857-4562; Ilic, Milena/0000-0003-3229-4990; Fischer, Florian/0000-0002-4388-1245; Ayanore, Martin/0000-0002-4095-3047; Shiri, Rahman/0000-0002-9312-3100; Bhutta, Zulfiqar/0000-0003-0637-599X; Bairwa, Mohan/0000-0001-7763-2530; Zhang, Yunquan/0000-0002-2618-5088; Usman, Muhammad/0000-0001-9747-8892; Khater, Mona/0000-0001-8499-5858; Shannawaz, Mohd/0000-0001-5114-5814; Waheed, Yasir/0000-0002-5789-4215; Das Gupta, Rajat/0000-0002-7680-676X; Ribeiro, Ana Isabel/0000-0001-8880-6962; Gomes, Nelson/0000-0002-8727-9678; Bizuwork, Ketema/0000-0003-3335-8052; /0000-0002-0131-0700; Nabhan, Ashraf/0000-0003-4572-2210; Linn, Shai/0000-0002-0867-2958; Lami, Faris/0009-0007-1552-8325; Tessema, Belay/0000-0003-1475-7357; Padubidri, Jagadish Rao/0000-0002-8671-7664; Pourshams, Akram/0000-0002-7950-3983; azzopardi, peter/0000-0002-9280-6997; Acharya, Dilaram/0000-0003-2270-7667; Olagunju, Tinuke/0000-0003-4019-8755; Pakhale, Smita/0000-0002-4051-962X; Kimokoti, Ruth/0000-0002-4980-3256; Menezes, Ritesh/0000-0002-2135-4161; Dhimal, Meghnath/0000-0001-7176-7821; teklay, girmay/0000-0002-3392-6585; Norheim, Ole F./0000-0002-5748-5956; Iwu-Jaja, Chinwe/0000-0003-0765-7497; Bhaumik, Soumyadeep/0000-0001-9579-4453; Balakrishnan, Senthilkumar/0000-0003-4117-9695; Dhillon, Preeti/0000-0001-9757-6492; Alvis-Guzman, Nelson/0000-0001-9458-864X; Rabiee, Navid/0000-0002-6945-8541; KM, Shivakumar/0000-0002-8062-9209; Deribe, Kebede/0000-0002-8526-6996; Zandian, Hamed/0000-0002-1284-5823; Heibati, Behzad/0000-0002-1640-8428; Adedoyin, Rufus/0000-0001-6877-6997; Wang, Yuan-Pang/0000-0001-7076-8312; Hariyani, Ninuk/0000-0003-0807-0081; Mamun, Abdullah/0000-0002-1535-8086; Kisa, Adnan/0000-0001-7825-3436; Bhattacharyya, Krittika/0000-0002-9914-7031; Mahasha, Phetole/0000-0002-5750-3595; Diaz, Daniel/0000-0003-2302-1982; Samy, Abdallah/0000-0003-3978-1134; Bhattarai, Suraj/0000-0001-6843-6677; Khafaie, Morteza Abdullatif/0000-0002-1651-3017; Arzani, Afsaneh/0000-0002-0562-4176; Pescarini, Julia/0000-0001-8711-9589; Rafiei, AliReza/0000-0002-1766-6605; Ji, John/0000-0002-5002-118X; Tabuchi, Takahiro/0000-0002-1050-3125; Bennett, Derrick/0000-0002-9170-8447; JHA, RAVI PRAKASH/0000-0001-5230-1436; Krishan, Kewal/0000-0001-5321-0958; Paudel, Deepak/0000-0003-3562-5337; Khan, Ejaz/0000-0002-7072-8035; Guo, Yuming/0000-0002-1766-6592; Haririan, Hamidreza/0000-0002-5714-8669; Wonde, Tewodros/0000-0003-3323-8202; Davis, Adrian/0000-0001-7134-7528; Farzadfar, Farshad/0000-0001-8288-4046; Franklin, Richard/0000-0003-1864-4552; Prada Rios, Sergio Ivan/0000-0001-7986-0959; Negoi, Ionut/0000-0002-6950-9599; Hasanzadeh, Amir/0000-0003-2893-4369; kasahun, yawukal/0000-0002-4758-7780; Rahimi-Movaghar, Vafa/0000-0001-7347-8767; Nnaji, Chukwudi/0000-0002-4132-1922; Rezai, Mohammad Sadegh/0000-0003-4585-9954; Yisma, Engida/0000-0003-0703-1515; Folayan, Morenike Oluwatoyin/0000-0002-9008-7730; Mereta, Seid Tiku/0000-0001-8036-4995; Tabares-Seisdedos, Rafael/0000-0002-1089-2204; Ross, Jennifer/0000-0001-5677-939X; Yilgwan, Christopher/0000-0003-4741-7009; Abrigo, Michael R.M./0000-0002-8187-4302; Senthilkumaran, Subramanian/0000-0001-5262-8367; Singh, Ambrish/0000-0002-4618-7507; Culquichicon, Carlos/0000-0001-5349-3521; Rahman, Muhammad Aziz/0000-0003-1665-7966; , KR/0000-0002-4536-2684; Muthupandian, Saravanan/0000-0002-1480-3555; Thirunavukkarasu, Sathish/0000-0002-2016-4964;

Gopalani, Sameer/0000-0003-0611-305X; Lacey, Ben/0000-0003-0139-2934; Damiani, Giovanni/0000-0002-2390-6505; Vollmer, Sebastian/0000-0002-7863-0462; Kengne, Andre Pascal/0000-0002-5183-131X; Carvalho, Felix/0000-0003-3858-3494; khader, yousef/0000-0002-7830-6857; El Tantawi, Maha/0000-0003-4989-6584; Etemadi, Arash/0000-0002-3458-1072; Moradi, Ghobad/0000-0003-2612-6528; Silva, Diego Augusto Santos/0000-0002-0489-7906; Duko, Bereket/0000-0002-4419-0016; Demoz, Gebre Teklemariam/0000-0002-2534-821X; Babaee, Ebrahim/0000-0001-7969-9122; Kugbey, Nuworza/0000-0002-0413-0350; Osarenotor, Osayomwanbo/0000-0002-3041-4445; Fareed, Mohammad/0000-0003-4311-8693; Hay, Simon/0000-0002-0611-7272; Britton, Gabrielle/0000-0002-1758-2495; Abbastabar, Hedayat/0000-0002-5713-4806; Soboka, Matiwos/0000-0003-2820-0947; Sabde, Yogesh/0000-0003-1787-2553; Mathur, Manu/0000-0001-5518-1935; ausloos, marcel/0000-0001-9973-0019; Rasella, Davide/0000-0002-7260-4386; Elhabashy, Hala/0000-0002-4865-3903; Moazen, Babak/0000-0002-2552-0438; Barac, Aleksandra/0000-0002-0132-2277; Yaya, Getinet Ayano/0000-0002-9137-4141; Costa, Vera Marisa/0000-0002-0471-2756; Desalew, Assefa/0000-0001-6065-0708; Guido, Davide/0000-0002-3291-3686; Postma, Maarten/0000-0002-6306-3653; RIOS BLANCAS, MARIA JESUS/0000-0001-7100-9262; Kissoon, Niranjan/0000-0001-8847-9973; SAGAR, RAJESH/0000-0003-4563-7841; Chatterjee, Pranab/0000-0001-6443-608X; Campos, Ismael/0000-0001-5939-3396; Atalay, Hagos Tasew/0000-0002-0886-815X; Pepito, Veincent Christian/0000-0001-5391-3784; Khalilov, Rovshan/0000-0002-8684-1390; Musa, Kamarul Imran/0000-0002-3708-0628; Topor-Madry, Roman/0000-0002-3091-6760; Haj-mirzaian, Arya/0000-0002-0724-2649; Naghibi Irvani, Seyed Sina/0000-0002-4566-7402; Pourmalek, Farshad/0000-0002-2134-0771; Ogah, Okechukwu/0000-0002-2093-7787; Gezae, Kebede Embaye/0000-0002-5874-3304; Bekele, Alex Yeshaneh/0000-0001-5876-7325; LASRADO, SAVITA/0000-0001-9261-9238; Sathian, Brijesh/0000-0003-0851-4762; yadollahpour, ali/0000-0002-1216-2109; Ndwandwe, Duduzile/0000-0001-7129-3865; Martins-Melo, Francisco Rogerlandio/0000-0003-3690-5023; Resnikoff, Serge/0000-0002-5866-4446; Kabir, Zubair/0000-0003-1529-004X; Younis, Mustafa/0000-0001-8448-808X; Alanzi, Turki/0000-0001-6598-1274; Zhao, Xiu-Ju/0000-0003-4962-6285; Bekele, Muluken/0000-0002-3464-2584; Islam, Sheikh Mohammed Shariful/0000-0001-7926-9368; Wiysonge, Charles/0000-0002-1273-4779; Mouodi, Simin/0000-0001-7868-9360; KASSA, GEBREHIWOT GEBRETSADIK/0000-0001-6848-1048; Mirrakhimov, Erkin/0000-0003-2982-6108; Hoseini, Mohammad/0000-0001-5180-3047; Meretoja, Tuomo/0000-0002-2691-0710; Aremu, Olatunde/0000-0002-5832-2403; C Goulart, Alessandra/0000-0003-1076-5210; Kosen, Soewarta/0000-0002-2517-8118; Bijani, Ali/0000-0003-2233-8726; Briko, Nikolay/0000-0002-6446-2744; Haj-Mirzaian, Arvin/0000-0001-8977-6865; Gesesew, Hailay/0000-0002-3531-4400; Olagunju, Andrew/0000-0003-1736-9886; Miller, Ted/0000-0002-0958-2639; Homaie Rad, Enayatollah/0000-0002-9064-0380; Ahmed, Muktar/0000-0002-9524-7027; Sunguya, Bruno/0000-0003-3625-0725; Awoke, Nefsu/0000-0002-1037-5004; Grada, Ayman/0000-0002-5321-0584; Sudaryanto, Agus/0000-0002-5886-0295; Fernandes, Eduarda/0000-0001-6424-0976; Izadi, Neda/0000-0002-6373-1113; Abdelalim, Ahmed/0000-0001-6445-5539; Oancea, Bogdan/0000-0001-6987-5137; Mulaw, Getahun Fentaw/0000-0002-4173-3759; Rajati, Fatemeh/0000-0001-6426-664X; Lindstedt, Paulina/0000-0001-6623-430X; Mohammed, Shafiu/0000-0001-5715-966X; Al-Mekhlafi, Hesham/0000-0003-2582-7410; Wasif, Muhammad/0009-0004-1374-302X; Olusanya,

Bolajoko/0000-0002-3826-0583; Ortega-Altamirano, Doris V./0000-0003-4767-8268; RENZAHO, ANDRE/0000-0002-6844-0833; Servan-Mori, Edson/0000-0001-9820-8325; Endalamaw, Aklilu/0000-0002-9121-6549

Bill AMP; Melinda Gates Foundation [OPP1132415, OPP1127433]; Coordenacao de Aperfeicoamento de Pessoal de Nivel Superior [001]; Conselho Nacional de Desenvolvimento Cientifico e Tecnologico; Fundacao de Amparo a Pesquisa do Estado de Minas Gerais; Department of Health Policy and Management, Faculty of Public Health, Kuwait University; International Centre for Casemix and Clinical Coding, Faculty of Medicine, National University of Malaysia; Romanian National Authority for Scientific Research and Innovation, CNDS-UEFISCDI [PN-III-P4-ID-PCCF-2016-0084]; Australian National Health and Medical Research Council (NHMRC) early career fellowship; Public Health Agency of Canada; Alexander von Humboldt Foundation through the Alexander von Humboldt Professor award - German Federal Ministry of Education and Research; EU; Wellcome Trust; National Institute of Child Health and Human Development of National Institutes of Health (NIH) [R01-HD084233]; National Institute on Aging of NIH [P01-AG041710]; National Institute of Allergy and Infectious Diseases of NIH [R01-AI124389, R01-AI112339]; Fogarty International Center of NIH [D43-TW009775]; Sistema Nacional de Investigacion (SNI) de la Secretaria Nacional de Ciencia, Tecnologia e Innovacion (SENACYT) of Panama; Project of Ministry of Education, Science and Technology of the Republic of Serbia [III45005]; National Institute for Health Research (NIHR) Oxford Biomedical Research Centre; Portuguese national funds through Fundacao para a Ciencia e Tecnologia (FCT), IP, under the Norma Transitoria [SFRH/BHD/110001/2015, DL57/2016/CP1334/CT0006]; FCT/Ministerio da Ciencia, Tecnologia e Ensino Superior through national funds [UID/MULTI/04378/2019, UID/QUI/50006/2019]; Wellcome Trust as part of his International Intermediate Fellowship [201900/Z/16/Z]; European Fund for Regional Development through the Operational Program for Competitiveness [P_40_382]; National Heart Foundation of Australia; Deakin University; Ministry of Education Science and Technological Development of the Republic of Serbia [OI175014]; South African Medical Research Council; Research Management Centre, Xiamen University Malaysia [XMUMRF/2018-C2/ITCM/0001]; DST PURSE grant; UGC Center of Advanced Study; NIHR Oxford Biomedical Research Centre; British Heart Foundation Centre of Research Excellence, Oxford; NIHR Biomedical Research Center at Guy's and St Thomas' National Health Service Foundation Trust and King's College London; Sistema Nacional de Investigacion (SENACYT, Panama); NHMRC Fellowship; Egyptian Fulbright Mission programme; Ministry of Education, Science and Technological Development of the Republic of Serbia [175087]; Health Data Research UK; Instituto de Salud Carlos III-FEDER [PI17/00719]; [K43 TW010716-03]; Bill and Melinda Gates Foundation [OPP1127433] Funding Source: Bill and Melinda Gates Foundation Bill AMP; Melinda Gates Foundation(CGIAR); Coordenacao de Aperfeicoamento de Pessoal de Nivel Superior(Coordenacao de Aperfeicoamento de Pessoal de Nivel Superior (CAPES)); Conselho Nacional de Desenvolvimento Cientifico e Tecnologico(Conselho Nacional de Desenvolvimento Cientifico e Tecnologico (CNPQ)); Fundacao de Amparo a Pesquisa do Estado de Minas Gerais(Fundacao de Amparo a Pesquisa do Estado de Minas Gerais (FAPEMIG)); Department of Health Policy and Management, Faculty of Public Health, Kuwait University; International Centre for Casemix and Clinical Coding, Faculty of

Medicine, National University of Malaysia; Romanian National Authority for Scientific Research and Innovation, CNDS-UEFISCDI(Consiliul National al Cercetarii Stiintifice (CNCS)Unitatea Executiva pentru Finantarea Invatamantului Superior, a Cercetarii, Dezvoltarii si Inovarii (UEFISCDI)); Australian National Health and Medical Research Council (NHMRC) early career fellowship(National Health & Medical Research Council (NHMRC) of Australia); Public Health Agency of Canada; Alexander von Humboldt Foundation through the Alexander von Humboldt Professor award - German Federal Ministry of Education and Research; EU(European Union (EU)); Wellcome Trust(Wellcome Trust); National Institute of Child Health and Human Development of National Institutes of Health (NIH); National Institute on Aging of NIH(United States Department of Health & Human ServicesNational Institutes of Health (NIH) - USANIH National Institute on Aging (NIA)); National Institute of Allergy and Infectious Diseases of NIH; Fogarty International Center of NIH(United States Department of Health & Human ServicesNational Institutes of Health (NIH) - USANIH Fogarty International Center (FIC)); Sistema Nacional de Investigacion (SNI) de la Secretaria Nacional de Ciencia, Tecnologia e Innovacion (SENACYT) of Panama; Project of Ministry of Education, Science and Technology of the Republic of Serbia; National Institute for Health Research (NIHR) Oxford Biomedical Research Centre(National Institutes of Health Research (NIHR)); Portuguese national funds through Fundacao para a Ciencia e Tecnologia (FCT), IP, under the Norma Transitoria(Fundacao para a Ciencia e a Tecnologia (FCT)); FCT/Ministerio da Ciencia, Tecnologia e Ensino Superior through national funds; Wellcome Trust as part of his International Intermediate Fellowship(Wellcome Trust); European Fund for Regional Development through the Operational Program for Competitiveness; National Heart Foundation of Australia(National Heart Foundation of Australia); Deakin University; Ministry of Education Science and Technological Development of the Republic of Serbia(Ministry of Education, Science & Technological Development, Serbia); South African Medical Research Council(South African Medical Research CouncilUK Research & Innovation (UKRI)Medical Research Council UK (MRC)); Research Management Centre, Xiamen University Malaysia; DST PURSE grant; UGC Center of Advanced Study; NIHR Oxford Biomedical Research Centre(National Institutes of Health Research (NIHR)); British Heart Foundation Centre of Research Excellence, Oxford; NIHR Biomedical Research Center at Guy's and St Thomas' National Health Service Foundation Trust and King's College London; Sistema Nacional de Investigacion (SENACYT, Panama); NHMRC Fellowship(National Health & Medical Research Council (NHMRC) of Australia); Egyptian Fulbright Mission programme; Ministry of Education, Science and Technological Development of the Republic of Serbia(Ministry of Education, Science & Technological Development, Serbia); Health Data Research UK; Instituto de Salud Carlos III-FEDER(Instituto de Salud Carlos IIIEuropean Union (EU)Spanish Government); ; Bill and Melinda Gates Foundation(Bill & Melinda Gates Foundation) This work was primarily supported by a grant from the Bill & Melinda Gates Foundation (OPP1132415). L G Abreu has received support from Coordenacao de Aperfeicoamento de Pessoal de Nivel Superior (Finance Code 001), Conselho Nacional de Desenvolvimento Cientifico e Tecnologico and Fundacao de Amparo a Pesquisa do Estado de Minas Gerais. O O Adetokunboh acknowledges the South African Department of Science and Innovation and the

National Research Foundation. S M Aljunid acknowledges the Department of Health Policy and Management, Faculty of Public Health, Kuwait University and International Centre for Casemix and Clinical Coding, Faculty of Medicine, National University of Malaysia for the approval and support to participate in this research project. H T Atalay acknowledges Aksum University. M Ausloos and C Herteliu are partially supported by a grant of the Romanian National Authority for Scientific Research and Innovation, CNDS-UEFISCDI, project number PN-III-P4-ID-PCCF-2016-0084. P S Azzopardi was supported by an Australian National Health and Medical Research Council (NHMRC) early career fellowship. A Badawi is supported by the Public Health Agency of Canada. T W Barnighausen was supported by the Alexander von Humboldt Foundation through the Alexander von Humboldt Professor award, funded by the German Federal Ministry of Education and Research; the EU; the Wellcome Trust; and from National Institute of Child Health and Human Development of National Institutes of Health (NIH; R01-HD084233), National Institute on Aging of NIH (P01-AG041710), National Institute of Allergy and Infectious Diseases of NIH (R01-AI124389 and R01-AI112339), as well as Fogarty International Center of NIH (D43-TW009775). G B Britton is supported by Sistema Nacional de Investigacion (SNI) de la Secretaria Nacional de Ciencia, Tecnologia e Innovacion (SENACYT) of Panama. A Barac is funded by the Project of Ministry of Education, Science and Technology of the Republic of Serbia (number III45005). D A Bennett was supported by the National Institute for Health Research (NIHR) Oxford Biomedical Research Centre. The views expressed are those of the authors and not necessarily those of the National Health Service, the NIHR, or the UK Department of Health and Social Care. V M Costa acknowledges her grant (SFRH/BHD/110001/2015), received by Portuguese national funds through Fundacao para a Ciencia e Tecnologia (FCT), IP, under the Norma Transitoria DL57/2016/CP1334/CT0006. F Carvalho acknowledges UID/MULTI/04378/2019 and UID/QUI/50006/2019 support with funding from FCT/Ministerio da Ciencia, Tecnologia e Ensino Superior through national funds. K Deribe is supported by a Wellcome Trust grant (number 201900/Z/16/Z) as part of his International Intermediate Fellowship. C Herteliu is partially supported by a grant co-funded by European Fund for Regional Development through the Operational Program for Competitiveness (project ID P_40_382). P Hoogar thanks Centre for Bio Cultural Studies, Directorate of Research, Manipal Academy of Higher Education, Manipal and Centre for Holistic Development and Research, Kalaghatgi-Karnataka. S M S Islam is funded by a Fellowship from National Heart Foundation of Australia and Deakin University. M Jakovljevic and the Serbian part of this GBD contribution was co-funded through grant OI175014 of the Ministry of Education Science and Technological Development of the Republic of Serbia. A P Kengne is supported by the South African Medical Research Council. r Y J Kim's work was supported by the Research Management Centre, Xiamen University Malaysia, grants number XMUMRF/2018-C2/ITCM/0001. K Krishan is supported by a DST PURSE grant and UGC Center of Advanced Study awarded to the Department of Anthropology, Panjab University, Chandigarh, India. M Kumar acknowledges K43 TW010716-03. B Lacey acknowledges support from the NIHR Oxford Biomedical Research Centre and the British Heart Foundation Centre of Research Excellence, Oxford. P T N Memiah acknowledges the Council for the Development of Social Science

Research in Africa. M Molokhia is supported by the NIHR Biomedical Research Center at Guy's and St Thomas' National Health Service Foundation Trust and King's College London. I Moreno Velasquez is supported by the Sistema Nacional de Investigacion (SENACYT, Panama). G C Patton is funded by an NHMRC Fellowship. A M Samy received a fellowship from the Egyptian Fulbright Mission programme. M M Santric-Milicevic acknowledges the support of the Ministry of Education, Science and Technological Development of the Republic of Serbia (contract number 175087). A Sheikh acknowledges the support of Health Data Research UK. M R Sobhiyeh acknowledges the Clinical Research Development Center of Imam Reza Hospital, Kermanshah University of Medical Sciences for their wise advice. R Tabares-Seisdedos was supported in part by grant PI17/00719 from Instituto de Salud Carlos III-FEDER. B Unnikrishnan acknowledges Manipal Academy of Higher Education, Manipal. M R Weaver was supported by the Bill & Melinda Gates Foundation grant OPP1127433. C S Wiysonge was supported by the South African Medical Research Council. 52 25 26 4 64 ELSEVIER SCI LTD OXFORD THE BOULEVARD, LANGFORD LANE, KIDLINGTON, OXFORD OX5 1GB, OXON, ENGLAND 2214-109X LANCET GLOB HEALTH Lancet Glob. Health AUG 2020 8 8 1038 1060 10.1016/S2214-109X(20)30230-8 http://dx.doi.org/10.1016/S2214-109X(20)30230-8 23 Public, Environmental & Occupational Health Science Citation Index Expanded (SCI-EXPANDED); Social Science Citation Index (SSCI) Public, Environmental & Occupational Health NG0WI 32710861 Green Published, Green Accepted, Green Submitted, gold 2025-06-24 WOS:000563709300024
J Krishnan, K; Thomas, A Krishnan, Kavita; Thomas, Antony Correlation of Cervical Cytology With High-Risk HPV Molecular Diagnosis, Genotypes, and Histopathology-A Four Year Study From the UAEDIAGNOSTIC CYTOPATHOLOGY English Article cervical cytology; HPV; ASCUS; genotype HUMAN-PAPILLOMAVIRUS GENOTYPES; INTRAEPITHELIAL NEOPLASIA; YOUNG-WOMEN; PREVALENCE; CANCER; LESIONS; INFECTION; GRADE; COLPOSCOPY; TIME Background: Cervical cancer is one of the most common cancers among women in the world and in the Middle East. Its prevention and screening strategies assume great importance especially in view of the ability to identify the illness at an early stage and disrupt its progression toward neoplasia. Cervical cancer screening program of our center in UAE as well as this study is oriented in this direction. Methods: Cervical cytology data encompassing 4 years (20112014) was compiled and analyzed for 14,950 cases to assess the abnormal smear reporting pattern, correlation with biopsy histopathology, HPV high-risk screening data, and HPV genotype prevalence among patients with abnormal smear test, as well as in relation to the respective nationalities of patients. Results: Abnormal smear rates as well as HPV high-risk positivity correlated well with established data. Cytology-histology correlation was good. HPV high-risk type 16 was the commonest, type 18 which is the second common type worldwide was less frequently detected, several other high risk subtypes such as 51, 31 as well as rare types such as 66, 56, and 59 were detected in a significant number of patients. Conclusion: Assessment of cervical cytology reporting pattern, correlation of cervical cytology with HPV molecular diagnosis, and biopsy histopathology is a useful way to audit our work and a good

quality control practice. Higher prevalence of HPV HR types such as type 51, 31 as well as 66, 56, and 59 found in the present as well as many similar other studies apart from the commonest subtype 16 may imply necessity for development of more targeted and multivalent anti-HPV vaccines for protection of women in our region. HPV genotypes correlation with the nationalities of patients is useful from an epidemiological standpoint to plan preventive strategies. (C) 2015 Wiley Periodicals, Inc.
[Krishnan, Kavita] Prime Healthcare Grp, Premier Diagnost Ctr, Anat Pathol, Dubai, U Arab Emirates; [Thomas, Antony] Prime Healthcare Grp, Premier Diagnost Ctr, Dubai, U Arab Emirates
Krishnan, K (corresponding author), Prime Healthcare Grp, Premier Diagnost & Med Ctr, POB 7162, Dubai, U Arab Emirates.
drkavita@primehealth.ae
46 9 9 0 5 WILEY HOBOKEN 111 RIVER ST, HOBOKEN 07030-5774, NJ USA 8755-1039 1097-0339 DIAGN CYTOPATHOL Diagn. Cytopathol. FEB 2016 44 2
91 97 10.1002/dc.23391
http://dx.doi.org/10.1002/dc.23391 7
Medical Laboratory Technology; Pathology Science Citation Index Expanded (SCI-EXPANDED) Medical Laboratory Technology; Pathology DH7DT 26610743 Bronze
2025-06-24 WOS:000372952600004
J Golshani, SA; Golshan, SE; Zohalinezhad, ME
Golshani, Seyyed Alireza; Golshan, Seyyed Ehsan; Zohalinezhad, Mohammad Ebrahim The Life and Works of Sadid al-Din Kazeroni: An Iranian Physician and Anatomist
MIDDLE EAST JOURNAL OF CANCER English
Article Sadid al-Din Kazeroni; Breast cancer; Anatomical illustration; Al-Mughni; Persian medicine Background: One of the great physicians in Iran who had expertise in medicine, surgery, and pharmacy was Sadid al-Din Kazeroni. He was a 14th century physician. No information is available on his birth and death - only "Al-Mughni", a book, has been left to make him famous in surgical and medical knowledge. Methods: We used desk and historical research methods in this research, with a historical approach. This commonly used research method in human sciences was used to criticize and study the birthplace and works of Sadid al-Din Kazeroni. Results and Conclusion: Sadid al-Din Kazeroni discussed the exact issues in the field of anatomy, surgery, and gynecology. He was fluent in pharmacology. In his pharmacology book, for the first time, he named drugs considered necessary before and after surgery. In this study, we reviewed the biography and introduction of the works and reviewed "Al-Mughni", a book on breast cancer.
[Golshani, Seyyed Alireza] Ferdowsi Univ Mashhad, Dept Hist, Mashhad, Iran; [Golshan, Seyyed Ehsan] Marvdasht Azad Univ, Dept Foreign Languages, Marvdasht, Iran; [Zohalinezhad, Mohammad Ebrahim] Shiraz Univ Med Sci, Persian Med, Shiraz, Iran; [Zohalinezhad, Mohammad Ebrahim] Shiraz Univ Med Sci, Tradit Med & Med Plant Incubator, Eessence Parsiyan Wisdom Inst, Shiraz, Iran
Ferdowsi University Mashhad; Shiraz University of Medical Science; Shiraz University of Medical Science Golshani, SA (corresponding author), Ferdowsi Univ Mashhad, Dept Hist, Fac Literature & Humanities Dr Ali Shariati, Mashhad, Iran.
s.alirezagolshani@mail.um.ac.ir Zohalinezhad, Mohammad/T-9061-2018; Golshani, Seyyed/ABH-8396-2020 Golshani, Seyyed Alireza/0000-0003-1635-4578; Zohalinezhad, Mohammad Ebrahim/0000-

0002-5538-8247 Shiraz University of Medical Sciences Shiraz University of Medical Sciences(Shiraz University of Medical Science) The authors disclosed financial support for the research, authorship, and/or publication of this article from Shiraz University of Medical Sciences. 13 0 0 0 0 SHIRAZ UNIV MEDICAL SCIENCESSHIRAZ NEMAZEE HOSPITAL, SHIRAZ, 71934, IRAN 2008-6709 2008-6687 MIDDLE EAST J CANCER Middle East J. Cancer OCT 2018 9 4 323 327 5 Oncology Emerging Sources Citation Index (ESCI) Oncology GW2HK 2025-06-24 WOS:000446703700009

J Zheng, JL; Merchant, AT; Wirth, MD; Zhang, JJ; Antwi, SO; Shoaibi, A; Shivappa, N; Stolzenberg-Solomon, RZ; Hebert, JR; Steck, SE Zheng, Jiali; Merchant, Anwar T.; Wirth, Michael D.; Zhang, Jiajia; Antwi, Samuel O.; Shoaibi, Azza; Shivappa, Nitin; Stolzenberg-Solomon, Rachael Z.; Hebert, James R.; Steck, Susan E. Inflammatory potential of diet and risk of pancreatic cancer in the Prostate, Lung, Colorectal and Ovarian (PLCO) Cancer Screening Trial INTERNATIONAL JOURNAL OF CANCER English Article dietary patterns; dietary inflammatory index; incidence; pancreatic cancer; cohort study MEDITERRANEAN DIET; INDEX; METAANALYSIS; ASSOCIATION; VALIDATION; SCORE

Inflammation plays a central role in pancreatic cancer etiology and can be modulated by diet. We aimed to examine the association between the inflammatory potential of diet, assessed with the Dietary Inflammatory Index (DII (R)), and pancreatic cancer risk in the Prostate, Lung, Colorectal and Ovarian Cancer Screening Trial prospective cohort. Our study included 101,449 participants aged 52-78 years at baseline who completed both baseline questionnaire and a diet history questionnaire. Energy-adjusted DII (E-DII) scores were computed based on food and supplement intake. Cox proportional hazards models and time dependent Cox models were used to estimate hazard ratios (HRs) and 95% confidence intervals (CIs) with participants in the lowest E-DII quintile (most anti-inflammatory scores) as referent. After a median 8.5 years of follow-up, 328 pancreatic cancer cases were identified. E-DII scores were not associated with pancreatic cancer risk in the multivariable model (HRQ5vsQ1 = 0.94; 95% CI = 0.66-1.35; p-trend = 0.43). Time significantly modified the association (p-interaction = 0.01). During follow up <4 years, there was suggestive evidence of an inverse association between E-DII and pancreatic cancer (HRQ5vsQ1 = 0.60; 95% CI = 0.35-1.02; p-trend = 0.20) while there was a significant positive trend in the follow up >= 4 years (HRQ5vsQ1 = 1.31; 95% CI = 0.83-2.08; p-trend = 0.03). Similar results were observed for E-DII from food only. Our study does not support an association between inflammatory potential of diet and pancreatic cancer risk; however, heterogeneous results were obtained with different follow-up times. These divergent associations may result from the influences of undetected disease in the short-term. [Zheng, Jiali; Merchant, Anwar T.; Wirth, Michael D.; Zhang, Jiajia; Shivappa, Nitin; Hebert, James R.; Steck, Susan E.] Univ South Carolina, Arnold Sch Publ Hlth, Dept Epidemiol & Biostat, Columbia, SC 29208 USA; [Zheng, Jiali; Wirth, Michael D.; Shivappa, Nitin; Hebert, James R.; Steck, Susan E.] Univ South Carolina, Canc Prevent & Control Program, Columbia, SC 29208 USA; [Zheng, Jiali] Univ Texas

MD Anderson Canc Ctr, Div Canc Prevent & Populat Sci, Dept Epidemiol, Houston, TX 77030 USA; [Wirth, Michael D.; Shivappa, Nitin; Hebert, James R.] Connecting Hlth Innovat LLC, Columbia, SC USA; [Antwi, Samuel O.] Mayo Clin, Div Epidemiol, Dept Hlth Sci Res, Jacksonville, FL 32224 USA; [Shoaibi, Azza] Med Univ South Carolina, Biomed Informat Ctr, Charleston, SC 29425 USA; [Stolzenberg-Solomon, Rachael Z.] Natl Canc Inst NCI DCEG, Metab Epidemiol Branch, Div Canc Epidemiol & Genet, Rockville, MD USA
University of South Carolina System; University of South Carolina Columbia; University of South Carolina System; University of South Carolina Columbia; University of Texas System; UTMD Anderson Cancer Center; Connecting Health Innovations LLC; Mayo Clinic; Medical University of South Carolina; National Institutes of Health (NIH) - USA; NIH National Cancer Institute (NCI)
Steck, SE (corresponding author), Univ South Carolina, Dept Epidemiol & Biostat, 915 Greene St,Rm 454,Discovery 1 Bldg, Columbia, SC 29208 USA. ssteck@sc.edu Wirth, Michael/C-6330-2013; Hebert, James/IUO-5628-2023; Stolzenberg-Solomon, Rachael/Y-9280-2018; Merchant, Anwar/B-5233-2009; Zheng, Jiali/I-5084-2019; Steck, Susan/G-5736-2013; Antwi, Samuel/ABB-4836-2021; Shivappa, Nitin/X-2215-2018 Zheng, Jiali/0000-0003-1434-2856; Antwi, Samuel/0000-0003-2372-9489; Shivappa, Nitin/0000-0003-0441-8896 Support to Promote Advancement of Research and Creativity (SPARC) grant by University of South Carolina Office of the Vice President for Research; National Institute of Diabetes and Digestive and Kidney Diseases [R44 DK103377]; Cancer Prevention & Research Institute of Texas [RP170259]; National Cancer Institute [ZIACP010202] Funding Source: NIH RePORTER Support to Promote Advancement of Research and Creativity (SPARC) grant by University of South Carolina Office of the Vice President for Research; National Institute of Diabetes and Digestive and Kidney Diseases(United States Department of Health & Human ServicesNational Institutes of Health (NIH) - USANIH National Institute of Diabetes & Digestive & Kidney Diseases (NIDDK)); Cancer Prevention & Research Institute of Texas(Cancer Prevention & Research Institute of Texas); National Cancer Institute(United States Department of Health & Human ServicesNational Institutes of Health (NIH) - USANIH National Cancer Institute (NCI)) Grant sponsor: Support to Promote Advancement of Research and Creativity (SPARC) grant by University of South Carolina Office of the Vice President for Research; Grant sponsor: National Institute of Diabetes and Digestive and Kidney Diseases; Grant number: R44 DK103377; Grant sponsor: Cancer Prevention & Research Institute of Texas; Grant number: RP170259 50 31 33 0 24
WILEY HOBOKEN 111 RIVER ST, HOBOKEN 07030-5774, NJ USA
0020-7136 1097-0215 INT J CANCER Int. J. Cancer
JUN 15 2018 142 12 2461 2470
10.1002/ijc.31271
http://dx.doi.org/10.1002/ijc.31271 10
Oncology Science Citation Index Expanded (SCI-EXPANDED)
Oncology GD3GB 29355939 Green Accepted
2025-06-24 WOS:000430390800005
J Nejatollahi, F; Silakhori, S; Moazen, B
Nejatollahi, Foroogh; Silakhori, Samira; Moazen, Bahareh
Isolation and Evaluation of Specific Human Recombinant Antibodies from a Phage Display Library against HER3 Cancer Signaling Antigen MIDDLE EAST JOURNAL OF CANCER
English Article Breast

cancer; HER3; Specific scFvs; Antigen targeting MONOCLONAL-ANTIBODY; BINDING; EXPRESSION; INHIBITORS; RECEPTOR; GROWTH; CELLS; ERBB3 Background: The human epidermal growth factor receptor family comprises four homologous members: EGFR (ErbB1), ErbB2 (HER2), ErbB3 (HER3) and ErbB4 (HER4). This family plays an important role in the signaling pathway and cell proliferation. The heterodimerization of HER2 with HER3 leads to tumor cell proliferation. Monoclonal antibody to the human HER3 receptor blocks HER3 heterodimerization and inhibits the growth of breast cancer cells. Due to their human origin, small size, rapid penetration and high affinity properties, recombinant single chain antibodies (scFv) have been introduced as the most desired agents for cancer immunotherapy. In this study, we use a phage display system to select specific scFvs against HER3 for their use in cancer targeted therapy. Methods: A phage antibody display library of scFv was panned against an immunodominant epitope of HER3. Phage rescue was performed on the library. The supernatant that contained the appropriate scFv (109 PFU/ml) was added to an immunotube which was coated with the peptide. Elution was done using log phase E. coli TG1. The clones were amplified by PCR and DNA fingerprinted to select the specific clones against the epitope. The specificity of the selected antibodies was tested in ELISA. Results: The results represented two predominant patterns with the frequency of 25%. The other patterns showed the frequencies of 5%-10%. scFv1 and scFv2 demonstrated positive ELISA with absorbances of 0.63 and 0.46, respectively while the absorbances of wells without peptide were 0.19 and 0.11, respectively. Conclusion: In this study two specific scFvs were selected against HER3 antigen in a successful panning process. Phage ELISA represented the specific binding of scFvs against HER3. The selected scFvs reacted only with the corresponding peptides. However, no reaction with the other peptides was detected. The selected anti-HER3 scFvs have suggested that these human high affinity and small antibodies that bind specifically to HER3 epitope can be considered in HER3 targeted approaches.

[Nejatollahi, Foroogh] Shiraz Univ Med Sci, Shiraz HIV AIDS Res Ctr, Shiraz, Iran; [Nejatollahi, Foroogh; Silakhori, Samira] Shiraz Univ Med Sci, Dept Immunol, Recombinant Antibody Lab, Shiraz, Iran; [Moazen, Bahareh] Shiraz Univ Med Sci, Student Res Comm, Shiraz, Iran Shiraz University of Medical Science; Shiraz University of Medical Science; Shiraz University of Medical Science Nejatollahi, F (corresponding author), Shiraz Univ Med Sci, Dept Immunol, Shiraz, Iran. nejatollaf@sums.ac.ir

Nejatollahi, Foroogh/V-6434-2017 Nejatollahi, Foroogh/0000-0002-8928-8519 Shiraz University of Medical Sciences; [2857]

Shiraz University of Medical Sciences(Shiraz University of Medical Science); The authors would like to express their appreciation to Shiraz University of Medical Sciences for financial support. The present article was extracted from a thesis written by Samira Silakhori, grant no. 2857. 33 8 8

0 0 SHIRAZ UNIV MEDICAL SCIENCES SHIRAZ NEMAZEE HOSPITAL, SHIRAZ, 71934, IRAN 2008-6709 2008-6687

MIDDLE EAST J CANCER Middle East J. Cancer JUL 2014 5

3 137 144

8 Oncology Emerging Sources Citation Index (ESCI)

Oncology V34EQ 2025-06-24

WOS:000215816700004
[truncated: 2,174,044 more chars]
